# What Are the Reliable Plasma Biomarkers for Mild Cognitive Impairment? A Clinical 4D Proteomics Study and Validation

**DOI:** 10.1155/2024/7709277

**Published:** 2024-05-27

**Authors:** Zhitao Hou, Ailin Sun, Yan Li, Xiaochen Song, Shu Liu, Xinying Hu, Yihan Luan, Huibo Guan, Changyuan He, Yuefeng Sun, Jing Chen

**Affiliations:** ^1^ College of Basic Medical and Sciences Heilongjiang University of Chinese Medicine, Harbin 150040, Heilongjiang, China; ^2^ Department of Systems Pharmacology and Translational Therapeutics Perelman School of Medicine University of Pennsylvania, Philadelphia 19104, PA, USA; ^3^ Key Laboratory of Chinese Internal Medicine of the Ministry of Education Dongzhimen Hospital Affiliated with Beijing University of Chinese Medicine, Beijing 100700, China; ^4^ The First Hospital Affiliated with Heilongjiang University of Chinese Medicine, Harbin 150010, Heilongjiang, China; ^5^ Pudong Hospital Affiliated with Fudan University, Shanghai 200120, China

## Abstract

**Objective:**

At present, Alzheimer's disease (AD) lacks effective treatment means, and early diagnosis and intervention are the keys to treatment. Therefore, for mild cognitive impairment (MCI) and AD patients, blood sample analysis using the 4D nonstandard (label-free) proteomic in-depth quantitative analysis, looking for specific protein marker expression differences, is important. These marker levels change as AD progresses, and the analysis of these biomarkers changes with this method, which has the potential to show the degree of disease progression and can be used for the diagnosis and preventive treatment of MCI and AD.

**Materials and Methods:**

Patients were recruited according to the inclusion and exclusion criteria and divided into three groups according to scale scores. Elderly patients diagnosed with AD were selected as the AD group (*n* = 9). Patients diagnosed with MCI were classified into the MCI group (*n* = 10). Cognitively healthy elderly patients were included in the normal cognition control group (*n* = 10). Patients' blood samples were used for 4D label-free proteomic in-depth quantitative analysis to identify potential blood biomarkers. The sample size of each group was expanded (*n* = 30), and the selected biomarkers were verified by enzyme-linked immunosorbent assay (ELISA) to verify the accuracy of the proteomic prediction.

**Results:**

Six specific blood markers, namely, APOE, MMP9, UBR5, PLA2G7, STAT5B, and S100A8, were detected by 4D label-free proteomic quantitative analysis. These markers showed a statistically significant upregulation trend in the MCI and AD groups compared with the normal cognition control group (*P* < 0.05). ELISA results showed that the levels of these six proteins in the MCI group were significantly higher than those in the normal cognition control group, and the levels of these six proteins in the AD group were significantly higher than those in the MCI group (*P* < 0.05).

**Conclusion:**

The plasma levels of APOE, MMP9, UBR5, PLA2G7, STAT5B, and S100A8 in cognitively healthy elderly patients and patients with MCI and AD were significantly different and, more importantly, showed a trend of increasing expression. These results indicate that these six human plasma markers have important diagnostic and therapeutic potential in the identification of cognitive impairment and have value for in-depth research and clinical application.

## 1. Introduction

Mild cognitive impairment (MCI) is a cognitive condition between normal aging cognition and dementia characterized by a higher-than-normal age-related cognitive decline that is not enough to result in significant impairment of daily functions [1]. The American Academy of Neurology guidelines estimate the prevalence of MCI to be 6.7% in 65–69-year-olds and 25% in 80–84-year-olds [2]. MCI is progressive and is closely associated with oxidative stress, inflammation, and atherosclerosis-related diseases, especially Alzheimer's disease (AD), and the incidence of progression to dementia can be as high as 10%–15% each year. An increase in age is the main risk factor for MCI, in addition to the living environment, education level, marital status, smoking, hypertension, hyperlipidemia, diabetes, heart disease, and cerebrovascular disease [3]. The main clinical symptoms of MCI are impaired memory, impaired executive function, cognitive impairment such as visual–spatial function in language use, and neuropsychiatric and psychological abnormalities. Since the symptoms are not obvious when the disease occurs and activities of daily living (ADL) are not significantly affected, MCI is usually ignored or missed. Only when the patient's cognitive impairment develops to the AD stage will it be taken seriously, which limits the treatment options at this time [4]. Although research on drugs aimed at protecting brain function is on the rise, there are currently only a handful of drugs approved by the Food and Drug Administration that can be used to treat MCI and hinder the progression of MCI to AD, such as Aduhelm, Donepezil, and Ebixa, among others. AD brings not only great pain to patients but also heavy mental pressure and medical care burdens to families and society. Therefore, early diagnosis and treatment of MCI can play an important role in delaying the onset of AD, improving the quality of life of patients, and alleviating the burden on families and society.

There is currently no gold standard for the diagnosis of MCI. The main diagnostic methods include neuropsychological assessment, imaging, and fluid testing, and their use depends on the clinician's knowledge of MCI [5]. The excessive deposition of amyloid-*β* (A*β*) in the brain and the A*β* cascade reaction are important mechanisms of the pathogenesis of AD. Therefore, early detection of A*β* deposition by A*β*-specific positron emission tomography (PET) imaging can help identify patients with MCI, but there are problems such as high cost, long operation time, and increased radiation exposure. Similarly, although humoral tests, such as cerebrospinal fluid (CSF) tests for Tau protein and A*β*42, can detect early AD, they are limited by the invasiveness of obtaining samples by lumbar puncture [6, 7]. Therefore, it is important to find and identify standardized and easy tests for the diagnosis of MCI.

Blood testing has become a research hotspot in the diagnosis of MCI due to its characteristics of easy sample acquisition, minimal trauma, and high acceptance by patients. Some blood markers have been studied extensively, such as A*β* Tau protein and P-Tau NFL, which can also be used to predict the progression of AD [8, 9, 10]. In recent years, proteomic technology based on mass spectrometry (MS) has been widely used to screen blood biomarkers. Proteomics is essentially the large-scale study of the characteristics of proteins under different conditions to obtain a comprehensive understanding of disease mechanisms, cell metabolism, and other processes at the protein level. 4D proteomics refers to an advanced approach that adds the fourth dimension of ion mobility separation (also known as collision cross-section or CCS) to the traditional 3D proteomics analysis based on retention time, mass-to-charge ratio, and peptide intensity. CCS describes the volume occupied by an ion during its migration, which depends on the ion's molecular structure and interactions with air molecules. By incorporating ion mobility separation, 4D proteomics can identify and quantify proteins with higher precision, accuracy, and reliability, making it a groundbreaking method in the field of proteomics [11, 12, 13]. Through liquid chromatography–MS (LC–MS), the corresponding protein can be quantified by comparing the signal intensity of the corresponding peptide in different samples. In this study, we applied a high-depth blood proteomic technique to effectively quantify the plasma proteome of patients and finally identified candidate biomarkers for MCI.

## 2. Materials and Methods

### 2.1. Grouping of Patients

A total of 90 patients in the First Affiliated Hospital of Heilongjiang University of Chinese Medicine from October 2021 to April 2023 were enrolled. According to Petersen's MCI diagnostic criteria published in 2001 [14] and the AD diagnostic criteria published by the National Institute on Aging and the Alzheimer's Disease Association (NIA-AA) in 2011 [15], the study patients were divided into the proteomic study group (10 patients with normal cognition, 10 patients with MCI and 9 patients with AD) and the validation study group (90 patients; 30 with normal cognition, 30 with MCI, and 30 with AD). The patient's name, gender, age, education and other basic information, medical history, and related examinations were recorded (*Supplementary 1*). All participants were screened using the Montreal Cognitive Assessment (MoCA), ADL scale, clinical dementia rating (CDR), and Hachinski ischemic score (HIS), and those who passed the five examinations were enrolled. Please refer to the attachment for specific criteria (*Supplementary 2*).

Patients were excluded if they met any of the following exclusion criteria: (1) infarction, infection, or other lesions of the central nervous system; (2) persistent severe neurological deficits or brain structural abnormalities due to a history of trauma; (3) serious sleep disorders or other mental diseases or a history of drug or alcohol dependence and abuse; (4) an inability to communicate, serious hearing or a visual impairment, aphasia, or an inability to complete the screening tests; (5) other mental/neurological diseases leading to dementia, certain diseases that can interfere with the evaluation of cognitive function, extrapyramidal dysfunction, or severe cognitive disturbances; (6) serious liver, kidney, and heart diseases; (7) participation in drug trials; and (8) other conditions making the patient not suitable for inclusion in the study as judged by the researcher.

According to the Ethical Review Measures for Biomedical Research involving humans of the Ministry of Health of the People's Republic of China (2016), the Quality Management Code for Drug Clinical Trials of the State Medical Products Administration (2020), the Quality Management Code for Clinical Trials of Medical Devices (2016), the ethical principles of the Declaration of Helsinki of the World Medical Association (2013) and the International Ethical Guidelines for Human Biomedical Research (2020), this study was reviewed and approved by the Ethics Committee of the First Affiliated Hospital of Heilongjiang University of Chinese Medicine (No. HZYLLKY 202101201). As required, all participants signed written informed consent to participate in the study.

### 2.2. Plasma Sample Collection

Blood samples were obtained from all patients by venipuncture, collected into 5 mL EDTA-containing vacuum tubes, gently mixed by turning upside down 8–10 times, and immediately placed on ice. After centrifugation at 1,300 RPM at 4°C for 10 min, plasma samples were obtained and stored at −80°C until proteomic analysis.

### 2.3. Proteomic Analysis

#### 2.3.1. Protein Extraction

The samples were removed from the −80°C freezer and centrifuged at 4−8°C and 12,000 RPM for 10 min. The cell fragments were removed. The supernatant was transferred to a new centrifuge tube. High-abundance proteins were removed with a Pierce™ Top 14 Abundant Protein Depletion Spin Column Kit (Thermo Fisher Scientific). The protein concentration was determined using a bicinchoninic acid kit (Thermo Fisher Scientific).

#### 2.3.2. Trypsin Enzymatic Hydrolysis

The proteins of each sample were enzymatically hydrolyzed in equal quantities, and the volume was adjusted to be the same as that of the lysate. Then, dithiothreitol was added to a final concentration of 5 mM, and the protein was reduced at 56°C for 30 min. Then, iodine acetamide was added to a final concentration of 11 mM, and the mixture was incubated for 15 min at room temperature, away from light. The alkylated samples were transferred to an ultrafiltration tube, centrifuged at 12,000 RPM at room temperature for 20 min, replaced with 8 M urea three times, and then replaced with replacement buffer three times. Trypsin was added at a ratio of 1 : 50 (protease: protein, M/M), and the samples were enzymolyzed overnight. The peptide was centrifuged at 12,000 RPM for 10 min at room temperature to recover the peptide. The peptide was then recovered once with ultrapure water, and the peptide solution was combined twice.

#### 2.3.3. LC–MS Analysis

The peptides were dissolved by mobile phase A and separated by an ultrahigh-performance liquid chromatography system, Easy-NLC 1200. Mobile phase A was an aqueous solution containing 0.1% formic acid and 2% acetonitrile. Mobile phase B was an aqueous solution containing 0.1% formic acid and 90% acetonitrile. The gradient setting was 0–68 min, 4%–20% B; 68–82 min, 20%–32% B; 82–86 min, 32%–80% B; and 86–90 min, 80% B. The flow rate was maintained at 500 nL/min. The peptides were separated by an ultrahigh-performance liquid phase system, injected into a nanospray ionization source for ionization, and then analyzed by an Orbitrap Exploris™ 480 mass spectrometer. The ion source voltage was set to 2.3 kV, and the field asymmetric waveform ion mobility spectrometry compensation voltage (CV) was set to −45 V and −70 V. In the MS analysis, a peptide concentration of 1 *μ*g/*μ*L was injected, with a 4 *μ*L sample loaded for analysis. A high-resolution Orbitrap was used for the detection and analysis of the peptide parent ions and their secondary fragments. The scanning range of first-level MS was set as 400–1,200 *m*/*z*, and the scanning resolution was set as 60,000. The scanning range of secondary MS was fixed as 110 *m*/*z*, the resolution of secondary scanning was set as 30,000, and TurboTMT was set as off. In the data collection mode, the data-dependent scan program was used; that is, the first 15 peptide parent ions with the highest signal intensity were selected to enter the higher-energy C-trap dissociation (HCD) collision cell successively after the first-level scan. Fragmentation was performed at 27% of fragmentation energy, and secondary MS was also performed in sequence. To improve the efficiency of MS, the automatic gain control was set to 75%, the signal threshold was set to 1E4 ions/s, the maximum injection time was set to 100 ms, and the dynamic exclusion time of the tandem MS scan was set to 30 s to avoid repeated scanning of parent ions.

#### 2.3.4. Database Retrieval

Secondary MS data were retrieved by Proteome Discoverer (V2.4.1.15). Retrieval parameter settings were as follows: the Homo _sapiens _9606_PR _ 202107 21 was used; the FASTA (78,120 sequences) anti-library was added to calculate the false detection rate (FDR) caused by random matching; the enzyme digestion mode was set to trypsin (full); the number of missing cuts was set to 2; the minimum peptide length was set to six amino acid residues; and the maximum number of peptide modifications was set to 3. The mass error tolerance of the primary parent ion was set as 10 PPM, and that of the secondary fragment ion was 0.02 Da. The carbamidomethyl (C) was set as the fixed modifier, and the oxidation (M), acetyl (N-terminus), met-loss (M), and met-loss + acetyl (M) were set as the variable modifiers. The FDRs for protein, peptide, and peptide-spectrum match identification were all set at 1%.

#### 2.3.5. MS Quality Control Test and Sample Repeatability Test

Most of the peptides were composed of 7–20 amino acids in accordance with the general rules of trypsin-based enzymatic hydrolysis and HCD fragmentation. Among them, effective sequence identification could not be performed for peptides with less than five amino acids due to too few fragment ions generated. Peptides with more than 20 amino acids were not suitable for HCD fragmentation due to their high mass and charge number. The distribution of peptide length identified by MS met the requirements of quality control. To test whether the quantitative results of biological or technical replication samples were statistically consistent, three statistical analysis methods, namely Pearson's correlation coefficient (PCC), principal component analysis (PCA), and relative standard deviation (RSD), were used to evaluate the quantitative repeatability of protein results.

#### 2.3.6. Power Analysis

Power analysis was conducted using the “pwr” package (version = 1.3.0) in R, which contains a range of important functions. For each function, three of the four quantities (sample size, significance level, power, and effect size) can be specified, and the software calculates the fourth quantity.

For the *t*-test, the pwr.t.test() function offers many useful options for power analysis with the following parameters:

1. “*n*” specifies the sample size;
2. “*d*” specifies the effect size, which is the standardized difference between means;

(1) $$\begin{array}{c} d=\frac{\mu_{1}-\mu_{2}}{\sigma }, \end{array}$$

“where *μ*1 is the mean of group 1, *μ*2 is the mean of group 2, and *δ* is the variance of error.”

(3) “sig.level” specifies the significance level (default is 0.05);
(4) “power” specifies the desired power level;
(5) “type” specifies the type of *t*-test: two-sample, one-sample, or paired. The default is two-sample.
(6) “alternative” specifies whether the *t*-test is two-sided (two.sided) or one-sided (less or greater). The default is two-sided.

#### 2.3.7. Differential Protein Screening

For three or more replicates: firstly, select the samples that need to be compared, and calculate the fold change (FC) of each protein by dividing the mean relative quantitative value of each protein in the multiple replicates of the samples in group *A* by the mean relative quantitative value of each protein in the multiple replicates of the samples in group *B*. For example, calculating the protein FC between sample group *A* and sample group *B*. The formula for calculation is as follows, where *R* represents the relative quantitative value of the protein, *i* represents the sample, and *k* represents the protein.

(2) $$\begin{array}{c} {FC}_{A/B,k}=\text{Mean }\left(R_{ik},i\in A\right)/\text{Mean }\left(Rik,i\in B\right). \end{array}$$

In order to determine the significance of the difference, a *t*-test is performed on the relative quantitative values of each protein in the comparison group samples, and the corresponding *P*-value is calculated as the significance index, with a default *P*-value of less than 0.05. To make the test data conform to the normal distribution required by the *t*-test, the relative quantitative values of the protein need to be transformed by logarithm base 2 (Log2) before the test. The formula for calculation is as follows:

(3) $$\begin{array}{c} P_{ik}=T.test\;\left(\text{Log}2\;\left(Rik,i\in A\right),\text{ Log}2\;\left(R_{ik},i\in B\right)\right). \end{array}$$

For two replicates: first, select the samples that need to be compared, and calculate the FC of each protein by dividing the mean relative quantitative value of each protein in the two replicate comparison groups. For example, calculating the protein FC between sample group *A* and sample group *B*. The formula for calculation is as follows, where *R* represents the relative quantitative value of the protein, *i* represents the sample, and *k* represents the protein.

(4) $$\begin{array}{c} {FC}_{A/B,k}=\text{Mean}\left(\left(R_{ik},i\in A\right)|/|\left(Rik,i\in B\right)\right). \end{array}$$

In order to determine the significance of the difference, calculate the coefficient of variation (CV) of each protein in the two comparison groups as the significance index, with a default CV of less than 0.1. The formula for calculation is as follows:

(5) $$\begin{array}{c} CV=SD\left(A_{1k}/B_{1k},A_{2k}/B_{2k}\right)/\text{Mean}\left(A_{1k}/B_{1k},A_{2k}/B_{2k}\right). \end{array}$$

For no replicates: first, select the samples that need to be compared, and calculate the FC of each protein by dividing the relative quantitative value of each protein in sample *A* by the relative quantitative value of each protein in sample *B*. For example, calculating the protein FC between sample *A* and sample *B*. The formula for calculation is as follows, where *R* represents the relative quantitative value of the protein, and *k* represents the protein.

(6) $$\begin{array}{c} {FC}_{A/B,k}=R_{Ak}/R_{Bk}. \end{array}$$

Based on the above differential analysis, when the *P*-value is less than 0.05, a change threshold of greater than 1.5 is used as the significant upregulation threshold, and less than 1/1.5 is used as the significant downregulation threshold.

#### 2.3.8. Search for MCI-Related Modules Based on Mfuzz Analysis

Eggnog-mapper software (V2.0) was used to carry out Gene Ontology (GO) annotations for the identified proteins, mainly including the three aspects of biological process, cell components, and molecular functions. The protein pathways were annotated using the Kyoto Encyclopedia of Genes and Genomes (KEGG) pathway database. Fisher's exact tests were performed for the GO and KEGG pathway enrichment significance analysis of differentially expressed proteins (with the identified proteins as background). A *P* value < 0.05 was considered significant. Cluster analysis of expression patterns revealed significant changes in protein abundance in the normal cognition control group compared with the MCI group and AD group and identified modules associated with MCI.

#### 2.3.9. Protein Interaction Network Analysis

The protein numbers or sequences screened from the differentially expressed protein databases of the different groups were compared with the STRING (V.10.5) protein network database, and the results were compared according to the extracted differential protein interaction relationships with a confidence degree >0.4 (high confidence). Then, the R language packet network D3 tool was used to visualize the differential protein interaction network.

#### 2.3.10. Enzyme-Linked Immunosorbent Assay (ELISA) Validation Test

A human proteinase ELISA kit (Provided by Hangzhou Jingjie Biotechnology Co., Ltd.) was used to determine endogenous protein levels in plasma according to the manufacturer's instructions. It mainly includes reagent kits for the following types: APOE, UBR5, MMP9, S100A8, STAT5B, and PLA2G7. First, standard diluent was configured according to the instructions: 50 *μ*L of sample was accurately added to the standard coated plate, 40 *μ*L of sample diluent was added to the sample well, and 10 *μ*L of the sample to be tested was added to the well. The incubation plate was closed with a sealing plate membrane and incubated for 37 min. A washing solution diluted 30-fold with distilled water was added for washing 30 times, and the sealing plate membrane was carefully removed after incubation. The liquid was discarded, and the plate was dried by swinging. The washing solution was added to each well and allowed to be set for 30 s, and then the washing solution was discarded; this process was repeated five times. The plate was dried by patting. Horseradish peroxidase-conjugated reagent (50 *μ*L) was added to each well, except for the blank well. The incubation and washing steps were repeated for color. Chromogenic agent *A* (50 *μ*L) was added to each well. Chromogenic agent *B* (50 *μ*L) was added, mixed gently, and incubated at 37°C in the dark for 10 min. Finally, 50 *μ*L of stop solution was added to each well, the stop reaction was set to zero at the blank well, and the absorbance of each well was measured in sequence at 450 nm. The standard curve of each protein was determined by the continuous dilution of a standard sample of known protein concentration provided by the manufacturer.

#### 2.3.11. Multiple-Group Analysis Based on Logistic Regression: Using Biological Markers and Participant Scores

We set the total sample size to 90, including three groups: 30 with normal cognition, 30 with MCI, and 30 with AD. Using ELISA measurements of six biological markers (APOE, UBR5, MMP9, S100A8, STAT5B, and PLA2G7) and four participant scores (CDR, MoCA, ADL, and HIS), we constructed binary classification models for each pair of features using logistic regression. Prior to constructing the models, the 11 features were standardized using *Z*-scores to ensure that they had the same scale and mean. The total sample data was then divided into training and testing sets in an 8 : 2 ratio, with the proportion of each group being equal in both sets. Logistic regression models were trained on the training set, and the model's performance was tested on the testing set. MATLAB programing language, please refer to *Supplementary 3*.

### 2.4. Statistical Analysis

SPSS 22 software was used for statistical analysis. The measured data were normally distributed and are statistically presented as *x* ± *s*. An independent-sample *t*-test was used for the comparative analysis of differences between groups. Median and quartile spacing were used for statistical analysis, and the rank-sum test was used for intergroup difference testing. Count data were analyzed by frequency statistics, and differences between groups were tested by the *χ*^2^ test.

Differences in protein screening conditions: FC > 1.2, *P*  < 0.05. The first step was to calculate the differential expression of proteins in the two samples from the groups being compared. First, we calculated the average quantitative values from analyses repeated many times for each sample, and then we calculated the ratio of the average calculated value between the two samples, which was the final difference in expression from the control group. The second step was to calculate the significance value of protein differential expression in the two samples. First, the relative quantitative evaluation of the data was carried out to make the data conform to a normal distribution, and then the *P* value was calculated by a Welch two-sample two-tailed *t*-test. When *P*  < 0.05, FC > 1.2 was the significantly upregulated change threshold, whereas FC < 1/1.2 was the significantly downregulated change threshold.

## 3. Results

### 3.1. Study Design and Patients

**Figure 1:**
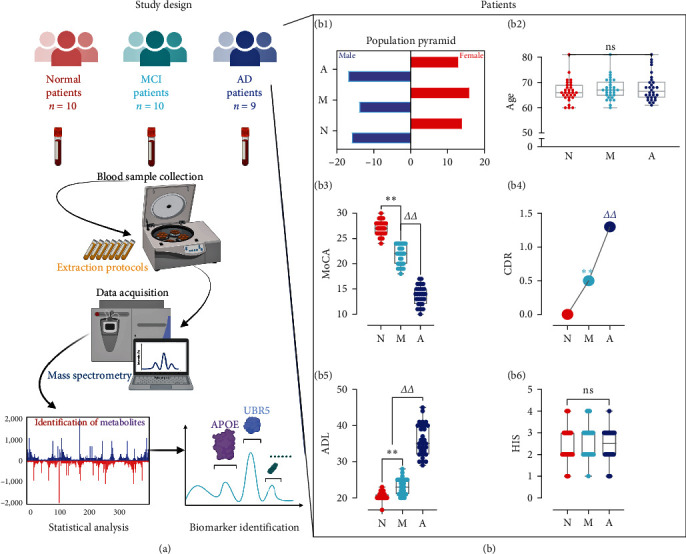
Study design and basic information about patients. The study design process is shown in (a), and the basic information of all patients included in the study is shown in (b): (b1) sex distribution; (b2) age distribution; (b3) MoCA scores; (b4) CDR scores; (b5) ADL scores; (b6) HIS scores. *n* = 30;  ^*∗∗*^*P*  < 0.01, compared with the normal cognition control group; ^*ΔΔ*^*P*  < 0.01, compared with the MCI group; ns, not significant; N, normal cognition control; M, mild cognitive impairment; A, Alzheimer's disease.

We collected blood samples from the cohort of study patients in the First Affiliated Hospital of Heilongjiang University of Chinese Medicine and carried out the study in strict accordance with the experimental protocol design approved by the Ethics Committee of the First Affiliated Hospital of Heilongjiang University of Chinese Medicine (Figure 1). Basic information was obtained from the patients enrolled in each study group according to the inclusion and exclusion criteria. This basic information included sex and age as well as the MoCA, CDR, ADL, and HIS scale scores, which were used for the diagnosis of MCI and AD (Figure 1(b1), 1(b2), 1(b3), 1(b4), 1(b5), and 1(b6)). There were no statistically significant differences in age or sex among groups (*P*  > 0.05). According to the MoCA, CDR, ADL, and HIS scale scores, significant differences were found between the MCI group and the normal cognition control group (*P*  < 0.01) and between the MCI group and the AD group (*P*  < 0.01). These findings suggested that with the progression of the disease, the cognitive function of the patients changed significantly. The collection of basic information involved in the study fully met the basic requirements of the ethics committee.

### 3.2. Plasma Proteomic Analysis

**Figure 2:**
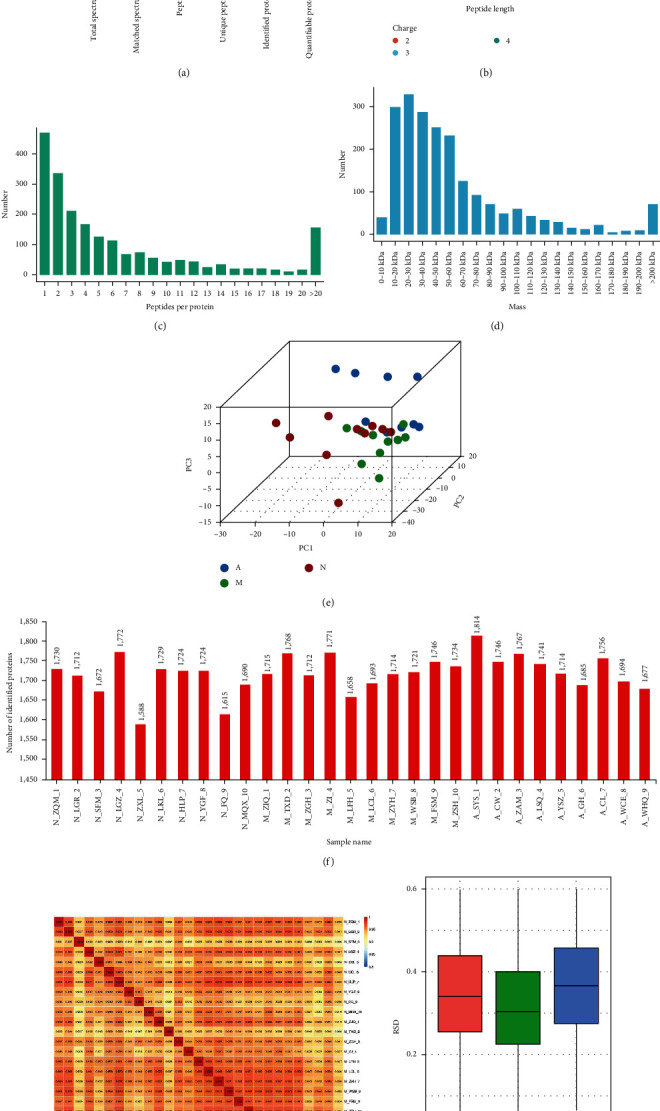
Plasma proteomic analysis of the patients in each group. (a) Number of proteomic spectrograms. (b) MS identification, indicating that the peptide length distribution meets the quality control requirements. (c) Quantitative peptide number distribution of proteins. (d) Molecular weight distribution of protein peptides. (e) Test results of PCA. (f) Quantitative analysis results of each group sample. (g) Test results of PCC. (h) Test results of RSD. PCC, Pearson's correlation coefficient; PCA, principal component analysis; and RSD, relative standard deviation. N, normal cognition control; M, mild cognitive impairment; A, Alzheimer's disease.

Plasma proteins in normal cognition control, MCI, and AD patients were identified by MS. A total of 2,030,350 spectrograms were detected, including 520,692 matched spectrograms. A total of 14,283 peptides were identified by spectral analysis, among which 12,941 were unique peptide segments (Figure 2(a)). SDS–PAGE Electrophoresis Results and Protein Concentration Measurement Results are available in *Supplementary 4*. To evaluate the reliability of the proteomic data, a series of quality control evaluations were carried out to ensure that the results met the standards. Most peptides were composed of 7–20 amino acids, which conforms to the general rule based on enzymatic hydrolysis and MS fragmentation mode. The distribution of peptide length identified by MS met the quality control requirements (Figure 2(b)). Most proteins were composed of more than two peptides. During quantification, one protein corresponded to multiple specific peptides (or corresponded to multiple spectrograms), which is beneficial to increase the accuracy and credibility of quantitative results (Figure 2(c)). The molecular weight of the identified protein was uniform at different stages (Figure 2(d)). PCA found that the quantitative results of biological or technical duplicates were statistically consistent (Figure 2(e)). Each patient sample yielded a protein count ranging from 1,588 to 1,814, with a total of 29 samples analyzed (Figure 2(f)). When assessing the correlation of protein abundance changes among the samples, we calculated PCC, a commonly used statistical metric for evaluating the linear relationship between two variables. PCC values were calculated for protein abundance across sample groups, and the resulting correlation coefficients are displayed (Figure 2(g)). Furthermore, we generated box plots based on the RSD of protein quantification values among replicates within each group. These plots demonstrated consistently low RSD values, indicating excellent quantification repeatability (Figure 2(h)). Based on these results, we have comprehensively characterized the plasma protein profiles of all study samples and confirmed the high reproducibility of our samples, making them suitable for further investigation. The results showed that there were high numbers of differentially expressed proteins in the plasma samples of each group of patients, suggesting that there may be a single biomarker or a combination of biomarkers in the proteomic data.

### 3.3. Power Analysis and Differential Protein Screening Results

First, we conducted a power analysis on the actual data from the proteomics project in this study, individually analyzing the power for each protein within the three comparison groups. This involved calculating effect sizes and statistical power for each protein within a comparison group, with the condition that each protein in each group had a sample size of 10 and did not contain any missing values (NA). Please refer to *Supplementary 5*, “Power_analysis_result.xlsx,” for detailed data tables.

Based on the performance of the real data with a sample size of 10 in each group, it appears that there are a sufficient number of proteins with power values exceeding 80% or even approaching 100%. This suggests a high degree of confidence in identifying proteins that exhibit differences between the two groups. These results indicate that even when the sample size in each group is 10, highly differentially expressed proteins can still be detected using the *T*-test method, demonstrating the effectiveness of the *T*-test approach.

**Figure 3:**
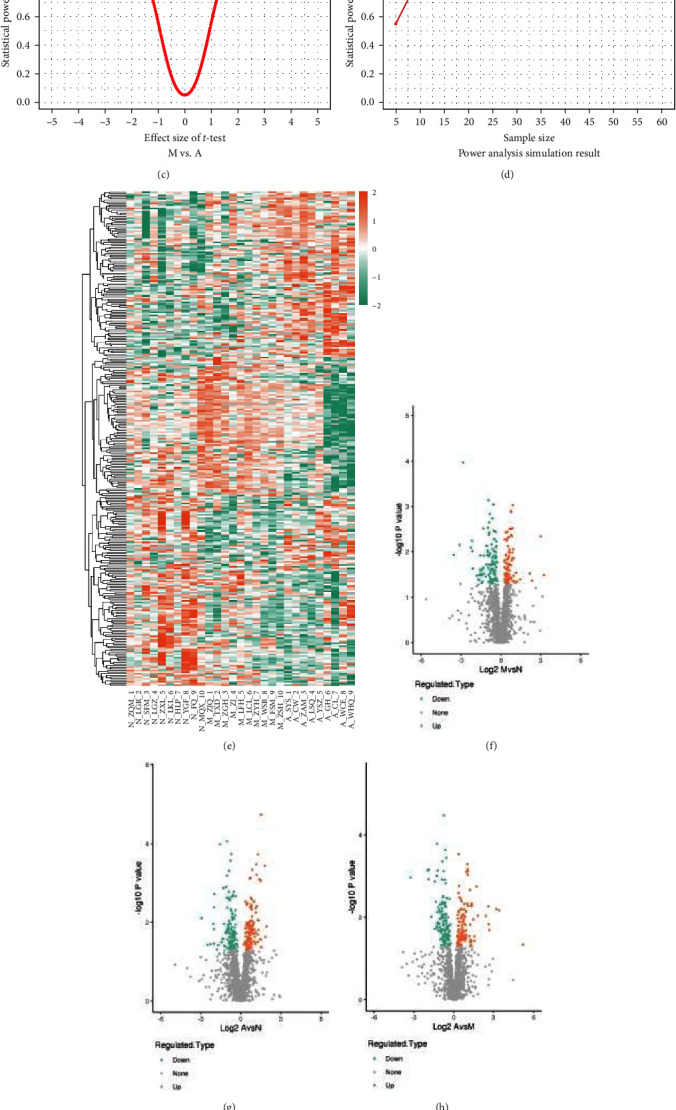
Power analysis and differential protein screening results. (a) Effect size vs. statistical power relationship plot for the N vs. M comparison group. (b) Effect size vs. statistical power relationship plot for the N vs. A comparison group. (c) Effect size vs. statistical power relationship plot for the M vs. A comparison group. (d) Power analysis simulation result. (e) Heatmap result. (f) Volcano plot results for the N vs. M comparison group. (g) Volcano plot results for the N vs. A comparison group. (h) Volcano plot results for the M vs. A comparison group. (i) Summary result of all differentially expressed proteins. N, normal cognition control; M, mild cognitive impairment; A, Alzheimer's disease.

Furthermore, we conducted power analysis through simulation to investigate the impact of sample size on statistical power, with a fixed effect size of 1.5. The experimental results are as follows: at an effect size of 1.5, with only 10 samples in each group, the power can reach 88.7%, which exceeds 80%. This suggests that choosing a sample size of 10 for each group is reasonable, as there is sufficient statistical power to detect differences between groups (Figure 3(a), 3(b), 3(c), and 3(d)). Please refer to *Supplementary 6*, “Power_analysis_simulation_result.xlsx,” for detailed data tables related to the simulation study.

In the end, we visualized the differences in protein abundance between different samples or groups using heatmaps and volcano plots. In the heatmap, differences in protein expression between different samples or groups are determined by comparing the shades of colors (Figure 3(e)).

In the volcano plot, each point represents a protein, allowing us to identify proteins with significant differential expression (Figure 3(f), 3(g), and 3(h)). In the final analysis, we discovered the following: in the N vs. M group, there were 36 upregulated proteins and 73 downregulated proteins. In the N vs. A group, there were 89 upregulated proteins and 96 downregulated proteins. In the M vs. A group, there were 71 upregulated proteins and 88 downregulated proteins (see Figure 3(i)).

### 3.4. Target Protein Screening by Mfuzz Analysis

**Figure 4:**
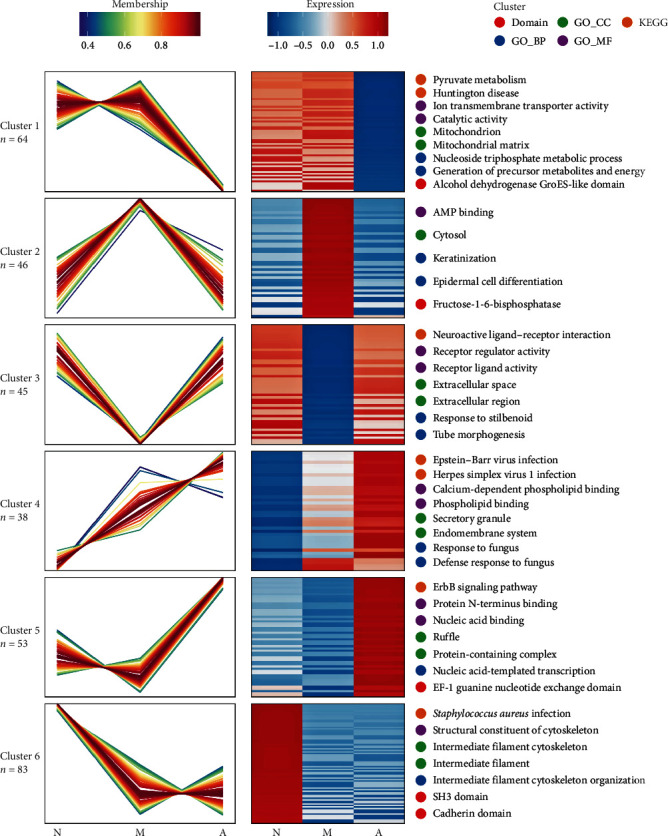
Mfuzz analysis of the differential protein expression of the three groups of patients. Red and blue represent high expression and low expression, respectively, and the darker the color is, the higher the degree of expression. N represents the normal cognition control group, M represents the MCI group, and A represents the AD group.

Based on the above most commonly used biological information sources for proteomic studies [16], we conducted gene expression profiling and pathway enrichment analysis for the differentially expressed genes and signaling pathways of MCI. Combined with relevant results, we found that the factors related to lipid metabolism for Mfuzz analysis should be addressed.  Figure 4 shows a protein heatmap, with the ordinates representing different proteins and the ordinates representing abbreviations for groups.

The protein functions of cluster 4 were related to the structure of the lipoprotein membrane. Cluster 4 has four enrichment functions that are highly related to cell membrane structure: calcium-dependent phospholipid binding, phospholipid binding, secretory granules, and the endomembrane system. Cluster 5 is closely related to gene expression, transcription, and protein synthesis, for example, protein N-terminus binding, nucleic acid binding, protein-containing complexes, and nucleic acid-templated transcription. According to Mfuzz analysis, the variation trend of different protein expression differences among groups can also be obtained. As biomarkers, the variation trend of protein expression at different stages of disease should be monotonic. That is, marker proteins with low expression in the normal group should be highly expressed in the AD group, and their expression showed an upward trend in the MCI group.

### 3.5. Protein Interaction Network Analysis

**Figure 5:**
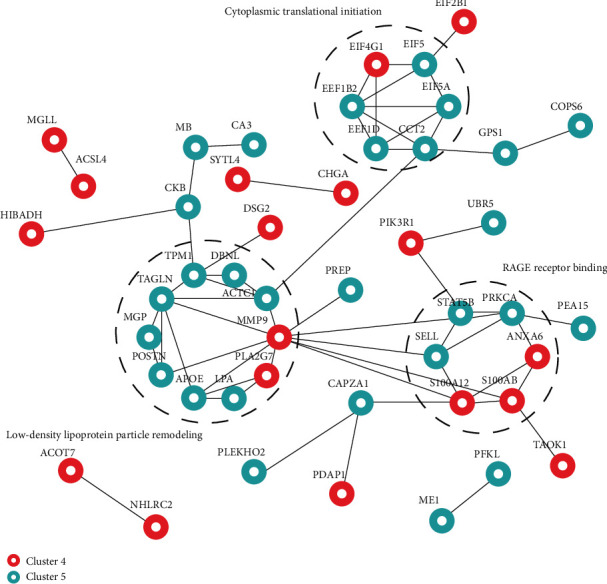
Results of protein interaction network analysis; the lines represent interactions, the red circles represent proteins in cluster 4, and the turquoise circles represent proteins in cluster 5.

We further analyzed the protein interaction networks of clusters 4 and 5. The proteins PLA2G7, S100A8, and MMP9 were derived from cluster 4, and the protein STAT5B was derived from cluster 5. In addition, we found that the expression of the proteins APOE and UBR5 showed an increasing trend in the normal cognition control group, MCI group, and AD group. Therefore, we added APOE and UBR5 to the protein interaction network for joint analysis and found that these two proteins were closely related to the proteins in cluster 4 and cluster 5. In addition, cytoplasmic translational initiation, RAGE receptor binding, and low-density lipoprotein remodeling are important potential biological processes changing from MCI to dementia. Most importantly, plasma APOE, MMP9, S100A8, UBR5 PLA2G7, and STAT5B play a potentially crucial role in the progression of cognitive disorders (Figure 5).

### 3.6. Validation of Biomarkers

To further validate the feasibility of the biomarkers screened in proteomics, we expanded the number of patients in each group to 30. Plasma levels of APOE, UBR5, MMP9, S100A8, PLA2G7, and STAT5B, according to ELISA, showed that the measured protein levels were increased in the MCI group compared with the normal cognition control group. The plasma protein levels in the AD group increased significantly.

**Figure 6:**
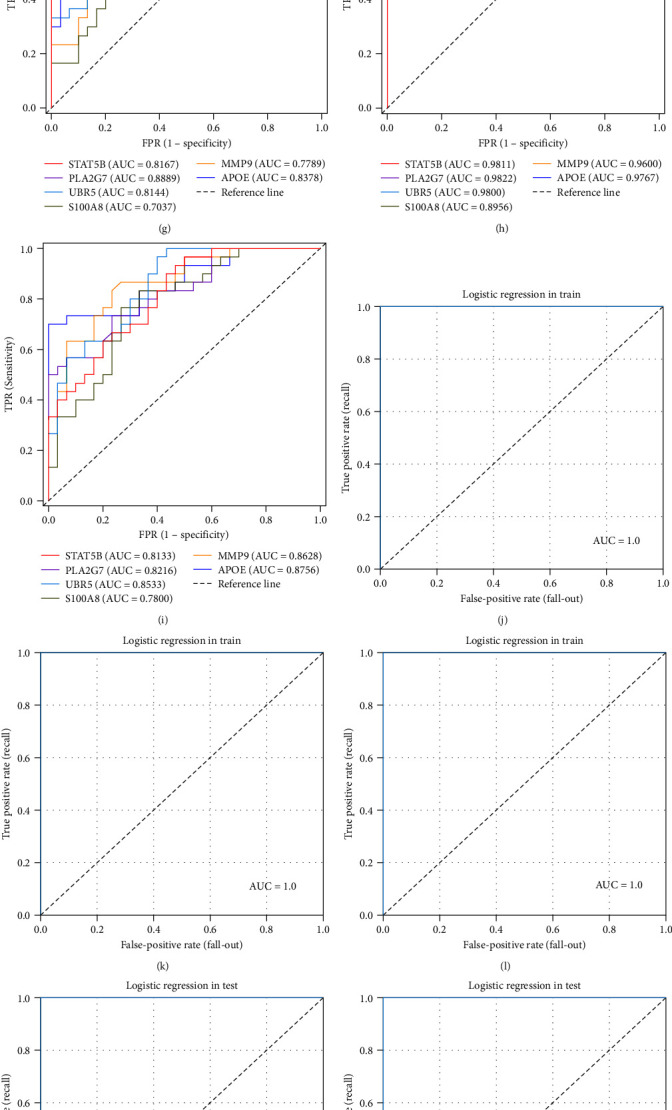
Serological validation of plasma biomarkers and the multiple-group analysis based on logistic regression. (a–f) Levels of the different biomarkers in plasma. (a–f) Correspond to APOE, MMP9, UBR5, PLA2G7, STAT5B, and S100A8, respectively. The bold dotted line in each box represents the median; the two thinner dotted lines on the top and bottom represent the values of 75% and 25%, respectively; the top and bottom represent the maximum and minimum values, respectively; and the difference in width represents the situation of sample aggregation. The wider the box is, the more samples that were gathered there. (g–i) ROC curves of different biomarkers. (g) Patients in the normal cognition control group and MCI group were identified using six different biomarkers. (h) Patients in the normal cognition control group and AD group were identified using six different biomarkers. (i) Patients in the MCI and AD groups were identified using six different biomarkers. The horizontal coordinate is the false-positive rate (FPR), and the vertical coordinate is the true-positive rate (TPR). The value AUC is the size of the area below the ROC curve. (j–o) Multiple-group analysis based on logistic regression. (j) Results for N vs. M on the training set. (k) Results for N vs. A on the training set. (l) Results for N vs. M on the testing set. (m) Results for N vs. M on the testing set. (n) Results for N vs. A on the testing set. (o) Results for N vs. M on the testing set. ^*∗*^*P*  < 0.05,  ^*∗∗∗*^*P*  < 0.001, compared with the normal cognition control group; ^###^*P*  < 0.001, compared with the MCI group; ns, not significant; N, normal cognition control; M, mild cognitive impairment; A, Alzheimer's disease.

All proteins showed statistically significant differences among the different groups (*P*  < 0.05; *P*  < 0.01; *P*  < 0.001). However, compared with the normal cognition control group, the elevation of S100A8 in the MCI group was more conservative than the elevations of the other five markers (*P*  < 0.05) (Figure 6(a), 6(b), 6(c), 6(d), 6(e), and 6(f)).

To further compare the differences among the selected biomarkers, we conducted receiver operating characteristic (ROC) prediction judgment analysis for distinguishing between two patient groups based on the results of the six biomarkers detected by ELISA (Figure 6(g), 6(h), and 6(i)). When distinguishing between the normal cognition control group and the MCI group, the six biomarkers, in the order of the highest to lowest area under the ROC curve (AUC) values, were PLA2G7 (AUC = 0.8896), UBR5 (AUC = 0.8112), APOE (AUC = 0.8096), STAT5B (AUC = 0.7712), MMP9 (AUC = 0.7504), and S100A8 (AUC = 0.6432) (Figure 6(g)). When distinguishing between the normal cognition control group and the AD group, the six biomarkers, in the order of the highest to lowest AUC values, were UBR5 (AUC = 0.9776), PLA2G7 (AUC = 0.9744), STAT5B (AUC = 0.9744), APOE (AUC = 0.9664), MMP9 (AUC = 0.9568), and S100A8 (AUC = 0.8512) (Figure 5(h)). When distinguishing between the MCI group and the AD group, the six biomarkers, in the order of the highest to lowest AUC values, were MMP9 (AUC = 0.8680), UBR5 (AUC = 0.8592), APOE (AUC = 0.8496), STAT5B (AUC = 0.8272), S100A8 (AUC = 0.7856) and PLA2G7 (AUC = 0.7800) (Figure 6(i)). The AUC values for each marker in each group were between 0.5 and 1, indicating that these six markers showed good clinical diagnostic performance.

### 3.7. Biological Markers and Participant Scores' Results in Multiple-Group

We separately calculated the accuracy, ROC curves, and AUC values of the model on the training and testing datasets. The results indicated that all three classification models (N vs. A, M vs. A, N vs. M) achieved an accuracy and AUC value of 1, enabling them to completely and correctly differentiate between different groups (Figure 6(j), 6(k), 6(l), 6(m), 6(n), and 6(o)). Additionally, we derived the logistic regression equation for comparing two groups as follows:

1. N vs. A

(7) $$\begin{array}{c} y=0.3630212726297349\times \text{ APOE }+0.503837278589717\times \text{ UBR}5+0.45480591796503533\times \text{ MMP}9+0.1283158996025137\times \text{S}100A8+0.2883415540718194\times \text{ STAT}5B+0.3709678607850625\times \text{ PLA}2G7+0.6783376714393615\times \text{ CDR }+-0.9097053097540923\times \text{ MoCA }+0.676209124052747\times \text{ ADL }+0.11146359143518329\times \text{ HIS }+0.2891868688116039. \end{array}$$

  (2) M vs. A

(8) $$\begin{array}{c} y=0.26246573735459194\times \text{ APOE }+0.3506063771419758\times \text{ UBR}5+0.4803959904062068\times \text{ MMP}9+-0.0028488379192457155\times \text{S}100A8+0.17682273291933967\times \text{ STAT}5B+0.08672123471665183\times \text{PLA}2G7+0.7517438825017654\times \text{ CDR }+-1.1615725126015188\times \text{ MoCA }+0.9360695574690933\times \text{ ADL }+-0.08734839499641048\times \text{ HIS }+0.2237026159877827. \end{array}$$

  (3) N vs. M

(9) $$\begin{array}{c} y=0.3021360272736927\times \text{ APOE }+0.2521693867082287\times \text{ UBR}5+0.3267493349482505\times \text{ MMP}9+-0.031002765088110354\times \text{S}100A8+0.04775536548813992\times \text{ STAT}5B+0.4606650763906919\times \text{PLA}2G7+1.6059593104806753\times \text{ CDR }+-0.9833403308009726\times \text{ MoCA }+0.4445285285315958\times \text{ ADL }+0.19724010957261218\times \text{ HIS }+0.3713351800237155. \end{array}$$

## 4. Discussion

Research on blood markers of cognitive disorders, such as dementia, is a popular topic in the field of psychiatry and is the key to the diagnosis and treatment of these disorders. At present, the existing methods used in clinical practice for the diagnosis of MCI have some limitations. Clinically, the diagnostic methods of cognitive disorders mainly include the MoCA, ADL, CDR, HIS, and other scales [17, 18]; magnetic resonance imaging (MRI) [19, 20]; and ^18^F-labeled deoxyglucose PET (^18^FDG-PET) technology [21]. These methods have provided some help in the diagnosis and judgment of cognitive disorders, especially AD, but their limitations are relatively numerous, as shown in the following: (1) These methods are essentially not effective until patients have advanced to the middle and late stages of AD and cannot be used to prevent the underlying MCI stage or to diagnose abnormalities in neuronal metabolic activity at an earlier stage. (2) The MRI and PET equipment involved in imaging diagnosis is large in size and high in cost, and MRI and PET require high data analysis ability of researchers. The MRI and PET experimental results involved in imaging diagnosis are subject to large transnational or transregional fluctuations due to different instrument brands, parameter settings, data analysis, and other factors. In addition, the accuracy of various clinical scales, including the MoCA, ADL, CDR, HIS, and so on, can be affected by the temporary performance of patients and subjective factors of testers. (4) Detection without the use of biomarkers cannot distinguish AD from dementia caused by other causes. In contrast, biomarkers have attracted much attention in recent years due to their ability to diagnose disorders in early stages, low cost, and high sensitivity.

How to combine cutting-edge science and technology with existing clinical diagnostic testing methods to solve psychiatric problems related to dementia is a very urgent problem to be solved. For patients with cognitive decline, CSF and brain biopsy collection are invasive and costly, which, to some extent, hinders the identification of patients in the presymptomatic stage and cognitively normal-MCI stage. Blood samples for the analysis of multiple candidate biomarkers are easier to obtain; therefore, the development prospects of biomarkers may be broader. However, the detection of blood biomarkers is also challenging. First, the existence of the blood–brain barrier (BBB) makes the concentration of brain-derived biomarkers in the blood low, which requires higher detection sensitivity [22, 23]. Second, some biomarkers related to AD pathology are also expressed in the periphery [24, 25], which may interfere with the detection results. Moreover, the protein content in blood samples is relatively difficult to detect by general proteomics based on MS [26], which largely restricts the types of blood markers reported by current researchers in the premorbid stage of AD, the MCI stage.

In this study, our research team used cutting-edge 4D label-free deep quantitative proteomic analysis to obtain proteins with a higher accuracy than ordinary proteomic analysis. A total of 2,067 proteins were identified, among which 1,986 proteins were quantified. This is the highest number identified in plasma proteomics currently known in the field of dementia. This technology has greatly solved the bottleneck of common proteomics technology based on MS in the study of disease mechanisms and the discovery of new blood biomarkers and has laid an innovative foundation for the auxiliary diagnosis of blood markers for cognitive disorders. Our results reveal the clinical changes in the proteome in cognitively healthy participants and MCI and AD patients and suggest that lipid metabolism and ubiquitin modification may be important in the pathogenesis of the progression of cognitive impairment, in which biological processes mainly involve cytoplasmic translational initiation, RAGE receptor binding and low-density lipoprotein remodeling. This study reports the current mechanism of AD and reveals that a total of 6 markers, PLA2G7, UBR5, APOE, MMP9, STAT5B, and S100A8, can cross the BBB into the peripheral blood. These markers, although not found at high levels in the peripheral blood of healthy persons, can be stably detected by routine ELISA.

**Figure 7:**
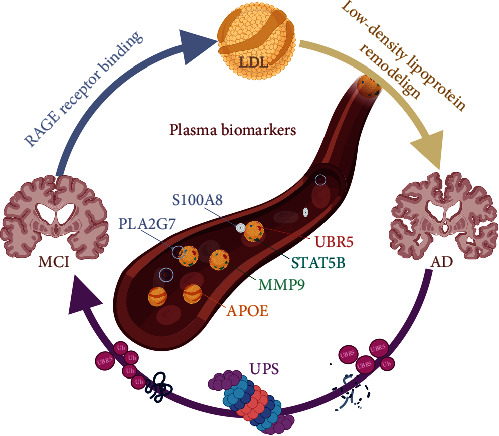
Key biomarkers in the pathogenesis from MCI to AD; the crucial involvement of six significant biomarkers (APOE, PLA2G7, UBR5, MMP9, STAT5B, and S100A8) in the pathogenesis of Alzheimer's disease (AD) from mild cognitive impairment (MCI). It emphasizes the roles of three main pathogenic processes: (1) activation of RAGE receptors, highlighting the impact of neuroinflammation and neuronal damage; (2) LDL (low-density lipoprotein) remodeling, pointing to its role in amyloid-beta accumulation and plaque formation; and (3) the UBR5-mediated ubiquitination cascade alongside proteasomal degradation, underlining the ubiquitin-proteasome system (UPS)'s crucial function in maintaining protein quality control. These processes signify pivotal biological changes that occur as the disease progresses from MCI to dementia, suggesting that these biomarkers and their associated pathways offer potential targets for therapeutic intervention.

In this study, six markers found in 4D label-free deep proteomic analysis were verified by ELISA, and the samples were amplified to 30 cases in each group. The blood levels of these six specific proteins were found to be significantly higher in the MCI and AD groups than in the normal cognition control group. This finding also suggests that PLA2G7, UBR5, APOE, MMP9, STAT5B, and S100A8 are predictive of MCI in the early diagnosis of disease markers and that the level of cognitive impairment can be predicted to a certain extent (Figure 7). These findings add to the ongoing work in the field of clinical dementia. In 2018, the NIA-AA published three biomarkers for AD, i.e., A*β* accumulation-related biomarkers (A), Tau accumulation-related biomarkers (T), and neurodegeneration (N)-related biomarkers or other biomarkers of specific pathological processes, referred to as ATN.

APOE, short for apolipoprotein E, is a widely recognized biomarker for AD risk prediction [23]. APOE promotes A*β* degradation to neurotoxic fragments, including A*β* oligomeric and fibrous states, and the amount of A*β*42 depends on the presence of the APOE*ε*4 allele, with the highest concentration of A*β*42 found in patients homozygous for the APOE*ε*4 allele [27]. In addition, the APOE*ε*4 allele interferes with the clearance of A*β* in the brain by affecting the integrity of the BBB [28]. Epidemiological data showed that the risk of AD in APOE*ε*4 homozygous individuals was more than 50%, and the incidence of AD in the APOE*ε*4 heterozygous APOE*ε*3 population was approximately 20%–30% [29]. In normal cognition, elderly APOE*ε*4 carriers, the degree of ventricular dilation was positively correlated with the decrease in CSF A*β*42. In patients with APOE*ε*4-positive AD, ventricular dilation was associated with CSF Tau content [30]. Previous studies have suggested that combining APOE analysis with core CSF biomarker analysis can improve the accuracy of the clinical diagnosis of AD. Our study also found that plasma APOE levels in patients with cognitive impairment were of diagnostic significance, and the ELISA detection method we used is more convenient and easier for patients and medical staff to accept.

PLA2G7, short for phospholipase A2 group VII, is a member of the lipoprotein-associated phospholipase (LP-PLA2) family, which plays an important role in lipid metabolism [31]. Davidson et al.'s [32] study found that the LP-PLA2 level of MCI patients was higher than that of healthy controls, and the higher the level of LP-PLA2 was, the more severe the degree of cognitive impairment. Yin et al. [33] study found that LP-PLA2 was associated with the APOE*ε*4 gene, and individuals carrying the APOE*ε*4 gene were more likely to have elevated LP-PLA2. In addition, LP-PLA2 is involved in inflammatory and lipid metabolism processes, and it mediates the biological activity of phospholipid substrates, thereby exacerbating inflammatory and oxidative stress responses [34]. Our results showed that PLA2G7 levels were significantly higher in the MCI and AD groups than in the normal cognition control group, which is consistent with previous studies. The APOE*ε*4 gene is one of the important potential risk factors for MCI, which indirectly demonstrates the association between PLA2G7 and MCI; therefore, PLA2G7 has the potential to be an important biomarker for the diagnosis of MCI and AD.

UBR5 is a member of the E3 ubiquitin ligase family associated with ubiquitination. It has been confirmed by a genome-wide association study that UBR5 is an important biomarker in the progression of AD [35]. Over time, UBR5 levels changed significantly over the course of the disease. In addition, there is an association between A*β* abnormalities and ubiquitin–proteasome system dysfunction in AD [36]. Early in the disease, hippocampal proteasome activity is reduced, and protein aggregation reduces proteasome function, most likely due to Tau and A*β* oligomerization. The expression of the ubiquitin-binding enzyme E2K (E2-25K/HIP-2) is upregulated in A*β*-induced neurons and has been shown to inhibit proteasome function through its association with the mutant ubiquitin, ubiquitin B + 1 (UBB + 1) [37]. The results of this study also showed that the UBR5 level in the MCI group was significantly higher than that in the normal cognition control group. The level of UBR5 in the AD group was significantly higher than that in the MCI group. However, the mechanism of action between UBR5 dysfunction and cognitive impairment remains to be further studied.

The fact that matrix metallopeptidase 9 (MMP9) levels are significantly elevated in the course of many neurological diseases has been demonstrated repeatedly, and postmortem studies have observed higher levels of MMP9 in various brain tissues in patients with AD than in cognitively healthy patients, for example, in cytoplasmic neurofibrillary tangles, amyloid plaques, and blood vessel walls in the hippocampus and cerebral cortex [38]. The inhibition of MMP9 promotes A*β* passage through the BBB, thereby promoting A*β* transport, and eliminating MMP9 regulation alters brain A*β* levels by promoting lipoprotein receptor passage through the BBB [39]. High levels of MMP9 degrade the base proteins in blood vessels, leading to the destruction of the BBB and brain damage. On the other hand, the injection of A*β* increases MMP9 expression, and this increase has been associated with the development of cognitive impairment and neurotoxicity [40]. Our work also found that plasma MMP9 levels were significantly increased in the MCI and AD groups compared to the normal cognition control group, and the level of MMP9 in the AD group was higher than that in the MCI group. Therefore, MMP9 is closely correlated with the progression of AD and can be used as a biomarker for diagnosis and treatment.

STAT5B is short for signal transducer and activator of transcription 5B. STAT proteins are major proteins involved in the regulation of synaptic plasticity, neuroprotection, and cognitive function. A recent continuous cohort study [41] found that STAT5B may serve as a tumor-associated molecular determinant of neurocognitive deficits in patients with diffuse glioma. Cognitive performance may be affected mainly through mechanisms such as affecting neuronal communication. Previous studies [42] have also found that mice without STAT5 genes show obvious memory deficits. STAT5 affects cognitive processes by inhibiting the expression of its target gene, IGF-1, suggesting that STAT5 plays an important role in learning and memory. In this study, ELISA verified that STAT5B levels were increased significantly in the MCI group and AD group compared with the normal cognition control group, consistent with the trends observed in previous studies, suggesting that STAT5 plays an important role in the progression of cognitive disorders, and its blood level can be used as a potential biomarker for diagnosis and treatment.

S100A8 refers to migration inhibitory factor-related protein-8, also known as MRP8. S100A8 has been shown to activate the MAP kinase [43] and NF-*κ*B [44] signaling pathways and has been shown to mediate the toxic and proinflammatory effects of A*β* [45]. S100A8 is a calcium-regulating inhibitor of A*β*42 aggregation. Increased levels of S100A8 promote the formation of A*β* plaques, which in turn aggravate neuroinflammation [46]. S100A8 alters APP processing, increases *β*-secretase activity, and leads to more A*β* peptide production. Positive feedback between S100A8 and A*β*42 has been observed, and the increase in S100A8 levels was closely related to hippocampal A*β* deposition, which may, in turn, be a promoter of brain A*β* deposition [45]. Our experimental results also showed that S100A8 is the main disease-causing gene of MCI and AD, and S100A8 has high connectivity in the protein–protein interaction network of MCI and AD. The level of S100A8 is significantly different in different stages of disease and can be used in the diagnosis of cognitive impairment.

**Figure 8:**
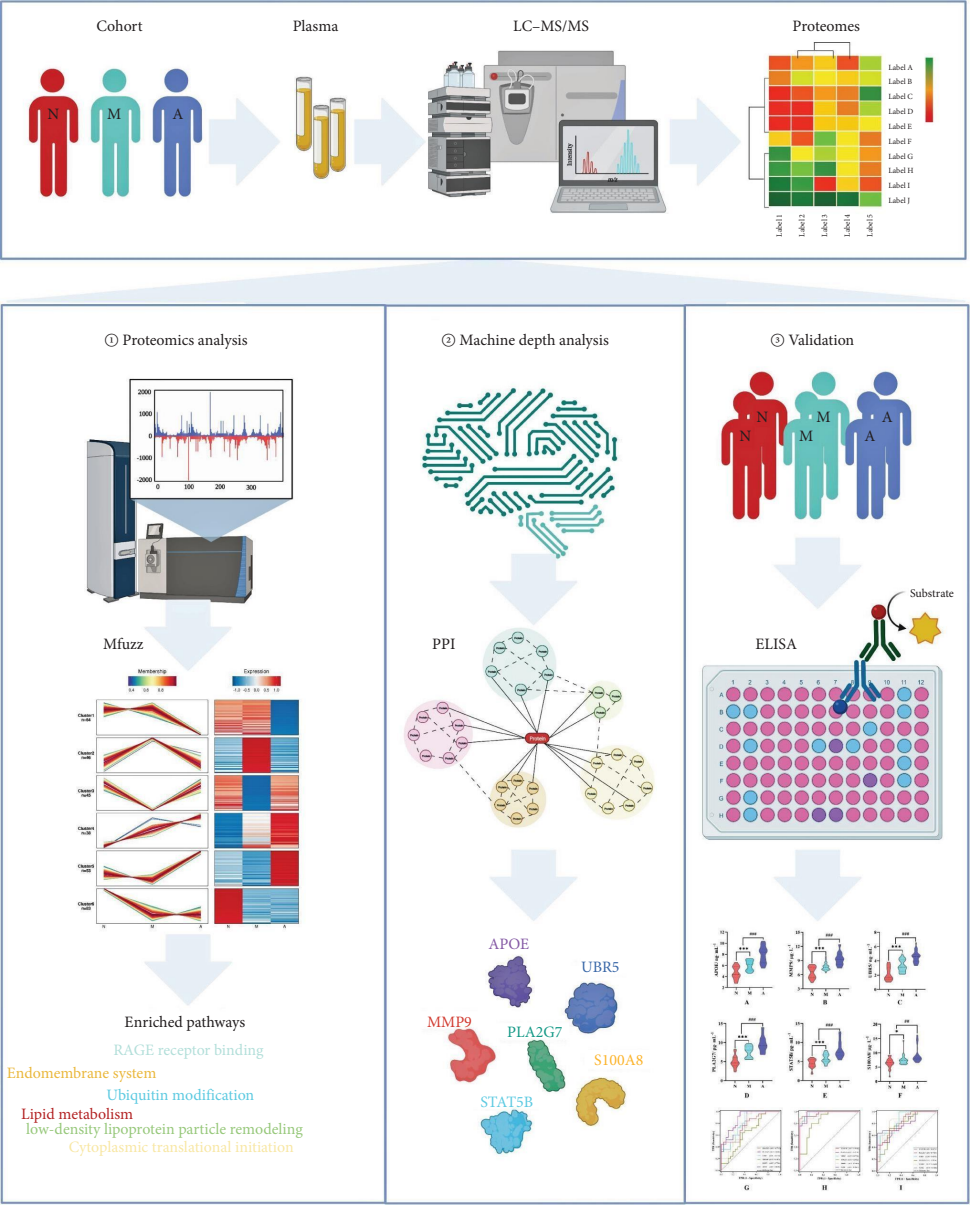
The paradigm and results of this study.

This study presents a novel exploration into the pathogenesis of MCI, particularly focusing on the roles of lipid metabolism and ubiquitination modification as significant precursors to AD. Our findings introduce six proteins (PLA2G7, UBR5, APOE, MMP9, STAT5B, and S100A8) as highly sensitive predictive markers. These markers hold promise for identifying the transition from normal cognitive function to MCI and subsequently to AD. By determining critical detection time points, these biomarkers could significantly enhance early clinical diagnosis and the initiation of effective treatments for AD (Figure 8).

Our years of dedicated research in cognitive impairment have led us to propose criteria for the ideal MCI biomarker: it should reflect MCI's basic pathological features, be detected through minimally invasive methods, yield easily interpretable results, be cost-effective, and possess high specificity and sensitivity. Moreover, an ideal biomarker would indicate the optimal timing for intervention and reflect the efficacy of subsequent treatments. While the search for such comprehensive biomarkers continues globally, our study contributes to this endeavor by highlighting potential candidates.

### 4.1. Limitations and Future Research Directions

Acknowledging the limitations highlighted in this study, including the study's small sample size and cross-sectional design, future research should aim for larger, more diverse, and longitudinal studies. These studies would better establish the causality and predictive value of identified biomarkers across different populations and stages of cognitive decline. Expanding the range of validation techniques beyond ELISA and incorporating advanced diagnostic comparisons, such as with MRI or 18FDG-PET technology, will also be crucial.

## 5. Conclusion

In summary, our investigation underscores the emerging significance of lipid metabolism and ubiquitin modification in the progression of cognitive disorders. The plasma levels of PLA2G7, UBR5, APOE, MMP9, STAT5B, and S100A8 emerge as pivotal biomarkers for predicting the evolution from normal cognition to MCI and further degradation to AD. These findings offer a valuable diagnostic reference and pave the way for proactive disease management strategies.

## Data Availability

Raw data are available upon reasonable request from the corresponding author.

## Supplementary Materials

Supplementary 1 Demographic Characteristics of Study Participants by Group.

Supplementary 2 Petersen's MCI Diagnostic Criteria and The NIA-AA Diagnostic Criteria for Alzheimer's Disease.

Supplementary 3 Matlab Program Language File.

Supplementary 4 SDS-PAGE Electrophoresis Results.

Supplementary 5 Power Analysis Result.

Supplementary 6 Power Analysis Simulation Result.
