# Supplementary material for: What Are the Reliable Plasma Biomarkers for Mild Cognitive Impairment? A Clinical 4D Proteomics Study and Validation
Source: Mediators Inflamm. 2024 May 27;2024:7709277. doi: 10.1155/2024/7709277 (PMC11178428; doi:10.1155/2024/7709277)
Supplement: Supplementary 1 — Demographic Characteristics of Study Participants by Group. [file 7709277.f1.docx]

Supplementary Table 1: Demographic Characteristics of Study Participants by Group

| **Demographic** | **Total Participants (N=90)** | **Normal Group(n=30)** | **MCI Group (n=30)** | **AD Group (n=30)** |
| --- | --- | --- | --- | --- |
| **Age (years)** |  | | | |
| **Mean ± SD** | 67.43 ± 5.07 | 66.73 ± 4.835 | 67.43 ± 4.392 | 68.13 ± 5.935 |
| **Minimum and maximum, Range** | 60~87(27) | 60~85(25) | 60~82(22) | 61~87(26) |
| **Gender** |  | | | |
| **Male** | 57 (60%) | 18 (60%) | 20 (60%) | 19 (60%) |
| **Female** | 33 (40%) | 12 (40%) | 10 (40%) | 11 (40%) |
| **Comorbidities** |  | | | |
| **Hypertension** | 23 (25.56%) | 6 (20%) | 8 (26.7%) | 9 (30%) |
| **Diabetes** | 14 (15.56%) | 5 (16.67 %) | 4 (13.3%) | 5 (16.67 %) |
| **Hyperlipidemia** | 9 (10%) | 4 (13.33%) | 2 (6.7%) | 3 (10%) |
| **None** | 44 (48.88%) | 15 (50%) | 16 (53.3%) | 13 (43.33%) |

Age (years): Mean ± Standard Deviation (SD) and the range (minimum-maximum) of participants' ages are provided to illustrate the age distribution across Normal, MCI, and AD groups.

Gender: The distribution of participants by gender (male, female) is expressed as a count and percentage of the total participants in each group, indicating the gender balance within the study population.

Comorbidities: This section details the prevalence of specific comorbid conditions (hypertension, diabetes, hyperlipidemia) within each group, presented as a count and percentage of participants. A category for participants without any listed comorbidities is also included to reflect the overall health profile of the study population.

The total number of participants (N=90) is divided equally among the Normal, MCI, and AD groups to facilitate direct comparisons across groups.

Percentages have been calculated based on the total number of participants in each group, providing insights into the distribution of demographic characteristics and comorbid conditions.

This table aims to enhance the understanding of the study's demographic landscape, addressing potential confounding factors and supporting the external validity of the research findings.
