# Supplementary material for: What Are the Reliable Plasma Biomarkers for Mild Cognitive Impairment? A Clinical 4D Proteomics Study and Validation
Source: Mediators Inflamm. 2024 May 27;2024:7709277. doi: 10.1155/2024/7709277 (PMC11178428; doi:10.1155/2024/7709277)
Supplement: Supplementary 2 — Petersen's MCI Diagnostic Criteria and The NIA-AA Diagnostic Criteria for Alzheimer's Disease. [file 7709277.f2.pdf]

1. **Petersen's MCI diagnostic criteria:**

The Petersen's MCI diagnostic criteria include the following:

a) Subjective report of memory impairment by the patient or informant.

b) Objective evidence of memory impairment on neuropsychological testing.

c) Normal general cognitive function, which means that the patient should not meet the criteria for dementia.

d) Intact activities of daily living.

e) Absence of dementia.

In addition, the criteria require that the cognitive impairment is not due to other factors such as medication, depression, or other medical conditions.

2. **The NIA-AA diagnostic criteria for Alzheimer's disease:**

The NIA-AA diagnostic criteria for Alzheimer's disease include the following:

a) Presence of beta-amyloid plaques and tau protein in the brain, which can be detected using imaging or biomarker tests.

b) Evidence of cognitive impairment, such as problems with memory, language, or spatial orientation, as assessed by neuropsychological testing.

c) Functional impairment, which means that the cognitive impairment interferes with the patient's ability to perform activities of daily living.

The criteria also include three stages of the disease: preclinical Alzheimer's disease, mild cognitive impairment due to Alzheimer's disease, and Alzheimer's dementia. The preclinical stage refers to individuals who have beta-amyloid plaques and/or tau protein in the brain but no cognitive impairment or functional impairment. The mild cognitive impairment stage refers to individuals who have beta-amyloid plaques and/or tau protein in the brain and evidence of cognitive impairment but no significant functional impairment. The Alzheimer's dementia stage refers to individuals who have beta-amyloid plaques and/or tau protein in the brain, evidence of cognitive impairment, and significant functional impairment.
