# Supplementary material for: What Are the Reliable Plasma Biomarkers for Mild Cognitive Impairment? A Clinical 4D Proteomics Study and Validation
Source: Mediators Inflamm. 2024 May 27;2024:7709277. doi: 10.1155/2024/7709277 (PMC11178428; doi:10.1155/2024/7709277)
Supplement: Supplementary 3 — Matlab Program Language File. [file 7709277.f3.pdf]

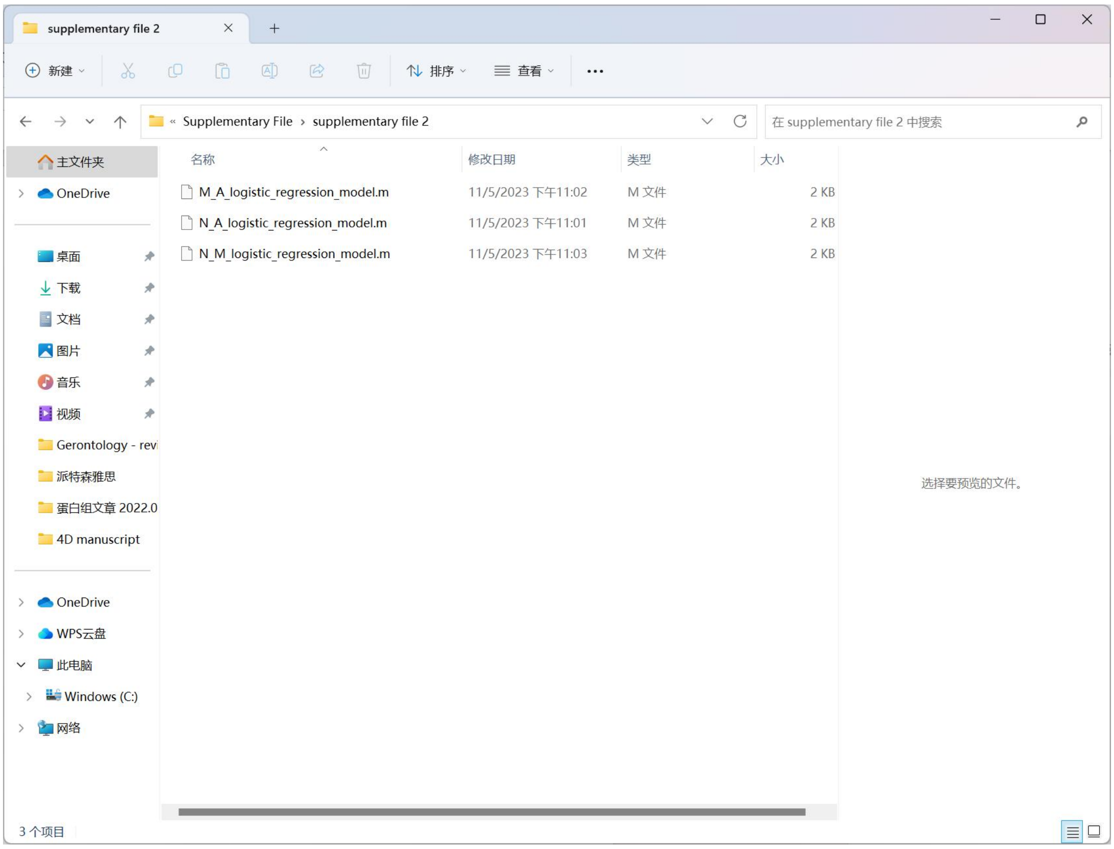

Supplementary file 2 is a Matlab program language file and cannot be uploaded to the Hindawi system, so please refer to the email attachment of 18745020244@139.com. We have submitted it to the email attachment, please check!
