# Supplementary material for: What Are the Reliable Plasma Biomarkers for Mild Cognitive Impairment? A Clinical 4D Proteomics Study and Validation
Source: Mediators Inflamm. 2024 May 27;2024:7709277. doi: 10.1155/2024/7709277 (PMC11178428; doi:10.1155/2024/7709277)
Supplement: Supplementary 4 — SDS-PAGE Electrophoresis Results. [file 7709277.f4.pdf]

SDS-PAGE Electrophoresis Results

To assess the efficiency of total protein extraction after removing high-abundance plasma proteins in samples from the three groups, SDS-PAGE electrophoresis was performed to analyze the protein band patterns. The results demonstrated clear and well-distributed protein bands with no degradation. Good parallelism was observed within each group, and there were no significant differences in electrophoretic behavior between the groups. The total protein content was found to be sufficient for the proteomic analysis conducted in this study (Fig 1).

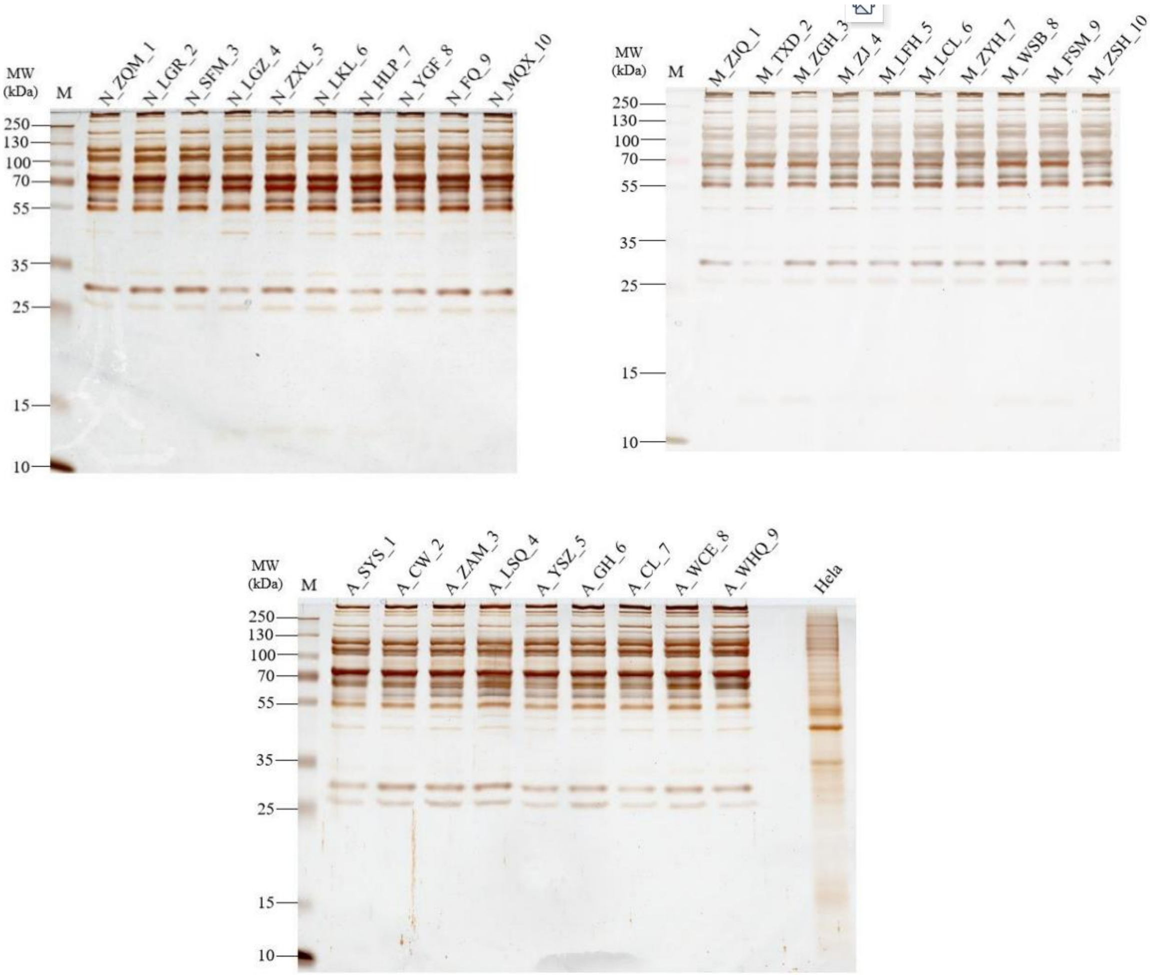

Figure 1.SDS-PAGE after Removal of High Abundance Proteins

Protein Concentration Measurement Results

Subsequently, the protein concentrations were further determined for a total of 29 samples from the N, M, and A groups after removing high-abundance proteins. The results indicated that the protein concentrations and total protein amounts in all samples were adequate for the proteomic analysis in this study (Table 1).

Table 1. Determination Results of Protein Concentration after Removing High Abundance

Sample ID	Protein Concentration (μg/μL)	Sample Volume (μL)	Total Protein Amount (μg)
N_ZQM_1	0.20	200.0	40.0
N_LGR_2	0.20	200.0	40.0
N_SFM_3	0.20	200.0	40.0
N_LGZ_4	0.20	200.0	40.0
N_ZXL_5	0.20	200.0	40.0
N_LKL_6	0.20	200.0	40.0
M_ZYH_7	0.20	200.0	40.0
N_YGF_8	0.20	200.0	40.0
N_FQ_9	0.20	200.0	40.0
N_MQX_10	0.20	200.0	40.0
M_ZJQ_1	0.20	200.0	40.0
M_TXD_2	0.20	200.0	40.0
M_ZGH_3	0.20	200.0	40.0
M_ZJ_4	0.20	200.0	40.0
M_LFH_5	0.20	200.0	40.0
M_LCL_6	0.20	200.0	40.0
M_ZYH_7	0.20	200.0	40.0
M_WSB_8	0.20	200.0	40.0
M_FSM_9	0.20	200.0	40.0
M_ZSH_10	0.20	200.0	40.0
A_SYS_1	0.20	200.0	40.0
A_CW_2	0.20	200.0	40.0
A_ZAM_3	0.20	200.0	40.0
A_LSQ_4	0.20	200.0	40.0
A_YSZ_5	0.20	200.0	40.0
A_GH_6	0.20	200.0	40.0
A_CL_7	0.20	200.0	40.0
A_WCE_8	0.20	200.0	40.0
A_WHQ_9	0.20	200.0	40.0
