# Supplementary material for: What Are the Reliable Plasma Biomarkers for Mild Cognitive Impairment? A Clinical 4D Proteomics Study and Validation
Source: Mediators Inflamm. 2024 May 27;2024:7709277. doi: 10.1155/2024/7709277 (PMC11178428; doi:10.1155/2024/7709277)
Supplement: Supplementary 5 — Power Analysis Result. [file 7709277.f5.pdf]

Protein accession	Protein description	Gene name	Coverage [%]	Peptides
Q00266	S-adenosylmethion	MAT1A	2	1
Q8TDZ2	[F-actin]-monooxyg	MICAL1	1	1
P18669	Phosphoglycerate n	PGAM1	42	9
O43242	26S proteasome no	PSMD3	3	2
Q6AHZ8	Beta-ureidopropion	UPB1	10	1
A6XMV8	Protease serine 2 p	PRSS2	16	5
A0A7I2V4E4	Eukaryotic translati	EIF4H	17	3
H0YLU7	Electron transfer fla	ETFA	4	1
G3V2W1	Protein Z-depender	SERPINA10	26	14
H7C0V9	Amyloid-beta A4 pr	APP	18	8
B7Z7P8	Eukaryotic peptide	ETF1	9	4
B4E1Z4	C3/C5 convertase C	--	45	85
F5GWI4	Adenosine deamina	ADA	2	1
Q96L35	Receptor protein-ty	EPHB4	2	2
B4E1E0	CTP synthase OS=H	CTPS1	5	2
A0A4W9A917	Immunoglobulin he	IGHG3	45	22
A0A024R6I7	Alpha-1-antitrypsin	SERPINA1	54	39
Q5T4U5	Medium-chain spec	ACADM	3	1
A7XZE4	Beta tropomyosin is	TPM2	32	11
Q5VY30	Retinol-binding pro	RBP4	68	14
A0A1W2PSE7	HLA class I histocon	HLA-A	33	10
Q86X91	Inactive tyrosine-pr	PTK7	8	3
G3V3A0	Alpha-1-antichymot	SERPINA3	75	29
B4DPQ0	Complement subco	C1R	48	34
H0YLE3	Protein phosphatas	PPP1R3E	41	1
H0Y8P3	LIM and calponin h	LIMCH1	1	1
A0A0G2JL54	C4a anaphylatoxin (	C4B_2	56	119
Q5R349	p-selectin (Fragmer	SELP	22	13
F5H1T9	Keratin-associated		KRTAP2-1	21	2
A0A3B3ITQ1	Inositol 1,4,5-trisph	ITPR1	0	1
V9GYM3	Apolipoprotein A-II	APOA2	32	12
M0QXI5	Dipeptidyl peptidas	DPP9	4	1
A0A3B3ITN0	Exopolyphosphatas	PRUNE1	5	2
C9J6H2	Insulin-like growth	IGFBP1	6	1
H0YDV5	Myc target protein	MYCT1	12	2
E9PCY7	Heterogeneous nuc	HNRNPH1	4	1
A0A1B0GVN9	Uroporphyrinogen	UROD	15	5
Q53Z42	HLA class I antigen (	HLA-A	39	12
A0A2R8YEL6	Glutamate--cystein	GCLC	7	3
K7EQM4	Eukaryotic translati	EIF3K	8	1
A0A7P0TBI1	Anoctamin-6 OS=H	ANO6	3	3
A0A2R8YDI2	Myosin regulatory l	MYL10	10	2
A0A3B3IS80	Fructose-bisphosph	ALDOB	52	15
E5RIA4	Aspartyl aminopept	DNPEP	7	3
A0A024RA28	Heterogeneous nuc	HNRNPA2B1	20	4
B4DF51	Myeloid cell surface	CD33	6	1
C9JQ41	Coiled-coil domain-	MIX23	7	1
A0A0D9SFB1	Dynamin GTPase O	DNM1	5	4

A0A5F9ZHB4	Dipeptidase OS=Ho	DPEP3	4	2
H3BRQ0	Phosphopantothen	PPCDC	7	1
J3KQ18	D-dopachrome dec	DDT	38	5
M0R061	Coatomer subunit e	COPE	14	1
A0A7P0T9A7	Adenylosuccinate ly	ADSL	2	1
Q5QPQ0	Acyl-protein thioes	LYPLA2	16	2
A0A0J9YVU5	Immunoglobulin he	IGHV2-70	6	1
H0Y804	Aldo-keto reductas	AKR1C1	4	1
H0YNG5	Alpha-mannosidase	MAN2A2	5	4
H0Y614	Ubiquitin-fold modi	UFM1	9	1
F8WC81	WD repeat-containi	WDR75	7	1
A0A494C1L5	Lipopolysaccharide-	LRBA	2	5
E7ETH0	Complement factor	CFI	53	34
A0A7P0TAG8	NAD(P)H-hydrate e	NAXE	6	2
H7C3N6	Anoctamin (Fragme	ANO10	4	1
J3KMZ9	Low-density lipopro	LDLR	8	7
A0A2R8YEE1	FYN-binding proteir	FYB1	14	1
Q5T7F0	Neuropilin OS=Hom	NRP1	19	12
C9JFR7	Cytochrome c (Frag	CYCS	41	5
B7Z6Z4	Myosin light polype	MYL6	29	6
A0A7I2V506	Nucleolin OS=Homo	NCL	4	1
E9PC41	Interleukin-1 recep	IL1RL1	4	1
H0Y2Y8	Zyxin (Fragment) O:	ZYX	45	18
D6RD58	Leukocyte cell-deri	LECT2	11	1
A0A669KBE7	ADAMTS-like protei	ADAMTSL4	7	6
A0A0D9SG88	Complement factor	CFH	79	52
H7BXG7	Protein transport p	SEC31A	2	2
C9J542	PDZ and LIM domai	PDLIM4	7	1
J3KR44	Ubiquitin thioester	OTUB1	11	3
K7EPH3	N-acetylglucosamin	NAGPA	5	1
H7C2R7	Spermine synthase	SMS	5	2
C9JS68	Thromboxane-A syr	TBXAS1	7	1
F8W031	DUF3456 domain-c	--	27	6
I3L1R7	CTP:phosphoethan	PCYT2	6	2
H3BMA1	Mesothelin (Fragm	MSLN	13	4
A0A140TA69	Keratin, type I cutic	KRT34	44	17
A0A3B3ITJ4	Heterogeneous nuc	HNRNPL	6	1
A8MXL6	GATOR complex pro	SEC13	7	2
M0R2W8	N-acetylmuramoyl-	PGLYRP2	10	2
A0A286YEY4	Immunoglobulin he	IGHG2	40	22
K7ESE8	Bleomycin hydrolas	BLMH	29	6
H3BMD8	cAMP-regulated ph	ARPP19	21	3
V9HW50	All-trans-retinol del	ADH1B	27	12
A0A087WVM2	CD177 antigen OS=	CD177	2	1
E7EUJ1	Hepatic triacylglyce	LIPC	3	1
M0R0R2	40S ribosomal prot	RPS5	4	1
A0A0U1RQC5	Neurexin-3-beta OS	NRXN3	1	1
A6NKZ2	GlcNAc 2-epimeras	RENBP	13	5
X6RLJ0	Complement C1q st	C1QA	22	3
H0YHX9	Nascent polypeptid	NACA	12	2

F8VZX2	Poly(rC)-binding pro	PCBP2	10	3
V9GZ45	Alpha-methylacyl-C	AMACR	4	1
C9J8E1	MAP kinase-activat	MAPKAPK3	6	2
E7EX73	Eukaryotic translati	EIF4G1	2	2
A0A087WVC6	Protein-tyrosine-ph	PTPRJ	8	8
E7EMS7	Dermokine OS=Hon	DMKN	17	2
A0A0A0MSV6	Complement C1q su	C1QB	25	7
E7EQB2	Lactotransferrin (Fr	LTF	32	22
A0A3B3ISS6	Transmembrane gly	GPNMB	3	2
H0Y755	Low affinity immun	FCGR3A	14	4
F5GX88	Endoglin OS=Homo	ENG	9	4
C9IY88	3-hydroxyanthranila	HAAO	6	1
A0A0G2JMH6	HLA-DRA OS=Homo	HLA-DRA	3	1
A0A4W8ZXM2	Immunoglobulin he	IGHV3-72	44	9
K7ELL7	Glucosidase 2 subu	PRKCSH	20	9
B3KSI3	Branched-chain-am	BCAT2	5	1
A0A0D9SEN1	Prolyl endopeptidas	FAP	13	10
H0YFL1	cGMP-dependent 3	PDE2A	2	1
A0A494C1B8	Tripeptidyl-peptida	TPP2	19	20
H3BP09	Mothers against de	SMAD3	11	1
E9PGN7	Plasma protease C1	SERPING1	42	37
A0A7I2YQI6	Eukaryotic translati	EIF3I	5	2
C9JL93	Serpin B13 (Fragme	SERPINB13	4	1
A0A087X089	C-C motif chemokin	CCL14	23	3
A0A096LPE2	SAA2-SAA4 readthr	SAA2-SAA4	36	11
A0A1W2PQF8	Malic enzyme OS=H	ME2	8	3
P02743	Serum amyloid P-co	APCS	52	14
Q9Y5X3	Sorting nexin-5 OS=	SNX5	5	2
P05543	Thyroxine-binding g	SERPINA7	34	12
Q86WC4	Osteopetrosis-asso	OSTM1	4	1
P55809	Succinyl-CoA:3-keto	OXCT1	4	2
Q9P0Z9	Peroxisomal sarcosi	PIPOX	5	2
P55000	Secreted Ly-6/uPAR	SLURP1	9	1
Q13296	Mammaglobin-A OS	SCGB2A2	23	2
P40227	T-complex protein 1	CCT6A	10	6
P13727	Bone marrow prote	PRG2	17	4
P28827	Receptor-type tyros	PTPRM	4	5
P61026	Ras-related protein	RAB10	18	4
P06703	Protein S100-A6 OS	S100A6	17	2
P50454	Serpin H1 OS=Homo	SERPINH1	5	2
P0C7P3	Protein SLFN14 OS=	SLFN14	1	1
O15400	Syntaxin-7 OS=Hom	STX7	9	2
P16949	Stathmin OS=Homo	STMN1	8	1
Q99983	Osteomodulin OS=H	OMD	6	2
P27918	Properdin OS=Hom	CFP	34	14
P28070	Proteasome subuni	PSMB4	9	2
Q14644	Ras GTPase-activati	RASA3	13	9
Q06141	Regenerating islet-c	REG3A	12	2
Q8N392	Rho GTPase-activat	ARHGAP18	14	9
P34096	Ribonuclease 4 OS=	RNASE4	35	4

P25398	40S ribosomal prot	RPS12	18	2
P08865	40S ribosomal prot	RPSA	8	2
Q8WXD2	Secretogranin-3 OS	SCG3	1	1
Q9BXM0	Periaxin OS=Homo	PRX	1	1
Q9NRF8	CTP synthase 2 OS=	CTPS2	4	2
Q5T6V5	Queuosine salvage	C9orf64	2	1
Q99969	Retinoic acid recept	RARRES2	21	3
P10301	Ras-related protein	RRAS	8	2
Q9Y3I0	RNA-splicing ligase	RTCB	6	2
P35237	Serpin B6 OS=Homo	SERPINB6	25	8
Q9H7B4	Histone-lysine N-m	SMYD3	3	1
Q99961	Endophilin-A2 OS=H	SH3GL1	9	3
O60279	Sushi domain-conta	SUSD5	4	2
Q9NY15	Stabilin-1 OS=Homo	STAB1	3	8
P09486	SPARC OS=Homo sa	SPARC	40	9
Q92734	Protein TFG OS=Ho	TFG	5	2
Q3LXA3	Triokinase/FMN cyc	TKFC	7	4
P25325	3-mercaptopyruvat	MPST	19	5
P30626	Sorcin OS=Homo sa	SRI	25	5
Q9Y3Q8	TSC22 domain fami	TSC22D4	6	2
P0DJD7	Pepsin A-4 OS=Hom	PGA4	2	1
Q6P2Q9	Pre-mRNA-processi	PRPF8	0	1
P01033	Metalloproteinase i	TIMP1	23	4
P15090	Fatty acid-binding p	FABP4	23	3
Q5NDL2	EGF domain-specifi	EOGT	2	1
P06744	Glucose-6-phospha	GPI	38	19
P11413	Glucose-6-phospha	G6PD	30	16
Q9UJ14	Glutathione hydrol	GGT7	2	1
Q14520	Hyaluronan-binding	HABP2	31	15
P49773	Histidine triad nucle	HINT1	36	4
P08779	Keratin, type I cytos	KRT16	66	37
P33176	Kinesin-1 heavy cha	KIF5B	4	4
P05771	Protein kinase C be	PRKCB	9	7
P01700	Immunoglobulin lar	IGLV1-47	43	5
P62714	Serine/threonine-p	PPP2CB	4	1
Q13228	Methanethiol oxida	SELENBP1	15	7
Q92835	Phosphatidylinosito	INPP5D	1	1
Q9Y2Z0	Protein SGT1 homo	SUGT1	4	1
Q9H4B7	Tubulin beta-1 chai	TUBB1	46	17
Q9UI15	Transgelin-3 OS=Ho	TAGLN3	18	3
O00391	Sulfhydryl oxidase 1	QSOX1	34	20
Q93091	Ribonuclease K6 OS	RNASE6	7	1
Q9NQC3	Reticulon-4 OS=Hor	RTN4	3	2
Q9UJC5	SH3 domain-binding	SH3BGRL2	11	1
Q96ST3	Paired amphipathic	SIN3A	1	1
P68366	Tubulin alpha-4A ch	TUBA4A	60	34
P78356	Phosphatidylinosito	PIP4K2B	9	3
P27169	Serum paraoxonase	PON1	50	17
P25789	Proteasome subuni	PSMA4	19	5
P61586	Transforming prote	RHOA	32	6

Q684P5	Rap1 GTPase-activa	RAP1GAP2	5	3
Q9UNH7	Sorting nexin-6 OS=	SNX6	11	5
Q9NZQ3	NCK-interacting prc	NCKIPSD	1	1
Q7L7X3	Serine/threonine-pı	TAOK1	4	4
Q06643	Lymphotoxin-beta (	LTB	4	1
P00441	Superoxide dismuta	SOD1	31	4
Q9Y6E0	Serine/threonine-pı	STK24	26	11
O94804	Serine/threonine-pı	STK10	3	3
P43487	Ran-specific GTPase	RANBP1	18	3
P62328	Thymosin beta-4 O!	TMSB4X	89	7
Q15126	Phosphomevalonat	PMVK	16	3
Q9Y265	RuvB-like 1 OS=Hon	RUVBL1	2	1
P78324	Tyrosine-protein ph	SIRPA	14	8
Q9H3N1	Thioredoxin-relatec	TMX1	3	1
P20742	Pregnancy zone prc	PZP	29	41
Q14141	Septin-6 OS=Homo	SEPTIN6	22	11
Q13596	Sorting nexin-1 OS=	SNX1	4	2
Q13093	Platelet-activating f	PLA2G7	12	5
P62191	26S proteasome reg	PSMC1	11	5
P49247	Ribose-5-phosphate	RPIA	6	1
P25815	Protein S100-P OS=	S100P	24	2
Q8TAQ9	SUN domain-contai	SUN3	2	1
P22531	Small proline-rich p	SPRR2E	36	2
Q96G03	Phosphoglucomuta	PGM2	13	8
Q6UWP8	Suprabasin OS=Hon	SBSN	18	7
Q9HCB6	Spondin-1 OS=Hom	SPON1	6	3
Q9Y3F4	Serine-threonine kiı	STRAP	11	4
Q9NSD9	Phenylalanine--tRN.	FARSB	2	1
P00747	Plasminogen OS=Hc	PLG	73	58
P17980	26S proteasome reg	PSMC3	4	2
Q07960	Rho GTPase-activat	ARHGAP1	21	8
Q13885	Tubulin beta-2A cha	TUBB2A	37	16
P37802	Transgelin-2 OS=Ho	TAGLN2	69	18
Q86UF1	Tetraspanin-33 OS=	TSPAN33	5	1
O95071	E3 ubiquitin-proteir	UBR5	1	2
O43865	S-adenosylhomocys	AHCYL1	11	6
P04279	Semenogelin-1 OS=	SEMG1	9	4
A1L4H1	Soluble scavenger r	SSC5D	16	17
Q8WWA1	Transmembrane prı	TMEM40	5	1
Q04323	UBX domain-contai	UBXN1	4	1
P21281	V-type proton ATPa	ATP6V1B2	10	4
Q6EMK4	Vasorin OS=Homo s	VASN	12	6
P38606	V-type proton ATPa	ATP6V1A	16	10
O75340	Programmed cell de	PDCD6	10	2
Q96PD5	N-acetylmuramoyl-	PGLYRP2	32	17
P11216	Glycogen phosphor	PYGB	12	10
P51692	Signal transducer aı	STAT5B	8	6
P22528	Cornifin-B OS=Hom	SPRR1B	34	2
P62937	Peptidyl-prolyl cis-t	PPIA	55	13
P21549	Serine--pyruvate an	AGXT	6	2

P02787	Serotransferrin OS=	TF	57	61
P62820	Ras-related protein	RAB1A	29	6
Q9UBG0	C-type mannose re	MRC2	4	5
P36955	Pigment epithelium	SERPINF1	45	19
P22059	Oxysterol-binding p	OSBP	1	1
P27105	Stomatin OS=Homo	STOM	11	3
P50225	Sulfotransferase 1A	SULT1A1	6	2
Q4LDE5	Sushi, von Willebra	SVEP1	1	4
Q16762	Thiosulfate sulfurtr	TST	16	4
P49368	T-complex protein :	CCT3	26	14
P02776	Platelet factor 4 OS	PF4	60	9
P78527	DNA-dependent pro	PRKDC	0	2
Q9BRP8	Partner of Y14 and	PYM1	12	2
Q14242	P-selectin glycoprot	SELPLG	2	1
O15389	Sialic acid-binding Ig	SIGLEC5	2	1
P40763	Signal transducer a	STAT3	4	2
P67936	Tropomyosin alpha	TPM4	48	18
Q12974	Protein tyrosine ph	PTP4A2	20	3
P49721	Proteasome subuni	PSMB2	23	4
Q06124	Tyrosine-protein ph	PTPN11	15	8
P54727	UV excision repair p	RAD23B	11	5
Q9UL25	Ras-related protein	RAB21	11	3
P52758	2-iminobutanoate/2	RIDA	20	2
P61086	Ubiquitin-conjugati	UBE2K	36	6
Q8TF42	Ubiquitin-associate	UBASH3B	15	8
Q96RL7	Vacuolar protein so	VPS13A	0	1
Q96QK1	Vacuolar protein so	VPS35	4	3
Q8WWY7	WAP four-disulfide	WFDC12	9	1
P04070	Vitamin K-depende	PROC	15	6
O75558	Syntaxin-11 OS=Ho	STX11	3	1
P01624	Immunoglobulin ka	IGKV3-15	31	5
P22897	Macrophage mann	MRC1	20	30
P26447	Protein S100-A4 OS	S100A4	38	5
P05109	Protein S100-A8 OS	S100A8	48	7
P0DJI9	Serum amyloid A-2	SAA2	54	11
P36952	Serpin B5 OS=Hom	SERPINB5	10	4
Q96QR1	Secretoglobin famil	SCGB3A1	13	1
Q9UIB8	SLAM family memb	CD84	6	1
O43278	Kunitz-type proteas	SPINT1	4	2
P09619	Platelet-derived gr	PDGFRB	6	6
P08237	ATP-dependent 6-p	PFKM	3	2
P30044	Peroxiredoxin-5, mi	PRDX5	31	6
P41222	Prostaglandin-H2 D	PTGDS	29	5
Q99747	Gamma-soluble NSI	NAPG	3	1
O75563	Src kinase-associate	SKAP2	11	4
O95630	STAM-binding prot	STAMBP	7	2
Q9H0U4	Ras-related protein	RAB1B	36	8
Q15166	Serum paraoxonase	PON3	24	8
P28066	Proteasome subuni	PSMA5	25	4
P25787	Proteasome subuni	PSMA2	13	2

Q13200	26S proteasome no	PSMD2	2	2
P13489	Ribonuclease inhibi	RNH1	37	13
P06702	Protein S100-A9 OS	S100A9	68	7
Q86U17	Serpin A11 OS=Hon	SERPINA11	13	5
P03973	Antileukoproteinas	SLPI	32	4
Q15833	Syntaxin-binding pr	STXBP2	19	9
Q05209	Tyrosine-protein ph	PTPN12	3	2
Q99460	26S proteasome no	PSMD1	1	1
Q9NPR2	Semaphorin-4B OS=	SEMA4B	6	5
Q7Z406	Myosin-14 OS=Hon	MYH14	2	4
P05120	Plasminogen activa	SERPINB2	2	1
O15534	Period circadian pr	PER1	1	1
Q9UQ80	Proliferation-associ	PA2G4	25	8
Q01813	ATP-dependent 6-p	PFKP	19	12
O14818	Proteasome subuni	PSMA7	25	5
P05387	60S acidic ribosoma	RPLP2	10	1
P10153	Non-secretory ribor	RNASE2	15	4
P17900	Ganglioside GM2 ac	GM2A	13	3
P00390	Glutathione reducta	GSR	16	8
Q8WUM4	Programmed cell de	PDCD6IP	17	14
P05164	Myeloperoxidase O	MPO	20	14
P33764	Protein S100-A3 OS	S100A3	11	1
Q7KZF4	Staphylococcal nucl	SND1	18	14
P04179	Superoxide dismuta	SOD2	36	8
O60749	Sorting nexin-2 OS=	SNX2	11	5
Q14CX7	N-alpha-acetyltrans	NAA25	1	1
Q04721	Neurogenic locus n	NOTCH2	2	5
Q9UMX5	Neudesin OS=Homo	NENF	13	1
P07737	Profilin-1 OS=Homo	PFN1	83	15
Q9UHD8	Septin-9 OS=Homo	SEPTIN9	3	2
P00558	Phosphoglycerate k	PGK1	50	23
P43686	26S proteasome re	PSMC4	3	1
P35813	Protein phosphatas	PPM1A	28	9
Q9H939	Proline-serine-thre	PSTPIP2	34	11
P49720	Proteasome subuni	PSMB3	14	3
P55036	26S proteasome no	PSMD4	5	2
P61224	Ras-related protein	RAP1B	35	8
P62834	Ras-related protein	RAP1A	35	7
P31151	Protein S100-A7 OS	S100A7	23	2
P80511	Protein S100-A12 O	S100A12	9	1
Q96FQ6	Protein S100-A16 O	S100A16	23	2
O00151	PDZ and LIM domai	PDLIM1	60	18
Q15149	Plectin OS=Homo sa	PLEC	1	4
Q15293	Reticulocalbin-1 OS	RCN1	2	1
P62491	Ras-related protein	RAB11A	14	3
P61019	Ras-related protein	RAB2A	16	3
Q86UN3	Reticulon-4 recepto	RTN4RL2	14	5
P62280	40S ribosomal prot	RPS11	6	1
Q13835	Plakophilin-1 OS=H	PKP1	26	16
P61006	Ras-related protein	RAB8A	25	5

O43182	Rho GTPase-activat	ARHGAP6	1	1
Q15102	Platelet-activating f	PAFAH1B3	4	1
P28161	Glutathione S-trans	GSTM2	27	6
P10721	Mast/stem cell grov	KIT	11	10
P49862	Kallikrein-7 OS=Hor	KLK7	4	1
A0A075B6I6	Probable non-funct	IGLV1-50	24	3
Q15121	Astrocytic phospho	PEA15	11	1
O94903	Pyridoxal phosphat	PLPBP	7	2
Q86WI1	Fibrocystin-L OS=Hc	PKHD1L1	1	5
Q8IV08	5'-3' exonuclease Pl	PLD3	4	2
Q96BZ4	5'-3' exonuclease Pl	PLD4	7	4
Q9UBQ7	Glyoxylate reductas	GRHPR	12	4
P17931	Galectin-3 OS=Hom	LGALS3	13	4
P41218	Myeloid cell nuclea	MNDA	6	2
P01834	Immunoglobulin ka	IGKC	73	20
Q2M2I5	Keratin, type I cytos	KRT24	9	6
Q13576	Ras GTPase-activati	IQGAP2	16	25
P05107	Integrin beta-2 OS=	ITGB2	4	3
Q5T749	Keratinocyte prolin	KPRP	25	10
P17050	Alpha-N-acetylgalac	NAGA	9	3
Q99471	Prefoldin subunit 5	PFDN5	10	2
P14780	Matrix metalloprot	MMP9	15	10
Q8IUK5	Plexin domain-cont	PLXDC1	3	1
P80303	Nucleobindin-2 OS=	NUCB2	7	3
O00592	Podocalyxin OS=Ho	PODXL	4	2
Q04118	Basic salivary prolin	PRB3	4	1
Q86YZ3	Hornerin OS=Homo	HRNR	27	23
P01880	Immunoglobulin he	IGHD	14	4
P63241	Eukaryotic translati	EIF5A	45	6
Q9H492	Microtubule-associ	MAP1LC3A	6	1
Q9H1U4	Multiple epidermal	MEGF9	2	1
Q7L9L4	MOB kinase activat	MOB1B	11	2
Q14432	cGMP-inhibited 3',5	PDE3A	2	1
Q9UHY7	Enolase-phosphatas	ENOPH1	5	1
Q99714	3-hydroxyacyl-CoA	HSD17B10	12	2
P01701	Immunoglobulin lar	IGLV1-51	38	5
Q92882	Osteoclast-stimulat	OSTF1	9	2
Q96S96	Phosphatidylethanc	PEBP4	17	3
Q8N573	Oxidation resistanc	OXR1	3	2
P61758	Prefoldin subunit 3	VBP1	5	1
Q6PCE3	Glucose 1,6-bisphos	PGM2L1	1	1
O43157	Plexin-B1 OS=Homc	PLXNB1	2	2
Q53T59	HCLS1-binding prot	HS1BP3	3	1
P16402	Histone H1.3 OS=Hc	H1-3	10	2
Q15323	Keratin, type I cutic	KRT31	50	22
P05106	Integrin beta-3 OS=	ITGB3	27	20
P01042	Kininogen-1 OS=Ho	KNG1	52	57
O75594	Peptidoglycan reco	PGLYRP1	12	2
P01619	Immunoglobulin ka	IGKV3-20	43	7
P61626	Lysozyme C OS=Hor	LYZ	44	8

O60449	Lymphocyte antigeı	LY75	1	2
Q9ULC4	Malignant T-cell-am	MCTS1	22	3
A0A0B4J1U3	Immunoglobulin lar	IGLV1-36	13	1
Q9UNZ2	NSFL1 cofactor p47	NSFL1C	19	6
Q15365	Poly(rC)-binding pr	PCBP1	39	11
Q9NPG4	Protocadherin-12 C	PCDH12	2	2
Q9HBI1	Beta-parvin OS=Hoı	PARVB	17	5
Q96CX2	BTB/POZ domain-cc	KCTD12	7	2
Q86X29	Lipolysis-stimulatec	LSR	10	5
P14543	Nidogen-1 OS=Hom	NID1	21	22
P01714	Immunoglobulin lar	IGLV3-19	26	3
Q15746	Myosin light chain k	MYLK	11	17
Q8WWZ8	Oncoprotein-induce	OIT3	10	5
Q6UWY5	Olfactomedin-like p	OLFML1	4	1
P05121	Plasminogen activa	SERPINE1	16	5
P03952	Plasma kallikrein OS	KLKB1	48	31
P01706	Immunoglobulin lar	IGLV2-11	16	2
P04211	Immunoglobulin lar	IGLV7-43	15	2
Q7Z3B1	Neuronal growth re	NEGR1	13	4
P15531	Nucleoside diphosp	NME1	47	9
P48163	NADP-dependent m	ME1	5	3
Q9UEW3	Macrophage recept	MARCO	7	3
Q15691	Microtubule-associ	MAPRE1	37	8
Q8TCD5	5'(3')-deoxyribonuc	NT5C	10	2
Q9BUN1	Protein MENT OS=H	MENT	4	1
Q5VVQ6	Ubiquitin thioester	YOD1	2	1
Q13126	S-methyl-5'-thioade	MTAP	18	5
P13667	Protein disulfide-isc	PDIA4	29	16
P62136	Serine/threonine-pı	PPP1CA	20	8
Q92859	Neogenin OS=Hom	NEO1	8	11
P10619	Lysosomal protecti	CTSA	3	1
P53384	Cytosolic Fe-S clust	NUBP1	3	1
Q08174	Protocadherin-1 OS	PCDH1	1	1
P35998	26S proteasome re	PSMC2	11	4
Q13526	Peptidyl-prolyl cis-t	PIN1	18	2
P45877	Peptidyl-prolyl cis-t	PPIC	13	3
Q99952	Tyrosine-protein ph	PTPN18	7	2
Q06323	Proteasome activat	PSME1	27	6
P61020	Ras-related protein	RAB5B	16	3
P15153	Ras-related C3 botu	RAC2	33	6
P20936	Ras GTPase-activati	RASA1	2	2
O75116	Rho-associated pro	ROCK2	10	13
Q9UJ70	N-acetyl-D-glucosar	NAGK	7	2
P05976	Myosin light chain 1	MYL1	18	3
P51575	P2X purinoceptor 1	P2RX1	2	1
Q9Y446	Plakophilin-3 OS=H	PKP3	3	2
Q00722	1-phosphatidylinosi	PLCB2	3	3
P30405	Peptidyl-prolyl cis-t	PPIF	29	5
P28074	Proteasome subuni	PSMB5	8	2
Q9NP72	Ras-related protein	RAB18	11	2

P11234	Ras-related protein	RALB	16	3
P31939	Bifunctional purine	ATIC	17	10
P51003	Poly(A) polymerase	PAPOLA	2	1
Q6XQN6	Nicotinate phospho	NAPRT	20	9
O95897	Noelin-2 OS=Homo	OLFM2	2	1
P09211	Glutathione S-trans	GSTP1	56	12
P30740	Leukocyte elastase	SERPINB1	36	12
P01705	Immunoglobulin lar	IGLV2-23	15	1
Q9NSB4	Keratin, type II cutic	KRT82	3	2
Q05655	Protein kinase C del	PRKCD	7	5
Q8TF66	Leucine-rich repeat	LRRC15	6	3
O00743	Serine/threonine-pı	PPP6C	3	1
P29350	Tyrosine-protein ph	PTPN6	40	21
Q9NRW1	Ras-related protein	RAB6B	12	2
Q13637	Ras-related protein	RAB32	4	1
Q9UKU6	Thyrotropin-releasi	TRHDE	2	2
O95881	Thioredoxin domair	TXNDC12	14	2
Q96K76	Ubiquitin carboxyl-t	USP47	1	2
P14868	Aspartate--tRNA lig	DARS1	30	14
P37837	Transaldolase OS=H	TALDO1	37	14
Q8WZ75	Roundabout homol	ROBO4	7	5
Q13464	Rho-associated pro	ROCK1	4	6
Q13103	Secreted phosphop	SPP2	32	6
Q92890	Ubiquitin recognitic	UFD1	9	2
O75954	Tetraspanin-9 OS=H	TSPAN9	5	1
O75083	WD repeat-containi	WDR1	58	33
P55072	Transitional endopl	VCP	27	21
P45974	Ubiquitin carboxyl-t	USP5	2	2
Q9BRA2	Thioredoxin domair	TXNDC17	19	2
Q9H2K8	Serine/threonine-pı	TAOK3	5	4
O43294	Transforming growt	TGFB1I1	3	1
P41226	Ubiquitin-like modi	UBA7	15	11
O15231	Zinc finger protein 1	ZNF185	3	2
P02774	Vitamin D-binding p	GC	70	53
Q5T750	Skin-specific proteir	XP32	8	2
Q15819	Ubiquitin-conjugati	UBE2V2	25	3
P48147	Prolyl endopeptidas	PREP	8	5
P17987	T-complex protein 1	TCP1	25	12
O00534	von Willebrand fact	VWA5A	6	4
P16035	Metalloproteinase i	TIMP2	10	2
P07911	Uromodulin OS=Ho	UMOD	16	8
O75436	Vacuolar protein so	VPS26A	2	1
Q9NQW7	Xaa-Pro aminopept	XPNPEP1	17	10
Q9Y6W5	Wiskott-Aldrich syn	WASF2	4	2
P11684	Uteroglobin OS=Ho	SCGB1A1	9	1
Q96H79	Zinc finger CCCH-ty	ZC3HAV1L	8	2
P19320	Vascular cell adhesi	VCAM1	28	19
P51809	Vesicle-associated r	VAMP7	4	1
O75351	Vacuolar protein so	VPS4B	14	6
P30101	Protein disulfide-isc	PDIA3	42	21

P23470	Receptor-type tyros	PTPRG	5	6
Q9H4X1	Regulator of cell cyc	RGCC	15	1
P49321	Nuclear autoantigen	NASP	3	1
P35579	Myosin-9 OS=Homo	MYH9	30	54
Q86UD1	Out at first protein	OAF	11	3
Q16822	Phosphoenolpyruva	PCK2	6	3
P80108	Phosphatidylinosito	GPLD1	22	18
Q5VY43	Platelet endothelial	PEAR1	2	2
Q4G148	Glucoside xylosyltra	GXYLT1	3	1
Q14533	Keratin, type II cutic	KRT81	40	23
Q9BQG0	Myb-binding protei	MYBBP1A	1	1
P22102	Trifunctional purine	GART	3	3
P13716	Delta-aminolevulini	ALAD	23	8
P34932	Heat shock 70 kDa		HSPA4	20	15
P00738	Haptoglobin OS=Ho	HP	69	27
Q14012	Calcium/calmodulir	CAMK1	5	2
P11047	Laminin subunit gar	LAMC1	4	5
O43813	Glutathione S-trans	LANCL1	9	3
O00231	26S proteasome no	PSMD11	3	1
P04085	Platelet-derived grc	PDGFA	4	1
P00491	Purine nucleoside p	PNP	51	13
P13796	Plastin-2 OS=Homo	LCP1	50	32
Q9HAB8	Phosphopantothen	PPCS	14	4
P07988	Pulmonary surfacta	SFTPB	7	2
Q15262	Receptor-type tyros	PTPRK	3	3
O60462	Neuropilin-2 OS=Hc	NRP2	6	5
P61970	Nuclear transport fa	NUTF2	21	2
Q9UHY1	Nuclear receptor-bi	NRBP1	5	2
P08567	Pleckstrin OS=Homo	PLEK	27	8
P11908	Ribose-phosphate p	PRPS2	6	2
P25788	Proteasome subuni	PSMA3	12	3
P28062	Proteasome subuni	PSMB8	20	6
Q16769	Glutaminyl-peptide	QPCT	6	2
P54725	UV excision repair p	RAD23A	7	3
Q7Z7M0	Multiple epidermal	MEGF8	7	14
Q9NQR4	Omega-amidase NI	NIT2	25	5
A0A0C4DH69	Immunoglobulin ka	IGKV1-9	24	5
Q9BYE3	Late cornified envel	LCE3D	37	1
P18054	Polyunsaturated fat	ALOX12	18	11
Q9BU23	Lipase maturation f	LMF2	1	1
P26572	Alpha-1,3-mannosy	MGAT1	8	3
Q13162	Peroxiredoxin-4 OS	PRDX4	22	6
P10586	Receptor-type tyros	PTPRF	7	11
Q99784	Noelin OS=Homo sa	OLFM1	10	4
Q8NBP7	Proprotein convert	PCSK9	12	6
Q9UHV9	Prefoldin subunit 2	PFDN2	21	3
P16284	Platelet endothelial	PECAM1	11	8
P05186	Alkaline phosphata	ALPL	4	2
P40306	Proteasome subuni	PSMB10	4	1
Q9NS28	Regulator of G-prot	RGS18	20	3

P48739	Phosphatidylinosito	PITPNB	19	4
P30041	Peroxiredoxin-6 OS	PRDX6	44	10
Q6ZRP7	Sulfhydryl oxidase 2	QSOX2	5	3
P35625	Metalloproteinase i	TIMP3	4	1
O43617	Trafficking protein p	TRAPPC3	4	1
P02766	Transthyretin OS=H	TTR	76	20
O00161	Synaptosomal-asso	SNAP23	20	4
O60493	Sorting nexin-3 OS=	SNX3	6	1
P26639	Threonine--tRNA lig	TARS1	10	8
P23381	Tryptophan--tRNA l	WARS1	10	4
P07437	Tubulin beta chain (	TUBB	40	17
P30530	Tyrosine-protein kir	AXL	5	4
Q02818	Nucleobindin-1 OS=	NUCB1	11	4
Q9NTK5	Obg-like ATPase 1 C	OLA1	25	9
P27986	Phosphatidylinosito	PIK3R1	3	2
P98160	Basement membrai	HSPG2	14	49
P17858	ATP-dependent 6-p	PFKL	7	4
Q8IWL2	Pulmonary surfacta	SFTPA1	5	1
P10599	Thioredoxin OS=Ho	TXN	21	2
Q99832	T-complex protein 1	CCT7	13	6
P35442	Thrombospondin-2	THBS2	4	4
Q9NSB2	Keratin, type II cuti	KRT84	36	20
Q9Y3C8	Ubiquitin-fold modi	UFC1	5	1
Q9BRP4	Proteasomal ATPas	PAAF1	3	1
Q92954	Proteoglycan 4 OS=	PRG4	12	14
Q15714	TSC22 domain fami	TSC22D1	2	2
P24821	Tenascin OS=Homo	TNC	19	34
Q6ICL3	Transport and Golgi	TANGO2	11	3
P50990	T-complex protein 1	CCT8	25	13
P50552	Vasodilator-stimula	VASP	41	16
P35590	Tyrosine-protein kir	TIE1	6	5
Q92752	Tenascin-R OS=Hon	TNR	1	1
Q9Y4G6	Talin-2 OS=Homo sa	TLN2	6	14
P54578	Ubiquitin carboxyl-t	USP14	12	6
O43516	WAS/WASL-interac	WIPF1	32	11
Q9BRG1	Vacuolar protein-so	VPS25	6	1
E9PD35	Vascular endothelia	FLT4	7	7
A0A2U3TZL5	CD59 glycoprotein (	CD59	10	1
B1AHF3	NADH-cytochrome	CYB5R3	5	1
B8ZZZ0	3-hydroxyisobutyry	HIBCH	3	1
Q5T8H6	Ciliary neurotrophic	CNTFR	5	1
G3V158	2-deoxy-D-ribose 5-	DERA	5	1
F6SYF8	Dickkopf-related pr	DKK3	9	2
A0A5F9ZHM4	L-lactate dehydroge	LDHB	46	19
J3KQ66	Reelin OS=Homo sa	RELN	1	2
A0A494C1M4	10-formyltetrahydr	ALDH1L2	1	1
A0A087WWU8	Tropomyosin alpha-	TPM3	52	16
A0A7I2V2R5	Dipeptidyl peptidas	DPP4	38	27
A0A7P0TAK8	Neurogenic locus n	NOTCH1	3	4
H9KV31	Neural cell adhesio	NCAM2	14	10

A0A7P0T8X6	Tectonic-3 OS=Hom	TCTN3	2	1
F5GXE4	Arginyl-tRNA--prote	ATE1	4	2
C9JIZ6	Prosaposin OS=Hon	PSAP	25	13
E7EX17	Eukaryotic translati	EIF4B	8	4
A0A7I2V3T0	Tetraspanin OS=Ho	CD9	14	4
A0A075B7B8	Immunoglobulin he	IGHV3OR16-12	9	1
J3QQX6	Intercellular adhesi	ICAM2	8	2
A0A0B4J2B8	Immunoglobulin he	IGHV1OR15-9	10	1
H0YAW3	2,4-dienoyl-CoA rec	DECR1	23	1
A0A1X7SBS1	Heterogeneous nuc	HNRNPU	3	1
A0A087X2I1	26S proteasome re	PSMC6	10	4
A0A087WX80	Laminin subunit alp	LAMA2	2	7
H0YAH3	Polypeptide N-acet	GALNT7	6	3
J3QLV6	Coronin OS=Homo	CORO6	6	3
Q5SR44	Complement recep	CR1	2	2
A0A7P0TAE1	Endoplasmin OS=H	HSP90B1	34	24
H7C0L5	Inter-alpha-trypsin	ITIH4	58	58
B1ALD9	Periostin OS=Homo	POSTN	17	14
B1AN48	Small proline-rich p	SPRR3	10	2
B4E0K5	Mitogen-activated	MAPK14	23	6
F5GZS6	4F2 cell-surface ant	SLC3A2	20	9
E7EUI6	Integrin beta OS=H	ITGB1	22	15
A0A7P0SBL1	Ribonucleoside-dipl	RRM2	5	1
J3KS22	L-xylulose reductas	DCXR	8	2
A0A087X0R6	Sorting nexin-12 OS	SNX12	20	4
M0R1T5	Charged multivesic	CHMP2A	7	2
F5GZQ3	Trifunctional enzym	HADHB	2	1
B8ZZK2	Gamma-glutamylcy	GGCT	20	3
M0QZQ3	Spectrin beta chain	SPTBN4	1	2
A0A6Q8PFE5	Calpain inhibitor OS	CAST	7	3
A0A286YFJ8	Immunoglobulin he	IGHG4	44	24
A0A494C165	Xaa-Pro dipeptidase	PEPD	33	15
A0A0G2JMM7	Osteoclast-associat	OSCAR	4	1
C9J931	GTP-binding proteir	RHEB	27	2
B5MCH7	Basic leucine zipper	BZW2	3	1
F5H4W5	Porphobilinogen de	HMBS	17	1
D6RER5	Septin OS=Homo sa	SEPTIN11	15	8
A0A5H1ZRQ7	Immunoglobulin lar	IGLC7	37	9
D6RE86	Ceruloplasmin (Frag	CP	43	9
H7C024	Glypican-1 (Fragme	GPC1	5	1
A0A087WYT3	Prostaglandin E syn	PTGES3	28	7
E9PBC5	Plasma kallikrein OS	KLKB1	44	22
A0A087WWD4	Neural cell adhesio	NCAM1	21	17
A0A0A0MTH3	59 kDa serine/threc	ILK	38	16
A0A140T9U0	HLA class I histocon	HLA-C	37	11
A0A7P0Z4I7	Cyclic AMP-depend	ATF6	2	1
G8JLG2	Corneodesmosin OS	CDSN	7	3
C9K0C5	Protein FAM234A (I	FAM234A	4	1
A0A1W2PRU0	Alpha-endosulfine (	ENSA	56	6
A0A1B0GV97	Coiled-coil domain-	CCDC33	3	1

K7ERP4	Glutathione peroxic	GPX4	32	4
A0A087WZZ6	Mucin-1 OS=Homo	MUC1	9	1
K7ERE3	Keratin, type I cytos	KRT13	28	14
A8MU27	Small ubiquitin-rela	SUMO3	8	1
E5RJ77	Protein-tyrosine kin	PTK2B	8	2
E9PBS1	AIR carboxylase (Fra	PAICS	7	3
Q5VZ73	C-C motif chemokin	CCL21	10	1
E9PEX6	Dihydrolipoyl dehyc	DLD	2	1
H0Y512	Adipocyte plasma n	APMAP	36	14
H7C3P9	COP9 signalosome (	COPS3	5	1
F8W6I7	Helix-destabilizing p	HNRNPA1	12	2
A0A0A0MT32	Lysosomal acid lipa	LIPA	4	1
C9JYY6	Neuronal cell adhes	NRCAM	6	6
A0A087WYX9	Collagen alpha-2(V)	COL5A2	1	1
A0A087WTB5	Interleukin-6 recept	IL6R	4	2
Q5T5C7	Seryl-tRNA syntheta	SARS1	12	6
C9J0E4	Cystatin-A OS=Hom	CSTA	43	3
E5RFU2	Bifunctional epoxid	EPHX2	5	3
G3V2U7	Acylphosphatase OS	ACYP1	19	2
A0A2R8YCW8	Band 4.1 OS=Homo	EPB41	2	2
H7BXY5	Nexilin (Fragment) (	NEXN	2	1
E9PJA2	Macrophage colony	CSF1	4	1
A0A5H1ZRS9	Immunoglobulin ka	IGKV2D-29	47	6
A0A1W2PRS4	Junctional adhesion	JAML	8	2
H7BYY1	Tropomyosin 1 (Alp	TPM1	41	12
U3KPS2	Myeloblastin OS=Ho	PRTN3	4	1
C9JF17	Apolipoprotein D (F	APOD	32	13
E9PEK4	Macrophage colony	CSF1R	8	4
C9J2C0	Tubulin alpha chain	TUBA8	36	23
C9J5C3	Programmed cell de	PDCD10	11	2
H3BSA6	dCTP pyrophosphat	DCTPP1	14	1
H3BPK7	Alanine--tRNA ligas	AARS1	13	12
E9PQY2	Prefoldin subunit 4	PFDN4	9	1
F5H365	Protein transport pı	SEC23A	2	1
H7C131	3-ketoacyl-CoA thic	ACAA1	19	4
A0A0S2Z4G6	Tropomyosin 1 (Alp	TPM1	31	11
L7P8G6	Methylenetetrahyd	MTHFR	3	1
F8VUA2	Charged multivesicı	CHMP1A	4	1
E9PGA6	C1QTNF3-AMACR r	C1QTNF3-AMACR	11	3
E9PGM4	1,4-alpha-glucan br	GBE1	5	3
F5H2F4	C-1-tetrahydrofolat	MTHFD1	9	8
C9JIG9	Non-specific serine,	OXSR1	2	1
A0A5K1VW95	Malate dehydrogen	MDH1	32	10
H0YDZ7	Guanine deaminase	GDA	6	1
D6RCP9	Deoxycytidine kinas	DCK	4	1
A2A2V1	Major prion proteir	PRNP	4	1
A0A6I8PRN4	Glutamine--fructos	GFPT1	2	1
F8W881	Constitutive coactiv	FAM120C	1	1
H7BY64	ZNF511-PRAP1 reac	ZNF511-PRAP1	7	2
F6U1T9	Calcineurin subunit	PPP3R1	6	1

B4E3Q4	Adenosine deamina	ADA2	19	7
Q32Q12	Nucleoside diphosp	NME1-NME2	39	10
B0QZ18	Copine-1 OS=Homo	CPNE1	14	8
F5GZZ9	Scavenger receptor	CD163	22	18
S4R471	Alpha-1-microglobu	AMBP	54	14
Q5JXI8	Four and a half LIM	FHL1	47	11
A0A087WXM8	Basal cell adhesion	BCAM	6	3
H0YCV9	CD44 antigen (Frag	CD44	14	4
Q68DW7	Cohesin subunit SA-	STAG1	1	1
A0A0G2JII1	HLA class II histocor	HLA-DPB1	5	1
A0A7I2V2U8	Twinfilin-2 OS=Hom	TWF2	39	11
E7EVW7	Hematopoietic line	HCLS1	14	5
C9IZP8	Complement C1s su	C1S	39	2
J3KNB3	Dual adapter for ph	DAPP1	5	1
B1AK88	F-actin-capping pro	CAPZB	32	10
B7ZKJ8	ITIH4 protein OS=H	ITIH4	59	80
D6RD47	40S ribosomal prot	RPS23	20	1
C9JIX8	Ephrin type-A recep	EPHA4	14	1
C9JWB2	Interleukin-1 recep	IL1R1	9	1
A0A0B4J1R6	Transketolase OS=H	TKT	44	16
M0QXB5	Persulfide dioxygen	ETHE1	5	1
A0A0G2JPP1	Keratin-associated	KRTAP4-8	10	1
B7ZAX5	N-acetylgalactosam	GALK2	2	1
A0A7I2V2S0	RNA helicase OS=H	DDX5	5	1
Q8NBY1	Serine/threonine-p	STK26	18	7
B8ZWD1	Acyl-CoA-binding pr	DBI	37	3
A0A0U1RR22	Protein kinase C an	PACSIN2	12	5
A0A7P0Z497	Peptidyl-prolyl cis-t	PPIB	52	10
G3V4U0	Fibulin-5 OS=Homo	FBLN5	20	8
A0A0C4DGZ8	Glycoprotein Ib (Pla	GP1BA	15	11
F5GXS2	Actinin, alpha 4, iso	ACTN4	29	26
K7EQF8	Lon protease homo	LONP1	9	1
A0A2R8Y5S7	Radixin OS=Homo s	RDX	18	13
A0A0G2JSC0	Immunoglobulin lar	IGLV5-45	15	2
A0A2U3TZV8	Phosphoinositide pl	PLCH1	0	1
A0A7I2YQK0	Glycogen synthase	GSK3B	2	1
E9PNF7	Lysosomal Pro-X ca	PRCP	14	6
H0Y449	Y-box-binding prote	YBX1	3	1
X6RFL8	Ras-related protein	RAB14	39	7
E7EPV7	Alpha-synuclein OS	SNCA	50	4
B4DDF4	Calponin OS=Homo	CNN2	36	10
X6RLR1	Dynactin subunit 3	DCTN3	9	2
B8ZZC1	Fibulin-7 OS=Homo	FBLN7	3	1
A0A1B0GVI3	Keratin, type I cyto	KRT10	58	54
B1AHL2	Fibulin-1 OS=Homo	FBLN1	33	20
E7ENY0	Alpha-adducin OS=I	ADD1	3	1
A0A7I2V3M3	Cathepsin F OS=Ho	CTSF	8	3
D6REY1	Chitotriosidase-1 O	CHIT1	13	4
E7EW33	Cytoplasmic FMR1-	CYFIP2	6	6
C9JPM4	ADP-ribosylation fa	ARF4	36	5

A0A0G2JMC9	Leukocyte immuno	LILRA2	8	3
Q5T7C4	High mobility group	HMGB1	13	2
Q5URX0	Beta-N-acetylhexos	HEXB	13	4
A0A1B0GUE3	Acid ceramidase OS	ASAH1	12	4
A0A0G2JHC2	PPP1R18 OS=Homo	PPP1R18	1	1
M0R0X1	Ras-related protein	RAB4B	16	3
A0A0J9YXZ5	Ras GTPase-activati	IQGAP1	3	4
K7EKP8	Cytosolic acyl coen	ACOT7	8	2
D6RAR0	5-phosphohydroxy-	PHYKPL	7	1
C9J1E7	AP-1 complex subu	AP1B1	5	3
A0A0A0MT99	Immunoglobulin lar	IGLJ3	16	1
E5RGN3	Copper transport p	ATOX1	14	1
Q5SRR8	Dimethylargininase	DDAH2	12	3
A0A7I2V2U2	Small glutamine-ric	SGTA	8	2
B1AQM9	Consortin OS=Hom	CNST	6	1
F8VV59	Nucleosome assem	NAP1L1	24	8
H7BYD9	Peptidylamidoglyco	PAM	10	6
A0A5F9ZH72	Adenosine kinase O	ADK	6	1
H9KV28	Protein diaphanous	DIAPH1	9	11
A6NFX8	ADP-sugar pyropho	NUDT5	23	6
A0A7P0TAE9	Calnexin OS=Homo	CANX	10	7
F6WIT2	Serine/threonine-p	PTPA	22	6
I3L3P5	Protein disulfide-isc	P4HB	40	19
F6U784	Ras-related protein	RAP2A	10	1
A0A7I2V3F9	Hepatocyte growth	HGS	3	2
A0A087WUR8	Inactive serine prot	PAMR1	2	1
E5RH18	LSM1-like protein U	LSM1	16	1
Q2L6G2	HLA class I histocon	HLA-B	21	6
F5GX71	Mannose-6-phosph	MPI	4	1
A6NDF3	Protein PBDC1 OS=	PBDC1	9	2
A0A087WVQ9	Elongation factor 1-	EEF1A1	14	7
B4DDD6	Drebrin-like protein	DBNL	36	12
G3V114	Docking protein 2 C	DOK2	26	3
J3QQU6	Secreted and transr	SECTM1	13	1
F8W8N3	Cyclin-A/CDK2-asso	SKP1	5	1
A0A087WVE2	Ficolin-1 OS=Homo	FCN1	6	2
F6TLX2	Glyoxalase domain-	GLOD4	14	7
D3DRR9	Chromosome 10 op	PROSER2	12	2
Q5SRP5	Apolipoprotein M C	APOM	58	7
A0A6Q8PFJ0	Prelamin-A/C OS=H	LMNA	11	7
E9PJC7	Tetraspanin (Fragm	CD82	8	1
A0A5H1ZRQ2	RNA helicase OS=H	DDX17	3	1
H7C3Y7	Neurobeachin-like	NBEAL2	2	2
C9J6A7	Ribulose-phosphate	RPE	10	2
K7EKM4	Spermidine synthas	SRM	9	1
G3V272	L-2-hydroxyglutarat	L2HGDH	3	1
K7EPJ4	Cartilage intermedi	CILP2	2	2
B4DIP2	Erbin OS=Homo sap	ERBIN	1	1
E7ENL6	Collagen alpha-3(VI	COL6A3	16	41
G8JL96	Protein-tyrosine-ph	PTPRS	4	4

A0A087WZF1	Lipoma-preferred p	LPP	12	4
A0A7P0TB04	Basigin OS=Homo s	BSG	11	2
D6R9Z1	Receptor of-activat	RACK1	5	1
C9JQD4	Peptidyl-prolyl cis-t	PPIH	7	1
D6RA82	Annexin OS=Homo	ANXA3	33	9
F8W876	Mannan-binding lec	MASP1	43	11
A0A087WTD7	A-kinase anchor pro	AKAP13	1	1
H3BP35	Diphosphomevalon	MVD	8	2
A0A087WXC4	Tenascin-N OS=Hon	TNN	1	1
B4DNG0	Olfactomedin-like p	OLFML3	6	2
H7C003	Protein phosphatas	PPP1R7	9	3
C9IZG4	Protein CutA OS=Hc	CUTA	10	1
H7C2Z8	Rho GTPase-activat	ARHGAP4	3	1
A0A087WTA8	Collagen alpha-2(I)	COL1A2	2	2
J3QTR3	Ubiquitin-40S ribos	RPS27A	17	2
A0A140TA68	Receptor-type tyros	PTPRC	6	3
A0A3B3ISV5	Uncharacterized pr	--	4	1
C9JVE2	DCN1-like protein C	DCUN1D1	8	2
B0QYF8	Myoglobin (Fragme	MB	34	4
A0A7P0T989	Cathepsin Z OS=Hoı	CTSZ	15	4
A0A2R8Y4Z1	Protein HEG homol	HEG1	9	2
A0A3B3IUC4	Alpha-galactosidase	GLA	2	1
A0A7I2YQK6	60 kDa chaperonin	HSPD1	24	6
H0YBS3	Golgi-resident aden	BPNT2	12	1
A0A0A0MSQ0	Plastin-3 OS=Homo	PLS3	19	14
C9JH19	Cathepsin D OS=Ho	CTSD	20	9
H0YM70	Proteasome activat	PSME2	4	1
E9PGZ1	Caldesmon OS=Hon	CALD1	29	16
A0A3B3ISU0	Desmocollin-2 OS=I	DSC2	6	4
A0A0B4J2A0	FGGY_N domain-co	--	3	1
G3V180	Dipeptidyl peptidas	DPP3	8	6
A0A1B0GVH5	Alpha-ketoglutarate	FTO	1	1
C9JZY6	Ubiquitin-conjugati	UBE2H	12	1
F5H442	Tumor susceptibilit	TSG101	3	1
H0Y8C6	Importin-5 (Fragme	IPO5	2	2
E7EWX8	Monoglyceride lipa	MGLL	7	2
F5H3Z4	Folate receptor bet	FOLR2	10	2
E7EWW9	Glutathione transfe	GSTM1	27	8
K7ER74	Apolipoprotein C-II	APOC4-APOC2	40	9
A0A286YEY5	Immunoglobulin he	IGHA2	41	17
A0A0G2JIW1	Heat shock 70 kDa		HSPA1B	39	22
Q53YU7	Foveolin OS=Homo	GKN1	11	2
A0A494C1A0	Alpha-actinin-2 (Fra	ACTN2	16	17
J3QRU1	Tyrosine-protein kir	YES1	7	5
A0A140T8Z4	Lymphocyte antige	LY6G6D	7	2
A0A096LP69	CD99 antigen OS=H	CD99	38	3
H7BY55	Complement decay	CD55	13	7
F8W809	Thioredoxin-disulfic	TXNRD1	9	4
C9K052	Secernin-1 (Fragme	SCRN1	3	1
F8WAG2	Serine/threonine-pı	NEK7	16	1

E7ESC6	Exportin-7 OS=Hom	XPO7	3	3
G3V4G9	Ig-like domain-cont	--	5	1
A0A3B3IUA6	Vitamin K-depende	PROS1	37	5
A0A6Q8PHA5	Histidine--tRNA liga	HARS1	7	4
A0A0A0MRV4	Prostamide/prostag	PRXL2B	7	1
B7ZM79	PCDH9 protein OS=	PCDH9	1	1
H0YDU8	Serine/threonine-p	PPP5C	2	1
F6T1Q0	2',5'-phosphodieste	PDE12	3	1
F8WAN9	GMP reductase OS=	GMPR2	6	2
H7C096	BCL2/adenovirus E1	BNIP2	8	2
H0YNE9	Ras-related protein	RAB8B	24	5
A0A7I2V5Y5	Kinesin-like protein	KIF23	1	1
Q5SZE1	Ceramide synthase	CERS2	5	1
A0A0A0MSN4	Angiotensin-conver	ACE	10	12
M0QX76	40S ribosomal prot	RPS16	20	1
E9PHX8	Receptor protein-ty	MERTK	1	1
D6RBW1	Eukaryotic translati	EIF4E	7	2
D6RFN0	COP9 signalosome	COPS4	11	4
A0A1W2PNX8	Protein unc-45 hom	UNC45A	5	6
A0A3B3IS75	Tartrate-resistant a	ACP5	27	1
E9PNJ7	Nucleosome assem	NAP1L4	40	5
F8WF18	Eukaryotic translati	EIF2A	17	1
G3V3E8	Epididymal secreto	NPC2	24	4
Q5TCU3	Tropomyosin beta	TPM2	33	11
F5H5V4	26S proteasome no	PSMD9	16	2
H0Y7Z1	Fibronectin (Fragm	FN1	56	65
H7C1A3	Fibulin-2 (Fragment	FBLN2	12	1
C9JMK5	Phosphoinositide-3	PIK3IP1	8	1
M0R009	Alpha-1B-glycoprot	A1BG	53	23
E9PK34	Coatomer subunit	ARCN1	12	1
H7C1W1	Peroxidasin homolo	PXDN	6	4
A0A0G2JMB2	Immunoglobulin he	IGHA2	52	19
A0A087WY55	Chromosome 6 ope	VTA1	3	1
I3L0A0	HCG2044781 OS=H	PEDS1-UBE2V1	9	3
A0A2R8YDP2	Casein kinase II sub	CSNK2A1	4	1
E9PLT1	Glycoprotein IIIb O	CD36	8	3
B1AKQ8	Guanine nucleotide	GNB1	48	4
H0Y7G9	Serine protease HT	HTRA1	4	1
E9PLK3	Aminopeptidase OS	NPEPPS	21	18
E5RGS4	Prefoldin subunit 1	PFDN1	9	1
Q5TAW7	Calcium-binding pr	CAB39L	8	2
B7Z2U2	TOM1-like protein	TOM1L2	9	4
A0A0B4J231	Immunoglobulin la	IGLL5	25	11
A0A7P0T906	ATP-dependent (S)-	NAXD	2	1
A0A087WZ85	Roundabout homol	ROBO1	1	1
A0A0G2JMX5	Leukocyte immuno	LILRB5	9	4
B4DP31	Phosphoribosyl pyr	PRPSAP1	6	1
C4P0D4	Disrupted in schizo	TSNAX-DISC1	16	2
Q5T123	SH3 domain-bindin	SH3BGRL3	38	5
A0A494C0F5	V-set and immunog	VSIG4	15	3

A0A024R571	EH domain-containi	EHD1	27	14
A0A087X2J4	Vacuolar protein so	VPS11	4	1
I3L145	Sex hormone-bindi	SHBG	27	9
A0A140T9L8	C6orf25 OS=Homo	MPIG6B	35	6
E9PCN2	Guanylate cyclase (	GUCY1B1	2	1
F8W1A2	Protein kinase C-bir	NELL2	3	1
D6R9W4	Drebrin (Fragment)	DBN1	6	2
A0A2R8Y7U1	Tripeptidyl aminop	TPP1	4	2
J3KP74	Proteoglycan 4 (Fra	PRG4	6	4
A0A087X0K1	Calcium-binding pro	CAB39	14	5
A0A2R8YFQ7	Lambda-crystallin h	CRYL1	8	2
B5MDF5	GTP-binding nuclea	RAN	28	7
Q6ZN40	Tropomyosin 1 (Alp	TPM1	30	12
E7EUF1	Ectonucleotide pyro	ENPP2	13	10
F8VXI7	Dynein light chain (I	DYNLL1	52	1
E9PG02	Inactive C-alpha-for	SUMF2	11	2
A0A0J9YWQ1	Rab-interacting lyso	RILP	4	1
A0A087WVQ6	Clathrin heavy chai	CLTC	3	6
A0A140T902	Tenascin-X OS=Hon	TNXB	15	37
E7EV05	AN1-type zinc finge	ZFAND2B	3	1
Q5W0H4	Translationally-con	TPT1	16	3
H7BXH2	Serine/threonine-p	PPP6R3	1	1
A0A7P0T9B0	Anthrax toxin recep	ANTXR1	7	2
J3KPA1	Cysteine-rich secret	CRISP3	29	8
A0A3B3IRS3	Guanylate-binding p	GBP7	4	2
E7EM64	COP9 signalosome	COPS6	7	2
P01833	Polymeric immunog	PIGR	6	4
Q15147	1-phosphatidylinosi	PLCB4	1	1
P25786	Proteasome subuni	PSMA1	35	9
P07225	Vitamin K-depende	PROS1	38	24
P36873	Serine/threonine-p	PPP1CC	15	6
Q8TER0	Sushi, nidogen and	SNED1	1	1
P08294	Extracellular superc	SOD3	51	11
P54920	Alpha-soluble NSF a	NAPA	25	6
P02549	Spectrin alpha chai	SPTA1	7	15
Q9BYE4	Small proline-rich p	SPRR2G	36	2
Q6UX71	Plexin domain-cont	PLXDC2	17	8
O00194	Ras-related protein	RAB27B	10	2
P07998	Ribonuclease pancr	RNASE1	40	4
P60903	Protein S100-A10 O	S100A10	10	1
P23526	Adenosylhomocyst	AHCY	21	9
Q6ZMJ2	Scavenger receptor	SCARA5	2	1
P48061	Stromal cell-derive	CXCL12	9	1
Q9HAT2	Sialate O-acetylest	SIAE	14	6
P12931	Proto-oncogene tyr	SRC	15	9
Q5R3I4	Tetratricopeptide r	TTC38	2	1
Q6GTS8	N-fatty-acyl-amino	PM20D1	8	4
Q99436	Proteasome subuni	PSMB7	6	2
P28072	Proteasome subuni	PSMB6	9	2
Q06830	Peroxiredoxin-1 OS	PRDX1	55	11

P49908	Selenoprotein P OS	SELENOP	17	8
P50453	Serpin B9 OS=Homo	SERPINB9	25	9
Q5T5C0	Syntaxin-binding pr	STXBP5	1	2
Q9NR12	PDZ and LIM domai	PDLIM7	13	5
P32119	Peroxiredoxin-2 OS	PRDX2	28	7
O00487	26S proteasome no	PSMD14	6	2
P31948	Stress-induced-pho	STIP1	23	13
Q01970	1-phosphatidylinosi	PLCB3	4	4
P48556	26S proteasome no	PSMD8	2	1
P51148	Ras-related protein	RAB5C	14	3
Q9Y3A5	Ribosome maturati	SBDS	10	3
P0DJI8	Serum amyloid A-1	SAA1	61	12
Q92854	Semaphorin-4D OS	SEMA4D	1	1
Q15019	Septin-2 OS=Homo	SEPTIN2	23	7
Q02383	Semenogelin-2 OS=	SEMG2	13	5
P10124	Serglycin OS=Homo	SRGN	17	3
Q14515	SPARC-like protein	SPARCL1	20	11
Q6GMV3	Putative peptidyl-tF	PTRHD1	12	2
P05451	Lithostathine-1-alpl	REG1A	7	1
Q9BW04	Specifically androge	SARG	5	1
O75326	Semaphorin-7A OS=	SEMA7A	4	3
Q16181	Septin-7 OS=Homo	SEPTIN7	35	12
O75368	SH3 domain-binding	SH3BGRL	49	6
P02814	Submaxillary gland	SMR3B	18	1
P60900	Proteasome subuni	PSMA6	33	7
P16885	1-phosphatidylinosi	PLCG2	1	1
P49593	Protein phosphatas	PPM1F	8	3
Q9HCY8	Protein S100-A14 O	S100A14	28	3
Q96P63	Serpin B12 OS=Hon	SERPINB12	10	4
P54136	Arginine--tRNA ligas	RARS1	3	2
P63000	Ras-related C3 botu	RAC1	46	8
Q8IW75	Serpin A12 OS=Hon	SERPINA12	4	2
P11277	Spectrin beta chain,	SPTB	3	6
O15031	Plexin-B2 OS=Homo	PLXNB2	3	5
P14923	Junction plakoglobi	JUP	36	24
Q00577	Transcriptional acti	PURA	3	1
Q15404	Ras suppressor prot	RSU1	33	9
Q9HBR0	Putative sodium-co	SLC38A10	7	6
Q14247	Src substrate cortac	CTTN	51	25
Q9NSE4	Isoleucine--tRNA lig	IARS2	1	1
Q6UXB8	Peptidase inhibitor	PI16	15	6
P48426	Phosphatidylinosito	PIP4K2A	22	8
P62906	60S ribosomal prote	RPL10A	6	1
Q9NR31	GTP-binding protein	SAR1A	12	2
Q9P270	SLAIN motif-contair	SLAIN2	3	1
Q13043	Serine/threonine-pi	STK4	8	3
O43776	Asparagine--tRNA li	NARS1	7	3
P36639	7,8-dihydro-8-oxogi	NUDT1	7	1
P36405	ADP-ribosylation fa	ARL3	12	2
Q9Y5C1	Angiopoietin-relate	ANGPTL3	8	3

P61604	10 kDa heat shock p	HSPE1	38	5
P20023	Complement recep	CR2	15	13
Q7L591	Docking protein 3 C	DOK3	9	4
P50148	Guanine nucleotide	GNAQ	13	4
O60234	Glia maturation fac	GMFG	27	4
P33151	Cadherin-5 OS=Hon	CDH5	13	9
P36222	Chitinase-3-like pro	CHI3L1	11	4
Q9UKY7	Protein CDV3 homo	CDV3	35	6
Q96KP4	Cytosolic non-speci	CNDP2	15	6
P21709	Ephrin type-A recep	EPHA1	2	1
Q08830	Fibrinogen-like prot	FGL1	31	9
Q12841	Follistatin-related p	FSTL1	15	5
P16401	Histone H1.5 OS=H	H1-5	10	2
P01889	HLA class I histocon	HLA-B	33	10
P00739	Haptoglobin-relate	HPR	42	17
P31937	3-hydroxyisobutyra	HIBADH	8	2
P31947	14-3-3 protein sigm	SFN	18	6
Q01433	AMP deaminase 2 (	AMPD2	6	6
Q13895	Bystin OS=Homo sa	BYSL	3	1
Q6ZUT6	Coiled-coil domain-	CCDC9B	3	1
P29692	Elongation factor 1-	EEF1D	9	2
P36980	Complement factor	CFHR2	38	10
Q03591	Complement factor	CFHR1	41	15
P14324	Farnesyl pyrophosp	FDPS	10	4
P19440	Glutathione hydrol	GGT1	8	4
Q02413	Desmoglein-1 OS=H	DSG1	22	18
P19823	Inter-alpha-trypsin	ITIH2	42	60
P13798	Acylamino-acid-rele	APEH	6	5
Q8IZ83	Aldehyde dehydrog	ALDH16A1	7	5
P63151	Serine/threonine-p	PPP2R2A	4	2
P52209	6-phosphogluconat	PGD	27	11
P53396	ATP-citrate synthas	ACLY	11	12
P67870	Casein kinase II sub	CSNK2B	12	2
P05160	Coagulation factor)	F13B	49	28
P00742	Coagulation factor)	F10	27	12
P15170	Eukaryotic peptide	GSPT1	6	3
Q12805	EGF-containing fibu	EFEMP1	43	17
A0A0B4J2D5	Glutamine amidotr	GATD3B	17	3
P10912	Growth hormone re	GHR	2	1
P19086	Guanine nucleotide	GNAZ	6	2
P13224	Platelet glycoprotei	GP1BB	5	1
Q9Y251	Heparanase OS=Ho	HPSE	17	9
P01344	Insulin-like growth	IGF2	18	3
Q8IZP0	Abl interactor 1 OS=	ABI1	14	6
Q8TD06	Anterior gradient p	AGR3	10	2
P08571	Monocyte different	CD14	34	10
P10645	Chromogranin-A OS	CHGA	13	5
P06681	Complement C2 OS	C2	41	27
Q01459	Di-N-acetylchitobia	CTBS	24	8
O94919	Endonuclease dom	ENDOD1	15	6

Q8NI99	Angiopoietin-relate	ANGPTL6	14	5
P05023	Sodium/potassium-	ATP1A1	1	2
O43852	Calumenin OS=Hom	CALU	25	7
O95810	Caveolae-associate	CAVIN2	37	14
P55290	Cadherin-13 OS=Ho	CDH13	21	14
O00299	Chloride intracellul	CLIC1	71	15
O94985	Calsyntenin-1 OS=H	CLSTN1	7	7
Q14118	Dystroglycan OS=H	DAG1	8	6
P31946	14-3-3 protein beta	YWHAB	48	13
P00326	Alcohol dehydroger	ADH1C	22	11
P61769	Beta-2-microglobul	B2M	46	12
Q8N1Q1	Carbonic anhydrase	CA13	3	1
Q8NEX9	Short-chain dehydr	SDR9C7	7	2
Q9BX68	Histidine triad nucl	HINT2	15	2
P01876	Immunoglobulin he	IGHA1	55	28
A0A0C4DH32	Immunoglobulin he	IGHV3-20	17	2
P40926	Malate dehydrogen	MDH2	25	8
P14151	L-selectin OS=Hom	SELL	19	6
P16581	E-selectin OS=Hom	SELE	7	3
P27816	Microtubule-associ	MAP4	3	2
P08727	Keratin, type I cyto	KRT19	21	14
P53990	IST1 homolog OS=H	IST1	10	3
O00629	Importin subunit al	KPNA4	3	2
P49327	Fatty acid synthase	FASN	7	17
Q92820	Gamma-glutamyl h	GGH	33	11
P32754	4-hydroxyphenylpy	HPD	26	10
P22692	Insulin-like growth	IGFBP4	38	8
Q14CN4	Keratin, type II cyto	KRT72	9	6
P04746	Pancreatic alpha-an	AMY2A	10	4
P05089	Arginase-1 OS=Hom	ARG1	9	3
P12110	Collagen alpha-2(VI	COL6A2	1	1
P13671	Complement comp	C6	51	43
P07360	Complement comp	C8G	55	11
P42574	Caspase-3 OS=Hom	CASP3	17	4
P07327	Alcohol dehydroger	ADH1A	24	10
Q86SQ4	Adhesion G-protein	ADGRG6	6	6
P59998	Actin-related protei	ARPC4	28	5
P02748	Complement comp	C9	46	29
Q12860	Contactin-1 OS=Ho	CNTN1	24	23
Q12864	Cadherin-17 OS=Ho	CDH17	3	2
P07451	Carbonic anhydrase	CA3	15	2
P08603	Complement factor	CFH	67	108
Q8IY22	C-Maf-inducing pro	CMIP	5	3
O95433	Activator of 90 kDa	AHSA1	6	2
Q03154	Aminoacylase-1 OS	ACY1	19	8
Q6UB99	Ankyrin repeat dom	ANKRD11	1	1
P04083	Annexin A1 OS=Hor	ANXA1	26	8
P15289	Arylsulfatase A OS=	ARSA	6	2
O15511	Actin-related protei	ARPC5	58	6
P07357	Complement comp	C8A	36	17

Q9Y240	C-type lectin domai	CLEC11A	4	1
P07307	Asialoglycoprotein r	ASGR2	16	3
P25705	ATP synthase subur	ATP5F1A	7	4
P47755	F-actin-capping pro	CAPZA2	31	7
Q9NTU7	Cerebellin-4 OS=Ho	CBLN4	6	1
P10909	Clusterin OS=Homo	CLU	45	30
O15551	Claudin-3 OS=Hom	CLDN3	6	1
Q99704	Docking protein 1 C	DOK1	9	3
Q96FN4	Copine-2 OS=Homo	CPNE2	3	2
P23528	Cofilin-1 OS=Homo	CFL1	64	18
P07741	Adenine phosphoril	APRT	19	3
Q9NP61	ADP-ribosylation fa	ARFGAP3	2	1
Q9H4A4	Aminopeptidase B (	RNPEP	5	3
P08519	Apolipoprotein(a) C	LPA	40	29
Q9P232	Contactin-3 OS=Ho	CNTN3	7	7
Q8IWV2	Contactin-4 OS=Ho	CNTN4	7	6
A8K7I4	Calcium-activated c	CLCA1	4	4
Q9NQ79	Cartilage acidic pro	CRTAC1	28	14
P30043	Flavin reductase (N	BLVRB	40	7
P13501	C-C motif chemokin	CCL5	12	1
P12830	Cadherin-1 OS=Hon	CDH1	10	8
P55273	Cyclin-dependent k	CDKN2D	7	1
P60953	Cell division control	CDC42	25	4
Q9BRF8	Serine/threonine-p	CPPED1	9	3
P53621	Coatomer subunit a	COPA	4	4
P04424	Argininosuccinate ly	ASL	15	10
P49407	Beta-arrestin-1 OS=	ARRB1	15	6
Q5VTR2	E3 ubiquitin-protein	RNF20	1	1
Q9UI42	Carboxypeptidase A	CPA4	2	1
P20160	Azurocidin OS=Hom	AZU1	3	1
Q09666	Neuroblast differen	AHNAK	3	5
O60488	Long-chain-fatty-ac	ACSL4	2	1
P06727	Apolipoprotein A-IV	APOA4	76	46
P50995	Annexin A11 OS=Ho	ANXA11	10	5
P15144	Aminopeptidase N (	ANPEP	31	30
P14384	Carboxypeptidase M	CPM	3	2
O15335	Chondroadherin OS	CHAD	7	2
P01024	Complement C3 OS	C3	75	215
Q12882	Dihydropyrimidine	DPYD	3	3
Q9BT09	Protein canopy hon	CNPY3	7	2
Q9UHL4	Dipeptidyl peptidas	DPP7	7	3
Q8TEA8	D-aminoacyl-tRNA (	DTD1	18	4
P02750	Leucine-rich alpha-2	LRG1	43	17
P68032	Actin, alpha cardiac	ACTC1	40	26
Q13790	Apolipoprotein F OS	APOF	12	3
P09525	Annexin A4 OS=Hor	ANXA4	18	6
P43251	Biotinidase OS=Hon	BTD	19	9
P55957	BH3-interacting dor	BID	12	2
P08236	Beta-glucuronidase	GUSB	8	5
Q96MW1	Coiled-coil domain-	CCDC43	9	2

P55212	Caspase-6 OS=Hom	CASP6	3	1
Q13098	COP9 signalosome (	GPS1	4	2
Q15067	Peroxisomal acyl-cc	ACOX1	1	1
Q99941	Cyclic AMP-depend	ATF6B	5	3
O15145	Actin-related protei	ARPC3	26	6
O15144	Actin-related protei	ARPC2	39	12
O43866	CD5 antigen-like OS	CD5L	40	11
Q9Y6Z7	Collectin-10 OS=Ho	COLEC10	12	3
P04080	Cystatin-B OS=Hom	CSTB	70	5
Q06481	Amyloid-like protei	APLP2	4	3
O95445	Apolipoprotein M C	APOM	56	9
Q07075	Glutamyl aminopep	ENPEP	4	4
O43150	Arf-GAP with SH3 d	ASAP2	2	2
P0C0L4	Complement C4-A (	C4A	57	123
Q9BPX5	Actin-related protei	ARPC5L	13	2
Q93088	Betaine--homocyste	BHMT	11	4
P07858	Cathepsin B OS=Ho	CTSB	25	10
P09668	Pro-cathepsin H OS	CTSH	10	4
Q9Y259	Choline/ethanolam	CHKB	5	2
O00501	Claudin-5 OS=Homo	CLDN5	6	1
O75131	Copine-3 OS=Homo	CPNE3	3	2
Q9P1F3	Costars family prote	ABRACL	16	2
Q96IU4	Protein ABHD14B C	ABHD14B	31	5
P02760	Protein AMBP OS=H	AMBP	44	20
P48960	Adhesion G protein	ADGRE5	2	1
Q9NZP8	Complement C1r su	C1RL	25	10
P50238	Cysteine-rich protei	CRIP1	9	1
Q14393	Growth arrest-spec	GAS6	2	1
Q9UBW5	Bridging integrator	BIN2	32	15
P08311	Cathepsin G OS=Ho	CTSG	9	2
P09871	Complement C1s su	C1S	41	29
P06276	Cholinesterase OS=	BCHE	23	12
P11597	Cholesteryl ester tr	CETP	13	6
P32320	Cytidine deaminase	CDA	10	1
P01031	Complement C5 OS	C5	39	65
Q9ULV4	Coronin-1C OS=Hor	CORO1C	32	19
B5ME19	Eukaryotic translati	EIF3CL	2	2
Q00688	Peptidyl-prolyl cis-t	FKBP3	28	5
P30153	Serine/threonine-p	PPP2R1A	12	6
Q9NP58	ATP-binding cassett	ABCB6	1	1
P21399	Cytoplasmic aconita	ACO1	5	4
P27482	Calmodulin-like pro	CALML3	32	4
P09326	CD48 antigen OS=H	CD48	3	1
P19022	Cadherin-2 OS=Hon	CDH2	8	7
O75339	Cartilage intermedi	CILP	3	3
Q8IUI8	Cytokine receptor-l	CRLF3	10	4
P15924	Desmoplakin OS=H	DSP	26	78
Q8NEU8	DCC-interacting pro	APPL2	5	2
P00352	Retinal dehydrogen	ALDH1A1	38	15
Q8TD30	Alanine aminotrans	GPT2	6	3

O00468	Agrin OS=Homo sap	AGRN	2	3
P61160	Actin-related protei	ACTR2	35	11
Q9NZT1	Calmodulin-like pro	CALML5	42	4
Q9BY67	Cell adhesion mole	CADM1	10	4
Q03701	CCAAT/enhancer-bi	CEBPZ	1	1
O75356	Ectonucleoside trip	ENTPD5	3	1
P07148	Fatty acid-binding p	FABP1	39	6
Q13451	Peptidyl-prolyl cis-t	FKBP5	8	3
O14561	Acyl carrier protein,	NDUFAB1	9	1
O43747	AP-1 complex subu	AP1G1	1	1
P28838	Cytosol aminopepti	LAP3	25	11
P08758	Annexin A5 OS=Hor	ANXA5	24	8
P13497	Bone morphogenet	BMP1	5	5
Q9HCK8	Chromodomain-hel	CHD8	1	2
P02452	Collagen alpha-1(I)	COL1A1	5	6
P04406	Glyceraldehyde-3-p	GAPDH	65	26
Q99999	Galactosylceramide	GAL3ST1	2	1
Q8N335	Glycerol-3-phospha	GPD1L	5	2
Q9NZD4	Alpha-hemoglobin-s	AHSP	42	4
O14791	Apolipoprotein L1 C	APOL1	27	13
P50583	Bis(5'-nucleosyl)-te	NUDT2	5	1
P15291	Beta-1,4-galactosylt	B4GALT1	9	3
P41240	Tyrosine-protein kir	CSK	19	7
P42126	Enoyl-CoA delta iso	ECI1	3	1
Q9NV70	Exocyst complex co	EXOC1	1	1
P31751	RAC-beta serine/th	AKT2	5	3
Q15848	Adiponectin OS=Ho	ADIPOQ	12	2
P02730	Band 3 anion trans	SLC4A1	12	10
P46109	Crk-like protein OS=	CRKL	18	5
Q7L576	Cytoplasmic FMR1-	CYFIP1	10	12
Q8IXL6	Extracellular serine,	FAM20C	4	2
P17174	Aspartate aminotra	GOT1	34	11
P28332	Alcohol dehydroger	ADH6	2	1
P49913	Cathelicidin antimic	CAMP	29	7
P07384	Calpain-1 catalytic s	CAPN1	37	26
Q14315	Filamin-C OS=Homo	FLNC	4	13
Q92496	Complement factor	CFHR4	21	9
P01008	Antithrombin-III OS	SERPINC1	53	52
O43505	Beta-1,4-glucuronyl	B4GAT1	12	5
P02747	Complement C1q su	C1QC	36	8
P24387	Corticotropin-relea	CRHBP	16	4
P02741	C-reactive protein C	CRP	19	5
Q14203	Dynactin subunit 1	DCTN1	1	1
Q16531	DNA damage-bindir	DDB1	6	7
Q8TDD1	ATP-dependent RN	DDX54	2	2
Q9BPW9	Dehydrogenase/rec	DHRS9	2	1
Q9BXR6	Complement factor	CFHR5	35	17
Q96RD9	Fc receptor-like pro	FCRL5	4	3
P04217	Alpha-1B-glycoprot	A1BG	51	33
P61163	Alpha-centractin OS	ACTR1A	3	1

Q06187	Tyrosine-protein ki	BTK	22	14
P80723	Brain acid soluble p	BASP1	30	4
P04003	C4b-binding proteir	C4BPA	62	36
P19876	C-X-C motif chemol	CXCL3	12	1
P26196	Probable ATP-depe	DDX6	2	1
P63104	14-3-3 protein zeta,	YWHAZ	67	19
P04075	Fructose-bisphosph	ALDOA	69	28
P31749	RAC-alpha serine/tl	AKT1	4	2
Q9NR19	Acetyl-coenzyme A	ACSS2	3	2
P61158	Actin-related protei	ACTR3	35	12
O75882	Attractin OS=Homo	ATRN	24	33
O76071	Probable cytosolic i	CIAO1	2	1
P12111	Collagen alpha-3(VI	COL6A3	14	43
Q76LX8	A disintegrin and m	ADAMTS13	10	12
P10643	Complement comp	C7	54	39
P07358	Complement comp	C8B	45	24
P06576	ATP synthase subur	ATP5F1B	19	9
Q9H2M3	S-methylmethionin	BHMT2	9	3
O75531	Barrier-to-autointe	BANF1	24	2
Q86WR0	Coiled-coil domain-	CCDC25	4	1
P54289	Voltage-dependent	CACNA2D1	16	16
Q6YHK3	CD109 antigen OS=	CD109	10	14
Q9Y3E7	Charged multivesic	CHMP3	8	2
P22792	Carboxypeptidase N	CPN2	24	14
Q9BR76	Coronin-1B OS=Hor	CORO1B	22	9
Q08554	Desmocollin-1 OS=I	DSC1	16	11
P55884	Eukaryotic translati	EIF3B	7	5
P00505	Aspartate aminotra	GOT2	11	4
P14550	Aldo-keto reductas	AKR1A1	18	5
P54802	Alpha-N-acetylgluc	NAGLU	9	6
P62330	ADP-ribosylation fa	ARF6	10	2
Q10588	ADP-ribosyl cyclase	BST1	21	6
P16152	Carbonyl reductase	CBR1	17	4
Q14574	Desmocollin-3 OS=I	DSC3	3	3
O95336	6-phosphogluconol	PGLS	37	8
P49189	4-trimethylaminob	ALDH9A1	2	1
P12814	Alpha-actinin-1 OS=	ACTN1	50	48
P53004	Biliverdin reductase	BLVRA	21	5
Q9NY97	N-acetyllactosamin	B3GNT2	19	5
P08185	Corticosteroid-bind	SERPINA6	25	12
Q9H0W9	Ester hydrolase C11	C11orf54	6	2
Q96HD1	Protein disulfide isc	CRELD1	6	2
Q13618	Cullin-3 OS=Homo s	CUL3	1	1
P00367	Glutamate dehydro	GLUD1	6	4
P09172	Dopamine beta-hyc	DBH	27	11
P06733	Alpha-enolase OS=I	ENO1	40	22
P03951	Coagulation factor	F11	38	22
Q562R1	Beta-actin-like prot	ACTBL2	35	18
O43488	Aflatoxin B1 aldehy	AKR7A2	24	7
O15143	Actin-related protei	ARPC1B	36	11

Q01518	Adenylyl cyclase-as:	CAP1	45	21
P13688	Carcinoembryonic a	CEACAM1	8	3
P00746	Complement factor	CFD	42	9
Q9BY43	Charged multivesicı	CHMP4A	7	1
Q9UHD1	Cysteine and histidi	CHORDC1	7	2
Q99459	Cell division cycle 5	CDC5L	1	1
P49747	Cartilage oligomeric	COMP	38	21
P31146	Coronin-1A OS=Hor	CORO1A	36	18
P51452	Dual specificity pro	DUSP3	26	4
Q96GK7	Fumarylacetoaceta	FAHD2A	4	1
P15121	Aldo-keto reductas	AKR1B1	16	5
Q9ULH1	Arf-GAP with SH3 d	ASAP1	3	3
Q93084	Sarcoplasmic/endo	ATP2A3	1	1
O00499	Myc box-dependen	BIN1	3	3
P20851	C4b-binding proteir	C4BPB	41	9
P55291	Cadherin-15 OS=Ho	CDH15	1	1
Q7LBR1	Charged multivesicı	CHMP1B	4	1
Q9NR30	Nucleolar RNA helic	DDX21	1	1
Q16610	Extracellular matrix	ECM1	57	23
P01009	Alpha-1-antitrypsin	SERPINA1	54	40
Q8NFV4	Protein ABHD11 OS	ABHD11	5	2
A8K2U0	Alpha-2-macroglob	A2ML1	1	2
P62258	14-3-3 protein epsil	YWHAE	61	16
Q99424	Peroxisomal acyl-cc	ACOX2	3	2
P40394	All-trans-retinol del	ADH7	2	1
P11766	Alcohol dehydroger	ADH5	26	11
Q9UEY8	Gamma-adducin OS	ADD3	2	1
P43652	Afamin OS=Homo s	AFM	52	37
P02656	Apolipoprotein C-III	APOC3	69	10
P55774	C-C motif chemokin	CCL18	9	1
P27797	Calreticulin OS=Hor	CALR	53	22
P52907	F-actin-capping pro	CAPZA1	38	7
P00918	Carbonic anhydrase	CA2	47	13
Q96KN2	Beta-Ala-His dipept	CNDP1	35	15
P81605	Dermcidin OS=Hom	DCD	41	3
P61221	ATP-binding cassett	ABCE1	2	1
P09972	Fructose-bisphosph	ALDOC	29	13
Q16853	Membrane primary	AOC3	17	13
O95861	3'(2'),5'-bisphospha	BPNT1	3	1
Q96CG8	Collagen triple helix	CTHRC1	7	2
P08319	All-trans-retinol del	ADH4	28	10
P35858	Insulin-like growth	IGFALS	36	20
Q96P48	Arf-GAP with Rho-G	ARAP1	3	4
P02647	Apolipoprotein A-I (	APOA1	81	64
P07355	Annexin A2 OS=Hor	ANXA2	42	12
Q6Q788	Apolipoprotein A-V	APOA5	5	2
P00966	Argininosuccinate s	ASS1	5	2
P11021	Endoplasmic reticul	HSPA5	44	28
P53634	Dipeptidyl peptidas	CTSC	12	5
Q9H3H3	UPF0696 protein C1	C11orf68	4	1

Q16543	Hsp90 co-chaperon	CDC37	14	5
Q9UDT6	CAP-Gly domain-co	CLIP2	1	1
Q6UVK1	Chondroitin sulfate	CSPG4	3	6
Q99715	Collagen alpha-1(XI	COL12A1	1	3
Q9UJU6	Drebrin-like protein	DBNL	34	12
Q9HB71	Calcyclin-binding pr	CACYBP	16	4
P01023	Alpha-2-macroglob	A2M	54	86
Q8IZF2	Adhesion G protein	ADGRF5	6	6
P61204	ADP-ribosylation fa	ARF3	34	7
P00915	Carbonic anhydrase	CA1	51	13
Q9H444	Charged multivesic	CHMP4B	16	3
Q9HCU4	Cadherin EGF LAG s	CELSR2	0	1
Q9BWP8	Collectin-11 OS=Ho	COLEC11	19	4
Q08495	Dematin OS=Homo	DMTN	23	7
Q96EP5	DAZ-associated pro	DAZAP1	4	1
P60981	Destrin OS=Homo s	DSTN	35	6
Q9H4A9	Dipeptidase 2 OS=H	DPEP2	14	6
Q9BS26	Endoplasmic reticul	ERP44	21	7
Q02985	Complement factor	CFHR3	31	12
P62993	Growth factor rece	GRB2	43	9
P05019	Insulin-like growth	IGF1	12	2
P09417	Dihydropteridine re	QDPR	24	5
Q13011	Delta(3,5)-Delta(2,4	ECH1	9	3
Q9UNN8	Endothelial protein	PROCR	19	4
Q16595	Frataxin, mitochon	FXN	6	1
P02749	Beta-2-glycoprotein	APOH	61	33
Q96IY4	Carboxypeptidase B	CPB2	28	11
Q9HBB8	Cadherin-related fa	CDHR5	1	1
P42830	C-X-C motif chemok	CXCL5	8	1
Q96C86	m7GpppX diphosph	DCPS	3	1
P26641	Elongation factor 1-	EEF1G	13	7
P16930	Fumarylacetoaceta	FAH	27	11
Q9H4G4	Golgi-associated pla	GLIPR2	28	3
Q9BX10	GTP-binding protein	GTPBP2	1	1
Q3LI77	Keratin-associated	KRTAP13-4	17	2
P06732	Creatine kinase M-t	CKM	27	10
P50502	Hsc70-interacting p	ST13	21	7
Q9NZ08	Endoplasmic reticul	ERAP1	16	14
P08709	Coagulation factor	F7	18	5
P01742	Immunoglobulin he	IGHV1-69	37	6
A0A0B4J1V2	Immunoglobulin he	IGHV2-26	6	1
P01780	Immunoglobulin he	IGHV3-7	45	10
A0A0B4J1V0	Immunoglobulin he	IGHV3-15	39	7
A0A0C4DH34	Immunoglobulin he	IGHV4-28	19	2
P06331	Immunoglobulin he	IGHV4-34	37	4
A0A0C4DH35	Probable non-funct	IGHV3-35	26	7
A0A0B4J1X8	Immunoglobulin he	IGHV3-43	21	3
A0A0A0MS15	Immunoglobulin he	IGHV3-49	44	6
P01767	Immunoglobulin he	IGHV3-53	35	5
A0A075B6Q5	Immunoglobulin he	IGHV3-64	29	4

Q9Y4L1	Hypoxia up-regulat	HYOU1	9	9
A0A0C4DH42	Immunoglobulin he	IGHV3-66	35	5
A0A0B4J1V6	Immunoglobulin he	IGHV3-73	28	3
P0DP04	Immunoglobulin he	IGHV3-43D	21	3
A0A0J9YXX1	Immunoglobulin he	IGHV5-10-1	15	3
O75874	Isocitrate dehydrog	IDH1	30	12
A0A0B4J1U7	Immunoglobulin he	IGHV6-1	18	2
A0A0J9YX35	Immunoglobulin he	IGHV3-64D	38	9
A0A0J9YVY3	Immunoglobulin he	IGHV7-4-1	31	3
P0DP03	Immunoglobulin he	IGHV3-30-5	32	4
P48735	Isocitrate dehydrog	IDH2	34	15
Q14116	Interleukin-18 OS=H	IL18	13	3
Q8N1N4	Keratin, type II cyto	KRT78	24	15
Q5XKE5	Keratin, type II cyto	KRT79	12	12
Q9NPH3	Interleukin-1 recep	IL1RAP	13	8
P07099	Epoxide hydrolase 1	EPHX1	6	2
O14775	Guanine nucleotide	GNB5	5	1
P21266	Glutathione S-trans	GSTM3	19	4
P06310	Immunoglobulin ka	IGKV2-30	31	5
P09382	Galectin-1 OS=Hom	LGALS1	35	4
P23919	Thymidylate kinase	DTYMK	4	1
P01011	Alpha-1-antichymo	SERPINA3	61	51
P24298	Alanine aminotrans	GPT	15	7
P31944	Caspase-14 OS=Hor	CASP14	19	4
P00403	Cytochrome c oxida	MT-CO2	4	1
P31327	Carbamoyl-phospha	CPS1	3	2
P14770	Platelet glycoprotei	GP9	13	2
Q02747	Guanylin OS=Homo	GUCA2A	17	2
P00338	L-lactate dehydroge	LDHA	39	17
P12259	Coagulation factor V	F5	25	51
Q9UBQ6	Exostosin-like 2 OS=	EXTL2	14	4
P30040	Endoplasmic reticul	ERP29	22	5
P00740	Coagulation factor I	F9	36	14
P30273	High affinity immun	FCER1G	23	2
P42685	Tyrosine-protein kir	FRK	3	2
P04259	Keratin, type II cyto	KRT6B	63	47
P48668	Keratin, type II cyto	KRT6C	60	46
P15586	N-acetylglucosamin	GNS	6	3
P40189	Interleukin-6 recep	IL6ST	4	4
P08729	Keratin, type II cyto	KRT7	6	5
P12277	Creatine kinase B-ty	CKB	17	5
P60709	Actin, cytoplasmic 1	ACTB	65	55
O15467	C-C motif chemokin	CCL16	20	2
P28906	Hematopoietic prog	CD34	2	1
P00748	Coagulation factor X	F12	32	19
P62942	Peptidyl-prolyl cis-t	FKBP1A	42	6
Q10471	Polypeptide N-acet	GALNT2	12	7
O75715	Epididymal secreto	GPX5	5	1
O76003	Glutaredoxin-3 OS=	GLRX3	15	4
O76013	Keratin, type I cutic	KRT36	25	12

A2NJV5	Immunoglobulin ka	IGKV2-29	40	6
Q14847	LIM and SH3 domai	LASP1	53	14
Q9NZN3	EH domain-containi	EHD3	38	19
P20930	Filaggrin OS=Homo	FLG	3	6
P07954	Fumarate hydratase	FH	7	4
Q14376	UDP-glucose 4-epin	GALE	2	1
P08238	Heat shock protein	HSP90AB1	27	18
P01591	Immunoglobulin J c	JCHAIN	35	5
P08648	Integrin alpha-5 OS	ITGA5	1	1
Q04760	Lactoylglutathione l	GLO1	29	6
Q96AG4	Leucine-rich repeat	LRRC59	3	1
P58107	Epiplakin OS=Homo	EPPK1	7	7
P46926	Glucosamine-6-pho	GNPDA1	15	3
P28676	Grancalcin OS=Hom	GCA	8	2
P11142	Heat shock cognate	HSPA8	54	29
Q96NU7	Probable imidazolo	AMDHD1	4	2
Q92598	Heat shock protein	HSPH1	5	4
P18065	Insulin-like growth f	IGFBP2	50	12
Q04695	Keratin, type I cyto:	KRT17	47	27
Q9UIJ7	GTP:AMP phosphot	AK3	25	5
Q52LG2	Keratin-associated p	KRTAP13-2	20	3
A0A0C4DH26	Probable non-funct	IGKV6D-41	10	1
P19256	Lymphocyte functic	CD58	4	1
Q13449	Limbic system-asso	LSAMP	22	7
P50570	Dynamin-2 OS=Hon	DNM2	4	3
Q96N76	Urocanate hydratas	UROC1	4	3
P07476	Involucrin OS=Hom	IVL	2	1
O95678	Keratin, type II cyto	KRT75	19	18
P08514	Integrin alpha-IIb O	ITGA2B	22	22
Q8WWA0	Intelectin-1 OS=Hor	ITLN1	17	6
P10644	cAMP-dependent p	PRKAR1A	11	5
Q9H8L6	Multimerin-2 OS=H	MMRN2	4	3
P49257	Protein ERGIC-53 O	LMAN1	10	4
Q8N423	Leukocyte immuno	LILRB2	5	2
Q9NWV4	CXXC motif contain	CZIB	11	1
O00429	Dynamin-1-like pro	DNM1L	18	12
Q16555	Dihydropyrimidinas	DPYSL2	30	13
Q96AP7	Endothelial cell-sel	ESAM	16	5
P38117	Electron transfer fla	ETFB	24	5
P23141	Liver carboxylester	CES1	21	10
O75369	Filamin-B OS=Homo	FLNB	2	8
Q9Y6R7	IgGFc-binding prote	FCGBP	29	78
P02671	Fibrinogen alpha ch	FGA	45	46
P19652	Alpha-1-acid glycop	ORM2	25	5
Q04917	14-3-3 protein eta (	YWHAH	35	11
P24534	Elongation factor 1-	EEF1B2	12	2
P59665	Neutrophil defensir	DEFA1	20	4
O43583	Density-regulated p	DENR	8	2
Q9H479	Fructosamine-3-kin	FN3K	21	5
Q9BTY2	Plasma alpha-L-fuc	FUCA2	17	8

P02794	Ferritin heavy chain	FTH1	32	4
Q9UJJ9	N-acetylglucosamin	GNPTG	25	6
P22352	Glutathione peroxic	GPX3	36	8
Q14019	Coactosin-like prote	COTL1	52	9
P13639	Elongation factor 2	EEF2	15	11
P00451	Coagulation factor '	F8	1	2
P35555	Fibrillin-1 OS=Homc	FBN1	11	26
O14672	Disintegrin and met	ADAM10	2	2
P03950	Angiogenin OS=Hor	ANG	48	6
Q15582	Transforming growt	TGFBI	25	15
Q7Z7M8	UDP-GlcNAc:betaGa	B3GNT8	7	2
P01034	Cystatin-C OS=Hom	CST3	55	9
Q9BTE1	Dynactin subunit 5	DCTN5	4	1
Q9BVJ7	Dual specificity pro	DUSP23	8	1
P15086	Carboxypeptidase E	CPB1	3	1
O75976	Carboxypeptidase D	CPD	1	1
Q15369	Elongin-C OS=Homc	ELOC	31	3
Q01469	Fatty acid-binding p	FABP5	69	9
O00757	Fructose-1,6-bisphc	FBP2	3	1
Q93063	Exostosin-2 OS=Hor	EXT2	6	4
Q9BYJ0	Fibroblast growth fa	FGFBP2	11	2
Q15485	Ficolin-2 OS=Homo	FCN2	14	4
O95479	GDH/6PGL endopla	H6PD	22	17
O43681	ATPase GET3 OS=H	GET3	3	1
Q8NBJ4	Golgi membrane pr	GOLM1	14	5
P61981	14-3-3 protein gam	YWHAG	45	12
P27348	14-3-3 protein theta	YWHAQ	52	15
P02649	Apolipoprotein E OS	APOE	74	34
P25774	Cathepsin S OS=Hor	CTSS	21	6
P15085	Carboxypeptidase A	CPA1	4	2
P0DP23	Calmodulin-1 OS=H	CALM1	44	9
Q9NPY3	Complement comp	CD93	18	8
Q13740	CD166 antigen OS=	ALCAM	21	10
Q15370	Elongin-B OS=Homc	ELOB	25	3
P55056	Apolipoprotein C-IV	APOC4	22	3
P04114	Apolipoprotein B-1(	APOB	56	298
Q7L1Q6	Basic leucine zipper	BZW1	6	3
P15169	Carboxypeptidase N	CPN1	43	16
P29279	CCN family membe	CCN2	9	3
O75390	Citrate synthase, m	CS	12	6
Q15828	Cystatin-M OS=Hon	CST6	28	3
Q96HE7	ERO1-like protein a	ERO1A	6	2
Q6ZVX7	F-box only protein !	NCCRP1	15	3
P55899	IgG receptor FcRn la	FCGRT	5	2
O00170	AH receptor-interac	AIP	4	1
Q9NUB1	Acetyl-coenzyme A	ACSS1	3	2
P20933	N(4)-(beta-N-acetyl	AGA	7	2
Q9Y6D5	Brefeldin A-inhibite	ARFGEF2	0	1
Q86VP6	Cullin-associated NI	CAND1	11	12
P02461	Collagen alpha-1(III	COL3A1	2	2

P04632	Calpain small subur	CAPNS1	14	3
P05141	ADP/ATP translocas	SLC25A5	19	4
P20073	Annexin A7 OS=Hor	ANXA7	8	4
P08133	Annexin A6 OS=Hor	ANXA6	6	5
Q92484	Acid sphingomyelin	SMPDL3A	7	3
P07711	Procathepsin L OS=	CTSL	11	3
P04040	Catalase OS=Homo	CAT	54	27
P00450	Ceruloplasmin OS=I	CP	62	88
Q5ZPR3	CD276 antigen OS=	CD276	3	1
Q9HCU0	Endosialin OS=Hom	CD248	6	5
P39060	Collagen alpha-1(X\	COL18A1	5	8
P00533	Epidermal growth f	EGFR	7	7
P02675	Fibrinogen beta cha	FGB	58	30
P02763	Alpha-1-acid glycop	ORM1	39	11
P08697	Alpha-2-antiplasmir	SERPINF2	43	28
Q12904	Aminoacyl tRNA syr	AIMP1	5	1
O15204	ADAM DEC1 OS=Hc	ADAMDEC1	3	1
Q9Y646	Carboxypeptidase C	CPQ	10	4
Q8N163	Cell cycle and apop	CCAR2	1	1
Q9BYE9	Cadherin-related fa	CDHR2	11	12
P26885	Peptidyl-prolyl cis-t	FKBP2	26	3
P06396	Gelsolin OS=Homo :	GSN	53	65
Q08211	ATP-dependent RN/	DHX9	1	1
O75636	Ficolin-3 OS=Homo	FCN3	35	9
O15117	FYN-binding proteir	FYB1	11	6
P49915	GMP synthase [glut	GMPS	1	1
P02768	Albumin OS=Homo	ALB	79	151
A1A5B4	Anoctamin-9 OS=H(	ANO9	1	1
P16157	Ankyrin-1 OS=Hom(	ANK1	5	9
P02654	Apolipoprotein C-I (	APOC1	49	8
Q08722	Leukocyte surface a	CD47	6	2
Q9Y696	Chloride intracellul	CLIC4	43	9
Q9H0R4	Haloacid dehalogen	HDHD2	12	3
P32019	Type II inositol 1,4,!	INPP5B	1	1
P19013	Keratin, type II cyto	KRT4	17	10
P21695	Glycerol-3-phospha	GPD1	13	4
P69891	Hemoglobin subuni	HBG1	60	11
Q92619	Rho GTPase-activat	ARHGAP45	8	7
Q14532	Keratin, type I cutic	KRT32	15	7
P53582	Methionine aminop	METAP1	8	2
O60268	Uncharacterized pr	KIAA0513	17	6
P00995	Serine protease inh	SPINK1	23	2
P02533	Keratin, type I cyto:	KRT14	58	39
P80217	Interferon-induced	IFI35	11	3
Q15555	Microtubule-associ	MAPRE2	29	8
Q9Y624	Junctional adhesion	F11R	10	3
P14618	Pyruvate kinase PKI	PKM	60	32
Q92876	Kallikrein-6 OS=Hor	KLK6	5	1
Q92520	Protein FAM3C OS=	FAM3C	23	5
P16452	Protein 4.2 OS=Hon	EPB42	5	3

P02765	Alpha-2-HS-glycopr	AHSG	56	43
Q14789	Golgin subfamily B	GOLGB1	0	1
Q04756	Hepatocyte growth	HGFAC	34	18
Q8TDY8	Immunoglobulin su	IGDCC4	1	1
Q9NWZ3	Interleukin-1 recep	IRAK4	4	2
Q13201	Multimerin-1 OS=H	MMRN1	13	14
Q08380	Galectin-3-binding	LGALS3BP	38	21
P55268	Laminin subunit be	LAMB2	2	3
Q07954	Prolow-density lipo	LRP1	12	48
P19957	Elafin OS=Homo sa	PI3	14	2
Q9NPD3	Exosome complex c	EXOSC4	4	1
Q96KP1	Exocyst complex co	EXOC2	1	1
P11362	Fibroblast growth f	FGFR1	9	6
Q14697	Neutral alpha-gluco	GANAB	25	19
Q96C23	Galactose mutarota	GALM	10	3
Q9HCN6	Platelet glycoprotei	GP6	17	4
Q08623	Pseudouridine-5'-p	PUDP	6	1
Q9NR34	Mannosyl-oligosacc	MAN1C1	3	2
Q9H8J5	MANSC domain-cor	MANSC1	5	1
P01709	Immunoglobulin lar	IGLV2-8	16	2
A0A0B4J1Y8	Immunoglobulin lar	IGLV9-49	8	1
P30711	Glutathione S-trans	GSTT1	9	2
O75144	ICOS ligand OS=Hor	ICOSLG	13	3
P17301	Integrin alpha-2 OS	ITGA2	5	6
P11279	Lysosome-associate	LAMP1	5	2
P07942	Laminin subunit be	LAMB1	8	13
Q12907	Vesicular integral-m	LMAN2	21	7
P33908	Mannosyl-oligosacc	MAN1A1	14	8
Q8WZA0	Protein LZIC OS=Ho	LZIC	4	1
O00754	Lysosomal alpha-m	MAN2B1	2	2
A0A075B6I9	Immunoglobulin lar	IGLV7-46	15	2
Q13232	Nucleoside diphosp	NME3	21	3
Q96PD2	Discoidin, CUB and	DCBLD2	1	1
Q00796	Sorbitol dehydroge	SORD	12	3
Q16775	Hydroxyacylglutath	HAGH	10	3
P02042	Hemoglobin subuni	HBD	83	24
Q9BYR7	Keratin-associated	KRTAP3-2	22	2
A0A0A0MT89	Immunoglobulin ka	IGKJ1	67	1
P04180	Phosphatidylcholin	LCAT	26	8
P21291	Cysteine and glycin	CSRP1	63	11
P02775	Platelet basic prote	PPBP	38	8
Q02108	Guanylate cyclase s	GUCY1A1	5	3
P01599	Immunoglobulin ka	IGKV1-17	33	9
P13929	Beta-enolase OS=H	ENO3	20	7
P08263	Glutathione S-trans	GSTA1	4	1
O14498	Immunoglobulin su	ISLR	12	5
A0A0B4J2D9	Immunoglobulin ka	IGKV1D-13	28	6
P01717	Immunoglobulin lar	IGLV3-25	54	6
P14174	Macrophage migrat	MIF	17	3
Q6P179	Endoplasmic reticul	ERAP2	6	5

Q5D862	Filaggrin-2 OS=Hom	FLG2	6	6
P04066	Tissue alpha-L-fuco:	FUCA1	18	9
O14908	PDZ domain-contai	GIPC1	7	2
Q16778	Histone H2B type 2-	H2BC21	19	3
Q8N1G4	Leucine-rich repeat	LRRC47	5	3
P26038	Moesin OS=Homo s	MSN	43	30
P28799	Progranulin OS=Ho	GRN	16	8
P68871	Hemoglobin subuni	HBB	88	34
B2CW77	Killin OS=Homo sap	KLLN	4	1
P37235	Hippocalcin-like pro	HPCAL1	29	5
P35908	Keratin, type II cyto	KRT2	61	45
P56199	Integrin alpha-1 OS	ITGA1	1	2
Q14974	Importin subunit be	KPNB1	11	8
Q9HA64	Ketosamine-3-kinas	FN3KRP	4	1
A0A0C4DH67	Immunoglobulin ka	IGKV1-8	30	5
P18428	Lipopolysaccharide-	LBP	14	6
P69905	Hemoglobin subuni	HBA1	52	17
P04196	Histidine-rich glyco	HRG	62	32
O95998	Interleukin-18-bind	IL18BP	4	1
P24593	Insulin-like growth	IGFBP5	22	5
Q9BX67	Junctional adhesion	JAM3	3	1
Q8IUC0	Keratin-associated	KRTAP13-1	15	2
P17252	Protein kinase C alp	PRKCA	7	5
A0A075B6P5	Immunoglobulin ka	IGKV2-28	31	5
Q5TCX8	Mitogen-activated	MAP3K21	3	3
O00533	Neural cell adhesio	CHL1	21	20
Q5VTT5	Myomesin-3 OS=Ho	MYOM3	4	5
Q9GZT8	NIF3-like protein 1	NIF3L1	4	2
Q9UJM8	Hydroxyacid oxidas	HAO1	2	1
P01871	Immunoglobulin he	IGHM	41	18
P20042	Eukaryotic translati	EIF2S2	3	1
Q9C075	Keratin, type I cyto:	KRT23	9	5
Q6A163	Keratin, type I cyto:	KRT39	5	3
Q96CN7	Isochorismatase do	ISOC1	22	5
Q13976	cGMP-dependent p	PRKG1	4	3
P13473	Lysosome-associate	LAMP2	4	3
P01718	Immunoglobulin lar	IGLV3-27	44	4
P55083	Microfibril-associat	MFAP4	11	3
P31995	Low affinity immun	FCGR2C	8	1
P23142	Fibulin-1 OS=Homo	FBLN1	41	23
P02751	Fibronectin OS=Hor	FN1	52	143
Q7Z7M9	Polypeptide N-acet	GALNT5	3	2
P02792	Ferritin light chain	FTL	21	3
Q8IWJ2	GRIP and coiled-coi	GCC2	1	1
P32004	Neural cell adhesio	L1CAM	2	2
A0A075B6I0	Immunoglobulin lar	IGLV8-61	7	1
A0A075B6K5	Immunoglobulin lar	IGLV3-9	30	4
Q9UGM5	Fetuin-B OS=Homo	FETUB	34	10
Q8TEQ6	Gem-associated pro	GEMIN5	1	1
P04899	Guanine nucleotide	GNAI2	16	5

P01594	Immunoglobulin ka	IGKV1-33	22	5
P11226	Mannose-binding p	MBL2	46	9
Q14696	LRP chaperone ME!	MESD	18	4
P78417	Glutathione S-trans	GSTO1	44	14
P51858	Hepatoma-derived	HDGF	16	3
P0DOY2	Immunoglobulin lar	IGLC2	51	10
P60842	Eukaryotic initiatior	EIF4A1	22	9
P17540	Creatine kinase S-ty	CKMT2	3	1
P31025	Lipocalin-1 OS=Hon	LCN1	18	3
P54296	Myomesin-2 OS=Hc	MYOM2	2	2
Q13287	N-myc-interactor O	NMI	7	2
P11166	Solute carrier famil	SLC2A1	6	3
P84243	Histone H3.3 OS=H	H3-3A	29	2
Q8N6C8	Leukocyte immuno	LILRA3	17	5
O00187	Mannan-binding le	MASP2	22	12
P17813	Endoglin OS=Homo	ENG	11	6
P48507	Glutamate--cystein	GCLM	5	1
P48723	Heat shock 70 kDa	HSPA13	4	2
P17936	Insulin-like growth	IGFBP3	55	12
P04264	Keratin, type II cyto	KRT1	69	73
P12035	Keratin, type II cyto	KRT3	13	15
O76015	Keratin, type I cutic	KRT38	7	4
Q05315	Galectin-10 OS=Hor	CLC	9	2
A0A075B6H9	Immunoglobulin lar	IGLV4-69	8	1
O43639	Cytoplasmic proteir	NCK2	17	7
Q13442	28 kDa heat- and ac	PDAP1	13	3
Q16777	Histone H2A type 2	H2AC20	27	3
P01857	Immunoglobulin he	IGHG1	55	32
P27930	Interleukin-1 recep	IL1R2	5	1
P13647	Keratin, type II cyto	KRT5	60	49
P23229	Integrin alpha-6 OS	ITGA6	13	14
Q9GZP8	Immortalization up	IMUP	8	1
P19827	Inter-alpha-trypsin	ITIH1	41	44
O43790	Keratin, type II cutic	KRT86	51	27
P05362	Intercellular adhesi	ICAM1	19	9
P55010	Eukaryotic translati	EIF5	4	2
Q14623	Indian hedgehog pr	IHH	3	1
P41091	Eukaryotic translati	EIF2S3	10	4
Q14210	Lymphocyte antige	LY6D	15	2
P55103	Inhibin beta C chain	INHBC	13	3
Q14525	Keratin, type I cutic	KRT33B	50	20
P20774	Mimecan OS=Homo	OGN	13	5
P08253	72 kDa type IV colla	MMP2	18	12
Q9UKX2	Myosin-2 OS=Homo	MYH2	3	5
P28482	Mitogen-activated	MAPK1	4	2
Q99972	Myocilin OS=Homo	MYOC	10	5
Q08209	Serine/threonine-p	PPP3CA	7	3
P07738	Bisphosphoglycerat	BPGM	12	2
Q8WZA1	Protein O-linked-m	POMGNT1	2	1
Q8TAT6	Nuclear protein loc	NPLOC4	2	1

P04792	Heat shock protein	HSPB1	55	11
P07900	Heat shock protein	HSP90AA1	30	20
P24592	Insulin-like growth f	IGFBP6	16	3
A0A075B6J9	Immunoglobulin lar	IGLV2-18	25	2
Q9NQ38	Serine protease inh	SPINK5	5	5
P58166	Inhibin beta E chain	INHBE	8	2
A0A0C4DH29	Immunoglobulin he	IGHV1-3	32	5
P35527	Keratin, type I cytos	KRT9	78	58
P31323	cAMP-dependent p	PRKAR2B	6	2
P78386	Keratin, type II cutic	KRT85	40	24
Q3ZCW2	Galectin-related pro	LGALSL	21	4
P01721	Immunoglobulin lar	IGLV6-57	32	3
P15529	Membrane cofactor	CD46	7	3
Q96HC4	PDZ and LIM domai	PDLIM5	13	7
Q14165	Malectin OS=Homo	MLEC	2	1
Q9C0I1	Myotubularin-relate	MTMR12	3	2
Q9UM47	Neurogenic locus no	NOTCH3	5	8
Q8NBF2	NHL repeat-contain	NHLRC2	11	7
Q13177	Serine/threonine-pr	PAK2	11	5
Q15113	Procollagen C-endo	PCOLCE	33	11
P19012	Keratin, type I cytos	KRT15	21	13
P48059	LIM and senescent c	LIMS1	36	13
O15212	Prefoldin subunit 6	PFDN6	16	2
Q13508	Ecto-ADP-ribosyltra	ART3	11	4
P03971	Muellerian-inhibitir	AMH	2	1
Q16836	Hydroxyacyl-coenzy	HADH	9	3
P02790	Hemopexin OS=Hor	HPX	76	84
P26927	Hepatocyte growth	MST1	48	27
P06312	Immunoglobulin ka	IGKV4-1	22	4
Q14767	Latent-transforming	LTBP2	1	1
P43034	Platelet-activating f	PAFAH1B1	16	6
Q9Y2E5	Epididymis-specific	MAN2B2	1	1
Q13496	Myotubularin OS=H	MTM1	1	1
Q9Y2A7	Nck-associated prot	NCKAP1	6	6
Q86X76	Deaminated glutath	NIT1	7	2
Q14554	Protein disulfide-iso	PDIA5	20	11
Q15084	Protein disulfide-iso	PDIA6	22	8
P09467	Fructose-1,6-bispho	FBP1	5	2
P01602	Immunoglobulin ka	IGKV1-5	32	6
O75342	Arachidonate 12-lip	ALOX12B	4	2
O95274	Ly6/PLAUR domain	LYPD3	6	2
P48740	Mannan-binding lec	MASP1	31	16
Q9Y316	Protein MEMO1 OS	MEMO1	10	3
O14950	Myosin regulatory l	MYL12B	41	8
Q8NCC3	Phospholipase A2 g	PLA2G15	8	3
Q9Y5Y7	Lymphatic vessel er	LYVE1	15	6
P43121	Cell surface glycopr	MCAM	25	13
P32942	Intercellular adhesi	ICAM3	5	3
Q16270	Insulin-like growth f	IGFBP7	20	4
P05154	Plasma serine prote	SERPINA5	37	14

Q7Z794	Keratin, type II cyto	KRT77	11	12
P30085	UMP-CMP kinase O	CMPK1	32	6
A0A087WW87	Immunoglobulin ka	IGKV2-40	31	5
P23490	Loricrin OS=Homo s	LORICRIN	3	1
O14745	Na(+)/H(+) exchang	SLC9A3R1	25	8
P09104	Gamma-enolase OS	ENO2	26	10
Q9BYR8	Keratin-associated	KRTAP3-1	41	3
P08581	Hepatocyte growth	MET	4	4
Q969H8	Myeloid-derived gr	MYDGF	5	1
P12883	Myosin-7 OS=Homo	MYH7	6	10
Q9H8W4	Pleckstrin homolog	PLEKHF2	4	1
Q15063	Periostin OS=Homo	POSTN	18	15
Q00169	Phosphatidylinosito	PITPNA	5	1
P24666	Low molecular weig	ACP1	15	2
Q9GZP4	PITH domain-contai	PITHD1	4	2
Q9Y5Z4	Heme-binding prote	HEBP2	14	2
P00492	Hypoxanthine-guan	HPRT1	37	7
Q96RW7	Hemicentin-1 OS=H	HMCN1	0	1
P61978	Heterogeneous nuc	HNRNPK	8	3
P29218	Inositol monophosp	IMPA1	11	3
P58546	Myotrophin OS=Ho	MTPN	31	4
P08590	Myosin light chain 3	MYL3	13	2
Q15223	Nectin-1 OS=Homo	NECTIN1	7	3
P23083	Immunoglobulin he	IGHV1-2	31	5
Q9BS40	Latexin OS=Homo s	LXN	5	1
P19367	Hexokinase-1 OS=H	HK1	16	16
P48200	Iron-responsive ele	IREB2	1	1
Q6KB66	Keratin, type II cyto	KRT80	19	8
O00505	Importin subunit al	KPNA3	3	2
O94819	Kelch repeat and BT	KBTBD11	2	1
P60985	Keratinocyte differe	KRTDAP	9	1
Q02750	Dual specificity mit	MAP2K1	2	1
P07203	Glutathione peroxic	GPX1	41	8
P40197	Platelet glycoprotei	GP5	16	8
A0A075B6R9	Probable non-funct	IGKV2D-24	11	1
P11717	Cation-independent	IGF2R	13	28
Q14232	Translation initiatio	EIF2B1	12	4
O75791	GRB2-related adapt	GRAP2	16	4
P11169	Solute carrier famil	SLC2A3	7	4
P68431	Histone H3.1 OS=H	H3C1	29	2
P29622	Kallistatin OS=Hom	SERPINA4	37	16
P62805	Histone H4 OS=Hon	H4C1	39	4
P09960	Leukotriene A-4 hy	LTA4H	22	12
P34896	Serine hydroxymetl	SHMT1	6	3
P48637	Glutathione synthe	GSS	25	12
Q7LDG7	RAS guanyl-releasin	RASGRP2	4	1
O43464	Serine protease HTI	HTRA2	2	1
P78318	Immunoglobulin-bi	IGBP1	3	1
P05198	Eukaryotic translati	EIF2S1	13	4
P02538	Keratin, type II cyto	KRT6A	63	49

Q9NPH2	Inositol-3-phosphat	ISYNA1	2	1
Q5VWZ2	Lysophospholipase-	LYPLAL1	10	2
P00568	Adenylate kinase is	AK1	34	6
Q14624	Inter-alpha-trypsin	ITIH4	59	81
P54819	Adenylate kinase 2,	AK2	40	9
Q86V88	Magnesium-depend	MDP1	13	2
Q9UNW1	Multiple inositol po	MINPP1	14	6
Q9UJ68	Mitochondrial pept	MSRA	26	5
P00488	Coagulation factor	F13A1	36	24
Q96A65	Exocyst complex co	EXOC4	1	1
Q9UM22	Mammalian epend	EPDR1	5	1
P05546	Heparin cofactor 2	SERPIND1	41	22
Q08431	Lactadherin OS=Ho	MFGE8	3	1
Q9ULI3	Protein HEG homol	HEG1	6	7
P06865	Beta-hexosaminida	HEXA	8	5
P05783	Keratin, type I cyto	KRT18	8	4
O00462	Beta-mannosidase	MANBA	8	7
P55145	Mesencephalic astr	MANF	41	9
P08493	Matrix Gla protein	MGP	11	1
Q8WUA8	Tsukushi OS=Homo	TSKU	3	1
Q9NYU2	UDP-glucose:glyco	UGGT1	2	3
P21283	V-type proton ATPa	ATP6V1C1	2	1
Q86UX7	Fermitin family hon	FERMT3	54	32
Q70J99	Protein unc-13 hon	UNC13D	6	6
Q9GZX9	Twisted gastrulatio	TWSG1	7	1
P63279	SUMO-conjugating	UBE2I	12	2
P35443	Thrombospondin-4	THBS4	23	17
P07996	Thrombospondin-1	THBS1	53	49
P61081	NEDD8-conjugating	UBE2M	21	4
Q9NVG8	TBC1 domain famil	TBC1D13	8	3
P78371	T-complex protein	CCT2	7	4
P18206	Vinculin OS=Homo	VCL	56	70
P22735	Protein-glutamine	TGM1	3	2
Q08188	Protein-glutamine	TGM3	11	7
P08670	Vimentin OS=Homo	VIM	15	8
P42765	3-ketoacyl-CoA thic	ACAA2	3	1
Q92973	Transportin-1 OS=H	TNPO1	2	2
Q9Y490	Talin-1 OS=Homo s	TLN1	52	116
Q86YW5	Trem-like transcript	TREML1	5	1
Q8TBC4	NEDD8-activating e	UBA3	4	2
O95292	Vesicle-associated	VAPB	5	1
P56192	Methionine--tRNA l	MARS1	2	1
O75347	Tubulin-specific cha	TBCA	27	4
P52888	Thimet oligopeptid	THOP1	1	1
P48643	T-complex protein	CCT5	23	13
P05452	Tetranectin OS=Ho	CLEC3B	57	10
Q8WZ42	Titin OS=Homo sapi	TTN	0	7
P55327	Tumor protein D52	TPD52	9	2
P60174	Triosephosphate is	TPI1	64	18
P21333	Filamin-A OS=Homo	FLNA	40	97

Q9Y613	FH1/FH2 domain-cc	FHOD1	1	2
P35754	Glutaredoxin-1 OS=	GLRX	10	1
Q06033	Inter-alpha-trypsin	ITIH3	30	26
Q8IUC1	Keratin-associated	KRTAP11-1	15	2
P54577	Tyrosine--tRNA liga	YARS1	18	9
Q9BYX2	TBC1 domain famil	TBC1D2	1	1
Q7Z7G0	Target of Nesh-SH3	ABI3BP	9	8
O43493	Trans-Golgi networ	TGOLN2	11	4
Q15631	Translin OS=Homo	TSN	13	3
A0AVT1	Ubiquitin-like modi	UBA6	2	2
P19971	Thymidine phosphc	TYMP	25	8
Q14508	WAP four-disulfide	WFDC2	11	1
O43399	Tumor protein D54	TPD52L2	33	6
P61088	Ubiquitin-conjugati	UBE2N	22	4
Q9BWD1	Acetyl-CoA acetyltr	ACAT2	17	5
P53999	Activated RNA poly	SUB1	17	2
Q4G0F5	Vacuolar protein so	VPS26B	6	2
P62837	Ubiquitin-conjugati	UBE2D2	12	2
P50991	T-complex protein 1	CCT4	15	8
P00734	Prothrombin OS=Hc	F2	56	41
Q6PL24	Protein TMED8 OS=	TMED8	9	3
O95497	Pantetheinase OS=I	VNN1	14	6
O75695	Protein XRP2 OS=H	RP2	3	1
Q96C24	Synaptotagmin-like	SYTL4	5	3
P68371	Tubulin beta-4B cha	TUBB4B	40	16
P20061	Transcobalamin-1 C	TCN1	5	2
P25311	Zinc-alpha-2-glycop	AZGP1	55	30
Q969T9	WW domain-bindin	WBP2	3	1
P68036	Ubiquitin-conjugati	UBE2L3	40	6
Q5JSH3	WD repeat-containi	WDR44	26	20
Q8NBS9	Thioredoxin domair	TXNDC5	6	3
Q99536	Synaptic vesicle me	VAT1	7	2
Q9GZM5	Protein YIPF3 OS=H	YIPF3	3	1
P04004	Vitronectin OS=Hor	VTN	46	33
Q5THJ4	Vacuolar protein so	VPS13D	0	1
Q9C0C9	(E3-independent) E	UBE2O	5	7
P22314	Ubiquitin-like modi	UBA1	22	20
Q9UDY2	Tight junction prote	TJP2	5	5
Q16620	BDNF/NT-3 growth	NTRK2	1	1
P06737	Glycogen phosphor	PYGL	12	11
P51149	Ras-related protein	RAB7A	38	7
O95980	Reversion-inducing	RECK	3	3
O43665	Regulator of G-prot	RGS10	24	3
P47929	Galectin-7 OS=Hom	LGALS7	15	2
Q14766	Latent-transforming	LTBP1	16	22
Q92686	Neurogranin OS=Hc	NRGN	59	2
Q9P121	Neurotrimin OS=Hc	NTM	7	2
P07585	Decorin OS=Homo	DCN	3	1
P10451	Osteopontin OS=Hc	SPP1	8	2
Q99497	Parkinson disease p	PARK7	51	9

P68402	Platelet-activating f	PAFAH1B2	12	3
P55058	Phospholipid transf	PLTP	16	7
Q8WVV4	Protein POF1B OS=I	POF1B	12	6
O60664	Perilipin-3 OS=Hom	PLIN3	18	6
P12273	Prolactin-inducible	PIP	18	2
Q8TD55	Pleckstrin homolog	PLEKHO2	9	4
Q13094	Lymphocyte cytoso	LCP2	15	8
P62310	U6 snRNA-associate	LSM3	20	2
P51884	Lumican OS=Homo	LUM	35	21
P22891	Vitamin K-depende	PROZ	28	10
O76074	cGMP-specific 3',5'-	PDE5A	17	15
Q9BQ51	Programmed cell de	PDCD1LG2	9	2
Q9UHG3	Prenylcysteine oxid	PCYOX1	31	14
P30086	Phosphatidylethanc	PEBP1	62	9
Q9ULV0	Unconventional my	MYO5B	0	1
P22061	Protein-L-isoaspart	PCMT1	28	6
Q9UFN0	Protein NipSnap ho	NIPSNAP3A	9	2
P20618	Proteasome subuni	PSMB1	39	9
Q9Y3C6	Peptidyl-prolyl cis-t	PPIL1	20	3
Q9UNP9	Peptidyl-prolyl cis-t	PPIE	6	2
P30048	Thioredoxin-depen	PRDX3	24	6
P24844	Myosin regulatory l	MYL9	41	7
P80188	Neutrophil gelatina	LCN2	39	6
P00480	Ornithine transcarb	OTC	3	1
Q8NHP8	Putative phospholi	PLBD2	1	1
A0A0A0MRZ8	Immunoglobulin ka	IGKV3D-11	30	4
Q16706	Alpha-mannosidase	MAN2A1	16	16
Q99650	Oncostatin-M-speci	OSMR	4	3
Q01995	Transgelin OS=Hom	TAGLN	27	5
P01137	Transforming grow	TGFB1	9	3
P04155	Trefoil factor 1 OS=	TFF1	11	1
O95858	Tetraspanin-15 OS=	TSPAN15	4	1
Q9H900	Protein zwilch hom	ZWILCH	2	1
P35030	Trypsin-3 OS=Homo	PRSS3	11	4
Q16851	UTP--glucose-1-pho	UGP2	36	16
P42768	Wiskott-Aldrich syn	WAS	15	5
P04275	von Willebrand fact	VWF	40	94
O60844	Zymogen granule m	ZG16	26	4
Q9Y4E8	Ubiquitin carboxyl-t	USP15	6	5
Q9NP84	Tumor necrosis fact	TNFRSF12A	9	1
O43396	Thioredoxin-like pr	TXNL1	18	3
O43895	Xaa-Pro aminopept	XPNPEP2	7	4
E9PLD1	Sterol carrier protei	SCP2	5	2
H3BR70	Pyruvate kinase OS	PKM	50	20
A0A0D9SEI7	c-Myc-binding prot	MYCBP	16	1
U3KQU7	Conserved oligome	COG5	6	1
H3BUU9	Cadherin-11 OS=Ho	CDH11	4	2
C9JH92	Quinone oxidoredu	CRYZ	16	3
U3KQT1	S-formylglutathione	ESD	39	8
M0QZL2	Multiple epidermal	MEGF8	14	2

E7EVN1	Caspase-8 (Fragmer	CASP8	6	1
J3KNF4	Copper chaperone f	CCS	15	4
A0A6Q8PHH9	Diadenosine tetrapl	GARS1	8	6
F8VVM2	Phosphate carrier p	SLC25A3	8	1
E9PMV2	HLA class II histocor	HLA-DQA1	11	2
G3XAH0	Septin OS=Homo sa	SEPTIN5	23	8
C9J480	Type 2 lactosamine	ST3GAL6	3	1
A0A494C0X0	Tubulin-folding cofa	TBCB	16	5
A0A7I2V649	Polyadenylate-bind	PABPC1	6	3
J3QRU8	ARF GTPase-activat	GIT1	2	1
H0Y555	Proteasome inhibit	PSMF1	11	1
E5RGR0	Acyl-protein thioest	LYPLA1	11	2
A0A6Q8PFA6	Kinesin-like protein	KIF2A	11	8
A0A1W2PNV4	Uncharacterized pr	--	19	10
K7EKI8	Periplakin OS=Hom	PPL	1	2
E5RIM3	Phospholipase A-2-a	PLAA	7	2
A0A494C0U4	Protein-tyrosine-ph	PTPRZ1	2	4
A0A0G2JPR0	C4a anaphylatoxin (	C4A	57	123
A0A6Q8PF60	Bile acid-CoA:amino	BAAT	3	1
A0A7I2YQN2	Heterogeneous nuc	SYNCRIP	2	1
E7EN89	Toll interacting prot	TOLLIP	3	1
A2ACR1	Proteasome subuni	PSMB9	17	3
J3KR97	Tubulin-specific cha	TBCD	1	1
H0YET5	EGF-containing fibu	EFEMP2	12	2
A0A087X0S5	Collagen alpha-1(VI	COL6A1	12	10
A0A7I2V3F1	Aconitate hydratase	ACO2	14	8
B1B0D4	ADAMTS-like protei	ADAMTSL2	3	3
J3QRN2	Beta-2-glycoprotein	APOH	64	20
A0A3B3ITK7	Phosphoglucomuta	PGM1	37	21
G3V0E5	Transferrin recepto	TFRC	36	22
G5E9W8	Glycogenin 1, isofor	GYG1	7	1
E9PF17	Versican core prote	VCAN	7	9
H0Y938	Coatomer subunit b	COPB2	3	3
A0A2R8YHD2	Tropomyosin alpha	TPM4	45	16
A0A0A0MS54	cAMP-dependent p	PRKACB	8	3
A0A7I2V4Y9	Dynactin subunit 2	DCTN2	8	3
F8WE86	Transcobalamin-2 C	TCN2	4	1
C9JEU5	Fibrinogen gamma	FGG	67	36
D6RHI9	Ribonuclease T2 (Fr	RNASET2	17	3
A0A140T9Z6	HLA class I histocon	HLA-A	39	11
A0A0J9YY99	Ig-like domain-cont	--	35	4
E7END4	Lysyl oxidase homo	LOXL3	4	2
F5H5D3	Tubulin alpha chain	TUBA1C	50	29
A0A087X0K0	Collagen alpha-1(X\	COL15A1	3	3
H7BXZ5	Non-specific serine/	KALRN	1	2
H7C0X7	Methionine adenos	MAT2B	6	1
H0YLH9	Pro-interleukin-16 (	IL16	1	1
M0R165	Epidermal growth f	EPS15L1	1	1
A0A3B3ISU3	Low affinity immun	FCGR3B	15	4
A0A0A0MSA9	Poliovirus receptor	PVR	21	7

F5H157	Ras-related protein	RAB35	10	2
A0A0J9YVP2	Immunoglobulin he	IGHJ6	38	1
D6RFH4	Cytochrome b5 typ	CYB5B	25	1
A0A7P0T862	Nicotinamide phos	NAMPT	5	1
A0A7P0TBH1	Angiotensinogen O	AGT	37	23
P55285	Cadherin-6 OS=Hon	CDH6	3	2
Q00013	55 kDa erythrocyte	MPP1	16	7
O95834	Echinoderm microt	EML2	7	4
Q9NUQ9	CYFIP-related Rac1	CYRIB	16	4
Q15075	Early endosome an	EEA1	1	2
Q14126	Desmoglein-2 OS=H	DSG2	7	8
O00115	Deoxyribonuclease-	DNASE2	3	1
P31150	Rab GDP dissociatic	GDI1	42	16
P50395	Rab GDP dissociatic	GDI2	43	18
P07093	Glia-derived nexin (	SERPINE2	3	1
P52565	Rho GDP-dissociatic	ARHGDIA	17	4
P52566	Rho GDP-dissociatic	ARHGDIB	51	12
P52306	Rap1 GTPase-GDP c	RAP1GDS1	4	2
P36959	GMP reductase 1 O	GMPR	37	9

PSMs	Unique Peptides	MW [kDa]	M/N Ratio	M/N P value
1	1	43.6	0.594	0.165053943
1	1	117.8		
153	9	28.8	1.192	0.318707395
2	2	60.9	1.508	0.121181591
1	1	20.9	1000	0.001
25	2	26.7	0.969	0.470522556
32	3	27	1.201	0.192916053
1	1	31.7	1.206	0.308389323
437	14	55.1	0.856	0.313868151
75	7	55.1	0.738	0.16445231
12	4	47.4	1.15	0.186229878
5419	66	140.9	0.879	0.178456367
4	1	38.3	0.546	0.129990733
2	1	102.5	1.141	0.341412706
13	2	49.7	0.625	0.087291879
1126	3	41.3	0.712	0.239165315
1357	1	46.7	1.347	0.653845976
1	1	50.2	0.699	0.570444422
252	2	33	0.928	0.901456018
776	14	23	0.945	0.714433322
91	2	40.7	2.189	0.616517353
28	3	50.4	0.692	0.091901395
2580	1	23.4	0.875	0.260868333
1602	34	81.8	0.864	0.724959115
5	1	5.4	0.821	0.49034453
28	1	103.4	0.913	0.453578944
15626	1	187.6	0.001	0.001
229	11	86.4	1.059	0.382383436
9	2	13	1000	0.001
58	1	229.2	1.071	0.435314285
1446	12	14.9	1.026	0.898136137
2	1	24.8	1.08	0.67223422
17	2	50.2	1.081	0.793679398
8	1	22.9	1.26	0.770403789
4	2	24.3	0.574	0.512918959
1	1	47.1	0.001	0.001
68	5	33.2	1.495	0.012504726
82	3	40.9	0.821	0.310168088
22	3	72.9	1	0.907233623
1	1	19.7		
7	3	89.9	1.159	0.417375492
12	1	19	0.001	0.001
232	14	37.3	1.363	0.801609345
28	3	52	0.929	0.608340284
7	4	31.1	0.935	0.486006928
5	1	22.3	0.914	0.596635044
5	1	15.3	0.841	0.430160897
9	3	94	0.717	0.513274987

2	1	56.4	1000	0.001
4	1	18.7	1000	0.001
56	5	14.2	0.639	0.023722744
1	1	16.4		
23	1	57.9	1.171	0.161916978
5	2	17.6	1.294	0.260399083
18	1	13.4	1.395	0.062188391
1	1	28.2	1000	0.001
15	4	106.4	1.36	0.546386571
13	1	8.7	1.307	0.054094942
1	1	11.1	1.212	0.221145105
25	5	317.6	0.932	0.740814186
1686	34	66.6	0.889	0.438957122
11	2	29.5	0.973	0.947190767
23	1	20.8	0.742	0.243211792
12	7	104.6	0.809	
12	1	7.8	0.609	
224	12	79	0.932	0.541156055
42	5	11.3	0.708	0.243357639
115	6	26.7	0.714	0.636078178
1	1	66.1		
1	1	35.6		
666	18	57.6	1.064	0.468699929
29	1	8.9	0.947	0.822879462
74	6	111.9	1.067	0.509669448
5052	2	51	0.744	0.415135028
2	2	105.6	0.846	0.702480384
2	1	12.9	0.532	0.031626142
32	3	31.4	1.176	0.173529581
8	1	34.1	1.544	0.050346192
17	2	33.5	0.759	0.444007718
1	1	17.2	0.001	0.001
13	5	29.2	1.296	0.048537663
3	2	41.4	1.229	0.240465669
35	4	37.6	0.401	0.003072174
96	5	49.4	3.429	0.253561909
1	1	59.2		
5	2	31.6	3.538	0.304362172
44	1	19.5	0.755	0.213335748
1156	3	43.8	0.556	0.165458312
69	6	26.7	0.413	0.59395196
31	2	14.5	0.835	0.779678782
74	2	39.8	1.223	0.997940228
3	1	46.4	0.534	0.409396574
21	1	55.6	1.184	0.916696741
12	1	25.3	1.054	0.964717583
4	1	162.2	1.19	0.467991362
36	5	47	0.592	0.864979628
145	3	23.3	0.906	0.467299785
8	2	25.3	0.48	0.320893693

33	1	33.8	1.401	0.212497787
1	1	28.5		
14	2	37.4	1.121	0.821552272
2	2	158.5	1.481	0.380051575
101	8	146.5	1.065	0.603780922
4	2	13.3	0.001	0.001
671	7	24	0.79	0.05549892
286	21	76.6	0.877	0.48535089
42	2	65.9	1.015	0.907753097
68	1	28.9	1.441	0.438862085
40	1	51.1	0.897	0.487774699
1	1	20.4		
1	1	28.6	0.766	0.178080545
409	1	11.2	1.196	0.643928109
143	9	60.2	1.115	0.161327765
1	1	39.9	0.794	0.99266302
166	10	87.5	0.743	0.06287792
1	1	37.1	0.986	0.756176361
118	20	130.2	1.174	0.083538693
11	1	12.7	1.162	0.781379546
8080	37	59.5	0.888	0.082954081
4	2	44.9	1.16	0.489341576
2	1	17.9	0.515	0.139374676
45	3	13.1	0.763	0.148778264
955	5	23.3	1.271	0.090002765
8	3	53.6	0.684	0.12166896
1186	14	25.4	0.943	0.802787722
15	2	46.8	0.947	0.531006412
864	10	46.3	0.833	0.096214917
4	1	37.2	0.746	0.426306273
8	2	56.1	0.826	0.700627931
2	2	44		
4	1	11.2	0.391	0.04503107
8	2	10.5	0.491	0.256120778
92	6	58	1.119	0.274451485
71	4	25.2	0.867	0.237352603
51	5	163.6	0.713	0.10161336
138	2	22.5	1.395	0.318288602
52	2	10.2	1.054	0.393560268
4	2	46.4	0.738	0.300458836
6	1	103.8	1.328	0.317292906
5	2	29.8	1.48	0.09836408
25	1	17.3	0.772	0.159785907
11	2	49.5	0.758	0.196251838
591	14	51.2	1.036	0.51112668
52	2	29.2	1.17	0.403067032
79	9	95.6	1.158	0.268633964
8	2	19.4	0.924	0.951866246
111	9	74.9	1.322	0.161487116
81	4	16.8	0.809	0.131429744

11	2	14.5	2.006	0.558376686
13	2	32.8	0.846	0.764279466
8	1	53	0.834	0.237969712
26	1	154.8	1.177	0.215927267
2	2	65.6	1000	0.001
1	1	39	0.8	0.392087679
38	3	18.6	0.786	0.040297812
12	2	23.5	1.267	0.039584944
2	2	55.2	1000	0.001
109	7	42.6	1.152	0.135135212
1	1	49.1	1.21	0.752082618
17	3	41.5	1.014	0.893889195
3	2	68	0.706	0.500172537
81	8	275.3	1.003	0.800603942
260	9	34.6	1.076	0.482283659
11	2	43.4	0.857	0.539844236
36	4	58.9	1.806	0.403278791
28	5	33.2	1.26	0.223069394
64	5	21.7	1.001	0.933608162
8	1	41	1000	0.001
21	1	42	0.907	0.811921735
1	1	273.4	1.223	0.124083133
67	4	23.2	0.95	0.624560138
10	3	14.7	0.88	0.474464102
10	1	62	0.99	0.98711448
342	19	63.1	1.053	0.36850184
266	16	59.2	1.211	0.060938335
1	1	70.4	0.457	0.034635084
605	15	62.6	0.927	0.757804164
8	4	13.8	0.856	0.621862423
761	15	51.2	0.686	0.295886459
64	4	109.6	1.037	0.665894331
80	5	76.8	1.391	0.040741615
120	3	12.3	1.045	0.800427765
20	1	35.6	0.774	0.166098257
94	7	52.4	1.21	0.808166184
34	1	133.2	0.8	0.297482701
11	1	41	0.894	0.66820016
373	14	50.3	1.11	0.155848233
98	1	22.5	1.448	0.04405292
507	20	82.5	1.148	0.279639974
5	1	17.2	0.919	0.694465562
25	2	129.9	0.963	0.951468638
26	1	12.3	0.774	0.192176775
18	1	145.1	1.066	0.514547694
555	5	49.9	1.524	0.010133282
50	1	47.3	1.04	0.585222831
601	16	39.7	1.261	0.104871741
99	5	29.5	1.132	0.373441048
79	6	21.8	1.17	0.157745349

17	3	80	1.475	0.039353906
19	5	46.6	0.982	0.929399625
5	1	78.9	1.186	0.106838164
16	2	116	1.162	0.787291401
4	1	25.4	0.862	0.509250229
146	4	15.9	0.705	0.072466283
97	4	49.3	1.07	0.643425515
8	3	112.1	0.795	0.449149951
16	3	23.3	0.222	0.064090226
159	7	5.1	0.869	0.550712908
11	3	22	0.913	0.867440544
1	1	50.2	1.029	0.999744439
45	8	54.9	0.864	0.772215473
1	1	31.8	0.53	0.208428178
1319	32	163.8	1.327	0.880627898
155	5	49.7	1.001	0.742495321
3	1	59		
107	5	50	1.376	0.616864374
28	5	49.2	1.246	0.79336048
12	1	33.2	0.577	0.066727639
7	2	10.4	1.202	0.18834921
14	1	40.5	1.025	0.74789627
40	1	7.9	1.052	0.485616827
90	8	68.2	1.095	0.344474718
53	7	60.5	0.649	0.01605946
27	3	90.9	1.027	0.771414573
35	4	38.4	1.045	0.793701514
14	1	66.1	0.929	0.830708127
5936	56	90.5	1.009	0.936713271
21	2	49.2	0.547	0.550465239
35	8	50.4	1.333	0.095072995
403	2	49.9	0.471	0.8773825
1111	16	22.4	0.815	0.23010217
9	1	31.5	3.131	
2	2	309.2	1000	0.001
40	6	58.9	1	0.749427287
4	4	52.1	0.887	0.757764773
239	17	165.6	0.745	0.125031119
2	1	25.5		
7	1	33.3	0.858	0.188490525
26	4	56.5	1.408	0.321830278
196	6	71.7	0.824	0.043866645
118	10	68.3	1.619	0.058692266
38	2	21.9	1.116	0.389734092
990	16	62.2	0.996	0.917325497
183	7	96.6	1.164	0.410083045
27	6	89.8	1.026	0.513946295
2	2	9.9	0.773	0.931935617
460	13	18	0.909	0.442727749
2	2	43	5.782	0.349037465

2328	60	77	0.871	0.190713994
134	1	22.7	1.147	0.452423768
62	5	166.6	0.985	0.904512447
1258	19	46.3	0.731	0.031898425
1	1	89.4	1.266	0.27396131
42	3	31.7	0.96	0.710179035
17	2	34.1	0.952	0.714177268
55	4	389.9	1.217	0.346004583
21	4	33.4	0.776	0.684441357
130	14	60.5	0.867	0.258676738
143	9	10.8	0.342	0.024711291
6	2	468.8	0.724	0.425984632
5	2	22.6	0.493	0.073524057
12	1	43.2	1.166	0.419805635
3	1	60.7		
13	2	88	1.005	0.434360837
487	5	28.5	0.833	0.742426487
7	3	19.1	0.811	0.86893353
47	4	22.8	1.693	0.116804186
32	8	68	1.091	0.339794544
132	3	43.1	0.972	0.985031571
22	2	24.3	0.956	0.934093746
3	2	14.5	1.589	0.052281266
27	5	22.4	1.359	0.198812815
58	8	72.6	0.936	0.764323071
5	1	360	1.434	0.030286627
11	3	91.6	1.121	0.578616925
1	1	12	0.905	0.901371937
222	6	52	1.074	0.742856612
3	1	33.2	0.705	0.149807756
95	4	12.5	0.688	0.102840137
338	30	165.9	1.028	0.891861139
264	5	11.7	0.957	0.890708765
151	7	10.8	1.359	0.410650658
133	3	13.5	0.257	0.875987037
9	4	42.1	0.174	0.488099976
10	1	10.1	0.989	0.999951117
3	1	38.8	1.136	
11	2	58.4	0.796	0.707256294
86	5	123.9	1.052	0.592190924
10	1	85.1	0.93	0.793962061
73	6	22.1	1.146	0.227951512
155	5	21	0.653	0.202740306
2	1	34.7	0.969	0.668560873
30	4	41.2	1.144	0.498570295
11	2	48	1.749	0.247766459
147	3	22.2	1.066	0.605487964
203	7	39.6	0.839	0.311751772
98	4	26.4	0.78	0.114761186
17	2	25.9	1.119	0.381162068

26	2	100.1	1.064	0.945211389
190	13	49.9	0.86	0.052562184
112	7	13.2	1.227	0.74492492
91	5	47	1.44	0.090961961
55	4	14.3	1.147	0.786364797
71	9	66.4	1.26	0.101989163
2	2	88.1	1.593	0.722711574
1	1	105.8	1.246	0.191399592
62	5	92.7	0.981	0.992718486
57	1	227.7	1.822	0.086987412
1	1	46.6	0.001	0.001
3	1	136.1	1.156	0.16616048
117	8	43.8	0.937	0.812793229
133	10	85.5	1.107	0.372810953
20	5	27.9	0.767	0.697342035
3	1	11.7	0.836	0.234434876
30	4	18.3	0.69	0.133948425
45	3	20.8	1.012	0.585503419
160	8	56.2	1.033	0.538464103
120	14	96	1.053	0.612954587
106	14	83.8	1.736	0.82935646
1	1	11.7	2.072	0.264692297
76	14	101.9	0.83	0.321293931
122	8	24.7	1.03	0.622847644
14	4	58.4	1.212	0.165994225
3	1	112.2	1.061	0.535326496
12	5	265.2	0.756	0.375761306
1	1	18.8		
940	15	15	1.055	0.474399278
7	1	65.4	0.946	0.92679309
545	23	44.6	0.895	0.365320281
2	1	47.3		
51	9	42.4	0.797	0.176221051
87	11	38.8	1.117	0.317755963
38	3	22.9	1.16	0.378122074
11	2	40.7	1.238	0.145829308
215	3	20.8	1.366	0.073611275
46	2	21	0.839	0.185722556
47	2	11.5	0.516	0.002981746
4	1	10.6	1.693	0.127623042
6	2	11.8	0.334	0.179430276
405	18	36	0.924	0.544992766
9	2	531.5	0.872	0.49017575
332	1	38.9	0.729	0.025575857
8	3	24.4	1.086	0.356203606
15	3	23.5	0.536	0.063323561
22	5	46.1	0.94	0.511087598
2	1	18.4	0.51	0.010150812
57	15	82.8	0.63	0.33270544
159	1	23.7		

15	1	105.9	0.55	0.045623525
2	1	25.7	1.097	0.944806314
17	2	25.7	0.906	0.71763699
112	9	109.8	0.95	0.574131954
2	1	27.5	0.858	0.534073429
82	1	12.3	1.034	0.845685741
1	1	15	0.656	0.431828792
10	2	30.3	0.966	0.822895986
64	5	465.4	0.772	0.174201812
18	2	54.7	0.72	0.433638086
27	4	55.6	1.209	0.308259934
14	4	35.6	0.948	0.677582865
61	4	26.1	1.278	0.481697192
4	2	45.8	0.959	0.51546878
476	20	11.8	0.974	0.647715767
126	1	55.1	0.766	0.170302875
211	23	180.5	1.239	0.033874181
26	3	84.7	1.303	0.257355016
143	10	64.1	0.372	0.243223309
30	3	46.5	1.099	0.400343252
16	2	17.3	0.915	0.693041107
55	10	78.4	1.451	0.095744385
3	1	55.7	0.622	0.131642994
5	3	50.2	0.922	0.883318146
23	2	58.6	0.835	0.39897607
4	1	31	0.458	0.471159037
238	23	282.2	0.366	0.048225077
8	4	42.3	0.785	0.951895317
112	6	16.8	0.966	0.847225438
6	1	14.3	1.5	0.01967079
1	1	62.9		
9	2	25.1	1.036	0.840957386
1	1	124.9	0.857	0.280896418
6	1	28.9	0.617	0.134623162
3	2	26.9	1.548	0.031573774
120	2	12.2	1.258	0.694411284
28	2	23.8	1.138	0.235780786
18	3	25.7	0.542	0.012611748
13	2	97.9	1.03	0.721969261
22	1	22.6	0.832	0.418004112
2	1	70.4	1.09	0.721183861
18	2	232.2	0.914	0.523560857
5	1	42.8	1.761	0.431905625
14	2	22.3	5.55	0.350575372
142	7	47.2	9.48	0.032091875
288	20	87	0.988	0.836176847
4959	57	71.9	0.887	0.103893712
34	2	21.7	0.987	0.998795583
147	5	12.5	0.643	0.539865512
184	8	16.5	0.925	0.477225107

9	2	198.2	1.223	0.005404247
24	3	20.5	1.319	0.166354091
6	1	12.5	0.939	0.949424104
54	6	40.5	1	0.801620153
109	9	37.5	1.355	0.098453848
6	2	128.9	0.482	0.200357026
258	5	41.7	1.283	0.155367482
11	2	35.7	0.559	0.093468628
37	5	71.4	1.457	0.332680991
363	22	136.3	0.857	0.139229998
5	2	12	1.072	0.347474786
247	17	210.6	1.022	0.56854289
82	5	60	0.962	0.987495898
1	1	45.9	1.007	0.529009118
105	5	45	0.948	0.886399374
2096	9	71.3	0.873	0.182399973
28	1	12.6	0.714	0.005278279
23	1	12.4	1.529	0.598847511
48	4	38.7	0.763	0.036431685
211	1	17.1	0.61	0.049982425
14	3	64.1	0.585	0.061891013
53	3	52.6	1.904	0.258243552
153	8	30	1.302	0.079697701
17	2	23.4	0.97	0.843314226
1	1	36.7		
29	1	38.3	1.196	0.092163485
13	5	31.2	1.492	0.042309818
168	16	72.9	1.21	0.015468565
68	3	37.5	1.05	0.909691228
90	11	159.9	1.158	0.344091561
8	1	54.4		
16	1	34.5	1.292	0.01795124
1	1	114.7	0.001	0.001
22	4	48.6	1.391	0.018522771
26	2	18.2	0.669	0.554074156
81	2	22.7	0.285	0.056988115
16	2	50.5	0.89	0.839720845
99	6	28.7	1.333	0.081634871
26	1	23.7		
51	1	21.4	1.634	0.064144695
8	2	116.3	0.475	0.018991112
78	9	160.8	1.159	0.164195881
7	2	37.4	1.019	0.960319331
31	2	21.1	0.695	0.003417918
7	1	45	1.039	0.820651799
3	2	87	0.001	0.001
43	3	133.9	0.81	0.447048126
77	4	22	1.165	0.410627172
4	2	28.5	0.612	0.063467808
2	1	23	1.452	0.706243117

51	3	23.4	1.363	0.032424657
105	10	64.6	0.928	0.983893482
70	1	82.8	1.264	0.470410931
110	9	57.5	1.181	0.016458969
16	1	51.4	1.088	0.83603408
180	12	23.3	1.321	0.079874733
241	12	42.7	1.004	0.881322691
1	1	11.9	0.954	0.987397847
4	1	56.6	1000	0.001
43	4	77.5	0.988	0.996667533
5	3	64.3	1000	0.001
2	1	35.1	0.823	0.716146299
269	21	67.5	0.95	0.550936538
8	2	23.4	0.983	0.879033254
2	1	25		
3	2	116.9	0.905	0.865798027
36	2	19.2	0.71	0.103382101
3	2	157.2	1.391	0.222247084
128	14	57.1	1.204	0.02200888
174	14	37.5	0.652	0.088383447
91	5	107.4	0.798	0.374969829
43	2	158.1	0.932	0.480712898
156	6	24.3	1.278	0.061310741
8	2	34.5	2.663	0.073543932
3	1	26.8	0.106	0.197882011
725	33	66.2	1.012	0.635868706
309	21	89.3	1.099	0.191774201
16	2	95.7	1.376	0.063260259
43	2	13.9	0.893	0.792562623
22	2	105.3	0.776	0.18586926
1	1	49.8		
87	11	111.6	1.194	0.078426302
10	2	73.5	1.239	0.216480555
6884	53	52.9	0.863	0.146112185
36	2	26.2	0.889	0.677430219
29	1	16.4		
19	5	80.6	0.759	0.859621974
127	11	60.3	1.273	0.104225316
18	4	86.4	5.387	0.154525419
28	2	24.4	0.874	0.290701042
102	8	69.7	0.819	0.979123165
1	1	38.1	0.856	0.217625573
130	9	69.9	1.904	0.008795676
6	2	54.3	1.347	0.316344834
32	1	10	0.946	0.292488561
13	2	32.9	1.057	0.644489133
302	18	81.2	1.015	0.887257564
1	1	24.9	0.774	0.576002094
78	6	49.3	1.255	0.061855128
367	21	56.7	0.904	0.710893434

140	6	161.9	0.527	0.000734699
5	1	14.6	0.838	0.346631819
1	1	85.2		
464	51	226.4	0.886	0.424551605
75	3	30.7	0.987	0.833457586
7	3	70.7	1.066	0.377059708
786	18	92.3	1.095	0.399124255
3	2	110.6	0.867	0.923164922
1	1	50.5	0.494	0.535259034
103	2	54.9	7.788	0.540751188
1	1	148.8	0.342	0.076429753
28	3	107.7	1.383	0.119839393
78	8	36.3	0.753	0.472928716
102	13	94.3	1.025	0.647693057
793	13	45.2	0.939	0.67944546
9	2	41.3	1.449	0.263523086
26	5	177.5	1.077	0.842817213
15	3	45.3	1.07	0.476326082
14	1	47.4	0.943	0.674328904
8	1	24	0.873	0.542597982
268	13	32.1	1.02	0.702920824
759	24	70.2	1.072	0.448777902
44	4	34	1.325	0.236326765
47	2	42.1	0.537	0.26233935
26	3	162	0.361	0.085520461
35	5	104.8	0.924	0.51674738
7	2	14.5	0.001	0.001
3	2	59.8	0.001	0.001
161	8	40.1	0.758	0.466688031
15	2	34.7	0.87	0.304307047
49	3	28.4	1.124	0.451614635
72	6	30.3	0.881	0.397270443
2	2	40.9	1.001	0.834347745
66	1	39.6	1.188	0.384872571
171	13	302.9	0.927	0.659939429
17	5	30.6	1.001	0.888101511
56	1	12.7	1.415	0.018361218
2	1	9.4	0.001	0.001
172	11	75.6	1.311	0.065746929
29	1	79.6	0.647	0.013893639
10	3	50.8	0.911	0.829617822
103	4	30.5	0.813	0.876426298
85	11	212.7	1.559	0.269102715
76	4	55.3	1.337	0.284808452
99	6	74.2	0.414	0.030294703
38	3	16.6	0.751	0.042037385
69	8	82.5	0.878	0.631446195
15	2	57.3	0.976	0.9315281
7	1	28.9	1.178	0.364780955
17	3	27.6	1.119	0.415808418

13	4	31.5	1.122	0.99380536
291	10	25	1.263	0.104819154
5	3	77.5	0.805	0.621447925
2	1	24.1	1000	0.001
27	1	20.3	1.085	0.684402154
652	20	15.9	0.884	0.349544645
47	4	23.3	1.104	0.579824282
1	1	18.8	1.307	0.095614848
146	8	83.4	0.895	0.489678641
13	4	53.1	0.474	0.456168095
441	2	49.6	1.453	0.055517068
60	4	98.3	0.994	0.534813292
16	4	53.8	0.62	0.041278102
86	9	44.7	1.117	0.351859897
4	2	83.5	1.5	0.246814732
602	49	468.5	1.007	0.98858754
21	2	85	1.118	0.974672232
2	1	26.2	0.619	0.184829705
59	2	11.7	0.776	0.070737183
25	6	59.3	0.813	0.586795827
128	2	129.9	2.131	0.354407819
69	16	64.8	0.808	0.151783311
29	1	19.4	1.207	0.196145914
2	1	42.2	0.49	0.00739901
330	11	151	0.984	0.974550643
6	1	109.6		
431	34	240.7	0.73	0.058906434
28	3	30.9	1.271	0.20697442
102	13	59.6	1.281	0.128693293
311	16	39.8	0.872	0.367498271
70	5	125	1.064	0.38725535
13	1	149.5	1.264	0.511020534
499	1	271.4	0.889	
80	6	56	0.784	0.038442497
109	11	51.2	0.93	0.788240423
3	1	20.7	1.213	0.126444493
122	6	146.4	1.009	0.864671772
56	1	13.3	0.124	0.22531639
1	1	16.7	1.032	0.960740416
2	1	30	1.043	0.405255637
12	1	28.8	0.866	0.942202939
5	1	25.1		
22	2	39.9	0.99	0.386227162
484	16	37.4	0.839	0.083238085
13	2	388.7	1.348	0.802465555
1	1	102.8	4.488	0.029814068
348	11	26.4	0.953	0.862187452
517	27	84.5	0.9	0.20671572
10	4	186.2	0.622	0.181441832
113	10	91.1	0.935	0.63456981

13	1	56.9	0.769	0.260479898
8	2	58.1	1.411	0.041981271
222	13	58.4	1.099	0.507471429
24	4	69.7	1.126	0.517449579
73	4	24	1.153	0.226082927
30	1	12.9	0.88	0.359869244
36	2	23.7	0.863	0.243455579
11	1	12.9	1.722	0.037807621
10	1	4.8	1.209	0.100175607
1	1	81.7	0.001	0.001
14	4	45.8	0.747	0.013828836
25	7	343.5	0.857	0.284613069
6	3	52.5	1.07	0.653429085
53	1	48.3	2.535	0.271060292
8	2	223.6	0.994	0.831072378
362	23	90.7	1.071	0.448493282
5587	1	80	0.341	0.012114933
181	1	90.1	1.125	
2	2	17	0.731	0.655357157
104	6	32.3	1.233	0.174455037
31	9	64.8	0.79	0.332869976
196	15	88.7	0.973	0.888857718
1	1	39.6	0.448	0.179704628
7	2	23.8	0.906	0.987381508
10	4	19.8	1.014	0.807326812
14	2	24.9	0.813	0.629824994
3	1	49.6	1.188	0.273855999
33	3	18.4	0.929	0.667094646
73	2	225.4	0.711	0.202453342
20	3	66.3	0.988	0.958149594
1063	4	43.8	1.042	0.570396118
230	15	57.7	0.855	0.321718152
4	1	29.3	0.001	0.001
19	2	8.7	1.708	0.11774093
13	1	39.4	0.884	0.463915621
1	1	6.9		
138	2	49.8	0.998	0.743214705
123	1	11.3	0.532	0.137100071
300	1	20.7	0.226	0.009718532
10	1	32.9	0.694	0.219029961
82	7	19.1	1.111	0.372196018
1293	1	57.6	0.85	0.132286136
270	17	97.3	0.795	0.150864888
585	16	54.6	1.423	0.096130696
75	1	41.4	1.455	0.839076653
1	1	67.2	0.783	0.449037602
15	3	51.6	1.109	0.722894714
28	1	26.4	0.73	0.14367718
57	5	13.1	0.86	0.764619998
5	1	30.6	0.813	0.167206878

11	4	17.6	1.155	0.590966922
9	1	13.3	0.968	0.912488243
204	5	45.2	1.001	0.841517153
14	1	16.9	1.006	0.71859453
5	2	21.5	0.696	0.042004772
29	3	45.6	1.474	0.013916333
5	1	13.6	0.605	0.074552539
13	1	51.8	1.177	0.311376895
219	14	45.4	1.167	0.44206308
3	1	39	1.065	0.885826638
2	2	33.1	0.02	0.110840975
4	1	32.5	1.261	0.397568218
52	6	133.7	0.66	0.078516063
1	1	106.9	1.435	0.101489656
8	2	38.8	0.767	0.513028043
70	6	61.3	1.153	0.290355449
20	3	7.1		
25	3	58.8	1.47	0.980128809
7	2	14.1	1.06	0.256725388
10	2	84.7	1.365	0.01499215
5	1	68.9	0.949	0.559149624
1	1	31.2	0.259	0.120949817
50	1	11.2	0.378	0.146337034
22	2	25.3	0.883	0.862450791
268	5	28.7	0.805	0.48165902
4	1	23.6	1000	0.001
1208	13	24.1	0.989	0.890724848
92	4	74.2	1.146	0.675440765
367	1	52		
15	2	23.6	1.099	0.508225523
14	1	7.6	0.952	0.657750916
112	12	110.1	1.198	0.193251881
1	1	15.6	0.212	0.678779989
1	1	82.9	0.001	0.001
4	4	30.3	13.384	
235	1	32.7		
1	1	47.3		
3	1	19.5	0.001	0.001
19	3	31.1	0.52	0.286649529
10	3	75.9	0.68	0.371917068
50	8	104.6	1.441	0.048282674
11	1	51.8	1.04	0.88697687
142	10	38.6	0.871	0.330889871
1	1	19	1.396	0.687396068
2	1	22.9	0.85	0.365137868
3	1	27.3	1.289	0.384211835
2	1	68.5	1.197	0.34386278
19	1	99.3	0.595	0.020133854
36	2	36.1	0.612	0.162714043
11	1	18.2	0.829	0.729552466

84	7	54.3	0.837	0.326588057
251	2	32.6	1.168	0.086596356
59	8	59.7	1.119	0.385139659
285	18	120.2	0.936	0.734015384
884	1	21.4	0.896	0.808993858
256	11	29.1	1.017	0.749622704
18	3	63.7	0.744	0.263212788
123	4	30.6	0.737	0.164351776
12	1	114.9	0.906	0.736971346
4	1	25.8	0.001	0.001
59	11	33.7	1.163	0.074087557
44	5	49.7	1.034	0.736486904
56	1	11.8	0.616	0.29487127
1	1	30.2	0.808	0.229594608
181	10	33.8	1.113	0.261532019
6961	1	103.8	1.595	0.130650325
1	1	14.8		
3	1	10.3	0.957	0.671016777
2	1	13.1	0.626	
249	16	49.9	1.165	0.313697936
1	1	28.4		
1	1	19.6	1000	0.001
25	1	47.6	1.165	0.139955549
4	1	47.2	0.264	0.608401778
84	1	43.8	1.292	0.023636913
78	3	11.1	0.944	0.601084107
26	5	51.8	0.592	0.690420361
369	9	20.2	1.001	0.751543831
122	8	50.8	0.841	0.610244168
370	11	68.9	0.724	0.056385259
412	10	104.8	0.989	0.818219186
1	1	20.3	0.001	0.001
203	4	69.3	0.586	0.081921309
2	2	13.2	0.001	0.001
43	1	188.6	1.12	0.679325035
3	1	52.4	1.115	0.562972542
87	6	49.9	0.988	0.818690713
2	1	42	0.512	0.870056875
150	6	20.4	1.039	0.640395993
74	4	11.8	0.856	0.542669336
174	10	32.6	1.346	0.083661322
2	2	19.9	0.916	0.378699862
1	1	39.4	1.107	0.390502106
1626	44	63.3	0.656	0.047461101
563	4	78.3	0.936	0.878995091
5	1	73.4	1000	0.001
23	3	52.7	0.952	0.569522544
17	4	40.2	0.14	0.000107033
39	1	122.4	0.968	0.711204911
18	2	14.5	0.89	0.602010322

21	2	53.4	1.852	0.299426841
3	2	18.3	1000	0.001
46	3	38.2	1.103	0.915152499
18	4	37.4	1.078	0.431168974
15	1	68	1.154	0.412118929
67	2	21.3	1.591	0.226480447
54	2	135.9	0.854	0.333648453
5	2	30.8	1000	0.001
3	1	13.9	1000	0.001
10	3	65.3	0.834	0.38062691
27	1	5.5	1.435	0.371922101
2	1	6.3	1.239	0.324044205
11	3	24.6	1.203	0.582765769
7	2	30.9	1.469	0.178699046
10	1	14.7	1.767	0.00453891
115	7	38	1.132	0.205775538
88	6	75.6	0.932	0.772209173
9	1	25.9	0.785	0.350648137
91	11	136.8	1.313	0.023199337
55	6	25.9	0.876	0.687750478
33	7	71.3	0.646	0.048321375
42	6	32.9	1.031	0.794381515
376	19	53.2	0.807	0.942580243
7	1	12.3	0.749	0.427774482
3	2	84.5	1.136	0.570610523
9	1	46.5	0.962	0.643846609
8	1	4.9	0.903	0.687513774
50	1	40.5		
3	1	41.2	0.001	0.001
24	2	26.6	0.739	0.234689351
91	7	49.8	1.315	0.16059554
211	1	45.7	0.96	0.547961522
34	3	13.6	1.046	0.929674367
1	1	15.9		
21	1	17.9	1.184	0.404571204
4	2	39.2	0.069	0.289966212
72	7	54.7	0.936	0.919147467
7	2	24.8	0.983	0.96251352
165	1	14.2	0.936	0.895675966
16	7	80.9	0.742	0.109973551
1	1	17.3	0.001	0.001
1	1	80.4	0.001	0.001
5	2	121.6	1.045	0.996610934
17	2	17.4	1.095	0.341465592
1	1	10.5	0.868	0.504707771
25	1	25.2	0.813	0.411398421
24	2	126.8	0.717	0.114836034
2	1	68.5	1.276	0.628991442
528	3	277.9	0.678	0.330080698
27	4	168.3	0.832	0.326761343

30	4	63.3	1.178	0.154363507
24	2	28.9	1.079	0.913333788
3	1	26.3	0.841	0.382576881
5	1	16.5	1.275	0.235714516
85	9	32.1	0.892	0.394334207
342	1	40.6	0.818	0.623899097
125	1	161.1	0.785	0.013507813
6	2	29.4	0.669	0.66025897
10	1	124.4	0.827	0.448880214
17	2	39	0.91	0.462129842
29	3	38.7	0.684	0.322385914
23	1	14.4	0.55	0.098318542
11	1	32.5	0.693	0.159123142
24	2	129.1	1.258	0.291700096
26	2	12.2	0.72	0.075188938
43	3	56	0.922	0.366543301
6	1	28.6	1.105	0.728839971
13	2	28.3	0.89	0.659175397
7	4	16	0.661	0.509710285
68	4	32	0.116	0.007130563
7	1	36.3	1.145	0.293011834
2	1	53.2	1.055	0.441363855
13	6	51.2	0.714	0.092791648
1	1	9.3		
320	6	69.3	1.012	0.948035696
149	9	48.6	0.939	0.892486504
9	1	26	1.81	0.00302481
222	16	61.7	0.842	0.512101375
43	4	83.8	1.066	0.654138214
1	1	51.5	0.307	0.126344025
26	6	84.3	0.863	0.78578221
16	1	64.1	0.851	0.426057216
1	1	13.8	1000	0.001
2	1	40.9		
5	2	123.8	1.296	0.253530947
28	2	30.7	1.711	0.003147085
26	2	23	0.786	0.435765812
19	4	27.6	0.688	0.168698365
1630	8	20	1.17	0.567103733
796	4	42.3	0.62	0.141903612
275	18	70.1	0.786	0.069850836
4	2	20.3	1.268	0.599942833
217	1	99.7	1.702	0.367878227
39	1	61.3	1.119	0.592516949
36	2	40.9	1.11	0.286818333
18	3	18.8	0.609	0.329518869
87	7	58.9	0.711	0.00989525
32	4	54.6	1.277	0.169851022
1	1	27.1		
1	1	9.2		

17	3	124	1.015	0.73521758
3	1	30.4		
207	1	11.6	1.196	0.223989978
44	4	53.4	0.86	0.128002852
4	1	20.7	0.774	0.302997329
1	1	131.6		
16	1	55.2	1.09	0.558557909
5	1	52.1	0.92	0.526830896
13	1	38.2	0.85	0.635551357
2	2	24.2	0.945	0.657470267
185	1	21.9	1.022	0.651481975
1	1	111.6	1.032	0.529080903
1	1	36.4		
133	12	142.3	1.108	0.309437605
15	1	5.6	0.989	0.876701694
29	1	91	1.1	0.649623808
7	2	28.5	0.994	0.983655563
13	4	49.7	0.966	0.918395154
17	6	118.3	1.221	0.885290552
6	1	7.5	0.676	0.55877736
100	4	16	1.28	0.080730967
7	1	6.4	1.058	0.4691122
58	4	19.2	0.894	0.235158465
325	1	32.8	1.183	0.495926294
4	2	16.9	0.77	0.287719155
3711	2	120.7	1.213	0.293383432
4	1	11.6	1.814	0.010354439
3	1	16	1.636	0.1026353
1545	1	33.3	0.661	0.012685969
17	1	9.1	0.762	0.152893175
28	4	79.8	1.143	0.784532913
945	2	36.5	1.142	0.963552074
2	1	31.1		
27	1	42.2	1.083	0.855731598
1	1	40.6		
28	3	44.7	0.778	0.508335517
40	4	12.2	0.932	0.502129321
29	1	23.8	0.988	0.789280857
175	18	102.9	1.382	0.032971471
4	1	13.5	1.455	0.066816389
15	1	32.4	0.334	0.158104424
20	4	42.9	1.7	0.206062928
225	1	23.1	0.801	0.065125566
4	1	39.9	1.048	0.581756502
7	1	166.6	1.178	0.319395866
61	4	67.9	0.642	0.097227999
1	1	31.2	1.339	0.509046066
5	2	16.1	1.012	0.89674336
125	5	9.4	0.815	0.221479273
21	3	32.8	0.855	0.196105923

174	9	61.9	1.329	0.148301711
14	1	20.8		
219	9	37.5	0.957	0.952821489
58	6	24.9	1.05	0.749100014
16	1	73	0.903	0.723881358
6	1	36.4	1.581	0.003133885
2	2	36.4	0.321	0.814448331
15	2	43.7	0.81	0.125820771
105	1	89.3	0.65	0.040356226
30	4	39.4	1.342	0.180972835
16	2	25.3	1.238	0.452522958
89	7	26.2	0.758	0.807541357
259	2	37.4	0.84	0.233675439
179	10	101.5	0.988	0.93835281
1	1	4.8		
17	2	20.5	0.856	0.305212751
9	1	24.8	1.295	0.200858124
102	6	191.9	1.135	0.354016942
623	37	455.9	1.098	0.624775764
26	1	23.4	1.068	0.457413104
28	3	21.5	1.16	0.43870221
2	1	92.4	0.693	0.774447859
7	2	39.2	1.406	0.100932829
148	8	31	0.767	0.109106607
9	2	57.4	1.291	0.167815192
8	2	36	0.842	0.575160833
23	4	83.2	0.784	0.354193252
5	1	134.4	0.76	0.316485859
100	9	29.5	0.881	0.499454571
1116	20	75.1	1.002	0.740435177
49	1	37		
29	1	152.1	0.945	0.910272412
179	11	25.8	0.503	0.118062227
52	6	33.2	0.865	0.501780642
44	15	279.8	0.994	0.88661064
22	1	8.2	0.185	0.127072478
189	8	59.5	0.741	0.222483124
28	2	24.6	1.152	0.284027887
75	4	17.6	0.523	0.022618168
1	1	11.2	0.865	0.420854225
139	9	47.7	1.07	0.317817167
1	1	54	0.001	0.001
2	1	10.7	0.774	0.096242673
52	6	58.3	1.666	0.001265848
116	5	59.8	1.509	0.004758985
2	1	52.8	1000	0.001
18	4	55.7	0.209	0.008110399
46	2	29.9	0.582	0.707199837
46	2	25.3	0.985	0.931853332
196	8	22.1	1.115	0.185148148

368	8	43.2	0.885	0.453295238
111	8	42.4	0.801	0.10033346
34	2	127.5	0.773	
41	5	49.8	1.179	0.539173717
147	6	21.9	1.443	0.05932419
15	2	34.6	0.95	0.97671449
202	13	62.6	0.855	0.586000944
29	4	138.7	0.646	0.042138678
11	1	39.6	1.382	0.094621583
34	1	23.5	1.145	0.47402014
41	3	28.7	0.859	0.792223932
163	4	13.5	0.41	0.997279247
17	1	96.1	0.841	0.522715737
59	5	41.5	1.272	0.257549651
5	5	65.4	0.854	0.391555494
118	3	17.6	0.986	0.985507486
197	11	75.2	0.809	0.076970077
9	2	15.8	0.926	0.683676017
28	1	18.7	0.844	0.723439763
7	1	63.9	1.09	0.900064207
23	3	74.8	0.8	0.262217893
175	11	50.6	1.205	0.152666646
171	6	12.8	0.948	0.96585995
3	1	8.2	0.312	0.040847855
124	7	27.4	1.119	0.298282053
2	1	147.8	0.825	0.709663536
19	3	49.8	0.678	0.116568619
7	3	11.7	0.352	0.459794604
38	4	46.2	0.532	0.045749124
3	2	75.3	1.162	0.660734787
102	3	21.4	1.359	0.032785481
10	2	47.1	1.142	0.664242042
21	6	246.3	0.885	0.999355216
65	5	205	1.095	0.922292548
221	24	81.7	0.286	0.033227575
27	1	34.9	0.949	0.970815582
318	9	31.5	1.262	0.085770918
59	6	119.7	0.883	0.865444964
484	25	61.5	0.783	0.42524953
9	1	113.7	1.506	0.095636557
221	6	49.4	0.875	0.446058103
104	6	46.2	1.027	0.811305223
1	1	24.8		
22	2	22.4	1.343	0.83489323
1	1	62.5		
12	3	55.6	1.76	0.015500122
26	3	62.9	1.005	0.804952412
1	1	22.5	0.811	0.996596864
7	2	20.4	0.526	0.076443394
46	3	53.6	1.066	0.997603805

53	5	10.9	1.114	0.38972209
124	13	112.8	0.866	0.255605272
19	4	53.3	1.046	0.522839705
21	3	42.1	0.485	0.391387348
81	4	16.8	1.26	0.033295987
235	9	87.5	0.883	0.720551019
29	4	42.6	0.812	0.777870035
78	6	27.3	1.147	0.145428093
27	6	52.8	0.762	0.468153766
2	1	108.1	0.714	0.135483362
155	9	36.4	0.703	0.009804994
41	4	35	0.918	0.652505203
2	2	22.6	1000	0.001
77	3	40.4	1.055	0.829560279
405	3	39	0.819	0.877561351
7	2	35.3	1.615	0.546992974
207	2	27.8	1.112	0.51409323
71	6	100.6	1.218	0.130867763
1	1	49.6		
3	1	57.3		
4	2	31.1	0.779	0.268556921
556	5	30.6	1.133	0.807029732
909	4	37.6	0.843	0.065194222
52	4	48.2	1.344	0.059342579
6	4	61.4	0.623	0.082077078
137	18	113.7	0.375	0.030890764
6961	60	106.4	1.033	0.590744818
33	5	81.2	0.754	0.275610373
39	5	85.1	1.09	0.397440501
5	2	51.7	1.279	0.047975595
215	11	53.1	0.927	0.287897029
111	12	120.8	0.079	0.255214345
7	2	24.9	0.983	
1061	28	75.5	1.005	0.973855343
665	12	54.7	0.92	0.433137792
12	3	55.7	0.778	0.610505613
463	17	54.6	0.94	0.771182165
22	3	28.1	1.212	0.16020855
4	1	71.5	0.489	0.130291448
3	1	40.9	0.875	
17	1	21.7	0.682	0.654197731
139	9	61.1	1.074	0.668774163
81	3	20.1	1.03	0.748299006
40	6	55	1.332	0.204857424
6	2	19.2	0.617	0.066351672
240	10	40.1	1.117	0.43627845
26	5	50.7	1.673	0.35129469
1283	8	83.2	0.898	0.136878446
169	8	43.7	1.054	0.524502197
33	6	55	1.221	0.106781254

33	5	51.7	1.54	0.269360681
11	2	112.8	1.117	0.475479057
73	7	37.1	1.118	0.319865932
302	14	47.1	0.818	0.362338259
224	14	78.2	0.832	0.200222645
268	14	26.9	1.014	0.894067825
64	7	109.7	1.138	0.131379561
32	6	97.4	0.643	0.001842874
455	4	28.1	1.021	0.531618695
64	2	39.8	1.544	0.645597849
281	12	13.7	0.847	0.296805157
2	1	29.4	1.073	0.877568437
3	2	35.2	0.608	0.554487162
32	2	17.2	0.849	0.341985722
1044	14	37.6	1.13	0.418369284
5	1	12.7	1.367	0.350079912
98	8	35.5	1.143	0.316469954
104	4	42.2	0.654	0.158419985
28	2	66.6	0.81	0.803984035
3	2	120.9	0.814	0.759598412
342	1	44.1	0.474	0.137979837
20	3	39.7	1.473	0.201666373
35	1	57.9	1.298	0.228087673
116	17	273.3	1.008	0.956822212
229	11	35.9	0.719	0.098533728
26	10	44.9	1.674	0.732873592
175	8	27.9	1.045	0.941531414
56	2	55.8	0.325	0.032246039
32	4	57.7	1.134	0.969286264
10	3	34.7	0.523	0.382250705
5	1	108.5	1.065	0.706298343
2009	43	104.7	0.854	0.115391321
510	11	22.3	1.011	0.697530594
19	4	31.6	1.074	0.728973781
54	1	39.8	4.615	
78	6	136.6	0.883	0.178348729
139	5	19.7	1.131	0.274684452
1259	29	63.1	0.988	0.799329143
304	23	113.2	0.879	0.26564003
5	2	92.2	0.841	0.274227878
3	2	29.5	0.888	0.584010692
12352	49	139	0.87	0.150215517
7	3	86.3	0.883	0.773899391
3	2	38.3	1.659	0.27716811
45	8	45.9	2.369	0.151138861
7	1	297.7	0.869	0.992268574
48	8	38.7	1.1	0.693210683
12	2	53.6	0.901	0.681941163
144	5	16.3	1.012	0.837609559
1390	17	65.1	1.116	0.270414043

8	1	35.7	1.209	0.292619682
10	3	35.1	0.803	0.735228649
11	4	59.7	1.423	0.064020817
110	6	32.9	0.942	0.989307323
6	1	21.8	1.166	0.150682624
3902	30	52.5	1.12	0.207720476
3	1	23.3	1.855	0.000946345
4	3	52.4	1.472	0.439369527
29	1	61.2	0.96	0.923403291
484	17	18.5	0.831	0.208103615
36	3	19.6	1.332	0.115816481
1	1	56.9	1.106	0.8741126
26	3	72.5	1.388	0.404236588
892	27	501	0.812	0.13167435
86	7	112.8	0.483	0.129117505
52	6	113.4	0.79	0.664330887
8	4	100.2	0.974	0.985568464
287	14	71.4	0.845	0.221525624
105	7	22.1	1.209	0.257931392
52	1	10	1.175	0.28504986
153	8	97.4	0.704	0.279579661
12	1	17.7	0.755	0.123560673
29	3	21.2	1.557	0.424758005
24	3	35.5	0.658	0.910788752
22	4	138.3	0.939	0.675851183
61	10	51.6	1.724	0.275654659
90	6	47	1.674	0.019941156
9	1	113.6	1.369	0.03140493
27	1	47.3	0.74	0.164940522
5	1	26.9	1.108	0.748262306
28	5	628.7	0.822	0.115086337
1	1	79.1	8.435	
4524	46	45.3	0.914	0.380964258
59	4	54.4	1.093	0.532422308
513	30	109.5	0.751	0.135426933
16	2	50.5	1.181	0.040678069
14	2	40.5	1.22	0.81617699
39632	214	187	0.925	0.218845733
11	3	111.3	0.797	0.066557743
20	2	30.7	0.617	0.319779586
15	3	54.3	2.191	0.028777506
20	4	23.4	0.621	0.080962789
829	17	38.2	0.679	0.000906942
3055	3	42	1	0.988810823
109	3	35.4	0.905	0.465173673
21	5	35.9	0.811	0.46866981
318	9	61.1	0.778	0.126653956
2	2	22	0.857	0.845531876
26	5	74.7	0.799	0.828058944
3	2	25.2	1.03	0.531494898

4	1	33.3	0.001	0.001
6	2	55.5	0.783	0.268615578
24	1	74.4	1.511	0.133374331
26	3	76.7	1.104	0.397089445
114	6	20.5	1.064	0.472906919
294	12	34.3	1.128	0.132301998
197	11	38.1	0.846	0.297608555
99	3	30.7	1.063	0.41142944
32	5	11.1	0.727	0.454033116
13	2	86.9	0.624	0.841854709
221	3	21.2	1.072	0.58627647
44	4	109.2	1.113	0.506326114
9	1	111.6	1.254	0.231779312
14184	1	192.7	0.579	0.010839376
35	1	16.9	0.598	0.124132561
11	2	45	0.871	0.343652306
89	10	37.8	1.118	0.774245249
42	4	37.4	1.558	0.193150901
13	2	45.2	1.323	0.045457765
5	1	23.1	0.856	0.508592202
52	1	60.1	0.822	0.181962218
9	2	9.1	0.585	0.338651203
98	5	22.3	0.984	0.914276875
1391	7	39	1.136	0.262950108
1	1	91.8	0.554	0.011910849
223	10	53.5	0.86	0.532370042
10	1	8.5	0.969	0.692117306
1	1	74.9		
242	13	61.8	1.395	0.047308741
10	2	28.8	0.855	0.466944498
1934	27	76.6	1.049	0.50794319
360	12	68.4	0.654	0.008734413
49	6	54.7	1.41	0.035505205
4	1	16.2	1.8	0.49708985
3463	65	188.2	1.069	0.285430585
477	17	53.2	1.028	0.603941323
29	2	105.4	1.049	0.620312263
48	5	25.2	0.732	0.535244624
34	6	65.3	1.341	0.049636701
25	1	93.8	0.72	0.451769801
37	4	98.3	0.995	0.741594823
117	2	16.9		
6	1	27.7	0.951	0.530409699
77	7	99.7	0.821	0.875055166
9	3	132.5	1.34	0.392422922
20	4	49.7	0.621	0.86080566
476	78	331.6	0.662	0.120012948
4	2	74.4	0.866	0.62909001
151	15	54.8	1.616	0.730988234
6	1	57.9	1.308	0.043816373

5	3	217.2	1.31	0.966775208
249	11	44.7	1.245	0.033702141
28	4	15.9	0.442	0.09150341
70	4	48.5	1.047	0.697955769
5	1	120.9	0.785	0.275694107
2	1	47.5	0.001	0.001
20	6	14.2	1.255	0.701461253
4	3	51.2	0.085	0.011650481
1	1	17.4	0.906	0.937874563
7	1	91.3	1.567	0.092504922
66	11	56.1	1.165	0.185663058
151	8	35.9	0.927	0.779524597
55	5	111.2	1.252	0.210271438
39	2	290.3	1.051	0.409554961
59	6	138.9	1.207	0.126370787
968	26	36	0.955	0.953849241
18	1	48.7	1.384	0.03977971
6	2	38.4	1.593	0.03594395
9	4	11.8	1.066	0.523366134
355	13	43.9	1.197	0.239109543
7	1	16.8	0.714	0.2247946
51	3	43.9	0.402	0.196384811
97	7	50.7	1.933	0.033261239
1	1	32.8		
1	1	101.9		
11	2	55.7	1.538	0.316015268
36	2	26.4	1.118	0.237080335
117	10	101.7	1.321	0.617605071
99	5	33.8	0.437	0.454196865
80	7	145.1	0.975	0.675861643
38	2	66.2	0.961	0.590142507
135	11	46.2	1.129	0.697033747
1	1	39		
137	7	19.3	1.157	0.402946481
432	26	81.8	1.293	0.083096425
172	2	290.8	0.843	0.827247714
368	5	65.3	0.978	0.946883363
8332	52	52.6	0.886	0.2687861
85	5	47.1	0.788	0.20765704
509	8	25.8	0.922	0.447590763
63	4	36.1	1.138	0.352199687
94	5	25	1.132	0.398158666
5	1	141.6		
25	7	126.9	1.074	0.445586175
8	2	98.5	0.397	0.077479642
3	1	35.2	0.757	0.958598691
552	16	64.4	0.832	0.134988142
19	3	106.4	0.755	0.823806198
2974	11	54.2	0.782	0.005937098
14	1	42.6	1.186	0.494615198

111	13	76.2	1.182	0.174621727
27	4	22.7	0.548	0.003772639
7978	36	67	0.976	0.888288532
3	1	11.3	0.853	0.74654381
7	1	54.4		
661	12	27.7	0.986	0.871196285
674	21	39.4	0.934	0.944099008
8	1	55.7		
3	2	78.5	1.946	0.076651536
217	12	47.3	1.348	0.08520485
1398	33	158.4	1.059	0.66043464
1	1	37.8	0.964	0.78633477
583	5	343.5	1.072	0.33121305
265	12	153.5	1.276	0.125279394
2129	39	93.5	1.037	0.581292588
969	24	67	1.198	0.282228754
47	9	56.5	1.087	0.371516549
10	1	40.3	0.166	0.376201674
28	2	10.1	0.72	0.028637376
4	1	24.5	0.904	0.470597989
198	16	124.5	1.007	0.983558645
205	14	161.6	0.227	0.102131856
5	2	25.1	0.918	0.853475378
848	14	60.5	1.046	0.47431862
210	9	54.2	1.407	0.052405685
28	11	99.9	0.456	0.469270596
35	5	92.4	1.153	0.950809657
16	4	47.5	1.103	0.296309143
36	5	36.6	0.934	0.656403776
88	6	82.2	0.945	0.810188371
9	2	20.1	1.94	0.043699334
116	6	35.7	0.635	0.018244522
44	4	30.4	0.995	0.898504884
16	3	99.9	1.526	0.018512193
91	8	27.5	1.015	0.535676577
3	1	53.8		
927	26	103	1.054	0.694989421
71	5	33.4	1.28	0.105430871
77	5	46	1.074	0.372258994
956	10	45.1	1.006	0.803594778
32	2	35.1	1.225	0.076137092
5	2	45.4	0.919	0.817030121
4	1	88.9	1.036	0.640557509
7	4	61.4	1.084	0.573987575
282	11	69	0.707	0.096903671
636	19	47.1	0.889	0.309797567
598	21	70.1	0.779	0.097314505
1521	2	42	1.072	0.507208948
79	7	39.6	1.31	0.141227347
175	2	40.9	1.335	0.072813509

548	21	51.9	1.231	0.093149043
40	3	57.5	0.927	0.790598779
264	8	27	0.638	0.018977966
26	1	25.1	1.142	0.306348243
2	2	37.5	0.644	0.046265465
23	1	92.2	0.696	0.017661437
444	17	82.8	0.896	0.372697157
369	18	51	1.253	0.06045365
100	4	20.5	0.993	0.943696539
11	1	34.6	0.593	0.039397807
28	5	35.8	0.95	0.556014218
3	2	125.4	0.542	0.108509646
1	1	109.2		
44	1	64.7	1.011	0.475256438
867	9	28.3	0.985	0.98772528
1	1	88.9		
14	1	22.1	1.153	0.295628808
1	1	87.3	1.258	
958	23	60.6	1.052	0.551051992
1387	2	46.7	0.929	0.639314191
6	2	34.7	0.495	0.205322259
521	1	161	0.865	0.79716463
379	12	29.2	0.971	0.724485862
2	2	76.8	0.001	0.001
6	1	41.5	1.286	0.346985737
194	11	39.7	1.094	0.223278507
1	1	79.1		
3670	37	69	0.874	0.286965649
1185	10	10.8	0.829	0.362572479
1	1	9.8	0.001	0.001
250	22	48.1	0.92	0.614586026
83	6	32.9	1.02	0.617739718
203	13	29.2	0.976	0.946048314
507	15	56.7	1.13	0.30977458
62	3	11.3	0.62	0.006992851
1	1	67.3	0.732	0.089571723
77	6	39.4	0.815	0.191331704
261	13	84.6	0.966	0.772818606
14	1	33.4	1.267	0.071282791
3	2	26.2	0.001	0.001
17	10	40.2	0.545	0.245547526
861	20	66	1.226	0.046622382
29	4	162.1	1.111	0.438955684
11233	64	30.8	0.998	0.883948485
69	12	38.6	0.754	0.062765663
5	2	41.2	2.323	0.04509631
3	2	46.5	1.699	0.054710834
636	26	72.3	0.823	0.095123125
49	5	51.8	1.189	0.314216231
1	1	31.4		

9	5	44.4	0.966	0.696787136
2	1	115.8	1.216	0.422045983
29	6	250.4	0.733	0.403409954
204	3	332.9	0.697	0.102303352
222	1	48.2	1.086	0.323824524
46	4	26.2	1.415	0.063508373
2191	77	163.2	0.848	0.201045229
115	6	149.4	0.846	0.957144398
76	4	20.6	1.408	0.014100013
411	13	28.9	1.065	0.457016814
8	3	24.9	1.091	0.967262917
1	1	317.3	0.892	0.538696461
135	4	28.6	1.01	0.688333361
104	7	45.5	0.855	0.627304175
4	1	43.4	1.664	0.155915078
193	5	18.5	1.092	0.32000335
37	5	53.3	0.792	0.838284263
72	7	46.9	0.986	0.602159147
418	3	37.3	0.62	0.047159647
150	9	25.2	0.727	0.990016214
68	2	21.8	1.224	0.372263006
48	5	25.8	1.381	0.115200973
11	3	35.8	1.402	0.166225855
178	4	26.7	0.84	0.30026188
3	1	23.1	0.001	0.001
4334	14	38.3	0.775	0.040152878
413	11	48.4	0.921	0.405195218
12	1	88.2	1.016	0.788419609
2	1	12	1.152	0.764093069
1	1	38.6		
89	7	50.1	1.429	0.022037171
114	11	46.3	1.163	0.827915377
51	3	17.2	0.88	0.443927208
10	1	65.7	1.264	0.166356476
6	2	17.7	2.83	0.47246354
45	7	43.1	1.147	0.291152582
107	7	41.3	0.747	0.012226021
141	14	107.2	1.384	0.142925534
79	5	51.6	1.088	0.504397325
104	2	12.7	1.039	0.969457233
6	1	13.2	2.193	0.089826297
457	2	12.9	0.953	0.706858721
150	5	12.9	1.09	0.872265952
38	2	13.1	1.191	0.397314762
69	4	13.8	0.727	0.069845397
373	1	12.8	1.02	0.930966974
25	1	13.1	0.769	0.157394507
59	5	13	0.899	0.674555266
59	1	12.8	1000	0.001
98	1	12.9	0.001	0.001

125	9	111.3	1.012	0.922572907
64	1	12.7	2.705	0.040401324
35	1	12.8	0.001	0.001
55	1	13	0.854	0.533555238
46	1	12.8	1.006	0.804884161
205	9	46.6	0.725	0.464824239
25	2	13.5	1.26	0.128774079
391	1	12.8	1.415	0.109416097
70	2	12.8	1.43	0.147496484
84	1	12.9	0.928	0.785032872
239	12	50.9	0.908	0.351791877
21	3	22.3	0.878	0.572747513
136	9	56.8	0.571	0.352119439
119	1	57.8	1000	0.001
257	8	65.4	1.076	0.180751183
13	2	52.9	1.062	0.576979906
8	1	43.5	1.033	0.376249655
27	4	26.5	0.947	0.698912362
78	2	13.2	0.813	0.229587927
54	4	14.7	1.074	0.550769021
1	1	23.8	1.402	0.023373239
6857	21	47.6	0.878	0.259725772
22	5	54.6	1.057	0.657772957
54	4	27.7	0.495	0.011878732
4	1	25.5	0.561	0.852647873
4	2	164.8	1.065	0.552624777
5	2	19	1.111	0.209786848
3	2	12.4	1.14	0.231393271
459	14	36.7	0.975	0.781289072
1446	50	251.5	1.004	0.781245267
60	4	37.4	0.786	0.168419786
25	5	29	0.822	0.742740171
527	14	51.7	0.933	0.473982223
16	2	9.7	0.863	0.600243927
23	1	58.2	0.604	0.15974474
680	2	60	0.208	0.085647627
699	1	60	0.001	0.001
10	3	62	0.704	0.305315904
29	4	103.5	0.678	0.024758471
43	1	51.4	0.222	0.005757809
11	2	42.6	0.911	0.587477224
4099	28	41.7	1.193	0.137986341
18	2	13.6	0.824	0.864171294
11	1	40.7	0.927	0.485448436
1083	19	67.7	1.143	0.167048082
71	6	11.9	0.97	0.958729474
39	7	64.7	0.953	0.765746902
3	1	25.2	0.803	0.539163259
49	4	37.4	0.75	0.211755684
60	7	52.2	8.003	0.00457518

47	1	13.1	1.042	0.496943071
200	14	29.7	0.87	0.460518455
366	14	60.8	0.835	0.259273556
48	6	434.9	0.322	0.077656715
10	4	54.6	1.431	0.090754696
20	1	38.3	0.637	0.025446188
259	8	83.2	0.878	0.240323586
99	5	18.1	0.932	0.73178228
13	1	114.5	0.826	0.153581036
59	6	20.8	0.781	0.022204179
3	1	34.9	1.168	0.405593141
14	5	555.3	0.945	0.835005222
5	3	32.6	1.298	0.061370791
23	2	24	1.513	0.154767575
731	24	70.9	1.18	0.39237347
2	2	46.7	1.31	0.1055409
6	2	96.8	0.786	0.016682634
201	12	34.8	0.617	0.041360346
544	7	48.1	0.417	0.114201906
17	5	25.6	1.259	0.331291472
4	2	18.7	4.745	0.347698889
10	1	12.6	0.826	0.284982146
4	1	28.1		
29	7	37.4	1.385	0.053974736
17	2	98	0.805	0.265752037
4	3	74.8	1.962	0.014049371
2	1	68.4	0.112	0.318264393
163	2	59.5		
586	22	113.3	0.401	0.022497034
47	6	34.9	0.921	0.97749933
67	5	43	1.158	0.18228299
41	3	104.3	0.876	0.438153098
16	4	57.5	0.792	0.261202995
18	1	65	1000	0.001
13	1	18	1.552	0.10315682
93	12	81.8	1.037	0.500223869
176	13	62.3	1.082	0.571018868
59	5	41.2	0.713	0.483052403
37	5	27.8	1.11	0.584386007
45	10	62.5	0.715	0.799960308
100	1	278	0.843	0.463365384
1500	78	571.6	0.878	0.534625319
1353	46	94.9	0.804	0.067842585
257	3	23.6	0.766	0.1669219
377	6	28.2	1.251	0.046642033
23	2	24.7	0.916	0.60588184
79	4	10.2	0.981	0.803664025
12	2	22.1	1.119	0.23728214
17	5	35.1	0.964	0.224974754
164	8	54	1.037	0.621256474

18	4	21.2	0.678	0.456109972
131	6	34	0.925	0.912790582
1814	8	25.5	0.546	0.002248646
115	9	15.9	1.199	0.218809651
39	11	95.3	1.021	0.514876356
3	2	266.8		
238	26	312.1	0.994	0.902014583
8	2	84.1	1.366	0.005669446
178	6	16.5	0.907	0.546353204
433	15	74.6	0.94	0.661834388
13	2	43.4	1.003	0.929620641
250	9	15.8	0.835	0.226772547
17	1	20.1	1.399	0.018489156
2	1	16.6		
1	1	47.3		
13	1	152.8	0.835	0.350931258
8	3	12.5	1.167	0.721970236
62	9	15.2	0.312	0.052839227
2	1	36.7	4.08	0.652754916
22	4	82.2	0.835	0.654134177
29	2	24.6	0.591	0.177286265
75	4	34	0.961	0.893868735
208	17	88.8	0.883	0.234517755
4	1	38.8	1.204	0.162429875
76	5	45.3	1.204	0.477128155
362	6	28.3	0.639	0.688981983
385	8	27.7	0.95	0.927089996
2245	34	36.1	1.32	0.034115466
84	6	37.5	1.019	0.892132166
7	2	47.1	0.833	0.827491284
212	7	16.8	1.042	0.523627303
138	8	68.5	0.8	0.038540018
300	10	65.1	1.003	0.944246562
16	3	13.1	1.335	0.129028693
133	2	14.5	1.35	0.36876186
25786	297	515.3	1.156	0.249949696
6	3	48	1.15	0.439200636
446	16	52.3	1.161	0.187159483
36	3	38.1	0.918	0.588837999
43	5	51.7	0.966	0.647023325
58	3	16.5	0.541	0.004237408
2	2	54.4	0.326	0.041446214
8	3	30.8	0.19	0.247206709
42	2	39.7	0.811	0.300432016
4	1	37.6	0.99	0.928051666
2	2	74.8	1.562	0.008864819
46	2	37.2	0.585	0.042766383
2	1	201.9	1000	0.001
81	12	136.3	1.358	0.15237529
19	2	138.5	0.926	0.412345634

46	3	28.3	1.603	0.039345892
5	4	32.8	1.393	0.253104219
45	4	52.7	0.815	0.259208861
12	4	75.8	1.823	0.013788991
48	3	51.2	1.105	0.768028338
8	3	37.5	1.148	0.245176613
449	27	59.7	0.911	0.610020313
7841	80	122.1	0.823	0.017175512
3	1	57.2	1000	0.001
55	5	80.8	0.838	0.349229509
147	8	178.1	1.16	0.201389688
83	7	134.2	0.929	0.907144209
1008	30	55.9	0.774	0.080768482
228	9	23.5	0.716	0.019338209
1404	28	54.5	1.04	0.516293079
1	1	34.3	0.87	0.785644372
38	1	52.7	0.94	0.557773897
43	4	51.9	0.689	0.147847143
10	1	102.8	1.395	0.227747368
24	12	141.5	0.529	0.412394195
69	3	15.6	0.738	0.081743704
3764	65	85.6	0.967	0.830551319
2	1	140.9		
932	9	32.9	1.273	0.15872779
43	6	85.3	0.708	0.760457652
1	1	76.7		
16722	151	69.3	0.808	0.116411882
29	1	90.3	1.149	0.205446677
59	9	206.1	2.089	0.047517029
1084	8	9.3	1.072	0.854596605
38	2	35.2	1.058	0.554963153
146	8	28.8	1.283	0.068589117
46	3	28.5	0.842	0.175239618
2	1	112.8		
121	3	56.1	3.296	0.6066718
5	4	37.5	1.975	0.642040207
107	7	16.1	1.183	0.087649014
58	7	124.5	0.985	0.980799737
40	1	50.3	0.987	0.813620155
6	2	43.2	1.099	0.312457384
35	6	46.6	1.282	0.119069994
22	2	8.5	0.869	0.723208764
843	13	51.5	0.366	0.032549112
8	3	31.5	0.71	0.390825077
71	8	37	1.109	0.302197473
12	3	32.6	0.958	0.844275074
835	14	57.9	1.239	0.067970723
4	1	26.8		
41	5	24.7	0.93	0.767191719
5	3	77	1.692	0.467086115

6308	43	39.3	1.041	0.567154644
28	1	375.8	0.801	0.186072467
686	18	70.6	0.845	0.22282922
3	1	134.1		
9	2	51.5	1.26	0.450484434
142	14	138	0.918	0.543450884
543	21	65.3	0.769	0.0217296
11	3	195.9	1.274	0.148707142
675	48	504.3	0.536	0.144665636
15	2	12.3	0.589	0.077453442
8	1	26.4	0.966	0.911136458
5	1	104	0.985	0.809706577
50	5	91.8	1.24	0.109306464
218	19	106.8	1.108	0.191047368
17	3	37.7	1.184	0.179667729
30	4	36.8	0.909	0.401388424
4	1	25.2	0.774	0.487146865
7	2	70.9	0.944	0.602930453
1	1	46.8	0.001	0.001
54	1	12.4	1.037	0.821463591
10	1	13	0.95	0.918730873
5	2	27.3	0.448	0.370985987
41	3	33.3	1.507	0.119370368
32	6	129.2	0.958	0.62692989
34	2	44.9	0.799	0.15235091
158	13	197.9	0.807	0.130745929
73	7	40.2	0.881	0.425059346
122	8	72.9	1.036	0.619508768
25	1	21.5	1.021	0.868195474
2	2	113.7		
35	1	12.5	0.844	0.24473186
26	3	19	0.92	0.905026762
10	1	85	1.109	
33	3	38.3	1.426	0.436203558
37	3	33.8	1.09	0.474211066
217	5	16	0.204	0.9369395
6	1	10.4	5.065	0.042889208
8	1	1.4	1.383	0.692728564
342	8	49.5	1.141	0.167580239
258	11	20.6	1.213	0.305834539
839	8	13.9	0.92	0.670257934
22	3	77.4	1.155	0.682370268
88	4	12.8	0.878	0.149300218
31	2	47	1.214	0.399019472
2	1	25.6	1.134	0.994686971
89	5	46	1.099	0.786243011
83	1	12.6	0.906	0.742267969
55	4	12	0.851	0.495888274
27	3	12.5	0.731	0.6289295
52	5	110.4	0.394	0.023749564

39	6	247.9	0.34	0.030823108
157	9	53.7	0.811	0.199455796
11	2	36	0.707	0.195040075
23	3	13.9	1.148	0.867869054
8	3	63.4	1.131	0.305841369
616	21	67.8	0.949	0.83513717
70	8	63.5	0.931	0.647021949
367	15	16	0.655	0.101526231
122	1	19.9	1.177	0.922818209
49	5	22.3	1.077	0.767347413
908	29	65.4	0.931	0.950687594
10	2	130.8	1.054	0.576380287
85	8	97.1	0.978	0.763588589
2	1	34.4	1.518	0.308814624
62	1	12.5	2.732	0.302097646
79	6	53.4	0.662	0.346218291
306	17	15.2	0.277	0.960071909
2254	32	59.5	0.902	0.375270738
16	1	21.1	0.836	0.182808658
81	5	30.6	1.017	0.758944373
1	1	35	1.133	0.479999896
4	1	18.3	1000	0.001
28	3	76.7	1.003	0.937853767
84	1	12.9	0.329	0.136017282
134	2	113.9	0.576	0.025300355
327	20	135	0.821	0.222732078
7	5	162.1	1.874	0.068968922
3	2	41.9	1000	0.001
1	1	40.9		
628	18	49.4	0.794	0.271171686
20	1	38.4	1.19	0.136640138
19	2	48.1	0.001	0.001
14	1	55.6	1000	0.001
89	5	32.2	1.13	0.272767731
9	3	76.3	0.768	0.012688867
208	3	44.9	0.985	0.978759569
17	2	12.2	0.338	0.076724779
19	2	28.6	0.997	0.488072514
1	1	35.6		
690	7	77.2	0.962	0.896085974
7867	80	272.2	1.197	0.55140847
5	2	106.2	0.611	0.143864962
29	3	20	0.809	0.457394668
13	1	195.8	0.829	0.440855891
2	2	139.9	0.792	0.113596635
12	1	12.8	0.618	0.098491225
33	4	12.3	0.703	0.197005731
438	10	42	1.102	0.321105915
3	1	168.5		
37	5	40.4	1.584	0.010465701

52	1	12.8	0.88	0.543313273
288	9	26.1	1.112	0.965795851
13	4	26.1	0.674	0.420272755
309	14	27.5	1.072	0.347367137
5	3	26.8	3.424	0.983390042
222	1	11.3	1.004	0.739463612
137	9	46.1	1.442	0.007542768
1	1	47.5	0.989	0.453578276
21	3	19.2	0.122	0.05069634
16	2	164.8	1.404	0.003636522
4	2	35	0.624	0.164109767
9	3	54	1.618	0.688048784
7	1	15.3		
52	3	47.4	0.533	0.941258794
305	12	75.7	1.143	0.210773508
84	3	70.5	1.036	0.570832657
3	1	30.7	1.689	0.00428809
29	2	51.9	0.764	0.809273313
332	12	31.7	0.995	0.993697721
2326	56	66	0.476	0.019515599
230	1	64.4	0.405	0.012295221
34	1	50.4	1.563	0.454405052
22	2	16.4	1.488	0.118490799
6	1	12.8	1000	0.001
75	7	42.9	1	0.678724343
8	3	20.6	1.91	0.088500786
22	3	14	0.916	0.879843101
1448	9	36.1	1.058	0.691219326
1	1	45.4		
920	27	62.3	0.566	0.056506204
139	14	126.5	1.178	0.071001484
3	1	10.9	1.109	0.922814341
2713	44	101.3	0.95	0.639400378
128	4	53.5	8.739	0.095006447
179	9	57.8	0.846	0.196793569
2	2	49.2	0.902	
2	1	45.2		
15	4	51.1	0.589	0.0518916
17	2	13.3	0.469	0.007387221
99	3	38.2	1.087	0.451135953
113	6	46.2	6.192	0.062208814
44	5	33.9	0.951	0.91312743
186	12	73.8	1.013	0.617285494
5	2	222.9	1.119	0.321338003
40	1	41.4	1.239	0.742132409
68	5	56.9	1.069	0.70384084
23	2	58.7	1.044	0.715998842
41	2	30	1.172	0.258281094
8	1	75.2	0.702	
8	1	68.1	1.007	0.770771824

185	11	22.8	1.076	0.735398457
276	11	84.6	1.206	0.079087512
54	3	25.3	0.728	0.018811092
3	2	12.4	1.277	0.65308285
18	5	120.6	1.146	0.26092735
7	2	38.5	1.25	0.517428516
101	1	13	0.67	0.203706317
1892	57	62	0.357	0.025852738
8	2	46.3	1.053	0.914262869
115	11	55.8	2.656	0.524836955
101	4	19	1.08	0.702502064
25	3	12.6	1.169	0.554228002
50	3	43.7	0.89	0.329027947
67	7	63.9	1.703	0.001334236
3	1	32.2	0.71	0.253870338
4	2	86.1	0.94	0.56295815
55	8	243.5	0.819	0.054853059
66	7	79.4	1.695	0.085003711
46	5	58	1.269	0.121026116
324	11	47.9	0.702	0.018798667
300	2	49.2	0.001	0.001
379	13	37.2	1.241	0.081816979
30	2	14.6	1.027	0.443727055
78	4	43.9	0.776	0.284325442
1	1	59.2	0.433	0.096815136
28	3	34.3	0.785	0.092319106
20880	84	51.6	0.883	0.118421311
970	27	80.3	0.832	0.257929276
62	3	13.4	0.992	0.962537114
2	1	194.9	0.001	0.001
64	6	46.6	1.143	0.294391145
1	1	113.9		
2	1	69.9	0.929	0.600095323
8	6	128.7	0.61	0.085995749
6	2	35.9	1.253	0.656726987
144	11	59.6	0.885	0.778742743
99	8	48.1	1.117	0.447419688
2	2	36.8	3.444	0.274454831
77	3	12.8	0.415	0.116362722
2	2	80.3	0.376	0.129648609
27	2	35.9	0.882	0.280122395
506	6	79.2	1.104	0.328375308
21	3	33.7	1.152	0.470845225
68	2	19.8	0.804	0.657726406
19	3	46.6	1.095	0.596432517
150	6	35.2	0.689	0.029078341
293	13	71.6	0.771	0.010235806
103	3	59.5	0.982	0.877228113
27	4	29.1	0.712	0.111526795
415	14	45.6	0.617	0.141973227

168	2	61.9	0.001	0.001
108	6	22.2	0.887	0.468127384
78	1	13.3	0.904	0.772359197
2	1	25.7	0.391	0.377593297
99	8	38.8	0.89	0.397099683
154	5	47.2	0.745	0.122873096
11	2	10.5	1000	0.001
29	3	155.4	1.189	0.39919993
21	1	18.8	0.928	0.912519749
17	7	223	1.207	0.212026928
14	1	27.8	1.006	0.803008992
171	2	93.3	0.961	0.616091033
6	1	31.8	1.126	
73	2	18	0.948	0.530694279
2	2	24.2		
4	2	22.9	1.084	0.574395959
107	7	24.6	1.156	0.252688194
1	1	613	0.232	0.023357014
25	3	50.9	1.417	0.063033686
25	3	30.2	1.071	0.5329114
278	4	12.9	0.977	0.915167627
30	1	21.9		
11	3	57.1	0.814	0.766009608
66	2	13.1	0.001	0.001
1	1	25.7	0.595	0.639045655
202	16	102.4	0.828	0.724502609
11	1	105	0.97	0.961575817
19	8	50.5	0.716	0.028273868
34	1	57.8	1.43	0.118306017
1	1	65.7	0.798	0.401448609
11	1	11	0.865	0.359405237
6	1	43.4	1.05	0.887297029
132	8	22.1	0.915	0.952655474
194	8	60.9	1.093	0.420444287
25	1	13.1	1.609	0.789544162
296	28	274.2	1.286	0.003769672
29	4	33.7	1.818	0.044690265
25	4	37.9	0.962	0.802841189
63	4	53.9	1.263	0.196142647
8	1	15.4	1.206	0.747478589
1100	16	48.5	0.95	0.878760504
14	4	11.4	0.801	0.543781822
145	12	69.2	1.277	0.061136959
12	3	53	0.655	0.560602702
174	12	52.4	0.97	0.612729587
1	1	69.2		
1	1	48.8	0.97	0.826747082
1	1	39.2		
21	4	36.1	1.45	0.080497794
705	4	60	0.37	0.031881969

8	1	61	0.887	0.546554247
19	2	26.3	1.322	0.258995294
128	6	21.6	0.953	0.618018205
7005	2	103.3	0.767	0.008052779
139	9	26.5	1.205	0.030345185
9	2	20.1	0.689	0.288130467
98	6	55	1.131	0.386587272
33	5	26.1	1.005	0.99740469
696	24	83.2	0.934	0.828231435
7	1	110.4	0.973	0.559849716
4	1	25.4	1.07	0.711324539
2102	21	57	1.208	0.170078896
14	1	43.1	0.883	0.6453522
52	6	147.4	1.064	0.488354046
18	4	60.7	0.977	0.944735017
40	1	48		
60	6	100.8	0.887	0.807848797
89	9	20.7	0.537	0.098054585
26	1	12.3	0.813	
18	1	37.8	1.386	0.09072358
6	3	177.1	1.258	0.09164895
18	1	43.9	0.981	0.701462681
793	32	75.9	1.321	0.147446656
50	6	123.2	1.357	0.106122251
12	1	25	0.62	0.049134436
25	2	18	1.173	0.269767538
360	13	105.8	0.948	0.74434303
1455	47	129.3	1.173	0.192085411
14	4	20.9	1.225	0.272531911
10	3	46.5	1.389	0.214313899
29	4	57.5	1.101	0.249159975
2658	70	123.7	1.188	0.088280775
3	2	89.7	0.519	0.368688199
74	7	76.6	0.487	0.064660828
80	7	53.6	1.144	0.417376312
1	1	41.9	0.856	0.30181547
15	2	102.3	0.542	0.335799606
3606	103	269.6	1.224	0.17374947
32	1	32.7	1.008	0.586681996
17	2	51.8	1.412	0.808315015
2	1	27.2	0.001	0.001
9	1	101.1	0.989	0.896908628
61	4	12.8	0.802	0.786563982
1	1	78.8		
163	13	59.6	0.947	0.933972951
510	10	22.5	0.812	0.103638222
32	7	3813.7	1.001	0.910268548
5	2	24.3	1.074	0.940504223
435	18	26.7	1.026	0.635584563
1446	86	280.6	0.797	0.525662144

12	2	126.5	0.618	0.33798151
28	1	11.8	1.068	0.690276154
1723	26	99.8	0.813	0.074661137
2	2	17.1	1000	0.001
82	9	59.1	1.494	0.07536659
79	1	105.3	0.825	0.389661308
213	8	118.6	0.818	0.08706279
22	4	45.9	0.985	0.443164333
3	3	26.2		
7	2	117.9	0.571	0.035479353
136	8	49.9	1.283	0.012102368
1	1	13		
98	6	22.2	1.14	0.419351154
37	3	17.1	0.986	0.632627182
66	5	41.3	1.078	0.461574312
15	2	14.4	1.152	0.102818294
28	2	39.1	0.939	0.654447302
4	2	16.7	1.18	0.615957399
50	6	57.9	1.145	0.502028843
5284	41	70	0.956	0.684075097
4	3	35.7		
50	6	57	1.533	0.073785246
2	1	39.6		
13	3	76	1.267	0.535734659
418	3	49.8	1.312	0.752316399
19	2	48.2	0.582	0.029312539
3422	30	34.2	0.891	0.164349059
5	1	28.1	1.115	0.422916186
101	6	17.9	1.103	0.353794803
254	20	101.3	1.17	0.102899852
32	3	47.6	0.958	0.958877606
18	2	41.9	0.891	0.26133101
26	1	38.2	0.743	0.079253259
5465	33	54.3	0.897	0.18592396
18	1	491.6	0.626	0.630498541
23	7	141.2	1.385	0.260729664
235	20	117.8	1.164	0.237662432
14	5	133.9	1.184	0.164825846
9	1	91.9	0.557	0.112931974
152	8	97.1	1.083	0.208924505
78	7	23.5	0.791	0.429298758
52	3	106.4	1.221	0.099058144
14	3	21.2	0.824	0.290411708
11	2	15.1	0.527	0.47247212
249	22	186.7	0.881	0.220315063
26	2	7.6	0.912	0.698081212
22	2	37.9	0.752	0.289237408
8	1	39.7	1.206	0.450069758
27	2	35.4	0.804	0.089114755
61	9	19.9	0.876	0.441991962

8	3	25.6	1.007	0.919317961
166	7	54.7	1.326	0.067748005
30	6	68	1.149	0.50341054
58	6	47	1.305	0.011499862
3	2	16.6		
13	4	53.3	0.646	0.628770837
143	8	60.2	1.427	0.022412466
36	2	11.8	1.065	0.406372686
1553	21	38.4	0.893	0.159748763
299	10	44.7	0.742	0.427221587
175	15	99.9	1.22	0.13786671
15	2	30.9	0.775	0.536053741
265	14	56.6	1.111	0.548736632
150	9	21	0.924	0.930668509
4	1	213.5	0.731	0.276646527
46	6	24.6	1.187	0.304015021
30	2	28.4	0.988	0.865049251
89	9	26.5	1.143	0.160835956
28	3	18.2	1.326	0.029142597
44	1	33.4	1.222	0.076280786
20	6	27.7	0.625	0.108570557
56	1	19.8	0.902	0.634969047
128	6	22.6	0.976	0.877287006
2	1	39.9	1.055	0.88782364
12	1	65.4	0.777	0.586352432
88	2	12.6	1.058	0.570089878
256	16	131.1	0.939	0.181096551
62	3	110.4	1.016	0.93536971
6	5	22.6	1.086	0.602344276
89	3	44.3	1.182	0.063951018
2	1	9.1	0.724	0.051150793
2	1	33.1		
1	1	67.2	0.567	0.001472776
29	1	32.5	0.78	0.305817479
190	16	56.9	1.263	0.151561758
57	5	52.9	1.046	0.556305631
1582	94	309.1	0.839	0.372972337
44	4	18.1	1.299	0.165848066
33	5	112.3	1.084	0.576313688
1	1	13.9		
47	3	32.2	1.152	0.386221487
41	4	75.6	0.875	0.534877977
2	2	39.5	1.064	0.696259507
454	2	40.2	1	0.724456592
1	1	6.5	0.001	0.001
21	1	19.2	1.124	0.173496774
4	2	73.7	1.025	0.679240385
37	3	21.9	0.741	0.29998389
63	8	29	0.931	0.655870118
30	1	19.3	1.036	0.935754861

2	1	14.9	0.99	0.939840298
58	4	27.1	0.935	0.78883429
32	6	79.3	1.206	0.752675424
1	1	36.1		
2	2	23.7	0.79	0.817841164
75	8	43.8	1.174	0.126812315
24	1	29.8	1.079	0.484882992
43	5	31.1	1.1	0.435759942
29	3	65.1	1.158	0.253009419
29	1	76.8	0.879	0.621128962
2	1	11.6		
42	2	20.8	1.263	0.153806554
40	8	78	0.733	0.742978293
148	1	75.8	0.918	0.996225143
18	2	204.4	1.081	0.445658147
3	2	66.7	1.066	0.675113052
21	4	227.6	1.177	0.075365654
15633	1	192.8	0.761	0.003660979
1	1	30.3		
3	1	62.5	1.14	0.536519165
3	1	27	1.278	0.054944273
51	3	20.9	0.925	0.676437866
2	1	136.5	0.997	0.96427234
7	2	19.7	1.229	0.29969501
119	10	108.3	0.962	0.612808226
84	8	86.1	0.786	0.00748125
29	3	116.2	1.183	0.530326008
2259	1	19.9	0.542	0.186631909
279	21	63.9	0.851	0.13469587
504	22	75.9	0.983	0.959143366
3	1	21.5		
30	9	176.7	0.97	0.841146635
9	3	101.5	1.183	0.142116231
406	3	28.7	1.027	0.745372541
46	3	41.3	1.399	0.025145394
29	3	41.6	0.931	0.866451243
33	1	44.8	0.874	0.488853163
1101	35	50.3	1.068	0.39724955
33	3	29.1	0.505	0.009441224
66	3	38.4	0.801	0.374727875
77	1	13	1.368	0.06391739
17	2	77.1	1.049	0.558383491
455	4	57.7	1.573	0.025497877
19	3	140	0.843	0.70153198
13	2	336.1	0.546	0.018916536
20	1	22	0.723	0.238274892
3	1	64.6	0.973	0.94279633
5	1	83.4	0.954	0.674087898
58	1	26.2	0.921	0.863373907
135	7	42.9	0.863	0.59643641

55	1	21.2	1.048	0.65067976
2	1	2.6	0.001	0.001
1	1	15.2		
2	1	44.9	1.572	0.571096492
2156	23	51.8	0.98	0.797693649
17	2	88.3	0.799	0.702936377
39	7	52.3	0.916	0.732475956
49	4	70.6	0.983	0.935196043
43	4	36.7	0.651	0.281766195
15	1	162.4	1.133	0.297374831
112	8	122.2	1.587	0.192281509
2	1	39.6	2.733	0.482273973
377	10	50.6	1.083	0.331405396
281	12	50.6	0.705	0.03956747
1	1	44		
103	3	23.2	1.371	0.048820598
130	11	23	0.807	0.679169528
10	2	66.3	1.393	0.005069864
72	8	37.4	1.259	0.459196395

M/N Effect_size	M/N Power	A/N Ratio	A/N P value	A/N Effect_size
		0.758	0.273881298	
1.025510749	0.583130227	1.779	0.004720036	3.243095157
		0.758	0.412445802	
		0.29		
-0.737165066	0.345061529	1.331	0.446617653	0.770112488
		1.299	0.149683955	
		0.331	0.064444105	
-1.036115538	0.591844351	0.949	0.743462262	-0.335634578
-1.449302761	0.865148643	1.26	0.342103206	0.970174191
1.37426634	0.827822667	1.002	0.898903185	0.13029723
-1.400194201	0.841404953	0.99	0.884739152	-0.146121804
		0.626	0.179673849	
0.977218759	0.542989683	1.074	0.606024303	
-1.808336871	0.968541358	0.577	0.051908816	-2.079373932
-1.217418318	0.730674299	1.331	0.772011843	0.289455193
		0.45	0.682114854	
		1.045	0.808863204	
0.125580466	0.058153484	1.004	0.895325766	0.134432821
-0.371730529	0.123523219	1.108	0.313082658	1.065991231
		3.624	0.229458318	
-1.780369542	0.964142665	0.922	0.655406153	-0.451782189
-1.160847726	0.689880911	0.888	0.299610371	-1.057707189
-0.357385625	0.117826739	1.063	0.619742069	0.519355472
		0.915	0.732246023	
-0.766044824	0.367766159	0.753	0.047529026	-2.108366452
		5.116	0.893549035	
0.895415227	0.474196003	0.951	0.509961168	-0.666237725
		1000	0.001	
0.797926469	0.39338526	0.58	0.043110476	
0.129835943	0.058718198	0.915	0.383698761	-0.86394015
		0.902	0.943126048	
		1.077	0.916530577	
		0.95	0.78755441	
		3.549	0.023505696	
		0.001	0.001	
2.774351999	0.999949529	1.38	0.070508168	1.960994969
-1.044302889	0.598541153	0.817	0.34910397	-0.964593183
		0.736	0.568908447	
0.83006537	0.419693747	0.519	0.012709919	-2.991871928
		0.364	0.754163226	
-0.25500595	0.084067073	0.678	0.195909117	-1.351146374
		0.25	0.001894924	
-0.711313005	0.32520625	0.81	0.152888772	-1.417230317
		0.884	0.582471501	
		0.67	0.095382972	
		1.528	0.396916072	

		1000	0.001	
		1000	0.001	
-2.470365862	0.999424688	0.797	0.161923053	-1.431002736
		1.234	0.113318847	
		1.589	0.103854304	
		1.911	0.015767386	
		1000	0.001	
		1.846	0.859790949	
2.060600805	0.991582474	0.862	0.190472223	-1.343269559
1.267454517	0.76435667	0.702	0.038133643	
-0.335921318	0.109749899	0.952	0.806296703	-0.251064831
-0.791502405	0.388181126	0.931	0.656805528	-0.45587119
-0.067165014	0.052324482	0.654	0.020137125	-2.496710825
-1.206583316	0.723074116	0.612	0.047980171	-2.161551002
		0.892		
		0.584		
-0.622910175	0.261329945	0.93	0.635702837	-0.479161286
-1.20619537	0.722800051	0.925	0.778146634	0.297585918
-0.481334593	0.174956376	0.85	0.963496368	-0.047699224
0.740241195	0.347454761	1.29	0.084646708	1.868561083
0.227133738	0.076936067	0.611	0.099787786	-1.711627867
0.672721305	0.296502922	0.954	0.773183219	-0.290981022
		1.378		
		0.716	0.533316825	
		1.374	0.334587502	
1.417102549	0.849872429	1.159	0.802593183	0.248870859
2.097399423	0.993199491	1.365	0.221814322	1.270193072
		1.165	0.654086108	
		0.001	0.001	
2.201549897	0.996389374	0.817	0.317244166	-1.021552379
		1.079	0.981409794	
		0.586	0.042639874	
		0.666	0.199672591	
		2.175	0.018429684	
-1.290125267	0.778814609	0.652	0.056520421	-2.118401019
-1.445663802	0.863477874	1.561	0.446909835	-0.754449151
-0.542760957	0.209793632	0.431	0.735646456	-0.345634641
-0.283963739	0.092400211	0.995	0.802846155	0.258824908
-0.002617631	0.050003526	0.555	0.437649034	-0.82019604
		0.579	0.386511024	
		0.397	0.216965172	
		0.866	0.282876456	
		1.271	0.877831859	
-0.173332336	0.065596487	0.955	0.306270962	1.068917995
-0.742609161	0.349301283	0.897	0.415246237	-0.831108458
		0.484	0.304879373	

		1.231	0.090416734	
		1.244	0.109999313	
		2.176	0.068402612	
0.528246178	0.201183737	0.874	0.149355362	-1.522199746
		0.286		
-2.047411436	0.990925568	0.906	0.423732337	-0.812647034
-0.712398337	0.326030197	1.254	0.23142742	1.237392781
0.117515287	0.05713572	0.646	0.033272371	-2.273425603
0.791669576	0.388316298	0.975	0.950969043	-0.06305115
		1.077	0.651678711	
		1.003	0.900386415	
0.470101351	0.16904552	1.005	0.678018582	0.439756294
1.460722264	0.87030023	0.927	0.477662363	-0.73865704
		0.886	0.873545276	
-1.982676614	0.987008796	0.733	0.064550132	-1.970581377
		1.131	0.986472413	
1.832079763	0.971916085	1.104	0.37586664	0.920857039
		0.855	0.560231528	
-1.83586197	0.972424747	0.879	0.109947894	-1.639928349
		1.461	0.079651789	
		0.43	0.051166146	
-1.508502269	0.890367448	1.149	0.507016239	0.676977103
1.791737368	0.965987927	1.186	0.171863396	1.465129041
		0.693	0.186059997	
-0.253456559	0.083647865	0.853	0.300788089	-1.083154118
		1.187	0.570470817	
-1.755284175	0.959779526	0.905	0.384993799	-0.887226441
		1.219	0.915536742	
		0.732	0.270490007	
		0.845	0.464977813	
		0.71	0.176944879	
1.127222087	0.664422825	1.327	0.011834135	2.857548646
-1.222317618	0.734076122	1.437	0.476172888	0.739403989
-1.725229219	0.953993711	0.693	0.060582598	-1.975482469
1.02642395	0.583882304	0.877	0.735069055	-0.346323238
0.874131478	0.456340392	1.388	0.075201561	1.957839483
-1.066125715	0.616244433	0.631	0.09901386	-1.792193849
		1.11	0.387391867	
		0.711	0.663829542	
-1.466424271	0.87282076	0.889	0.411080729	-0.831330277
		1.132	0.908921147	
0.670378624	0.294799491	1.133	0.405502176	0.838234468
0.856338669	0.441479862	0.998	0.951936722	-0.060630394
1.141468157	0.675310079	1.158	0.268455636	1.172981851
-0.061210315	0.051930137	1.29	0.13680102	1.615059909
1.460135513	0.870038908	1.552	0.061961511	2.084406489
-1.580389289	0.916193308	1.375	0.038921193	2.202427026

		1.213	0.699518988	
0.304446547	0.09886981	0.794	0.8033698	
		0.664	0.029745057	
1.28253124	0.774029166	0.503	0.157592625	-1.470763716
		1000	0.001	
		0.808	0.527967285	
-2.209988043	0.996576448	1.168	0.310932477	1.028141596
2.218923754	0.996765009	1.482	0.019104741	2.518989023
		1000	0.001	
1.585185364	0.917737741	1.143	0.288399914	1.101404167
		0.864	0.791737907	
0.135283368	0.059469085	0.56	0.010801181	-3.095966
		1.904	0.273601114	
0.256328415	0.084427017	1.073	0.601439971	0.529601707
0.717484167	0.329902719	1.001	0.98195323	0.02300395
		1.156	0.37552202	
		1.057	0.70608616	
1.26196423	0.760778304	1.473	0.123677409	
-0.084478917	0.05368029	1.385	0.069579058	1.940747003
		1000	0.001	
		0.675	0.291584287	
1.613254638	0.92634908	0.835	0.243881769	-1.173568426
-0.497932741	0.183953276	0.874	0.229792492	-1.230420611
-0.730537047	0.339926471	0.917	0.752634063	-0.333801027
		0.86	0.595520812	
0.922434491	0.496934032	0.904	0.191253509	-1.339372163
2.034762286	0.990253792	1.233	0.140828581	1.544948731
		1.181	0.800741048	
-0.313099125	0.101747084	0.961	0.873227752	-0.164622499
		1.049	0.569979126	0.592175955
-1.076579414	0.624641627	0.838	0.849463514	-0.200064882
-0.442604187	0.155188857	0.879	0.302807789	-1.039778993
2.305403475	0.99816127	1.525	0.025904662	2.519146537
0.2565602	0.084490305	0.888	0.561969017	-0.588682511
		0.702	0.084215733	
0.246392733	0.081770772	0.654	0.080113272	-1.89539176
-1.07291675	0.62170602	0.919	0.64394341	-0.468237713
		1.223	0.961509979	
1.481194027	0.879190214	1.134	0.069856203	1.966992508
		1.074	0.878065618	
1.114704025	0.654740003	1.25	0.172947159	1.494770878
		1.091	0.865259969	
		0.79	0.348533987	-0.973506481
-1.355010964	0.817268411	0.674	0.049769591	-2.14574013
		0.631	0.016025059	
2.872298791	0.999978759	1.646	0.004039913	3.414952782
		1.351	0.041851292	
1.707750132	0.950332184	1.363	0.207919253	1.310106178
0.912743744	0.488773775	1.227	0.065793415	2.009684263
1.474040354	0.876133841	1.373	0.072539334	1.872658816

2.372646457	0.998839517	2.525	0.015570436	2.763138737
		1.335	0.18469589	
1.697426614	0.948062529	1.119	0.345763118	0.983181429
		2.038	0.097077271	
		0.812	0.551212298	
-1.908048635	0.980752189	0.758	0.163927856	-1.438578316
0.470818697	0.169418665	1.417	0.017390242	2.770970251
-0.773701843	0.37386968	0.823	0.473636905	-0.724761058
		0.175	0.037605426	
-0.608107453	0.251322291	1.067	0.476019936	0.74411491
0.169310608	0.064875415	1.026	0.65413725	0.471137329
		1.016	0.926307383	
-0.293874587	0.095470765	1.06	0.551927247	0.620516064
		0.001	0.001	
-0.152321316	0.062021325	0.955	0.503534274	-0.673003055
0.333655095	0.10892832	1.298	0.076841707	1.932236117
0.509102622	0.190183	2.016	0.015084999	2.742038106
		1.5	0.336547087	
		1.002	0.874602592	
		1.166	0.955707064	
0.32638653	0.106333312	0.862	0.528461565	-0.634931279
0.711958274	0.32569601	0.845	0.623854443	-0.505068276
0.97087967	0.537678423	1.046	0.477971782	0.762869741
-2.656709761	0.999864583	0.909	0.564951843	-0.584084509
0.294939923	0.095807495	1.38	0.035511411	2.343322546
0.265420463	0.086954934	1.05	0.60451942	
		0.933	0.788936636	
0.08051864	0.053342663	1.078	0.399163394	0.848090769
		0.842	0.710721155	
1.761828352	0.960957409	1.393	0.078147673	1.906735033
		0.35	0.844589343	
-1.242205751	0.747657539	0.917	0.763416074	-0.314598477
		1.651		
		1000	0.001	
0.324329402	0.105609962	1.135	0.858382642	0.178088224
		0.942	0.588937859	
-1.608922922	0.925067135	0.68	0.042588367	-2.276120429
		0.913	0.414645839	
		1.124	0.936973533	
-2.167325848	0.99553296	0.973	0.68204879	-0.407337597
2.018499736	0.989326499	1.717	0.015860429	2.790302004
0.881372072	0.462406627	0.81	0.208825121	-1.27830575
-0.105270378	0.055721512	0.972	0.619513574	-0.488553652
0.843381949	0.430709319	1.569	0.01327835	2.71170033
		2.434	0.031149635	
		1.031	0.422955329	
-0.78488789	0.382843924	0.892	0.364687215	-0.955738191
		0.839	0.399861498	

-1.359702633	0.819876592	0.919	0.447991366	-0.770834072
		0.808	0.239027637	
-0.121664808	0.057650781	0.68	0.040928108	-2.154733526
-2.326042026	0.998400372	0.727	0.027904403	-2.4592373
		1.613	0.045096803	
-0.377551056	0.125902981	0.72	0.237005674	
-0.372080476	0.123665181	0.642	0.007209884	-2.986773942
0.967727101	0.535034432	1.421	0.392702477	0.86952285
		0.801	0.758622825	
-1.16639507	0.693998575	1.085	0.709785048	0.372114612
-2.450629765	0.999335009	0.477	0.069459155	-1.928839629
		1.256	0.829891327	0.217113867
		1.02	0.500109604	
0.825660885	0.416063843	1.291	0.074312209	1.935000422
		1000	0.001	
-0.803484353	0.397903383	0.648	0.405406159	-0.837037943
-0.333747851	0.108961831	1.04	0.579927947	0.576026594
		0.628	0.288928301	
1.647483667	0.935895739	0.431	0.065849541	-2.024878405
0.980584688	0.545806774	0.649	0.404022617	-0.880003316
0.019023595	0.05018624	1.097	0.376312274	0.929082005
		1.542	0.469473944	0.73056349
		0.617	0.059014865	
1.334077264	0.80534533	1.713	0.034627511	2.351627932
		0.887	0.940649768	
		1.269	0.079571304	
		1.67	0.105433352	
		0.797	0.673645818	
0.3331683	0.108752617	1.061	0.877290494	0.153733682
		0.798	0.256282731	
-1.718592391	0.952629851	0.642	0.058300867	-2.088715141
0.137886179	0.059838981	1.019	0.869637401	-0.16162079
-0.139365575	0.060052432	0.995	0.948010114	0.066701419
0.842340004	0.429845328	2.355	0.09872077	1.666305096
		1.62	0.404751231	
		0.033	0.119625023	
		0.949	0.940501717	
		0.756	0.250813788	
		0.709	0.526505274	
0.545374043	0.211368018	0.71	0.023717503	-2.393260038
		0.91	0.868799857	
1.248197816	0.751676381	0.973	0.911246992	0.117123447
-1.342340302	0.81010726	0.635	0.170231524	-1.454839803
		1.057	0.709590746	
0.690691354	0.309721101	1.157	0.684603445	0.407512898
		1.193	0.937168868	
0.525737162	0.199718834	1.138	0.416814754	0.810930076
-1.040790073	0.595671303	1.018	0.782472454	0.284252754
-1.657416572	0.93847759	0.783	0.126523837	-1.590098817
		0.984	0.471958752	

		1.349	0.134084054	
-2.075358436	0.99226763	0.763	0.004886128	-3.185560982
0.332963194	0.108678668	1.876	0.142276709	1.470122209
1.785968427	0.965061506	1.479	0.08857051	1.872647585
-0.277993234	0.090604406	0.992	0.751872712	-0.312488923
1.72318882	0.953577815	1.331	0.080231511	1.90744704
		1.515	0.45287194	
		1.802	0.013789811	
0.009253733	0.050044064	1.1	0.765958093	0.297575549
		1.349	0.189304816	
		0.001	0.001	
1.443134572	0.862308298	1.059	0.676113075	0.42767072
0.240326131	0.080203243	0.951	0.755220118	0.331605459
0.913975174	0.489810532	1.085	0.44940962	0.788613026
		0.91	0.774328042	
		0.931	0.373524678	
-1.569477693	0.912598444	1.193	0.637131316	0.475571934
0.555332725	0.21743556	1.323	0.14956117	1.547999046
0.627105217	0.264204421	0.918	0.725870571	-0.365869442
		1.18	0.244551963	
0.218684117	0.074944903	0.923	0.596129814	-0.531870107
		0.904	0.747845887	
-1.019889458	0.578494114	0.75	0.125297635	-1.617970804
0.500412672	0.185324278	0.746	0.10309377	-1.734268055
1.443732639	0.862585475	1.804	0.007109592	3.007126829
0.632009186	0.267585773	1.01	0.921788994	
-0.908221121	0.484966827	1.263	0.850874874	0.185784004
0.730645787	0.340010476	1.157	0.133906647	1.62190625
		0.853	0.752167767	
-0.928723223	0.502230459	0.965	0.791502683	-0.269191897
-1.407818226	0.845261068	0.829	0.287564463	-1.113739632
1.027586633	0.584839395	1.16	0.237058545	1.232420983
0.9036387	0.481110949	1.149	0.294908579	1.098559456
		0.912	0.829934334	
1.899733962	0.979917596	1.072	0.810456637	0.246246907
-1.390745782	0.836539216	1.129	0.940726072	0.074760722
		1.127	0.844144476	-0.193460752
		2.871	1.80657E-05	
		0.131	0.007765491	
-0.616950674	0.257275386	1.211	0.073595538	1.933152478
-0.704436188	0.320006069	1.608	0.258588222	1.166255324
-2.433967414	0.99924943	0.718	0.016622281	-2.627183905
0.946951884	0.517577142	1.239	0.227076385	1.265426748
-1.978989675	0.986747193	0.86	0.442168303	-0.778607686
-0.670441404	0.29484508	0.964	0.935665144	-0.082946785
		1.5	0.394495031	
-0.995463802	0.558230033	0.455	0.041320775	-2.226728836
		1.6		

-2.147480016	0.994957034	0.706	0.243620848	-1.209093154
		0.329	0.062124948	
		1.307	0.961364672	-0.048276288
-0.5723994	0.228079228	1.088	0.621702286	0.493831768
		0.89	0.842676027	
-0.197452889	0.070288858	1.476	0.610955296	0.496650754
		5.075	0.080573298	
		0.936	0.85155932	
-1.414772799	0.848724001	0.94	0.505347833	-0.661169295
		0.464	0.21524116	
1.048552532	0.602005822	1.647	0.044301279	2.270033881
-0.422613348	0.145664506	1.037	0.797261818	0.26005316
0.718458768	0.330646963	1.168	0.175717289	1.448313692
		0.957	0.336289801	
-0.46470335	0.166256564	1.009	0.780840879	-0.271694267
-1.428388342	0.855353063	1.052	0.549889059	0.628521419
2.296397199	0.998047085	1.188	0.150348136	1.526534934
1.169757699	0.696482696	0.928	0.486223941	-0.694138127
-1.206552677	0.723052476	0.363	0.282592462	-1.148013256
0.86140821	0.445706412	0.798	0.171172122	-1.423632251
		1.284	0.404830812	0.844606896
1.757972509	0.960266849	3.343	0.090878675	1.78990631
		0.681	0.245663574	
-0.148861318	0.06147798	0.637	0.13326656	
-0.863961307	0.447837358	1.029	0.7190227	0.37213503
		0.939	0.930122515	
-2.119338461	0.994027014	0.685	0.830586257	-0.225160258
		1.732	0.603812518	
0.195455968	0.069876348	2.434	0.008743275	2.948154653
2.560312331	0.999708658	1.939	0.011055101	2.839518258
		1000	0.001	
		0.923	0.822452305	
		0.309	0.038093442	
-1.566583818	0.91162598	0.718	0.249988685	-1.179371072
		1.38	0.218247381	
0.399243013	0.135119999	0.702	0.007869291	-2.999299871
1.22658911	0.737024103	0.844	0.402975788	-0.870115196
		0.666	0.038553108	
0.361452334	0.119417328	1.366	0.083447009	1.894360905
		0.683	0.098849699	
		0.877	0.700833316	
-0.650540501	0.280564657	0.864	0.352245793	-0.96789026
		1.142	0.827163648	
		2.243	0.347913525	
2.323065274	0.998367733	1.219	0.467128039	0.72803252
-0.209804246	0.072937288	1.082	0.665542212	0.43156373
-1.712946865	0.951444338	1.006	0.934053494	0.084052227
0.001530614	0.050001206	1.25	0.433601784	0.78464365
-0.624919892	0.26270493	0.738	0.625765831	-0.501343933
-0.725913735	0.336362359	0.734	0.102322455	-1.649040254

3.16117242	0.999998689	0.83	0.067014735	-1.91388633
1.442438716	0.86198532	0.995	0.665036342	0.454654445
		0.887	0.936801233	
0.254991736	0.084063215	1.05	0.535959716	
1.742646408	0.957423118	1.453	0.055010304	2.142543317
		0.508	0.191497287	
1.483018221	0.879961011	1.412	0.118536861	1.729592196
		0.589	0.065576302	
0.995515498	0.558273104	0.702	0.007743904	-3.036500563
-1.547143856	0.904883147	1.121	0.195706447	1.356530145
0.964706543	0.53249969	0.782	0.75248304	
0.580851122	0.233463127	1.215	0.218371543	1.312228449
-0.015975617	0.050131338	0.803	0.192717375	-1.321769477
		0.809	0.720472319	
-0.144900773	0.060871722	0.991	0.902783405	0.126293741
-1.386929542	0.834546674	0.98	0.940160939	-0.078144803
-3.171918056	0.999998826	0.666	0.002027987	-3.661960982
		1.34	0.337507323	
-2.260317398	0.997522164	0.779	0.16596999	-1.384445635
		0.877	0.594057878	
-1.990925748	0.987577971	6.257	0.066467718	1.887897449
1.167495724	0.69481267	0.651	0.969416672	0.039383155
1.857368661	0.975173089	1.479	0.012214304	2.87999926
-0.200530268	0.070933093	0.86	0.210834315	-1.273578897
1.778816427	0.963884298	0.988	0.922653063	-0.09866284
2.254422987	0.997425185	1.452	0.269066066	
2.674421049	0.999882854	0.807	0.097236046	-1.809041909
0.115034613	0.056836476	1.501	0.181006573	1.398669259
0.971670752	0.538341628	0.563	0.041404007	-2.16811525
		0.749		
		1.245	0.132720474	
		0.525	0.28782797	
2.588994574	0.999767116	1.102	0.803317346	0.249539338
-0.60293393	0.247875153	0.853	0.713189016	0.388520589
		0.347	0.10325701	
		0.663	0.458298226	
1.84448356	0.973555561	1.254	0.111755412	1.698535761
		1.668	0.283078801	
		0.636	0.038443004	
1.450233238	0.86557359	1.284	0.124655769	1.609866315
0.050450111	0.051310701	1.079	0.650694119	
-3.36903868	0.999999858	0.876	0.209988966	-1.257583427
		1.042	0.707789228	
		0.001	0.001	
-0.777351813	0.376790589	1.058	0.843375214	-0.196801063
0.842383098	0.429881056	1.899	0.093572562	1.766600693
		0.568	0.046868198	
		1.124	0.881275166	

2.317983281	0.998310604	1.43	0.06873663	1.957963613
0.020470214	0.050215645	1.044	0.708837416	0.383048265
0.737353255	0.34520776	1.584	0.027633809	2.493573114
2.645083414	0.999851172	0.699	0.001667088	-3.618886766
		0.945	0.886104911	
1.856179747	0.975027403	0.988	0.911669693	0.114479315
0.151427541	0.061879745	1.009	0.963019479	0.0470917
		0.639	0.201349703	
		1000	0.001	
		1.229	0.859434232	
		1000	0.001	
		1.413	0.171578016	
-0.607762728	0.251091778	0.968	0.792483309	-0.267472246
		0.639	0.033255453	
		0.559	0.724216672	
-1.715682108	0.952021649	0.62	0.031347133	-2.357056481
		1.66	0.151389096	
2.50649226	0.999560509	1.037	0.617486469	0.514903291
-1.801599441	0.96752477	0.598	0.046449085	-2.166947405
-0.90976175	0.486263524	0.79	0.365831569	-0.931453485
-0.72009606	0.331898808	0.527	0.013540656	-2.618130396
1.995831256	0.987906088	1.43	0.028727579	2.320171065
		4.226	0.100148536	
		0.773	0.978218808	
0.48163521	0.17511654	1.068	0.477389425	0.744016375
1.356299314	0.817986971	1.231	0.033031447	2.362925572
		1.155	0.527843982	
-0.266922837	0.08738162	1.013	0.518721833	0.665027919
-1.375449724	0.828458333	1.123	0.923474646	0.09535261
1.865976241	0.976206695	1.306	0.032019169	2.338471181
1.280919158	0.773005799	1.621	0.009712511	3.041877693
-1.519079391	0.894490432	0.986	0.90830181	-0.117286885
-0.422826422	0.145763572	0.654	0.069872182	
0.179407327	0.066718826	1.762	0.096473756	1.832430523
1.769221663	0.962254142	1.381	0.260573781	1.166543703
		1.018	0.618693136	
-1.088577673	0.634206671	0.902	0.414785816	-0.831850589
0.026534272	0.050362367	0.898	0.817884971	0.242597672
		0.979	0.57348805	
2.937822181	0.999988357	2.467	0.000186541	4.879522099
		1.957	0.177787399	
-1.105773206	0.647768774	1.3	0.946167909	-0.069430249
0.469300907	0.168629846	1.189	0.141980702	1.563216085
0.143798103	0.060705908	1.111	0.668337598	0.426359243
		0.728	0.343439649	
1.991227913	0.987598403	1.166	0.182309886	1.4393402
-0.376572716	0.125500218	1.047	0.554751355	0.616551777

-4.060064067	1	0.676	0.049560465	-2.106116043
		0.73	0.165111745	
-0.817105723	0.409034146	0.933	0.771409193	-0.307968928
0.213342487	0.073726826	0.945	0.962523426	0.04936489
		0.3	0.562530167	
0.863684309	0.447606087	1.134	0.263840054	1.1726293
		1.238	0.614565169	
		0.951	0.693956287	
		0.736	0.561880684	
		0.557	0.5024226	
		1.225	0.43409407	
-0.733114992	0.341920199	0.779	0.396394557	-0.873897
0.464735672	0.166273164	0.819	0.158914968	-1.494551826
-0.420015019	0.144460703	1.155	0.402638504	0.852967534
		2.208	0.017734478	
0.201175465	0.071069475	1.063	0.658014064	0.454556672
0.727417404	0.33751991	1.023	0.781067242	-0.271616019
		1.283	0.901041511	
-0.620667782	0.259800324	1.024	0.741255277	0.338120985
0.387512558	0.130067493	1.119	0.279908952	1.140817161
0.774347165	0.374385573	1.164	0.180756058	1.42778179
1.225102915	0.736000305	0.902	0.97704966	0.029836327
-1.15714389	0.687118232	0.668	0.596702014	-0.551853532
		0.509	0.527447867	
-0.661377867	0.288298437	0.971	0.839103307	-0.205934013
		3.084	0.480610372	
		0.771	0.986266941	
-0.743645119	0.350110263	0.873	0.75293367	-0.323622473
		0.966	0.624837661	
0.769435109	0.370464524	1.052	0.450372507	0.795847644
-0.867154446	0.45050465	1.082	0.395139325	0.89023171
		0.885	0.671076403	
0.890639945	0.470184023	0.91	0.710765429	-0.387582252
-0.447377651	0.15753171	0.825	0.194497813	-1.386556122
0.142713914	0.060544137	0.815	0.41916862	-0.811028455
2.593165405	0.999774642	0.757	0.190768343	-1.32888316
		0.001	0.001	
1.959337939	0.985274654	1.29	0.116576742	1.706650068
		0.777	0.035643843	
		0.788	0.595358065	
-0.15772897	0.062896172	1.201	0.947354081	0.066327898
1.140311818	0.674431799	1.416	0.046896488	2.209367198
1.102403406	0.645125204	1.523	0.053881885	2.071515241
-2.351387124	0.998655084	0.447	0.067300662	-2.037163303
-2.188777985	0.996088727	0.739	0.099272138	
-0.48799268	0.178527786	0.785	0.277785489	-1.065283506
		0.684	0.299290957	
0.929792938	0.503131367	1.149	0.215201809	1.325192923
		0.596	0.132118514	

		2.324	0.587425856	
1.708028488	0.950392267	1.152	0.248952463	1.240992814
		0.859	0.515348572	
		1000	0.001	
0.413114763	0.141302004	0.85	0.552599848	-0.608406298
-0.960468894	0.528941392	1.04	0.518784696	0.66316395
0.563834871	0.222699501	1.031	0.485613952	0.742945221
		0.74	0.157023535	
-0.70525441	0.320622931	0.791	0.145494957	-1.532111698
		0.461	0.425405138	
2.047242926	0.990916892	1.79	0.017305803	2.741309021
-0.639994703	0.273139988	1.024	0.82898149	-0.211300556
		0.595	0.034901417	
0.955749277	0.524975658	1.024	0.767779525	0.30400077
		2.307	0.107998708	
-0.014503875	0.050108252	1.125	0.46672169	0.721160866
		2.494	0.648697313	
		3.237	0.681081466	
-1.920828768	0.981977399	0.645	0.005978409	-3.128165718
		1.104	0.980720669	
		0.971	0.670727817	
-1.509473402	0.89075076	0.716	0.229964072	
1.342422354	0.810154183	1.15	0.432680771	0.804997309
		0.612	0.103726164	
-0.032347854	0.050538605	1.14	0.484858284	0.708072392
-2.01661185	0.989214022	0.659	0.018459354	-2.58851568
1.309077475	0.790500625	1.358	0.181446141	1.464114204
1.592445454	0.920034657	1.435	0.026222782	2.518333174
-0.924414188	0.498601337	1.009	0.790428526	0.276459896
0.88608786	0.466362393	1.002	0.448058062	0.815913559
		1.21	0.168146216	
		1.249	0.246718433	
-2.233557829	0.9970537	1.046	0.794747052	0.262042495
-0.272630291	0.089025833	0.875	0.635987132	-0.489783889
1.602515946	0.923139876	0.657	0.064600024	-1.929878826
0.172884	0.065515238	1.051	0.830046277	0.215437561
-1.255599543	0.756593045	0.13	0.279097902	-1.174067661
		1.23	0.459954428	
		0.856	0.784541347	
		1.208	0.804376218	
0.888049839	0.468009179	0.819	0.592148272	0.575266345
-1.834021663	0.972178216	0.847	0.107371922	-1.74019598
		0.42	0.250158691	
		0.001	0.001	
0.177966858	0.066449092	1.048	0.614887506	0.51972987
-1.309857853	0.790973874	0.86	0.121947261	-1.568352151
-1.390130926	0.836219246	2.044	0.030265104	2.390030393
-0.48350029	0.176112523	0.981	0.958083324	-0.053903707

-1.161828371	0.690610583	0.663	0.067408472	-2.017777285
2.189449475	0.996105076	0.707	0.12432415	
0.676262105	0.299086319	0.802	0.036803785	-2.212981784
0.660257279	0.287493966	0.467	0.001259885	-3.688733576
1.253439556	0.755163727	1.104	0.610231752	0.512593275
-0.939584732	0.511376625	0.773	0.075405649	-1.906028468
-1.205934952	0.722616004	1.179	0.482510709	0.698776293
		1.612	0.302821243	
		1.149	0.688306392	
		0.001	0.001	
-2.727180457	0.999924509	0.767	0.019565439	-2.574485883
-1.102865407	0.645488057	0.977	0.715249617	-0.363893926
		1.068	0.934638638	
		0.913	0.94331679	
		1.039	0.783999142	
0.774841064	0.374780567	1.287	0.002960382	3.503455204
-2.78915337	0.999955603	0.545	0.208138422	-1.322904077
		1.313		
		1.573	0.555274946	
1.413897083	0.848290815	1.117	0.25768867	1.23593018
-0.995115962	0.557940214	0.623	0.099188503	-1.776186918
-0.141742591	0.060400269	0.885	0.227782408	-1.28046575
		0.431	0.111610257	
		1.015	0.764172424	
		1.207	0.778760454	
		0.873	0.963036334	
1.128669967	0.665535881	0.647	0.05896022	
-0.437300928	0.152616952	0.872	0.320466111	-0.997525637
-1.322827656	0.798746405	0.778	0.334742209	-0.99345573
0.053211398	0.051458233	0.921	0.613215646	-0.508466278
0.578043953	0.2316667	1.074	0.502503951	0.694498034
-1.018970569	0.577735208	0.987	0.902957932	0.127300562
		0.755	0.695316806	
		1.804	0.297646566	
		0.724	0.181977086	
		1.381	0.221467858	
		0.387	0.026408138	
		0.801	0.333646364	
		0.869	0.479037658	
0.915178326	0.490823548	1.127	0.713625084	0.36363574
-1.576659614	0.914977279	0.939	0.516528445	-0.654330137
-1.500332992	0.887104656	0.722	0.041669344	-2.262611656
1.755764522	0.959866955	1.887	0.019567036	2.710987824
-0.206034129	0.07211115	2.795	0.000869061	
		0.592	0.18309106	
0.360192894	0.118922673	1.207	0.892510544	-0.135364701
-1.528880683	0.898209655	0.606	0.024951291	-2.492647091
-0.303992181	0.098721092	0.79	0.453595026	-0.800565926
-1.439381549	0.860560219	0.534	0.001677601	

0.547192539	0.212468017	1.351	0.047655546	2.206952366
		1.326	0.049709609	
0.202863557	0.071428462	0.747	0.341208051	-0.995551645
		0.741	0.186390657	
-2.299009524	0.998080851	0.701	0.05848768	-2.059007676
2.72421775	0.999922599	1.622	0.021517312	2.591147238
		0.896	0.858104068	
1.041620443	0.596350161	1.115	0.948464576	0.064163867
0.786051317	0.383781082	1.1	0.572645666	0.577794496
		1.17	0.758558787	
		0.001	0.001	
		0.001	0.001	
-1.86536452	0.976134454	0.807	0.331651276	-0.987671783
		3.223	0.107455472	
		0.709	0.343609354	
1.08938285	0.634845645	1.085	0.369612121	0.962695369
		1.293	0.19168187	1.390828532
		1.073	0.24434727	
2.689177196	0.999896281	0.722	0.077266703	-1.811480044
0.595153396	0.242741079	0.518	0.122919794	
		0.548	0.474884498	-0.734416847
		2.026	0.328963777	
0.17575209	0.066038748	1.517	0.253151676	1.184893527
-0.718522227	0.330695447	1.651	0.009602605	3.035518585
		1000	0.001	
0.139344927	0.060049437	1.048	0.635268089	0.484913036
0.425605282	0.147060403	0.931	0.747277138	-0.328468149
		1.283	0.517563741	
-0.45046919	0.159063136	0.959	0.565397012	-0.572923995
1.351580839	0.81534667	1.194	0.224041202	1.290508993
		0.41	0.955698792	
		0.001	0.001	
		1000	0.001	
		1.78		
		0.885	0.974882539	
		1.086	0.665867864	
-0.915724547	0.491283468	0.521	0.074712525	-1.900707248
2.118731229	0.994005369	1.347	0.296690165	1.059703762
		1.015	0.977404502	
-0.99931006	0.561432594	0.851	0.296697372	-1.057922905
		0.426	0.065565365	
		0.947	0.858478311	
		1.76	0.156074726	
		1.465	0.073415455	
-2.549189548	0.999682513	0.554	0.018510526	
-1.455633602	0.868021737	0.65	0.215947046	-1.273082844
		1.104	0.79685208	

-1.00848294	0.569053623	1.051	0.692051241	0.407198099
1.812668365	0.969180907	1.073	0.346822843	1.004370378
0.89012877	0.469754729	1.291	0.133930195	1.612707636
-0.345104942	0.11314017	1.01	0.742730664	0.343597898
-0.245306861	0.081487175	0.697	0.160827607	-1.514189535
0.324066937	0.105518023	0.954	0.979538448	0.026257144
		0.542	0.225777806	-1.270116981
-1.449667416	0.86531529	0.743	0.203352536	-1.330052561
		0.585	0.029610833	
		0.82	0.790814374	
1.896309054	0.97956499	0.753	0.006662519	-3.040967331
		1.204	0.752170253	
-1.07891629	0.626510851	0.807	0.451684911	-0.772726197
		0.965	0.765266106	
1.19095111	0.711926617	1.066	0.427774426	0.84610326
		2.625	0.000807501	
		0.945	0.781661526	
		1000	0.001	
1.036490665	0.592151791	1.666	0.016260819	2.690773393
		1000	0.001	
		2.004		
1.544131348	0.903805159	1.008	0.837232178	0.211719537
		0.001	0.001	
2.472115745	0.999432073	0.881	0.262314643	
-0.53221703	0.203516286	0.858	0.278005052	-1.111703712
-0.404764288	0.137553685	0.515	0.366304587	-0.955163876
0.321487905	0.104618837	0.937	0.807769871	-0.25103473
		1.169	0.246602283	
-2.039239314	0.990496373	0.805	0.224484772	-1.282719857
-0.233223879	0.078420278	1.16	0.037006068	2.306813819
		0.001	0.001	
		1.06	0.78099447	
		1.084	0.795541649	
0.420182922	0.144538254	0.861	0.260566149	-1.136687239
		1.018	0.925053435	
-0.232607267	0.078268131	0.867	0.28592971	-1.083517546
		0.819	0.365982266	
0.475148066	0.171683211	1.129	0.419713196	0.83869397
-0.620556901	0.259724813	0.634	0.0607496	-2.090557364
1.880456758	0.977863779	1.546	0.024840471	2.561605024
		0.761	0.053530721	
		1.316	0.243762018	
-2.127456041	0.99430977	0.812	0.365057844	-0.943274282
0.154422247	0.062357493	0.967	0.854128256	0.191596322
		1000	0.001	
-0.579366607	0.232512109	0.899	0.334838122	-0.957629034
		0.403	0.12073293	
		1.209	0.161275607	
		0.892	0.803018798	

		1.294	0.335881055	
		1000	0.001	
0.108048524	0.056028546	0.966	0.456754024	-0.734662922
0.805277722	0.399364251	1.142	0.289684556	1.109373686
0.839649055	0.427615547	0.485	0.121638696	
		2.201	0.004360073	
-0.993471844	0.556569921	0.949	0.747644312	-0.32470248
		1000	0.001	
		1000	0.001	
		0.729	0.22387362	
0.915714688	0.491275167	0.825	0.457328121	
		0.969	0.814768368	
		0.853	0.806248791	-0.250002808
		1.06	0.792100642	
		0.931	0.472622496	
1.312700432	0.792692369	1.124	0.661136247	0.434149501
-0.293882966	0.095473409	0.817	0.244922235	-1.186800742
		0.844	0.378662193	
2.481130819	0.999468753	0.839	0.198475754	-1.306210517
-0.408465166	0.139204881	1.049	0.503799035	0.698776735
		0.774	0.303531017	-1.047984897
0.264523737	0.086701471	0.997	0.974250686	0.032861569
-0.073039446	0.052749563	0.724	0.529073661	-0.67012841
		0.672	0.187582197	
		1.406	0.120817737	
		1.05	0.735353005	
-0.408793557	0.139352169	0.772	0.198406266	-1.364594066
		0.001	0.001	
		0.628	0.11109086	
1.463424539	0.871499039	1.634	0.025367347	2.559139368
		1.873	0.938343045	
		0.868	0.286975232	
		0.75	0.072740843	
		0.205	0.913759059	
-0.102941679	0.055470389	0.894	0.714832837	-0.377629633
		0.797		
		1.106	0.711079737	
-1.681298948	0.944352522	0.852	0.376960384	-0.902466375
		0.001	0.001	
		0.001	0.001	
		1.898	0.147480917	
		1.209	0.113256016	
		0.886	0.563614223	
-0.840968781	0.428708814	0.867	0.779612772	-0.285990091
		0.553	0.023303234	
		1.098	0.530064762	
-1.001029051	0.562862605	1.122	0.625680713	0.501787974
-1.008111798	0.568745752	0.701	0.166787997	-1.424834686

1.545505987	0.904298167	1.164	0.513809491	0.670476432
-0.110374393	0.05629183	0.607	0.014949036	
		0.616	0.059907226	
		0.909	0.665607373	
-0.872672525	0.455119263	0.949	0.647695585	-0.461840302
		0.813	0.704346774	
-2.738206405	0.999931232	0.916	0.364457037	-0.919550116
		1.029	0.787191805	
		0.615	0.289938319	
		0.878	0.424050508	
		1.91	0.061692354	
-1.743403043	0.957567276	0.579	0.147797123	-1.497383526
		0.9	0.811157019	
		1.78	0.012048201	
-1.888463224	0.978737395	1.007	0.991398997	-0.010889189
-0.926301412	0.500190784	0.665	0.007789795	-2.903778324
		0.778	0.151168801	
		0.84	0.68408364	
		1.61	0.769575915	
-3.242139067	0.999999436	0.748	0.732733675	-0.347752764
		1.384	0.116288474	
		1.468	0.629166542	
-1.775109602	0.963261482	0.678	0.050474073	-2.034241314
0.066088731	0.052250484	0.849	0.12686292	-1.583103368
-0.13708348	0.059724139	0.595	0.010777994	-2.945129989
		1.486	0.059790854	
-0.668813794	0.293664254	1.342	0.096644153	1.791336258
0.455582435	0.161620285	1.589	0.000771754	4.092635628
		0.221	0.170064141	
-0.275880511	0.089978638	0.752	0.388475118	
		0.96	0.822393993	-0.225577245
		1000	0.001	
		1.249	0.495431145	
3.406328634	0.999999906	1.973	0.009987761	2.852640944
		0.758	0.474262474	
-1.434065108	0.858058145	0.812	0.362224417	-0.9188764
0.583034353	0.234865885	1.12	0.808255747	0.24193385
		2.194	0.568558939	
-1.92748798	0.982588996	0.833	0.167710012	-1.409625674
		1.845	0.118018007	
		2.02	0.324083698	
		0.969	0.704355035	
1.097664302	0.641395551	1.175	0.342567383	0.970554724
		5.651	0.05242439	
-2.968136521	0.999991237	0.995	0.77309358	-0.285427414
1.429982583	0.856116238	1.305	0.118285965	1.731275003

0.34347885	0.11253275	0.548	0.009199188	-2.8674518
		0.001	0.001	
1.259350662	0.759064475	1.332	0.215225911	1.289214957
-1.595521461	0.920993053	0.561	0.00269859	-3.352534626
		0.776	0.353207867	
		1.095	0.965716292	
		1.038	0.999050469	
		1.918	0.052822203	
		1.209	0.33583126	
0.459349885	0.163523709	0.998	0.982649361	0.022092564
		0.736	0.764918452	
1.045927526	0.599866631	1.197	0.204049338	1.29479511
		0.532	0.22282758	
		0.956	0.839842015	
		0.971	0.875256749	
0.103903154	0.055573384	1.132	0.460156512	0.770423692
		0.976	0.776691264	0.29191165
		1.612	0.998158358	
1.907133166	0.980661768	1.218	0.21334031	1.322520471
		0.948	0.774453488	
-1.228286336	0.738190779	1.17	0.348166652	0.939040077
		1.421	0.684558547	
		0.884	0.779205179	
1.082351756	0.629253383	1.393	0.091745627	1.835905404
		1.64	0.012220188	
		2.688	0.079821539	
-2.767619909	0.999946514	0.658	0.021984292	-2.489028028
		1.06	0.856505439	
0.277533489	0.090467803	1.557	0.043012453	2.186424634
-0.046336748	0.051105548	0.999	0.860890279	-0.175361945
0.18443839	0.067678555	0.952	0.671971022	-0.424084946
		1.263	0.619425045	
-0.68490438	0.305435535	1.118	0.448192364	0.765472776
0.271255554	0.088626428	0.716	0.200331411	-1.352207966
2.30973699	0.998214018	1.666	0.000736314	4.252954973
		0.927	0.503451611	
-1.472694315	0.875552745	0.89	0.611858927	-0.512994117
1.311830444	0.792167305	1.758	0.01208695	2.904455144
-1.964314155	0.98566026	1.011	0.967801428	-0.040391671
		0.82	0.196829039	
1.02401135	0.581894719	0.88	0.653752191	
-1.749534539	0.958720952	0.923	0.960465821	-0.051634128
		1.054	0.684574813	
		0.646	0.233254717	
-1.266498429	0.763735664	0.91	0.528717333	-0.633574135
-1.342548176	0.810226123	0.749	0.026088899	-2.430630452

1.510381299	0.891108239	2.004	0.01941339	2.769900722
		2.12	0.162148461	
0.059994006	0.051854108	1.086	0.56044031	0.610849037
0.324769017	0.105764134	1.082	0.881778876	0.14744813
		1	0.766522775	
		1.151	0.476096514	
		0.468	0.904940837	
-1.605335845	0.923992719	0.85	0.214836644	-1.322202714
-2.209262288	0.996560707	0.846	0.296988447	-1.045740436
1.391702924	0.8370365	1.379	0.405198364	0.836638156
		1.133	0.7146223	
-0.247212681	0.081985792	0.919	0.610955665	0.536493562
-1.232344877	0.74096979	1.09	0.292331357	1.128082455
0.078428102	0.053171024	0.84	0.358687514	-0.951387581
		0.708	0.063864301	
		1.556	0.037316906	
0.951371324	0.521294771	1.186	0.529461392	0.630518128
0.49762071	0.183781264	0.715	0.022357147	-2.454849657
0.759453065	0.362538777	0.913	0.684155132	-0.426056927
0.791950851	0.388543763	1.301	0.059300289	2.063478
		0.809	0.809775188	
		1.403	0.11698296	
-1.685718425	0.945389417	1.115	0.377687827	0.912660672
1.437208552	0.859541198	0.581	0.168060987	-1.420416059
		1.763	0.037065127	
-0.951014293	0.520994502	0.695	0.150557974	-1.534427595
		0.904	0.878109078	
-0.689251307	0.308652151	0.759	0.103427713	-1.763504539
0.336432483	0.109936035	1.282	0.056748676	2.128638377
0.114290883	0.056748022	0.594	0.062286069	-2.045616587
-1.641440562	0.934284169	0.501	0.12927825	-1.642198673
		1.104	0.737657928	
0.144629304	0.06083078	1.018	0.997172229	-0.003564003
		0.165	0.09078576	
-1.263633036	0.761869109	0.865	0.572162315	-0.58294678
1.117487591	0.656902203	1.185	0.298060418	1.103948664
-2.587466268	0.999764301	1.081	0.559177595	0.606538945
-0.823765404	0.414503912	0.617	0.029998838	-2.343683853
1.027452963	0.584729387	0.993	0.722891371	-0.364722131
		1.433	0.148224749	
		1.006	0.98948657	
3.815860882	0.999999999	1.103	0.957050193	-0.052926345
3.219039912	0.99999928	1.192	0.383107117	0.897044917
		1000	0.001	
		7.696	0.79235642	
-0.381635041	0.127596332	0.98	0.806530574	0.250480392
-0.086717654	0.053878382	1.009	0.981401473	0.023551135
1.377821623	0.82972789	0.908	0.205448337	-1.277555702

-0.766533927	0.368155036	1.312	0.137714531	1.548513229
-1.732227609	0.955397475	0.834	0.202222029	-1.317705191
-0.411921081	0.140761208	0.714		-2.425287008
0.625998275	0.263444311	0.948	0.507167882	-0.658167448
2.012947048	0.988992729	1.085	0.435785948	0.815224693
		1.362	0.301344288	
-0.554589878	0.216979294	1.006	0.660147209	0.465348414
-2.187567925	0.99605911	0.702	0.123047492	-1.55921821
		1.769	0.044328782	
0.731281955	0.340502101	1.27	0.320758993	1.023499393
-0.267369745	0.087509037	0.844	0.579034665	-0.569069433
0.003457632	0.050006152	0.684	0.912345485	-0.112873727
-0.657534864	0.285544121	0.742	0.176362857	-1.456143577
1.169261709	0.696116856	1.297	0.146666881	1.542666069
		0.873	0.68285227	
0.018418674	0.050174583	1.076	0.758442094	0.309200711
-1.875987757	0.977363096	1.048	0.555599644	0.61090773
		1.016	0.705109608	
0.359451422	0.118632317	0.987	0.694980496	0.403249231
		0.9	0.750826776	
-1.157449114	0.6873463	0.68	0.063119674	-2.030750522
1.534771525	0.90039851	1.879	0.01207639	2.884286331
-0.043400703	0.050969806	0.989	0.740377999	0.351295147
		0.289	0.049317951	
1.071087949	0.620237585	0.882	0.246830564	-1.169364182
		0.924	0.958126571	
-1.663973558	0.94013689	1.076	0.906132155	-0.117515091
		0.288	0.256232845	
-2.146088137	0.994914261	0.592	0.121821361	-1.668616633
		0.96	0.881024457	
2.312527495	0.998247251	1.204	0.617653418	0.499576451
		0.562	0.402679035	
0.000819413	0.050000345	0.823	0.787571306	-0.284842585
-0.09892326	0.05505042	0.756	0.144870324	-1.581901954
-2.305918326	0.99816761	0.438	0.194109392	-1.376185157
		0.962	0.919919232	
1.817848641	0.969931592	1.195	0.237133123	1.260390052
-0.171885882	0.065335137	2.609	0.055758171	2.012991235
-0.815852763	0.408007026	0.58	0.060105716	-2.085607165
		0.561	0.049018605	
-0.779074728	0.378171867	0.888	0.552637003	-0.597282316
0.242276056	0.080702595	1.065	0.506275346	0.68900906
		1.338	0.452819065	
		0.835		
		1.733	0.00353032	
0.250611975	0.082885239	0.59	0.047868395	-2.068287086
		1.021	0.912603238	
		0.614	0.221976901	
0.003045171	0.050004772	1.143	0.851075982	0.187415506

0.881394856	0.46242573	0.973	0.558517809	0.623419767
-1.174229327	0.699772003	0.65	0.006017271	-3.118881617
0.651683806	0.281375627	0.833	0.351210266	-0.95614854
-0.878237759	0.459779504	1.218	0.479116183	0.729122869
2.304902894	0.998155087	1.394	0.011230215	2.825609339
-0.363383572	0.120179423	0.734	0.162188141	-1.494629345
		0.619	0.704365752	
1.52181918	0.895539854	1.084	0.421263661	0.839487607
		1.038	0.768993252	
		1.103	0.893662226	
-2.887570244	0.999981507	0.9	0.335746527	-0.983724708
-0.457897797	0.162788133	0.978	0.905730917	-0.119876784
		1.017	0.912887475	
		1.274	0.918475041	
		2.592	0.008908429	
		0.83	0.53241762	
1.582847891	0.916987731	1.252	0.333487668	0.984950847
		1.876	0.36339906	
0.247884207	0.082162451	1.225	0.214722761	1.292982952
-1.963762241	0.985617926	1.039	0.88388515	0.144852923
2.012786263	0.98898293	1.066	0.516341858	0.67700267
		0.987	0.476909658	
-2.341824412	0.99856369	0.49	0.215773397	-1.344387471
0.54752272	0.212668124	1.199	0.153351079	1.543379824
-1.124411	0.66225767	0.885	0.501816979	-0.682056551
0.866835676	0.450238271	1.053	0.530160266	0.657822786
2.121976352	0.994120236	1.216	0.244117387	1.191919833
-1.095130871	0.639396155	0.76	0.002167962	-3.526090404
-1.175231528	0.700506994	0.074	0.241322998	-1.281805873
		0.806		
0.033231965	0.050568459	0.936	0.449526581	-0.760569536
-0.801780734	0.396516967	0.969	0.797529665	-0.263442207
		1.052	0.50879323	
0.295249131	0.095905472	1.205	0.202393162	1.388197751
		0.908	0.617862195	
		0.742	0.542350676	
		1000	0.001	
		0.552	0.267853242	
0.4349424	0.15148363	1.044	0.617141042	0.517848388
0.32584526	0.106142511	0.932	0.687651356	-0.407755143
1.31548619	0.794368361	1.362	0.195278151	1.375771244
-1.954535028	0.984894042	0.827	0.770625873	
0.796222926	0.392003293	1.33	0.080152675	1.909481324
		2.789	0.027033232	
-1.556998552	0.908347258	0.897	0.183551525	-1.373335475
0.64904943	0.279508771	0.801	0.195844876	-1.369451664
1.697723079	0.94812884	1.264	0.064482319	1.989716328

1.139676094	0.673948529	1.587	0.103541222	1.6924007
0.728835645	0.338613138	1.233	0.403232238	0.879738005
1.022988926	0.581051773	1.255	0.135991979	1.607497498
-0.934651323	0.507222802	1.372	0.128571062	1.5984006
-1.329701541	0.8027944	0.856	0.287889695	-1.083226499
0.13505416	0.059436856	0.943	0.780883198	-0.284910665
1.580608471	0.916264362	1.061	0.68153699	0.411148923
-3.647251816	0.999999994	0.821	0.094402879	-1.74551498
0.645817943	0.277227302	1.073	0.461217455	0.773303302
		0.25	0.30224583	
-1.074469665	0.622951547	1.28	0.110709583	1.685965019
-0.15625851	0.062655181	0.551	0.010873433	
		0.514	0.329669628	
-0.97602949	0.541993804	0.837	0.310067443	-1.02507454
0.828262209	0.418206838	1.169	0.666507554	0.425812915
		1.233	0.459959632	
1.030399554	0.587152819	1.211	0.213991738	1.324511316
-1.47151347	0.875041394	1.318	0.187973774	1.393122043
		0.82	0.53752312	
		0.778	0.614891279	
		0.533	0.142473192	
1.325245826	0.800176113	1.326	0.090440462	1.834096968
		1.305	0.210368081	
0.054900883	0.051552388	1.114	0.245170016	1.21248041
-1.77044615	0.962465472	0.531	0.002371374	-3.738401025
0.346650216	0.113720245	0.59	0.116403339	-1.661388576
-0.07437616	0.052851295	1.224	0.209299577	1.274595671
		0.442	0.08054701	
0.039042517	0.050784728	1.39	0.142262734	1.546623692
		0.422	0.120982442	
		0.803	0.478992877	
-1.654336937	0.93768595	1.017	0.798813489	0.261760623
0.394936436	0.133246427	1.026	0.716657682	0.378760078
0.35193471	0.115724925	1.349	0.067741202	1.987238849
-1.400559551	0.84159117	0.892	0.222517413	-1.245234564
1.12665628	0.663987466	1.045	0.593467782	0.555094931
0.258005912	0.08488642	1.21	0.362445104	0.957805384
-1.148889452	0.680923374	0.81	0.133600371	-1.513875732
		1.075	0.559227725	
		2.221	0.759642512	
-1.502865251	0.888123376	0.883	0.215401047	-1.306431137
		1.126	0.547510241	
		1.012	0.657492824	
1.499267319	0.886673962	0.819	0.232033394	-1.192437231
		0.769	0.934655614	
0.400902647	0.135847799	1.105	0.514248101	0.666271204
		0.677	0.304125698	
0.207941148	0.07252708	0.908	0.354732168	-0.930324837
1.137084915	0.671975717	1.19	0.076193635	1.951027748

		1.087	0.565367631	
		1.564	0.938423327	
1.973267415	0.986332157	0.666	0.183418705	-1.360862781
-0.013589056	0.050095026	1.254	0.12731243	1.635315356
		0.871	0.491349832	
1.306831266	0.789134922	1.265	0.030539767	2.454670074
3.946396754	1	1.199	0.850149598	0.186257423
		1.019	0.923439408	
-0.097504488	0.054906183	0.742	0.14194402	-1.52992294
-1.305680187	0.788433039	0.884	0.378705544	-0.88678711
1.652267159	0.937149446	1.378	0.222121867	1.294532439
		0.875	0.719195293	
		1.041	0.468637553	
-1.579321926	0.915846648	2.002	0.256835344	1.136384749
-1.618161711	0.927780939	0.413	0.059826135	-2.065359455
-0.444476889	0.156104848	0.942	0.98388235	0.020789481
-0.018341167	0.050173116	1.233	0.172220847	1.455443641
-1.266365905	0.763649514	0.623	0.013156325	-2.689729968
1.16828973	0.695399355	1.002	0.898490317	0.129896048
1.101832896	0.644676946	1.063	0.79430231	0.265712078
-1.114848551	0.654852393	1.12	0.529950348	0.65320569
-1.615653944	0.927051877	0.641	0.024809932	
		1.219	0.553000557	
		0.947	0.461384238	
-0.425031731	0.146792004	0.904	0.4103395	-0.828210453
1.124303751	0.662174956	0.91	0.409746104	-0.828745762
2.553788434	0.999693577	1.608	0.043188487	2.220925881
2.333712489	0.998481753	0.918	0.626762107	
-1.447534513	0.864338544	0.814	0.396477239	-0.884051015
		1.644	0.281762829	
-1.655825677	0.938069628	0.512	0.011762109	-3.115696652
		9.168		
-0.898147106	0.476492419	0.935	0.437825044	-0.769586767
0.636562357	0.270745416	1.226	0.248414813	1.226849256
-1.563152361	0.91046243	0.814	0.374521807	-0.94494347
2.205280837	0.996473193	1.031	0.659349185	0.451492176
0.235895518	0.079084382	1.147	0.568840984	0.583122849
-1.274063911	0.768624873	0.844	0.028387875	-2.355146951
-1.952907328	0.984763148	0.89	0.21380992	-1.319076047
		1.138	0.556441747	
2.376542367	0.998870689	1.176	0.583104047	0.561567174
		0.793	0.457803483	
-3.965485661	1	0.945	0.443943728	-0.795708125
0.014220088	0.050104057	2.125	0.296159882	1.055883917
-0.746213087	0.352118577	1.413	0.033292106	2.35422446
-0.740292087	0.347494408	1.543	0.127181822	1.611545901
-1.601571579	0.922852641	0.835	0.316386127	-1.056942292
		1.441	0.646910542	
0.220375466	0.075337152	0.457	0.133378102	-1.573300876
		0.949	0.991064115	

		0.695	0.702082526	
		2.496	0.655261979	
		1.051	0.682094345	
0.867493822	0.450788272	0.714	0.051566626	-2.00999915
0.733151626	0.341948563	1.35	0.075133568	1.924547399
1.576590722	0.914954694	1.22	0.106834708	1.723953486
-1.072628578	0.621474749	0.876	0.664077226	-0.451500607
0.840911935	0.428661711	0.805	0.803219964	-0.261873967
-0.765262229	0.36714422	1.251	0.599096475	0.536105212
		0.476	0.970387703	
0.554178632	0.216726955	1.209	0.374597601	0.899409973
0.67811056	0.300439133	0.866	0.49040297	-0.716454892
		1.481	0.120561238	
-2.841007514	0.999971872	0.627	0.043647076	-2.09726461
		0.642	0.119994276	
-0.972578408	0.53910243	0.569	0.051259423	-2.049776975
0.291176199	0.094623662	1.302	0.534103663	0.611286885
1.351902365	0.815527339	0.647	0.155247648	-1.495246985
		0.853	0.24169667	
		0.739	0.483851067	
-1.388390507	0.835311328	0.744	0.054626872	-2.067333188
		0.808	0.275259037	
-0.109168244	0.056154586	1.038	0.688833402	0.411564331
1.155610651	0.685971489	1.356	0.010804163	2.810866717
		0.875	0.473109017	
-0.636644425	0.270802544	1.112	0.407922467	0.870643479
-0.402415067	0.13651384	0.97	0.581608234	
2.179555647	0.99585788	1.751	0.004045365	3.498449048
		1.007	0.987538568	
0.675501408	0.298530421	1.205	0.174974555	1.471196587
-2.941049289	0.999988702	0.613	0.005089393	-3.112309812
2.273108859	0.997721339	1.768	0.078905749	1.84926216
0.693105571	0.311516888	0.81	0.569243042	-0.576471692
1.100933862	0.643970154	1.09	0.342989006	0.954810124
0.528010274	0.201045708	1.25	0.126203985	1.675038356
0.504090154	0.187370082	1.376	0.233368425	1.202847464
		0.876	0.637375272	-0.476797519
2.104642644	0.99348327	1.342	0.295168314	1.060618777
-0.769166974	0.370250874	0.797	0.782143558	-0.291394857
		0.749	0.17829553	
		1.068	0.876263653	
-0.159494725	0.06318862	0.646	0.461152474	-0.784402773
		1.238	0.250486944	
0.17787753	0.066432439	3.778	0.012778946	2.789872441
-1.632177556	0.931753094	0.532	0.015378001	-2.795230816
		1.008	0.791946327	
0.34920372	0.11468487	0.802	0.530115799	-0.649136329
		1.259	0.079484776	

		1.018	0.598710876	
2.298913893	0.998079625	1.376	0.010765746	2.861605039
-1.782735356	0.964533312	0.531	0.306978576	-1.091837023
0.394350027	0.132992984	0.91	0.356387141	-0.923740527
-1.124208228	0.66210128	1.486	0.133085143	1.582322565
		0.784	0.384372691	
-0.389520578	0.130920989	0.604	0.190931576	-1.392874379
		0.553	0.240840722	
		0.722	0.474826946	
		1.375	0.287324451	
1.376127215	0.828821577	0.488	0.000643606	-3.997078433
-0.284168187	0.092462423	0.996	0.90896632	0.117235706
1.299198509	0.784455244	1.48	0.297912572	1.035694738
0.844352167	0.431514136	0.835	0.043498343	-2.179725931
1.602848564	0.923240849	0.804	0.146194904	-1.516687549
0.058685474	0.051774026	1.12	0.867169635	0.167027723
		1.455	0.041036093	
2.267013336	0.99762831	0.95	0.866915588	-0.170688571
0.650849125	0.280783455	0.653	0.070263911	
1.217568632	0.730778994	1.034	0.997302546	-0.003369323
-1.257073129	0.757565554	0.594	0.057340214	-2.055169334
		1.063	0.744319644	
2.305418421	0.998161454	1.529	0.11630726	1.638570585
1.031396047	0.587971637	0.819	0.877297363	-0.158503272
1.223055964	0.734586891	0.991	0.834993074	-0.208672033
0.508024694	0.189575708	0.952	0.771205866	0.302046397
-0.764980187	0.366920164	0.352	0.174801011	-1.483314197
-0.425017115	0.146785169	1.123	0.518882642	0.640697813
-0.54841843	0.213211563	0.93	0.410525583	-0.823813795
-0.395621877	0.133543179	0.679	0.130887713	-1.643069465
0.856562567	0.441666391	1.37	0.17170167	1.425626207
1.834938908	0.97230132	1.236	0.148406287	1.572808854
		1.163	0.68460624	
-0.067556609	0.052351704	0.925	0.671772043	-0.440324226
-1.141092696	0.675025011	1	0.819899126	0.239970859
-1.307022011	0.789251099	1.225	0.310714986	1.044602057
-0.776408474	0.376034982	1.05	0.712629287	0.372274324
0.955058416	0.524394933	0.903	0.835352867	-0.214257594
0.865490469	0.449114408	1.353	0.067991623	2.008058782
0.779896844	0.378831525	1.196	0.300769771	1.089427292
		0.916	0.908856759	
		0.591	0.647241823	
-1.565023525	0.911098317	1.088	0.289002305	1.12714413
-0.225923725	0.076646078	0.76	0.481601986	-0.720242171
-3.118271907	0.999997973	0.769	0.011406018	-2.776547612
		1.329	0.01402039	

1.41332119	0.848005491	1.341	0.074189531	1.991015105
-3.32437825	0.999999767	0.968	0.823586114	-0.225308699
-0.142473678	0.060508461	1.015	0.833336072	0.216293205
		0.834	0.640771609	
0.164467057	0.064030133	1.215	0.061505344	2.053922139
-0.07110405	0.052605557	1.061	0.433602536	0.827021883
		1.134	0.844573761	
1.821426473	0.970441152	1.674	0.004275688	3.374034847
0.446678726	0.157187024	0.826	0.220859628	-1.277207687
		1.055	0.628121838	
1.013832743	0.573486625	1.131	0.23407039	1.259808357
1.607793047	0.924729969	1.194	0.257382217	1.217866913
0.561632662	0.221328687	1.277	0.075126016	1.930923362
1.108522192	0.64992003	1.326	0.128789363	1.682903502
0.916509297	0.491944252	1.276	0.065947685	2.018890973
		0.062	0.147356778	
		0.809	0.164246561	
-0.737037909	0.344962735	0.977	0.926745648	0.095400602
-0.020895832	0.050224707	0.651	0.005917035	-3.056788999
-1.722415777	0.953419459	0.269	0.221955648	-1.316881676
0.187358397	0.068248176	1.193	0.314706721	
0.730781101	0.340115022	1.039	0.671908358	0.437754765
2.076887091	0.992335713	1.108	0.3459307	1.008925641
-0.739277284	0.346704164	0.368	0.342927023	-1.013635774
		0.815	0.308794449	
1.092587604	0.637385139	1.131	0.442785458	
-0.452374423	0.160012418	0.961	0.981247491	-0.023990334
-0.243740247	0.081080346	0.809	0.194937564	-1.330402879
		1.042	0.973197379	
-2.596199856	0.999779974	0.624	0.024560381	-2.422745043
0.129363155	0.058654512	0.963	0.970849936	-0.037726086
2.589266608	0.999767614	1.075	0.695932344	-0.3850332
0.631461246	0.267206838	1.392	0.109258897	1.740988461
0.398444244	0.13477086	1.629	0.00915537	2.960056633
1.742293182	0.957355684	1.406	0.064582153	2.032508942
0.915055048	0.490719749	0.889	0.512434897	-0.680114304
0.252395769	0.083362408	0.951	0.612336358	-0.515959628
1.881790012	0.978011326	0.844	0.279141993	-1.128755531
		0.696	0.651196206	
		1.209	0.749485681	
		0.975	0.752764937	
-1.751369545	0.959061227	1.298	0.239453562	1.209341837
-1.045126606	0.599213335	0.905	0.420897736	-0.840047055
-1.749045993	0.958629973	0.886	0.489927971	-0.719326122
0.676685519	0.299395948	1.797	0.086025779	1.837120683
1.538882107	0.901905338	0.785	0.242929902	-1.190879852
1.905514914	0.980501048	1.235	0.116294747	1.695222592

1.832815488	0.972015639	1.477	0.011586338	2.995464252
		0.799	0.238736717	-1.186243485
-2.577425633	0.999744996	1.046	0.808717478	-0.23809595
1.052829027	0.605484237	0.736	0.059419684	-1.979596307
-2.140402506	0.994736166	0.742	0.193445619	-1.372119285
-2.611643376	0.999805332	0.686	0.017623718	-2.585737573
-0.914197721	0.489997904	0.913	0.417310698	-0.806236417
2.067895365	0.991927536	1.441	0.023044004	2.542227034
0.071616898	0.052643337	0.922	0.701908362	-0.397133171
		0.539	0.066651969	
-0.59995544	0.245902615	0.932	0.527007166	-0.631002555
-1.688779129	0.946098509	0.418	0.039376174	-2.222605913
		0.659	0.373328919	
-0.015599796	0.05012523	1.035	0.701165344	0.396600993
		1000	0.001	
		0.855	0.163529217	
		1000	0.001	
0.607584785	0.250972836	1.048	0.593183117	0.541150181
-0.476696273	0.172498233	1.007	0.807198018	-0.243412242
		1.174	0.46241974	
0.264057887	0.086570155	0.859	0.888894667	
0.358028915	0.118077064	1.144	0.29241406	1.109631213
		0.813	0.753083953	
0.965710321	0.533342177	0.625	0.501009279	
1.288775406	0.777968254	0.99	0.987618598	0.015912583
		1000	0.001	
-1.097317904	0.6411224	0.901	0.434378491	-0.801037341
-0.934184512	0.506829709	0.803	0.263393951	-1.144874107
		2.052	0.249534622	
-0.512422147	0.192061346	1.067	0.603921767	0.528384735
0.507828809	0.189465488	1.886	0.175473217	1.416640928
0.068620417	0.052426458	0.992	0.946215299	-0.068564014
1.045177733	0.599255047	1.064	0.485191721	0.739126571
-3.043368459	0.999995742	1.167	0.597572036	0.529011438
		0.669	0.039862918	
-1.357717922	0.818776128	0.801	0.122380834	-1.571792881
-0.293072547	0.095218113	0.905	0.348684915	-0.938441669
1.916766368	0.981595367	1.318	0.035955803	
		2.179		
		0.316	0.214072891	
2.136506116	0.994610932	1.262	0.068191373	1.930420007
		1.237	0.432940383	
-0.148050894	0.061352563	0.914	0.285926169	-1.086364123
-1.983609044	0.987074244	0.808	0.154742374	-1.490259566
		1.114	0.627991856	
		1.448	0.203749003	
-1.761539638	0.960906043	0.969	0.847225347	-0.197172216
1.035348879	0.591215849	0.984	0.966842942	-0.042348341

-0.39596217	0.133690708	1.231	0.400384526	0.846848908
		1.021	0.943113649	
-0.855701998	0.440949525	0.682	0.293432179	-1.1067593
-1.72148793	0.953228818	0.961	0.219383389	-1.231774962
1.0144207	0.573973264	1.12	0.273305382	1.16698753
1.977467476	0.986637866	1.512	0.011381346	2.932642617
-1.327159733	0.801303329	0.825	0.148922356	-1.53052788
-0.054918283	0.051553373	0.769	0.622757327	-0.504348059
2.718055716	0.999918481	1.074	0.95278104	-0.058778111
0.760132808	0.363076641	1.123	0.396493079	0.888743904
		1.01	0.756992571	
		0.001	0.001	
0.407656701	0.13884281	0.994	0.84752835	0.199254147
-0.493965623	0.181774531	0.94	0.918269841	0.108298861
		1.069	0.833122055	
1.035805942	0.591590575	1.209	0.353222863	0.955254764
-0.207064028	0.07233528	1.047	0.573196514	0.588161122
-0.530633075	0.202583768	0.612	0.000183447	-4.74391704
-2.130691929	0.994419127	0.847	0.446329326	-0.7704365
0.01268809	0.050082843	1.202	0.460686744	0.758944509
0.915047195	0.490713137	1.204	0.323074037	1.027999944
1.655265703	0.937925528	1.173	0.384025791	0.901753623
		1.481	0.04278348	
-1.066573626	0.616605388	0.95	0.879618572	-0.15640798
		0.472		
-2.21179293	0.996615313	0.869	0.243817312	-1.22162908
-0.852393137	0.438195041	1.115	0.377205523	0.896923786
0.272393491	0.088956883	1.559	0.018582161	2.637315948
		1.158	0.707542943	
2.505874716	0.999558461	1.709	0.002376832	3.577767951
-0.220562651	0.075380757	0.946	0.534214485	-0.622503269
-0.782791113	0.3811567	0.813	0.207473309	-1.310790475
1.442430147	0.861981339	1.33	0.156164746	1.534136535
		0.622	0.442709655	
1.087526711	0.633372083	1.115	0.321440363	1.056830593
-2.784889042	0.999953928	0.794	0.073544359	-1.85508587
1.531934073	0.899348539	1.48	0.087932969	1.880636397
0.681228888	0.302727744	1.19	0.32843994	1.013702304
-0.038825071	0.050776007	0.967	0.68795461	-0.403816997
		0.773	0.320705981	
-0.382103078	0.127791638	0.742	0.047816437	-2.133928741
0.163088322	0.063794136	1.311	0.873769294	0.154500898
0.867071363	0.45043522	1.045	0.768199405	0.299472615
-1.927529071	0.982592714	0.968	0.744920596	-0.327030903
-0.087848604	0.05398044	1.09	0.769410804	
-1.475357961	0.876700818	0.946	0.730565372	
-0.426843245	0.147641027	0.753	0.167251869	-1.421511359
		0.604	0.214722601	

0.098565133	0.055013813	1.195	0.079442857	1.835688278
		1.217	0.425633481	
		0.001	0.001	
-0.63478454	0.269509413	1.164	0.232775171	1.265957089
		1.121	0.839624655	
-0.746806354	0.352583151	0.714	0.450075157	-0.792893231
1.592086448	0.919922229	1.323	0.068210026	2.021503243
1.684137271	0.945020219	1.706	0.015117871	2.737266113
		1.163	0.841767138	-0.204369348
-0.276871885	0.090271643	1.056	0.704877136	0.387556116
-0.955887628	0.525091948	0.986	0.782052667	-0.276751344
-0.574488968	0.22940342	0.925	0.960722008	-0.051721947
-0.955221535	0.524532052	0.477	0.145619457	-1.556660846
1.392447005	0.837422408	1.21	0.092458251	1.744366962
		1.605	0.020044755	
		1.302	0.282386922	
-0.393031193	0.132424459	0.755	0.184376421	-1.320322468
-1.243634565	0.748618947	0.807	0.17867683	-1.434401202
0.608020949	0.251264436	0.777	0.145894208	-1.470514668
		1.286	0.089768302	
-1.16373538	0.692027375	1.227	0.423094585	0.796756555
0.450438031	0.159047646	0.975	0.761712761	-0.30494695
-2.7983466	0.999959021	0.759	0.459982042	-0.777452434
		0.208	0.251214232	
		0.8	0.581269911	
		1.032	0.377828954	
1.238629278	0.745242533	0.94	0.873054627	
-0.281829239	0.091753545	1.029	0.808618294	0.244206236
0.281887286	0.091771063	0.949	0.890230789	-0.14386822
-1.435055193	0.858526401	0.494	0.004215547	-3.280586064
-0.333325181	0.108809211	0.853	0.848653058	-0.197820354
-0.731345595	0.340551296	0.957	0.654080786	-0.456858847
		1.002	0.667501157	
-1.46657713	0.872887857	0.753	0.479535312	-0.728577958
-1.895383881	0.979468844	0.277	0.24370164	-1.232712742
		0.001	0.001	
		0.806	0.503870333	
-2.44970649	0.999330513	0.73	0.093245346	-1.790298923
-3.242862094	0.99999944	0.24	0.011049011	-3.033660028
		1.866	0.285335248	
1.552338186	0.906720901	1.492	0.011278809	2.955137792
-0.173530166	0.065632407	0.408	0.026175526	
		0.996	0.889309131	
1.439949854	0.860825891	0.777	0.173760513	-1.456678503
-0.05247338	0.051418029	0.978	0.983925141	0.020601711
-0.302489043	0.09823079	1.263	0.207665342	1.314046342
		0.249	0.004167585	
-1.29479089	0.781725634	0.79	0.325802919	-1.003312418
		1.806	0.75107425	

0.693345154	0.311695352	1.014	0.938202269	0.078654606
-0.75413893	0.358343427	1.145	0.204004475	1.37515318
-1.164880923	0.692877069	0.662	0.050488152	-2.074050348
-1.871246477	0.976821495	0.536	0.792962092	-0.268234676
		0.65	0.370687893	
-2.436432474	0.9992627	0.34	0.0001033	-4.917127109
-1.214302669	0.728499624	0.897	0.331876088	-0.982366964
-0.348127985	0.114277572	0.923	0.852038319	-0.194304426
		0.697	0.17918595	
-2.502244331	0.999546244	0.846	0.112257993	-1.709544475
		0.888	0.507036384	
-0.211328395	0.073275705	0.729	0.11774333	
1.995321677	0.987872358	1.408	0.412778512	0.814119471
		1.56	0.265386788	
0.876372379	0.458216809	1.444	0.048227986	2.169638069
1.704218566	0.949564847	0.759	0.126420664	-1.554511847
-2.638691201	0.999843278	0.979	0.724331075	-0.352465551
-2.196935359	0.996283231	1.345	0.658271291	0.436237555
-1.660162036	0.939176678	0.393	0.088458549	-1.877716619
		1.377	0.505495576	
		1.207	0.901808929	
		1.456	0.082611553	
		0.96	0.949447125	
2.061744391	0.991637412	1.059	0.798464401	0.261064267
		1.52	0.598049864	
2.719746932	0.999919631	0.833	0.53263079	
		0.162	0.439943448	
-2.495939993	0.99952428	0.48	0.10786633	-1.76128536
-0.028738826	0.050425097	1.026	0.93966715	0.076206817
1.387319685	0.834751096	0.813	0.205393627	-1.279292904
-0.792917412	0.389325711	0.909	0.647863658	-0.474608675
		0.715	0.117749716	
		0.952	0.814369037	
0.696420774	0.313990375	0.913	0.794181855	-0.271380663
0.577101705	0.231065542	1.194	0.352020526	0.964631114
-0.716207715	0.328929009	0.878	0.933805329	0.08519377
0.557002277	0.218463169	0.868	0.393072828	-0.877506505
-0.257175273	0.084658543	1.3	0.088048821	1.89052319
		0.497	0.027450156	
-0.633107245	0.268345994	0.975	0.923354876	0.100320907
-1.942859005	0.983933313	0.994	0.975231425	0.03184952
-1.440401755	0.861036899	1.068	0.857065949	-0.179071342
2.136292381	0.994603986	1.126	0.42608438	0.79220853
		5.611	0.696240028	
-0.252304765	0.083337978	1.6	0.012325931	2.743682658
1.255043183	0.75622532	0.961	0.869257237	0.173758377
		1.096	0.901808372	
0.502719782	0.186605959	0.906	0.363894967	-0.948653433

		1.053	0.625281643	
0.111069198	0.056371583	0.753	0.438802313	-0.818183099
-3.557793621	0.999999984	0.779	0.135131504	-1.531681528
1.274168055	0.76869178	1.747	0.018386216	2.585369394
0.664367885	0.290450319	1.081	0.403003338	0.896807691
-0.124864727	0.058060385	0.951	0.422764956	-0.802742399
3.139328384	0.999998362	0.734	0.083270505	-1.78144505
-0.614842967	0.255849603	1.15	0.565671922	0.57757926
-0.444704396	0.156216404	0.898	0.420998912	-0.822654848
		1.141	0.497389302	
-1.251501151	0.753877189	0.902	0.452255043	-0.758297629
2.589859552	0.999768696	1.277	0.144433028	1.549034405
-0.957639725	0.52656446	0.973	0.753999305	-0.314800132
		1.445	0.169410219	
-2.072662416	0.992146256	0.435	0.352899454	-0.989156998
		0.272	0.168635794	
		0.93	0.892343585	
		0.787	0.70646487	
0.135309622	0.05947278	0.794	0.266768571	-1.117674613
-1.230037322	0.739391613	0.932	0.507868124	-0.673032568
1.456673807	0.868489733	1.409	0.078353518	1.928750224
0.72607581	0.336487052	0.987	0.920017444	-0.101130297
		1.235	0.373278187	0.930583818
-0.092796859	0.054442675	0.992	0.851589588	-0.187684257
2.292887456	0.998000869	1.568	0.010667531	2.806374419
-0.137538285	0.059789123	0.849	0.129412376	-1.5644228
		0.944	0.875987035	
0.650435202	0.280490025	0.894	0.531734545	-0.636917353
-2.232293111	0.997029701	0.8	0.035232397	-2.310295103
-0.070916034	0.052591775	0.894	0.158717422	-1.445833622
		1.133	0.342580342	
0.921922154	0.496502548	1.179	0.468141785	0.740842538
1.18884256	0.710406875	1.358	0.003404821	3.396620014
		1.226	0.368565236	
1.371224241	0.826181671	1.166	0.248669613	1.219773276
-0.550359961	0.214392482	0.835	0.36799023	-0.928034883
0.465689129	0.166763382	1.21	0.24954265	1.210106734
-3.271742276	0.999999588	1.133	0.752124383	0.317720467
		0.246	0.014802852	
		0.319	0.481280809	
-1.066186694	0.61629358	0.487	0.002400371	-3.55054314
		0.905	0.903582689	
2.934205984	0.999987958	0.579	0.062170424	-2.13863391
-2.298864427	0.99807899	0.538	0.033757877	-2.260937697
		1000	0.001	
1.494477752	0.884723753	1.67	0.05311555	2.138945553
		0.407	0.001347229	-3.793287919

		1.749	0.026609862	
		0.144	0.220350226	
-1.165044929	0.692998633	0.737	0.142264848	-1.555046098
2.728535619	0.999925367	3.455	0.000365977	4.456731195
0.299448105	0.097246794	0.99	0.681395302	
		1.826	0.031736288	
-0.519092105	0.195872664	0.719	0.149503806	-1.504678205
-2.624888034	0.999824875	0.896	0.221074846	-1.249631592
		1000	0.001	
-0.961112958	0.529482361	1.046	0.996400036	0.004502463
1.326097787	0.80067836	0.69	0.107973776	-1.650381596
0.118294778	0.05723109	0.851	0.486205075	-0.70840431
-1.8502127	0.974285338	0.855	0.303289185	-1.086755625
-2.632771894	0.99983562	0.748	0.081408614	-1.806760375
0.662103309	0.288819816	1.224	0.088386213	1.811530475
		0.827	0.591717013	
-0.597258641	0.244124269	0.932	0.423786233	-0.793451405
-1.512178309	0.891813316	0.639	0.095067529	-1.75541815
		1.107	0.577760491	
-0.839145253	0.427198343	0.674	0.860618394	-0.18229352
-1.843767674	0.973463166	0.72	0.070652356	-1.920807869
0.217127168	0.074586616	0.99	0.7853433	0.286837178
1.470363504	0.874542003	1.276	0.118500752	1.720526461
-0.309550481	0.10055664	0.853	0.724578931	
-1.649379465	0.936394919	0.725	0.020015678	-2.599932492
1.313697141	0.793292946	1.293	0.116450987	1.684500366
2.12685776	0.994289343	1.421	0.08327836	1.912411722
0.185907093	0.067963904	0.983	0.549817426	-0.589273333
0.601568407	0.246969723	1.016	0.783192741	-0.270241062
1.937098332	0.983440308	1.37	0.154043115	1.506599793
-1.411190332	0.846946663	0.797	0.212159132	-1.298007782
		0.525	0.057830674	
		0.732	0.82163772	
1.806124473	0.968210481	0.557	0.030670141	-2.577176311
-0.024402898	0.05030648	0.988	0.994818875	0.006795019
		0.491	0.282853532	
		1.266	0.153020489	
		1.176	0.357297018	
-0.359765642	0.118755286	1.054	0.444123492	0.788966443
-2.431440714	0.9992356	0.362	0.03182704	-2.444926431
		0.781	0.523427626	
1.062180928	0.613061131	0.844	0.054737922	-2.031754231
0.199283191	0.070670775	0.583	0.111535127	
1.995390414	0.987876912	1.234	0.077547386	1.946864161
		1.225	0.181224566	
		0.748	0.171546075	

0.58295707	0.234816146	1.116	0.688917622	0.39609326
-1.374782664	0.828100201	0.605	0.045214538	-2.070211831
-1.26264747	0.761225231	0.856	0.281448979	-1.147182209
		1000	0.001	
0.771389874	0.372023303	1.011	0.780437236	
-0.619342922	0.258898854	0.935	0.653479881	-0.463583559
-2.512629024	0.999580384	0.74	0.025722883	-2.57670472
		1.317	0.216623444	
-1.524885465	0.896705327	0.933	0.644154395	-0.466578871
		1.104	0.91666191	
		1.093	0.867470291	
0.244372048	0.081244087	1.258	0.617570796	0.494442009
1.684696934	0.945151125	0.875	0.561288562	-0.595908186
1.389883142	0.836090184	1.24	0.090871103	1.805156212
1.396094753	0.839305633	1.001	0.889066584	-0.1402918
-0.85946027	0.444081622	0.769	0.240593093	-1.200765236
		1.327	0.502541812	
		3.491		
		1.113	0.767025076	
0.228983077	0.077382412	1.01	0.921845158	0.099962879
-0.103474084	0.055527301	0.595	0.004636916	-3.259404548
		1.168	0.369845795	
1.643043388	0.934714643	1.029	0.841188998	
-0.494506257	0.182070401	0.859	0.108478106	-1.680699615
-1.494571882	0.88476231	0.811	0.173096092	-1.4057617
-1.583382073	0.917159583	0.983	0.928238013	-0.091685983
-0.816194065	0.408286746	1.098	0.588035465	0.548700397
0.505257076	0.188022424	1.179	0.215877533	1.323364479
0.168336688	0.06470342	0.878	0.37954809	-0.913573399
-1.20254866	0.720217341	0.733	0.079882147	-1.812510018
		1.243	0.095698529	
		0.542		
		0.972	0.689649585	-0.398973581
0.730961557	0.340254466	0.778	0.295444748	-1.083967288
-0.080230158	0.053318707	0.115	0.311147273	-1.079993878
		1.865	0.156759906	
		0.682	0.127428224	
1.43804708	0.859935022	1.082	0.535325033	0.634920225
1.053981463	0.606420173	1.188	0.193694235	1.396691649
-0.432860858	0.150488773	0.919	0.702444967	-0.398724516
		0.969	0.810807986	
-1.506449932	0.889554199	0.923	0.392727718	-0.845855094
		0.386	0.480184401	
		0.862	0.55142859	
0.275271013	0.089799051	0.999	0.898900231	0.130561322
-0.333961468	0.109039043	0.818	0.483778776	-0.717594367
		0.513	0.057861422	-2.006287657
		0.776	0.944667075	-0.070863673
-2.4698203	0.999422367	0.724	0.184755968	-1.366686606

		0.518	0.422651482	
-1.332078587	0.804182661	0.715	0.051928726	-2.08591119
		1.202	0.828914772	
0.168757774	0.064777659	0.871	0.923228358	0.100531418
		1.14	0.968482197	
-0.211156721	0.073237459	0.969	0.980628799	0.025677166
-0.465691087	0.16676439	1.163	0.43130222	0.800010463
-1.725703211	0.954089894	0.966	0.845346864	0.201349312
0.098251797	0.054981894	0.912	0.813588638	-0.238851869
0.300355409	0.097539274	0.858	0.29483015	-1.061625908
-0.062711224	0.052026068	1.02	0.435825813	0.839752709
0.56901145	0.225941918	0.807	0.400541132	-0.856939625
-0.305368576	0.099172324	0.982	0.686079858	-0.396714311
		1.669	0.100858006	
		2.186	0.5176635	
		0.819	0.536616938	
0.050764967	0.051327125	0.225	0.690615314	-0.424134536
-0.909175766	0.485770303	1.039	0.979256578	-0.025677457
		1.109	0.999567275	
0.311573762	0.101233605	1.508	0.013115077	2.837425782
		1.408	0.103304792	1.718876054
		2.46	0.020414593	
		7.322	0.735549322	
-2.439218913	0.99927744	0.593	0.03781856	-2.201769927
-1.262923978	0.761405972	0.856	0.376707042	-0.927226327
		3.411	0.493983143	
		1.756		
-1.135225816	0.670557301	0.623	0.069660507	-1.919476087
1.558005119	0.908695747	0.83	0.2715918	-1.125061057
		0.348	0.150295895	
1.131322049	0.667570877	1.16	0.321686524	1.022883609
-2.767513037	0.999946465	0.585	0.000270891	-4.46629799
-0.026996515	0.050375105	0.909	0.379758546	-0.909337354
		0.204	0.03606313	
		0.87	0.363957389	
-0.132465107	0.05907668	0.981	0.986843151	-0.016981007
0.60703549	0.250605867	1.509	0.018076894	2.720178531
		0.84	0.289351952	
-0.759484681	0.362563788	1.103	0.916157566	0.106011298
		0.68	0.131817026	
		1.437	0.729245849	
		0.417	0.011076028	
-1.339722084	0.808606227	0.663	0.136147757	-1.591530675
1.020296987	0.578830597	0.927	0.725021747	0.374959703
2.857313976	0.999975689	0.907	0.237579994	-1.167131443

		1.339	0.093709283	1.822652172
0.043482242	0.050973456	1.396	0.20068567	1.351264003
		1.147	0.447937267	
0.964927411	0.53268508	1.093	0.355410704	1.001056831
		2.609	0.703626574	
-0.337743244	0.110414718	1.316	0.415118167	0.843862031
3.008612984	0.99999404	1.204	0.167184656	1.464692629
		0.001	0.001	
		0.126	0.076556098	
3.341006286	0.999999806	0.705	0.080618644	-1.978774472
		0.618	0.111605709	
		0.898	0.767254141	
-0.074723632	0.052878044	0.194	0.442018832	
1.297704836	0.783532452	1.087	0.396603074	0.899299622
0.577383396	0.231245165	1.154	0.36430133	0.971159631
		1.644	0.088656451	
		0.636	0.415302823	
-0.008009239	0.050033009	1.147	0.430736665	0.811891885
-2.564095486	0.999717083	0.716	0.421811999	-0.840944129
		0.505	0.046169767	
		0.953	0.984554485	
1.639394392	0.933731428	1.689	0.196054954	1.323059027
		1000	0.001	
0.424842048	0.146703324	0.78	0.15483063	-1.47441547
		2.915	0.042777325	
-0.153331025	0.062182298	0.721	0.266600265	-1.159375735
0.403657925	0.137063169	0.953	0.614325622	-0.507625685
-2.0381332	0.990436937	0.661	0.230331444	-1.29464335
1.962600364	0.985528453	1.059	0.826923986	0.218145229
		0.935	0.73048207	
-0.476572884	0.172433177	1.086	0.378010331	0.913638625
1.762211835	0.96102555	1.012	0.835937223	0.213204885
-1.34038751	0.808988407	0.648	0.008432969	-3.026617894
		2.723		
-2.081937891	0.992556924	0.731	0.251915972	-1.169158623
-3.01818721	0.999994565	1.196	0.513891362	0.658389783
0.770262657	0.371124169	1.051	0.660810098	0.458610902
2.095505825	0.993123524	1.283	0.462020135	0.731235468
-0.110638335	0.056322067	1.55	0.242843938	1.186185676
0.508489695	0.189837525	1.137	0.385165817	0.923322556
1.019793956	0.578415253	1.191	0.267584019	1.208409038
0.33414416	0.109105119	0.823	0.187879488	
0.386247677	0.129532278	0.867	0.254661053	-1.137553984
		1.568	0.030682209	
1.167400281	0.694742114	1.128	0.501907914	0.689655599
		0.657	0.11713763	
		0.975	0.885724145	

0.343234278	0.112441656	1.16	0.302011228	1.085889523
1.861484509	0.975671934	1.237	0.072320835	1.977846518
-2.581635953	0.999753264	1.094	0.735702462	0.334197004
		0.771	0.339886544	
1.160698827	0.689770054	0.661	0.045864475	-2.064304798
		1.318	0.191511038	
		0.466	0.0188238	
-2.428742815	0.999220574	0.444	0.131936763	-1.645306824
		1.789	0.00261218	
0.648519537	0.279134014	0.674	0.372885037	-0.933190711
0.388088505	0.130311814	0.974	0.99211086	-0.01026941
		0.674	0.091471923	
-1.003269936	0.564725531	0.821	0.140825056	-1.5171451
3.792249761	0.999999999	2.257	0.000334112	4.496264419
		0.672	0.171000158	
		1.305	0.761015017	
-2.053440916	0.991231249	0.931	0.427862737	-0.823006911
1.822702795	0.970621183	2.298	0.000619618	4.208400927
1.62741689	0.930423282	3.899	0.188768987	1.315554812
-2.581950867	0.999753872	0.841	0.210920871	-1.275731702
		0.001	0.001	
1.843286151	0.973400866	1.23	0.149633392	1.540580942
0.783140757	0.381437891	0.843	0.737152131	-0.351135374
-1.103545951	0.646022312	0.919	0.822938643	-0.231880838
		1.469	0.967626228	
-1.777896056	0.963730472	0.657	0.002115693	-3.742503135
-1.639725728	0.933821177	0.974	0.76458104	-0.304608858
-1.168295117	0.695403333	0.875	0.452012319	-0.780405681
0.047628097	0.05116807	0.878	0.555776307	-0.612179301
		2.113	0.023046918	
1.080023534	0.627395475	1.293	0.02259941	2.442518754
		1.126	0.504123578	
-1.816433336	0.969728019	1.107	0.588108347	0.558303724
		1.41	0.390456899	
-0.285205118	0.092778681	0.873	0.746145398	-0.334755004
0.7767058	0.376273086	1.801	0.009023455	3.038284531
		0.535	0.559746563	
		0.866	0.884387736	
		0.591	0.576272798	
-1.113548849	0.653841197	0.618	0.002268254	-3.500316208
1.004661673	0.565881806	1.1	0.358122113	0.967303897
0.736621228	0.344639076	1.232	0.28552398	1.077918728
-0.450503839	0.159080363	0.878	0.853337765	-0.191336799
		0.86	0.289107416	
-2.371457745	0.99882985	0.834	0.230340995	-1.213200128
-2.867627285	0.999977844	0.886	0.276307482	-1.08803649
0.156696627	0.062726741	0.954	0.96717388	-0.042695866
-1.673454999	0.94247393	0.98	0.545912328	
-1.535821859	0.900785135	0.681	0.323928624	-1.052710271

		0.695	0.333212451	
-0.741208985	0.348208994	0.488	8.64036E-05	-5.213379949
-0.293683447	0.095410487	0.834	0.521259961	-0.662464006
		0.525	0.466115543	
-0.867474623	0.450772226	1.01	0.799539247	-0.250521883
-1.618824644	0.92797273	0.87	0.508554474	-0.684218667
0.863542876	0.447488008	1.011	0.777761355	0.288121758
0.111415635	0.056411539	0.951	0.928160853	0.093571781
1.293988022	0.781226283	0.873	0.374856937	-0.88826756
		0.918	0.530555887	
-0.510228714	0.190818817	1.825	0.000457282	4.242412618
		1.014		
-0.639278025	0.2726391	0.977	0.803961901	-0.250621994
		1.456	0.514859329	
1.181735787	0.705257035	1.085	0.695304586	0.398242639
		0.298	0.09290474	
		1.511	0.09905254	
0.63579461	0.270211291	0.915	0.483891428	-0.711981531
0.108029179	0.05602638	1.014	0.696942492	0.408866966
		0.472	0.123511024	
		0.556	0.547928571	
		1.438	0.3379676	
-0.358006147	0.118068196	0.5	0.014693001	-2.728276493
		0.841	0.734635649	
-2.385166795	0.998936983	0.835	0.185887661	-1.387659316
		1.617	0.056677107	
		1.033	0.605991761	
		0.586	0.025248953	
		1.056	0.960056225	
0.060695723	0.051897784	1.013	0.658683512	0.462575837
0.824506069	0.415113299	1.163	0.315641905	1.030204483
0.270907732	0.088525714	0.307	0.161690518	
3.324734296	0.999999768	1.341	0.030282106	2.331446501
2.157933377	0.995268151	2.179	0.035421634	2.361723005
0.253386273	0.083628912	0.958	0.956337617	0.056702045
1.342432631	0.810160059	1.315	0.378313408	0.906575723
		0.552	0.083761515	
-0.154724149	0.062406189	1.083	0.478671914	0.738217675
-0.618829159	0.25854973	0.671	0.37327212	-0.922402256
1.997308492	0.988003412	1.552	0.011162515	2.826666957
		0.481	0.28440232	
-0.515131303	0.193603429	1.026	0.930135052	0.086663837
		0.914	0.970608723	
1.852013763	0.974511242	1.527	0.072379842	1.973368002
-2.472880637	0.999435274	0.368	0.032084288	-2.487847467

		1.049	0.859434188	
		0.921	0.319571083	
-0.50742373	0.189237696	0.843	0.122925882	-1.585618828
-2.978512991	0.999992056	1.037	0.789884124	0.266123964
2.350570481	0.998647492	0.793	0.056537565	-1.993103282
		0.782	0.517459018	
0.887362286	0.467432017	0.969	0.935996146	-0.082209721
0.003298213	0.050005598	0.793	0.104612114	-1.682789803
-0.220150582	0.075284816	1.211	0.10969447	1.749085302
		0.754	0.571697738	
		1.081	0.663025132	
1.429178159	0.855731495	1.194	0.183588898	1.441266488
		0.716	0.175117229	
0.707437012	0.322270889	0.949	0.554870958	-0.590217174
-0.070293654	0.052546414	0.642	0.060195562	-2.027962527
-0.246809212	0.081879894	0.643	0.11971869	-1.693825996
-1.802715484	0.967695017	0.701	0.461994402	-0.772456177
		2.527		
		0.962	0.38554509	
1.781869157	0.964390691	1.186	0.307809212	
-0.389518613	0.130920152	0.986	0.848550288	-0.192786126
1.513765326	0.892433257	1.598	0.055046352	2.167380756
1.701166018	0.948893997	1.247	0.310728288	1.056490505
		0.977	0.427571816	
1.13867437	0.673186435	1.085	0.681890946	0.416231124
0.331166398	0.108032934	1.002	0.956974543	0.055126335
1.355303167	0.817431539	1.322	0.107107953	1.762842821
		1.25	0.316241193	1.056264095
		1.603	0.064496141	
		1.905	0.055194333	
1.864682329	0.976053673	1.394	0.010292544	2.979684836
		0.245	0.034933998	
-1.968063862	0.985945045	0.51	0.089174584	-1.814074904
0.830063881	0.41969252	0.649	0.016804859	-2.553943139
-1.063046246	0.613760075	0.549	0.018718025	
		0.95	0.961510064	
1.416339677	0.849497021	1.488	0.030100363	2.467656738
-0.553573525	0.216355993	1.222	0.19247291	1.363875835
		0.857	0.965058052	
		0.001	0.001	
		1.918	0.386781294	
0.27484653	0.089674227	0.887	0.593939642	0.564144355
-0.084013592	0.053639772	1.252	0.135271534	1.610029385
-1.714311347	0.951733022	0.731	0.013546139	-2.740185492
-0.114295829	0.056748609	0.543	0.000492524	-4.184452876
		0.77	0.414336502	
0.48204306	0.175333999	0.893	0.836263667	-0.217809115
-0.652985441	0.282300326	1.156	0.259333039	1.174986734

		0.961	0.905555171	
0.404964033	0.137642396	0.806	0.157160334	-1.467829812
-1.89221029	0.979136117	0.973	0.601981059	-0.517466332
1.887207245	0.978602312	1.288	0.182570602	1.451587953
-0.881510259	0.462522492	0.978	0.932311523	0.087991086
-1.809760221	0.968752717	0.837	0.152174672	-1.505132296
		1.824	0.061281747	
		47.472		
		0.596	0.052596515	
2.789638087	0.99995579	1.155	0.175811821	1.419490824
0.826483364	0.416741139	1.224	0.481808939	0.704974634
		1.09	0.905546075	
0.752337019	0.356924786	1.092	0.555004165	0.59500993
1.718709958	0.95265429	1.02	0.928278474	0.089775586
		0.991	0.848263283	
		0.911	0.662019104	
0.685067432	0.305555913	1.194	0.209848175	1.369587282
-0.413569426	0.141508425	1.063	0.394109587	0.897018172
1.898480677	0.97978917	2.002	0.00295942	3.612632878
0.631370354	0.267144008	2.148	0.03429691	
		1.526	0.053957836	
		0.769	0.147631567	
-1.449677275	0.865319793	1.108	0.224366465	1.252333639
		0.926	0.493737094	
0.951821332	0.521673218	1.657	0.030436195	2.311792727
1.718271079	0.952563007	1.282	0.018504082	2.610815252
-0.052284857	0.05140785	1.198	0.396698425	0.869760885
		1.052	0.936600993	
-1.860363752	0.975536939	0.898	0.5135203	-0.663688499
-1.375270106	0.828361947	0.914	0.319155399	-1.031387054
-0.489357624	0.17926617	0.646	0.438579605	-0.801835966
		1.624	0.085819392	
1.221478204	0.733494829	0.983	0.902070476	0.125373716
1.447949472	0.864528951	0.688	0.041389405	-2.158398505
-1.666438921	0.940751614	0.566	0.1442971	
1.303219554	0.786928051	1.075	0.37475708	0.891878311
-0.808608892	0.402081582	1.135	0.534410085	0.637185669
1.739278122	0.956776558	1.203	0.638299619	0.464229194
-1.089251727	0.634741614	0.765	0.163222575	-1.484329632
		0.295	0.074341321	
-1.269834224	0.76589843	1.266	0.014231499	2.75619086
0.39417704	0.132918297	0.931	0.641031249	0.496851588
-1.091992509	0.636914032	0.914	0.775811034	
		0.975	0.973078803	
-1.797126203	0.966834947	1.212	0.415973824	0.802336612
-0.786175865	0.383881448	1.08	0.343938993	1.008823

		1.242	0.278983636	
1.943592867	0.983995204	1.559	0.014531122	2.700973806
0.682826889	0.303903658	0.602	0.332065904	-1.001459678
2.813466069	0.999964107	1.195	0.037223386	2.286541341
		1000	0.001	
		1.639	0.073803439	1.943737219
2.497752518	0.999530693	1.527	0.009976708	3.13464476
0.850215956	0.436384271	0.986	0.978146512	-0.028186976
-1.46656219	0.8728813	0.835	0.052671941	-2.038320176
-0.812319428	0.405114024	0.691	0.39314738	-0.902416816
1.60312804	0.923325612	1.365	0.058222267	2.121119347
-0.630871134	0.266799054	0.733	0.353212561	-0.972809036
0.611157142	0.253366661	1.441	0.110745335	1.653015813
-0.088848452	0.054071779	0.882	0.55857861	-0.601488202
		1.074	0.994566525	
		1.778	0.014719634	
-0.172396687	0.065427172	0.489	0.016382344	-2.612829713
1.462536172	0.871105786	0.874	0.264950122	-1.127701316
2.37037809	0.998821005	1.144	0.424695955	0.820227316
1.880785101	0.977900193	1.201	0.116414821	1.670591234
-1.688466169	0.94602634	1.006	0.836404693	-0.204491017
-0.48741157	0.178214069	0.834	0.278181896	-1.114844558
-0.156620808	0.062714343	1.238	0.454491716	0.753283236
		0.948	0.754827462	
		0.635	0.169913743	
0.578507503	0.231962784	0.876	0.686726642	-0.423651587
-1.39128793	0.836821011	0.861	0.041059244	-2.129195719
0.08223206	0.053486733	0.886	0.327997709	-1.003622297
		2.925	0.091338193	
1.973837725	0.986374019	1.066	0.660256458	0.442594203
		1.037	0.92373463	
		0.725	0.117576319	
		0.504	0.016974743	
1.49762551	0.886008125	1.231	0.127114685	1.643771659
0.599508523	0.245607402	1.148	0.440924243	0.797845432
-0.913659639	0.489544872	0.999	0.666941701	0.458675182
1.444258817	0.862829019	1.179	0.870937754	-0.157000588
0.569111707	0.226004994	1.018	0.947526686	0.065650543
		1.337	0.297487559	
-0.632711486	0.268071867	0.829	0.45355007	-0.761884522
		0.457	0.017723959	
0.358068706	0.118092564	0.931	0.788723609	-0.283341093
		0.001	0.001	
1.417216435	0.849928418	1.081	0.374712854	0.926142699
		1.643	0.045564664	
-1.067206184	0.61711497	0.669	0.165921434	-1.440120034
-0.453129638	0.160389865	0.924	0.595421523	-0.551301502
-0.081740857	0.053445118	0.918	0.507895016	-0.672284874

-0.076531755	0.053019262	1.494	0.065217699	2.019310925
-0.271845541	0.088797576	0.844	0.301720778	-1.065518735
0.319969842	0.104093147	1.114	0.914742273	0.106017351
		0.492	0.151247164	
1.600858494	0.922635212	1.065	0.777007634	0.286755142
0.713172942	0.326618782	1.08	0.717822115	
0.797138762	0.392746078	1.412	0.169424821	1.429303447
		1.021	0.713230052	
-0.502904788	0.186708995	1.004	0.727341041	0.35771642
		0.952	0.921895113	
0.333004368	0.108693509	1.076	0.254003923	1.241990517
		1.144	0.647664962	
0.779771432	0.378730872	1.358	0.00356577	3.46373351
		0.983	0.645640688	
1.887213856	0.978603024	1.005	0.752866938	-0.310411413
-3.337973998	0.999999799	0.764	0.018301998	-2.536309455
		0.961	0.949266608	
2.0525855	0.991188439	0.86	0.310636365	
-0.424212108	0.146409117	0.567	0.002661491	-3.53677329
		0.98	0.770800303	
		1.705	0.037082827	
-0.515016463	0.193537894	1.091	0.68553807	0.401265395
-3.012376386	0.999994252	0.833	0.055664513	-2.04830356
0.639857407	0.273043995	1.216	0.395939694	0.857712438
-1.372949217	0.827113393	0.848	0.593659611	-0.541174855
-1.566272709	0.911520956	1.073	0.486730246	0.715137596
0.051946636	0.051389679	1.051	0.775271218	0.290377631
		1000	0.001	
-0.203344776	0.071531369	1.229	0.077265266	1.886059339
1.535236664	0.900569862	0.904	0.342055992	-0.995256435
0.329780674	0.107537481	1.289	0.618215587	0.502762354
2.442196319	0.999292889	0.604	0.020261293	-2.476489134
		0.927	0.848425016	
		1.005	0.924479608	
0.867193616	0.450537383	0.934	0.541815686	-0.636663316
		0.658	0.0787674	-1.844878229
		0.5	0.0856212	
1.974112683	0.986394162	0.958	0.880572391	
0.596325455	0.243510597	0.873	0.556401376	
2.435448428	0.999257429	1.837	0.016773469	2.745618664
		3.036	0.190906131	
-2.578971468	0.999748061	0.55	0.032769621	-2.311217531
-1.219821339	0.732345565	0.744	0.382601239	-0.89182286
		0.923	0.827135833	
-0.427496921	0.14794833	0.812	0.084913791	-1.811914689
		1.387	0.180542946	
-0.539080972	0.207588992	1.484	0.103562875	1.744292506

		1.426	0.003258887	
		0.814	0.521433466	
		0.001	0.001	
-0.260159163	0.085480754	0.919	0.393749258	-0.868577743
		0.955	0.886551627	
		1.186	0.32112618	
-0.082453553	0.05350558	1.209	0.069650163	1.976436172
-1.10962368	0.650780665	0.999	0.582425932	0.581561332
1.073163815	0.621904265	0.639	0.083973631	-1.77787248
1.354676138	0.817081377	2.223	0.005667192	3.112987193
		1.178	0.613465114	
1.015420096	0.574800183	1.084	0.439753795	0.811889736
-2.21914464	0.99676955	0.753	0.1152686	-1.72988475
2.113091626	0.993801002	1.455	0.07922173	1.902659195
-0.420399758	0.144638456	0.991	0.541410743	0.628201896
		1.136	0.384268065	
0.756398677	0.360125337	2.305	0.003897163	3.391467225

A/N Power	A/M Ratio	A/M P value	A/M Effect_size	A/M Power
	1.276	0.029821408		
0.999999441	1.493	0.032021864	2.325802354	0.998397767
	0.503	0.055798959		
	0.001	0.001		
0.37100444	1.374	0.219912927	1.29086007	0.779274547
	1.082	0.63565852	0.480888191	0.174718731
	0.275	0.000696401		
0.109645619	1.108	0.489242753	0.686826354	0.306855864
0.537086895	1.707	0.028178658	2.346932107	0.998613189
0.058780564	0.872	0.19460193	-1.341852267	0.809828019
0.061056847	1.126	0.279918012	1.099947721	0.643194308
	1.146	0.991855051		
	0.941	0.700386026		
0.992445343	0.923	0.525337553	-0.636643695	0.270802036
0.094087727	1.869	0.213255867	1.279111575	0.771855216
	0.334	0.381445391		
	1.494	0.396381565		
0.05934977	1.081	0.965953541	0.042040713	0.050909947
0.616136038	1.172	0.150856875	1.539763781	0.902226346
	1.656	0.471011751		
0.159716888	1.332	0.22114778	1.282827508	0.774216955
0.609441721	1.015	0.862587536	0.177583472	0.06637768
0.196024171	1.231	0.16348092	1.4136714	0.848179044
	1.114	0.766004225		
0.993625042	0.825	0.167735437	-1.425442889	0.853935879
	1000	0.001		
0.291799952	0.898	0.152659037	-1.471431649	0.875005908
	0.071	0.069792658		
	0.542	0.032341812		
0.447819693	0.892	0.359647437	-0.915709022	0.491270396
	0.835	0.584743495		
	0.995	0.722423285		
	0.754	0.663372332		
	6.187	0.014722966		
0.985404037	0.923	0.47558163	-0.718628391	0.330776565
0.532404536	0.995	0.920148739	0.102313301	0.055403601
	0.736	0.687739829		
0.999993004	0.448	0.005746875	-3.403477882	0.999999903
	1000	0.001		
0.815102364	0.497	0.403449771	-0.874005661	0.456235068
	0.269	0.00119179		
0.849935242	0.866	0.199279527	-1.267080206	0.764113652
	0.968	0.955475693		
	0.796	0.360797545		
	2.132	0.156678235		

	0.897	0.887784221		
	1.653	0.818545582		
0.856603162	1.247	0.742053872	0.325326002	0.105959787
	1.054	0.655604748		
	1.228	0.422381571		
	1.37	0.250259852		
	0.223	0.391287668		
	1.357	0.826042851		
0.810638257	0.659	0.008826706	-2.965535621	0.999991019
	0.579	0.01253797		
0.083006041	1.022	0.940869873	0.074971847	0.052897229
0.161765593	1.047	0.773559738	0.287409628	0.093455114
0.999527017	0.672	0.026079087	-2.556333722	0.999699545
0.995371761	0.824	0.308091934	-1.05471212	0.607013247
	1.102			
	0.96			
0.173801543	0.998	0.870304614	0.167387234	0.06453673
0.096649448	1.307	0.158973159	1.547107924	0.904870342
0.051171564	1.191	0.522017008	0.635294412	0.269863595
0.976509924	1.212	0.127403617	1.58601136	0.918001548
0.951163964	0.645	0.038539037	-2.14602604	0.994912346
0.094562713	0.894	0.354351639	-0.927260825	0.500998816
	1.853			
	0.845	0.820060075		
	2.581	0.005102983		
0.082422926	0.985	0.500107204	-0.654709831	0.283527679
0.766130432	0.884	0.527361798	-0.636477399	0.270686284
	1.536	0.215121589		
0.579866766	0.631	0.012139942	-3.081556094	0.999997075
	0.878	0.336412178	-0.952260926	0.522042887
	1.461	0.148419671	1.536421069	0.901005211
	0.194	0.090036521		
	0.615	0.633443076	0.503752911	0.187181839
0.99399357	0.863	0.697366419	-0.410394264	0.140071908
0.358587864	2.81	0.81488554	0.23189367	0.078092581
0.1133387	1.045	0.851098237	0.190144168	0.068800251
0.085111862	1.193	0.455288912	0.746783356	0.352565137
0.411570142	0.454	0.522481154	-0.678969073	0.301068413
	1.084	0.850377691		
	0.335	0.230749834		
	0.821	0.37986384		
	1.068	0.758135353		
0.618492962	1.613	0.058629712	2.05709134	0.991411894
0.420554422	0.99	0.815476906	-0.232808583	0.078317759
	1.007	0.851331352		

	0.879	0.777349345		
	1.11	0.344657118	1.010167511	0.570450479
	1.47	0.237311946		
0.895685021	0.82	0.084040716	-1.871940219	0.976901417
0.40538204	1.147	0.368679211	0.904800912	0.482088739
0.744404778	1.43	0.073391444	1.904013002	0.980350868
0.997726082	0.637	0.024288373	-2.417120354	0.999152678
0.052048119	0.676	0.42502628	-0.84995119	0.436164155
	1.2	0.246549511		
	1.309	0.342499007		
0.153803668	0.84	0.82874571	-0.22166044	0.075637269
0.346221505	0.831	0.014984681	-2.683299167	0.999891115
	1.115	0.357549965		
0.986133498	0.986	0.710605744	-0.369977381	0.122814172
	1.148	0.769504759		
0.495605534	0.94	0.394277523	-0.849377222	0.43568705
	0.736	0.376336196		
0.933876014	0.99	0.800063398	-0.252244074	0.083321691
	1.26	0.295329318		
	0.836	0.309350392		
0.299609261	1.507	0.030596677	2.270117234	0.997676103
0.872251232	0.933	0.625710396	-0.502000512	0.186205737
	1.013	0.562955422		
0.629892965	0.904	0.297183124	-1.064655162	0.615058658
	1.254	0.356462747		
0.467317991	1.086	0.5484409	0.599312951	0.245478279
	1.632	0.738288408		
	0.887	0.582174878		
	2.159	0.225737912		
	1.446	0.754468943		
0.99997574	1.186	0.089286371	1.815115879	0.969537495
0.346802794	1.657	0.163117305	1.423134465	0.852818687
0.986494128	0.972	0.89236015	-0.138282881	0.05989599
0.113597271	0.629	0.195926899	-1.367956984	0.824408154
0.985156803	1.317	0.044992725	2.108093267	0.993614735
0.96606036	0.856	0.355916236	-0.952666253	0.522383724
	0.836	0.794222524		
	0.48	0.010885132		
0.420737499	1.152	0.654567262	0.451723254	0.159687501
	1.494	0.240252514		
0.42644432	1.093	0.620464777	0.482612693	0.175638037
0.051893696	0.853	0.417349052	-0.834220093	0.423124168
0.698855994	1	0.975104211	0.031797368	0.050520424
0.926878355	1.395	0.066436736	2.009049201	0.988753035
0.992662978	1.174	0.317568323	1.04233624	0.596935116
0.996409239	1.699	0.011071186	2.923176887	0.999986662

	0.605	0.755172133		
	0.938	0.93952333		
	0.796	0.229644677		
0.874715959	0.427	0.014344731	-2.970566126	0.999991436
	0.88	0.538969456		
	1.01	0.790300309		
0.58529605	1.486	0.010419798	2.837575241	0.999971
0.999600099	1.17	0.402911474	0.840222484	0.42809051
	1.044	0.994312379		
0.644339954	0.992	0.796499151	-0.255312762	0.084150405
	0.714	0.708558401		
0.999997465	0.552	0.009561898	-3.167380623	0.99999877
	2.698	0.254232547		
0.201978052	1.07	0.671431828	0.41603198	0.142630634
0.050272342	0.93	0.49839913	-0.686310724	0.306474522
	1.349	0.097530004	1.752689408	0.959304565
	0.585	0.555078924		
	1.17	0.51369758		
0.983754047	1.384	0.069438168	1.950078971	0.984533382
	2.109	0.162968298		
	0.745	0.317767534		
0.699286867	0.683	0.03170617	-2.249149959	0.997335535
0.739654095	0.92	0.420694067	-0.814305699	0.406739694
0.108981046	1.042	0.537622567	0.649573323	0.279879533
	0.869	0.539776367	-0.637672046	0.271518399
0.808405065	0.859	0.04256495	-2.139650174	0.994712189
0.904098534	1.018	0.934262788	-0.081353402	0.05341247
	2.586	0.061247097		
0.064056868	1.036	0.880842469	0.148092135	0.061358929
0.240792461	1.225	0.223284561		
0.070835002	1.222	0.183563341	1.40213363	0.842391824
0.594844318	0.848	0.787794354	-0.280106024	0.09123526
0.999600576	1.096	0.53310053	0.624779942	0.262609056
0.23851758	0.85	0.420118896	-0.823036654	0.413904533
	0.907	0.665521854		
0.979469664	0.541	0.122047443	-1.685313028	0.945294945
0.168078862	1.148	0.681688299	0.404050019	0.137236844
	1.368	0.653979837	0.446691717	0.157193425
0.98586418	1.022	0.656956391	0.460362216	0.164037956
	0.742	0.092436979		
0.88484379	1.088	0.366924657	0.939488419	0.511295541
	1.187	0.584351921		
0.539880195	0.82	0.348772822		
0.994903517	0.871	0.220183343	-1.250553713	0.753247044
	0.591	0.000758087		
0.999999915	1.08	0.499389282	0.693402723	0.311738241
	1.299	0.019684683	2.551612731	0.999688388
0.791124335	1.08	0.969045443	-0.038191858	0.05075089
0.988792395	1.084	0.378034281	0.925985263	0.499924519
0.976983958	1.173	0.407972738	0.826943455	0.417120125

0.999944414	1.712	0.225191948	1.212013181	0.726896023
	1.359	0.11661925	1.702916965	0.949279662
0.547978521	0.944	0.490060777	-0.695266522	0.313128202
	1.754	0.097472061		
	0.942	0.893013238		
0.860184134	1.076	0.942040504	0.072211074	0.05268745
0.999948035	1.324	0.032912983	2.404339446	0.99907176
0.335476079	1.036	0.988917735	0.014079376	0.050102008
	0.787	0.221611635		
0.350477352	1.228	0.103265137	1.715135819	0.951906788
0.169584598	1.125	0.568062867	0.568451536	0.225589851
	0.988	0.96006073		
0.259697006	1.226	0.303115972	1.073116066	0.621865954
	0.001	0.001		
0.296708103	0.72	0.748482266	-0.333088679	0.108723905
0.983014164	1.297	0.048061713	2.132190868	0.994469147
0.999933434	1.466	0.139017376	1.600939055	0.922659799
	1.204	0.481591331	0.725213036	0.335823489
	1.738	0.05885523		
	0.97	0.418137224	-0.789435197	0.386510754
0.26961132	0.841	0.362391898	-0.9206068	0.495394792
0.187916776	0.803	0.15542337	-1.454059421	0.867311317
0.36524508	0.956	0.580937584	-0.575473002	0.230028592
0.23554237	1.401	0.066242072	1.977251326	0.986622279
0.998578373	1.343	0.041924659	2.240858509	0.99718885
	1.004	0.805516009		
	1.004	0.913774904	-0.108312349	0.056058125
0.434618042	1.068	0.47395878	0.725673343	0.336177448
	1.54	0.954177337		
0.980622331	1.045	0.813943203	0.238584207	0.079760746
	0.744	0.940049399		
0.102254415	1.126	0.181764183	1.357665182	0.818746828
	0.527	0.185268748		
	0.934	0.622336729		
0.066471732	1.135	0.955061046	-0.055459608	0.051584176
	1.062	0.65369277		
0.99776607	0.913	0.815633802	-0.23803397	0.079621673
	1.064	0.722253145		
	0.798	0.413261584		
0.138700109	1.181	0.174959306	1.41339402	0.848041594
0.999956045	1.061	0.502820277	0.704367722	0.319954474
0.771341226	0.726	0.057024317	-1.9801771	0.986831939
0.178830995	0.976	0.68318268	-0.403978964	0.137205358
0.999914018	1.349	0.103662006	1.714867516	0.951850296
	2.372	0.01501644	2.777743412	0.999950987
	1.334	0.253827492		
0.52496634	0.982	0.721068775	-0.35679085	0.117595721
	0.145	0.105216176		

0.371579874	1.055	0.710971267	0.36978152	0.122735181
	0.705	0.12168231		
0.995174786	0.691	0.050130538	-2.055921577	0.991354366
0.9993756	0.995	0.896461245	0.134450865	0.059352293
	1.274	0.279897585		
	0.75	0.55622604		
0.999992656	0.675	0.014172743	-2.678227663	0.999886466
0.452484489	1.168	0.908294815	0.114286426	0.056747494
	1.031	0.961141839		
0.123679037	1.251	0.20632282	1.325387726	0.800259818
0.982710944	1.395	0.887526209	0.141157543	0.060314099
0.074583567	1.736	0.342689649		
	2.069	0.000768312		
0.983257575	1.108	0.374794909	0.913429153	0.489350821
	1000	0.001		
0.425454153	0.645	0.988743494	0.01442842	0.050107128
0.23038074	1.248	0.199726095	1.299481582	0.784629868
	0.775	0.255919403		
0.989699	0.254	0.000727057	-4.219626075	1
0.46125915	0.595	0.010255321	-3.153524738	0.999998582
0.502532625	1.128	0.278374759	1.124258083	0.662139734
0.339946898	1.613	0.516342407		
	0.388	0.004175422		
0.998657315	1.26	0.304080417	1.073351469	0.622054818
	0.947	0.715781624	-0.367508535	0.121821741
	0.885	0.394449319		
	1.489	0.160424697		
	0.881	0.825040021		
0.062246796	0.987	0.888200568	-0.142491513	0.060511107
	1.131	0.670160381		
0.992844894	0.933	0.600961316	-0.533692516	0.204387419
0.063545184	0.992	0.803835809	-0.247702792	0.082114676
0.052292461	1.039	0.819301249	0.228522052	0.077270787
0.940718374	1.733	0.328749753	0.983242016	0.548029174
	6.301	0.315982606		
	0.191	0.160858638		
	0.959	0.927718298		
	0.666			
	0.891	0.513516214		
0.998995928	0.675	0.012368588	-2.695439242	0.99990153
	0.978	0.774107751		
0.057088021	0.849	0.108302797	-1.710390821	0.950899835
0.867663829	0.971	0.863253496	-0.175668427	0.066023351
	1.091	0.915965721		
0.138778487	1.012	0.863496416	-0.172180621	0.065388208
	0.682	0.273307716	-1.127108973	0.664335807
0.403977886	1.067	0.715461387	0.365187327	0.120895134
0.092488169	1.214	0.182402849	1.410827957	0.846766112
0.919297611	1.005	0.801649529	0.25893344	0.085141793
	0.879	0.458508514		

	1.268	0.218699905	1.32886486	0.802304339
0.99999898	0.888	0.163020518	-1.438263776	0.860036674
0.87443704	1.529	0.313295546	1.024715148	0.582474752
0.97698267	1.027	0.866626187	0.170524591	0.065091236
0.101541353	0.864	0.936308878	0.083743336	0.053616344
0.98069281	1.056	0.714271135	0.369719423	0.122710146
	0.951	0.707208346		
	1.446	0.006182573		
0.096646133	1.122	0.751879399	0.312349263	0.101494326
	0.74	0.552362584		
0.14803012	0.916	0.339008475	-0.95252753	0.522267075
0.108190379	1.015	0.837497599	0.208560668	0.07266306
0.385847003	0.98	0.901258256	-0.125798777	0.058181988
	1.186	0.414614165		
	1.114	0.940085802		
0.171906075	1.727	0.086164014	1.838437802	0.972766746
0.905187536	1.307	0.146819386	1.502591243	0.888013463
0.121166778	0.889	0.164516428	-1.431760268	0.856964013
	1.121	0.375358711	0.93283477	0.505693079
0.203311807	0.532	0.599684991	-0.549349323	0.213777265
	0.436	0.097296158		
0.927725636	0.904	0.56026968	-0.596050231	0.243329774
0.955800187	0.725	0.01843874	-2.570410403	0.999730644
0.999993954	1.488	0.054424494	2.015896217	0.989171117
	0.952	0.616511814		
0.0679399	1.67	0.419442554	0.802729601	0.397288998
0.928859131	1.097	0.219656125	1.276852529	0.770412652
	0.902	0.792146656		
0.088030884	1.078	0.540116024	0.616261204	0.256808512
0.653989699	1.041	0.575476445	0.572639182	0.228230951
0.741021756	1.038	0.774349436	0.289926	0.094234005
0.642101084	0.99	0.891286899	0.140363683	0.060197756
	0.737	0.111719282		
0.08173261	0.785	0.205430233	-1.330799988	0.803436663
0.052880907	1.346	0.200497035	1.360543201	0.820341393
0.069468538	2.183	0.082623363		
	1.696	0.030250901		
	0.393	0.417695136		
0.983095188	1.311	0.012572569	2.803565221	0.999960849
0.693895144	1.844	0.09736998	1.764211005	0.961379217
0.99982807	0.985	0.99164652	0.010729066	0.050059235
0.763038504	1.141	0.576165205	0.559028419	0.219714246
0.377797278	1.605	0.395803467	0.853230472	0.438891806
0.053547733	1.025	0.543175031	0.640010939	0.273151341
	2.944	0.028974602		
0.996922006	0.722	0.051131803	-2.015589003	0.989152653

0.724843972	1.282	0.517400379	0.644968188	0.276628935
	0.3	0.264355179		
0.051200103	1.443	0.808530222		
0.181701328	1.145	0.361266727	0.932360081	0.505293328
	1.037	0.75594622		
0.183247263	1.427	0.576282203	0.559670132	0.220111393
	7.735	0.101649133		
	0.968	0.946398413		
0.28814862	1.217	0.517797495	0.666308267	0.291850927
	0.644	0.592004209		
0.997674831	1.362	0.05889972	2.064139037	0.991751432
0.085451373	1.094	0.529676687	0.644334009	0.276182795
0.864695925	0.914	0.835053148	0.220290072	0.075317272
	0.998	0.818950973		
0.088753656	1.037	0.959545383	0.050443404	0.051310352
0.265178583	1.372	0.034118524	2.373875995	0.998849439
0.897328353	0.959	0.544649603	-0.603387284	0.248176165
0.312286355	0.712	0.139421212	-1.532278462	0.899476406
0.680262771	0.974	0.916662045	0.106798851	0.055889432
0.853060081	0.726	0.026456095	-2.383898241	0.998927462
0.431725486	1.404	0.266317577		
0.965696098	2.304	0.620180111	0.490539129	0.179907027
	1.095	0.784674403		
	0.691	0.105357796		
0.123687325	1.233	0.184029261	1.397370944	0.839961108
	2.049	0.588975545		
0.076463942	1.87	0.007010345	3.066678316	0.999996612
	2.206	0.53883508		
0.999989427	2.52	0.00474449	3.17157338	0.999998822
0.999971497	1.292	0.358685541	0.920424453	0.495241227
	1000	0.001		
	0.891	0.975411109		
	0.361	0.595634976		
0.703534125	1.165	0.798801025	0.257725896	0.084809514
	0.892	0.447954421	-0.763993245	0.366136494
0.999993483	0.558	0.05647239	-2.122896573	0.994152451
0.452979842	0.742	0.019778871	-2.536199367	0.999649219
	1.229	0.476924391	0.73509379	0.343453621
0.979362087	1.327	0.072022713	1.941897316	0.983851897
	0.821	0.322835037		
	0.805	0.25737714		
0.535171309	0.945	0.827067951	-0.225450263	0.076533051
	0.648	0.513532114		
	0.404	0.80707425		
0.337993891	0.129	0.076034098	-1.976971588	0.986602082
0.149871366	1.096	0.566760472	0.577885801	0.231565734
0.053643128	1.134	0.097913131	1.763681267	0.961285758
0.382647273	1.267	0.433892036	0.808280917	0.401813828
0.185840905	1.148	0.9549487	0.056336482	0.051634716
0.936305825	0.793	0.191708758	-1.303583009	0.787150743

0.981320362	0.678	0.000359153	-4.268633639	1
0.161153951	0.754	0.137470685	-1.527027226	0.897513767
	0.945	0.882183789		
	1.05	0.6908407		
0.994803866	1.073	0.55934389	0.601042945	0.246621803
	1.055	0.652453466		
0.954872963	1.101	0.434814214	0.801548482	0.396328061
	1.053	0.83469943		
0.999995448	0.482	0.017076625	-2.75045322	0.999938038
0.818115526	1.309	0.019875247	2.639643197	0.999844478
	0.73	0.133147832		
0.792407611	1.189	0.209526862	1.272434354	0.767576574
0.798118843	0.835	0.144760871	-1.45841267	0.869269494
	0.803	0.660610491		
0.058246801	1.045	0.753274822	0.320410845	0.104245588
0.053148114	1.122	0.124536507	1.639480506	0.933754762
0.999999995	0.932	0.476938958	-0.724300615	0.335122311
	0.876	0.94064934		
0.83324135	1.02	0.559145405	-0.56655153	0.2243976
	1.437	0.113127775		
0.978676634	10.698	0.006792087	2.978171089	0.999992031
0.050798487	0.342	0.250519455	-1.211916935	0.726828507
0.99998019	1.135	0.271855675	1.148593917	0.680700621
0.768313138	0.887	0.22799952	-1.21120034	0.726325564
0.055023787	0.826	0.074689709	-1.856551065	0.975072981
	0.974	0.544133739		
0.968646199	0.667	0.008213126	-3.313909834	0.999999739
0.840626147	1.43	0.232915562	1.244177579	0.748983816
0.995554601	0.486	0.004905033	-3.120520947	0.999998018
	0.963	0.634197388		
	1000	0.001		
0.082600023	0.793	0.113174239	-1.728612332	0.954676683
0.130495362	1.275	0.261922721	1.20392781	0.721195472
	1.218	0.858515475		
	0.745	0.225570813	-1.246426149	0.750491708
0.948310267	0.941	0.752343643	-0.325112088	0.105884603
	1.021	0.720454984		
	1.34	0.55752056		
0.925347768	1.108	0.581239425	0.54744236	0.212619411
	1.059	0.71162661		
0.757901838	1.261	0.413834208	0.812282274	0.40508363
	1.003	0.890948518		
0.07015373	1.305	0.694205008	0.390108962	0.131171969
0.961798529	1.63	0.283115987	1.094278238	0.638722388
	0.928	0.778075722		
	0.774	0.765828858		

0.985166596	1.05	0.960477464	0.048955732	0.051234147
0.128186832	1.125	0.6225916	0.486302332	0.177616304
0.999515782	1.253	0.154528531	1.511409528	0.89151208
0.999999992	0.591	3.36697E-05	-5.376709205	1
	0.869	0.692404383		
0.056770378	0.748	0.058527171	-2.025343348	0.989725703
0.051141891	1.005	0.924699356	-0.094438551	0.054601682
	0.67	0.132109135		
	0.523	0.61005783		
	1.244	0.859328563		
	0.18	0.067947492		
	1.717	0.004732803		
0.087538293	1.019	0.70715979	0.390787946	0.131462098
	0.65	0.051766106		
	0.618	0.865197852		
0.998706725	0.873	0.242895773	-1.178994995	0.703259692
	1.193	0.327071155		
0.193473326	0.861	0.042960658	-2.173585025	0.995701995
0.995522552	0.916	0.590737729	-0.544892863	0.211077552
0.504529839	0.99	0.877818302	-0.153869991	0.06226867
0.999815145	0.565	0.029402426	-2.275712558	0.997760059
0.998335418	1.119	0.52905645	0.634385366	0.269232297
	1.587	0.490630942		
	7.311	0.205869162		
0.350400346	1.055	0.606928213	0.508909579	0.190074147
0.998758268	1.119	0.120039654	1.605205156	0.923953354
	0.84	0.22209202		
0.290926381	1.134	0.277271436	1.162647002	0.691219126
0.054691435	1.448	0.212168319	1.346046679	0.812219695
0.998530322	1.094	0.445078766	0.771978306	0.372492953
0.99999568	1.308	0.032389628	2.39885139	0.999034881
0.057107897	1.142	0.177185101	1.393045351	0.837732299
	0.735	0.084981519		
0.971963585	2.322	0.002170873	3.547535351	0.999999982
0.694108567	1.085	0.845564928	-0.199970873	0.070815216
	0.189	0.230613575		
0.421167005	1.033	0.839465521	0.205421106	0.071978294
0.080785364	1.096	0.685255998	0.406538163	0.138343129
	1.143	0.84174166		
1	1.296	0.130113722	1.627354297	0.930405666
	1.453	0.442087103		
0.052484153	1.374	0.525847052	0.639189166	0.27257703
0.910484141	1.125	0.272350367	1.16802134	0.6952011
0.14741381	1.095	0.743298153	0.324899938	0.105810091
	0.94	0.547623599		
0.860540875	0.929	0.408900336	-0.846400644	0.43321433
0.257005217	1.158	0.185892794	1.363038951	0.821716987

0.993539695	1.282	0.237766881	1.186476116	0.708696771
	0.871	0.72285768		
0.100030746	1.052	0.430300097	0.81353464	0.406108424
0.051254878	0.957	0.808804506	-0.24821337	0.082249228
	0.282	0.080465293		
0.69859689	1.035	0.689311391	0.414115849	0.141756825
	1.428	0.476122614		
	1.923	0.224494095		
	0.094	0.261063247		
	1.629	0.384732558		
	0.886	0.42366856	-0.794493967	0.39060212
0.456144107	1.035	0.839380584	-0.204709095	0.071824503
0.884754095	0.799	0.021721799	-2.485114934	0.999484253
0.438672989	1.229	0.229984822	1.237218893	0.744286849
	1.523	0.103384471		
0.161104876	0.986	0.841252099	0.206785453	0.072274541
0.088730948	0.956	0.511143258	-0.643373526	0.275507803
	1.361	0.860992828		
0.110553037	1.173	0.305487915	1.045639256	0.599631526
0.674815745	1.097	0.319108477	1.020948753	0.579368617
0.855061986	1.085	0.39512286	0.871352842	0.454015069
0.050458194	0.681	0.167501385	-1.453786684	0.867187964
0.215303688	1.244	0.460372215	0.748216071	0.353687965
	1.41	0.116278687		
0.072089424	1.051	0.651571862	0.466328261	0.167092577
	1000	0.001		
	1000	0.001		
0.105362512	1.15	0.692094871	0.385482979	0.129209614
	1.11	0.661957558	0.443581143	0.155666207
0.391699038	0.936	0.952090013	-0.062164614	0.051990861
0.469841177	1.228	0.134763725	1.626485224	0.930160715
	0.883	0.616615446		
0.130097037	0.766	0.175916351	-1.452476189	0.866594154
0.834350862	0.889	0.290362308	-1.104582041	0.646835129
0.404058309	0.815	0.342670795	-0.949700652	0.519889605
0.802315065	0.535	0.00472372	-3.09842444	0.999997527
0.950094169	0.983	0.803053257	-0.250186097	0.082771843
	1.201	0.222601205		
	0.865	0.739398374		
0.052266824	1.478	0.832464212	0.207433843	0.072416044
0.996562987	0.908	0.65983903	0.465994473	0.166920596
0.992094104	1.139	0.445118261	0.790312635	0.387219493
0.990384552	1.08	0.459898574	0.775602945	0.375390141
	0.985	0.533270877		
0.615565455	0.894	0.32044285	-0.969185477	0.536257741
	0.7	0.18103969		
0.8001449	0.976	0.795872423	0.269272363	0.088054015
	0.532	0.024085714		

	2.071	0.600599133		
0.746839864	0.912	0.429052613	-0.813465881	0.406052142
	1.067	0.739805486		
	1.317	0.083154373		
0.251522219	0.783	0.314851883	-1.037848248	0.593263937
0.289582927	1.177	0.120064328	1.706303624	0.950019023
0.349563636	0.934	0.988305766	0.015489836	0.050123471
	0.566	0.00324331		
0.899414504	0.884	0.349904631	-0.955421158	0.524699854
	0.972	0.991559377		
0.99993302	1.233	0.183322187	1.37951956	0.830632996
0.0732695	1.03	0.724485344	0.36202538	0.119643007
	0.959	0.72103186		
0.098723901	0.916	0.484918388	-0.705572232	0.320862676
	1.538	0.354850656		
0.332713972	1.117	0.489871275	0.690709358	0.309734476
	2.23	0.502240089		
	5.231	0.157858636		
0.999998165	0.831	0.196948471	-1.337407906	0.807273452
	1.358	0.57409112		
	0.456	0.293766765		
	0.886	0.826900952		
0.399135733	0.953	0.681904023	-0.410909248	0.1403041
	1.249	0.762250677		
0.322751303	1.158	0.437182731	0.781814014	0.380371234
	0.759			
0.999766237	0.903	0.602733952	-0.531829399	0.203287823
0.871803756	1.069	0.630840095	0.488060798	0.178564585
0.999598106	1.121	0.336149343	1.009073273	0.569543231
0.090149742	1.157	0.145450014	1.52986491	0.898577783
0.408056848	0.942	0.831117092	-0.220658587	0.075403121
	0.957	0.603354637	0.548306113	0.213143372
	1.405			
0.086004865	1.335	0.036191919	2.259459212	0.997508253
0.179497197	0.941	0.833115683	-0.216150235	0.074363168
0.982804206	0.542	0.004245582	-3.170903434	0.999998813
0.074200827	1.041	0.933483094	0.082968529	0.053549597
0.699653365	1.05	0.850134586	0.188491608	0.068471733
	1.192	0.49299574		
	0.821	0.046912175	-2.187982312	0.996069275
	1.395	0.709858734		
0.229897216	0.827	0.280338914	-1.126990214	0.664244437
0.956953528	1.009	0.881662506	0.151972273	0.061965932
	0.312	0.150152815		
	0.001	0.001		
0.196239683	1.1	0.562775291	0.565159584	0.223526566
0.91222117	0.956	0.579692578	-0.554142307	0.216704675
0.998972775	3.285	0.001793476	3.753687548	0.999999999
0.051496458	1.05	0.652569511	0.462035381	0.164890486

0.989283578	0.862	0.209336117	-1.306012597	0.788635871
	0.501	0.0192202		
0.996640695	0.73	0.028019371	-2.405654362	0.999080401
0.999999997	0.415	0.000986088	-3.838074791	1
0.192158511	0.958	0.625913109	-0.485029397	0.176932046
0.980552175	0.878	0.341709606	-0.981993562	0.54698524
0.315753073	1.366	0.134899055	1.534822193	0.900417186
	0.936	0.407840786		
	0.951	0.79386522		
0.999739071	1.026	0.764481004	0.307306882	0.099811456
0.120381542	1.14	0.612625099	0.506844014	0.188912019
	0.998	0.65607958		
	0.36	0.237543862		
	1.046	0.926872445		
0.999999969	1.202	0.027891208	2.445490462	0.999309626
0.798791681	1.597	0.1766877	1.374021034	0.827690711
	1.167	0.914897693		
	2.152	0.293552656		
0.743411971	0.906	0.293276954	-1.064468372	0.614907963
0.963443391	0.789	0.880184809	-0.159007885	0.063107655
0.772717499	0.91	0.111458081	-1.654946695	0.93784332
	0.963	0.856326504		
	1.12	0.712594479		
	1.191	0.65685235		
	1.075	0.482076556		
	0.545	0.019572391		
0.559947297	0.938	0.418110499	-0.796711841	0.392399776
0.556556487	1.094	0.720752623	0.36618147	0.121291219
0.189824334	0.932	0.564160994	-0.5766629	0.230785896
0.312554757	1.03	0.857400584	0.181270359	0.06707102
0.058379438	1.155	0.150504291	1.520017249	0.894850517
	1000	0.001		
	1.056	0.86358413		
	0.819	0.817715759		
	1.384	0.308622122	1.045126159	0.599212971
	0.727	0.788842581		
	3.535	0.009199898		
	1.251	0.715397089		
0.120279253	1.014	0.806354396	-0.240954302	0.080363647
0.283257201	1.104	0.457643105	0.756796305	0.360439206
0.997559008	0.908	0.526906632	-0.658620452	0.286320861
0.999913504	1.326	0.046988328	2.194176948	0.996218454
	1.922	0.026997573		
	0.755	0.355674928		
0.059480535	1.088	0.661172469	-0.439773943	0.153812223
0.99951242	0.831	0.116980464	-1.610330995	0.925485702
0.395529158	0.92	0.397802413	-0.878667209	0.460139359
	0.656	0.211596703		

0.996510175	1.169	0.239415572	1.267486169	0.764377214
	1.37	0.03197149		
0.558303218	0.746	0.197357418	-1.349603884	0.814233372
	0.736	0.044116879		
0.991505411	1.006	0.814783774	-0.240035971	0.0801293
0.999771029	1.1	0.687954623	0.412828269	0.141172057
	1.481	0.055520012		
0.052121138	0.948	0.489127024	-0.686585725	0.306677877
0.231507456	0.943	0.829820233	-0.219258858	0.07507784
	1.098	0.707622979		
	0.001	0.001		
	0.001	0.001		
0.551730515	1.224	0.49924037	0.686744523	0.306795331
	2.246	0.543457212		
	0.925	0.704890606		
0.530811242	0.941	0.760083785	-0.31690273	0.10303914
0.836582248	0.88	0.389334843		
	1.013	0.909527282		
0.969006532	0.529	0.0104646	-3.201369225	0.999999134
	0.546	0.020496564		
0.342928743	2.117	0.439499642		
	5.363	0.009248252		
0.707550485	1.718	0.20355811	1.285319571	0.77579304
0.999995404	2.05	0.000507206	4.431716346	1
	0.996	0.99716287		
0.176869589	1.06	0.639874501	0.463574731	0.165677678
0.107070247	0.813	0.485345482	-0.722570249	0.333794172
	1.167	0.81891488		
0.228411245	1.007	0.836751544	-0.20587121	0.072075801
0.779054864	0.996	0.987434437	-0.01591777	0.050130388
	1.937	0.162186604		
	1.211			
	1000	0.001		
	1000	0.001		
	2.089	0.080800873		
0.980016852	0.766	0.192501772	-1.329327755	0.802575557
0.611058217	0.935	0.543554817	-0.606917375	0.250526997
	0.976	0.89761818		
0.609616464	0.978	0.999883223	-0.000149633	0.050000012
	0.305	0.194606723		
	1.115	0.398077835		
	1.365	0.477754908		
	1.224	0.172674431		
	0.932	0.464339892		
0.767994067	1.062	0.869224645	-0.163003193	0.063779631
	1.331	0.522221113		

0.138637764	1.255	0.180587741	1.425033698	0.853738263
0.56563984	0.919	0.174768784	-1.39491972	0.838700562
0.92618813	1.154	0.344753812	0.971727498	0.538389196
0.112577116	1.079	0.487078131	0.734057341	0.34265012
0.892598532	0.778	0.069078065	-1.939278224	0.983628369
0.050354835	0.938	0.64540823	-0.487075482	0.178032805
0.766081248	0.728	0.685130254		
0.80299978	1.008	0.899323762	0.128869685	0.058588291
	0.645	0.021032676		
	1000	0.001		
0.999995642	0.647	0.000230146	-4.580903087	1
	1.164	0.926447203	0.090903102	0.054262752
0.373090151	1.31	0.866504528	0.167367725	0.064533315
	1.194	0.474202263		
0.432967429	0.958	0.516679346	-0.643053057	0.275282775
	1.646	0.02177124	2.579949615	0.999749983
	0.987	0.806255768		
	1000	0.001		
0.999897643	1.43	0.063404452	1.973068699	0.986317545
	1000	0.001		
	0.001	0.001		
0.073362964	0.865	0.140890072	-1.52514079	0.896801946
	0.001	0.001		
	0.682	0.084751022		
0.652403749	0.91	0.612667471	-0.517893079	0.195183874
0.524483583	0.87	0.310141629	-1.016303573	0.575530913
0.082998004	0.936	0.493762334	-0.692816879	0.311301905
	1.389	0.052029152	2.123578599	0.994176227
0.774148731	1.111	0.393070545	0.878500101	0.459999328
0.99817859	1.173	0.051161814	2.163865517	0.995436982
	1.108			
	1.808	0.03707272	2.248781472	0.997329166
	1000	0.001		
0.671672515	0.769	0.201226948	-1.325674974	0.800429199
	0.913	0.674383389		
0.630182544	0.878	0.442139962	-0.784670729	0.382669075
	1.602	0.250069953		
0.426824701	1.087	0.616215876	0.50489938	0.1878223
0.992921451	0.74	0.151642764	-1.522554662	0.895820271
0.999711563	1.149	0.257835779	1.177101323	0.701876063
	0.83	0.283490558		
	1.189	0.555592105		
0.514482354	1.237	0.19487482	1.364708621	0.822633544
0.069091383	1.032	0.947694255	0.065962526	0.052241886
	1.545	0.058938717		
0.526555476	0.944	0.610865394	-0.507371025	0.189208072
	2.886	0.374852541		
	1.25	0.366858733		
	1.002	0.375603527		

	0.699	0.783088888		
	1.119	0.534439358		
0.343119505	0.876	0.438735115	-0.771104934	0.371795951
0.650585403	1.059	0.646513064	0.462023593	0.164884468
	0.42	0.018335906		
	1.384	0.11662559		
0.105740785	1.111	0.542657073	0.62436223	0.262323009
	1.671	0.525701129		
	1.897	0.627523453		
	0.874	0.7071236		
	0.575	0.156283052		
	0.783	0.217076089		
0.082723102	0.709	0.427816512		
	0.721	0.188373737		
	0.527	0.009193222		
0.151104076	0.993	0.668500695	-0.421542486	0.145167424
0.70893164	0.877	0.252522893	-1.138528779	0.673075612
	1.075	0.969093521		
0.788756584	0.639	0.005400405	-3.092693902	0.999997381
0.315753404	1.198	0.214462082	1.304416576	0.787660962
0.601543499	1.2	0.268039221		
0.050555854	0.967	0.819899416	-0.229866673	0.07759701
0.294617829	0.897	0.184632412	-1.35750219	0.818656257
	0.897	0.468022663		
	1.238	0.205154356		
	1.092	0.510588735		
0.822570754	0.855	0.246847795	-1.197878189	0.716892562
	0.47			
	0.849	0.560332043		
0.999705998	1.243	0.120310995	1.670200051	0.941679869
	1.95	0.648557421		
	0.83	0.293706553	-1.05712982	0.608973907
	0.633	0.020423784		
	2.962	0.07097046		
0.125935378	0.955	0.724708213	-0.355029272	0.116913915
	0.811			
	1.182	0.592466921		
0.480124767	1.148	0.458825226	0.76300164	0.365349693
	1.816	0.130933095		
	1.104	0.385221036	0.88456487	0.465084471
	1.021	0.936768298		
0.093018908	1.067	0.442167827	0.815232179	0.407498535
	0.772	0.393686243		
	0.86	0.957846283		
0.186087586	1.653	0.09175194	1.747440243	0.958329795
0.853642087	0.843	0.77354709	-0.294730546	0.095741213

0.294870517	0.988	0.495397908	-0.661917762	0.288686419
	0.563	0.070301239		
	0.733	0.436861766		
	0.713	0.128532386		
0.164790921	1.063	0.846867596	0.197791964	0.070359334
	0.994	0.963794698		
0.494504907	1.168	0.196477657	1.318868498	0.796392381
	1.538	0.382404289		
	0.743	0.775232659		
	0.965	0.785333899	0.288185291	0.093694435
	2.791	0.011258136		
0.885909753	1.053	0.853146446	0.188137254	0.068401677
	1.299	0.219858707		
	1.415	0.098419997		
0.050061016	1.398	0.097384692	1.748530786	0.958533852
0.999984052	0.722	0.04792855	-2.084471847	0.992665768
	0.705	0.550945395		
	0.944	0.942232108		
	2.437	0.346878567		
0.114135821	6.443	0.020358639	2.729529883	0.999925991
	1.209	0.347861417		
	1.392	0.327569436		
0.990225215	0.949	0.916249554	0.108483218	0.05607732
0.917069954	0.839	0.125038013	-1.597453487	0.921590547
0.999989124	0.634	0.004347099	-3.341133356	0.999999806
	0.821	0.147449083	-1.492858103	0.884058892
0.965924174	1.593	0.008816185	2.95900865	0.99999045
1	1.491	0.004062719	3.350748862	0.999999826
	0.721	0.644386752		
	0.872	0.477205594		
0.076563341	1.128	0.616387285		
	1.158	0.175078411		
	0.964	0.75268354		
0.999974649	1.153	0.771519941	0.288091537	0.093665472
	0.964	0.990936837		
0.493937551	1.181	0.690854936	0.404511783	0.137441609
0.080614653	0.957	0.798312167	-0.257915964	0.084861706
	3.541	0.733421014		
0.846166069	1.059	0.871607797	0.161838171	0.063581914
	1.455	0.268168177		
	1.187	0.733146189		
	0.866	0.349971483		
0.537405973	1.059	0.869314231	0.162030714	0.06361449
	9.284	0.006329048		
0.09284664	1.399	0.044483111	2.174406542	0.99572375
0.955208453	1.022	0.628553502	0.494834727	0.182250322

0.999977809	0.54	0.007098748	-3.372219469	0.999999862
0.778244053	1.113	0.756697643	0.307435916	0.099854157
0.999999829	0.653	0.024879259	-2.377375051	0.998877251
	1.002	0.850884378		
	1.004	0.71335336		
	1.128	0.722756231		
	2.256	0.028705829		
	1.279	0.16904539		
0.050251187	0.976	0.671285652	-0.421972399	0.145366825
	0.713	0.194446159		
0.781728258	1.079	0.645370282	0.460313822	0.164013346
	0.537	0.317062628		
	0.869	0.730582752		
	0.976	0.856377883		
0.371252576	1.171	0.42093056	0.824186081	0.414850001
0.094853718	0.799	0.921403339		
	2.384	0.675262482		
0.798564347	0.951	0.562543237	-0.576869278	0.230917394
	0.896	0.222614013	-1.233692354	0.741889056
0.51091809	1.31	0.08406872	1.78631721	0.965118099
	1.202	0.84154803		
	1.148	0.263313474		
0.972430543	1.149	0.449199582	0.786620901	0.384240143
	0.904	0.67275704		
	1.643	0.488623419		
0.999499069	0.995	0.932209606	-0.085429426	0.053763756
	1.39	0.435538383		
0.996030943	1.362	0.208866203	1.340599575	0.809110105
0.065967013	0.875	0.920116632	-0.102753577	0.055450353
0.146349783	0.879	0.587156117	-0.550309347	0.214361645
	1.624	0.265022926		
0.36731151	1.2	0.203195587	1.313583737	0.793224666
0.815698958	0.725	0.048499218	-2.090245197	0.992908529
1	1.205	0.080175046	1.91055536	0.980997941
	0.637	0.245641309		
0.192386236	2.663	0.47731695	0.708129933	0.322794825
0.999984151	1.034	0.530200514	0.669197855	0.293942686
0.050839927	1.263	0.117279129	1.639279414	0.933700261
	0.783	0.056665127	-1.986068554	0.98724551
	0.747	0.109781948		
0.051372996	1.438	0.073177665	1.973485121	0.98634815
	0.787	0.394275756		
	0.638	0.350830486		
0.268669579	1.116	0.707687991	0.373611602	0.124287989
0.999231115	0.876	0.480942771	-0.732095891	0.341131505

0.999947554	1.508	0.036514999	2.337402377	0.998519539
0.253159708	1.135	0.51846548	0.668781852	0.293641103
0.061259737	1.03	0.930270626	-0.08659115	0.053867049
	1.107	0.927311341		
	0.728	0.056689029		
	1.458	0.888507929		
0.79837592	1.049	0.800979183	-0.249801536	0.082669623
0.59971405	1.301	0.516092502	0.653495183	0.282662866
0.42512342	1.028	0.786607935	-0.270497153	0.088407004
	0.915	0.704573148		
0.206047718	1.212	0.2119295	1.291033389	0.779382953
0.665084407	1.297	0.000292894	4.460326222	1
0.521308443	0.85	0.260684	-1.157323505	0.687252452
	0.826	0.171724387		
	1.202	0.284560595	1.108913616	0.650225954
0.266555274	1.045	0.924903398	-0.09351637	0.054512017
0.999355204	0.651	0.019345805	-2.569983805	0.999729747
0.147272025	0.854	0.142006385	-1.557942095	0.908673956
0.991720095	1.122	0.313066608	1.043425957	0.597825224
	1.166	0.982051309		
	0.998	0.779466634		
0.488703838	1.454	0.024321316	2.522516928	0.99961066
0.851495737	0.45	0.021127914	-2.811920922	0.999963616
	2.094	0.000928305		
0.900271671	0.887	0.476894265	-0.72770113	0.337738504
	1.19	0.445999971		
0.961254539	0.861	0.171899512	-1.426763044	0.854572215
0.994349946	1.28	0.002582294	3.511696732	0.999999972
0.990832787	0.629	0.004816889	-3.192354987	0.999999049
0.93448805	0.996	0.855233709	-0.182166495	0.067241768
	1.276	0.340517179	0.987948193	0.551961326
0.050006536	1.024	0.897200394	-0.127590063	0.058417775
	0.887	0.444772663		
0.234809524	1.167	0.480994263	0.716905863	0.329461427
0.646338322	1.029	0.949194102	0.064050245	0.052113623
0.250274395	2.068	0.000676997	4.057997784	1
0.998581894	0.714	0.140284819	-1.53810627	0.901622225
0.120710187	0.928	0.350863359	-0.933802734	0.506508215
	1000	0.001		
	1.3	0.1207886		
0.051442638	0.662	0.028743777	-2.297286376	0.998058639
0.475565821	0.79	0.072347525	-1.998410736	0.988075587
	0.59	0.15038474		
	36.886	0.045833598		
0.082850181	1.684	0.483835569	0.703651288	0.319414806
0.050285455	1.025	0.916604732	0.104958719	0.055687573
0.770862228	0.814	0.042975068	-2.15810143	0.99527301

0.905370203	1.483	0.029970396	2.33557049	0.998500888
0.795697589	1.041	0.935228662	0.080692427	0.053357136
0.999200929	0.924		-2.135207365	0.994568603
0.28599661	0.804	0.299700154	-1.056645304	0.608581205
0.407492402	0.752	0.161420003	-1.473244999	0.87579071
	1.433	0.248711443		
0.166588084	1.176	0.052266207	2.048175699	0.990964824
0.909114428	1.087	0.998801726	-0.001494548	0.050001149
	1.28	0.39598237		
0.581472678	1.109	0.693221041	0.397367597	0.13430144
0.225978397	0.982	0.653739918	-0.444360021	0.156047565
0.056581089	1.669	0.883525015	-0.145986939	0.061036321
0.868251322	0.882	0.201594483	-1.317841822	0.795779266
0.903277587	1.02	0.780274986	0.286948429	0.093313141
	1.022	0.648669286		
0.100440088	1.091	0.745095134	0.322398281	0.104935366
0.253199125	1.295	0.020799391	2.607185572	0.999798308
	1.097	0.950845763		
0.136882332	1.17	0.904711875	0.119000974	0.057318044
	0.826	0.738592091		
0.990031839	0.849	0.6770907	-0.437012687	0.152478099
0.999980946	1.559	0.020954339	2.475943463	0.999447924
0.11548058	1.043	0.544127943	0.631930249	0.267531165
	0.928	0.596546047		
0.696192455	0.788	0.068224086	-1.911915761	0.981130189
	1.12	0.606184366		
0.057135696	1.586	0.201025592	1.338750993	0.808047649
	0.817	0.457497479		
0.941290482	1.113	0.703389375	0.376552681	0.125491982
	0.826	0.45971483		
0.184861208	0.886	0.276191702	-1.147572996	0.679930623
	0.492	0.801147111		
0.092667972	0.929	0.615158876	-0.513300097	0.192560189
0.916682754	0.69	0.365246657	-0.97389703	0.540207448
0.82885262	1.529	0.396711868	0.835843817	0.424466444
	1.014	0.863144412	-0.17082615	0.065145094
0.759746849	0.947	0.50691864	-0.663734104	0.289993546
0.98899542	2.954	0.037143188	2.205487052	0.996477774
0.992714078	0.74	0.171539422	-1.461150899	0.870490901
	0.372	0.001353387		
0.244139849	1.014	0.946374729	0.067461933	0.052345108
0.308472493	1.036	0.688716933	0.413966212	0.141688766
	0.997	0.464187452		
	0.985	0.850740355	0.196087847	0.070006407
0.991945708	0.587	0.027291759	-2.320213265	0.998335893
	1.259	0.913379533		
	1.167	0.58206524		
0.068259409	1.072	0.872273281	0.163172785	0.063808534

0.261678227	0.873	0.619800123	-0.517838874	0.195152773
0.999997985	0.75	0.188673304	-1.392831012	0.837621334
0.525311248	0.797	0.101844686	-1.786491587	0.965146365
0.33883471	2.509	0.087898114	1.787889131	0.965372223
0.999967752	1.106	0.428581794	0.803103753	0.397593534
0.884785843	0.832	0.1157634	-1.636498832	0.932943084
	0.762	0.457311314	-0.757766922	0.361205768
0.427481842	0.945	0.481947179	-0.698874226	0.315826453
	1.363	0.227132876		
	1.544	0.255650721		
0.548432703	1.281	0.076379625	1.915934015	0.981516246
0.057426615	1.065	0.754111947	0.318434905	0.103564314
	0.001	0.001		
	0.963	0.770551382		
	1.555	0.801058149		
	1.605	0.095335142		
	0.747	0.219295149		
0.549457523	1.028	0.831368089	-0.209843995	0.072946081
	2.407	0.154566462		
0.780600241	1.082	0.459521568	0.77478064	0.374732236
0.0608645	1.233	0.120598944	1.599056437	0.922083663
0.299627969	0.793	0.117423594	-1.639117046	0.93365623
	1.585	0.417170078		
0.81127584	1.306	0.058338909	2.053663415	0.991242354
0.903534839	1.161	0.107509829	1.680919585	0.944262795
0.303336531	1.174	0.706598867	0.382426009	0.127926542
0.285750029	0.967	0.822051198	-0.225200342	0.076473489
0.712623559	0.951	0.599785132	-0.524057228	0.198741874
0.999999976	0.82	0.074002941	-1.91556972	0.981481525
0.77356902	0.941	0.491212869	-0.701565214	0.317845664
	0.82	0.686860605		
0.363422358	0.932	0.448756024	-0.76533434	0.367201513
0.086396979	1.054	0.576212618	0.563648566	0.222583333
	1.352	0.194015789	1.377205315	0.829398593
0.835210573	1.283	0.082492913	1.891523616	0.979063526
	0.749	0.04993976	-2.116775116	0.993935171
	1.518	0.32412107		
	1000	0.001		
	0.809	0.38695508		
0.195158232	0.973	0.98221059	0.023045832	0.050273335
0.138886857	0.905	0.456618195	-0.750197757	0.355243163
0.828630781	1.022	0.940041593	0.076535494	0.053019558
	1.34	0.161733285		
0.980892975	1.19	0.195615771	1.358475945	0.819196927
	1.667	0.264210839		
0.827321587	0.999	0.909721636	-0.112948916	0.056589893
0.825220911	0.76	0.023895023	-2.467076075	0.999410561
0.9874959	1.036	0.74652219	0.328974957	0.107250425

0.946928091	1.03	0.739156749	0.342206696	0.11205968
0.461036769	1.104	0.784869036	0.274787525	0.089656892
0.924641584	1.123	0.431747047	0.806191943	0.400109521
0.921882193	1.677	0.008712517	2.873829004	0.999979051
0.629950644	1.029	0.935306028	-0.080309421	0.053325281
0.09268875	0.93	0.67242292	-0.432758452	0.150439958
0.140412268	0.932	0.40744777	-0.828959302	0.418781531
0.957967562	1.277	0.101020839	1.745578149	0.957979487
0.373551187	1.051	0.739625324	0.32791144	0.10687267
	0.162	0.153901861		
0.945446819	1.512	0.009429304	2.900324899	0.999983539
	0.513	0.047928787		
	0.845	0.600960966		
0.582770875	0.986	0.829003318	-0.216243128	0.07438437
0.147157662	1.034	0.883848108	-0.14456279	0.06082076
	0.902	0.723729377		
0.799742493	1.059	0.683561874	0.414728933	0.142035942
0.837771991	2.016	0.004626081	3.266976136	0.999999567
	1.012	0.498585786		
	0.956	0.693821854		
	1.124	0.734384645		
0.97218834	0.9	0.958799329	-0.054357034	0.051521757
	1.005	0.838739935	0.208095453	0.072560909
0.727223661	1.105	0.280909807	1.124617929	0.662417235
0.999999998	0.738	0.010002335	-2.915520023	0.999985684
0.939486979	0.352	0.117536337	-1.687067184	0.945702797
0.768966383	1.171	0.291628152	1.09399482	0.63849833
	1.358	0.170376618		
0.904697652	1.226	0.219551338	1.2982671	0.783880088
	0.807	0.212217945		
	0.754	0.270724055		
0.085926169	1.19	0.052747595	2.083614678	0.992629107
0.126402253	1.015	0.976900001	0.028790817	0.050426637
0.98732631	1.256	0.1572578	1.511763412	0.891650819
0.749693232	1.01	0.974427799	0.032373465	0.050539459
0.21728944	0.924	0.502433687	-0.685870167	0.306148872
0.526703665	1.226	0.381214266	0.900034923	0.478079781
0.89247629	0.922	0.481002674	-0.702799879	0.318773975
	1.279	0.564888907		
	2.502	0.671825825		
0.788891095	1.015	0.830480781	0.217938273	0.074772935
	1.274	0.225734714		
	0.61	0.418525385		
0.712995469	0.346	0.052624203	-2.039389167	0.9905044
	0.885	0.926462361		
0.291824144	1.005	0.837851953	0.210040092	0.072989487
	0.752	0.564397928		
0.503579324	0.897	0.280473333	-1.131815169	0.667948714
0.984610787	1.066	0.38144853	0.914999044	0.490672594

	0.899	0.482989768		
	1.946	0.757820559		
0.82051791	0.468	0.006731941	-2.948516391	0.999989463
0.932618789	1.332	0.029722448	2.328948581	0.998431667
	0.747	0.030180092		
0.999354356	1.13	0.061183782	2.013465675	0.989024284
0.068032315	0.646	0.043527842	-2.281027431	0.997837249
	0.692	0.376957779		
0.898599458	0.773	0.140282709	-1.525335642	0.896875637
0.466949243	1.064	0.939695739	0.074846986	0.05288757
0.78156496	1.035	0.789374337	-0.265417313	0.086954042
	0.791	0.648439437		
	0.75	0.742290612	-0.345432149	0.113262768
0.67144181	2.466	0.047032309	2.143945735	0.994847794
0.991809015	0.855	0.505095793	-0.68525916	0.30569749
0.050222425	1.192	0.577440966	0.552342619	0.215602592
0.867936149	1.266	0.151465099	1.529788252	0.898549146
0.999896755	0.738	0.125493472	-1.585182519	0.917736831
0.058726312	0.829	0.314321066	-1.031140934	0.587762047
0.087037556	0.905	0.429225913	-0.796379996	0.392130656
0.282456943	1.592	0.034749365	2.261443503	0.997540311
	0.85	0.385730387		
	0.783	0.638044897		
	1.44	0.319025606		
0.418164177	0.963	0.526262713	-0.621778126	0.26055713
0.418605467	0.528	0.125741871	-1.627497951	0.930446091
0.996805954	0.96	0.795900598	-0.26149075	0.085850906
	0.671	0.018341933		
0.464653383	1.1	0.481306172	0.732096973	0.341132343
	1.483	0.324627293		
0.99999792	0.623	0.038692675	-2.388502901	0.998961655
	1.087			
0.370585383	1.023	0.874431087	-0.156294008	0.062660972
0.7372031	1.122	0.482958709	0.72287854	0.334030645
0.515887135	1.084	0.324886619	0.993767115	0.556816065
0.159572317	0.873	0.087945067	-1.824345997	0.97085162
0.234922849	0.94	0.777261333	0.291277362	0.09465527
0.998689537	0.913	0.218738476	-1.260189502	0.759615267
0.796516192	1.117	0.428171844	0.828661837	0.418536276
	1.843	0.056460084		
0.221288001	0.537	0.092984514	-1.757192265	0.960125909
	1.277	0.175524569		
0.391585942	1.391	0.085889772	1.785558138	0.964994836
0.607963873	2.124	0.28387208	1.078369815	0.626074001
0.99868116	1.561	0.012853164	2.853428596	0.999974827
0.925845409	1.902	0.032033413	2.349104485	0.998633765
0.608821928	1.073	0.534055174	0.627792087	0.264676661
	1.683	0.585277002		
0.9138709	0.572	0.039566674	-2.151860144	0.99508955
	0.921	0.521466607		

	1000	0.001		
	3.186	0.272676162		
	0.696	0.277417663		
0.988811866	0.646	0.02095238	-2.441042139	0.999286936
0.982321148	1.268	0.10384837	1.6879534	0.94590793
0.95373403	1.081	0.478700917	0.693467803	0.311786729
0.159576518	1.036	0.44464072	0.805218041	0.399315612
0.085957802	0.757	0.069550993	-1.901467931	0.980094136
0.205817015	1.721	0.215227128	1.28778182	0.777344099
	0.763	0.528830009		
0.477554255	1.127	0.648496742	0.456328166	0.16199575
0.329117469	0.778	0.161889436	-1.48452385	0.88059458
	1.181	0.406435588		
0.993194107	1.083	0.811155414	-0.236418372	0.07921528
	1.074	0.663613049		
0.991046591	0.653	0.24389202	-1.190311509	0.711466023
0.253453837	1.165	0.722587058	0.354622023	0.116756806
0.885038569	0.415	0.016599453	-2.701075558	0.99990604
	0.644	0.040768625		
	0.863	0.638633106		
0.991901392	0.904	0.514522038	-0.667792494	0.292924459
	1.381	0.807617131		
0.140599906	1.055	0.609605881	0.525846508	0.199782532
0.999963277	1.194	0.157263959	1.48008364	0.878719324
	1.579	0.546907523		
0.453421685	1.294	0.03532053	2.243937129	0.997244144
	1.001	0.826700001		
0.999999967	1.256	0.066447497	1.979609384	0.98679148
	1.178	0.470717172		
0.874903921	1.148	0.148935688	1.503455645	0.88835994
0.999997848	0.937	0.712009363	-0.373874404	0.124395162
0.974165439	1.253	0.760098167	0.299003966	0.097103964
0.230664104	0.45	0.282314275	-1.126031301	0.663506321
0.524186209	1.02	0.903393701	0.120608095	0.057517892
0.942857107	1.216	0.103702998	1.751009861	0.958994709
0.720429402	1.311	0.35781544	0.913761674	0.489630779
0.172551628	1.196	0.983655593		
0.611798401	1.001	0.668700868	-0.421390465	0.145096966
0.094691996	1.108	0.465533222	0.755464027	0.359387946
	0.753	0.345119743	-0.968838055	0.535966349
	1.123	0.504128208		
0.382453361	0.786	0.335795771	-1.002790976	0.564327475
	0.924	0.931364335		
0.99995588	6.084	0.005414094	3.515546436	0.999999973
0.999957892	0.804	0.027805361	-2.3677514	0.998799232
	1.164	0.474495314		
0.279570252	0.497	0.410169254	-0.870387782	0.453207819
	0.962	0.771924586	-0.295254637	0.095907217

	0.777	0.67036668		
0.999976609	1.105	0.376676535	0.897468611	0.475922001
0.636790907	1.2	0.283154307	1.114076903	0.654252168
0.498033974	0.869	0.240576033	-1.188813726	0.710386067
0.916818466	1.894	0.020894733	2.575753065	0.99974164
	1000	0.001		
0.837643789	0.481	0.578777241	-0.588110233	0.238146103
	6.475	0.142594825		
	0.797	0.319754627		
	0.877	0.356540971	-0.923279491	0.497645688
1	0.419	0.000164649	-4.623221795	1
0.05710167	1.075	0.666186955	0.437976262	0.152942653
0.591499412	1.183	0.823761568	0.219475683	0.075128086
0.99586225	0.794	0.003570886	-3.311133349	0.999999731
0.89356806	0.666	0.005564021	-3.124946412	0.999998105
0.064473866	1.172	0.878516051	0.148756592	0.061461734
	1.051	0.730134965		
0.06512051	0.596	0.026339239	-2.442903159	0.999296511
	0.613	0.044438917		
0.050005842	0.864	0.33118624	-0.987382708	0.55148911
0.991317193	0.831	0.281679204	-1.098058873	0.641706599
	2.643	0.115247849	1.603084008	0.923312263
0.933507873	0.791	0.511262854	-0.675708023	0.298681362
0.063024002	0.532	0.208807876	-1.304312005	0.787596994
0.072687548	0.886	0.227082813	-1.230524729	0.739725373
0.098086898	0.721	0.765494304	-0.312928934	0.101689661
0.880085744	0.805	0.08725829	-1.806439078	0.968257706
0.273631852	1.152	0.382298639	0.889694051	0.469389673
0.41454372	0.967	0.756423728	-0.312668689	0.101601917
0.934721628	0.601	0.48075639	-0.753619542	0.357934308
0.854024354	1.184	0.499669139	0.6842327	0.304939874
0.913707924	0.956	0.67348374	-0.428545769	0.148442449
	1.379	0.50131306	0.668512922	0.293446221
0.154079154	0.946	0.700931854	-0.396501212	0.133924678
0.08011272	1.128	0.087604217	1.835324885	0.972352987
0.598785319	1.554	0.021841794	2.470321423	0.999424499
0.123743881	1.138	0.28409835	1.093208963	0.637876814
0.07393327	0.793	0.177335424	-1.389437586	0.835857945
0.988691412	1.196	0.24118974	1.255000656	0.7561972
	1000	0.001		
0.634880903	1.114	0.681170817	0.417302929	0.143212583
	2.309	0.050730451	2.129722697	0.994386569
	0.78	0.583851939		
0.664362854	1.307	0.006932672	3.1504968	0.999998538
0.332010616	1.007	0.553661716	-0.594904144	0.242577611
0.999950477	0.984	0.788472373	-0.268224563	0.087753382
	1.12	0.255479393		

0.987584016	1.135	0.156160979	1.47159138	0.875075177
0.076499305	1.767	0.009461542	2.961824565	0.9999907
0.074395803	1.04	0.693082796	0.401243063	0.135997479
	0.978	0.850174994		
0.991255252	1.232	0.010698392	2.82812491	0.999968462
0.417184735	1.136	0.179009396	1.392017387	0.837199665
	0.583	0.066316454		
0.999999865	1.242	0.117115624	1.671181235	0.941920136
0.770639785	0.78	0.100153633	-1.74533102	0.957932817
	1.095	0.402076248		
0.759365088	1.055	0.56942688	0.564216128	0.222937344
0.730986688	0.936	0.527935035	-0.631459299	0.267205493
0.982897512	1.232	0.062088685	1.962441754	0.985516202
0.944730766	1.107	0.266719037	1.194309257	0.714339184
0.98934968	1.174	0.085368169	1.840881678	0.973087943
	0.372	0.131940489		
	1.124	0.631073733	0.480983274	0.17476933
0.054696172	1.08	0.395538959	0.90071711	0.478653482
0.999996266	0.647	0.008323535	-2.921988702	0.999986514
0.795204886	1.184	0.518344398	0.651071894	0.28094144
	1.299	0.268870815		
0.152835771	0.993	0.792165736	-0.259594567	0.085324411
0.569420798	0.787	0.035741939	-2.194121439	0.99621714
0.573323572	0.807	0.809704505	-0.250835919	0.082944949
	0.707	0.344292607		
	1.025	0.856122998		
0.050296203	1.028	0.670044666	0.439911793	0.153879058
0.80320462	0.856	0.213003845	-1.257332166	0.75773629
	0.537	0.056751843		
0.999186189	0.982	0.801472061	-0.25202985	0.083264233
0.050732678	0.968	0.849629818	-0.193302578	0.069436394
0.129020149	0.704	0.089819997	-1.880960666	0.977919643
0.957105852	1.371	0.059352096	1.974157342	0.986397432
0.999990544	1.546	0.018000728	2.628377517	0.999829709
0.990129662	1.098	0.559987652	0.588020547	0.238087916
0.301908804	0.827	0.059132116	-1.992378754	0.987675954
0.194076553	0.946	0.384097632	-0.867326106	0.450648106
0.665601613	0.689	0.003431306	-3.364725563	0.999999851
	0.757	0.805422107		
	1.167	0.966451911		
	0.899	0.410280396	-0.823473558	0.414263851
0.72501903	1.835	0.015387793	2.70311718	0.999907625
0.427945194	1.017	0.89743121	0.128300716	0.058512259
0.331309887	1.137	0.241137424	1.197527538	0.716642178
0.972592314	1.676	0.156332771	1.463995652	0.871751412
0.71187532	0.599	0.020408546	-2.48886973	0.999498477
0.94756744	0.925	0.690629014	-0.410831916	0.140269213

0.99999324	1.199	0.029708293	2.373581481	0.998847069
0.708528406	0.861	0.286832788		
0.079637322	1.641	0.122831786	1.598307643	0.921853605
0.986790547	0.645	0.010385454	-2.814466352	0.999964421
0.826665516	1.152	0.435495415	0.796194248	0.391980041
0.999761077	0.987	0.889186313	-0.140977488	0.060287653
0.400145786	1.018	0.760628328	-0.29748101	0.096615914
0.999665052	1.15	0.284643314	1.076059009	0.624224963
0.13419941	0.929	0.596224509	-0.544024086	0.210553746
	0.91	0.578653111		
0.266889842	0.981	0.966737715	-0.042337676	0.050922854
0.996839951	0.77	0.630797059	-0.491142516	0.18023492
	0.652	0.030609134		
0.133968025	1.051	0.646616924	0.468544762	0.168237853
	1000	0.001		
	0.741	0.047700005		
	1000	0.001		
0.208826824	0.996	0.965555324	-0.043803854	0.050987917
0.080995515	1.085	0.920602533	0.098101963	0.054966667
	2.373	0.040434859		
	0.994	0.829303605		
0.650786548	1.178	0.234680759	1.199488818	0.71804127
	1000	0.001		
	0.486	0.086227915		
0.050130303	0.905	0.181806003	-1.428404849	0.85536098
	1000	0.001		
0.395912403	1.031	0.970709022	0.03757452	0.050726801
0.677891325	0.969	0.781520509	-0.280204092	0.091264665
	1000	0.001		
0.201264836	1.16	0.261545442	1.128612787	0.665491952
0.849645341	1.85	0.188696945	1.320485231	0.797355635
0.052422465	1.017	0.878054076	-0.157445292	0.0628495
0.346586859	0.942	0.633945936	-0.487202172	0.178101118
0.201631917	1.883	0.007372603	2.997722134	0.999993384
	0.914	0.671403774		
0.913370667	0.982	0.466009766	-0.72245352	0.333704653
0.510414285	0.936	0.458603051	-0.73808308	0.345775088
	1.04	0.668995347		
	20.661			
	0.58	0.927920692		
0.982852603	1.029	0.875452572	0.156566333	0.062705438
	1.114	0.889222379		
0.632448114	0.915	0.439245998	-0.796661134	0.39235865
0.882986509	1.071	0.605195554	0.530001496	0.202212707
	0.479	0.180614724		
	0.852	0.739229991		
0.070230616	1.177	0.113996414	1.634751329	0.932463808
0.05092332	0.827	0.295077011	-1.085780831	0.63198425

0.433586548	1.275	0.287333456	1.094237065	0.638689841
	0.84	0.333048966		
0.648541006	0.93	0.812243685	-0.243545565	0.081029981
0.740580477	1.38	0.916740222	0.10460263	0.055648921
0.694436906	1.031	0.753600577	0.315873389	0.102687838
0.999987782	1.068	0.476576521	0.730251409	0.339705844
0.898825205	0.972	0.699615403	-0.388173366	0.130347846
0.187514126	0.908	0.411537142	-0.835454826	0.424144798
0.051779637	0.763	0.090009495	-1.883195596	0.978165976
0.468591875	1.055	0.847689022	0.194544747	0.069689561
	0.926	0.75253006		
	0.001	0.001		
0.070664686	0.984	0.808457478	-0.243380854	0.080987403
0.05605661	1.099	0.300823344	1.068338178	0.618026382
	0.643	0.189309842		
0.524559985	1.107	0.826053289	0.215103424	0.074124906
0.238179123	1.323	0.411676	0.859271133	0.443923912
1	0.62	0.052069509	-2.16905122	0.995580139
0.37126279	1.367	0.311611076	1.027167016	0.584494035
0.36213655	1.654	0.291250079	1.040251601	0.595230928
0.585179502	0.984	0.968058699	0.041109217	0.050870049
0.479525246	0.849	0.44668439	-0.778888786	0.378022719
	1.056	0.694308539		
0.062679572	1.131	0.325833107	1.03480616	0.59077079
0.733599358	1.121	0.276188944	1.135383206	0.670677479
0.475463995	1.21	0.130361365	1.588078822	0.918659055
0.999841529	1.535	0.015451432	2.692104209	0.999898766
	1.006	0.948046528		
0.999999987	1.196	0.247735315	1.195901375	0.715479616
0.261052021	0.813	0.797904572	-0.264354243	0.086653665
0.79153862	0.923	0.476908378	-0.716130271	0.328869971
0.900164237	1.052	0.829174683	0.217880471	0.074759633
	0.22	0.273351035		
0.608731395	0.972	0.887174161	-0.146577478	0.06112634
0.97489273	1.063	0.938525209	0.076242395	0.052996434
0.977883708	1.069	0.652753215	0.463489131	0.165633832
0.573378648	1.094	0.644106737	0.465071364	0.16644564
0.137133607	0.931	0.778292416	-0.289798136	0.094194253
	0.353	0.237185119		
0.994526642	0.779	0.132624612	-1.594045066	0.920534143
0.06237017	1.203	0.960970143	0.048359388	0.051204241
0.097254683	0.877	0.564263914	-0.597886927	0.244537928
0.106560898	1.332	0.177613322	1.40461864	0.84365039
	1.069	0.735332931		
	1.231	0.260994366		
0.852029735	0.837	0.261413333	-1.136872476	0.671813761
	0.001	0.001		
	1000	0.001		

0.972401557	1.182	0.109144804	1.674380453	0.942698138
	0.45	0.079552753		
0.763383648	1.363	0.025426363	2.44792239	0.999321746
	1.114	0.657475535		
0.389306143	0.985	0.931685399	-0.08683583	0.053888984
0.989503336	1.05	0.61203173	0.524322757	0.198896085
0.999930682	1.206	0.304382466	1.073977947	0.622557299
0.071751315	0.813	0.287203389		
0.130085957	1.137	0.476507844	0.722406479	0.333668581
0.090235957	1.086	0.592174039	0.541731908	0.20917566
0.051377674	1.053	0.534114121	0.658435398	0.286188381
0.908230119	0.836	0.326167829	-0.992307924	0.555599436
	0.001	0.001		
0.957750353	1.125	0.326028975	1.035342891	0.591210939
	1.512	0.028068185	2.341913543	0.998564568
	1.261	0.630777257		
0.797258784	0.798	0.243344481	-1.155070227	0.685566862
0.858217217	0.994	0.614307307	-0.502807936	0.18665505
0.874607728	0.723	0.07412209	-1.830783913	0.97174002
	0.917	0.564151024		
0.392436042	1.397	0.12645757	1.559384545	0.909171725
0.099033869	0.923	0.478874099	-0.709729827	0.324005924
0.376871214	1.532	0.018823048	2.64901749	0.999855844
	0.37	0.137569685		
	0.751	0.247274426		
	0.929	0.372430667	-0.884286292	0.464850758
	0.825	0.227974186		
0.081201071	1.055	0.620590649	0.5000665	0.185132485
0.060716414	0.945	0.560670241	-0.590653326	0.239799425
0.999999625	0.628	0.129318943	-1.604460056	0.923728623
0.070365241	1.037	0.873147033	0.162068205	0.063620838
0.162263331	1.025	0.801865892	0.254605167	0.083958378
	1.161	0.947284395		
0.338414401	1.246	0.443722537	0.788719842	0.385933218
0.741220921	1.332	0.139623418	1.546984826	0.904826464
	0.687			
	1.145	0.565105592		
0.965758844	1.077	0.744311258	0.323509762	0.105323113
0.999995321	1.082	0.475811675	0.748340138	0.353785258
	2.048	0.368803639		
0.999990097	1.25	0.022702479	2.509618039	0.999570739
	0.495	0.007617578		
	1.075	0.546666156		
0.868491843	0.68	0.00035954	-4.343396869	1
0.050218425	1.009	0.939572511	0.076955982	0.053052888
0.793503116	1.325	0.130752084	1.595729264	0.921057483
	0.31	0.009699681		
0.564760834	1.054	0.760765319	0.311672126	0.101266637
	0.226	0.081580519		

0.053189401	0.973	0.567332706	-0.565936902	0.224012735
0.828299183	1.317	0.001737353	3.736187992	0.999999998
0.992208948	0.793	0.404948544	-0.853839444	0.439398666
0.087756277	1.663	0.084086008	1.928174095	0.98265099
	0.454	0.014181804		
1	0.533	0.010624753	-2.826210026	0.999967923
0.547297507	1.021	0.799679227	0.259926332	0.085416237
0.06964045	0.991	0.832723906	0.219986944	0.075246769
	0.843	0.406926356		
0.95071847	1.083	0.315686648	1.045009065	0.599117435
	0.76	0.240052946		
	0.772	0.128570992		
0.406587206	1.084	0.78416178	-0.266030091	0.087127766
	1.031	0.853169751	-0.185890892	0.067960744
0.995596084	1.224	0.242832998	1.230561185	0.739750327
0.907482095	0.58	0.015718267	-2.588034784	0.999765352
0.115928093	1.246	0.082391277	1.825399216	0.970998528
0.152105179	2.18	0.067103015	1.915551291	0.981479768
0.97755791	0.943	0.568414058	-0.581743358	0.23403581
	1.094	0.902743215		
	0.254	0.308096994		
	1.762	0.034195843		
0.085732136	0.764	0.073695658	-1.823762983	0.970770033
	1.889	0.190616801		
	0.424	0.007517455		
	1.449	0.484381146		
0.960860758	1.197	0.240993021	1.221184761	0.733291469
0.052993633	1.113	0.905887483	0.115537403	0.056896604
0.771970786	0.702	0.053032252	-2.205814471	0.996485037
0.171399903	1.037	0.705840635	0.384638153	0.128853933
	0.903	0.506980854		
	0.001	0.001		
	0.613	0.188764585		
0.088662688	0.88	0.166849444	-1.403730202	0.843201193
0.532436375	1.103	0.634860099	0.480638374	0.174585839
0.053742975	1.231	0.380466636	0.889481312	0.469211034
0.459166831	0.781	0.175678526	-1.416827421	0.849737111
0.978957385	1.817	0.030799561	2.502209203	0.999546124
	0.59	0.090948381		
0.055194571	1.11	0.310614355	1.035870614	0.59164359
0.050522133	1.235	0.043951213	2.161123965	0.995359637
0.066655711	1.395	0.394617108	0.844770271	0.43186105
0.388752181	0.9	0.381954184	-0.888178733	0.468117385
	6.125	0.44307617	0.775567	0.375361375
0.999934358	1.632	0.012057986	2.78016509	0.999952003
0.065673896	0.858	0.041664062	-2.151433609	0.995076784
	1.137	0.293657687		
0.519008684	0.874	0.11052113	-1.687006994	0.945688843

	1.554	0.826913315		
0.409917838	0.815	0.139153855	-1.530493305	0.898812312
0.899254697	1.427	0.351094067	0.930824639	0.504000246
0.999760385	1.456	0.102718196	1.689552332	0.946276482
0.475366405	1.059	0.608742952	0.521354815	0.197176838
0.397299414	0.957	0.493083165	-0.687576714	0.307411188
0.96432069	0.537	0.006050942	-3.584713956	0.999999988
0.231370109	1.268	0.271600087	1.110343921	0.651342996
0.413590587	0.955	0.610363423	-0.509286812	0.190286901
	1.137	0.52385535	0.649497477	0.279825841
0.361625143	1.08	0.81179933	0.236996196	0.079360294
0.90555509	0.913	0.372293398	-0.894637415	0.473542329
0.102322846	1.165	0.677280383	0.407962303	0.138979584
	1.238	0.366579681		
0.552970505	1.391	0.13150323	1.632327978	0.931794789
	0.067	0.163536087		
	1.113	0.729442965	0.349442999	0.11477565
	1.331	0.33800561		
0.657047293	0.826	0.206459184	-1.265730101	0.763235959
0.296729599	1.056	0.632207251	0.486395995	0.177666724
0.982702901	1.17	0.3106867	1.019443886	0.578126155
0.055278985	0.82	0.459572662	-0.758016742	0.36140316
0.503797433	1.932	0.11393589		
0.068312318	1.044	0.891246657	-0.134563168	0.059368005
0.9999618	1.187	0.352906664	0.939838282	0.511590078
0.910894538	0.833	0.263305374	-1.164921899	0.692907443
	1.132	0.653714752		
0.270992574	0.858	0.175112228	-1.372094934	0.826652363
0.99822071	1	0.917122574	0.106903104	0.055900975
0.863556158	0.891	0.241005461	-1.213137108	0.727683831
	0.849	0.253729709		
0.347923336	0.873	0.756061193	-0.322666823	0.105028918
0.999999895	1.174	0.134361637	1.601951782	0.92296838
	1.067	0.854051778		
0.732312189	1.004	0.947311675	-0.066222053	0.052259585
0.501650735	0.91	0.80003203	-0.261453212	0.085840444
0.725557129	1.253	0.267879024	1.11700974	0.656531386
0.103319095	2.094	0.00525808	3.120925588	0.999998026
	0.755	0.746874848		
	1.679	0.535757128		
0.999999982	0.6	0.015313716	-2.675009865	0.999883419
	0.914	0.784348107		
0.994679646	0.37	0.019095646	-2.899535883	0.99998342
0.997532175	0.92	0.289927258	-1.127575516	0.664694657
	0.755	0.407945672		
0.994689645	1.23	0.358621401	0.943375598	0.514567626
0.999999999	0.44	0.07083425		

	1.092	0.728465776	0.349085486	0.114640039
	0.104	0.001073037		
0.907668478	0.904	0.774727473	-0.294224681	0.095581279
1	1.895	0.026469981	2.396004819	0.999015227
	0.896	0.937751604		
	1.59	0.023732169		
0.888848666	0.79	0.368878018	-0.926396732	0.500271064
0.752632907	1.089	0.461760896	0.73349651	0.342215641
	0.411	0.638677586		
0.050010431	1.249	0.427149859	0.808699456	0.402155525
0.936657562	0.595	0.02925945	-2.608561803	0.999800501
0.323002389	0.916	0.341577917	-0.947588422	0.5181127
0.632759348	1.105	0.314902857	1.055801486	0.607897021
0.968305877	1.045	0.77309358	-0.296409218	0.096274035
0.969013948	1.177	0.121534896	1.583917568	0.917331588
	0.951	0.696967371		
0.389757907	0.992	0.717533738	-0.359229085	0.118545376
0.959803926	0.927	0.597787292	-0.528688311	0.201442597
	0.793	0.402909373		
0.067266042	1.275	0.413847101	0.831714671	0.421054798
0.981975451	0.976	0.743981853	-0.32798071	0.106897235
0.093278931	1.023	0.877148446	0.154057298	0.062298759
0.953030608	1.002	0.801287341	0.262085956	0.086017007
	1.204	0.8766335		
0.999786371	0.897	0.409315885	-0.855370907	0.440673774
0.945105176	1.125	0.469040229	0.725463106	0.336015765
0.981178207	0.68	0.512434835	-0.671959127	0.29594821
0.238901444	0.917	0.526389161	-0.640524277	0.273510411
0.088333057	0.96	0.520122087	-0.63653288	0.270724899
0.889613728	1.068	0.985002232	-0.018280569	0.050171974
0.783719798	0.946	0.589997525	0.568138491	0.225393154
	0.159	0.42075452		
	0.37	0.547292955		
0.999744498	0.471	0.014395643	-3.069709088	0.999996712
0.050023759	1.003	0.963157952	0.046642421	0.051120192
	0.498	0.188636856		
	1.152	0.417912112		
	0.917	0.49847915	-0.679891524	0.301745239
0.386132281	1.213	0.183445566	1.424063856	0.853269166
0.999306786	0.989	0.979310376	-0.026397467	0.050358639
	1.1	0.80990818	0.244800925	0.081355491
0.99008778	0.761	0.016140431	-2.680536839	0.999888605
	0.609	0.035240288		
0.984268612	0.996	0.968909433	-0.039522859	0.050804165
	1.316	0.092138323	1.854345094	0.974801183
	0.442	0.140217828		

0.133747576	1.073	0.942760976	0.069809301	0.052511392
0.992034479	0.755	0.154727281	-1.427538423	0.854945087
0.679635677	1.012	0.820284787	0.233649618	0.078525575
	1000	0.001		
	0.802	0.438870133		
0.165682201	1.019	0.888158289	0.141674888	0.060390278
0.999743555	0.962	0.754485325	-0.315861469	0.102683777
	1.034	0.924015085		
0.167221785	1.74	0.422265911	0.800951646	0.395842729
	1.875	0.104932194		
	1.131	0.699596718		
0.182035223	1.277	0.684372747	0.394926431	0.133242099
0.24323648	0.706	0.016581641	-2.557670642	0.999702637
0.968064769	1.119	0.32190993	0.981171727	0.546297859
0.060187255	0.846	0.183337714	-1.355743349	0.817677106
0.718950015	0.846	0.822470717	-0.229936314	0.077613961
	1.714	0.215443961		
	3.697			
	1000	0.001		
0.05515745	0.974	0.895206078	-0.134796002	0.059400623
0.99999953	0.626	0.001221393	-3.780041305	0.999999999
	2.608	0.052935912		
	0.683	0.235408756		
0.944210716	0.897	0.194953761	-1.328928844	0.802341841
0.844227074	1.015	0.856986115	0.184817925	0.067752069
0.054336678	1.218	0.156686786	1.502752608	0.888078201
0.213382816	1.246	0.206729587	1.301644655	0.785961524
0.799064323	1.139	0.266913564	1.150214082	0.681920976
0.489472264	0.86	0.288235987	-1.108733866	0.650085479
0.969157718	0.868	0.551328258	-0.605708654	0.249720673
	1.352	0.156746705		
	0.489			
0.135002148	0.682	0.363403848		
0.630540795	0.714	0.035159517	-2.305799707	0.998166151
0.62737179	0.563	0.150459943	-1.512774024	0.892046323
	0.368	0.234990565		
	0.493	0.135767313		
0.269603643	0.948	0.493264976	-0.68537527	0.305783244
0.839612428	0.979	0.848817382	0.198577569	0.070523104
0.134893281	1	0.953878787	0.059183609	0.051804303
	0.839	0.522234855	-0.662820544	0.289335746
0.432761412	1.05	0.951680276	0.059535918	0.051825872
	0.318	0.070713784		
	0.76	0.677292208		
0.058816371	0.909	0.868104863	-0.171391384	0.065246307
0.329986837	0.903	0.680541905	-0.417713464	0.143400963
0.988580481	0.602	0.261735499		
0.052587943	1.061	0.664734093		
0.823715482	1.838	0.605518811	0.510825945	0.191156602

	1.523	0.086704072		
0.992726967	0.882	0.307993625	-1.035305835	0.591180555
	1.7	0.171960251		
0.05521646	0.758	0.918476198	-0.107448217	0.055961515
	1.008	0.348970566		
0.05033933	1.021	0.689915784	0.411370156	0.140512173
0.395077714	1.25	0.227527644	1.238034536	0.744839762
0.071106301	1.475	0.034809123	2.279382561	0.997813622
0.07982852	0.775	0.753115323	-0.320713401	0.104350302
0.61261263	0.796	0.244598455	-1.202308823	0.720047071
0.427701394	1.095	0.390135769	0.927911177	0.501546549
0.441980546	0.766	0.142181373	-1.495022083	0.884946589
0.134017266	1.004	0.825770703	-0.216006993	0.074330494
	1.1	0.591919849		
	0.8	0.770605359		
	1.237	0.830334167	0.214855627	0.074068682
0.14637292	0.812	0.264320089	-1.153195234	0.684161285
0.050339337	1.152	0.562173683	0.57129716	0.227382556
	1.326	0.348726699	0.945854199	0.516653518
0.999970961	1.482	0.006525553	3.118907748	0.999997986
0.9526888	1.243	0.313105093		
	0.001	0.001		
	2.453	0.005302718		
	22.283	0.330681414		
0.996394366	1.029	0.717584917	-0.360042885	0.118863879
0.500969761	1.043	0.668653393	0.43885875	0.153369059
	1.82	0.768667477		
	1.511			
0.98185095	0.785	0.456931249	-0.761430581	0.364104299
0.662758839	0.698	0.016654121	-2.579312988	0.999748734
	1000	0.001		
	0.001	0.001		
0.580964921	1.026	0.954464845	0.056997849	0.051673362
1	0.761	0.032331813	-2.286237025	0.997910561
0.48590631	0.923	0.311989768	-1.031789349	0.588294707
	0.602	0.532460789		
	0.872	0.8882416		
0.05014839	1.02	0.896467769	0.131244254	0.058909308
0.999919923	1.261	0.13321104	1.646105195	0.935530865
	1.375	0.897641063		
0.055802604	1.363	0.43501045	0.781151771	0.379839157
	0.82	0.264109648		
	1.814	0.24240304		
	0.676	0.436358734		
0.919747946	0.942	0.794085642	-0.266456615	0.087248937
0.12483861	0.841	0.123127088	-1.622276799	0.928965149
0.694543337	0.573	0.068769754	-2.083799153	0.992637011

0.970614059	1.522	0.069860938		
0.815168528	1.256	0.234142865	1.255810317	0.756732277
	1.703	0.082046961		
0.562885709	1.019	0.872535499	0.162314538	0.063662583
	0.762	0.814626833		
0.431107521	1.311	0.317748151	1.05494989	0.607206191
0.872058937	0.835	0.112031004	-1.6624485	0.939754129
	0.001	0.001		
	1.033	0.572769306		
0.986731784	0.502	0.02234267	-2.799432044	0.999959408
	0.991	0.822860724		
	0.555	0.552965201		
	0.364	0.461437969		
0.477461464	0.951	0.561485569	-0.588841899	0.238621102
0.537913141	1.115	0.495575497	0.70083042	0.317293749
	0.973	0.72735594		
	0.832	0.391703008	-0.872886254	0.455298124
0.404764315	1.153	0.433999445	0.807361997	0.401063884
0.428688388	1.504	0.04778452	2.121894682	0.994117369
	1.247	0.647957318		
	0.61	0.250197821		
0.798883465	1.135	0.978508604	0.026828122	0.050370439
	1.722	0.306886155		
0.876295443	0.78	0.058790251	-2.147057558	0.994944086
	1.527	0.496415175		
0.688784234	0.787	0.275110686	-1.125099261	0.662788284
0.18935124	0.901	0.425679118	-0.817854132	0.409647961
0.78163392	1.167	0.202612406	1.330541156	0.803285439
0.074820591	0.899	0.270034285	-1.168531312	0.695577757
	0.843	0.729685928		
0.48952718	1.143	0.139938828	1.541854029	0.902984298
0.073695864	0.116	0.121019899	-1.721738985	0.953280463
0.99999499	0.765	0.37446721	-0.941361618	0.512872442
	1000	0.001		
0.696040794	1.24	0.675003917	0.415542338	0.142406937
0.28615573	2.548	0.004660057	3.248140789	0.99999947
0.163149063	0.967	0.696201527	-0.389814918	0.131046492
0.340466166	0.207	0.140049951	-1.606964422	0.924481964
0.70848656	1.631	0.201061232	1.29877664	0.784194845
0.497681957	1.123	0.481836713	0.716096612	0.328844313
0.72436211	1.064	0.445303808	0.785089366	0.383006166
	0.664	0.279198808		
0.672333206	0.811	0.189692009	-1.329150098	0.802471491
	1.501	0.027567137		
0.30895209	0.962	0.697936685	-0.388648781	0.130549859
	0.936			
	0.968	0.643825366		

0.632070702	1.077	0.547470611	0.629035214	0.265532475
0.986665162	1.026	0.784934177	0.275668923	0.089916246
0.109124239	1.501	0.033337996	2.30066462	0.998101969
	0.604	0.160587467		
0.991759274	0.577	0.014435072	-2.604803668	0.999794458
	1.054	0.583620852	0.571724462	0.227652482
	0.696	0.185745229		
0.935318803	1.246	0.18489139	1.366965898	0.823867914
	1.699	0.006416372		
0.505992826	0.254	0.232506533	-1.289785712	0.778601884
0.050054268	0.902	0.665870475	-0.452488437	0.160069359
	0.577	0.047803763		
0.893744956	0.923	0.589317081	-0.547611532	0.212721969
1	1.325	0.117059461	1.614392761	0.926683099
	0.947	0.523970131		
	1.388	0.390336384		
0.413880073	1.137	0.652669317	0.444502426	0.156117367
1	1.356	0.166137054	1.482982559	0.879945975
0.794409544	3.073	0.527376332	0.615466432	0.256270904
0.769695026	1.198	0.342346876	0.971769452	0.538424365
0.902523175	0.991	0.850560578	-0.188072534	0.068388896
0.115419614	0.822	0.098577988	-1.721237784	0.953177314
0.07808943	1.185	0.316423075	1.043726184	0.598070367
	3.393	0.142946559		
0.999999998	0.838	0.334521952	-1.005371185	0.566471061
0.098922993	1.104	0.216924778	1.266945155	0.764025937
0.37923999	1.052	0.739323679	0.332287092	0.108435252
0.254053907	0.885	0.500176646	-0.698819713	0.315785606
	1000	0.001		
0.999294543	1.132	0.315375623	1.040760437	0.595647069
	1.211	0.25618581		
0.219266269	1.813	0.009647583	2.825231987	0.999967644
	1.125	0.669936175		
0.109326331	0.986	0.94636577	-0.068200855	0.052396835
0.999995526	1.612	0.008450576	3.005947437	0.999993885
	0.155	0.27246102		
	2.086	0.15179755		
	1.574	0.25573169	1.164917062	0.692903857
0.999999968	0.701	0.018862233	-2.547619903	0.999678652
0.534679378	0.996	0.977087811	-0.029148965	0.05043732
0.625713283	1.069	0.730754279	0.348650231	0.114475139
0.069039184	1.092	0.764509158	0.297393592	0.09658798
	0.785	0.154926848		
0.727727971	1.211	0.778458459	0.288393793	0.093758882
0.633776987	1.15	0.520808988	0.633398216	0.268547632
0.050938544	0.972	0.80382089	-0.252015818	0.083260471
	1.377	0.55329596		
0.605387756	1.104	0.450393978	0.777251837	0.376710486

	1000	0.001		
1	0.55	0.000438967	-4.426983228	1
0.289079221	0.923	0.696592036	-0.397961747	0.134560324
	1.344	0.859957215		
0.082861233	1.134	0.762821369	0.296270456	0.096229869
0.304929522	1.168	0.330398102	0.992869377	0.556067622
	0.086	0.134575953		
0.093674807	0.85	0.565152405	-0.589454485	0.239019212
0.054517379	1.025	0.987554909	-0.015689915	0.050126682
0.468191958	0.723	0.079697264	-1.799075408	0.967137007
	0.913	0.473126034		
1	1.9	0.000800147	4.072462708	1
	0.901	0.691951956		
0.082887909	1.031	0.676839442	0.435000802	0.151511615
	1.343	0.711178782		
0.134682857	0.939	0.519630519	-0.636827679	0.27093013
	1.285	0.808910294		
	1.066	0.879323985		
0.325713669	0.855	0.201525396	-1.316867172	0.795196202
0.139385111	1.038	0.663645406	0.443537156	0.155644691
	0.58	0.105347464		
	1000	0.001		
	2.415	0.035097504		
0.999925204	0.604	0.004464314	-3.168389898	0.999998782
	0.867	0.649131594		
0.834928918	1.166	0.26056489	1.18141723	0.705025207
	1.131	0.520469038	0.660190512	0.287446069
	1.294	0.081243522		
	0.678	0.167089089		
	1.005	0.873089748	-0.159554741	0.063198619
0.165166553	1.107	0.498418381	0.675974653	0.2988762
0.586992484	1.064	0.721773751	0.35651493	0.117488689
	0.191	0.165834274		
0.998458114	1.043	0.923008873	0.094348387	0.054592876
0.998747862	1.199	0.64120682	0.47049033	0.169247784
0.051656021	0.996	0.806037421	-0.244505651	0.081278769
0.483582126	1.041	0.819779439	-0.225508434	0.076546925
	0.458	0.29498723		
0.345879755	1.141	0.276387014	1.119752754	0.658657925
0.496906883	0.837	0.925042841	-0.098019459	0.054958293
0.999968053	1.216	0.244463935	1.188412315	0.710096312
	0.734	0.177541178		
0.053873559	1.058	0.636601125	0.478722597	0.17356909
	0.942	0.688329696		
0.986339548	1.053	0.830813914	0.216838684	0.074520523
0.999494641	0.993	0.962935535	0.047815152	0.05117727

	1.182	0.474105307		
	0.697	0.113999906		
0.917876261	0.884	0.184148154	-1.336885559	0.806971839
0.087154417	1.351	0.012996755	2.775449019	0.999950005
0.987724561	0.658	0.001488214	-3.648114021	0.999999995
	1.134	0.384206133		
0.053484835	0.857	0.296768386	-1.057050061	0.60890927
0.94470403	0.789	0.127103721	-1.588514709	0.918797168
0.958637299	1.296	0.007482817	3.053596276	0.999996147
	0.774	0.836856016		
	1.01	0.953653915		
0.861440066	0.989	0.973351027	-0.034235379	0.050603318
	0.811	0.287760309	-1.088901882	0.634464003
0.239515405	0.891	0.259111397	-1.138873154	0.673337725
0.989875002	0.657	0.054644076	-2.066828778	0.991877872
0.947251786	0.724	0.113589387	-1.706857204	0.950139056
0.372874502	1.306	0.177017547	1.372339061	0.826784191
	3.107	0.484911192		
	0.694	0.174383803		
	0.943	0.641135622		
0.069331631	1.005	0.825705431	0.228719965	0.077318677
0.995534468	1.209	0.158717114	1.511297188	0.89146801
0.608455716	0.919	0.56150593	-0.584510204	0.235816915
	1.576	0.450836682		
0.142721695	0.926	0.544007622	-0.608182251	0.251372323
0.051565176	1.057	0.777148103	-0.277664451	0.090506691
0.96113746	1.128	0.404373749	0.844084541	0.431292105
0.608272155	1.02	0.929997895		
	1.154	0.547146961		
	1.731	0.021424336	2.566974751	0.999723343
0.999992144	1.173	0.042661908	2.15113468	0.995067819
	0.473	0.107856713		
0.969386251	1.047	0.978966554	0.026598381	0.05036412
0.999693943	0.567	0.008457978	-2.90531053	0.999984275
	0.642	0.132757032		
	1.753	0.197679401		
0.999413078	1.216	0.110874763	1.706613421	0.950086225
0.822176764	1.212	0.284370871	1.157621839	0.687475331
	0.607	0.762331057		
	1.11			
	1.939	0.227470889		
0.222892557	1.106	0.593472245	0.540269861	0.208299635
0.925396196	1.322	0.06303715	2.016913488	0.989232062
0.999932378	0.901	0.611012953	-0.527917367	0.200991364
1	0.542	0.001202471	-3.824210675	0.999999999
	0.717	0.532760945		
0.074743218	0.87	0.327924811	-1.026458657	0.58391088
0.700327544	1.451	0.010419919	2.84657125	0.999973234

	1.556	0.217114222	1.279708236	0.772235372
0.873436788	0.754	0.087439084	-1.80332827	0.96778818
0.194939111	1.197	0.40383952	0.836659492	0.42514107
	0.001	0.001		
0.866190639	0.862	0.384182044	-0.894263555	0.473228163
0.053993392	1.185	0.245464794	1.199838561	0.718290411
0.889029801	1.023	0.876147442	0.156790696	0.062742133
	1.853	0.071158084		
	1.044	0.780027229		
0.851043647	0.9	0.228467611	-1.235398857	0.743050813
0.320411949	1.074	0.9579167	0.052595591	0.051424648
	1.105	0.595324019	0.553225883	0.216143048
0.242646982	1.013	0.976055303	-0.029989166	0.050462902
0.054157407	0.885	0.199310884	-1.320848178	0.797571504
	1.055	0.500421065	0.71681135	0.32938933
	0.771	0.350421966		
0.825294537	1.043	0.344438552	1.010925041	0.571078341
0.475543339	1.113	0.134849614	1.589706067	0.919173754
	0.965	0.829155124		
0.999999992	1.306	0.094998216	1.826289482	0.971122226
	1.695	0.140051032		
	1.163	0.128116475	1.689665931	0.94630259
	1.321	0.616766642		
0.754430164	1.243	0.028119021	2.425833577	0.999204066
	0.831	0.103456713		
0.998238556	1.503	0.084685055	1.772994694	0.962902215
0.999804044	1.096	0.338824176	0.983022731	0.547845837
0.452683538	1.251	0.337916084	0.978462538	0.544030916
	1.181	0.345245249	0.980953683	0.546115463
0.289960692	1.209	0.350987155	0.938700425	0.510632136
0.587964248	1.02	0.855667729	0.181384592	0.067092737
0.396561895	1.033	0.601171624	-0.518669358	0.195629632
	1.173	0.34542532	0.995351853	0.558136761
0.058126536	0.845	0.172965275	-1.461209986	0.87051717
0.995281588	0.581	0.003061897	-3.33222801	0.999999786
	1.016	0.91436547		
0.471224167	0.993	0.867879556	-0.166549216	0.06439041
0.27117946	1.436	0.124305128	1.587329555	0.91842123
0.166013184	0.985	0.606439556	-0.501915544	0.186158496
0.880512986	0.929	0.782294281	-0.285301363	0.092808098
	0.56	0.059564162		
0.999941002	1.438	0.003602485	3.46940431	0.999999954
0.183357743	1.02	0.831764881	0.217127256	0.074586636
	1.215	0.491337944		
	0.808	0.402047338		
0.396969199	1.507	0.064924056	1.971551925	0.986205565
0.569335674	1.233	0.008769043	2.95608621	0.999990185

	1.233	0.298810463		
0.99990596	1.176	0.374558232	0.906727952	0.483710228
0.563220711	0.524	0.084173042	-1.812087708	0.969095802
0.997914773	0.916	0.347709098	-0.986943123	0.551121978
	1000	0.001		
0.984007354	2.536	0.038415812		
0.999998282	1.071	0.368010722	0.948857947	0.519180728
0.050408924	0.925	0.31966034	-1.011957795	0.571934022
0.990447006	0.934	0.461908663	-0.744483515	0.350765476
0.48008308	0.932	0.95535605	-0.057553572	0.051706184
0.994090092	1.12	0.237396768	1.229642922	0.73912138
0.53929572	0.946	0.585756128	-0.547016256	0.212361228
0.937343918	1.297	0.293635292	1.072589451	0.621443344
0.2469166	0.954	0.467979943	-0.716826677	0.329401021
	1.469	0.229536188		
	1.497	0.025021484	2.469872332	0.999422589
0.999807162	0.495	0.034412715	-2.281496914	0.997843951
0.664791393	0.765	0.033135966	-2.239737982	0.997168477
0.411595828	0.862	0.185719532	-1.338728262	0.808034562
0.941775753	0.983	0.875625183	-0.158576966	0.063036202
0.07177751	1.61	0.27674238	1.111805408	0.652483032
0.654849288	0.925	0.326616529	-0.96746424	0.534813904
0.35766949	1.269	0.391689907	0.867522429	0.45081218
	0.899	0.906010267		
	0.817	0.182363359		
0.146147719	0.828	0.214442282	-1.313681793	0.793283706
0.994368797	0.916	0.198053968	-1.294984437	0.781845914
0.565018331	0.872	0.314990163	-1.037342492	0.592849704
	2.692	0.129849485		
0.155183985	0.901	0.246526267	-1.168991766	0.695917663
	1.432	0.073990265		
	1.277	0.363046949		
	0.646	0.087443667		
0.934909511	0.974	0.960817344	0.051047262	0.051341938
0.39331949	1.098	0.727839585	0.347297771	0.113964152
0.163181626	1.191	0.045757204	2.230340148	0.996992295
0.062776509	0.907	0.440339191	-0.759000305	0.362180672
0.052220701	0.94	0.668757962	-0.43780857	0.152861729
	1.16	0.684263205	0.405034436	0.137673675
0.364463993	0.948	0.882961119	-0.149251523	0.061538617
	0.43	0.013247158		
0.09221104	0.931	0.251996188	-1.18534357	0.707876669
0.500057113	0.963	0.611426748	-0.514935104	0.193491475
	1.603	0.02686629		
0.86090538	0.904	0.648800936	-0.460938078	0.164331012
0.214966623	0.992	0.85833927	-0.178989042	0.066640269
0.296185228	0.886	0.637549673	-0.486055809	0.177483643

0.989374513	1.508	0.071279273	1.978842743	0.986736674
0.615755129	0.902	0.320382059	-0.999524226	0.561610801
0.055803269	0.923	0.854160535	-0.188403153	0.068454233
	0.623	0.174411101		
0.093253713	0.907	0.275247547	-1.078794602	0.626413588
	1.001	0.854811205		
0.855791464	1.283	0.384012105	0.875022335	0.457086235
	0.882	0.302387263	-1.064356962	0.614818073
0.117955402	1.142	0.424907721	0.83479882	0.423602479
	0.754	0.048753514	-2.043568895	0.990725923
0.747512545	1.468	0.101587615	1.792526868	0.966113123
	1.246	0.59697979		
0.999999951	1.257	0.016016856	2.730419408	0.999926546
	0.923	0.955181955		
0.100844122	0.854	0.160606707	-1.399492375	0.841046834
0.999649514	1.005	0.870050459	-0.162158479	0.063636129
	0.842	0.47333745	-0.750196396	0.355242094
	0.673	0.011894832		
0.999999979	0.613	0.004734321	-3.253556622	0.9999995
	0.983	0.760819049		
	1.387	0.250631897		
0.136007303	1.134	0.455720313	0.755857386	0.359698222
0.990971377	1.06	0.529126244	0.63482329	0.269536322
0.44262455	1.027	0.852315963	0.189572837	0.068686338
0.208841613	1.566	0.483769835	0.709480669	0.323817189
0.328113609	1.26	0.028242878	2.389442616	0.998968509
0.094374562	1.069	0.766946763	0.294245845	0.095587965
	1000	0.001		
0.978478217	1.267	0.036000891	2.259465966	0.997508363
0.558057259	0.764	0.110872183	-1.757823138	0.960239898
0.186629665	1.255	0.783934247	0.271795944	0.088783173
0.99945015	0.431	0.000734105	-3.957390117	1
	0.996	0.966489609	-0.042534495	0.050931459
	1.151	0.625846179	0.48557183	0.177223402
0.270815695	0.875	0.080683013	-1.864496903	0.976031677
0.973606384	1.303	0.507161937		
	0.624	0.239864326		
	0.701	0.059780848		
	0.832	0.179810363		
0.999935431	1.168	0.401656889	0.843429628	0.430748863
	3.601	0.080595666	1.779893254	0.964063594
0.998231721	1.008	0.732017127	-0.339094355	0.11091022
0.471177587	1.029	0.698404533	0.399149495	0.135079084
	0.949	0.683798312		
0.969070406	0.851	0.156696869	-1.464391808	0.871926269
	1.506	0.0851067		
0.957736235	1.72	0.0140715	2.718861678	0.999919031

	1.36	0.00325649		
	1000	0.001		
	0.001	0.001		
0.451694292	0.938	0.562375459	-0.590140555	0.239465531
	1.196	0.795253939		
	1.295	0.29247765	1.088181616	0.63389223
0.986563353	1.23	0.039791863	2.256697812	0.997463011
0.23391891	1.535	0.059587293	2.08782536	0.992807656
0.963726524	0.564	0.034269085	-2.505461015	0.999557085
0.999997862	1.4	0.173056545	1.430840793	0.85652594
	0.431	0.654989264		
0.404762557	1.002	0.874909659	-0.155424014	0.062519448
0.954931432	1.068	0.366023009	0.937162319	0.509337134
0.980214657	1.061	0.907801287	0.114191462	0.056736242
0.264958627	1.229	0.127389382	1.630673899	0.931335212
	0.816	0.066524041	-1.918126283	0.981724016
0.999999889	1.831	0.028304705	2.441931811	0.999291529

Intensity N_ZQM_1	Intensity N_LGR_2	Intensity N_SFM_3	Intensity N_LGZ_4	Intensity N_ZXL_5
				250964.2545
94842703.83	133407986.1	114806816.8	148576230.8	102322619.9
5422.980619	354630.5747		408769.6762	
4302867.505	2727487.466	3414752.313	4932501.029	5280229.391
264302.8826	1034610.801	4351692.335	2029239.971	252916.4519
		558323.7502	3878270.837	2974303.515
117136029.3	122717107.3	45785536.77	91466940.39	65577671.5
1373288.018	3353915.023	6265964.877	4516975.786	1134260.33
2116307.531	1556049.257	1714187.808	2545571.963	1542987.448
12862395923	9413873450	13068696570	9754648582	12614474899
33120.60311	23778.70471	28542.90825	59131.94346	65559.33569
1392536.457	1021155.104	660183.3236	857442.6008	690251.0524
5432751.542	1587359.313	1457325.087	3615669.389	5391239.412
31845847.6	27928950.31	71807803.16	22509045.13	6935489.591
		103027.2222		
		1085126.169	564019.5566	
15782929.57	18463913.18	46609424.3	24056540.71	7233423.743
2144642422	1646188616	2476976722	1337428765	2212842304
560405.624	306959.0952		1543816.815	729646.1567
25506700.29	44920122.59	76270830.38	76151524.72	87954663.08
2199340.871	2343745.875	1752001.015	3123094.437	2295382.346
2702205355	3299984396	674879581	2766152123	2365724976
	13576.99688		83633.90136	
138937664.5	93276811.4	132158791.3	86637303.41	100840499.9
				1243768.395
42195537.49	40751974.75	59387814.86	49023923.73	24383795.4
6063548.715	5589773.339	11488221.25	2191543.451	5119815.607
7578434381	9031594341	12904804552	7853896584	9437720777
	322890.5612	510591.4812		
2121633.074	737240.3455		1545190.398	
990718.54	488492.5121	302577.9607		
	2663725.174	7515109.921	1312050.808	
21884.57819	7221.900267			
5807286.66	7650211.422	4277676.21	4375912.206	2783203.934
33137359.03	20998837.84	18662979.88	38076696.03	21907595.01
425264.9495	303092.1926	516773.2247	1567789.033	
22395134.14	24572780.36	17700011.31	16629398.36	18378819.2
840731.5803				
30021561.21	16830029.83	43684649.76	14565796.63	35670901.27
	3313982.826	1547141.918	5041130.268	8088489.814
59702448.23	70798544.66	90629625.88	68883038.95	87488536.49
628395.7435	771741.1833	868366.0565	539020.8221	1011623.057
197656.6451	132644.2462	485441.4759	144580.2576	
158307.9862			618724.2995	

976360.5911				
3740458.761	1832666.331	4003910.071	4381955.923	3012620.97
750499.8118	960136.6492	1286079.944	1449727.065	
	741196.5711	87989.5458	839342.262	282097.8226
1183858.848	501171.199	984063.8185	620861.8437	480434.1782
330192.9778	148347.1833	105524.9701	562662.3556	351443.0876
963525.506	1288163.011	736673.5815	1199578.41	604191.2111
6763529.41	5065488.44	6892362.811	4373966.52	5626777.283
2887257.685	1777611.131	770562.9526	2301151.606	2151410.531
2506335408	1866719588	2857797316	2047761242	2083412557
2766779.86	2522391.011	3186003.534	2488623.388	2553130.467
1298903.348	1537425.262	1329875.101	2965680.579	3691747.898
2891784915	2103011491	3936180680		149498.1728
			927130.5818	
50118746.6	31912795.18	27281461.85	28298648.58	29513971.19
3735547.309	3602525.345	17658364.84	5527255.713	3705131.911
13771942.2	13940231.87	42154075.48	28035470.87	1942730.94
145339409.7	160911029.2	275132261.9	286295917.6	55849373.22
5759108.083	3913239.3	3079400.695	2326532.032	4493217.456
7164432.521	7285910.223	5088131.879	16522469.66	9008245.497
	36043.0143	100585.8522	40259.91001	60367.33974
445288.6738	30065.19239		90071.78086	344278.7431
195072.8776	458628.4022		289145.0329	459569.9233
3249536.174	3104048.006	1300442.273	3359958.564	1336654.513
2776078.957	1952699.628	1350877.779	1964375.573	678870.827
3283057.808	3194249.175	2074442.88	1832525.23	
3306545.568	3272934.224	4334808.13	2979267.847	1355088.059
2168105.092	2818299.56		2383239.412	1521987.48
2593022.882	2790934.946	2005083.171	1551947.976	3792098.024
10203540.84	14320173.12	13363392.59		
219220.1839			237279.8668	337768.6396
30717002.41	24801428.98	18974276.18	43517112.88	43289910.79
6507756.914	694260.0083	2970610.761	1948378.345	21474369.24
774492543.6	709715792.7	13003983.82	10915561.32	51776036.03
12593657.8	35215227.64	64704312.81	37635918.54	15478222.47
8440036.849	3565843.296	7454196.176	4404749.86	6506248.912
			671876.6432	1490436.91
102256.7767	283791.9092	22878.29676	1471750.545	100628.5186
495517.6607	448821.0852		571524.0079	371074.3074
199981.8788	158126.9282			
7187501.04	722214.1674	11806373.55	17221769.87	1751821.144
1201420136	1877699704	886240433.5	986367090.6	1316932860
	2057007.422	580697.7464	2339503.961	

944101.5821	997184.0633		984342.3869	
705295.905	479396.6684		1370782.153	
	107129.15		28548.13724	
34534311.26	38410263.79	53096918.19	29553511.15	24832403.04
447211823.4	428818924.5	296165711.8	296755249.9	476674431.1
13999709.05	13674549.55	14677874.1	14929453.25	37684880.37
7820807.585	8339008.34	6718468.649	11398849.24	7651342.777
7221725.763	14523693.25	14776491.2	11035270.8	12451956.97
547189.1432	470009.963	783542.0066	632024.3424	684951.6679
	421609.8545			391974.5666
24878553.15	14715302.98	23328853.5	7310047.25	17826775.93
23642887.1	27875683.89	30706619.67	29196210.84	13070053.78
		395443.9842	73033.56515	
12255622.63	11426789.86	7069798.457	13741961.08	18042153.07
37359.02884	28974.7497		45484.88729	
12403478.94	15120087.29	20749912.53	16038804.59	5661851.315
	74185.40362		275364.8801	
10382563672	8728066023	11557460530	9906159263	10469294192
101394.0241	97454.56261	88185.29914	172287.8211	
23593.70715			42049.86677	
4017275.695	2101421.515	5487539.282	3724656.262	1504031.053
612211021.6	1224907843	857786474.3	922285962.5	922147851.1
558619.2767	242177.9497	588684.8316	1044825.794	
2633487173	3770912595	3579470769	2486385067	2323082539
89865.52832	74811.50213	35566.37237	136009.395	
585328844.6	895419044	981980643.8	1400132841	1146537744
78501.97179	29796.01076	88010.93079	57477.42012	103546.273
286639.4401	253042.2429	974391.2471		183226.1779
355063.5927	548947.5695	924893.6456	342676.4987	859941.9019
1138229.381	480095.5149	1892363.19	936529.23	578781.749
4601106.02	3969376.798	3325640.223	8435277.654	3297334.021
8267765.474	9272574.407	8071368.573	10487183.1	11426188.11
4839480.66	3612701.175	2950175.689	3917317.676	3125209.376
325231.4923	336411.179	418419.8456	642822.9765	472825.2451
12742515.35	21553110.61	33837568.39	26345174.48	7666857.858
26024139.84	53502739.82	59702311.78	25272341.68	52199772.2
16142.78949	24450.94991	42511.39864	91812.59276	
3575629.796	427912.6028	10467524.42	4308926.357	3657616.563
541099.7176	715905.7203	1613298.486	1027594.548	885477.4116
	387335.1431		312235.341	
269160491.2	291858631.1	323938581.6	192482748.9	256776435.1
2483337.013	3474582.916	5869959.717	7634233.431	2384883.813
148858683.2	207984631.2	60363895.05	142284969.5	158977193.1
5334931.648	4946392.253	1058523.69	1950965.002	2958510
9799663.578	6416553.7	2449810.551	6744250.834	928487.9029
6161783.841	4127114.759	4033689.189	5607392.265	6384947.699

70994.64587		1200988.85	193167.6868	98982.82031
508988.8959	348343.295	507266.7732	484432.283	3652.846201
409732.5504	380430.4835	564460.8665	507228.47	310902.1047
16378875.34	1445014.453	32242513.02	6702273.162	8138828.681
275727.7528	143072.638	707999.4682	365646.9319	200010.5898
17462346.34	19153824.64	14234036.43	25742566.32	16025395
1318195.841	1065004.004	932779.7129	1342840.284	1028833.212
			519962.0944	
108025629.5	120616389.6	120832320.5	67706637.19	85039499.11
150390.1743			52113.48014	
24421566.86	22988310.63	29200863.13	27165194.72	21509851.19
	510981.6448	476449.4688	340773.051	
5596517.344	7712876.801	5103748.468	3738238.243	4596912.072
51632211.39	68030902.66	115959556.8	79701505.43	30935780.69
952664.1775	1403167.765	627762.5035	2460784.498	930542.064
1425877.413	2081622.202	155658.1152	1809984.862	333666.5453
829401.6665	858714.3051	284143.5727	3669746.684	640386.8676
2754731.759	4775776.849	4670204.264	5774366.156	1784557.846
365192.977				
268261.5803	1092775.243	334586.143	1212446.974	1104830.7
3939155.075	3903831.606	2140025.19	2076415.176	2609837.106
372864458.3	536871328.8	489103811.7	524116271.6	310861191.5
1394038955	1428520251	2022814547	765952436.2	1010647511
46588.61432	43570.34798	130615.8266	160310.4332	49069.19036
110530849.2	117691187.5	181372652.3	128838663.7	87091260.14
18748278.42	23250325.53	15940108.44	32580019.65	10684203.94
135033.9255	33638.52365	51924.76212	77651.6878	136514.2297
623116865	753145433.4	572652527	346579812.8	719611685.7
2555748.755	4935648.398	8184086.5	3404075.803	2338353.488
299938711.1	182122847	493656550.5	471718305.9	478488957.8
20483330.57	12515053.68	18787122.64	20423486.31	28176788.67
5470592.751	8959172.012	2814173.368	10756133.81	1678469.197
8985349.66	6476328.488	12258300.73	12307187.09	12474025.23
961119.0089	710262.2808	1215760.58	1587935.5	559907.6258
8988608.541	3659918.431	5537973.956	15728166.86	4764478.589
62890945.33	90057880.61	159189467.6	51716830.13	57755466.41
49603.66717	47692.69513		235486.3366	
163091263.1	121716638.2	121276819.1	152665601.4	101155643
2444156.836		4930179.694		
146973327.2	122947201.5	13516890.2	128074875.3	92047200.36
412211.7325	358908.8412		765466.6255	
502399.6143	914332.3203	2428880.666	1451249.592	574340.8895
1863424.976	2716681.446	6558323.239	7614896.569	2579381.198
1257127.952	1425794.861		1468859.639	1673555.237
131473128.7	84280515.26	42986727.85	137363944.3	34735279.08
1012827.37	955262.211		1458363.069	
659121024.8	970638769	207831207.7	368863853.3	548287237
7111907.563	8074363.197	16539560.98	8231294.775	4806432.158
6800377.681	7511834.967	7746392.034	5379198.119	3197809.336

983460.6107	442391.1152	13548.40211	1036017.935	19154.07768
1085829.075	983846.7752	228581.4747	2879013.019	
15782415.57	34875871.22	41179033.91	37724818.14	35754231.34
348579.8334	422944.4584		904125.5806	
338527.6184	155788.395		520037.7068	
46947194.53	36024894.37	77953935.87	91844515.46	45550943.38
6275826.872	5437938.115	3125522.259	9842580.986	3051997.829
740624.6147	5522685.366	1420436.56	753767.163	652385.9055
	79602.94943	203855.3733	295672.0798	1546394.042
231667253.1	281101615.4	863482642.4	403580199.4	200503881.3
1442823.055	1457434.236	188375.0774	1278894.671	791919.91
			417392.0304	
637087.1845	4250947.419	4575114.181	2263697.445	3381838.832
		655352.9754		
126119700.4	194766758.3	338655140.5	434323314.8	641326646.3
16958576.93	26882410.08	9855751.881	32690013.14	8166989.134
5831836.874	5764126.533	1815713.504	9526494.905	7471638.561
6691569.786	1224807.829		4305772.092	
807126.9397		1966101.148	765616.3085	724945.2226
1489747.756	1583488.905		2912622.896	
971784.3405	2173944.773	1390569.333	829836.4267	901444.9791
1374209.128	935297.1574	669612.5305	2237787.604	2465350.851
12136438.33	15485255.78	15985090.38	27234588.15	13482698.08
6418095.31	5981944.881	8978544.68	4833387.952	8186699.855
744461.8938	899834.4255	1087571.822	975231.9428	1220418.413
2636930.501	1887000.345	4634546.952	5798199.547	1295601.149
281403.2985	101062.8734		149569.6166	
14117731910	12808361591	14121337102	11825092973	12074741623
175565.6798	797192.9411	3054706.727	1509403.308	81960.47165
1573473.653	538798.7722	1575088.231	1611861.046	1071159.41
23336.28635	12155.93135		17433.13516	17255.31255
470479609.8	499775291.4	1304956794	710642235.5	372247785.2
		239835.9263		
19122664.96	4939866.21	5909196.939	7211122.577	4684344.835
4268070.071	2005287.601	2020186.271	1414737.985	455322.7088
41008570.74	31632968.04	34996398.76	18691424.15	14944145.72
385901.834	490950.7385	817000.7549	884943.6114	
725717.7172	1208818.108		1251236.75	644478.8337
44709064.94	35640257.37	44793401.4	42690395.2	36105911.34
6467863.892	4491475.066	977039.819	5942132.851	5096251.874
4921089.365	4903595.972	3552189.87	5524725.632	6835586.016
1508116137	1353765069	1032675199	1220323960	1427442749
3302067.527	3124744.46	2307253.812	3805060.744	1834183.648
371741.8735	233449.3333		636336.723	
	786667.8375		499327.5193	680690.7652
94146998.38	101556948.7	193409154.8	149299168.8	77451291.69
	391027.3993			

1248496494	1113232625	1644862323	1260189963	1539728288
1531240.3	2158712.247	2190000.01	4258201.544	1265473.627
5973522.185	3071182.144	4466011.359	3157141.671	6127274.879
2877373688	2192215747	2801919024	2208879692	3679801267
84024.62216			210927.0631	
1270755.987	4323469.605	2787539.506	6494490.262	1306008.284
4767768.848	3230211.417	3188095.832	5121698.598	2494655.139
2848482.886	1820296.733	572336.2666	3507414.069	1497125.283
1477930.186	1264409.864	424171.6869	2158034.757	916921.2009
208492144.1	228269134	275684958	323575905.2	301785897
24047332.02	59081921.7	145709074.6	29325311.45	32482045.9
2314571.257	266322.7294	1564658.152	2776429.593	2342167.13
396119.3365	675638.4534	1899275.646		
72147361.8	61744130.52	42554475.5	41176487.84	44780363.6
809374.2227	76480.0925	128961.2659	857767.4627	193464.7901
137141826.9	180079724.8	578695102.6	280249430.1	74439279.83
1427946.124		655981.5946	1378876.696	
12805410.09	15556427.8	9853286.163	12612431.11	55537942.18
7084683.587	7451724.582	365540.6045	5520422.875	3415421.968
9074711.122	12617537.37	22448002.75	16526685.62	6475112.359
4785922.046	2100635.111	3502850.103	2617951.893	21057261.11
1647034.934		1802526.994		1441258.227
1835493.356	820161.319	390754.6264	6707927.053	378097.0915
6705772.944	763466.2817	2792590.545	10921873.29	6359783.423
457152.376	141808.6896	109512.253	423507.0956	126900.7712
	404669.7029	674117.6077	819852.762	78361.80555
193279.7041		1466500.232		2503252.586
111210450.5	94194731.8	62782449.86	96783801.18	84955403.22
181860.5615	149165.6392	320450.3305	322670.1747	
15355098.95	9120883.366	20828613.88	7791575.916	25703200.8
48189428.37	64801958.33	52176114.12	49667117.97	53766547.97
58667768.45	58744596.41	108043650.8	126760120.9	42642462.87
21866258.64	25155130.17	28318342.51	24175058.99	23374483.95
282464.2404	609401.9192	525560.892	2196657.601	454522.5043
58844.99026	50260.07848	77458.44368	357846.3828	
1395107.625		1471964.071	1052970.317	
		519402.5431		
1242113.999	1275574.201	1350076.74		796461.906
12410804.24	12045380.22	17237078.65	11178928.51	13625626.71
782028.8296		194574.3085	200567.4242	
559537274.7	533930530.5	184596391.1	405580134.7	329481743.9
23228356.45	16340995.37	33524987.24	18615620.25	44315561.12
60797.95704	214427.811	386955.7169	340890.3884	
1168062.745	3544091.151	1692222.471	4667452.97	4280903.762
564513.8159		89742.13037	244598.997	
24319710.7	23294087.21	27603379.82	37022579.4	16851298.53
12996793.96	14421767.76	7762126.018	17452512.5	14678902.31
3411146.066	5213289.22	6992200.463	10152731.81	5398423.753
1268980.836	1584514.795	4380453.14	2269079.192	

585243.9988	1045091.054	212547.1381	1023874.652	117345.8603
1824783935	1746669196	1932014342	1863823507	1368147931
24780564.27	32467709.19	28568316.21	52904096.25	38216345.95
7668064.489	5236040.888	3070583.442	7825850.822	3072940.499
9869575.879	11665979.75	13652279.73	8931365.018	9512445.329
4356317.667	5009144.481	3084605.864	9087143.541	1661435.706
1054653.201	768950.6393	2780492.792	259030.2388	2753740.026
205717.4146	176003.2641	42334.27217	92963.42552	77607.09297
6754735.184	3435771.336	2536137.701	3766152.41	2328909.944
1729195.134	1253268.621	3278235.011	1315478.1	1523805.082
				302259.1171
658046610	1114268185	301680238.8	765906908	371306082.2
10341290.32	12179056.48	33678209.58	15567209.69	8129933.209
14716916.27	9127402.95	12158916.47	13812111.22	5874710.491
101750.5947		2136706.995	2353969.797	280105.023
743417.8019	298637.4301	496313.2104	827832.73	
3916373.908	1058318.692	2391881.966	3049288.439	4024925.62
3134624.368	2067611.722	463421.1085	1582697.429	1911377.746
15434587.34	17224989.94	37242680.31	27317411.69	12074411.68
7746679.452	7922973.503	1757551.491	12090870.11	
8645083.956	17655745.47	12253813.44	8740281.972	8156631.026
170259.8976		167699.4878		
13686205.83	10611239.78	6791396.17	18212427.66	20385207.85
11559285.09	15238851.87	44356958.08	16268443.58	12615290.98
2109873.119	2633077.742	1852126.689	3173930.324	2011286.445
65503.12985	58558.03595	64164.91255	106478.3166	33001.66014
2114229.419	1345347.299	2369105.045	1019978.991	998338.7531
561352267	630413457.4	874854165.9	866912354	339332121.3
	920772.5472			
107943430.1	100608875.9	217165148.3	126389043	79248117.87
300452937.4	355716198.6	274276818.5	228709784.3	479965842
14191737.64	19509941.3	15980017.66	17509303.15	7952286.552
1072883.565	914561.9107	5101086.98	1068006.072	936074.8605
1350532.858	1927596.204	294191.1071	1777248.801	614593.2863
44561245.66	33145326.77	45972081.16	47884773.67	25460113.79
339162.445	1529597.66	3737445.52	2156263.069	1318849.832
4675990.012	1964704.778	5468776.923	3135308.507	6449073.308
	1107375.688	859493.6104	909217.8929	517853.1957
				2831433.797
75460937.05	74637000.5	125162599.7	146461645.7	51570731.84
22826284.55	28363343.08	43868400.59	28334266.83	28187336.56
473458372	319596821.5	592861599.4	412836005.8	225309132
2322778.699	2797570.571	1431454.914	2501341.112	907612.6197
4019642.982	1005342.214	1144255.998	2854001.822	2406758.363
2411399.945	3840279.387	2484361.02	1811133.117	1126294.086
590071.1288		383703.6559		
12534527.97	12482070.62	9873284.674	9744571.404	35657214.69
	701353.4728			

1596451.928	765736.9696	1323519.081	1214638.513	670223.6227
			269037.9138	
616589.164	384685.5789	934109.0939	278583.2899	188346.076
8408706.126	6705306.964	9356565.714	10329522.75	8079363.493
1089766.421		1098211.955	1451947.77	
9729300.69	6623162.653	11898560.12	8454383.696	7038104.292
3259.222162			1508.455293	5066.917371
233348.5313	452741.8889	722424.9878	857071.7981	538287.4744
1978165.753	1307238.01	2931880.527	1656918.493	2908305.842
621882.3087	86799.62534	78781.93799	814517.269	523741.981
3779744.022	4765525.52	1157889.639	3142256.032	3229966.133
1925135.695	2786720.822	5519667.959	2518639.844	1878922.364
5145168.593	2205249.708	5477759.958	5861037.635	2450836.01
674927.3021	623082.7363	774993.6137	468728.4878	761743.5383
200094755.5	207388146.8	236351554.7	284847462.6	226262451.4
2238565.701	2121431.294	1517158.885	815023.1398	650284.7442
18568982.08	18483353.1	11304581.45	29153877.63	11829907.3
1080877.394	1131153.865	1347050.83	830813.0923	392831.8267
4846193.118	7021755.473	16884838.04	5674035.556	65875737.61
6722080.37	5695775.637	5911576.874	3870284.959	6085360.979
685230.2039	808859.7392	2905391.966	2319642.566	544361.7957
2066615.971	879078.367	198250.6997	2810716.789	2301261.34
295177.488	330592.6415		978629.2494	
638619.2201	875858.2722	2884013.473	1895930.411	969557.9874
2578295.84	2510912.422	2885964.287	2276185.345	3656208.567
839465.4504				
26618551.16	35014559.58	45236037.36	53735141.38	96281921.3
173938.2306		2140090.877	1568540.563	
25145459.56	28661222.77	63608198.83	61316603.77	77777503.2
291179.5205	693579.1335	715894.8831	396883.5937	109263.8187
354355.4683	95865.80185	370831.7805	1037904.657	
			124043.4261	
6219548.943	1640439.095	1241531.206	4989199.575	4072441.04
284137.5486	246390.6208	137390.5849	439853.2201	
19740633.8	17405359.53	18961739.6	23917528.68	18182238.23
3044029.155	3879135.068	6600218.411	3824622.644	1213764.909
6752122.982	5149972.346		4208533.57	3154821.096
2192851.907	1995197.84	518914.1087	913645.8121	1071525.486
594504.2686	806551.8416	796741.8731	1247120.242	
	177374.0276			
1832002.956	3267864.393	1424262.575	2307216.113	1585029.455
369445.3614		567177.5212	260742.3588	
502927.3663	867163.7088		419895.3343	
11957100.27	21909769.88	17197167.24	15733506.67	14578496.75
16623242.35	24030768.43	22172668.7	38822898.94	17621858.05
11307334365	10951743196	9332939910	10708958309	10134209266
2361839.662	1315965.426	1587218.503	2054898.081	2313607.58
14215507.24	24081092.78	36556676.16	21941223.58	32274700.44
2751815553	2530299552	2606725962	4135639833	3508405781

3282928.599	3550764.767	3027415.137	4324544.142	2698028.923
601617.5613	936320.0341	1724872.802	1138131.86	372919.8825
	699755.0558	322373.7007		726454.0964
1559536.296	4082260.393	4618397.845	5193216.566	431797.0197
9370664.795	7944287.083	3997877.778	18907323.02	3334386.995
374130.4099	604229.9149		924539.4596	
39815021.86	28106574.84	938206.9835	77268541.61	11184916.76
393067.405	302123.934		483951.8612	591954.5365
6755558.636	5356080.224	7086889.681	7247219.312	6433380.217
85718116.41	85723516.86	65713681.44	103088613.8	79840139
580937.852	8899222.713	10694060.29	2806169.617	4425480.515
26913375.86	21436873.77	7013171.738	33924647.52	12419710.82
12840767.22	9939355.602	7017900.55	7550084.914	5687117.83
304093.5328	182421.6643	126116.8695		
2972185.567	3153546.081	4605098.036	4910537.551	1980263.388
2689075023	2962284171	2645641648	2508054200	2612490160
122749916	96530444.32	185168907.5	155969806.2	98761710.99
328122.0844		391055.2608	405225.6597	
2886578.761	2347504.3	2692948.515	3240983.755	4370566.34
	811662.0533	1370161.375		
2826161.592	2773382.435	4177325.298	2943326.191	1473991.879
484182.8067	476724.2159	1018813.542	371767.2649	4216654.83
22705707.49	23666390.33	12897060.34	21853050.95	7243287.246
1481871.064	1027536.11	1290061.327	1432317.902	1319373.038
919558.1458	1146950.534	540720.3421	1596270.981	852819.7896
479564.3161	2055390.307	772005.6832	563322.342	1294253.354
40627592.42	44721783.08	68034615.44	47716344.54	33429007.91
1468346.562	743608.3202	441835.1801	3381389.88	770504.144
4045600.752	14283190.09	15224963.26	12563434.14	26685049.76
		5676575.924		
295400.5217	304668.3499		507879.7839	
13023.56057	5811.672438		35920.77675	
4552674.079	3118347.887	2843416.624	3727531.332	1569350.117
38224.64639	146892.9657	717693.6174	438217.8046	416829.2375
553064.8026	282172.4196	293250.8578		400784.731
4821549.538	1653317.612	5352956.799	2378569.558	
7575760.927	5569347.195	3304637.687	15371979.58	2993749.88
552746.4511			207172.9744	
			556850.7036	564195.3858
13609782.62	23442857.6	10175002.14	24088716.41	7615572.483
177759.9425	211414.977	319231.173	267540.5215	346388.9866
106418592.5	84974404.85	128879257.1	149357822.4	97300181.96
257582.6921	301691.7944	369037.8368	468010.9408	346223.172
249181.7426	139900.8944	176297.39	407303.8606	133660.1155
6527925.382	3905300.487	39040949.95	8596540.036	2852857.474
2318305.923	1372468.28	2080118.072	1925444.819	415654.231

1352519.099	1477698.272	700605.1947	2469629.512	466015.0892
11221621.32	7239699.062	19809242.39	10204549.35	6087783.634
99199514.3	30749899.18	13964766.32	36884443.35	12159156.17
8305010.573	9066049.91	12709918.24	14130854.53	9522098.481
	641708.8353		423247.72	314341.2651
42283807.05	51085318.54	35903821.79	36030187.7	34229793.36
42909859.31	40774047.58	65645933.75	69748367.65	29580397.71
89245.80937	112848.3032			
1821362.134	736436.239	1561854.506	3091840.141	
		877444.4967	588633.6121	
44816942.14	35405655.45	68896248.1	85481369.58	38334017.83
447180.5782			877304.1241	
1582556.763	105282.0162	173747.8466	1660698.246	197114.7219
871848.2308	1248457.875	3070553.501	1224664.192	740378.0446
12346.63992	2105.59089		483240.6058	
23259124.21	25568826.25	32728345.17	25557877.93	12547771.72
454018465.7	423504681.2	684534138.3	816634561.6	1078210950
13796677.02	6377750.622	7529870.293	6698294.838	12733469.06
6025010.232	7311712.177	8392648.361	5950997.836	5748831.992
97612908	137387441.6	93933153.27	108748609	104284017.1
1727562.132			590827.5283	3328959.097
234592077.8	254400712.4	494209616.8	380754059.2	159692122
57235689.6	46511436.83	39207175.78	59280171.31	26562750.87
839976.965	673581.8426	254209.3994	808731.9561	
1518086.902	1155720.485	3696543.821	2388180.3	667972.4109
1286334.068	945560.7266	983549.442	2119669.393	1525980.053
11706233.51	11540950.4	8859419.311	7855499.848	6363369.747
2153425.451	3257265.987	2500243.317	3446667.046	2355301.87
15215584181	15688691934	17880359787	11325095129	13248091593
5128814.791	7781735.735	10103088.89	4724459.732	11345393.79
3521175.141	96153.38581	3186819.148	4208655.405	4270395.101
8448244.731	6092479.132	1860398.813	13767594.06	3610675.874
348088.8488	185001.707	129201.0477	1451387.358	
2238327.502	3262228.999	3604722.14	4461490.678	3263537.332
6744345.025	16476774.6	12484914.67	6956751.865	11307131.58
108664.5251	87692.50072	152729.539	204038.657	132649.9992
20617222.42	27417545.22	7081362.387	28153132.5	16586833.5
718993.0821			499451.3772	
2478729.054	2068331.995	2846031.135	5260229.269	2939295.101
640361.1732	855985.6284	395962.3393	998081.939	432718.5361
95649646.81	50336896.07	116406295.4	87412687.91	74047026.37
941349.1989	341871.6985		226945.1537	
4405175.952	3336324.565	1593973.734	6481520.864	2035211.3
86755008.15	94254715.98	257206269.3	142470572.4	62075330.61

13409273.96	10644841.93	9884026.226	14341948.82	17816407.16
561291.8135	501979.3468	804379.8157	928485.181	
1480881919	1525409203	2044281681	937970571.7	1058702001
8191227.838	7820694.702	1859596.556	9270608.251	7746045.022
162443.6265	200224.1767	477067.6543	47660.18949	
316157376.3	325150823.6	68048038.31	237257617.6	93360872.32
	749517.3982	1636064.09	805065.3646	576656.3253
	37482.27732		212456.7368	205308.9688
583497.7996				
32475.26438	5761.940731			4625.911354
599779.3761	1059523.101		1527411.556	
5660869.58	3958510.587	12376862.2	9489051.553	3600148.513
29318486.09	26057283.66	39686917.57	23674190.18	15050684.75
964324611.3	1322585287	1478493616	855511442.1	705161450.3
370203.6914			636638.7551	
4435016.669	1980800.094	2675801.591	4348715.749	2434505.058
18930184.02	17495068	16214996.18	10546852.34	15730755.74
105041.8739	74207.16051	10646.93579	266598.0966	
1714132.699	1392578.448	4210635.295	2517682.001	1034408.289
33777561.32	32428334.78	60112847.6	59126564.81	21707704.03
93423131.48	108722329.6	55523642.08	144736951.1	103679141.5
1604666.302	1744599.465	1785674.933	5244359.785	6708597.221
3541787.723	1927029.417	612683.275	2542205.701	2353531.754
527551.4531		5334444.361	417536.831	412570.9622
1804322.955	2251349.368	1482515.974	2885284.288	2099430.727
			243160.5464	299517.5976
			553241.6725	
181611924.4	663470919.5	342840158.1	227731227.6	122979146
1394463.225	1253309.332	654671.9036	1688155.219	
4768706.937	5366751.354	12084417.45	5805030.682	1783990.081
9055328.328	7102321.48	15061021.98	7988498.446	3861350.261
	469783.3523	769081.7351		825637.5517
1430724.241	863043.0006	1324200.64	1252032.158	404737.6182
8985189.038	11880479.99	9505180.074	8796747.257	14293900.97
1409657.971	792186.7696	1778019.269	1692932.484	2292268.085
3207830.572	4061694.341	2080245.144	3786371.887	2250183.602
				713075.4679
5638632	6239693.858	14497673.42	19391800.79	2676004.461
3214698.663	2084777.014	4130196.721	2936082.914	2524693.981
3692429.844		3092269.607	248139.7231	4075465.859
183316.8866	1359933.208	1503089.572	1839281.844	863879.2683
26818664.95	42505198.63	8690014.906	39226888.21	21183301.05
5256225.618	5259830.889	3188656.114	4113299.647	1487368.124
7493177.283	6888413.326	8175119.909	9244447.778	10668226.87
3413787.047	3606781.325	4283992.81	4235844.49	1464683.298
6283883.955	5312777.793	2974657.381	9906159.459	3518835.017
	3977902.819	3979313.335	2830450.002	2688565.815
457607.3663	333796.8877	2263961.907	917262.7449	486664.6155
	676560.3163	4361986.37	1310540.423	1386265.102

			554162.5122	
75255050.51	61242021.36	25532172.58	129621165.1	27373453.14
227820.0792	857292.6064			117876.5026
698044.3412	282467.7324	1103080.466	497348.9411	417887.9435
1867523205	1630252405	1615705741	1060446355	1404281640
1431274.013	4132566.486	10204489.59	5269140.259	2104676.77
909154.9095	809990.9301	732759.1238	1325082.225	
6183843.442	5792968.456	12404433.22	9342510.512	3982651.255
	304298.059		2843124.749	
75402110.7	61314248.63	8686890.093	84589310.98	11632812.89
6130969.171	4928230.475	6813096.747	5104486.464	4905321.367
	305267.7097	308823.7374	531544.9262	715377.3453
4319227.559	6453425.244	3957406.317	8410736.481	2631418.415
	49349.40836		717538.7973	
70982893.06	66841560.73	69945066.2	78849475.84	54070304.93
			668201.893	
829544.8921		473472.4997		
9416211.189	5799377.98	15838086.69	12016298.25	11815264.87
2613838.814	1933703.685		2760369.456	736292.5577
359555.3631				272399.9404
1266733.28	310692032.2	1644100.583	381740800	119130504.7
1883216.002	2038209.167	1332724.349	3604283.587	989650.0303
202777.9382	85888.1451	238763.49	259565.4199	
156000623.1	179008887.2	97007538.89	102611348.2	123228599.7
368463835.9	241351071.8	223118541.4	528230991.1	415264030.4
16080820.26	13485207.05	428318.1215	14366310.99	9156977.832
14073459	18746931.96	6679851.156	25738341.66	8371404.157
81736081.24	81348392.32	88242766.74	148351583.9	90219378
5663378.824	4975142.753	291466.9417	3028536.382	4504190.054
1050896.208	527386.9297	257799.2153	589266.071	
			714920.4687	
21978218.75	31166730.28	26631251.94	30006270.67	23720465.47
5185239.125	5447421.718	15099022.08	6098875.183	2805448.095
8378195.286	9563587.92	10795742.69	7895438.032	6674373.929
4925811.368	4915952.696	3128001.091	6426413.365	4988691.49
180943240.5	3236662.744	3044201.755	1718725.181	3656094.261
275941.7105		364888.2761	551017.4985	259716.4714
	128170.0827	551284.019	234959.2339	92698.17376
529112.6126				
13197117.27	27853333.57	13110805.2	14898271.87	8699077.871
451723781	389407148.9	462107676.7	678906569.5	531119768.8
45823.7741	541931.7533		64592.23385	
48816.40008				
59845589.27	75359335.57	157762421	94653151.25	29102709.62
55870671.77	63359855.3	79607406.54	68645434.28	70046079.72
1387340.775	448819.9306	1285171.767	310242.8544	169257.3702
5260047.177	4970196.931	4376011.31	5143242.128	6465860.606

333529.5032	202979.2801	107561.0428	251093.5537	313133.7249
5268996.999	1105060.788	6210090.855	6514654.684	4816431.326
66919135.51	74017582.42	90907829.25	74480765.8	81598079.29
88406608.06	86688423.91	96188841.06	95378114.76	103385970.2
10387693.89	16480657.58	9838673.04	28293835.71	12180332.81
7218963.73	6190423.176	12209074.78	9370368.353	9293990.138
23684039.99	17055365.13	25589216.64	18110610.51	26995389.78
1409107.893	1158935.123	1117280.504	1343070.012	1022427.278
		1147392.517	1200656.963	
210423.1618	79100.32932	75523.71117		
8329727.642	7955238.292	8259148.545	13578702.58	12819855.54
23791616.12	39575952.83	56151542.78	38676705.1	45913268.31
675284.4471	918885.2332	205777.0384	196185.1326	1077365.26
313155.2925			1588851.072	
283121.3742	94151.53737	90323.36157	35835.28719	
81011235.05	58942411.15	56129518.42	95127342.25	53795005.44
1301590.322	5283260.974	1084681.621	1097104.066	1120393.397
		406447.9057	100995.9431	
3449441.863	4004438.464	11068.44056	7906200.057	1561902.928
14679368.82	23072147.95	36110138.21	5251511.903	6035204.227
33047280.31	28461878.57	29682242.1	23268097.32	31200122.37
28425.70788	7210.981663			
905501.9665	956238.7524	738967.3676	997748.7176	1073623.566
4454269.373	3027620.437	5052363.39	3766166.286	
1347033.059	1872828.612	4271991.599	2344121.136	1298053.045
6818961.868	11541698.33	11186176.56	18108607.97	11527545.64
4343876.375	2188207.91	4354335.761	4084457.237	1716362.06
53634985.23	103907462.9	68385043.06	63207420.48	48371051.11
1894074.688	2479194.716	3690974.752	2595821.484	3324511.167
257939634.3	457965218.6	398212152.9	479173597.6	299927814.7
48036823.97	64900409.56	91038656.38	71651543.63	42048586.8
		678104.0456	1106071.942	1686716.866
319359.7582	158447.8855			
			439487.0749	
644443.2954	820267.1494	607200.7201	4969003.931	275622.2639
613077.9148		1879915.848		
2623515.008	1484994.62	3123743.763		
542884.5235	471544.3152		505798.9651	757582.2152
5460920.808	4808130.147	10190342.06	8212859.159	2837454.493
1190206.807	1567027.526	1786239.57	1023700.991	1245176.651
39905783.56	55762169.4	70939230.14	56236683.41	64723872.01
87570540.71	65836601.19	3568705.43	111771274.9	19083211.16
798399.9795	981976.12	1213603.203	1314799.708	1569234.514
423729.9459	240237.7357	204580.1895	153788.3811	432345.8426
36276240.42	39600455.13	1647560.668	1507790.393	10281241.5
224134.2019	200825.3616	155459.2663	239564.2513	212965.1942
6109634.525	9070399.122	26744088.52	8038574.29	5643268.308
403106.1435	553208.9224	444254.4546	614204.6753	547213.7934

879599.0738	551158.6994	687035.4603	1438822.465	388399.2303
386604.597	312771.7105	251152.1681	213183.3258	236004.1922
2200288.151	7445316.958	8898943.073	11644799.93	9233888.03
10182983.75		22742952.31	10700178.89	
3605503.371	6033781.285	2897436.628	4345438.606	3522156.174
1015992.925	983496.6722	243169.6299	1379020.918	486143.5906
552939.6142			196482.6678	435416.0396
2187417.298	2709002.905	1025227.821	4107868.143	2343845.614
63302407.96	65744790.65	27997305.46	46478605.84	30941607.08
96631.94399			151645.007	
441663.5071	114875.2462	49501.80522	4389.209615	
	979521.9163			
3327468.096	1788689.39	1633398.812	2715344.467	4204394.153
204829.3965	260428.2639	321450.9288		304433.1954
	244012.494	515981.3915	853312.5701	1578183.96
1533521.312	6909573.323	8421066.687	7174812.239	4937794.751
	1506447.84	2463808.25		
366114.1782	954741.9824	182434.3133	609676.0895	161093.1755
115070.5406		2323612.811	672449.2932	
15789266.82	8451552.345	16112223.05	12653752.79	11037888.71
12911941.01	6953108.444	2049374.477	5108336.69	11850972.81
161141.4412	90273.1859	199264.1472	188038.5861	207760.5886
268026.4835	401343.2121	2998741.592		1249075.759
400436.8427	243121.6951	31729.4847	147250.1401	259423.4334
21721127.04	23853924.27	38048416.1	46038545.89	9912662.554
2205752559	3254636007	2219791928	2821208602	1731885978
7990103.773	12137521.33	9420141.956	5373359.662	13335374.84
794758.2187	934837.6041		2131257.685	
127018.5363	105115.8108	193550.1026	154994.6142	72359.49302
7206754.28	7690702.692	3010148.408	11138380.04	2235369.698
614869.2309		38940.78048		
	1010575.769			
		127525.7516		
			1876230.391	
1102920.355			351008.029	
526256.6397	1592692.746		1162642.808	
7254192.258	4710183.952	7999199.545	4303825.314	6310126.401
3305840.91	3312844.912	2068683	3755809.634	1815372.289
806326.1782			1014546.75	
11840372.17	11521469.46	21191787.9	16268305.62	7878468.234
244525.7414	233393.4721	223545.5585	440755.783	
240472.609	188058.3993	80174.34739	329288.9265	
1148223.046	1053498.113	647613.2131	1851029.552	2217557.8
143778.7889	184604.6541		175785.0556	37438.56037
812157.9125	748243.1044	722926.6165	643086.1102	997295.4057
1501358.683	1494483.066	3776429.938	3854676.682	2383594.144
	1275511.03	1150060.779	1099156.972	

6204812.489	2707609.872	2675176.889	7412132.169	7356686.463
36663970.29	36316950.82	114987922.3	53898987.3	32993678.14
5484595.029	5641344.734	3577688.632	6538627.223	3454427.982
217901229.9	363934947.4	272827898.7	247647902.5	245165511.5
6245725.381	4681329.012	1136082.649	5364065.857	4928781.47
30572723.15	22953844.73	52838054.03	73517407.19	28543839.47
5194953.48	13838118.79	6249784.053	5623739.299	287758.6129
39478799.45	38617507.05	42857431.13	23150992.9	68131587.14
1674047.596	4072267.232	2961757.802	4673820.28	2291228.419
				2389490.552
25588463.86	20460892.45	28044854.95	22316048.12	22170135.39
827119.7834	1117684.312	2878682.47	2964228.831	448125.4252
8307455.213	6690148.224	1232335.438	13008651.31	11923313.64
15215836.18	73775631.79	113332426.4	152185790.5	54303209.25
837382.7811	975136.0095	510251.3918		
769729.5769			675240.1427	
	273349.2238			
25816383.86	19753034.71	47455149.76	17883775.97	8118467.887
541305.8246	405759.1399	807475.6556	542512.8691	231541.8437
168854.8119	37904.12967	13285.35825	3270.504206	
3964179.887	3711175.606	2386785.103	3334818.691	2627114.518
3312536.919	2608082.921	6611675.974	5709948.262	3013406.951
1496775.042	4674735.98	4406514.423	7800759.772	2139313.982
48034474.2	55069437.11	165855094.5	65198809.6	48093433.69
5295490.295	2776594.68		4652139.016	2798166.729
65902157.89	45719909.22	106693303.5	72337753.36	82469926.69
9887641.476	10158089	11713039.35	18257385.76	7494576.399
20726.70518		5562.058447		
476390.6044		1515937.291	1480219.15	982934.8805
3112511.785	3439404.556	3193034.212	5549905.484	3297493.511
			1125651.765	1383617.421
4195473.284	5643110.265	9593080.735	4089939.53	6889623.907
14245.18981	7382.736815	97082.28668		4502.801248
6170023.968	6084424.034	9463546.804	11028488.45	4527579.576
8344827.453	3642900.694	12897625.79	10465254.03	4994221.14
27409244.95	43187516.97	7762921.93	51761741.85	10289481.86
1100084.411	956911.2839	1130314.893		
213538.6067	306565.8602	206720.9618	264819.8238	238554.8239
1020867099	1074023297	2449066907	2224653115	3045379995
15104582.6	18557577.69	2477693.248	12248768.7	9995608.117
1470408.485	1945448.237	1812246.674	3311854.9	1524995.967
	3563800.709		2820458.05	3942175.85
1061615.307				836129.0863
503823.2158	126012.5884		827137.1857	

319279.5863		294560.9548	896405.3477	279446.3139
	308215.7898			
803242.6336	1534982.428	4531438.828	2487617.878	1342581.67
20392827.44	21819799.46	10217311.98	33006741.06	20607102.56
8382021.926	3510692.526	18788273.18	2255708.708	1842968.042
121208.8985	427709.4717	468133.7277		
2361642.325	1973172.954	1409136.72	2450513.77	2384031.711
		174521.8878		
1434586.24	1223667.007	510197.1707	2140431.263	500287.4897
3106764.129	2135014.226	6435117.773	3359617.505	3549892.619
247599.9418		878755.6493		
364178.7173	366257.4103	4374174.657	1813283.96	224898.7674
	563960.5249	1411789.412		
863351.6571	1552273.847	1156766.105	1450167.12	
13512979.37	9596526.284	8937214.207	15252749.71	6978782.659
7606357.092	7169305.301	3428165.725	10171374.93	7815794.063
	390461.0589	750945.9656	407450.6367	
79073434.92	92914955.55	86067423.78	61751358.05	48362419.2
771870.2928	1157032.049	3607877.822	1216995.089	1198410.066
6482756.611	4860933.743	9032838.639	6803010.862	1912092.302
9111032.033	6062105.344	7244574.042	13375605.84	3129532.102
97659216.49	78947684.11	90845679.19	65513679.2	46847666.64
1215367.799		1803722.199	569592.0048	2451092.354
716709.3033	366875.6358	403806.2354		373424.8068
761779.3286	836696.1233		548597.9656	
303977.6886	257463.2923	530341.4266	374435.702	260644.3778
		3609509.049		
618431.4517	1235965.237		1965524.471	
4671085.721	7010667.158	698632.1327	8460911.674	2350943.464
4781718.297	2441853.386	931672.8494	4979040.98	
2834648.362	2964452.067	2041063.866	2493801.2	
1683997.412	2253811.692	2314893.216		
938192.5672			29539794.53	81583.26944
4027055.732	4059242.037	7556224.962	6995085.012	1169700.139
		204089.2786	417180.6433	
1090745.104	974851.517	1862033.856		1417338.631
18925701.48	14422982.92	35211095.93	12850726.22	21769109.58
852011.9396			856620.629	
55647.24335	14653.49407	14254.73156		
46425.16381	41888.60943	11094.68716	116604.5936	
605028.8637	598043.3771	1294156.507	1010268.321	
	139966.4351		297407.5034	
1577826.928	785721.1681	3037244.397	1895814.455	2787302.298
1059317.876	497692.368	374360.7604	746824.2157	1016134.368
	33629.8032		363897.9854	
6267592.987	9413901.754	10644892.31	5560635.347	1632139.349
1046417.344	919771.3855	454741.5637	1373070.697	1077458.401

214925.4827	1060763.1	2258837.832	2669418.36	330430.1445
383826.326	521885.2895	321624.9926	968956.4822	329989.4968
		664562.5471	729651.2693	832971.7977
981866.5063		1963502.227	1541407.336	
20318053.16	28259867.04	31068315.93	17237105	30193609.47
1006801.4				280318.1703
376639894.9	422917533.4	532328242.7	320511472.2	371031401.4
448181.1663		2084294.225	417580.8044	
	46270.6472		232853.1039	
2502982.592	1581268.984		1358828.101	707513.2192
2802473.718		2080030.949	3901193.563	387686.9688
756439.0037	297367.848	626084.4669	2870464.191	1309221.818
56757.09843			21648.91042	8480.196104
2040834.054	1209348.096		1459743.236	769474.9349
21223038.91	15711724.15	17727014.08	7752520.276	11990596.73
2445049.2	1732237.285	2330197.094	2218438.568	2422733.026
260533.5232			229462.6871	
	1239552.457	1014838.106	490162.3574	329776.6495
715236.894	1836329.586	169658.8176	4447623.188	
438535003.4	191338530	16803575.88	22525895.29	276933450.1
	334156.3956	343512.9497	404674.2416	
1735341.204	731266.083	68204.34102	832056.5776	88994.71395
3750875.784	2273384.433	2349504.836	2383066.216	1511238.822
14421059.11	13983774.54	10507498.24	16499955.77	14915259.35
13722632.34	12054659.23	22558539.19	16461484.4	10319966.76
701782.4921	1539812.794	292550.4208	1037791.495	472336.478
29921359.51	45261073.36	69664566.59	50603601.05	10189991.58
190690406.8	209731402.3	121267413.2	201186669	161684870.1
			1741267.444	
1255053	3692142.342	4380199.073	1273318.882	1208862.999
1212062.35	958354.3066	1773445.358	694701.0552	653735.9061
168996.4675	159685.2489		350999.7097	
360317.6758	487608.5667	592391.0623	1038273.05	309528.1986
881375.11	1254855.678	1123523.845	1191580.84	2295572.488
2949347.923	3668929.752	7740800.291	2786453.087	5653507.034
2786443009	1183851653	3094687499	1639546048	2203093029
	1615301.428	4906961.472		
21474318.42	18047629.73	36830663.85	49571533.27	17285115.06
1088016.464		783701.9627		520002.6547
	202306.6741			
1108861.692			1295783.798	
1065090.549	1512759.984	693746.5711	2288252.888	863873.3487
9709776.431	11507375.63	11049053.25	9128499.064	9761529.492
556041.3875	1208259.162	4347551.878	2917509.982	987844.656

2617039.6	3331363.423	3487648.431	4182729.182	971292.7478
		945902.362		
2118658.27	3712962.27	758953.5122	2204591.379	2733668.418
3810333.265	4872597.189	4507536.48	6242094.145	4718218.203
268431.7852			382752.5531	
889879.1131	861790.608	983360.9862	1129733.165	379888.9156
	166620.3951			
	301701.907	321963.2753	861052.6597	
55839.90385	21704.76707		58944.62287	44797.44989
31291958.33	37178651.09	83555255.86	42587002.62	17982189.89
729115.9178			366331.2659	
23765987.86	16816543.92	20050991.36	22862176.67	27221529.53
47964.8442	25696.47507	10869.7997	37881.97362	
2721915.463	2051438.7	2780752.498	773500.1709	1210509.468
279589.5794		303306.5017	328684.6375	135725.759
2645748.114	1199734.759	1826578.052	2357022.375	1092787.8
1299601.879	315958.8231	63521.5309	3868255.26	571755.4698
60819.42173		35638.40081	30606.55981	146775.3203
7371836.226	5005738.245	2580307.132	13023756.51	2192046.585
456856.0445	343299.2637		323569.4934	
5961342.759	7165942.755	9714596.564	7526841.967	6011907.717
		288039.8831	555176.8877	
332627.2583	522883.9556	909860.364	456930.6714	
51185917.59	26781095.42	12602380.43	23048432.32	6801386.857
2668791.159	2448371.31	1788780.679	2775815.443	
218603.4328	301501.2203	320972.8469		
7179919.425	4771430.806	10175771.63	7946773.502	12379368.34
695535.4034	396979.2438		1069082.505	
421963.7577	661436.7752	252777.0162	591093.1635	191041.9373
249172113.5	68836860.9	156884390.8	59154632.7	109096146.1
3010502.408	2243494.427	1043259.578	4722276.143	1696352.276
1103744.956	2005419.297	359857.2926	1142888.279	568144.3157
2651099.346	2704453.186	2452297.584	3228811.506	1788266.034
780945.3698	412353.9046	164109.2658	717485.5731	552055.3066
15214374.9	13176686.7	20433658.39	17833474.62	5532901.03
	307991.0788	373731.7758	598516.6101	
10698.18201	5962.50177	6363.543236	29278.57471	18178.04537
1434663.712	856509.293	497818.6065	971083.1053	990871.2789
178238759.3	141201771.1	249416468.4	258808764.5	240334105
1478899.372	918472.695	980866.8084	786351.3896	580643.0111
448971.0902	504111.5132	174695.4645	486221.1007	406254.3091
3847093.523	2060288.825	1997534.564	900613.6167	4504404.621
30463.93987	31701.31502	28027.6031	51395.37907	
	406166.8082		814383.1486	936931.5042
23554255.35	31027832.72	44656923.29	26929056.97	11791224.52
53820386.41	42194784.55	61492101.32	46312440.83	40260807.65

6819199.54	4449926.503	784996.3889	14057677.02	318779.0107
	1513821.849			
115188288.1	80401221.42	9912007.575	190059688.9	63516040.99
2497058.45	2408593.729	4472835.129	4212067.599	2623365.631
375251.208	180431.8001		322236.7363	
437831.7354		304067.4805	628829.413	
			114751.0256	2328.843656
2663229.486	2280068.692	2248525.985	2620828.634	968725.0546
6623121.718	5647612.308	6151604.179	5111971.9	6843163.956
2932140.964	1099942.594	1379950.642	1922873.513	729489.5723
592269.9104	594588.3469	1059238.864	2149456.763	
8823690.273	6986427.071	15006334.01	11620377.62	37782907.35
44607849.33	39139185.47	100891226.4	76248958.92	32007260.11
14988206.18	15426446.5	10200239.57	14210038.4	21090745.77
310991.1685	501951.5233	859249.0367	1079748.288	
	66497.26773		85608.36813	16835.5204
569409780.1	1270741595	481162321	727141541.2	603953542.6
979833413.2	1547207403	1006448484	1415408796	1529578193
184898.5151	148815.2103	384755.7527	212966.9439	107063.0779
72483711.79	61231250.11	42818008.01	41461350.92	45119754.9
	2754375.095	3106851.429	2582248.574	1094089.081
1431335.708	137690.3092		390404.3426	1250148.461
81282879.27	75359721.64	67288394.86	60275176.68	133903304.4
5332249.25	3476874.127	3751526.322	170758.9182	2033156.421
			784860.4536	
5428773.086	8033320.336	5637763.885	5280745.222	5419352.394
17221.42406	7464.161631	5559.984778	34795.0946	11461.43189
7500344.625	10253886	24971550.53	18260793.97	8657891.249
800867797.6	754592395.4	235035041.7	817964464.2	609351388.6
3921371.506	3192434.186	733407.636	3758558.084	3231452.341
15279777.59	14691656.13	19059098	16383741.8	20536467.93
4638204.945	5256925.29		4768808.996	1914617.049
274616408.4	275653597	375312362.2	202100379	220049422.5
	558889.0501	1988492.804	622140.959	8057591.54
56603180.6	34618706.88	9231196.659	45515140.64	49011047.99
607983.0648	515528.682	305829.9405	1194849.454	160804.3461
11070830.36	13965873.71	4958130.676	5334825.439	20499651.34
921396667.3	419575271.1	749553057.1	826562128.5	1235475077
8924041.236	8962943.362	11075841.99	10287054.79	7594648.366
579290.3742	1424292.02	892219.4027	524785.9348	1322169.108
410422.5992		617711.5385		417102.5137
5973643.352	5865060.933	6586643.033	4061217.406	3358121.087
10202933.51	10690094.48	3660256.692	11046852.67	4856581.645
		518684.9737		
				2143451.951
45174501.69	6092802.11	33057059.79	4023741.917	5130442.486
3038738.155	3750009.609	5527899.424	3308555.874	1748824.935
78491762.97	77388720.45	92458771.41	75865842.71	65462272.6

333126098	366329696	452561591.2	260113561.3	292442052.8
8082371.184	9818745.288	16441629.46	17465796.45	11547141.05
3379139933	4721474875	3149580744	4368009795	3855645351
4248908.663	2206628.629	1982044.545	5604066.26	1632077.038
54093846.68	31924656.93	17162748.94	115019336.5	22750114.68
772355.4313	954235.3116		900069.0805	
22914147.64	31017515.92	40622335.4	26071862.53	9928328.159
902622.9971	504387.6995	1289259.253	545776.4728	449862.0832
845122.3461	463802.4179	74337.91253	78267.29937	67374.71257
1498335.32	2656561.015	3557491.743	2873248.392	900540.4269
2708941.135	3702588.091	5663345.447	2686069.873	6855745.105
32022178.55	33925119.74	50825631.71	190850235.8	34462773.9
461371.7114	658695.9925	1690232.805	1330517.839	796233.0352
4567581.762	5807589.47	3101349.392	7153526.672	2045541.103
573195.9223	778075.2564	231154.8541	277352.5485	332557.6765
16720715.88	19331875.69	29129335.25	30182419.45	9372017.444
11469903.58	15527712.45	11866045.15	18825188.77	18431845.51
	576082.0715	2005867.362	1859754.274	427281.1025
14609225.78	7716741.239	1442739.043	1682111.173	1621190.267
337016.7411		518964.6126	575317.6694	147307.7142
4838674.576	2158933.457	5922774.448	1992504.16	2565182.838
5763511.58	12434810.39	9908405.057	15358581.18	1204257.837
17783301.4	18361370.91	42355976.38	22349957.77	12713331.84
93569.90241		98043.29174		340062.0187
37677118.47	43734875.96	55999408.41	33065676.27	29622159.24
335901.2279	231156.4928	83093.94134	862731.2937	
353409.0685	1567969.968	266677.2842	1769156.089	827933.4608
	511060.0497	354797.4033	691657.6075	6150509.306
4522120.412	4741364.855	6882261.627	6195451.149	13790725.45
385314.5323				
19132019.85	20391449.53	15137388.31	21159658.24	4505898.119
650066.7302	49596.584	990694.86	113334.7062	970306.0343
56908923.39	48407770.97	9444028.369	88401162.64	51016743.8
5518193.14	10578364.2	6733992.263	5214042.196	7861179.048
16763004.45	14257457.74	28637505.29	32713988.87	113476819.9
775034.3635	465044.4186		1003356.756	301696.8332
119579886	91815559.18	28348660.88	127872165.8	55353244.39
1531324.857	1338827.824	962609.8589	614664.9627	1183790.372
188048043.7	753352218.1	218882464.6	211360446.2	148967377.4
6423562.91		9669365.029	6827582.904	15019469.85
69217679.89	25815908.08	103549464.6	118147979.6	37904738.97
23484009.84	15893483.95	42126197.8	19378732.08	14490545.28
1572009.631	201840.8633		1097696.277	634468.2003
		555142.3703		
519223.8397			693525.112	
10032022.61	8248129.444	15807971.3	4887401.567	5114452.229
258572.0979	40900.84271	421059.6484	394760.4397	
2716505.516	6512112.634	4108138.821	5890311.137	4801698.999

20502358.29	36023821.17	6655422.333	63301123.62	21617877.77
20143642.59	9750126.772	11109142.47	12405591.49	12719937.42
30640678.27	19641902.63	8870001.419	13725928.85	15795465.72
7341742.107	10559846.54	27355546.96	17282194.4	1660215.046
36216171.85	52370925.86	35207648.17	38557629.24	25528983.27
99296385.1	64753174.52	38274693.11	82363431.15	105426692.3
5497668.102	1707781.418	7113340.836	2922128.629	946931.0769
3137029.19	3774259.928	10278426.58	4023519.995	2025454.315
2486936.648	1711538.074	4131157.107	3157976.491	683229.9469
50153851.72	63543664.61	98527386.78	117351023.5	69078361.89
7354014.668	2912571.749	1337093.829	3036794.404	2335360.267
		1670559.792	778074.3684	978670.199
3592148.475	668374.3488	6942657.833		
1018274.846	47766.53727	581936.0357	318295.5439	117637.1711
3144039.865	1430876.69	8629307.658		7951417.988
1372719.333	3685414.401	4346340.191	5451370.726	2308118.119
	941419.4348	1106872.084	573763.7926	
95875553.78	218437837.2	120090929.3	110607280.4	99066259.43
1341492627	1509522270	1884714904	1550513653	1190313970
5393423.007	5107609.974	17908867.92	18086746.61	3187816.964
315402.384	182178.665	135459.3895		84357.45241
13965421.94	15290126.64	16301716.35	22321466.67	56901494.61
15947715566	16173602112	4572114937	14226003426	11760159768
8556123.277	1348229.843	6202427.335	4543263.25	959257.1785
4657322.619	1827741.586	4192837.626	2964271.439	238519.0629
1693464.244	2501294.655	2599122.633	2103344.243	1829098.003
206971633.3	239853169.8	310935729.2	301175979.1	242455397.6
12572470.65	9991708.715	7708433.649	15494553.23	711295788
			610359.6205	
439637978.6	681668320.7	501639240.8	656252354.4	446674979.9
505424169	562455636.3	506049752.7	321344845.7	451005595
1003103.69	915371.4739		2810367.166	369165.1043
112178364	98911034.74	19568439.61	181118066.8	74312134.85
	925078.224	2558571.092	1121626.256	327494.3312
347140.9555	213043.0054		478426.7269	855063.0869
103027.7729				
438741.9458	1078515.459	623311.4216	726924.4044	468975.1973
14469143.1	15877418.1	32306839.55	18356482.79	7139869.129
39601033.17	71037096	65073545.21	21131130.26	42271777.37
4100222.706	4596602.098	1973590.058	5271108.189	1935462.368
7339354.835	3826419.859	8324175.881	4258795.329	9418585.326
90482062.12	104536561.9	30252157.12	124288513	63740307.78
992585.2985	1343550.989	1082046.731	1559263.043	1013549.062
464624332.1	532071278.1	606106197.5	624038537.1	639762637.3
27492917.75	26237333.25	54889824.55	25269398.89	20320382.1
12366092.79	14473401.33	23222003.18	14105726.86	11655157.83

1603628.184	1329415.907	606787.6356	2250372.091	1214617.465
2204334.774	2807644.39	196573.5563	2827152.221	1572565.223
4103772.706	5675764.369	13760906.18	6596914.979	1492455.067
50794673.09	65483306.84	91501244.56	99108212.4	20557748.53
40099893.06	40243622.1	25508805.82	48697687.84	45356457.44
69171219.49	64508672.14	143505034.1	65765023.91	46069544.3
6758242.005	7588468.518	5359389.569	7425621.524	5735564.661
3419333.79	4473412.516	4090314.992	5696292.73	4406635.26
12172640.87	17661389.19	33559291.45	22671222.63	7521438.636
	96385.68593	530552.526		138203.9685
48605470.57	46288165.33	71080835.99	48891318.22	44367677.16
1862440.051	1738932.672	2812056.305	2713780.119	2845986.386
801020.281		397008.1231	448852.5422	1212242.541
1006765.285	803043.9719	1904186.216	1236336.223	732328.9837
799346230.7	655403854.1	1171094863	689140295.4	885000381.7
	239117.7748			
9500481.301	7902172.478	13818753.67	8354090.315	2621677.019
1635758.098	3472215.721	4533140.817	3439302.192	8666722.222
	400525.8643	154759.56	291896.4059	447245.5084
574815.8368	332420.1647	1191611.355		
1122728.691	825449.9181	2384459.65	4199677.539	5638918.854
4058971.899	4549194.742	1487734.927	5032788.876	2053324.073
386374.7084	209643.0448	12968.10148	458582.1955	87420.25394
13001405.79	9482544.532	7970441.698	11399066.51	6068063.745
72259632.41	46633568.51	161420360.6	70656839.6	98657984.58
3467748.407	2475484.786	5051902.542	1837092.77	5164488.108
53707475.57	27364547.03	57093004.33	52640728.46	28228079.8
	1816025.547	3363137.334	2612056.631	7325581.293
540778.379	1025463.091	634842.6297	1136566.805	678414.6423
478805.9861	546967.5199	1048363.638	921278.4897	4377358.875
	674622.5792		3131831.557	964168.1703
1674287764	1587401949	1900178677	1787520152	1896817024
450392533.1	411528677.6	159445457.4	435075639.4	416700909.1
672004.7332	1071321.465	3474954.487	1696045.699	912315.7594
9516356.546	7616964.792	6913433.44	7003301.999	6821974.057
22361657.97	23521213.62	21770273.28	55331912.22	12546611.05
2104931630	1504311981	313643415.4	3615738995	1380107952
44838878.24	34582492.47	49355496.91	58489263.42	61765449.97
1590432.766		1435234.424	2095911.999	795104.3153
787822.0385			1242810.913	967298.1475
20430871494	21080932076	27001168052	19799531915	21897378605
1228918.131	672374.3513	303809.9269	1013696.961	
401141.5779	231229.0685	158519.9585	795011.1305	2241153.454
2734254.351	2685433.796	1064542.382	2733815.729	1263698.613
127522.6599	65242.10957	116674.1537	601577.9169	184275.5584
4960781.751	2845613.45	4257312.671	3704264.548	2117187.256
233311.4803	222416.842	459203.4853	913015.3447	
25186894.51	17982718.51	26944152.47	32621640.98	18234703.76
1456601639	1462941973	671431456.1	1361064223	1079492696

536371.0121			838772.6956	
612957.207	149251.6705		319298.2595	241106.1206
4801323.742	6121657.702	1109539.483	4367136.67	3016131.085
9176386.844	8892311.663	4150439.469	17423314.61	3613404.703
	6488582.448	5279343.802	4630838.512	5062144.136
3993553054	4719305827	1726196665	4635823965	2882458808
4015075.079	4614816.627	3161177.379	2329245.856	2448107.692
			374893.231	
1336023.948	1148612.68	1282286.509	1437357.595	731718.1253
586841274.1	464123573.2	966401142.9	534175846.8	390262162.1
1401390.578	2072810.572	568532.4765	3589035.48	612537.9899
38388.3195			122470.5097	
1248163.138	406221.2633	314854.6899	365763.9801	229378.1937
214850148.4	289712259.7	103731918.9	185865102.8	256338733.2
42956484.37	37536025.46	90316960.53	180616688.7	219315350.7
4449466.566	2114114.981	3654005.496	2352347.502	1413740.143
1061615.307	5399469.254	1265982.074	3796392.555	1666394.525
51810440	88439924.89	69353358.82	153692597	86466411.87
16102376.81	10431200.53	7411788.292	37814700.25	7872542.046
17469668.77	17848115.62	23670384.37	18468977.87	4729333.262
13848108.24	20028327.35	3563490.116	18134477.82	16002139.59
345978.8365	265835.8927	1061944.476	435377.7621	225156.5877
	3521517.542		4181132.07	
1332336.807		8814245.121		1024707.293
2858811.331	1841116.121	3107337.63	4261925.311	3569645.313
2794351.025	2453454.252	3214081.283	4202957.974	1494985.004
6037484.955	5574363.656	1226365.666	9562138.897	3129104.234
1247432.955	905199.8069	1245415.865	1527552.856	1743480.723
273108.1406	300097.2697	607978.4151	445774.0187	766775.9718
			307366.146	
2028358.775	2460514.386	4178145.377	2240545.883	2214425.228
9696224952	12620797555	10130814119	12843828790	15482691942
1298903.731	1045212.233	315706.1007	3028904.197	888994.6382
81218218.53	89372907.13	109555431.6	92281971.51	93889402.47
266072845.7	333387298.7	219710822.3	378899229.5	227023855.1
10683570.11	6757850.501	2232173.279	15426107.4	725998.236
91457402925	86118065713	94354818550	84974050772	91853445618
1292152.288	1534882.318	1792102.081	2337096.955	1385929.71
536154.4504	354347.7619	2523459.6	641004.4884	
5927781.623	519881.6625	6670280.952	938594.952	536120.2079
3029675.878		2870578.95	2075868.608	1112044.153
1256951572	1192229576	1684038015	1544343684	1142527649
42754970.45	25629053.19	9654696.644	103024586.4	21120958.98
19389734.33	24540755.98	12182012.94	32008965.36	12787270.77
1154041.779	1018576.31	2150237.775	6029438.917	2807089.893
268932970.7	277053827.3	285191808.6	130366513.6	259843850.9
	405139.6854	469220.0232		985268.8537
190271.9274	862925.0952	1997128.158	207697.2183	801964.3258
		1289480.512	451134.3089	559602.5296

		1433056.586		
518443.8727	319003.1308		916892.1991	
376015.1674		624305.179	611786.4273	231797.1177
1436479.729	3037640.937	2728144.735	3095991.404	2060502.625
16711410.92	22772822.61	26705648.2	27193015.36	8641994.863
45331725.64	55054493.69	58879398.01	80130096.44	26555207.94
84878656.04	92161058.79	76735490.98	47915170.47	105985274.6
18954334.93	10466503.46	2042802.556	18062874.03	15555179.86
1367319.401	492230.9825	2998889.065	153278.6879	1388450.046
			9027756.096	
254016915.4	288972939.8	277606400.5	221730883.5	223002736.1
3640480.373	2069375.879	4919231.96	5079672.296	1756163.462
	237496.4322	196897.5282	471917.2179	
622626384.2	278155189.5	552604869.6	282481721.1	534013828.8
185657.879		274608.8517	111680.4198	133206.3716
1857462.883	1253395	3393563.189	3822348.619	1678629.4
13032449.7	10079109.42	11872084.81	10789459.16	6453378.94
7879028.323	2874182.69	2235015.32	2636889.124	8931337.837
682124.6298	904046.9662	909525.7858	635446.5588	771056.3362
			1078582.559	
8702190.277	4904460.195	10602903.36	4842519.853	5445106.785
141563.2242		670239.1528	2596526.68	
4860670.156	4227157.081	3429746.863	5902487.73	5586051.133
2027604025	2114030049	1270093486	1719819838	1387266912
91947804.05	90540229.99	34869427.41	114962329.5	102592106.7
672906.6718	606822.6774	672120.674	906315.7768	743178.5983
29109405.15	21221053.9	15519055.02	26895075.51	5734067.521
282254.8771	596477.9237	184954.8906	632239.3486	673469.3044
2146122300	2338211671	566541813	1945307669	1735692159
121236629.8	105279654.2	200090191.8	119452456.3	168492280.5
5683247.054	8892181.543	2172579.415	5434038.107	3829316.714
942144.9054	1482039.743	1165868.011	1177422.689	1397911.208
3447074259	4584066926	3242872365	4063423075	3482290503
68868777.57	63054207.6	25988106	98812388.57	15948693.57
98552758.94	58089289.93	45948259.16	50208633.8	50428779.88
2125505.776	755683.3383	5010673.197	2800114.746	365643.6854
3513975.219	2706354.682	1972572.284	4398631.454	941885.1336
42061860.56	56897085.5	180149231.7	93692103.33	47182630.76
694606.2749	1331101.538	1750068.145	1389968.015	752855.3482
465651.9368	471136.5352	692140.3524		369665.3376
7141930.223	6104441.839	526513.8891	5979295.764	4863901.181
400925.897	389757.2399		381188.0444	278659.4732
1188728.175	347387.9395	127957.9273	1464712.712	7419903.384
170564671.9	157228821.1	226139063.2	205246652.5	511662858.6
147455.9348	46855.34569		107581.9879	
6641507.888	13724844.26	10102124.49	8347136.294	4260083.507
1100360.676	1401179.372	1079554.258	1495734.614	957598.7084

1242097.456	737310.4443		551722.8074	812247.8948
24691906.45	25795692.65	24750196.15	42252264.42	14462988.03
7595889.414	9243554.686	19200056.44	15145779.95	57074175.57
10026967.64	11829733.13	6476838.901	9742723.943	9543871.473
47046531.85	60942676.45	8672569.277	23138561.8	30097750.67
			311107.7075	
3132853.272	2352978.098	3463170.079	1282104.89	1669374.468
16754120.23	3158421.134	4123777.692		43029977.76
	147369.0682		215139.291	133776.1565
1036941.819	777356.0916			
82484246.36	86602991.77	120757594.1	137825909.6	106739810.9
9435096.37	12190752.1	27337968.82	11078473.68	5075715.242
225228516.6	95139651.98	142425285.8	85496411.05	108011819.6
75128823.29	64929070.57	70934564.67	71534324.78	73559237.05
11153577.71	20163883.57	22692497.38	19949335.73	19207905.77
397978256.8	413334309.6	1140087750	588043791.3	298042527
7891824.523	5058393.039	6367818.006		3449061.528
197981.5452	274615.733	440507.0982	268072.2816	105422.9018
3454551.783	2851728.392	1708259.447	3918700.799	2206984.234
245716529.8	157771249.6	133938510.1	179159311.9	188171544.6
337869.2017	199274.1737	433146.4399	275220.8503	491580.2133
341414.9937	128367.9456		309781.4228	159663.6573
5086398.44	4621761.215	2433716.272	8402344.672	6594483.796
3117693.183	9896319.772	11708049.85	11720025.84	9640648.022
52056604.91	67344569.07	79527445.61	83154557.03	84161992.18
17215146.94	5301473.502	20530296.03	16526544.58	3218256.427
88343035.27	4838750.538	11747028.73	7315870.305	2952106.353
121216249.3	107109451.8	85259118.9	122318357.7	130543526.1
2144973.185	1540875.301	2548174.476	1812552.536	1780107.678
5811454.373	7637877.707	16998338.4	24595961.89	8937325.221
17571656.67	20544797.67	9067252.252	6967385.944	17171976.08
52679241.55	36228495.68	13210259.25	62591535.2	21953265.57
477546.4047	735927.0913		547240.2186	2083188.797
73642417.79	114122084.5	91474643.28	125182396.3	83739547.11
16089866560	17546674470	20833559474	12837889105	14523500434
46074178.85	37931898.68	60728282.08	44990557.15	54298386.16
1490940452	1745656395	1192062578	906268196.5	1105745165
1567801.956	1869492.589	331136.5169	1033616.31	1085549.29
663785700.6	366841008.7	173828587.3	516968886.2	187886831.4
2621896.328	5975723.145	10102389.23	2297839.981	936854.6139
5016949.805	4149868.305	6843496.356	19822003.51	82605354.13
1245434	1506726.298	5018898.958	7119612.547	
281987719.5	359883950.8	462541584.5	303390845.6	220863941.8
1381076.415	1847031.203	1233950.953	4429313.406	2906934.272
8649764106	8030344832	9928408891	7787481243	9242155273
626049.6386	650957.0378	670387.8302	1368849.765	480335.8961

20768220.19	14021404.52	3361379.563	17086092	5023056.918
689568.7918	1286171.489	890013.7141	862412.7516	817820.4583
10425776331	9848722784	5738488832	11295063961	10395312470
		1251569.058	526311.0956	
152561399.8	182251766.2	330073051.6	184600423	106638929
290874520.9	331046609.6	942041182.9	493597663.6	230867187.7
254748.5861	268758.2165		1721885.26	835702.8727
35807042.31	40653313.48	51068770.49	76300821.42	18004299.34
1822949989	3185927369	2382262168	1274213494	1500942792
		332772.3671	277279.7306	
172466418.4	208976258.1	117697785.4	205345950.2	75818009.94
43753236.62	31557931.96	3965721.434	30756649.09	11177859.04
1943737341	1857114829	1047270273	1828027416	1811800784
761234294	727118367	57416620.42	796979632.5	409612655.7
9838120.312	6864156.446	6752688.772	8330209.971	2494999.418
		9960.919282		455137.7243
1234984.637	1239930.011	2150527.097	1127580.46	707483.0858
3291378.758	5109664.853	4746924.083	2642793.364	3981865.509
23799214.76	28009383.01	24604167.47	25407082.19	27365840.43
295815976.3	8528346.034	7889475.121	15662668.45	21026939.71
228443.6468	643851.2445	1156885.016	835280.9698	141515.5892
1171670307	1560027321	649043746.6	1278193409	1047201932
36541929.11	47390951.21	5018109.063	34517361.26	4191458.767
2964545.82	1688390.86	1896508.112	1270885.429	11202822.8
777516.3412	1016743.6	501446.1963	2002898.586	
615558.8968	957267.8493	2211439.522	2159767.032	983429.7456
2548134.608	771946.5353	3041966.998	1759308.046	1568080.802
11917060.31	6178239.874	11648337.89	5069683.171	5215723.401
	348669.7945		495809.3902	432507.6026
11149498.92	8928461.967	9890596.401	14883026.57	6948047.418
7106623.284	8593959.1	19324647.43	10022300.34	2873633.567
3924595.308	6316598.822	5475742.279	6107213.78	4733936.692
5403691.098	4133365.098	1090326.467	7897611.785	1878014.682
138334104.8	167987384.1	79721481.2	332256329.2	69666982.13
3012941.514	4070124.809	2192217.249	7621655.785	970912.7236
6033717.087	7077452.545	3890228.567	5569391.258	6274717.885
1412946450	1458824403	2107538855	1499553739	1463667619
730773.3788	1101994.542	2108260.415	1156146.303	549309.8784
	61009.46014		136783.3783	
1021531.123	977423.6447	1191084.292		679457.3409
79395400.75	70738564.66	68466697.96	51935720.11	25963728.42
192993915.2	181420135.3	399036212.9	327358132.8	158401235.2
200730310.7	238339395	288887015.2	265896918.6	326178353.3
12089077.1	17647208.37	6388964.034	17910799.23	4789009.124
35655276.36	26776061.42	7378649.375	20646745.73	13076105.25
34171888.26	46568318.57	48534702.23	63553339.61	17807909.07

193357429.6	196447494.2	85749219.03	251125893.6	64887933.76
3326182.435	3149407.243	4237106.724	3160889.721	3056624.208
72280416.67	46223613.91	44374954.44	51584857.12	69050543.25
783550.1616	543066.0846	1446414.135	1263596.938	684686.7927
3183475.167	3074351.303	3724509.824	3935289.917	7636142.199
3879098.651	3759025.763	4369300.666	6197481.378	5111174.391
103924852	155822496.6	76621728.01	113002000.9	129283572.6
83487473.4	75601748.8	41533849.71	102163137.6	33723382.06
5824969.914	7201288.531	14023357.43	17501670.98	6999818.381
193309.3449		332745.0446	495676.7954	
6609149.464	7022643.245	10480929.75	7142458.287	6565449.192
651146.485	137901.1644	1683678.301	1653001.327	1613914.212
	1442303.916	4085479.664		
406416044.2	367688341.9	177207121.9	509307364.4	329395969.6
825631.3856	745133.0558	1324821.221	944917.4214	567884.7601
			740443.6719	
661920227.1	548009860.8	882904190.2	674164695.3	597731688.4
808529382.9	609654910.4	1734819317	781353626.6	669714762.7
271187.9022	235142.3297	184165.3099	1361802.746	
979117.8761	1288583.976	150584.7778	1170848.842	818643.3683
24438054.98	32272671.94	76359228.62	38105912.65	12321481.18
		233806.8617		
753559.862	529638.6588	1436896.343	1174440.335	900929.5008
12467371.74	18865251.35	32039679.64	26924913.45	10926581.42
3127686689	3347737139	4679388219	2464322101	3431758546
3648149796	1799296215	2214991008	1612972722	4050247702
168060.35	114224.6817	75924.77866	267854.904	209671.7378
46142357.05	62320248.76	116831987.3	65669930.12	41657941.02
1425330.015	1234462.321	199249.3898	1954188.487	1123337.319
1545603390	1606986537	573570441.6	1548068656	1115226896
360917263	497152390.8	112633072.1	297626278.8	279069308.8
11326844.2	11387971.17	25373593.59	14788789.45	15299346.43
	297642.5259		677873.793	
3919895.516	2224390.169	5502657.018	3077973.824	3399009.67
34963700.78	34422989.15	37371931.08	40890622.65	45032426.05
706377.048	828493.6671	388000.0595	792144.5722	306176.746
		1704548.466		
3974223.451		560847.8488	747933.6689	1121142.158
1021487820	1416091429	1141617432	764101638.3	726189399.1
678261.0943	418128.0437	273417.5685	1164347.941	
33450345764	32262189114	44264819543	38920944261	35554037726
29056801.73	42296526.46	48196817.37	61861633.95	72794266.72
			641916.2058	581125.9234
344719.4082	195921.5355		143232.9498	
113282471.4	97432337.26	196541005.8	127112205.3	77958015.1
2231167.963	2762692.57	5004697.864	1905355.671	1105749.589

1926694.671	2355159.887	3472424.268	2567137.897	1480786.839
337668.3412				
473472.7459	3317179.175	827136.6103	2073671.351	2308160.156
235860457.2	98304007.09	84514862.97	382497105.7	196570025.6
29895519.21	39795079.26	52266897.21	37216398.94	17104871.19
4431853.986	3515775.696	2503149.321	10488367.91	2563288.102
693999318.2	710813504.8	1104209377	1233067129	1451474945
15940946.49	8162979.935	4650313.565	11457113.97	16739597.25
9534937.315	9878475.687	5460182.716	9152648.556	4252977.694
146484339.5	40069759.73	95347845.78	277743153.5	26719075.81
621078.5602	492654.7135	3036662.222	899758.5042	
				3207905.114
17250896.61	15671551.94	8484285.484	19061144.06	11278204.76
21794758.23	10671386.81	19422076.5	15301708.44	3752379.529
	415393.9289		1407714.19	1037217.99
59864701.73	66108261.09	51092633.66	156923647.9	38289941.8
2959189.602	2618256.431	3882693.672	436499.2605	553829.5096
14862228.89	17709087.83	22721400.42	22787207.99	12799758.76
98951207.84	153657253	156492064	173605094.5	44947582.78
8750936.548	8937428.024	19641515.56	11482723.88	3833369.246
39140502.86	24605533.24	32371733.97	13931362.33	20051835.09
3705662.437	2414963.075	4263179.481	1906545.539	709279.9618
788139.3561	1038424.11	648039.8979	1232106.585	
44107677.8	26447518.44	32795524.56	25694850.97	53993909.01
		2468098.32	806060.0179	
10043437785	6802353328	9167387706	7228634612	9650820703
292698241	248864793.6	334223654.4	301320538.6	274215744.9
2432846.299	2571471.25	872836.0571	3257037.741	963289.6644
286207.3123	395743.9848	591124.4052	535319.54	
6705209.539	11569092.48	8532495.226	10553199.35	3832557.267
28180784.84	14817252.01	33076612.5	15628888.39	12782840.29
2611754.23	4660828.645	3358468.566	4797478.754	5885127.617
445264.9928	2654992.843	308719.1773	1956295.388	899292.8927
3310680.06	3613625.161	853545.1124	1646099.109	
57731423.95	61745888.19	17630962.38	60802325.14	40938567.91
1393954560	882230855.6	1132609656	1446844037	1392409109
8655757.816	8712594.77	6834689.369	5285725.571	2083176.88
14077027.06	10587512.86	3970447.273	11091150.23	10199168.51
30438405.72	41978167.72	69254598.64	37324193.85	42228973.21
		449109.3241	1366619.07	731234.8238
474259229.9	726075409.8	450744714	521043956.8	754585526.5
18764921.94	17576904.24	18681242.85	11774594.54	19640503.16
14659722.32	12542987.39	21861373.23	12040080.63	11801653.61
5257807.438	3506350.682	6897940.731	7061286.856	8198918.917
6603870.354	4777098.307	4491429.798	2723794.362	4395974.862
3529390.357	6536064.902	7187325.036	3469249.46	6069213.65
5089301.844	4558377.167	10276421.75	4917439.673	4524558.422

17416897.59	24675702.38	15491603.03	18435481.38	14230184.54
291692.6627		1852359.6		
				587398.528
170799536.2	221010077.3	667375979.7	320833167.5	162714985.6
2141277.81	3317784.29	431203.6233	747594.6657	2806297.798
14504940.48	10223660.75	49820703.68	14949606.2	8270484.839
434578090.1	413962555	117002792.9	294247287.4	340362526.9
8985569.936	11943234.52	16814361.63	10648827.72	2795742.753
3209251.646	3307074.069	6584760.647	4194442.488	4557878.459
10653452.86	5764006.3	8219392.075	8337594.452	10108532.77
122041415	121015738.8	96498663.61	113251606.8	129856233.4
749350.7133	1395834.021	3176871.261	1929514.848	1262646.832
9219240.917	8946070.441	11465240.04	14282494.18	27811999.58
25456317.13	35090215.32	33224874.81	32052518.81	35077180.76
1249843.809	1633625.23	2096320.358	1792636.986	973397.2176
208175.8788		100882.045	743293.4953	
3619936.762	5441796.726	8152694.376	5473647.735	2823317.471
9747609.062	2993161.036	7880590.197	4637987.322	4699623.649
7255714.739	4338693.287	8271777.51	5909825.988	4058749.204
131478.5596	135607.3741	127944.5502	292459.1306	75371.63709
5800979944	6484876151	5826111880	9620191521	4499050490
13566212.53	14685032.02	19320962.2	11870395.31	12485155.02
3658623.998	4680086.378	8550866.705	6453853.314	9131295.094
	20485.66399	15433.91146	45325.34719	
1017929.726	2872406.316	1183095.36	2799105.35	1923092.163
3541426.748	4266392.998	2990633.931	334576.3667	
3839530.079	3213190.887	4045081.908	3229575.211	2441015.291
79795951.59	59769511.34	139767152.4	92237875.62	64593453.66
382059150.3	370485092.4	156621387.7	461347962.5	333421897.5
6271719.838	5153913.766	4063980.027	4131091.27	8830431.42
8119347.591	2918361.346	17035128.43	13054146.29	2282778.038
214063729.9	160600163.1	125308076	188931773.1	191672907.4
1592895.643	1689682.885	1294934.648	1099349.054	
539849.0591	351490.434	127650.3366	523935.5683	766470.1619
1559814.887	2708999.628	19044685.35	24817251.98	30977631.51
		469344.4966	479842.998	4698627.853
	410273.1019	1813014.666	478015.9979	
2656733.705	2304286.569	2967487.315	1338411.08	2383025.924
29022130.42	42774793.39	118248697.8	157886156.4	231282369.4
918818.1978	661167.4044	2622390.151		364663.3618
2740935001	2840324832	1394161444	4320741504	1183617729
3109850.833	484181.4717	555675.0971	462253.83	715645.1397
	995982.9971	1542594.659	1720214.046	1513320.878
1685440164	1185898898	3160131261	919502905.9	1762616127
4615766.887	5145179.777	11410989.73	7369767.707	2800890.692
253599696.5	399375969.7	164953675.4	200500543.5	164375542.7
193406.1754	133075.8177	91606.92777	297260.1331	
1833521.588	2519148.438	2383720.259	1545711.269	979052.4775
4809228.917		600235.9734	2841467.274	899065.1247

42653450.76	30165971.18	38551521.86	31935019.38	28029075.48
52821129.34	37881967.07	84251646.46	76442831.61	56388553.54
741875361.5	424424877	1419604689	490875458.5	751201777.8
792014.2606	2875408.236	3626920.469	6971696.43	13628687.11
221110.3027		2345401.993	1243267.671	437631.6687
819124.491	926316.1316	916431.5935	706232.2989	1749255.703
240392381.9	162221804.9	130803847.4	151126452.1	148944193.4
27718742.42	47272207	59156016.53	30367664.01	30129808.64
	886682.6925		922097.6349	678177.9867
16474034.95	26871713.98	28890327.97	26703569.27	21990477.75
		632605.5995	618064.1203	321039.4344
4721677.612	5780381.266	5443026.433	6115404.332	4186770.451
10109276.31	6283179.357	3512208.507	5699718.271	1789578.473
234280.3022	237272.9689		361187.5664	80636.32289
1055958629	1276065424	1978368478	894585601.7	438638037.4
2757226.174	5421976.041	4302551.082	3144089.119	2397539.955
15389368.93	15484175.32	14697176.06	14520228.82	13353583.99
40701881.54	57355351.87	91020456.32	39152757.89	24986597.63
11489853.93	10381022.7	25929945.79	32418953.35	22686304.17
			496177.3663	
3257143.389	2779938.147	576629.5035		
	739020.6023		1238504.539	1039205.3
477262.4809				803417.6199
843070.6771	4640296.767	5223498.634	5079835.775	4048483.234
334582.6712	238757.9317		1035810.09	
523077.4901	1584152.907	1020064.792	1157167.814	875490.0026
	42875.54741			
147677746.1	206939730.6	228351073.5	332176815.8	1035745623
7144731.95	2585866.054	2633025.28	11121526.83	4019340.868
9339423.962	8095861.708	7827531.269	11136624.8	3829243.408
9075883.734	6194228.439	4344389.823	4818993.022	4697739.552
	1975221.166	3889579.73	1833105.107	
			480938.4465	343420.1856
233387382.3	286249383.6	195457175.3	282071099.8	338097715.3
6769359.689	10843314.74	11730086.22	12803726.94	2854985.508
2498296.356	1893245.621	11308570.88	4180020.144	1491671.048
2307542.794	3046292.816	4304885.728	3303746.684	2121074.826
7042681.903	199274.1737	7084462.409	529403.1742	1325805.789
		562605.2138		
436383670.9	481652415.3	271439839.1	254799931.2	251625513.4
694613163.9	578712537.4	1161035573	1078536205	694638963.1
12956282.56	12619464.27	19548513.35	23292246.32	22848641.44
32253888.52	30481946.91	33903835.19	38446121.5	19179435.94
441626.2981	176390.8306	2835453.713	2514613.218	
37775300.75	45354256.17	65795017.34	54179955.34	45805914.12
4762314.871	6978879.688	8183501.995	9807393.175	4474892.123
	3216725.029		1623129.382	2037722.971
19424267.51	17241123.21	30068210.95	17623184.21	14098124.13

	203256.4908	1240264.955		
5963929.993	4361415.826	1080596.444	7815057.841	7244309.769
1146902649	1536245205	911600357.4	1499502320	1738697112
19685619.78	31208584.37	25668285.5	33082880.34	16673016.04
6650522.324	10338943.08	7226681.444	5584111.169	6596790.028
33281956.21	45218906.11	39325733.9	47427564.45	33040245.36
5014399.987	5688091.026	4096584.147	5222436.458	4034229.668
31574959.9	45704553.13	40389054.36	27028755.95	32780151.76
114995681.9	91127422.03	47274019.06	91810946.03	82868146.1
250657.4645	124177.7927		134562.5047	249761.8393
57880190.13	51902473.53	44901689.95	59551643.05	65802752.04
83783.62779	178005.5316	82987.71671	240561.5706	93841.52236
96985.0488	71479.68497	59208.68515	137164.283	92383.24122
1417727.625	599847.6955	1202262.237	639568.9447	1170842.677
3422879.185	2250623.564	9200189.227	8608871.427	29003544.93
	269461.1043		198872.6174	369469.0977
6884015.029	3003861.62	2042608.254	3143656.644	3328732.043
957164.6027	1664767.335		2474568.666	5676063.655
168045964.1	933303573.2	235524459	328851782.5	303803336.4
58097026.16	44309717.07	68435623.12	47311412.95	59968529.42
268255.3764	302152.8749	340151.7816	320702.2606	48945.40517
5977729.151	11729653.09	5169425.569	7952083.459	6995548.882
7476075.922	1468049.58	3904783.269	1992709.621	409857.2487
13959984.04	48667132.12	78611914.64	55223560.74	36869048.69
3876029634	3258452001	2003064528	2577565317	1947908825
10319587.42	9361734.672	10069536	10022796.9	12647208.89
966626.4177	247836.2693	801587.4457	893597.4741	460379.5668
47076492.68	59034533.1	143009139.4	96597342.01	35751550.22
9346412.001	7616348.899	7785242.147	9876681.586	8514689.033
37420356.63	45817078.52	38017029.31	38370171.68	38757192.34
1735010.286	1383205.319	4288498.658	4704943.395	1627470.462
38403619.4	12857477.96	36694104.63	14811282.64	21659330.02
30136827091	38287602256	21804551348	30704540122	26721147745
478707882.7	716186427.3	237105805.3	668525771.5	500107416.8
11948562.72	11208805.69	10673139.01	13004152.6	18435088.34
4495968.79	2581301.809	12443814.04	4676164.458	2041004.816
6982101.345	3801023.823	3481390.739	2775925.489	4144152.99
36682.46961	16324.7796	9462.981505		4486.382884
		136774.5511	217007.0363	1294288.256
1908045.812	2627396.618	1519185.292	1487913.275	4057708.584
65790.58638			303172.9336	
1660821.311	2434161.761	5079337.27	2084243.738	1755822.621
2886552.251	1244610.83	6676150.345	1317837.551	1657003.969
3516097.543	3015782.396	486813.5863	4121553.7	688023.6165
1793245.371	1813059.644	2215282.974	1104250.563	1558204.147

9480030.791	9252578.699		5988639.325	1554905.955
1850664.108	267457.3749	996310.3073		
23755507.44	28173249.63	16024486.18	21490826.26	10660984.45
1066650.717	518988.5916	300621.1124	1259175.531	208420.506
2341188.88	2544723.632	3608582.197	826463.4194	672787.6182
295172.8351	354627.6729	233217.5413		
739523256.2	145252186.3	389268379.8	463003648.9	167636010
14390618442	16285290558	20408057596	15096307993	14620971370
4901254.797	6235672.786	1751584.419	4508201.03	5452327.114
28074175.57	28548569.82	35317786.24	33513495.62	26207118.7
4438650.225	1663235.928	5571308.272	10533858.62	1849684.441
273644762.4	294594982	476875794.9	502152473.1	378993654.9
369748724.3	268462600.5	610648338.5	364308574.8	493679781.1
3249824959	3399585455	2360239068	2428358888	2037206972
128077.5539	289428.4486	460933.5684	370774.2498	
1640059.003	970306.4911	1133804.29	1272671.583	1060759.443
4216243.874	1316329.31	6451481.603	5541439.785	1565794.38
1490994.662		3152800.878	2061259.16	
4376865.184	3931970.137	2332631.613	1873027.271	1955177.348
5156697.683	4362338.359	13938572.5	9939040.867	5081404.479
3462950860	3420433172	893381485.8	4340933798	3339013114
1048767463	405918586.2	1060399650	1295960196	1645415956
3641505.815	5368795.469	6891394.021	9704342.294	1075900.922
35546929615	27310408819	34649323380	41612775305	55919225814
5996167.805	4285269.62	2116069.943	6613837.094	4869623.293
2687618.916	976910.3999	1993213.437	5585626.308	912543.5761
3513173703	2877802632	2321931852	2724210675	3349190556
4408833.223	3164897.752	4220809.556	5149173.527	2687316.773
6428198.787	10912786.67	8717488.014	11306933.4	3904957.97
1170631.041	889278.3603	1938614.16	1895415.739	1317127.787
1586562.619	872073.1313	4247440.373	3866753.724	1886437.281
113610.0987	110906.4131	184513.3768		509650.6207
14409799.17	17051658.16	20337414.84	21484129.52	14127725.21
10390615.51	11312473.49	10592160.48	10299486.29	8752268.401
473259.7073			1872500.65	2348088.547
266914.1105	300940.709	570374.1254	419792.5156	222688.766
1822385.967	2927245.72		2790072.108	456253.3885
288714.2013	1013258.595	1961814.732	1037992.764	891292.1448
175165026.2	158064496.3	372475758.1	564742820.1	948622287.7
3637428.001	2815744.119	533475.1039	5081374.732	6189596.071
31881709.16	21309089.99	28155841.32	28884728.63	25103848.95
1144265.461	965677.3615	755672.3728	3297917.266	1810745.262
316402398.5	324900564.5	223850191.3	518789711.8	147671218.5
2873505.477	983602.8261	5145294.492	2989522.964	888734.9772
		712804.9705	414783.6592	184912.3324

11683332410	11665156266	6115702751	10525887500	10891398398
768084.7798	558755.8549	641538.6939	794902.273	576532.159
239413684.4	221088528.6	228110397.6	282109359.3	264239991.5
2501251.323	1249963.92	3467172.188	877232.4305	1016409.039
32365931.85	33228041.57	38681958.41	29330682.18	13589088.08
340055393.9	270793778.1	307671313.2	302912165.6	344506212.8
1792363.384	542803.5306		2363234.244	697407.969
124686665.1	106385728.5	78181170.22	197590923.3	138265309.2
1448101.034	356740.9178	796560.6234		583571.6884
	214583.9317	2094763.958		528776.7175
1204237.865	2108968.454	1482063.226	3469793.587	1012560.662
155205398.4	124358616.7	34814527.65	144888599.4	118885811.6
9062252.904	13027973.34	22799863.03	16759406.6	7290596.923
6114133.189	4757090.606	2487772.753	4552436.988	2400469.216
11368634.07	3124643.186	13320581.52	11677665.09	6113540.894
583598.8235				
			112887.9297	
		37139.22205		
1699953.093	3596799.059	4342949.65	3566559.868	3189697.947
52046198.47	42241922.85	44132257.37	80682538.25	62837752.21
264407.6427	235664.5324	190905.6716		
842955.4073	1024148.719	116283.0023	183753.717	196503.4752
21720636.78	18272925.53	19420318.04	19899612.32	20554263.57
6588546.82	4805697.658	5184679.937	5778470.94	8610292.783
99808861.19	82002142.73	108852556.9	55966008.91	70091107.24
5569045.499	7634803.211	7814135.761	4908693.173	3726108.574
28793568.42	26518666.47	9536496.428	22336664.48	16395957.24
616466.6563	493709.6971	1395618.91	710960.1001	536824.8874
6249862.074	7476356.359	13197831.53	8376408.8	12483046.36
380751.5703	787492.3134	1950043.087	19177.55263	52062.40783
		385203.3861		
859859.5557	1307866.293	1236655.551	1112307.235	715931.1634
1415311.171	916185.5812	2469506.014	2320186.435	595162.1663
2161886.409	623522.2111	1141638.727	96402691.72	709695.7309
1970407.787		2029469.569		5942230.823
		1157177.362	1064767.908	
122431926.7	142152855.1	73751973.49	119922208.1	163706003
38506228.05	32976613.58	66985391.38	46269808.89	15577132.52
1480275491	1764262936	3313166702	1274867439	793098783.2
469202.5539	843152.5154		1628125.133	
17277394.22	12948789.24	19638455.54	20416375.28	14799121.22
	94124.55193		240061.8028	
657050.7512	521036.2445	326045.9914	502609.1428	411782.4672
17580275.65	10688453.23	4628508.651	17012007.11	9354332.21
1912128.126	4244282.145	10744660.96	5920265.471	6680747.151
1275987.935	2408585.796	7326600.383	7940945.95	4073390.809
732720.9147	1081173.478	1086107.26	1386268.403	11973549.48
35952728.06	12409241.29	13411942.17	19409618.28	59483651.38

1692581.989	3836893.711		6410423.91	9453984.873
22249766.55	16339338.35	24166276.08	18963507.02	24304500.71
1314575.909		1328236.08		1005763.432
1155774.057	316792.5509	542004.0548	1605738.893	636047.1089
106445.8872	74989.48684		906254.0888	1224040.223
166336518.8	187542820.4	417342763	247309209	159227095.5
8155142.847	3919083.556	10412328.46	3773327.88	6591356.325
2004081311	2044837123	2308763999	6508713145	1092684736
19149750.36	16020021.92	9728449.715	111522609.5	72938000.96
6105025.263	4740931.263	9968743.544	9490064.403	4618236.478
2515304325	2385801526	2641700477	285125725.5	265530332.7
1377578.377	1235859.158	717118.4658	1045319.137	1508077.349
45375081.15	44701831.82	37900019.42	47066976.48	39566602.59
110943.2297	394495.9874		214268.078	
		789279.4763		
10240456.98	4674484.842		22427996.05	5520476.127
51296924.31	15364589.92	56160372.33	1442006526	12798530.88
2580351129	4084960842	3956606165	2349213843	3037871722
4042379.247	4757470.488	4510868.113	3674204.187	3317087.656
19419286.02	19265247.46	15997902.62	6875438.428	15033463.99
4781414.038	4941124.636	2525884.193	5954400.266	3964913.012
142510.275	163710.5677	289540.0599	401791.0126	
18482.84724				29280.13531
555131103.2	339596802.1	443546765.9	563638127.5	559271193.1
56932490.77	71621806.49	124442229.1	61828865.12	53302126.15
309921.5525		95065.53102		551664.7082
			929667.4199	
155990851.7	339428533	589906240.8	352568299.1	340448450
8723356.92	6199483.877	3626497.194	4643487.434	4887722.771
	197370.0521	416738.074		717109.5757
4306770.787	4825782.815	2687850.859	5519486.016	2256028.874
250283974.2	174729330.7	181369878.5	261908244.2	246819490.9
32583672.79	36190718.9	66119769.82	33918650.75	35758668.92
				967628.1548
279230.3472		468969.7772	551090.9267	488029.2773
505454876.4	575814225.4	232717935.6	685553472.7	454455748.4
13393029325	6860938991	3994241063	9099510717	3727740978
524075.8571	572482.4857			
5994723.524	3152989.779	7633634.378	3564199.759	6289499.094
313402.4525	205720.7885	88418.98114	366116.0243	
532141.4135	478992.5945	357326.1706	827234.6403	448997.7844
	3480593.536	5604859.096	3688683.995	4740428.567
4252817.689	2700795.756	4885196.476	2716689.365	5828838.953
350096373.4	433797311.1	36433412.96	179081408	149146890.9
98975207.35	156526731.9	72712360.66	142958820.1	55156538.24

4570247.98	5141390.045	11847735.5	13199513.22	3190121.177
293108267.2	148389695.8	249058412.3	44846725.37	255431032
	809872.0377	4136282.602	956823.5171	552494.819
1718676.341	80140208.08	199127269.6	116328247.7	50224994.51
	18896.65064	20890.61433	14221.03715	33716.57738
77845674.85	133848004.1	173399300.7	187252837.1	100657434.3
14890969.37	13341103.64	6602397.508	17367786.11	5655706.769
462443.7591				
	583517.0536	710062.4954	1300752.117	16866111.76
83263503.54	99784100.55	57929788.73	72481449.12	59693017.33
			785999.9558	
1086667.539	564044.8521	1470232.719	850067.1387	51290.18774
1513806.753	7786899.582	11648769.81	1892286.135	899354.0677
97684458.92	75118641.2	31915447.41	72239056.32	62799036.07
6611969.286	6302288.084	481842.9923	3830362.731	4637331.18
748908.7052	855869.2315		1061089.368	
995531.5533	460798.5605		213347.6077	111249.2553
273307116.9	193400775.1	309712472.5	86498630.49	115388687.7
2866761822	1920848370	4483969869	4075178307	8923346276
859384.1538		1462338.776	1814663.388	5943311.731
		591558.7224		
834594.8596	1649154.592	972135.5621	912601.8855	736883.937
		470118.8626		
7401120.929	5816073.169	4674890.855	9411134.62	3518982.602
	899960.6462	2236453.037	697894.3786	
1073389.595	888801.876	486840.6043	1297714.667	603450.8932
2028995243	1626466497	2352783577	2583420871	1874244276
380644162.6	550879369.4	716988163.9	690504709.5	1083965003
10173289.14	18790534.85	15329400.1	12399480.59	6622844.292
448739.6697		1149728.769	1080384.408	
8646278481	9370853542	4697696990	7417097030	6101497457
13756686.22	18969120.61	30300172.82	22047871.91	20293973.24
21173025.47	16672851.34	21845935.81	15224547.17	24351410.05
44033003.17	29494703.73	37738091.59	50558227.03	74169201.74
407899.5494	458628.4022	416261.7379	553964.6961	1224553.463
6807140.162	6826592.789	905855.5148	8228937.685	6868679.81
6089662.131	8324384.653	5885820.7	4480523.355	4212408.135
2475390.403	1579850.284	1000215.879	2105828.532	863068.4895
11388792.75	19181160	1681604.007	20382079.36	13042259.64
12244697.04	12752663.92	395517.832	11178785.27	5506064.271
3865203.16	4387943.463	4661086.748	3567793.308	3035952.544
6794919.97	5234653.4	3994712.8	8227198.086	4392352.728
672055.8196	1013556.571		560906.8564	
2990738.112	1629491.909	1372644.186	3962334.298	915336.8397
384439.0943			525355.983	
1485105.407	776958.2938	737454.4431	1480660.699	948305.3722

15927788.7	14180172.12	29498698.75	56776103.52	17202162.76
31300316.27	44008029.56	26266962.63	70655938.52	13768485.1
17073673.32	9448741.316	16167686.72	11045306.56	10223556.32
116196873.5	92804760.96	103046882.6	100655722.6	68595699.27
401286.8715	147259.9811		302728.9917	294055.8042
87522.98494	38164.50704	50003.94964	76909.69687	218828.7603
1011559771	1598109721	2467583117	1996136151	7894724534
690625.2915			432994.1403	
6447372.602	6824225.52	19616463.55	17340211.48	9003582.579
11536074.41	10954894.64	13505386.19	14801903.73	14507560.16
1587105.287	883806.5363	2760739.964	1389048.781	1668453.13
2239499.252	3026846.277	1857712.16	2487012.577	2456854.638
2129825.772	1643092.291	1304460.489	3872805.929	667673.0397
221335.8257	138676.7583	441358.2349	253751.5355	80247.97879
144661.0423	121058.3858		12743.07492	
3743089.604	4529393.855	3596468.133	4543151.084	3902454.728
1069253.293	1238584.771	780057.781	2519948.735	586092.203
4140374.841	4857482.344	1915814.038	4066727.797	1796001.29
45060174.36	31515024.28	26921964.34	45557052.84	37050355.54
		613864.8794		
94400681.15	85976604.86	45571154.04	188453245.3	38702958.2
1825778.691	2277529.528	6077331.586	2113702.822	512458.578
5410679.092	3954755.532	925347.1251	3141565.804	3023108.888
304103.6812				
5095502.959	2711817.287	7823015.92	5248924.539	5244395.57
29739205116	29130358754	42417809502	29183530655	32357193126
358026302.2	164155019.1	459838719.5	341463630	357663824.1
6725456.953	5084140.538	15049983.1	9450157.882	15517587.18
			213652.6913	
16129357.61	12562180.01	15972389.46	16722020.74	11355369.06
	55641.08966	110456.0445	67855.72919	32399.72476
1800759.437	1396679.332	2210298.029	627803.9799	1165124.279
662809.2644		178976.4002	927676.5394	267336.5302
15264015.02	7795774.142	30577661.83	19720245.94	3099996.154
6214450.857	5070084.404	1298838.802	13191442.88	2454893.946
		52202.24427	378512.4122	
279437.0862		268696.628		1455443.677
148334.446	236642.6382		493744.2463	
3083763.47	2290916.541	2945874.084	2923698.925	3792378.598
160746665.2	130641406.4	61276549.8	125994328.4	103769061
334035.3884	488899.2864	826128.6435	662297.5724	336784.1318
12359565	10986195.08	19101785.14	16295509.23	3474164.974
433282.1768	375588.8226	673895.4961	290540.7146	488627.5007
30656821.91	30773764.21	23604576.12	22200873.12	42584593.9
45769929.04	48410701.06	41015025.28	57067135.04	45332953.91
6636101.561	2119679.792	6331043.863	5044064.055	8996099.645
3673182.692	5444403.269	3849768.29	1540757.468	3592627.304
159273524.8	109911120.1	159697510.2	152529493.6	380029411

891628.1509		1324649.004		
22641239.56	21931595.98	35156684.38	29424321.47	42023749.13
10827838.57	7312403.481	28651046.53	13677420.14	16962294.59
141673.2284	1231697.329	147351.0874	2121953.504	1873197.285
8175305.474	7570710.827	14640915.86	15799080.25	7188819.849
8953477.236	6596430.241	13068260.07	9043693.949	11057143.57
1755098.141	1900468.407	987718.6927	865630.9044	657645.6529
9187213.826	3490843.161	3498357.165	6639311.988	5493633.465
6123199.678	6831634.652	3955718.598	4447717.591	2547645.676
			1392311.357	
9944305.11	16506408.3	15051301.98	22066269.89	8065565.503
			535945.896	
2274381.99	2692936.419	4862083.394	3250560.385	1750032.118
464462.6278		712396.23		
3423118.13	3063773.011	1804173.613	5560545.12	1337121.774
				675936.376
806456.3833	1402085.182		1613351.195	457233.5904
697048223.2	631039333.4	855737652.2	944844185.2	626944143.1
21241393.19	18229680.69	42893721.26	34398160.29	12255825.16
4303980.644				
186196.1432	124499.3925	97036.57335	262893.1368	730610.2296
	370324.4457			
				236074.7899
94371880.94	110150952	128566975.7	156070446.6	290835040.9
15737.61699	10013.8259		26829.27188	4001.964878
71759005.29	73103995.62	93995502.64	102108168.6	92982198.61
428560.5944	346376.8888		824148.4719	72027.47463
395828.6344		661234.8115	485031.5704	1190336.788
1307186.079	1002653.857	1250275.606	1112324.195	2143923.186
691135.1595	623212.7282		666313.8955	
19855159.97	23034080.47	46585445.78	38637990.88	7034394.105
18732514.38	17656566.34	12059285.53	21378081.74	10562163.38
7084402.631	81615494.83	6614501.756	11523966.39	6089238.031
32888641.22	30528438.09	44436644.61	44683419.26	22044133.65
176440.9455	123148.2581	7140.718712	745974.2346	40817.96087
83011055.74	75542115.31	27997603.44	164068830.7	70626797.64
1763498.071	10401898.76	1629504.6	8119121.389	3125348.495
	411063.6691	337684.2564	840973.2287	591578.9885
1175553985	884590737.3	434424601.5	782441765.3	967677261.7
2079584.631	1574973.406	1547995.013	2933348.983	1599125.235
215551940.8	152916739.6	154275394.4	119151338.3	89671075.47
		1687820.066	190581.3345	
16231101.14	14081694.12	20628955.77	18557956.59	12533511.02
91252.37565	102722.4687		322326.3213	
347137.1642	1019989.684	96781.90219	297162.1646	71854.67813
156327016.2	197157139.1	359028689.3	509715427.3	613069974.2

662117.5688	951940.3015	1945346.808	1077263.999	
1460522.067	1318880.114		1800213.645	836675.444
15743873.76	12898984.3	25283113.92	24139252.53	11497909.71
12325382444	11420499015	9228592524	11833450674	10034905881
45898638.18	45195980.64	56918099.28	38469633.42	26367300.02
	130281.7166		614765.5049	
9344723.77	11588916.02	3394394.981	9073619.214	12047815.21
3020370.712	2232897.399	5835147.229	2527832.935	2129571.978
256994622.9	330062730.2	503342531.2	500070260.9	261870134.2
1432732.464	1098402.443	2655686.182	3030549.988	1510227.165
792732.4568	833085.6242	2258155.583		
3193520039	2930280559	660303732.6	1528493738	1666868402
159105.3203	87267.95029	99945.91632	164954.8146	208666.0328
17506365.46	18297482.58	24490152.69	14967923.66	20347045.36
2345227.48	2766906.526	2307532.72	1860976.583	1918726.783
2141341.649	2033377.619	5244715.892	1830385.855	1810155.099
6180987.536	37049044.21	33088692.6	18840662.22	7818669.796
541084.0547	807094.0832		574760.8871	246123.0819
1875168.559	1131498.101	1859890.648	1586058.68	965809.6438
1402724.51	1459550.163	895842.9149	1810697.495	1188049.767
333482306.3	269787730.4	12781530.9	432199924.5	61566686.15
4635467.468	3459594.704	2919025.662	5910620.44	2776109.449
84871.23311	49347.59723	104355.3041	176683.0734	125881.0051
963412.7637	899562.1035	1593248.44	1604259.686	451799.3761
34524421.72	31688030.9	5719505.208	27248819.15	28868394.46
623787004	675652784.2	209667163.8	955039325.2	119637651.8
990509.5607	1048800.263	1074067.339	2477867.089	3627472.304
543955.3458	594993.3706		983692.6148	198945.6236
16930859.06	22953571.71	305952.5667	42647117.66	
1128905602	1014962900	547091749.7	1451546700	461576185.7
		305621.9367	642253.1532	1010737.417
2011307.514	4135712.627	9723632.681	9724774.556	16996008.28
7471288.701	6297290.433	10361722.1	6780961.076	8886769.421
232406.7845	294319.797	500634.9471	469577.1199	766265.294
				594153.7204
1356179989	1199515049	341051743.5	2129123382	364711960.7
932855.5357	806746.6433	2399967.756	1829476.225	478652.1581
435153.4543	10006248.95	434470.9898	10599149.08	6762668.894
455089.5439		895908.704		
	43199.87086			
2227008.464	2255415.398	22071213.99	4293714.322	637138.5546
26439192.67	12928610.01	7026006.994	21551843.2	4891827.574
708789879.8	651819537.5	814582408.3	863648535.4	921557176.3
32105852.76	37438944.03	39473152.4	63540913.42	50112718.87
	296323.2005	942241.1545		
106690446.1	97753510.2	293926143.4	274048545.9	82986397.06
235132968.4	262618267.7	112243320.2	441459952.1	76340508.88

198665.1604	43481.95158		1038700.948	
2695507.106	1891427.149	3182845.985	2758492.813	1673509.307
921403991.4	1084614925	811470378.6	1473235918	1041530767
6588178.875	4955194.908	1300842.833	8538693.074	220465.4818
2259683700	1305380434	2123530289	682500519.7	1715238062
30687706.81	30836401.87	33088321.43	20282674.81	22676657.96
411062.1745		498127.6155	527978.3589	347382.9983
			753991.2057	
15315167.14	17861436.85	13431313.99	20279683.65	8001641.6
15631661.12	7736953.454	19289461.19	12809707.36	6749016.503
1163026.105	1184773.877		2225981.789	842261.6307
2326132.209	1687109.696	2782563.614	2503465.744	1840224.739
1138631.868	1464837	1381862.689	1239836.396	1002207.954
1086649.808	1245242.865	1746000.795	1703339.341	
		632382.5542		637411.4739
2612446.132	2609383.872	256283.147	4353971.765	258626.1522
6716914160	7316536442	4462931075	6482018499	6812974884
34617984.71	72179832.53	9064836.123	51126377.07	25285247.21
685599.3737	773924.676	520188.1953	702687.1261	445359.7406
8666439.195	8821571.617		215638.9143	31322.98217
1133905.345	1167789.029	1599661.532	696234.6316	1067284.013
3556991384	4480405985	3442927113	4371828324	3527881447
722024.9467	873145.7979			
7400923.63	8434553.781	16917533.78	9759944.557	5119033.198
24433159.79	18273323.02	17836887.47	28564313.37	12501030.76
2009921.404	2191646.281	4716837.2	1879886.479	942580.358
680602.8669	530726.5128		1031491.639	584062.6449
1251405.15	916742.3957	691058.9845	972263.4169	1089335.162
8204154504	6744327336	8291850460	7261466272	7538874401
348004.1006	822724.1839	1171017.134	546119.0093	244580.6204
483452.5594	1066958.831		1767448.706	19188.46781
16544926.78	19600233.77	18566022.18	30093760.64	11267168.41
9460172.518	9977905.094	15632698.37	9765510.549	5222716.741
467385.8861	291174.6617	82629.98249	358250.9847	678953.1504
24326551.74	28340142.78	33954527.41	29811096.06	21733876.37
4191185.859	1699500.613	7757124.025	4625991.898	2083593.61
2743210.664	1710761.996	2741834.619	3640667.803	2152534.061
2285239.949	1233404.265	5975544.419	3102558.698	1248966.643
1462751.638	1401883.489	724136.9237	3962946.971	2833442.604
34650255.31	39826477.09	32591722.11	61083560.66	23562030.37
29288530.72	21478806.16	93742865.31	33545065.95	15095803.71
398907.2963	584175.2078	1547505.797	2765182.167	2062518.154
629583.5972	619895.4374	410253.3579	574293.4007	540713.1289
3490179.007	5408575.471	5042588.369	3896136.71	3186264.107
34881579.45	28909506.74	59546718.95	26521159.28	17564439.2

1110757.785	1827419.101	1013213.091	1866202.143	1021684.226
373245081.1	324606291.2	241221417.9	155319569.2	211142810.1
1176995.736	2480316.856	1954145.914	2284705.65	6932287.747
4112111.384	2518800.914	2637621.489	4194549.327	2176585.813
58426.15101	1064221.992	1961958.955	1616386.757	14655.36443
8207649.155	11458402.42	3113095.764	14028779.83	2773057.408
947572.6209	1048604.12	1490403.351	1298530.128	633666.4388
2400898426	2526239703	2158338147	2385993744	2785528634
247104472.4	229160035.4	15312308.04	161755427.5	122318161.1
20080372.1	17439263.64	6447533.763	31772066.76	3344679.338
524297.1292	713970.5905	250497.6068	1455104.54	1324558.888
47166388.27	71175579.18	25795972.84	34391739.14	19967910.04
10535709.55	29157780.78	40961472.54	31648389.31	13135703.18
	636799.9808	554426.2219	408892.0906	
2106494.081	2278896.493		3266197.121	445320.5413
5748376.5	3006697.484	4577401.878	1299284.068	4565171.122
12941727.75	11497649.49	17483802.51	15291465.56	5838048.051
1819954.157	1473320.82	2956156.742	3023490.859	902408.4682
5853233.827	8676067.394	9884183.584	9060022.005	3715138.912
1033627.179	3609820.446	4641797.224	3707440.755	1194467.1
2960477.599	3545755.197	2414397.091	4453542.252	3353535.205
5905813.95	5636299.412	5258817.196	4911826.672	7152335.007
310991.1685	501951.5233	841293.3713	1079748.288	
73796.76547	42234.06589	228327.6155	56916.32963	78691.13983
15753050.54	23730504.57	22170981.91	27836276.37	27478916.76
14489619.04	12998150.12	15723966.1	13316859.62	11670807.47
3079145.86	4032108.578	3873687.852	5384004.341	4848094.579
829816.6188	1389485.85	248921.045	410507.4466	312114.7697
5831573.515	5203699.034	5767542.287	9477409.186	2658370.047
754724.8915	816097.722	1108119.813	1339427.511	1290830.153
	679226.0829	850374.9782		
984388.388	934152.7811	2039959.032	993129.4225	1259453.701
16945238.45	10610829.25	5981994.79	28479954.44	5552027.443
35414084.1	28519635.06	9252657.191	18613399.61	8441544.55
1489267723	1598515009	661425689.8	1603900847	1321929794
2465892.628	2264700.959	2423825.689	3190719.796	1576761.107
2055370.039	2129519.849	2716448.387	3101781.495	1260069.181
3548847.598	4664650.856	4473866.298	6399696.455	1673073.168
4929546.8	5031833.593	2330744.699	1083104.785	2749576.952
856116.0051	690831.5725	1204342.802	818387.8599	1100424.807
3399720.758	4267148.106	8530349.582	5764433.114	4072586.355
			682127.2961	187611.7821
1355059.324	1115425.59	1767449.339	1369709.813	1923425.518
		87323.47496	119793.5606	
2300463.073	1990477.046	2614492.35	3480695.216	4866122.503
8764468.084	10923998.57	17456052.65	8291589.729	10170914.96
1024188.074	865756.3688	819696.0551	582426.3922	1002078.825

514178309.9	311736650.8	100415849	360668424	85777621.3
11403830.81	14522302.15	7503505.799	9811159.919	4942362.599
2436165.123	2954206.632	4249667.071	3019503.445	2048011.033
97209.63349	313620.085	268846.6173	195868.582	701060.9822
4525206.36	5237822.634	4105839.188	8897873.568	1684258.97
567800.1932	434266.1187	249611.5152	399901.5375	349191.5189
1927186.579	5126409.01	1767523.086	2901988.752	3143631.531
1635232.874	1848143.286		3176369.515	685989.8903
8441100.816	2229991.65	1446878.901	2849497.262	1338175.274
1902599.256	4384919.008	1722447.956	6768872.213	852879.4076
2708130.282	5913495.956	58507.09614	6415664.349	59140.94194
	544109.3354	1023170.721	796025.9235	
1889462.018	1648173.059	2576995.595	1766408.024	1262441.737
332369.7202	164443.9794	97576.99343	168350.708	
34427094.9	32720470.97	21915153.57	21081211.29	32857665.02
34555961640	44563194172	34678580249	36530622072	36663062665
509595.4388	392358.4947	1072855.834	339591.4224	
732747.4153	610943.6091	612746.2425	698247.7076	697388.345
12751618.37	14196575.19	22241261.12	16118806.81	27081941.35
1324603.388	2912074.949	1682826.92	1995674.228	2107637.3
901454.9614	651242.2752		660933.7224	
129999095.7	109382588.1	86512724.08	128020507.7	140490480.4
11559235.8	13305819.3	19011546.11	19032339.34	12935248.5
1349671.704	1240380.42	1128121.141	1178606.277	845491.447
3716491.981	1368744.383	1137586.541	3153203.305	5513780.098
56123745.51	61483251.12	54075346.48	39689906.54	28728838.98
105975942.9	110657492.4	51551372.19	133632878.2	122753834.8
11735266.14	17911334.29	11490062.71	13276746.45	12246756.51
9721703.451	4620011.962	4487812.639	5224830.503	4037344.27
2590074.943	4753200.196	7796174.644	8756987.256	729484.5589
14647662.45	10870218.58	7918337.554	14333767.91	11650340.22
832305.011	1479597.533	1194191.088	2346792.046	
388647.8218	266837.222		549401.6285	468450.799
610201069.6	905297823.5	821905984.6	965043965	779351944.9
1076714.889	1717353.558	2540440.844	616109.4399	579020.0264
5170279.456	7347184.416	1649089.345	6939719.576	
1061622.124	2980311.795	2050879.079	1633161.742	1071359.834
1036194.671	399842.1959	811931.9341	919277.6839	337420.5977
33725302.17	21298762.92	3835606.423	34374268.85	4086074.92
82495.7555	35990.87318	14441.6929	88803.05667	114296.9253
3253986.367	2175615.981	1089858.197	1147529.031	3564806.875
3485780.907	7474638.379	8128357.224	1015140.985	6405411.008
376623.9529		1270187.05	1557433.056	
2497254.485	2060271.747	3177543.563	2103306.212	2524045.059
612507.578	813437.617	792437.35	187189.1992	
13911504.54	16223700.62	18394800.68	3398610.671	11154226.15

247102.0987	346873.8567		545331.7922	
31042.62984			18817.66093	
3230553748	3485948137	3239208821	3596228138	4213559850
768367.7845	1198755.836			
1523253.822	1563097.783		5635942.934	1243295.57
9907954.634	15066459.19	13604731.86	8449691.743	9055068.913
918050.0258	1715397.867	119122.4964	1180331.39	1598895.878
4768131.507	4782731.317	6008392.676	3342238.086	4282459.085
15712282.97	35195706.98	26256667.34	17397791.03	9053129.415
350833.3431		1185528.722		
19148517.47	22184387.11	48515780.12	31995764.37	8033766.966
38607424.7	39356711.91	78533773.71	74804834.64	68350118.93
12698075	13414726.59	4627791.65	17463087.94	2142526.412
36365227.25	26976836.92	26800544.67	76467143.2	11588921.88
341314.4676	246374.3811	212473.5136	417565.3543	
129222373.2	656652427.5	356428401.4	357984016.7	194914485.3

Intensity N_LKL_6	Intensity N_HLP_7	Intensity N_YGF_8	Intensity N_FQ_9	Intensity N_MQX_10
797746.7728		375519.2113	424175.8024	
103617189.9	138109727.7	119396761.1	88247985.64	40862146.96
	345265.6139	300478.3637	234069.1855	492959.4637
1306435.768				
4531320.108	2753944.603	8456465.999	9982399.251	6310513.053
2831097.618	1497788.695	2069870.811	1527377.927	
4982885.476	3099480.371	1518946.171		2428522.275
137260040.5	88979238.33	114726488.9	65922702.5	88463680.96
2534421.936	2979353.308	2571993.337	2717965.446	1836197.945
4163997.71	3361572.998	2060560.97	1508986.561	2841707.454
12315649052	13931575861	10141741351	13598995419	10831827610
49134.45975	43090.632	234387.4491	149958.8339	38235.19134
1013040.33	656199.4776	854947.0672	797932.5086	807067.2227
4905567.714	3377358.486	6577829.083	5778319.062	2963164.912
36420754.62	16556982.08	43927439.19	36395543.73	65220647.56
	4480777.313		5412994.476	565272.8062
	275647.1669			
23857704.1	21211946.31	6050413.031	10644195.52	14522041.97
1132444313	1242819321	1670473707	1734533990	1921467685
794291.3035	2294947.46	3442963.321	2468695.792	902161.5612
62174465.38	38831203.6	126312503.5	34005055.36	54602639.96
2441080.607	3076595.655	1614204.761	3050239.032	2342692.485
3999692200	2958456529	3192530935	2062199815	4707460019
34771.48609	15796.75103	55139.21316		25098.78918
70399891.32	85303613.44	133120624.7	100791606.6	120476115.1
536470.5809		65545705.96	37611750.97	
36990652.79	41214935.78	35624687.99	34069401.65	44555057.93
3625155.766	4989615.159	4868685.339	3980402.074	9963386.638
7805408703	8341715571	6841973008	8375141830	6109546453
86653.71974			355546.6427	303213.8281
	1657651.003	628772.0684		1465568.774
	600274.7394			
			3828397.408	2119847.227
			4717.784352	7895.786635
8637027.838	4686367.7	4743373.21	1436646.016	6385276.481
19195968.7	15269362.22	18512684.51	54149638.38	28567691.96
1123578.228	524787.6563		127859.0232	1425495.25
18036344.55	18056368.06	8297940.136	13774878.11	29068560.11
	4702.286118	1817.811623	1251.928303	
119660192.7	11394028.79	50828939.11	35687106.09	56883091.69
8539922.984	3539722.163	1393914.033	986158.1288	2919108.427
61878728.34	60402647.81	51399841.08	74911447.31	58022836.63
559987.1525	453354.173	338072.6029	319220.8296	648369.9738
170568.6166	153756.0507	154467.6472	145079.151	
174595.3435		432510.7412		

6824181.944	2507119.514	7066646.106	5521750.605	4541630.878
1069205.034	1051203.327	528219.3791	678254.0606	
1077084.365	734696.9452	932804.3359		
	546567.8082	613669.8382		1159092.158
290125.9611				
306353.7394		1136390.396	851528.644	192449.1552
1421551.527	1251118.227	894009.3711	744621.3754	1020372.304
4116059.414	5891285.422	5091546.968	4742780.629	7704191.188
3000636.685	1869059.781	1848243.196	2304939.04	3285985.915
1802664457	3111395425	2624786682	2976834360	1366370298
2278097.642	3867718.88	1927384.447	3371737.731	5494176.263
3182082.029	2119092.15	1173388.681	861435.6792	1010487.216
	1536260513	1579967442	2304199449	697728.7741
57022718.7	21245475.81	23092715.57	21998821.63	48023215.6
5888593.317	4182009.421	6090156.494	3346156.315	1939879.725
12419831.91	24154143.85	8955702.234	8796965.148	38168681.61
141641647.1	158767573.6	131354692.4	136481483.3	95781530.91
2301073.976	7470772.339	8763424.236	6889976.41	8628683.673
4312150.482	7180572.578	8478298.35	4030998.28	7553122.245
	65225.88703		64601862.49	
	75128.53538	355769.9838		455153.5899
123851.2956	283045.1169	185624.9504	124129.5007	
4557824.427	3488041.341	3056056.801	541548.471	1154693.84
722178.0853	1724112.458	1199310.349	1894369.948	11750885.61
1704915.051	1120350.162	2470268.627	1948843.911	358387.2193
	86557.19455			276544.6811
1303086.087	1878257.466	2069051.935	2185015.685	5343034.596
1695585.304	323905.8723	1517506.62	1070467.639	1774273.979
	1841450.452	2040682.035	810641.1936	5061486.524
	10159302.03	4125511.694	3784332.566	9596631.062
292201.3247		338686.7831		968604.6781
29407287.83	21569986.88	42460365.49	69625840.56	45997671.9
10069517.27	2018475.159	11165167.51	11612796.78	10632608.43
8158362.651	11576715.4	12690283.7	8155533.176	9740661.449
41520695.3	29211879.15	18919540.95	23846063.72	7010710.118
72487527.45	5116707.622	7450257.309	7442797.813	13652290.11
83616.62797	623556.9045	152746.4442		579438.0581
498417.1563			238736.0192	524504.078
		211136.0412		281087.0188
865064.6631	5029011	1778742.894	1209094.919	591614.763
1318571716	1576143310	1097089339	1003835411	1053534325
	320876.1126	1135866.389	203744.601	

1078770.328	1001007.418			
1080677.407	1042945.198	835439.2691		542832.285
293977.7384	105081.0704			202004.2532
28602123.25	37600592.64	29766159.81	28332880.14	38466466.89
316546.9974		729470.9187	951649.7791	
458082789.2	267031127.3	334630858.6	279728402.3	379485180.3
22959654.06	12676737.08	12003707.2	16123500.35	23035669.36
12830416.69	5074089.443	4363534.083	3112550.529	7329106.913
11656102.9	16707943.56	9401929.645	5419972.019	12526222.38
915985.5173	640770.5712	367941.1962	318307.1673	
393381.7627		367639.1299		340087.0599
17319658.87	16389047.19	9158404.123	11699716.16	24683275.41
22707893.9	24300416.61	22103904.06	25294299.33	37341400.85
	178340.4907			
19992998.62	10372461.65	17110377.88	9768108.672	10654294.86
62360.09082	30806.22229			21606.7702
13902550.5	10483539.87	8690095.71	7142111.828	13413581.66
170802.5908	171877.4746			110848.0401
11112054804	9762183609	10787527200	10678201078	10303142486
85489.2161	138667.1581		102656.369	
41911.04356		57989.99808	141970.6198	55026.86217
1408638.242	3833634.779	2709788.271	2145563.762	3917184.333
996471792	1727944972	447958818.1	598112530.9	1283662469
888681.7914	626440.9161	1425682.801		811943.5869
2241614241	3868547837	2013200626	4043293233	2217871902
144362.7768	136578.6195	166321.8098	60815.22663	98037.93586
1246366328	1256703442	1168877277	1063858143	1045351731
130681.6752	55043.66617	160351.7427	42212.84707	58596.81671
323812.343	682173.2482	297240.2957	158747.6779	269099.9083
		701301.149		
353811.8922	175480.1137	4504863.937	1094578.348	490577.7162
6034297.703	4180849.387	4464934.411	2986938.894	6393273.625
13044279.26	5460797.157	9761524.386	5614142.449	7131438.652
2866083.604	6809047.241	6327398.302	4909163.741	7974994.827
370645.2241	532960.2784	1116642.319	304660.414	2022802.789
25264847.79	21153823.67	8674021.293	16074931.49	4588399.537
36574290.41	128244546.3	29030769.23	66243401.68	42674109.06
124622.2721	29643.758			
6041781.764	608935.5674	6778196.404	3164332.635	6828654.872
1410167.561	667176.4056	1177937.663	1139444.894	457544.9522
	495574.1866			
272738106	264276625.8	232642508.8	304739368.2	259818505.9
3033514.495	4025706.255	4475869.915	3792530.063	3795967.71
102593038.4	214846688.8	132590201.9	71298036.25	152723856.4
3518205.511	1258419.531	4174600.875	2808197.005	6026739.544
12354731.95	10744577.31	11109883.72	545708.7281	4280448.734
4869514.575	4482085.243	4943218.105	5666889.794	4085839.23

138090.4716		314102.4976	280461.9765	
752657.0311	600225.1502	716371.6231	606396.3938	681820.8588
275838.4384	342374.5978	544821.0787	597732.8876	641257.8457
10738737.38	7493651.955	812615.0351	9652989.061	14988141.77
350661.6645				
199124.7871	376799.5757		138838.6245	237354.4757
15191949.02	13125100.07	12828010.61	19687882.81	23125194.76
1458019.362	1497647.029	956559.3048	903984.5418	768134.44
71285013.97	78250455.33	74742314.91	53208041.63	125276661.6
122555.6082	59765.78189	120852.8723		
17606384.33	23049334.46	21385369.44	25817162.56	47622494.07
386779.3089		417461.3207	965404.7575	1215226.539
5626157.195	4370950.733	5769440.884	5043199.602	6576778.589
76360143.43	68696668.38	61152543.27	70776445.98	52442550.44
962162.8036	1872923.331		535964.3123	700203.1491
3366923.493	459642.0861	1099115.315		1329324.228
4869051.686	2720406.209	2178867.812	1401260.067	1596339.677
4591127.149	5610161.445	2500374.64	3422079.347	2055523.514
1487039.265		824028.3182		1260199.167
1486176.468	2592771.209	2702367.736	2543265.455	3555193.675
423901263.1	484685161.2	263974830.9	495438586.5	407686396.8
844909569.9	1613802458	1268170774	1749063349	1286418055
83666.22323	105291.9751			49359.37297
119399749.2	100273955.2	117320308	91693200.89	113003684.9
26084821.11	24660107.4	20650496.66	12655231.68	12807342.05
174315.2685		88053.12546	191468.658	
628669164.6	859661881.9	584685040.1	830726270.8	769246046.7
6397347.707	2125478.534	2245542.791	2689717.218	1679716.206
238673258.3	401619831.1	516118937.1	682910890	84655427.02
17669688.16	16561221.12	12711244.13	12477682.98	18231016.77
8654776.895	9366123.386	7700098.685	3217335.082	4292071.032
6119594.36	5480853.446	10953455.98	9689858.888	20148411.93
1386852.321	909116.9653		1733474.531	
16703466.11	4325111.475	5401042.755	5001150.912	12176915.52
71498924.86	83743245.29	48202236.63	46443374.05	34195457.88
152403.4041	100143.6312			84798.01405
104412400.2	105619302.7	98122107.65	79385854.12	166940623.5
	1314467.534	1001785.104	1621072.558	2063093.713
161238638.5	108065637.4	181415229	54367250.99	192629597
				736678.1866
1050672.993	1727887.757	795017.0437	954596.8267	2024509.55
6418437.639	3280561.782	3506849.312	3751112.07	2505935.733
1894560.6	1336985.007	818254.7141	669117.6495	2071282.367
81611589.6	91866080.82	111191906.3	72477036.87	138825338.9
989933.8171	1212383.025	1087303.579	382973.5201	567829.6272
404591810.9	563177095.5	552238302.5	566863586.3	708840681.7
8244166.27	4822041.75	7513594.532	6060123.445	6685292.343
6277626.393	5713582.295	5085669.398	4996084.459	3844522.06

703738.9577	448508.7052	24070.29755	18893.91799	34580.67264
2766438.097	2338674.986	1411866.805		368614.1639
31766076.07	37217173.2	36872236.56	30118873.84	50435646.96
297012.4698	736734.2419	800892.8288	7183.630488	
1059503.529	387026.6438	787120.7647		
80552006.27	43546096.01	46734799.82	60461080.64	19975120.3
12971669.18	7576570.283	5644798.429	2501763.555	4337369.759
3663768.206	1984216.522	2258508.917	759483.2048	479259.3616
2373642.851	138636.2268	349523.2649	292636.2978	
357125995.2	339112064.7	227973624	326895545	209011187
2174481.076	1843222.788	1280037.935	789786.588	2221995.417
		324300.5507	355656.1004	437245.1431
4485497.942	1243378.377	4840867.282	1981951.515	1141494.96
				625938.2039
669850241.5	603121914.5	550758943.4	207607955	726005375.8
21518189.36	20429714.2	15394946.97	8015427.053	11192002.71
5053608.405	1786481.121	1861261.708	5126065.811	11550712.6
19794741.99	1119712.41	595891.5655	2745244.434	8573798.128
794816.5577	392130.5434	937348.7169	608766.7071	
2515244.963	2654368.591	1202404.684	352268.4396	1906487.263
709076.3086	4834407.105	765023.5665	433569.0588	1339955.149
1667911.422	1922025.726	3417804.95	773738.6143	2976273.662
20763906.28	14223267.47	13413234.39	3058028.387	13478597.57
5947480.377	4307468.667	11088620.63	4892200.811	4909681.01
682993.9827	666304.5828	1600129.652	1737143.32	1668192.585
5222518.252	1829567.759	2502771.124	1950713.356	1476286.25
189486.636	142413.172	321837.7597		129915.1343
11081202024	12770138576	11331119586	11952983970	13543833868
276873.3798	1299381.555	508778.2241	1771631.226	170405.3495
900247.9351	1477223.178	2824915.678	450360.1513	1329352.433
324895.5956		64473.28235	1678890.336	26391.37048
743820326.5	592340364.2	443248346.4	446115567.3	306178352.8
		504741.1739		
5233468.808	5572116.737	3124491.676	3393447.519	5253960.176
	1404283.103	2401471.208	8027906.574	3743887.221
15079020.85	23794043.36	28860470.21	36374855.58	41083879.12
786564.5931	694006.5819	628249.5943	502478.4487	
1327925.849	1103676.868	514813.864		734593.0122
58455333.21	41247741.95	36467483.3	47633399.44	58548082.65
5681725.717	5449360.856	6658740.145	1786648.103	1453614.123
3826917.429	6371862.571	5685568.822	2324921.347	6993758.258
1266614554	1291447763	1057821293	1320791891	1203066825
4772709.79	3081699.289	1763528.112	1795129.229	2433390.712
468613.5235	494311.2023	376386.3142		65887.06679
163772.014	558252.9411	3082720.903		816258.9981
158848627.1	107268888.2	96496159.63	108344158.6	73138662.44
	314483.8143	341005.6014		

944215109.8	1001606938	906552478.1	1032791598	1265108773
1696184.079	1688469.144		1375817.622	1564764.276
6317215.43	3294567.761	4510640.097	3131905.532	5936905.704
3091580893	2523762304	5019787702	2834118370	3796984186
80595.33014	55417.51133	126353.6024	48748.44812	
2709260.862	4209311.19	1211565.393	2517305.657	4400809.862
4656378.984	4266073.845	4805497.727	2526273.373	7229205.251
3165747.001	2234995.616	3336512.847	1753846.914	5617698.703
2042944.055	3484526.457	3212219.981		641543.7645
306016223.6	277077069.6	160831323.3	343291829.5	249390614.3
34909146.26	66848437.28	196603964.4	243093086.1	26326790.69
1263431.346	2140294.163	173382.8778	1832154.561	2927387.381
	411942.57	325638.8116	389470.8303	
31248211.08	45834681.92	67074862.23	78014839.21	47706429.28
844697.6868	2313997.477	740340.3866	434441.0229	4069602.514
184532415.6	174688933.4	130514925.4	151124376.7	80709874.69
328792.9173		993056.2653		2623648.599
22245355.53	13213251.71	8897068.22	2940781.394	40851745.16
4936584.448	8690904.33	3778754.373	6284905.53	9523121.143
13440988.64	13320789.9	8711730.672	7000023.633	6603979.559
5825767.069	1752432.72	3461556.934	1991594.837	1119855.7
610787.1035	1380905.045	1679211.991	1084744.549	
603170.075	1942285.941	909132.1277	860279.7915	2131090.813
6536505.699	4979958.053	2566900.704		5118335.551
222028.2648	334262.4391	278214.43	136793.7316	
267013.8702				
		266841.1455		
77490228.26	65698096.48	98024058.2	111153059.5	94245554.64
		342741.8359		278470.7329
15747771.01	9684332.324	18175782.85	7076090.249	9792656.705
57131637.92	46109190.12	58679564.8	53056321.28	76071616.34
110611214.9	70729754.69	43254154.08	50581661.55	53311690.99
27159152.08	22631579.86	21449888.57	30003803.26	50397252.36
40338884.94	141015.2873	209157.5627		
		79654.66718	8621397.164	119614.5797
		2405707.995	1111788.923	1819208.207
		342018.506	278204.2345	
818517.4028	1169370.824	4886653.86		2221654.627
12471020.4	12237885.41	10930730.18	15061899	16458350.67
267993.2734	344979.7675			
307926854.6	494801701.1	350219684.6	294161191.9	606654210.9
20124395.13	13244864.83	85141838.78	33652001.82	39603760.08
207388.4431	204438.2697	88751.32039	150498.2514	89151.26048
2343922.728	2308784.233	1710563.696	1706177.292	2515297.394
154294.8671	130981.8031			361534.3743
24399926.91	29025046.27	23244450.09	16892955.53	16353019.26
20449067.08	7038095.329	8699257.393	9355129.009	9060717.02
10105639.43	3983640.393	5647972.186	4777707.686	4614890.798
1067276.032	1673533.722	1114095.888	1302382.183	9694.530165

416443.1922	675582.1103	541140.3742		535170.2945
1587855676	1603107698	1719687179	2169485756	2099631115
45631928.01	41961496.54	25305359.84	32645625.09	46116928.26
626605.5688	6426715.669	5957950.832	4811589.26	6165919.797
10559828.41	11607911.27	10632200.18	8585684.709	7666870.599
7679426.404	6526735.752	4921900.113	1916515.278	6026444.39
178318.6287	251690.9515	379833.6961	223819.549	
			117157.5918	96721.07801
4217463.773	5643964.928	2335955.337	3434379.749	4601095.499
1387050.411	1788512.419	2291964.672	3322461.742	1929318.682
		1068108.838	324653.6738	
655301590.3	899766116.7	438648169.8	497010375.5	701703116.1
11296309.17	12769148.96	9475931.168	7389871.772	4233167.545
22453762.27	8212439.998	21459355.53	6008765.972	8504150.837
		1235040.115	793492.039	125979.7724
1087044.67	463049.2197	552732.5649	650893.1466	
3477599.353	1139307.324	3636161.519	2104366.178	2622752.935
1881637.032	1220717.839	2241556.974	1199439.993	4725359.929
24395069.65	17401391.66	18786373.24	11186963.86	9607541.865
12462049.26	10675623.85	14595927.42	3086350.297	4191221.955
16987708.6	13288993.44	5020772.636	15371682.43	10222730.58
				374974.581
14643741.85	18345905.45	12991385.6	12893448.22	9180200.718
22754029.48	19309674.78	13834844.94	13922725	13313274.89
4326836.107	2653541.888	2880902.338	972728.847	2407848.562
99197.02471	80727.3885	101208.4335	34778.8266	73181.86825
4986889.679	929503.0226	1366987.911	3203939.644	880077.7809
829083443.1	806826474	542016087.5	453438240.7	359578965.7
	347133.6398			827742.0994
116529480.1	114333083.9	119997862.7	104523049.4	72631634.11
225928454.6	417969105.8	390018635.2	309541738.2	247427517.2
13504246.29	18221847.05	9740376.612	6647194.381	13423645.41
1239483.1	1056660.691	1803655.523	943135.7417	881213.6744
1911573.73	1968006.09	197571.9851	549971.4169	3668992.767
42241122.01	41969020.23	40676401	38099351.65	37118727.46
1532943.384	2342851.638	2197048.946	1615593.191	2511001.57
2744195.841	3008767.946	6888422.276	3241295.656	4278765.294
		943009.737		1131567.061
620262.1301	237022.3895	4037228.505	1601879.822	
70170669.51	71339856.45	60198363.52	50215656.5	67289824
18728739.04	29716278.83	16520715.04	23570671.6	3359869.43
692829470.6	473487865.6	397882079.4	455178099.9	518366747.8
2423835.432	2898469.053	2453248.859	1675654.993	7482090.634
1753485.299	1841902.369	6979381.013	4658811.185	1503078.739
1838948.813	3617377.322	1456527.639	2776019.105	3371455.57
		338771.269	475596.2891	
11806123.39	9182538.288	55770593.75	25044929.18	12654774.8

2502545.591	3170475.982	5045764	1234285.379	1472567.063
408782.0615		451208.8546		
718902.9979	232519.4227	837240.2617	124893.627	206039.8181
10527107.21	8806311.82	6365724.816	6602314.611	5000536.044
430140.3107	949274.9528	1338269.294		534011.7228
9803302.019	9245238.966	10040831.72	9228403.461	18112267.14
	1608.373619	4031.228702	5513.231654	
686138.2431	957270.4529	668276.338		
2411068.15	1187901.642	3169918.938	1236664.272	3322591.858
696862.0415	84262.05493		35300.39072	1156004.517
5250851.847	5099822.281	4465936.517	262136.3978	4019567.952
1912648.205	2916787.169	2089235.204	2282181.197	2616349.907
2602219.539	2085521.453	2703159.498	2160527.755	2738725.81
537682.2394	827831.3085	756389.677		734064.834
229420156.4	317095241.1	187393454.2	199067638.4	269228830.8
1327635.461	1865545.305	1515316.775	1954813.302	1487824.986
27906740.61	21927693.5	17540241.7	9473540.082	17679045.93
1092187.627	711876.4468	981734.225	1100334.793	1860264.83
10011354.85	7166318.723	83336548.76	2687288.482	15406985.31
6898030.356	5115310.481	3196823.511	6057563.162	10055748.48
1645978.72	635150.8375	860761.7641	771051.4408	693557.9162
250376.6168	1180907.791	1857619.059	344969.4485	9299542.664
1681692.306	573303.588	583433.3661		586535.0109
1723498.949	1321996.559	471837.0297	535466.0824	1105138.076
2039692.383	2277278.502	3856002.717	3793178.78	1157368.864
	268738.6802	215759.3812	69144.59814	48193.15004
30551375.06	34362353.54	188796312.2	20031910.65	6084266.842
364166.5598		207583.2715	239220.1267	503730.3194
49476051.55	98270180.86	105523552.7	104787239.8	142633767.3
285417.2243	249185.7218	303876.7776	241694.6007	363088.5584
			3264958.533	
936040.9766	256884.4836			141500.8386
87996.63499	37177.21055	74987.10345		
5032835.278	1593100.574	9531714.847	8103339.582	2915165.175
194297.8875	377291.5362		91369.10277	206469.672
19362587.74	12993827.98	11611810.29	12517732.68	28618011.75
3100143.678	3120889.313	1897178.964	2556714.811	2274188.727
7293386.557	3287471.969	4888109.146	5536621.714	3745366.546
2570286.61	1507906.295	2478973.6	1308921.438	2914157.496
1355334.607	728908.8915		245526.3613	
173893.4857				384244.8996
1952981.211	1655454.909	2312915.264	1677362.294	3780133.804
	554885.2663			
1255885.277	435398.5542			897834.5382
20027378.7	17207666.67	27962961.86	19986897.94	31734099.44
24983145.66	28973311.06	20761058.03	19312747.18	37042259.54
9322517747	9819454794	9281738959	10806249987	10608487182
1619594.85	1089482.91	2057249.454	2222658.812	2898615.994
19522057.07	27698819.64	13320117.28	14655935.5	198685358.8
2816450282	2731144994	3404020458	4247382408	3676812746

3167834.396	3787187.351	3056704.989	3630560.073	5184070.179
1478361.364	1280121.474	747133.5245	48947.41698	892134.1742
			1038241.631	2084228.213
2293705.256	5989919.336	1052757.543	2664001.819	2132212.254
13576082.95	13443353.84	7900127.519	1540732.142	12968500.53
		1255350.474		2636218.698
57722694.34	44256729.72	49401830.93	8039870.714	25567924.96
765742.5273		776275.705		485290.1158
11272262.08	5606200.081	6325870.495	5003775.996	12697502.21
74874824.12	92504154.16	67766080.33	44597044.51	85717751.94
2741025.506	3129507.636	6766321.284	4674751.092	14697794.03
27645290.76	28203468.38	35555058.33	19264169.18	14091991.57
6705157.459	11859811.4	8086063.865	8052519.071	15859843.77
	209199.8443		162481.4794	1409288.168
5283471.943	4416372.5	1583082.984	2339597.982	1790538.392
2463332830	3282895718	2414019250	3095564656	1849773699
226959947.9	98889794.92	129722590.5	160068485	154695793.3
		115091.8157	265287.3756	
3478570.097	2924786.123	4001076.66	3807265.794	2404554.066
1493747.639	2100240.384	392280.4582	2254274.297	4022345.469
647731.06	633579.0541	819604.3962	407928.8642	11620732.45
21288371.23	22770312.93	16301075.29	7453615.64	10132051.57
1388773.045	1038419.066	1249289.859	1464100.945	803178.5625
1079847.495	1158282.674	1304282.216	966693.3161	835370.9636
2255866.379	2151316.005	324604.6937	810961.5019	1343470.39
54182992.54	37121092.61	32703727.87	37049325.72	60798778.25
2399837.259	998768.4807	2970488.438	1909165.585	1102996.352
19890299.26	15224914.39	15280808.31	21277764.37	17234888.14
528553.6945	454700.91	331893.2938		
18352.7068	7730.318426	166294.9577		34808.55678
2652994.454	3775052.852	1630195.012	1252354.5	5312314.612
978866.6219	351538.3491	943663.4047	563964.2229	31315.56661
575873.9178	569353.2399	5166477.405		1545511.514
	2036467.16	840107.959	1396433.432	10673859.66
12249590.61	10444768.32	8247027.487	3678693.447	6317320.536
689031.8759		662391.6833	621747.1698	653391.158
12609489.25	19264679.53	13602682.53	10768190.09	18277094.68
186682.4482	224945.1476	247980.2741	282798.5894	291240.196
75696479.55	148345777	76460755.9	83175341.05	117814869.2
258575.0863	248737.0456		335126.9274	404839.9725
		433348.1247	595757.7177	
262067.1864	268954.1675	806227.6501	206921.5169	190065.2867
5833244.579	9835493.973	4968789.96	34798980.99	13319041.34
	1554150.793	2492822.391	1536884.339	686360.8451
840502.5777			102645.5659	

1968379.305	1781144.17	1546534.491	1342547.311	1424493.825
10540767.41	6908097.283	5478392.82	4995177.172	3146584.772
10150971.82	32161440.08	15830873.4	20610656.17	93167167.98
12333817.68	9198369.396	7650772.628	7553920.508	11341313.31
		794787.7225	779489.1606	858503.0764
57343544.65	34189792.93	33889478.78	12479552.47	17059596.96
68093743.35	48165967.88	47963041.71	38600609.39	25899724.61
	75280.09243		237799.3948	102814
1076857.996	2386536.41	1335010.365		3110923.916
352441.7753		266373.5417		
68498564.46	61346941.2	61846349.95	59623934	32337965.18
562274.7128	749228.9951			
1402567.396	1062388.582	415618.796	3125353.81	1567140.981
1449646.592	1267511.502	1020088.566	609683.0811	557573.6895
248735.2938	444209.694	111542.5364		
19851898.38	26986140.57	20866053.08	17696551.88	25889313.66
1003166325	799177442.4	327967560.9	454982955.5	213459484.3
6335065.283	5689152.254	6799211.531	6325136.631	22306089.79
4359257.147	5566985.953	4362692.195	3580709.749	8183524.147
100051022	112929730.8	79194201.17	79286269.93	77606793.61
		96042.54822	96166.5955	
670148.5415	549941.1857	14890440.39	12775849.19	376380.0665
353049217.4	289212998.8	217596108.2	218206636.6	133519183
50145135.53	57279128.49	49775559.03	24731778.67	53828948.33
1167332.538	954934.2517	480331.4032		401549.6703
2579622.26	1956781.464	1316626.298	727518.3565	463869.1279
2249715.294	1969734.053	1918692.292	2335458.732	1679656.535
7676445.856	12964876.66	10839971.82	8301453.414	12932511.59
3210770.616	2211400.357	2565817.59	460194.7253	1154770.832
13650411046	11787304608	12057194122	15500645031	10549374788
5685183.878	8045808.432	14657160.72	3855014.375	9317221.617
7472277.887	9010317.973	4800983.744	6215512.676	1179549.829
5811829.576	9923377.334	5431654.294	1676040.983	7123942.56
2016469.378	295272.0651	794368.312		261564.2049
5036174.254	2928283.296	2301727.149	1918883.27	2216434.321
13190855.2	15898557.99	6144927.601	5414586.843	623801.0068
144630.4469	110235.1259	179678.5087	104110.338	90349.70366
22533039.53	33913006.47	54178163.46	15916267.03	21651048.86
	616878.5561	412443.7929		
2624412.598	2893445.382	2975171.169	2155008.979	2246585.183
807779.9833	558647.317	1430782.962	978825.4461	1191075.063
106463015	97021131.02	76281406.15	77614139.03	159596638.6
	365628.683	284809.1881		397489.7909
4353136.684	4730767.102	4109893.949	2136202.973	3464395.625
107118743.6	124057836.6	96140181.2	91840843.2	57778768.14

26872025.22	8593477.015	12985596.52	22005562.69	17891938.26
946110.964	609232.6069	732477.2055	1455551.179	375602.7078
928591872.9	1745812623	1332946078	1785418490	1445370866
11798103.92	8703415.11	7704289.915	5859206.362	15152021.22
2291188.911	45998.59696			
321521132.1	145285052.1	347515000.7	109719087.8	381311348.5
122497.6619	226197.2931			
		478056.2096		
				1572509.985
3902.43419			4953.568191	
2305922.945	1482516.207	710088.4198	102645.5659	
42689966.77	29797402.93	7164970.773	11389830.21	12981941.47
23467134.38	30764927.75	15379048.77	26862092.15	40219168.25
1112698047	683895531.7	903838673.4	1414026113	732926298.4
502339.3092		626736.8026		
4309248.709	1613233.372	1120300.213	1204138.382	1739403.257
17727942.59	19787047.58	17115422.25	12792338.42	19600555.19
	109263.9919			106202.1418
1595523.252	1221530.678	1168163.685	1012473.348	1188337.835
49555321.46	41546463	43124659.26	35454846.5	21532296.13
126713938.7	128519878.4	141444579.8	66982300.82	141855540
7136325.041	5401960.487	4762731.413	1217069.804	10123525.71
3105421.354	1839393.403	12280418.63	9114068.721	2888827
659463.3361	2707775.481	636572.6638	329943.9553	
2356819.832	1266054.217	1695548.939	1840100.777	3649280.577
578440.3532		754570.6339	343150.7293	174897.4464
		13900.28755		
186072043.8	376999916.4	186567993.2	142225661.3	105324881
1431856.445	1316411.538	1510504.352	1314670.329	1002581.632
1454288.78	2871042.531	3301880.871	1857141.918	2698985.658
6658188.724	5291572.812	7465583.621	5163574.124	7424916.806
	516641.9894	1484636.932	451064.6887	633800.3218
468779.214	1029995.151	1030454.281	588289.375	891918.4231
10036594.73	8417129.267	10477890.15	14264875.55	15941488.95
3205453.698	1875580.374	550356.3524	667222.002	2649712.225
3692651.379	5698964.26	1052976.545	2116969.067	3875888.535
		1636948.263		
13223011.19	12692800.59	12485185.9	9867821.894	5068729.315
2613472.159		2818761.001	3145471.002	2361060.522
3807216.799	4443597.052	2770603.226	3363844.376	6863930.866
980207.1453	238689.6942	2244140.128	305596.9768	807224.8907
17250546.54	14008643.02	15831570.53	8829130.511	12144596.38
2717177.201	1935050.139	5804799.849	3730340.65	2308518.425
81629959.62	7998061.012	9922502.354	11206795.54	8715415.709
3542013.897	2779218.457	4060028.315	4905725.912	3120770.897
5961416.923	6953682.521	5223259.884	3993361.23	13655481.82
1545365.826			1508253.18	861035.8496
845990.569	419327.8571	527645.1816	558762.6205	444643.2264
3512440.931	1287772.294	729721.645	800639.9325	2132872.973

994851.4102	450552.2404	290288.1377	3125330.742	
116960857.7	109370497.9	74250748.81	29124886.46	77781844.22
916326.2547	567902.9279	441639.9173		377898.3487
			5335708.011	
865522.493	1644881.956	1107237.337	539822.1255	510419.4783
1275388834	854143086.6	860931963.3	1130737566	1240335663
4548419.537	4153191.496	4594803.54	4324359.317	1927867.234
1233360.578		881494.5283		2064774.73
10441352.31	5452798.894	6166092.934	5891642.551	3766270.641
492951.51	1541961.833	7924480.199		2174333.172
40181727.94	53148732.27	55700942.83	29522437.44	89814911.01
6693871.565	4092736.145	5354857.149	3636391.128	6676447.394
836096.7474	327449.6596	567912.3004	655323.8608	593616.7884
11452386.88	5752724.571	5400444.612	3786824.807	2149715.419
	40651.13019	65557.09506		
80977435.15	48761321.04	49262804.76	39249975.48	66955919.96
		386318.5218		
11357422.82	8311047.637	11469491.78	6698470.227	7044159.306
2563288.083	884094.1831	786230.8591		92676.1497
			327701.3394	385432.0885
284100041.8	364922639.9	199942094.2	187463821.2	187799047.1
3297883.9	2491756.552	2472271.082	748120.2196	2076006.784
171772.7997	83682.62261			
114316526.7	155464927.9	121490126.2	143475256.7	211504832.7
511658759.1	281973173.5	236307828	226398191.5	216671031.3
11367902.28	16276945.95	12885958.33	19962046.32	18722595.38
19120344.35	18731936.87	10883371.99	5250755.807	21840760.05
120305096	98744435.23	65502758.47	42258721.84	51007285.57
4933040.977	3662788.969	5086919.459	1544719.02	11373122.04
185952.3106	678929.0506	285824.6485	471898.7093	2778091.332
528246.6044	753650.3699			
32418106.32	18946437.06	25401695.16	28021420.83	16370759.7
6267037.394	7044313.375	6081360.485	5857984.929	2622586.037
7109325.482	7654638.862	4971595.998	9385733.097	24140122.38
4367489.085	5390349.596	5307823.073	7236854.83	5128913.37
3305838.342	2588296.207	1960393.603	2393613.688	5309638.328
405161.3233	340727.9169		171394.7664	345190.0114
213103.6467	184113.6145	162331.7388	109174.8277	110565.6122
1481393.658	247395.2676	1690271.292		1246398.752
13607976.07	6984041.575	2424385.447	61757.28783	14543032.67
807431264.6	414535014.2	490970410.9	451169097	374037960.9
317200.6342	30846.88766	352067.5228	78061.85159	155191.7409
	46840.89642			54936.72545
80847876.8	68844899.13	38861073.98	49014920.04	49309354.42
67533339.1	55149880.69	57514604.51	41553240.18	62044773.57
326576.6081	545925.8758	1499852.576	1233228.908	1053169.063
5669955.237	3644226.874	5207474.402	2180183.362	4881514.417

446886.111	333745.3652	525863.773	357227.3896	347670.4225
5284897.712	4682136.181	4264114.614	4856843.204	11835387.14
103571076.1	85096646.92	63469519.05	61760102.14	108404464.2
123975442.2	78414506	52292864.98	59124988.96	88543356.17
10636638.23	14412000.49	12669915	14955373.42	15838612.89
5202502.488	9994626.505	7308591.14	6123736.003	13498249.64
16432266.3	16907951.62	16663268.09	22540683.49	25480587.19
	397706.2844	472421.4825	2994899.092	776136.7962
	1206090.578			
				76284.52153
11720610.18	7923345.8	10683903.36	7765401.267	10739098.55
40999241.62	49068156.05	27337162.5	32222059.12	24858237.19
551335.3124	497773.9998	684683.7631	767643.4591	222703.8079
		401827.671	466676.5748	373999.7009
59458.27323	59864.37588		277675.312	445204.2521
77321636.34	85902477.45	59380790.2	45166687.09	60930848.15
689203.1121	1070634.083	281158.9617	359356.8498	609064.3666
				968218.7987
	599290.9601			175455.8298
9384585.338	7220374.621	3805047.106	464495.7314	2865172.817
26275943.65	18029648.98	13668922.98	16531971.56	23210776.66
38719516.33	28689785.14	24725277.03	24331655.3	46026989.64
				14523.38822
4368498.974		1329574.576		1768098.199
967403.9294	4264274.358	872400.3989	4795425.718	2458186.8
665186.388	1990547.046	1003620.025	1305400.124	358387.2193
10618098.99	9787756.929	12351711.78	14733767.31	27666699.21
2894656.606	2177231.118	3762251.847	2191887.504	2067390.289
53807215.08	42984517.65	35336327.84	22433543.06	12207081.56
2680657.407	3510294.716	1344439.562	823160.828	1345529.019
407980251.5	312305046.4	405702544.5	539626592.5	956937496.6
67846963.71	53154427.88	51708095.29	49390418.67	23242944.96
1626684.354	575139.3455			
269313.7057	213978.7928	386312.931		143555.626
542673.4511	305363.0108			
830919.446	909875.8929	459071.2689		461497.4166
997313.5147		354599.353	1221526.016	
		1864777.39	3788282.906	
995067.9202	432428.8684		230903.3809	245861.8835
6610554.957	6888880.11	5940609.046	5252967.466	3576721.036
1260889.526	1885358.607	1229976.136	1270218.934	1321285.255
92173999.31	45059092.39	33306458.31	78376894.96	80828616.54
69815940.71	83959738.95	98083916.79	12748191.88	33272040.1
279818.2861	1363447.373	2138480.473	1964372.136	1780026.826
513129.7211	128392.1832	541687.9592		465714.5034
62847815.26	2051753.887	8582612.731	1665644.434	3512890.932
501984.1561	169987.8597	478549.8668	419731.4588	271340.4478
8137016.693	7367603.243	4608143.53	5456458.779	5377468.313
667806.6266	330186.3823	345404.7478	312863.5816	232944.1825

1977209.062	887397.8081	1003121.639	461473.0002	366070.7001
199424.564	269408.4358	282319.5609	349066.7302	566746.6533
14441120.71	15464209.6	3658792.604	7664241.041	16737427.25
11950703.93	12527194.63	5685215.575	6282884.603	
8922915.346	3839186.89	5334470.786	5756286.077	2697456.055
1012175.003	1274829.888	884762.3636	346471.401	909340.1236
285055.7148	538323.4234	282944.4277	239848.3994	144384.6317
2500834.007	2453751.374	4104874.063	1774612.329	3118161.714
21399506.91	60642160.93	61896596.7	25759664.5	89131764.83
				10568.2489
		418448.9227		
3106203.17	785095.6024	2588911.655	2698210.357	1811687.388
	259474.4399		167470.5936	
381450.684		490487.3811	538013.6163	
5911618.168	7630350.695	10465993.13	9781828.221	3188115.163
609158.0891	1082268.21	938835.3114	197284.3049	521066.7014
167447.5444	223372.5849			
9829357.46	9346194.116	11056545.42	14482011.69	25846025.45
7334133.223	11824273.39	5033453.623	876021.3108	7906411.652
177982.2047	83769.22664	1011679.972	2698483.015	230605.2216
			2390048.563	
165542.0225	171809.4488	545610.8121	104712.5229	484196.427
16907610.54	19512163.84	5761038.696	12681070.68	39053507.58
2739686818	1885348661	2231656126	3602587506	3229806035
11456592.2	6867322.207	10073433.38	5337301.523	17383260.72
1978003.916	2608841.304	1777663.698	580065.6753	1431185.117
184242.6887	148218.945	129655.6378	67024.77444	96041.32245
6188679.194	7309690.189	6830075.106	2636476.648	6338606.667
		2190056.342		
		239380.8403		
482051.5783				376530.4378
326677.9229		186252.5726	147206.6831	
6409169.414	3898607.704	32697318.44	4463965.414	5836667.773
2546606.878	2724988.147	3692578.348	773542.0975	2403085.382
622187.285	475857.0879	851431.2132		430650.1391
19669156.83	8969814.632	13853286.41	16335578.79	6774794.072
	179985.0994			627929.2235
224706.4091	225456.7312	226197.4102		232091.6744
478931.2521	463362.7298	147622.083	1055610.828	343641.4755
	200369.5097	151877.6081		195964.9659
828489.9023	785454.2033	1096418.667	2382600.365	812994.0769
7883980.012	1337067.307	5604739.866	7022513.501	8214378.018
462415.9071				

2975856.261	4819464.669	7684356.557	6993370.271	8850495.411
55706951.39	42980611.32	43616436.89	55899297.76	32107022.05
1501426.44	7028409.838	3090688.09	1608075.138	3569961.891
342996537.7	305685165	169281592.9	223641397.4	63341108.64
6002012.41	5146535.821	3598100.115	2738852.496	6944152.871
49858658.35	32855077.99	37243104.01	34607342.58	18802112.39
13227258.2	15274139.01	6132261.353	6900858.052	11186261.44
47191620.18	23831251.33	80944690.96	48090793.77	31453610.36
4715429.537	3013000.74	1234513.233	1218339.626	3005223.493
1452134.447				
24195391.15	20803978.77	23555226.51	29703854.11	25699919.02
1433778.486	1350997.924	479483.4131	429479.2555	
17647833.87	7966240.05	30777222.37	17420408.61	18102111.22
346848.4478	379047.7384			
132046061.4	80942131.28	52161729.67	41253562.03	43167345.7
	435686.1717	1438148.301	1820397.785	1250214.397
509780.9502				1080444.992
19838313.39	18024341.06	34579766.36	34934986.6	18127063.08
		132190.2235		
529749.3969	533663.8432	381393.6746	225669.4835	320457.5098
	3773.38595			11498.89095
2836304.975	5384363.734	3741302.621	3169029.316	6423026.05
7348719.614	2869431.532	3688171.678	3850706.748	3416250.236
8063640.348	3301172.195	4361427.765	33039950.38	7289028.007
68799460.79	62359714.09	65342406.39	45496301	61017143.89
5552972.647	4567008.149	7613131.497	1787027.613	12226904.83
78219709.69	48752104.22	92275268.19	125565314.4	67720532.04
9502634.607	10789355.73	8452873.869	9758849.35	11570242.55
	6195.087661			
1919647.883	677639.3058	386325.2948	647240.8397	306647.9079
3243702.034			241718.4962	1705526.251
1904207.426	2606766.562	2923086.308	3328137.133	3085795.911
997892.0856				
9270189.906	4391436.313	6231058.184	5047963.996	5967634.052
	11292.48363	1533.29374	2388.309165	916.152422
7983353.486	3274732.109	5859175.174	5286251.108	2414502.788
6486988.431	7473052.805	10059317.28	13418536.14	3153003.927
30639118.44	40551430.04	24658861.59	13777384.04	10256083.57
975006.0395	862017.833	824736.2172		
259643.3831	372499.9424	210676.2167	82332.46448	254428.8775
1722167811	1195553430	3213791337	1328684284	1402915094
22327272.15	13391603.23	28486416	9860609.692	19759543.21
2919211.946	1547525.779	2187804.345	1125961.763	1597060.795
3033362.133		7904527.177		
998085.7641		916777.984		842359.2651
	223438.0781			115927.4771

296745.1818	341586.0229		176700.3678	
697649.0147	568760.9055	2277744.137	1855149.562	1281191.731
20662274.99	22857692.39	21733786	12986013.69	28601704.45
1025126.818	2630270.931	1894703.01	3239714.506	7591116.05
	529990.0347	550276.8263	176528.1339	
2515661.248	2909122.093	2934023.569	2525592.643	5604909.216
		232122.7823		
950442.3973	1123912.236	560999.2465		1701736.9
3797642.796	950694.9756	2866641.469	1231047.571	3391239.079
		265294.2017	259063.1991	182955.1827
294849.7135	337418.2616	2343748.009	413102.9613	3073972.166
425589.9998			408516.1607	
1920721.863	1767635.641	984697.1906	620310.9067	2553125.021
17647019.52	14330667.56	14376741.76	9910792.528	14900000.4
11104284.5	6914904.072	7242003.038	4852978.374	11738976.34
447460.0443	225461.9912	248640.1237	323373.8846	
72388798.02	52343620.11	60329110.48	79320961.83	117272460.8
846485.7109	1232767.949	2751417.341	1066869.805	904175.0226
4989489.681	4955473.092	6973595.301	3835975.789	1174000.249
8240715.86	11039100.13	4876677.461	2278370.532	6044650.382
62056750.47	411361880.8	70674834.31	74555631.9	96092232.73
1193272.75	832156.9052	1031093.237	964665.5304	
434775.818	530010.57			101895.9303
811297.5905	673334.2532	694952.293		790608.1238
378986.7958	288584.1796	272868.479	91855.89565	158143.3502
2450418.689				
	1625998.771		1705030.732	2425667.305
3463956.123	4885391.004	8057947.831	4220145.755	2509288.627
467735.2243	998469.1588	241075.7263	343875.002	
2824589.823	2325022.825			2471978.341
1878029.947	1738464.145	997716.1695	1264165.23	
	746141.2471	53576.41127		
6352108.395	4829940.533	3010931.158	2046724.314	4232005.083
	398650.3568			125125.119
680259.5542	810125.9735	349893.0286	1537455.471	1138757.344
17122673.58	9076876.056	24488960.49	11718469.92	25570311.72
				680966.7266
31026.27738	110010.976	48698.03208		205615.0035
765659.6334	530304.7403	597456.2723	590055.9483	559137.7642
147279.0789	303152.1817	246766.977		
2378476.642	1776272.525	1236543.45	3893014.385	2563456.642
1182163.411	913152.1109		542758.7574	392127.9138
90456.73689		145135.8423		166741.5917
4417986.345	6054753.732	1363238.127	7692500.526	2370494.508
3346458.526	862256.9947	1300180.184	1292866.403	1862395.215

1820680.308	1787853.29	427924.2849	482848.745	1152281.039
951530.1041	755893.5737	925111.6601	545739.086	804846.6341
771531.4793		850867.4625		
1271768.345				
22502198.58	27273106.79	33312801.89	25155092.82	21840549.58
529737.8514				
500389119.5	374412994.5	317321573.9	394099556.9	313622410.5
548581.0036			212590.463	
196457.2477	106820.2263			224632.8657
1188326.229	1349020.24	1565917.917	1341175.303	1949040.132
3654107.418	3130680.491	1750241.953	1972161.439	173650.6713
1920864.69	858996.1494	5528758.827	3284133.501	672434.5195
28064.3305	22009.30063	75285.24819		21487.66162
1352132.23	1644785.292			3065603.566
11370914.93	12950504.22	9404454.032	15768252.87	7514437.892
2284348.088	1697952.87	2027193.647	1323717.709	2707376.827
283267.0746	316622.066			223270.4335
699112.6304	1005479.941	1134297.688	1451177.975	2308789.784
1025877.501	1531933.618	493135.3735	808045.1644	1048703.195
298334072	24065826.26	16601437.75	170957405.3	26770086.19
372245.9585		381353.3245		237901.8291
138363.0551	983995.374	774158.9404	658594.3863	1806204.569
2098784.189	2204503.914	4110365.6	3330463.322	2118154.138
17704969.88	15675216.12	15797589.86	16772700.12	20549138.67
19949755.6	18945898.03	46479001.65	23192712.56	23720309.22
1399455.308	1003791.995	821390.0699		1566954.174
41881706.06	28505189.29	23998849.87	19293224.33	18946615.51
132089520.1	179595342.4	163318550.6	209001948	154014943.2
	174311.5614			346527.9595
5171578.036	1651870.14	791736.4852	1567155.533	1142196.175
1137348.669	716206.1878	397813.6232	436608.5539	757195.0074
333163.1058	291948.6842	136833.475		119609.4041
770235.273	966526.5464	988320.9642	331987.9413	773856.1851
1472252.887	636017.1612	667309.2784	461083.0305	3787903.561
3093678.765	2033983.342	5589200.837	1549157.412	1181359.966
1317089387	1165731405	1270540551	1320842613	2600477192
12590056.87	10254454.7	19503333.92	5579653.892	9253682.681
26395010.55	22493067.01	21518591.68	17491345.78	18998472.82
		831666.7146		
				477581.6417
1042871.237	1329057.958	736320.3965		
1670769.85	1251615.824	1520686.399	921676.5916	1645330.113
	850278.152	271438.1446		295083.0394
9912191.963	6293253.422	8790675.539	9293267	9745953.265
1804973.47	1224905.773	1881181.461	2053072.881	138476.4193

2525300.421	4303918.389	3242133.716	3566569.485	4719749.913
	292089.5884			
2695948.088	3889118.417	1637085.255	2042672.089	2354596.52
6139767.479	4449838.351	6588281.249	4860701.484	4303448.16
395415.9389	210346.3293			131114.5532
1067618.829	527406.6755	363187.046		
	238774.9965			
617686.4965				
53597.35256	47971.93354	106325.7263		
36680151.25	42109276.4	37200406.47	26132843.34	27366830.52
350211.164				1964877.771
21878215.96	18492311.69	18170015.37	18473471.68	29889676.9
24149.49294	13828.06677		120581.6241	41225.37826
1390549.078	1861476.297	1237157.104	3180551.539	2948169.77
244479.88	327904.9417	432305.4767	438388.5981	756875.0411
2682381.462	1773571.088	908868.862	754867.8819	350944.8949
962739.2665	797168.0271	658452.5212	1442479.017	1331205.409
212275.9077			303562.1035	53048.19368
9594546.508	9218973.823	6251822.776	3301627.229	4606090.745
262820.2205	476317.5238	373711.0624	148910.6563	368581.2922
7306329.616	5057949.214	7247136.504	4182372.057	9241644.517
	262922.2898	526230.853	1370479.127	734143.668
	303408.3809			252840.7792
6235563.705	25901348.76	13438581.38	21341317.39	126589908.5
		2290760.368	2651569.173	2470370.438
398547.1619		206084.489		
7555205.678	10002250.42	10353093.99	7664235.688	5873629.621
	525187.6287			539990.5513
789718.6811	475708.2051	417623.0983	227092.1425	701183.7608
103308588.4	115329192.3	110678016.2	77610751.76	40773363.78
5523876.758	3511429.494	3198979.22	1803051.057	2223912.984
		839852.1291	165411.411	1981503.652
2388209.022	3619667.246	1829263.399	1629402.475	3820794.657
658826.6486	843738.5383	1041451.604	344951.2881	857387.5701
11885111.68	14194337.22	16772873.38	11114131.71	6826579.443
	407314.1956		405222.1095	
39169.86939	19599.68297	263050.0613	91366.94566	8867.452464
909374.7125	2077882.216	426054.3969	186439.1618	359317.6401
272215358	280402628.1	277858085.6	278368842.4	231293431.2
732871.3337	1412304.656		825184.4579	1136471.642
406257.3325	578144.8064	576274.5059	189923.1661	786755.7292
2801262.316	3205082.084	4501949.852	2443107.271	4670275.65
64402.42335	25275.41266	56771.31778	56361.92884	
471838.6021	350893.0694	175699.7153		
33870035.44	22928057.02	25455797.09	42033676.92	23828297.44
48684824.84	41529279.08	58044822.02	77205939.44	63622427.07

10396474.59	10354545.24	11110267	968018.2284	4781068.552
	709253.6376		184646.7781	
122199238.3	79975139.9	125221496.8	20348092.37	101505324.4
3322321.84	3065558.193	1288507.932	1576925.456	2608003.876
249217.4319	612450.4513	294995.9956		
	444137.4144	487860.9535		535257.3586
1737515.218	2383360.965	2109507.92	2078206.129	3020029.599
6941135.185	7224708.365	11609301.43	14806801.12	9658019.008
1265042.268	896815.0887	1925136.952	1791826.118	753004.2933
573647.9766	531944.0536	1842987.274		2324757.6
8899959.719	7719702.384	10020180.5	3641424.362	4782498.219
62332584.64	42777869.71	42429900.37	46334076.13	30722702.22
17078610.25	25769143.44	32530302.6	13896673	18090654.01
807284.436	663374.7543	857535.2732	655604.0073	704247.7804
50799.53747	56752.16532	104193.507	45731.39581	54645.67349
796210404.7	763207082.5	686837472.3	414324000.9	1219661002
1997442305	1588630175	1192711549	1201120686	1806380392
232388.4295	287093.0849	124030.6788	120685.4822	160464.3721
31872475.86	46318674.65	68573492.86	79569528.04	48402101.09
372030.0646	1484090.767	551718.8296		277025.5637
1523447.103	1599372.229	1291728.908	1321415.574	1016103.097
97295750.44	58488123.05	73525360.7	66541449.55	57103832.9
2660822.722	1984656.139	2933853.032	3591748.006	7316831.738
336258.5249	334958.0341			
3425243.049	3117286.62	1285016.071	2667156.001	5350415.492
29827.17584	15095.96792	111991.5625	16385.39582	9015.999566
12789188.52	9953265.979	15793783.02	15515628.15	9120121.946
708524318.3	885566175.8	629331595.2	587926233.6	774388090.9
4670050.38	3200089.814	4392812.057	1574389.887	5484117.314
158795022	18128559.2	18987024.84	21570968.22	15925437.52
3278699.216	4554670.28	1632155.36	1274021.463	1316484.04
251251687.1	123391848.7	128606721.3	237513791	190581472.1
2308157.188		12891728.81		518260.7726
44680109.87	33258129.03	34837662.91	30998501.64	38676630.97
1129975.441	1229814.734	1201934.54	275484.9245	1153545.341
10954249.26	9133176.752	17839714.18	14203221.55	23380238.53
1063620511	380068970.8	1265552855	684111956.7	989626458.2
7833908.269	7969509.326	7970609.098	6987243.42	9286977.134
	548593.3862	558224.1339		
6230477.989	5538228.067	2668910.991	4909288.607	9603344.705
11758161.87	16712570.06	10725297.73	6843498.215	8087870.746
2478583.513			3252923.534	1231507.728
3036190.19	2898870.726	3788449.376	40014129.23	4432343.567
3511586.549	2681445.164	3766896.632	3101043.625	2454810.076
70897170.86	66451957.15	78467394.49	75991291.8	93594084.71

195451454.5	241781153.1	276912114.9	483095405.8	296428887.8
11757211.91	13858294.88	10377836.37	9497634.221	5971736.427
4698965522	4536817714	4141831927	3025072712	5071057495
3602378.332	2695687.896	5140153.019	2883986.138	2485995.234
33656551.77	21382341.84	21559976.71	14373444.05	43650586.62
204254.2688	516100.8647	710081.5511		516110.8243
26952938.58	19449951.03	16467424.76	18511247.23	8424771.336
823723.957	664934.6186	840814.5801	376836.2089	252017.0461
264713.8077	238359.0403			130530.3887
1680375.408	3419970.361	2407239.444	522694.991	1941696.108
2419583.909	1883472.303	2616009.704	1928393.415	1581342.45
1951815817	257613030.4	20309676.59	20667468.1	53043637.28
1725847.123	750454.5573	563270.9259	446942.7665	613432.5875
6839147.245	4963925.008	2584684.228	1304406.376	3002512.689
501934.4717	422762.6898		4125942.188	1318823.158
22962030.96	19574635.85	19839541.22	12446583.53	11509258.24
15180060.75	13530448.03	10406966.36	15790844.13	14640683.85
1642031.401	1490770.811	549932.5969	112014.6275	283682.9902
1331363.871	4761887.831	2099996.808	1810337.534	2427984.497
307946.3336		358068.7549		
2462834.094	3055045.562	2622301.597	3165706.069	6844753.545
6240608.477	7573016.361	3615922.587	2321839.414	3360785.908
24136056.54	20624549.56	16098410.84	14807837.96	8093511.191
112429.5636		322831.1123	860704.0591	159363.2048
37106852.31	34393672.98	27564126.57	30867094.78	48377530.02
521078.7467	235042.717	276092.9691		679260.547
1161408.526	1135875.254	1636378.538	245223.891	1136120.332
		8982844.962	1148214.649	727026.4006
4901632.232	3780137.075	23615306.21	6002014.203	5229595.906
264473.2825	499115.6715			
21038235.64	17657527.92	15719328.55	8960374.835	16283246.57
115172.6269		882463.7922	615582.1773	1238985.07
76400927.94	50444693.06	107510634	27738193.11	90261975.09
5553496.458	2849953.157	4181980.287	3736127.946	6500864.053
20195957.3	14494175.73	197404877.7	101765131.4	24519122.49
542328.0599	486732.6767	972044.6528	324239.615	269052.3946
84499680.57	138450817.9	83080695.12	35047111.75	109578184.6
1600220.833	1175218.615	2050923.457	7011269.817	1377512.13
196231932.4	189956987	126336085.8	172831836.3	519160661.3
		5178160.742	10914747.49	12579328.97
37419068.57	66777761.82	49153115.99	36658152.86	157529383.2
25471181.9	26869768.34	17851128.73	13163329.1	14925602.19
900664.4153	372923.5052	1507948.721	597951.3804	
212806.74	366091.4213	350084.3319	372917.8119	225021.7754
6154704.727	9773918.703	3257339.601	4372952.955	12267437.97
	177706.1737		690953.4128	302876.2382
201919.3266	134361.7246	408111.9925		
1899880.728	5560123.655	2152911.353	5137178.149	3124860.763

58775507.53	69528955.26	54857101.35	7050140.629	49628383.82
9153524.805	12295917.17	11348174.71	10350919.51	20248635.39
19932522.81	23159797.5	25537046.31	18571011.39	34207703.62
7369114.287	1802711.708	1239364.744	385824.845	799930.9499
39539140.99	52856701.75	30039042.74	27115018.69	26465845.09
91666877.15	56236133.81	86714990.97	66623596.23	188129089.3
1793779.648	4291591.536	464528.2385		32940950.81
3621804.328	4164041.192	2955530.868	2509198.036	2007693.856
4501167.678	5929201.906	2768892.613	1369404.342	
	413372.3335	424043.4914	304800.5819	807593.7525
83381682.73	57955231.09	56785571.64	63053362.85	95688867.13
6217478.565	2675927.619	4710534.287	3049495.262	2809690.684
4552132.185		2331962.023		3763440.967
853374.0773	517622.2864	1309191.744	1916843.457	599288.2199
162925.0108	772028.0019	803938.5592		242496.6502
2621553.212	3654266.141	13953553.39	4594624.431	9993571.996
4986025.65	3782102.296	3718610.811	1942415.294	2927889.342
318587.9841	134619.7214	829399.46	184302.2221	295403.8056
199539991.1	130948248.7	158060854.4	246570229.9	140743975.7
1379260632	1171251271	927906947	1497202814	1275825893
7960071.894	8232670.317	8232121.097	4438442.601	13774708.34
175271.2401	104815.9363	399874.3833	303389.9928	584273.5508
10427081.95	11088908.32	96683574.95	14038244.56	9180581.901
13040056250	12208092175	13710730855	10572513499	13033986705
6031658.59	1776681.113	2483335.208	1328399.889	426280.9234
2613088.272	4935940.43	2175499.141	1045960.339	1090586.29
2545965.909	2214939.894	1587683.114	829195.0911	1681592.902
253288850.5	232973549.9	191934320	208602951.1	289787047.9
849435600.8	12898573.71	13302295.11	7127405.495	15290943.97
619316682.9	610950446.2	410724642.5	585587122.7	533437020.8
412810421.8	479677151.7	443421409.9	482987805.9	588770387.1
3194089.904	2379561.899	234402.2729	852743.5088	195271.1926
182123438.5	227667657.5	216157794.4	45295828.24	221662121.4
766802.2461	1253759.916	94420.52712	571387.8562	425830.7216
238989.278	195666.3264	1131150.256	907073.6415	
950769.4974	1480757.614		651364.7869	6677428.965
15421235.14	26000653.16	16533124.82	10186018.62	12718993.47
49590924.86	31913432.06	29417593.2	32458965.71	31875892.45
2776248.166	3725851.393	1496829.621	1607868.348	420503.4587
1527772.472	10657377.82	5974098.266	8840761.963	7301553.162
110017047	130511839.3	109428759.8	46313442.81	106143868.1
1440073.189		1423570.813	2529715.481	3059904.002
616564670.4	550208897.5	483622853.5	615920323.4	564168632.8
21915705.11	23819959.44	37380919.49	43051977.18	45706866.5
12481302.35	9293803.105	15448062.57	18700479.86	18458132.21

1509458.854	1571168.644	1619701.825	594416.0503	2366197.872
2025892.04	3684644.16	2548781.624	3212363.488	2520001.569
4790132.2	4569660.393	4424053.511	3555287.026	2313391.101
47528208.29	55124795.66	50138537.71	22835099.29	34219133.46
39608345.06	20035165.54	26601144.29	23013270.2	38598980.83
86358557.42	89187025.36	71002631.57	48496069.63	37492389.28
7332052.54	6541073.651	5922519.522	3186701.313	5647415.326
5282369.599	2679750.558	3041441.327	3895799.112	4582186.94
17639068.66	17999735.58	12694447	9817845.242	7810681.503
3918904.639	151110.3773	168356.165	104163.2972	471990.4503
50745460.76	39778031.28	79053631.63	75056417.75	104373174.6
3933352.436	2590572.848	3060838.458	1458152.807	6657575.736
1030694.169		2842251.218		202445.5007
1223237.406	1552849.607	1428739.166	494888.0403	578152.169
645053721.9	1460518872	984309125.8	571993391.7	712342279.9
	899542.3142			468237.5315
4636095.274	10000126	8574676.645	7923632.155	3142006.884
3450064.649	5197596.99	8246896.565	6663239.273	2201739.873
	320587.0429		786188.0943	1153847.684
	457539.8928	152719.2566	342845.3183	
612832.598	2201543.083	8287894.969	4817371.362	4074580.62
3586794.96	4268185.354	3274077.286	1105692.746	3861806.917
346698.4215	434561.9132	381089.2441		258831.4376
9951755.234	14210267.36	11667016.21	9277634.872	11383185.95
94486309.27	57764470.62	141672769.7	97073082.15	56511159.21
17410051.18	2773233.649	4488726.631	3627367.697	6838783.177
39802493.03	41039320.16	43852608.62	27868155.92	56179905.92
		14912657.78	3817107.905	2580269.33
1540187.812	449951.0774	1460737.686	2092349.316	936995.1929
673262.5112		3160636.157	488382.489	1014296.751
1405466.218	927995.7229	848759.2281	577235.5164	602171.2494
1571091723	1766596148	1992126091	1932488431	1180643570
484699576.6	459959617.7	526925136.7	464488356.5	373942519.7
1770541.433	1564712.305	2120540.8	1106935.38	988950.5706
1233740.93				
8158038.867	7432124.467	9651156.059	7918075.837	10638806.89
37340626.84	34644106.79	27352742.57	17906247.28	16700273.49
2545034088	3333776028	1901764200	1066081752	1856738014
54444342.19	43643911.62	33527864.43	27183540.18	46363079.05
		174020.907	1123591.72	1182137.655
728032.0292	573672.0359	600739.2908		1275531.753
18952009078	24539053197	20701425052	28916058944	20548217398
	889590.2892	1256200.557	749280.514	1410191.704
2081842.44	1271737.212			3079881.348
7647990.644	2668580.392	2746854.63	2496299.384	5212312.25
169499.2334	456639.4234	697810.0865	582076.5915	
2124382.813	2254303.071	3040871.386	4714966.027	3709937.554
1059078.26		1245904.743	1120102.534	545386.5607
29501293.27	24229040.46	18709784.69	14582932.36	13475979.58
1283294044	1734409979	1282956920	1204111886	1236821977

1115029.21		550356.3524		1285556.474
471935.7009	266733.0088	1157340.317	992748.0625	1085451.001
1197646.701	5946161.207	2720873.268	4998105.571	6195530.627
10255558.59	7638569.203	7760155.941	4847906.471	5308876.369
4744165.57	6072911.181	5056693.722	2879070.26	9203926.51
3625381450	3282804333	4362592491	3435312006	4279632300
4376576.394	4405149.503	2080254.481	3399588.458	7974084.745
	701593.8034	687866.146		
1880169.103	1343369.088	2179567.756	330057.5688	2084727.786
521993955.3	1039001442	420537951.7	463764276.2	457246874.9
2812496.199	3027781.581	2007436.544	247383.9029	1450078.859
108678.8011	62073.96082	65117.48552		
1447099.712	227423.174		241052.8219	309530.1738
366325919.9	527995963.8	191172944.3	252215203.3	297596863.2
45477363.76	45750203.69	247606052.3	267261908.1	69658994.76
5313054.25	1230647.124	3667306.754	534473.0739	1312373.18
1999276.482	1888803.499	2748371.974	1908136.125	3004472.194
70553824.01	108340282.5	89821952.09	67378334.52	62047385.02
13216561.01	10169887.13	6508764.948	6785323.502	17607023.29
7454138.684	16446109.93	12652289.85	11800474.48	6659400.894
13105817.31	7919005.256	28226044.31	15831198.71	26462711.03
551068.2747	420389.8102	304727.1638	371503.1727	343196.3494
2947322.594	3853247.843	1993860.508	3537538.742	
921279.4733		365065.6398		
1944107.121	2726794.115	2276181.011	2154045.554	2963179.748
8707426.456	2235736.234	2792360.651	2414130.301	4746670.205
7898458.814	6755012.312	5316737.081	3022308.783	5484244.922
904930.9216	884236.2828	1882293.468	1795878.828	2785427.841
332129.8833	309894.7216	881165.3383	352323.8024	533789.0385
263465.5994				947801.1749
2458960.004	2598857.613	2420433.21	3571694.048	2752238.985
				9009.384611
11685429200	11869168431	11798752260	13045995663	13387637961
1893776.918	1209737.334	2272119.249	814826.8605	1536776.901
100529562.5	81282480.25	105954663.3	250958558.2	97923040
314749765.7	336425120.2	239481310.9	266972291.1	336849375.4
385089.9403	7165264.244	4011281.359	591512.3209	854686.0504
83502996591	90225393604	1.00581E+11	83967392910	98162084190
1509938.835	1881718.51	1326167.776	1429869.832	1896813.368
	447170.8578			672392.9207
1731483.325	6335532.287	6120952.563	3724814.953	689066.2193
2724810.57	2419202.14	2034668.535	1226550.612	518932.0739
2336223363	1603943438	1276967368	1493114958	1649827055
50921940.18	53380628.8	82598185.4	34081817.29	38356984.86
26687790.19	20798739.25	19570168.37	20656292.46	8717168.39
4457251.586	1103665.09	1175283.936	981234.7843	696772.2207
206484205.4	186022330.8	218446741	323092431.1	171759769.7
1649366.927		504156.3058		326995.3034
1146589.863	185623.5885	2745559.802	2079960.975	1105869.352
554841.3742	891405.7505		1107841.471	2633559.577

507679.6417				
	663372.4874			
523734.6595	665249.8644	368575.0083	301224.8919	327765.7775
3447111.732	2810341.218	2330336.584	2776228.3	3705919.67
29087260.5	28831398.18	14466522.84	12476039.25	8429638.435
61214660.67	71810687.54	53968071.51	38856567.82	29280954.46
67694004.83	34958179.15	77416129.47	28329429.85	94334080.53
15590529.13	12872800.28	29876737.82	18002934.33	35393592.75
590717.4718	700127.4856	2840083.816	546671.5723	3338176.074
410329.9332	655746.3738		739371.2073	
221070054.4	207747591.1	130201940.5	396729007.5	232765397.2
3166032.395	4231173.377	2753819.104	2255507.951	7464972.21
56714.78834	117286.2613	183622.3193		146606.9535
852585592.8	290760755.2	436607579.2	359839854.7	425127775.6
406188.0272		325459.9359		
4591954.191	2432728.308	1033323.192	1649076.228	6725031.187
20052234.51	7097236.3	9209825.476	8866588.884	14569767.31
2599379.118	3058427.338	3020196.767	1877680.679	4357735.263
935554.3317	816261.5502	937755.2845	708370.6208	1768062.478
752252.4206				
10425230.7	5069731.471	8450256.946	6964102.264	9879064.95
571785.9366	403584.5373		271590.6166	43137.73963
8072665.073	5561318.193	6604746.596	3056331.009	4252228.512
1621272720	1881421087	1636131166	1427908041	1938424948
608842.0805		370421.4307		570874.5307
101439057.8	132588602.8	146972024.5	111639822	82130888.6
620235.6461	414649.0788	921386.005	373423.2968	623537.451
18450517.28	35021472.6	13290782.83	7596451.339	8910493.325
1264536.377		705593.7176	210477.7842	1031480.628
2974225984	2643367874	2433835906	1554060051	1893152174
136491333.9	163375932.9	165504590.8	139794373.6	111999587
7140046.624	7435171.6	6014328.645	2768963.096	12822017.81
1021828.445	9682340.166	1128972.951	1457028.612	1194654.544
3686136600	3752700166	3830768696	3413475989	4032625539
75754107.83	60796413.5	69784611.07	46129426.69	22320136.08
44552832.58	91554123.74	47650383.34	47976355.53	91325607.18
1700687.333	1856457.443	2561119.252	2700982.089	622775.0979
1770353.624	3540677.216	2507203.496	2353230.19	3423504.836
72516786.21	60645704.44	33298233	38656167.59	9922953.294
1450888.606	669373.2788	1453330.4		2715736.142
		362988.0432	429623.3326	667740.6468
8094266.207	7532884.244	9942746.452	8128299.505	17388511.77
328031.3377	327263.0978	226587.2371	214716.9825	767428.5215
2013995.306	2013729.582	1232013.941	432046.8108	607078.4369
142852102.4	182224527	493179862.9	285790201.8	201332898.9
109525.4556	91004.53286	289591.9635		
30250136.29	3710914.63	12319543.36	4123064.326	16428637.2
1597998.135	1506188.525	1504873.043	1656709.717	

801798.7797	3092864.107			1393429.73
36035601.85	30683921.71	31555911.94	16213768.28	22260731.88
7661089.21	5405045.273	21872310.27	6081071.696	4546098.357
13339037.71	10515185.23	11540462.13	7538757.987	11025892.74
24845937.49	34546004.83	27237048.39	25155454.51	20504416.53
349157.218				
3862057	2914979.164	3030538.665	1040655.759	12643458.93
30121587.69	6290752.372	153887189.4	182764843.5	8824624.906
		232325.6667	423965.938	844607.9256
367246.4606	1259456.654	1132055.936	998971.6403	34547.93119
118256937	120922998.3	74516679.71	85036699.59	171812257.8
22823121.57	10002683.97	13000840.24	9661873.135	10150231.88
103960289.9	175536348.5	135889477	91528612.74	103910697.8
65775548.9	70278390.13	62266530.56	62889726.07	110110136.1
26329934.46	28410588.91	17534202.92	20857369.59	36300904.27
576647088	428310678.2	427369812.4	377678564.7	240248690.6
4825914.783	5724717.784	9490770.563	7416698.903	7183422.756
366051.3258	374510.9978	170643.6266	98644.45925	174532.9481
3830068.192	2658539.549	1566566.233	1727662.441	4196054.248
141187095.6	256072605.4	242002716.7	182347769.9	273260733.7
424253.9681	281963.3989	820779.2282	843714.6537	280291.8667
413061.7023	223281.0839	2954628.966	125745.0545	508840.3979
9048327.903	8312002.92	6107755.3	2565503.162	6594506.155
842549.6735	11177233.17	7962380.338	336769.8501	281181.61
74695902.43	106446498.6	61799773.02	73521361.23	67015290.34
4924938.536	3091366.017	4568419.428	4783463.339	4436419.907
7822436.35	6049486.898	7275421.541	5616602.853	3616812.984
99018878.29	105416334.3	125537266.8	97302511.55	124408259.6
1828805.953	1819052.684	2512465.648	1723654.679	2530474.446
30587353.69	7361924.693	9720987.261	6257505.445	9873222.626
8268840.373	10309710.67	14900367.27	8861121.579	18565280.68
56577740.82	60237217.51	43542832.91	12469942.52	30788341.03
2200511.902	620861.208	668936.3153	786689.9869	487288.02
128484295.1	105692872.9	77722858.34	176165796.1	89107305.79
12663877503	12297824911	16224538822	18839733358	15157732298
32341298.77	38224430.62	51682953.68	44981896.38	16329071.14
1264944941	1431128284	1351641137	1063435635	1617287610
3276188.368	1018815.239	2069925.154	668022.2973	1593624.824
810442774.6	921638233.8	169694898.2	321371038.8	1253653967
4406965.927	4384764.65	1197072.71	2479415.774	2056401.258
16166037.03		79190615.29	12763078.38	6923928.696
1053361.629	727533.0032	862562.1418	769363.9672	237690.7323
316277233.5	326992800.5	238509471	479927621.3	317728884.3
40072001.73	26628593.29	5814346.998	9404090.324	9024831.904
8590302950	7731989287	7892172389	9092240743	7616197292
436839.1497	702436.8201	527725.69		736958.0813

16877211.78	22228925.24	17369282.34	9654212.255	17774454.42
866463.9355	1434225.853	764694.8877	1687651.04	1386024.665
8651504711	11185041955	7710131143	10479223779	10810706504
307818.3605	437830.9763	276275.6466	374349.7784	266844.4844
168617764.3	194658853.5	144608482.7	139242729.8	106870175.9
451539860.5	383225571.3	275876994.6	316206544.1	211309747.4
867713.0844	1113777.2	207039.7438	96372.03127	
44450531.58	54304468.12	32400862.15	14674728.45	20399937.43
2491145883	1325999730	1373136308	1717664345	1480478495
	198298.1326			
173259733.4	181456420.9	120317229.2	80495784.11	180497811.2
40104420.06	36494775.77	38563340.41	8882284.43	44758135.78
2196171766	1727846567	1685411337	687006906.5	2485476571
774890758.2	855598384.6	960702241.5	398324075.9	825101292
6282760.561	13107136.87	12153791.6	7232410.17	12467450.92
222482.9489		407519.0096	388369.4206	
1196176.534	1354339.02	1137885.936	1691233.563	
2213938.606	2220359.429	2211701.894	2773911.656	2459243.883
46070066.24	22573804.96	22644154.62	30771440.52	32193872.78
12078329.8	8417671.295	15011597.74	9381402.688	14176242.84
361550.1481	307486.9525	240919.4138	185474.3498	86857.14682
668750460.2	1759115907	1321199665	814000839.9	1079011545
33178259.03	36332491.38	44713929.08	6840336.39	33594778.95
881927.1587	1033749.294	20217196.72	622165.5516	3146876.866
	1289647.545	800817.2603	245132.0247	934475.3405
1523356.912	1311159.791	374424.776	313632.568	355291.8203
2486206.517	974953.5967	596710.2595	3834117.79	660662.2238
8217431.658	8248063.129	10477598.67	8123806.736	5007499.571
		360962.5215	456362.5069	460300.0703
14371721.25	4725193.721	18715028.73	13653825.24	12659572.53
9690701.876	8466783.572	8107738.202	7680134.108	8392044.11
2046324.284	8745822.252	8124356.994	4129594.724	9147221.092
7489114.072	5932302.401	9255438.945	3305051.71	5510070.361
142456418.1	143478888.9	127242502	76959666.05	172485378.5
6212463.128	5719849.168	5373394.277	1732649.548	3113013.091
6599708.99	6000948.368	5385717.066	4448408.256	13094101.24
1101150321	1508636249	1307494569	1337991573	1097349879
1001750.187	1114406.275	1035858.473	737660.7099	702893.5419
			224939.9237	918742.6443
89727.41002	91864.28596	229039.5013		97885.00707
4204890.348	676784.3877	2395932.1	1210188.704	4038324
67869663.02	49433412.76	33455400.63	28453840.35	123863762.8
279385976	203127294.6	243028139	197177988.1	153321204.5
193314978.4	180631100.6	206049267.5	221127874	114470102.7
30841713.86	65271853.83	4367564.903	4675386.073	12044701.26
17928006.24	19622425.44	21532191.41	15715108.95	39653743.76
52076053.27	46902347.56	34532366.05	17061644.23	21393364.11

244948608.7	250854540.4	157932535.4	93182470.75	91655716.81
1367447.884	3149082.785	2792897.733	4542623.798	4887428.738
59960194.4	34247045.5	57098813.43	85590430.84	53408641.18
1488044.499	1248980.719	714097.5796	770121.1556	695990.8136
6032024.932	4295005.896	2358353.324	2909100.844	1739030.42
9525274.578	4391372.484	3151101.5	4381097.591	4361976.979
106862771.8	103701686.2	78930789.67	79945115.98	110303385.8
94068876.12	110664928.3	59032570.8	33690245.07	45698278.74
18785668.18	12802123.55	10039803.7	8561316.433	7403331.617
	340915.2579			
12116539.15	4950910.424	7173791.669	4008504.63	6387138.534
1478783.441	221717.1196	689670.2376	919948.2387	1593046.733
	85100.36954			
989618.0678		1084498.819	1194249.061	4740703.764
338273607	483368162.2	308493425.2	401686986.9	292864523.2
944150.6984	890977.7247	623012.8408	561074.3342	
832962907.1	494107400.5	583990829.8	511368730.3	838976077.7
709072704.6	946732106.5	771260215.9	935612866.5	1122907252
1063496.073	2028461.547	222834.0749		
698972.9172	1265691.578	576062.2448	108511.8741	662927.2626
27492646.77	37953986.29	23105871.22	21093149.39	16011624.32
	274479.2877		806361.2479	
8622648.989	842676.9039	956898.3347	1075409.93	1608218.694
23238904.46	21500858.27	12763290.43	15272500.57	17951438.73
1002714.137				
2410759425	3642300274	4553112609	3784399072	3548796211
1746970802	1333240368	2616232282	3457488410	3230557874
150446.4302		107731.7644	119938.146	
56309263.05	37802846.45	39578713.21	44351209.25	55973536.51
2226553.624	1832836.503	2675324.605	1205899.989	1343251.693
1377272107	1231170562	1186555843	952005941.5	1469327081
463172647.1	558149485.5	372727738.9	178374833.1	526115635.9
11568855.84	8922240.718	16846591.4	9462060.173	8263757.206
589802.9471	428691.7185	396879.3421		
4251103.697	4476952.497	5542370.984	5930780.117	5199172.034
55295586.69	23937547.13	37317999.97	21538900.2	36169291.89
641395.7736	809941.5016	491130.9373	371851.4089	410511.295
		1809539.922		
30360260.37			5620096.961	7297035.502
865840015.6	905572605.2	715022825.9	953947240.5	1054187500
387093.3069	774291.732	522101.0025	58595.94271	618109.6547
34796276431	36039851182	36972746478	45534452337	36181447878
58829379.57	58955632.04	32768243.39	47584921.78	49214452.89
548007.4271	720236.7996			955684.965
	237449.3527			455307.2005
93295267.13	96452906.46	103960717.6	78775615.24	65679614.97
2476023.856	2378008.588	2269197.201	1342041.755	3506149.993

2506909.391	2816459.234	1514303.032	1589132.618	1038654.961
				688845.3774
2352792.603	1019718.033	1840132.085	598748.2067	874329.4426
320786574.2	106346422.1	197367947.1	339181315.5	218998185.5
28364677.6	34698981.58	22352055.64	22019124.28	14577918.66
3326708.503	2017267.702	2695574.167	1930024.364	6558081.916
1020884965	764639436.2	988409282.3	856390761.5	1400915015
6576535.345	2716997.623	6903808.331	15392498.59	17252140.4
6625382.847	13207594.18	8764088.335	5969949.441	19970139.79
47611383.41	34074260.15	58320442.35	60002543.61	176247026
2929540.247	1136048.852	610229.7803	558996.9263	
3651860.575		2389298.078		
18555205.1	13440371.75	23084708.63	25269154.6	32130194.11
9979221.492	10546437.84	6180472.793	7460909.125	8759788.433
1744177.313	1681641.984	1678160.525	488164.1722	1701560.115
106985421.1	124006372.6	91471696.2	31800702.84	70375867.2
1174130.324	156185.7099	4294282.015	4172280.99	2413100.551
21162459.35	15539209.52	19658835.27	23680584.53	28566224.19
192185671.1	314952860.8	104225958.6	209479594.4	109143329
10351748.95	68704842.42	7786796.322	7881788.253	6856422.992
30731714.82	12147719.72	9363353.747	26528685.12	19962921.83
2068266.099	3199004.235	1850846.941	2019291.274	4812811.027
680532.3322	1298037.421	878736.38	492895.5783	692685.6027
36856336.13	43902726.09	58133281.83	34472526	24984217.57
7854046283	6349154048	9886184714	10609212544	8636311706
259328093.8	287361583.8	315004506.2	405284764.5	253791298
1880788.018	3128209.248	2714363.491	1469443.789	1633016.794
	574034.4268	292065.7126		
8771918.641	10393443.3	10582786.41	3666573.15	8882715.69
17607643.24	12842103.83	20296196.82	9460149.135	22540941.76
3538579.898	4093044.661	4287626.651	1916315.873	3609831.009
2274110.367	1738138.344	2599616.879	1274229.144	1935591.271
54315775	47092634.92	45265033.43	36080134.31	62475139.09
1507432748	1334515921	1685738428	1625787425	1630114067
10078276.73	9019276.138	11496754.15	6138412.255	774027.427
9237191.566	11204638.88	12242389.62	8352079.346	17851251.04
29717234.09	49679170.54	32451240.21	45150870.68	68680315.73
			660264.3917	936932.8433
601498634.6	790885124.4	344234886.6	499143049	584147718.1
20244792.51	13727655.56	15166845.3	16664164.07	25152114.96
12103237.01	21716892.25	11984603.72	17674873.49	26888657.84
5883925.443	6939459.627	7215533.929	10619006.67	9255769.831
5208360.518	2930037.692	2589743.863	2607680.14	6319316.549
3886230.821	8142329.385	4642211.82	3924767.836	5208273.097
5491049.087	2874859.626	4784553.908	4728480.686	13615306.14
	2563123.373			
11029787.2	18120856.32			

22160401.99	20928014.06	20256597.95	14097337.52	19578367.19
		688173.6115	156063.2866	
				387212.4478
288037979.7	274339068.1	173642269.8	263668223.5	135794457.2
201365.5006	6622270.189	211869.9777	3856313.499	
29755453.5	11610145.98	14027138.74	40870927.36	7105056.151
594645623.8	565908392.5	408079757.6	347719517.9	324947394.7
3679101.664	13300688.69	5961794.981	3080682.407	3798237.461
5784116.387	4770396.916	4193311.927	1980338.51	11568288.56
12864371.28	18533914.4	4060997.739	5734507.367	10182616.85
128778858.3	175913462.1	150648363.1	103470127.4	172226866.6
2001928.957	3604411.902	1722234.766	1462810.255	3576908.4
11351927.24	7984813.267	88840300.69	8286057.643	20431598.47
31833784.1	29991544.64	25225561.34	29006664.17	27780019.84
899070.7509	1677471.885	1897672.11	1868798.393	
240254.1883	824704.779	172210.7294	139910.3806	1054765.27
7025254.462	7336392.992	4565343.846	6446418.064	3349159.117
5330227.784	4131048.917	3755581.365	3860210.871	8313957.314
7040449.796	8276170.264	4835775.348	4236625.696	12019152.56
212883.1476	147212.7089	117543.6087		
8520059010	8574460598	5734274901	9793528873	6855258603
14868911.78	6949958.036	10897078.98	15314701.46	6627460.146
4029110.718	3509861.539	12894113.07	2570119.678	2424655.626
	118525.5933		129436.1674	485013.324
2940853.253	1811265.512			6288256.527
255041.1176	4374741.252	4803154.389	3943645.036	8527413.858
2325527.547	3205984.98	3448307.675	3610786.962	8912246.449
104244732.1	81257424.48	95529871.53	85959967.41	85492994.47
350884982.6	433776486.7	539685920.3	335547980.1	587683360.4
7372343.938	5124112.922	10283158.75	5751698.431	2868664.156
6158214.259	7354995.396	3663305.613	1392609.236	9551597.539
176990413.1	209554719.2	204563805.8	173513494.8	222175769.2
	1354132.286	5018267.945	4568855.937	1665763.069
530881.1127	591979.4902	1575503.339	1262061.585	627718.6116
2476701.504	5130429.384	34777227.22	81686083.7	6021159.734
		2664585.835	538531.0753	600359.279
702870.844	950928.3283	924536.3923	260604.4648	
2155653.037	1007245.176	1549453.369	1054249.65	2193942.286
37635107.46	32052902.78	306537704	338916812.5	68774898.14
419870.6125	678076.7536	1358947.052	3347407.366	888268.3982
3594088095	3850792601	2683436122	873469579	2219146226
507464.4289	557464.3662	354033.9091	587051.8122	1597101.206
		933464.7605	1122343.988	1019611.502
1892292147	1766203377	926538863.9	1080717674	1607207794
5216992.316	4235381.872	2954786.245	6694807.795	2728666.061
243290548.9	249956911.5	121778558	251103543.7	113108185.2
549997.3348	127385.77	922518.1707	303009.5673	192804.2477
1133999.947	1605554.163	2239213.763	859348.6743	336711.4511
	826917.8662	832618.2156	1071774.933	1030971.165

34151083.17	42479397.19	14181285.9	14406775.76	95505054.31
57330684.08	59697920.18	50122561.02	45998388.92	21023941.61
1206588670	462623390.9	528616350.1	844272121	808245233.9
1094678.263	779208.3548	12989236.62	5148787.216	614667.7569
356797.1428		280194.6497	607901.1879	407604.7146
1516224.56	946694.6254	701986.2693	578527.3491	673771.8169
200583335.8	149413562.2	212112315.2	176619247.7	150355788.5
16020824.4	17034362.69	24133828.73	22165663.23	36195678.5
654026.2615	683096.8828	723431.6368		
34709106.16	21297511.76	17968471.78	20746229.45	16630862.66
321335.9364	413482.882			
4174375.76	3998813.171	5355771.558	10400384.32	8733689.717
6414444.592	4980754.314	5484670.88	4424977.89	6570182.357
361085.1509	947149.9485	1163626.268	82451.71285	336044.1013
377689710.2	875656073.5	720168525.3	700590479.7	474742226.7
5130097.714	4929782.76	1902484.98	2054635.383	6545120.459
10887952.24	14904162.91	9618523.375	13183525.04	12125649.94
40307334.79	23867151.87	15491704	22621917.67	38187508.29
19366679.19	19007841.31	61288900.82	145926097.9	13283222.19
453821.7295	855261.0185	317995.2396		
		975927.9703	1134549.16	1128118.169
1049749.615	1590647.041			742385.1686
7209826.813	5576635.263	7267678.804	4239276.454	8973719.653
354996.1788	272043.1287	338310.55		274111.5655
1771116.76	984042.3797	551321.0477	795593.1487	2962228.504
		875239.6627		
			2017944.077	
214250042.8	127072502.2	873861010.9	150785390.7	307114056.1
8035240.69	3019674.351	3574672.164	4472132.553	8697409.389
6377410.769	10417202.77	8169786.372	9326626.853	19074392.83
7011925.019	7044443.954	9740021.034	9709312.571	8476793.398
1937526.947	1313818.092			1090050.088
643023.1664	261280.9575	463385.4662	582037.3586	
550949779.9	367916411.1	396773443.2	100106697.7	340988874.9
10264291.22	9611543.612	8942313.826	8670641.034	3533320.386
2234630.558	1531439.143	918124.6791	1161640.339	1900574.76
1225717.807	3653301.148	2482987.4	3088062.055	2613743.633
424253.9681	3903931	9725115.825	13637838.1	9086174.591
	775577.1643	829207.949	730200.9165	
545604627.1	438966201.6	242228383.2	539046522.1	558871559.8
707683697.5	930207529.8	552229723.2	1014915932	1139759326
12910020.55	21003876.71	5690075.92	12727680.1	13139686.58
37150140.78	30054363.29	20794915.29	18347252.96	28666019.92
1459586.944	243465.2024	5296014.34	5258538.841	3277172.214
42015429.81	54869200.1	66468706.16	72502675.17	76907413.93
10197875.43	6436124.161	4682429.355	1714159.114	3437999.581
3246517.666	3079359.085	1506876.553	2166560.235	501292.9858
22455735.97	16782352.9	13288706.77	18774639.81	27745716.21

	474853.2249	1484741.952	1617753.643	
7956797.05	6362718.709	7970275.132	4855656.545	12251077.78
1948222409	1509062165	2206829677	2860336857	1539308867
34652297.83	36815451.37	11828991.45	13815707.33	11633542.67
5056068.109	7265928.288	11135252.37	9431545.913	968357.8976
36993412.67	35131958.91	32856717.97	35945565.92	42264345.84
5351988.893	5219289.228	3681592.143	2537912.239	10553692.71
17909701.71	45991320.17	30681373	32613638	26956114.16
108093107.2	70080336.8	93486177.5	90845888.5	112687707.2
274390.5518	100688.3068	168266.1521	116922.027	324702.62
104467512.7	62677998.54	32841592.19	40394154.97	83227085.51
148083.7108	188404.304	178795.0406	51708.39434	150897.117
120227.3063	89454.63066	150699.5335	157822.4465	193353.0678
866224.7886	787853.7548	596492.088		
2797795.156	862566.3164	21658370.34	4882030.389	2589225.028
3925320.159		665447.4253		
5177042.126	4335310.207	714703.6451		5514881.103
1358336.43		1689964.53		1802658.541
389489241.5	454400721.1	276831609.7	233893700.2	179872951.3
57141096.5	43590796.11	71977944.44	60066552.98	63916080.14
120823.6332	288204.6096	226907.0185	323178.1074	317781.759
12115033.57	6382310.967	7509491.126	3849730.257	16325317.49
1876823.899	7292616.444	9019005.32	942964.6171	299454.6762
56592749.67	41700968.15	33467185.71	35823143.84	27390802.73
2059236626	2498877246	2495586583	2630886839	3155129606
13139743.84	10051815.91	14961256.82	8949900.771	12812914.32
254957.6723	304745.5188	226177.5227	978997.6624	1697911.69
82642088.84	69062859.27	51037199.46	62379123.1	43679975.1
11100172.43	5596634.44	7135518.165	7502249.258	12702822.91
43652172.68	38831567.55	46843786.11	32576372.2	50684587.79
1497048.833	2214016.791	208328.278	519509.658	
14407239.64	26944827.89	27685525.21	21527960.99	33130622.56
30496953151	38035618937	30254286213	24386359987	34203691580
	806093.7324	229998.7017		
339324220.8	723376489.6	396034957.6	256169887.5	467379571.8
6998669.843	11697681.04	16028566.32	20420458.83	20074122.94
2323992.648	6824095.869	3048117.935	1410718.697	4204418.751
4467344.307	3902735.44	6562692.222	7044219.838	9799238.861
		3105.90672	4790.087588	8877.319595
117077.0948		2088449.695	95183.15748	106657.2434
2708862.964	1224787.379	2402297.026	1245759.072	2020943.208
105106.0042				
1707437.905	2016001.729	952245.1914	884026.1699	2473515.209
2025726.52	1347477.28	3638487.965	2909948.428	2354009.955
3794000.362	3528933.727	3835972.205	457501.8817	1701573.811
2915909.584	3812901.062	2697810.32	734066.7329	3852090.358

11131912.55	10572327.52	4703308.065	1736047.986	1859874.442
51162.38071	278635.2008		61394.86314	342955.5565
17638765.1	11017828.29	6381710.435	12726498.18	10402377.31
953393.4413	328479.911	848508.5977	335806.8473	1652334.935
1771755.394	1009054.66	5453331.251	2126368.874	1790580.161
	782172.6367	157101.0394		355837.2344
357260367.7	213128063.2	190796459.6	249280656.8	329253250.2
15049423567	17161429083	16493131350	19814771145	15705054983
8190977.898	3721357.698	4053764.025	3553540.034	4452692.781
8793972.618	34217136.47	27921952.06	30497815.11	61121484.25
13803142.96	4921791.333	3120558.744	4813389.185	4732049.127
408553465.7	418149357.8	237495223.4	492659614.9	680830677.5
287332173.2	449483008.5	279123268.4	390864066.7	285211076.8
2146256289	3286674477	1590318263	1912412781	2259317052
		115420.6064	146752.9863	
1450617.319	903900.3919	1356048.621	1339444.547	2141925.02
5344646.588	3184123.502	7582620.058	5732356.952	5239588.191
1777891.002	1674121	523427.5625	1229068.598	
2004819.379	2670451.73	16391261.95	2568763.615	30889754.91
8509157.056	4902141.729	8287945.127	6062079.175	3812787.671
3443951779	3312353267	3489000553	1590097315	2833811831
767347418.9	934067208.5	2030277187	175159425.7	1625958824
1881449.986	2158572.183	951175.7352	253758.3197	965539.2177
54709424548	38078548474	31999794895	32454940829	25970790337
3636211.919	4049313.132	4565574.956	2665516.372	11415114.35
759545.1056	122486.2267	707561.7732	720365.5501	500106.1726
2459239286	2399015092	1816210357	2540128536	2946600019
4387416.75	2808025.047	4048838.219	2910137.03	5772488.48
11594683.26	10216115.9	6861516.233	4050144.55	2061018.332
2280359.452	1059907.882	915666.9677	1905447.438	809530.7692
2664017.964	3530298.299	12438239.68	1345874.097	4245621.454
1410405.281	131148.0687	192422.2734	164556.0875	527267.5596
16249908.99	18562641.16	18882876.9	15373644.81	37377794.94
8141375.429	12973295.05	10611084.67	12760600.05	12885607.45
2496063.781		777407.3595	490655.5333	
397085.1029	281436.1593	200267.9631	262125.9204	
2485006.769	3412988.022	1205525.296	1337075.85	1290593.318
665916.5419	357675.9182	483986.1981	592473.7036	1224038.592
212012142.2	189086295.9	1181539818	1124904512	264470660.1
4920195.342	5278332.508	6546141.99		4043143.106
31511135.07	33366984.17	23368890.88	18115834.86	23198701.7
1777499.85	1016772.221	311075.4767	940254.0588	2687552.034
463701217.6	377297310	382346811.6	131272377.7	248956422.9
3817477.708		5391029.679	4390280.806	3538688.493
	526261.1999		230028.8811	

11690233163	12402766165	9265319880	9020859715	9679355888
755737.5157	900269.239	1683043.297	422404.9677	738119.9741
246753116.2	112807110.4	279189472.1	269164422	314651027.3
2213516.547	2291889.399	1086355.497	1817109.941	2458024.817
20877764.28	33986400.46	23577294.01	24570488.29	41209615.65
342774342.9	314697337.9	384639766.6	292465082.5	265602441.5
1532507.51	1310795.417	332089.6769	336770.6601	874508.8069
214519985.3	139537433.8	915481571.6	511831433	147962536.4
407996.2558	690877.9981	638899.4411	1414244.228	1654442.642
2258539.351	1398746.821	944425.5352	900890.899	2567613.609
112411575.9	134539732.8	167875997.8	82202335.64	167885572.5
13656443.7	16525842.99	9589902.119	8382383.046	17145511.42
5815658.683	3188470.599	3966454.428	2223779.68	5929493.27
11584561.77	11880940.27	16242424.75	9418486.975	4684037.52
469759.6387	297057.6312		160536.5618	152414.2719
159003.7661				
			49569.09065	52728.85165
3142172.572	2936220.759	3102709.419	3018401.656	5071072.809
32097942.12	32705711.13	26387307.34	28294708.85	47041900.17
270945.8335			1805809.309	
251855.1022	135187.0044	678682.2275	657843.7661	1633802.007
15517314.55	18343188.6	18706512.34	16925653.65	27953144.71
6379941.66	4501747.673	5656569.464	6594873.544	3883275.467
84817340.12	75983119.61	152797535.8	62738999.24	88946625.86
9204846.551	6103124.431	4393075.307	3512720.431	12203325.06
27546359.32	34732570.59	39691736.77	25241591.17	30390326.27
599351.572	695363.343	744456.1561	723646.3417	459308.8755
11934500.58	7799567.743	5890950.955	5151311.299	13243414.04
800415.1553	45478.67613	748027.168	530811.619	24597.55408
1265373.71	759077.6266	693228.9016	991207.1313	1174423.776
1006600.615	901662.7138	598293.8793	1317578.778	2154056.344
340063.115	607944.58	1066955.555	804471.3057	3045301.405
				703424.9685
2531735.919	664946.5201		800258.2392	760495.6608
140809761.1	126010482	132694639.2	131889313.4	138922319.4
34526142.88	52656367.58	18424476.51	18739887.4	14048298.54
1329781156	1516853909	1215088053	1341637589	1369194671
992047.871	1097147.021	762842.3793		706185.4198
14110151.09	12562297.81	13986679.69	18071638.67	14229672.91
	27375.71978	1018788.307		43193.07131
1332706.389	500087.9744	414936.0718		525262.9403
11083518.86	9778106.867	10480365.4	3196244.198	16635324.9
4996730.962	6623902.808	1460669.446	3802842.159	7878860.423
4914627.732	4642625.899	1095531.285	6582550.11	7418657.949
17102763.02	1158441.431	498317.3685	542505.9269	696141.7424
38873774.61	19074003.56	97856438.28	83708731.94	23178248.75

5013954.408	2329465.347	17054471.69	2961436.137	708526.1563
23432270.58	14888259.64	27936666.74	39191095.07	27999289.23
1864208.978	1067631.597	1192228.164	842515.056	336369.8855
614239.1882	405791.5794	508095.468	2622164.343	1041718.056
811127.9515	804151.2695			54908.28993
268198884.8	213495383.9	182576207.5	203985859.5	130979055.4
6721517.621	6560837.275	6801352.208	4573914.731	6232964.715
1195282553	1228864315	1224224211	1891054406	1072956016
42940527.08	191301884.2	14678969.78	196687606.8	129169932.9
7210548.762	9615783.499	5521202.568	2894349.617	7428737.65
2337054907	1968230376	2819925375	2364536387	3133085603
1607152.916	845876.7546	288889.5595	286213.4914	1206048.436
64586719.58	43831736.33	33337419.58	29830476.27	42369797.62
	380957.9505	280389.8507		280684.7762
		287785.1564		
47031846.61	11940608.57	69361568.96	10184196.79	20857113.59
17382521.14	30862426.15	11842521.45	20870795.37	53741869.29
2683310040	2247699605	2813845040	1965876163	2673981827
5558256.841	2245372.798		2348528.015	3679226.645
11156227	14198288.99	9831636.202	18243888.02	10343265.12
2182247.45	3410813.676	5396709.165	3449292.979	1979217.999
	287129.5684			
17671.8097		99904.1172	97459.27454	
694802400.4	787040572.6	1053352634	1563743499	802144840.8
82356209.29	138553871.7	60724755.18	84278952.62	51646975.38
		262268.6344		654879.1138
115195021.1	255029894.8	201784859.3	458735760.9	319122960.4
5285838.976	5309038.277	4066923.158	4083346.327	10578755.39
		2219060.716	603858.1365	
6310717.064	4657088.347	5876277.655	4210679.844	2841523.314
239739824.9	237731803	213587783.9	257945837.8	188030615.1
33793466.01	34091412.96	33334723.83	38330726.3	35187963.66
3213196.932	1675199.831	100797.0898	749173.5215	1103733.706
		921203.3276		838335.518
669328233.6	525573457	661183591.4	302294953.7	614898975.4
3166249079	7082544800	4296551852	5527569871	21476630270
894734.5177	310720.7009	368449.1021	347244.1601	1587802.088
4365572.521	3391611.402	11300366.01	9471077.758	3596263.485
281121.9192	221603.1353			361753.8102
503432.6914	357930.1692	434639.8376	700937.3289	660205.1616
10777463.88	3142878.873	3277368.867	2319755.843	13047964.94
4397270.876	1252174.938	4044253.232	4627726.031	9131420.737
330482797.7	341835094.8	472344112.5	342301851.5	497266752.1
128262150	117445279.2	66887669.06	35952476.49	235318390.3

9865206.38	10887632.64	3294686.612	4230743.786	2431564.318
245280347.5	367283449.3	203477297.2	68567422.56	216602730.1
538653.3239	1284861.601	521844.326		
128279052.8	88209051.94	86360579.82	83634726.53	55811061.79
				61413.75121
83111364.6	70573559.13	10358852.77	64602498	54235920.23
15370564.78	12525584.34	12011242.81	7564113.549	17294424.2
262444.4737		152545.269		453642.4257
			2098911.674	
63018011.8	110059994.2	78845262.39	73978789.83	121425428.4
964918.669	315449.4226	316241.3061		514927.5868
376206.3881	202328.2551		310403.8904	534816.2292
1610678.048	347009.525	170370344.2	3048537.683	2676238.564
72736020.08	81883695.52	119052775.1	58030847.35	135506383.8
5873571.943	6255055.944	7593203.562	4093136.94	8171901.85
626681.9765				
1145109.254	1362507.533	1345853.124		1058750.267
91430580.34	128439124.9	114294214.5	182313388.1	154647516.3
3653406467	2606990439	12716378607	3120301227	2464103328
1457489.211	1995777.93	4239570.023	1154832.793	1664508.9
			192129.8067	
4322644.636	756111.1287	384355.6498	704011.2848	648059.2921
12680652.22	7143861.282	4222431.669	4215134.436	3965989.119
440306.0347		188720.3874	215448.0919	148570.2891
469710.2484	544154.698	459101.0563	2122047.283	917959.5888
1737706286	1907198803	1570999668	2427013993	3118484486
375529651	322034535.9	1437787153	1341943179	512674798.9
11591805.73	18031384.91	12068763.97	11125912.01	15760885.54
693396.3241	1272133.642	636004.4085	619224.0068	409309.8761
7106802538	7514900158	7514307387	6798751163	6463953949
12460963.84	21310780.99	19484155.68	23649720.8	17289942.82
42251758.18	16490192.7	26264122.49	19041743.26	34585605.85
				363769.6048
53886630.26	30500635.24	108821340.1	116612746.5	50720458.91
303813.2732	283045.1169	761662.7871	438564.4067	284426.6287
4671305.269	7379293.405	10134166.16	5743424.299	8655058.065
7557354.044	9257244.233	3408695.933	7158875.908	5652564.261
4436184.892	2350906.769	1949759.383	951711.9566	4534944.325
14297959.62	10862085.3	11876921.09	5988396.416	13651942.29
8617575.208	9245112.017	9884020.733	10484184.4	12868104.68
3915486.011	4496784.713	4928931.39	5846030.791	5982005.455
6634466.961	4351795.611	6983864.917	4268542.806	8825790.86
	748831.7855	943944.6571		550469.9005
1566607.097	1189940.645	1981641.374	882075.0143	5005762.798
		484784.3177		
611571.6119	1147479.379	1366557.399		1872294.242

17384747.41	43106621.77	39846482.33	31122789.27	15462360.1
37647350.88	36858334.59	40207646.44	22420656.69	34519092.88
12502886.15	8900334.751	9692205.196	13029158.63	14209787.97
817083.6237	1109759.977			1390288.102
85663874.46	93411031.27	100358802.3	98563040.09	234317590.8
289669.2014	414004.9502	874733.6736	687262.2075	1717241.637
139105.0301	64727.14788	122159.0656		158297.5759
1386871206	1048690442	8798127897	1092766755	1382784566
376171.7436	412332.4476	438862.792		492365.348
10981078.18	6869900.226	16793103.39	40085461.38	42781466.67
23204864.82	12035083.68	20329015.24	12520979.58	8072747.527
4384052.023	1555055.477	1156624.272	1273363.097	2994589.336
1792104.582	1591895.84	2729162.185	1632509.906	2695933.579
1905266.561	1726314.238	3163401.238	1263706.409	1744297.306
253537.6927	121012.2093		100850.2458	451475.1689
225709.5042	172756.0893	239632.1935		
4600625.117	3923093.833	3349197.7	5369404.233	5573833.873
1800614.665	2565231.623	882287.8557	629603.3778	841092.1391
1712755.335	3626717.062	3031356.017	1464036.636	5247489.289
33642402.97	20779572.36	47039440.02	31882782.95	31546941.46
		916269.8225	1080033.851	
116880357.2	106964680.3	85777219.37	44320471.11	65830880.7
1740518.047	2113070.602	1505056.168	1443180.904	1283625.38
4336342.529	2479002.488	3174779.027	2977816.077	5461414.619
		1346712.072	583941.9867	
6483284.669	4447596.564	3714689.603	5707629.575	4394869.093
32088144256	31804343342	31714066896	38536421033	36033491196
310200995	272389887.9	397146087.6	495389060.8	290209405.4
11233122.31	13051766.01	7880541.896	10175143.49	16167233.45
				203014.805
16884500.78	18572164.2	11924705	8958503.012	13097266.84
95543.82305	57375.307	148581.8904	89829.69477	
1008977.098	641324.8547	1590778.088	1490128.239	358937.0696
410606.5705	584303.9291			
13305705.1	8195364.763	17294139.43	9863032.976	8515949.907
6263338.027	7003652.606	7514296.033	3423133.6	5970073.246
957013.5551		268689.8362	220284.9937	488848.9919
	197063.9986	556114.366	417908.5718	436977.5147
654750.0589	332869.0883	2859491.81		173271.8583
3219111.117	3174331.969	1970478.593	2052963.37	3962627.971
114758842.3	102868679.5	163721062	78485125.9	161651152.7
575825.6603	690217.9656	638060.9514	346100.8057	442248.6309
7435024.195	12924598.37	4354336.075	4323454.15	25389713.69
536422.7126	355270.6994	528385.4437		582136.1113
20739886.45	18209126.34	16572573.82	14375636.51	26282525.91
63551684.32	36252962.02	31193197.64	46075701.62	41035602.3
7210825.626	5618934.344	8078126.053	4001257.739	7666344.845
2638455.136	2387832.212	4046318.114	3253846.282	1916571.472
107864098.7	88135184.09	482187709.7	180752947.2	121996788.4

				1476637.342
61326740.35	31874053.73	22399321.8	26657448.52	23695745.05
10865367.22	17615596.02	6785544.906	8679938.18	28472426.74
1320804.303	892975.5327	3756262.62		
10417105.73	13932944.97	6183549.876	9108849.791	6695030.866
11709294.7	9984820.06	22108082.57	17504818.91	8954813.984
943965.387	821524.6224	815469.5699	2474746.739	1874135.06
8512703.656	5113991.55	9501673.96	552753.9609	9691167.599
4005168.52	4840437.579	3721285.799	1303052.03	3461199.062
1322797.389	1393504.674			
15805399.52	12307519.45	9118186.395	9243900.633	13535049.46
3005733.33	3305989.575	2506361.224	2104727.585	2333393.562
377445.2806	441011.8059	295185.7016		
5828899.006	3704972.862	3389477.822	933724.414	1848406.164
		1182595.778		656336.4281
1534998.381	1755952.988	1083172.028	200626.1118	630888.3356
616342241	698357982.3	475760112.2	897343707.7	1275446676
25993964.12	23132100.83	25017196.79	24173000.47	11802370.6
567531.0164	202546.0514			234967.1042
			1680665.749	
486980.2304		573187.3274	65506.26396	592006.5574
131982045.6	393862275.5	79119083.7	89448888.52	218609380.3
14524.82919	19105.17883	39571.10036		10543.17692
58585307.25	65237373.03	61631302.3	62523619.46	40507642.81
699194.9081	782784.7094	704388.2477	96831.26603	529816.7929
1043475.948	622868.1546		437595.7162	
896535.6115		1117644.649	1404492.029	1142017.95
608402.0553	891619.9759	773414.9084		549846.6948
36405981.79	14156709.91	14997804.77	20495549.87	11253348.53
22377294.14	21530115.31	22415758.74	9126905.036	26492858.59
5054805.21	4193635.613	1810063.227	4674646.635	4170942.654
26579146.91	37464913.89	35507831.16	44070330.82	43242802.42
537930.2725	542731.4622	436550.1715	55024.09143	123548.3658
227862580.4	96882134.99	90028920.7	50569283.21	109142147.2
3253529.826	12603598.21	10177344.68	9368983.83	14742518.91
	284053.4251	351791.3022	1546195.714	541489.8522
905524582.1	643422076.3	1307959036	716154157.1	960427700
548782.2711	1402065.544	1771550.808	8559247.142	2618071.892
112911561.4	207647230.3	139720825	151574551.3	237742656.8
	704139.5722			1251410.87
14946295.17	12926851.54	14759134.03	18099674.19	15961964.25
	69751.4302	126171.6678		107923.1937
1371981.979	547825.0366	441752.054	167408.7683	291748.7142
223905764.4	106900448	662360682.3	1414060864	281017416.6

733904.0697	869249.7831		773903.0826	
1475118.24	1983964.148	1624816.538		1450347.893
18800888.73	16096978.45	12417817.76	14855188.46	13829402.18
11174267651	12285354134	11446985290	12931868924	9791056112
36990266.56	42612511.02	40682778.12	41949684.61	60751943.62
381793.9673	263056.0922			
12049009.23	15206324.97	17434529.08	14375627.49	16710859.41
2802617.263	2638363.431	2297296.502	2207217.329	2319770.269
324320617.3	335134028.6	235262530.3	217745219	123351891.4
475670.9208	1152500.217	2780235.739	1447502.865	402361.2583
	1157104.364	993668.0654	732129.8803	1271722.994
2369629502	2347226053	2622835980	1018440076	2401500764
195696.187	58544.7669	229386.598		232733.7645
16390359.21	16705821.43	17626089.74	15379787.05	13265036.45
601391.5209	2096831.922	2611078.531	1947533.799	3291846.59
1584635.985	1917777.807	4284555.335	4876054.2	1214035.738
11465014.95	12743222.56	32822474.19	6744244.131	6139018.391
		3079981.636		
321741.7792	304890.9569		142830.9178	2545257.134
1097403.911	1404790.047	1837836.205	1801968.454	1673179.091
1610108.817	1660114.808	1948742.565	1061079.313	1593846.66
341593844.8	441877946.6	395383590.5	58115668.83	217185947.9
7438725.024	2420516.396	4516652.447	704080.9638	2206537.491
205764.7961	83422.96992	241409.1095	148807.5378	197935.8407
1463290.876	1202735.584	690114.0243	341777.894	869765.966
35169258.18	44623788.06	24563509.28	5103350.935	31346538.05
537712144	638867918.1	486395299.4	144428716.8	477352146.6
4398779.052	1289430.646	4022948.882	457876.2145	158516.8057
447975.7637	472933.4757	636294.734		492821.7708
26222712.37	36438785.63	13291479.04	361891.67	11349623.37
1146231248	1220796770	871517315.8	495355776.7	690903562.7
378686.2243		2271844.123	231413.7963	361984.0094
4815994.627	8621451.038	21050223.1	2875400.784	4203289.621
8124385.297	9408655.138	7522583.936	6820768.335	14447430.34
785330.7842	526393.674	623783.1658	428062.8906	678155.8041
		2381594.061		564940.7433
1330321823	1723399603	1128206938	372690375.6	1123863713
1215155.832	992885.882	833818.6537	923769.1874	1161193.817
730139.5969		8687022.509	10248137.72	
	89520.30096			
6907258.562	6582249.28	7005859.403	2330366.834	3367857.229
20659529.57	13408741.65	10649680.25	7010077.836	14404020.67
999382766.4	546317901.1	918430046.3	594155383.5	540181653.8
48252953.88	39585888.26	25857957.42	27473874.95	39501298.84
		733452.827	595210.4289	
147058775.9	124504454.5	112463543.8	108601227	51308882.78
186141229.3	377393321.1	129340074.5	100031289.3	370487496.5

638332.2911	97297.38711			96715.12667
1576496.443	1285825.651	1501859.217	1027197.528	2255759.989
962165799.2	974506343.3	1476871131	1300085252	1156659984
8778187.07	9007518.661	4779085.445	1570018.093	3314202.144
1206334525	927398062.5	1292147913	1879253515	1850603903
32835594.32	24269963.4	17544517.64	16934488.49	17847447.12
	447045.6451	341892.2564	250712.415	209984.007
			200498.4816	
766763.799	554547.794			
19291372.28	18528137.61	12360421.4	8754593.431	16626425.8
7636279.343	6392633.282	10415647.15	7810510.466	7536597.523
1608235.986	2105822.952	1349194.751		1181792.478
4752799.573	2710176.29	2511293.324	1607511.737	2129323.091
1160621.115	1077715.636	1113205.44	1218992.71	1762863.312
1587284.909	1161027.579	765330.8403	359782.4601	
656620.7498			204991.7254	479567.4252
4000544.876	2216273.239	3289301.569	4754642.883	4760006.608
6844349724	6852317677	6117104360	7510650622	6777648104
63421862.71	38911465.17	112602989.2	32740298.15	45187360.11
956866.8044	708211.6264	1015286.27	992218.6293	502435.3518
201426.4144	28247.81262	6399130.285	6368900.34	12044369.45
1177168.875	884831.991	596146.0935	1241310.115	762992.8473
4259696676	3467702250	2926410154	3891841992	4758392224
1290803.976	1084099.327	767124.7725		
9117073.553	8498959.963	6916620.352	5795695.655	4651799.593
24200465.29	24753855.89	21562196.99	13835558.74	14653335.69
1427495.143	3225026.674	1808035.5	668306.9297	1805099.936
881105.2167	667758.2288	1147211.495		743978.7697
1232963.557	609728.8267	1005643.837	823208.5132	2239736.029
7695923676	6359959598	5670885800	8386627904	6073123791
229128.0658	186005.4432	62346.57805	56970.25486	113243.5084
1215729.968	407221.5856	911603.6884	313472.2191	324001.4908
28951323.81	25154206.8	19421472.76	8199741.197	14386025.89
7787555.433	12945716.8	11552696.34	11418046.31	19468206.44
676561.7274	251917.4502	1008683.781	807959.7254	397135.5469
29802495.12	26448279.17	20910938.35	17947428.74	25039849.15
4504528.887	2882745.157	10016900.14	5671409.134	3472936.628
1513175.362	3386884.717	1254763.05	1186370.041	2802459.82
2712465.898	2016483.519	2763977.382	2104114.228	2161718.856
	1415596.671	4639694.973	14401199.47	
43166227.73	39726430.56	29560830.6	21469830.66	35421769.06
27454389.38	35586670.23	17981764.52	25052318.62	3831203.511
1727746.483	1231088.15	1772765.91	1191274.579	1785652.744
	688124.9463		442531.8096	1065985.979
4839726.258	3441877.417	3640615.08	2668006.148	5387673.343
30653554.98	31128541.5	16555504.43	26348156.19	21387586.36

814458.715	1009608.952		785552.9365	1133076.543
243364100	361744168.5	223969196.8	197381270.7	186908524
375531.7482	1725539.459	4826621.205	1072365.716	585572.1789
3098971.585	4149165.518	3878751.642	2411281.054	4604749.985
53083.06823	523614.154	72904.07578	39383.04481	32542.11502
12103187.27	7205717.323	7538571.791	4682669.873	8732179.532
1454500.36	964201.733	767379.2047	813827.3366	617643.7594
3168100258	2514723460	2253637019	1886788921	2587187829
138891834.3	107147010.2	141119774.5	118017093.9	135454018.7
12351470.07	28463960.59	13960398.5	4100323.107	13057724.16
1540729.93	685951.0612	1627269.425	719512.0931	1976281.117
45445962.58	30157113.89	35513186.06	28244601.12	29076035.28
27093316.98	18585779.15	14802173.19	13852133.69	11690620.12
4231938.715	2734507.003	1394062.583	3790680.525	3971901.637
3457756.074	5821705.159	2390305.073	2599694.479	8205645.041
10667738.88	12694576.1	10164774.09	6239852.593	11391166.35
4390072.922	1266875.91	1737553.251	1471371.274	1291465.341
6790980.213	5599064.369	4120532.823	5732415.555	4793110.061
2008475.451	2530916.627	1090120.326	559933.6905	1116060.515
2365007.616	4804763.803	2651016.141	4172600.171	6454736.332
9575037.772	3471061.426	5131395.308	5207461.431	13402371.67
807284.436	663374.7543	803915.5248	592741.7131	704247.7804
80371.34472	43348.859	209956.1136		120758.0245
14204393.6	17117366.61	10482605.46	15190535.53	47691380.03
14410282.93	11332548.73	12932783.12	11757721.82	13065475.23
5979882.722	3187277.837	3603778.483	3213912.203	6911802.171
690877.2057	231148.7413			694589.0371
5988354.116	5291095.622	4425132.42	4182335.43	6636070.581
996916.2337	1263681.798			667815.6022
	794708.6143	939745.5038	838396.5147	
482087.3498	2183908.802		1399896.213	1788475.836
30435818.14	16918423.1	24687542.83	6452394.147	22334104.54
29125052.03	14873091.21	32926923.99	15071837.55	35232559.85
1401076498	1798154855	1242401334	973645500.3	3071577742
2859701.162	1869927.593	1335743.151	2347613.243	3455612.197
1847111.982	1847111.416	1697012.435	1781658.311	1670814.725
4372776.117	1384472.352	3885156.415		2340923.811
2299154.508	2609959.683	5790243.095	1772497.132	6219077.2
903917.5793	789697.6945	1782680.395	510242.6584	1282187.847
6088655.184	4084569.65	3370651.55	3647438.19	2426383.769
1671148.667	1575852.76	1435737.912	1876833.304	1979762.081
168777.1062	42343.68995	253626.5344	138238.2357	139803.592
7851465.69	1906418.617	9603496.797	5420246.016	2761720.618
11832726.71	9621841.601	7028847.465	8020079.976	6855764.709
806668.3748	784997.0439	1227432.708	851565.6054	750984.2828

83061608.54	268242898.3	110425325.6	190072764.2	400623046.1
8121744.627	4531504.18	6909736.69	7088559.663	7471313.355
3241685.014	4415542.624	2443284.32	2058452.656	1285855.386
484028.6432	291848.5671	1707245.024		292621.1004
6347149.087	4725405.679	4024433.282	1829720.079	4520625.054
502147.9981	410155.9413	278084.2747	345458.1465	476484.7046
2870082.229	2524059.465	7785736.039	3376506.82	1777020.394
1780132.64	1834823.827	873599.0799		1986025.457
1048646.698	2393242.608	1905834.386	2272896.659	8643297.736
5566407.815	6476414.41			3009024.544
6241435.422	2856624.536	3581471.899	2027069.641	1359243.915
235470.26	787905.3276	144193.4145		
1550009.312	1456101.146	1870428.571	2611843.197	1818438.097
	236302.8676			985785.1131
29656474.95	41322378.46	25882546.93	24378114.71	34379482.95
41454005494	32713965762	36757893364	34013289628	27812630423
307345.6447	537749.7824	487985.3582		
662179.7653	968287.2471	720792.7496	760546.5614	1565937.671
25519523.05	15855855.02	12497517.39	7784335.344	17239524.19
1967391.778	2128502.201	1292531.309	2047089.138	1742674.821
	307260.2235	481396.2868		585834.959
107123576.9	111839486.5	134026528	101547247.2	137974651.2
15363029.16	14870238.21	12767382.27	19493924.16	15320108.44
1557778.248	1555443.513	2784080.24	897562.6511	2413812.36
3222678.668	1689719.094	9711432.396	13926028.83	5132197.248
58055626.34	49053590.79	37888751.73	53143104.08	39776160.97
166195627.1	76166807.91	137162496.3	77560609.16	112727974.8
14620474.27	14566974	13074710.08	18610298.5	20276672.57
6080543.225	6063951.755	5755051.777	5613663.906	10334991.33
7609581.482	4821933.043	2043146.535	4053638.182	1402444.589
9669513.728	13537779.76	10559456.63	6277713.148	22998622.09
1333272.198	1064096.028	480394.7556	605623.0722	
435518.2309	337497.7814	924505.0036	451342.8058	592529.354
919028087.8	1068644381	496896880.8	757277115.4	1048173820
1070863.788	1027929.264	2407640.996	1131210.717	1346816.763
		5440359.427	7083866.01	972616.5347
1327509.888	1293102.373	1602225.363	2146685.894	4792717.152
536004.5135	437253.2619	423811.806	832773.5986	385065.1176
13687328.12	19447330.53	23517958.26	13643717.58	32726105.59
108505.1163	77027.28888	91888.12985		87578.56781
2960974.639	2522144.86	5116745.378	2572711.114	2015989.517
5783793.568	7295397.018	3133876.722	7182181.11	11124421.21
1198997.1	917841.3384		650741.7794	
2284606.499	2381733.659	3092481.91	3233324.54	3370450.945
759936.549	637986.6324	1199417.374		
17832814.08	16982605	17369677.47	14978531.27	12421152.09

336786.3087	374088.4976	238094.7502		385277.6931
		503491.4723	498543.4992	
17771.08537		126775.8164		
4375569875	3767794868	4200113005	4601035391	4516340006
392127.7168		1187146.133		2404879.923
5957029.042	3424354.183	6026312.994	261857.0833	
12936456.99	14858175.99	6629499.709	8974580.957	10712670.97
1398176.095	1435333.181	1181411.689	1189259.727	196170.3053
4257388.942	4735762.423	991741.7416	3596355.656	7871389.808
11274898.37	12758447.37	13806411.97	6558293.316	15143684.42
		4521872.428		520340.7108
32905542.63	28544392.19	20094245.11	28691639.47	19532939.96
78737519.13	55627940.22	106068540.1	59872372.31	32283415.95
10972218.88	14756740.67	9859610.451	5656055.044	10498172.86
71057607.69	34013176.9	73920150.71	12692081.97	14330424.88
236822.4911	364015.2978	313121.0616	154747.8039	338899.3376
126168858.7	227663250.5	134128107.4	126493750.8	62044961.4

Intensity M_ZJQ_1	Intensity M_TXD_2	Intensity M_ZGH_3	Intensity M_ZJ_4	Intensity M_LFH_5
			380090.6657	
98231986.27	119692264.5	81675271.46	206415476.2	170032014.2
730298.667	796129.9086	306254.4607	579344.7455	314311.894
	628474.1109			
5430217.684	1390330.794	19894800.52	4464627.955	7862741.011
1802178.453	2381922.33	3025386.562	2129879.701	2643295.797
4852078.145	2392682.043		4684540.786	4266048.807
109335826.9	88680602.68	104893538.7	106394546.3	64787951.09
1685365.829	2704965.565	2285941.3	1560703.89	1370866.53
3220742.838	3256880.164	2288232.235	5287845.786	2654895.761
8992552450	17063066026	10497891459	9935531882	10222087109
	29935.38158	75837.54393	146648.824	64602.46624
1186120.669	1440986.112	759206.5016	1655726.099	1199277.847
2129277.196	1554156.22	3285651.736	5245505.565	4360798.177
48124001.48	6963415.875	29090615.64	9986687.761	11632544.54
				5086152.138
	646481.1146			
18531338.3	25428864.77	23724216.14	16458683.49	8893325.883
1983452822	1167809620	2042997274	1978506312	1606375030
4672421.439	257730.2542	1099799.125	2714674.525	1494775.658
43225404.35	29587487.64	20899289.59	64421369.87	80094445.49
2107493.502	2589265.616	2456595.073	1615419.113	2699932.679
2354562038	3286996547	1790025873	3069088774	2400098671
12640.48524		33309.52277	111237.9698	16308.07072
132273500.7	143746895.8	74393649.28	75597434.58	105475089.8
			575454.9957	
48352687.14	55459319.96	54140003.23	56898185.58	30075808.96
	51021565.5	5331802.208	37781701	
9004159.482	8679482.828	9909989.123	7652492.111	4808490.731
9501454626	8994333806	14366606970	6591563661	9389002634
	598568.0053			
1644070.645	608531.5798	1885894.102	3037537.829	1563451.574
	406808.6112			
1816983.358				
9871344.831	11329913.05	5553883.438	11757943.02	8343102.131
40327311.07	17644792.67	53477060.81	17840853.65	15486737.51
766363.0859	1985542.594	1471483.488	774140.2152	895887.2697
32894936.61	46517988.25	15587981.37	21436508.63	22657692.39
27318393.66	410014226.4	36536378.29	41215551.67	52744608.81
2311932.026	4555180.321	3733826.858	6706975.21	4454741.116
70275351.37	50872701.39	97034724.96	76570954.69	69138640.24
	1275961.061	753483.2197		739362.5647
163289.2443	176066.3283	311919.0783	235963.4827	
			429201.5051	

592767.5702	33224.93603			
2043173.564	5773148.661	2867677.482	6623841.279	2418302.622
920476.5378	1484908.702	1525369.211	1317816.791	785986.1234
700700.2468			992998.5275	
1427109.547	1433059.032			648240.1449
	1161298.348			
723513.2106	102313.5522	443821.1334	941443.9221	435892.2585
1786817.427	1904451.082	1265533.485	1930548.467	1613264.297
11770941.99	8979187.362	6479420.732	5006078.071	6010978.071
3037801.088	1489391.668	1705671.631	4913770.875	2262370.733
1551864103	2390964968	2205746759	2294029652	2394653531
4278715.348	4439840.596	3502296.418	2341872.276	3033297.868
1475393.304	1952476.084	2218587.814	970396.0752	1353428.961
2113038269	1884153673	3549187168		2319265.58
30411459.57	70988728.99	21202974.81	25698493.64	35008077.33
4155865.127	4880046.005	3695034.96	7086647.448	3561996.298
11851068.15	12059176.73	17363585.39	23849502.93	8664448.061
150078984.5	171191853.3	236237132.1	269269388.7	98917372.62
5245294.992	8385249.797	5885607.285	4862855.597	6595370.137
8566036.417	6917118.597	7177658.77	9555072.583	7734230.973
15714346.73	91287.07612	25282067.8	55698.01603	
562251.9076	205770.0477	552622.5373	229728.9803	
80595.09872	96813.47967	77851.79804	51186.19525	63305.14338
3686327.4	2962716.796	2973311.829	5070424.6	2544934.156
6883206.184	2363479.544	1534920.651	7552478.835	1693128.431
	2260905.886	2518646.063	1338579.482	1630594.841
3765272.737	5463743.619	3511443.054	4067361.029	3175557.709
2915036.548	2515742.96	1837345.371	1572232.983	1434203.151
2127615.364	2511742.258	371401.3351	551165.8825	970295.9431
14264407.89	189032103.2	28696071.42	38714124.94	6957755.73
776502.7217	275753.7183	476420.9138	198778.9506	4417307.562
30797094.76	11543554.25	43614530.28	46170572.76	46440626.03
671712.253	357065.8771	11809549.71	15420452.34	2799175.025
9824990.422	5245813.034	10905639.34	120145743.1	407914625.9
25123901.15	25579631.86	21124811.47	16013835.72	22915708.89
7838672.228	111324880.5	8577916.4	11807295.57	12540358.42
			475789.4673	
390119.3233	21072.91659	547648.0429	689065.8908	191654.4616
886845.1301	300837.8731	459565.9514	243983.3393	514648.5502
399114.9718			265751.0694	283622.331
1448766.276	740281.4291	3665582.695	5577320.718	1923783.517
1183357308	1480955758	823287202.6	1095168617	1230830229
	709263.1535	707404.7318		

2045495.919	718640.5734		2306972.31	757774.0221
	937652.1804			
1134631.303	469392.8852	310025.6062	1805670.672	848837.8185
258315.0872	163503.9809		626450.5941	
45057258.04	58188274.58	54433700.2	32323113.8	35235871.95
256318900.3	458647036.3	401959947.2	324891003.7	266261271.9
13279564.34	24371054.28	42622317.31	24047806.93	12810928.38
7771623.767	17123254.85	5171697.433	6327853.976	7823448.965
10813414.47	76120756.14	5897682.137	9791017.729	13266171.55
372325.4432	823740.5613	579807.5609		516154.6149
	130462.317			
	838592.144		265634.6971	149605.9732
29499935.44	17131166.78	30086502.86	7703440.257	17308689.75
34161337.26	36803034.22	33897158.23	29509873.53	20547358.1
181275.4631	186185.8696	210700.1599	166857.1237	142487.1246
8287092.769	8082108.887	14301737.77	11816521.76	12294755.87
60389.42483	66327.92822	13859.212		41879.55813
17253594.33	15013685.26	14319940.73	20088584.61	10429376.46
170359.3919			574037.5047	
10415697172	9397147566	11174976170	11127250580	10195698304
	193128.9531	120221.6658	274704.7506	60832.456
25728.73844	64689.38955		37778.15914	
2916961.02	2527374.344	2684668.844	2267957.22	2396666.008
1142717619	1291643745	994119061.2	1544070368	1587586541
347513.7049	183119.9627	813008.1681	1342500.126	847458.7769
3094231992	3658689054	2513304800	2263143220	3389420369
100561.1638	10106.1762	83920.60682	191446.5321	118502.8273
787545458.5	1050399102	993768989.7	1251161721	1208752370
40928.95476			76479.24901	46602.50582
		483240.2654		
	1286595.153			
				80051.00724
			743962.0528	
5563049.034	6952814.033	4735167.4	9111090.954	3229577.041
7655693.754	7585874.09	5557142.694	8068515.573	6055523.591
5025127.862	5185920.138	4013700.157	3761312.525	4748515.177
1569375.615	2383102.402	404821.5049	2289034.498	1232009.73
22509181.48	18802435.09	22850631.97	22847984.23	18746843.32
31075053.89	84842778.03	30486890.17	43602137.8	35099082.19
64837.04213	59055.36034	23243.518	204923.5103	
5964711.695	6365766.348	8196852.506	5085134.008	8499940.564
583245.3809	819785.551	1078722.205	2193640.473	936744.8509
249048.6601				
261174062.1	398385037	320548081.9	315069291.6	265062015.3
3128641.438	11561926.5	3741157.117	6063468.872	3968096.115
254451902.6	170081988	90779280.21	199503096.5	162074588
3035428.741	5401299.702	2236985.282	1127731.564	2239660.821
10539345.74	7955386.109	5912080.507	18764062.97	5992121.063
3379951.937	3457982.266	1226121.591	3639575.583	6177175.903

		119042.2671	1513775.877	
396779.786	558788.2707	648254.7043	886761.5096	397687.3374
508870.0255	544467.3515	487821.395	198562.3731	429583.8479
16718086.13	17353304.44	23021492.59	8315815.435	10810932.19
		323752.7574	345789.902	
	502307.6815	328813.4066	238966.4158	171259.2532
17189514.57	15405533.31	11894948.89	12879137.46	10563228.09
1719377.03	1178372.066	1008331.377	2114723.23	1059069.443
244362.029	240040.0241			
121615376.4	117021226	99353921.94	102436026.7	83351433.24
			225618.7935	
43421572.6	35545542.69	33688033.69	20727492.19	25532588.62
638840.5618	500918.8707		404311.4815	
6285436.394	7153747.444	4777443.256	7549088.579	4593694.185
57975983.43	118062984.1	101034237.5	110717306.1	43144453.59
814153.169	1796924.485	636733.0608	2269646.22	597928.3654
147270.0646	10092649.8	842232.65	4358444.508	732700.8967
3609359.441	4455222.531	1191876.228	3168370.231	951473.2403
3208142.548	2879642.556	7757935.417	6818225.968	2575039.003
	63141.29346			
1421644.018	514789.4399	643082.5875	992124.1786	833224.9973
4209500.079	4355663.291	4130529.657	3874386.894	3005690.635
552068931.2	611253406.4	339265342.9	336124236.7	370755909.6
1036317035	1736428964	1706499085	871221357.2	962384797
85597.41633	72895.89578	106532.7609	193495.0981	42863.64251
111945234	160148921.6	149859776.9	126902437.9	134542843.1
25295829.58	32255381.41	18677799.19	37078243.26	18791589.31
56056.40714		58456.84343		
650802692.3	738962917.7	578820887.3	798596276.8	637981367.5
1347815.875	3355554.303	3373447.866	2563014.078	3715782.773
225654786.7	310476100.7	286056049.7	380333056.4	353415665.8
34468964.97	44753492.23	18777374.26	22680120.49	11111386.86
7190242.472	9620827.382	6646654.525	12427897.2	5947543.302
15701503.75	13424258.21	12407142.01	6040945.745	9705581.209
364733.1319	379788.0904	1294160.328	2469378.667	1101113.784
11509118.83	33644250.86	6496711.175	17276853.78	11302022.07
47317243.38	85664257.84	52736233.23	41414490.42	55444838.22
65343.05837	42250.03173	91296.81845	316462.3266	92656.64025
170706144.2	168945608.1	143083864.8	190687184.5	96265966.95
6135497.297	4663555.481	2998964.911	3373059.005	2393288.702
156369646.6	245676730.5	83785920.86	171703687.5	160805036.4
478694.2442	737130.8959	744818.3046		
1226058.855	2487575.647	1205677.524	1804314.613	
3003356.543	2858962.491	4102256.171	5933323.855	3212550.06
1946315.205	1107649.865	1696821.255	2279288.958	1651496.5
155313139.4	114862016.3	114825581.4	269050416.7	102986951.5
995159.5972	1208000.381	1106334.449	817163.9786	753430.6744
991911568.6	733771821.2	525026950.4	605914275.9	691353754
6227272.435	20257232.78	9980873.252	9813206.128	6638675.779
5650601.277	7256821.104	10883940.16	10700026.45	4538767.449

323106.865	674343.8328	509421.1589	1375232.224	313248.8805
993121.7942	2892610.125	840890.7776	5364100.818	334872.2421
63498073.42	49516956.79	39935847.48	43903224.94	43758051.67
588906.7766	946071.6361	4283.499945	1992684.174	217873.8372
	327180.425	145008.9935	1869154.733	491223.2654
38792761.95	48273727.04	21505007.8	90508174.18	38226586.5
3285467.342	8780768.678	5103758.502	13109424.65	2989873.073
4614493.747	377828.8187	2733749.392	2340946.153	284375.7339
130461.489		288357.0145	256672.6921	95050.18088
211344763.6	305708451.6	478100510.9	371706921.8	238819119.6
1902322.527	1243972.219	1071816.166	1682752.977	1150883.095
		315497.9988	635872.1836	
770895.9122	6308353.097	1717930.389	3585107.658	2254468.905
		467491.3716		
83963576.26	52911447.4	719266620.4	1260167285	630994858.4
17301833.8	18480977.33	16586004.67	29141065.36	9728809.964
9739619.642	17748425.61	5474749.572	929272.6645	5852775.614
1610978.485	24226565.32	6578567.652	15800814.92	10287310.34
532600.7996	431300.9807		910482.3316	
1997294.637	4240162.245	2014118.561	2910247.429	1482902.948
895649.4532	1554180.372	999847.8795	1071060.026	1694254.592
3426364.271	2395050.43	1833187.419	1924947.583	1973054.018
17807126.9	18105332.66	11722326.39	24676811.99	15283669.59
2993815.962	8265510.415	3589848.967	3362958.497	8168327.557
1134140.427	1356334.496	2172520.542	745052.6767	1211065.542
2607711.242	7566185.151	3174305.542	4173267.303	1637313.544
246101.0824	185397.6014	131805.2382	242164.3699	119861.4602
15419984233	11336094872	14178911868	12850402214	12548195648
531721.5643		196052.4006	545180.7545	285433.618
2875238.146	2499739.506	863052.9745	1846396.619	1907412.087
388044.4624		24648.83002	14652.35477	
499036223.3	541153989.6	654147730.6	640988173.9	390285440
240582.2035			1545583.466	766855.3747
366145.1113			662315.7949	601156.7763
6596663.391	7276911.516	5504433.01	19993723.41	4546665.132
4409515.163	3916197.548	4405328.917	1065929.327	1654483.359
16849189.54	32071966.52	39900726.9	18852053.68	18589495.14
	470802.8286	807919.7824	882874.4632	507679.1187
3074657.168	1458801.58		2881645.905	862979.622
32803363.81	58044236.95	26949294.74	40374321.73	48880922.36
8665896.375	8193353.196	3890115.398	11505034.62	4643922.528
4112768.085	4890730.029	5221607.296	8611848.909	9161005.888
1449817954	935422204.7	1254130295	1474748945	1710573102
4358186.954	2874833.124	5445254.029	6227366.549	1863343.389
68769.45308	412250.6888	132046.9109	766583.1979	43990.08075
1762480.953	647629.2723	324384.306	1088234.614	
112312242.3	107185049.2	142153004.6	128224841.4	100659714.6
410801.9291	6802542.402	391244.2743		

1194322528	1465176713	1557552103	1008570241	838992800.8
2828834.381	3862548.059	2079728.844	1997545.575	1499806.365
6386899.099	5574293.427	4977008.359	4470863.026	6467956.753
2945349386	2419027975	2644209289	2892165695	3915216351
120841.6724	200114.0264		225285.8615	
3226478.651	2089273.802	5215077.743	6445885.118	3576792.964
4662382.954	4619177.749	3721648.364	5799503.94	1891278.15
4615181.839	2345028.036	2643368.29	4144681.928	3661356.351
1636855.859	2589976.386	1286389.001	1111820.158	1191544.371
274480427.1	316956776.5	218023182.4	148707243.4	95586170.24
20186059.14	8180289.704	98213252.28	43325872.08	19725580.95
2076916.796		1760343.15	2087457.951	2099812.239
239566.0119			394080.7458	460402.4802
43036069.28	48273511.59	38674576.92	58876093.91	97024561.73
75956.43886	15470.09514	55612.63563	1399337.783	2107856.037
152655679.8	173202310.5	253845271.3	195385950.3	108788180.7
	676012.4876	972011.9178	892149.32	
49380370.05	65329131.59	50050842.77	131281445.8	15581390.33
6283373.559	9349544.915	8147085.423	6030164.504	5871433.732
7255328.172	15798346.42	12690273.31	14004023.51	7600457.191
1818351.602	3944189.546	1910071.803	9490585.475	2784828.182
2640344.179	4974625.95			1878546.764
2120477.465	3009417.566	6073437.457	3980042.202	574418.2824
4208487.776	4720765.864	3211468.486	11268771.58	2769470.571
469422.3089	256102.7311	347897.7005	461325.3962	269059.0645
	477908.8993	355387.1188	247499.9613	
96631846.7	53418854.66	151091164.7	141681708.2	127316106.7
			409370.8597	
12988675.9	11978557.87	9983594.037	7040348.575	7225200.968
49569603.05	119257119.9	65692363.24	58651623.16	59490762.49
51139662.98	77296649.37	82653393.37	108886333.6	45303206.6
25991387.86	90741498.27	96124759.01	61679814.31	23629953.79
	6661127.327	268249.0135		266131.9406
88521.19398	2096197.597	175169.0494	57920.66034	42662.38742
1408556.533	1214076.416	1594844.344		1595155.027
		473278.2996		
1457224.579	1050716.386	1824191.057	2121622.582	1582493.664
15716049.68	26252591.43	16070777.65	13424227.61	9895355.631
316852.0902	433134.1036		402967.9082	
677871459.7	444246081.3	398537233.6	493143019.5	619436722.2
24948009.54	21062583.86	27061989.14	22645980.05	29090563.84
	178401.7702	246835.8106	241199.7938	133181.1963
3960877.935	3009722.88	6388886.993	4139874.859	2200724.75
97911.83495	645673.954	562905.4508	870131.9309	96185.79114
18829028.42	34149456.02	27097679.96	51590258.34	19283743.56
13174881.11	5459223.786	13028012.21	19274658.52	9636996.625
3820838.645	11017057.99	7193657.032	8519455.772	4286722.432
	3953725.293	1534417.916	1792172.475	1323911.053

752831.6068	1261386.605	74819.40705	945271.226	84350.78453
1902055088	1588596935	1963148107	1847755500	1187342006
33968220.15	96845500.38	110035012.2	76070945.44	35658402.64
7580491.382	4425233.261	10532351.25	10665758.76	6544062.573
12977312.08	13410551.81	12944798.15	6349155.897	11175459.41
6325716.513	8632369.248	4014891.914	10847998.75	4135441.036
933648.0765	273190.2742	270058.9719	6744972.321	5867063.976
104091.8075	202566.069	197225.6586		
5065264.508	3981951.523	3697449.926	4257310.799	2760220.513
	2870737.529	3287440.088		1908715.917
982934906.9	726210834.3	605422704.2	776654308.2	732532539.2
11044832.43	17846125.65	12806740.13	17556027.12	8300711.847
14648256.59	14664356.34	16097954.31	26771722.72	10869375.86
344131.3108	2889847.933	1010996.189	145075.2539	
346490.3438	382428.3978	1139213.844	1339405.169	778327.1181
2200566.828	4372184.518	2481759.984	2505390.636	1760154.804
2110037.328	3706161.146	1778511.14	1539000.487	2254086.618
24582240.9	26189442.57	17472714.32	22366833.78	18997408.94
10343973.2	11251298.62	3198840.839	18486108.92	4028796.059
13127237.06	142626345.8	13724648.66	26219281.88	4738461.886
			1103080.973	
14224693.52	15419880.15	14328039.91	18739895.44	3682455.55
16665700.48	38589218.17	20284525.83	23852982.79	12625429.07
2687746.58	3150063.523	5984063.494	4329350.673	2454772.509
66452.49432	83186.42682	72755.85871	127136.7398	48429.98396
2674461.014	2040288.775	1436341.699	680192.6902	511847.7082
573630611.8	681720325.4	844072925.1	916202931.1	508365015.7
	1393538.064			
95135731.04	122153984.8	147772912.3	179049135.8	105928165.8
132652701.2	150999950.4	186622651.2	267605115.6	370393747.1
21105463	15832123.46	12098581.92	26317952.52	10612469.07
1293949.426	4492358.844	1426307.414	4158569.989	947902.7602
1276495.669	2886915.435	1179942.667	2513398.113	2309252.326
47191598.8	31246658.8	108834372.3	110129918.8	49583199.7
2026510.451	293044.2975	6170027.5	1975199.166	2234426.265
3773984.269	2359168.729	3071065.185	2777697.205	2583346.217
1467138.315	3459612.659	2830864.2	1577681.248	
830494.6509	103284.811	53378.12528		1047307.398
71578191.36	77069422.44	85992488.96	88172782.07	40675875.59
42897113.15	18666089.98	57372409.61	3636472.899	11235830.25
321875573.2	688405663.7	243339019.1	212008964.9	287131787.8
2664755.858	3279487.993	4173876.665	3355944.248	3119137.908
1514890.506	1200182.69	2369498.116	4033396.95	2116607.48
3122477.979	4607491.746	6072743.208	3336065.51	926655.5728
		200362.1647	307693.3685	
14281282.85	22906782.19	12667897.18	14976853.99	13422679.99

971188.2777	1285587.281	487423.3024	1899399.094	1123511.485
			772228.2796	168200.5534
244583.4873	198685.2145		1118013.024	782167.6678
8365036.554	8215513.634	7514625.684	10241635.93	4321945.083
626372.3972	905942.8059	595953.6777		737994.6407
18523688.82	13672858.31	13954430.97	4266709.302	6098550.058
			5938.471451	
656439.8177	388134.4445	879206.7319	1074793.19	
1446143.039	2872794.916	202205.0546	2054418.875	659825.3584
142366.0636	149838.1369	97419.04063	1284616.86	42597.92296
3121103.372	5229461.443	1859691.116	4930328.581	4668579.873
1749803.113	5210732.168	2395258.666	1780214.136	2704029.957
3237147.767	12796465.76	3900926.686	9430317.226	1582075.113
637586.6054	1470849.904	1272754.407	610888.5712	
304904732.3	223461344.6	264319190.8	181695996.7	234892954.3
977624.472	782474.9305	1176522.558	1741309.701	1506202.057
22031370.96	23590840.14	17835101.03	33031491.45	20591824.03
1312997.999	3397793.184	1479881.624	521095.7864	1547900.765
8493084.211	10018661.46	4114175.875	9087715.989	13943751.37
9757472.84	7880001.087	6355055.688	6791821.485	3504176.025
1215755.317	2563486.385		868353.6287	415155.422
4104499.664	2722343.155	2768037.859	3390888.192	3308465.372
		371129.3175		599767.6053
1284703.035	2310821.946	912225.6029	742759.3784	839452.9023
2034457.218	2156908.724	1996357.989	3219357.435	3795910.836
	299854.4636		35678.06773	
12054250.41	11473161.19	24498539.46	13894097.15	26223229.48
469430.9335	541556.2734			
71187629.54	94056171.57	73179180.26	102067424.2	24213273.37
527743.9863	798327.9405	470685.3723	752590.9301	685012.4481
155404.2631	148663.9588	505308.0799	2045421.903	299131.935
24109.86794			293066.6819	
1530271.986	634699.9672	3209642.616	11757730.45	3332969.448
497705.0199	649207.1266	378707.2056	402965.6672	143746.0432
33959379.91	22573705.38	19529072.69	13603882.08	10632616.47
4550301.386	5594424.618	4589155.263	4517991.054	2119542.103
3354909.441	5991230.025	1562144.149	1763371.323	3091039.665
1293383.268	1106633.127	1824999.11	2222013.13	2944661.98
487909.2007	695915.4849	405897.0983	1840489.18	449123.4773
370253.566	407622.9598	155337.6635	198525.7167	177447.0289
3547439.337	2535817.055	1872633.755	1386398.042	1850921.037
475340.5574			2262875.815	
599884.9172	5797963.65	19839812.88	3104898.692	
28046302.31	1487788002	151207755.4	488389517.9	20770406.2
18398425.67	28227122.2	25268693.38	46960442.64	22535326.34
8950476997	8727829100	11887651354	9027413808	9076440845
2035682.002	2093328.916	3060698.649	2013446.95	1922703.274
27309432.37	25713841.47	19000222.3	23124757.14	18088913.91
3015048864	2211114711	4373088135	2990738888	4439098317

4467812.38	4335993.567	4387287.567	5452045.02	4293828.84
1064055.238	3032969.528	1051838.675	1069706.76	1502347.2
1820136.653	519643.8084	1440892.027		
3292935.163	4642877.214	3207651.827	2626750.012	445592.4029
13307390.63	10441058.78	6551983.56	28294524.84	10283947.22
	970477.3188	471733.8813	475960.3887	
48420094.5	41092454.71	31802411.34	104812584.3	32274114.15
			489396.2996	
8916414.747	49791953.59	8033379.833	7938535.474	7844808.087
76487850.08	108895919	44392542.13	49925795.84	60030098.46
12461122.05	6661668.197	6290265.849	4064315.108	7968338.127
24995888.85	32988577.88	20552518.88	32364831.78	19474622.14
7886585.801	13044685.61	9725848.242	8904583.904	7402511.754
877519.3737		257177.9793		
3752183.939	3341256.858	3714762.421	4618467.551	2876241.424
2599883711	2216688276	2750354463	2490643613	2284824336
108733601.3	181705452.1	95530728.02	84570207.59	84958235.42
	894379.9571			
2832332.694	2107565.832	2423255.352	3050050.978	2817500.171
628504.0337	415767.5956	601016.5006	1052230.791	678849.5545
1702811.003	2208393.533	968640.1514	666645.7783	3138621.985
628220.2947	24646758.58	1013280.385	4204053.881	4968829.797
20646554.8	25682658.27	30680075.63	39422072.63	13640200.77
1399434.421	1518931.062	1567675.993	1684218.903	730027.8983
1655840.502	1908406.571	1348668.797	1626145.503	816059.3469
1829615.956	3905124.391	1683681.548	1266877.04	1036127.791
63357305.34	73981679.54	65755019.95	57550248.8	47677314.65
904847.3844	454490.1123	3791573.801	3953321.212	1506915.503
23371557.02	39001665.76	12885846.73	15959403.17	24351486.42
596117.5337	705127.1503		792116.4797	421576.0322
	42665.3374			
5353804.113	5826192.779	4751549.081	6759525.926	2836052.858
12051.31757	270249.2028	120399.4423	1127987.858	390761.6704
432812.8311	314127.9901		401126.3331	239346.069
5881219.223	1770515.899	2077155.35	4987890.476	1094482.751
10329135.23	15072411.31	11156147.1	20858234.66	8454722.447
		641567.2533	879578.4027	545994.6863
203782.8201			886682.3629	451185.9516
24427015.94	20808128.14	15522823.78	20388536.4	15483787.23
309661.3871	354339.7214	454874.6583	422230.3691	192199.93
70738472.74	79552048.84	89131005.81	57598690.57	81328365.29
360577.077	406055.1686	282736.5055	510930.9648	290411.9044
	2207164.774			
166769.9756	140456.1842	246460.4659	660910.914	167262.307
26507937.57	9396857.013	26988627	5751847.657	5229501.389
1241839.846		1714846.672	1281229.132	516576.0956
			1068910.864	

1740441.397	1610301.898	2697166.74	3433552.138	1981853.725
6305661.263	9652608.145	8116119.033	11985283.55	5099030.311
53378568.12	14091100	12443850.52	114541669.3	16555546.92
15193890.48	17227611.89	13624137.98	14214563.73	12783527.77
538730.7507	668301.8611	875555.6117		1365802.466
58269047.15	34548086.85	51877170.78	49185830.94	60234016.04
53102431.24	42816133.06	56624091.23	88686948.95	52005277.74
	104686.62	103193.5785		129295.1086
	229730.3491			
3342718.913	969250.4768		2740216.949	1226700.369
384762.4792	5443596.566	1196024.442	1838392.806	
			610676.6359	
35720053.35	26524099.6	78308459.9	132579925.3	38029714.25
	695543.34		1168133.099	
174448.9951		2108225.613	2077135.441	2277559.32
830618.3758	897396.9858	1173588.402	1682428.493	888700.0743
396340.6396	472879.7673	11309.72345	396999.3432	185986.2948
28340913.56	32505132.51	29645828.5	28492388.61	25093212.92
285373622.7	724948120.5	676666562.8	262721130.2	364588634.8
7831681.187	18076147.34	6896657.448	7316178.152	11318429.11
8719996.088	6714080.59	8881630.389	4666695.176	5340612.13
140696308	119729751.6	96158645	137432483.9	103847599.3
	201886.6146			
		390396.3549	921787.5418	421323.5037
244986876.5	310447621.1	320029701.4	370780006.7	221327225.3
62175511.22	71319101.39	52506999.56	64565213.21	40257027.92
995570.5605	1047639.122	783170.2228	915941.6409	1278837.677
889249.2654	1758577.647	2518041.947	2912433.515	671210.6668
1190098.577	48481.78519	2858488.188	2005778.114	1779173.79
17595274.56	10391167.35	10038467.99	15263280.02	11638989.04
4326561.514	2736555.847	1871990.033	4547391.373	2718103.66
14467434879	12071027038	13702313517	11429672616	10600686669
11775223.93	8249924.366	6324694.575	9363818.12	5316221.238
2056983.662	5837302.499	3966591.028	3266777.309	3747448.108
8944306.937	10245403.85	5088424.689	13789498.19	6145023.723
770912.313		368504.5453	2054719.241	154780.0353
3810933.263	2951476.783	3485653.24	1918707.193	3351665.657
11981314.53	7042605.548	5813890.105	5798119.637	6445267.93
108990.7539	71103.07342	218607.4732	197199.5556	81515.31033
59186458.22	48474616.67	14898715.09	89734951.96	45829481.91
	1655781.019	500984.3172	935170.8423	539950.1688
3124894.792	3515716.742	1652825.787	951851.2705	3218036.721
1318760.817	790161.5491	879923.3485	958756.5636	1241375.129
102789340.6	170738581.4	86345513.67	98040559.89	125192353
436185.6993	421757.5668			
4298965.764	5381660.527	3483382.847	8121920.708	3495958.527
93351251.89	104346243.8	117059545.2	125294865.3	74779113.46

7864937.814	9435890.901	9507698.421	10038384.05	11336752.97
598144.5442	314451.8798	1148364.192	1808736.442	273978.9746
1117029619	1862357290	1740259039	990851613.9	1035457410
10908229.98	11653807.05	7193373.971	17195387.34	9228157.078
	2020985.2		647912.2843	
195409227.1	351002265.8	147772642.2	328656193.5	289428778.7
187989.0104	555275.9911			
			250914.8664	
886605.4329	30246345.54	3642540.191	34400558.42	
	4116.091119	2611.807855	5521.86904	
1551247.02	2000594.637	1293569.819	2948439.97	568824.4205
5088536.671	43264589.4	10918589.66	11923646.51	10777281.2
36269171.27	37666727.4	38852514.85	27446973.82	23631270.48
1129277914	1003714244	876677445.5	641153344.5	660930885.7
	784647.7087		2145738.747	393522.1537
1295290.335	2452510.883	3396625.053	2122335.312	2472753.662
19369109.32	24889201.23	15867823.45	14256712.19	19042160.97
	91470.99435	105817.6224	215040.9861	
1074095.655	2027918.478	2208776.051	2341077.181	1229884.221
48520576.53	41981055.44	43507113.16	64772161.28	28405694.82
85189054.05	153982075.6	104663331.6	199302768.2	130788199.7
3100753.101	3320291.893	4304323.348	14469001.29	6574397.695
2238968.603	1164848.963	1448311.397	5799324.357	2051095.607
295351.2398	909517.5249	253766.8689	978984.1519	617909.5578
3385370.069	2118523.838	2721527.206	1011379.276	2725264.015
			11601.65462	
169957730.3	270674632.2	191716184	299593447.5	204241124.4
1377494.966	1051737.461	1466931.464	2360447.743	511236.9283
3075751.499	12164495.85	7405235.304	4875668.899	1616337.061
5997978.232	8316952.24	8977251.992	9710320.36	1775907.425
952988.7303	1102022.838			
969120.6422	2986228.584	1645891.142	1201985.27	695047.6558
12029186.25	16308472.15	11222062.4	11404645.37	14849998.22
2510523.453	3371046.357	2713216.518	1884343.93	1609932.563
4024971.62	4912870.544	5820704.109	7070767.166	4823730.883
16988688.12	11085398.59	12325172.5	16319848.23	10698024.83
1102277.935	1565366.475	2432349.226	750291.4423	
915915.4107	4971487.493		453950.8431	4850468.561
546513.5979	469009.3653	658324.8446	1473072.98	1517235.193
23665511.91	54545672.49	23375529.3	9367035.113	17207462.84
2696734.967	11489017.09	6076147.873	2871635.931	3530372.061
4660590.379	6316446.109	4405642.523	10100761.98	8298369.235
2596029.611	3252813.19	4097045.449	2247067.957	1786292.855
5962157.875	7081027.859	6509959.219	8517919.97	5355826.977
4022507.726	4273971.392	2161028.418	966362.1895	2121123.062
575740.4936	2591919.988	830466.1602	665208.7585	379557.8437
984918.7288	2489117.13	3346295.644	3404649.892	2291108.344

		197845.3605	5359573.697	
112637026.1	95512506.65	58561686.28	158854866.4	78991672.42
352556.7889	1122663.225			224711.6322
				1275395.384
927381.0511	1156189.717	909475.4514	1007768.585	507978.0763
1913561791	680117423.7	1910549782	1352482071	1122826222
1102668.539	4859559.417	6688073.413	5773766.886	3952653.481
1271255.769	1892841.655			
4601587.885	5998934.805	8823432.141	12024162.07	5079927.71
	1824014.297	417842.7885	3732245.796	1084386.979
81747105.31	61398633.06	43928048.84	132246810.1	53012362.61
5077277.926	15869517.54	3757748.279	4917983.579	6440001.309
229132.8461	869834.4924	312358.1887		670493.1402
7535578.702	7884535.879	3405328.574	12664189.99	5427786.76
118913.2318	148727.2129	461949.1378		
83474007.26	72863169.61	53705677.52	56383626.56	51928155.37
524588.9475			1426568.86	
634040.256			305056.4845	330362.7304
5608393.697	7164088.898	12318015.97	14177077.93	8158622.591
1428428.023	1824161.375		4159327.361	
316154.7251	2986471.345	256463.7429		342916.6577
541682.4286	536565359.7	353698671.4	379072063.2	373674.902
3158003.541	3670006.518	2483432.542	4453523.155	1364309.53
71766.0801	61194.93615			
108116822.5	102607100.5	196077332.9	186058672.4	118036338.7
			830740.6461	
166499843.2	254916232.8	188144969.7	374375339.5	288534519.8
15658486.35	18530676.32	20401459.34	20883840.64	24927363.16
26228199.5	21811645.08	8607308.91	33599665.91	19588777.5
79613680.29	107481714.6	94075833.88	74556619.75	50001557.77
4894198.867	11274023.75	3500758.977	5707401.209	4761177.757
1473983.964	182603.759	175311.2533	2400136.78	519337.7789
			697904.0711	
21670798.25	28943639.21	20517040.72	17954931.44	12613824
3636860.426	5394983.158	9361206.123	13128112.55	2776686.09
19235178.64	14554051.41	16315386.28	13741541.98	10109633.78
4840368.725	8453242.799	5498262.814	4972457.542	6358281.753
2982191.368	5058446.781	2706587.322	1915349.38	2587446.248
310080.6451	392581.9023	270445.0439	372265.1364	162771.8982
225689.0255	277936.7274	264209.9846	212841.8991	118527.5243
403733.3132	1445139.079	1030573.827	1462000.55	
14446390.73	19100669.72	8446439.908	6828038.079	8659621.87
419926457.1	385887769.6	590479638.1	578055017.6	446990636.6
1211282.945		62180.0872	396711.9295	66795.78854
	398243.1439			
69409595.58	79914824.55	81347081.48	73538955.67	67398999.27
56341898.87	94119064.1	55258822.64	64904173.11	54360124.42
473390.4612	757302.1481	1184983.623	111494.6542	115499.442
4861036.45	2826736.015	6961416.311	5132660.846	4226039.464

223225.8378	317040.0505	328116.3859	443684.3225	262542.8076
12509026.56	13421818.52	8643921.472	6831679.273	7407755.698
139049600.5	162102260.7	103775969.6	74233114.81	65973944.07
165596382.9	177478932.7	122587430.2	76657982.93	80935372.78
15811687.25	15429125.27	19863953.89	18459952.87	15739690.32
8347156.799	7106415.274	12583070.24	6519489.055	5150514.804
14399850.84	28866694.34	28953196.71	13734307.58	23293196.78
3609306.893		1570968.875		1157943.444
			1815064.053	
6251155.188	13158116.96	5715028.55	11268043.42	9651357.092
29441574.41	38770065.94	21346852.55	21821914.26	39575734.05
601655.3113	496211.6159	661538.9846		419887.7447
2019347.719		3324707.501	411279.7362	
77437.22634	162135.9673	64431.41117		776393.3962
58130802.97	123591230.4	64126220.87	91483745.17	53614031.29
600300.1221	711084.5166	350518.9512	243691.1723	695678.0681
171575.0915	71605.2786			
4965287.164	5331262.742	4758317.329	8392154.665	3823746.532
25071039.58	23047031.02	18758405.02	16949963.99	830096.486
39473855.81	33552614.49	31838992.4	42144936.17	24331941.33
			12539.86334	
1009848.707	675055.5298	1853824.761	1267868.49	1690519.924
5329706.642	1889191.438	2751672.948	4023789.321	5508186.992
570437.0876	1502446.02	2709768.759	1928578.692	661440.8697
30626604.7	18543728.9	21474753.76	11695082.39	15761117.79
2809313.648	3622798.165	2410357.143	4527135.825	2941990.155
11313486.59	48136578.98	38448762.89	28466014.36	8573284.735
1605687.144	3539041.451	3018744.769	2392266.502	2585164.175
457129773.6	615946908.8	497231780.2	325737313	451939720.1
36318799.33	78272509.08	50041635.53	46211272.01	49564561.41
	2848301.493			
336226.182		226159.5811	1190305.278	689629.2359
228709.4879	180750.8213		1190392.499	356595.5806
594346.2442	726041.2547	1376328.558	4403944.585	398860.7917
456588.0005	561210.0879	450830.9559	85560.75659	756265.7691
	1619682.097		292836.9786	
		546663.2344	401229.206	403819.984
6299927.695	4651372.79	10482071.14	10410279.83	6440903.3
1248531.384	1734696.063	1121245.118	935906.1064	809100.3623
77196741.25	53316812.37	63432961.07	75134747.65	51111187.89
65604794.12	76552587.46	36708936.48	159296385.9	60263169.35
1683362.224	7764584.883	180256.1527	2058754.888	786481.792
168852.5539		258606.1479	578303.7795	280497.7903
3556729.747	56815572.03	2085675.583	2003925.511	20102834.06
282233.8993	263257.584	207832.6499	336898.7911	174079.5245
6576872.444	6691892.011	11267870.43	7565419.199	6979855.62
161834.4013	595873.8121	561926.696	156634.6762	212408.4686

933053.4993	1278276.844	1326855.719	2131522.218	234983.6978
311606.6291		439766.182	248381.3889	187829.5648
13092464.7	15640913.59	14354165.65	13164391.06	6194280.248
		11967728.75		
3200319.753	4005426.364	3719540.564	4729525.431	3336492.916
1656958.987	1458386.547	1267787.009	1674268.342	1007073.682
	441194.5923	207111.8706	136624.5457	186299.0414
5272152.575	2597596.948	2072069.051	2406636.7	2368881.645
79767354.43	19004167.13	66192141.2	59947574.07	77436159.42
	57093.53693		249334.8312	
	2474.117013	3288.096637		
	1196751.886			
1993854.846	4922639.852	989476.2571	2014394.185	2830477.768
		553281.9528		
585121.7677			247601.2157	858856.1911
11437544.84	9078755.732	10590416.12	5381430.232	5803868.764
162367.3575	4451898.744	340809.4398	1478141.358	365242.8174
		758167.1222	1111343.321	
21924303.79	17061879.25	30675433.25	22247921.33	14094022.2
11698470.69	7501034.725	8702391.259	6238879.053	6707491.627
77829.04945		250112.8034	263059.2096	155132.181
850573.1889	616366.1028	972380.9262	324894.7422	
257904.9781	470247.6302	71810.81271	293999.2259	282846.1281
20490381.44	6978277.88	20234682.51	22070943.82	8357750.014
		566335.8419	497882.2503	
2977688687	3074049574	2129317631	3380678266	2365542074
6049227.759	36482970.27	10551955.12	15405840.3	10162805.96
2348529.415	1149789.169	1164666.554	3058163.712	1516989.486
175688.9779	108860.0487	140410.2025	232906.961	109302.9927
5637767.699	8461174.767	5815655.068	11856216.94	5336020.737
			357926.5973	
	1963949.562			
		912974.6622		
	435795.1843	276077.7093		
4711337.354	3208415.72	5484096.596	8100892.084	11317117.94
4779888.082	7148048.857	2999549.681	7001143.526	2101646.856
	495077.307	441501.8865	1959266.31	653943.0167
4350818.623	17345997.73	23230822.29	23671243.33	8664042.498
	1267605.387			303466.2121
167063.1905	264683.3649	82144.37899	190770.5124	198445.7467
1522491.748		506926.1		1542211.904
187607.4555			306753.9592	
555406.859	619613.6016	636980.7764	849510.4765	700953.6798
1950327.941	2032656.629	3272454.665	4600823.41	5627481.808
			918903.1865	

2928088.848	16933798.38	4176419.76	2197476.35	6809849.528
61778304.78	79567287.11	69441301.75	81365931.01	49233071.8
4646209.521	3614800.906	3814990.368	8765090.788	4437586.124
262644412.2	438463133	172157076.9	200879783.4	289481004.5
6556592.914	3767609.506	3382051.961	5861317.916	4864276.677
33136384.9	30188181.51	48314987.06	74898662.72	39232296.49
53372.3031	71349.39901	7255357.382	17827067.14	7727202.486
22650278.42	67352176.79	25493634.64	47036699.17	69864089.71
3190167.451	2978863.89	3329640.249	2932413.063	
	4038687.812			
35630053.31	34779648.56	36291762.07	35339309.69	36033124.44
806024.4846	1186263.53	2030597.799	1873722.174	459772.165
4140445.803	3153436.188	12239328.67	19045042.67	9915530.129
389729.3618	253216.6431			
83602719.53	101446055.4	79803871.25	140183254.1	83502731.19
921534.674	1320923.894	1215549.716	741922.6642	1243135.134
	401795.0771		653642.88	1388113.061
17151049.67	39198359.94	44559875.21	33431682.38	20610090.1
			626306.7072	239750.1321
540283.4568	712599.3759	604115.9016	756441.2666	328836.3011
18853.30802	4007.458123			
5065036.687	4681915.653	4591284.104	7465925.41	5219190.676
2909992.018	3959885.092	7498219.027	8243327.555	2129696.345
6532957.811	5841608.186	5320917.765	5914782.252	3958074.001
61129453.82	87614604.84	117476993.1	95954920.85	52541850.75
5781030.618	4972863.377	1515235.088	6046557.007	4476111.144
57393830.23	64399541.39	68902371.58	77730362.23	74409697.39
14131862.19	9065737.901	10723407.07	12570658.29	9383059.389
	12915.32297			
194329.3447	302928.5887	824261.2611	770673.4112	468653.6391
7204514.421	3593740.143	3439999.929	3236621.166	2993297.63
	1715793.247		1290143.969	
6134965.731	13060128.78	4947102.858	3095615.481	5112977.892
5695.621926	14026.97329	7522.189595	2102.388687	
4233850.409	6984412.929	7076895.656	12996878.71	4300308.461
3488323.492	5633955.337	10649179.35	17104888.53	7888769.756
35083522.13	28819684.16	30263499.04	38341113.11	29584079.22
			1596402.843	
279213.0363	265128.3		323449.6121	421781.8314
1349229064	901908411.1	902647592.9	1538123417	2083255894
17399964.26	15760534.37	7407445.832	12069322.49	15663388.19
336090.9694	332798.1794		304702.4764	
1492803.067	1453589.371	2488968.885	2117513.818	2664372.406
890986.7164	445654.5333			420664.9887
			858908.0551	
117458.648	85219.49495		1447478.106	342870.5638

	1879677.998		1269457.899	199845.506
	866408.4205	1220943.79	667653.0226	
437874.372	4940584.243	3879934.682	1129426.354	1104069.567
22819327.94	21077955.02	18173291.01	33456185.58	31637529.71
21051132.33	3589735.153	8135663.237	5801323.638	2801499.293
780437.6717	973087.1373	113476.7963	1185894.369	226938.7882
2690144.99	1063962.034	1772416.154	5276654.049	2888599.893
	319943.3417			
	298546.3736			
1544603.2	1286828.355	15600.88334	1182251.308	1008031.648
6315603.282	7207678.566	4738829.03	4075965.504	1355587.4
		478229.6549	779980.816	
3618886.473	3422465.588	392881.249	881620.6014	
	1703536.136	1386039.852	928338.1152	
4147976.988	3704937.73	937530.4141	2747726.674	2399291.503
11802062.75	21902531.48	15252353.08	26730552.76	9838050.707
9880767.779	8917533.451	8025183.308	6601197.692	8605709.667
		383725.9022	320419.8046	
119233487.4	175397973.4	104735774.9	100692614.5	103102001.9
1056769.464	1144163.828	1600200.595	2281305.403	924785.1463
810145.5699	12430480.48	1536604.512	1311371.829	6985941.114
5863267.762	5759513.593	9657607.774	16573797.96	6150261.146
77347972.35	97279077.29	118104013.9	102238597.6	95969960.02
	1260821.577	877294.0551	822272.7657	1115973.114
563150.2653	358552.588	239655.0804	683994.6673	230399.3452
437630.9517	775407.8186	620885.9156	770654.4516	881412.8557
253879.5761	317563.4303	300135.7423	522556.9948	169670.8829
			1493258.985	
1244829.58	577464.5698	2168433.366	1884414.077	
3537464.004	9333900.834	3452089.584	14036609.48	6162103.715
	5663421.06	5350693.847	177465.7361	204628.8284
3586635.244	3761720.31	1991799.466	3681985.817	1016059.811
1796576.392	4357971.107	2118349.52	2173922.732	
	101883.2416	62178.53844	18839.39944	794390.0081
3674935.328	4534515.111	6030077.048	7608280.471	2846162.236
			486690.4067	
1070761.292	576313.5849	1755883.639	765168.2503	1002348.841
11104285.44	39558550.97	12836903.23	14232873.13	23476415.69
109397.4668	36751.26506	73351.351	202297.1288	208872.0824
850294.8461	820328.2903	753465.5692	1041103.225	471175.0498
249155.8781	130629.3294		321477.8451	200201.9399
1989241.264	1894999.003	1449710.667	1099361.113	739514.8935
262326.8513	309068.8843	780289.4533	1587356.996	802476.8118
176060.2469		98652.21684	588650.0871	
5308377.505	2138755.744	4157646.537	2601892.978	4370111.765
2409556.852	899510.032	456040.4171	239802.8809	2049245.779

1171216.695	1786486.432	1457907.009	2233272.915	1335376.708
258278.2501	437273.8686	785196.3128	1839917.189	1069660.813
	979911.7631	806523.0457		
1807841.499	1690732.165	2490985.215	1702122.224	
20933392.67	37128490.78	28872571.25	30856110.11	19522170.31
587905.0543		926144.2794	421375.549	338850.0501
370992000	307485739.3	335076194.6	280657984.1	347202634.6
426464.9092	862084.2363	648744.1795	420045.5284	341035.2063
82866.43679	46605.13618	239093.8729	335505.8801	111314.8378
2593835.623	1416638.029	295770.4182	1389464.595	1724865.837
3123024.72	2160794.238	3171913.682	1340346.393	263567.0389
197950.5817	116435.4096	1849597.51	3844747.534	2850743.288
7864.32263	3447.366234		130849.69	26818.66855
3061955.008			2484869.084	908912.8657
7199124.887	7374646.379	16340955.77	10568963.28	6336462.962
2231435.32	3858731.968	3020485.56	2676191.133	1669288.795
208570.6839	131324.8369		952395.7999	172858.4587
256094.607	1598513.347	1793080.624	1464511.831	298812.5951
739898.6048	919903.8416	847069.1901	605895.2672	606670.1194
12197505.07	60207864.79	19375839.96	12080023.2	17789650.41
413945.2328		556885.4797	394534.461	403448.5149
1400461.876	17506.97438	38770.42196	2695372.74	106342.1102
1374910.563	440852.2295	2348907.59	3264769.971	2262474.25
15489522.48	13154139.67	16630923.95	24061403.51	24213382.23
16937262.14	41526620.89	16220940.05	31319741.86	26623163.98
1811588.24	2789405.987	873642.6632	2630920.493	1689645.904
23592658.68	33654969.21	31969908.02	20851383.91	15322771.08
204009649.7	121852089.5	157932328.5	209574898	159409050
	191584.6068			
1532984.886	3663898.712	2040311.592	2526107.333	2599704.342
	628327.093	707161.4941	1016746.633	765463.8567
		323714.8479	337183.6482	
	601727.4995	155342.5435	428577.2954	156300.5529
1632555.284	1099272.818	716254.6729	2224446.865	821500.2667
904687.1818	3261288.208	576613.1255	1703662.614	758178.1381
1288213.06	5137546.005	340592.33	1401316.811	4438503.256
1525380270	1311511802	5042058184	1472275459	3015992229
327877.3825	11327830.36	14540020.93		159409.229
18020300.75	23255296.37	33538824.74	35979786.72	12901391.41
	2185858.974	847373.5959	506407.4721	864783.815
750101.0986			361073.0082	
1000012.829	1268715.401	662994.3027	1764779.957	
1901106.161	1402071.568	1557335.106	2507094.415	1197824.582
	439346.9018	200538.5827	285393.15	354967.5752
4424526.822	8782098.403	8455404.267	6315094.583	8777826.9
1222508.394	3867852.298	3560476.625	3044566.555	1091610.915

4371082.742	4920823.501	2353395.777	3540570.877	3870277.368
3198835.007	2979667.217	1675231.656	2514567.066	3433545.148
5047021.625	3712511.618	6601281.666	7371269.46	5260804.608
			407648.6272	196291.5081
1144000.23	835628.6896	458406.4095	1310686.97	612325.5561
220854.1743	222924.7115			
			672522.2708	
30225.29313	34411.42654	26401.85354	186373.2809	54296.88452
43928667.71	50673189.73	57348440	43848435.69	27432077.77
1233240.721			1522758.194	
27961842.05	21187725.47	28306383	27074547.76	25462386.32
16214.42114	124188.5768	86112.90379	120807.3502	
2343273.138	4971421.603	3294941.288		730236.3887
343109.5934	381259.5148	232754.1931	653526.561	274788.0714
993190.7702	2472230.395	756540.4508	2019823.001	794116.0563
1081274.647	376460.3738	426684.8824	4453333.879	54720.99463
		26980.21702	318657.483	75561.80717
5929815.114	8195198.119	10230588.11	11478229.21	5152784.948
478247.2273	454440.096	251305.603	482251.1119	345477.1653
6274822.432	10364325.19	7839408.191	5596950.669	6017449.434
1015705.217		394458.3165	1480019.821	
354096.2989	702460.4151	313783.2054		259326.2026
79578872.73	12761462.23	15806862.47	52436809.37	12946457.37
	9076963.628	2877727.918		
622054.2192	807714.2734	239594.4775		
7102995.765	7436741.776	4634323.315	4575983.293	5517975.813
684505.0881	501522.1327	254443.4785		
627985.3059	776662.4825	502806.1416	80399.77157	728126.5289
193623022.2	41271429.3	132005673.3	80992809.8	142319406.8
3222617.717	981706.8377	3896713.021	5822447.879	2289623.085
881355.9541	919005.1602	1436250.816	382925.6832	491333.8415
1848565.275	1807162.639	2653989.976	3836078.319	2654071.152
636383.4066	689782.3773	459675.7235	1031536.824	737465.0898
10332032.96	16553546.63	24323598.86	21935015.31	16806781.12
		722159.8505		
9733.012688	6352.425179	21836.62189	98158.98397	19202.44175
1749883.326	2120901.101	1770640.388	2866457.419	1214961.119
224142648.9	165880544.9	254500688.6	152241336.6	149472072.9
923421.1464	1899882.027	956571.2121	1260315.226	835793.4108
538650.3018	1161607.718	386061.8106	390394.2519	640954.2676
569160.2854	1917574.246	2387857.522	2027006.33	511035.8761
37723.1347		93104.32049	179164.0467	
226638.0114	1444312.51		830607.3239	244148.5384
33989789.32	28187563.36	32622082.15	17139350.76	27124293.18
69032091.62	33374960.17	52634532.99	51743411.05	41648194.3

12908555.36	8883936.445	1964050.866	15884585.4	5721410.663
145407842.8	26552627.36	35337908.31	89346704.89	154854518.6
3327827.761	2336126.22	2387863.152	5284501.348	1951482.126
369044.4998	276109.4507	216149.8808	383885.724	
932123.2	829194.4062			622536.3161
		3354.513555		
2870026.571	2670683.606	2065458.027	963007.4359	1526193.767
3727812.637	2561650.028	9937154.54	10250229.46	5812441.248
2922773.031	3779583.527	1553938.465	4409335.988	706842.4295
	2889120.607	1187134.418	701916.0448	855819.2148
5610990.483	8910452.739	13402855.73	14786243.2	7235097.1
47820366.39	57050387.56	47200912.12	59837030.14	34942427.46
12480218.06	22832849.86	19823898.92	24803256.11	30850345.57
587628.1254	1110276.827	743981.7754	993561.3229	405472.307
73044.42574	81505.64687	46388.09432	171549.19	39409.59721
1367367660	894429926	545363299.2	1066040030	1126548873
2175485042	1738544091	1988275296	1057518245	2150666309
227571.0442	226353.4908	313967.5139	232201.7371	115938.1462
42984123.26	47436888.95	39478846.84	59890785.6	97858108.18
841418.8872		558818.4263		
810946.3357	1806970.193	1408646.158	1297099.382	1403920.952
38764353.65	49711311.29	46709468.83	77499356.34	90169166.48
6857083.433	5168393.907	7823559.72	3606974.046	3355719.956
412442.7189	585398.119		289499.3349	
4847325.086	4508369.797	4603484.694	1945910.72	4454090.109
5094.542079		19226.8524	121711.2057	12764.76364
9767615.244	22666102.55	15027340.42	16396922.76	8864685.101
746767828.2	769157649.7	678426307.2	764709902	650128379.1
3616668.555	3005786.75	3150629.793	6225229.748	2526618.063
12534473.25	12112192.94	20688090.62	23618429.71	21225371.95
3978468.342	1237817.643	737214.9624	6346718.178	1529419.355
230951735.4	305008598.4	349145461.4	200687203.3	312528587.2
	1300533.457	890490.6263	736057.697	523762.7263
14640763.34	31313723.68	14167924.77	45781242.28	36481765.9
966455.8138	641822.69	801132.8626	2185491.67	723774.0963
6549445.546	7425978.468	5039537.783	9453333.873	6541166.208
658071846.5	533499078.5	558188846.6	1645659829	1332030322
10404526.68	13656290.48	10546259.53	14797396.88	6168809.906
	368895.136			
14189091.44	11940402.56	6767263.187	12165124.95	11358284.06
15379172.23	16861492.38	12685620.44	25271300.54	10326883.44
		475341.886		
		545361.2712		125224.1069
5017275.305	16735218.95	28177814.88	3442099.557	4879958.639
2877903.74	6989216.91	2601748.944	3917248.06	2632729.13
88309859.04	127045188.5	120046478.8	90110102.7	72329365.77

225901047	251524245.3	293920951.7	254934092.5	447465537.2
7172899.161	10731309.21	11125924.72	10059513.48	12649894.96
5484973856	237691.6858	3416897992	1028245.468	3776054443
3682971.757	1184787.853	5099057.123	7894037.358	3279810.079
92592368.76	115230596.6	44087516.65	80669411.2	50566030.23
508903.487			1110766.315	462500.1073
16558097.58	23797853.81	23994999.26	18680822.07	20410996.25
166023.7409	131876.7888	822079.8707	1591171.334	314822.9677
508260.0608	348690.6274	168686.2529		191444.9786
3430911.424	1978384.878	2363882.215	4235717.085	1144730.26
3448202.305	2405277.659	3425376.102	3701966.005	1964892.815
96693762.38	283286787	38986252.47	50740203.76	124423770.7
685665.1168	1262696.965	670195.4006	1191893.975	707384.1722
3700030.175	8457503.675	2298058.105	9837621.342	3014927.563
818736.7294	699183.2179		1337039.287	
13816532.1	21975279.77	26296126.16	31539840.12	13422133.6
11678835.87	9328394.613	15304478.54	10891691.09	13347895.58
739771.6398	1139475.014		820036.5241	626383.2187
2676170.187	3280228.641	1309164.118	3347203.036	5056569.572
		547016.766	610295.4511	
1910756.276	2968796.857	5292158.935	4124901.189	3263032.522
7587114.331	11356138.8	7454983.323	7150987.253	4612093.478
13257342.86	22872885.19	22091025.13	33998086.9	18672753.39
		128533.4406	180401.8836	141156.6298
51937291.29	86805179.18	45636290.76	47173508.95	30414721.72
338392.5053	180287.8205	365235.2692	819452.2969	
171896.4983	905578.7834	127996.5351	2246707.048	139038.4175
1192317.072	1088115.802			1591478.312
5762309.537	4690017.928	4022110.097	5042812.285	6412241.183
	876707.4333	385730.103		367711.6665
23165298.1	17937683.14	31127416.35	33981291.63	13806910.54
2930820.39	868496.8779	1709337.375	823540.0113	728575.0305
63150435.09	33979716.49	56661730.17	99096363.66	51009183.41
3645405.895	16394843.19	4128952.763	3840058.065	3337755.4
19453983.32	24982346.84	20465360.48	31057005.52	21480473.87
761127.2156	549462.6521	441481.9753	1009156.125	364915.7834
144090850.6	124406237.3	73921279.27	137231954.3	86767283.89
2081543.798	7873252.565	1653909.385	956580.2957	1001296.714
184864802.6	199746102.9	187664766.9	173333357.7	156356185.3
25862911.19	24529719.38	10884325.69		12368057.38
40759332.18	47348815.96	38078506.88	49598379.35	186402308.7
13559268.26	14534025.63	27452670.05	24364401.23	19727899.22
210830.8878	187574.8361	3064825.518	1123684.304	3243193.564
1163113.004	710534.6567	297505.2533	772510.86	570356.4969
13362038.79	12404355.99	6011863.845	6344634.467	4096631.017
			474662.596	
63014.82993	44964.05177	130196.6085	629458.774	148044.8146
6746967.373	1278892.266	9350000.634	2246282.323	3027123.539

52613862.86	47772280.93	13250381.03	41311625.46	68800609.17
11880689.88	27167870.73	8237164.02	9881047.129	5838511.003
24244876.11	44299786.5	26181209.87	24719488.4	15839617.88
2069042.325	10389692.95	11721946.56	1752338.254	690177.2984
60160745.86	45202194.17	29322095.68	69229302	49674263.43
106701815.6	100589612.2	69959496.51	94769408.39	89226468.72
1531445.918	2177515.519	9152366.427	2015556.255	6501565.348
4633760.915	5878784.607	4577849.468	7967675.409	3075384.527
		1247561.715	3245291.804	1963913.018
			399888.0872	442642.4085
27146096.25	42595829.14	51313122.6	66445478.45	64594183.05
1404061.905	9675706.397	3753697.664	2763196.353	4105777.999
	1369308.103	1296646.7	719751.1969	
1212653.039	3175481.462		3677546.416	4138353.072
329421.5111		1195997.255	1845422.581	891050.3136
72467.30391	1077433.024	358316.2207	1922441.246	856646.0556
7965916.516	4681370.374		8369353.617	9054262.441
4509795.014	3802962.316	5408685.158	5989112.899	3005536.612
	111026.4775	1385561.636	212249.9884	
407719603.5	344139494.5	231960550.5	107756847.3	172765524.1
1481954158	1394547597	1260051769	1236728411	1274683163
12404090.1	22402825.11	12246771.7	9478625.108	16611756.91
150382.3104	766318.4445	153695.6566	81454.50938	93570.06609
6649251.883	14490838.91	9501736.944	7543049.758	15772555.75
16466237564	12324571608	11275046018	13764209168	12391252947
655496.7464	3599330.2	1914308.553	2309309.432	367366.3855
3409643.602	2654675.288	6086321.873	2079590.732	1734596.969
3002940.302	2456898.944	2031810.792	3601238.879	3817915.502
279037265.9	456339841.1	253519701	187041880.5	203000813.6
18390075.5	20700643.04	13786198.22	20035254.84	8894154.374
290893.6548		518803.0576		
660728926	757325156	594376130.6	585192077.5	614125054.9
499407854.5	598113002.5	644088899	465234824.9	369901931.3
236728.9277	2804126.03	756426.2442	3050144.531	1127150.753
135224519.9	130148379.1	49077703.2	147406523.3	195041929.3
1351911.652	967470.2573	1977005.221	1167851.506	749603.832
377426.8314	273840.3777		475158.4279	158128.9928
478328.336	1209654.571	1014441.622	2687115.585	411879.8584
15008431.3	13157655.23	31875862.96	21702428.83	16618892.64
67523085.17	45995523.45	35789378.08	54580397.88	32116370.51
5726653.723	5125137.941	2929346.531	5567385.093	822310.193
239889.6455	8039339.822	15927117.47	773606.1794	4233960.297
97784533.44	138182129.2	56365019.44	96014228.29	107133229.3
2025756.414		1329652.17	600694.5454	8077202.019
517909558.6	911087605	544246183.8	550195512.4	498534471.3
32784233.37	50762029.24	39487951.53	25011276.02	29094894.99
19882651.93	20712307.86	28868778.48	14756150.45	16861783.27

2809858.103	507768.5706	1085554.556	1981277.776	1728730.527
2964151.14	1560113.079	2176988.161	3860208.285	2566768.159
4660623.116	6244703.326	7420917.051	9001788.521	2602198.691
34745713.9	53388935.46	33056574.43	84123775.05	32370028.98
26856859.32	44977738.71	40600589.73	22681601.05	33746585.8
63086200.61	154973398.2	89940550.42	106547169.6	68284449.77
9019141.551	9000598.818	8148930.419	6080594.986	6816014.482
3126972.371	4764531.079	3710716.281	2404720.382	3113967.617
14581522.06	21855805.02	20461473.24	20284838.3	12201016.48
415537.2678	6837563.129			290667.0622
29830106.72	89386763.81	45414441.29	57255390.18	66752300.34
1854335.433	1112538.64	4050162.69	6208355.739	3410296.651
248253.1021		361506.1918	662000.2752	829250.4717
1099480.044	949503.2569	1629294.372	1669617.663	448276.1041
1200628234	611327851.9	1098772955	945953046.4	1002772232
	660038.5281			
8866359.677	8191570.736	15145074.38	13067344.04	6960024.462
2048487.138	2222536.766	7173086.944	5336145.558	3022009.371
266264.2755	1100306.065	464990.0008	504045.8764	442276.3747
358872.2156	584299.7021	346473.4446		
1095352.473	1799793.154	1364233.143	5110529.539	1549687.769
5057968.165	4771010.582	736589.1088	7338729.95	5807882.043
543053.2175	336161.5962	212997.5066	1033951.848	189400.4822
11192224.16	18352227.01	8890471.907	13680285.06	8774001.584
49562730.07	126858790.8	53394026.05	70500118.1	66730843.42
4061833.326	64535107.13	9434699.156	3381204.162	4692997.13
30217759.23	45398728.02	43490973.59	37370130.83	43548397
209557.9149	1836927.334	1804436.761		3307270.948
1612163.021	747567.9177	945864.8389	2953602.613	3564253.084
694470.0997	829516.7146	780450.1337		1325464.271
1964825.897			637690.7439	1515273.316
1468206951	1304303110	1577087292	1947134394	1529800575
435680628.6	491555375.5	357279666.2	517386653.1	517824758.8
933075.2021	1719701.296	3019188.249	2919274.601	651682.2962
	6454431.149			
6144425.604	13107462.77	8485011.387	8790902.649	8030177.961
27837956.78	23133495.06	37687347.52	59011746.94	33263499.95
1654595396	2679821047	591669792.4	2048821944	2149233000
45164199.9	25195163.86	57328818.67	39417681.93	45232907.89
	64076.79519			
852966.9559		522779.7664	1403342.198	669223.4746
21529048239	23482294909	18486471938	18681399473	23128362763
643373.3174	1158803.142	922023.9756	1629766.225	
2466563.683	5461314.997	286956.865	812997.8377	1307964.915
7291906.474	50755891.2	2062211.401	4021355.16	4058172.941
	176705.2905	434691.4037	961834.6416	331453.4555
3322013.679	5045186.242	8471385.172	6820764.497	2647216.651
953617.6644	1740482.71	1310142.069	366451.0365	286724.6188
19974727.98	27261830.48	27325944.06	41090617.94	19242828.35
1605106452	1451377675	1079066958	1428341361	1370055353

1437286.261	948450.7089	816261.8587		936823.98
	777755.0368	818886.1744	393423.4902	
9414569.447	10863509.96	5915710.705	5013969.532	6125185.824
7194632.801	8722708.293	5374300.569	9586705.996	5743871.09
8929134.556	7933830.994	6760888.228	6971420.857	4483611.344
4925479114	4274990715	4181768133	4767740346	4617099287
11892047.98	8137590.457	4412044.8	9635206.907	9771522.786
	689960.5909			
1920166.11	1736450.669	1080374.7	2243827.957	1253142.354
505983481.3	622779958.7	680899163.8	547298897.1	427111820.1
2833079.509	2548230.216	1463334.936	5943053.458	1541717.153
			176065.2127	
206454.8828	1289068.365	1204282.135	1855682.254	1094572.292
111964582.1	71775575.39	48554600.82	85933131.74	419940915.4
67822470.37	169801309.4	74940384.44	60577397.85	88373163.17
2035120.321	3105540.425	1741584.977	2079674.93	1837039.562
4195808.215	1296596.081	1145574.196	3539872.749	3041955.905
118055751.5	45409510.58	82901932.7	78343940.14	112294105.3
26288256.51	31681894.01	18249413.63	25886020.94	13512689.41
17399175.6	22300424.2	26939568.36	11550557.14	8903419.079
10410340.26	19142119.46	5944979.683	19015513.58	18139813.42
326509.8927	292923.0365	432533.7393	736932.5438	169436.2309
11345306.9	5221972.23		5187748.134	581104.1136
565423.6653	1894127.726	1987295.416	2975858.883	
2546308.66	3838873.763	3075360.777	3470917.036	2245088.33
3856037.545	33486353.09	5442372.156	7075742.56	2973299.316
12367908.13	8946912.151	5155557.142	11790282.76	4935012.8
2608099.961	2316769.652	4028726.815	2694663.87	1703038.275
554019.9993	344914.0224	474358.6751	391211.1883	487795.5141
422468.0083	1075787.147	377958.755	1011427.298	
2984203.321	3551532.026	3372929.481	1570134.244	1759658.642
			93070.0458	
13477775364	11616740374	11986242452	8305275594	13299223261
2114880.992	860482.512	2058248.169	2904793.082	991996.8876
75845728.61	131573260.5	86725147.92	109704479.2	80093274.64
450923266.3	483903219.9	348801212.4	299287737.9	336538298.5
198041.8231	6395646.613	5446178.897	24277821.05	3714473.437
84229449397	1.06557E+11	81432620249	84203964989	1.05153E+11
1422627.926	1592461.128	1374432.868	1799696.465	552866.8477
	580339.8519	764238.4579	614164.7828	420081.5439
11852027.62	1497585.362	12719338.06	10486449.61	8254079.032
1000655.1	261163.7776	1403228.043	3543368.757	949038.3793
993036208.7	1708054511	844824887	795967967.8	972525069.2
26725139.8	22291911.83	56294535.32	129986078.4	52471133.96
19594828.93	10735668.57	17406412.95	21150653.84	15311488.94
883678.0735	744848.6145	1080374.624	5592627.587	3602762.609
177657170.6	245986005	272556181	156673412.5	187817894.8
791993.01		1092256.484		
1279064.876	2160570.297	699665.5765	1056851.892	985427.9545
1800833.066			1276728.191	

400606.0333	519218.9038		956701.5947	
520389.0157	623311.6182	1638272.214		335799.0268
3108154.023	5462773.867	2273337.39	3147845.656	3312239.882
14007015.21	24963042.81	21446861.15	22289362.94	18497300.1
60653716.77	66823979.3	60523746.62	87670471.28	50957447.63
47995930.28	229887988	53951329.43	37078257.19	81112245.61
17917896.87	21533136.58	21404158.99	19299314.64	20328268.57
430989.4525	2147163.927	213036.8724	3037997.129	540268.0264
2625530.366	1050214.379			
250237702.1	180085870.6	398387207.5	286545411	322331107.3
4331448.819	3556210.05	7169276.393	2891378.07	5199499.389
261889.6843	250466.5275	134507.3073	652510.9219	238736.1714
206804572.7	111811677.3	355706162.5	526888192.5	395204067
	47004.87991	113293.2703	457585.9362	
2683579.144	11667057.68	1606588.358	1221406.496	2182252.517
12690436.13	35337182.5	6281233.632	8706984.684	12850017.63
19197346.95	12818001.8	4381372.075	8066960.002	8712413.188
2107586.17	1379710.413	1354644.239	1031468.278	1123294.585
		801228.5057	1472123.504	
9067476.113	7317623.961	8422630.384	5251407.42	4143432.158
56216.81007		655707.9141	1217833.502	57161.76743
5699873.819	3190595.706	5209368.557	8605991.608	4375032.138
2117484558	1825879377	1701056270	1422467150	1728394892
	359707.6461		369459.7144	
80874177.18	77630270.44	74850700.95	139757392.2	90022168.4
810929.275	932507.993	747638.9929	456814.6025	659734.2646
23931100.21	26366567.5	20740271.73	28180940.95	14159638.37
337184.0281	1538123.511	1308979.875	850542.1939	
1921234502	2846150584	1704761122	2090721728	2118727982
72296309.9	51959427.27	125803380.8	212816772.1	112031590.8
13215947.39	6240168.87	5956128.449	12150980.77	8123511.214
1536495.252	24793455.03	1412748.442	1091846.977	1696674.1
4680836443	4817031054	3556947440	3970830303	4197073442
61501656.94	71718946	35025631.91	88355073.59	31432076.08
75257994.06	70550175.25	81908132.63	39650168.49	60647624.25
852763.6421	1004257.462	1517900.878	4141991.295	1754902.778
4755189.447	5717559.834	3258633.697	3149240.559	2449930.316
41380016.65	67424505.89	42420291.76	33438409.26	23344065.99
487886.4375	3246336.425	726664.1396	2752268.557	1583873.712
643351.6381		796883.0058	595415.6907	479204.6772
7571703.884	16058664.93	5527112.506	2510658.605	7008900.412
354161.266	351679.6471		510924.1529	341459.8987
416737.6153	1322413.001	952520.6843	1798935.66	1000594.801
196975843.7	263288265.1	176897701.7	212236617.3	192856729.6
	31277.67124		274429.907	117753.9244
6568451.797	117466841.3	7503800.103	10756373.79	13702294.79
2376778.523	2714932.722	1987801.064	1310051.947	1428950.171

	743646.7387	5546734.118	614125.7253	764074.2798
30451263.13	37234402.69	27818393.33	60608831.4	21857639.96
8451288.678	20172338.55	4956725.353	5608168.373	8301592.14
9414851.324	17865798.71	8965802.929	10004334.06	11093092.08
19971201.42	11215270.81	12738014.68	9736720.449	18057977.62
			380199.7636	
1823690.92	28342424.51	1279989.936	1620854.163	1551402.721
5456293.729	3234599.819	16208452.45	51417.36146	
268323.1922		179416.0586	683401.6182	247378.714
1697303.117	1643171.554	572041.995	1800691.579	2128716.473
190476074	226863472.1	134324095.5	107760723.8	142179016.9
9547218.353	12753677.96	21903157.19	20165207.95	11951236.73
194290871.5	96354385.54	259268541.1	129963038.3	128746142.1
97424231.38	103639574.1	78986719.65	79425241.45	78511682.19
40058602.43	36998553.12	35358541.01	21980920.9	21287249.4
454572400.9	587842673.7	601493954.5	702192014	318047383
10806344.77	9576954.676	7476093.261	5749426.87	7122886.378
269619.7296	417375.7831	431800.9394	503897.9343	197330.3158
4379628.658	3556604.094	4563810.194	3328818.179	2514683.898
211626316.2	468858965.6	236561646.1	406180449.6	321531833.9
246783.2249	290863.0614	549349.3268	648778.6118	455930.6624
194141.9133	119948.8632	164501.7893	601896.5795	367149.5217
6981352.602	10292224	8118612.815	11908835.26	27217187.23
25138626.02	16181718.7	1824460.071	846339.2187	9431496.529
77346735.29	105788797.4	78485035	123882942.6	77787514.02
9041810.053	40536841.3	7014085.705	28400308.62	2330675.224
6056130.499	4588783.46	8233907.765	11438726.37	5481820.96
104839339.1	84837613.58	98140443.03	155275038.3	131046524.5
1874994.598	2475115.261	1432031.869	1845915.799	2612292.063
10488785.66	81802756.34	9608162.52	10530480.55	9999785.505
19020309.59	8333164.888	22074414.61	11928712.07	15609310.94
58475790.6	45552371.98	32626727.49	89904084.11	49895772.59
796740.3901	615903.7968	372952.8397	2227112.455	867880.2264
164924010.7	132202329	157069277.3	127783818.2	110198711.5
17097188715	13803580996	17303707822	12648199117	12163896207
25671003.61	51928396.55	33373638.54	32523784.23	38604891.26
924377905.8	1815350636	1054637103	1217153631	1343010899
2066134.005	2577428.379	732961.4375	2157918.804	1195418.821
728210052.1	897247658.6	145717784.7	970705763.6	419222174
			284646.5207	
3825518.36	6598956.672	4220555.444	2534962.125	1504206.465
3066944.065	2305869.917	3990892.935	16348085.47	19191609.8
	2117208.344	2719651.645	521364.6718	784245.2126
315863593.3	266654141.8	447269292.3	275068328.9	275822706.5
2203336.53	42354921.79	2842111.511	3058301.943	7893743.454
6761820673	7632097560	6898360265	6246314679	8784644928
961679.473	529273.5738		937618.5212	529783.6321

20299720.89	20193497.92	12355639.69	25249005.78	17003835.7
226035.1631	704412.3071	326238.9992	984759.4967	653214.6902
11195645477	9442287680	11051291835	9224395723	10160889546
309759.1794		375670.4509	921677.2699	
162392311.9	165364028.3	225742550.4	233607448.5	136714840.4
303697823.1	393213355.6	411861798.9	377646806.9	294118493.1
1755362.64	1497006.336		2463035.76	1571052.203
37710729.15	105365444.3	39325448.66	70595711.68	27971087.61
1511402486	2160036174	2626428018	1261486069	1837051091
	382517.7682	248419.747	393234.8186	196142.9911
175531289.1	172119499.3	136492031.6	205393932	148872463
45090723.55	45106629.83	23499122.87	63805433.21	36878864.19
1926247568	2996322097	1444525398	1989412126	1562174308
709113629.3	790849076.8	372636112.5	1093296357	789314717.3
13397942.96	11026545.14	6936692.858	10326216.78	9038870.031
	91408.81845	39413.1516		
1141405.835	1622404.815	1080743.709	709303.5103	741665.3557
5538041.917	2967297.783	4610100.488	1929720.61	1525344.241
31279196.22	52780364.42	34106550.67	28853444.95	39087730.2
7782397.27	11854096.22	8631355.847	13577780.35	13996370.76
542934.9477	517715.2533	685522.2909	203240.2108	239252.84
1400890840	1274358304	1103671884	1301628968	1339065166
46595912.83	41388497.97	52412384.03	41252956.14	45489795.81
3352276.132	503363.9418	1737766.036	2735731.934	4527350.926
1228455.803	1838823.627	1885146.217	2297100.953	204771.9455
1823303.38	1637248.287	1232006.303	952303.1005	856307.9305
388286.242	2688838.634	3752517.776	6046493.467	1133866.341
8624391.792	9584604.876	10513277.56	7711875.839	10763737.21
	578445.2479	2105041.828	1257139.219	589307.0344
7230691.77	8205944.439	5281877.217	10962430.94	16026262.91
6803344.044	12938134.95	15293733.78	12695625.79	4553257.73
15270579.51	10303937.08	4564825.576	10232362.3	10881630.04
5324492.663	8538787.879	5196606.108	9553563.744	4270653.613
207373722.8	139779299.7	111862868.7	231927453.3	59413644.36
4917699.44	5832815.543	3130195.595	9491021.085	3872906.099
5892943.659	10489742.1	7655072.049	8967101.877	6608023.333
1322807973	1678808864	1609791914	1531088964	1590149778
1283796.371	1637748.617	1001191.143	2091925.899	1021387.886
	927777.2575			383409.6874
115619.5165	135541.573	120660.7762	214635.3814	90176.80584
1791365.075	3130363.394	4258360.284	1448381.624	1362267.703
75241339.78	31993761.34	46044150.28	74508669.84	29067757.28
179416678.9	266951653.4	245368545.4	318297811.6	191439032.6
175866566.7	163563956.5	186801399.9	238283350	192167012.1
72768699.63	19048735.81	8282286.512	14874468.21	11338197.97
52911941.4	35539785.68	19173334.15	17255272.7	31267014.86
37815829.29	59913936.06	44065956.75	53168943.31	30084176.59

162697526.8	243702652.2	189934613.9	264301506.8	159144633.1
3298709.268	6150553.773	3970691.725	3011378.506	
13689165.21	23683636.27	45922011.46	28230902.35	40969027.39
1215130.342	1499112.601	1228413.275	2119753.51	1126061.192
3006529.821	3478087.072	3237913.667	3539561.783	1754345.839
4680077.563	3924582.022	4600552.092	2594502.901	2212965.408
102811964.1	121979477.1	123542623.3	81441252.94	107554923.2
76795282.95	117310014	83560143.22	113396536	58131979.97
10025276	9782214.399	13168081.59	23504581.83	11754810.42
182587.1406	297458.1875			
7058135.551	14580471.52	3203603.712	5973762.775	6087828.911
876026.9336	88974.47615	339710.066	742037.1335	849461.1102
2550223.51	2862721.702	2965723.202		
372824723.5	329400326.5	471968154.2	469591035.2	350036677.7
	231511.2634			
1110330.365	1317406.296	1485287.805	1262435.006	515554.7937
			1018716.266	
871649054.8	565203335.9	749957450.3	813944372.9	607875447.3
860489267.6	1210009317	901073878.2	1037064884	1135307634
246213.084	264957.3462	107351.9765	1627222.307	
1013150.458	741437.9727	588233.8901	763145.2958	570623.7429
36619339.78	42825882.05	39188659.01	36744639.9	20526563.08
	981484.1066			
1577609.679	10786567.75	1927363.855	2005696.299	1859905.331
19634555.86	25074325.4	21732372.18	30250892.57	16523389.11
3843347663	3604429623	3807400432	3926604695	3491407217
1982916981	926050302.8	3925844295	2307453422	4530444045
66644822.85	53939873.9	78810635.93	63279750.88	47063815.81
1123600.898	2233134.75	1225173.931	3267128.507	817396.7063
1415684337	1667941883	1075464114	1531465061	1132368024
504346875.8	571239746.5	238882135.3	734057516.6	330708249.4
7694011.698	10792828.13	10725173.17	6483726.052	10211795.06
378767.5756	240214.7396		837206.417	315810.952
1979627.362	4228486.712	5487506.989	4748109.491	3679334.61
32583442.49	49387352.14	38038074.65	28938870.24	35023639.44
592877.1918	1155457.599	947381.8251	986032.4782	400300.2919
	226096.2575			
9800428.119	23117192.48	5544979.807	1154322.122	1173899.711
1693466645	937491236.4	1342273702	1729235066	1012610483
606493.0168	396704.2769	356732.4256	1389780.204	
30925542938	30894492064	57616220409	42461301469	40006570059
55550010.79	52847398.02	26931237	28938826.12	21086569.62
			1197499.27	2601188.877
668337.594	1241467.453	400342.3656	338548.5644	412600.2085
71440586.92	93192582.97	116165698.8	135721180.2	69450540.7
3162326.75	6836734.069	2286612.432	3311101.96	3467196.652

952459.945	2099183.179	3221322.375	1242065.814	2025958.162
	859024.1756	911331.2532		
1669174.329	1951957.352	426298.767	1305747.023	1233178.985
63481590.53	35928010.43	324959658.6	245370534.6	312364635.1
35162598.58	42860938.48	33449858.94	34382237.61	23950487.14
8045147.035	11420999.82	3871438.642	9469089.156	3247417.689
776678129.5	1060825619	829901298	1086539955	798286738.9
6601700.33	10529342.81	7576305.254	13426370.82	12228528.4
17817674.33	11066427.8	12301852.89	15955867.75	12495157.14
187887046.7	217121721.2	136513971.1	111642512.7	66141206.14
	3846866.649	3592139.331	597904.5258	
18152706.88	27893119.15	17737843.97	22800691.94	23271295.04
9426615.997	11883758.93	9649249.047	15022897.5	5873100.375
		1882268.491	1827582.254	1469952.881
98094956	76796078.15	75806805.16	155212974.5	75022526.38
1258694.996	2646623.868	4517589.829	1308213.324	1336446.733
27183005.25	25103439.88	17786178.08	34744092.31	31049473.78
104169300.6	63371493.32	110321937.8	127842753.2	101423145
10456737.4	14113502.8	11570220.03	21091444	7328310.803
36341151.54	34833242.04	27369051.57	51441059.59	10276155.68
3935578.997	9786524.843	2616167.746	4064294.725	1501825.78
1423433.405		2132577.471	1717763.962	
19393438.07	22313197.5	35106609.14	38214635.81	55235076.22
7387349701	4477834873	8842841312	9824581812	6864096234
405165174.1	277722130.9	401378550.5	252459776	236686036.3
1773011.228	2779976.334	894826.214	3361385.629	1920542.647
259026.9621	290557.8844	484388.8953	677877.7111	198941.0978
13853490.21	13673794.95	6460607.999	10184202.25	11217633.6
11473515.05	102980697.3	14873083.73	17962687.29	18823369.09
4091816.78	3832087.09	2034477.495	3611011.027	3486453.329
2719569.985	2129653.492	2976259.853	1415045.667	3013327.546
	24519603.32	901328.7261	3381400.962	
66949925.81	71620441.69	36668509.92	48050537.1	48310904.76
1014740982	943494321.6	1375930798	1548501276	1201127467
10889130.46	19427915.05	7815447.422	13165033.29	7260210.997
11431386.21	8119525.494	18081321.27	12841324.98	14973090.21
63287474.71	70434903.67	69357052.54	16913409.68	23386211.59
740277985.4	919419524.6	553603281.5	294258372.6	515642953.7
28270641.52	17633735.49	25949607.63	16430794.46	14641539.01
29385540.41	28241273.2	32151385.98	11028470.26	14540563.6
9692203.243	6126692.315	5399562.374	4816291.006	3336120.353
8961358.226	3485120.544	5022811.417	4593809.125	2040309.943
417000.6412	5749334.308	2945869.786	2619748.178	6081471.725
3801396.629	6931718.085	8033265.148	3754871.832	2761718.284
917944.9662				
			951143.8234	

20219937.67	19060988.08	17044410.37	22116182.25	24274126.16
1336130.19		1587492.456		
		410074.2228		
144408793	214387721.4	344833821.4	291640603.2	186391352.5
8655238.452	471802.435	2518642.759	1416918.154	117629.169
14630822.08	39869762.39	15729033.52	17684922.8	10733980.42
427388307.7	729800352	269019324.9	485095748.6	523271153.8
6948460.746	12990326.29	13542870.8	4345079.135	7253617.819
	14842735.83	14325624.8	5215086.868	3643791.666
8353457.545	7762377.123	8588926.831	7397749.247	6350491.472
148082030	110807472.9	110510438.7	201344707.5	160404943.7
2483906.24	2871224.729	2775713.053	1519734.398	1442415.036
12745866.95	11170580.9	22039983.12	12966099.99	17671871.14
1538475.511				
39881160.36	40041312.69	34726630.71	39475728.41	30879929.34
1827208.333	1626034.366	1028156.318	2689510.832	2160996.788
	678184.6908		426220.8575	
4054566.091	7223775.104	8379906.621	7890269.405	2336553.983
7096261.622	1594681.299	5911819.749	5056203.803	3545135.394
5646903.391	15847638.81	9413377.281	8936913.719	5172681.232
169881.7531	229333.1421	257972.2951	405048.3463	258580.3172
6130004766	8349190832	5935486515	4695379889	7616781813
11922996.41	27278331.55	13389085.86	13151102.11	14988706.52
2284057.945	2176303.676	2987488.785	3457737.667	4872244.223
184912.7239	43230.01499			
2595849.861	4875923.635	4509248.426	1344145.862	2655869.133
3953936.614	3986981.565	3219112.02	5958188.374	4353551.371
7332028.627	7016536.953	5212681.362	3836639.496	3771406.508
81246576.41	83574029.63	125250105.3	150390753.6	75741536.31
458399161.7	373515262.6	327572117.3	507993890.9	496986933.5
2777091.957	4648846.939	5653007.143	6324986.695	14609124.94
7723975.07	3197498.225	8112055.547	12123724.16	4463954.98
179187000.5	145782516.6	191957111.7	206492220.6	225519670
2505738.382	1935270.969	1327931.986	9654203.115	1474995.297
383046.8488	98870.23215	257459.7786	992120.6215	898811.9406
5756469.523	13044148.53	7275282.965	5910590.57	3853690.115
394588.4137				
43223.50966	1041540.421	911623.7417	954261.1502	
1599987.196	1949962.788	1832265.767	1413718.706	1363548.451
54721200.39	43126581.72	41762676.38	42160598.44	44350281.36
	1197051.193	1258013.939	983768.562	1534928.956
3066486048	4464175218	2172121826	4128675503	2036076395
1191650.811	768819.1377	522014.6652	588413.1806	1532689.801
		1161157.236	316427.8222	
2134145424	1870304414	1789664633	2031987993	1957805476
3549806.358	4362376.922	9299711.155	8180142.526	7188265.104
232700156.5	264373939.3	145195957.1	114805608	200937720.1
186253.3621	96993.71791	333417.86	218166.5166	839123.6095
386448.8661	1390083.384	1087504.153	3165644.347	723401.7448
1369892.993	71699091.75	8634602.749	7038305.061	3034875.579

67275942.21	46997154.39	32985460.54	31832527.02	31116742.43
37602494.75	48628906.46	58702858.88	59834690.16	37949359.24
403905508.5	607061785.5	1262168797	349715286.9	498723168.4
5054727.694	2774420.223	545476.4532	6964.41631	2355005.076
1797963.628	1796207.413	1505316.814	836922.3781	364158.8334
693164.384	953261.7041	1144057.756	326190.4856	558294.0509
103142063.8	98134750.39	147173422.5	219996391.4	219699218.2
45113674.96	37928293.31	38805267.69	17152651.96	14790405.13
	466719.7288	637834.217	840337.9946	865780.7993
20895541.56	23574796.05	12790951.65	22997415.22	16052175.63
629007.4637		704775.5036	551842.5602	
8543002.678	8798069.991	7835589.337	4228744.064	4052437.079
8232673.953	7163317.126	4441743.106	11626279.45	7953733.196
1064007.567	381507.2361	954877.9384	1987248.391	274532.7019
1456344433	1466387287	947952519.9	443797276.4	471774970
7740709.053	6911101.191	6324668.839	6961015.749	5222969.109
11396886.21	13835151.45	12653059.41	8798670.872	6953909.815
40773351.69	12532441.05	19481992.81	8921976.039	18700623.35
19661702.1	26339248.87	17829895.71	17207778.87	19033920.37
751277.3868	530729.9993			
	35797160.87	4469100.766	7616895.938	
801885.726	829475.6953		639479.8509	
8425736.964	11991526.59	6008972.033	6283528.596	7752334.34
229484.4507	136464.364	151351.999	1054109.894	545505.4412
2626038.745	9675107.687	2152946.708	1932203.611	1619982.506
63505.97113	60359.20338			
158231676.9	132898366.7	166462194.7	233808944.3	305780810.5
7622331.357	6223978.793	4578927.626	6211438.726	4332094.396
15292284.59	15499152.53	14424117.74	14855806.44	7611505.9
5711129.667	7153715.01	6866590.72	6931466.172	7588243.427
455799.3469	3129258.22			511440.2569
	586594.9153	404604.4821	2418753.795	
		692747.2561	1315910.724	
385955673.1	361299271.1	267959345.2	404279701.3	274033994.1
4383500.938	13139916.48	14687271.32	12915896.79	6247313.087
1683094.956	3911236.079	1676040.623	1825110.315	1235318.082
4956517.277	2782855.642	5854519.367	3032014.918	1637141.998
3977430.754	595548.7842	8281123.514	3267517.375	5465421.771
611278.262				
286441662.6	608306047	314305793	381993802.6	336289217.4
762106478.1	665914333.5	811878967.2	685211154.1	511835031.7
10528665.26	28586606.71	13247239	12817692.15	6429847.584
48376482.29	51438886.13	26892583.31	28751411.47	28764789.03
136486.8186	50447.52005	3996231.922	6507366.882	2629542.684
47155516.48	81158859.02	97085685.85	67286932.82	73107993.88
7031152.735	9755976.085	5817477.59	8173624.91	6454365.112
315523.0104		114549.9116	4128721.126	413449.3816
20860488.53	34811464.71	21683723.37	23961174.79	23737423.74

	167300.4845	1560352.146		
5727887.615	10036696.68	5574184.758	6995251.737	9463356.157
848446077.4	571248924.3	926394801.7	2085849406	999095129.2
13366852.81	27857027.41	33597088.77	36226192.05	26239761.01
7787266.246	9026368.545	8116437.218	11346488.49	8930342.42
44721914.48	47831461.34	41198556.35	36957082.82	40655211.57
9896812.585	8789802.393	7911484.033	8115769.674	6671193.393
33213196.01	40483195.52	22418860.68	23572912.8	28394931.06
88633847.61	116721805.3	71654995.18	88083995.35	134198082
203956.1364	293264.7045	101598.5019	259798.3293	111498.6312
47417927.29	81285192.71	51811633.52	56916590.16	66794790.21
146708.3834	240452.2539	166567.2478	199395.548	198577.0227
102778.8222	130575.8988	105827.741	133139.7513	78318.66402
	1027304.032	1589645.186	3199765.332	704466.7013
497543.6979	11468621.4	2857053.791	5719411.127	1204917.225
	19410152.08		579988.1994	
1358176.156	3310489.353	3770176.706	6403827.994	1627950.334
2345353.875	671595.3869			2616461.57
487466540	221186749.7	276927613.7	360095076.6	338188505.4
62473955.93	76064828.17	79546546.39	42391694.11	41740691.93
365866.038	354389.1027	465275.6258	294050.8758	203668.7087
4197990.562	33395647.6	10497221.98	11238107.11	9700195.348
7736048.177	1915658.27	2947041.761	2327534.6	566079.6783
39911436.36	88336057.62	38699500.53	56686964.8	25936251.65
4221391077	3640264267	4549784550	2754922624	5234611042
8779753.073	29593299.76	11345176.21	14122516.21	9909994.197
589479.0626	460042.9234	1284104.458		720402.1801
72244658.07	100000585.2	111476530	89356156.88	49222561.83
6471994.581	7316616.696	7361882.372	10617213.42	10970689.72
51928982.71	55160551.79	31606723.3	62925927.93	52625552.25
2506959.631	5320667.752	2460288.874		
36909814.32	7596487.82	52620586.07	57481352.3	30606448.14
43968552497	32729683993	20772535362	50657076924	48157683655
	684363.4659		698792.1506	625303.2229
772217834.3	584619894.1	410477350.2	615249731.1	634245498.5
12863388.53	4550608.679	23524175.75	12311795.3	21073130.43
5208744.284	4669724.996	7270941.987	4472888.382	2676696.181
2249448.615	3247578.889	2678635.372	4642147.03	3837782.06
5195.500845			1216.552828	6125.596886
133090.9825				156083.7243
1466616.239	5134203.78	2351639.814	2270863.87	1755060.471
		124798.4153	362441.6595	
4634790.544	6067211.343	3535433.028	3381423.875	2394355.37
1494455.904	1500921.399	2274548.003	2015338.334	1650172.95
	270883.3685			
3607604.293	6388158.143	1354977.925	6961002.837	2010189.867
4112617.744	4183863.056	4313189.548		1147998.236

11326742.6	9949357.827	4536545.657	18129825.27	11005390.29
998101.4723	910733.8245			
11040010.31	13256961.86	10061892.94	9605073.423	7905478.314
1044686.268	2173302.784	3297219.745	1649421.716	768299.2691
1744410.044	7512128.487	1229556.254	1809296.445	2503794.523
559107.2254	390535.5125	240826.556	631312.9059	456965.4113
127358581.3	197513829.9	398230442.7	440245711.6	368869018.9
13437382912	15896380698	15356734349	13302622217	13304758711
	1428563.774			
2522774.272	7161528.017	1217036.661	2686947.688	5091717.046
47138410.77	54262071.72	41559806.97	33539121.14	37460184.65
5025186.773	5010078.177	5165382.24	11727221.45	6987364.961
386303585.7	329044810.3	350425575.5	255855931.8	192387679.2
291519433.9	389693856.7	383520474.9	281239389.7	182135948.8
2887564312	2563349919	2994623638	2823151267	2330587476
162565.3457	307768.9168	372050.8385	340954.9803	137130.2397
1832440.244	1389514.912	1252620.104	942745.3431	894100.4767
1417086.567	5836048.175	3604830.907	3677895.23	2597397.88
2342200.109	794698.0658	1094128.393	3383905.2	2226911.048
2942931.177	16162986.16	2142923.357	2550285.961	2654815.185
4148801.507	4274403.197	8127634.87	9118542.626	6645990.527
3363464430	2599808827	2765531667	3324639175	3217110807
923728113.2	2193898486	2198603307	1778220517	1950208837
3498213.286	2626992.78	1110005.144	1443579.464	757801.922
32375513032	44328965868	47123684795	27186463139	27453271405
4101027.259	7539475.821	4981601.423	6153529.44	7465203.532
1234213.719	6423564.863	4159872.684	9080044.338	5328904.345
3095640354	1254162649	3881621786	3606148252	4166899147
2959225.495	5316538.494	4160451.855	6206806.029	4168817
8629237.671	9276569.597	11837917.36	15879122.54	7734085.562
1085122.479	402198.4418	1305012.861	5027469.218	1440336.392
558833.1241	101292301.4	2499209.433	6148219.602	289556.3607
174676.0618	5535055.281	381265.5492	413705.5564	402102.4025
31052269.55	27518830.91	23306472.8	26763544.64	16413314
10971721.4	13396240.11	10821692.23	13014760.22	10851837.6
			2443203.692	
342806.7082	490586.3141	532782.4239	355368.0063	
2645918.741	4473329.639	1670558.122	3627210.154	2112319.243
1057419.406	920737.9391	842636.4812	781485.6352	940337.0016
221487279.7	428374971.4	250941966.6	208687567.6	254099086
2802127.61	2502407.427	541270.095	5266658.228	4922640.152
39110682.86	47525205.85	39957450.85	37359204.37	21995943.22
739218.8187	639638.0756	1730556.091	2369577.486	1334974.727
407473063.2	443248069.2	291957592.9	510079625.9	300278524.6
1768268.797	8718120.413	2046504.873	952449.015	4207961.623
	1574117.009		1057905.593	144661.9043

10414061964	11157234088	10894651832	11537771349	10728318825
981548.6913	602051.0059	739221.0977	1069375.766	706045.454
187141400.3	225968143.2	234825893.3	284587431.3	263557283.5
4656868.882	2115580.797	4561389.903	1227320.614	2139754.048
22409667.68	39340166.89	34492439.11	23374384.9	26038489.97
144500089.7	369543086.4	221426691	224745687.8	393121504.4
1715306.231	1746347.515	634431.1927	2290318.275	1253648.84
120730169.7	79731916.31	127244877.5	174604003.7	459319303.8
488290.2843	1117189.541	328761.6506	187229.2038	739552.7993
1291889.426		2306131.707	792053.0201	
1696804.683	2209597.086	1725291.418	2477866.04	1171469.297
210519385.7	162893542.4	158557996.5	153515310.6	162264672.1
15441613.01	16330722.34	18111419.06	20762521.63	11114533.07
4474254.041	5937333.893	3799189.425	6449749.95	6415973.521
2622366.797	6026309.357	14820324.97	32038064.61	5550699.402
212885.8693			671578.4862	232184.2147
43777.01113	30120.39382		138728.8701	264344.3888
		23202.47027		
3358867.602	4166683.722	4821301.014	2263214.456	2397110.405
43554232.68	57579445.08	56119619.33	32179868.2	44666717.87
219503.8865				140590.3644
1304712.423	2125577.736	1326141.221	170597.7754	1078143.968
22288752.05	18915731.13	24400507.81	19553989.77	17854534.86
1542301.617	6114040.633	4742417.384	4542465.424	5485891.75
100149764	65955438.24	44486328.6	74625128.05	55713697.87
5509569.701	13098004.54	4388473.128	5755553.573	5753334.368
33045050.79	35827364.93	30008284.95	18878564.3	20537267.34
576160.4585	672415.1025	1002301.733	1619971.988	796367.2828
13001051.43	15818291.45	12013105.66	4474262.156	3333782.356
29601.94086	500084.4842	327089.3613		
	491844.2559			
769502.7607	6103032.407	1113552.806		1123853.13
1786093.434	1926864.692	3008763.284	2087777.953	832270.5775
2967118.494	5060291.979	1046285.725	3197315.733	717768.6669
	43182858.7		18046574.66	4866308.481
897922.7589	471348.6827	1280729.953		
178846410.4	127024137.7	167394374	199019444.6	176246447.5
71203304.18	28791565.94	47203336.14	59469321.7	68693787.81
1262668408	1803896700	1876424037	1440146940	858070179.5
1512258.856	1333110.239	276543.4857	2308198.817	623978.5517
14759154.81	18544302.21	20984901.54	13935515.57	11625240.15
	1256679.536			
321424.5989	2574187.373	334424.3947		646055.5888
21125315.1	9932226.174	3832668.937	9887086.474	20751277.29
5982828.013	9350393.453	4644280.695	2502158.639	1956912.74
3781017.397	4435350.61	4050415.797	1098679.615	
	1270153.569		20725613.15	1016622.185
12451515.64	8594280.482	33507378.32	25278357.19	29001832.36

1081863.993	166175.1367	287126.9358	2224508.19	5761309.519
20005120.36	25193469.41	28658477.64	30153286.24	26145572.92
377434.5606	1281648.371	598721.8058	1157389.208	871814.5168
1141991.241	5257865.059	1021003.526	1367755.843	298981.0196
570429.0109	953124.7134		1014560.423	403487.7971
163201780	273642130	267826904.1	319787058	169580096.7
3915323.572	10085873.49	6763926.113	5378543.475	6385599.311
1519161159	1018068080	1471190249	848195695.3	899246035
143865478.1	217635314.8	240396726.4	16259691.93	16052122.96
11534223.98	10977786.08	9965757.037	11541835.78	4479480.678
2738424664	2491936218	2329956878	2087533893	2792272439
886657.5574	1884327.366	586312.8119	819200.7348	883426.589
38034373.49	59223926.4	37945236.41	41239537.57	49207619.23
461134.282	306157.4228		968951.9411	699849.8253
				1131787.993
9988996.884	10700133.04	4974927.883	59071471.73	29237129.77
44291891.65	84771895.2	40102427.86	72194192.54	31506761.07
3165846802	3022458561	2382582283	2647575690	2197356906
2342969.251	7986837.16	1660583.832	2388555.535	3501997.68
13250267.16	10330024.79	12837531	22647609.7	10052597.72
3400906.037	9429730.575		2341320.348	4960487.501
	32607669.27	646087.5191	8083388.886	
294909.1479	209278.311		254124.5948	
		32353.77398	16632.46219	18191.66409
494638936.5	257547814.1	558240603	892062496.3	620189773.8
70236531.88	65972904.83	90319220.85	48649577.13	51362110.93
	1236842.669	981665.5215		666265.5567
717071.573	1423677.97	899405.3438		
	1413892.657			
586634430.9	167245276.5	446702213.4	126955153.6	156860381.6
9698259.25	9145693.469	6656652.009	8075107.329	4184440.938
	331659.6776	69515.85159	550245.6818	
4063100.254	6988338.477	5364600.633	7514324.285	4236002.725
196573677.8	203316875.3	210878653.4	171615401.6	226611576.4
34573252.23	50072157.76	48124934.66	47817277.26	24857362.93
	128835.9679			
154335.3108	308530.9609		387162.7182	1712574.849
481230790.5	591853859.9	310774318.1	508105801.3	558943478.5
14261274051	2921301328	3113316574	18153727841	4520559689
	588960.2831			218786.726
3463375.44	4002563.158	7275522.421	4219582.111	8755853.433
185875.0826	177507.3596	206656.0879	482157.5943	262696.2305
303308.9644	533149.3248		254665.1579	328042.2924
8016159.607	2034548.657		2319551.124	3083242.802
4224495.433	5338640.577	2675811.988	4046689.632	4594593.968
370738406.3	472788887.6	378986935.1	494025359.4	276002504.1
307532769.9	221488839.3	90608538.29	226448317.5	189219156.7

675286.644	6001785.025	5324714.448	4650691.745	
277162977.4	853017770.5	81182600.57	122989814.3	213970844.8
625447.4942	900154.1935	751928.7589	1054207.218	498857.6708
70022335.17	139218250.7	106398905	165796736	76398657.42
19130.47703	149640.7225			
129059637.6	96775435.55	155011714	9594126.979	13796931.59
22099505.64	24963357.45	12125154.41	30257169.88	11921835.03
	1274831.592		193989.9162	288066.5842
602882.0744	1571544.676		598810.6046	
174196233.3	119712750.8	150023169.2	122232084.5	96143198.79
		225728.2308	595584.6556	
653914.143	3293099.58	488840.7084	2727919.186	26300.5419
668952.3187	89283146.42	1062198.038	7088323.259	2732330.787
90503106.59	149626502.5	91354144.46	101561693.8	93521394.9
7959977.483	2979812.869	6489502.317	6164933.512	6966120.791
	2389817.591		1469570.605	
602050.4393	693947.6116	860244.4698	1080690.172	132478.0697
228619861.8	84889630.89	246391469.4	269823328.1	110163470.1
1594272807	1313176946	2588422190	2516673951	3484208009
749966.3573		581433.3193	1817532.15	1638508.316
		492750.8827		
1500368.279	5198369.73	2549240.198	3467776.103	771037.4996
		23598.69618		
6849829.165	8883564.575	5834893.26	8264956.171	6044655.816
	3729895.88		2000717.028	674729.6177
663624.1289	2439456.287	1106499.803	1502105.759	566142.1958
3341396441	2877403614	2611630855	1593809129	1425435912
551621760.2	664368939.2	328048389.5	390459344.2	436706557.4
14922834.5	19155258.15	16343016.13	22738518.12	11168556.54
498158.3991	1644157.024		511097.333	
7984507098	7357063821	5994233461	6325770172	6234836706
16913147.94	1201482887	186907637.4	631057281.3	18012276.61
12894918.37	74219942.55	16515997.86	26771981.21	20937335.64
			402356.7533	
41330535.68	19073497.49	43885966.82	47175826.26	46618153.9
80595.09872	312557.3896	277470.0987	180083.9494	206597.6854
9827989.429	6912850.694	4566868.947	12010418.84	10640679.28
8043560.302	288684364	32744500.8	90048348.41	4156725.565
3258042.894	1975658.542	916682.1792	1166848.201	2317398.441
13714821.16	12024964.65	6397133.236	12267793.43	12485788.83
15354501	9303542.534	10665151.4	11158869.33	10270441.87
9353803.245	11653601.71	7406805.241	6875128.081	4822047.007
9489046.883	6167885.874	4456698.902	10782545.75	9142649.577
796880.5512		1267671.455	734089.5227	
3920759.929	5158375.123	2731247.89	3127420.468	2431920.01
			400732.6627	
852353.6214	2037784.009	1218496.74	1295893.898	1231477.391

7740471.193	39015429.47	41894958.54	59749728.2	27565791.66
34089713.78	59917031.32	54340386.2	58892810.52	30126432.43
7262870.291	16137345.64	9457365.381	5173246.748	9177035.796
603218.857	1742853.733	1192311.665	784017.9743	1448894.234
211309205.7	129543314.5	183387128.1	134905507.4	90389868.61
196357.015	842613.7868	230800.0311	1887286.979	1166597.669
46897.94041	45191.30829	146235.0508		100829.4641
1156466019	1154575930	1172123001	1048632355	1905308181
577010.6152	531687.4363	245679.5293	1355047.228	218083.4228
11490616.98	353254608.7	34221675.16	118292150.2	8750870.099
10035085.24	12611262.79	32349671.87	19995633.52	19416038.92
3387819.684	1799323.78	1492734.575		647770.0291
2998135.377	2788761.945	1670068.995	2232486.677	2427800.356
2042790.1	5111266.126	3206559.084	4307372.217	3110508.248
218137.4806		165210.7138	218909.0907	111104.4416
184700.2908		19370.35506	401345.2271	10091.63623
3497053.776	5012341.469	3158601.622	3608011.384	4411701.44
2046262.481	2251529.185	1023156.261	4804468.265	336355.7363
6376968.727	2468680.738	3459765.267	6875517.021	3796056.776
31201406.47	16543027.82	23971993.1	16043046.28	27580399.26
		747246.7693		
101232941.1	98367395.9	97472756.65	207806235	75957687.94
2699330.42	2814184.341	2175733.802	2364369.01	1143101.126
5072888.678	5088016.214	2174524.7	1585053.133	2338519.046
517223.8443		200489.6525	240421.666	404435.2993
5150705.474	3878132.047	6700837.59	5349048.073	4289105.093
33658652767	29703663740	31569515262	31663866624	33969223753
348475906.9	222798576.1	336327593.7	361792977.7	291828021.4
13764058.66	17625971.67	15893726.21	5874400.121	7911757.907
12384007.62	11347747.74	24498719.9	20226632.49	14554264.05
73320.71255	42682.50466	141377.4801	221740.0175	31036.11381
638332.0831	806214.8488	1098313.125	697784.9953	659731.6374
750556.1744	987080.5607	1141325.383	468376.6393	727109.3068
8411148.686	17517445.85	14264259.94	11445039.59	5056103.656
8637581.949	6250728.776	5042841.692	12713576.15	2911661.31
510071.9047	5059853.034		258206.7511	
		513635.6794	184690.4261	
183290.6089	127063.4995	149270.0774	1145355.066	133496.6804
3269037.152	3708994.906	2945599.16	2423710.941	1361889.775
123566407	225745482.8	125899656.1	135662338.9	153384950.7
490082.0277	581077.1934	738009.2129	1371930.272	444607.6479
12900500.44	8142083.031	10901742.78	15582390.55	7070555.571
518810.9072	1323088.646	529020.5207	509233.2766	457471.9152
13608816.86	29367320.9	16772241.28	14237785.04	21568656.83
32956520.97	43299503.76	42982846.26	34717562.33	38914763.4
8938334.667	8803566.772	5882382.896	6673616.559	5601547.156
3679962.04	6011416.324	1174174.259	2105348.737	458978.6505
118889786.7	92092091.68	141015575.5	163828524.2	193140373.1

			1078719.721	
34666719.5	48865733.56	39844972.6	39535091.51	22543174.72
20068743.42	22572553.29	18153112.05	6453965.677	9850385.913
472271.9529			243071.3024	936508.593
6046432.501	8544841.272	12197502.82	12679202.47	6299241.684
7562473.928	12840064.36	8853316.451	11976076.42	5107293.691
	55392079.32	5643968.35	25628602.22	
2395626.61	2508901.383	2761726.079	697890.7674	1169127.824
4052211.7	5818555.35	9094982.188	12778302.2	8108970.306
8802427.408	7608191.214	3561226.227	4304427.817	4704445.209
937857.4576	1377535.64	882271.7557		
10853075.18	10927934.57	12890723.76	8275085.45	18247954.47
	790514.2865			
2279683.637	2946160.564	3098271.989	4592356.241	2783436.928
	518852.9885	669094.4618		
3409578.394	2936522.931	4565207.54	8227034.163	2723682.659
395481.6698				
1750295.609	2054122.829	675790.1729	2506201.774	1066045.015
1175596154	1080032621	994220281	659376225.3	534555276.2
24393479.86	24180760.59	34638683.7	34263268.86	15969445.69
121272.418	139843.199	270122.3354	802368.3449	358117.9159
196663383.4	245847481.6	193505877.8	120301839.1	149127595.9
17503.31038	7297.996053	9380.605267	53879.87368	15581.65886
59534358.24	104943583.2	51684961.55	27158539.89	48882570.92
883989.0766	620892.7001	250220.7496	1669743.213	494680.2401
555882.1532	650728.9147	342049.4305	637534.5847	392613.7573
1364091.436		2454252.361	1272135.085	1405105.982
997421.4035	757054.1633	498506.1181	1021210.543	445990.219
21208890.39	26608161.44	29620599.12	34077103.98	13646461.65
22312867.95	22254083.15	15651840.4	39675538.55	13469258.48
100798429.5	4246827.142	6757396.386	1924004.652	2523409.417
49527060.87	51291466.46	45728059.77	47629063.1	51357714.55
596622.9314	428233.0133	150552.912	934526.2145	489685.895
73752168.36	93998056.75	84021551.27	136574981.2	145391034.2
13673537.58	8926445.881	2567703.23	11410285.13	11311177.58
305938.941	4795079.149	408439.1609	687842.7272	
1084373069	652310559.7	994011534.6	1188728210	925979141.1
103045.5006	7632815.89	1579860.943	3373019.663	1524162.025
236055664.5	164732452.2	127905688.5	225807128.3	228140777.5
834619.7834	1371269.603	957705.7999	517097.9702	529491.7509
16176369.39	31596335.06	20439225.22	19990299.5	12682537.03
173186.3669	130708.6006	97688.31937	241498.1858	
413458.2978	518515.4895	1522468.27	1118640.399	307501.0199
227426519.7	199134713.7	299157412.4	222008049.9	182946977.1

877074.0064		609246.718		
2480739.796	2871186.729	2875459.96	1901826.213	1555451.7
17827342.76	16749688.04	20457396.33	25231864.67	15559523.77
6670118846	7957684801	10428479892	9117961278	8030818514
66310574.46	73676132.09	67060091.18	51511211.75	44245242.67
199328.6519	129212.9767	205473.4612	462277.789	
18618772.88	14175923.87	16560612.62	15016316.34	23047524.7
3619973.181	4107177.239	4711718.977	1904964.406	1675288.348
225103173.8	296592642.9	332018630.5	307026084.7	272292372.4
		205764.151	968117.4324	1112609.527
1831380.27	1386491.462	1611844.293		
3219914993	1989950906	1866014582	2930569743	2849798364
195562.7294	77982.2679	246181.2859	172289.0784	192855.7098
21107962.71	31644646.64	19838920.66	18349335.86	19776049.93
2278489.039	3781976.152	3189613.76	880620.6849	2473004.161
2660192.34	4858292.083	3245236.993	1460568.226	1580583.872
6035987.471	11432614.64	11419733.17	7823574.878	8521131.21
		179134.2593		
1942182.762	939216.4733	288664.3983	1357450.478	876325.5815
1303404.127	3564494.612	1631012.035	3188043.366	1863887.481
1779510.764	763900.8921	1654916.979	2024713.138	1741585.089
354437767.6	354327985.6	131179395.3	556276158.6	242834281.5
7112999.269	5590026.492	4725272.917	4686649.077	1845976.174
47049.48018	38546.2468	159365.3348	181493.1834	104826.7985
1025573.191	1565419.81	1789445.404	2324761.076	660932.4952
30194605.88	39604254.16	15245657.68	20743347.35	22424365.24
772171404.2	689498198.8	353616243.5	756723354.4	387215639.5
1574862.635	3217207.626		2364991.868	628210.5998
662774.6967	1065052.592	575346.5938	1262583.908	173589.0617
27742436.61	23287988.89	6060258.678	21951895.93	8924899.612
1087428432	1204917257	1118792999	1324355873	757389213.2
446305.5763				542918.3925
3926926.593	6129077.761	2613934.394	7986034.975	6461568.719
13609877.04	17050663.46	12302636.01	11762269.31	9616730.31
403997.8944	531354.346	262672.1091	755143.7585	903358.1581
1008824.692			353505.4795	
1482926746	1334765306	757981775.6	2371022290	814996814.7
998358.7957	57240.37509	1632161.431	2025437.517	814413.8709
18005537.24	13537000.78	14752755.05	9607576.06	
			707462.2909	
	32912.14272			
2215598.058	4608403.276	7889049.732	4120662.234	3579710.858
	1443288.848			
9038496.391	12878989.35	7334824.151	26445052.88	8981494.655
461641583.9	371383329.5	1027643604	605841798.6	680843520
70315468.26	60713547.37	53765825.63	34736458.62	38666176.79
	572473.6299	1753198.155		500049.3015
92510282.79	161195113	134706607.4	251736733.2	172657291.8
162211310.7	176823012.8	154722132.9	243400168.3	106001288

76605.65012	42832.92182	37975.58178	1078449.644	69191.72916
2522755.891	2502039.367	4293157.323	3147367.147	1357704.756
1122220701	1201501960	654296398	806159836.2	953419745.3
	2560224.127		725062.9554	
8855921.916	7275915.743	5714392.175	12528569.37	2458404.824
1992251932	988277820.2	1796389392	1045591958	1130417438
23715136.73	31352991.92	28371300.22	18776549.76	19317733.15
	216588.6157	381351.8019	62264.10315	304013.1232
300449.7297	348452.3224	278647.3291	1083253.778	218989.1016
18995683.75	33924070.88	19033795.53	21725992.46	14747119.64
8950348.392	7737791.436	17401854.69	19832640.35	18055308.39
1473330.028	2270356.551	1234641.118	2231944.425	167919.278
2344094.523	5178660.748	3229357.35	3744997.488	1647638.339
1384136.265	2060495.679	1317843.119	1656910.916	1513154.265
407965.8582	1509828.037	1410342.106	1992861.939	243529.8173
598250.7726	706216.3187	447262.9039		293061.3882
2828986.737	2951754.168	1195565.268	12937014.78	1668830.856
7799993547	5142769037	7323662216	8061429055	5824442831
26301907.97	84713062.69	31970930.9	107108750.2	88252605.29
567494.4113	2721450.098	1876625.549	1084205.282	482721.334
14103011.56	12499.38386	89465.9219	10104839.22	7076444.009
	73397.8374	1116782.814	635948.8758	312734.4112
4508742677	3457224196	4476015349	3163117696	2866319101
1429407.171	1402257.641		1196096.897	
5868966.531	14913414.71	9979542.248	11032963.09	7224253.51
18630636.49	23586986.01	25490907.15	35386473.02	17499556.67
2566504.49	3647614.376	2953263.257	3557720.707	1032288.3
855700.8515	510344.7614	534036.0438	1897717.662	652489.3989
765278.7932	1286901.013	799563.164	776399.2581	1293957.653
6779641880	9035376256	7510545134	7304771350	7160869619
478132.7514	303819.1057	298986.2211	190815.0213	143749.405
1425390.556	1625518.581	306722.567	2388908.387	236418.9915
33199306.58	22790386.19	26910100.4	38892048.81	18242484.06
14945441.56	24343060.28	19835386.22	12957712.08	9061650.953
181062.9946	147281.3177	365163.0587	436895.9531	679855.08
32606473.03	36461294.16	35847451.82	31820674.9	20377011.46
3413098.531	2830234.339	5813907.527	8311111.079	2367180.577
3066932	4168094.432	2220584.211	3166325.614	2263104.706
1707245.318	1422201.322	4327030.015	3982781.683	1386121.21
1892459.596	7692154.15	1287412.1	1782276.468	1468732.557
34575143.92	35555066.5	20279771.32	38978723.25	17859443.78
20112552.86	32594779.24	45393179.15	40075460.89	20257070.45
1199228.089	1997170.23	1012148.014	434461.6847	1590184.646
1291934.858	1696812.993	405514.2651	592556.1287	872420.8674
4052070.974	4419342.531	1245235.937	3141493.003	4575975.688
26734112.01	42338218.27	20399948.57	32632175.71	22106235.54

1509139.612	1718377.464	896394.3258	554289.5276	944511.2205
343397826.5	301827956.5	156723882.5	521332623.8	435241998.7
2737314.08	3705285.963	1074705.457	1145169.809	9778738.782
4386392.386	4312332.929	6543182.75	6303901.988	4585597.05
7908.094993		548639.5464	670459.2272	504811.1608
11575363.33	17841866.14	12212292.41	15680629.9	8994126.759
1494196.666	1286376.232	952346.5704	1434890.043	829407.8828
2038600039	2546264196	2293870779	1858707244	2290950348
218248779.3	155474690.2	152445884.2	166514916.2	47276838.8
21739545.48	17104999.21	16968438.14	20645310.18	14028435.06
970104.6684	1222656.858	1260574.217	1017316.809	938571.4202
57789625.53	19364315.1	31555589.72	80162805.85	52029446.32
17921224.61	24132987.25	23150290.68	17337759.26	24341848.87
208342.3212	780203.6771			
1467580.913	3594554.492	2168629.026	5791629.019	4106015.025
7209011.76	3527572.716	4075186.224	808556.2308	3154767.675
15525664.22	21704177.09	11670554.62	12259384.67	15222389.89
3120324.993	3798257.074	2971315.192	3800236.275	2945481.294
6112372.882	8898058.041	6436748.616	10905430.23	5943659.182
880247.0484	267669.4854	2344233.028	1599167.76	905500.239
4356624.94	3988086.068	3698974.172	3810752.579	2951082.096
11579617.42	9319237.657	10240765.99	4030784.575	3640556.338
587628.1254	2538840.601	719787.4674	946997.8161	405472.307
57497.03498	90872.99156	130683.0079	97450.8254	59641.33272
29443210.15	24819030.58	28108557.55	20132763.77	19959668.13
12082170.05	15922370.91	14825335.95	15631137.74	12131651.37
4969872.763	7008491.152	3925819.949	7523535.041	6172772.414
341301.9322	1541255.356	489122.0539	505534.9729	
7139504.293	6675696.087	7388600.538	9589354.601	4105033.437
567447.6124	995280.496	559299.0763	545022.1765	
			976968.0628	
			468802.8933	530796.3929
1043362.34	1499684.596	2359432.986		
19783040.88	36225775.22	11015472.26	45762201.48	18263552.59
17250418.89	28363284.55	20182015.75	24216279.52	16745688.52
1940314199	1102763191	834324638.2	1757672870	1239954697
5481465.349	2133095.832	4357875.668	2964765.892	1583943.418
2737730.508	2573085.394	3246927.37	3596326.519	1242718.716
1673108.313	4128153.735	5565462.076	8352062.568	3592055.21
5388171.379	1195432.367	7788930.383	3334589.433	4263444.747
1327225.577	2375870.35	749075.5519	783891.8376	984390.6644
4906222.701	4285288.292	5804977.387	6761520.068	4223555.421
1449137.043	1815613.536	2280839.027	2343040.295	2147271.959
	201370.5187		150979.5278	148644.7946
2757408.359	7881045.594	2711839.258	7108966.821	5517521.509
11039247.34	12718143.53	9288808.887	11875411.59	10347162.09
830822.2551	608314.7907	681607.7067	965763.3779	1986182.39

241721463.5	53457001.56	91704665.41	366953064.7	117991995.2
6686896.462	11404029.23	10424854.69	11723375.79	5909800.961
5375505.061	5728780.32	5713326.037	1812044.117	971784.7844
199299.1257	1337799.56		716905.6858	
6144936.198	6773944.411	5501639.971	7774752.25	4223822.737
448808.7763	500881.0141	391945.5554	727308.7301	490113.658
3350116.064	2946274.421	2842903.099	4032913.497	1991132.088
2126650.99	3779655.146	1288637.375	2570162.494	1249905.366
4580470.239	607902.7286	684309.9714	8921829.28	973844.8939
5201331.816	7258844.421	3238566.127	4602141.407	3809647.958
1629901.554	1954218.069	2450148.251	4415829.345	1695763.877
186054.7848				
2638705.981	1985426.484	3179529.654	2185230.361	1565910.012
551763.9899			575704.2812	101159.5952
39087438.73	49259779.7	34740318.1	43871725.49	37290404.13
28636000914	27405484030	27218729394	38933656274	33315079419
	1199918.808			
1214857.277	372447.0931	663910.433	496237.5282	805751.9519
1743872.045	1149230.425	1515711.695	1022899.054	675266.8627
18438942.11	31392872.62	18325686.41	15261547.94	12466780.57
	2797307.19		2220781.003	2711124.258
		768503.8784		
108440627.7	93242044.99	101075252.7	155309882.8	139235750.5
9721409.019	15978903.81	13500923.39	14344766.33	10647994.1
873770.5001	2555535.561	841727.6111	2316506.554	3320845.86
1927890.83	551548.4739	6471848.582	6847789.703	3734979.874
48280940.61	52566375.69	36121226.29	44784485.97	30341653.8
105136950.2	109115469.2	100120590	128987946.1	180333202.2
20920599.69	18140825.51	11851710.2	13478859.05	14815770.45
13025935.82	8841351.822	6272220.957	6662356.81	6722875.355
1871264.447	2545435.99	9275716.828	6311185.429	3913680.21
29868819.86	26204946.62	12380378.25	13844377.65	16571300.12
1270779.957	956198.3773	1021167.765	2288957.094	591062.3805
482641	233678.9382	551741.0559	701091.3538	483899.5529
1178591685	1044903883	686187918.1	1004829836	955635999.1
511406.5928	1616667.56	839102.3824	896659.7046	
2627730.481	17698676.55	975749.5515	7508814.891	2270455.33
4712373.057	2045057.846	2968891.423	3118829.367	1467170.586
638782.2164	673205.2995	938927.4986	638944.0721	728224.7024
36362039.71	30155874.36	27911800.41	46279326.61	19193724.21
57081.25865	57560.50312	54881.63992	204424.9571	64694.75118
1521432.86	1637218.122	1405314.513	3665270.662	1830267.097
9672216.147	468610.1158	2234892.655	7379225.999	1588884.169
818050.9108	1493368.554	728220.3177	916630.5908	627652.249
3464640.412	3148042.722	3291010.531	2305074.78	3535514.82
223150.0547		393451.4992	914362.8328	916031.0261
10095876.23	18914149.78	15892726.61	14593005.84	17750340.83

428429.6154	393557.9801	281215.7805	515088.9242	346359.5501
			1178274.835	
		26019.53935	164865.6412	
4713081994	4134862440	6503684886	4418672821	4292994811
1120743.666	1407841.684	319454.4922	1315968.848	1119100.125
3128942.863	2022234.666	3144934.791	5399976.086	2501332.133
12819595.23	12312425.72	6866516.748	13121914.16	10616445.97
222289.7888	220558.553	914644.5251	2413580.125	172164.2261
6203858.921	8420562.306	7139254.074	4790350.127	5272385.797
41435045.25	21535949.24	9326639.986	17523370.01	41957367.53
	10435870.94			
26016670.7	34399063.89	36704468.17	37577379.28	20272908.76
35521522.9	55048337.1	45867117.34	72524332.17	51942131.33
12357126.7	19872874.87	10512227.46	22161312.1	10335487.83
22921772.63	28384584.39	19710148.65	81096184.08	29430174.37
418879.4787	402268.213	504679.3136	549049.1614	329828.0972
261237596.2	280021053	138352587.1	78149023.17	136703645.9

Intensity M_LCL_6	Intensity M_ZYH_7	Intensity M_WSB_8	Intensity M_FSM_9	Intensity M_ZSH_10
	287329.0897			
102211629.5	89120434.26	130263722	238658017.8	175832490.2
515902.9188	359594.296	502457.6417	387683.493	515161.4071
			110746.1662	
2348489.115	4955090.356	2344961.569	3132404.311	4218463.476
1713787.601	1381527.09	3828038.427	1454792.748	1801437.607
3577378.352	3104059.207	1951370.693	3229040.629	
91204200.7	80001675.63	79842354.59	74793333.14	72617111.29
3282918.466	1991821.124	3651853.659	1714441.05	2582069.866
2689521.105	2772132.814	2173353.348	2619594.342	2155719.608
12576074465	9829625644	11118383401	11381444549	13140946626
23282.84561	22981.20941	26812.76944	15850.96094	11633.19166
1325087.901	840785.0541	652054.1843	747126.0321	1126123.896
2537443.872	3698169.588	1772152.301	2594183.784	1429562.168
21532568.74	42316259.29	32129912.78	29344096.4	37632595.74
	6187322.072		349606.936	
	216752.8312	539613.2478		
13259867.14	19948328.27	21184403.28	18580765.24	19268931.62
2291819941	2035490142	2442065818	1256891585	1294189571
13013188.14	420590.0832	796834.6515	534654.3719	8644733.685
102896164.7	28445499.53	26976840.89	16322096.48	47581804.83
2349772.861	3722768.934	1349729.022	1330354.149	2672300.116
3044829045	2782619564	2847885261	2421600354	2951089701
19640.79848	52739.32991	12423.44214	18706.09259	17375.75736
67074636.91	104875549.9	107826907.4	153541843.5	99868518.62
36405446.74	38075463.02	48305315.02	53128117.69	48494658.43
			1846128.469	
6232780.753	5518562.256	5938985.381	5804098.925	4252664.048
10037626125	7355823794	12209665657	7753554315	7600232297
		308231.8714		289290.7375
825280.4083	1779690.646	784710.9845	1574636.882	1803717.369
		1497857.969		564024.4056
		2470424.622		
4544592.155	9262432.036	7544823.273	7217439.118	6494666.928
23598858.45	26022412.04	14666116.44	18069140.9	15189746.1
79523.98932	593741.4196	346927.048	614567.1698	257537.8492
20647089.53	21625754.03	24278919.45	10709347.28	21234947.37
4455.574894				
17846476.3	15157918.93	20542661	28710536.33	9992051.312
3675660.485	3272351.422	5208338.488	3322718.318	2999323.936
59102448.41	121323372.7	55473324.05	40343326.41	58443249.94
529518.4617	246986.6829	432206.5124	555114.2241	387377.629
76423.60627	152232.1619	85516.26377	217542.3801	204806.4572
	174771.2398		368647.1703	96373.92027

				36810.05841
217752.7075		252693.2239		
2036522.596	2745188.092	2230692.412	2805080.689	1717157.74
878323.2247	941422.1651	1332012.011	1733959.607	1151613.027
649207.6656	689006.1421		1213895.715	1281520.161
661168.5108	1080982.125	1648251.535	1010238.259	
			143208.2175	
134397.9397	355383.4235	1024343.999	2042132.425	
1367772.166	1350072.874	1486879.995	854358.644	728533.3063
8961322.621	8760002.394	4150604.597	7151149.927	6682677.777
1365252.469	1931246.306	1716017.64	2275860.091	2238708.232
2253589633	2572280672	1789868025	2396264780	2804805263
2999676.415	2578553.641	3596780.033	2148104.345	3347122.296
851748.4567	880459.7932	2526161.784	2373124.659	1009089.171
1509463402	1786716321	2059281573	147961.8574	1389407245
			534667.1338	
50717508.31	15103072.95	24932470.83	44326711.12	23464578.61
259010.4401	2298000.917	4843765.581	7117660.648	5005148.315
13713211.64	14172731.29	13505360.77	20034984.02	9244977.059
148239464.1	152596733.2	191912598.7	183700921.4	207991116.2
6869242.655	5836265.602	3725445.816	4884345.349	3945863.112
14040687.27	8481082.421	7234926.606	10434606.27	7086846.182
254874.8895	78372.19205	34303.18664		31833.49971
374383.3435		358781.5588	101171.2934	191540.6834
2701380.851	2630495.08	2327520.19	4033223.931	2794956.326
5179796.163	5700237.058	2157405.509	5698697.196	4358967.857
956498.6512	1294495.453	362766.8456	1703826.627	2744551.262
			74336.24259	
4893200.973	3951384.323	3439113.36	3824821.042	2956306.353
3541994.828	2408827.902	1882915.472	1575555.201	2803548.037
2077732.393	622400.003	677773.3991	332434.5665	801845.1566
	14276948.65	13208694.35	2032505.87	
466169.5543	424869.859	6668188.974	255607.0752	487849.5027
28711903.31	38339402.81	19747327.23	24910742.68	18910161.07
2797453.123	13531553.57	1145639.119	2459189.36	963188.7946
9339848.658	8802821.033	7151135.62	111700628.9	8132345.878
22896232.05	26252097.76	37933076.02	22911402.54	31018689.78
2938929.062	3950753.904	4241327.723	21501166.34	3506863.162
		430188.3832	1281229.874	
565922.23	1487542.42	27470.55354	538940.6975	45762.01276
594209.1965	495589.0373	487513.3559	551485.9353	479696.3365
283276.3839	161365.4329	348936.949	171649.2133	227743.3124
4128321.422	2233553.966	2855167.134	3264734.89	3177818.203
1429067719	1210819984	1435984590	972952481.6	1339021901
	186016.6251		547109.2507	705602.5029

	1326018.999		1187541.644	2061635.77
671213.386	1014080.354	963219.0916	1454940.131	1391241.251
		94724.95369	238319.0864	83150.47107
37593545.56	40533730.29	32987096.6	34910125.76	25128020.02
384264180	321779251.9	269956756.4	282933849.9	224051372.9
16346326.32	11454872.47	10499983.33	13270202.62	11505306.58
7676507.526	5736062.242	8524810.594	10511791.2	5156946.314
17923144.97	13474157.3	13555620.97	14796703.49	11903725.46
538569.6975	600177.0814	600446.1007	555951.3883	551774.9503
254384.9974	120556.0745		450856.1841	
9491470.197	17463723.19	38554209.06	38367492.45	11663118.77
27674932.96	34860158.83	29669788.46	30372502.62	31019297.73
8950248.283	10161662.96	10948187.13	13865185.61	7208432.119
19523.48484	17568.25411	43061.09632	41477.82483	36632.60928
16382618.6	14333875.71	15390950.79	17362449.44	14054090.91
121573.529	131564.269	98488.28582	125914.8077	150506.3718
11800266851	10045106588	8870106096	8876641410	8266884879
	121384.8804		127804.2999	121465.7148
35257.29776	30913.28545			24976.45874
1944941.272	2739759.05	3081024.725	1921400.204	2543299.026
841478386.1	950571396.3	1677905374	1152441521	2023793728
479794.784	712387.462	217370.5319	286421.6912	253395.8172
2577706362	2987159422	3216160940	2873255200	3553680285
77449.76185	229534.5476	44832.67576	89336.19395	94323.15642
629830896	861673157.6	1118562425	1014301741	911203192.1
	85156.73686		81502.30125	
158881.0606	315818.3935			344141.4674
466793.3472			203760.8682	
718131.1387	1273235.717		128562.0883	247383.5437
5809886.734	6008728.548	6902739.281	4713333.759	4580674.003
17210504.91	4879833.194	11916816.3	9746957.482	3988747.779
3955150.344	3339452.495	4038939.074	1988803.812	710724.9635
450654.997	374285.9596	392555.307	613330.7922	498261.2257
14461134.46	13547060.49	19534302.78	26000601.14	20422616.47
41834071.4	49479077.54	22705208.92	33232132.08	56879712.03
45461.77699		50762.21678	70953.95404	90901.59192
9540514.511	7913636.502			4919508.148
338702.4436	717361.8441	544514.0632	477989.9344	622982.6015
272136.7142	414743.2564	328458.2347		256369.6597
285302528.2	302203151	333593538.7	317563123	252744528.5
5253001.5	2429403.574	6060588.298	5747747.686	4363167.026
155148494.3	134668874.5	184552207.4	181515525.2	232625973
3339056.218	4425067.378	5038753.958	3418230.405	4243927.567
4463708.511	7729002.068	4216321.847	9659418.068	17630793.84
6414513.702	5291402.781	4079841.235	3801288.871	6536109.726

307029.826	729509.0609	166167.773	402616.2057	297525.5079
487299.8962		374423.5869	307799.8858	377741.7424
15213928.76	16918008.98	10387423.79	6437141.687	10146924.7
	224725.977			
132337.366	224206.7327	253394.3853		181097.3021
10270473.62	18357011.95	22158698.18	18870845.05	13775100.66
1460741.567	1449911.41	1650720.869	2082643.472	1821181.329
			351737.3793	291981.8444
134114857.9	107887750	124734570.8	120621521.8	117956674
			68642.43986	
28713456.3	24303902.23	28476634.82	20575807.9	24738454.22
6188963.569	5132386.249	6508772.003	6588416.488	5102531.869
62886506.31	67938635.96	91566403.42	72020594.61	69667275.49
845733.7099	692858.8855	1293512.757	1261549.324	739004.2989
1135736.617		2376015.725	3236577.554	1597209.023
1465784.876	1383475.841	2570278.812	2710068.762	4231004.248
1774435.057	3071980.796	4458394.445	4260563.67	4414788.629
			126629.9247	186180.048
1617862.034	823170.7131	1288117.456	409985.5321	486046.5303
4267646.78	2875913.402	5211072.105	2242482.62	2969318.372
361352847.7	428686516.8	490819108.7	418224630.1	544598202.1
904416303.1	1461545107	1634842054	1533740051	1161559508
55001.38723	49258.84043	90672.62531	58515.65347	117506.1388
116620859.1	135311748.4	119559993.2	128867441.6	139341736.6
19392397.37	24253425.88	26393441.84	25633582.68	31355176.1
43794.97091		64919.56455	58243.51769	
795467004.7	602785771.4	670219329	570960826.5	829502389.2
	3092634.275	4240430.687	5031617.058	3503285.623
329727034.5	110199526.6	254494643.3	323871563.2	374711459.8
6576049.437	5148030.29	26908209.78	28124944.1	10594709
9171113.716	9829456.16	9989291.655	10553432.43	12006596.55
5678491.868	15812397.96	14004918.66	18674159.61	8164140.949
1055911.926	929287.1699	694309.2778	641599.4463	680826.7051
2986495.676	3574026.636	9868193.876	9708325.391	2975680.026
69215788.13	66300811.5	61219409.33	60952963.45	66206906.26
91969.5977	99655.49797	46603.80749	75457.51861	98419.95618
146019562.6	132808531.6	125224553.5	133991362	156588129.8
2837191.505	2611498.947	3508580.587	2212512.156	3963221.695
140577335.8	125174707.7	143879466.9	147920616.7	124613337.7
			478878.4473	311752.6614
788006.9948	628873.4301	1200674.441	1882982.969	691526.984
2287680.458	3418383.063	2851474.105	2765967.976	3330045.083
1690485.509	1575606.838	1150150.56	1609544.612	1526520.16
144456689	180165582.6	118240655.8	135374648.4	190145712.9
950385.6721	953692.5081	1032912.222	1278025.475	1409160.228
760679255.8	866043542.3	795461310.6	684794372.1	1053289388
8383600.412	5913750.12	11381982.89	10406045.9	7572556.083
6813806.459	6147645.502	5518588.282	7194047.271	7511629.966

261017.5814	585377.9154	731224.7746	514872.3375	538124.1613
468434.3492	1363404.326	350754.1701	2277874.586	1330179.159
48759718.04	35269841.07	44332113.29	44009177.78	39029390.85
6476.006926	259149.5615	348109.7942	1234357.133	893189.2254
388551.8392		255424.7107	297570.6561	297184.5854
27488423.87	49789154.37	26583858.83	43495043.07	40583714.03
5696880.296	5404635.357	7385435.187	10490948.95	8120321.095
442254.398	991047.5092	402146.2275	1543883.652	2099262.248
	130807.9953	146722.0917	146092.6468	124589.4946
227979838.6	343429504.7	280802824.1	321368295.2	423654543.2
1091796.335	834803.1072	1396042.315	1746209.825	1154790.431
4043147.67	3218278.844	2002179.895	1420488.591	2302146.702
			223833.836	
282510266.2	1265492839	467437263.5	142717063.2	1552218394
19135586.58	18740303.51	23460785.52	20076302.27	11378241.18
9054583.272	6284385.277	13803362.49	1368973.101	13146321.64
11430285.64	1270535.897	1376157.117	1267425.793	963112.8449
	469351.2662			
1519850.972	2136627.571	1977489.575	2493329.213	2487455.579
1409241.145	1659935.566	402207.6046	2113168.418	3926140.104
1908595.304	2015131.264	1226313.994	1566704.603	2804449.807
13672449	15768332.99	17102968.57	22741743.56	17415995.77
6507766.525	3303317.317	3267425.073	3497483.472	3292633.301
658317.9569	1097732.827	1415621.731	1756195.455	1325312.111
2461348.711	3014406.094	1197620.744	3274318.421	3786186.357
103471.1793	137802.6404	157632.7417	360541.2689	183998.3107
14083587889	14827792797	13124618029	13666216067	15502099353
	875507.928	207402.454	436620.9668	1412600.203
2382126.229	1594365.297	1394982.694	1564538.998	2122746.958
18475.79587	453793.4661		17072.6297	
412045141.5	437026616.9	568100401.2	559590383.1	444354777.5
318189.6368	914739.2949			
530746.8619		369748.5999	612862.9319	807527.9162
4963191.648	5245721.67	5873074.426	6785867.3	5188859.243
2679009.294	2856383.545	2065786.742	2497280.803	3145898.315
26413711.56	31700220.53	13436382.24	12155810.85	23158686.59
440124.2626	411517.1513	517396.145	557868.9077	725267.3952
409990.0868	1326406.976	801777.9181	1313098.959	874974.1679
42050254.84	46881126.58	41567517.89	31801111.93	29136709.31
6193217.816	7012188.229	2756347.278	15942840.45	9953111.943
5229849.574	5068514.663	5723841.068	8262875.318	5667906.565
1456311678	1596735657	1396009934	1314094784	1151382027
4247643.308	2229881.415	3077720.26	2608243.328	2471687.438
50325.77916	462781.2719	296199.9685	466018.2001	1421060.444
525107.4701				617967.3139
90168296.78	114623447.1	106100784.1	113910614.3	115337463.8

966255558.6	960469518.1	1321991427	1240175129	908806683.9
3129645.03	1458905.471	2516431.483	1282337.861	3274521.037
6785185.122	2690879.87	3575788.85	4639075.396	3447734.774
2234273878	2159125618	2059580865	1814048685	1693090428
62051.58726	99511.23075			131763.258
488973.7985	2652154.445	1987659.091	5201076.781	1446986.905
4648338.124	5239235.224	3314258.988	4534312.377	4988842.929
6269557.293	3859152.605	1958607.909	2679783.279	1565206.301
841615.1106	828404.3567	1259862.328	1631231.336	2119184.472
188775344.2	279183593.8	388435188.4	357670527.2	289176470.7
40393284.2	43957267.16	18475267.55	15165835.93	22526821.48
315558.4052	392832.5531	412952.7758	389309.9326	2783689.98
		260036.0021	305478.4284	492309.9946
85527962.39	71595712.54	50577771.12	97107039.72	87067463.4
1465048.312	145047.6466	3221563.207	2308655.759	69526.60298
117393470.9	171574627.5	217837007	187081638.6	165348391.8
		1340240.007	1080496.658	1429749.395
21265589.59	17221576.82	16259265.85	11617536.21	14181523.99
7762915.678	6654675.099	4542911.13	7519267.964	5911283.972
11110417.26	8937819.092	13593672.19	14387080.29	15695993.23
3371039.72	2021202.253		22567968.48	2352810.971
	2040130.143		2013918.193	1496121.231
803528.5054	1958913.084	2075750.355	970201.9691	2162755.171
5161130.022	5172980.207	5234671.738	4652068.147	5887021.988
256175.0435	520765.8123	317503.1826	532658.8131	377364.1122
		744108.5854	496218.9424	799136.0646
323304.5735				1976656.267
102147218.9	132876122.3	76344680.52	85475407.53	84700743.54
182844.1761	99066.16959			120222.5402
7150839.028	11294033.67	15370368.28	12683021.32	10503579.26
70002733.75	54665809.88	58805851.73	47698000.62	49579635.26
54957975.97	77739132.06	75821340.14	90563037.7	76442923.41
21316574.23	16650645.32	24145600.37	20194581.62	39617979.53
	341747.1154	659231.7571		1116048.481
469751.3983	101378.5348	56938.70813	43090.76259	42144.29539
2566866.721	1246261.428	1512428.312	1511728.987	1625390.433
1395058.373	2074226.094	1090211.331	1273727.094	988666.3514
16119825.93	14110363.98	14700967.76	14127986.79	14982501.21
496406304.5	482214860.8	503957694.3	496157435.4	416634915.4
29652698.96	18296503.71	30349602.15	15828396.52	17282254
168229.4663	182410.381	143595.6975	260552.661	219252.6279
3414960.704	1747162.923	3456601.225	1240699.802	3011135.697
86892.3951	816982.6701	528319.3258	596090.4534	501175.0754
19364458.51	22613604.38	23828946.82	36775290.61	26132020.02
12491804.03	10389189.08	11224139.28	6604576.835	9759385.957
1773059.636	4314531.85	3841457.235	2255692.257	4867140.382
1755070.686	1334285.917		2098580.725	

697040.6293	617586.4366	698139.3029	800015.7768	652957.5093
1657816496	1969527999	1427812514	1487215857	1781087372
29367526.17	16903056.46	21742216.19	26047323.66	56344171.03
4397611.535	12806169.46	6416822.294	8130898.363	8177806.215
4208502.357	6289448.957	9681343.238	44168211.27	8308757.506
6239265.736	5590253.568	6234008.103	6600217.346	8882970.968
	354264.5839	375231.1402	441074.185	599362.8613
	192233.8745	136656.2624	137421.8817	172308.171
3186338.948	2854641.065	6181071.214	6215087.023	3480195.904
1930636.21	3823262.252	11918586.44	1939374.513	
902644779.9	768805583.3	753447393.4	881449900.9	828786844.7
8787540.454	10773052.32	13687198.85	13601604.25	12202702.06
11915048.1	9742723.279	12602066.58	9097240.087	19759616.82
416432.8926			1074908.15	102578.4083
		269268.5378	208655.99	201256.7181
2625498.895	1170575.724	1724450.691	1975097.196	289083.6536
2556542.25	1700369.577	3296103.064	2071316.57	1505791.026
21057346.49	14568811.6	19946472.55	25719436.8	18725965.73
6073873.933	8966240.608	8247175.793	10407534.75	13227729.01
8190390.696	8682519.926	9511274.525	5198277.459	9757623.588
		229828.0993	383580.3006	
11082586.55	12907427.44	13755978.56	13823270.11	10414053.36
13332158.16	20114753.91	16351035.74	23845330.41	20157569.47
2599186.57	3265644.865	2552595.242	2180889.895	3369298.253
62182.96804	72169.5222	95143.72587	120572.5872	71043.55851
1007787.882	1636698.902	3024847.726	1606649.322	1250988.093
594894146.9	595279516.8	713639905.6	766192656.8	895192245.1
349074.7472	618385.7989	605808.8276	743943.3173	
74603536.55	91548705.55	101697863.5	100508924.3	112841701.5
354710756	218699665	361434272.1	421645335.5	362901948.5
11905081.66	16769894.38	12958577.08	17722892.45	19859216.86
1202028.671	1006964.348	1766139.611	2033910.975	1144586.321
	1713320.739	2037614.066	1632405.166	1605722.194
41288311.61	60251055.28	49883513.97	48285164.61	44064825.51
290804.5787	2473418.209	928156.425	413741.0632	355374.3016
1947533.289		1772481.128	1619913.055	1674489.092
922268.1905	557546.4393		1302388.693	
1373389.332				
96485066.43	83028367.82	72654471.39	90602347.75	71801755.46
15374748.58	10373346.53	24169458.07	14942750.47	31095511.93
414370462.9	349845369	323498877.3	237091704.4	497446236.5
1456842.702	2216658.039	3024366.663	4367039.152	3694073.499
683053.7594	2240705.385	984161.8851	1382571.994	642974.951
1475771.445	822241.2693	1705817.907	2417949.499	1575720.677
294133.7786		235970.3676		
16172512.57	12547263.58	12870894.68	8348681.784	12617060.61

628546.433	1109204.984	1589195.639	1079592.334	1271216.424
191978.8989	632489.1251	268613.2396	248595.3637	264827.9295
9992015.584	9163317.151	11060104.27	8564098.732	5381706.408
1088531.568	453510.7934	1663520.852		708221.2345
6156480.661	9830059.076	16778974.42	14545069.19	8319862.043
		1601.367972		1566.140906
685313.8329	224297.9956	642896.2398	717526.2274	739161.9474
3136018.307	973510.3308	3259170.508	2714116.875	1495881.713
105902.1148	1380817.192	102107.6005	168348.2724	117607.5721
4340733.106	2996373.384	3535627.853	10080856.63	5220216.809
1977754.812	3023733.003	1839547.373	3376377.841	3150181.981
2954172.867	1927807.767	3378667.884	5708004.502	3086052.362
553295.2488	592947.7032	671293.0291	189113.9014	559960.8717
163584938.3	272809460.8	345702254.4	251753932.2	232744111.1
1738689.219	588190.1489	1006745.309	1260957.911	2124054.291
23670254.45	25512728.44	27730433.1	22861022.41	26268872.51
1660908.764	1188431.185	1430953.104	1301586.916	977282.4121
22135437.78	6101611.722	5804027.716	2961683.943	8674134.658
8416729.071	6628277.783	7577771.485	7499143.558	7372962.333
517913.2238	794267.3953	707789.9887	1245856.58	2142819.682
6646598.305	1483343.451	2367638.735	5122914.789	561575.5301
378940.5126	549507.1758	306458.0441	398300.093	413908.4957
1655074.818	886003.0554	1622149.543	1050876.073	767367.9429
2888467.302	1045273.843	2184512.155	3122202.845	2640571.614
34692647.68	20471786.51	25486881.91	21629477.31	23749761.83
238265.6267	279243.1983	1063338.054	1038848.698	477820.3647
93289849.72	75167768.74	58946506.25	94640804.66	129338820.7
258718.0478	480069.7782	411629.5496	727626.0313	793139.4642
202274.7414	364568.9301	116156.4168	160816.9002	908880.4731
		18072.35887	38043.9029	20177.85033
2666865.436	4650627.456	1484124.738	1775511.985	1064725.25
254696.6316	307908.5947	351299.1366	488637.6333	569212.282
9898521.746	28118106.36	23551561.94	74280633.4	14603401.72
2860205.179	2768929.596	4118276.829	4430475.404	3217905.555
4089927.377	358383.2025	3609150.301	2954098.968	2373980.881
1766214.496	3132406.331	1399335.037	2102790.263	1482853.339
	671319.9901	646818.5095	713869.9302	707558.3391
	270786.8086	197685.801	397490.0336	375792.6198
1513868.376	1517800.658	3075305.555	2731148.45	1582991.912
		405607.7734	383399.3003	
	253324.6674		376222.6067	495848.0462
18088175.35	22345314.19	21490166.09	59323984.7	25628146.77
18622918.95	20614959.51	20584772.81	43138432.54	25332821.12
11048001785	10307138594	10573831545	9625002968	9480253902
2311615.038	1940581.47	2288972.867	1039651.981	2368402.836
13106220.62	17018716.68	32477455.73	23157044.25	77286143.75
3135149576	3911975970	3115252052	2467087073	2814330488

4146848.823	5404571.915	4950663.543	5574490.005	4405032.69
907948.1355	992197.8562	1187351.018	1243075.77	801769.5852
		910548.8982		543442.9167
1871367.1	3891659.899	3894802.934	4877392.591	3528958.273
10578566.9	11561326.79	11891194.42	13684051.05	16790863.24
	597752.4356			
38105500.37	48648419.54	35172209.02	43801015.26	43962661.77
		245633.4476		
5581960.492	7600600.177	9671741.045	10583911.03	5306766.916
91857415.02	68999514.33	71931847.91	64405003.39	88086391.91
5518961.421	4373503.289	8735615.781	7105879.288	5319722.632
20501258.66	18131242.32	21814601.47	26080697.01	35499019.74
10421906.64	9245651.871	10319066.25	9511023.464	11515290.58
392813.3934		193252.9291	473674.8005	309527.4838
1327450.408	3409607.091	3927157.036	3559708.861	3082482.648
3144913659	2148504223	2348222649	2370031213	2943802480
102341153.9	115626965.6	117975612.5	97307828.65	116346015.4
	191730.4764			
2494240.061	3650310.217	2707145.502	2439926.734	2506353.051
490647.2511	599608.9319	645649.7062	798240.0309	1065030.864
1631843.283	392726.5446	2858704.487	498821.0105	957829.9368
955014.1501	302728.7144	897199.2734	7746113.816	1244124.71
14082046.61	14193984.33	21194676.48	28316741.78	27948606.03
1212881.028	1085010.411	1434172.723	1304272.209	1492783.304
913590.3237	1148963.528	1401952.007	1532532.281	1360109.453
1939604.783	1733782.318	2296007.478	2094673.482	2044514.265
58719575.76	57817209.15	62148523.46	46208040.28	60946640.96
1423417.788	1979050.984	1249467.505	842792.4945	2235070.156
23066835.17	10890196.31	24397232.33	19044642.67	16786585.13
358249.1527	502142.8912	593196.3308	443597.536	526049.082
4999075.382	3851643.46	3930786.86	3175128.899	4025043.605
359825.989	334584.2155	164634.1841	262430.8854	415883.4328
470589.8764		463585.9519	395365.8019	219900.9233
3059905.234	3806527.709	4125039.919	4827745.85	2614145.67
4653302.799	11694043.08	7238603.744	9937718.814	9464014.074
562367.7685			545011.4839	683507.9401
	482751.3419	128748.7531	121058.9127	79125.5644
21723708.89	12143357.03	19853396.11	18898611.44	21443233.08
123148.3933	223067.0937	351596.5213	289567.9603	210029.2258
96715430.3	79271510.54	108888632.4	71335605.94	57743352.17
254121.2897	282304.0471	296487.9845	670607.9128	441483.7459
225236.0954	363796.6109	175144.0228	244746.5466	146615.8379
20351113.69	28468078.3	7495955.272	26846989.16	6640538.176
897873.3813	2708679.907	308556.25	523393.5382	226148.0479
664047.3185	1002228.769			88521.86731

1476049.448	1818466.23	1944049.103	2308080.712	2265383.66
10405893.77	6428541.719	8217109.962	10456085.99	8648981.528
33282435.12	98803425.99	25901611.28	91331162.97	43705892.3
10548761.59	10286838.68	13262882.61	11836593.88	10935671.52
415788.434	428846.9432		1212273.112	569818.2529
24806925.85	57977936.89	45598015.71	60911776.74	58429130.32
36422127.27	48803473.82	43026604.39	48349703.82	46440399.2
44034.09714	127163.3501	185955.5618	209166.0887	178409.5426
	103126.4228		886922.5927	
	1781828.921	1172250.997	2099844.866	2235172.715
	270130.053			208932.9484
368767.5589	328704.7663			
36874165.02	70190429.58	55182560.25	55453840.59	50954638.73
440436.7399	680829.0258		474823.6332	
827722.6509	1528947.616	645147.0778	147789.6944	157301.1245
799830.3185	827431.8529	719860.6859	633037.6182	734111.3704
223780.4944		428695.5407	357248.3276	312011.9228
27701414.12	24646162.76	31838199.19	30394283.59	39913319.97
345184157.1	301671452	628649899.2	626059730.8	279164324
6567486.383	6089519.522	8483548.693	5768207.425	5112120.277
7943025.327	4936528.505	5474258.394	2600701.528	4170280.071
152320811.8	108689664.9	130828374.5	132359542.3	254930245.8
445226.1591		429918.0301	285112.2575	154326.8611
247566901.8	260005804.1	352258733.8	317906984.7	323695289.6
50921004.46	54524132.97	59158869.09	47403356.97	47178429.28
	608558.4719	947448.2772	1199047.551	1227252.575
1441158.866	1632758.445	1353157.466	609691.925	1883098.611
1499064.322	1887462.324	768975.4652	673623.0856	1467721.002
10063636.89	12112459.09	12886422.89	13858337.72	14755969.58
1538697.053	2720729.161	2847732.29	2808528.598	5051511.387
14715055265	13813499803	13896274599	10833685340	13322952182
11691046.83	4365329.892	8309674.706	8637870.751	5057536.827
5094795.644	3437455.638	1787912.182	2830232.865	4431043.105
7778993.89	7181097.602	9203864.043	9287608.267	9129179.996
337744.7021	14472337.54	595936.3945	13352992.36	1223405.127
3233396.62	2420524.668	2378998.884	3296972.977	2092937.774
11360361.4	6638571.061	9197868.478	12587524.06	7553486.223
85674.92731	104234.8152		128600.3457	109803.555
27102127.57	46684703.58	55391096.48	82442636.32	52750776.04
559544.5689				
7928450.225	1605807.232	2257279.115	1002464.355	2758670.618
854083.7735	402978.1713	846270.183	1020765.718	1146120.368
98213382.39	61074754.87	80009088.95	108816692.3	100260083.1
249280.5341		380744.7083	301676.5178	
3912009.84	5350673.375	5324448.175	5301458.418	5026535.067
82442097.54	164975028.4	112467459.3	103148653.2	104519513.5

7684550.246	10693067.2	9560389.627	7846621.463	5379764.85
755886.327	598739.9126	742277.4149	519853.5285	376323.0949
1001202017	1550134530	1736602146	1649401104	1277810078
6557649.458	10291848.63	6131525.331	5170105.695	6943799.983
	314171.485	262943.7193	427992.6753	126389.791
324582985.5	335237322.6	316149051.3	313380652.6	153810573.2
1180999.953	374057.8497	1030129.179		337226.7952
				45628.7037
		791510.8696	536816.1364	448922.0472
2144.571678	5016.595832		5567.792117	
1339985.747	2068400.636	1853419.664	1261499.482	1329176.192
3179782.172	6186404.622	7530606.377	7160307.441	9377417.784
29096433.93	27260401.65	27727110.73	26258965.34	25181161.98
949820388.5	1400148285	1127347997	1077700247	1537018979
455415.2632		666283.3335	697740.4208	717138.0578
4094295.576	2457625.332	3990805.524	1141573.769	5734231.084
19301702.64	17115536.65	25048832.68	19207050.43	19244707.46
119458.2746	105856.2274	49321.3355		85745.57236
983041.6619	1061531.746	1817572.611	2028686.887	1251128.494
31709983.46	39053902.35	39807233.83	47058050.35	54231386.77
128776688.4	113380857.4	151513383.6	119270888.1	112863882.7
3059077.913	9049973.1	5584172.504	4045694.621	12553108.96
3874822.519	1874932.387	1452668.728	2450241.871	2283386.813
750887.2531	351238.4641	203597.3398	390927.8678	498637.9103
1558457.182	1386787.117	2255567.72	1859530.117	2033315.472
195739271.8	157406401.3	172905773.3	239929130.9	200945229.6
1131905.239	646377.9672	1451616.609	570772.5981	1597437.402
4667549.423	4701227.714	3533272.376	5590351.101	3554959.762
7429403.559	6234827.739	6806216.117	9081287.456	7407207.84
			594469.563	
604076.8301	1029234.106	962343.6767	1279858.367	777723.1198
12778180.09	9702795.751	8713219.09	10493960.62	8103659.276
1060287.413	2274758.219	816362.0495	1395759.57	764384.7626
4236331.68	4771949.966	3851774.897	3301739.106	5790163.879
11286786.45	13297232.62	11220167.7	18520482.83	19311905.42
2162403.273	1951880.808	2573733.986	2443185.343	3196679.591
4519310.576	4260455.781	4416568.161	5082744.911	2415354.082
1312396.252	905892.9927	461277.9687	646927.6767	817791.5696
38664808.6	14884580.12	23609476.71	27167787.75	113956598.6
7878277.629	1972883.818	7585082.666	4436737.891	3943036.252
10416586.07	6122434.083	3065393.932	5394178.845	10995799.77
2769966.591	2994248.469	2727424.703	2587245.538	3751523.354
6334646.373	5392325.17	4475584.023	7028303.507	3409750.496
2856010.587	2418238.71	2004675.091		2796782.749
533079.7806	773902.678	908145.692	1051033.206	1006123.427
1909371.663	2240851.692	1245514.469	957487.6529	2422813.186

	473605.3085		498617.1969	1390403.535
78528200.58	112252503.4	71977314.22	96137563.72	110857784.4
473962.2157	321096.938	342142.9452	439940.3729	228092.4047
				1718157.804
553962.434	1417898.129	380436.0888	1794445.418	429273.8162
1145130883	1186062054	1355139499	915097576.3	848135256.8
5143183.977	6403077.14	6101324.875	4501453.512	5824351.715
1885094.946	1511728.758		1093986.604	1425814.688
3806189.959	5618533.303	6704707.385	7894576.332	6806770.828
487014.1363	688051.2685	1117814.988	1491652.33	
77158678.66	87194929.05	69830246.77	84674286.68	102549725.8
3996085.426	2729335.626	5939154.377	7841223.96	3174424.827
267680.8501	417315.2817	147086.7725	282720.4404	91324.13757
4951495.105	5732962.66	5098930.363	7021793.751	5967526.741
123313.9156	124585.9636	167706.6484	183246.9569	1140221.277
81926901.01	57348561.69	65053020.69	68344946.74	82798653.72
		317657.5677	421103.5381	449042.326
			496398.2863	347992.1305
7087002.236	10318895.53	6190235.963	7152232.513	6224895.868
107335.2234	1014287.987	1165607.326	1591744.567	113204.0897
	758375.1827			664784.1542
158764.1486	315777097.2	299340026	911347.5118	846099.5657
2321418.108	1977757.361	3148335.818	1601090.601	2974775.25
107593.7881	103655.8106	80790.08836	97792.31295	68479.04228
177097076.4	145045582.7	141686166.5	167090663	172423528.1
435001565.2	312644720.7	157907605.2	139340731.3	226612668.7
16023296.44	17246904.54	11289398.64	14894330.88	25105818.24
15310371.05	17461006.93	16471143.12	24940510.99	20834275.26
71092591.74	60105405.1	112189487.9	92542504.89	74098205.06
5448749.48	4873810.483	2854849.24	6227676.861	3370773.922
1004460.012	1420166.4	472704.744	1840660.662	821725.0567
16202625.44	18583069.42	25525190.26	25965231.76	31558792.65
4352225.236	4457512.151	8092474.749	6261270.494	6307119.285
12570194.35	11760432.95	9933523.468	7589645.987	10935926.69
5867439.748	7025995.951	3763729.779	5304040.356	5465494.659
2340889.124	2539239.172	2511462.127	2542098.565	3009360.714
		679874.6588	410049.3791	427381.8828
158145.3582	220857.767	191822.4816	275059.3601	248551.8749
418728.8828	1224883.766			
9418857.963	11110485	20209223.09	9236456.039	14267741.65
424230591.3	467771278.9	405990494	401208044.8	465014000.4
77008.22239	123448.6019	79353.78774	57654.46661	532887.8329
			137081.0285	
55222463.44	59192907.5	81534406.11	69244913.87	72747454.87
76923908.52	36167746.71	62423074.85	65597811.78	44049393.45
340436.0759	1192917.009	292641.4182	664054.9326	504947.7842
4917234.634	4704113.02	5568720.149	5391585.887	3781104.16

278312.8707	261740.7299	247377.5855	242671.3484	145904.1739
6439020.678	7858398.21	6464191.741	7258694.848	6779783.79
77850393.49	66013930.61	114126583.7	92346897.38	78230221.51
75788571.38	64003904.42	127736944.6	116207930.4	67312909.12
12568323.77	13773100.85	16913703.19	26941557	26240323.19
5300114.781	10593439.39	10102143.49	8574773.912	8107982.792
13389175.09	20523546.82	21596480.49	16640480.76	17747705.62
		2914270.043	2124674.401	2340039.816
	1270038.989			
8359760.41	7019901.392	5265297.461	8557489.585	6741282.776
39305966.17	42305002.76	33829484.14	40132435.9	43187485.92
325081.036	814986.8976		1889620.84	434197.0035
2947879.573	360011.3232	410955.2313		
134700.8523	92838.09356	121402.4594	70349.70824	39031.21729
63112075.2	76213282.41	69183031.27	95234186.86	92144975.84
365158.8008	349840.6168	659734.1593	471694.7517	353055.3027
		1319388.694		906270.649
	418217.5687	220962.8268		303484.4361
3374935.756	5524460.825	5850658.922	6419705.233	4549351.235
20582512.47	4596095.109	18456250.29	14253089.03	14902251.69
27793764.58	29648591.45	30191593.67	34569311.61	34783497.97
			8200.083216	5509.540803
1596399.992	1422331.206	1338009.018	1615958.981	1702043.01
140826.8153	3623735.576	2683621.855	4322743.836	5507335.511
694204.4645	1710412.774	1233761.58	2199807.122	
17233467.69	18581747.18	14755045.33	11975828.11	10910889.97
3224415.644	2732195.692	2130251.738	3202668.714	2250380.519
51159549.21	42835871.8	52290296.47	32963455.6	74023460.1
2954232.99	1291177.307	2509023.068	4317061.676	1233753.439
558415627.5	599841253.7	447387466.8	681819793.7	445933575.8
60827894.2	43550850.59	64256792.91	52846638.26	37227041.16
331605.1743	541931.1539	172079.6135	320732.4503	300940.7464
272188.4065			360532.4974	142339.2393
333327.7524	1020640.167	927514.7557	1076360.849	809605.7144
	1407920.88			276065.3782
		340202.5552		
	397594.0927		381896.5608	242416.6891
4869486.806	6381894.947	7730156.827	7602309.799	6324692.1
1819263.008	1142049.025	1269008.077	1462600.345	1232057.64
60777183.37	44701424.21	43646625.53	32818489.05	34769765.57
73322846.93	95596171.69	89252699.55	109688686.5	127312946
192530.2776	5028279.575	2700842.119	2199641.33	229222.9692
206032.4124	522208.8641	165117.2563		164013.3557
3469677.235	29779048.57	42495407.17	1986018.993	39659242.32
268848.2274	169972.5802	262962.6475	190957.8236	166101.8769
7720811.471	6399787.878	10031868.34	8175085.862	7335890.122
374590.9372	846101.345	347007.7562	426363.8417	236616.563

839041.6231	420020.6812	1536728.691	963683.2252	1149429.171
414509.1096		285167.6483	354191.7634	
9210930.303	2486412.052	9384013.581	14173485.74	8770389.397
		11822526.95	11231300.53	12655378.1
3677105.172	3730345.202	2766080.058	3033714.459	3633342.046
902186.6746	1394857.504	1396548.092	1248151.365	1497010.406
279175.9397	195326.8522	159785.4819		207175.1562
3270570.134	3107246.826	4849270.139	3793702.056	3549598.313
59104946.75	84618748.69	55844310.04	56434676.64	53845614.33
				128513.3178
		914738.5238	1054862.372	
693678.2107	653560.0534	1626375.437	1174234.806	1619466.284
		291365.5066		385633.8883
759531.3578	849891.0829	571246.5274	364489.6211	169654.1879
8563524.618	5226348.396	9433654.526	7546596.366	10209441
	881145.2808	477192.5083	479398.7104	85496.16395
	475751.6298	757805.5863	735684.838	
20961771.62	18337774.42	15330326.02	20150041.27	18851985.32
6944805.567	6178045.853	9193458.604	5514765.992	5900227.774
134222.4687	240198.1149	76182.33618	87527.58345	113515.2072
	278173.7004	401731.7815	947716.0439	840681.1926
304182.3166	162352.8151	204215.3283	246641.9398	206138.309
26510292.14	18097064.99	17564632.07	30819128.05	24600058.12
2778573974	2214118024	2835793163	2969641491	3419206110
13487420.2	7103751.962	6955169.187	15623447.47	7023783.886
1064222.869	2263992.491	1727902.717		1648645.101
60206.00328	107902.9451	140338.4151	137584.9439	99231.98971
4738374.833	6078680.806	8202888.734	10474549.54	11535366.33
	326450.6146	104972.9349		
134732.2068	141279.7708	265822.6849	846837.2479	466876.2848
6388193.043	4254009.795	8239904.666	4817341.343	5084476.264
2610618.126	2845180.574	4557057.681	5565553.941	2412527.756
	925496.2933		537654.8817	474476.9153
3816036.664	15810720.95	9562779.411	10582947.68	12479576.68
201368.5424	180933.701			
100193.8123	149281.4778	158386.7974	387605.5629	241739.0047
1711743.33	1312485.056	511361.1357	422479.9388	2788132.128
135237.0267	121645.3565		218389.4828	244967.3761
777116.0441	1005736.197	419616.5984	578932.7469	457151.3899
1684528.394	3139743.046	1084291.555	3410198.139	1647944.741
875417.887	733154.1141			

3936949.144	2686561.06	2498111.521	7453360.849	4871506.296
47951883.57	48749149.41	67001867.57	67940034.84	68247660.44
3660720.651	5558442.835	7710677.842	3044849.829	5106590.812
246209806.2	227038658.3	261115468.4	56946326.28	347591727.5
4508565.203	5537636.32	3069947.202	4435008.243	3318310.708
32781569.65	47338228.11	34101880.91	38960986.05	38225790.53
	8964547.432	7932035.345	3824873.266	7574101.704
37618749.14	25624783.97	28578279.66	25699709.46	17072748.48
3822717.247	2701169.471	2038170.984	1454536.193	2660824.152
25673278.69	28106030.49	28585386.22	27176925.58	21550356.04
524088.837	1195162.099	2561118.511	2209264.766	1541931.965
6826114.124	16768515.03	5942961.791	5449090.199	7303860.696
263523.0365		280201.9119	319239.3481	
71383535.67	74531888.12	102101183.3	78769571.99	80609856.02
2091391.835	1381880.058	2626118.331	2823849.535	4143387.914
	919067.5394		517888.5003	448197.1591
		193281.5441		
35536301.21	21292833.87	30255881.13	19587169.91	49301265.52
387323.1018	449082.0059	597205.3314	612831.3472	682321.6426
5120997.83	5594407.997	3867008.172	4740678.062	6456938.469
4000409.38	4228393.643	2544658.114	3668441.467	4282280.044
3545302.425	4360160.374	7095795.524	7101780.73	2829523.333
55385698.91	60327887.53	75181125.72	69370208.49	65945409.93
3728771.294	5817742.237	3842388.202	6128098.892	5196994.859
78870595.23	58253495.72	59867825.5	54180746.77	32392696.49
15557363.29	8601303.608	11696010.5	10813884.76	10805548.63
634425.2134	395620.4859	1020089.316	516079.0992	648037.5937
				458854.8864
3281503.447	5671278.022	2770685.147	5496919.991	1730207.021
1642711.226			1340938.754	
7303543.97	4665802.937	5132392.853	8287710.988	8514807.535
7230.125512	4896.860927		23025.33812	10640.09212
4760828.81	6863182.229	7445092.215	8633149.082	7018386.865
4553595.352	8508017.768	6100772.632	4881060.201	8087275.578
26053672.34	29522925.22	70963871.57	42536870.42	41713707.88
638252.1135	657225.5264		1022879.672	967052.9985
287719.976	204696.2512	269916.5032	280837.8561	235185.0367
2336543338	796288533.9	1081432983	887671254.3	1308089033
18129988.04	10660067.05	23175401.88	17399262.71	15994769.15
1747078.553	1388199.489	1490725.22	1277249.24	3385675.866
733846.9195				680981.1765
				1232197.435
147009.267	472695.943	114382.9193	187778.8169	138276.424

	267861.004			385255.3503
		84615.77777	158120.8309	181314.2011
449945.656	2095558.78	2144046.158	2431986.521	2569138.317
22253023.09	23008003.92	23093744.45	24198255.32	27361939.66
5680159.512	4192229.719	3477843.797	2866533.391	4609758.337
365756.7943	665962.4136	834822.2669	907065.0166	750408.5399
1727477.135	3702049.052	1953953.921	2003524.376	2246456.1
			321228.4696	378988.7844
		100671.1774		
992151.8993		1041898.608	1066829.024	765752.7265
3271233.834	1651296.688	5039475.478	13352491.99	1495783.089
			493396.2707	321166.8832
620653.0006	339666.3805	4184627.171	1932204.972	352562.2351
			1240139.409	619976.1598
2375784.728	2181109.547	2514051.501	2862725.156	2716430.804
13153073.91	12778114.93	14849120.46	15270139.19	14017470.77
7148492.395	8434069.771	6065739.183	6950989.49	7320187.633
		394991.5015		260627.3041
72282450.76	110677238.9	97717625.75	71339072.97	121916598.2
847518.8332	704086.93	1463771.186	3010759.377	1225947.663
	1644957.774	707678.7711	1056653.025	5789722.318
3372471.742	10562857.14	6089917.713	7515251.986	7178517.275
66179720.91	94525952.58	107222163.4	97508655.69	98334656.12
		1475659.076	981645.3255	
247689.1634	708082.7782	575776.7995	906492.0873	713044.9648
817972.9598		849635.1768	1011425.001	485266.5333
237300.5033	324476.7232	280569.6654	305769.1282	147959.0511
1418758.568	1994571.799	1319940.786	357507.2921	551604.6688
4843279.674	6078297.536	4440203.487	6771176.137	8984160.354
301807.0642	199992.2705	4435163.15	693838.1469	616543.0686
2345957.708	2031262.624	4345214.005	1663499.804	3741679.93
1473069.992	1267393.825	3665455.54	2030856.03	1748347.492
833291.6614				
2080870.618	4931275.561	4163426.108	3770887.242	4460528.935
	146043.2081			
1021740.877	1881893.062	1141044.247		965576.8593
8292920.256	19930831.18	11579093	8055754.085	7254670.505
29863.95505	19279.9851		32853.08149	27690.69944
609189.1437	890835.6468	899737.2603	1056593.395	1051390.486
177737.3895		152836.6217	182363.0179	228071.1668
1748701.831	2289625.675	2311858.105	3086367.789	3149726.359
383574.9861	989899.7478	194463.5804	405443.3948	227200.4689
	161480.247		74586.01214	
5072252.578	3433303.657	4741748.604	4841106.136	3272470.306
1783463.285	866038.8779	1090461.741	936606.9599	935632.5696

1065897.216	1315688.198	1442224.078	1373317.163	1784696.21
468208.7691	1065679.779	258800.5813	889280.5098	536307.4874
			442672.3939	
20248438.33	29154352.89	24633781.81	23167305.44	21892429.23
499651.6097				396187.7522
277266280.7	331932489.4	438722247.7	322693355.4	325374386.3
				428059.7941
102666.5007	252294.7945	29308.23915	86117.51815	62508.71977
1436713.925	1848834.613	1222961.102	1442192.369	1430322.497
223555.0454		138947.9776	1843023.135	2330313.304
381181.5879	821664.1245	289048.7486	366786.7594	417110.2203
16942.36946	18885.60582	7915.205611	12505.94277	11760.69835
1318333.386	1784442.298	1652048.694	3531820.767	2779154.333
18385816.19	8724042.242	13643884.89	8622523.217	6209281.404
1922685.924	1681219.555	1451135.187	1693438.232	1331798.195
194833.987		101465.8464	233226.9367	482960.6969
	1516446.004	1133062.005	836288.7266	846445.4619
1723958.609	398342.8323	751535.3988	720253.4297	1669790.236
13937876.6	19329066.74	23316032.42	5942492.907	15499538.84
330329.4098	394009.2362		290911.6092	
1126537.171	1599012.686		28945.59127	1141115.319
1594281.599	2801690.667	258285.2288	3796494.611	2261241.661
13948210.05	16043153.79	16541793.39	17662817.46	15112386.22
14468729.06	14402405.65	15470335.4	16156308.61	22763501.34
1485531.46	1801154.846	1947011.414	1903306.355	2110462.781
21446119.16	34270091.13	56081023.02	33353269.49	30287448.54
195469962.1	235992377	185778533.6	228793851.2	308651246.8
168205.0534	573785.0229	108018.7906	146406.1787	113235.8992
728422.878	927929.4411	2142330.979	3440752.261	1167390.737
1117347.131	249039.445	1137844.609	728867.1335	785813.688
	238162.4896	256744.1291		
173375.7856	256693.8061	212005.3569	438684.0996	342571.5636
753117.6114	818337.2866	1161893.41	1672921.045	1330879.575
1885445.083	552100.1524	560335.5099	752023.7326	
2646828.484	5137711.862	1169121.427	2359687.191	3121003.505
2588434170	2375274294	2507131990	1362212904	1927509650
7033624.808	5721090.594		208583.2398	9277163.358
15299891.81	11125723.68	19866930.08	20182463.89	22210460.13
1408982.89				1031026.066
686956.1465				
835280.5047	1532834.965	1283830.412	1915637.719	1408922.847
1175806.876	1530145.673	1355374.185	2056625.694	1431353.113
8923133.247	5447579.508	11152170.53	7157805.228	4149052.783
1394761.484	2917861.574	2743523.977	1951512.472	2033707.362

3613067.008	3468934.549	3017655.827	4256571.272	2510665.161
2853815.19	3459411.887	2967758.761	3756092.234	4683459.921
4110978.072	4901511.043	3408420.605	3677448.377	3221985.58
196854.9904	285544.4422	129168.9391	164997.2438	129032.0677
	826954.1504	718828.1337	1334011.801	887581.9634
		194956.2181	207574.5243	
383392.1612			211384.8676	534245.6696
41540.08258	38671.92105	42363.95955	74764.66904	58678.52868
29394535.77	35502754.23	35410338.04	40027972.94	55290987.43
630943.7657	728899.5024	490860.8943	1325602.363	521187.8497
33681031.03	31017050.38	20713769.1	22285235.2	23392576.45
23872.23469	22821.39009	8442.518676	19470.03481	14384.27249
2054586.594	1468911.946	3017508.266	2794422.374	1565705.647
291064.9421	559121.8405	144655.6865	663516.5243	386891.0481
1373324.96	1750053.873	1914033.569	3008560.57	1223415.945
1271027.622	1593639.586	1223703.414	3214117.823	
29190.60105	56446.52028			
7365958.499	7931661.935	10697236.75	8670122.165	9997333.067
318943.157	425049.7281	369205.7069	399587.3863	380398.2785
5307913.353	5980947.637	8164102.783	5137691.991	6401690.573
561513.7114				
205726.6988	300300.33	314149.2539	445430.5409	422015.0632
35164871.14	55943465.4	26101374.32	74982879.78	42148934.03
4122195.384		5382868.17	3348157.524	
		319718.035	606783.1576	
4548928.54	6294946.332	3510851.84	5872062.393	11552125.41
500817.7692	456776.5236	429370.8392	679887.2672	502111.6997
450691.3388	602063.6661	652541.3112	618059.8284	613033.5262
78036926.99	72199546.29	253231716.7	333027985.4	25203541.71
1859142.135	3149492.034	2660202.622	7170484.137	2697557.161
790800.5541	875528.5755	1961109.034	749844.2088	59700.27954
2677468.866	2370535.259	2302354.16	3826756.453	2148900.081
565383.0859	887115.3667	439200.6326	992106.0759	427284.3359
17436670.93	14778450.88	26516922.72	23799983.35	24557257.21
				557238.2956
7593.347176	15644.08859	7930.692434	5826.621332	5122.458854
78470.6667	1058969.285	1803805.129	1183402.033	2596849.042
172232202.3	180508635.9	230260551.2	338499540.2	233126523.3
820860.0862	1062825.103	1100632.916	1572135.817	906152.6396
702290.5768	475080.8119	481486.6786	669068.7323	375968.9532
1856661.034	2573081.26	3858844.306	4328353.341	2295753.947
49667.80265	68520.90599	27113.4082	28614.06475	30422.77159
	696503.4073	769649.1921		153792.8211
12753226.69	22713990.63	29043112.51	28170668.63	20859965.53
48664834.33	88098992.84	31168853.05	37834499.08	41575632.68

7415412.362	9060040.303	7157759.14	7505324.063	12928113.11
63986219.67	124025940.7	67769917.86	61309216.47	128134299.2
2245831.327	3574907.991	2735886.613	5471022.976	2579434.726
357202.3783	356703.9942	191489.8654	488507.3793	289949.8693
673788.26		716110.502		
				30696.07029
2091776.52	2201958.04	1974897.94	962974.8845	1810520.651
7638825.67	8149044.782	2890592.559	3551057.097	3720833.336
1075020.926	1935103.932	1760052.765	2697514.517	1456248.776
734037.8008			1919742.978	2727390.325
7753241.6	6695647.417	12060727.79	12094380.22	11850956.79
41557778.82	41764905.52	52023992.17	48867959.89	36660386.53
22459332.37	18744346.68	14740683.26	15357516.13	16121425
473891.3659	466367.9218	653301.1039	692309.3566	487700.9696
62975.15445	56520.95966	88925.65043	128480.3125	111315.0025
586367441	624917249.3	856200022.5	1128792550	1061661235
1822483466	1650643842	1844866763	1533142249	858182987.5
209888.6904	187859.5146	271747.1458	239752.0779	212666.4342
86897611.84	72289283.8	50462531.97	96950283.71	87120363.87
	1858853.291	892211.4066	1544731.432	924482.8337
1953087.527	2421037.669	1875235.083	2016453.703	2257366.073
98029755.52	54508670.72	82940627.68	81379875.98	29916855.18
4166312.513	4113144.162	3844495.164	3785782.774	4268988.072
		338254.889	391192.3286	472311.2151
2876874.955	3136949.345	5619015.412	2799868.157	3803080.467
7680.977297	24564.30355	3630.850871	5261.01233	7139.637174
8934046.381	9651342.767	13661710.48	12619461.98	11118668.07
708851614.8	732954442.7	684141636.8	659420837.1	1019762541
4361250.41	3927941.426	2357249.687	3619190.022	2360486.048
18147764.83	18694830.5	13851889.74	14109641.73	12607988.69
1471617.697	3847062.358	2716718.91	4492147.166	3803225.974
215751281.2	232418403.4	214058493.1	215595398	180785585.2
1007349.012		745044.7364		620396.7197
24884331.12	27937432.97	40164141.21	36939394.5	36825274.93
654286.9854	959412.6219	846973.6818	1165426.719	659681.8346
7421801.882	6198442.832	15233972.76	6838457.461	6694125.449
781253800.2	979591532.6	474135708.5	781829255.4	365754331.3
7814760.067	10103545.59	9629479.561	9714984.269	9453608.743
		343259.872		
421722.5415	344020.8139	692047.2238	349214.7059	
8263373.732	5602871.257	8212552.234	11051020.84	9292139.077
11876054.76	16810716.84	14001954.01	16078773.46	16841320.4
			594951.0887	
677717.3878	271484.8023		1098295.075	
3518896.88	16662728.26	9180907.522	4240509.525	5434344.291
2806601.785	3089134.666	3175572.72	3977538.281	3509162.095
112081213.2	72190476.94	89713096.73	93486518.91	77497840.55

318649297.9	239387686.9	359062146.2	287800565.8	406040218.1
6574370.401	9387442.754	8276285.337	10407222.21	12582518.16
4311234769	4013883096	3977730565	4085148368	3930656689
4550038.455	5182111.723	2604887.371	4473776.422	3204950.956
31088604.78	30546832.28	55615492.91	48694149.63	30082122.84
502868.188	349080.7942			989371.1552
18838138.46	17867043.46	20652649.77	20585220.04	19922100.11
413391.4011	455013.8901	409481.5785	310526.034	143276.7997
470009.3635	230210.0388	518526.9647	518685.2518	597369.7499
2633916.417	1463861.872	2667340.722	4955706.69	1480436.339
3021433.473	4315989.059	2734419.068	2040532.32	3583158.684
50537042.67	74323662.7	31726854.05	39836563.86	300533621.1
734427.0577	604659.803	744425.763	965566.9388	591158.1416
3056522.618	5666564.496	10131439.34	5508558.034	5302230.21
717112.4119	802094.1104	624706.8562	1835444.348	1007387.754
20032297.98	14957570.06	23848848.01	19432152.29	17183719.45
15660881.91	15826823.69	9302509.857	15373192.13	11010518.75
1407999.705	342397.5135	1063649.313	1436628.227	925105.4091
3610405.987	3023355.188	6349750.281	4837300.319	2687917.908
	186496.1305			
2553994.15	5105671.71	2004023.733	2858929.268	821571.7579
8941693.2	7881819.744	12064639.84	9175034.051	9208259.274
12908681.97	16934625.34	18954844.95	21863958.61	21321316.42
57383.89511	109165.4161	86234.23029	42257.28765	103220.9735
35976932.29	40930061.16	49202386.47	33718445.13	43366877.78
	411870.7787	339141.1241	232587.1863	145081.8051
1057727.397	1438205.781	131776.5124	145500.0006	1206428.391
1677368.284	204309.4321	547741.4111		
4713223	3770642.347	4920208.19	3609706.628	4222311.226
273580.09				
20927187.29	17901170.86	22113433.09	28785716.79	22919006.99
714375.36	61285.84399	44642.62081	59623.59351	43483.50393
57618229.85	74873980.5	46883487.78	46989824.8	48364498.54
5241930.91	4216204.875	3548050.13	15363843.54	12509337.28
18548260.6	10145028.68	13932783.56	12446999.49	16990682.39
376734.1362	568634.0644	615072.0896	639125.0722	596595.6156
104378991.5	130729320.2	127575708.7	120423376.7	134220153.6
1077513.253	2000852.566	1079153.566	756434.9571	1625037.124
195064839.3	612058432.3	228908833.6	178453011.8	178893250
22365861.74	9137367.497	11060270.25	20986192.44	7430049.701
34831002.9	39214563.16	38526093.38	82898239.42	69761350.08
27964898.05	25214286.18	28575785.67	24133303.8	27126663.82
1321579.23	567505.5842	138697.7567	1048055.999	
586653.7315	455166.3176	726785.1348	1315437.658	740802.2975
10120749.69	6231735.767	10435730.07	8633844.689	8660681.45
	341653.0248			
95056.87968	123142.0009	80587.92671	120118.7782	65813.39536
3394178.325	6829374.603	4923378.063	4616186.985	6053038.043

42652569.88	43111849.57	44658691.15	59967523.57	37812784.91
18458780.54	10782356.66	14682594.47	10361893.62	6573568.709
21389628.93	22000955.96	22300494.74	20691708.8	22215209.63
5537078.178	1096390.024	1791538.029	1997814.019	1135380.866
39250069.28	50151940.14	49461439	49774709.42	55715085.59
65189539.75	88777514.37	49197314.66	92185944.77	93523173.81
1631621.736	12268471.96	971461.2624	7253677.133	9234046.359
4286999.694	3758064.549	4761465.421	4568291.596	4201648.379
1918623.804	2762727.282	2590549.266	2539353.387	2667220.188
270310.6764	308982.3247		455407.5446	
57944739.87	58973719.08	81914919.11	43405802.72	71637312.37
2492847.647	2120000.631	1863924.134	4247838.759	4570454.575
262251.4501	1153551.537	5244063.107	644202.0414	3861739.63
3278586.376	2188294.399	1233529.871	619434.1703	4124655.811
68763.24841	1094901.758	1493042.297	82819.96528	968682.5878
14593907.35		5415378.486	3189932.208	6998103.992
3058791.816	3144661.758	6626999.911	4486364.341	4998813.554
			132097.8629	
98139288.82	126652643.9	238395506.4	89724990.58	96087558.87
1235017067	1510205762	823469699	1112687213	1199726110
8485184.11	9829687.496	12011353.2	16344353.51	9790276.601
104029.3591	99390.79931	114028.5874	154017.5479	110211.2077
15264694.94	10292935.03	10488977.93	11199800.04	10513234.91
14875140404	13795138118	14984250223	15102607689	15747061790
1599612.692	450401.2198	13033150.59	1382653.638	1766078.94
1533109.128	2462217.668	2639564.131	3499568.298	3446815.875
2066671.695	1840860.556	2552231.202	2932961.72	2577730.076
225886974.4	218142018.9	219648993.9	225094015.8	237980094.5
11272145.77	13441515.26	12864723.55	17546041.5	13722969.66
				817145.6854
562208113.2	548459727.8	764373181.4	519790312.3	389842928.4
428990017.2	506504935.5	442861191.6	390252811.8	471280709.5
750113.1234	263463.1689	370441.4293	316449.654	1629600.269
120707618.5	129443914	143014870.6	185640575.1	134887343.7
886340.1641	983854.7955	727199.6185	1227176.784	1334561.725
		257724.1075	346538.5511	361790.8379
	93954.02958			
517841.4279	744874.4122	584074.4095	2276286.767	
9281054.896	13445529.76	22463245.75	29046192.46	21260562.05
63540591.27	53742301.48	52924097.44	28401064.99	31152348.22
2803488.966	3190709.087	4186173.241	5326763.343	5030600.266
4757153.09	4236278.401	248734.9876	3523074.983	4325212.619
145669487.7	103674670.1	94984931.67	91566611.11	159915938.1
2731365.477	3223254.07	1784354.367		2172147.032
461128383.3	515019133.9	548350836.8	556693528.4	546222969.7
30871408.81	38135288.57	42474169.73	36583640.27	49724779.19
15239341.83	14525203.58	20435321.46	20590123.94	27731120.3

2323865.99	2986318.896	2564429.555	1522346.494	6212294.579
3295411.851	1166096.276	3074758.798	3052569.552	5151979.818
3759475.434	6456317.743	6057090.927	6935225.558	8347232.138
49496239.41	43325794.03	51422432.16	39891199.72	47431157.12
30667945.03	28324547.1	24607995.73	35465242.64	26353426.92
34324470.1	70933897.57	56635784.35	76196123.23	81417977.15
7636109.642	7528768.185	7649566.051	7583914.569	5873135.757
2268863.889	1996738.915	4086753.712	1869577.314	1776850.894
12612573.08	16644325.51	20208084.68	18329923.62	16943274.77
286520.6633	140859.9877	294628.2179	177571.0453	
45133938.72	49353630.66	63259812.79	64952515	54847099.77
2411669.512	5318001.599	2475557.675	936095.3553	6321959.841
1038611.688	675549.1718			
869342.08	1113828.178	610223.0375	467354.2187	1188867.169
631261543.4	817872167	1421758548	1516828051	1268260269
		554336.7688	1276133.254	739022.0759
7059972.038	11612875.32	4964955.954	8984663.824	9939007.621
4103020.469	4619601.311	1654217.778	2828960.461	2156172.98
314334.8201		501875.7401	477592.7727	236018.4216
238317.9186	408463.0206	612980.9714	635932.7955	414738.798
1859074.461	1615239.219	831911.8329	1754697.548	
4570645.578	4852228.623	4770223.114	7190106.26	7325841.24
380636.973	340476.076	254613.4062	336259.968	376747.1724
9274264.559	7287511.56	9918640.845	13334065.89	14814201.22
72348755.86	57010972.64	63061180.64	76741970.95	77389033.51
2956305.143	2937005.448	4088547.125	4281976.729	2874161.632
53709697.54	38067858.72	44601855.2	31466028.65	105035658.1
3581536.634	999979.8555		1136401.745	
627549.4961	659092.2327	650591.2386	619340.1315	694020.1603
732697.5848	736042.4949		673547.3614	
1070886.852	1135735.199		1036701.051	
1674412289	1753231610	1448502878	1385944847	2093453509
519364516.4	487235452.7	405998953.8	438229768.1	461864854
843327.7962	1858793.72	1770305.447	2211546.846	2069980.929
7220548.266	8569520.288	4878747.115	7728648.243	6433634.271
27660893.25	29466972.83	26080906.17	28595528.08	31333572.28
2058011647	2343635488	1883220013	2401158793	2835295377
56249958.34	55886793.79	39980373.62	27064839.18	33919356.92
		2106967.134		
25713697743	19957718857	20361685244	22067160140	19448886464
681290.7117	774027.5502	771336.8918	895654.1273	861567.7757
651547.1418	3427096.029	4156418.698	4545930.807	475752.2871
3276654.129	3385096.646	3465507.271	3248929.585	3439858.462
153107.7539	370008.8786	243937.4161	145595.3759	153057.2762
3541874.094	3518768.356	2649983.661	2062785.367	3097755.844
	190540.9834	233768.4088	712326.1946	745407.7293
16951127.69	21478631.71	25041260.71	27460799.24	18354190.43
1624258849	1596719405	1532207461	1891855805	1926018714

	1075942.527			
377962.2741		128850.112	543318.7467	750498.2668
6128818.424	5153901.161	3818854.113	4453002.116	6799189.705
5902671.913	7597769.052	10958028.11	8766585.031	9466449.22
6389345.947		7811631.941		6552362.073
4796460395	4572860797	3847064849	4125711209	4721091386
6485629.859	4314451.575	6864200.478	8136460.359	8284828.369
1093431.754				
809727.7797	1572307.48	1277674.646	509914.961	1778905.658
557707492.9	482036035.5	599745049.7	568976114	254345006.9
2510413.222	1776558.66	1780764.966	2608457.137	2372399.857
58360.89707	49650.15721			
210656.7872	211884.8656	847852.3013	895737.2489	268906.99
300513790.7	104889846.6	178050089.4	183882059.3	787406197.6
59189453	36338065.12	44802234.35	39761255.99	54278539.83
2554226.999	1250832.585	2481966.225	3297403.343	1483279.497
1977048.019	2539526.947	2258394.458	2065680.224	3877326.37
95461526.48	55995002.03	56956680.59	65331662.86	49391624.82
7262623.146	10065775.63	14643945.73	16076015.48	10034794.78
12742358.05	17132595.86	20585128.21	23660055.94	12658693.25
10123091.53	12949158.74	9366959.994	12380386.75	9980113.164
284740.3692	270684.3678	421732.8254	425296.6248	190537.0169
7015578.085	5332137.206	6018665.255	5391435.719	4588294.387
1104115.936		1256834.908	2728661.483	
2021889.722	2620076.185	2883531.246	3758348.526	2174827.284
3267566.967	2480484.495	2560061.149	4360275.855	2754311.134
7377635.308	18569302.63	10953300.13	9268231.537	7328048.092
1606945.453	1750099.81	1975283.319	2292377.426	1722906.927
608661.466	148418.8255	244004.863	325882.0003	291689.4495
			218230.5993	
2293087.318	2465742.062	2208958.87	1883877.298	2146514.739
12474013606	14105415366	13058706446	14612134559	9205795430
943534.63	2369363.852	1071021.941	1627527.097	1816102.706
100625002	82050973.02	92386740.21	97511305.2	78461203.92
355858039.4	328236928.5	325402038.8	391523654.6	407856753.1
1658690.481	7908627.268	220410.0374	6101249.084	9535456.874
89948097770	91409048630	81103461974	1.00156E+11	87847341250
1676763.527	1711948.018	1736248.226	525504.9892	1766099.024
	471335.1398		482980.5445	543098.9494
8824212.932	9814774.645	6834481.669	6046557.035	1635927.144
1186484.736	1799345.452	1653083.169	1434940.12	242987.5219
929673182.9	1133788030	1211789184	859951797	1681119695
31357251.66	87710435.51	22903697.15	28788241.11	39352966.48
20131731.4	24167615.95	12053151.71	8752250.486	41426424.27
1069058.604	884244.9765	824017.4906	1446624.035	2500122.606
191274752.9	170828147.5	222437116.1	190916954.7	196827204.2
		398862.1021	414629.162	
236531.3199	759514.3851	932116.8292	1408080.939	823815.5448
1055320.696		933276.7351	1181805.876	934418.3962

482486.1578		401634.664	478730.3493	313358.8379
	500053.1609			650609.7834
3235890.668	2148436.608	3070402.025	4325924.82	2923049.595
17251927.63	17414181.52	27201846.61	28417749.33	29924800.6
51578290.72	56016743.51	68316698.36	69598092.44	61117151.7
31670156.16	61237375.1	45479467.23	51888434.49	38033658.17
20987747.4	22354690.27	11279631	18149644.72	31516620.28
218086.2626	219067.6351	1091689.64	2607906.531	1550097.273
		1266941.766		
169315770.2	390584316.9	283310932.5	294559058.5	314566642
4256515.86	6047978.608	3267949.539	3619985.098	2893142.826
273158.4762	264838.433	169757.1061	174485.4375	203251.4559
375669046.3	365138318.8	242904398.7	180054969.4	136474991.1
		64096.91678		
1706398.504	1839578.89	1477897.266	1559814.091	1197172.31
7772514.072	14160962.2	12763821.43	10058460.85	15615868.97
3854431.131	4662238.111	1948155.764	3175557.683	2999420.301
1189498.929	954974.5545	1228415.951		
	516153.9676		542522.7292	580901.306
8002016.64	8028674.526	6246349.078	3740119.33	6377923.548
36580.33296	572050.4193		610612.9261	33416.43131
6429171.122	5683065.766	4115300.848	5678734.612	5925746.123
2216487924	2443216692	2318532247	2528196355	2562499205
234496.8266				
93789935.72	106614334.7	88853684.74	94610054.57	110633036.9
905629.3672	363268.134	977888.0594	438095.3214	545004.0454
29532340.33	21951265.24	29357290.81	29934006.02	45090069.78
362974.6826	118442.2712	295420.0688	307641.1152	288150.1309
2503053288	2435200550	2489168647	2139818150	2820101254
88205699.48	160507701.4	80601309.41	73868759.07	55579175.03
8144218.482	8181502.744	10222881.31	11244104.42	10861348.24
1188736.528	1569663.491	1170656.23	1042479.585	7874097.883
4567779986	4173958483	4301817898	4727467845	4623168387
39816485.05	60411335.62	83963105.13	84285601.92	54856274.44
79454595.99	65905183.63	81096913.97	78330973.46	75573258.45
1824952.369	1010136.31	1325139.571		1182524.767
2139798.641	4587309.668	4589387.278	4794753.645	3857656.655
30980140.44	28744007.87	67025705.06	74875531.22	78527454.67
526836.662	707781.6332	1180220.477	2039815.245	1486137.992
511673.7587	678653.1375	457679.7288	402300.8856	86347.75145
4740889.19	5446481.658	5336117.456	9076962.779	5232201.998
292258.2406		527389.7865	1614105.346	352994.8701
1163756.747	864502.0771	1945761.101	1026982.56	1822739.885
235162986.1	141458106.6	164287910.4	177290399.5	170528226.1
				89194.94662
4557927.55	4714461.698	5461398.022	16407149	14652478.24
1566228.307	1658252.942	2261948.899	1471298.3	2846239.975

3745298.282	745209.0241	668657.8116	647657.3108	
34452842.26	34546438.1	37655998.72	38948039.78	37402428.76
8937863.482	5341886.763	6376554.716	4956356.982	6245139.934
11744622.42	11495379.94	13778216.55	14242677.48	7592769.359
43428448.66	16027126.57	63059795.29	27925631.22	34568672.46
3134429.766	7785091.901	1813488.134	2110374.244	842284.2487
2714702.222	10634380.47	3584032.755	1922635.596	1489542.205
	532646.4327	254326.5212	234123.9763	
909789.4852	983363.8087	1305528.486	1880531.333	1207093.121
126836371.3	112882970.4	107016272.9	129142896.6	112908822.8
8094683.943	8015066.798	11478908.52	9496472.27	16451997.87
106160559.7	157484001.9	195039899.2	300968830.3	155208420.7
82641040.72	73720699.22	74518967.53	77301232.26	86367469.08
26583456.26	35078597.74	27565367.31	24745903.1	21555067.94
377602480.4	348224879.9	528483552.7	579211379	546777595
14274876.28	10627412.52	8555989.465	12517731.68	9906245.969
250006.3064	476457.4423	305199.343	293017.564	1038799.257
2983131.534	2062004.258	3327832.367	2550735.835	2880289.632
234102521.5	243133000.9	146708252.2	226122058.1	166249880.3
238552.9417	262886.138	398779.4617	237914.8191	226361.9779
235655.8103	341753.9379	159900.0267	182831.5652	163020.606
6153748.274	5377009.004	8971483.093	7823551.298	29074263.46
1679520.491	12753293.28	14607000.86	12942321.66	15199866.34
77719298.12	125326402.4	84147997.1	64562128.51	106606044.1
5106344.168	2786237.83	9511973.524	16074784.05	5168097.96
6583468.382	5181858.392	7240223.412	8579137.854	5751222.628
111112964.2	191471080.4	101984903.7	84053390.64	125048243.1
2749512.278	1733210.221	1962235.131	1944028.552	2301454.096
4794445.089	6194157.477	8059074.537	12719732.18	6601140.801
27827988.61	14727168.13	15783486.54	15335599.43	15365998.39
48836724.32	59590082.9	57099639.54	49426740.22	47615776.5
916756.6899	870083.4082	806479.4842	715600.6519	1030759.032
90198459.6	101404160.3	110971590	72256540.28	80297702.62
16362232311	17066564031	18335437093	13435447872	13853686022
49686845.62	32337935.94	36252108.4	24695112.55	44615003.49
1077538804	1359262417	1761851348	1248221591	1499135960
1091611.927	1348975.706	2343945.982	2742132.475	1860232.023
606734740.3	763358332.4	499486351.5	788443559.9	736337237.6
4042662.841	4928984.536	6403576.233	2623182.237	5057242.863
5618529.061	776227.9195	2715786.831	63265373.04	3833635.607
2863276.517	876890.3465	1408820.055	1116061.869	
258051212.1	379545637.9	208914686.5	276041169.8	290527867.5
2841219.648	3456056.355	6068086.8	3943744.862	11467675.44
8000983486	7524052745	6744870202	6171865815	7082277159
789519.7309		1792211.065	530338.1839	808646.2826

16907849.6	17145756.08	16928495.62	16280443.98	21151167.85
632508.4377	777044.371	1243591.487	438023.5688	521736.707
11054677135	10007942830	8100549544	8678403327	13661804053
400864.9192	377346.4668	270617.1178		449026.5914
147899110	172220076.4	202987592.2	188634323.6	184412782.4
346199723.2	333419157.8	437697274.2	484799411.6	519891179
224863.7664	1863399.378	318555.6786	1376930.227	1556093.476
39525838.36	40210205.89	87545536.15	55707929.37	58973974.43
2530231906	2310497087	2686790344	1663540331	2602252770
229251.3382		197288.5089	314045.4704	152710.2737
154393102.4	173047736.6	192702548.4	211639953.4	169655668
42958807.42	38136002.61	31360232.02	34405965.04	36014794.24
2020855214	1555324425	1883382601	1843860620	2246145310
1029514322	838308303.4	679346873.1	981148798.1	1191930393
6792129.834	8555197.477	11469791.9	12805033.46	10081109.91
539696.9191	1060080.116	1145996.354	1486896.196	918156.0054
3966026.006	4356398.191	2390490.118	1833000.373	2121778.562
31958891.84	24318161.08	22017992.83	24346725.52	22812363.96
14397121.76	8814684.663	8839666.478	8233763.404	4403751.399
245957.9668	171729.957	542534.6528	271852.138	591914.5178
1146323854	1291457613	1144246394	1323363487	1613983189
37752883.27	33848726.22	38594445.34	39659255.58	43285423.31
3809928.463	2117608.045	2387266.661	773724.8587	780413.1392
138753.8103	661713.1062	1245661.719	1317444.002	1056096.036
1111494.468	756415.7901	1417871.759	1324608.913	1415158.965
558169.4479	1042528.856	881975.2423	2516273.411	882917.9051
6188205.964	7281284.148	5180956.098	9878942.756	6527383.9
		493590.4771	1191079.809	521749.7183
5565399.31	3209355.645	6667820.975	8306657.786	7909515.03
6548619.787	7958450.206	9963364.972	11656391.87	8794660.912
8332119.289	9272023.586	7621494.689	10513538.11	10265578.35
3477631.409	5068523.64	6747573.554	4870106.028	4394879.623
210007610.9	135749633.8	175810676.8	202563899.8	149377916.1
3623185.403	6420376.758	5361109.393	5719323.367	6283158.045
8493564.745	7095550.763	5011874.05	8016958.377	7365612.633
1483224968	1523557065	1577294279	1910038196	1445257005
1425392.573	906924.4739	1697606.387	1077035.666	1405884.104
1061654.328	214385.0041		192559.0441	
113778.5818	170271.0399			70369.42014
763328.8239	1163622.08	1402542.554	2356251.422	3677515.475
57735024.75	19686929.06	38662685.61	16926758.66	62020061.59
159844971.1	224087274.1	203728193.7	240909066.6	217164194.5
216776300.8	131805818.2	212786387.1	200620054.8	199611709.3
13040286.95	16242952.08	14336045.36	13337626.47	15023029.89
35462124.92	34455691.64	27353312.98	21974518.96	30005642.11
44499944.06	43290421.5	105555304.7	58805249.87	65922923.51

165236564.3	189107707.9	283856075	255787520.6	240958286.1
2015994.286	3172250.35	3780195.214	3351186.918	3052893.97
48198463.87	48057317.33	59607529.08	41936295.97	49969936.68
750680.8959	789512.1563	1009115.338	1522937.606	715379.0364
1753111.96	1911919.433	4293016.015	3808043.848	1415246.186
1879833.008	2538421.316	5309025.076	5169161.881	4153336.412
96515940.35	79757585.34	109354217	128172966.6	86592193.6
72425748.63	77264422.24	120238078.4	96546733.88	101286422.6
8151502.324	11950244.81	7929969.59	10120852.92	10127249.68
		160705.9117	208538.1591	
8322040.193	4582097.146	8737247.155	10967646.64	5176596.436
229515.1021	398652.0903	1175699.442	1412618.878	93471.87151
2070326.844	2373426.828	1618855.088	1904701.504	2885895.156
415454153.7	419269715.2	225008154.7	320406512.9	501759948.1
545123.6258	857826.0459	1211747.572	1071224.155	1008689.754
690744465.2	810260084.6	733092201	991360007.9	671143633.2
575866037.2	1031765136	758571239.3	680590325	963889361.1
170406.6522	228796.6963	310165.1359		
738330.5689	830684.0711	720994.8044	657976.363	570457.3194
21013003.16	32417417.88	30697564.54	30184615.02	31510094.66
923616.9501	866214.2683	1484128.206	3024675.585	819578.7223
18854306.45	21095356.54	25468014.87	22660989.82	24105243.49
2429545965	3009529115	3353607668	3454177372	2864676005
2118545990	2495096162	1675837897	1556029437	1832537612
44088960.03	50800559.73	55555978.48	48492421.23	51047484.81
1269265.951	2144440.755	1863048.248	1630576.942	1672712.35
1340370587	1391778801	1192216065	1518536603	1161622828
448957122	501055192.2	415542903.8	391271330.9	383565613.6
11675774.46	7159894.305	10090422.21	4252612.906	9501515.355
265420.6642	368587.651	229877.3555		275486.9239
3717854.881	6042306.526	2727639.579	2436312.638	3857696.341
52940115.71	33211714.8	37913861.76	47244856.14	27085637.55
513283.2845	684996.1889	860441.8382	1063622.743	769671.0938
693777.511	347817.0042	376624.8671	564123.0849	217998.5507
1188970545	1306427694	1250785100	1059697454	1290148153
460035.4482	794561.4712	433392.7877	770515.4931	584515.0708
44230220412	43870146874	46186827257	35963503916	33902268671
32608936.37	45846538.61	44133068.56	49472326.83	58908959.36
			1134390.337	
253237.9297	426617.0068		347837.2888	444039.8606
70400542.48	91546750	92817535.38	106085994	92327658.5
3041969.175	1558894.784	3775899.611	2675615.415	1935882.152

1847542.574	2023152.764	2184108.114	2547434.291	3693034.17
497295.9831	510312.1888	522048.5115		
1207205.362	873350.6187	486177.5	2237972.971	1295992.505
108848020.6	326703206.1	57588933.81	81229983.75	67373283.65
26656161.98	34659362.27	39476380.97	32565071.22	45847660.94
2795061.017	5394106.943	3752957.821	6217645.648	7024637.658
639764976.2	1122032145	813927829	1297640017	1123595433
11226395.26	8845107.455	11807382.11	7154465.531	10732913.47
14693003.56	10410950.81	12218589.22	19337078	13806694.16
48577942.41	58621716.99	91148201.6	107960198.6	62199730.46
442954.0572	554546.8881	666763.4922	1601168.904	767082.2239
	2665834.211	3164137.924	3518282.484	
22723244	20204928.03	13877915.79	14763845.75	21751915.17
10060769.27	7866896.73	11553005.33	12977275.8	10353718.69
630976.8907	3082071.683	1700291.897		5010484.044
79761933.61	105951643.7	84357749.42	93464386.36	79801652.5
70282.71073	2345120.411	2612269.735	2180020.129	1870083.069
21931094.26	19816665.67	6704137.536	20533639.34	8695656.024
78349039.94	107618069	119336726	36577444.04	186421090.2
8607182.756	11033823.5	11420445.21	15113107.63	11183814.82
43903352.26	25561050.05	43878566.79	12998274.48	23459515.44
2342154.996	3963350.698	3932597.408	4850832.127	3967010.403
1306938.814	889484.5222	670352.467	1645756.802	488612.9708
34284755.87	32557548.38	54094179.13	41022247.69	19132418.38
7297649017	7284820055	8661321248	6423701462	6572083294
339712445.3	301540492.5	207302150.4	314730025.5	256400517.2
2181485.257	3101487.523	2471656.537	2339952.808	2211873.434
642863.242	976555.8836	703117.2637	550168.2995	541941.5492
9375194.71	11955873.21	16385012.57	16356762.88	17606805.68
8031043.178	12217441.96	14749630.7	30582728.55	14344011.79
4510084.459	3531695.649	4013640.243	4368947.411	3709760.457
1153932.356	1872379.054	2129781.155	2347108.496	2036875.499
3063404.5				
65941672.64	46378226.13	72805066.6	79480506.05	65451200.68
981186066	1622178314	897054696.2	869355863.1	1011683763
2902006.558	7796434.375	12074900.97	14717320.59	10246769.19
10533853.14	14562420.98	16657307.05	10636747.51	10091329.65
41045556.29	39734727.69	65308849.57	58679080.58	58403711.32
	1163474.891	2732460.808		
507717339.9	520725238.2	917886372.7	582100796.4	475845547
9291459.201	14679955.93	24697753.52	46091634.94	15684666.29
8428332.286	19977482.82	23282398.7	23864833.88	21532660.29
5088198.548	7662318.975	7904928.285	4736589.197	2216341.226
2437164.568	4767137.757	5306092.248	8000392.375	3005575.108
6185205.073	2567529.971	6848115.502	5678351.674	4727652.98
4946125.964	7980975.781	7329685.613	6837494.899	6495697.004
	2672671.59			

17078451.14	24794094.55	23224161.06	21411503.07	15300242.53
1180979.493	5672407.793	2457564.468	1396517.001	790737.5939
175872234.9	261586707.1	231481871.1	258493473.7	343146651.2
	2703653.858	6913664.662	429502.6993	278697.0454
6457460.292	10843106.53	16671363.18	14411534.39	16647755.06
470277059.5	550325223.6	601701495.3	541698905.8	704388152.4
10514304.35	16711927.97	17553406.4	16066820.66	14794043.77
3922493.38	6026831.788		9668365.011	5304023.14
6264109.682	11686577.77	17390995.3	12901550.43	8136534.23
83242770.76	149499140.4	90875772.76	115304253.9	136082270.8
1952628.816	485025.5338	2100826.411	2186377.71	1873728.965
13673855.99	14578607.96	8327420.859	7418106.111	10093291.78
		566822.1382		
33311523.72	38169895.58	31639844.38	33934383.26	37545784.59
1434200.355	1868759.279	2081729.171	1857499.04	1672142.104
	353719.625	549774.2059	547485.105	375335.4467
3029502.255	4913042.021	6157167.486	5070541.053	7530712.243
7815171.507	2924170.694	4203374.396	5672138.634	3967279.971
6740130.059	6164610.697	5673195.092	6666938.257	7389709.839
120479.0623	213326.7219	259498.2364	180876.7108	193341.1216
7867636172	7140185972	5597265729	6588727852	7772040446
17089332.12	8203326.91	12560890.54	18251269.45	10878892.92
6586653.428	1686597.561	1993647.54	1960321.274	2711722.53
52749.88072		28219.15204	98280.04073	
2824838.106	2311044.936	2822952.016		2252011.621
3365759.327	5099014.998	5499403.839	5175239.246	4132917.47
3734058.694	4194873.973	4063473.595	3853434.276	4398718.882
75336737.43	94279283.54	75081244.56	85684226.8	96566249.7
519902933.4	398351393	343906867.8	459914553	404684394.6
3653330.184	5002222.735	3598235.552	2913637.938	2050084.594
2619770.484	9156580.676	4001860.069	6037717.283	4008607.69
206353731.6	205293505.9	180963828.6	157627957.4	189355233.7
1055881.874	2142432.248	1336761.722	1039443.914	1068815.26
392529.0949	661996.6306	209943.57	418830.4178	351451.8873
2701833.735	3192439.184	3526700.582	3410482.928	3954390.807
		334927.0683	554887.2584	518388.3405
1686054.695	1201236.846	1033143.705	1268538.467	1133218.003
52375258.61	11359649.03	22273641.14	14452135.61	19233408.13
916647.9468	1038498.939	1186653.703	1201612.463	2199561.211
2873220527	3210824787	3291503302	4130584605	3445344775
325447.3602	538089.873	991730.812	623870.1468	822126.3267
1588597.316	1028753.671		1922242.274	1582609.289
2238315501	2237785638	1876099244	1875871842	1660423738
4586072.039	4613327.243	3369120.885	3587164.425	6506047.661
200423833.5	183500888.7	248924631.4	167216713.4	484026162.6
179717.0241	163144.4251	186653.462		108687.4959
252037.2774	1968522.285	359838.7675	1349197.685	2283266.156
	9267003.8	6140609.213		

26225743.4	35863172.6	44456442.76	51342095.71	46357438.22
52555220.82	46336961.58	48951917.03	54412592.32	65563408.71
1488787190	761335471.7	552331523.7	405936587.1	464159677.7
1961289.342	40891.53122	1413123.575	2959231.079	631722.8363
	906350.1995	1137975.582	715410.7672	1104551.024
750459.6719	394438.1994	771770.5228	754878.1866	349107.0927
153623244.9	156871429.6	127426339.6	292131248	130796261.4
32287264.29	44773155.54	21302709.78	24200021.58	36092932.09
		536645.2067	809075.6569	
14814366.23	16785381.43	20683323.49	24559575.75	24730365.88
		408927.4507		
6723481.517	4843818.234	6512015.71	4800318.125	5130627.788
5264433.169	8328279.253	6733162.781	9455205.339	8435907.683
172577.8613	294085.6995	708432.167	904380.034	393302.308
1205077904	1064993552	1199898785	1821151890	1089124996
5151083.779	5406670.679	3312091.035	2331920.278	4889566.43
13144630.9	11123747.04	13561935.5	12567509.48	11156419.8
37324215.44	39363508.77	18968724.55	20382522.12	35343758.58
14361089.35	10252882.59	13941572.37	21399462.85	16278700.61
			617834.9073	883784.1776
		1170399.638		321382.4774
581136.7132		1183659.723	1048190.958	1626412.425
7365802.298	7920990.05	7241797.029	8372183.956	8033831.316
307388.0563	274621.8079		86496.44055	246414.1264
1851652.274	2415418.269	1583906.806	1288219.468	1502350.314
		54261.01583		
154321573	81593140.46	138448686.2	139352218.3	127842849.5
6443512.636	4921797.686	3729884.456	3777536.407	6188211.538
9549930.422	11728166.96	7985736.507	9814984.358	10987443.29
10639704.37	5214395.433	6585645.492	5545276.925	5745961.827
1052570.286	787516.232	4194870.77		1300089.702
	560894.9875			
329994138.1	272887371.4	365255356.9	469751309.7	342879458
7739946.909	6873955.358	9852385.851	13476326.1	11820703.61
1826307.667	948220.3358	4424602.788	2275890.057	2111618.025
2669912.157	4422969.359	2416049.971	2895401.681	3321359.961
357479.3136	9271608.889	7442859.323	237914.8191	2918232.9
				731423.1759
401326791.4	312920961	433703145.7	391813644.4	422807682.8
661804414.8	913817753.9	681738004.1	788817794.4	953705375.2
11939858.52	14454852.64	9915307.937	12107371.01	13477529.95
45170671.92	38264064.8	34176032.63	36704072.79	46786487.37
177099.17	2673155.384	3159773.423	5039990.055	285076.2616
63190075.73	51099403.04	41724699.88	34429299.85	46417747.52
6487183.14	5567355.596	7101083.98	7745327.285	8002431.785
4205985.527	690427.4836	8992997.413	551476.6745	1525389.765
14118456.97	21433340.04	19790810.43	22622484.89	19745947.25

		261779.2163	1377009.96	547696.9678
6381860.498	7304276.044	5049826.562	5076873.919	4521773.441
1082663451	1269022524	780348876	896565090.1	882844293.8
20000057.35	30164397.92	38288796.52	35299426.28	41419208.43
6139434.332	5253064.572	11431457.89	7065251.714	5413586.175
38772814.82	35835752.47	47008814.05	36811570.67	40826928.52
6697408.533	5549788.395	8233490.235	6544626.525	7173024.087
31478616.03	39572123.42	49488707.29	25581309.48	35728129.86
69418623.92	85009502.97	95198209.15	90273641.29	85560705.48
282590.7438	120754.6744	217389.8937	353357.4979	217307.7037
37474606.33	52481622	68127320.82	29673486.45	54331152.53
171451.7029	168679.8875	258482.5365	272284.4984	263578.0317
151942.0346	75399.62682	93085.29442	117716.9379	82514.24134
617013.9278	1449436.592	564695.3174	543533.1388	1070085.45
2134791.136	461009.9562	1283818.635	4868737.138	2324235.477
		158521.3896	750833.8285	
4417126.168	5463933.722	2907605.693	2048312.925	2831220.967
	1756094.586	694884.9706	1004362.901	1015272.714
634102162.2	404669990	332186404.2	368603355.2	203379149.1
50083450.08	54553022.11	42922526.34	63241063.47	44983553.16
309875.6772	297727.2609	343881.3714	368011.7115	321994.1596
6263457.823	8998185.683	8436277.872	11597083.68	8107432.838
	2263240.031	1283709.712	1009894.874	1642582.574
27331657.61	27593258.66	47146780.53	45406174.45	51522675.63
3443796636	2515857555	3528569076	3623996100	4239738768
9978161.214	9166131.595	16543793.26	9451387.165	9317626.43
620246.9188	227678.7487	403353.3349	531347.6147	565760.714
53522944.09	69661860.02	65764368.45	82721255.91	80081541.7
6912191.293	7623756.873	6161950.5	6168998.562	5782809.298
43586484.52	36984536.1	37228953.99	43940203.21	35227036.94
2720428.792	2108890.46	2905626	1976622.787	2688169.359
34854171.93	62737633.89	26592114.5	36515401.73	18139603.47
27783248538	35992635141	35301329587	44762249812	43785561072
	472037.878	822848.0841		
537966107.2	558367416.6	625516135.7	505937227	767498268.2
13322899.56	18117468.99	13683169.28	10474500.05	10814260.28
3490867.007	4746223.45	4396083.924	3107079.001	4799394.095
2040978.076	3947593.195	4879897.219	2494547.389	1772865.806
1692.685321			6975.669849	3714.673576
112281.4995		71560.2718		
1394674.699	1216041.544	1172106.848	1223903.268	1705855.736
112564.7488	147412.646	53829.91075		130432.9664
3481968.427	2176871.958	2097230.9	3969955.588	3685533.819
1591561.99	1388248.444	1431526.063	1751457.565	1428307.977
		221412.8813	263075.805	101185.1594
1671275.802	2521631.037	3766751.45	4486344.897	4534319.972
897584.4738	3460952.332	562899.7366	899089.2939	574686.2557

7775952.633	13019119.42	12067542.5	8460315.743	11248923.39
	607414.5061			
8204329.752	19195716.68	10896674.28	35848496.57	13987684.15
1120484.817	832331.0176	692625.631	1370249.411	1582305.49
733760.9779	2634590.774	3951676.363	1093674.108	4094336.156
361445.7587	499805.2004	462576.3971	468642.7426	399044.0345
294939456	653107961.9	196652789.4	270064462.6	175767787.7
15391053643	15260486534	13098300171	14993567769	18112108640
		137405.5961		
4680389.994	4064838.838	4610258.044	4998854.792	5682103.252
35382170.7	44102216.11	35951530.96	30833395.5	36558044.47
2867104.485	3710013.975	4626551.694	4384331.025	5026982.793
329756281.1	489346638.3	305103349	405634454.3	442221816.5
295247156.7	297810543.1	314989591	267036616.1	297867693.5
2826716027	2519028858	3013380951	2547152322	3336731257
215006.3626		213858.6012	179220.5652	183381.4088
1093660.107	1386473.988	2102825.765	1779099.051	874387.4492
1423831.911	3167745.728	4483778.838	6195172.844	2514492.26
2632937.279	2512237.479	3080174.702	2332003.852	4030559.09
2986477.165	3248460.488	1873380.198	3130360.396	3133316.806
3785808.453	6371720.407	4108739.482	5338966.288	3584862.483
3109288975	3184988561	3705200471	2967898429	3192783396
915385970.5	1540625498	1013181809	915386291.2	1835131034
1013177.149	3100165.985	5998861.896	3122488.745	1428032.395
24511472525	23389855557	45616335507	42525897690	22907875156
6389317.769	6009942.541	5055749.825	6830164.95	7480211.58
1075039.893	1104725.404	1265799.824	2839904.619	1145736.034
3157555577	4194737726	2749288071	2165931996	2982835171
3859056.639	3557426.758	6598824.445	4065059.587	4349464.164
8099860.015	10523958.39	10486360.26	10522279.14	11688061.92
1115930.191	1356344.19	498599.1925	460700.7504	682679.6046
2788907.784	4109265.666			141558.656
201401.7799	82524.84685	137859.5526	518575.1948	
31272713.59	25593619.12	22651235.86	19496217.6	22848553.12
12755017.5	9545015.963	11537958.32	13096055.68	11405027.44
			1031741.16	
261417.4218	361056.1883	410411.6598	396486.852	357062.1392
1504862.856	2692977.287	1971983.133	3420498.142	3391193.375
641889.4252	763513.0088	1273080.226	435611.3854	273511.2681
204433078.5	139314346.1	192834254.7	135392780.6	163203303.5
2546751.864	5142125.676	3122291.886	3260033.549	3280796.718
20594597.24	24420242.4	35392968.51	32575884.9	23694576.92
1275747.541	1352085.119	1505445.313	2566723.345	1567382.024
342735818.3	361912250.7	473775662.9	486749612.4	520132973.5
2202339.807	2480266.481	4081763.847	4718774.517	2643394.763
			572626.1199	

12866236214	10769627215	14872306124	10860830229	12682616230
697246.5415	733653.1189	342070.7809	489305.4061	444798.3265
183374379.2	301665153.2	230845852	210180064.5	152929907.4
3308348.892	2933249.243	1752215.839	1071951.469	1500294.983
23310424.81	30396536.82	27448145.92	36133227.2	25582408.17
251160510	269238978.5	178045698.4	281140809.7	318526000.1
1360499.678		1493943.824	1182397.891	1601458.649
102542354.4	142415683.5	96463507.3	98865908.43	99359803.41
697197.3363	346566.3087	788983.4013		507514.7542
1033861.277	773148.9472		321211.5667	235999.3018
1403086.179	1189785.497	1969787.885	2561055.715	1873391.957
182729317.6	125599503	168487344.9	149760369.7	194483655.6
13426719.2	15086245.12	16256340.83	17927595.1	14786236.43
5892902.447	6567029.762	5144453.861	4517979.205	3164050.814
2684793.739	4239783.627	10395688.28	11932764.78	11582269.71
	152559.3157	167045.1672		
3895056.986	5555679.857	3629738.612	3882374.454	3719727.224
45040096.98	42736165.72	56422508.46	43678372.4	38245750.09
128991.0696		148346.0486		800774.4479
656739.9449	557067.712	879467.7483	759576.431	659870.2715
24276812.94	19453615.32	18030190.74	20901019.96	19521934.02
7912769.63	5646630.531	4770480.441	5420411.433	4748390.454
50591893.61	66987035.65	60035250.9	123123615	135587607
4859725.443	5374310.109	8804245.99	3767651.767	4757669.4
25776558.26	31040255.32	27053108.1	36774853.09	36188253
653052.1962	559504.6174	624147.601	582851.8861	729957.2112
3934634.615	6608894.432	7801331.074	13822669.74	4727861.88
15958.09914	495805.7987	1218429.178	1404111.058	285219.2417
1168148.029	851364.0432	643090.1167	1641154.921	1096177.866
928824.3297	1306342.635	1315469.309	1541611.059	1324890.898
497033.0155	927323.6971	3225090.289	3326084.028	470631.6515
	6244149.011			
		3831386.25	1840055.923	
170950208.6	165356703.4	145340742.9	153582279.2	138021864
30926489.32	21311870.97	31254181.01	33890018.77	38702844.55
1127780277	1988402291	1677354413	1651452686	1622081004
928977.4676	962370.0431	987670.1706	1261138.504	1111622.028
8845957.053	15894007.55	16368717.05	15628577.64	15585309.23
	343105.034	146781.4942	160332.9888	115663.7614
478776.4881	347100.5643	474190.8381	656118.926	554659.3733
18054851.35	12036546.86	9378136.312	13232759.7	8391107.782
2868812.824	5757318.068	9000111.045	3086027.698	8362329.63
6854069.428	4209959.899	9608957.095	623745.8785	4179437.339
728942.3869	612438.9984	603157.2102	677045.8479	1072661.309
15630624.5	24013160.55	9078846.585	13956022.53	8601151.873

3877755.961				324326.5481
16648993.27	22023153.7	19243259.68	16649156.14	11811390.01
	1140850.784			792550.703
245814.2812	703487.5957	794214.8159	1576586.739	523361.5684
	759784.2647	1067974.747	966122.1747	119886.7618
199893526.5	203273807.9	220758302.8	225150498.8	204662529.5
6772169.137	3773374.334	5482666.355	8375123.461	7862617.456
1661985304	1457294173	1797323092	1499928897	1825457367
123848959.5	16153328.61	258233238.9	17407433.8	16874796.39
5297273.67	2969044.291	7613298.363	5930483.565	8409201.088
2112540151	2107177150	2266139287	129396814.8	1697599120
1614067.641	908633.8422	1220381.305	1829064.18	957995.7931
41896323.28	40060513.25	44724584.18	60796842.08	39642001.44
344122.4381	429306.7309	167642.1233	122636.0494	548321.0183
	651050.9955	3532813.217		522775.6818
11740909.98	11402373.62	3422288.429	9682687.981	12463920.64
15969620.94	33187037.5	39265072.13	63566595.11	37446072.85
1666443044	3177743800	3698778228	3033129143	3065909415
3618074.813	1900482.203	3419050.902	3994315.182	3684376.892
19313541.32	23181390.07	16641005.54	15635377.67	15008468.81
5045676.536	4195551.699	4269432.406	4824766.121	4758237.358
179346.4656	154847.5889	291978.1651	217988.4247	517889.5237
	14515.35104			
601739218.9	526800297.4	218759474.3	311768958	237919347.9
67324503.29	96170808.77	82975502.09	61195743.86	60715930.98
	540617.1114		622665.7868	
		1110612.344		
297700931	441706852.4	115986212.4	176071105.1	171956574.7
8906145.037	8031683.908	6562722.742	5747515.374	7435183.944
217650.0535				
			61222.18828	
3173556.126	5639787.905	5534580.947	4025741.456	7230356.788
134929747	260708796	172114234.1	163708198.1	148003324.8
44356426.21	35932480.05	38399140.38	44495344.82	41177439.09
279475.433	1654277.224	94013.85816		135545.5362
	360441.3921		737142.1392	
551031930.9	548960678.9	547869512.8	639214115.3	667966328.6
9581047136	12825775979	7300548551	18395640658	10720474587
	350581.9552	591586.6908	457081.6848	406661.1654
5693955.426	3450260.122	6476130.81	3737977.744	5062880.537
248680.6482	176121.4535	183384.7374	192153.1298	135365.5812
354806.2448	388633.3939	795985.0994	487928.8407	670999.3368
2201936.011	2711154.084	8161074.044	3839991.462	244642.6988
2784866.199	4552406.526	2096954.906	1269270.299	2416251.71
342196893.3	498908203.2	456169118.9	310575381.3	222124684.6
128637467.5	159636856.8	171030453	179587089	197307794.8

6180794.157	5068309.345	10307635.72	9732299.692	9534773.289
133391559.3	247361474.2	465791011.1	117883256.1	98615143.52
	212534.5426	1096118.459	1119538.747	1821214.122
76232937.91	73628742.03	106302947.9	122546370.5	98371631.52
	18363.7746	5025.542361	4389.561448	481854.7745
64837342.07	118636877.6	220038716	62699457.11	149135084.2
14682227.05	18058074.15	19637836.09	21135073.76	17110304.1
58799.317				62247.07072
480750.1647	271772.5207	599904.3765	867490.5047	639679.7644
131215739.8	111451698.5	98956106.87	119106933.1	129349654.4
	459814.827			229146.6223
232513.0513	443688.8181	974394.4442	1918815.384	291356.4605
10403367.92	937211.1259	16034515.29	1556191.28	821966.4466
82197257.71	124477659	95396516.13	72574815.92	107173784.5
6803070.96	6310613.046	4225718.777	8060441.609	4378065.226
1388733.435	1202337.619	1151376.141	1613234.941	1099672.534
608200.9377	927448.9868	587613.5626	759665.7454	661286.12
175339706.4	222039220.8	257707621.2	81996710.7	120494235.2
3093360112	2465901883	2354931237	1855369790	3181816561
1486715.868	1121492.104	599409.8161	616364.3172	679175.1782
348082.3069			1336656.635	422256.4531
784284.5473	792023.2448	1505561.621	2282975.912	907028.9353
	437920.2546			
7570223.723	5499406.796	5809987.059	5868760.887	6531982.996
460298.7367		1081838.64		815892.6672
388359.5088	637697.4551	623073.9984	801744.2738	723929.4917
1928340747	2612105511	3120924259	2406220145	2519593082
535717613.2	434674370.3	359678751.3	384768800.2	653302786.6
13392403.9	17031848.06	21061146.46	17887499.68	15978438.62
547689.8063	672215.502			1767148.665
7939467491	7948262696	7538487695	7601152170	9091006674
17688203.12	15670083.87	14109418.34	48481450.12	18594880.13
14622555.21	15232194.31	14707607.45	19948441.22	11472616.67
47153973.91	56350340.12	32555513.93	38515672.74	18789483.93
676611.9466	283770.65	358781.5588	101171.2934	191540.6834
6561028.42	7660875.468	5798777.564	8979983.603	6182422.608
12299348.57	7122720.54	3237134.407	9902046.387	13642408.2
1668922.855	1846650.527	4349783.542	2423990.686	2176815.175
13064628.34	11643247.88	15598649.27	21884996.17	14626284.5
11309025.54	12311758.97	12216799.95	10216751.77	10465158.03
3899959.795	704813.67	6479378.719	5627224.198	5096097.849
6635870.15	6037592.354	4370487.595	6003673.958	5468561.997
804648.1414	599949.5028	781054.6004		823375.9029
1573939.401	2326578.581	2164923.823	2296694.179	1265118.437
1125801.609	1140713.842	873932.2005	1098866.988	1650419.479

16920797.96	33075173.6	45095303.54	33431382.64	27081357.68
37958038.75	38494835.55	48681043.84	53258568.1	47187836.95
11926860.1	8776042.207	12124833.28	7310070.333	9172809.749
	1975249.29	3191388.436	1057575.495	1148222.827
120941635.3	134974073.1	108974499.1	92111961.01	145727483.6
536624.3308	1142552.917	195144.0817	995765.3848	764453.9419
44434.41763	118160.2745	100292.2739	35319.63463	46792.50086
1202180059	764260143.1	1118596609	872325153.1	1120308853
	547767.9226	364239.8951	601678.3119	450973.7093
7153423.458	8805310.273	7860693.63	24418974.1	7537786.19
9443534.56	14503413.19	17586112.39	14751962.82	15468387.2
1710814.254	3309867.846	3021087.363	4182258.134	2259043.32
2554925.53	2395870.439	1273305.15	1249914.349	2194130.678
2357525.475	3474707.591	3195660.476	5278969.343	3467032.84
		144727.3371	183795.1907	158938.9498
5558.733828	219490.6597	198019.2466	242991.8635	
4299630.994	3280407.139	3188396.418	5541192.032	2626703.926
1949892.916	2395647.377	2430457.841	3190798.849	3266352.335
4524036.78	4563852.941	3631550.932	3526017.462	4183848.206
25810764.21	39154770.07	32093208.48	22430319.05	29632986.18
115330099.2	118938175.5	109617155.1	117790373	112309091.7
1990646.831	1791745.328	2321649.481	3103641.727	2465665.136
2983038.154	3470660.29	3178001.959	1558719.417	2040916.736
480975.8573		501044.5483	180275.3966	
2190123.854	6182324.3	4389476.143	2726540.019	2632315.379
34156684837	30644365678	31088982932	29339539710	34085992432
284860734.5	349413443.7	260486662.7	350768797.2	340007327.4
7791983.608	13083511.61	15133619.52	13598097.1	9632857.439
16936493.91	21114140.51	13131677.71	20017070.2	19651397.67
53913.15963	54293.51414	112963.4993	72603.67633	52340.15006
978073.727	761519.7081	1239421.994	630688.529	738212.0471
		848592.0495	222132.1203	164930.2739
9593523.486	8768258.912	17420413.48	23733971.84	10351505.76
3921061.152	6400731.147	7855532.096	11518627.46	5407607.073
	440343.7239			
	225613.86		121360.7556	
225655.5453	285416.5906	98581.58612	251074.7635	348169.8255
3070035.508	2993094.066	3229295.108	3387976.469	2043874.178
109484349.8	178782345.8	128483718.9	116295433.7	153652375.4
284086.1867	518664.7715	526827.176	939629.3418	851370.3513
5586208.836	10202212.23	11326573.5	7306706.617	10263155.55
	406341.2942	447546.594	465280.8345	512422.85
19478852.99	19572432.91	21478387.76	18260423.82	12800516.88
47902010.52	39138924.99	29610686.57	39637346.74	30320777.31
6405637.332	5517352.22	7307301.721	8328607.277	3264325.475
3454133.862	2188242.596	1812981.094	3384235.545	1870007.507
137669177.7	126027182.3	181709172.5	101356580.1	85074866.78

19781265.13	19531124.85	31910015.02	40134233.41	15448408.94
10478847.34	17142571.06	18245027.91	14411704.77	8659061.714
618932.5584				
7125224.962	9310365.57	10319105.54	9503558.819	13743978.24
8763031.346	6804154.095	10829180.85	13960192.24	12886944.82
			2470798.209	
1078376.938	2431294.4	927955.7054	1967665.001	1354167.943
4584575.378	6925958.568	3047509.209	3706717.314	3650414.683
2711912.747	5402866.935	5288180.701	5710368.631	5642422.087
1594694.876	1065464.954	1465390.407	2091207.144	1435677.402
8624337.197	11319255.48	18310993.08	18958003.21	14668155.06
			439291.0606	
943634.1551	3441298.361	2223689.347	2471805.231	4088315.784
	480834.4211			432906.124
2663257.193	3455689.349	3292043.365	3564391.219	3378386.462
	99154.3582			160047.4632
1113700.414	1715259.56	1685032.948	1724323.56	1796225.585
765180135.9	1070102883	1059415605	922517215.9	718189053.2
19489528.53	19365317.88	25979856.09	26469928.27	27669700.56
219627.4024	213818.3349	159692.3984	212831.2139	
		252625.3058		246910.9575
136382936.2	120356926.2	116729929.3	140950895.4	127004624
16058.47553	18839.2781	11610.22312	15695.28201	17664.2954
53184043.22	49284693.53	65317061.57	52122876.66	54482257.96
654186.3549	789658.9286	546598.3763	753673.4596	928845.4762
893115.1386	775949.2154	587815.0174	665951.1445	
		931633.2774	626826.0465	496742.0055
662774.0271	782460.256	543019.9388	630494.5785	1278173.383
18618893.87	17379916.88	23312754.87	21677468.72	20922597.62
18557141.39	20420964.02	26740864.7	21693893.35	16220378.72
2947613.674	8233218.198	17265720.18	4219627.476	83137844.96
51912037.8	46131267.39	52380657.17	56044298.38	49783013.47
161189.4164	624149.7128	338499.926	988249.2591	595385.1867
110633908.3	92658306.54	72225773.33	103054611.5	94773686.27
9374409.92	10569623.71	9720091.629	13567045.64	12298837.8
214677.3228	409758.0856	359027.7834	938558.6615	224782.4669
802924926.6	1048027635	911724785.1	940224335.5	604204324.6
1444754.546	1406936.363	1851021.906	2870642.151	1926837.902
185287597.8	267408963.8	256095309.4	268701337.2	220577722.9
274422.231	673825.9357	451087.274	554590.46	331266.5621
11052970.31	15914839.28	15895795.23	14714843.54	12171371.94
104497.4379		122846.7337	150664.9006	102870.8599
365565.3322	532312.8076	1108259.054	852284.5939	541197.8628
176208642.9	146899289	179559705.9	124434270	175506183.6

673218.9136	644048.209	1347019.081	1063470.052	1194095.592
	2699033.663	2077323.821	617305.7599	2067616.655
12743236.16	15000347.17	13809414.46	17210897.87	15721638.35
11323953354	11073446441	9202197911	7858593417	12181420261
51272252.76	57552544.72	52312284.35	46109737.93	59753698.77
		253062.919	215413.5933	270035.6443
7571590.938	11016339.19	17443866.91	16183380.81	11341378.01
2757559.569	2937398.757	2721892.793	3083132.785	2826758.811
281290873.2	243537691.8	439062058.8	383146955.4	309305306.6
	983700.9257		1757519.617	4960743.974
560308.2065		1579400.492	1253534.94	1110195.496
3105227544	3163205013	2709804044	2180498640	2988485310
155351.4616	113149.019	80763.15106	169601.0447	98068.84028
13716443.65	22834764.64	21348748.6	22004140.83	15041929.52
2298379.084	2752144.839	1587279.905	2286124.746	1640121.51
1869658.724	2806311.716	2626510.925	1419938.541	4140840.385
8888219.414	10306409.41	10235939.94	10422225.91	14445430.46
		5409109.806		
880430.788	882046.6188	544451.9698	1450719.374	942640.4769
2123747.226	1191961.879	2327819.761	2319080.699	1731796.253
1561307.736	1943487.272	812365.1226	1833295.853	1294667.622
306676848	331947222.7	310680722.6	459540241.7	566903421.8
4120719.44	5056200.653	5156775.019	6829963.579	8196746.475
49754.84961	154271.0988		77600.63434	50435.41816
740578.3892	895445.9923	1261446.407	1151350.573	1277468.541
23847898.66	20409257.46	31611171.58	44950949.42	27988499.19
503659697.6	574124573.3	702371752.9	725869847.9	592853575.8
1354968.954	3253593.684	2143639.516	4568497.725	4813851.121
522226.2267	840340.2397	738614.1881	1018838.053	1315224.034
25953466.9	13419054.43	23293627.04	28059654.29	36708491.98
984903278.7	1094826589	1349008336	1279619129	1291157620
485815.9384		244890.9284		276976.4715
9069723.448	2009189.094	2568348.224	1494537.23	3028577.662
11829519.91	10704740.36	6152545.167	5111624.467	9421806.594
225192.8998	729251.8014	170107.8753	212752.3462	756766.9887
	455512.3161		1118415.25	730687.0542
1273406680	1478366890	1347012879	1766010293	1817660121
710468.4107	1469527.577	1205174.345	2124141.827	1322952.669
13682801.63	349179.4774	44382.21126	9295305.393	8662349.033
60792.48831		113139.4683	77803.42211	
4675475.494	4866074.405	5091193.497	4773401.553	6048866.637
12386382.53	16556325.73	10522554.96	21984395.76	15463490.92
768613297.6	816619187.3	670681747.9	787966845.8	417161077.7
36279519.12	27567833.51	44444426.53	43812903.58	27157442.59
	249351.5305		834585.411	687131.6619
166000606.8	191070540.7	96061378.6	124613457.9	131070196.6
207977049.2	163472696	240295467	267772475.9	211406518.8

78531.62393	80230.50078	570337.5021	153986.8667	77758.40426
1805885.43	1390698.539	2368393.319	2042660.504	1540521.677
828736296.3	1141124788	857691835	880485198.4	1498396038
6130940.133	10342780.33	7282188.511	9155863.323	7997792.955
1364962205	1542150962	1535897229	1131278125	1266737043
21412962.61	16945762.79	22948965.98	20900102.27	15970632.97
735514.2455	281383.1811			723274.3973
	326923.3071	219091.505	293740.3418	577030.5964
16801567.48	17420451.67	17781085.46	24161484.25	23841489.6
10406689.67	10064585.66	9406792.239	9058617.728	13325595.47
1224987.337	1252280.097	1474090.643	1917677.173	2323667.784
2106256.931	2437391.484	3052966.207	2910729.171	2625455.551
2107255.292	1256230.783	1574687.88	1315928.633	1520894.589
1101164.395	1174078.323	1315391.456	1436395.878	1446099.413
		1292887.073	797176.9174	
2372269.949	2754504.665	6461053.243	2398871.955	2129217.224
6730800410	7525526621	7039525504	6490798718	7150384916
	201738.2037		359546.2941	356284.0334
86589302.36	78002428.82	86626172.47	96135942.63	122365737.9
385006.221	789784.446	557593.825	1340941.012	720262.8116
9394911.274	8196733.573	9944360.593	25419.51276	9044839.341
897531.4606	747510.24	828659.0746	488946.93	757668.8342
3800358716	3799347548	4716551131	2774464064	3750900807
839919.5871	809396.2865	1456800.483	986987.2448	
6513692.108	8247543.732	11048347.38	11855853.04	11973079.75
19428612.07	21384106.61	30957860.41	30492629.07	31129366.07
1299657.287	1507510.355	1076768.171	2655134.048	1356374.185
409939.7622	1544159.012	343319.5903	308809.6177	524121.1785
767637.1897	707852.3719	921776.2357	578280.7993	807121.039
6205487381	8056509070	6097593357	6085557140	6843767935
114314.5502	115924.6164	329564.3037	370741.2855	171667.4402
1144530.186	383386.5276	1185512.929	717421.3584	1353991.865
17941924.31	19879733.18	15789750.63	17229732.36	28400198.53
11549058.49	15922116.67	12655829.42	14230052.35	10832211.07
198135.9635	470287.9404	178245.8596	284171.7935	156857.9082
32455074.31	26815291.04	30307696.9	27498015.1	28064382.91
2399384.669	3702932.65	3219655.688	4709023.797	4223752.173
3762225.002	2031968.323	2940585.762	3837181.214	2923191.244
1884302.118	2140059.165	2318684.776	1618196.733	1841644.96
2509862.237			1584479.679	1821413.338
36639562.12	26623966.2	54276868.36	38500474.05	37356698.11
29979460.21	18668782.72	38585265.55	25673249.52	24707317.66
1765744	1138454.49	584436.5266	1479116.551	874640.413
740439.4812	417959.9486	820515.5294	719111.4862	705074.3161
1937566.082	4216697.228	4977895.549	2719083.166	4166469.449
24056148.2	26288498.91	25805720.4	33420379.23	25628435.31

1420734.792	984068.179	1313205.596	1456975.18	1896317.257
325488323.4	356002248.4	431463643.4	446658903	339503089.2
3306519.894	1514834.393	1775359.215	1698835.001	2862051.5
4626547.735	4507415.437	4674877.528	4480006.384	3061873.25
27767.741	155724.6927	1270850.238	19637.3286	13460.28365
9105753.66	6642344.843	13858858.85	13107911.33	13484275.25
822343.1446	1091546.464	1173056.496	1149394.166	1298002.297
2248474213	2606030556	3069726091	2946383917	2036936033
96086370.53	55151569.29	171096684.3	67561877.89	43562746.47
17582270.99	15540283.99	22440251.37	17398047.29	29622606.17
675954.579	1316683.608	682475.3611	508631.2628	533899.0736
41470426.43	36585066	41353669.77	46696589.57	35417990.77
16757326.83	19067558.5	19721280.28	20742784.56	23306985.4
175763.5442		413221.3193	533470.1524	381525.3065
2830268.371	5051227.424	3338921.786	2924718.901	3333860.097
6364588.224	6608367.692	4825479.363	4556765.605	2816731.488
11147569.76	11937046.41	14239824.58	13033020.82	12460884.61
2696568.424	1734160.958	2565886.996	2109173.235	2813935.194
7554321.689	6625684.921	10568344.08	12418739.85	9469533.587
3186607.021	300540.0922	1416898.717	1542355.897	1390661.634
4039395.17	2786382.552	3853782.696	3660236.141	3475072.327
7510758.692	7234496.439	6214755.761	4633720.941	5056840.506
473891.3659	466367.9218	790706.7	692309.3566	487700.9696
61991.90875	112722.7267	52957.37054	74572.97263	124101.3724
13184250.57	28907670.59	44867016.6	21952320.75	23356009.97
13890970.45	10691166.15	12014697.53	15291432.04	12425043.68
4094121.856	3050723.869	3462359.057	3382461.87	5283725.857
653787.0367	938391.3958	1037679.764	678933.4014	279673.6302
6147075.842	8069813.875	7452718.315	6920281.736	6703444.412
711075.2383	1118702.33	1097478.482	877241.4085	
		585410.5294	634001.2719	393977.6094
979295.6821	1367798.575	659036.2026	425227.018	799623.1751
15018432.05	19580670.05	17536056.86	23523337.79	22920465.69
24331087.06	37812804.44	33694304.76	37015435.84	17555419.83
1295936179	1316478368	1368894503	1643788303	1272326354
4330718.705	2057769.06	3817488.266	3330628.594	2945053.504
1353153.969	1743734.214	2765665.8	2199195.792	2343979.478
3402642.453	4052315.335	4451747.041	4085487.854	5146603.013
1025630.973	1706715.646	3072997.582	3565575.598	1834884.796
1076110.869	1299029.756	1017040.821	850465.932	
4149963.778	4368896.758	5321759.686	5015460.369	4412446.103
1861293.926	2093487.024	1701821.847	2122683.11	2012816.843
	175541.9176			85716.96831
1196366.047	2475293.759	1234782.86	2472327.715	2730811.843
8414661.678	7571930.78	11982348.95	9985630.748	7424434.457
1171328.634	740727.8207	1012919.359	1035259.373	716134.8263

214209918.1	362849601.9	216837182.1	593259770	331960301.4
8885178.447	9239568.584	6502985.892	7325480.961	6223151.598
1106129.482	4750784.093	3317314.325	3114961.024	5068829.026
311778.7195	243533.6634	403001.6232	301920.6781	122427.0378
4192205.051	4707784.832	7352560.898	5694000.297	4816833.942
575730.6581	359303.8786	518363.3616	313548.0018	395045.4814
5940606.283	4208616.935	5985968.716	3306424.937	5665071.729
1930940.893	2086814.956	2050457.217	2234354.056	1917362.727
2398651.513	3428384.376	2032082.59	5846854.256	2297360.53
4168749.06	5058990.54	5192865.737	5067875.176	6521752.845
2620810.509	2350089.394	246819.0844	3761724.401	3216931.855
564980.3016	463030.1528	681826.9622	409008.4389	896235.4593
1515360.953	1670817.285	2718605.576	2247017.717	2178855.907
	444241.1783	124214.4847	467363.9195	
34872309.06	30760858.66	50246920.52	38031350.24	29836052.82
34727809279	30817753134	21626315943	25619088273	29907863075
537651.2264	324549.6349	538050.8848	643636.3024	495432.479
1097578.532	847463.0211	969309.7474	1083662.206	1077751.071
12980014.85	10354144.51	23558828.05	22218190.03	10175791.36
1632434.963	1652239.796	1474990.715	2380897.284	
589290.6494			919151.4197	
117544268.1	203257211.3	103702714.2	87880106.17	133566110.7
10760983.87	15618322.96	12135312.85	12539808.3	16787306.08
2478019.01	1093917.897	1265962.749	2194465.955	2018185.301
1686027.734	4726527.634	1612247.911	1652037.867	1285191.058
38328658.09	33312878.41	55834025.81	44332441.69	60938804.37
99931999.36	106413806.6	121217300	99157639.51	109491492.8
11110442.2	14821422.81	17496632.18	16487329.66	18284593.64
7272617.659	7131392.248	9070694.901	7781179.45	6517165.767
3259467.393	8816252.72	4656147.052	2359374.167	4633693.072
19945771.16	17425203.42	13189359.37	18187404.22	16894654.13
1115385.651	876666.3357	1294032.577	1054843.926	1004759.056
467535.2886	464883.1573	467961.2544	459761.4912	391656.8328
838961991.7	907921084.2	980390923.6	968555350.8	1122399166
461639.7771		456058.7409	373729.7487	1037454.895
1607319.721				876707.4693
2238406.854	4608847.045	3001688.289	3152108.558	2324482.276
656225.2324	481392.2847	1024853.202	672457.0677	462827.6817
38140040.32	29889802.7	26003536.56	33596575.14	49504198.89
71617.3856	75534.32462	44882.28543	54266.75238	43923.17892
1475288.509	1719293.098	1117926.56	821692.7767	1167539.878
7363322.284	5961369.463	5767114.689	1312388.021	6543483.489
966640.6345	863294.8402	1521729.124	1083391.835	
1938532.715	2764260.819	2531979.184	2264608.029	2617933.773
734719.6375	958223.8501		853512.7083	478711.2603
10882365.19	13982287.11	5802623.357	8999103.91	18218037.68

223324.5752	324135.621	418842.7498	496670.615	432097.1542
3529998926	4180375509	4185071181	3502974795	2747387939
1122885.742			819677.0515	
823742.2874	5212992.557	1777120.337	3951656.719	3114317.73
10618184.92	9187587.141	11782785.46	14550112.51	15944601.48
225556.8259	773442.6815	1136829.003	1200640.301	995666.675
4438469.5	4656276.992	5966398.801	4662539.071	3561894.726
15064039.38	75091574.01	14177621.19	17165773.2	18397238.53
				713085.7197
22854319.69	29191977.83	27569647.27	35873689.24	32109022.27
38063743.29	39432126.37	41658863.73	55065816.33	54750627.36
9428316.386	14289372.18	15245131.62	17916774.68	18803890.31
25532584.43	19768561.18	29970864.57	42947254.91	32704309.8
328535.5977	554514.8919	298162.1307	500727.2738	412494.0845
263755170.9	262037238.1	522873211.9	704833713.2	570916704.4

Intensity A_SYS_1	Intensity A_CW_2	Intensity A_ZAM_3	Intensity A_LSQ_4	Intensity A_YSZ_5
135011015.4	153894395.8	313108975.2	275226544.1	155696473.3
591974.2332	443687.2172	393902.9556	494155.8032	4756.445869
5921742.913	5298917.012	5068986.863	3246722.06	4813107.187
3264006.812	2668412.533	1659806.264	3524460.955	1539333.844
914168.2855				1398420.845
88815288.4	93261072.33	89446487.94	68614442.37	84822160.8
7038370.623	3662034.227	3552504.245	4375475.978	4030418.446
3624811.49	2817941.709	2269715.518	2065845.943	1562104.914
13304284473	10351684607	9465773056	10223960395	13570963884
	20421.32316	20952.8894	16833.10508	19293.31052
1140869.94	799729.7705	1461930.666	975728.737	
3055133.203	2171561.354	1686923.992	1132275.894	1589845.117
30293719.48	47304662.04	66848032.41	14432087.42	51641913.8
	3801223.461			258684.3939
		497270.6465		775577.7691
36854156.64	19669304.05	22867686.09	16628168.25	19890881.14
2010176751	2062780056	1866926973	2649560484	1969723536
10650609.32	964503.8095	548079.9812	8776813.032	468969.9799
40039290.48	88036999.41	69801869.14	27542898.73	133114321.7
2256060.554	2007843.278	1814205.534	1739841.233	2632385.936
2966671745	3295087136	2729596945	2572308588	2559118700
51585.64676	45000.55622	69162.94899	23867.71593	12684.05347
111506403.7	81151928.88	64032351.95	41986560.17	104404697
53949879.81	50382446.6	57809904.79	43891904.16	33853574.31
			949655.5947	1314929.549
3446231.764	5821872.167	5085388.267	6357722.744	6177773.743
5774343494	8708473747	9610523396	9900361249	7949329214
434339.4914			266364.3558	
3592611.651	1278028.174	2865681.893	1936775.91	916459.4691
				287691.7419
	8044949.042			
8444903.75	6992800.566	9782051.099	5480487.331	5290892.973
23623625.16	13343497.69	40063617.75	15250429.48	17523083.56
937954.1006	858800.2421	490797.2401	325690.5865	584814.1322
21528005.48	11639410.06	11172598.3	17475277.85	20029330.46
4679.516438	170016.8641			
12534422.5	16937086.55	18608782.55	21204070.91	29202626.04
396735.3492	381020.4668	530966.0603	2926169.476	2034131.089
71012722.68	75891433.26	80022811.65	73492452	99374669.66
506049.3452			461438.6454	715879.0993
185622.8204		218586.7011	111509.1464	147478.0309
366852.9251		894852.038	766443.8657	

			33187.34467	
			180360.116	
4046088.408	3239885.318	1889675.88	2386375.622	1401806.959
1583920.168	1267364.43	915529.8482	1579307	883319.9772
1636548.88	809097.0018	1412923.769	1107428.367	
980137.5453	2475123.76		814377.9653	1995879.802
				86004.70687
42719.99527	107113.804		955149.0988	109942.2362
1551968.95	825909.1507	1103393.862	1154551.952	1131439.557
6581218.864	5237366.332	4649055.028	4519773.368	5712370.539
2177174.558	2462951.998	4719658.322	2651252.616	1607289.438
2048144071	1549569540	2074114555	2017389060	2500204746
2525409.563	3815839.121	2037281.743	4650308.26	2335351.27
996721.6571	890735.0077	944843.0469	1174054.598	985803.9823
2000781265	241999.4538	1771669628	2695545155	698157.6569
709064.9561	700551.859	344187.405	409045.2007	
43376560.62	33447579.47	15493628.39	24876560.62	38089164.34
6511644.282	6681041.35	6241297.953	6299621.389	4471620.429
28358329.13	6958003.452	21888377.04	20147482.66	16056496.81
320809431.4	163242362.4	306900709.3	267405056.7	144355200.7
2430233.064	5196297.812	5042023.767	4367270.628	4979478.494
4106279.243	5926756.206	9888364.244	11322644.54	10053187.54
32100.79467	40670154.73	37671.00789	1498.944064	
347676.1283	390920.008	513116.3996	67921.41925	24691.11528
539339.9646	306254.9125	204595.609	479454.8911	297650.6811
4444256.052	3411319.491	3232184.021	3639156.957	2114329.649
3977556.885	6848735.422	5701800.839	5496984.758	6232169.42
2123819.54	567975.3588	2345045.501	3507782.005	4635376.358
3755703.362	3238172.932	4159769.016	3769535.236	1131156.378
2074492.634	1867164.27	2344478.31	2725342.527	2359543.723
886732.495	740182.1409	1218178.712	925497.3186	1656276.295
14486389.79	11069079.41	6742608.414		
799720.4786	626603.205	1709958.715	276701.017	933802.8891
32345614.35	36079855.73	26311927.77	22773855.73	29799237.12
6564991.976	9274998.447	4533960.299	1739784.352	211922.9608
13619683.64	9629825.623	342557336.7	8179981.056	6598779.981
37028896.51	20300886.59	25041595.78	41913467.81	28434476.83
5478499.284	7980982.58	7899224.021	5815306.598	13974044.09
	487767.7449			1038965.479
248623.4985	77877.67124	534458.8426	76680.16252	47311.17356
379808.7373	316581.6582	499664.6936	188196.5439	569110.7833
202132.8701	147504.8198	183771.8452	270266.9373	
4853805.744	4883826.434	3847904.746	5201220.092	2396342.974
1213104120	1230057318	1094732647	1116383829	1521216387
649891.0433	567623.1137	672757.2243	482671.7004	366106.1966

1680734.808		1763504.106	1449443.065	860734.2276
1463356.949	988571.9219	1443054.084	959302.2945	1025555.786
470361.7831	407739.2392	596902.5246	206234.5376	
35841269.2	28368773.19	33975343.05	44162007.38	36913933.59
251246662	336613475.6	314375661.9	268830997.9	287663535.8
28439221.03	14069954.58	31770740.47	12022973.5	36197517.88
4808566.661	4871723.564	4329061.304	5254613.706	7836343.774
14356774.11	12925565.66	6607019.168	10779885.82	13973250.46
852698.6705	560135.6052	817355.8309	807059.0132	688197.9652
346509.0832				404738.6595
22558972.16	21221266.38	22119480.3	13733532.81	14362944.68
39355396.53	27726375.71	28814836.46	25710375.3	25958170.92
6935625.349	10380877.23	10050850.09	10802271.13	7759928.27
139842.7161	20048.0869	35505.45846	30857.19248	29857.39249
18858692.57	15293126.2	20690217.37	16522437.76	10459407.63
202374.8361	183634.2261	162963.7465	184196.824	62001.7806
9772255263	7274025563	8820669237	9262083726	8728130818
281558.828	125522.2834	231802.8722	223946.4596	127371.2487
24435.57347	34109.04055	27612.98423	38666.88093	22804.65776
3758150.33	2342873.069	2006446.749	2921368.965	3864239.866
1267747310	944744276.2	1145208111	1069221928	1137142427
794894.191	588154.9386	565957.9116		478219.7553
3659551375	2787725774	2335479736	1778970882	2488518952
243054.5704	59113.76787	140735.4056	242199.5606	67054.73616
808300190.2	997414610	818286594.6	850433301.5	1033318702
57095.99575			36943.38024	
377690.5685	261049.8124	242251.0052	117614.7523	423026.4549
353471.3952		180847.77	524165.2732	636186.23
208658.7381	148863.6052			3271117.35
10446292.29	6641297.016	9070102.999	6313470.829	5074878.322
9403712.283	13072519.51	8094164.874	13777156.32	29533315.69
9632828.949	4135574.734	1563362.843	3627634.362	4177649.537
754219.9383	437456.791	1824204.923	700752.0373	372150.5071
32057589.45	31920089.25	24304498.94	18288349.49	20891994.53
34397443.98	39885891.12	38810517.84	35330721.22	36254884.96
71494.60153	71675.63618	73091.01517	78292.05972	49795.60978
		3962220.014	5588042.444	5165142.456
985050.5548	659034.7043	716309.9989	481872.5712	724360.4186
	758063.0449	274757.5071	219781.0182	323227.2693
275916812.9	260770590.8	282305155.8	396606205.5	337665800
4722258.653	5413666.369	4479225.326	6425976.446	3573628.486
147468383.8	136246369.1	220060888.3	194215968.4	224275609.7
5255846.981	4512468.9	4048695.423	2492055.685	6729841.768
13807054.49	10691619.25	19960828.37	11419299.55	4516428.274
6669307.818	6266349.038	5551559.577	6208302.232	6244828.057

108148.6426		834587.3565		
686161.3984	361032.2759	775794.1452	350588.9353	360960.9346
496573.3366			450698.1151	384824.2926
5817387.955	9581101.083	10995943	15077732.17	5510984.964
		468394.6572		
395169.0769	224315.6973	328918.452	246785.1136	182339.8003
29701507.36	13411891.61	17608797.18	29833673.2	25381657.02
2333843.538	2122847.91	1177140.598	2336574.302	1069795.845
226202.8316				
99680829.71	125917151.1	91727733.04	139038597.2	117644916.4
67931.0133	131549.3118			
24339173.86	26587169.02	19387543.94	26486013.39	22497989.34
2627199.675	744926.0101		935817.3193	674319.13
5076323.808	6423513.104	4565953.343	5927938.018	4882939.929
99886346.04	75688268.02	74029437.86	58811507.33	61730029.33
1772086.699	1522770.9	1098715.163	1565726.915	1571274.646
2895636.776	689165.6533	3493448.315	334095.6201	895844.9544
3611770.573	2708600.207	2613159.481	955058.5275	2169378.965
5590578.981	4917188.963	6995036.836	5304782.749	2629030.53
513330.037		309668.4561	208970.9057	
527094.1002	1039316.361	667830.209	475784.7308	586361.3638
2837801.446	4208557.342	4614307.186	3305517.57	2923760.351
584342711.6	367804625.4	346034660.1	221709011.9	375519399.8
1396719036	1485760266	1137952697	1280601690	1446308961
78144.9567	55604.46688	102305.9347	59065.09559	61700.96608
130162963.1	120656556.6	102414464.4	72734011.58	105365217.6
40618035.53	23581975.64	19266347.9	21232546.95	21421994.43
91310.57434	135110.7887		76271.34527	
599309288.8	642342360.2	742771943.4	825436816.4	679955836.7
4678834.612	5973067.183	2805358.603	3783639.734	3976942.341
309434691.2	241100625.9	359831928.3	252189296.4	298841549
13032105.11	20549734.82	17945031.99	11202582.17	8288142.325
14730359.66	10039607.55	13337265.53	9906323.373	6318515.27
10061919.77	11831989.4	10752543.94	3270033.82	10534052.92
1745718.507	673372.8664	822298.2695	442973.5069	422173.7366
5837759.049	9616807.314	3099220.636	6626552.268	6839302.026
66637402.31	47347527.8	80110628.01	70344005.08	62953701.33
148132.1415	183153.9569	127657.523	59960.38319	64379.51057
190075107.7	154025175	178919272.5	149390622.5	114792803.5
5195440.113	2409802.039	2976725.049	3990122.532	2340965.513
145350470.9	151707262.6	127104758.2	151925686.9	154145551
847278.6033	769625.6051	363332.1915	567825.5612	652410.5244
1246581.402	1116117.006	1541005.069	1229367.883	749824.227
5586676.611	2887097.506	2962970.106	2216719.333	3526295.057
1182267.98	1343532.936	777977.811	943244.267	1023601.213
257343923.1	141948685.6	226862694.3	164001928.3	108238308.4
1998392.686	1361117.365	1651950.141	1002289.512	963274.5599
646543090.7	1173530985	860785021.4	714416033.5	452192124.8
12471146.19	8931941.648	8128397.888	9219431.979	10497251.24
9903988.836	6491963.654	13284175.92	12167783.91	4828150.582

1858316.457	1040603.694	1334633.082	806816.1792	66527.38362
2757695.21	1086502.946	2463870.287	982596.4902	1769694.483
35676253.29	33285860.72	41387819.37	36443917.49	39824513.22
2227140.972	1163108.652	1788780.736	1050899.462	
393055.7786	372949.5547	316138.616	378645.5461	
68454443.22	49386928.44	37443187.08	33718987.63	34889628.69
11441987.24	8839154.818	10706876.39	8001022.702	7859687.88
2417235.629	543353.1	526462.4869	432742.9629	339565.6512
169661.7301	163966.3178	151202.4596		
500021996.7	390193424.4	426269805.9	349319708.7	334212314.6
1100651.836	1335999.203	1717012.447	1007966.777	1436701.084
341120.0611	586913.4233	438047.5234		
5013842.133	2521754.653	3387274.593	2625228.245	4095153.317
420316588.6	168550969.6	663357807.8	245451886.8	1437793915
36099828.77	20957138.14	34854074.7	27445495.62	14769015.99
17758492.21	15857946.6	17819498.34	16264409.19	4260125.279
6273509.383	6508031.755	2659868.703	14899213.86	974382.777
732771.2994			739821.704	
3309193.091	2166597.999	4320450.362	1761146.272	156899.2824
639708.4703	660128.2333	1049380.378	322448.6952	2521686.338
2477222.682	1660021.276	1664540.135	1740102.536	2788066.696
21528128.28	16393883.31	16496697.09	18981062.5	13939890.64
9022327.402	4080138.312	3236944.589	6842207.891	5789977.181
1418912.853	1807686.59	1614156.559	1110582.046	1948916.145
5759240.724	3482690.908	2923485.369	3459437.859	1695995.689
290903.3689	266601.0549	290023.6321	221483.9094	96805.05065
11868425416	13824407565	14343233581	14215781673	15541016942
414053.5012	636096.9339			16326.61124
2637760.664	1885030.159	3234683.184	1807824.461	1689235.142
375616.8667			16066.92462	
753176271.4	633384771.7	467065318.8	605792155.1	374740450.9
	371180.0969		245269.2931	261985.4079
458291.6302	710171.0805	593868.961	316024.2621	411399.2721
19826532.57	4155255.899	21271505.75	7613554.153	3265460.378
4937669.479	3094500.239	3965160.666	3292204.208	2500080.508
29408661.88	22427505.01	26104936.77	23725176.59	14749504.2
779898.0737	534814.0737	873566.7485	595915.5314	430822.2815
3649891.849	1269540.319	877156.9262	876639.5297	716966.5141
48970406.14	59231277.14	26778744.53	46305459.67	43021441.56
13220300.51	9118272.42	10602105.58	9465134.753	5867009.713
4670259.99	5937757.759	5279834.176	4245350.751	7217635.082
1105100820	1300488929	1225241394	1549272104	1606430910
7594775.968	6862635.751	6905737.947	5873330.632	2010880.844
2247604.926	488298.5282	1972877.521	453197.157	267023.789
425441.8142	805184.8161	1596167.13	1865625.931	1049490.7
161705728	106970471.7	106647063.8	116230549.9	100329335.9
392244.2557		305772.3434		263671.1731

815206066.8	1449356315	1096935667	1065461044	1094292562
2103954.685	2166630	2478149.732	2253146.527	1205700.046
3156234.857	4466203.973	2470123.088	4018552.285	5366763.659
2897006098	2702436776	1901828462	2249007928	2909489405
263434.2839	152246.4956	265635.9232	240265.9633	
2356216.763	3583118.721	2052229.245	4588455.205	1382425.837
4586741.209	4736981.919	4444541.633	3061970.904	1526066.312
2253353.16	1880069.76	1795115.612	6382622.313	1959781.344
1215854.067	1390623.517	1999948.263		
249836762.2	300813158	245342798.6	190293336	386967598.7
23102378.64	33890768.85	13363513.42	13702606.86	14542837.55
622589.3416	2378208.658	3195196.773	1867490.643	261943.4671
917461.3351	884008.9191	572590.7785	1237833.549	452002.2892
90646316.51	54503729.93	65919595.33	85046510.25	69200758.89
811344.9482	1555153.18			
879921.7463	796777.5919	921373.0405	825358.476	42921.96096
326505213.9	176356425.3	202893890.3	178299766.7	141323686
1532904.018	1294494.339	1326633.141		
16176296.92	10792057.33	15305840.95	10402476.18	11306366.66
7621641.133	5539097.374	2368585.917	1957688.757	4800278.708
18628633.73	13114774.7	14317862.93	13521967.76	12676039.88
3569841.122	28120138.57	5689825.394	27244183.24	843357.0901
1173473.7			1596267.003	1862588.865
4273904.016	2681519.233	3155928.056	7305670.307	2013770.799
7455452.356	6140792.831	5880110.543	5598168.302	2016730.088
483080.7601	398673.6521	357045.827	288238.3706	323744.1388
1206999.481	306165.0541	825967.2193	905479.618	
				1220565.681
76197878.94	104855828.9	102471278.1	67956905.74	72866263.14
371389.9355	253976.3098	347421.2663	147221.5038	76044.41502
8604375.1	10737753.6	11565839.19	6810015.817	5923923.826
63045351.11	36044730.6	55118839.57	46468275.34	45715914.03
95267319.51	97996012.46	74470425.31	54908984.71	71339114.97
56420662.85	17143750.78	82461577.56	12155738.9	31046761.14
31037018.2		736207.902	366568.5987	312346.2777
	106933.2851	68656.86513	51306.11416	40510.06564
1928436.862		1313551.686	1536786.393	
551621.3729		316581.1835		
1222811.183	1330717.304	1410155.396	1129194.29	1731265.221
13754655.7	12468570.87	12058853.99	14064122.96	12469237.15
	492468.5593	390777.8548	288263.4286	
549905036.9	477066581.6	479135454.2	452791956.9	527025334.9
38577620	23347717.68	18182176.64	20559334.01	27986481.95
359911.4988	133058.2768	247116.5135	214447.1103	
6874265.926	3008301.057	5738625.519	2551363.271	1614081.552
888380.4512	80948.39536	692680.2808	54733.1546	92988.75259
46451551.5	35360931.3	46579836.41	31968000.42	17960294.99
8946317.075	15015226.96	18010270.49	17850631.39	8792922.049
6091429.572	6374059.299	6082158.375	4159321.338	6196497.841
2055668.72	753287.3231	1146659.288	2508518.907	1824892.249

1397172.846	797525.021	1205321.983	1200140.76	617689.3309
1347428147	1406251541	1756748808	1265320248	1623170236
71493438.27	24342471.88	95034080.13	15391409.74	50323675.95
5157949.666	7535226.308	8007281.65	9995136.479	6363528.716
11969927.61	12450163.71	8276131.36	10189820.01	10693426.19
11907574.55	8114681.499	10191905.2	7763811.506	5212403.29
1062014.766	243038.8359	1457062.349	825608.3576	
257282.7418	201519.0337	219742.0131	244678.8529	
4908734.47	2993947.281	1966753.501	3896182.9	7004739.976
2317272.29	2051381.765	2276929.213		
763970414.3	564650873.2	713103505.7	640814271.3	755875954.4
18276869.82	13758964.38	10913333.68	12506417.78	10490056.08
17992049.38	19315131.57	18474567.76	14610495.82	6602515.856
1874168.058	835920.7541	730701.3981	662725.0665	740420.7032
	361830.9532	470551.6067	192560.874	
4561845.521	2116668.798	1390000.089	1748136.032	7335595.004
5400809.798	2045964.691	1518691.277	2840697.394	2455241.078
25533622.38	17308298.59	13555885.2	20664110.72	15666884.28
18046730.55	9566118.8	12190695.81	10980047.3	8613778.332
12635075.69	10128219.02	15100237.5	9644264.617	7226507.22
268600.2459	212001.1861		336919.6866	143496.5585
18684720.5	13385082.64	12974898.59	12052750.33	5279503.318
15690478.5	11796001.71	15484464.98	13867528.39	20223129.71
4214809.155	3076870.784	5184262.55	1956666.783	3457814.298
141590.0167	65845.18514	77560.61173	76123.74484	
573448.0185	4757952.447	1201636.754	2061216.467	3016655.915
1156199261	705358660.9	776842827.8	800465289.6	660613357
903671.8409	544655.6803	807047.5119		641830.1234
183227613	112591380.5	126545171.1	104363091.8	77857350.66
384292889.1	401732621.9	180749252.6	347422761	277778258.7
23781653.68	18289225.06	26111212.59	20367943.85	14487392.23
2196245.959	1368041.219	1597060.526	2123338.319	1535621.287
1300860.969	1804650.597	2160270.967	786258.4404	2352329.513
60780244.61	47560235.82	80585210.76	63954985.2	30377778.59
1262141.508	588237.647	1024364.706	3373573.609	4392809.23
3195289.716	5016890.102	5394034.579	1014218.725	4486581.77
		3080990.603		
318088.8652	145022.398		82560.12092	576907.8281
146962676.8	136423062.5	130205497.7	97570048.94	79765230.98
30740659.94	17681123.39	38932825.28	49191733.32	27423749.82
372886862.2	286557706.9	216360208.6	308904219.6	529208901.5
4766877.28	4611204.67	6003667.377	5240794.263	2783957.825
1723044.274	1969564.274	1974342.675	1251796.413	921222.0506
2997159.825	4812860.713	2891268.84	1708644.774	1536715.814
			345710.7997	
11892906.52	15940509.48	13232253.12	12830344.21	15060177.51
1388694.835		1771511.55		

2185701.553	1669522.001	999875.6944	2683137.845	813153.9907
		84692.11599	174744.8814	
207596.8095	638119.7152	349002.4016	510749.6028	444035.5821
11274696.38	8166120.978	7974240.748	7354714.979	8513552.34
			819924.0981	
8165620.882	13114475.09	19609061.17	63203538.94	9777967.14
	3617.82228			
766781.3475	661743.4728	1009586.916	506732.1655	436930.9096
2416180.635	2053617.192	1217512.963	3461681.324	882456.796
153573.7172	117461.8645	130718.0634	84924.39886	88582.16618
6917206.778	4331411.274	7249277.426	4359157.409	4724669.207
3350537.276	3952253.292	3866252.918	4190473.061	1864350.378
4424747.528	5260531.434	4345015.318	3322053.892	3719344.825
510287.9508	638766.9666	572110.4807	370075.7663	453928.3405
242931411.3	423253140.4	206795525.1	116994678.1	305820464.8
2261040.264	1820457.993	1803735.261	1536581.47	1202951.196
33766051.23	25748384.53	27755812.66	29436585.76	12334852.86
1183037.378	1293293.675	1267970.316	2015671.905	978728.2259
7459948.125	14179643.68	12275750.65	3689295.907	14235873.65
8554244.163	7732140.84	6927879.981	5496509.001	5561002.174
2902621.331	2082316.493	1779380.464	1952369.787	754751.9201
6309774.589	3445487.162	717156.1759	1114662.902	3891697.307
329526.0324	515649.4886	535484.3871		559494.5366
1256690.247	925976.4483	857809.721	1263008.137	667231.2983
2670668.554	2977113.025	3470851.918	2613774.598	2632145.148
34992813.41	33201436.81	27093217.31	28554625.19	48759598.6
	182839.7808	220343.6132	335779.0068	5147597.34
148435405.5	83125801.59	85318252.05	219823009.6	77646322.31
713046.6866	507822.3731	284936.3191	1052965.365	634393.5077
		2193220.464	2586458.237	
990880.6034	262978.5419	1042177.957	431048.6758	74928.78293
		33170.20202		17156.12981
2970100.164	1791595.845	2728843.754	1283491.833	1508807.706
460386.3758	347358.4865	471582.0814	207661.7373	286203.265
17178413.88	16438369.31	22311037.12	7795559.243	12857593.86
4333906.264	2905175.867	2724663.809	3971217.071	2785233.713
7037553.574	4548645.642	1733171.968	3831644.617	2126171.212
2397455.637	3697960.758	2835599.057	3371273.61	1957301.946
899776.8136	662886.366	648172.0083	714854.8059	384907.8517
220725.0986	220968.2478	263931.8855		235429.4549
2720501.644	2293968.815	2050756.575	1314740.618	1802246.589
608311.5654	324693.8665		435121.1871	
1792153.44	336134.0646	5320027.936		
23914354.96	17608885.05	16523061.63	17516994.72	54398234.17
49999743.89	25505402.26	40026447.47	41964075.19	23473161.33
11857513195	9821265209	10870490998	10597453679	9678939515
4971494.112	1799575.43	1779424.78	1253731.466	3941820.579
15537205.52	119425914.6	18919449.19	13860981.58	23810003.64
2424529803	3121633985	3398756278	2902724969	2964425180

4242905.37	3998806.791	3349312.443	4658081.562	3072486.358
1349720.023	1411082.675	1104497.713	988353.618	889062.2639
	1191246.245			
4145191.444	1820334.433	2407094.122	3172398.556	2662224.091
24138985.14	13531842.22	20564380.19	15060831.28	7579650.753
807893.9217	688117.8458	780193.6255		
78026395.24	51599400.94	65728584.3	47052246.63	30854077.1
340171.4564				161309.7297
8372549.441	6011739.342	4947540.337	6301707.991	6890052.886
103651077.5	85750678.21	85500026.5	97885243.38	84749205.54
4279243.968	6172353.051	8500860.203	7684244.498	8195100.999
40831215.35	26407653.14	30903692	21934962.64	24365527.17
14318967.79	9087540.966	12137008.69	9038876.087	10242883.64
425114.5578	378180.7465	226353.4567	351365.1971	401983.7178
4635773.744	2941520.199	2742847.627	3444659.161	3606522.604
3000232839	2762407110	2712569584	2961930069	3024429802
120471209.5	94471000.6	81003933.89	67246072.09	115312025.6
336973.7357	478627.4593			
3831827.124	2917748.881	1913738.212	3405556.217	2768556.383
1453095.109	1375858.943	751617.9154		1033512.281
55484567.94	2556861.557	3525385.849	4843036.566	2598089.077
1655816.051	545249.9249	1090233.579	935956.7913	225599.8316
40349116.21	26624913.59	32495303.03	29583982.62	21155933.47
1921765.228	1866476.418	1416325.612	1018883.176	735068.5466
1616528.018	1350206.191	1179653.083	858486.1469	1096860.462
827537.2596	2914627.121	1824885.779	611489.3465	2019656.734
70458245.97	58168850.2	42106189.18	54186363.65	45131814.52
3632037.528	1640813.675	3558535.172	1214157.276	1200338.308
17607017.62	13885183.99	4223314.15	5371059.051	18404999.44
753310.8929	618383.0893	559572.0959	475989.2692	493985.4645
11387.41254	6728.150846		6764.102936	2190.01553
5176912.986	6153022.894	5157977.361	5961036.744	2901758.798
623922.2178	258713.7584	253619.3502	226069.1898	269028.7861
1110470.865	551704.0226	319681.8727	262729.3805	375740.5381
3885755.863	3104107.093	3389821.645	3188521.641	3403537.438
15596696.32	9328420.513	11873049.32	10000428.73	7299294.671
866855.4637	562042.5754		1262369.579	
285789.4585		159178.3129	184996.3567	182308.4478
21389047.9	26579130.98	23080938.84	18203719.89	21377726.28
396982.4459	406699.6929	380181.6845	398155.5519	198245.3555
97114280.36	64112553.1	65772710.15	110430979.7	80135317.11
378825.2208	384921.5603	361497.8096	436940.3945	328625.5018
465835.1132	141121.4736	258556.4376	175389.4851	149144.5299
55952447.79	30520492.29	37422353.25	3379956.756	12931468.25
	485638.1963	418217.6018	1918636.181	914434.3795
	692787.316		71401.99151	727194.6197

4314741.913	2206826.139	3497948.893	2805633.75	1360081.018
10026953.16	10660115.39	8269525.928	15083489.59	7074244.79
72556142.77	97565182.81	69937833.81	78963113.23	34663727.91
9505047.384	10138680.02	7222619.186	8135557.861	10325461.89
644117.8426	833281.7177	428880.9721	500837.8717	874665.1617
61487768.78	23188098.48	43498150.7	46489089.1	32917220.03
79254143.67	39567019.08	53292016.99	44908525.18	42754712.51
	46275.22732	64698.98964	108562.4319	52777.69198
		150971.2502	111570.3311	
450422.6801	2612667.558	5174289.18	1415829.118	3764596.085
311577.8133	271026.2453		231893.1891	
918220.2858			745337.475	
111698019.3	55255900.35	60732769.47	46291252.58	39105534.14
649201.0611			413072.3231	
1185838.196	675415.7644	617347.2372		523515.6839
980004.8879	539751.3028	862530.4664	786512.0187	576102.8015
774755.6162	417686.8885	378574.2445	370330.3586	201584.4405
28438135.79	26479266.65	31923846.35	30797984.43	23000089.82
286422582.8	517213654.8	265991532.4	313255707.6	308289728.9
10201096.04	12847831.02	2891544.71	8057751.258	12037142.64
5560347.755	5248303.941	4571644.477	5392391.454	6556825.937
106051216.7	127067917.2	160338190.9	154687922.4	231981215.4
208853.2255		1276767.546	449678.844	
	351732.6799	473570.4354		
468679036.8	305891397.5	301096809.2	363188792.1	288619677.5
85330168.11	63416143.34	77789214.76	55908714.38	40455830.42
1655746.432	1040317.983	1015070.843	1021972.35	383755.4027
2590276.963	1623693.264	1963463.081	1612418.613	1730920.665
2240504.24	1313635.02	1364499.542	943695.2593	1214905.252
15997976.64	13650590.26	22056031.84	19228006.26	9178283.189
3566045.514	3177162.515	5395127.156	4953352.87	3693307.82
12859283423	15593716231	11624788114	13070015749	13747949562
9227922.862	8715709.714	4940479.256		4236136.833
5334108.878	6355583.204	6585259.46	4973807.03	6033604.6
17196193.83	11582588.18	18362160.46	9322885.675	4875079.438
1758588.805	1483290.904	448445.8146	377642.8201	241973.624
2306797.261	3366169.272	2378345.873	2634893.162	3760232.004
3999629.661	11011705.04	12040705.26	10433209.14	4677452.386
228204.9252				162409.4525
50226319.35	59209419.52	87285540.21	78975885.04	36723619.26
2517313.817	347332.0121	1457650.702		
10987356.8	2540522.624	727031.0436	6986142.312	1485868.944
1396321.15	1087326.049	1466694.388	937749.634	893915.1766
122162277.6	59329650.42	54544239.26	116513090.3	121269165.7
305315.6243	669081.6906	367597.9043	289964.2637	
7088387.585	5229749.697	5519517.725	5113948.07	3207482.213
190436394	113012695.1	113975903	103543595.3	157247392.2

13017657.2	10811574.62	5360507.277	8626714.04	8619029.046
1338379.152	607526.488	968361.2672	451843.33	439377.2704
1539992158	1565650906	1259209482	1385347859	1530043623
9058796.836	6933842.427	7787549.795	7148676.77	6925103.81
		33278.26019		180283.2641
282176884.3	434056077.3	287176272.8	361689254.9	231494587.1
				435860.1565
		640041.4015	585939.1182	1064682.975
		3474.643384		
1284036.676	1765877.12	1880349.719	1437542.148	1906164.222
12585869.11	7585710.408	7069665.322	6734176.579	5433619.817
30833416.72	28216090.55	32547524.96	27691437.49	27156929.72
1675045915	1122970876	1256607252	877885693	674899382.3
950737.0455		2207334.701	1487198.951	
2746195.507	2203761.792	3371734.496	5271172.818	2339642.362
14713206.46	20043938.88	19680806.38	21921966.08	23254318.78
287331.5825		177314.8388		
2023791.824	2153221.96	2268267.629	3025874.23	859499.617
67131090.48	38101500	47646897.78	45091040.82	38172745.93
137881499.7	112473656.8	138558630.5	108061639.4	147543065.9
5231912.354	2190290.831	6859988.13	3070413.164	8564854.575
3587314.872	1491344.211	3671808.586	2970973.865	1720333.384
571405.3619	820622.7104		966348.6441	857145.1103
2437604.549	2033631.03	1864708.599	2027146.133	2595150.221
				53334.8317
			232700.3699	
309698231.3	195688769.1	553883672	257050043.7	205578135.8
898563.6376	551671.8281	2097497.052	1144764.109	1430135.891
8685142.987	4139903.669	2650999.417	5302664.151	3023590.244
10810170.24	10226529.48	7749033.394	11839657.14	5302930.069
		318539.3608		796222.8839
1513994.237	1123037.15	912696.2847	658734.8202	658661.4036
11695963.93	10489574.67	9594273.92	11012901.5	12659101.48
2494035.422	1794767.8	1646256.26	1070744.193	2333042.789
3609043.028	4703615.795	2651199.9	2053950.398	4062393.908
22308840.15	12903316.32	20867815.22	12886423.02	10326473.98
1912035.972	2971945.526	2391788.109	1850120.065	2043834.12
4084934.856	5618438.472	286270.3288	4672672.041	4098826.245
986407.0774	832233.5043	4650297.763	499702.7935	1873214.301
31962053.16	23182852.18	25774027.09	24340522.3	29583025.54
2879020.605	9972394.687	6783562.22	6249143.714	5561615.037
8586339.685	6000267.554	6677067.166	6885113.087	7862573.739
3905364.921	2826431.226	1007627.324	3897127.036	2710103.7
8788681.416	7041483.138	6764632.62	6718027.439	5240416.166
1882491.14	1017185.906	2630671.002		
1119185.358	968418.0822	531671.5488	802120.1925	1183555.096
912633.802	1008070.046	932905.5367	1679246.846	1996521.186

2380187.455	1755156.253	671350.8053	109825.2025	
134286983.7	102859954.4	129827083.6	82431699.82	68006733.9
659505.5093	147403.7049	375649.9995	284303.4865	275598.3015
			2434958.435	
1361208.053	1094019.596	783291.0217	339391.8641	650580.6627
1505887038	1606697139	1305837548	1477073317	1413702701
7145173.251	5592042.465	5457231.247	4714627.996	4689079.215
1143624.068				1065523.51
9502511.337	6702178.647	6751149.235	5958895.452	4399129.73
2208908.795	578394.6877	449220.1032	1392714.076	1094490.136
169910756.9	92517379.1	153517818.8	128039131.2	59314592.16
6800627.111	5271362.894	3233583.574	4952535.446	7618991.605
647791.3528	275592.446	218626.4965	407586.299	
8282605.363	5101329.218	3493425.394	6709652.021	3933058.125
1424136.546	751571.9797	973776.3943	310970.9314	56372.57367
73061741.42	78406761.07	59191144.89	115530051.6	90497773.24
721204.5979	442740.6225	745912.9092	394114.7091	
450588.5019	728079.5411	10535882.89		
11819145.64	7261596.641	6352797.071	6896529.731	6137827.947
3935936.175	787585.0242	3337309.854	699827.9829	68363.17762
		647210.8918	390375.3317	
1629275.698	328809112.3	2711504.958	320258130.1	409392.9582
4744450.069	2890311.54	2804446.826	2759050.47	2218080.62
186701.7774	155368.2748	85463.51155	117054.0686	49823.60763
134921611.7	171088187.6	206910816.7	176418220.2	121118843.9
635770.0914				
200100752.9	193167436.4	139980889.9	154683674.9	285099913
18893750.35	15041077.44	31785397.16	14963320.53	21088207.15
30927273.33	21143261.13	36160455.41	27277697.74	17534498.3
164989858	95954061.11	94396737.91	87812688.22	70618123.1
5194640.599	6740275.074	3723399.782	3498425.51	5487779.902
1207072.019	1594785.665	1238233.192	1029910.874	1109940.111
1016956.748				
27294436.03	28322914.04	26230166	22011810.45	21653808.72
11949195.89	6594948.443	7961462.131	5277772.648	2822737.125
9547641.899	9471436.719	10408185.13	9119248.533	10090088.88
5838083.852	6920911.139	3248293.141	4461778.438	5948819.849
4919110.378	2570592.701	1845364.668	2962700.17	3802580.822
340195.0283	261765.7427	264486.3787	309974.1817	569630.0535
243260.465	173687.0503	180854.8956	186008.5563	195977.4536
1506623.124	567846.1559	347738.4757	2261455.734	
21807769.88	10165461.65	6625860.029	11927045.59	9874639.13
489686954	379321234.8	522112759.3	449598262.6	405993009.5
73028.09983	115280.3622	87902.99128	65936.14447	71120.87392
131656714.2	88525845.01	87019243.09	91624257.49	64354168.32
41439698.82	54950869.75	54132564.78	60177399.53	49942178.79
1423559.72	1920077.892	920766.7613	2363118.321	629415.2922
4095249.12	4869969.604	3851704.04	6392281.08	4542268.994

206903.0163	210267.7103	242532.382	203331.1895	165570.1862
6158855.944	6715851.751	160977.9971		6751008.859
106172420.9	75590643.85	57412852.88	82058455.13	81208905.83
63672308.33	72827862.88	57573024.58	75453241.64	52216962.29
20955042.95	13991778.18	24595784.55	23490914.57	18404188.47
9655792.329	12812068.52	9284318.201	6695282.607	6079550.606
28526442.36	23868986.54	9094567.973	29744633.3	25511350.84
	2118580.735	7312309.034	1492593.929	757949.8827
1164842.193		1839249.02		
10955083.57	10019007.24	6025504.741	7735530.661	7316948.141
37098253.22	24666043.13	30724008.14	51296775.92	35434727.27
193545.2908	271886.3936	762215.5741		859497.2879
	693663.4842		441369.8295	
46435.44428	58377.46483	58269.91347	49508.45296	67433.09903
98849377.84	86019074.41	77757878.9	99658866.25	76138932.53
543465.0232	876741.6701	353177.8928	567205.9122	882933.4268
956931.1212	1823999.15	460653.5203	1162979.627	
297009.8862				
6496163.182	5143191.625	5906571.202	6276995.474	4687207.403
14799029.94	12029928.12	21863425.24	13334341	19456334.35
34253472.35	28528689.13	29703807.27	33579476.8	28559969.32
8387.087982	10823.41529	4928.500755		
1684085.152		1811385.105	1336968.04	125201.8476
6268125.25	3401050.409	6146262.485	5343947.248	702410.6246
1699074.549		2159925.274	1702629.552	956051.0701
10730385.68	16711932.21	14167657.05	16158038.65	12098863.14
5296129.629	3085138.187	1043017.957	3388720.438	2067994.449
21507932.86	51250125.51	23864199.62	19704863.34	57613772
6314415.554	2156080.463	2601798.808	1610165.329	2119663.779
794115349.5	784003058.7	594225929.4	431645894.5	477373751.1
57325529.76	68966768.3	48612135.97	77607217.28	61425158.69
1547577.238	335712.6381	581882.8741	483973.7972	131258.4394
505538.0862	292619.0183	263000.6481	144761.8073	
1629986.501	1032125.598	1434890.063	3965684.851	399950.6736
304502.0937	536012.9207	501336.7685		
1750322.85	1922137.409	2258833.133	1130135.198	
708917.7985		337473.9892	202747.9555	311645.1267
9672210.359	6485330.999	7587052.587	8703198.235	5774470.53
1800585.589	1112200.26	1446395.778	2288604.451	1606897.764
63068913.77	63819113.12	56847313.2	48397494.78	45259390.33
183387871	127096747.6	155848395	121343247.7	64573306.34
2623536.501	2402880.967	6251042.439	3322655.131	6589453.182
213820.0155	197888.1919			102581.9416
55852260.05	76608007.59	3360100.334	45940866.74	3005707.309
139623.7087	176166.5058	197223.8488	190473.7143	140696.3615
10453614.1	6567717.168	6583618.381	7980853.99	7200704.333
324955.2627	224132.56	330565.1873	219615.931	378560.5857

1168413.933	1032911.328	1419109.482	1156509.786	691875.6401
	428372.8554	433290.5361	502390.9334	478366.4719
6077103.388	4419593.525	5056518.432	9503698.877	8782461.704
14547041	6838402.898		12493558.99	10846474.34
3513408.846	2592465.987	3661265.365	2405418.21	2678256.293
2104278.446	1349443.226	1717935.722	1365886.348	919601.0687
375685.9839	262977.0346	318447.0445	387706.3408	
1577044.29	4559295.008	1258413.29	5973357.008	2830887.648
61950713.88	50262543.63	58976130.81	59917367.68	69201946.37
	221293.3615			
			888211.9242	
2117641.08	1429545.29	1313271.426	2074016.062	2234230.17
				2387892.899
623007.6326	1067658.489	403949.9886	362238.7427	216319.2546
9570148.203	8691339.677	5312000.622	6438373.6	8565638.085
1258204.393	709184.7632	832283.2954	1436406.494	383509.491
890970.0409			784188.9982	
16917980.4	18083172.93	18393003.69	14220267.8	15039843.83
5916521.2	7513165.498	6009086.578	4095464.158	5156979.786
163105.9881	224438.9676	131453.5333	197241.6005	66257.47603
	8443880.675		396674.5898	2404540.507
589132.3984	230384.4665	50944.43707	249340.9685	466500.8393
57539515.59	29990095.23	45269581.17	57550874.68	35136670.71
520802.9109				
3683978991	2985494329	3988952942	3547733779	2016970366
10053654.41	11855712.9	5198346.144	7755688.756	11468232.12
2709348.159	1597557.093	3227699.629	2290235.185	278712.1496
155430.8562	89228.20417	133428.6408	99314.7298	80817.14575
11964262.2	7703578.993	8013991.55	8520779.918	6747428.323
		428736.0046		
437585.7317				
				288822.9966
674111.2854				531712.5726
356323.1116	406778.691	748754.2867	920921.5323	
1477063.049	5564650.054	5350415.925	5987077.267	4492843.399
7922113.339	5974457.458	6133425.886	4323012.137	2692687.793
998684.3773	818547.2508	943133.1302	646283.6546	362650.0321
18709860.46	7042759.758	12533305.72	16168862.25	5363714.72
206643.8919				
316528.8763	182166.113	245683.9364	268517.1007	151345.4515
4797550.071	648889.7819		1473612.409	721287.3647
299379.13	208614.2955	253275.4361	340381.6574	
639873.6972	471997.3744	437067.579	1353720.506	696228.8106
4400123.03	3465772.823	1876417.82	1276518.188	3400144.659
1441393.444		573742.7416		877535.481

7969349.094	6275095.809	2537520.242	5286769.551	7900470.993
78254960.31	64912792.55	57352895.87	56408891.42	50878467.5
7385734.762	4820971.096	8217209.982	7742513.827	5386562.644
258679926.9	206231189.3	188044888.8	255232317.3	301414162.8
4554217.236	3970209.6	3244189.754	3061720.477	4200707.205
57219141.83	41585228.52	41379089.66	42142100.31	30839128.52
7086991.424	5704921.8	3514252.562	7436514.288	8203925.674
28465823.27	32519819.34	21677712.26	36619083.07	21670039.59
2652737.196	1600182.119	1615558.838	1336451.967	2627040.447
30385480.07	12474179.43	19053521.8	21625978.41	17299579.4
1998186.635	99905.96784	1918329.516	1470516.24	1499792.138
9531558.127	9575667.062	10235007.8	3114015.88	6105985.735
477803.7606	200274.0014	448593.0147	274778.5603	
103776654	61773822.25	70964827.13	111435312.5	93079999.44
2422608.831	2504007.087	2566690.929	2188701.5	1739190.865
677650.2049		697647.7427	786005.4001	844158.9994
			207813.0657	
50370799.74	42333944.72	30633309.09	18665969.46	32439658
				269642.1365
579759.5452	370966.1758	621353.2103	668380.4824	452053.3132
3902861.533	4265455.79	6569259.009	4964552.477	6025046.574
5049162.889	5106048.362	3127157.499	3198821.397	2728544.783
6114077.444	4808599.761	6648328.984	4418030.418	4610108.405
102237234.6	60529848.37	71825777.52	73684556.89	61989250.32
7583465.807	8077217.579	6016087.566	3683033.909	7740612.934
84641252.14	75107831.77	44378800.8	89109738.34	58055838.71
14187682.78	13973016.87	18658267.46	14288856.42	12151748.03
			12540.45302	
2048775.441	834588.1822	1388792.339	1258322.414	332322.8123
				707045.3413
5116589.847	3555323.841	4581667.828	2553806.374	2776928.04
3967518.598	5504203.105	4315431.235	5466034.985	6825340.259
13812.27845	14522.29345	8561.673252	47619.90824	4071.107707
6462047.915	4041974.23	11382008.44	7984842.641	5376350.506
8900849.095	5391667.127	6706437.688	4389516.536	4886936.874
57381031.98	35868923.69	64738814.86	44971540.11	28927407.01
1391211.906	1009958.349	971344.8587	990559.7266	597788.356
331750.1636	201007.3043	243738.2986	346697.5953	253803.7476
1762567474	1853798450	1391929947	972940248.4	3021832429
14677922.73	15187172.52	19962345.28	24803600.7	10815620.68
553836.804	585372.3026	657327.1364	403102.8278	243780.3833
1557024.916	2040602.608	2884791.536	1858582.258	2324814.53
471898.3859				3470991.051
	998137.3056	1175417.695		1163580.46
341031.1271	301808.9097	536455.9966	312305.9265	

660684.4055		366910.239		282568.2296
205518.2371	28230.75921	146228.2908		
1003988.416	543423.6533	2080104.899	677551.156	44812.0509
21565063.6	38203368.36	28142275.24	27212775.27	21769802.55
3004394.339	3200578.043	4342709.047	6616158.438	974616.0939
1024558.153	1125157.394	1313709.9	1081203.503	686254.0653
2718046.943	2485246.054	4649716.238	3355063.87	1764920.472
369591.364	241876.8018			
			174858.3729	
1041039.871	1034856.773	1375748.429	645877.9599	968556.5908
2504054.989	4705674.539	1177115.035	1325873.468	3906209.285
525082.972	315322.3451	417037.9177	327887.2551	626417.2695
4081556.739	522784.2495	453601.1649	382283.9995	184211.4774
1423836.747	331247.5428	1237298.94	931134.8441	
1974517.323	1731228.127	1936365.176	2830758.029	1581568.18
21052423.33	15980251.87	16379359.44	13774847.11	12820988.91
8275504.119	7947517.602	8143908.348	7477613.619	8102892.827
431959.6142	181031.1911	328679.9403	216732.0634	414485.3329
83894268.45	90801570.61	102930074.4	84883367.62	92417498.54
2504314.95	1574678.122	1463581.039	1786298.271	1614148.289
5577584.632	2848807.835	5607317.744	1450463.585	743059.5956
11585193.41	13948568.36	5940262.132	6969946.465	5006898.99
100035584.6	113345063.7	90872874.6	88770795.87	83977481.03
940973.1167	956409.5922		872659.862	433940.9131
990701.3969	536908.8384	956683.5338	638357.9285	815292.3079
	820239.1405	877217.6518	536577.9294	863861.4588
409541.2897	174876.7762	251989.352	209566.9954	151809.3442
2154121.5	303571.0822	1789677.992	1615440.569	1422255.144
10336558.25	6420955.748	12798684.18	8689724.397	6450329.089
863046.2473	13129055.27	368695.1325	432846.7453	
3778992.065	2959649.373	2991034.56	2847319.793	3537508.923
2008462.901	982886.1043		1291330.394	
1154165.501	197800.0726		993309.2722	2604734.398
6097773.445	4648702.582	5095007.149	3388388.938	3809321.124
963871.3577	1796204.25	2731622.322	1526972.703	358509.8799
19012327.94	13073806.88	7297240.751	14940078.53	14082754
170000.3588	114340.107	222232.8324	84191.26084	75866.80716
1318883.269	768997.9487	906294.4969	1387263.717	714659.2513
355934.0439		284376.7097	110432.7067	199621.3931
1645212.361	2341679.208	1719718.569	2368242.81	2538576.167
270171.1471	416678.6187	394134.3952	155294.0905	290173.755
236986.0996	129970.3829	286525.9382		
4179866.266	4770873.995	4224737.815	7586769.489	6734249.761
1248024.832	1341829.529	946570.1171	1478700.733	760877.9623

2702458.401	1845941.057	1505290.883	1650155.718	1684291.002
676407.7313	466805.4497	647373.1802	299498.8845	432390.8777
1537289.744		1129300.771	1548544.619	
30207066.97	19102838.16	19572952.14	38691050.76	20937428.28
		835182.366	508369.5483	294725.1635
339143294.3	363913400.2	268890582.2	406095628.5	378120136.1
698915.7706		440196.8449	567466.2163	
		109048.0203	45846.50761	96770.84051
1178624.883	1533293.692	1404191.674	1511858.388	1427058
		6344554.948	5390473.974	2264108.367
697021.0894	1015393.065	728090.3928	327708.5819	183935.0552
35411.86021	22759.75646	22126.65484	35440.48273	
3408598.993	5533835.987	3001515.679	4473008.878	2231981.349
6927877.707	25549691.88	11318711.91	14095517.32	8515582.786
2128782.221	1651014.368	764006.2723	1358939.5	1932076.059
318631.7642	234161.3041	312078.6893	201702.3195	88116.67003
1422634.874	268142.7746	1313630.57	942261.8527	964721.2214
506453.0804	1196063.045	651465.4046	1562177.625	690653.0688
10184753.19	193817185.1	406628515	14941371.13	18777203.7
763835.1479	530427.6673	265391.5882		463991.5136
1420902.482	1715036.738	1509898.223	1257706.095	1783236.332
2715328.897	2031959.74	2482327.222	479605.816	1849130.127
16128942.46	15411603.81	20326909.16	20808509.19	13883717.33
14816337.2	13219472.96	16025623.3	8431486.27	11356641.36
2587141.073	953689.3354	1956540.363	2401160.031	1720501.864
56274728.38	48445712.21	91467164.2	44205912.21	36038614.21
303253223.4	339416167.7	378943914.2	306608477.1	212376561.2
229646.9055	210865.7811		81993.67232	
2187314.858	3666082.458	657589.8341		819746.3442
1268051.456	824850.8694	802268.6454	1156872.917	730163.6189
392727.221	330673.6082			
426299.031	125612.6829	307324.4554	191539.8925	
1328494.686	769675.0756	3422090.23	1856451.914	680296.8298
1623802.687	768241.6691		1468474.738	1624321.052
2045518.586	6271897.334	1856341.21	6228387.492	1429529.55
1382086755	2189370554	4011394966	2350893696	1552389893
133177.2102	81488795.57	17805783.58		13771923.89
32222997.02	21830100.26	26857538.61	19589775.71	12295412.5
2357920.787	1373731.095			
554474.8833		1053241.365		
2219175.097	1278640.989	1078110.283	1248036.369	
2702122.53	2339174.118	1956997.675	2502676.368	1510851.145
12661529.62	7023236.725	6056216.117	8717339.571	8806194.08
3502171.78	2836816.099	3321111.617	2325537.466	1556506.142

3808958.85	2895565.225	2039158.283	1812059.177	3420994.121
3587483.37	3077348.522	4171847.978	3854841.597	4755586.572
4970288.483	3640208.404	4786302.544	3418129.262	2940625.653
263934.8351	188129.197	190046.6934	323187.6005	
918072.7565		1259216.714	1281887.733	1203631.995
		312954.95	141514.4345	
848486.8715				813725.7183
93895.34	73147.24706	85381.77199	70745.54816	
72790859.35	42264928.06	43497727.37	36429764.28	27473514.2
587868.342	1112141.745	848440.6394	546199.1485	693873.6053
24742709.21	36904798.37	23535664.01	34775046.47	29480894.01
29524.74303	17042.47755	57653.03424	18706.13503	7454.246434
2720670.292	2467576.269	1694248.943	1549681.148	2153112.783
437272.1947	498045.2327	393824.8138	323323.1523	147475.7287
3674923.503	1976225.848	2490096.924	1408148.342	1602510.21
1977152.625	533870.415	1596255.94	1717679.454	1205417.73
27570.48821	28615.89688			
12634675.62	8969933.183	8650306.494	8368051.752	4620953.995
469874.9883	326835.203	379357.8331	364004.1445	219444.5509
17125843.78	7751114.693	5103180.323	8986786.452	8438785.246
881784.109	614194.6807	297537.7365	319424.9906	
639194.1997	416784.4778	353437.9749	448296.0772	332774.4327
52222712.97	84350917.53	56130048.93	75295236.74	54152294.86
	7240175.063	5112763.731	2930830.698	2269922.514
2614541.375	373509.2666			
4955038.754	6814313.877	2375655.193	6381954.584	7449622.633
852442.3531	785889.4049	1359187.717	892216.1407	191407.9861
675459.2416	507733.2955	486662.8523	517109.7241	524314.9461
285585142.8	167272332	149065713.9	22136443.44	77633764.41
5674960.426	3619368.432	2343933.659	1849274.547	2623884.408
2484490.696	1082975.374	1199829.321	3637276.469	1504726.505
6049471.381	2545795.711	3762837.932	4254972.854	2050799.856
567649.9074	461425.8852	399658.7502	441253.5324	297921.1553
38987039.03	24002366.75	22334323.59	27267250.09	18511048.07
740642.9265		400814.8808	239360.6832	341828.877
16111.17116	12412.33975	12368.73359	7875.565545	4540.790826
1450165.9	1606040.735	2784258.95	1682343.553	1698662.306
270743443.8	319965647.4	341838131.5	167967232.2	273663501.9
1507649.732	1213018.581	1024426.175	1060279.014	803215.2605
322198.7172	500905.5141	457748.9613		448336.1162
4234973.549	2610350.292	2327889.375	4254104.028	2302363.56
79932.80182	48513.95882	57046.89919	28719.39549	12198.52639
645067.3954	610960.2402	297056.341	223429.7524	256985.0664
44985138.92	31748670.31	25806640.75	24508137	21281662.98
36656563.64	43237818.93	46870921.73	41283581.65	35029671.94

18734081.55	12821657.32	20204845.7	10957422.43	6810267.544
86305070.57	74406807.65	55530585	102903999.5	71496562.44
4066811.26	5364912.691	5175843.487	2689665.681	2638604.19
877853.6121	532873.3244	409878.5856	173952.9006	214029.6747
			623116.8629	619477.207
56345.3315				
4032342.544	2956479.592	2152919.951	3871964.4	2271080.374
5769114.482	6548551.771	10376671.09	3128461.621	5672675.529
6571752.267	4013595.602	1941052.424	973551.9465	1642146.157
2445752.57	2893308.29		2415920.919	680006.0929
16795974.68	11089894.45	12816622.16	7438767.899	8339785.477
77550074.73	59203437.7	57110577.07	71540427.84	44970461.27
15237209.32	18800076.11	20877290.54	15176121.28	20105186.52
760747.7396	546679.9497	431657.0473	544102.1501	573909.0887
159406.7867	98869.94822	111766.5698	102995.2381	50488.34491
646793920.3	665518889.9	1978692256	1683469088	844717070.3
880665839.2	1197566783	1322475598	1724885274	1569904738
259745.437	197604.1619	250858.5052	215567.3248	137664.9617
91053017.98	54551940.47	65899919.2	85107755.21	69576172.43
2232723.331	957642.4115	690423.6291	396147.9478	
1879557.433	1757510.293	1627459.232	1820270.073	1652688.252
101181961	51918626	78586068.12	103471973.4	97055611.97
3411495.186	4501830.742	3946468.819	3016090.393	2854416.008
1218735.165	579185.5966	816406.8446		
2568976.821	4144339.005	2491245.773	4622745.662	4029530.462
14016.72769	11559.58064	8587.897096	7769.952739	
15366075.55	10643280.1	10011344.5	12462704.14	9911709.626
943921800.4	814328431.6	1140783695	897563814.5	840389477.7
2796984.897	3228680.201	3348246.936	1780248.508	1460453.508
14676234.02	17094600.18	11449917.49	21554373.82	17345822.64
7332704.267	4625769.391	5515720.714	3037636.337	5376484.659
258437137.4	119498179.6	285665294.7	347107282.3	252777686.1
432000.0286	769887.4507	901875.6678		956792.2091
24190016.02	36737006.91	24118396.46	50884194.27	16920884.13
1504463.686	641174.8252	1954112.158	1145922.89	496337.3017
22741172.97	8756453.951	7946076.648	19691774.2	10307672.22
534258475.5	678680392.4	526735645.7	444322012.7	422431791.4
14378298.93	9907358.796	8341599.657	7825040.915	5975512.857
	1658917.621			
488360.8455				580988.07
8949972.349	8357578.552	12993056.38	8998402.91	8141573.241
19722428.91	14144097.56	19598886.06	19331494.09	8496427.51
531717.4421			338222.7441	284420.8264
7419576.85		753094.8461		120091530.8
15538302.96	6363805.117	4227642.151	15321572.93	56072517
3939370.582	3287706.159	3280667.085	4400597.349	3867809.62
135930932.2	77103636.89	82745196.97	83853744	85787830.56

324622950	582366292.8	382845253.8	849880815.7	242290943.6
13106070.52	9207679.278	8581273.397	6879359.118	15300969.64
3930195301	3541578861	3972834568	3078633946	5025609606
6655704.002	3694257.044	7331956.258	3444580.466	2467050.553
44467941.85	76872983.93	35171760.63	27213386.46	55676105.55
1772330.226	701750.7447	1929748.207	1099814.483	392949.1241
33293875.09	18945145.24	24795949.25	20945140.92	16534656.9
808268.1733	609352.6436	720273.6279	856444.5683	85818.98318
867028.1266	715049.7373	928733.796	664371.7308	223810.7283
4704208.803	2656492.742	4772215.545	4020630.278	2808320.566
3688601.866	2792569.586	5690070.38	1863331.427	1903256.056
169365546.3	39330001.11	70462684.84	46635955.34	75364396.19
946403.5199	581549.357	823917.9164	1029094.565	578811.5206
10685563.46	5126887.59	6620020.4	6680549.659	4883169.311
1198132.868	2277105.843	209903.2383		943710.4639
29601639.22	22216003.72	26674800.94	17474088.26	13712072.82
18797011.55	12339692.85	14926197.94	19489160.52	14975889.18
1612668.758	1215725.813	836429.9942	442251.9347	1761810.583
7723901.987	1999967.581	1628230.842	1793845.657	2662284.037
481242.555	392456.6841	258984.5726	367147.9703	
2324757.396	2258481.994	4021560.997	3603614.789	1568446.752
23706773.51	12303639.35	18913219.78	15567203.83	9362684.448
25880527.03	17366303.72	20178457.77	24324907.11	19308976.42
44268.70939	110552.8125	44052.93288		72935.21157
47853493.51	38789936.88	39024003.66	45405627.06	37773951.59
483126.3797	422120.3305	771868.4884	312568.0176	206822.1836
1635264.43	1034599.256	2092748.363	123079.9652	1325264.163
		353663.054		2328702.679
3929936.698	5605264.875	4342418.815	5207308.393	5871202
512992.11			440226.0366	322949.6109
40880213.27	18179494.19	38793338.15	31711713.63	11047802.76
491921.0039	494519.1291	255555.3019	66391.46518	1096333.56
76772394.76	59947026.65	70746732.84	39941818.49	51012701.6
5077348.716	6088572.201	4330456.616	7380650.609	3859712.934
23027781.97	20297927.87	17942223.34	14702905.35	15237520.47
913477.7828	629493.9224	779675.5975	535373.4131	354804.7939
185555689.1	147369374	168166201.5	120047698.2	81873461.96
2508904.524	11187180.83	954190.2727	1770286.723	1608979.837
216135037	115128875.3	180914737.9	195039910	192853450.7
8657332.592	6102094.487	10377009.25		9345011.808
75482112.59	132751896.4	31622963.42	39312816.21	89490821.76
23061533.13	31932648.35	30397430.01	29007011.2	19474742.15
1261423.567	422650.7079	2572027.935	1794201.85	730941.8256
553828.1783				
833921.3983	709684.0477	930730.6896	557069.0429	658748.4383
7799809.474	8998884.068	6416563.396	9812551.865	4705085.368
		149752.1615		
155675.5641	187739.965	86588.22813	97319.74117	35252.09559
5318199.542	5386377.962	6459566.105	6717391.31	10963911.12

54579236.78	30806118.4	48970825.68	29779462.47	52437340.61
8089747.925	11992900.8	11573879.48	8981577.726	14151887.61
22332193.69	30158153.45	24549589.76	18125935.52	21241394.93
21795686.16	15029920.85	3063780.849	2882216.985	13097077.58
58649886.07	34335088.16	72552034.85	72568945.03	44002649.15
86921537.47	100932662.1	63460084.64	84478937.49	77261748.03
1695922.483	13758020.06	999821.4432	667203.9065	4514350.279
6873992.524	4406705.369	5003985.431	5120523.414	4715582.192
6720076.837	1559140.808	2462799.254	3358144.647	1428850.686
			354610.6771	
105065398.1	56636540.03	50923993.27	65185044.95	71735115.79
5285979.625	7494643.063	5619808.073	2351662.829	1859881.722
		423399.966		
3683837.13				2581383.933
1436136.285	979608.5463	489705.1374	589890.3746	
1734051.657	1444618.204	2733321.402	1287710.119	531935.8029
8044695.866	10523592.01	7688532.094	2531108.825	11592564.71
8830146.219	4763408.265	8790699.984	5345240.121	4125863.54
2340463.099	632999.0976	2408996.207		
398398481.6	188387940.4	196558354.4	234028028.4	128377808.3
1906669005	1424900063	1188878264	1436970657	1924376613
14212222.37	10613264.59	16843064.88	12732775.53	6866192.466
218704.6011	174205.1181		120647.0274	111285.2909
14661831.82	17802846.93	11686685.29	15786081.72	16350785.73
14003494004	16333218114	16211075154	16235024947	14656626319
2246452.985	1540890.924	2714741.183	1621015.603	240487.1626
2623103.616	3198080.21	3350404.853	3529836.096	2077740.585
2578548.381	2287755.79	4182171.627	3100875.618	2643010.696
248591986.3	230283393.4	180487668.7	233240377.5	238611614.6
21257908.76	17032423.1	18417155.36	11345376.58	11877983.68
331439.3035		486034.3271		
753676105.2	562767037.6	667909786.2	749639273.2	497754868.7
481608627.7	547985186.8	499181148.5	451525472.6	404006065.5
1915019.723	512616.2925	414219.2379	1379206.436	1285390.139
208825477	137934588.2	175348855.2	154414664.4	169753603.9
1126752.473	782675.1391	1311249.788	528709.2373	743439.123
237324.2125	239986.6438	604730.3593	518217.9425	
177301.5226	99461.75791	324936.1997		
1497934.658	582056.004		2446988.763	383052.9259
31045629.17	13277820.44	20053502.37	20434123.23	17220584.71
28349779.14	49006490.3	40843907.3	25478010.45	46907551.45
6200007.501	4585861.91	6165602.751	4559036.865	3413814.521
6220347.16		5447765.831		
153111654.6	111561583.1	91194326.46	143795227.2	117650814.8
6685685.77	3761915.239	9913208.046		1162933.845
604879384.4	505018011.8	437833529.9	502252599.4	487067752.9
24282795.6	45306943.85	29935720.99	26464987.32	34977302.31
21734829.86	23368338.24	20013110.02	11998862.07	18433802.64

3027187.826	862995.7621	4847952.608	3197319.633	2623837.343
7595240.269	3565475.372	4580877.354	4341873.343	2879402.818
10788503.17	7357893.497	6232694.36	7763762.899	5865841.839
178600687.5	80318673.6	121975190.3	69362761.72	30539185.64
27228692.17	31129274.87	28451737.69	49865861.97	45289293.68
112595822.9	74717575.54	76688955.79	57423236.86	62913529.5
8922506.424	7725140.654	9665298.307	8124048.672	7215806.85
5480841.953	2491505.335	2520286.711	3551747.525	3014288.611
27276714.96	13991018.11	16792686.6	17303889.4	19071889.23
240149.4476	257852.3782	146740.9979	160056.6785	124604.4019
155584078.5	71398778.52	50924631.82	72260068.5	78129788.72
1966096.402	1085261.894	2341559.463	709005.2092	4593945.167
241666.4567	471134.1535		391806.6389	414959.4196
1398625.951	873922.6004	952229.8192	251533.5199	760974.8306
1206752764	1945944760	962000987.8	297365431.1	1320282514
	488030.5181		1088332.627	613000.9655
9826201.688	8621805.662	11174804.79	7582923.403	8879500.358
6805023.396	5500925.61	6713200.183	2987131.778	4257990.545
				263306.5516
529608.4939	468163.1819	434504.5643		146122.7755
2215645.547	1695339.473	889576.5761		1466122.77
5923159.202	3777943.668	6822074.11	4736373.857	4407894.118
579526.9113	445276.6405	468365.6712	373630.5519	179687.8742
17222292.46	11663248.08	14311040.68	13550986.35	10980242.56
57448406.12	51087620.54	57251565.16	63265176.23	52169414.45
3169972.411	4097340.543	3048166.389	4784928.303	3148057.349
77973404.36	48357648.5	42825622.63	43419300.67	76256634.34
2829936.753	3685909.561	2154770.873		4277785.951
1766857.289	1556694.494	1017155.476	665866.913	1727522.261
684415.8081	957167.1859	796935.2114	759576.833	841479.1605
1064000.537	849875.81	958099.3854		681202.2921
1591376398	1610611586	2038554859	1893333253	1932087653
434352779.5	486815586.2	379308763	350932432.3	396127967.5
3545242.427	2072797.748	2191093.859	1480073.469	1444170.593
7941566.298	6705093.955	5967466.288	7003292.561	6813007.473
41527629.29	26508872.73	37870441.86	32957473.44	21576219.25
3182634975	1764549413	1411340949	1431617126	2031321844
39276962.19	47133678.95	40403487.25	52422242.41	20578268.43
	480046.4112		172925.8468	934510.5974
986084.673	512977.6587		397857.238	572984.8865
22221250011	19585597920	22396314787	16842462976	20153716974
1680220.965	1274272.236	1089790.119	1182382.752	681151.913
1085002.275	532482.2759	954242.1192	4352919.585	
3467720.594	3985419.872	4062344.32	2490721.554	3280912.064
473274.4836	191569.2877	220761.7343	302550.7314	230842.842
4948061.709	3558975.969	6940218.473	2739429.238	2023972.631
680679.0365	922227.5151	340506.7714	666985.2826	
29532988.55	17563818.07	33763465.75	19797577.7	11743346.76
1632450716	1657322623	1754878173	1490119259	1772322835

914909.5758	853603.1772			1220932.166
1756871.137		159361.513	139557.0522	225625.5049
6390486.576	4730797.146	3478246.599	2876500.731	4828810.133
12540725.81	7401353.382	10600166.54	9423283.719	7181544.434
		5601344.626		5573516.831
4749765747	4721821301	4903857427	5150744721	4913811477
6552617.209	7054275.283	10001692.91	8274815.322	6585918.188
		393724.8323		645376.1533
851149.1663	1103637.271	667144.8879	858708.172	1571530.842
314518543	550377138.1	634116584.8	656871255.5	525744383.7
3756536.561	1792603.733	2919877.406	1573097.943	1567416.387
95251.01674	72022.32554			
1292145.287	735242.535	847657.6438	735756.7639	421640.9578
193129041.6	307544931.2	1248149725	311627232.3	186884128.6
75846190.27	90263396.78	61905611.95	48691992.35	85396767.85
2988695.515	1528054.809	1426662.898	3004900.71	3054003.818
1891130.827	3558691.137	3620139.512	2427073.217	3210238.713
56592773.5	36493544.85	51656167.92	95859421.41	74088427.83
17262815.72	23771805.81	11285331.6	8276492.886	12822956.97
22488662.98	31492250.79	11745520.13	25569869.45	13956419.25
11091063.19	19212203.08	12625321.12	12686422.46	18718257.42
549350.4324	234800.6711	356232.8408	243927.2072	175283.6437
7335500	5198282.119	7360831.763	6827751.85	3447896.852
3886361.893	3140221.823	2838347.337		2121974.195
3183988.255	3623060.725	4032124.889	3286567.335	1133804.616
4933839.233	2632731.423	3317224.993	2081544.664	2934318.304
9143236.672	8711056.592	12874300.64	7257421.257	16277130.57
1603232.383	1868504.63	3114826.37	1974139.63	1292038.945
477308.7492	578570.2691	384261.8344	322205.9741	672817.8613
913197.1499		1574771.925		
2841106.059	2640455.346	1854807.424	3008297.269	1093791.529
8963197861	14523697410	15063334260	16748875393	15354533684
3395233.72	1211655.283	2133137.862	1565863.581	1338256.939
86747215.11	108693271.3	91402223.34	102196906.4	82080477.9
389479851.9	254188872.3	324514609.5	284558965.5	353442226.6
8866252.741	228975.3314	10033532.43	5152369.701	2021576.703
82979179084	84322619478	67001493864	65823234319	90441451075
1130371.115	1244426.718	1819869.424	1775312.262	1823833.894
796401.8772		593771.8819	513510.2171	601581.9458
8610804.486	6268767.032	6371634.249	4378330.303	518504.5034
2102988.048	1269834.204	2035470.505	1404831.032	
1334381813	1028403805	798656964.1	1128121291	1721279033
97017861.79	69981353.68	79447928.46	21712662.19	39688325.91
25987976.45	35627713.55	30577766.82	20894664.77	26078426.64
2976423.588	1643801.768	7112445.054	7214154.941	1228406.835
179777541.3	200410221.1	185066997.6	254754160	188907426
485447.55	1493866.239	1735340.08		
189441.4805	1249745.256	1029092.558	891773.301	115027.5162
1852988.545	1578990.057		1319207.563	690555.4638

7730789.837	744973.2389	838358.7762	419347.5993	
793672.8075	460425.9636		427071.2852	476445.9028
2457330.723	1038658.017	3019077.284	2923968.326	2798763.263
45865970.07	26495488.65	27541290.18	32691009.14	20457931.11
100211292.6	71165165.95	85720619.47	69087640.2	57707656.65
86541526.17	71143085.85	34275182.2	60509761.52	76083381.94
18449528.73	15451768.44	20027906.45	15463950.21	15931710.62
1541333.937	2173220.238	1453373.659	1054921.566	607018.8584
1658879.2	1586114.576		719179.5504	
209329771	289929584.1	589764663.5	361589523.7	233341379.5
3782048.205	4823974.484	3549477.651	4315885.501	3270365.999
421059.3972		621405.3406	329527.8278	
341077453.3	315233197.5	203567211.4	130716553.6	279073139
88171.06789	122134.7164	86459.056	72158.70554	
2260728.846	2440714.196	738155.8839	850827.6811	2209230.487
16651187.42	11974368.61	5398813.25	14012397.22	18547159.45
3498429.608	2357637.508	2211942.256	3544010.003	3646227.665
1142990.692	2278951.497	762876.5045	857680.0613	953908.0589
466704.2973		941589.4646		
4148624.289	6442793.207	7878802.211	9749468.64	5812967.944
522799.4647	33836.99168	316872.6022	31130.34023	21574.86647
8441505.733	5850723.387	5510734.899	3984972.762	3798106.468
2540317722	2532393779	1913209578	2434857352	2170991526
			309909.0961	254531.0915
112355160.4	136981989.2	101910021.4	99547047.64	85667908.42
684810.5829	491349.9472	662119.1005	557668.4379	1195835.057
48556237.35	32968704.77	35198190.72	29457341.22	20672209.76
742542.218	247205.949	692868.2809	259192.4661	264130.3002
2412841588	2282993975	2198548317	2222046005	2514152384
90056658.19	102597454.9	111834606.5	81411516.36	57578559.92
9970390.832	11887793.55	12213980.91	16985180.93	4802290.8
6692436.279	771806.5567	4302995.623	1088872.259	1342567.398
4756453310	3383111262	3926210841	3625443304	4311209116
110097167.8	75192671.22	76326897.35	69059665.75	41534642.58
68797576.23	126027792.6	74740414.54	139357640.5	84786226.08
2947030.859	2714997.163	1852443.014	640122.0716	836048.805
7285650.477	4484748.825	5802842.132	3747220.995	2073660.988
79722854.71	38743727.07	53151491.7	70112637.09	56251174.21
927628.9618	1676698.966	1775407.923	1779290.421	1121721.423
	307952.4836	440928.2312	652868.0058	496923.0036
5586110.8	6158547.682	5656225.627	2270135.075	6039239.509
437184.7107	488549.2815	550049.2096	557070.2535	575521.4531
2750029.522	1806813.924	2491089.755	1293651.301	1472168.801
178597672.1	166837554.5	160359805.2	208332990.4	160258630.6
161762.6272	127959.3315	194605.5411	74540.88828	
6920123.161	6545407.498	12593692.74	8822800.285	6188247.581
1982338.757	1452141.308	1564396.033	2551358.351	1588620.682

6053884.248	1203832.728	512507.9343	1016093.89	
60370835.34	42975163.29	42904077.92	33495901.28	28007510.12
7152851.023	6268354.804	9190750.176	11713278.66	13900786.45
10115086.33	10658665.34	8603129.551	8922685.628	13360866.46
43381833.62	45819795.94	38422037.43	49583814.76	41612061.01
241612.6761	191146.8757	295324.8057	116027.1589	150124.7973
3226474.957	1856286.953	1553726.226	2337401.757	2054015.644
6517375.023	3720504.059	3399641.331	1392755.583	6494185.5
185316.1988			166252.3512	124046.344
457331.9459	2096916.771	2273874.179	1317701.927	1090621.766
84718244.26	78704959.05	69924377.34	66759331.36	111041188.6
14726937.58	13110464.75	19576991.5	13399444.27	9715019.699
270744177.6	354423519.9	239607435.5	41419935.43	97109498.59
75229475.63	61861398.87	56442261.35	50499340.82	83070098.36
24351610.39	24605488.12	21130390.99	10019188.07	25840580.17
709081051	491976985	296209367.1	511651900.6	366561068.3
12533632	8446046.863	7074620.262	12736111.17	12430273.1
346799.2341	282094.6807	384742.5787	279566.3025	181829.9
732126.6765	2843408.25	2075512.377	3448232.217	3789203.879
181243218.5	328868418.4	338371026.3	264714126.3	206440775.7
171205.8246	299721.5462	355150.1614	303114.2687	145953.9822
528022.0516	329873.752	285531.7518	235039.3304	141014.523
7372303.685	5971519.884	6069648.343	6921330.975	4305823.071
910407.2913	13161895.31	10223964.09	7329404.402	11921166.39
71316365.05	55530976.81	76785041.29	78240167.13	120292693.5
9309579.857	18940829.62	7565353.828	6114769.663	7533940.616
7625132.379	6191620.558	5065718.439	4036906.353	2945693.107
94070625.87	121374746.9	113490320.2	104144550.3	94065318.82
1962897.194	2724935.198	2803339.979	1857699.398	2265343.605
9544876.591	8226962.922	6765659.807	11682290.81	7569874.679
17215697.91	17112351.36	41982384.37	21030581.54	16545829.58
78201471.79	56913684.69	79594575.2	38145433.78	38043707.4
854572.2708	718830.034	1254291.056	239466.6419	961620.0039
150737881	119477521.2	163345333.6	104007318.9	83501633.54
17634962004	16330634400	15943244380	18123479470	15121248927
79793938.87	53334749.88	41943231.79	67335997.4	37449650.88
1385746482	1562455731	1197290960	1559321937	1925048618
2542925.586	829671.3114	915652.1832	1600328.436	816996.4906
684629190.2	995603319.7	693657031.4	728521507.9	924074610.6
290219.7816		747245.0026		
6162552.208	2580728	3915000.131	4083183.114	5171232.917
12583680.83	15027010.4	5343109.635	106634692.4	15496373.37
1122950.107	1027503.279	601701.1492	1320040.628	1127201.943
493330979.7	438963592.5	351169875.7	316333252.1	410956943.4
4390868.864	2273410.903	767159.9476	4927382.073	3302114.691
6687177856	6782973891	4720818632	7654476634	7077294148
1138794.193	1028132.404	1255368.229		666352.0647

26987982.16	24577571.05	23724235.57	19454411.41	12765799.07
2393795.883	1475169.961	806412.8698	889284.6744	596580.9773
9650329842	8850297725	11521217736	10180933723	12580746812
547717.6827	230995.5619	454470.0023	506273.3954	
285248326.8	222039161.3	211695111.3	215218350.3	181705731.5
707156255.5	431234454.8	409181911.2	467136184	409541069.1
268631.0865	1869330.033	331986.7319	1661296.82	2180355.107
85609402.66	61236598.39	68795034.73	91549659.56	45064215.69
1463371132	1444323251	1464398369	2139507132	1623386348
355951.4441		280057.5829	317486.8149	279092.3937
204982815.7	203236997.8	227767641.2	207521174.8	137411833.6
50050229.89	51049227.91	40336468.76	39799091.59	34189466.53
2547479401	1897580131	1796631138	1883954867	2549834369
939173374.6	991038672.5	1101790912	759015902.6	975401742.5
13972148.89	12462835.71	13863097.88	12744021.22	10867919.04
			18682.11345	
1765794.543	997914.0315	489593.77	1064252.974	1091846.415
4388497.175	2543951.932	2644032.754	3921736.779	3543839.169
14665229.94	20919627.91	19542107.12	30951528.04	31449945.93
7535146.41	12489904.12	11637971.79	13485593.62	6526492.366
799755.7901	203686.7093	752198.6784		529886.3299
828692422.5	1346553890	1520368620	1671326707	1400149425
68349246.92	44610859.53	54106371.95	21418173.51	24229170.72
1919220.989	1170248.613	747430.5887	1571178.299	4001171.253
2368427.53	289520.3125	1148010.06	1483722.53	374878.7006
1003174.69	761268.8079	1367195.757	628096.0763	1624470.004
2105539.436	879086.5698	1513552.541	1119514.366	3359177.2
4774690.553	12241696.74	5652406.333	7011492.537	3844147.939
416001.9815	706901.2378	706027.8007	543186.6854	450172.9112
3166982.99	9245283.784	6169397.394	7166092.352	5368529.239
16620626.51	10717451.58	9366308.772	9598768.313	7024999.92
6039361.754	6472101.864	9771931.654	12138263.13	11881797.26
8858552.531	9315765.042	7547811.014	5464172.881	4441601.396
340778690	296893418.4	515045717.8	247643373.1	154441323
8644722.663	8661706.389	5831725.451	5611559.357	4359829.941
9194344.396	8074967.929	7671118.82	5118576.264	5789325.312
1450057107	1726882842	1294866145	1762962881	1325613629
1185565.082	551606.1324	1288597.038	900305.7435	952350.9646
284091.8859				
134789.195		157465.0473	340253.9075	35881.61945
3187693.387	2670507.877	5395300.291	1518804.64	498206.7751
30264653.24	110748567.2	94495985.65	95849943.79	77509156.14
321099487.5	212257019.2	206212492.6	213689156.8	173261004.3
172880957.4	205488267.3	225301729.8	226803522.7	241988711.6
21555846.88	18527931.68	19430125.45	14540790.83	17171571.21
29108689.38	27971262.6	31605415.64	18495669.12	30610898.92
72437542.96	47433053.2	50574744.49	49945362.85	38987828.85

395759794.8	220968266.6	306614491.4	256661007.5	203990753.7
2124670.771	4524019.286	487443.6857	3482259.987	1867467.298
100524338	21568286.3	21226733.44	83932771.11	32685411.99
1557459.77	692681.7275	963180.0937	901206.9113	502498.2438
3167475.975	3571659.508	2210656.302	2053813.52	2013624.284
2609870.109	5771331.91	3117770.966	2398229.468	3178402.592
79826867.1	111911421.7	116300809.3	132498674.3	95985339.67
176217332.2	110556445.2	93448201.87	97299510.17	87337625.04
14440029.1	10070597.4	10100539.05	10275158.71	7478496.401
	274652.1594	174764.5497	123052.2745	
7829878.336	8563694.454	2746104.614	6164551.469	7286644.158
363035.2689	265456.5287	190462.2656	406348.7306	1105825.041
1257278.617	1291964.698		2930392.259	1810524.816
417669795.4	345981649	460738617.4	250067510.2	484760601.8
		187344.0136	184589.6437	
1073224.169	857203.5444	840052.4696	771718.3087	805825.4429
1148272.655	760611.0827	835826.3104		
673140619	760747049.3	781632901.4	888909249.4	696178351.7
801758843.3	781656182.7	686666375.3	632608249.9	1038288173
441934.4266			415830.3816	
705573.478	727259.8001	748145.7657	873995.0877	1017053.792
57854251.74	34588301.79	36765738.06	27603388.12	34553779.41
156122.8089				
1054518.059	1101130.317	1025219.478	1125833.513	2010429.976
29657475.87	22486832.06	19849819.26	26713730.62	20831271.19
947422.0226		1332869.422	867125.9508	
2436078817	4466583483	4103884105	4574401891	4349427175
1564094337	1813694373	3105470620	2600634191	1411189325
168199.8214				
84361276.71	75846666.37	64427401.69	64728494.37	60206968.44
2716057.732	1545550.365	2089139.432	2330833.854	1364215.858
1100064457	1437038423	1128505889	767962924.7	1466573421
470783480.9	564176235	295540842.6	460743590.5	475203293.7
13944487.77	10827512.84	15148654.38	10953559.24	36020686.18
612643.0833	284080.3206	478560.3158	302785.6315	183471.1625
5878332.405	4647993.995	2546153.9	1766649.139	2707588.507
31533632.46	34856568	24918228.85	53652089.64	25253128.56
1106634.433	855654.1142		827526.9134	636038.0309
434760.8498	4683369.237	381875.4896	3960667.166	876224.6388
789797154.6	1584908647	1639518402	1540867442	1223332909
1158610.063	710208.5408	1544958.065	775907.1603	327146.3702
29860785270	35078787721	42519164738	45982579557	36173781897
51696134.54	35008193.57	38836432.91	24584003.36	53690738.14
657147.7912			967378.2537	
403820.0291	402582.0888	568350.2192		
162667181.2	111895837.7	85315939.11	97736521.73	81883974.87
2671019.308	2138454.825	2360927.896	4002090.254	2039483.959

2654180.868	1852333.974	2195905.674	3007800.028	3322931.904
595176.1006		908691.9673	630932.7486	
923551.0844	596145.3452	1182255.13	1070120.797	994165.8572
169832483.6	132808387.4	93337695.97	39411329.01	100716928.2
47428431.52	27390648.08	35999111.21	36375922.16	35528763.07
6240781.089	5724319.205	6073171.061	5569750.598	5187118.962
926576261.5	774638288.5	1026196706	771304953.7	1043849159
10692181.94	14008133.65	8375675.946	7117643.928	7962422.867
13323732.24	14088078.33	24723520.5	16602552.96	9718451.856
157704986	150475613.2	88107035.91	77351967.26	79433868.04
2397230.067	357382.8633	1441950.642	787951.1662	
2537831.474				
15284026.17	15093845.11	20459748.21	16079146.35	17981093.57
17610849.66	13018503.14	13393607.72	13475883.07	9856052.249
1001480.668		1678406.26	790026.2195	3335912.192
160764315	88730194.95	91470660.56	75346018.67	86935989.34
2727415	3390291.145	2575643.191	2439949.043	498553.3733
19263053.98	14312586.41	9306289.258	14375753.18	13619211.38
270216109.3	156791184.7	81578834.36	125800333.5	165040419.1
17979120.18	12408019.32	11765252.12	12535309.52	11973549.82
25017835.95	57743074.49	34728256.78	31082439.71	32154467.3
5502636.792	4166694.435	4022485.091	5119143.972	3034596.503
1041624.263		1656697.305		1108212.996
37285205.72	32297880.76	34827035.42	30807053.01	33960874.93
8338867095	6663421770	7668346090	8056580986	7536014147
399356756.4	352173815.9	281722688.8	364270040.3	236815448.4
5388135.437	2853872.219	4954957.362	3663883.805	2354420.533
422144.0799	584666.0731	350038.6065	688558.6776	892457.1565
31318682.53	16818937.04	16477634.01	19987456.62	8741348.41
11381566.94	12931547.22	18913882.25	18798932.98	14858216.97
4175122.536	3281155.217	1588832.282	3423702.566	3575194.574
2173493.5	1653716.189	2382816.051	1635999.324	3591315.781
2781643.036				
54760067.74	68504251.19	57152378.06	70336302.44	61834122.52
1262807707	1334262391	980276477.9	713859075.4	1002747224
23151298.42	14971904.09	13095927.05	9307949.17	4784772.007
10531461.85	12980906.89	19317135.19	19306246.17	12841590.79
40302482.37	105737613.2	41585111.63	27833077.33	49061780.24
		579042.3896	143375.0916	
436935509.4	565758058.2	465525165.2	393345387.6	671387635.4
21433598	26092125.99	26981177.25	86020665.48	6284188.823
19881368.02	23981693.16	18467509.16	15541511.06	29607689.87
5052421.724	6614844.041	6221394.654	5515509.487	7547055.971
4015202.148	7005442.993	10696952.48	2991079.191	
4769363.194	4305305.79	4472133.38	5495988.354	5513908.672
7638397.099	9985884.491	6999690.493	2599771.869	4304561.73

39082619.24	22236887.24	17572691.35	27559557.8	23673540.93
	1066093.715	1366861.785		
384483186.2	298253951.4	334691490.2	294188127.2	269157744.1
138424.0104	5489602.595	900571.5558	1149699.624	841637.1775
17285997.09	7900574.64	18342105.81	12935628.71	25898071.27
550345074.2	562439068.9	535309915	486687788.9	697733565.8
20581768.37	19944476.67	8882468.939	7852396.996	9088543.02
9196737.472	5853250.042	11210908.49	1568507.13	1044623.347
8572134.251	10056856.47	14340908.07	5887277.729	9595155.618
133842595.3	138496766.6	178170042.1	149402364	93181085.76
2592729.936	2922180.178	2713151.545	1816120.581	1768864.661
10706381.34	14186361.65	12729523.87	12221700.07	15889977.8
	877056.0394			
27872245.9	33837968.68	38247983.73	49390538.18	37934105.84
2548938.113	2382756.462	2715354.039	1609650.582	1304717.209
915164.1085	813366.9981	914683.9317	382321.0869	490148.7335
4925204.849	3789351.401	5077601.861	4274191.003	5834862.79
8289346.241	13189271.95	5331266.934	2174996.433	1784345.452
8194790.077	6243480.372	4578087.827	7614532.013	6886754.685
249992.0487	154861.4828	300325.9047	202449.5647	216897.4319
7534742975	5836252896	6303142362	6723124282	8248869678
11041366.39	15797052.3	8304858.252	10217923.15	10165090.54
3342412.455	5953181.256	4639590.467	3103095.168	6344102.108
		23137.44813		
3499467.249	1892724.13	858638.9677		2949564.201
2822526.509	3829774.022	5248463.302	7537672.371	5118078.013
	4850364.191	4681971.874	2239227.928	3915922.349
141300769.2	96849829.12	102560155.3	95194779.77	60944134.43
412634351.4	487917806	387551856	490384749.5	382289015.1
3707042.42	2837000.686	2543538.271	2025507.001	2312687.085
14206330.74	9466151.39	5869224.438	4113780.557	3876089.49
248183655.6	155438467.2	211206420.5	186203101.7	197948282.7
845667.8268	1393745.664	8220680.48		831253.7781
379183.2248	382774.581	466677.1498	668467.955	321372.7018
9891974.751	5684807.691	10891333.71	7361064.238	6509253.332
331655.318				
1319972.079	519101.715	553928.4851	273891.2667	
1512727.151	2039076.452	977147.9487	2179512.725	872000.9454
48554769.6	50338096.17	38738644.92	36756909.17	57267115.15
1590111.344	1405091.037	1913529.412		3326553.431
5939182413	4089960037	4073093388	4132056560	3061934905
	350017.485	206569.9909	410847.3826	430345.9374
1025757.87	1116018.529	1525504.86	1818811.325	
947072388.9	1642629562	1673014285	1699464901	1143592120
5317888.867	4165322.944	7388206.152	7863392.103	6147381.704
254077748.2	293749417.5	152039535.4	261873911.3	353006290.4
119543.4992	130133.0944	96487.34709	81770.85927	63479.99177
1416466.043	1093432.636	2092393.274	461557.2485	903011.92
3726936.963	3673986.91	138055.5909	5726867.211	

48048141.83	91822217.3	55776315.18	29918013.06	30692160.87
87920892.01	57997781	63859368.09	62173419.07	54740907.56
543791407.7	973633267.8	450581759.6	397422712.7	448983357.6
1233786.104	1593599.393	3353649.167	3410341.509	4500370.023
776339.5047	782127.4277	972884.3568		212639.9315
178442.6915	436441.9558	425491.2282	111236.431	360422.5756
111587585.5	188862868.2	188167159.9	202803397	168496627.8
30415799.86	39429779.45	24517922.63	29741555.12	31633448.03
		894098.3262	388525.1091	695429.0513
20780405.25	20309548.64	17724259.28	25889528.02	18151032.41
332636.2691			332605.4342	344443.3234
6004276.839	8163996.454	4547454.852	6535307.069	6951262.75
1290876.193	7842454.01	9524572.89	8820302.545	4425365.492
1729507.903	285767.8816	262051.4385	253049.84	218914.3878
895550794.4	1456797465	1010862660	1247264550	1429596442
4740829.354	4129011.009	3954432.775	4358027.125	6742464.064
12643820.95	14924836.01	11662588.02	10186511.03	12157364.53
69897517.89	16976693.84	7617095.107	44963946.74	38743513.08
20158129.87	15644802.55	16145290.1	15776532.1	26201617.38
501890.2672	443501.6946	623390.5318		488631.3406
882598.1972	1195990.076	1036336.728	2839404.29	751561.1955
1702522.601				1907821.851
	514170.0127		593970.1881	
8387960.28	7028487.26	10430298.07	7378565.794	7130617.212
863740.7654	156949.0878	1323221.754	972647.7101	
1797364.78		1422802.265	1114289.587	1464292.783
		99893.24569		
240055541.4	186419933.4	152460038.6	158282107	269510652.4
6539638.829	5827090.759	10862606.29	8302264.582	4783359.219
14634538.54	11605048.85	15952799.64	10843344.59	9383974.495
10161922.21	7849713.336	7314807.267	5001420.955	7410568.433
1038929.771	235797.5202	301581.9981	1138423.574	2715105.905
325764.1459				
188687119.7	275131553.3	369906473.5	426681036.6	252973515
19190087.43	9361732.605	11011829.94	7609138.304	10284277.9
3690919.319	3122864.9	2031852.123	3250726.507	2759823.939
2875726.813	3024358.395	3998540.64	1385982.259	1736210.954
5300536.845	8483197.708	7597104.813	8518922.624	5657481.883
557143.8704		366573.904		
701510888.7	501075258.4	311823801.1	525581815.1	378612388.2
1180875852	727298237.6	949475268.8	668063220.4	944904242.5
13877057.6	9097352.349	6675389.084	9570063.325	27227813.27
51223747.66	36986205.78	32423237.18	48488775.92	42067307.12
6617017.324	395854.2466	108892993.3	114608.448	184840.9856
129928623.9	55365140.13	78582589.87	52918031.44	102417888
8299887.897	6587723.64	6827757.219	6565297.551	5752254.382
5086143.387	3361503.86	4609079.91	727813.0514	3995022.278
20265436.4	24318317.75	21640798.43	18363025.58	17317649.56

1639808.765		406573.9149	234865.2979	398972.4016
4298744.684	3541596.465	3339025.798	6330887.161	5648017.822
1420932436	739284737.9	946419824	703750404	832849124.7
47323336.26	28209803.47	23074089.01	61452390.89	50164034.13
8369169.372	9753537.371	9727994.563	9892196.161	5634339.786
41565400.56	38626711.27	39139507.99	46436826.55	47844325.59
5607790.186	5415907.303	5953961.8	5066866.92	7055331.224
49255598.34	44767925.53	22220931.16	31250426.29	47747527.05
78267792.1	80325529.67	82616124.33	85256712.91	93913596.34
349932.1133	374531.0634	266158.3791	253450.7498	127136.4985
78021385.6	38748027.83	30224003.43	34854501.72	72845028.95
299943.5934	247434.4021	239770.2418	264343.2618	128280.3032
122342.6992	126282.8047	100959.5623	107721.709	78148.69231
3600323.851	1438876.268	1923907.275	1084391.785	
2321811.011	3521415.46	4611847.827	2700661.519	1806196.266
		196026.3787	160614.0762	330165.1102
3540494.087	4436272.259	4316339.459	4314028.495	2134541.48
	922907.8112	3606979.062	2356415.098	1915462.276
272294790.8	173728514.6	208169713.7	141731685.6	394566649.1
68824218.76	65409658.88	69908257.78	36202861.89	53815315.28
607357.6068	369389.305	693785.4904	489272.9236	237312.9563
10575323.2	6990759.079	13334165.92	3152451.87	12302397.31
2568702.583	2895617.247	2137035.5	1444764.087	6579244.64
85847421.01	38178144.8	12161275.74	32249162.77	41059782.22
2691537286	4786889719	6629157899	4767606980	3330628144
11299828.62	9562141.761	9905383.895	9304802.292	13424579.55
901655.8793	308051.5211	661025.0537	322625.9464	696832.2492
117781658.7	60147325.75	70443356.58	63769155.13	63387795.62
8491054.63	7305910.787	5905823.552	7483404.578	6977627.051
42947021.87	41635043.9	48314770.5	49124364.51	41425199.42
4686270.976	1849181.623	1836598.526	3659063.126	2374914.009
30585552.29	31280912.31	71388460.36	31428072.05	14969017.72
37422732185	39886907380	56643780325	54569476764	35889113691
960399.9196	399452.8504	512622.6134	1130096.534	611671.67
484803582.5	484072411.8	736120865.4	626521948.4	764690205.4
11247470.87	14685499.94	13930754.59	10876853.6	11207489.17
7843038.507	4056168.387	7317314.654	3803223.646	6263204.957
20152555.27	7092630.039	5719542.76	4699615.412	2342020.536
4947.396262				4163.962555
93753.40622		89660.23827		385968.5093
1322089.671	1392689.199	1049608.471	1087864.892	679382.9649
286607.2834	136922.7795	117666.9528		
3141483.754	2017077.34	1702930.132	2078899.893	2562849.912
1182402.627	2141896.181	1502789.254	2636042.345	373621.5833
			252291.1996	
6284511.231	3616031.398	4587721.514	3511142.207	2619236.747
840635.3216	666645.7343	1130544.381	513479.8937	2100375.102

19385142.98	12953607.37	18694244.32	10394454.19	13727361.09
		88242.76811		
17493602.95	12984296.52	10153114.05	6645477.675	9964150.909
2756484.451	1290782.465	2684727.137	1747030.59	1030744.368
1570930.262	4402208.601	1740014.016	3415036.206	2061809.697
815118.1374	536272.3701	1101114.754	555633.0085	500226.1675
413120150.6	474866148.6	264047708.1	207554240.3	139606155
17761646643	12809717555	12664452526	12971030308	15052699588
215382.2352	217518.6302			
4961499.855	6879382.989	3514355.855	5446662.293	6908072.769
41677547.92	34132160.88	33211853.23	30917817.78	42733225.45
2676573.748	10476077.31	2141093.348	3607882.481	3955315.048
389306708.7	351674015.3	490982971.7	256664293.4	392081719.2
301108455.6	204099364.3	136153431.2	158013250	335276584
2475739699	2756939403	4092826047	3882857135	2946312549
363566.7909	138565.9427	320302.6357	210233.0133	128347.1468
2812775.296	1437611.834	535118.2102	1036156.172	2190960.249
993899.9541	3248689.417	2365634.427	5480942.373	4459762.671
2686546.697	1460852.283	2920069.852		1737554.798
1859016.427	5851680.669	4625713.961	4242718.54	3019556.189
8579545.147	4889900.556	4594451.85	4190881.369	3727725.511
2776658776	3808587871	3563565083	3747738992	3274143723
1491554202	1310300229	2031059138	1362270287	1196908883
6269762.87	4041201.756	3749591.826	3747998.064	752759.6163
29295165182	30974175582	23335406496	27858622719	22767754145
6074713.174	8131356.619	10397601.18	7012221.757	4205067.757
2650531.358	4064030.276	2152602.695	1299026.75	1109695.969
1852813919	2864647780	3585975635	3285212765	2006147423
7321570.45	3535767.918	2849111.396	3894790.833	7590341.622
14941211.28	9752767.671	10739119.41	9257940.337	8426141.667
1855377.68	1329111.099	1516814.737	1053211.123	738600.1487
4587939.475	2868529.552	160977.9971		1896230.482
	556254.1839	321068.1708	154594.1559	354635.5975
17662913.91	19428786.18	16113679.76	24302848.22	19140891.27
13076848.15	12829643.31	15591802.21	12669454.77	10368927.53
				437015.1495
604697.4685	590547.4923	472711.6817	345100.6821	
2734814.741	3066809.227	3343584.907	3116805.472	2783079.522
1710393.708	1035309.949	985934.8534	634970.3631	917550.3954
224817907.7	334397673.5	206527270.6	169830065.6	266348143.4
5566908.089	3350467.677	2770669.458	1954871.981	1292179.261
29377316.75	29829234.9	28815801.43	24155486.17	20389598.4
910477.0034	373640.5179		2210220.686	652756.4418
535808363.4	390613837.1	509627701	414701437.2	327785767.9
7913535.259	4791456.469	5347056.817	3361825.645	2738421.489
116632.3788	156717.1081	210442.0292		

8289460375	13395086951	12126388751	12052049099	14172073388
46836.40488	677744.7119	368169.2533	366538.566	522815.5137
265535703	199931460.2	206023407.7	280830636.8	246216593.5
3500490.734	2840912.273	1428824.536	3357927.503	1488129.052
42348544.06	32345787.72	34999172.76	41524533.54	24335230.31
232785474	418573178.1	186686708.9	179620431	263599753.4
2274779.305	1164119.66	1924747.982	1508385.772	1474619.712
128692434	106491746.5	97387072.28	103529262.5	96867829.59
2223942.25	495380.7074	412359.1444		637626.5135
1505213.732	230134.003	300744.7327	944706.474	700250.6114
3459712.257	2388997.856	3184930.203	5239929.414	1586544.748
152292615.8	154417646.5	150662935.6	179361294.2	125144236.2
30774433.54	21132054.32	19365996.08	19435711.06	12230449.65
4408565.85	9514566.872	3917514.662	5572147.64	4030211.691
13719113.51	13171036.52	9877530.195	4928566.105	5706907.237
		588342.9688	215034.7833	
54434.33767				34678.77346
4599067.195	4059417.166	3572877.248	3049790.995	2188456.343
27378432.12	33194123.61	25749253.79	32500637.45	51476665.88
313283.3053			955941.0171	
624829.2741	1035524.952	891378.5487	957505.5317	868510.0423
21989431.99	17371769.98	20559216.25	15554927.88	14264443.14
3968044.961	4874877.885	4374743.809	6849852.291	5658301
73472490.49	91742366.19	90155943.33	104790887.5	57359168.4
11627089.66	5487635.835	3242582.87	8007016.296	9685929.884
52889956.24	26818292.02	25460190.76	29106034.29	34264355.27
779742.9597	645136.4041	1178968.022	620733.042	463260.3357
5534404.788	9488332.881	6044785.639	2352526.487	7491531.314
415211.1352	696397.2463	559206.9447	1379059.283	
1081610.736	689620.5882	1062293.274	1678599.795	1217751.187
1500784.413	1319627.728	1694457.166	1419518.36	1054138.477
854307.6066	3647124.209	1135426.029	598093.9829	1127577.278
490693.3185	918094.4129	725159.2664	721218.8614	1120479.808
141684864.2	151918694	192835762.3	183850849	133479301.6
41250449.67	51744253.86	67539790.95	51427868.67	24995128.58
2007026771	1736749052	1468953084	1496276202	1043567777
1843486.027	911114.049	853355.7151	1031547.125	805861.2744
16043170.6	14082282.1	18543960.38	5792127.71	21200812.98
409982.1302	40945.65985	66925.46001	42747.7441	
	479778.4205	458037.1102	450531.3945	550254.2701
13118512.21	11976609.76	8716279.722	8446380.001	16453886.14
5954654.93	3538422.626	2988398.503	6693902.684	6308447.242
2181285.451	6567262.349	3372533.789	1278162.768	2244302.373
1984678.703	605597.9131	667264.4786	790666.1993	776802.7769
15199490.65	13523305.92	15358018.82	5924556.436	13518359.07

2606981.909	2967321.043	3002581.651		4565243.48
14134411.02	18029517.74	16764596.02	17480402.99	15742504.45
865702.742	1313835.974	1571601.84	1264982.382	834978.7506
776436.9343	477298.2499	1140099.323	649438.2414	1035295.58
2032037.163	1229924.066	130958.9564	57636.98793	70206.31037
307939931.6	192515683.7	221851612.6	241445911.7	215388557.6
10098535.58	5602922.082	4546071.893	6467256.468	6843143.522
1935799183	2444656316	1379477945	1919481132	1741384371
22502854.14	122023859.2	11081495.88	176171106.5	9195932.575
9495447.794	6862347.605	7114279.51	8520863.49	5679447.828
2093725672	2674591310	2680326417	2156230681	2200067461
837793.1139	334131.5159	536500.9083	1715206.469	742981.9845
47153087.85	43907875.28	49238217.33	50266037.8	49979788.99
481582.7871		805841.1859	562134.4606	420577.9801
2512656.009	309654.6732			
17487097.98	9477238.474	12675074.1	5851011.721	5291364.803
33415949.54	55988691.88	27429815.78	33657541.28	54026869.61
2775220025	3563920686	3011553176	3399308007	2789227864
3950167.29	6939383.235	2142472.32	4421047.993	4793357.86
19401223.08	25351600.84	21175248.53	24550855.07	20121945.46
4403993.11	9111825.241	3756769.465	7102668.134	4942170.149
354781.1364	321965.0489	569534.7405	288537.8086	
18763.09743				
464137425.3	287057927.3	430368699.1	159915810.8	321140537.7
66723973.87	65061557.51	62075775.85	80714109.49	89684240.7
109483951.7	246983820	276703187.6	96104699.67	64410478.87
7115509.964	7538617.121	8152776.239	5885787.351	5324060.7
		239671.1348		356167.6097
8348647.616	6718001.223	5536412.217	3812770.839	3233530.527
140643030.4	118591205.5	90750239.83	189722166	147196692.1
35723891.27	40919182.3	40576388.24	41003677.41	34487625.14
			87337.61113	96261.47763
	486069.7487	705316.954	188116.777	254096.079
509089653.6	570792054.9	677009508.3	687810582.4	591100340.7
12628677489	17694313777	12953818621	13985612569	13818304045
326728.0315	1421280.67	237193.6829	325624.0357	363304.7097
3192126.591	5009384.738	6603338.479	6171863.849	3657169.732
251828.0602	177513.2424	173042.0562	202162.1273	180471.2765
	259379.7593	232900.4166	703201.0784	853413.5985
888217.548		1792090.384	717625.8738	5340012.89
3225866.138	4521542.092	4611233.622	1740554.849	2290225.796
323496262.2	432066116.3	207757511	321298343.9	254793369.2
152629228.1	146990949.2	236501805.2	199967202.8	168903247.6

12960012.01	8676569.305	10821929.04	13018320.89	8562180.236
162236546.3	178521983.9	358565328	313331377.2	212484883
3284586.049	597323.5537	933139.546	1493665.007	1988178.212
146677801.8	100628033.3	85194144.53	85905081.76	80891335.04
282756.3513	6306.089087	7889.94807	6498.616487	10281.9995
175884132.4	139313273.5	153921996.9	84185247.69	120651091.6
22548261.58	17642967.59	22240585.23	20088850.21	12463004.12
540781.0526	726880.5008	814150.1742	728253.6428	688044.3913
89669031.4	109510030.5	117622636.5	130438002.9	95564463.68
815082.7423	285973.3193	295605.7546	155627.7913	368473.4055
56369.60524	1467014.041	506411.4745	464785.8277	670652.9806
	387488.7292	331044.6293	9604496.116	486682.6795
105503361.7	125863715.6	104662190	67691687.67	88636392.28
5234756.644	8254839.096	7543593.673	5739627.99	5474216.277
1565311.246			2231166.919	1075093.033
943908.9617	681102.5676	520259.9736	623874.8308	402270.4237
131513286.2	259557763.3	253281045.2	272224334.2	213730329.7
4066724434	2438293749	2319329003	2161253343	4783171797
984424.9679	735319.1562	893212.3724	737743.038	2247953.783
477546.142	313544.5585	335622.7143	587949.7201	
2668762.868	475607.6357	3883472.272	869496.3203	1124114.915
	533091.4969		367427.7596	
8526719.977	5603809.709	6242622.753	8192347.708	6918636.562
2593175.052			597154.3681	433061.3928
543396.5172	420105.0413	831540.6162	375383.4401	757500.3405
1999513088	2278333145	2534605413	1913981828	2804014425
712332127.8	561508791.8	433013403	417954024.7	426716220.1
23067077.2	14151130.5	15186375.54	20286841.89	17505968.51
1299207.882	1011242.803	408728.5945	874511.6988	
8349481977	8682576313	8531649903	7274419077	7669190926
20994928.67	16730109.14	26933084.05	17701494.2	26151283.93
18026402.47	13572472.95	11059185.66	15802955.56	16968794.8
584077.5296				
34807182.67	52631284.44	52436436.4	50357558.47	30708101.81
1092785.414	687224.131	350999.8998	895521.7293	679403.0306
9527005.985	7084051.243	8215479.883	6954523.806	7278539.195
4396917.65	8083064.794	12230352.41	6992180.997	10100180.02
4461809.254	2544466.966	1745842.483	5227722.565	3636325.824
12821187.7	15394423.65	13202234.09	15967787.75	17696201.13
11513299.15	11461909.48	15736873.18	15691632.82	10059553.53
6729629.659	5751802.209	5269020.368	6054146.962	
6356390.029	6527768.091	6462761.269	8236992.025	5364894.92
1291470.028	1152266.229	1792495.519	1993252.32	
3184764.272	4638374.193	1843729.914	1606385.847	2904216.984
289198.9924		208940.9683		242216.8931
2039905.24	1187652.448	1948011.989	1170301.808	960798.3156

57235202.55	21830914.22	34156945.4	44424190.89	34258418.12
65357715.17	49274594.43	65686876.54	46508114.49	38097383.84
27858285.27	11882075.69	7509919.208	14233614.06	10766235.54
	1471891.422			756053.034
135201704.4	135369618.8	107791549.4	94920735.06	119520442.9
1033105.411	1033280.076	422430.3285	728107.5797	598644.1558
66607.95716	72405.84238	107763.0153	25333.03596	38469.92227
1453321744	1821113670	1132881160	1048019250	2171762041
1261288.548	690277.84	861095.6474	949446.9729	380812.3455
11424217.45	12418591.35	26565382.01	10711916.24	7511857.529
17173153.02	12037188.03	15786037.1	17812174.71	10116014.25
775789.4114	2205055.406	1103834.792		1135623.871
2838365.278	2769933.421	2300541.703	1921494.625	2009657.619
3951617.209	2685362.6	8846682.957	3187505.627	3670739.341
299290.5306	229024.8405	213962.3464	152317.1776	47412.3304
279700.3227	9120.12119	461604.0106	195635.3289	
3680679.117	2915628.289	3632786.464	6646546.217	5478805.165
3381200.81	5394321.603	2901477.661	1933424.107	1858868.116
4864022.671	4660683.824	4715493.334	5188813.796	3108881.035
30992746.7	30739042.42	22446682	35762856.89	40814878.35
184221453.1	143809582.1	142498081.6	117007938.7	80595091.89
2116937.118	1727042.926	2135796.672	3333717.266	1842368.138
4475360.724	2540949.635	2381953.837	2473194.921	2325279.9
	183465.6208	417088.1875		530160.6381
3774937.202	4720889.566	3953488.253	3785662.135	3766393.86
32671159107	33943786710	30984360093	37154831327	33763315643
370060925.5	356229598.8	285739795	332930341.8	286056432.8
13150828.27	16991064.17	14245127.29	7229427.368	8133814.348
532972.7427	342565.6494			
20146395.47	21395545.55	20273577.37	17967394.75	21008992.45
100508.7106	91653.74461	133290.3245	88071.73159	38508.45011
1186121.338	2374772.268	1355239.572	2541771.94	617176.3692
998074.4803			133918.6427	868643.1026
18845358.86	15120872.1	21966623.33	15904535.96	6651940.206
18744657.97	12251302.3	14875578.94	8776731.247	5781494.286
	112531.7468			
269157.1617	299199.4203			
538686.084	372904.9927	346929.5824	159140.7526	153998.0227
1880745.476	3310087.319	1716240.242	1722011.001	3264557.101
131276665	174718840.5	188692729	116372029.2	123886120.8
1261890.322	859930.7966	613773.599	344106.2383	449672.4525
14671284.63	11851590.91	13852166.4	13389410.36	10725466.2
320310.7656	453436.5221	243612.4009	322367.5255	423849.2898
22080836.12	14770026.33	11269340.43	18736446.66	10975222.77
67867188.61	45403406.73	34021991.74	45422590.59	38268734.56
6402302.364	7673696.44	5761169.711	10270130.86	6825026.178
4637018.522	2807656.375		4778805.071	425169.8602
135332536.7	181453727.4	131246907.8	129400053.1	135314232

	1067935.176	1333941.05		939108.9272
22483095.46	23156594.05	21437824.77	18294640.06	17192548.97
23533491.91	19238543.12	11796818.98	6253451.394	12296429.5
		120343.4094	332406.3148	980698.7083
16710375.95	10999193.07	6459403.686	9474075.177	13353236.64
10629948.48	9136639.352	12046915.23	7981772.145	8814712.78
				1634510.531
2150171.001	1840394.278	1524658.609	1528785.8	378105.9913
6593176.771	5262098.469	5255901.853	3547690.425	4507715.77
6634909.355	4753212.152	5438832.172	5916390.506	3358669.36
1235259.269	931620.0227	1672498.314	1363569.4	640469.3858
25670476.03	27318655.11	24443779.43	24876375.52	18379193.56
734336.9397				452807.4949
3654458.194	3199070.895	3144267.271	2051841.293	2101071.38
970164.0714		264683.3462		
5433527.979	4252568.716	4535303.779	3787563.293	2980947.303
				105357.9971
2723724.461	1696560.157	2344921.827	1972604.555	1270566.48
665371641.4	917120737.6	948989248.2	619860560	903971176.3
40205028.94	28060864.71	29279651.59	21515279.78	20992768.79
149970.1236	228132.2852		105705.8419	119867.431
	342570.0167		966714.292	
				497269.3869
109155264.6	136270034.7	87610914.36	88848499.95	119444538.4
24871.94316	18002.6634	31751.63187	9046.670158	9596.744199
67953383.55	70529723.66	63191844.56	63150534.53	47481938.17
1406498.704	1142195.049	1640285.952	718587.8227	405368.7567
1207151.807	1022416.015			721860.0956
296594.3093		1608198.687	2010287.496	508357.2696
1017751.503	765580.6222	1255493.847	1270238.733	405202.821
32978248.03	21315026.72	23805595.08	36851227.66	13342645.77
28918183.2	33520550.72	32349399.73	26539728.03	19785106.55
5822772.329	6889444.044	4050765.303		2048216.744
54539561.02	58636533.12	65221550.38	54479442.1	45372220.53
1422966.852	410314.8046	791871.8984	659956.3622	98388.91104
114756845.8	93901922.3	162790983	89352476.33	92309604.45
10349037.36	12709945.04	16740323.7	19751135.71	10495359.08
332726.3929	277988.4974	383077.3132	276354.9506	679950.339
903479965.6	1162277330	1150562629	1144125047	724526694.4
1588051.417	5230716.111	1426646.331	925295.1631	2256230.935
235721323.2	275054931.5	361897266.3	387302673.5	202615811.6
750964.2915	660911.026		823983.6191	425052.5837
24641937.88	14846536.88	17058477.17	10785409.39	14044790.15
211189.7139	120692.0052	142551.0991	105111.7506	143180.8291
1774286.403	577178.9511	628761.324	535123.8317	359412.7982
229304030.6	199510155	240833107.6	132178922.4	227565665.7

1659410.275	1527166.659	981558.5173	1146195.339	
2858165.7	2132057.802	2036641.296	640062.9527	1393847.418
20316922.13	19398803.45	15066115.27	15093245.34	13133287.97
11087692803	10331813457	9740244768	12567283536	10941904325
48315899.08	40860880.27	48980267.27	49591966.17	51023225.37
365764.227	255023.8081	229300.6761	209048.0207	267857.7451
8666692.153	11887381.74	17887385.13	14147139.36	12819541.04
4224079.708	2900637.03	2451748.311	3103554.601	2209914.583
491704746.4	423009645.6	435030691.6	441938653.6	308341725.2
1310678.697		1245439.966	1097579.627	
1751935.262	1013216.906	1215686.06	1852501.657	1646845.458
2358097368	2461821520	3220262086	2160705103	1955005224
156773.8267	108744.3677	240809.4606	119688.1626	64395.43768
14985436.59	12631232.58	11679320.41	25836321.22	16713121.06
2042122.232	1682479.222	2716112.56	1973701.345	1657002.958
2372702.442	1906608.014	1316527.715	1539293.544	2763360.393
15744795.4	12904855.27	9711506.112	12516700.36	13120014.36
1142713.015	595773.3119	485374.0851	914591.6818	648687.1285
2060061.807	1193461.053	2707398.092	2822708.742	
1653819.181	1493070.576	1664908.21	1396508.143	1610276.461
661936993.2	417342850.4	579904628.8	445011239.2	246151448.6
5718178.119	6638083.813	7832134.607	3265694.298	1472963.118
133278.8225	98111.92939	76828.33246	35638.3434	49733.61231
1454823.447	1145343.187	1319824.395	1267613.593	1010423.887
13613575	44200877.58	26280907.07	20076454.39	26611231.03
866346329.6	780787379.7	1141298632	664106488.7	474019765.6
6991752.259	3670953.753	1818146.874	2743855.946	585647.3134
1679475.567	554735.209	1393174.889	1155203.788	346198.2972
76600675.9	45748882.59	63718694.11	34665819.27	21789134.26
2030998670	1414934130	1476040524	1250684413	982898921.7
		400994.3041		394047.4317
3306496.051	3924000.713	3204348.627	3249684.119	8035085.629
10908213.13	5907394.222	9188035.273	5978914.604	5247050.322
381278.8998	235262.0798	602008.4237	83153.14566	195123.3378
1544791.432	711824.7852	1046245.27	839785.8392	695035.3346
2821232903	2018610192	2295312989	1475232382	1092426039
2326859.764	2162139.474	2091375.618	1242037.269	1032133.413
568236.976	488073.8047	8593594.888	14201255.65	10248093.8
791940.3045				
293238.7977	185461.0967		70200.9533	
6975314.933	4646030.713	6415409.777	5307254.58	4607742.163
36608044.1	20050815.51	21797714.86	14171511.31	10050983.83
635396886.1	553223152.5	630905759.3	584815125.7	525865781.4
29562768.66	26645655.94	22923289.56	36495198.45	30722441.08
706314.0247		697655.2589	499917.6414	652710.0042
174300331	120295249.4	107622196.2	128667207.8	113331439.4
533608405.6	296208463.1	304191904.7	360490026.6	188014191

259155.8401	123629.4683	658645.0716	108650.6348	29092.72111
3033688.58	1748272.555	1841740.853	1314481.736	2250140.796
1454103819	1013148681	665674993.4	899203672	774096446.4
10269360.56	4668648.027	10594865.09	6133701.845	4490960.059
1311166312	1229064722	1513432723	1833081736	1455394151
16922823.62	26475482.04	18093482.93	24608207.05	20853632.81
282968.4619	541670.1382	371078.6867	971293.1144	871846.2966
14748605.34				
809683.8303	204604.9468	265889.5233	617856.3229	279036.5144
31163972.73	19443055.39	18185587.07	22746073.11	15030918.23
571851.2217				
10416279.66	8519495.179	23383321.98	16098530.36	7615984.158
3296575.001	2813654.089	1954540.846	2117814.477	1184942.08
3993776.412	2757627.828	2811161.767	2844301.373	1993283.271
1477498.573	1194726.028	1255430.443	2059828.511	1306857.139
2415815.492	1032479.852	1392263.41	1200677.826	1150268.91
		462693.1115	992732.6294	630479.2015
5642079.501	3482026.048	4910915.25	3107519.44	3893735.018
7140998013	7991385116	7763354316	7016494955	7207820514
531409.7423		316157.8904	287424.8099	
100740876.5	108091937.4	143932378.9	83251683.85	97968755.69
1899483.486	2109257.795	2972204.118	2270576.791	601703.1997
12872056.78	7950625.738	12264002.24	8122503.515	8273459.527
546075.1919	574753.1208	2035704.254	87390.21576	802768.2265
4962689152	4783274883	3506104776	4172446952	4544064085
1077867.105	781129.3419	808922.9356	1260353.635	968066.0215
15461703.78	9829010.846	42619863.98	12598521.97	11447285.41
36868197.59	28063358.48	32965037.25	30014449.47	23820834.95
5142020.848	1244191.443	2580404.486	3865551.766	1026698.847
1356654.423	291920.9438	1126270.445	902644.7904	766274.6496
1815299.726	722559.7974	555194.1971	1005947.22	1083631.664
7022102397	6098970212	6661699994	6545743640	6779909009
219684.2881	287466.071	63568.98468	193245.205	113642.7178
2354505.65	1149342.315	2049181.742	1125060.872	961613.7692
28751868.28	18556722.33	20247450.61	19556049.93	16402009.87
15231494.7	13820123.98	10735538.67	10874319.24	9916623.775
130163.0739	368477.1161	211395.5922	168844.4768	330557.322
41541067.91	34182125.69	39249988.04	30914999.35	31780412.47
7893685.369	5742089.482	5239569.849	2758616.516	2330667.679
4203387.938	3624581.807	3123011.935	3433951.428	3507102.767
2989051.716	2185785.541	2199244.168	2109317.719	1443247.502
1742252.608	846893.2051	1620220.57		
56774074	54802683.89	63147844.81	37826938.17	37426632.93
58251910.97	26816332.73	23278002.96	35547707.41	25293150.1
1462616.03	452751.3949		1947244.799	1383766.258
678963.0227	663034.7773	654371.4519	455177.5509	693498.736
8891194.048	4822311.953	1859039.016	4500587.195	4075964.918
39505755.5	29410989.02	34682959.56	46831759.03	25988742.4

1864873.877	1414961.476	1112309.809	1940374.125	1583027.956
275632568.2	378601194.1	620202968.3	423335261.1	247274923.5
1458056.972	3165199.09	1378068.913	3581305.047	2471065.224
5462350.543	4639464.92	4580228.247	4663906.764	3760138
1234091.81	32761.20728	666822.9669	1304304.882	568145.2583
17005859.85	11809490.7	14183810.88	12646432.49	11737541.06
1525525.32	1072296.924	1098242.605	1224718.998	809298.5198
2196297596	2449309832	2119480936	2467987311	2523555607
46552972.73	147946385.8	118832540.9	66868240.59	100282224.8
32205252.23	25043685	25502370.19	15824485.2	16456037.46
527253.7502	904816.194	779457.2532	844724.9481	629340.5835
36952649.95	40819644.75	100933249.6	67388059.59	27497858.65
34052586.18	18772039.99	14048803.59	19366173.01	17588856.67
474114.0029		390915.7897	539129.1244	419511.4551
9189989.451	6700488.047	5934627.428	2678778.578	3817725.753
2712531.788	2829175.561	2767587.802	2041181.74	5587254.06
11920339.82	15102037.84	12065336.43	11283034.21	16729777.13
3411312.522	2173142.168	2713030.243	3055601.544	2585671.532
12081905.5	7251759.498	9385740.071	8580828.043	5848718.908
2253283.876	2038324.114	2513811.714	131210.3269	2315408.892
3032008.946	2986694.216	3394492.455	4964475.535	4231430.292
23354453.91	6017294.276	6622704.159	3301488.738	7780906.475
976129.9748	764198.5799	431657.0473	544102.1501	573909.0887
42899.54104	88051.87009	62010.45867	51216.3452	41970.78656
25376542.45	27240866.33	22881913.29	14866949.69	25217980.89
13711713.86	9152150.331	15134210.14	16588893.68	10187182.77
4115151.006	2837667.61	3536934.41	4001390.223	4580096.574
1494353.39	339030.5986	217816.2451	1187645.368	965036.3596
10377756.36	8009431.417	8839559.011	6443083.569	5665415.347
	1027717.393	726213.3318	1384000.789	922063.6612
				442293.3823
474355.8945	2516955.516	345492.9681	681915.6974	1037789.202
32219328.55	24489999.8	22616964.76	24921069.58	17250299.08
29984498.7	40257758.41	29198501.72	43170780.88	14834134.62
2001097409	1908619425	2049620703	1465545725	1647455205
3759989.242	4024292.609	3893817.056	1925326.721	2911231.737
3622505.647	2504299.166	2163351.321	1774753.584	1890423.022
7970493.406	5970911.199	5539023.493	4612874.516	5315015.321
1357376.974	3967848.159	4617601.885	5886206.305	1609426.967
988202.061		852683.4684	410716.4427	754171.3779
6726044.488	5108538.765	4831091.319	4736798	3437292.73
2316360.457	1748431.859	1770620.866	2622602.756	1690141.115
210827.4156				161725.7335
1994685.044	1381586.154	1393989.401	3976928.464	1216611.136
9216063.667	10620786.58	10766947.59	9337613.747	7956722.682
914464.3109	941969.9383	1235113.86	1233031.049	1128131.197

475638274.2	628183283.3	323451872.6	453020646.2	378171568.4
11146573.23	9961152.47	5299588.711	9597626.249	7497788.357
3223486.763	1311908.327	4801153.767	6965574.141	2765810.381
129816.261	270999.2878	115649.0081		166650.6497
10094372.68	6094217.766	8016210.847	5386957.852	4350051.861
559259.9943	438354.1388	501324.5638	482317.8898	365647.1232
9128303.342	3642439.367	4623931.003	8431673.206	2086535.538
2689418.834	1845315.816	2159098.223	2138099.323	1155016.182
3820960.443	7362564.668	3786186.255	4626932.702	1489497.243
5100071.32	2802582.175	2901022.852	4680328.662	5070177.944
6525567.725	1765012.585	3155597.804	3144911.623	2205915.965
1184624.732	642244.9717		604810.9122	572835.9349
2963754.618	2935008.409	2798884.778	2409050.399	2550940.905
335556.7402	555644.0354		180297.1057	332609.7153
18691184.94	37318509.52	25350415.97	45740926.25	40392673.86
35156986068	23448667006	23014607601	17653965422	28231204826
643415.1449	319007.3339	423251.7241	721856.6121	449047.1209
796388.195	895922.6036	1047403.447	950265.059	1010653.352
14846580.32	13649829.4	9308569.11	11609413.89	8680513.129
2186298.214		1783186.642		3324393.077
2025290.503		844910.071		896351.182
94584560.67	123321357.2	113545177.6	107571929.2	97639478.51
15915316.41	13793317.33	13422186.6	11257903.11	17029460.92
1846791.123	1110482.398	1860250.663	1824471.584	1780665.269
4749128.474	1936427.395	2441752.197	988061.1267	2282900.617
57623428.37	42870160.42	48009097	47643946.97	53550972.73
120139279.8	119919824.7	107986065.9	122598048.9	166225774.9
319068.7753		386763.677		
17839421.57	17886857.45	15166328.61	32007182.71	17494919.56
5386926.63	10841270.88	9349681.251	9997755.681	7558386.698
10781461.95	6690260.673	3521945.067	4614191.678	11120722.44
12304321.89	12059642.67	11195916.04	11661676.12	10036529.92
2052342.515	758109.8873	1311790.793	1293402.219	1069870.375
518614.0262	440848.8412	533543.0284	484126.5668	625853.0665
810073525.7	737707608.1	1090168953	880847486.7	939171481.7
2450289.99	1497271.275	339053.0036	390503.5813	1465817.736
85689.20655	220681.609	3749610.096	9183605.832	
4198428.379	2584745.117	1336240.139	2273203.506	3080618.105
641095.2758	686698.5847	889205.8225		484562.4572
63605783.14	41671185.54	64200419.03	52609767.78	24815796.72
1476245.67	94789.77553	59617.3371	60767.22295	33077.87294
1540776.833	1391415.795	1414755.144	1244354.103	990978.3306
6556074.184	7455405.259	4726270.305	599954.9907	5853877.786
	521126.023		1316067.926	896335.4162
2191772.415	2108654.642	1811022.928	3102710.851	3692630.389
999448.7191	912037.7601	458822.4128	1126644.018	897302.4371
18264182.7	15187775.52	18252176.48	14958453.58	16103548.67

706988.0196	463001.9058	560984.301	513791.4669	488311.5801
		264037.2623		879130.4133
3325364558	3643407924	3822047085	4639665738	4284591472
775009.1567	609762.8619	1025375.571	1609286.048	
6379622.679	4175572.372	4096142.734	1914428.91	2894594.18
16061828.06	11900824.14	17925658	14324611.89	14739589.11
2194244.349	898311.9301	1060526.129	1141845.19	1487715.906
3103058.004	5740348.175	5034285.719	5246064.949	4370724.871
24006902.89	46171665.81	53052810.57	46946202.65	23551708.3
				4322965.763
44025931.76	34332131.64	30463464.53	28734195.17	26256762.93
69842239.4	52301091.66	52083557.17	40728690.07	46349969.31
26403917.94	13112606.66	13234132.03	17567908.54	14427884
57262255.03	34486151.99	46341778.83	38601309.25	27641371.79
566971.1907	343057.9964	528443.9386	352823.4071	328983.1361
504223003.3	451350670.6	498690802.5	1059043505	286847540.1

Intensity A_GH_6	Intensity A_CL_7	Intensity A_WCE_8	Intensity A_WHQ_9	N_ZQM_1
294844.915	323408.8578	390474.2813	363719.8182	
178624643.6	236568497.8	248333249.1	106507693.3	0.721
120531.5757	39021.32343	4224.07373		0.017
			338399.5768	
8527892.586	4648187.456	5521218.375	22604116.67	0.807
1042477.191	3554329.456	2050522.188	1892020.477	0.141
		540173.625		
68818600.21	71567410.09	101855088.6	160349652	1.452
1098394.137	4602729.9	1360924.027	5338147.239	0.515
1943554.888	1912939.243	2164662.531	3515133.707	0.93
13404039421	14960242727	11757505998	12815493297	1.226
71212.35829	11686.27645	57066.0625	148993.0775	0.668
915151.8051	937185.5792	1168318.75	537414.659	1.592
3140310.504	752552.6492	4505762.844	4290473.187	1.93
132001736.9	45784048.15	19967398.25	8940121.068	0.996
		241571.9063		
8345029.112	34562056.74	8542212.406	10047005.11	0.936
2260166460	1608356884	2132587357	1789400732	1.299
12190905.31		649525.7656	9601919.602	0.188
39852778.65	35149205.62	36311049.83	72533452.46	0.512
1652763.916	1739896.446	2278598.391	4058715.721	1.068
5048536717	3163237247	3278933540	2269206350	1.057
17323.76707	5707.895115	55992.40234		
77872724.36	73672826.79	111924848	84628098.09	1.579
266731.4339		6214180.438	427080421.6	
26491469.97	33912201.11	25741056.13	42044135.6	1.108
			1880439.057	
1016512.111	508464.8304	866119.3125		1.235
11550768774	9523609105	6201594911	2492894511	0.992
309668.2974	1282954.953	545997.5		1.578
		1017451.438	335135.7674	1.721
10510566.65	17082699.4			
			15594.13801	2.153
6924058.719	8811661.554	9348652.063	3410406.524	0.963
13134602.94	41801419.31	25221207.38	21030743.16	1.501
	324118.2115	180035.6406	794077.2281	0.681
3959875.696	4296233.174	3645846.375	1988747.411	1.408
				6.457
23358775.83	20549975.92	13217563.58	97751521.03	0.739
	239125.971	1307144.688	481124.0255	
425595.4443	78936191.59	64447358.5	1135857.444	1.013
	335102.1679	849179.5		1.17
70683.34026	105345.1801			1.266
		197946.6875		0.458

			336134.9526	3.288
144257.4066	184172.0679		983087.5824	
2277162.732	1629094.714	5169046.742	10114609.19	1.145
			190500.4525	
766865.8403	1542886.337		1320908.708	0.741
405846.6891	1915071.162	868616.125	752632.5992	
1342643.857			1245034.005	1.227
	147722.1161			
1298564.363		1172227.688	2008958.849	0.586
396369.2465	757465.4924	606894.25	820421.1936	0.962
2459117.693		1180491.75		1.282
2088462.833	1280083.222	2010117.563	1234014.384	1.444
2479780678	2550479857	2282963198	2687711650	1.24
932798.3999	952153.2569	1175738.031	1038689.239	1.101
2169586.961	1303170.311	1303447	881001.5517	0.935
2610556681	1658821203	1959594458	2203758601	1.947
20947194.02	36240922.77	50041735.53	30111767.56	1.684
3253173.466	5919213.845	2920029.313	5771743.684	0.833
6674381.674	17889189.69	8972673.563	23759426.32	0.924
166230319.6	249818275.8	134305210	158205504.9	0.895
4023526.463	2720636.575	2260067	419971.621	1.333
7864298.993	4557292.459	8807658.719	4591742.837	1.014
			52204377.52	
39428.95158	254276.9002	42095.42969	202175.2754	2.141
341483.3427	1014576.451	253880.9375	116982.647	0.809
176791.2004	3259335.956	3018633.5	4287194.513	1.258
1757613.969	1222891.197	1875772.813	719884.6869	0.881
1901301.185	2035511.991	3319380.594	1020022.715	1.842
1073403.226	1668354.618	873015.0078	2530018.597	1.211
198145.7534	3524166.246	1712230.094	866132.4911	1.235
1247777.959	1387969.684	1466134.75	4075732.037	1.737
155440.0748			4028743.469	0.562
681816.9081	1670501.262	673395.1406	653863.7702	0.248
25779485.47	17021687.52	25034306.5	19009416.57	1.099
3076399.616	62404.61211	12006722	82316296.37	0.848
8108022.61	5038110.158	84831310.16	203293408.9	8.466
11876408.68	56220150.51	27038806.97	17076842.92	0.513
4391983.162	4346813.549	5300776.039	12418813.44	0.703
208972.8512	1079585.472		634806.6801	
92556.47749	23649.27861	140519.3906		0.34
	474683.5825		481473.0644	1.211
540830.3964				0.884
2360434.711	10500079.47	2238252.813	4447310.945	1.993
819042396.4	1612708909	1304259997	585219405.9	1.122
	305669.2464		892914.9548	

	933173.1172	1066980.5		0.827
601534.4534	1529908.502	817542.5938	901905.7338	0.8
216858.2673	78086.33329		348403.9339	
25725312.62	28062270.79	23063195.63	26109104.52	1.105
170561.4814				
450151544.3	404408414.5	335561211.9	423222107.5	1.473
12542485.48	24808711.93	27516801.05	32604199.95	0.787
5915557.151	5075001.576	4521721.813	1260830.724	1.276
9739601.892	12866154.03	12828170.03	11037292.74	0.578
421816.9496	974241.0884	652741.125	297326.3685	1.005
		494008.9688		
17869530.39	17918752.05	13549886.66	15801436.86	1.496
19558103.87	17107637.21	20913398.63	19866694.36	0.979
	176445.0291		175875.2305	
17375366.81	7240709.548	9257209	8026141.384	1.236
	25910.98279	39737.41406		1.042
6768805.633	14173355.17	10290360.98	15147256.88	0.998
92310.45382	82759.17985			
8244866338	8537912923	10915244360	13845616849	1.174
	111300.2504	73323.57813	227602.3016	0.784
		30432.7207		0.63
5376686.077	4782041.037	4025195.25	2595245.893	1.5
982805252	1847003117	1216842306	978966390.5	0.6
445839.7191	178573.1565	658627.4844	567242.4414	1.027
1615797177	3342242651	2683771342	3157338714	1.035
		108563.5859	83602.60506	0.9
1237871084	1011094181	1132419443	1089919937	0.647
			215786.4305	1.103
	123750.9189	328470.6563	464962.7623	0.96
	1152467.572			0.765
109057.1044			447945.4688	1.355
5835385.923	5452219.694	4298524.25	6117272.15	0.914
14292816.03	5117069.07	20205513.63	3223860.942	0.939
2820980.382	1571073.341	1735831.969	2960185.055	1.36
479060.9992	334093.6193	288477.1875	325750.1075	0.478
11384862.53	39569943.14	24782730	26204308.7	0.686
32168805.17	56682184	17925972	22325701	0.674
	29732.32443			0.277
958394.7949	946717.9108	3237149.75		0.792
304862.8041	741712.1845	1355693.5	2049696.434	0.681
	455185.5414	695793.6875		
457460772.8	265794162.4	287788570.1	242190374.1	1.032
4275492.814	1669321.117	3822036.875	3553357.166	0.619
129849123.3	207441320.1	156798041.1	106928894.3	1.04
3583673.209	6048366.918	4170227	4657951.436	1.585
7136828.606	12448379.96	6456647.391	8285077.172	1.267
7789277.434	10640979.66	10293938.23	5101425.43	1.266

94287.91953	518502.3212	122477.1875	750319.605	0.189
328735.4883	304999.6415	335770		1.19
380527.9452	182221.7788	161065.5938	147074.88	1.145
922694.9052	3932904.563	1063389.875	376473.9149	1.802
	77239.21294		202229.9318	
	187491.281			1.145
24046120.5	18220252.6	21817157	13168851.64	1.096
1538839.772	2936183.146	1119963.656	1130610.584	1.012
			350892.1049	
113466658.3	120750833.3	93712389.34	62524664.57	1.178
				1.527
6413836.055	5554962.89	5850964.5	6099322.782	1.158
1468528.414	2342027.676	972942.0625	104341.8321	
7446209.298	7372234.945	5839638.79	6345784.478	1.09
39264974.31	79096864.85	42048893.27	106244924.2	0.801
599741.1388	2171738.337	663491.5625	1542334.925	0.887
527218.2594	2075004.684	1313607.5	1049275.35	0.871
	5500423.357	2566049.813	2957609.65	0.386
7789534.589	6320418.336	3536660.031	4867457.575	0.709
	100681.2089			1.758
593004.5617	660398.9663	710747.6875		0.359
2024054.672	787632.0486	890019.1875	991750.2209	1.476
386076971.7	509108401.7	387741775.5	353025748	0.992
1143483029	1266234889	1391336806	1088227814	1.199
37271.54872	71963.52104	70385.29688	107467.804	0.649
96270960.31	117269627.2	112623071.4	121946231.5	1.042
12834914.94	27326617.72	23877185.7	37971958.17	0.894
205619.4408	26128.84863	238116.875	172910.6913	1.439
893096958.9	690152235.7	508636920.7	494953090	1.037
2135481.328	6480181.165	2888156.912	3010049.967	0.787
367746858.9	400574464.1	448790868.5	376518174.3	0.995
26932849.99	18491922.28	16251291.34	11831538.37	1.26
4604492.153	14068768.99	7508975.656	9524402.039	0.726
7292658.323	7969060.383	14819028.41	10808413.91	0.942
	302875.6863	732392.8125	1792965.001	1.1
3446013.947	4070662.066	3831738.75	5871338.274	1.22
110583854.2	78925929.84	49477388	24811099.61	1.08
70408.40866	23693.47827	68951.80469	437652.9818	0.486
121227053.4	119506938.1	140704017.4	126979336.5	1.342
691452.2797			960211.3891	0.951
136208608	175284067.8	183607735	160399437	1.176
799996.0759	280744.1948			0.799
554417.9463	1045087.898	826340.4375	881426.0613	0.473
1179616.632	2372154.307	2569391.5	2631474.133	0.606
		632173.625		1.02
91264394.19	118703571.9	144513823.6	193805263.3	1.103
835353.2731	1631013.824	984278.25	1569213.589	1.022
1524330755	649006329.2	673826996.1	368959476.7	1.06
9017144.978	9914719.126	7311994.063	12951285.33	0.884
9450749.219	7075388.366	5734373.359	4149542.438	1.102

964364.4353	953798.9399	768099.6719	745055.702	1.778
979013.0244	3349513.069	1646578.281	2871303.214	0.716
33359884.83	59788482.89	42477232.51	43838748.92	0.441
327924.1347	1202034.515	312638.4219	687383.276	0.523
345271.2543	175744.011		1066535.235	0.773
10283537.78	29435214.97	46905256.63	84871478.2	1.12
5566532.731	10517415.67	6388235.672	10002063.18	0.975
448841.9641	2337202.613	3582976.063	3285744.615	0.505
62981.86739	50280.32653	166722.1094		
207073644.6	407237165	235020973.8	583192817.6	0.752
655874.6517	2381341.111	1339325.938	1835198.617	1.193
2229267.345	4081560.668	4193636.516	962352.5688	0.244
437384.8251				
479197343.7	282540160.8	129681676.8	137897381.1	0.276
12620444.27	26155480.78	18547735.38	17894674.44	0.982
9410508.609	15453527.54	8157527.156	3967690.632	0.768
11185247.94	1491792.909	15144576.95	13587658.7	1.058
		1137059.375	1047597.941	1.12
2663714	1070940.567	1660092.391	2273923.793	0.792
320012.6972	2889202.526	869834.2188	2053609.985	0.767
519676.6875	787106.3602	1343945.219	2043736.716	0.824
14470221.28	16280685.61	12739354.81	12928629.09	0.849
8146355.201	7258094.527	6608539.594	4883273.941	1.252
1432236.881	1829416.429	1467594.25	2180111.992	0.625
	2686319.303	1986590.25	3958999.852	0.945
84298.37095	79910.85459	134072.4063	198660.9366	1.702
14798233407	16083222243	13168910253	12433137460	1.185
2050144.789	696267.6062	794891.4063	690263.3954	0.255
804958.9138	1012548.134	2564960.375	2041276.404	1.027
16412.61367				0.128
347028878.2	675411783.9	548089002.1	621557319.7	0.958
		565497.5625	407142.5594	
715736.2938	469617.7616	475510.2188	400012.6924	
2576549.131	4586540.016	4583742.906	5180175.433	2.985
		1372079.25		1.635
19755287.01	19503385.04	14187885.5	17030235.95	1.898
	449533.2587	379249.5	932337.753	0.702
343552.4879	1163083.614	707944.3125	752528.2415	0.689
47553544.97	39582755.54	42819454	49787950.93	1.171
4027269.088	9188821.335	4603783.125	7287426.76	1.078
1608471.233	4024568.857	5263963.711	1355842.791	1.055
1208917778	1497873496	1658603200	461025879.7	1.296
3265743.614	3224356.406	2645374.328	3916007.587	1.019
593752.0102	1414999.785	466217.25	679825.7816	0.71
560843.1605	855738.0719	723853.5	1409347.871	
62731718.78	115940012.9	85665201.75	118833303.4	0.943
	123047.9363	274249.6875	543628.463	

1328376640	1050531845	1303084739	1019505941	1.213
			343126.687	0.827
3531093.917	1422774.116	3878240.625	1048839.919	1.571
2447006312	1842011419	2449559552	1984650844	1.219
94292.42569	77648.63718	101849.4219	156551.0607	0.677
	1499793.658	1653857.125	1956466.32	0.485
1459907.702	3036048.471	1815740.5	1469735.828	1.394
4442031.108	1369345.531	5605385.125	7829163.604	0.995
655072.1292	2394191.028	1501210.781	867159.0803	1.079
434916065.7	335372141.7	279087219.7	247422345.7	0.863
39374412.37	8034549.892	56393357.31	182586204.1	0.5
2253222.039	3288132.544	428384.875	6076456.162	1.453
355815.543	658050.6945	687204.625	812149.8968	0.731
84768885.87	61390539.82	93698536	49438910.64	1.265
113951.1589	10527.58941	1407853.375	1322252.079	0.957
114597351.3	245951731.4	125108910.4	383163992.1	0.799
202697.947	221712.5434		452657.4782	1.539
3445708.967	4516000.737	7941034.938	4745640.342	0.624
3074467.847	1255858.576	3227830.859	5718219.711	1.442
7892105.899	19436467.78	8715450.188	10455481.06	0.833
2306019.2	3517560.675	2499558.875	5078966.399	0.852
506409.2207	324798.7536	371298.1563	602851.361	1.193
1222159.409	2182201.525	933449.0488	2373590.349	0.903
2310091.409	6180660.105	3699422.188	3957424.508	1.488
	277420.1503	286668.4375	275536.6791	1.594
400597.7498	638667.6875	397723.25	1242925.85	
859026.2931		610365.375		0.208
126343337.6	68769745.9	99847189.25	165013028.7	1.284
205709.1144	143505.1411			0.882
9978455.244	10154829.1	10824897.41	10134779.26	1.52
54151677.93	52931000.83	79404307.09	98385044.58	0.914
34383376.45	97027755.73	57974194.25	83154297.64	0.9
45206289.13	57960447.38	54847023.44	238907848.8	0.554
			15822353.75	0.059
19119.70134			46672.64625	0.119
1353285.663	1541161.462	1430053.25		1.012
249376.3569	166926.0769			
				0.909
9137958.19	5116546.178	8933550.813	3242166.408	1.067
173400.5159	308783.805			2.494
260418044.3	318190749.7	411703412.8	302839621.5	1.421
20027271.22	13258753.86	22727853.16	18202022.2	0.99
	287405.0085	83262.96094		0.34
3478422.417	3791124.615	1095714.188	1201827.024	0.435
165172.8287	390609.1907	151624.8594	368975.0206	1.723
21645270.75	26514933.07	15272034.63	18159238.08	1.021
11430292.28	12077322.27	14012821.94	10155629.75	1.217
1734458.562	2760727.15	4667902.688	6421519.299	0.712
1543395.572	1769783.239		1377734.479	0.829

318908.6107	790508.044	425011.5938	555845.5688	0.97
1519355354	1001590279	1383616776	1490178093	1.256
41272973.43	71059295.97	63634678.33	207058158.1	0.536
9696833.116	9407316.68	8686303.625	4444931.885	1.255
13870567.97	10387397.33	11567692.94	5266299.849	0.987
3525551.639	6824066.987	3963414.953	5377450.452	0.788
1460165.711	1646156.212	5378540.5	131139.2365	0.842
182695.701	275342.3149			1.392
6322328.152	4582866.392	3078159.234	4284512.93	1.844
1836349.813	3033901.544	2081291.5	5986753.785	0.698
775699613.8	1008623416	732127479.3	316076747.8	1.043
8006732.43	13833219.75	9541025.125	13396009.05	0.933
12017571.51	10351818.87	14075742.74	10462921.03	1.226
136426.2161	484696.9029	358272.75	2661390.498	0.123
			1404953.789	1.372
4231994.265	1932784.669	3119576.922	3810791.716	1.626
2545460.824	3177397.102	3906163.063	1729646.044	1.495
13399609.31	13088158.83	21102932.25	21113127.25	0.898
3903458.306	11472580.54	8999570.938	8020152.121	0.939
2437623.033	11939675.25	16446456.28	17324072.06	0.613
245288.095	88009.39168			0.619
4607437.886	9995043.298	13039039.47	11478798.02	1.217
5652670.399	16519547.48	11612822.42	16484331.27	0.732
5060240.855	4800980.791	5469439.469	7519758.801	0.702
49355.29039	39669.9435	69388.71875	89059.12417	0.964
2457047.604	4119127.3	3736971.688	277705.5578	1.207
			1077539.047	
459495650	840561306.9	592601770.7	798461578.3	0.911
		422824.1563		
66012956.71	135147538.9	94607600.63	159845550.7	1.053
259694437.8	291043627.8	275560381.3	180024056.7	1.135
8907954.521	16580460.08	10390227.88	11734536.78	1.025
770513.7352	1447595.301	1366846.313	3615076.257	0.695
	699940.1335	582302.8125		0.963
35078847.74	33419028.37	30032172.16	25694362.28	1.044
1190004.925	1923911.307	1730842.313	4273555.061	0.197
8505072.387	1177022.267	9797253.25	4546030.764	1.385
1908132.02	2946931.477	2392532.5	2436420.567	
201372.0813	166742.3054	59930.12109	655213.6123	
59493608.28	88153330.78	66015737.7	97348345.76	0.998
70533052.49	88241470.53	26200588.71	8577757.657	0.898
205438759	411063696.3	304124911.1	390516525.1	1.387
1502292.028	3256610.991	2222592.125	1538440.317	0.842
2441168.77	534157.9562	3138159.75	8762608.102	1.931
1827375.059	2998166.76	2212943.656	1805844.624	1.071
867947.1752			715483.2565	1.539
7245049.9	2533225.34	6917668.766	3853105.218	0.964
938763.874	925401.4806			

479977.1307	2745685.279	612147.875	736685.074	1.204
87876.59461	281819.9765	210612.5625	2628417.724	1.407
13211803.99	9490255.49	8731743.25	5499589.716	1.13
921915.2169		605816.5625		1.381
7336232.498	8239020.409	6819465.5	5993118.26	0.897
4391.680387		14054.69141	54170.99705	0.449
	715466.3285		416187.607	0.404
3320296.534	2814828.424	2652228.688	414383.0199	1.075
111702.7564	57910.44013	902979.8125		2.027
7810398.151	4876440.206	4912315.672	7959343.438	0.92
1560482.82	3578984.754	1647102.063	2169912.807	0.788
3161871.25	4622323.319	4704319.875	2466827.053	1.432
1792978.718	524262.2406	467748.875	429728.8363	1.112
218034018	283762077.5	201119759.1	223636022.1	0.927
1193139.141	1458600.636	2685873.75	1576814.717	1.666
14896261.95	22884416.57	18441606.77	20213010.48	0.96
1277835.941	431845.8551	299384.5313	458063.5544	1.023
9284993.444	3094567.371	7527961.164	6907555.302	0.407
2045735.635	2892513.315	3475330	4055343.239	1.234
346074.0464	2908168.012	888696.3125	755310.6939	0.589
814194.5974	3400036.776	6665571.941	37728581.53	0.574
454666.972	222190.236	665381.3125		0.617
	240927.7067			0.63
3634469.458	4403689.16	2531619.75	1642780.86	1.071
		445457.5938	81951.30181	3.697
56836538.46	51759379.31	44607127.45	18511496.11	0.795
	54444.61153			0.244
214910027.7	200372532.2	220715417.8	452574253.6	0.251
368358.5866	1387828.935	558459.75	994729.743	0.593
2909663.365	909192.0004			
170155.1072	115044.3997	362977.2813		0.865
6882127.93	544200.2077	8264487.438	3671142.029	1.899
83550.16953	619793.8217	261617.1406	390074.4353	0.945
10140616.82	13059408.15	10785506.81	11120718.55	1.161
1271210.456	3297146.691	1603215	2240976.536	1.043
5436484.937	2429230.838	3069026.344	845526.3489	2.045
1951616.297	1207946.986	2229031	2979168.326	1.208
248628.3578	487870.2837	691502.125	398566.5486	0.956
132248.4267	179499.8407		270699.4977	
1615668.624	2700216.137	2009222.875	1268699.623	0.979
	255733.7348		948808.6176	0.69
	882305.7227	596593.8125		0.233
24314725.41	21550967.11	13808435.03	33714044.6	0.142
18064742.08	26243877.25	19695355.23	14114909.99	0.698
11720174472	11367420907	10505535594	10221759018	1.242
1002189.041	1919819.772	3480687.875	3420456.875	1.204
23614809.12	16727162.69	24277364.14	21113591.38	0.486
221146083.6	1619061459	3249509832	3178378644	1.023

2707332.479	1762716.665	2290464.75	2149853.363	0.964
185879.3046	788259.1794	768806.7422	1054664.033	0.643
	765085.4962			
	5806974.628	2171119.453	3978243.074	0.557
7788222.927	14794684.28	10346609.98	11769911.89	0.871
358734.5646			487420.7156	0.517
33606056.11	33370790.81	35850620.25	69747892.43	1.036
324917.5057	63147.13544	475296.0938	531586.4328	1.034
3036851.718	4070180.197	3717063.547	5726329.516	0.905
86906463.22	124709604.4	72038386.53	79364599.89	1.202
1687877.669		1366650	2324574.928	0.109
13140113.73	32302382.01	25039419.16	45310220.71	1.191
4976997.795	5778318.829	5569709.563	1940448.818	1.586
				0.872
1643252.617	3047744.418	3183767.844	5065966.306	1.002
2812525836	2640223363	2498039041	2187185560	1.151
88601179.55	112230921.2	91071125.88	110336521.6	1.175
407829.8016				0.97
2986213.758	1814932.056	4080665.781	246007.8972	1.135
	447981.6426	474355.6875	1462116.902	
1812867.782	34327222.34	718122.9688	43435365.97	0.489
3065856.159	2332759.472	1987776.938	363074.4585	0.199
15736183.07	24102096.54	13824890.25	28459051.17	1.17
1026110.333	770575.0543	1123242.039	558346.6244	1.333
512647.5812	1541522.35	684753.6875	1017162.618	0.885
	2782561.831	757781.5	3067260.843	0.324
16320315.48	23170528.77	22613984.58	24464010.63	0.944
3473040.063	963848.4075	1514989.906	5126591.09	0.844
11393601.01	7833057.716	7774448.828	1678352.036	0.288
3886141.301	3080517.772			
	576429.4148	246804.6563	449480.5662	0.664
68796.46961	6794.397113	31178.08008		0.48
1072125.276	2293185.485	2069118.938	1547951.907	1.375
209825.8628	318521.0423	751614.4297	786304.8665	0.106
289043.8875	94286.75231	212780.4531	785653.1361	0.955
808867.3341	3017587.507	653210.9375	1289244.755	1.704
4827839.727	7602046.361	8483810.5	13303706.88	0.908
	363472.0677	348161.3438		1.01
337941.393		325689.5625	1367224.951	
24067826.72	23153696.16	15328949.88	8612165.796	0.846
	320604.042	216250.7188	138856.7142	0.707
182714634.7	102934036.2	98498015.75	40424444.3	1.287
209640.0766	494221.691			0.805
175067.529	86627.66559	447978.7813	894967.931	0.997
19413432.45	37387230.34	5096906.625	39178771.28	0.399
835499.0882	999328.4444	929582.25	801268.9142	2.185
		545966.3125		

1506547.411	1835500.835	1349279.031	1056514.125	0.793
2846522.872	13216687.54	7870906.531	8717207.619	1.44
69211208.23	35122893.4	50228825.56	32193796.28	2.301
5283063.349	4423644.878	4406254.375	7525554.281	0.909
564567.3791	767160.3584	587396.0625		
20710035.74	48518019.09	34608806.64	22117083.12	1.162
27242277.62	38961501.03	48707018.2	76895812.54	0.971
133372.8077	96483.51528			0.868
271858.4037				
939486.6567	3021152.999	2338282.594	902496.8585	1.001
	717089.3542		573093.0156	
31065492.53	42140707.81	55347753.72	73507489.16	0.887
273264.7509	350682.0397			0.842
	260007.7491	971971.75		1.751
278817.9961	682541.2356	728302.75	1506182.374	1.011
228724.7443	354347.4525	338806.3203	168977.3203	0.046
15930022.66	29045107.01	21099769.38	18545507.77	1.008
493066076.6	405909524.2	661184371.9	192155636.3	1.046
4691207.733	7396817.149	5576103.563	7762637.482	1.808
1809161.678	307319.5441	346852.2813	835624.1923	1.317
170261022.3	154946154.7	128835019	79486316.21	0.867
358141.2356	471435.9914		115068.3174	
4523108.444		2833752.688	9267681.359	0.576
146532821.8	324130319.5	230696324.1	320245372.8	0.907
42614009.23	49465341.95	56128427.49	65687814.35	1.206
226847.7653	869136.5421	631067.125	724951.1622	1.111
866625.2426	1265646.525	1487774.188	2229030.066	1.045
1362235.905	2329185.83	1825527.914	5246428.458	0.858
11670087.08	11501757.92	9626212.953	10620437.89	1.09
2822972.058	3429442.054	3645796.219	4372070.209	0.786
15760577077	16557078636	14010133651	12982442764	1.269
8025623.544	2155832.866	3673795.625	3693078.868	0.792
3636688.89	8824659.485	8994660.844	21533924.57	0.754
2862930.319	7976471.117	2576703.781	9902471.859	1.174
263463.7191		356218.4375	818967.8027	0.22
1874338.874	3163029.965	4232101.875	2217779.315	0.85
9140899.203	9352594.226	8720441.875	10399258.43	0.85
29099.32599	120097.328	78180.61719	190795.5261	0.948
80294114.87	52762373.86	57023641.5	66320343.76	0.503
589732.6217	1275369.565	433361.4375	1475449.348	0.892
972452.7379	4689214.263	5816798.5	1960023.719	0.875
880604.2323	761565.9113	1210312.75	736459.7071	0.772
121327094.1	148407196.7	121533097.5	88328449.98	1.069
	150767.8311		269010.2738	2.819
2921423.537	3874361.789	3068093.406	4335525.238	1.137
74359040.94	111432306	80952368.59	150269190.6	0.86

11345793.7	7109747.634	19761415.75	12999747.96	1.284
351152.2464	253929.0259	516919.0313		0.9
1190282680	1394615648	1485504517	1273990056	1.185
8248522.929	6163044.854	7518261	13804610.21	1.076
	193177.5077		186666.2652	0.385
215971740.5	185262054.8	286806491.6	201881532.1	1.362
	1211188.153			
170593.8785		279479.9688	185553.4022	
			887387.0605	0.112
	3241.62482		11526.94698	5.399
156755.501	1224312.683	1329671.156	1411255.198	0.483
3268634.778	5563147.747	7045757.594	43017857.87	0.525
15052518	17608457.58	14262939.83	17554053.8	1.228
772516040.8	1315900466	1222760494	2275057490	0.992
	1209481.648	547516.375	856419.3313	0.465
2082050.631	3442360.744	2834332.906	1147540.441	1.802
17570703.54	18765935.65	19415441.13	3943834.589	1.194
8170.390342	51664.73159		230929.7672	0.969
961925.3725	1780835.862	1420148.625	1815389.787	1.135
26638563.04	53247103.74	44219838.66	56354682.68	0.881
141685001	123781783.8	126590220.8	157656329.6	0.847
4384307.417	2638046.159	2425123.938	2991286.973	0.349
1629613.071	1007389.02	4474341	5829020.121	1.269
809632.3352	466620.5379	340186.4375		0.704
2441231.985	2754742.113	1976776.813	1065082.687	0.958
	19043.10319		3658398.652	
		6605.947754	337221.1278	
76504681.77	225294893.3	137431626.1	189474873.8	0.875
1460726.317	1223036.742	685244.9375	1789514.858	1.257
3958409.723	3483657.827	3709075.375	5940811.727	1.163
6051746.305	9235133.914	6654459.75	8103337.817	1.329
691696.983	796952.3634			
577572.9271	912790.6935	654165.4375	969702.6201	1.59
8130509.286	10783170.36	8726320.401	6348387.526	0.925
114712.1596	1301345.149	1480228.875	1008533.215	0.949
318818.5551	1089427.582	2267262.563	2415122.229	1.012
8739490.536	17053045.9	9991610.688	7867408.433	0.504
2627235.681		2497117	2216074.232	1.507
3276237.874	1319531.92	2271229.906	1510255.057	1.23
105930.6874	1096175.954	297858.4063	1530824.003	0.193
30898714.54	52247102.22	24931701.28	26823728.37	1.066
5133509.486	2871360.098	3999249.297	7661623.63	1.233
9188244.939	4528583.651	8272749.375	6691841.342	0.827
	3534109.205	3911837.125	667996.4276	1.243
11940.00827	4697527.887	4987973.969	3972221.112	1.194
	2313693.596	1677912.625	1532660.427	
643002.9174	1086652.604	588317.25	713360.4389	0.618
107753.6459		1327255.344	835704.0111	

9897518.936	474092.8379		2414291.327	
47121093.04	64729402.29	64667838.88	88556621.97	0.991
	188601.2544	1235509.719		0.555
	1828099.821	1756048		
664499.3188	427383.7688	614172.3438	279565.7282	0.994
1317731613	1140452193	1314523005	1614115363	1.604
3479620.779	4036081.632	2557196.938	3822378.074	0.348
519320.0159	595086.5386		791891.4115	0.872
2287190.779	4070217.044	5140773.688	7269691.114	1.071
752694.0636	894815.7753	792037.4375	2659355.859	
63182100.69	75434874.49	73768545.08	53118879.67	1.135
7558275.467	4997241.745	7186923.813	3269016.463	1.215
252349.9019		333044.0313		
2701647.864	6380873.101	5946090.313	8999734.432	0.823
	164685.2647	223842.75	193846.8279	
49019478.88	79309038.22	75568532.5	36364997.25	1.186
	4855837.658		388200.0067	
454360.2838	1169195.768		382049.7747	0.709
2934740.597	4207373.565	7038122.969	9098037.46	1.252
652934.9535	1933080.307	1275947.969	3418288.182	1.847
222607.8674	219261.4664			0.696
	723253.5566	232823229.4	323542108.5	0.008
687058.9478	2661637.717	1840880.75	2218338.451	0.868
78857.95934	19419.65421	157365.8281		1.933
281695997.2	120465399.1	119483510.8	152360855.4	1.158
147840241.5	199247344.8	385842120.1	263940673.4	1.552
13054818.05	15823255.48	15290884.94	26732756.55	1.072
11663814.73	19753226.52	16551424.78	20315388.55	0.828
69386038.41	108096692.5	65217665.31	70965731.89	1.064
3714672.285	5952352.689	4488009.25	3590795.582	1.319
797687.1269	388097.7297	789020.4375	412640.7575	1.291
	738731.8443			
37123128.36	36400906.2	27336161.06	20467296.16	0.997
3835558.46	5804613.795	3881631.266	4404963.189	0.948
4508604.246	2002035.176	1782653.125	4318720.486	0.963
4005195.983	9082611.503	4528132.328	7580031.041	1
1893487.131	3346915.066	2977632.125	1495329.84	22.567
516579.5549	662578.5272		360841.3169	0.827
125420.8837	154655.4015	116434.625	184038.0236	
243915.0777	1486695.586	2690291.25	1115159.281	0.53
6336777.543	8376706.096	7724242.438	6796203.256	1.321
303128119.5	483920330.4	491148917.9	487796303.3	1.082
84205.45539	36245.44756	154453.3125		0.265
				0.401
29117483.23	80654990.21	49343206.88	74251848.04	0.927
88590485.06	48786827.99	56175823.34	33517963.22	1.063
2817045.375	3461613.78	1295518.625	742200.9685	1.539
4471466.365	5064706.231	6227853.094	4172197.04	1.23

187942.6176	190490.8939	264282.1875	343020.6439	1.366
4653621.237	5847169.477	1249917.367	282250.0588	0.977
47986685.27	69940027.98	63779888.31	34019375.22	0.911
28066550.95	9379952.4	24285351.29	12617541.28	1.234
11044298.31	22029170.88	11744489.13	6660514.428	0.707
4215161.677	4723406.235	5463323.938	5392572.478	1.013
30022271.56	37727113	25867310	20908660.05	1.21
	1680339.867	1409646	546727.0055	0.88
				2.15
4956014.376	7865950.139	10994130.86	6824410.122	1.07
38928977.17	65044953.76	44908248.5	16155582.63	0.725
1030705.2	1076378.656	833829.875	114197.7255	1.194
277319.5481		350395.6875	997073.276	0.367
453210.2088	47062.16204	408025.6133	315881.0852	1.891
79036112.9	101445164.3	96536779.89	87065241.65	1.17
160182.2514	1854349.411	759336.1875	652182.9899	1.748
990412.6284	2031026.154	2301244.75	232030.9169	
824738.4591			202336.0255	
2413702.916	5009126.79	2732210.469	3279332.781	0.832
7206604.934	5821731.939	6723855.188	7008528.436	1.07
30321825.17	24891289.8	24327489.22	22308048.22	1.21
	8266.322146			2.945
	2613125.16		2080947.052	0.681
5758870.815	847430.4469		4972109.371	1.371
1277754.695	1774065.483	774070.8125	1529701.129	0.987
3643112.644		1572402.5	1185955.374	0.563
2375626.182	1254190.539	3365010.172	2512180.531	1.69
43846224.32	84753477.34	21761830.66	32299572.62	1.404
431102.9538	2615690.316	1590388.625	2149920.677	0.876
337972773.8	452782769.3	523831885.3	260491159.7	0.592
43197095.94	65026078.47	55662377.55	40967572.93	0.981
837711.846			683084.8214	
337312.6665	122426.9642			0.867
234304.6529	170852.688	310750	453429.5774	
678182.071	1102261.5	528111.625	2720944.132	0.579
617308.668		196515.4844	246913.6626	1.085
3114190.721	1065044.872	1979439	4268740.986	1.451
945998.9824	235154.0718	522570.25	397772.3037	1.318
1407379.023	10805204.45	6019687.25	7232517.418	0.917
1109112.959	1271364.961	883757.5	853867.2513	1
25249926.75	47249968.71	47909229.63	31859626.06	0.824
70189289.95	121637578.8	97943723.6	95026103.49	1.138
3096696.054	4846078.373		2946030.774	0.369
308687.3492				1.6
1678901.274	363632.797	889225.8125	18013493.88	2.06
209882.937	98010.59366	259968.3438	213212.1543	1.074
4452728.381	8615531.651	6083942.25	5792833.458	0.872
	210997.0235	129234.7656		1.204

988282.5547	1335107.661	1415319.406	1354448.472	0.958
368783.6933		311827.9063		1.28
11878465.29	5511383.953	4192479.188	9869242.944	0.267
3055570.17	10084209	4458722	6768739.163	1.108
3613880.94	3023882.742	4762029.5	4344340.773	1.04
722157.5637	832006.6146	1659243.5	2292702.63	0.949
334366.4282		431154.9688	152107.3782	2.124
2340356.411	5196202.692	2242208.25	1341273.439	0.823
54605795.77	16819859.35	61808040.13	71979748.85	1.285
91165.73892	88614.17047			0.804
				5.561
3883261.026	2696239.424	2236216.313	463833.0288	1.765
245595.0754	297309.6615		423343.6645	0.492
588259.907	68018.32301	870569.125	422328.6032	
6612264.82	7596072.159	6236454	8915268.954	0.231
404764.1783	529073.1104	367267.9063	1034852.675	0.543
	908413.2369	538880.375	549775.3916	0.177
3539409.356	3909959.355	3485535.109	2638879.489	1.199
505922.2863	431539.6873			2.233
148018.5959	41515.01167	441145	1369148.423	0.551
2042793.981			3047251.118	0.188
543884.8948	405982.3106	643584.8125	395394.6421	1.517
35436185.36	38378984.72	26777581.25	27113190.7	0.908
		488364.3438		
2374922510	2816075780	3014435454	1596400918	0.9
10849052.77	9858515.828	13669055.32	5475808.988	0.831
1434379.271	1650693.584	2105140.563	2755810.345	0.504
82147.45201	65059.52514	202729.9844	214454.5038	1.118
2721530.006	9228247.899	6709197.531	6399822.355	1.14
		355681.6875	442663.9748	1.232
1771244.895	72490.69602		374583.4416	
			2802657.814	
330752.2901				2.089
1323492.162	1231348.933	370031.0625	199728.6951	1.033
6196053.099	6628462.418	2906309	1924910.87	1.275
1219864.322	3071608.58	2182436.688	1367638.674	1.046
412687.863	341593.8461	852999.6875	1053115.487	1.241
4368435.157	10257245.21	11848468.56	22475461	1.039
	102639.0636			0.785
	282541.7362	149937.1719	123775.0787	1.304
883062.3226	2888023.797			0.998
	235131.5958		162338.1809	0.81
330549.5694	289483.074	617990.6875		1.206
2009214.209	350981.4116	3437454.625	6095244.2	0.502
	1628652.262			

8297484.06	7295624.918	5393431.57	5948093.445	1.2
33381104.65	63662696.78	43341110.38	63237707.83	0.723
2769515.672	7298267.709	4338672.953	3027737.462	1.255
241766349	354291872.2	313388417.5	187287090.5	0.981
2647514.05	3281037.945	4125565	1664670.879	1.662
27037234.39	35445783.4	33705427.88	31944317.68	0.877
5828196.865	725787.2037	826362.5938	3006053.382	0.875
47616693.68	21657285.71	51351795.64	48500688.13	1.152
			1149999.944	0.726
1452128.979		1640331		
18867947.52	16809456.05	15353819.72	21987341.15	1.153
1185622.111	2557587.096	1237097.375	2178057.095	0.645
5080510.88	2224196.601	15094772	40125743.79	0.836
	508431.4528	281622.8125		
42444650.75	93024485.98	79531423.88	84531831.15	0.208
3998567.807	4641928.076	2461463	3470240.653	0.474
	330197.1117	948125		1.192
			47757.06873	
49556028.35	45792819.36	46517852.47	60080580.79	0.906
		546082.625	389714.4752	
300307.0708	348444.7847	319853.9375	576377.1168	1.227
				5.825
396195.1583	1101501.142	1778031.625		1.048
1790624.786	2604735.62	3583397.063	6861931.475	0.903
946281.6228	4499973.277	2933967.25	5337737.055	0.289
32851696.99	61025727.66	50397231.22	90416122.87	0.773
4147655.035	4713971.984	8901212.5	6052948.934	1.102
62716489.4	45810562.16	83363481.89	61040243.34	1.069
9367418.671	11971588.62	11385053.95	10925900.59	0.957
				2.017
409966.82	560716.7052	718006.8438	1852056.896	0.638
2804944.114		1087167.5		
3499058.104	1162566.774	1656974.281	1892148.093	1.032
	1481083.477		913539.6024	
9797872.289	5400991.41	4133559.617	2858691.632	0.78
4746.908414		12301.0166	11927.48016	1.161
6779996.628	7477437.19	5558330.313	9396302.037	1.025
3451956.34	6293142.694	5497830.75	3861129.541	1.317
24380450.99	56985637.02	31133706.62	33241911.02	0.887
605147.8048	648106.2397	341343.5625	459758.5254	1.398
627633.5824	283539.0823	250249.2031		0.865
1476602601	1214109130	1454119680	1174346557	0.723
19279821.49	12533666.82	14506171.25	5955905.177	1.113
	492938.3346		321644.4249	
420240.4024	1506648.49	2833015.547	934038.1257	0.869
	409427.9286	2638100.031		
1228235.453				1.164
284994.3493	158283.3486			1.675

610613.0846				0.675
1423309.818				
3179110.448	2835556.755	2672070.172	1795946.95	0.492
28697831.31	20390170.32	18768608.69	21039488.52	0.972
471702.348		466455.875		1.927
	1028227.094	414407.0547	662384.7369	0.2
2326492.289	1001679.442	3174180.625	2894221.104	1.006
	374384.8092		1121353.197	
	110470.6949		664470.2406	
381550.359	613336.9016	738684.1875		1.627
2221899.165	2178291.532	2831440.016		0.979
208222.3798			224586.8981	0.682
287640.5744	3241447.72	1200188.844	605366.5315	0.285
384276.7959	747601.1545	562049.9844		
	516453.7931	466338.6875	373034.086	0.517
6407879.178	7421888.263	11181641.21	28247053.61	1.066
5040089.451	4559959.375	8659900.781	1856377.21	1.151
	439159.3018	210839.2188	553467.2499	
47459272.22	29386163.56	32976843.97	40802415.66	1.063
569708.404	1586466.415	1544355.406	2143111.703	0.576
2567117.694	5858249.991	5330996.5	6612513.867	1.707
2269078.32	5747335.494	7680328.891	7543854.153	1.374
43685926.66	56331419.21	79864023.91	94475743.19	1.14
1169860.325		1195747	677420.8508	1.26
334266.259	484845.6996	339699.875		1.525
	869542.3826			1.146
169406.9619	163458.8944	231362.1563	342783.3783	1.261
	543573.3954			
345694.5855	367622.5707		780652.9968	0.537
6955240.992	9113456.628	4359592.313	6758622.257	0.822
396089.9997	12680927.81	511013.2891	377429.1121	2.188
707783.9765	2349303.746	1058670.375	484317.7237	1.243
719667.5932	2041739.975	1244364.5	1258223.532	1.032
791025.9183	2434961.68	88526.73438	713071.4347	0.452
1316351.469	5242302.929	3268358.594	3826378.32	1.053
		201558.0625		
1446282.755	972281.8267	1069137.125	1009109.087	1.04
30050746.84	14728888.76	15283256.88	19232863.47	1.25
523595.4461				1.319
				2.226
	44718.04917	36829.85156	435670.4033	0.516
542518.5524	871917.183	606090.25	1023657.81	0.826
	95503.22962	182558.7813	231934.295	
1717227.499	1944867.91	2062341.625	1775619.365	0.863
624689.0521	127946.9135	1052312.125		2.016
	90536.93093	160783.3125		
10447230.84	12447028.97	2770779.891	2999212.399	1.353
328722.071	1260778.032	1470200.75	197312.9653	0.999

666237.0537	1923308.669	937930.582	192754.1892	0.174
260617.8073	209155.1498	371110.1875		0.693
	375392.4729	543842.1875		
				0.689
13126067.09	26279893.57	37853428.19	28281885.02	0.889
				2.056
348714864.3	576130044.3	334365514.5	334860428.4	1.161
	1584349.778	975271.5625	238554.5583	0.757
107367.8921				
1265601.878	880363.5569	1595851.938	1566783.934	1.941
2409909.42	5251168.771	1775364.281	5714622.435	1.242
1077399.432	93108.22741	1425149.125	4151232.522	0.635
22081.74913	7349.65812	71383.10938		2.141
2422870.772	2175761.488	2843695.563	1158659.346	0.981
12623961.57	14310640.51	18215830.25	13213156.23	1.93
1887519.185	1092242.423	1656282.078	632845.7417	1.432
178992.395	131942.9433			1.118
	1026616.97	889667.4375	1015429.457	
		757116.5	9331808.937	0.586
24536627.87	212747372.2	158824250.9	69023464.17	5.136
		486267.5		
943664.0528	9046.798455	1055905.453		2.058
1221181.658	603625.6122	2066858.375	3493817.149	1.932
11288023.07	13038411.59	8504741.5	8389036.89	1.031
13916394.76	10894865.38	15701267.55	11102167	0.833
769841.6552	1390164.164	1173584.625	791443.7787	0.532
37895208.66	54268195.1	23636822.3	28472415.08	0.923
273742008.5	308821638.6	323142860.9	163826825.3	0.983
654310.2408	2468815.346	1325374.063	2092600.15	0.698
721095.9879	938221.4529	1163429.375	216354.3815	1.615
215199.3451	363179.5978	111917.7109	479973.326	0.698
1472911.408	876550.334	1076203.625	627545.1862	0.381
216693.7702	765934.3067	1348365.719		0.794
329269.1248	2074404.259	3006708.75	5291232.086	1.05
3498999239	1986025388	1586184106	694481573.6	1.494
11278376.91	5616.980527		22914995.19	
10823991.12	11000301.88	20363356.28	39545811.4	1.069
1420963.472	2718263.969	1065367	535762.9007	1.004
551770.3053	1126017.079	739302	600267.6973	1.057
646648.5732	1077435.893	1203941.836	1218829.958	0.779
684941.5272	4306489.619		2720025.271	
11957986.71	10880053.18	13303149.72	8592483.874	1.236
1417546.211	2160215.815	1886165.797	2122218.543	0.293

679680.2943	504357.474	957865.0625	1394319.881	0.991
	456681.7641			
1705768.877	5140781.007	3552879.25	1030817.306	0.8
1185381.993	690297.3868	3386443.125	2631323.596	0.993
136530.824				1.286
504511.9612				
	862879.5337	441128.2813	210910.2329	1.174
	234336.4267			
	1428236.717		729968.6944	
55696.92725	28474.64056	93406.28906		1.023
19931063.19	63158083.92	27309331.28	28504310.04	0.88
	185934.0725			1.01
30952500.48	18318750.12	15494571.19	30312571.32	1.074
28838.14322	8461.47648			1.401
2292398.702	1674195.451	1820789.875		1.41
	158484.7823	424679.7813	619594.3109	0.836
1072715.918	2580901.652	691038.75	1264619.794	1.778
99015.68452	1626054.543	1127827.727	564599.5203	1.157
46385.54084		191008.2813	730737.3554	0.491
3080861.855	8841301.148	6219298.563	10376903.23	1.093
165196.1461	328723.7896	281793.5938	525173.1491	1.431
8011177.734	9204558.19	7553947.625	4561663.227	0.914
	334676.5416	1393814	2829056.554	
236344.3788	522659.0184			0.912
28934302.24	22818192.68	26755900	14994295.5	1.471
3081428.889	5493946.191	4059737.25	2704930.675	0.805
593590.9686		318956.3438	365541.3601	0.445
4836517.614	6060626.515	8204732.125	4781232.34	1.193
	335091.691			1.279
975897.5735	642654.693	967114.5293	1243277.571	0.815
66715862.63	82292561.74	64890204.17	160534124.7	2.315
1927774.448	618825.6942	2106327	5171589.243	1.11
739461.9493	290690.9326	329008.2813		1.156
2205109.751	2717169.215	2565329.313	1776088.166	1.081
734102.345	201333.6238	500950.375	589403.7706	1.465
16265757.91	24985470.21	16487757.76	20523707.49	0.922
	132674.3924		667487.3127	
56558.81073	2722.189762	39244.87891	249324.5596	0.321
580880.0961	1440853.176	1778424.828	1516208.337	1.182
117149726	322971897.7	191376610.5	322022827.5	0.852
298638.9397	501254.3481	689406.25	753028.3228	1.665
361395.5106	596664.17	389357.6875	211980.7764	1.037
4050306.077	2355743.455	3475680.203	1684705.699	1.562
22037.61467	10274.56626	58543.15625	113235.6015	0.666
335912.1627	440381.637	149927.0469		
7211751.715	19040479.67	35408559.94	40400017.8	0.976
45724549	26019554.78	45243997.02	54338619.45	1.254

7585286.356	16783358.31	9534441.906	14080772.18	0.823
1414792.58	1303753.623	2179475.75		
167021093.9	144960947.6	119577563.9	46181898.41	1.409
2066914.391	2991879.497	2951329.188	800928.6222	0.92
114287.1427	116559.9216	314664.9063	485781.1256	1.238
298052.0159				0.848
			3507.229743	
895417.1415	554010.0017	733527	962336.1281	1.459
7753290.319	1462826.389	8103951.5	15860114.41	1.064
712403.9533	1484055.86	714677.3945	2316076.933	1.671
343946.9618	1099638.934	824856.4688	659828.5447	0.479
6490450.006	17441975.9	9078633.242	15788201.19	0.899
43932528.77	65075454.68	44195184.13	61263570.39	0.966
19567873.84	8154487.933	15562886.28	11958803.64	0.93
	710382.049	506775.6055	175162.6753	0.547
39467.1668	91784.90106	85857.92969	153499.9612	
694074398.6	835576441.4	726483732.7	392077598.1	0.737
889104509.8	650554349.6	901500708.8	506211317.6	0.787
139908.5632	203017.0513	122541.8984	147635.3311	1.029
84785134.17	62464644.05	94818585.06	57886648.67	1.257
	2808929.57		852852.3922	
1695204.136		1333338.813		1.076
89052132.82	111222527	108089024.3	76578311.75	1.188
301091.1192	99558.87372	195723.6719	1224638.365	1.747
452327.0531	888598.374		972776.8597	
3153180.721	4439527.148	2458256.5	1722007.753	1.559
14710.79875		32814.5	75347.30231	0.8
7480398.714	6247730.431	9301041.641	13666843.58	0.688
825406368.5	1150991862	756401238.9	835790127	1.168
1912431.318	1399560.72	2367827.5	973144.2692	1.447
13899626.88	7013241.231	22340209.45	18488828.2	0.792
2814390.587	1391576.5	1031079.531	2691331.941	1.581
317034569.9	257333677.7	203958352.1	119580837.7	1.302
527536.9939		628015.8125	639981.4906	
44862497.39	24639230.76	42561038.63	38378161.34	1.87
605163.7618	690283.6358	631733.3438	967773.9565	0.764
20819531.86	18484744.29	15246876.13	12176763.33	1.049
479366790	164657618.2	1187833088	517990062.4	1.395
10591983.17	5824682.618	8305053.375	9813551.221	1.08
1310426.809	865890.6676	1263014	1819510.975	0.599
447387.9933	493059.3151	594809.9375		0.947
2548526.56	3981635.649	1329227	3595616.264	0.919
5480636.169	8745135.074	8558494.469	5500004.476	0.926
	146417.0802			
1399618.819	1102570.924	1061220.656	1907589.604	
5343370.303	4649583.829	2395000.25	29268203.4	3.845
1702278.214	2762861.33	2544174.75	5446073.496	0.997
43326606.55	48252043.79	56546849.63	67410951.28	1.07

469905878.9	434473262.2	385365455.2	328524887.4	1.059
4532063.128	9166219.732	8879619.078	14258755.97	0.87
958407.7403	2877020006	3365240500	2450808805	1.081
2447231.446	1186250.271	1983081.813	772576.0562	1.341
17308165.83	40062929.61	32261643.74	43420791.35	1.333
	792332.875	416482.1406	619905.4292	1.127
18358106.08	29550171.5	20722616.82	23327081.16	1.193
394182.5143	32688.82383	703979.5313	255287.9015	1.859
318234.1541	262358.0915	193661.7969		2.409
1700912.254	2844092.254	736221.875	1579013.478	0.665
2222006.009	2661374.448	1102919.75	4575192.153	0.993
33141577.03	32813141.51	24457529.62	1042513914	0.199
480617.5665	742960.4998	613087.75	421319.3733	0.644
2448773.686	7120007.6	2666926.5	4421473.735	1.002
999027.946	300315.2738	649560.5313	932747.9876	0.676
9605512.156	20664471.38	13259380.64	39008994.67	0.933
12668535.64	15433216.79	19829963.75	16234711.81	0.897
	1151800.161	294083.2188		
3239479.521	10396089.31	2277496.75	5228544.719	4.241
				0.971
1550004.269	2643084.389	1972035.625	2987442.252	1.768
4871466.473	17715890.67	6085231.219	10747143.46	0.692
14241449.75	25020780.3	18288561.69	16892461.66	1
101884.8687		173311.5938		0.655
31297870.58	39793355.09	15071369.59	21345747.35	1.062
	157884.9942		367139.7935	0.992
133992.3421	822931.8181	1265530.234	1877618.229	0.413
	180215.5655	469499.0313		
8639294.52	4285861.27	3481409.813	3031901.594	0.864
166483.7713		303507.5625	497980.5014	1.066
15839223.65	12025784.35	8966129.25	7556959.109	1.083
144804.2133	433459.8727		89587.93255	1.186
35736345.65	26854087.39	65136779.17	45275240.9	1.123
3597185.065	4520506.13	3861115.375	3833875.192	1.04
38178945.56	6244343.587	31293316.39	72155178.92	0.552
221581.9475	361135.6356	510862.75	943525.0001	1.5
53196226.89	89177663.74	76082205.8	81036727.93	1.278
7812338.764	1648713.082	12067719.88	7670913.679	0.581
117308959.7	203534643.9	173189078.2	98159063.19	0.944
1761513.4	2623104.603		3785446.143	0.656
69044750.6	42922141.64	72027910.53	13226077.11	1.194
14426670.18	26957721.02	22781282.59	14944192.74	1.165
1824500.762	474406.3679	609088.4375		1.612
450248.2214	656484.3319	440412.875	1043488.449	0.93
2967347.377	2727809.786	516242.6094	1802560.725	1.547
			737560.1826	
108890.7476		184002	419933.7003	1.572
5413031.089	1643742.424	1705921.906	2155056.926	0.652

30169996.26	34993400.41	43972565.63	21382945.47	0.565
6003141.445	6781678.919	7531999.832	4813569.192	1.976
21846897.02	7796075.988	8829407.938	12124877.3	1.613
888576.0801	14502497.1	949633.3438	12591956.46	1.208
52792777.73	59379258.27	41909807.75	38919405.55	0.886
46117249.66	40071861.21	78033149.63	41684823.59	1.382
2958080.274	3092288.491	6564492.375	1016843.14	1.191
1820031.815	5256777.035	2715799.438	3490007.552	0.817
2879207.515	4777607.886	2089242.5	3269112.293	0.982
631375.1963				
61006678.99	81681922.95	89279107.22	49664735.23	0.84
3963528.94	1320230.549	3448888.875	2712478.479	2.237
	1878624.98	3373824.125	588588.5935	
5403711.777	1474769.306	8351826.344	900571.5655	1.957
1098940.142		607933.1719	498605.4683	1.409
	3770282.94	1088487.125	1325113.895	0.544
1768559.047	2823124.643	2173685.75	3122689.522	0.37
868686.5553	109868.1726	195769.6719	1146458.791	
100041725.3	235998294.1	107566043.5	248239067.5	0.595
1168997167	2034296355	1561855992	772620155.7	1.105
5819732.531	9169397.099	4626126.5	10279134.27	0.563
87583.89123	140985.6276	113801.1797	1115420.579	1.55
15409174.62	13553879.49	14041605.86	7740802.933	0.904
16044971823	18991416015	16598847315	11479080947	1.283
11114024.1	2053792.426	1457294.781	1576561.036	3.28
3008601.276	2692418.166	3398688.656	693174.6265	1.907
1562204.103	3008608.755	2040614.375	1057702.307	0.803
136236097.3	232162976.2	153735747.4	127317014.9	1
8977451.782	14222521.12	9510611.156	10734705.5	0.213
	573155.2242			
464211583.2	464144923.7	463621712.7	278832127.4	0.877
571527092.8	523424897.9	371915904.6	483444409.9	1.188
1594100.282	2363643.797	1808811.313	1031761.987	0.89
218401948.5	155165976.5	176872733.6	115136838.1	0.861
417671.0292	796847.5231	869150.875	905919.2649	
580043.0638	160486.5358	268630.375	922370.3054	0.887
	41607.5124			0.829
244771.8763	394168.8783	486670.1875		0.426
9087738.549	18679619.14	14091483.13	22102535.08	0.895
55493935.58	52924345.36	37674895.22	19728555.25	1.047
1160072.753	2994622.199	1586557.328	5867981.045	1.275
8236656.483	4443805.993	5028046	5274055.812	1.45
100527490.4	172821182.7	158502708	79276327.47	0.944
9527698.827	2679728.59	2667544.625	959496.5249	0.379
510635389.3	670430876.7	617674165.9	463276314	0.941
17262100.11	20485604.4	27844308.11	21380071.09	0.947
21928843.79	17182281.53	19688514.66	23057619.7	0.767

2000765.608	3012193.502	1439759.344	849363.2159	0.87
1443257.625	1307106.214	2306607.023	1238830.903	0.882
2686686.308	6644686.251	4175830.469	8767462.44	0.775
41074616	67958391.49	57149123.94	56622237.95	0.971
28829994.86	22341067.82	32403070.92	13264920.95	1.394
29050665.12	65357928.42	66205276.8	98953330.13	1.043
6211135.518	6123655.532	4107355.867	3668091.756	1.113
4312760.778	2736059.505	4126675.34	3524388.043	1.091
9454537.849	24270354.94	13249913.16	17607444.6	0.808
58270755.68	57415408.4	106397707.6	94389400.75	0.828
1287802.992		489278.5313	1052320.467	0.761
		638756.6875	1058686.425	1.229
456124.9209	984427.7343	1320274.188	1516596.283	1.115
870947878.7	1387528572	566896705.1	924860913.9	0.915
	555484.3679			
4163786.618	12916398.68	11827581.53	11628049.97	1.205
10870849.71	6626775.185	5670876.938	10092528.02	0.376
88913.78548	301338.0159		1049111.263	
329985.7944		399083.5	581339.1668	1.426
5875123.484	868838.7275	818260.625	820657.5491	0.519
1701289.243	4337105.941	5105123.969	4719123.997	1.043
262063.1929	187636.4541	393696.5625	558790.7095	1.216
11849910.82	12159099.08	7103641.109	10908234.44	1.289
50156177.6	48836626.14	42761437.36	29185126.17	1.145
1743678.693	752512.8164	2481364.891	6085390.077	0.611
51543039.6	51056475.98	65915533.14	25551785.94	1.269
2045451.556	1212890.942	2527758.75	1445024.745	
1219869.244	951141.5934	1777760.313	3102721.316	0.473
632192.0126		269465.5625	418019.5308	0.543
941468.7353	1114393.391	1283427	415106.6143	
1672289826	1795762456	1862228950	2164069944	1.094
437258369	436772079.9	526457012.5	583845585.9	1.146
1378483.668	1821732.946	1839968.406	3620148.429	0.416
7297200.807	6238881.579	11220294.02	8673685.711	1.359
14802248.49	21051337.11	31472168.22	34367455.31	0.854
1570600927	2503128254	3058443074	4773378365	1.11
63612988.94	30190161.84	37220027.11	9471438.825	1.202
	4177353.482		659175.6345	1.477
590801.1084		1428207.625	9042227.324	0.69
19667804208	23820743039	18649028792	23750591925	1.071
719746.4116	508617.2507	812713.1406	2077574.216	1.392
1483715.435	845454.7442	398130.1875	1218521.907	0.263
132805.3918	259877.2119	887186.8125	5888397.196	0.635
	181287.9316	164436.5313	495448.0728	0.454
4220083.831	3501159.097	3373575.688	4079137.004	1.471
781290.1415	229501.1165	214421.7813	249390.39	0.4
11608587.86	12641498.1	20411615.56	32787091.56	1.256
1545517063	1728548341	1410796351	1298453560	1.119

	1077094.717	814383.6875	650518.3892	0.642
1122296.146	138686.0595	1116093.469	3005782.119	0.99
196763.5103	918740.9589	1483701.719	2323511.261	1.195
7853851.694	11385518.92	7348680	16968012.13	1.205
	5258956.547	3217734.25		
4319365298	4846127142	4647080024	5521141095	1.04
1909436.265	2381304.371	1329227	1535425.672	0.809
676035.8829	515874.9766			
373190.1055	701033.3296	632552.4375	2565957.781	1.172
752383393.1	584212217.4	454038042.3	234487193.5	1.215
883404.4757	1611592.032	1916155.906	6471638.296	0.698
		50801.74609	67184.31118	0.538
304120.8056	311185.4969	302567.0625	378955.6478	2.165
136765166	684005421.5	1767072819	218399713.9	0.7
36340375.88	23089253.26	44890000.84	50275727.19	0.571
3561372.442	1994459.098	3372937.219	975417.7584	2.084
3306577.62	4454489.445	2713815.625	2899643.056	0.441
54412676.59	35918156.72	70379824.32	15887490.34	0.807
4623304.157	10733196.01	11199997.89	23324313.22	1.222
7414628.166	11996340.8	7085146.5	8498156.621	1.266
31568252.83	16502726.35	22434141.03	21624421.85	0.991
	176732.2606	272561.2188	370799.1916	1.065
1654258.746	3591265.659	2647080.781	1728891.624	
660457.0848	4596401.593	1317582.5	837843.1431	0.677
2592338.224	1603328.502	2650653.25	1763769.011	1.165
1287953.685	1736729.299	1302558.18	9236090.264	0.688
4699522.274	9349392.631	3624167.5	8748880.337	0.853
808216.9196	526135.5275	902584.9375		0.792
422003.7584	231525.0451	311346.8438	329053.2196	0.72
	869161.8142	606172.125	259146.712	
924910.2251	754524.8102	238046.5	423364.7232	1.021
			77571.56205	
15707416855	6011593080	8442483760	7317200532	0.899
1717455.324	1034187.558	1242573.688	2694062.299	0.896
71006132.67	95450220.75	121592148.1	107561720.8	0.914
252198876.6	350055237	277704218.8	331654030.3	0.919
12110750.91	4248563.738	3729100.438	2935833.696	2.135
77142873285	79938316652	80504016795	87676808213	1.181
891529.2743	1890403.212	1933433.906	1049682.93	0.958
556916.0864	1927854.748		1605258.849	0.773
4420106.521	1072910.766	676487.0625	5759342.199	1.299
1362952.612	919255.3297	2376859.5	1604034.567	2.07
1231534567	2204975968	1358406366	2306768101	1.042
32159357.38	16885802.47	88844922.34	445708797.3	0.755
17323617.95	29048696.38	26593357.77	48223745.68	0.978
2442584.758	3939813.337	1311147.5	2974765.716	0.531
222513607.5	208508659.9	250818989.1	155330368.9	1.421
179147.155	499983.8756		2354764.659	
683496.7036	51511.22736	577904.2813	234363.7816	0.233
		319976.8438		

		543731.75	659284.6439	
	200035.1006		621803.8911	0.601
254527.3071	555331.7618			0.79
2527866.595	2035089.515	1044653.063	454535.0079	0.597
12229491.7	38567989.16	16343470.59	28114033.41	0.822
29310005.58	76271830.04	52059211.03	60325888.97	0.844
46172610.04	66850059.05	60521530.75	89540520.85	1.401
9107259.997	5330689.013	21067947.09	15450341.91	1.197
1678090.616	160342.283	3206530.984	4707373.973	1.038
	1238773.697		1104409.824	
428856937.2	268749902.1	271896845	154067870.8	1.019
2691718.592	3012610.501	2889587.406	1572056.192	1.077
139699.3809	201796.4681	224759.5938	188126.0268	
226821951.8	30863078.57	425119818.2	749507986	1.989
81705.84326		238005.375	377922.4182	1.117
2080828.924	1133657.559	214592.8438	2635888.069	0.866
10359478.15	21904674.26	29351393.41	4781674.546	1.118
4017421.382	816287.4012	2615580.688	1762633.379	1.926
151983.9892		241945.2031	446057.7932	0.761
4174971.006	6331163.198	4206885	3963895.768	1.462
382757.6094	52866.49338	2533931.906		0.31
4825791.076	4906745.208	5807197.016	7271142.213	1.013
1941179292	3885284306	2546216853	1682955489	1.114
790201.3819			218608.3879	
86549787.15	114406764	147841466.6	177669904.8	0.992
	925041.7246	663441.625	213185.8626	1.131
27841501.06	48160652.69	24550661.15	27304619.78	1.276
549419.6379	829508.7984	608889.625	1525793.527	0.514
2430304775	2954666516	2824722863	2813570213	1.064
161401127.9	32518493.65	108253059.6	67688226.98	1.209
25277429.4	5999552.936	8083717.375	3865002.559	0.727
811607.0759	531911.9409	1021023.563	615988.3725	0.379
3749900700	4606448723	4351902461	5588014873	0.944
53028549.41	72552601.51	54532493.18	88166773.85	1.251
104327797	105229481.2	79954435.38	21349159.17	1.506
210966.2008	1571785.925	1758540.656	4464680.834	1.288
1807575.422	2832791.688	1066204.781	5990458.89	1.132
26892908.87	78859411.92	35307780	32951997.8	0.862
447983.2214	508509.0566	1023915.203	240877.159	0.602
722741.3523	592385.8198	560625.0625		1.016
4951105.253	3158175.601	7206723.813	4688765.291	1.229
232548.2865	249220.9612	742139.7344		0.983
13921681.7	11949943	12157944.16	11460413.11	0.439
149564944.2	95890196.1	128642717.8	98111692.11	0.953
64225.49857			224494.8098	1.236
3312177.186	8076771.869	6216932.141	21896091.26	0.554
1045396.881	2743194.312	1449514.125	2015880.608	0.719

992996.4641	277686.9233	1023922.531	320519.7475	0.961
18581899.33	37716254.91	34478862.14	49087517.44	0.826
11324918.99	8223884.392	7004831.5	5108741.362	0.793
10407348.7	6788560.227	12870706.25	4318479.023	1.079
55169417.94	99724245.12	36695324	13495095.96	1.565
141609.024	55612.79663		711842.1277	
2282569.308	825498.1415	1842201.938	3589314.484	0.973
30054842.09	1318628.353	33150288	180136907.5	0.671
			561298.4307	
931191.4558	573205.5915	1612967.906	387970.2072	0.997
36156518.83	17901618.4	26494343.98	23713555.08	0.896
8829324.364	10795010.42	6967678.578	23597635.81	0.809
235741733.7	264735298.7	79344182.78	194898241.5	1.544
54051427.1	63404767.6	66179482.18	58718798.1	1.154
18791356.81	17124533.57	14443444.88	11837846.82	0.535
273876237.4	1582785069	370480500.8	455022013.4	0.868
				1.03
258719.6039	86865.73098	174600.2969	179860.1736	0.733
652787.4978		1422913	368913.5722	1.443
246395022.6	99603593.34	115076566.5	184661278.1	1.212
313545.9724	142296.9816	266145	498404.674	1.058
779796.1884	85235.57094	1106741.703	2017952.274	0.816
10804914.43	9032549.24	20085623.33	11504008.72	0.631
4652496.516	1882271.788	644357.7656	804378.2768	0.445
52084266.9	74957396.97	66717173.31	101509420.3	0.719
2782279.9	7352871.324	9544138.922	5517912.847	1.973
3784326.104	5532002.966	5577956.063	7409268.498	10.988
128423483.1	144141991.1	172295639.3	192929764.7	1.142
1305271.364	2280024.763	1683934.031	1003096.836	1.188
5143607.469	9438568.796	7953850.094	12185802.31	0.518
25873712.54	15655858.03	9722457.625	8826297.587	1.217
35199322.47	35538381.68	39673199.39	51719682.88	1.246
1041827.377	765501.8845	1916679.969	2764220.044	0.532
59976560.71	93716621.51	102367252.9	82212958.83	0.759
14425934089	18102971562	16677597552	16493606053	1.149
67729373.78	56647674.06	71964003.19	21413039.27	1.163
1278608317	1570571397	1627501877	859723759.5	1.236
2189292.205	658147.8187	1489878.781	1072670.653	1.146
618195500.7	535725480.2	481552432.3	1002883736	1.166
1813079.268	9554408.622	4297414.25	2758084.846	0.723
11166651.05	59369867.84	10657705.09	5720962.911	0.265
1063902.365	813770.4981		2246728.618	0.872
305290919.8	390918059.6	408217399.9	299333488.7	0.949
1082323.816	7572080.176	4263657.125	40694829.48	0.175
6788561154	7075641785	8877753490	5282640501	1.301
787308.3634	788726.6782	774563.3125	970191.7129	0.861

18544632.23	18006473.06	14590271.13	22180000.27	1.335
1012751.557	1098729.491	1237910.359	603921.8371	0.826
8874191951	13578953659	9340757095	7918171932	1.169
313000.2446	184106.1267	474946.2188	589369.1869	
141523947.4	218125811.7	171599859.4	300123762	0.911
258973536.7	499459719.1	279016290.2	444538626.6	0.81
98940.66435	281917.7733	155008.7656	233286.7157	0.298
28888558.64	78052410.72	70073041.16	70253458.79	0.755
2167009511	1655965966	1281089539	985005303.4	1.104
	207186.4199			
163580950.5	188214169.2	152743335.2	107568244.2	1.163
30837598.47	24728837.47	38547702.81	14248602.25	1.415
1766909524	3267246904	2627218717	2113823548	1.111
872952165.1	839257772.8	814389626.5	810719846.4	1.075
6120933.124	12997035.27	11727660.81	8323497.017	1.113
	23696.54659			
801898.9541	1083069.556	778295.75	2018559.933	1.204
3201620.813	4194094.951	2291737.25	2719916.936	1.165
17283165.07	14730723.5	15393932.47	8545432.712	1.013
16635940.97	6409304.592	18767984.64	7264446.26	15.875
	689468.0259	326987.4688	340344.4368	0.587
1069998139	1260406080	1135640107	864408818.8	1.083
21507815.47	22977566.16	20973826	21560358.53	1.192
1430485.816	2180198.409	1549030.563	1576084.179	1.157
	611446.4834	281575.1875	214530.6317	0.869
	2092109.077	1414020	1024334.451	0.584
816063.2692	3437918.364	675101.9063	2773577.255	1.52
7249991.761	6333248.82	10083955.69	3248976.317	1.749
	206756.5815	502281.2188		
10700206.88	3759151.943	13173831.97	6589940.87	1.399
4119136.045	10400091.36	5852411.875	8767826.125	0.861
8013153.172	2587549.068	928650.0625	1758323.211	0.595
4823850.308	6270391.416	5342440	14961694.28	1.002
139078818.7	201760104.9	137709965.6	196967329.5	0.853
2343829.543	5825213.563	3726243.188	7448764.475	0.67
4009825.079	3357373.892	6006225.063	5672066.713	1.012
1595264818	1331562385	1333528501	941459522.4	1.081
612553.3155	1157848.197	719029.4141	696131.3625	0.75
532856.099	325820.363			
	74018.51327	122072.1094	169280.0623	
395526.899	1720170.283	415759.9375	1713730.692	0.584
57328492.09	109815213.9	91456797.73	42085431.55	1.474
135687891.5	253786411	191915260.1	270240346	0.962
245447661.2	155556628.9	207915543.5	180319515.5	1.091
9014156.982	102882600.2	29982772.88	50430820.36	0.601
7244257.483	5059453.205	9714508.992	7426308.308	1.688
22984880.97	66404777.07	40348430.38	50469008.23	0.818

165162124.2	270263140.5	185662310.8	250894287.8	1.047
2870658.88	3463993.872	2903110.531	3442298.979	1.158
60238807.58	96943577.26	85311220.73	70529097.28	1.528
274214.864	334865.3038	736728.3125	855907.7558	0.901
4862512.798	3313465.683	2624630.5	3112325.332	1.107
4349896.276	2535174.62	4618421.5	2309753.159	1.083
131276348.2	85072092.24	120250460.8	30446162.14	1.133
53807215.18	106370261.1	59216599.9	137972963.4	1.084
5377551.298	10268297.67	11056440.81	14387870.36	0.597
				0.861
4296816.312	10380179.69	7475282.406	7715189.113	1.031
324360.9676	44757.87311	804962.5625	534129.2433	1.019
527688.158				
279885912.2	423412640.4	414307427.5	448897975.2	1.206
	286289.5489			
318100.8067	889376.465		472562.7111	1.054
440880.0276			634675.8284	
632427482.8	778250791	768941490.2	479194305.7	1.051
656240569	871276206.5	839349506.5	2124802799	0.991
	932924.5815	809217.625	1703712.347	0.46
	445875.3496	495053.2188		1.496
23982625.41	42001996.62	19067572.47	52069292.73	0.833
			657499.5732	
	943790.5217	603415.9375	1244257.928	0.455
11516560.67	20715204.43	14385396.38	12654552.22	0.684
2198612206	2507383732	2909688963	2630453968	1.033
4543234727	888663450.4	2382311928	1058562351	1.743
		651268.5	138017.7988	0.96
35407660.15	57666117.44	32189849.74	94843542.67	0.89
990573.2543	1361657.553	2268446.625	11983987.97	0.788
1368684915	1619296278	1476636634	1169173233	1.345
410429965.7	354601672.6	310799819.4	289039896.4	1
18406088.91	14305865.99	8044884.125	15145340.83	1.016
	176662.1428	227868.2656	387705.8915	
1543955.909	1006727.123	3146438.209	10270775.72	1.105
50948419.35	43074619.54	25581360	15817038.92	1.084
485472.6163	1056991.986	535420.25	780597.1101	1.115
	3728565.397			
	351384.5975		5339289.529	0.989
1231064770	1078219463	1043044309	1188391512	0.991
453703.0961	150058.2872	611427.3477	522236.0729	1.219
37410951245	30294639618	36547104132	27372950997	0.993
50269610.83	40349710.85	36549548.84	47787981.31	0.733
713259.6844				
				0.907
63654711.78	122097915.5	91336622.58	141504623.3	1.257
3209425.877	2725951.418	1996218.219	1278693.177	0.918

2168778.595	5412765.103	2416869.031	1040943.313	0.938
		299772.5	396147.6124	0.638
1617264.23	998765.3788	1819940.938	612863.5045	0.405
146274933.7	18936222.27	258876947.1	952188258.4	1.349
22888790.32	47702450.88	27761045.97	31371607.75	1.018
4330093.088	8302574.195	4540124.625	8837349.499	0.928
1104122990	753817583.1	757047537.3	715017685.3	0.821
5771585.374	4997985.839	8329818.406	12389407.77	1.825
6212892.991	4659212.17	4171602.5	3104166.482	0.946
46929202.7	52146392.32	103007283.1	228860906.6	1.578
943670.0985	1894643.202	926277.4063	1686864.702	0.513
22400269.44	18460942.2	18131131	25619193.44	1.013
7983053.892	8739467.536	6952635.25	9478650.866	2.234
1484416.777	975437.2313	296921		
36753225	68002722.75	85053873.38	199986376.3	0.749
5298188.644	2257850.364	739467.9375	2320903.193	1.487
7414819.875	10818851.29	10586322.63	16200388.21	0.919
61544248.01	212356379.2	156692456	32509779.48	0.839
6039508.152	15266150.02	9342650.406	72166283.48	0.642
16323267.4	15442853.64	35287019.94	16564688.71	1.593
1044094.209	2718834.202	1665165.375	3102475.859	1.233
1072285.699	1568298.762	1762713.688	625508.2548	0.794
47201777.66	47111326.15	45999275.06	31268547.3	1.343
	695509.3011			
9139238683	7354055722	9464137932	6441562862	1.424
482519964.6	322876666.4	316231903.8	348726370.8	1.055
2854447.265	3903355.014	1416503.5	3507084.338	1.063
551327.4212	393726.6516	208375.7188		0.641
9777856.378	13627774.34	9069259.188	10832708.7	0.631
18740254.69	9808884.835	12845370.53	42270869.71	1.552
3805823.337	3538489.228	3117201.656	2971138.845	0.81
2588616.972	2761472.637	1194725.25	1717393.326	0.253
	1810524	350378.4375		0.888
33871468.5	68681006.77	46272300.71	48441086.55	1.183
1010906275	612202737.2	1260820357	2288993655	1.267
6815819.429	10311273.5	8358043.192	8621956.714	1.03
16425149.86	8678461.208	13879715.38	7206143.351	1.286
24528215.72	54149554.86	34355991.81	35173695.99	0.728
1059779.986				
311125132.5	617430956.1	343943603.7	263997108.1	0.979
11372872.43	8300645.16	15485253.44	25527201.8	0.992
13320313.94	9474070.539	13064445.13	17913445.03	0.893
10782090.13	3523215.701	8230850.492	9903891.324	0.899
2782780.038	5143481.654	4388842.25	2809772.142	1.602
	9712275.901	4697931.5	3703747.764	0.801
4028467.321	1790029.328	4471145.133	1961182.306	1.018
11205288.08	4951892.804	6945009	7708185.581	

18476431.84	23072258.28	25151266.44	15539348.9	0.943
228512.0308	945462.0918	992852.9375		0.239
192829392.4	388880098.7	208999200	525892151.5	0.696
6301376.018	576728.5036	2797145.25	5333223.379	0.968
8552520.832	12942880.24	9734610.5	21852279.63	0.948
330966969	689819742.3	549959056.6	412837021.7	1.018
12191824.56	21747699.72	13225674	13491643.42	0.894
9588570.342	10133062.05	1899538.5	3455961.706	0.585
13061752.65	14735574.83	9010604.875	6407788.635	1.24
84034108.5	86288502.63	144980476.3	221775284.9	1.036
1362953.286	2267508.41	1263281.375	1486730.066	0.417
7239430.549	2536906.238	10721232.42	10260163.63	0.692
39500436.81	37930395.4	46012706.66	33219903.33	0.825
3885922.489	2612188.747	2553040.25	3589563.901	0.71
	166773.7894			0.465
137310.9281	6501018.735	3239734.531	5267597.478	0.79
2990275.747	1048457.301	2342992.5	6318435.195	2.174
825214.7911	2742969.836	5434539.875	6486609.085	1.23
	212974.5137	125427.9141		0.742
7562864357	10840469529	7080563055	20759243819	0.861
12430597.29	20459418.35	5818936.75	21194894.12	1.138
5156912.754	3322354.729	4801285.625	4360593.349	0.923
41011.92932	13716.45669			
				0.429
1690946.259	3637581.93	3661961.438	2673566.178	0.981
				1.025
61747405.99	77238553.68	96996615.57	131945823.7	0.968
399005549.4	231932113.8	322905831	412495077	1.061
3678822.512	1107312.419	5613985.719	4132527.252	1.475
3201833.007	2408011.585	7168347.547	6254614.69	1.401
169972070.6	149856909.7	127816093.2	249894173.3	1.284
821234.0195			3362407.732	0.752
766087.1409	139128.1181	701620.125	1087950.892	1.071
7679974.132	2454157.73	3869007.594	5259502.776	0.159
	541672.3587	271259.7734	1253990.959	
1414652.248	1095017.605	2034440.188	1320202.204	1.827
28853754.23	16698360.46	27483070	25166386.3	0.465
191331.3356	1405890.076	1552885.313	8642920.529	0.629
2231917220	4030113814	3138799684	4964790283	0.952
	380439.4637	365637.5938	498982.7877	4.86
1588563.016	1359992.086	905727.9375		
1137085903	1196185661	1171258355	1027061380	1.165
2452241.908	5125461.944	2760488	7692397.39	0.959
183314674.3	535075061	250506489.1	284437677.6	1.189
	32775.40694			0.927
249970.6299	2063964.326	786611.3438	2525626.268	1.499
343010.6481		5267642.5		0.819

36864703.53	18954132.34	14301822.25	29879322.48	1.22
64112320.76	75281357.07	51691179.94	62903706.95	1.057
832764742.7	156590652.6	364640950.4	538017887.4	1.265
3376822.473	1936064.93	3342677.781	2359314.119	0.283
	358761.4722	70160.70313	448105.4099	0.304
515513.5157	472954.8227	205975.9741	293480.5565	1.393
170962743.8	106606382.4	198384854.7	110319221	1.631
31779976.1	36577432.04	17940580.52	25329170.67	1.018
226479.4293				
16207112.25	21383132.7	23562995.73	19348363.45	0.878
538650.6763				
	964866.8314	838063.875		0.931
12272295.13	8975826.429	7488634.125	8894318.66	1.633
756215.2018	241103.4435	1186130.969	1196222.85	0.433
1499450847	2031753838	899808716.8	1105339114	1.095
849468.9308	638226.682	1166102.625	1600210.475	0.745
17476800.59	16199141.4	11615879	14833869.84	1.356
57248982.11	102497134.3	85031468.59	51820005.51	1.188
11292378.47	15349124.56	11550972.52	11702586.02	0.551
	1738993.056	554431.25		
5967826.855	760782.5818	1562832.875	2657812.469	0.955
	1358704.27			
	957489.5673	814120.5		0.776
3053022.272	4582264.285	4835571.453	1299143.17	0.148
358252.1252	93030.57051	648798.4375		0.843
387384.3568	993347.7657		446063.456	0.342
88247.65563	34730.76632			
209704867.2	90466261.98	196423306.7	193453529.9	0.694
5165829.126	4009467.056	6116125.047	1261943.536	1.44
2175878.901	3960278.55	3467393.563	2880160.782	1.067
3561104.5	5588510.07	7473635.188	8157424.466	1.472
871628.2426	4988692.336	436110.4688	523969.4955	
293484.3711				
		519318.4063	553171.5695	
243958521.5	308849394	294438832	257524341.6	0.831
3610814.957	11096511.01	8592763.078	16564853.2	0.778
2189338.638	3576092.689	2010681.5	585397.12	1.1
1361199.83	2882134.261	2462561.332	3535829.51	0.883
8006842.499	9213047.906	3802747.25	9611732.333	1.431
254818932.3	414427108.2	303943693.6	401443574.4	1.219
589627464.7	1254273851	849996663.5	836413464.1	0.947
35093549.63	17342400.22	16685082.24	13291244.46	0.965
13182113.94	27680298.81	25599315	30970575.07	1.073
343590.0204	5927648.361	3461717.625	11450269.93	0.076
83181120.66	70950445.19	122598033.6	147124036.9	0.615
3037692.941	7340208.099	4832826.438	4976361.276	0.833
	1966020.071	319201.3203	1583814.735	
19697689.12	14199950.82	16152146.69	14925365.17	1.082

	110887.853	1114398.871	4228762.253	
5691053.754	4757506.393	7426495.25	4543232.318	1.098
1335271435	648756743.3	2259248891	3472328844	0.947
26514216.1	70078671.62	18054653.91	54058796.4	0.702
7537925.112	8068351.121	4648208.594	9802890.786	0.97
31469244.19	36537942.07	35379364.92	24425717.43	0.96
1105200.044	3110339.084	1718398.031	1327071.109	1.004
26168044.75	52095434.54	68684590.53	19601244.84	1.009
84674964.22	114067716.7	95896807.22	42797932.28	1.454
173145.5867	134936.0081	349829.4063	113649.9736	1.308
41631472.26	52572409.03	75814598.94	79372708.23	1.144
74042.15579	159336.9022	116498.3203	176725.6654	0.528
147768.1391	42161.5744	102901.0078	230243.4025	0.961
	718315.9368	678949.8125	1072593.379	1.343
3612130.922	3034539.077	5199944.734	9391047.475	0.725
240200.2984	260075.6192		467756.1528	
1866112.412	1138130.6	3288448.781	7787979.364	2.149
971402.0061				0.578
570641525.5	35339867.13	456210304	252881949.3	0.57
31423453.89	62101829.63	69279994.15	55733630.26	1.154
267565.8703	250482.6204	238674.375	312193.474	0.938
8245605.009	7332320.756	9784852.063	5172639.663	0.715
3928778.758	10525533.38	919840.6875	7464066.253	2.489
30348884.08	67289709.5	35744542.25	55490541.96	0.358
6094396980	4052344512	3515825092	2747313721	1.232
4352325.262	11479627.49	12630604.25	8715897.687	1.019
1147064.668	642259.7794	556299.6875		1.684
20380104.46	64802324.52	48509157.54	76302509.58	0.751
7471742.203	6110521.707	8904442.723	6201629.68	1.344
35048593.87	26462082.87	37684244.56	25099515.02	1.011
699745.986	2075791.642	1766152.906	2422029.308	0.817
36884623.06	20597413.28	20509445.64	20944590.03	1.41
44509824927	39149412359	39636945530	40373463625	0.915
476857.2987	772027.2587		510131.2713	
428958847	742678622.8	578839934.8	377570529.9	0.977
18542216.41	5377317.24	10555658.56	14691560.36	0.996
1625627.113	7590118.666	4489115.438	6398512.389	1.063
4503026.998	4876608.225	7705619.75	3162979.5	1.574
	1896.574991	2831.42749	2111.174817	5.975
209403.6777				
2026717.641	533233.8692	1080469.125	577242.72	1.232
119624.2499	41142.57533			0.493
15080.67845	34794.77177	45986.23438	498498.237	0.792
2229065.833	274903.495	1825575.406	837764.0658	1.698
94215.47271	81704.89184			
1138762.826	5754070.576	4441994.422	7044000.572	1.133
602030.6698	295137.3211	1339664.344	916609.2915	1.109

4266430.701	11550773.96	8494163.438	5045057.472	1.115
64143.83716				3.842
10373813.65	21817305.36	11247015.69	8368425.085	1.907
4559440.679	5030303.215	1598733.5	2016667.706	0.78
1873296.997	1514649.399	1949376.5		1.087
951831.6767			345440.9958	0.668
203953707.1	79367468.01	185106858.6	241665549	2.819
13616361961	15464956943	14885876969	23728928819	1.041
		405154.875		
5523190.448	4866793.9	6593688.188	1094713.484	1.184
6923952.099	9755450.377	11800291.2	3052597.465	0.992
2767501.382	4774045.774	1792121.516	10179531.92	0.953
348446325.2	348999388.8	345422143.6	404378058.8	0.815
257340224.5	261678619.8	495567884	482736242.2	1.282
2964812376	4036080227	2648550560	2428501821	1.316
	212148.8871	138771.6563	250084.9286	0.617
752219.1082	1141530.266	1283418.625	700685.1499	1.385
3215347.301	1215830.967	3448455.344	2349203.53	1.278
1256009.365	3023232.887	1148181.5	998161.954	0.815
1888667.95	3147659.207	3709069.813	14445218.79	0.938
2367286.088	3185687.835	5784609.688	10073304.34	0.975
2475155331	3231574596	3286735561	1852376422	1.264
1758171408	1495760108	1056674703	1370801900	0.872
228664.5433	373090.4449		4697390.119	1.422
33530972344	26077347011	35240943426	27170085558	1.196
7456422.265	6910936.461	6766167.5	3535739.849	1.135
2800494.575	2199355.46	1420022.543	1321803.426	1.299
5235578537	2089441922	2470983609	1047114092	1.39
2741725.151	4244752.028	3435158.813	2117406.322	1.176
2997430.374	13348283.19	9163756.438	18778105.25	0.756
709526.8356	1702295.675	528067.25	1412913.479	0.997
4375164.003	1375132.333	126599.8672	282250.0588	0.273
217879.4618		80031.64844		0.239
4053068.361	1205899.343	2037775.813	1994763.935	0.862
8989349.284	9671616.92	9062040.133	10487555.89	1.033
		967596.9375	649257.6017	0.42
176132.2568	346431.2269	610777.1875	334640.1691	0.789
1441645.214	3010422.189	873202.75	1479099.376	0.858
879028.1346	555490.1196	822513.875	916082.6151	0.379
201187262.9	111394962.8	160559542.5	194041835.8	0.619
4417410.29	1131713.216	6041283	4997926.896	1.105
18550141.03	25910248.27	12583114.04	22254500.11	1.304
	971041.9356	734448.625	460546.0625	0.965
209467052.2	441823969.6	367304893.6	404347486.9	0.951
4113946.529	3942321.648	3840933.344	2775235.451	0.884
35397.9493			1049044.253	

14148399555	16346648854	11604277326	4700211647	1.17
788175.7476	390032.1108	407326.9688	663625.536	1.33
189705838.3	158634555.9	224014864.5	223229852.4	1.164
	463117.7657	538751.8125		
		407061.375		1.28
16779448.4	23684128.12	18491223.47	25720714.6	1.26
293730109.3	193460177.7	131395812.9	299125582.5	1.387
321930.9705	1292233.076	2274342.75	1088941.414	1.5
235895690.3	74754961.17	368935519.2	1014215063	0.647
	753747.9425	2268859.531	790745.0519	1.965
	317853.3454	223091.1094	4352655.687	
1464656.582	1982065.637	873207.25	476147.6498	0.7
85627331.74	61116425.22	99500830.5	40184909.81	1.281
8803675.929	18682805.81	11707536.63	15713176.38	0.657
3400988.723	2428375.922	3696316	2084244.248	1.505
3019206.291	1919943.938	9350227.875	11837462.2	1.352
		571864		1.912
344239.6258				
44775.39413		78484.46875		
3249687.658	2405911.203	3868303.5	4975229.452	0.538
24617428.23	16706973.08	20027492	17993300.43	1.469
	741559.49		1019879.198	0.577
171241.3488	55816.4292			1.293
18811215.29	12326182.62	15378810.03	23476723.41	1.263
4939091.531	6194137.382	5583490.063	2160091.75	1.403
116744060	77130564.35	90855606.94	99863366.13	1.326
7264321.264	6938217.549	7616071.875	5853239.617	0.944
35518354.03	27881820.38	33635466.95	25277016.25	1.111
393693.2875	517279.7029	681965.0625	561448.1247	0.977
6251172.02	3211819.807	6621250.5	15007820.01	0.854
635500.6087	611636.7488	500528.8652		0.745
	191644.2971			
755162.7844	1289475.796	729063.5625	725257.3592	0.802
630686.4851	1057912.401	599613.1875	748716.2841	1.163
152146.6041	720451.641	1127195.625	900698.8106	0.51
3702345.582		5521748.5	6133214.922	0.248
981674.3299	707902.125	776868.5	416946.7289	
181655055.5	105712977.4	142462004.5	93500985.09	0.944
33242447.16	36458126.41	29508543.3	36770972.11	1.101
1144467105	1603773560	975883090.2	1819166188	1.1
278951.7031	975360.7778	1003306.625	591983.322	0.527
11872007.48	15020027.46	15711397.47	18220339.75	1.265
	19819.06828		186055.27	
	719847.3423	409158.2813	444471.2585	1.227
13580052.68	8767281.467	14514044.38	4883747.465	1.703
5268415.673	7134284.294	1755215	1546093.49	0.42
3064463.15	1693302.146	2201500.906	229551.1054	0.368
5619723.376	1100643.872	962775.1875	12677794.07	0.253
22926584.68	7740953.498	40957334.56	142358301.1	1.365

3558236.833		2648248.656	1691592.082	0.521
24677979.84	9900654.776	24831590.56	18562745.61	1.181
619258.2035	4470056.999	1203326.375	559515.787	1.276
1327878.909	760524.339	886417	665375.2139	1.256
	1338306.768	128396.2109	869170.4617	0.176
159598750.6	227372152.1	182965501.2	230181749.8	0.849
8858093.372	6853650.161	13215441.34	6065586.395	1.353
2678215901	2721280329	1927063686	982536828	1.253
82928497.42	17813897.24	200608037.4	47507190.46	0.245
1812715.09	6569709.981	2963205.406	6018401.828	0.991
2480519691	1713425859	2002099654	1718900558	1.344
703707.5587	1481066.983	1037807.563	211180.8856	1.537
39966069.53	43989570.62	51738307.13	16131111.04	1.165
	354610.5445	144857.8906	661081.5794	0.301
			1125569.979	
25772386.42	2613581.651	26878461.25	56554323.48	0.613
11884085.62	34648195.09	36880369.89	29597114.55	0.673
3398040211	4905625957	2666039941	1083005616	1.004
4563686.757	6031831.449	4859131	985987.0834	1.189
24711470.89	23472333.06	28355822.63	13920509.76	1.269
6551260.323	7250842.349	5380734	2427903.14	1.137
540564.3423	1370105.535	690646.5625	662157.7184	0.411
8367.241723		58879.82813	1590501.899	0.134
529915837.6	109888821.1	621403409.3	1180046537	1.121
60035238.9	84910679.91	70353623.08	47328781.01	0.883
242751.6583	182411.6914		3501799.541	0.461
	1384486.22			
330258651.2	257205973.9	197654721.3	221052111.8	0.67
3328793.883	2496885.768	4150171	2103765.218	1.603
395368.3633		187607.0156		
2500397.055	5465629.828	5369298.891	7071201.545	0.969
130309296.4	121112000.2	206509390.4	90471245.68	1.522
35108530.56	27338472.48	24968380.8	43924818.73	0.957
690762.7145	40786.57117			
	176467.6112	1354604.125		0.546
454657160.3	507785052.2	527658791.4	271312243	1.071
11757258406	9128438256	11172592240	7141962239	1.506
412516.9213	233612.2071	1642662	76026.01543	1.06
6658879.829	4036137.91	4911047.625	20768053.13	1.14
139309.166	143451.4555			1.583
1488662.811	853367.5542			1.086
	2917878.593	3959899.75	593671.5179	
1481531.67	4869728.545	1828584.938	3546321.747	1.312
242988515.1	271868826.6	357001251.2	340781647.2	1.179
749647.701	16917237.74	30533338.34	28964208.85	0.816

10454133.32	9590164.422	4975262	4821596.347	0.687
415999021.9	335810940.6	529459096.1	159109816.7	1.301
367505.0243	1946866.354	1160125.469	1560949.033	
57469726.56	132037854.9	97626461.53	117537818.3	0.02
61606.90036	88409.42322	41521.09375	251891.1674	
83341200.79	336540272.7	29809107	55172542.61	0.807
8604940.448	11457691.51	9056251	15905073.46	1.071
				1.462
606776.3582	636763.7307			
7109480.205	15974066.21	10366262.63	4031979.032	1.026
	147979.4298	486545.1563		
		480788.0703	261803.2846	1.561
9821908.453	5449253.945	1377622.688	3279165.761	0.132
93045444.91	90452950.3	85213154.81	64394831.6	1.209
8466608.483	4729784.578	8510032.75	4007835.423	1.256
			782129.4435	0.658
133552.0875	187177.5032	798392.2969	745440.6772	1.626
200277359.5	151907547.4	219769715.3	78899603.05	1.709
4844427182	2705608035	3149771368	5111140016	0.911
2859695.17	903497.7745	764400.6875	609055.7943	0.644
3633027.129	1785659.156	3044832.438	511540.7753	0.549
275565.2494				
2757360.99	3406325.216	2543181.25	2443696.589	1.368
	1783098.885	4355351	2115781.058	
1065733.774	384830.3723	760299	815617.0959	1.446
2447671154	1853611880	1762568465	1254307816	1.028
357237185.8	552207640.2	526414478.3	829593880.2	0.733
8761194.676	19000417.64	9314067.668	6111425.89	0.765
	866880.1737	337911.5313		0.604
7896758200	10768576122	8103922499	5883737931	1.292
15002221.2	22053658.74	18081293.02	27413008.66	0.176
14272469.36	15842647.66	14340802	23519897	1.137
			1418568.028	
80537751.64	7532356.405	53839836	50653065.23	1.027
579649.3765	1014576.451	651729.2813	116982.647	0.961
7544869.017	4784831.5	11928628	2638715.773	1.053
6893744.257	7564293.355	15442697.25	2354261.746	0.329
2026978.953	6759461.683	4643703.531	521697.7254	1.066
17110346.09	13184620.28	16350779.84	6524514.946	0.969
7235442.074	11501492.05	11597438	11060862.22	1.282
1417537.086	2094363.039	2866028.063	2539883.945	0.876
7013688.556	2452574.223	4275894.906	1538503.687	1.259
948059.6322	859465.6032	686721.875		0.797
1295886.314	918048.0728	2750708.188	3220437.147	1.38
345066.4883	157220.3976	499886.9688		1.225
464522.7968	1336336.534		626078.3798	1.383

16514933.8	42911029.36	20680386.13	37039403.64	0.565
29169942.73	44616444.72	29573082.03	45599766.76	0.829
13619184.39	17617351.53	15610401	7965151.887	1.614
400629.6637			677026.5429	
36940108.37	10580350.52	35228201.94	30640549.31	1.205
1425671.785	598081.6209	835567.875	296424.0918	0.632
31129.6716	17739.31605	29239.36719	94629.52336	1.255
1718032865	923160469.6	1268829807	879727752.3	0.629
917427.4468	864364.9962	905338.7813	802776.3041	1.216
16302843.91	5749377.404	6374756.25	16364542.24	0.242
13368391.21	14874061.51	10923781.5	17669741.67	0.862
986346.6613	2079631.446	1216554.875	1199301.91	0.926
2281829.557	879870.9629	1172420.594	1478989.49	1.182
4825439.913	3867437.679	3348459.5	5558555.799	0.734
	119778.6037	115247.875	137024.3691	1.308
129178.1174	179359.1982	224661.3574		0.931
5138509.11	3693781.105	4526818.391	1681583.039	1.027
1103206.166	3303885.338	3817653.969	3731055.725	0.546
68204877.53	2085094.595	2593492.313	3324738.286	0.778
34795209.71	40798653.1	26565889.69	12531949.33	1.654
44380889.2	102992035.3	94711607.75	100352745.9	1.016
810825.0502	1568741.201	1191094.125	1720960.434	0.991
3228241.083	2555844.824	5375189.313	4556337.172	1.876
3720642.771	789488.3105	611970.6875		0.498
2270623.886	2837363.305	2946790.781	3693418.975	1.321
33507962662	27875072001	33583476517	41091823345	1.016
297023574.8	167478578	418687397	350342261.3	1.238
7582308.26	6662076.636	7786920	11629863.07	0.679
	473114.6583			
16188894.39	13791517.5	11598040.06	28422116.16	1.083
122090.1951	104401.6434	92139.04688		
1256551.431	1557841.112	1621889.5	578067.689	1.751
587943.1053	718499.2836	876699.6875	630655.6275	1.193
4469529.168	9976916.46	9602820.031	8214591.456	1.345
8545484.028	8784603.989	6337196.781	14317383.36	0.893
			296153.0515	
416170.1202		337685.25	1124046.073	0.71
88326.13688	243843.0261	672010.5	1263723.753	0.372
863690.2191	1129571.245	1817197.25	1624361.41	1.341
145066720.5	132097474.8	128629758.4	101423773.9	1.349
545220.0201	753725.7143	375510.625	985708.9983	0.598
4105930.527	7256546.622	4397580.005	15185698.55	1.298
659727.7396		419523.625		0.995
37823811.98	9055014.358	28650699.25	35228860.46	1.612
38192208.53	40369596.46	58595963.38	14556127.23	1.229
4530834.487	5468769.639	5995947.125	3300128.426	1.175
3286762.183	3641075.659	6353794.188	918613.9314	1.359
174440663.8	84074467.98	174201773.7	130324733.1	1.142

163103.3015				0.972
8091923.312	12453079.86	12547878.63	13682444.09	0.951
8253437.222	14096374.85	7001559	16475470.37	0.853
1028379.125		1111603.125		0.157
2301114.818	13765869.06	9183868.469	12874252.42	0.92
8249892.28	9896671.525	10359124.63	21349656.22	0.923
			1408495.684	
1184249.273	1369084.669	1309663	1629961.944	1.325
6076270.123	2002197.534	8590152.688	11434678.22	1.7
780662.8983	1880160.102	1137144.266	5171923.67	1.54
572192.3316	1133412.89	2145595.75		
25220732.88	26741043.12	35872221.38	11025568.12	0.671
	494395.5164		326211.1221	
2022478.754	3853197.894	3425524.188	2108916.905	0.901
			933575.8555	0.958
799729.4655	1566711.733	3220238.516	4893177.315	1.113
		407320.875		
396866.3393	1769905.529	1412555.75		0.627
878840670.1	435313626.8	651878232	550437437.4	0.979
14181062.8	26037427.31	20695588.84	28021049.71	0.964
				1.127
174076.5739	123189.7026			0.832
399849.6158		634217.3125	986803.5414	
	419420.5384		759682.7484	
132795958.3	42132802.49	39203781	45602309.74	0.764
11815.6873	6943.343666	12284.45898		1.029
89795601.45	67377402.56	45980005.5	46936474.04	1.267
447777.4441	592491.5185	747944.8125	485364.4361	0.688
661724.0221	616243.7615	419415.2188	683032.238	0.655
836968.6564	482796.7113	488916.2188	157114.5623	1.344
313056.8356	865738.681	424040.625	499868.3456	1.053
16527932.35	34103826.54	20150945.63	18083551.86	0.959
9197133.66	15663660.26	21221273.41	14880853.08	1.018
3100726.107	3874166.496	2927137.75	6314183.156	0.559
68617360.97	31643746.81	33717868.47	39620937.18	0.818
334570.5873	388778.6345	494491.6094	1021512.561	0.421
63643635.06	52673733.52	156656074.8	56020614.33	0.941
9055067.829	7310849.583	7630604.563	1078595.147	0.211
525490.1436	219471.3949	216522.8281		
1237999613	833141057.6	1074251664	720563917.2	1.43
1857164.83	1548855.618	1103851.344	506387.4364	1.049
332396307.9	153897156.1	181005600.8	168515676.5	1.163
	182688.6228		69217.74975	
13142396.53	22965150.84	15552560.37	20656044.11	1.099
80687.95806	92135.33934			0.761
404232.6993	1241822.633	347429.8125	872811.2871	0.608
148627146.1	147287404.4	160542592.8	183397957	0.629

387636.2519	1431458.239	669155.75		0.739
382033.6525	505324.398	880450.375	2374711.285	0.966
5431179.316	11582091.56	12486809.81	19614572.58	1.1
12940678486	14340255845	13507347452	13227196171	1.279
15300645.36	31666097.06	24832488.47	21501116.65	1.122
	186787.5375	356486.2188		
15637118.49	7089188.385	17042380.38	5438875.776	0.798
1300420.629	1439653.839	1002338.438	2732241.905	1.239
387199296.3	417330201.2	318749776	254400242.5	0.87
1203329.297	2271958.781	642190.9375	920621.1667	1.072
	645647.836		1313693.596	0.7
2711704156	2516678517	2921480080	2769525216	1.474
151329.0349	42178.42248	117175.1094	73233.65303	1.252
17016813.85	20354518.51	21468192.94	14310638.06	1.069
688501.7038	1207515.945	1180540.547	538955.8574	1.308
1051401.405	1948905.573	1599817.406	2167530.106	0.996
3909657.449	20865722.46	9056623.219	16232618.11	0.523
11749228.42			1717655.771	
672606.1264	597741.9516	402676.7188		0.754
2187726.536	1583043.875	1377456.5	1257572.577	1.146
1074724.596	865519.0188	2105370.313	1634959.791	1.053
259060231.8	508951529.4	327230287.4	381905168.1	1.09
2988574.46	7121887.469	3597156.063	4296775.338	1.138
68121.82189		296513.5625	374320.9292	0.757
305678.5191	1042972.791	738536.75	1841717	0.957
44742339.97	31759161.75	30121229.91	6514013.701	1.429
324231050.9	741846545.1	398277958.8	621748985.9	1.204
1016621.681	4285533.653	1425294.5	1806172.393	0.461
	1030936.255	494823.7344	834834.2619	0.796
23972567.07	31100483.27	14555331.28	24060413.03	0.74
940555752.8	1524287430	884377730.5	1291498905	1.142
	182458.0413	3044.054199	5613.8205	
8115320.467	1189544.159	6322019.313	2002955.514	0.39
1386622.631	2441477.608	4632816.789	9040343.356	0.983
	72884.58623		679588.2969	0.566
1167583.559	422013.1604		3332788.8	
1179598694	1602516475	1139088866	1740857591	1.085
933742.3325	1038077.823	737998.375	1717309.395	0.82
6297777.927	2650822.843	5325479	1074443.606	0.069
				0.72
46770.4434				
2926896.473	5907276.467	1942341.25	7935233.634	0.478
12664006.03	15011412.83	14618051.03	18932066.03	1.945
751538782.1	338093251.9	630558436.6	500049405.4	1.2
19971808.72	12804563.97	18572389.03	9308748.523	1.002
	275471.1531			
82149486.16	153399293.6	97336550.5	172176870.1	0.857
161747256.7	269240834.7	145261930.4	238720135.9	1.144

68801.86351	489634.7616	578741.125	533658.6558	0.775
1168663.238	706828.4493	1319297.875	1728556.978	1.521
669483356.2	1352429941	1204933210	2276176853	0.958
3696634.565	10617241.6	4308773.313	3708354.472	1.163
2080786641	1713154037	1783133268	1049034059	1.718
22046600.88	21415175.16	24589351.69	16360926.76	1.525
757479.8804	1281672.552	869415.7734	327839.1084	0.926
169997.0434	532744.7351	346889.3438	375299.2833	
11516490.13	20249987.33	12162119.16	13700854.82	0.957
21621767.07	10225870.55	10256696.63	5744579.822	1.503
1045037.456	694016.7595	1305223.344	736088.9943	0.835
1190939.216	3510268.165	2294761.563	4113575.952	0.955
712926.656	1618822.328	1296630.25	1229393.418	0.921
776277.6572	1176264.988	1106763.375	774323.1724	1.011
184035.9279	411603.8084		317830.3103	
1750946.389	3505659.372	2457292.214	4430251.872	0.854
6938369219	6614103265	7412274435	8151341896	1.091
	143979.9843		278601.7503	
100829261.9	126392706.3	90109560	58741003.6	0.514
231319.6684	2397744.911	1350916.469		0.684
4407817.283	6361303.344	5275595.719	6368451.487	1.498
802487.2488	1170641.03	675104.1875		1.509
3220907247	5157685942	4375432539	5544878993	1
617294.509	864316.5757		923542.7123	0.808
6419937.276	12188284.31	7467991.438	12605845.66	0.776
19128523.84	34802887.29	18832058.13	17624018.07	1.147
1336156.298	2357590.797	2741961.281	3071526.332	1
459501.098	614939.4866	784877.875	1414672.544	0.961
1461262.381	727196.5026	1000234.5	692585.5579	1.431
6441839773	6853357076	6671241224	8990441581	1.306
71113.41674	592190.3325	117282.3359	559111.6534	1.336
516066.0499	616469.5824	613620.0156	1623871.172	0.54
12861140.26	18852861.47	15268197	24832952.11	0.892
2863029.545	4358220.477	4018988.844	4507843.641	0.921
434366.8029	116259.3374	598280.75		1.408
18124872.81	24500349.78	19457073.47	24526000.74	0.964
3325615.296	5486534.755	5734867.781	11644453.37	0.993
2038707.832	2843953.682	3031122.875	415944.9061	1.125
1356161.758	1252821.42	1518574.125	3217441.378	1.124
1209241.462	864882.9685	1440833.375	1369630.977	0.632
40184683.5	56981442.18	37374334.45	39472420.99	1.006
17163840.01	31131358.23	24504360	23899456.04	1.107
2027737.717	1135820.696	1260346.813		0.33
547284.468	717460.5783	687104.9375		1.007
3393259.203	5380708.684	6672944.766	6990354.781	0.927
28555285.98	29046426.18	27711387.38	34165245.42	1.313

590978.3879	2662359.778	1169355.5		0.949
490458295.6	466029198.2	486894620.8	272443832.5	1.241
1044405.033	232436.5403	412805.7188	626724.7508	0.571
4345310.202	3357699.233	3424155.566	3470589.119	1.13
1644309.337			582712.1321	
728381.5995	1790049.704	1023240.523	419030.1764	0.112
10834050.32	14632275.36	8858214.75	10800242.15	0.852
549466.8851	1155028.905	776490.8125	1122765.061	1.003
2251955183	2008935888	2086484622	1206776351	1.156
83172468.66	94074616.33	93694159.75	163822289.9	2.305
14092787.98	21970352.69	15387822.55	23897150.93	1.228
1250136.487	514060.8969	1319998.625	447233.3012	0.631
78404823.29	36046847.13	65406530.72	34837752.09	1.188
9268323.999	33548585.61	11324439.81	17093497.39	0.581
	1097630.543			
2420633.25	4252326.054	5578745.688	5224520.338	0.636
246911.3894	1154850.018	1440232.938	1006249.681	1.783
5680109.665	9220935.32	4369970.156	7075039.44	1.22
959876.3196	3876916.272	1511883.5	1363308.602	0.844
3872985.578	8987387.16	7855621	9049233.586	0.862
1850568.225	2122929.726	1819836.672	4351071.393	0.618
1138269.149	2699805.27	2603349.375	4538046.99	0.936
4908168.951	6135810.963	12370777.41	8372214.526	0.902
	710382.049	878941.1875		0.472
90719.92743	27662.05921	121032.4922		0.967
14509857.15	22385886.65	17964950.88	13554675.38	0.78
12656385.68	9927239.992	9359537.641	9878182.269	1.269
5054021.593	4225633.423	4476822.25	2971308.494	0.782
1134075.301	1145469.602	2365298.563	6870225.609	0.901
3009177.457	4432460.865	3851979.688	5566120.388	1.049
1004289.786	1745051.109		657794.1075	0.883
		751822.8125		
243158.2011	96362.89755		491343.5699	1.024
9978692.339	19509838.41	17996175.78	23561523.93	0.938
32797838.51	11428839.2	21653829.38	21103012.17	1.588
1186009518	1234189253	1464139112	1385739548	1.125
3288726.563	2159215.117	3978514.75	169199.7378	0.973
936360.9335	2175923.048	2014622.391	2379833.158	1.06
1606617.21	4754109.63	1384229.875	8090297.613	0.915
3632044.135	4007732.969	1305444.625	924575.9756	1.691
241329.3766	132663.8093		125529.6442	1.05
2769697.762	4317669.31	3960443.75	4343700.334	0.823
1178206.549	2078194.524	1918187.88	1388177.174	0.842
151550.1735	211805.5721	314809.5625	264418.6837	
4214081.279	1965370.394	5129323.656	4984878.096	0.715
7198280.932	12997570.18	6875049.344	11016248.9	1.003
401178.7627	708079.9501	738842.25	502977.4385	1.275

454196911.6	251950996.2	274785120	140766092.1	2.002
4974561.191	9256987.713	3627867.906	4996770.095	1.6
1429603.435	5094035.445	1757729.125	2371551.597	0.84
608194.6394	147050.0335	234554.5	206258.3574	0.278
1594869.048	3868434.415	2680586.25	4458106.783	0.989
601699.1247	200554.3293	464945.5313		1.452
1282297.978	6411195.75	5762025.844	5112827.595	0.525
1487921.194	1813488.36	975806.125	1917389.729	0.972
1919821.322	1002333.986	5102166	2089487.889	2.889
1286781.927	5752937.835	3865748.5	1507435.948	0.509
2112981.931	4739847.156	3279845.156	4144909.742	1.022
	1022798.809	157761.25		
2593389.495	2757941.337	1814707.25	2853043.201	0.965
				1.061
34312622.1	39190442.57	28977496.31	10617323.91	1.164
25898921392	25908843452	38232738541	39787671500	1.234
454551.2279	480160.5532	374158.1875	639132.8759	1.049
	412448.9024	527013.125	383657.1742	0.917
5457382.435	11742721.31	12823023.56	5062683.823	0.948
864471.4356	2406205.265	1870343.125	1351143.473	0.749
519739.7962	1137761.5			1.26
137534849.4	151725943	177763721.8	196090569.2	1.171
7444567.505	13316694.21	13918975.28	15106975.51	0.929
2689053.286	2189011.865	2530941.813	700663.5772	0.875
3239275.452	878158.6795	11034154.34	12302896.28	1.022
60170302.06	56816850.29	45970387	62945432.5	1.312
110116394.2	89822885.01	176443202.7	58567515.59	1.042
15648361.2	19810439.99	16556024.72	19784333.43	0.804
5122695.292	1955713.737	1798283.906	1723628.939	1.63
630424.6562	8596127.513	1405172.5	6167059.063	0.581
1536953.547	5338964.358	5749763.625	2352596.809	1.263
658798.9998	1349919.129	682296.2969	792306.2808	0.823
406205.3835	162412.5261	847237.8125	636550.8301	0.891
680339637.1	791191294.9	727187995.5	682784253	0.785
535490.9294	827585.746	619556.3125	534379.3513	1.155
2100045.919		641270.875		1.385
1249995.346		1005782.813	879394.1859	0.506
222377.8625	699906.5081	434439.3125	469709.6924	1.862
15996463.48	40259810.8	23730152.18	25863575.13	1.239
60772.21674	407850.4807	148870.3281	127319.8636	0.668
2457402.106	363611.3232	2602120.41	1632331.604	1.886
2285977.623	2996752.379	5101402.75	8273338.036	0.744
617307.7241			1005802.048	0.439
1613732.214	2126783.698	2329227.75	1575451.055	1.087
1260458.884		825507.375	1444653.695	0.863
41662215.6	24970467.31	22367396.19	20754059.41	0.967

	560453.3655	377863.0625		0.668
	241197.506			
				0.545
4146496820	2491093659	3614303732	3932323492	0.916
2205869.572	890696.2076	1479788.938	377855.1385	0.782
1123112.71	3956851.7	3267159.359	7331708.161	0.505
13939494.52	14518912.99	11950061.91	9615209.712	0.918
367027.4618	1239160.283	736353.9063	1708937.988	0.999
1581664.588	331710.678	387593.6563	1965378.122	1.223
59257729.37	49029994.4	17159783.31	11919310.12	0.671
	450688.6263	1496164.875	1372487.333	0.156
13172393.57	32694089.99	20715577.53	34839323.62	0.757
34527964.02	45197551.79	49866951.08	61573184.55	0.802
4744671.715	20716425.72	10630898.63	18762290.57	1.057
21603297.15	31684534.89	31064957.66	61700463.88	1.114
179637.7302	390033.7014	213913.3438	209321.1196	1.073
646105152.8	1035080729	301025076.8	299392278.9	0.396

N_LGR_2	N_SFM_3	N_LGZ_4	N_ZXL_5	N_LKL_6
			0.888	2.089
1.056	0.801	0.958	0.95	0.712
1.126		1.057		
				2.232
0.532	0.587	0.784	1.208	0.767
0.574	2.129	0.917	0.165	1.363
	0.206	1.326	1.463	1.814
1.583	0.521	0.961	0.992	1.536
1.309	2.157	1.437	0.519	0.858
0.712	0.691	0.949	0.827	1.652
0.934	1.143	0.788	1.467	1.06
0.499	0.528	1.012	1.614	0.895
1.215	0.692	0.831	0.963	1.046
0.587	0.475	1.09	2.338	1.574
0.909	2.061	0.597	0.265	1.029
	0.04			
	1.952	0.938		
1.14	2.537	1.21	0.524	1.278
1.038	1.377	0.687	1.636	0.62
0.107		0.44	0.299	0.241
0.938	1.404	1.296	2.154	1.127
1.185	0.781	1.287	1.361	1.071
1.344	0.242	0.918	1.13	1.413
0.456		2.288		1.013
1.103	1.378	0.835	1.398	0.722
			0.025	0.008
1.113	1.43	1.091	0.781	0.877
1.185	2.148	0.379	1.273	0.667
1.23	1.55	0.872	1.508	0.923
1.09	1.52			0.254
0.571		0.974		
0.883	0.482			
0.545	1.355	0.219		
0.739				
1.32	0.651	0.615	0.563	1.293
0.99	0.776	1.463	1.211	0.785
0.505	0.76	2.13		1.626
1.607	1.021	0.886	1.41	1.024
0.431	0.986	0.304	1.071	2.659
1.251	0.515	1.551	3.581	2.798
1.25	1.411	0.991	1.811	0.948
1.496	1.484	0.851	2.299	0.942
0.885	2.854	0.786		0.987
		1.517		0.456

0.584	1.124	1.137	1.125	1.886
0.986	1.165	1.213		0.953
0.955	0.1	0.881	0.426	1.204
0.54	0.936	0.546	0.607	
				0.808
0.274	0.172	0.847	0.761	0.491
1.338	0.675	1.015	0.736	1.281
1	1.199	0.703	1.302	0.705
0.925	0.354	0.976	1.313	1.355
0.961	1.297	0.859	1.258	0.805
1.044	1.163	0.84	1.24	0.818
1.152	0.878	1.81	3.243	2.068
1.473	2.431		0	
		1.467		
1.116	0.841	0.806	1.21	1.73
0.836	3.613	1.045	1.008	1.186
0.973	2.594	1.595	0.159	0.752
1.031	1.555	1.495	0.42	0.788
0.942	0.654	0.457	1.269	0.481
1.073	0.661	1.984	1.556	0.551
0.003	0.008	0.003	0.006	
0.15		0.367	2.02	
1.979		1.017	2.326	0.464
1.251	0.462	1.103	0.632	1.593
0.645	0.394	0.529	0.263	0.207
1.865	1.068	0.872		0.864
1.248	1.457	0.925	0.606	0.431
1.67		1.151	1.058	0.872
1.945	1.232	0.882	3.099	
0.821	0.676			
		0.228	0.467	0.299
0.924	0.623	1.321	1.891	0.95
0.094	0.355	0.215	3.416	1.185
8.074	0.13	0.101	0.691	0.081
1.493	2.418	1.3	0.769	1.527
0.309	0.57	0.311	0.662	5.455
		0.824	2.629	
0.981	0.07	4.145	0.408	0.251
1.142		1.185	1.107	1.1
0.727				
0.208	3.004	4.05	0.593	0.217
1.825	0.759	0.781	1.501	1.112
3.235	0.805	2.998		

0.909		0.731		0.853
0.566		1.318		1.107
0.5		0.109		1.19
1.279	1.559	0.802	0.97	0.826
				0.595
1.47	0.895	0.829	1.916	1.362
0.8	0.757	0.711	2.584	1.165
1.416	1.006	1.578	1.524	1.891
1.21	1.086	0.749	1.217	0.843
0.899	1.321	0.985	1.536	1.52
1.322			1.441	1.07
0.921	1.287	0.373	1.308	0.941
1.201	1.167	1.025	0.66	0.849
	2.168	0.37		
1.199	0.654	1.175	2.221	1.821
0.841		1.076		1.571
1.266	1.532	1.094	0.556	1.01
0.526		1.59		1.05
1.027	1.199	0.95	1.444	1.134
0.784	0.626	1.13		0.597
		0.952		1.011
0.817	1.881	1.18	0.686	0.475
1.248	0.771	0.766	1.102	0.881
0.463	0.993	1.629		1.475
1.543	1.291	0.829	1.115	0.796
0.78	0.327	1.155		1.306
1.03	0.995	1.312	1.546	1.243
0.436	1.134	0.685	1.775	1.658
0.882	2.995		0.749	0.979
1.23	1.827	0.626	2.26	
0.595	2.067	0.946	0.841	0.38
0.821	0.606	1.422	0.8	1.083
1.096	0.841	1.01	1.584	1.338
1.057	0.761	0.934	1.072	0.727
0.514	0.564	0.801	0.847	0.491
1.208	1.672	1.203	0.504	1.229
1.442	1.419	0.555	1.65	0.855
0.437	0.67	1.337		1.932
0.099	2.127	0.809	0.988	1.208
0.938	1.864	1.097	1.361	1.603
1.169		0.768		
1.165	1.14	0.626	1.202	0.944
0.901	1.342	1.613	0.725	0.683
1.512	0.387	0.843	1.355	0.647
1.53	0.289	0.492	1.073	0.944
0.863	0.291	0.739	0.146	1.442
0.883	0.76	0.977	1.601	0.904

	2.931	0.436	0.321	0.332
0.847	1.088	0.96	0.01	1.589
1.106	1.447	1.202	1.06	0.696
0.165	3.255	0.625	1.093	1.067
				1.254
0.619	2.699	1.288	1.014	0.747
1.251	0.819	1.37	1.227	0.861
0.851	0.657	0.874	0.964	1.011
		1.564		
1.369	1.209	0.626	1.132	0.702
		0.449		1.124
1.134	1.27	1.092	1.244	0.754
0.686	0.564	0.373		0.451
1.563	0.912	0.617	1.093	0.989
1.098	1.651	1.049	0.586	1.07
1.36	0.536	1.943	1.057	0.809
1.323	0.087	0.937	0.249	1.857
0.416	0.121	1.448	0.364	2.045
1.279	1.102	1.26	0.56	1.066
1.524	0.411	1.378	1.807	1.8
1.522	0.736	0.66	1.193	0.503
1.486	1.194	1.182	1.009	1.018
1.278	1.596	0.558	1.06	0.656
0.632	1.67	1.894	0.834	1.053
1.154	1.568	1.03	1.002	1.016
1.154	0.698	1.318	0.622	1.124
0.373	0.508	0.702	1.776	1.678
1.305	0.875	0.489	1.462	0.945
1.582	2.313	0.889	0.879	1.779
0.629	1.503	1.327	1.938	0.715
0.801	1.06	1.065	2.115	0.981
1.238	0.343	1.211	0.272	1.037
0.707	1.179	1.094	1.596	0.579
0.846	1.277	1.541	0.782	1.434
0.517	0.69	1.811	0.789	2.048
1.61	2.509	0.753	1.211	1.109
0.487		1.959		1.35
1.042	0.916	1.065	1.016	0.776
	1.76			
1.024	0.099	0.869	0.899	1.165
0.724		1.258		
0.896	2.098	1.159	0.66	0.893
0.92	1.958	2.101	1.024	1.886
1.204		1.01	1.657	1.388
0.736	0.331	0.977	0.355	0.618
1.003		1.248		0.902
1.625	0.307	0.503	1.077	0.588
1.044	1.886	0.868	0.729	0.925
1.266	1.151	0.739	0.632	0.918

0.832	0.022	1.588	0.042	1.149
0.675	0.138	1.609		1.647
1.015	1.056	0.894	1.22	0.802
0.661		1.152		0.403
0.37		1.007		2.185
0.895	1.707	1.859	1.326	1.736
0.879	0.446	1.297	0.579	1.82
3.917	0.888	0.436	0.543	2.255
0.279	0.63	0.845	6.357	7.22
0.949	2.571	1.111	0.794	1.047
1.254	0.143	0.897	0.799	1.623
		0.932		
1.691	1.605	0.734	1.578	1.548
	1.407			
0.444	0.681	0.807	1.716	1.326
1.621	0.524	1.606	0.577	1.126
0.79	0.22	1.065	1.201	0.601
0.201		0.577		2.826
	2.504	0.901	1.228	0.996
0.876		1.313		1.208
1.787	1.008	0.556	0.869	0.506
0.584	0.368	1.138	1.804	0.903
1.128	1.026	1.616	1.151	1.312
1.214	1.607	0.799	1.948	1.047
0.786	0.837	0.694	1.25	0.518
0.704	1.524	1.762	0.567	1.69
0.636		0.767		1.035
1.119	1.088	0.842	1.237	0.84
1.203	4.065	1.857	0.145	0.363
0.366	0.944	0.892	0.853	0.531
0.069		0.081	0.116	1.611
1.059	2.437	1.227	0.925	1.367
	0.464			
0.802	0.846	0.955	0.892	0.738
0.799	0.71	0.46	0.213	
1.524	1.486	0.734	0.844	0.63
0.93	1.364	1.366		1.293
1.195		1.008	0.747	1.139
0.971	1.076	0.948	1.154	1.382
0.779	0.149	0.84	1.037	0.855
1.094	0.699	1.005	1.789	0.741
1.211	0.815	0.89	1.498	0.983
1.004	0.654	0.996	0.691	1.33
0.464		1.031		0.808
0.974		0.504	0.988	0.176
1.058	1.777	1.268	0.946	1.436
0.522				

1.126	1.467	1.039	1.826	0.829
1.213	1.085	1.95	0.834	0.827
0.84	1.077	0.704	1.966	1.5
0.966	1.089	0.794	1.902	1.183
		1.442		0.587
1.719	0.977	2.104	0.609	0.935
0.983	0.855	1.27	0.89	1.229
0.662	0.183	1.039	0.638	0.998
0.961	0.284	1.336	0.817	1.347
0.983	1.047	1.136	1.524	1.144
1.278	2.778	0.517	0.824	0.655
0.174	0.901	1.478	1.794	0.716
1.298	3.217			
1.127	0.685	0.612	0.958	0.495
0.094	0.14	0.86	0.279	0.902
1.092	3.095	1.385	0.53	0.971
	0.649	1.26		0.32
0.789	0.44	0.521	3.301	0.978
1.578	0.068	0.953	0.848	0.907
1.206	1.892	1.287	0.726	1.115
0.389	0.572	0.395	4.574	0.936
	1.198		1.274	0.4
0.42	0.176	2.799	0.227	0.268
0.176	0.569	2.055	1.722	1.31
0.515	0.35	1.253	0.54	0.699
0.799	1.174	1.319	0.181	0.458
	1.45		3.291	
1.132	0.665	0.947	1.197	0.808
0.753	1.426	1.327		
0.94	1.892	0.654	3.106	1.408
1.279	0.908	0.799	1.244	0.978
0.938	1.521	1.65	0.799	1.533
0.663	0.658	0.519	0.723	0.621
0.133	0.101	0.39	0.116	7.625
0.105	0.143	0.611		
	0.98	0.648		
	1.484			
0.971	0.906		0.711	0.541
1.078	1.36	0.815	1.43	0.968
	0.569	0.542		0.772
1.411	0.43	0.874	1.021	0.706
0.725	1.312	0.673	2.306	0.775
1.247	1.984	1.616		1.047
1.373	0.578	1.473	1.944	0.788
	0.251	0.633		0.425
1.018	1.064	1.319	0.864	0.925
1.405	0.667	1.386	1.677	1.729
1.132	1.339	1.797	1.375	1.904
1.077	2.624	1.256		0.629

1.803	0.323	1.439	0.237	0.623
1.251	1.22	1.088	1.149	0.987
0.731	0.567	0.971	1.009	0.892
0.892	0.461	1.086	0.614	0.093
1.215	1.253	0.758	1.161	0.954
0.944	0.512	1.395	0.367	1.255
0.639	2.037	0.175	2.683	0.129
1.24	0.263	0.534	0.641	
0.976	0.635	0.872	0.776	1.04
0.526	1.213	0.45	0.75	0.505
			0.736	
1.838	0.439	1.029	0.718	0.938
1.144	2.788	1.191	0.895	0.92
0.792	0.93	0.976	0.597	1.69
	2.365	2.408	0.412	
0.574	0.841	1.296		1.812
0.457	0.911	1.073	2.039	1.303
1.026	0.203	0.64	1.112	0.81
1.043	1.989	1.349	0.858	1.282
0.999	0.195	1.242		1.364
1.303	0.797	0.526	0.706	1.088
	0.559			
0.982	0.554	1.374	2.213	1.176
1.004	2.577	0.874	0.975	1.301
0.911	0.565	0.895	0.816	1.299
0.897	0.867	1.329	0.593	1.319
0.799	1.241	0.494	0.695	2.57
1.065	1.303	1.193	0.672	1.215
1.539				
1.022	1.944	1.046	0.944	1.027
1.398	0.951	0.733	2.213	0.771
1.467	1.059	1.073	0.701	0.881
0.616	3.03	0.586	0.739	0.725
1.431	0.193	1.075	0.535	1.231
0.808	0.988	0.952	0.728	0.894
0.926	1.995	1.064	0.936	0.805
0.606	1.487	0.788	2.331	0.734
0.771	0.527	0.516	0.423	
			4.693	0.761
1.027	1.519	1.643	0.832	0.838
1.162	1.584	0.946	1.354	0.666
0.974	1.594	1.026	0.805	1.833
1.056	0.476	0.769	0.402	0.794
0.503	0.504	1.163	1.411	0.761
1.776	1.013	0.682	0.611	0.738
	0.918			
0.999	0.697	0.636	3.347	0.82
0.719				

0.601	0.916	0.777	0.617	1.704
		0.773		1.251
0.914	1.957	0.539	0.525	1.482
0.938	1.154	1.178	1.326	1.278
	1.277	1.56		0.492
0.636	1.007	0.661	0.792	0.816
		0.176	0.852	
0.815	1.147	1.258	1.137	1.072
0.739	1.462	0.764	1.929	1.183
0.295	0.236	2.252	2.084	2.052
1.208	0.259	0.649	0.96	1.154
1.188	2.074	0.875	0.939	0.707
0.639	1.399	1.384	0.833	0.654
1.068	1.172	0.655	1.532	0.8
1	1.005	1.12	1.28	0.96
1.643	1.036	0.514	0.591	0.892
0.994	0.536	1.278	0.746	1.303
1.114	1.17	0.667	0.454	0.933
0.614	1.303	0.405	6.759	0.76
1.088	0.996	0.602	1.363	1.143
0.724	2.293	1.692	0.571	1.278
0.254	0.051	0.662	0.78	0.063
0.719		1.734		3.174
0.9	2.612	1.587	1.168	1.536
1.086	1.1	0.802	1.854	0.765
1.088	1.239	1.361	3.508	0.824
	2.759	1.869		0.462
0.298	0.582	0.519	0.947	0.446
1.47	1.338	0.685	0.272	0.525
0.243	0.83	2.148		2.063
		1.699		1.283
0.521	0.348	1.292	1.517	1.388
0.853	0.419	1.241		0.584
1.066	1.024	1.193	1.305	1.029
1.384	2.075	1.112	0.508	0.959
1.624		1.081	1.166	1.995
1.143	0.262	0.427	0.72	1.278
1.35	1.175	1.701		1.968
0.822				0.699
1.817	0.698	1.046	1.034	0.942
	0.971	0.413		
0.418		0.165		0.526
0.272	0.188	0.159	0.212	0.215
1.05	0.854	1.382	0.903	0.947
1.252	0.941	0.998	1.359	0.925
0.698	0.742	0.888	1.439	0.745
0.857	1.147	0.636	1.346	0.603
0.979	0.889	1.304	1.592	0.945

1.085	0.816	1.077	0.967	0.84
1.041	1.69	1.031	0.486	1.426
0.817	0.332		0.994	
1.516	1.512	1.572	0.188	0.739
0.768	0.341	1.49	0.378	1.139
0.868		1.083		
0.761	0.022	1.706	0.355	1.357
0.827		1.08	1.9	1.819
0.747	0.871	0.823	1.052	1.363
1.251	0.846	1.226	1.367	0.948
1.739	1.843	0.447	1.014	0.465
0.987	0.285	1.273	0.671	1.104
1.278	0.796	0.791	0.857	0.748
0.545	0.332			
1.107	1.425	1.404	0.815	1.609
1.319	1.039	0.91	1.364	0.952
0.962	1.627	1.266	1.154	1.962
	1.061	1.016		
0.961	0.972	1.081	2.097	1.235
1.12	1.667			
0.5	0.664	0.432	0.312	0.234
0.204	0.384	0.13	2.116	0.241
1.269	0.61	0.955	0.456	0.991
0.962	1.064	1.092	1.448	1.128
1.149	0.478	1.303	1.002	0.939
1.446	0.479	0.323	1.068	1.377
1.081	1.451	0.94	0.948	1.137
0.445	0.233	1.649	0.541	1.246
1.057	0.994	0.758	2.317	1.278
	1.393			
0.713		0.968		1.073
0.223		1.124		0.611
0.98	0.788	0.955	0.578	0.723
0.424	1.827	1.031	1.411	2.453
0.507	0.465		0.844	0.898
0.608	1.736	0.713		
0.694	0.363	1.562	0.438	1.325
		0.321		
		1.191	1.736	1.569
1.517	0.58	1.27	0.578	0.708
0.875	1.164	0.902	1.68	0.67
1.07	1.431	1.532	1.436	0.827
0.981	1.058	1.24	1.32	0.73
0.583	0.647	1.382	0.653	0.947
0.249	2.191	0.446	0.213	0.322
1.346	1.799	1.539	0.478	
				1.474

0.902	0.377	1.229	0.334	1.043
0.967	2.333	1.111	0.954	1.222
0.742	0.297	0.726	0.344	0.213
1.033	1.277	1.312	1.272	1.219
1.098		0.59	0.631	
1.461	0.905	0.84	1.148	1.423
0.96	1.363	1.338	0.817	1.391
1.143				
0.421	0.787	1.441		0.534
	1.573	0.975		0.622
0.73	1.252	1.435	0.926	1.225
		1.401		0.956
0.121	0.176	1.559	0.266	1.402
1.507	3.268	1.205	1.048	1.518
0.008		1.515		0.83
1.153	1.301	0.939	0.663	0.777
1.015	1.447	1.595	3.031	2.087
0.87	0.905	0.744	2.036	0.75
1.664	1.684	1.103	1.534	0.861
1.27	0.765	0.819	1.13	0.802
		0.167	1.355	0.202
1.024	1.754	1.249	0.754	1.233
1.02	0.758	1.06	0.683	0.954
0.928	0.309	0.908		1.395
0.828	2.334	1.394	0.561	1.603
0.656	0.602	1.199	1.242	1.355
1.118	0.757	0.62	0.723	0.645
1.238	0.838	1.067	1.05	1.059
1.362	1.369	0.801	1.349	1.028
1.251	1.432	0.619	2.138	0.793
0.021	0.626	0.764	1.116	1.445
0.881	0.237	1.623	0.613	0.73
0.122	0.075	0.779		1.152
1.289	1.256	1.437	1.512	1.727
2.161	1.444	0.744	1.739	1.501
0.797	1.223	1.51	1.413	1.14
0.697	0.159	0.583	0.494	0.497
		0.526		
0.76	0.921	1.574	1.266	0.836
1.074	0.438	1.02	0.637	0.879
0.585	1.194	0.828	1.01	1.074
1.066		0.576		
0.896	0.377	1.419	0.641	1.014
0.973	2.34	1.198	0.751	0.959

1.061	0.868	1.165	2.082	2.324
0.838	1.184	1.263		1.37
1.27	1.501	0.636	1.034	0.671
1.069	0.224	1.033	1.241	1.399
0.493	1.037	0.096		4.899
1.458	0.269	0.867	0.491	1.251
1.306	2.513	1.143	1.178	0.185
0.212		0.98	1.363	
0.997			0.939	0.586
0.888		1.043		1.677
0.382	1.052	0.746	0.407	3.573
1.136	1.525	0.841	0.769	0.887
1.417	1.396	0.747	0.886	1.034
		0.678		0.57
0.838	0.998	1.499	1.207	1.581
1.148	0.938	0.564	1.21	1.009
0.712	0.09	2.086		
0.96	2.558	1.414	0.836	0.954
0.88	1.438	1.307	0.691	1.167
1.026	0.462	1.113	1.147	1.038
0.395	0.357	0.969	1.783	1.403
0.719	0.201	0.773	1.029	1.005
	6.536	0.473	0.672	0.795
1.244	0.722	1.299	1.36	1.13
		0.342	0.606	0.865
		2.747		
3.325	1.515	0.93	0.723	0.809
1.176	0.542	1.291		1.166
1.362	2.704	1.201	0.531	0.32
1.085	2.028	0.994	0.692	0.882
0.742	1.071		1.529	
0.998	1.351	1.18	0.549	0.471
1.273	0.898	0.768	1.796	0.933
0.555	1.099	0.967	1.884	1.949
1.334	0.602	1.013	0.867	1.052
			0.835	
0.58	1.189	1.47	0.292	1.067
1.017	1.777	1.168	1.445	1.107
	0.945	0.07	1.657	1.145
1.493	1.454	1.645	1.112	0.933
1.758	0.317	1.322	1.027	0.619
1.284	0.686	0.818	0.426	0.576
0.791	0.827	0.865	1.436	8.131
1.367	1.432	1.308	0.651	1.165
1.051	0.519	1.596	0.816	1.023
1.972	1.74	1.144	1.563	0.665
0.469	2.806	1.051	0.802	1.032
0.463	2.632	0.731	1.113	2.086

		0.29		0.555
0.839	0.308	1.447	0.44	1.391
2.174			0.351	2.017
0.419	1.441	0.601	0.726	1.113
1.457	1.273	0.773	1.472	0.989
1.045	2.276	1.086	0.624	0.998
0.809	0.645	1.078		1.069
1.044	1.971	1.372	0.841	1.632
0.222		1.692		0.312
0.96	0.12	1.08	0.214	0.546
1.017	1.239	0.858	1.187	1.198
0.838	0.747	1.188	2.302	1.99
1.28	0.692	1.359	0.612	1.971
0.156		1.844		
1.162	1.072	1.117	1.103	1.222
		0.707		
	0.372			
0.803	1.932	1.355	1.917	1.364
1.422		1.654	0.635	1.636
			0.644	
1.987	0.009	1.99	0.893	1.577
0.978	0.564	1.409	0.557	1.373
0.852	2.088	2.098		1.479
1.384	0.661	0.646	1.117	0.767
1.058	0.862	1.887	2.134	1.946
0.936	0.026	0.812	0.745	0.684
1.148	0.361	1.285	0.601	1.016
1.102	1.054	1.638	1.434	1.415
1.206	0.062	0.598	1.28	1.038
0.674	0.291	0.614		0.206
		0.922		0.725
1.471	1.108	1.154	1.313	1.328
1.037	2.533	0.946	0.626	1.035
1.144	1.138	0.769	0.936	0.738
1.039	0.583	1.107	1.237	0.801
0.42	0.348	0.182	0.556	0.372
	1.004	1.401	0.95	1.097
0.76	2.882	1.135	0.645	1.097
				1.339
2.901	1.204	1.264	1.062	1.23
0.971	1.016	1.379	1.553	1.747
3.257		0.316		1.654
1.216	2.244	1.244	0.55	1.132
1.254	1.389	1.107	1.626	1.16
0.518	1.308	0.292	0.229	0.327
1.209	0.939	1.02	1.845	1.197

0.865	0.404	0.872	1.565	1.653
0.213	1.056	1.024	1.09	0.885
1.049	1.135	0.86	1.355	1.273
1.259	1.232	1.129	1.761	1.562
1.168	0.615	1.633	1.012	0.654
0.904	1.571	1.115	1.591	0.659
0.907	1.2	0.785	1.683	0.758
0.753	0.64	0.711	0.779	
	0.862	0.833		
0.841	0.708			
1.064	0.974	1.48	2.01	1.36
1.256	1.571	1	1.708	1.129
1.691	0.334	0.294	2.324	0.88
		1.581		
0.655	0.554	0.203		0.359
0.886	0.744	1.166	0.949	1.009
7.384	1.337	1.25	1.836	0.836
	1.331	0.306		
1.005	0.002	1.617	0.46	2.043
1.75	2.415	0.325	0.537	1.73
1.085	0.997	0.723	1.394	1.28
0.778				
0.749	0.51	0.637	0.986	2.968
0.97	1.427	0.983		0.269
1.428	2.873	1.457	1.161	0.44
0.992	0.848	1.269	1.162	0.792
0.886	1.555	1.348	0.815	1.017
2.831	1.643	1.403	1.545	1.272
1.193	1.566	1.018	1.876	1.119
1.093	0.838	0.932	0.84	0.845
1.38	1.706	1.241	1.048	1.251
	0.559	0.843	1.849	1.319
0.447				0.66
		1.183		1.555
0.767	0.501	3.786	0.302	0.674
	3.054			1.594
0.855	1.585			
1.191		1.041	2.244	2.181
0.841	1.571	1.17	0.582	1.003
1.37	1.377	0.729	1.276	0.956
1.198	1.344	0.985	1.631	1.718
0.89	0.043	1.232	0.303	0.819
0.472	0.515	0.515	0.885	0.117
0.944	0.709	0.492	1.992	1.75
2.341	0.086	0.073	0.713	3.224
1.002	0.684	0.974	1.245	2.172
1.348	3.503	0.973	0.983	1.049
1.72	1.218	1.556	1.995	1.802

0.625	0.687	1.329	0.516	1.945
1.078	0.763	0.599	0.954	0.596
0.941	0.992	1.2	1.369	1.584
	2.271	0.988		1.174
1.811	0.767	1.063	1.24	2.324
0.956	0.208	1.092	0.554	0.854
		0.64	2.041	0.989
1.06	0.354	1.31	1.076	0.849
1.389	0.521	0.8	0.766	0.392
		1.07		
1.505	0.572	0.047		
1.265				
0.988	0.795	1.222	2.722	1.488
0.651	0.708		0.892	
0.504	0.94	1.437	3.824	0.684
1.083	1.163	0.916	0.907	0.804
0.89	1.284			
1.473	0.248	0.766	0.291	0.815
	3.274	0.876		0.232
0.668	1.123	0.815	1.023	0.674
1.251	0.325	0.749	2.501	1.145
0.321	0.625	0.545	0.867	0.55
0.294	1.934		1.071	
0.959	0.11	0.473	1.2	0.566
1.037	1.459	1.632	0.506	0.638
1.383	0.832	0.977	0.863	1.01
1.314	0.899	0.474	1.692	1.076
0.617		1.146		1.133
0.963	1.564	1.157	0.778	1.465
1.266	0.437	1.494	0.431	0.884
	0.072			
2.319				
	0.172			
		0.767		
		0.564		0.825
3.253		1.935		0.579
0.862	1.29	0.642	1.354	1.017
1.091	0.601	1.008	0.701	0.728
		1.324		0.865
1.052	1.706	1.211	0.844	1.559
0.78	0.659	1.2		
1.061	0.399	1.514		1.1
0.953	0.516	1.364	2.352	0.376
1.082		0.84	0.257	
1.156	0.985	0.81	1.807	1.111
0.52	1.158	1.093	0.972	2.379
1.492	1.186	1.048		0.469

0.545	0.475	1.216	1.736	0.52
0.745	2.081	0.902	0.794	0.992
1.344	0.751	1.269	0.965	0.31
1.706	1.127	0.946	1.347	1.395
1.297	0.277	1.211	1.601	1.443
0.685	1.39	1.788	0.999	1.291
2.425	0.966	0.803	0.059	2.011
1.172	1.147	0.573	2.426	1.243
1.839	1.179	1.72	1.213	1.848
			1.498	0.674
0.96	1.16	0.853	1.219	0.985
0.908	2.061	1.961	0.427	1.01
0.701	0.114	1.11	1.464	1.604
				1.038
1.048	1.42	1.762	0.905	1.628
0.575	0.265			
		0.887		0.713
1.777				
0.721	1.528	0.532	0.348	0.629
0.957	1.679	1.043	0.64	1.084
1.361	0.421	0.096		
1.021	0.579	0.748	0.848	0.677
0.74	1.654	1.32	1.002	1.809
0.939	0.78	1.277	0.504	1.406
0.923	2.45	0.89	0.945	1
0.601		0.821	0.711	1.044
0.772	1.588	0.995	1.632	1.145
1.023	1.04	1.499	0.885	0.83
	0.497			
	1.863	1.681	1.607	2.322
				2.256
1.186	0.971	1.56	1.334	0.57
		0.815	1.441	0.769
1.092	1.637	0.645	1.563	1.557
0.626	7.26		0.448	
1.052	1.442	1.553	0.918	1.197
0.598	1.867	1.4	0.962	0.924
1.454	0.23	1.42	0.406	0.895
1.265	1.318			1.119
1.292	0.768	0.91	1.179	0.95
0.791	1.591	1.336	2.631	1.101
1.424	0.168	0.766	0.899	1.486
1.197	0.983	1.66	1.1	1.558
1.863		1.201	2.416	1.376
			1.119	0.988
0.436		2.332		

	0.572	1.608	0.721	0.567
0.752				
0.978	2.546	1.292	1.003	0.386
1.083	0.447	1.335	1.199	0.89
0.84	3.963	0.44	0.517	0.213
0.734	0.708			
0.875	0.551	0.885	1.239	0.968
	0.711			
1.444	0.531	2.059	0.692	0.973
0.7	1.861	0.898	1.365	1.081
	2.22			
0.299	3.143	1.204	0.215	0.209
0.74	1.634			0.485
0.967	0.635	0.736		1.038
0.788	0.647	1.021	0.672	1.258
1.129	0.476	1.305	1.443	1.518
1.256	2.13	1.068		1.249
1.3	1.062	0.704	0.793	0.879
0.899	2.471	0.77	1.092	0.571
1.332	2.182	1.519	0.614	1.186
0.951	1.002	1.711	0.576	1.122
0.959	0.973	0.649	0.667	0.654
	1.716	0.501	3.101	1.117
0.812	0.788		0.969	0.835
1.31		0.7		1.102
1.112	2.019	1.317	1.32	1.42
	1.845			1.233
1.117		1.447		
1.284	0.113	1.262	0.505	0.55
1.163	0.391	1.933		0.193
1.353	0.821	0.928		1.119
1.438	1.302			1.039
		12.063	0.048	
1.104	1.813	1.551	0.373	1.5
	0.747	1.411		
0.968	1.63		1.65	0.586
0.991	2.133	0.72	1.754	1.021
		1.125		
0.61	0.523			
0.485	0.113	1.099		0.311
0.849	1.62	1.169		0.944
0.775		1.342		0.708
0.447	1.524	0.879	1.86	1.174
0.986	0.654	1.205	2.36	2.031
0.211		1.861		0.493
2.114	2.108	1.018	0.43	0.861
0.914	0.399	1.112	1.256	2.886

0.896	1.682	1.838	0.327	1.335
0.981	0.533	1.484	0.727	1.552
	0.982	0.997	1.638	1.123
	1.265	0.918		0.806
1.286	1.247	0.639	1.612	0.889
			0.699	0.977
1.356	1.505	0.838	1.395	1.392
	3.232	0.598		0.837
0.41		1.682		1.511
1.276		0.894	0.67	0.832
	0.846	1.466	0.21	1.462
0.26	0.482	2.043	1.341	1.456
		0.693	0.39	0.956
0.605		0.595	0.451	0.587
1.487	1.479	0.598	1.331	0.934
1.056	1.252	1.102	1.732	1.208
		0.835		1.097
1.387	1.001	0.447	0.433	0.679
1.567	0.128	3.092		0.76
2.332	0.181	0.224	3.958	3.155
0.958	0.868	0.945		0.926
0.903	0.074	0.837	0.129	0.148
1.219	1.11	1.041	0.95	0.976
1.04	0.689	1	1.301	1.143
0.761	1.256	0.847	0.764	1.093
1.215	0.204	0.668	0.437	0.959
1.453	1.972	1.324	0.384	1.167
1.126	0.574	0.88	1.018	0.615
		4.89		
2.137	2.235	0.6	0.82	2.597
1.329	2.168	0.785	1.063	1.368
0.687		1.23		1.243
0.536	0.574	0.93	0.399	0.735
1.176	0.929	0.91	2.524	1.198
1.36	2.529	0.842	2.457	0.995
0.661	1.523	0.746	1.442	0.638
0.161	0.43			1.086
0.935	1.682	2.092	1.05	1.186
	0.664		0.586	
0.407				
		1.047		0.898
1.151	0.466	1.419	0.771	1.103
1.525	1.291	0.986	1.517	1.14
0.662	2.101	1.303	0.635	0.859

1.312	1.211	1.343	0.449	0.863
	1.732			
1.46	0.263	0.706	1.26	0.92
1.322	1.078	1.38	1.501	1.445
		1.556		1.711
1.183	1.191	1.264	0.612	1.272
0.907				
0.55	0.517	1.279		0.977
0.414		0.915	1.001	0.886
1.088	2.157	1.016	0.617	0.932
		0.43		0.438
0.791	0.832	0.877	1.502	0.893
0.781	0.291	0.939		0.637
1.106	1.322	0.34	0.765	0.65
	0.832	0.834	0.495	0.66
0.839	1.126	1.343	0.896	1.628
0.293	0.052	2.921	0.621	0.774
	0.264	0.21	1.446	1.548
0.772	0.351	1.637	0.397	1.284
1.119		0.859		0.743
1.144	1.367	0.979	1.125	1.012
	0.366	0.651		
1.492	2.289	1.063		
0.801	0.332	0.562	0.239	0.162
0.768	0.495	0.71		
0.639	0.6			0.733
0.825	1.551	1.12	2.51	1.133
0.76		1.667		
1.33	0.448	0.969	0.45	1.378
0.666	1.338	0.466	1.237	0.867
0.861	0.353	1.477	0.763	1.84
2.186	0.346	1.015	0.726	
1.148	0.918	1.117	0.89	0.88
0.805	0.282	1.141	1.264	1.116
0.831	1.137	0.917	0.409	0.651
0.797	0.853	1.262		
0.186	0.175	0.744	0.665	1.06
0.734	0.376	0.678	0.996	0.676
0.703	1.095	1.05	1.403	1.176
1.076	1.013	0.751	0.798	0.745
1.212	0.37	0.952	1.145	0.847
0.87	0.744	0.31	2.231	1.027
0.721	0.562	0.953		1.272
0.955		1.56	2.583	0.963
1.338	1.698	0.946	0.596	1.267
1.023	1.315	0.915	1.145	1.024

0.559	0.087	1.439	0.047	1.133
1.459				
1.023	0.111	1.971	0.948	1.35
0.924	1.513	1.317	1.18	1.106
0.619		0.901		0.742
	0.54	1.033		
		3.12	0.091	
1.3	1.13	1.218	0.648	0.86
0.944	0.907	0.696	1.342	1.007
0.653	0.722	0.93	0.507	0.651
0.501	0.786	1.475		0.419
0.741	1.403	1.004	4.697	0.819
0.882	2.005	1.4	0.846	1.219
0.996	0.581	0.748	1.597	0.957
0.919	1.387	1.611		1.282
0.943		0.989	0.28	0.625
1.712	0.572	0.798	0.954	0.931
1.293	0.741	0.964	1.499	1.448
0.862	1.965	1.005	0.727	1.168
1.105	0.681	0.61	0.955	0.499
2.414	2.4	1.844	1.124	0.283
0.108		0.249	1.147	1.035
1.146	0.902	0.747	2.388	1.284
1.186	1.128	0.047	0.813	0.787
		1.269		0.579
2.401	1.485	1.286	1.899	0.888
0.361	0.237	1.372	0.65	1.252
0.978	2.101	1.42	0.969	1.059
1.145	0.314	1.012	1.084	0.933
1.226	0.248	1.177	1.456	1.557
0.793	0.906	0.72	1.299	7.433
1.865		1.379	0.796	1.009
1.36	1.633	0.812	1.273	1.076
0.366	1.148	0.332	6.188	1.312
1.19	0.28	1.275	1.976	1.333
0.674	0.353	1.273	0.247	1.282
1.378	0.431	0.429	2.372	0.938
0.661	1.041	1.061	2.282	1.454
1.129	1.23	1.056	1.122	0.856
1.532	0.846	0.46	1.667	
	1.307		1.174	
0.939	0.93	0.53	0.63	0.866
1.01	0.305	0.85	0.538	0.964
	1.3			
			0.324	0.277
0.54	2.582	0.29	0.533	0.233
1.28	1.664	0.92	0.7	1.04
1.098	1.156	0.877	1.089	0.873

1.212	1.32	0.701	1.135	0.561
1.1	1.624	1.595	1.517	1.143
1.572	0.925	1.185	1.506	1.358
0.725	0.574	1.5	0.628	1.026
0.819	0.388	2.403	0.684	0.749
1.45		1.114		0.269
1.68	1.94	1.151	0.631	1.267
1.081	2.437	0.953	1.131	1.532
1.376	0.194	0.189	0.234	0.681
1.228	1.449	1.082	0.488	0.674
1.413	1.905	0.835	3.067	0.801
0.219	0.289	1.004	0.261	10.934
0.957	2.166	1.576	1.357	2.176
1.326	0.624	1.331	0.548	1.355
0.954	0.25	0.277	0.478	0.534
1.123	1.492	1.429	0.638	1.157
1.264	0.852	1.249	1.759	1.072
0.682	2.095	1.795	0.594	1.688
2.331	0.384	0.414	0.574	0.349
	1.372	1.405	0.518	0.801
0.821	1.986	0.618	1.144	0.813
1.553	1.091	1.563	0.176	0.676
1.074	2.185	1.065	0.872	1.225
	0.63		2.905	0.711
1.283	1.448	0.79	1.019	0.945
0.711	0.225	2.161		1.39
1.907	0.286	1.753	1.181	1.226
0.362	0.221	0.399	5.102	
0.942	1.206	1.003	3.214	0.845
				0.661
1.201	0.786	1.016	0.311	1.075
0.094	1.659	0.175	2.161	0.19
0.994	0.171	1.48	1.229	1.362
2.075	1.164	0.833	1.808	0.945
0.489	0.865	0.914	4.561	0.601
0.937		1.647	0.713	0.948
1.021	0.278	1.159	0.722	0.815
0.529	0.335	0.198	0.548	0.548
3.936	1.008	0.9	0.913	0.89
	0.906	0.591	1.872	
0.463	1.639	1.728	0.798	0.583
0.82	1.917	0.815	0.877	1.141
0.215		0.955	0.794	0.834
	1.036			
		1.054		0.344
1.324	2.237	0.639	0.963	0.857
0.259	2.349	2.036		1.109
1.628	0.905	1.2	1.407	0.412

1.033	0.168	1.479	0.727	1.462
0.995	1	1.032	1.523	0.811
1.076	0.429	0.613	1.015	0.948
1.808	4.129	2.411	0.333	1.095
1.333	0.79	0.8	0.762	0.873
0.938	0.489	0.972	1.791	1.152
0.385	1.414	0.537	0.25	0.351
1.022	2.455	0.888	0.643	0.851
0.704	1.497	1.058	0.329	1.605
1.107	1.514	1.666	1.412	1.261
0.922	0.373	0.783	0.867	1.708
	0.698	0.3	0.544	1.871
0.379	3.471			0.42
0.069	0.739	0.374	0.199	0.204
0.258	1.371		1.68	0.41
1.034	1.075	1.246	0.759	1.214
1.507	1.562	0.748		0.442
1.41	0.684	0.582	0.75	1.118
1.295	1.425	1.084	1.197	1.026
0.555	1.716	1.602	0.406	0.751
0.932	0.611		0.506	0.778
1.03	0.969	1.226	4.497	0.61
1.354	0.337	0.97	1.154	0.947
0.538	2.182	1.477	0.449	2.088
0.779	1.575	1.029	0.119	0.966
1.235	1.131	0.846	1.059	1.091
1.207	1.379	1.235	1.43	1.106
0.176	0.12	0.223	14.712	13.001
		1.126		
1.416	0.919	1.111	1.088	1.116
1.376	1.092	0.641	1.294	0.877
0.846		2.116	0.4	2.56
0.79	0.138	1.179	0.696	1.262
1.13	2.755	1.116	0.469	0.813
0.567		1.037	2.666	0.551
1.089	0.555	0.598	0.555	0.833
1.022	1.833	0.962	0.539	0.861
1.955	1.579	0.474	1.364	1.184
1.488	0.563	1.39	0.734	0.78
0.787	1.509	0.713	2.27	0.272
1.135	0.289	1.099	0.811	1.036
0.534	0.379	0.505	0.473	0.497
1.121	1.126	1.072	1.581	1.128
0.941	1.735	0.738	0.854	0.682
0.934	1.321	0.742	0.882	0.699

0.751	0.302	1.036	0.804	0.74
1.169	0.072	0.959	0.768	0.732
1.116	2.386	1.057	0.344	0.817
1.303	1.605	1.607	0.48	0.821
1.456	0.813	1.435	1.924	1.243
1.013	1.986	0.841	0.848	1.176
1.3	0.81	1.037	1.152	1.09
1.486	1.198	1.542	1.716	1.522
1.22	2.044	1.276	0.609	1.057
0.151	0.734		0.254	5.337
0.821	1.111	0.706	0.922	0.781
0.739	1.054	0.94	1.418	1.451
	0.559	0.584	2.269	1.428
0.926	1.935	1.161	0.99	1.223
0.781	1.23	0.669	1.236	0.667
0.407				
1.043	1.608	0.898	0.406	0.531
0.831	0.956	0.671	2.432	0.716
0.983	0.335	0.584	1.287	
0.859	2.713			
0.397	1.012	1.648	3.184	0.256
1.217	0.351	1.097	0.644	0.832
0.687	0.037	1.224	0.336	0.985
0.979	0.725	0.959	0.734	0.891
0.769	2.348	0.95	1.908	1.352
0.454	0.816	0.274	1.11	2.768
0.673	1.238	1.055	0.814	0.849
0.675	1.102	0.791	3.191	
0.934	0.51	0.844	0.725	1.217
0.646	1.092	0.887	6.063	0.69
0.732		2.768	1.226	1.323
1.08	1.139	0.991	1.513	0.927
1.09	0.372	0.939	1.294	1.114
0.691	1.976	0.891	0.69	0.991
				0.327
1.132	0.906	0.848	1.189	1.052
0.935	0.763	1.793	0.585	1.288
0.826	0.152	1.617	0.888	1.212
0.965	1.214	1.329	2.02	1.318
	1.223	1.651	0.901	
		0.924	1.035	0.576
1.15	1.299	0.88	1.401	0.897
0.792	0.316	0.973		
0.158	0.095	0.442	1.793	1.232
0.649	0.227	0.539	0.358	1.605
0.242	0.381	1.817	0.801	0.545
0.878	1.158	0.931	0.766	0.569
0.397	0.723	1.329		1.641
0.934	1.233	1.38	1.11	1.329
1.169	0.473	0.887	1.012	0.89

		0.851		1.205
0.251		0.437	0.475	0.688
1.585	0.253	0.922	0.916	0.269
1.215	0.5	1.94	0.579	1.216
1.28	0.918	0.744	1.171	0.812
1.279	0.413	1.024	0.916	0.853
0.967	0.584	0.398	0.602	0.796
		0.558		
1.049	1.032	1.07	0.784	1.49
1	1.835	0.938	0.986	0.976
1.074	0.26	1.516	0.372	1.265
		1.455		1.374
0.733	0.501	0.538	0.485	2.266
0.983	0.31	0.514	1.02	1.078
0.519	1.102	2.037	3.559	0.546
1.03	1.57	0.934	0.808	2.247
2.333	0.482	1.337	0.844	0.75
1.434	0.991	2.03	1.643	0.992
0.824	0.516	2.433	0.729	0.906
1.346	1.574	1.135	0.418	0.488
1.491	0.234	1.1	1.397	0.847
0.852	2.999	1.137	0.846	1.532
0.897		0.868		0.652
	4.11		0.636	0.423
0.781	1.162	1.473	1.776	0.716
0.629	0.726	0.878	0.449	1.936
0.82	0.159	1.146	0.54	1.008
0.598	0.726	0.823	1.351	0.519
0.823	1.471	0.997	2.467	0.791
		0.432		0.394
1.289	1.93	0.957	1.361	1.118
1.217	0.861	1.009	1.751	0.978
0.75	0.2	1.772	0.748	1.18
1.047	1.132	0.881	1.29	1.022
1.199	0.696	1.11	0.957	0.982
1.406	0.409	2.614	0.177	0.069
1.157	1.118	0.93	1.447	0.973
1.184	1.219	1.469	1.254	1.011
0.532	3.337	0.783		
0.119	1.341	0.174	0.143	0.343
	1.799	1.203	0.927	1.681
1.029	1.282	1.086	1.156	1.75
0.471	0.157	1.544	0.455	0.812
1.288	0.564	1.369	0.787	1.215
0.488	0.908	2.353	1.577	1.852
1.524	1.383	0.584	1.676	0.986
0.552	0.563		1.573	1.949
1.1	2.245	0.216	1.199	1.269
	1.175	0.38	0.678	0.498

	1.886			0.658
0.385		0.902		
	1.204	1.09	0.594	0.994
1.313	1.04	1.09	1.044	1.293
1.165	1.205	1.134	0.519	1.291
1.067	1.006	1.265	0.603	1.029
1.584	1.163	0.671	2.135	1.009
0.688	0.118	0.967	1.199	0.889
0.389	2.09	0.099	1.287	0.405
		4.691		0.227
1.206	1.022	0.754	1.092	0.801
0.637	1.336	1.275	0.634	0.846
1.086	0.794	1.758		0.225
0.925	1.62	0.765	2.082	2.46
	1.516	0.57	0.978	2.207
0.608	1.452	1.511	0.955	1.933
0.9	0.934	0.785	0.675	1.553
0.731	0.501	0.547	2.664	0.574
1.05	0.931	0.601	1.05	0.943
		1.298		0.964
0.858	1.635	0.69	1.117	1.582
	1.348	4.825		1.131
0.917	0.656	1.043	1.421	1.52
1.208	0.64	0.801	0.93	0.804
				1.517
1.017	0.345	1.052	1.351	0.988
1.061	1.036	1.292	1.524	0.941
0.968	0.624	1	0.307	0.731
1.131	0.309	0.977	1.497	2.081
1.206	0.258	0.818	1.05	1.332
1.093	1.831	1.01	2.051	1.229
1.184	0.255	0.589	0.598	0.825
0.621	0.431	0.402	0.687	0.372
1.306	0.815	0.943	1.163	0.911
1.193	0.433	1.523	0.354	1.243
0.924	0.644	0.651	0.941	0.615
0.476	2.785	1.439	0.27	0.93
0.907	0.583	1.201	0.37	0.515
1.214	3.388	1.629	1.18	1.342
1.2	1.391	1.021	0.796	1.135
1.07	1.386		0.984	
1.094	0.083	0.873	1.022	1.258
0.995		0.793	0.834	0.727
0.134	0.043	0.459	3.347	0.672
0.914	1.159	0.972	3.488	0.721
0.409		0.765		0.829
1.191	0.773	0.59	0.433	2.277
0.953	0.647	0.829	0.764	0.943

0.594		0.362	0.767	0.56
0.898	0.76	1.199	0.591	1.089
1.005	1.84	1.342	7.275	0.723
1.325	0.64	0.889	1.253	1.296
2.11	0.265	0.653	1.222	0.746
		1.112		1.328
0.761	0.987	0.338	0.633	1.083
0.132	0.151		2.102	1.089
0.541		0.644	0.576	
0.778				0.319
0.979	1.203	1.269	1.415	1.16
1.088	2.151	0.806	0.531	1.767
0.679	0.896	0.497	0.904	0.644
1.038	0.999	0.932	1.378	0.912
1.006	0.998	0.811	1.124	1.14
0.938	2.282	1.088	0.793	1.136
0.687	0.763		0.55	0.569
1.058	1.497	0.842	0.476	1.224
1.239	0.655	1.388	1.125	1.444
0.81	0.606	0.75	1.133	0.629
0.65	1.245	0.731	1.879	1.2
0.319		0.628	0.466	0.892
0.596	0.277	0.883	0.998	1.013

1.472	1.535	1.42	1.681	0.109
0.967	1.007	0.973	1.418	0.931
0.632	2.159	1.607	0.45	0.51
0.626	1.341	0.772	0.448	0.879
1.05	0.737	0.977	1.501	0.842
0.888	1.294	0.851	1.203	0.914
0.708	1.389	1.858	0.971	2.46

1.481	0.576	0.409	1.451	0.517
0.892	0.287	1.256	0.634	1.208
0.853		0.517	2.831	2.213
1.224	0.865	1.094	1.053	1.196
1.304	1.365	0.777	1.266	0.817
0.997	1.407	0.963	1.673	0.737
1.506	0.907	0.637	1.119	0.947
1.422	0.222	0.641	0.968	2.162
0.671	0.28	0.77	0.403	1.286

1.716	2.557	0.538	0.315	1.098
0.229	0.332	0.89	5.334	0.772
1.098	3.223	4.226		0.666
1.26	1.428	0.866	0.907	0.961
0.243	0.143	0.476	0.449	4.583
1.257	1.37	0.993	1.697	1.167
0.932	0.846	1.597	0.806	0.543

0.938	0.198	0.932	0.394	0.98
1.603	0.978	0.876	1.195	0.937
1.149	0.59	1.074	1.422	0.876
	2.893	1.124		0.7
1.132	1.808	0.935	0.777	0.909
0.96	2.408	1.166	0.785	1.136
0.327		1.707	1.192	0.916
0.892	0.988	1.365	0.463	0.846
2.009	1.324	0.655	1.11	1.363
	1.263	0.973		
1.466	0.728	1.174	0.624	1.055
1.062	0.118	0.844	0.441	1.171
1.105	0.549	0.886	1.264	1.134
1.069	0.074	0.955	0.706	0.988
0.808	0.701	0.799	0.344	0.642
	0.056		3.402	1.231
1.258	1.924	0.932	0.842	1.053
1.883	1.542	0.794	1.721	0.708
1.24	0.961	0.917	1.421	1.77
0.476	0.388	0.713	1.377	0.585
1.722	2.729	1.821	0.444	0.839
1.5	0.55	1.002	1.181	0.558
1.609	0.15	0.955	0.167	0.977
0.686	0.679	0.421	5.335	0.311
1.183	0.515	1.899		
0.946	1.927	1.739	1.14	1.306
0.479	1.665	0.89	1.142	1.339
0.944	1.569	0.631	0.934	1.089
0.638		0.739	0.928	
1.166	1.139	1.583	1.064	1.628
1.083	2.148	1.03	0.425	1.06
0.997	0.762	0.785	0.876	0.28
0.798	0.185	1.242	0.425	1.254
1.078	0.451	1.737	0.524	0.793
0.941	0.447	1.436	0.263	1.247
1.236	0.599	0.793	1.285	1
1.162	1.48	0.973	1.367	0.761
1.177	1.986	1.007	0.688	0.929
0.543		0.991		0.692
0.581	0.624		0.474	2.169
1.367	1.166	0.818	0.588	1.138
0.941	1.825	1.384	0.963	1.257
1.348	1.441	1.226	2.164	0.949
0.913	0.291	0.755	0.29	1.384
1.319	0.321	0.829	0.756	0.766
1.161	1.067	1.291	0.52	1.126

1.107	0.426	1.153	0.429	1.197
1.142	1.354	0.934	1.299	0.43
1.017	0.861	0.925	1.781	1.144
0.65	1.526	1.232	0.961	1.545
1.113	1.189	1.161	3.241	1.894
1.093	1.12	1.468	1.742	2.402
1.768	0.766	1.045	1.72	1.052
1.021	0.495	1.125	0.534	1.103
0.768	1.318	1.521	0.875	1.738
	1.36	1.872		
1.14	1.5	0.945	1.25	1.707
0.225	2.418	2.194	3.083	2.09
0.792	1.979			0.472
1.136	0.483	1.282	1.193	0.907
0.99	1.552	1.023	0.885	1.089
		0.888		
0.906	1.286	0.908	1.158	1.194
0.778	1.951	0.812	1.002	0.785
0.415	0.287	1.96		1.629
2.049	0.211	1.517	1.526	0.964
1.145	2.388	1.101	0.512	0.846
	0.467			
0.333	0.796	0.601	0.664	4.701
1.077	1.612	1.252	0.731	1.151
				0.984
1.151	1.419	0.691	1.384	0.719
0.895	0.971	0.654	2.362	0.754
0.679	0.398	1.298	1.462	0.776
1.25	2.067	1.074	0.98	0.98
0.711	0.101	0.917	0.758	1.112
1.456	0.458	1.143	1.185	1.082
1.434	0.286	0.7	0.944	1.159
1.063	2.088	1.125	1.674	0.937
0.922		1.711		1.585
0.653	1.424	0.736	1.17	1.083
1.11	1.063	1.075	1.703	1.548
1.361	0.562	1.06	0.59	0.914
	0.944			
	0.128	0.158	0.341	6.825
1.43	1.017	0.629	0.86	0.759
0.782	0.451	1.774		0.628
0.997	1.206	0.98	1.288	0.933
1.11	1.115	1.323	2.24	1.34
		0.63	0.821	0.573
0.537		0.32		
1.125	2	1.196	1.055	0.935
1.183	1.89	0.665	0.555	0.92

1.193	1.551	1.06	0.88	1.102
2.956	0.65	1.506	2.412	1.819
0.585	0.444	1.855	1.372	1.657
1.411	1.634	1.075	0.711	0.873
0.767	0.481	1.863	0.655	0.629
0.875	1.198	1.237	2.095	1.09
0.973	0.489	1.113	2.339	0.68
1.02	0.497	0.77	0.515	0.594
0.449	0.943	2.538	0.351	0.463
0.423	2.299	0.63		2.183
			1.462	1.231
0.958	0.457	0.949	0.808	0.984
1.138	1.826	1.33	0.469	0.924
0.304		0.838	0.889	1.106
0.861	0.586	1.665	0.584	1.208
1.37	1.791	0.186	0.34	0.533
1.139	1.289	1.195	0.966	1.181
1.356	1.218	1.249	0.465	1.472
0.682	1.322	0.715	0.343	0.686
1.042	1.209	0.481	0.996	1.13
0.836	1.301	0.538	0.288	0.621
1.089	0.599	1.053		0.619
0.838	0.917	0.664	2.007	1.014
	1.93	0.582		
1.004	1.193	0.87	1.67	1.006
0.934	1.106	0.921	1.206	0.844
1.169	0.35	1.207	0.513	0.742
0.922	1.214	1.016		
1.134	0.737	0.843	0.44	0.746
0.849	1.671	0.73	0.859	0.875
1.504	0.956	1.262	2.227	0.991
1.569	0.161	0.942	0.623	1.166
1.009	0.21	0.374		
1.317	0.332	1.057	1.024	1.005
0.835	0.945	1.116	1.545	1.238
1.079	0.746	0.533	0.302	1.083
1.007	0.333	0.859	1.137	0.762
1.046	1.521	0.758	1.233	0.642
	0.473	1.33	1.024	
1.559	0.854	0.912	1.9	1.121
0.967	0.907	0.528	1.268	0.967
0.795	1.221	0.622	0.877	0.666
0.624	1.082	1.024	1.711	0.909
1.206	1	0.561	1.302	1.141
1.544	1.497	0.668	1.681	0.797
0.949	1.886	0.834	1.104	0.992
				1.291

1.39	0.769	0.846	0.94	1.083
	1.393			
			1.751	
0.937	2.494	1.108	0.809	1.059
1.56	0.179	0.286	1.548	0.082
0.695	2.988	0.829	0.66	1.756
1.009	0.251	0.584	0.973	1.258
1.236	1.535	0.898	0.339	0.33
0.627	1.101	0.648	1.014	0.952
0.698	0.878	0.823	1.436	1.352
1.07	0.752	0.816	1.346	0.988
0.808	1.622	0.91	0.857	1.006
0.699	0.789	0.909	2.547	0.769
1.184	0.989	0.881	1.388	0.932
0.966	1.093	0.864	0.675	0.462
	0.207	1.408		0.485
1.236	1.632	1.013	0.752	1.384
0.695	1.613	0.877	1.279	1.074
0.765	1.287	0.85	0.84	1.078
0.797	0.663	1.401	0.519	1.086
1.002	0.793	1.211	0.815	1.142
1.282	1.487	0.845	1.278	1.126
1.228	1.979	1.38	2.811	0.918
0.259	0.172	0.467		
1.259	0.457	1	0.988	1.118
1.23	0.76	0.079		0.064
0.893	0.991	0.731	0.795	0.561
0.754	1.555	0.948	0.956	1.141
1.071	0.399	1.087	1.13	0.88
1.261	0.877	0.824	2.534	1.566
0.524	2.696	1.91	0.481	0.959
1.002	0.69	0.961	1.403	0.958
0.83	0.561	0.44		
0.726	0.232	0.882	1.856	0.951
0.287	1.778	2.141	3.846	0.228
	0.382	0.361	5.082	
0.691	2.692	0.656		1.027
1.65	1.873	0.781	2	1.339
0.714	1.74	2.147	4.527	0.545
0.471	1.648		0.305	0.26
1.026	0.444	1.272	0.501	1.127
0.788	0.797	0.613	1.365	0.716
0.906	1.238	1.276	1.615	
0.853	2.005	0.539	1.487	1.181
1.113	2.176	1.299	0.71	0.979
1.949	0.71	0.797	0.941	1.03
0.664	0.403	1.208		2.38
2.143	1.788	1.072	0.977	0.837
	0.094	0.411	0.187	

0.898	1.012	0.775	0.979	0.882
0.789	1.548	1.298	1.378	1.036
0.753	2.221	0.71	1.563	1.858
1.071	1.191	2.116	5.952	0.354
	2.958	1.449	0.734	0.443
1.639	1.43	1.018	3.63	2.328
1.146	0.815	0.87	1.234	1.229
1.807	1.994	0.946	1.351	0.531
1.526		1.293	1.369	0.977
1.49	1.413	1.207	1.43	1.67
	1.384	1.249	0.934	0.692
1.186	0.985	1.023	1.007	0.743
1.056	0.521	0.781	0.353	0.936
0.457		0.567	0.182	0.603
1.377	1.882	0.787	0.555	0.354
1.525	1.067	0.72	0.791	1.252
1.42	1.188	1.085	1.436	0.866
1.742	2.438	0.969	0.89	1.062
0.518	1.141	1.318	1.328	0.839
		0.717		0.699
0.849	0.155			
0.767		1.048	1.265	0.946
			1.594	
0.846	0.84	0.755	0.866	1.141
0.626		2.212		0.807
1.079	0.613	0.642	0.699	1.047
0.305				
1.012	0.984	1.324	5.938	0.909
0.543	0.487	1.901	0.989	1.463
0.962	0.82	1.079	0.534	0.658
1.045	0.646	0.663	0.93	1.027
1.427	2.478	1.08		1.215
		0.819	0.841	1.165
1.061	0.638	0.852	1.469	1.771
1.297	1.237	1.248	0.4	1.065
0.868	4.569	1.561	0.802	0.889
1.214	1.512	1.073	0.991	0.424
0.042	1.32	0.091	0.329	0.078
	0.902			
1.4	0.696	0.604	0.858	1.376
0.821	1.452	1.247	1.155	0.871
0.978	1.336	1.471	2.076	0.868
1.055	1.035	1.084	0.778	1.116
0.032	0.447	0.367		0.227
0.769	0.983	0.748	0.911	0.618
1.27	1.313	1.454	0.955	1.61
1.574		0.647	1.169	1.378
0.999	1.536	0.832	0.958	1.129

0.238	1.278			
0.836	0.183	1.22	1.628	1.323
1.32	0.691	1.05	1.752	1.453
1.159	0.84	1.001	0.726	1.116
1.569	0.967	0.691	1.174	0.666
1.357	1.041	1.16	1.163	0.964
1.185	0.752	0.887	0.986	0.967
1.52	1.184	0.732	1.278	0.517
1.199	0.548	0.985	1.279	1.234
0.675		0.596	1.591	1.293
1.068	0.814	0.998	1.587	1.865
1.167	0.48	1.285	0.722	0.843
0.737	0.539	1.153	1.118	1.076
0.591	1.045	0.514	1.354	0.741
0.496	1.789	1.547	7.501	0.535
0.169		0.102	0.273	2.142
0.976	0.585	0.832	1.268	1.46
1.045		1.266	4.18	0.74
3.296	0.733	0.946	1.258	1.193
0.916	1.248	0.797	1.454	1.025
1.1	1.092	0.951	0.209	0.382
1.46	0.567	0.807	1.021	1.309
0.509	1.193	0.563	0.166	0.564
1.299	1.849	1.201	1.154	1.31
1.078	0.584	0.695	0.756	0.591
0.962	0.912	0.839	1.524	1.171
0.449	1.282	1.32	0.979	0.401
0.98	2.093	1.307	0.696	1.19
1.14	1.027	1.204	1.494	1.441
1.289	0.943	0.879	1.278	1.065
0.678	1.854	1.879	0.936	0.637
0.491	1.236	0.461	0.97	0.478
1.21	0.607	0.791	0.99	0.836
1.521	0.444	1.157	1.246	0.625
0.972	0.816	0.919	1.875	0.527
0.635	2.7	0.938	0.589	0.496
0.892	0.72	0.531	1.14	0.91
2.767	1.414		0.892	
	0.4	0.586	5.033	0.337
1.765	0.9	0.815	3.197	1.579
		1.926		0.711
1.208	2.222	0.843	1.022	0.735
0.762	3.603	0.657	1.189	1.076
1.012	0.144	1.127	0.271	1.104
1.167	1.257	0.579	1.176	1.628

1.133		0.598	0.223	1.183
0.578	1.898			0.096
2.354	1.18	1.463	1.044	1.279
0.395	0.202	0.781	0.186	0.63
1.23	1.538	0.325	0.381	0.743
0.836	0.484			
0.576	1.362	1.497	0.78	1.23
1.226	1.354	0.926	1.29	0.983
1.568	0.388	0.924	1.608	1.787
1.05	1.145	1.004	1.13	0.281
0.372	1.097	1.917	0.484	2.675
0.913	1.303	1.268	1.378	1.099
0.969	1.942	1.071	2.089	0.899
1.433	0.877	0.834	1.007	0.785
1.45	2.037	1.514		
0.853	0.879	0.912	1.093	1.107
0.415	1.794	1.424	0.579	1.463
	1.581	0.955		0.878
0.877	0.459	0.34	0.511	0.388
0.858	2.418	1.594	1.172	1.453
1.299	0.299	1.344	1.487	1.135
0.351	0.809	0.914	1.669	0.576
2.182	2.469	3.213	0.513	0.663
0.956	1.07	1.187	2.296	1.662
0.844	0.368	1.062	1.125	0.622
0.491	0.884	2.29	0.538	0.332
1.185	0.843	0.914	1.617	0.879
0.879	1.033	1.165	0.875	1.057
1.335	0.94	1.127	0.56	1.231
0.788	1.515	1.369	1.369	1.753
0.156	0.671	0.565	0.397	0.414
0.243	0.356		1.307	2.677
1.061	1.116	1.09	1.031	0.878
1.171	0.966	0.868	1.062	0.731
		1.408	2.541	1.999
0.926	1.548	1.053	0.804	1.061
1.434		1.113	0.262	1.056
1.384	2.363	1.156	1.428	0.789
0.581	1.207	1.691	4.088	0.676
0.89	0.149	1.309	2.294	1.349
0.907	1.057	1.002	1.253	1.164
0.847	0.585	2.358	1.863	1.353
1.017	0.618	1.323	0.542	1.259
0.315	1.452	0.78	0.334	1.06
	1.478	0.795	0.51	

1.216	0.562	0.894	1.331	1.057
1.007	1.02	1.168	1.219	1.182
1.118	1.017	1.163	1.567	1.083
0.666	1.628	0.381	0.635	1.023
1.346	1.381	0.968	0.645	0.734
1.149	1.151	1.048	1.715	1.262
0.473		1.677	0.712	1.158
0.574	0.372	0.869	0.875	1.005
0.504	0.992		0.966	0.5
0.249	2.147		0.721	
1.275	0.79	1.71	0.718	1.185
1.069	0.264	1.015	1.198	0.838
0.982	1.516	1.03	0.645	0.893
1.219	0.562	0.95	0.721	1.293
0.387	1.454	1.178	0.888	1.245
				1.389
		0.691		1.037
	0.82			
1.185	1.261	0.957	1.232	0.898
1.241	1.143	1.931	2.165	0.818
0.535	0.382			0.534
1.635	0.164	0.239	0.368	0.349
1.106	1.036	0.981	1.459	0.815
1.065	1.013	1.044	2.238	1.227
1.134	1.327	0.631	1.136	1.017
1.347	1.216	0.706	0.771	1.409
1.065	0.338	0.731	0.772	0.96
0.814	2.029	0.955	1.038	0.858
1.063	1.654	0.97	2.081	1.472
1.603	3.499	0.032	0.124	1.414
	1.119			
1.269	1.058	0.88	0.815	1.066
0.784	1.863	1.617	0.597	0.747
0.153	0.247	19.287	0.204	0.072
	0.235		0.915	
	1.08	0.919		2.326
1.141	0.522	0.784	1.54	0.98
0.982	1.758	1.122	0.544	0.892
1.364	2.259	0.803	0.719	0.892
0.986		1.551		1.006
0.986	1.319	1.267	1.322	0.933
0.419		0.872		
1.013	0.559	0.796	0.939	2.248
1.078	0.411	1.398	1.106	0.97
0.97	2.164	1.102	1.79	0.99
0.724	1.941	1.944	1.435	1.281
0.389	0.344	0.406	5.046	5.334
0.49	0.467	0.625	2.757	1.333

1.229		1.674	3.552	1.394
0.902	1.177	0.853	1.574	1.123
	1.183		1.191	1.634
0.358	0.54	1.479	0.843	0.603
0.129		1.27	2.468	1.21
0.997	1.955	1.071	0.992	1.237
0.677	1.585	0.531	1.334	1.007
1.331	1.324	3.451	0.834	0.675
0.213	0.114	1.208	1.137	0.495
0.801	1.485	1.307	0.915	1.057
1.327	1.295	0.129	0.173	1.128
1.435	0.734	0.989	2.053	1.619
1.194	0.893	1.025	1.24	1.497
1.114		0.493		
	0.676			
0.291		1.138	0.403	2.542
0.21	0.676	16.045	0.205	0.206
1.654	1.412	0.775	1.442	0.943
1.457	1.218	0.917	1.191	1.477
1.31	0.959	0.381	1.199	0.658
1.223	0.551	1.201	1.151	0.469
0.492	0.767	0.983		
			0.259	0.116
0.714	0.822	0.966	1.379	1.267
1.156	1.772	0.814	1.009	1.154
	0.13		1.002	
		0.825		
1.517	2.324	1.284	1.784	0.447
1.186	0.611	0.724	1.096	0.877
0.417	0.777		1.778	
1.13	0.555	1.053	0.619	1.282
1.106	1.012	1.351	1.832	1.317
1.106	1.782	0.845	1.282	0.896
			1.813	4.454
	0.842	0.915	1.165	
1.27	0.452	1.232	1.175	1.281
0.803	0.412	0.868	0.512	0.322
1.206				1.635
0.624	1.332	0.575	1.46	0.75
1.082	0.41	1.569		1.282
1.017	0.669	1.432	1.118	0.928
1.032	1.466	0.892	1.649	2.774
0.867	1.383	0.711	2.195	1.225
1.521	0.113	0.512	0.613	1.005
1.344	0.55	1	0.555	0.955

0.804	1.634	1.683	0.585	1.339
0.685	1.014	0.169	1.383	0.983
0.786	3.541	0.757	0.629	0.454
0.964	2.112	1.14	0.708	1.339
0.28	0.273	0.172	0.586	
1.444	1.649	1.646	1.273	0.778
0.999	0.436	1.059	0.496	0.998
				0.749
0.429	0.46	0.779	14.534	
1.28	0.655	0.758	0.898	0.701
		1.727		2.257
0.843	1.938	1.036	0.09	0.488
0.705	0.929	0.14	0.095	0.126
0.968	0.363	0.759	0.949	0.813
1.246	0.084	0.617	1.075	1.007
0.782		0.79		0.497
0.783		0.295	0.222	1.689
1.259	1.777	0.459	0.881	0.516
0.635	1.308	1.099	3.462	1.049
	1.006	1.153	5.436	0.986
	1.2			
1.13	0.587	0.509	0.592	2.569
	1.385			
1.119	0.793	1.476	0.794	2.117
0.788	1.726	0.498		0.334
1.246	0.602	1.483	0.992	0.571
0.858	1.094	1.11	1.159	0.795
1.105	1.268	1.128	2.549	0.653
1.47	1.057	0.79	0.608	0.787
	1.42	1.234		0.843
1.457	0.644	0.94	1.113	0.959
0.252	0.355	0.239	0.316	0.144
0.932	1.077	0.694	1.597	2.05
0.716	0.808	1	2.112	1.135
1.125	0.9	1.107	3.523	0.647
1.099	0.129	1.08	1.297	0.653
0.467	0.291	0.205	0.277	0.368
0.708	0.395	0.769	0.454	1.725
1.698	0.131	1.471	1.354	1.098
1.39	0.038	0.993	0.703	0.815
1.035	0.969	0.686	0.839	0.801
1.009	0.679	1.293	0.993	1.11
1.251		0.564		
0.783	0.581	1.551	0.515	0.653
		1.42		
0.753	0.63	1.17	1.078	0.514

0.524	0.961	1.709	0.745	0.557
1.213	0.638	1.587	0.445	0.9
0.93	1.402	0.886	1.179	1.067
				0.673
1.002	0.981	0.885	0.868	0.802
0.241		0.404	0.565	0.412
0.569	0.658	0.935	3.829	1.801
1.034	1.407	1.052	5.988	0.778
		0.647		0.598
0.266	0.675	0.551	0.412	0.372
0.852	0.926	0.938	1.324	1.566
0.537	1.478	0.687	1.188	2.31
1.663	0.9	1.113	1.583	0.854
0.589	0.412	1.131	0.281	0.593
0.853	2.394	1.272	0.579	1.354
0.811		0.07		1.312
1.294	0.906	1.057	1.307	1.14
0.658	0.365	1.091	0.365	0.83
0.95	0.33	0.648	0.412	0.291
1.204	0.907	1.418	1.659	1.115
	0.757			
0.963	0.45	1.72	0.508	1.136
1.287	3.028	0.973	0.34	0.854
1.427	0.294	0.924	1.279	1.358
0.732	1.861	1.154	1.659	1.518
1.035	1.329	0.845	1.348	0.99
0.591	1.459	1.001	1.509	0.968
0.534	1.393	0.809	1.911	1.023
		0.579		
0.878	0.984	0.952	0.931	1.024
0.743	1.3	0.738	0.507	1.107
1.413	1.972	0.518	1.382	0.886
	0.296	1.416	0.587	0.667
0.715	2.473	1.474	0.333	1.059
0.758	0.171	1.608	0.431	0.813
	0.072	0.48		1.293
	0.626		4.511	
0.618		1.05		1.483
1.037	1.176	1.078	2.013	1.264
1.141	0.472	0.897	1.063	0.869
0.91	1.356	1.005	0.735	0.93
1.201	1.841	1.451	0.445	0.705
0.898	1.42	0.566	1.369	1.112
1.684	1.139	0.99	2.733	0.985
1.353	1.01	1.299	1.485	1.541
0.391	1.029	0.757	1.944	1.153
2.096	1.307	0.483	1.622	0.881
0.82	1.051	0.928	3.326	0.699

	1.325			
0.958	1.354	1.048	2.153	2.325
0.6	2.072	0.914	1.632	0.773
1.418	0.15	1.99	2.528	1.319
0.887	1.512	1.508	0.987	1.058
0.708	1.237	0.791	1.392	1.091
1.493	0.684	0.554	0.606	0.644
0.672	0.594	1.042	1.24	1.422
1.788	0.913	0.948	0.782	0.909
		1.026		1.038
1.16	0.932	1.264	0.665	0.964
		0.951		
1.111	1.768	1.092	0.846	1.076
	1.348			0.703
1.037	0.538	1.533	0.53	1.711
			2.021	
1.134		1.064	0.434	1.078
0.923	1.103	1.126	1.075	0.782
0.861	1.786	1.324	0.679	1.065
0.579	0.398	0.996	3.983	2.289
0.565				
			0.787	1.201
0.928	0.955	1.071	2.872	0.964
0.682		1.488	0.319	0.858
1.344	1.523	1.529	2.004	0.934
0.579		1.122	0.141	1.014
	1.004	0.681	2.404	1.559
1.073	1.18	0.97	2.69	0.832
0.988		0.861		0.837
1.158	2.065	1.583	0.415	1.589
0.999	0.602	0.986	0.701	1.099
6.705	0.479	0.772	0.587	0.36
0.79	1.014	0.942	0.669	0.597
0.305	0.016	1.508	0.119	1.158
0.891	0.291	1.577	0.977	2.332
1.293	0.179	0.822	0.456	0.351
0.746	0.54	1.244	1.259	
1.12	0.485	0.807	1.436	0.995
0.827	0.717	1.255	0.985	0.25
0.859	0.764	0.545	0.59	0.55
	2.637	0.275		
0.992	1.281	1.065	1.035	0.914
0.891		2.279		
1.859	0.155	0.441	0.154	2.169
0.826	1.326	1.739	3.01	0.814

1.105	1.991	1.019		0.739
0.908		1.01	0.676	0.881
0.938	1.621	1.43	0.98	1.186
1.234	0.879	1.042	1.271	1.047
1.15	1.277	0.797	0.787	0.816
0.551		2.118		1.401
1.03	0.266	0.657	1.256	0.929
0.953	2.196	0.879	1.066	1.038
1.163	1.564	1.436	1.082	0.992
0.855	1.823	1.923	1.379	0.321
0.766	1.83			
1.408	0.28	0.598	0.939	0.988
0.715	0.722	1.101	2.004	1.39
1.162	1.372	0.775	1.516	0.904
1.607	1.181	0.881	1.306	0.303
0.985	2.239	0.722	1.028	0.666
3.262	2.569	1.352	0.807	0.876
1.17		0.679	0.418	0.405
0.72	1.043	0.822	0.72	0.605
1.141	0.617	1.153	1.089	1.092
0.917	0.038	1.198	0.246	1.008
0.884	0.657	1.23	0.831	1.648
0.458	0.854	1.336	1.37	1.657
0.93	1.453	1.352	0.548	1.313
1.365	0.217	0.957	1.459	1.315
1.357	0.371	1.563	0.282	0.937
0.508	0.459	0.978	2.061	1.849
0.906		1.221	0.355	0.592
1.044	0.012	1.581		1.035
1.069	0.508	1.245	0.57	1.047
	0.657	1.275	2.888	0.801
0.834	1.728	1.598	4.018	0.842
0.863	1.251	0.757	1.427	0.966
0.746	1.118	0.969	2.277	1.726
			0.771	
0.999	0.25	1.444	0.356	0.961
0.738	1.935	1.363	0.513	0.964
1.648	0.063	1.422	1.306	0.104
	1.301			
0.5				
0.504	4.35	0.782	0.167	1.34
0.99	0.474	1.345	0.439	1.372
1.148	1.265	1.24	1.904	1.528
1.216	1.13	1.682	1.908	1.36
0.522	1.463			
0.817	2.167	1.867	0.814	1.067
1.329	0.501	1.821	0.453	0.818

0.176		3.435		2.248
1.111	1.648	1.32	1.153	0.803
1.173	0.774	1.299	1.321	0.903
0.91	0.211	1.278	0.047	1.399
1.033	1.481	0.44	1.592	0.828
1.595	1.509	0.855	1.375	1.473
	1.029	1.009	0.955	
		1.624		1.758
1.162	0.77	1.075	0.61	1.089
0.774	1.701	1.044	0.792	0.663
0.886		1.356	0.738	1.043
0.721	1.048	0.872	0.922	1.762
1.233	1.026	0.851	0.989	0.848
1.206	1.491	1.344		1.334
	1.141		1.53	1.166
0.888	0.077	1.207	0.103	1.181
1.237	0.665	0.893	1.351	1.004
1.115	0.123	0.643	0.458	0.85
0.803	0.476	0.594	0.542	0.862
1.587		0.032	0.007	0.031
1.617	1.953	0.786	1.733	1.414
1.311	0.888	1.043	1.211	1.082
1.016				1.304
0.92	1.627	0.868	0.655	0.863
0.893	0.768	1.137	0.716	1.026
1.135	2.154	0.794	0.573	0.642
0.78		1.235	1.006	1.123
1.091	0.725	0.943	1.52	1.273
1.118	1.211	0.98	1.465	1.106
3.288	4.126	1.778	1.146	0.794
1.239		1.673	0.026	1.225
1.099	0.918	1.375	0.741	1.409
1.011	1.396	0.806	0.62	0.685
0.913	0.228	0.915	2.497	1.841
1.169	1.235	1.002	1.051	1.067
0.419	1.686	0.929	0.602	0.963
0.73	1.032	1.266	1.077	0.56
0.631	2.696	1.294	0.749	1.204
0.631	0.287	1.453	1.495	
1.204	0.869	1.505	0.835	1.132
0.845	3.251	1.075	0.696	0.937
0.503	1.174	1.939	2.081	1.29
1.032	0.602	0.779	1.055	
1.496	1.23	0.878	1.033	1.161
1.132	2.056	0.846	0.807	1.042

1.626	0.795	1.353	1.066	0.629
1.123	0.736	0.438	0.856	0.73
1.253	0.87	0.94	4.105	0.165
0.72	0.665	0.978	0.73	0.769
2.129	3.461	2.635	0.034	0.092
1.238	0.297	1.236	0.351	1.135
1.155	1.447	1.165	0.818	1.39
1.266	0.953	0.974	1.637	1.377
2.225	0.131	1.28	1.393	1.17
1.11	0.362	1.648	0.25	0.682
0.894	0.277	1.485	1.946	1.675
1.866	0.596	0.735	0.614	1.034
1.673	2.072	1.48	0.884	1.349
1.491	1.145	0.78		
0.716		0.836	0.164	1.153
0.971	1.303	0.342	1.728	0.969
1.128	1.512	1.222	0.672	0.908
0.711	1.257	1.189	0.51	1.838
1.329	1.335	1.131	0.668	0.903
2.245	2.545	1.879	0.871	1.084
1.167	0.701	1.195	1.294	0.675
0.896	0.737	0.637	1.334	1.321
0.794	1.173	1.391		1.108
0.576	2.746	0.633	1.259	0.951
1.223	1.007	1.169	1.66	0.635
1.185	1.264	0.989	1.248	1.14
1.065	0.902	1.159	1.502	1.371
1.571	0.248	0.378	0.414	0.678
0.974	0.952	1.445	0.583	0.972
0.993	1.189	1.328	1.842	1.053
1.209	1.335			
1.012	1.948	0.876	1.599	0.453
0.611	0.304	1.337	0.375	1.522
1.331	0.381	0.708	0.462	1.18
1.257	0.459	1.028	1.219	0.956
0.93	0.877	1.067	0.759	1.018
1.144	1.286	1.357	0.793	0.861
1.252	1.058	1.399	0.526	1.018
1.797	0.734	0.315	1.151	0.712
0.882	1.355	0.851	1.647	1.001
1.075	1.894	1.183	1.203	1.33
		1.5	0.594	
0.721	1.008	0.722	1.459	0.938
	0.537	0.681		1.022
0.644	0.746	0.918	1.847	2.205
1.302	1.834	0.805	1.421	1.223
1.122	0.936	0.615	1.522	0.907

1.263	0.359	1.191	0.408	0.292
2.121	0.966	1.168	0.846	1.029
1.06	1.344	0.883	0.862	1.009
0.932	0.705	0.474	2.444	1.248
1.191	0.823	1.649	0.449	1.253
1.155	0.586	0.867	1.089	1.159
1.454	0.442	0.671	1.046	0.706
1.143		1.6	0.497	0.955
0.794	0.454	0.827	0.559	0.324
1.221	0.423	1.536	0.278	1.345
2.323	0.02	2.053	0.027	2.127
1.053	1.745	1.255		0.395
0.876	1.208	0.765	0.787	0.715
0.546	0.286	0.456		
1.151	0.68	0.604	1.356	0.905
1.657	1.137	1.107	1.598	1.337
0.84	2.026	0.593		0.571
0.795	0.703	0.741	1.065	0.748
1.099	1.518	1.017	2.458	1.714
1.714	0.873	0.957	1.454	1.005
0.948		0.784		
1.025	0.715	0.978	1.544	0.871
1.113	1.402	1.297	1.268	1.115
0.837	0.671	0.648	0.669	0.912
0.392	0.287	0.735	1.85	0.8
1.496	1.16	0.787	0.82	1.226
1.132	0.465	1.114	1.473	1.475
1.277	0.722	0.771	1.024	0.904
0.806	0.691	0.743	0.826	0.921
1.11	1.605	1.666	0.2	1.542
0.975	0.626	1.048	1.226	0.753
1.523	1.084	1.968		1.191
0.636		1.068	1.31	0.901
1.213	0.971	1.054	1.224	1.068
1.918	2.502	0.561	0.758	1.038
2.049	0.405	1.577		
1.479	0.897	0.66	0.623	0.572
0.748	1.339	1.401	0.74	0.87
0.814	0.129	1.071	0.183	0.454
0.303	0.107	0.61	1.129	0.793
1.312	0.58	0.564	2.521	1.55
1.661	1.592	0.184	1.669	1.115
	1.358	1.539		1.262
0.933	1.269	0.776	1.34	0.898
1.193	1.025	0.224		0.967
1.174	1.173	0.2	0.946	1.12

0.976		1.251		0.823
		0.28		0.282
1.029	0.843	0.865	1.458	1.12
1.27				0.36
0.539		1.584	0.503	1.783
1.452	1.156	0.664	1.024	1.082
1.943	0.119	1.089	2.124	1.374
1.276	1.414	0.727	1.34	0.986
1.564	1.028	0.63	0.472	0.435
	0.483			
0.912	1.759	1.072	0.387	1.174
0.851	1.496	1.317	1.732	1.476
1.163	0.354	1.233	0.218	0.825
0.86	0.754	1.987	0.433	1.966
0.806	0.613	1.114		0.673
2.094	1.002	0.93	0.729	0.349

N_HLP_7	N_YGF_8	N_FQ_9	N_MQX_10	M_ZJQ_1
	1.119	1.467		
1.033	0.933	0.801	0.295	0.703
1.036	0.942	0.852	1.429	2.096
0.508	1.629	2.233	1.125	0.958
0.786	1.134	0.971		0.904
1.229	0.629		0.93	1.84
1.085	1.461	0.975	1.042	1.275
1.099	0.991	1.216	0.655	0.595
1.453	0.93	0.791	1.187	1.332
1.306	0.993	1.546	0.981	0.806
0.855	4.858	3.609	0.733	
0.738	1.004	1.088	0.877	1.275
1.181	2.401	2.449	1.001	0.712
0.509	1.411	1.358	1.939	1.416
1.876		2.749	0.229	
0.532				
1.238	0.369	0.753	0.819	1.034
0.741	1.04	1.254	1.106	1.131
0.759	1.189	0.99	0.288	1.478
0.766	2.604	0.814	1.041	0.816
1.47	0.806	1.768	1.082	0.963
1.139	1.283	0.963	1.751	0.867
0.501	1.828		0.77	0.384
0.953	1.554	1.366	1.301	1.414
	1.127	0.751		
1.064	0.961	1.067	1.112	1.194
1	1.019	0.967	1.929	1.726
1.074	0.92	1.308	0.76	1.17
		1.376	0.935	
1.213	0.48		1.036	1.15
1.026				
		0.898	0.396	0.336
		0.554	0.738	
0.764	0.808	0.284	1.006	1.54
0.68	0.862	2.926	1.23	1.719
0.827		0.244	2.171	1.155
1.116	0.536	1.033	1.736	1.945
0.036	0.014	0.011		
0.276	1.285	1.048	1.33	0.632
1.263	0.52	0.427	1.007	0.789
1.008	0.896	1.516	0.936	1.122
0.831	0.647	0.709	1.148	
0.969	1.017	1.109		0.984
	1.285			

				1.878
0.755	2.222	2.016	1.321	0.588
1.021	0.536	0.798		0.855
0.895	1.186			0.816
0.557	0.653		1.141	1.391
	2.072	1.803	0.325	1.208
1.228	0.917	0.887	0.968	1.678
1.099	0.992	1.073	1.388	2.1
0.92	0.95	1.375	1.562	1.43
1.514	1.334	1.757	0.643	0.722
1.514	0.788	1.6	2.077	1.601
1.5	0.868	0.74	0.691	0.999
1.017	1.093	1.85	0	1.338
0.702	0.797	0.882	1.534	0.961
0.917	1.395	0.89	0.411	0.872
1.594	0.617	0.704	2.433	0.748
0.962	0.831	1.003	0.561	0.87
1.701	2.083	1.902	1.898	1.142
1	1.233	0.681	1.016	1.141
0.005		6.55		1.256
0.355	1.757		2.08	2.543
1.155	0.791	0.614		0.314
1.328	1.215	0.25	0.425	1.343
0.539	0.391	0.718	3.546	2.056
0.618	1.424	1.304	0.191	
0.658			2.032	
0.677	0.779	0.955	1.86	1.297
0.181	0.888	0.727	0.96	1.562
1.213	1.404	0.648	3.222	1.341
0.551	0.234	0.249	0.503	0.739
	0.394		1.043	0.828
0.759	1.561	2.972	1.564	1.037
0.259	1.495	1.806	1.317	0.082
0.124	0.143	0.106	0.101	0.101
1.17	0.792	1.159	0.271	0.963
0.419	0.638	0.74	1.081	0.615
2.037	0.521		1.829	1.219
		0.696	1.218	2.039
	0.959		1.181	1.66
1.372	0.507	0.4	0.156	0.378
1.448	1.052	1.118	0.935	1.04
0.477	1.763	0.367		

0.863				1.686
1.163	0.973		0.585	1.21
0.464			0.861	1.09
1.184	0.979	1.082	1.17	1.356
	1.559	2.361		
0.865	1.132	1.099	1.188	0.794
0.701	0.693	1.081	1.23	0.702
0.815	0.732	0.606	1.137	1.193
1.316	0.773	0.518	0.953	0.815
1.158	0.694	0.698		0.643
	1.138		0.974	
0.97	0.566	0.839	1.411	1.669
0.99	0.94	1.249	1.469	1.331
1.048				1.019
1.029	1.773	1.175	1.021	0.786
0.846			0.573	1.585
0.83	0.718	0.685	1.026	1.306
1.151			0.717	1.091
1.086	1.253	1.44	1.107	1.108
1.055		0.947		
	1.591	4.523	1.397	0.646
1.409	1.04	0.956	1.39	1.025
1.665	0.451	0.699	1.195	1.053
1.133	2.692		1.419	0.601
1.496	0.813	1.897	0.829	1.145
1.346	1.711	0.727	0.933	0.948
1.366	1.327	1.402	1.098	0.819
0.761	2.314	0.707	0.782	0.541
2.248	1.023	0.634	0.857	
	1.551			
0.206	5.509	1.554	0.555	
0.817	0.911	0.708	1.207	1.04
0.61	1.139	0.761	0.77	0.818
1.883	1.827	1.646	2.131	1.329
0.77	1.685	0.534	2.823	2.168
1.121	0.48	1.033	0.235	1.141
3.267	0.772	2.047	1.05	0.757
0.501				1.047
0.133	1.542	0.836	1.437	1.243
0.826	1.524	1.712	0.548	0.691
1.414				0.68
0.997	0.917	1.394	0.947	0.942
0.987	1.146	1.127	0.899	0.733
1.476	0.951	0.594	1.014	1.672
0.368	1.274	0.995	1.702	0.849
1.366	1.475	0.084	0.526	1.282
0.906	1.043	1.389	0.798	0.653

	0.858	0.89		
1.38	1.72	1.691	1.515	0.873
0.941	1.563	1.992	1.702	1.337
0.811	0.092	1.267	1.567	1.73
1.54		0.688	0.937	
0.81	0.827	1.474	1.379	1.015
1.131	0.755	0.828	0.561	1.242
				0.815
0.839	0.837	0.692	1.298	1.248
0.597	1.261			
1.075	1.042	1.46	2.146	1.937
	0.553	1.486	1.49	0.776
0.837	1.154	1.172	1.217	1.152
1.048	0.975	1.31	0.773	0.846
1.716		0.595	0.62	0.713
0.276	0.689		0.771	0.085
1.245	1.041	0.778	0.706	1.58
1.42	0.661	1.05	0.503	0.776
	1.134		1.605	1.792
0.956	1.04	1.137	1.266	1.484
1.268	0.721	1.572	1.031	1.381
1.365	1.12	1.794	1.051	0.838
1.443			0.654	1.122
0.93	1.136	1.031	1.012	0.992
1.157	1.012	0.72	0.581	1.135
	0.964	2.435		0.562
1.408	1	1.65	1.217	1.019
0.644	0.71	0.988	0.492	0.391
1.311	1.759	2.703	0.267	0.704
1.002	0.803	0.915	1.066	1.994
1.223	1.05	0.509	0.541	0.898
0.565	1.18	1.212	2.008	1.549
1.024		2.367		0.393
0.578	0.753	0.81	1.571	1.47
1.415	0.851	0.952	0.558	0.765
0.966			0.79	0.603
0.855	0.829	0.779	1.305	1.321
0.503	0.401	0.753	0.763	2.246
0.85	1.491	0.519	1.465	1.177
			1.357	0.873
1.6	0.769	1.072	1.812	1.086
1.05	1.172	1.456	0.775	0.919
1.067	0.682	0.647	1.597	1.485
0.758	0.958	0.725	1.107	1.225
1.204	1.127	0.461	0.545	0.945
0.891	0.913	1.088	1.084	1.501
0.59	0.959	0.898	0.79	0.728
0.91	0.846	0.965	0.592	0.861

0.798	0.045	0.041	0.059	0.549
1.516	0.956		0.231	0.616
1.023	1.059	1.004	1.34	1.67
1.088	1.236	0.013		0.832
0.87	1.847			
1.022	1.146	1.721	0.453	0.871
1.158	0.901	0.464	0.64	0.48
1.33	1.581	0.617	0.31	2.959
0.459	1.21	1.176		0.413
1.083	0.76	1.265	0.645	0.645
1.499	1.087	0.779	1.746	1.479
	0.878	1.118	1.095	
0.468	1.901	0.904	0.415	0.277
			1.392	
1.301	1.24	0.543	1.513	0.173
1.164	0.916	0.554	0.616	0.943
0.232	0.252	0.806	1.447	1.207
0.174	0.097	0.518	1.288	0.24
0.535	1.337	1.008		0.695
1.388	0.657	0.223	0.963	0.999
3.756	0.621	0.408	1.006	0.665
1.134	2.105	0.553	1.696	1.933
0.979	0.964	0.255	0.896	1.172
0.826	2.222	1.138	0.91	0.549
0.55	1.379	1.739	1.33	0.895
0.645	0.922	0.834	0.503	0.879
0.847	2		0.747	1.4
1.055	0.977	1.197	1.081	1.218
1.854	0.758	3.065	0.235	0.726
0.949	1.895	0.351	0.825	1.766
	0.364	11.001	0.138	2.005
1.186	0.927	1.083	0.592	0.956
				0.478
	1.104			0.733
0.856	0.501	0.632	0.779	0.969
0.529	0.945	3.668	1.363	1.589
1.083	1.372	2.008	1.807	0.734
1.243	1.175	1.091		
1.031	0.502		0.663	2.747
1.062	0.981	1.488	1.457	0.808
0.894	1.14	0.355	0.23	1.359
1.344	1.252	0.595	1.425	0.83
1.092	0.934	1.355	0.983	1.173
0.936	0.559	0.661	0.714	1.266
0.929	0.738		0.12	0.124
0.654	3.769		0.923	1.973
1.057	0.993	1.294	0.696	1.058
0.397	0.449			0.495

0.958	0.905	1.197	1.169	1.092
0.897		0.886	0.803	1.437
0.852	1.218	0.982	1.484	1.58
1.052	2.185	1.432	1.529	1.174
0.439	1.046	0.469		0.916
1.582	0.476	1.147	1.598	1.16
1.227	1.443	0.881	2.009	1.282
0.768	1.197	0.731	1.865	1.517
2.503	2.41		0.445	1.125
1.128	0.684	1.695	0.981	1.069
1.366	4.197	6.026	0.52	0.395
1.322	0.112	1.372	1.747	1.227
0.748	0.618	0.858		0.416
0.791	1.208	1.632	0.795	0.71
2.692	0.899	0.613	4.573	0.084
1.002	0.782	1.051	0.447	0.837
	1.1		2.688	
0.633	0.445	0.171	1.891	2.263
1.74	0.79	1.526	1.842	1.203
1.203	0.822	0.767	0.576	0.627
0.307	0.633	0.423	0.189	0.305
0.984	1.25	0.937		1.799
0.94	0.46	0.505	0.997	0.982
1.087	0.585		1.079	0.879
1.147	0.997	0.569		1.54
	0.295			
0.746	1.162	1.531	1.034	1.049
	1.708		1.284	
0.943	1.849	0.836	0.922	1.21
0.86	1.143	1.2	1.371	0.884
1.068	0.682	0.926	0.778	0.738
0.564	0.558	0.907	1.213	0.619
0.029	0.045			
	0.165	20.72	0.229	0.168
	1.793	0.962	1.254	0.961
	1.094	1.033		
0.842	3.673		1.545	1.003
1.035	0.965	1.545	1.345	1.271
1.082				0.95
1.236	0.914	0.891	1.464	1.62
0.556	3.729	1.712	1.605	1.001
1.124	0.51	1.003	0.474	
0.845	0.654	0.757	0.89	1.387
0.393			1.049	0.281
1.199	1.003	0.846	0.653	0.744
0.648	0.837	1.045	0.806	1.16
0.818	1.211	1.189	0.915	0.75
1.075	0.747	1.014	0.006	

1.102	0.922		0.843	1.174
1.085	1.216	1.781	1.373	1.231
0.893	0.562	0.843	0.948	0.691
1.034	1.001	0.939	0.959	1.167
1.142	1.093	1.025	0.729	1.221
1.162	0.915	0.414	1.037	1.077
0.198	0.312	0.213		0.701
		0.946	0.622	0.663
1.516	0.655	1.118	1.194	1.301
0.71	0.95	1.599	0.74	
	2.19	0.773		
1.403	0.714	0.94	1.057	1.465
1.133	0.878	0.795	0.363	0.938
0.673	1.837	0.597	0.673	1.148
	1.531	1.142	0.144	0.391
0.841	1.048	1.433		0.602
0.465	1.551	1.042	1.035	0.859
0.573	1.098	0.682	2.142	0.947
0.996	1.123	0.777	0.532	1.346
1.272	1.817	0.446	0.483	1.179
0.927	0.366	1.301	0.689	0.876
			1.295	
1.605	1.187	1.368	0.776	1.19
1.203	0.9	1.052	0.801	0.993
0.868	0.984	0.386	0.761	0.841
1.169	1.531	0.611	1.024	0.92
0.522	0.802	2.182	0.477	1.436
1.288	0.904	0.878	0.555	0.876
0.548			1.263	
1.098	1.203	1.217	0.674	0.873
1.553	1.514	1.395	0.888	0.471
1.295	0.723	0.573	0.922	1.435
0.673	1.199	0.728	0.542	0.788
1.381	0.145	0.468	2.487	0.857
0.967	0.979	1.065	0.827	1.04
1.341	1.313	1.121	1.388	1.109
0.877	2.096	1.145	1.205	1.052
	0.648		0.719	0.923
0.317	5.634	2.596		1.061
0.928	0.818	0.792	0.846	0.891
1.151	0.668	1.107	0.126	1.588
1.364	1.197	1.591	1.443	0.887
1.034	0.914	0.725	2.579	0.909
0.871	3.445	2.67	0.686	0.685
1.581	0.665	1.471	1.424	1.305
	0.908	1.48		
0.695	4.407	2.298	0.925	1.034

2.352	3.908	1.11	1.055	0.689
	1.571			
0.522	1.963	0.34	0.447	0.525
1.165	0.879	1.059	0.639	1.058
1.183	1.742		0.643	0.747
0.839	0.951	1.015	1.587	1.607
0.218	0.571	0.907		
1.63	1.188			1.069
0.635	1.77	0.802	1.716	0.739
0.27		0.137	3.582	0.437
1.221	1.117	0.076	0.93	0.715
1.175	0.879	1.115	1.018	0.674
0.571	0.773	0.717	0.725	0.848
1.342	1.28		1.149	0.988
1.446	0.892	1.101	1.186	1.329
1.366	1.159	1.735	1.052	0.684
1.115	0.932	0.584	0.869	1.072
0.663	0.954	1.242	1.673	1.169
0.593	7.198	0.27	1.231	0.672
0.924	0.603	1.326	1.754	1.685
0.537	0.76	0.791	0.567	0.984
0.323	0.53	0.114	2.454	1.072
1.179	1.253		1.165	
1.283	0.478	0.63	1.037	1.193
0.931	1.646	1.88	0.457	0.795
1.164	0.976	0.363	0.202	
1.009	5.791	0.713	0.173	0.339
	0.3	0.401	0.673	0.621
0.965	1.082	1.248	1.353	0.668
0.499	0.636	0.587	0.703	1.011
		1.851		
0.617			0.328	0.357
0.591	1.244			0.366
0.479	2.99	2.951	0.846	0.44
1.235		0.363	0.653	1.558
0.752	0.702	0.879	1.6	1.88
1.052	0.668	1.045	0.741	1.467
0.98	1.521	2.001	1.078	0.956
0.817	1.402	0.86	1.525	0.67
1.153		0.471		0.738
			1.626	1.551
0.87	1.27	1.069	1.92	1.783
1.019				0.835
0.199			0.396	0.262
0.202	0.342	0.284	0.359	0.314
1.196	0.895	0.967	1.477	0.726
1.061	1.048	1.416	1.108	0.925
0.546	1.077	1.351	1.404	0.976
0.931	0.468	0.598	6.455	0.878
0.999	1.3	1.883	1.299	1.054

1.094	0.922	1.272	1.447	1.234
1.345	0.82	0.062	0.906	1.069
		1.389	2.222	1.921
2.103	0.386	1.134	0.723	1.106
1.229	0.754	0.171	1.145	1.163
	1.781		3.459	
1.133	1.321	0.25	0.632	1.186
	2.098		1.213	
0.739	0.87	0.799	1.616	1.124
1.276	0.976	0.746	1.143	1.009
0.578	1.305	1.047	2.623	2.201
1.227	1.616	1.017	0.593	1.04
1.441	1.026	1.187	1.862	0.917
0.59		0.556	3.843	2.369
1.465	0.548	0.941	0.574	1.19
1.382	1.061	1.58	0.752	1.047
0.931	1.276	1.828	1.408	0.979
	0.35	0.935		
1.131	1.616	1.786	0.899	1.048
				0.784
0.358	0.07	0.466	0.662	0.277
0.256	0.346	0.2	4.542	0.243
1.154	0.863	0.458	0.496	1.001
0.919	1.154	1.571	0.686	1.184
1.097	1.29	1.11	0.764	1.499
1.431	0.225	0.654	0.863	1.164
0.849	0.781	1.027	1.343	1.385
0.565	1.755	1.31	0.603	0.49
1.065	1.117	1.806	1.165	1.564
1.006	0.767			1.261
0.281	6.302		1.22	
1.121	0.506	0.451	1.525	1.521
0.96	2.69	1.867	0.083	0.031
0.967	9.164		2.536	0.703
0.708	0.305	0.589	3.585	1.955
1.231	1.015	0.526	0.719	1.164
	1.716	1.87	1.566	0.483
1.178	0.869	0.799	1.08	1.429
0.88	1.013	1.341	1.1	1.158
1.765	0.95	1.2	1.355	0.805
0.765		1.249	1.202	1.06
	0.465	0.743		
1.059	3.314	0.988	0.723	0.628
0.592	0.312	2.539	0.774	1.525
1.441	2.414	1.728	0.615	1.101
		0.238		

1.028	0.932	0.94	0.794	0.961
0.872	0.722	0.765	0.384	0.762
0.734	0.377	0.57	2.054	1.165
0.991	0.86	0.986	1.18	1.565
	1.342	1.529	1.341	0.833
0.924	0.957	0.409	0.446	1.506
1.072	1.115	1.042	0.557	1.13
0.72		2.76	0.951	
1.29	0.753		1.624	1.728
				0.362
	0.535			
1.195	1.258	1.408	0.608	0.665
1.388				
1.157	0.473	4.126	1.648	0.182
1.446	1.216	0.844	0.615	0.906
1.615	0.424			1.378
1.15	0.929	0.915	1.066	1.155
1.811	0.776	1.25	0.467	0.618
0.733	0.915	0.989	2.778	0.966
1.197	0.98	0.934	1.7	1.794
0.987	0.723	0.84	0.655	1.176
	0.315	0.366		
0.18	5.102	5.083	0.119	
1.1	0.865	1.007	0.491	0.891
1.188	1.078	0.622	1.078	1.233
1.243	0.653		0.505	1.239
1.325	0.931	0.597	0.303	0.576
1.292	1.314	1.858	1.065	0.747
1.188	1.037	0.922	1.144	1.541
0.794	0.963	0.2	0.401	1.486
0.967	1.033	1.543	0.836	1.135
1.222	2.326	0.71	1.368	1.711
1.898	1.056	1.588	0.24	0.414
1.357	0.776	0.278	0.941	1.17
0.184	0.516		0.157	0.459
1.094	0.898	0.869	0.8	1.361
1.971	0.796	0.814	0.075	1.421
0.947	1.611	1.084	0.75	0.895
0.814	1.359	0.464	0.502	1.359
0.753	0.526			
1.004	1.078	0.907	0.753	1.037
0.663	1.772	1.408	1.365	1.496
1.067	0.876	1.035	1.695	1.081
1.077	0.876		1.132	1.229
1.201	1.09	0.658	0.85	1.044
1.21	0.979	1.086	0.545	0.871

0.81	1.278	2.514	1.628	0.709
0.961	1.207	2.785	0.573	0.903
1.374	1.096	1.704	1.099	0.841
1.124	1.039	0.918	1.891	1.348
0.107				
0.616	1.539	0.564	1.562	0.792
0.373				0.296
	2.672			
			0.287	0.16
		0.982		
1.175	0.588	0.099		1.175
2.717	0.682	1.259	1.143	0.444
1.267	0.662	1.342	1.601	1.429
0.692	0.956	1.736	0.717	1.093
	0.809			
0.645	0.468	0.584	0.672	0.495
1.227	1.109	0.962	1.175	1.149
0.992			0.931	
0.796	0.795	0.8	0.748	0.669
1.066	1.155	1.103	0.534	1.19
1.147	1.318	0.725	1.223	0.727
1.157	1.066	0.316	2.095	0.635
0.649	4.522	3.897	0.984	0.755
3.557	0.873	0.526		0.371
0.661	0.925	1.166	1.842	1.691
	1.284	0.678	0.275	
	0.084			
1.786	0.923	0.817	0.482	0.77
1.168	1.399	1.414	0.859	1.168
0.689	0.827	0.54	0.626	0.706
0.764	1.126	0.904	1.036	0.828
0.771	2.314	0.816	0.914	1.36
1.126	1.177	0.78	0.942	1.013
0.852	1.108	1.752	1.56	1.165
1.242	0.381	0.536	1.696	1.59
1.769	0.341	0.797	1.163	1.195
	1.614			
1.116	1.147	1.052	0.431	1.429
	1.358	1.759	1.052	0.486
1.456	0.948	1.337	2.173	0.287
0.248	2.431	0.384	0.809	0.542
0.548	0.646	0.419	0.459	0.885
0.447	1.399	1.044	0.515	0.595
0.868	1.124	1.475	0.914	0.484
0.996	1.519	2.131	1.08	0.889
1.3	1.02	0.905	2.466	1.066
		0.857	0.39	1.803
0.557	0.732	0.9	0.571	0.732
0.833	0.493	0.628	1.333	0.609

0.274	0.184	2.302		
1.417	1.004	0.457	0.973	1.395
1.361	1.106		0.875	0.808
		3.001		
2.304	1.62	0.917	0.691	1.242
0.722	0.76	1.159	1.013	1.546
0.993	1.147	1.254	0.445	0.252
	0.869		1.883	1.147
0.929	1.097	1.217	0.62	0.749
1.065	5.714		1.45	
0.787	0.861	0.53	1.285	1.157
0.798	1.09	0.86	1.258	0.947
0.849	1.538	2.061	1.487	0.568
1.079	1.057	0.861	0.389	1.351
0.121	0.204			0.339
0.802	0.846	0.782	1.063	1.312
	0.495			0.616
				0.51
1.087	1.567	1.062	0.89	0.702
0.615	0.571		0.062	0.95
		0.757	0.71	0.576
2.206	1.262	1.374	1.097	0.003
1.13	1.171	0.412	0.91	1.37
0.785				0.644
1.136	0.927	1.271	1.493	0.755
1.168	1.022	1.138	0.867	0.66
1.068	0.883	1.588	1.187	0.982
1.084	0.658	0.369	1.222	1.452
1.265	0.876	0.656	0.631	0.975
0.839	1.217	0.429	2.518	1.073
0.82	0.361	0.691	3.243	1.703
1.127				
0.845	1.183	1.516	0.706	0.925
1.267	1.142	1.278	0.456	0.626
0.865	0.587	1.287	2.636	2.079
1.077	1.108	1.754	0.99	0.925
0.318	0.251	0.356	0.629	0.35
1.005		0.613	0.984	0.875
1.032	0.95	0.742	0.599	1.21
0.244	1.738		1.186	0.38
0.688	0.249	0.007	1.383	1.36
0.977	1.208	1.289	0.852	0.946
0.175	2.089	0.538	0.852	6.581
0.379			0.429	
1.05	0.619	0.906	0.726	1.012
1.032	1.124	0.943	1.122	1.008
0.596	1.71	1.632	1.111	0.494
0.838	1.251	0.608	1.085	1.069

1.344	2.212	1.745	1.353	0.86
0.854	0.812	1.074	2.086	2.182
1.14	0.888	1.003	1.402	1.781
1.077	0.75	0.984	1.174	2.174
0.965	0.886	1.214	1.025	1.013
1.379	1.053	1.025	1.799	1.101
0.85	0.875	1.374	1.237	0.692
0.244	0.303	2.231	0.461	2.12
0.971				
			0.741	
1.002	1.41	1.19	1.312	0.756
1.472	0.856	1.172	0.72	0.844
0.866	1.243	1.619	0.374	1.001
	0.484	0.653	0.417	2.229
0.393		2.213	2.826	0.487
1.221	0.881	0.778	0.837	0.79
1.414	0.388	0.576	0.777	0.758
			0.868	
2.103			0.595	0.576
1.713	0.943	0.134	0.657	1.126
1.293	1.024	1.438	1.608	1.719
1.033	0.93	1.063	1.602	1.36
			1.43	
	1.028		1.264	0.715
1.292	0.276	1.761	0.719	1.544
1.435	0.756	1.141	0.25	0.393
0.795	1.048	1.452	2.172	2.38
0.834	1.504	1.018	0.765	1.029
1.107	0.95	0.701	0.304	0.279
1.597	0.639	0.454	0.591	0.698
0.705	0.956	1.477	2.086	0.987
1.068	1.085	1.203	0.451	0.698
0.508				
0.571	1.077		0.37	0.858
0.953				0.683
0.804	0.424		0.394	0.502
	0.645	2.58		0.76
	1.059	2.499		
1.033		0.669	0.567	
1.138	1.025	1.053	0.571	0.996
1.558	1.061	1.273	1.055	0.987
0.915	0.706	1.93	1.586	1.499
1.073	1.309	0.198	0.411	0.802
0.62	1.015	1.083	0.782	0.732
0.477	2.101		1.671	0.6
0.115	0.501	0.113	0.19	0.19
0.801	2.356	2.4	1.236	1.272
1.035	0.676	0.929	0.73	0.883
0.971	1.06	1.115	0.662	0.455

0.951	1.123	0.6	0.379	0.956
0.878	0.96	1.379	1.784	0.971
1.848	0.457	1.111	1.933	1.496
1.341	0.636	0.816		
1.089	1.58	1.98	0.739	0.868
1.172	0.849	0.386	0.807	1.456
2.034	1.117	1.099	0.527	
0.908	1.586	0.796	1.115	1.866
1.211	1.291	0.624	1.719	1.523
			0.126	
	0.533			
0.41	1.411	1.708	0.914	0.995
0.613		0.48		
	1	1.274		1.093
1.13	1.619	1.757	0.456	1.62
1.578	1.43	0.349	0.734	0.226
0.337				
0.698	0.863	1.312	1.866	1.567
2.011	0.894	0.181	1.299	1.903
0.282	3.555	11.012	0.75	0.25
		2.004		0.562
0.64	2.124	0.473	1.744	0.919
0.802	0.247	0.632	1.551	0.806
0.757	0.936	1.755	1.253	1.144
0.703	1.076	0.662	1.718	0.592
1.627	1.158	0.439	0.863	1.401
1.284	1.173	0.704	0.804	1.455
1.137	1.11	0.497	0.953	0.839
	4.507			
	0.542			
			0.678	
	0.375	0.345		
0.674	5.904	0.936	0.975	0.779
0.848	1.201	0.292	0.723	1.423
0.72	1.346		0.63	
0.774	1.249	1.71	0.565	0.359
0.569			1.917	
1.202	1.26		1.196	0.852
0.396	0.132	1.094	0.284	1.245
1.11	0.879		1.049	0.994
1.147	1.672	4.219	1.147	0.776
0.44	1.924	2.8	2.609	0.613

0.917	1.527	1.613	1.627	0.533
0.834	0.884	1.315	0.602	1.146
1.583	0.727	0.439	0.777	1.001
1.354	0.783	1.201	0.271	1.113
1.348	0.984	0.87	1.757	1.642
0.927	1.097	1.184	0.512	0.894
2.53	1.061	1.386	1.79	0.008
0.684	2.426	1.674	0.872	0.622
1.286	0.55	0.631	1.24	1.303
0.922	1.091	1.597	1.101	1.511
1.037	0.384	0.4		0.592
0.789	3.182	2.091	1.731	0.392
1.236				1.215
1.087	0.732	0.672	0.56	1.074
0.243	0.837	1.23	0.673	0.491
			1.59	
0.622	1.247	1.462	0.605	0.566
	0.483			
1.19	0.888	0.61	0.69	1.152
0.128			0.377	0.612
1.401	1.016	1	1.614	1.26
0.77	1.033	1.252	0.885	0.746
0.627	0.865	7.608	1.337	1.186
0.988	1.081	0.874	0.934	0.926
0.935	1.628	0.444	2.419	1.132
0.778	1.537	2.429	1.044	0.876
1.027	0.84	1.127	1.064	1.287
0.593				
0.893	0.532	1.034	0.39	0.245
		0.222	1.248	
0.85	0.995	1.316	0.972	2.246
0.803	1.19	1.12	1.055	1.073
0.905	0.128	0.232	0.071	0.437
0.535	1	1.047	0.381	0.662
1.16	1.631	2.526	0.473	0.518
1.29	0.819	0.532	0.315	1.068
1.078	1.077			
1.484	0.877	0.398	0.979	1.064
0.833	2.337	1.122	0.944	0.899
0.971	2.157	0.867	1.384	1.207
				0.842
0.9	1.329	0.794	0.897	0.83
	4.079			0.421
	1.033		0.878	
0.731			0.366	0.367

0.711		0.446		
0.343	1.433	1.355	0.746	0.252
1.072	1.065	0.739	1.296	1.024
0.595	0.447	0.888	1.658	4.552
0.86	0.932	0.347		1.21
1.219	1.284	1.283	2.269	1.078
	0.64			
1.254	0.654		1.834	1.648
0.295	0.928	0.463	1.016	1.872
	0.751	0.851	0.479	
0.26	1.886	0.386	2.288	2.666
		0.615		
1.041	0.605	0.443	1.452	2.335
1.113	1.166	0.933	1.118	0.876
1.029	1.126	0.876	1.688	1.407
0.686	0.79	1.193		
0.692	0.833	1.272	1.498	1.508
0.905	2.11	0.95	0.642	0.742
1.284	1.886	1.205	0.294	0.201
1.638	0.756	0.41	0.866	0.832
4.725	0.848	1.038	1.066	0.85
0.849	1.098	1.193		
1.109			0.206	1.127
0.997	1.074		1.131	0.62
1.178	1.163	0.455	0.624	0.991
1.389		1.766	2.001	1.017
0.846	1.456	0.886	0.42	0.586
0.45	0.113	0.188		
1.003			1.03	1.48
1.048	0.628	0.924		1.036
0.353	0.027			
1.242	0.809	0.638	1.052	0.904
1.564			0.474	
0.76	0.343	1.749	1.032	0.961
0.59	1.661	0.923	1.605	0.69
			1.002	
1.203	0.556		2.172	1.144
0.712	0.838	0.961	0.725	1.092
1.587	1.349			1.247
0.955	0.695	2.539	1.332	1.023
1.709		1.232	0.709	0.47
	0.899		0.956	0.999
1.285	0.302	1.98	0.486	1.078
0.81	1.276	1.473	1.691	2.165

1.428	0.357	0.468	0.889	0.894
1.343	1.716	1.176	1.381	0.439
	1.408			
				1.194
1.173	1.497	1.312	0.908	0.861
				1.13
1.135	1.005	1.449	0.919	1.076
		0.429		0.678
0.895			1.819	0.664
1.029	1.248	1.241	1.437	1.892
1.365	0.797	1.043	0.073	1.302
0.709	4.767	3.288	0.536	0.156
0.817	2.918		0.771	0.279
0.778			1.4	1.384
1.158	0.879	1.71	0.649	0.616
0.978	1.22	0.925	1.507	1.229
1.336			0.91	0.842
1.064	1.253	1.862	2.36	0.259
1.236	0.415	0.79	0.817	0.571
0.277	0.2	2.388	0.298	0.134
	1.079		0.623	1.073
1.148	0.943	0.932	2.036	1.563
1.117	2.175	2.046	1.037	0.666
1.102	1.16	1.43	1.396	1.042
1.131	2.898	1.679	1.368	0.967
0.749	0.64		1.13	1.293
0.865	0.761	0.71	0.556	0.685
0.911	0.865	1.286	0.755	0.99
0.568			1.091	
0.904	0.452	1.04	0.604	0.802
0.939	0.544	0.694	0.959	
1.187	0.581		0.47	
1.004	1.072	0.418	0.777	1.622
0.564	0.618	0.496	3.243	0.767
0.713	2.045	0.658	0.4	0.432
0.615	0.7	0.845	1.326	0.77
0.964	1.914	0.636	0.84	0.029
1.101	1.1	1.038	0.899	0.844
	0.789			
			0.877	1.363
1.246	0.721			0.897
0.901	1.143	0.804	1.144	1.308
0.906	0.302		0.304	
0.788	1.15	1.412	1.18	0.53
0.635	1.018	1.29	0.069	0.606

1.603	1.261	1.61	1.698	1.557
0.574				
1.445	0.635	0.921	0.845	1.137
1.141	1.764	1.511	1.066	1.238
0.992			0.597	
0.685	0.492			1.42
1.228				1.087
0.864	2			0.521
1.165	1.075	0.877	0.732	1.163
			2.587	1.607
0.822	0.844	0.996	1.284	1.189
0.397		4.203	1.145	0.446
0.949	0.658	1.965	1.451	1.142
0.965	1.328	1.564	2.151	0.965
1.172	0.627	0.605	0.224	0.628
0.698	0.602	1.532	1.127	0.906
		2.924	0.407	
1.344	0.952	0.584	0.649	0.827
1.468	1.202	0.556	1.097	1.409
0.763	1.142	0.765	1.347	0.905
0.358	0.748	2.261	0.965	1.322
0.819			0.659	0.914
0.732	0.397	0.732	3.459	2.152
	0.71	0.954	0.708	
	0.431			1.191
1.635	1.767	1.519	0.927	1.11
0.95			0.944	1.184
0.904	0.829	0.523	1.288	1.142
1.054	1.057	0.86	0.36	1.693
1.274	1.212	0.793	0.779	1.118
	0.904	0.207	1.973	0.869
1.452	0.766	0.793	1.481	0.709
1.557	2.007	0.772	1.528	1.123
0.846	1.045	0.804	0.393	0.589
0.996		1.202		
0.578	8.098	3.266	0.253	0.274
1.684	0.361	0.183	0.281	1.356
1.319	1.365	1.588	1.051	1.009
1.564		1.108	1.216	0.978
1.314	1.367	0.523	1.727	1.171
1.28	1.877	1.183	1.802	0.217
0.544	1.275	1.47		0.776
0.78	0.408			0.482
0.935	1.084	2.078	0.938	1.325
0.952	1.389	2.146	1.409	1.513

1.229	1.377	0.139	0.548	1.465
0.646		0.204		
0.962	1.573	0.297	1.18	1.673
1.111	0.488	0.693	0.914	1.154
1.987	1			1.145
0.846	0.97		0.985	1.698
1.285	1.187	1.358	1.573	1.479
1.142	1.916	2.838	1.475	0.563
0.503	1.127	1.218	0.408	1.567
0.423	1.532		1.788	
0.774	1.049	0.443	0.463	0.538
0.911	0.944	1.197	0.632	0.974
1.573	2.074	1.029	1.067	0.729
1.148	1.55	1.376	1.178	0.973
0.76	1.458	0.743	0.707	0.936
0.972	0.913	0.64	1.501	1.665
1.255	0.984	1.15	1.378	1.643
1.572	0.709	0.801	0.849	1.191
0.79	1.222	1.646	0.798	0.701
1.229	0.477		0.222	0.667
1.183	0.998	1.186	0.726	0.574
0.841	1.104	1.16	0.793	0.533
0.64	0.988	1.404	2.278	2.114
0.628				0.74
0.881	0.379	0.914	1.46	1.309
0.69	5.348	0.909	0.398	0.223
0.898	1.488	1.697	0.795	0.843
1.27	0.943	1.023	1.073	1.024
1.162	1.666	0.693	1.924	1.256
0.924	1.011	1.334	0.785	0.611
1.527	0.572	0.518	0.427	1.276
0.575	0.626	1.343	0.859	1.03
	8.334		0.31	
1.081	1.182	1.222	1.214	0.455
1.52	1.552	0.413	1.378	1.143
0.852	1.737	1.606	2.107	0.584
0.566	1.968	1.235	1.424	0.937
0.949	0.991	1.009	1.068	1.185
1.245	1.323			
0.838	0.422	0.901	1.404	2.054
1.492	1	0.741	0.698	1.313
		0.481	0.145	
0.243	0.331	4.063	0.359	0.402
0.865	1.269	1.213	0.765	0.888
0.891	1.099	1.236	1.212	1.132

0.756	0.905	1.832	0.896	0.676
1.468	1.148	1.22	0.611	0.727
1.428	1.361	1.155	1.542	1.651
0.837	1.666	1.086	0.746	1.093
0.518	0.546	0.422	1.022	2.146
0.741	1.065		0.716	0.699
0.996	0.881	1.149	0.417	0.811
1.347	1.779	0.926	0.493	0.322
0.668			0.354	1.363
1.494	1.098	0.277	0.819	1.433
0.679	0.985	0.843	0.551	1.189
1.572	0.129	0.153	0.313	0.564
1.031	0.808	0.745	0.814	0.901
1.072	0.583	0.341	0.626	0.764
0.49		5.802	1.477	0.908
1.075	1.138	0.829	0.611	0.726
1.041	0.836	1.473	1.088	0.859
1.669	0.643	0.152	0.307	0.792
1.36	0.626	0.627	0.67	0.731
	1.06			
1.098	0.985	1.38	2.378	0.657
0.894	0.446	0.332	0.383	0.857
1.14	0.93	0.993	0.432	0.701
	2.322	7.188	1.06	
0.954	0.798	1.038	1.296	1.377
0.683	0.838		1.907	0.941
1.306	1.964	0.342	1.262	0.189
	6.273	0.931	0.47	0.762
0.71	4.633	1.367	0.949	1.035
1.359				
0.983	0.914	0.605	0.876	1.234
	1.654	1.34	2.149	5.031
0.979	2.18	0.653	1.693	1.173
0.528	0.81	0.84	1.164	0.646
0.47	6.679	3.998	0.767	0.603
0.927	1.933	0.749	0.495	1.386
1.455	0.912	0.447	1.113	1.449
0.439	0.799	3.173	0.497	0.743
0.938	0.652	1.035	2.477	0.873
	0.543	1.33	1.221	2.485
1.133	0.871	0.754	2.583	0.661
1.311	0.91	0.779	0.704	0.633
0.376	1.589	0.732		0.203
0.645	0.644	0.797	0.383	1.961
1.483	0.516	0.805	1.799	1.939
0.48		2.263	0.79	
0.804	2.549			0.36
1.314	0.531	1.472	0.713	1.525

1.884	1.552	0.232	1.299	1.364
1.187	1.144	1.211	1.888	1.096
1.2	1.381	1.167	1.712	1.201
0.292	0.209	0.076	0.125	0.32
1.272	0.755	0.791	0.615	1.384
0.77	1.24	1.107	2.489	1.398
0.915	0.103		6.784	0.312
1.066	0.79	0.779	0.497	1.135
2.304	1.124	0.645		
1.048	1.123	0.937	1.978	
0.955	0.977	1.26	1.523	0.428
0.801	1.472	1.107	0.812	0.402
	1.09		1.628	0.519
0.277	0.733	1.246	0.31	0.169
1.051	1.143		0.319	0.094
0.622	2.482	0.949	1.644	1.298
1.003	1.03	0.625	0.75	1.144
0.204	1.31	0.338	0.432	
0.799	1.007	1.825	0.83	2.379
0.95	0.786	1.472	0.999	1.149
0.846	0.883	0.553	1.367	1.219
0.507	2.019	1.779	2.729	0.695
0.706	6.432	1.084	0.565	0.405
0.966	1.133	1.014	0.996	1.246
0.67	0.978	0.607	0.155	0.236
1.988	0.915	0.511	0.424	1.313
1.034	0.774	0.469	0.758	1.34
1.108	0.953	1.203	1.331	1.269
0.215	0.232	0.144	0.246	0.293
				0.595
1.199	0.842	1.394	1.012	1.24
1.11	1.071	1.355	1.316	1.105
2.078	0.214	0.903	0.165	0.198
1.718	1.704	0.415	1.616	0.976
1.447	0.114	0.8	0.475	1.493
0.492	2.969	2.765		0.907
1.413		0.754	6.156	0.437
1.581	1.05	0.751	0.747	0.873
0.83	0.799	1.024	0.801	1.68
1.14	0.478	0.596	0.124	1.675
2.071	1.212	2.083	1.371	0.045
1.339	1.172	0.576	1.052	0.959
	0.559	1.153	1.112	0.728
1.096	1.006	1.488	1.086	0.987
0.807	1.323	1.77	1.497	1.063
0.567	0.984	1.384	1.088	1.16

0.839	0.903	0.385	1.22	1.435
1.45	1.048	1.533	0.958	1.116
0.849	0.859	0.801	0.415	0.828
1.037	0.985	0.521	0.622	0.625
0.685	0.95	0.954	1.275	0.878
1.323	1.1	0.873	0.537	0.895
1.059	1.002	0.626	0.884	1.397
0.841	0.997	1.483	1.39	0.939
1.175	0.865	0.777	0.493	0.91
0.224	0.261	0.187	0.677	0.59
0.667	1.384	1.525	1.69	0.478
1.041	1.284	0.71	2.584	0.713
	4.479		0.295	0.358
1.692	1.626	0.654	0.609	1.146
1.644	1.157	0.781	0.775	1.293
1.448			0.728	
1.247	1.117	1.198	0.379	1.058
1.176	1.948	1.828	0.481	0.443
0.743		2.211	2.585	0.591
1.117	0.389	1.015		0.838
1.002	3.939	2.659	1.792	0.477
1.079	0.864	0.339	0.943	1.223
1.346	1.232		0.774	1.608
1.386	1.189	1.098	1.073	1.044
0.901	2.307	1.835	0.851	0.739
0.48	0.812	0.762	1.145	0.673
0.954	1.065	0.786	1.262	0.672
	5.469	1.626	0.875	0.07
0.387	1.314	2.185	0.779	1.328
	3.685	0.661	1.094	0.742
0.951	0.909	0.718	0.596	1.926
1.136	1.338	1.507	0.733	0.903
1.152	1.378	1.41	0.905	1.043
0.954	1.35	0.818	0.582	0.544
1.044	1.416	1.349	1.444	0.826
1.302	1.074	0.816	0.607	1.001
1.73	1.03	0.671	0.931	0.821
1.151	0.923	0.869	1.181	1.139
	0.166	1.245	1.044	
0.495	0.541		1.063	0.703
1.265	1.115	1.808	1.024	1.062
0.991	1.461	1.012	1.518	0.685
0.82			1.919	1.521
0.61	0.656	0.692	1.151	1.594
1.6	2.553	2.473		
0.657	0.926	1.667	1.045	0.927
	2.197	2.293	0.89	1.54
1.189	0.959	0.868	0.639	0.937
1.311	1.012	1.103	0.903	1.16

	0.677		1.462	1.618
0.424	1.921	1.913	1.666	
1.456	0.696	1.484	1.465	2.204
0.986	1.047	0.759	0.662	0.889
1.133	0.985	0.651	1.658	1.592
0.841	1.167	1.067	1.059	1.207
0.873	0.43	0.817	1.526	2.253
1.212	1.241			
1.16	1.965	0.346	1.739	1.585
2.115	0.894	1.145	0.899	0.985
1.483	1.027	0.147	0.686	1.327
0.855	0.937			
0.388		0.499	0.51	0.337
1.693	0.64	0.981	0.922	0.343
0.598	3.382	4.239	0.88	0.848
0.567	1.765	0.299	0.584	0.897
0.771	1.172	0.945	1.185	1.639
1.66	1.437	1.252	0.918	1.73
0.759	0.507	0.614	1.27	1.877
1.172	0.942	1.02	0.459	1.186
0.557	2.075	1.351	1.799	0.701
1.273	0.964	1.364	1.004	0.946
0.928	0.502	1.033		2.614
	0.191			0.27
1.094	0.953	1.048	1.148	0.977
0.542	0.706	0.709	1.111	0.893
0.939	0.772	0.509	0.737	1.644
0.552	1.228	1.361	1.681	1.558
0.804	2.386	1.108	1.337	1.374
			1.492	0.658
1.287	1.252	2.145	1.317	1.414
			0.161	
1.082	1.123	1.442	1.179	1.175
0.821	1.61	0.671	1.008	1.373
0.9	1.225	3.37	1.048	0.803
1.143	0.85	1.1	1.106	1.465
1.409	0.824	0.141	0.162	0.037
1.146	1.334	1.293	1.204	1.023
1.372	1.01	1.265	1.337	0.992
0.634			0.921	
1.366	1.378	0.974	0.143	2.443
1.626	1.428	1	0.337	0.643
1.309	1.088	1.477	1.301	0.775
0.928	1.499	0.718	0.644	0.444
1.032	1.014	1.243	0.418	0.93
0.5	0.556	0.539	0.305	0.383
0.967	1.186	2.037	0.863	0.883
	0.678		0.407	0.975
0.224	3.456	3.04	1.288	1.474
0.871		1.313	2.486	1.683

0.757				0.437
1.375	0.796	0.755	0.655	1.029
1.148	0.994	1.375	1.463	1.214
1.394	0.731	0.732	0.394	0.648
1.315	1.032	0.863	0.518	1.062
0.568	1.313	0.558	1.48	0.746
0.8	1.938	1.356	2.124	1.064
0.523	2.216	0.495	2.409	0.308
0.395		0.54		1.513
0.82	0.537	1.898	0.887	0.944
1.232	0.837	0.796	2.1	1.206
0.507	0.829		0.612	1.082
0.914	1.433	1.372	1.291	0.622
	2.012			
1.116	0.495	0.917	2.979	1.177
0.599	0.811	0.907	1.188	1.024
0.736	0.758	0.548	1.012	4.415
0.896	1.075	0.943	1.875	2.212
0.838	1.459	1.396	1.578	1.433
0.87		0.71	0.09	0.116
1.14	1.414	0.76	0.842	1.118
1.017	0.923	0.936	1.012	1.094
	1.05		1.497	
1.407	1.629	1.437	0.842	0.821
0.686	1.591	0.749	0.996	1.282
1.511	0.599	0.397	0.371	0.987
	1.321	0.457	1.786	0.578
1.289	1.24	0.919	0.892	0.896
1.603	1.696	1.663	1.062	0.678
0.936	0.79	0.423	1.559	1.59
3.835	0.467	0.7	0.457	0.582
1.011	1.077	1.115	1.049	1.205
1.087	1.303	1	0.385	1.051
1.377	0.748	0.875	1.327	1.082
1.106	1.594	1.952	0.359	0.486
1.122	0.83	0.904	1.048	1.441
1.223	0.701	0.945	0.193	0.798
0.57	1.293		2.236	0.398
	0.814	1.118	1.385	1.321
1.276	1.758	1.669	2.845	1.226
0.79	0.571	0.628	1.789	0.817
0.732	0.468	0.19	0.213	0.145
1.001	2.83	1.904	1.069	1.035
0.75	2.494			
0.304	1.055	0.41	1.302	0.515
0.968	1.01	1.291		1.461

2.355			1.025	
1.01	1.085	0.647	0.708	0.958
0.555	2.347	0.758	0.451	0.83
1.113	1.276	0.968	1.128	0.953
1.131	0.931	0.998	0.648	0.625
0.891	0.967	0.386	3.733	0.533
0.248	6.329	8.728	0.336	0.205
	0.842	1.784	2.832	0.89
1.192	1.119	1.146	0.032	1.536
1.292	0.831	1.102	1.773	1.946
0.844	1.145	0.988	0.827	0.77
1.184	0.957	0.749	0.677	1.253
1.062	0.982	1.152	1.607	1.407
1.34	0.864	1.193	1.654	1.807
0.919	0.958	0.983	0.498	0.933
0.735	1.273	1.155	0.891	1.327
1.364	0.649	0.436	0.614	0.939
1.092	0.672	0.861	1.665	1.721
1.243	1.227	1.073	1.282	0.982
0.869	2.641	3.152	0.834	0.727
0.525	7.257	0.359	1.156	0.437
1.014	0.778	0.379	0.777	0.814
1.571	1.169	0.057	0.038	3.38
1.445	0.876	1.211	0.879	1.004
0.349	0.538	0.654	0.483	0.975
0.74	0.93	0.833	0.428	0.709
0.977	1.215	1.093	1.114	0.929
0.991	1.429	1.138	1.332	0.977
0.645	0.89	0.665	0.836	0.879
0.702	1.06	0.732	1.222	1.239
1.402	1.058	0.352	0.692	1.301
0.68	0.765	1.045	0.516	0.835
1.072	0.823	2.166	0.873	1.599
0.864	1.19	1.605	1.029	1.148
0.95	1.341	1.355	0.392	0.61
1.167	1.151	1.052	1.274	0.721
0.732	1.554	0.582	1.107	1.42
1.593	0.306	0.674	2.094	1.204
1.19	0.339	0.816	0.539	0.993
	4.305	0.806	0.348	0.153
0.501	0.62	0.642	0.158	
1.083	0.825	1.927	1.016	1
3.318	0.757	1.421	1.086	0.263
1.144	1.22	1.632	1.089	0.957
0.951	0.746		0.964	1.245

1.406	1.147	0.741	1.086	1.228
1.689	0.941	2.411	1.577	0.255
1.234	0.888	1.402	1.152	1.181
1.085	0.715	1.125	0.639	0.734
1.143	0.887	0.992	0.606	0.912
1.05	0.79	1.051	0.56	0.796
1.281	0.249	0.134		1.931
1.127	0.702	0.369	0.409	0.748
0.79	0.855	1.241	0.852	0.861
0.807				
1.204	0.833	0.647	1.157	1.113
1.161	1.281	0.343	1.376	1.372
0.972	0.99	0.469	1.351	1.036
1.189	1.394	0.671	1.108	0.942
1.459	1.412	0.976	1.34	1.426
	2.564	2.838		
1.299	1.14	1.967		1.047
0.773	0.805	1.172	0.828	1.845
0.945	0.99	1.562	1.302	1.252
0.444	0.828	0.601	0.723	0.393
0.778	0.636	0.569	0.212	1.313
1.599	1.254	0.897	0.948	1.218
1.166	1.498	0.266	1.042	1.43
0.397	8.104	0.29	1.167	1.231
1.419	0.92	0.327	0.993	1.292
1.225	0.365	0.355	0.321	1.629
0.572	0.366	2.728	0.375	0.218
1.191	1.58	1.422	0.698	1.191
	0.652	0.957	0.769	
0.583	2.412	2.043	1.509	0.853
1.009	1.009	1.11	0.966	0.775
1.305	1.266	0.747	1.318	2.179
1.082	1.763	0.731	0.971	0.929
0.87	0.806	0.566	1.01	1.203
1.251	1.227	0.459	0.658	1.028
0.991	0.928	0.89	2.088	0.93
1.136	1.028	1.221	0.798	0.952
1.125	1.092	0.903	0.686	1.24
		0.597	1.943	
0.772	2.01		0.795	0.929
0.38	1.406	0.825	2.192	0.963
0.903	0.638	0.63	2.186	1.314
0.996	1.244	1.172	0.726	0.841
0.966	1.151	1.434	0.591	0.899
3.191	0.223	0.277	0.569	3.403
0.914	1.047	0.888	1.784	2.357
1.105	0.85	0.487	0.487	0.852

1.336	0.878	0.602	0.472	0.828
1.079	0.999	1.887	1.618	1.081
0.712	1.24	2.158	1.073	0.272
1.413	0.844	1.056	0.761	1.315
1.469	0.843	1.207	0.575	0.984
1.206	0.904	1.46	1.158	1.23
1.112	0.884	1.04	1.143	1.054
1.413	0.787	0.522	0.564	0.938
1.29	1.057	1.046	0.721	0.966
1.494				0.765
0.76	1.15	0.746	0.947	1.036
0.341	1.109	1.718	2.37	1.29
1.109				
	0.588	0.752	2.378	1.266
1.411	0.941	1.422	0.826	1.041
1.119	0.817	0.855		1.334
0.772	0.953	0.969	1.266	1.302
1.142	0.971	1.368	1.308	0.992
3.386	0.388			0.393
1.902	0.904	0.198	0.962	1.456
1.273	0.809	0.858	0.519	1.174
0.587		2.093		
0.501	0.594	0.775	0.923	0.896
1.16	0.719	0.999	0.936	1.013
1.184	1.546	1.492	1.115	1.195
0.627	1.284	1.971	1.467	0.891
	0.632	0.817		
0.717	0.784	1.02	1.026	1.209
0.997	1.52	0.796	0.706	0.585
1.054	1.061	0.988	1.215	1.159
1.522	1.061	0.59	1.386	1.315
0.787	1.552	1.012	0.704	0.649
1.255	1.213			1.06
1.242	1.606	1.995	1.394	0.525
0.73	1.188	0.796	1.065	0.95
1.257	0.796	0.7	0.616	0.88
	1.123			
		1.669	1.726	2.295
0.864	0.713	1.104	0.972	1.546
1.369	0.964	0.126	1.056	1.025
1.053	1.128	1.613	1.021	0.864
1.463	0.849	1.431	1.18	1.318
0.82			1.051	
0.615			1.139	1.654
1.053	1.185	1.042	0.692	0.746
0.963	0.959	0.659	1.371	1.224

1.349	0.757	0.923	0.48	0.436
			1.238	
0.859	1.619	0.612	0.712	1.345
0.598	1.16	2.314	1.191	0.342
1.163	0.782	0.895	0.472	1.127
0.416	0.58	0.482	1.306	1.586
0.89	1.201	1.208	1.575	0.864
0.306	0.812	2.102	1.877	0.711
1.289	0.893	0.707	1.883	1.663
0.361	0.646	0.771	1.805	1.905
0.922	0.517	0.55		
	0.917			
0.776	1.392	1.77	1.793	1.003
1.063	0.651	0.912	0.853	0.909
1.162	1.211	0.409	1.136	
1.526	1.175	0.474	0.837	1.154
0.077	2.217	2.502	1.153	0.595
0.945	1.248	1.746	1.678	1.581
2.628	0.908	2.119	0.88	0.831
4.959	0.587	0.69	0.478	0.722
0.486	0.392	1.288	0.772	1.392
1.047	0.633	0.801	1.522	1.232
1.286	0.909	0.592	0.663	1.349
1.315	1.819	1.253	0.723	0.556
0.886	1.441	1.795	1.164	0.986
1.019	1.167	1.743	0.87	1.374
1.344	1.218	0.766	0.678	0.729
1.264	0.671			0.545
0.963	1.024	0.412	0.795	1.227
0.696	1.148	0.621	1.18	0.594
1.249	1.366	0.709	1.064	1.194
0.971	1.517	0.863	1.045	1.453
0.949	0.953	0.882	1.217	1.291
1.194	1.575	1.763	1.409	0.868
1.056	1.405	0.871	0.088	1.219
1.007	1.149	0.91	1.55	0.982
1.17	0.798	1.289	1.562	1.425
		0.904	1.022	
1.606	0.73	1.229	1.146	1.437
0.714	0.824	1.051	1.264	1.407
1.301	0.75	1.284	1.556	1.683
1.167	1.268	2.166	1.504	1.559
0.699	0.646	0.755	1.457	2.046
1.818	1.082	1.063	1.124	0.089
0.566	0.983	1.128	2.588	0.715
1.386				0.475
2.31				

1.114	1.126	0.91	1.007	1.03
	0.579	0.153		1.03
			0.899	
1.099	0.727	1.281	0.526	0.553
2.944	0.098	2.079		3.679
0.747	0.942	3.187	0.441	0.9
1.304	0.982	0.972	0.723	0.942
1.301	0.609	0.366	0.359	0.65
0.855	0.785	0.431	2.004	
2.122	0.486	0.796	1.126	0.915
1.47	1.314	1.048	1.39	1.183
1.973	0.984	0.971	1.891	1.3
0.589	6.848	0.742	1.457	0.9
				1.642
0.957	0.84	1.122	0.856	1.217
0.938	1.108	1.267		0.977
1.812	0.395	0.373	2.239	
1.575	1.023	1.678	0.694	0.832
0.906	0.86	1.027	1.762	1.489
1.38	0.842	0.857	1.936	0.9
0.818	0.682			0.902
1.252	0.874	1.734	0.967	0.856
0.574	0.939	1.533	0.528	0.941
0.871	3.341	0.773	0.581	0.542
1.416		1.876	5.599	2.113
0.75			2.517	1.028
1.192	1.366	1.303	2.244	1.03
0.842	0.946	1.15	2.261	1.841
0.969	1.19	1.243	0.985	0.927
1.185	1.54	1.112	1.552	1.198
1.185	2.484	1.614	0.641	0.614
1.248	0.649	0.287	1.566	1.254
1.236	1.26	1.241	1.266	1.011
0.629	2.435	2.574	0.748	1.113
1.156	3.212	2.988	1.184	0.715
0.513	3.634	9.913	0.582	0.551
	2.426	0.569	0.506	0.329
1.514	1.537	0.503		0.066
0.682	1.095	0.865	1.434	1.035
0.506	5.05	6.484	1.048	0.826
0.457	0.956	2.735	0.578	
1.315	0.957	0.362	0.732	1.002
0.857	0.568	1.095	2.372	1.752
	0.839	1.171	0.847	
1.201	0.658	0.891	1.056	1.388
0.866	0.631	1.66	0.539	0.694
1.153	0.587	1.405	0.504	1.027
0.6	4.541	1.732	0.878	0.84
1.291	1.881	0.838	0.262	0.297
0.139	0.146	0.218	0.167	0.22

1.196	0.417	0.492	2.597	1.811
1.176	1.031	1.099	0.4	0.708
0.776	0.926	1.717	1.31	0.648
0.274	4.775	2.198	0.209	1.702
	0.396	0.997	0.533	2.325
1.584	1.226	1.173	1.089	1.109
0.998	1.479	1.43	0.97	0.659
0.616	0.911	0.971	1.264	1.559
1.111	1.229			
1.117	0.984	1.319	0.842	1.048
0.97				1.41
0.776	1.085	2.446	1.637	1.585
0.791	0.91	0.853	1.009	1.251
1.723	2.211	0.182	0.591	1.852
0.893	0.767	0.867	0.468	1.421
1.31	0.528	0.662	1.681	1.968
1.292	0.871	1.386	1.016	0.945
0.685	0.465	0.788	1.059	1.12
0.897	3.019	8.347	0.605	0.887
1.435	0.557			1.205
	0.294	0.397	0.314	
1.561			0.704	0.753
0.961	1.308	0.886	1.494	1.389
0.674	0.875		0.656	0.544
0.634	0.371	0.621	1.843	1.617
	6.148			0.409
		1.345		
0.587	4.218	0.845	1.371	0.699
0.599	0.74	1.075	1.666	1.446
1.171	0.959	1.271	2.071	1.643
1.124	1.623	1.878	1.307	0.871
0.897			0.719	0.298
0.516	0.955	1.393		
1.289	1.451	0.425	1.154	1.293
1.087	1.056	1.189	0.386	0.474
0.663	0.415	0.61	0.795	0.697
1.376	0.977	1.41	0.951	1.785
0.78	2.029	3.305	1.754	0.76
1.333	1.488	1.522		1.004
1.206	0.695	1.797	1.484	0.753
1.247	0.773	1.65	1.476	0.977
1.539	0.435	1.131	0.93	0.738
0.983	0.71	0.728	0.906	1.514
0.041	0.936	1.079	0.536	0.022
0.879	1.112	1.409	1.191	0.723
1.107	0.841	0.357	0.571	1.156
1.424	0.728	1.215	0.224	0.14
0.919	0.76	1.247	1.469	1.093

0.525	1.713	2.167		
1.153	1.508	1.067	2.144	0.992
1.226	1.872	2.818	1.208	0.659
1.292	0.434	0.588	0.394	0.449
1.042	1.668	1.641	0.134	1.068
0.997	0.974	1.237	1.159	1.214
1.028	0.757	0.606	2.008	1.864
1.446	1.007	1.243	0.819	0.998
0.872	1.214	1.37	1.354	1.054
0.517	0.902	0.728	1.611	1.002
1.219	0.667	0.953	1.564	0.882
1.168	1.157	0.389	0.904	0.87
0.872	1.535	1.866	1.822	0.958
0.734	0.581			
0.18	4.715	1.234	0.521	0.099
	0.413			
1.332	0.229		1.637	0.399
	1.048		1.034	1.331
1.517	0.965	0.947	0.58	1.556
0.852	1.469	1.424	1.207	1.168
0.992	0.815	1.349	1.056	1.204
0.751	0.923	0.549	1.856	0.472
2.388	3.084	0.374	0.095	2.423
1.052	0.882	1.096	0.667	0.963
0.781	0.815	0.998	0.953	1.262
0.976	1.517	1.054	1.202	0.815
0.522	0.405	2.035	2.812	0.966
1.084	0.836	1.187	0.662	1.084
0.792	1.054	1.287	1.736	0.875
1.032	1.301	1.05	1.302	1.32
1.026	0.101	0.292		1.111
0.973	1.044	0.943	1.156	1.275
1.136	0.944	0.883	0.987	1.256
1.381	0.411			
1.453	0.83	0.624	0.907	1.483
0.959	1.372	2.03	1.59	1.009
1.587	0.74	0.398	0.945	1.159
0.866	1.52	1.895	2.1	0.477
	0.52	0.931	1.374	0.796
	6.837	0.362	0.323	0.399
0.778	1.593	0.959	1.24	0.891
0.946	0.466	0.503	1.121	2.079
0.78	2.199	2.042	1.316	0.827
1.119	1.27	0.176	0.521	1.094
2.319	1.714	0.541	2.264	2.392

1.224	0.569	0.244	0.208	1.254
0.569		0.152	0.677	1.949
0.87	0.526	1.219	0.794	0.834
0.236	0.637	0.293	1.148	0.719
0.461	2.602	1.178	0.79	0.762
1.742	0.365		0.766	1.191
0.799	0.747	1.134	1.193	0.457
1.221	1.225	1.709	1.079	0.914
0.885	1.006	1.024	1.023	0.573
1.19	1.014	1.286	2.053	1.567
1.039	0.688	1.232	0.965	1.015
1.225	0.727	1.75	1.927	1.082
1.533	0.994	1.616	0.94	0.951
1.309	0.662	0.924	0.87	1.1
	0.571	0.843		0.736
0.751	1.177	1.35	1.72	1.456
0.949	2.361	2.073	1.509	0.404
0.9	0.294	0.801		1.204
0.563	3.608	0.657	6.29	0.593
0.912	1.61	1.367	0.685	0.738
1.189	1.308	0.692	0.983	1.155
0.764	1.734	0.174	1.285	0.723
0.829	0.382	0.118	0.358	1.285
1.26	1.106	1.303	0.83	1.025
0.754	0.888	0.602	2.054	0.73
0.058	0.351	0.415	0.23	0.561
0.934	0.738	1.199	1.108	1.152
0.737	1.11	0.926	1.464	0.743
1.181	0.829	0.568	0.23	0.954
0.888	0.801	1.935	0.655	0.869
0.598	2.201	0.277	0.695	0.091
0.271	0.415	0.413	1.053	0.345
1.092	1.16	1.097	2.125	1.747
1.269	1.084	1.514	1.218	1.026
	0.708	0.519		
0.819	0.609	0.925		0.954
1.58	0.583	0.751	0.577	1.171
0.462	0.653	0.928	1.527	1.306
0.657	4.287	4.739	0.888	0.736
1.577	2.042		1.167	0.801
1.343	0.982	0.884	0.902	1.505
0.843	0.269	0.946	2.154	0.586
1.116	1.181	0.471	0.711	1.153
	1.703	1.611	1.034	0.512
1.17		0.62		

1.222	0.953	1.077	0.921	0.981
1.534	2.995	0.873	1.215	1.6
0.539	1.394	1.561	1.454	0.856
1.154	0.571	1.11	1.196	2.243
1.301	0.943	1.141	1.524	0.82
1.263	1.612	1.423	1.03	0.554
1.079	0.285	0.336	0.695	1.35
0.712	4.879	3.168	0.73	0.589
0.922	0.891	2.289	2.133	0.623
				1.358
0.799	0.564	0.624	1.418	0.927
1.093	1.424	0.81	1.317	1.635
1.178	0.714	0.724	1.181	1.052
0.772	1.003	0.653	1.387	1.036
1.391	1.985	1.337	0.53	0.294
0.957		0.627	0.474	0.656
				0.297
		1.422	1.205	
0.914	1.009	1.139	1.525	1
0.908	0.765	0.953	1.262	1.157
		4.699		0.45
0.204	1.07	1.204	2.382	1.883
1.049	1.118	1.174	1.545	1.219
0.943	1.238	1.676	0.786	0.309
0.993	2.085	0.994	1.123	1.252
1.018	0.765	0.71	1.966	0.879
1.319	1.574	1.162	1.115	1.2
1.084	1.212	1.368	0.692	0.859
1.048	0.827	0.84	1.72	1.671
0.087	1.503	1.238	0.046	0.054
0.696	0.664	1.103	1.041	0.675
0.729	0.505	1.292	1.683	1.381
0.141	0.259	0.226	0.683	0.659
			0.084	
0.666		0.971	0.736	0.86
0.956	1.051	1.213	1.018	1.297
1.482	0.541	0.639	0.382	1.916
1.109	0.928	1.189	0.967	0.883
1.212	0.88		0.754	1.598
0.905	1.052	1.578	0.99	1.016
0.115	4.482		0.176	
0.919	0.796		0.932	0.565
0.932	1.043	0.369	1.532	1.925
1.43	0.329	0.996	1.644	1.235
1.319	0.325	2.267	2.036	1.027
0.394	0.177	0.223	0.228	
0.713	3.818	3.792	0.837	0.445

0.706	5.394	1.088	0.207	0.313
0.777	1.523	2.481	1.412	0.999
1.02	1.189	0.976	0.31	0.345
0.434	0.567	3.398	1.076	1.167
1.307			0.086	0.887
1.072	0.958	1.242	0.636	0.784
1.071	1.159	0.905	0.983	0.611
0.756	0.786	1.41	0.638	0.894
2.403	0.193	2.996	1.568	1.728
1.536	0.921	0.561	1.147	1.762
1.035	1.548	1.507	1.591	1.377
0.928	0.331	0.381	1.279	0.931
1.107	0.879	0.914	1.034	0.919
1.017	0.782		0.724	1.177
	0.276			
0.703	4.264	0.727	1.186	0.562
0.398	0.16	0.327	0.67	0.547
0.86	1.125	0.912	0.989	1.159
0.65		0.824	1.029	0.648
0.913	0.66	1.422	0.642	0.815
0.798	1.319	0.979	0.447	0.761
0.815				0.801
	0.744	0.843		
1.564	2.186	3.768	1.54	0.94
2.115	0.968	1.56	0.762	1.025
	0.401		0.926	
				0.706
1.077	0.89	2.349	1.302	2.369
0.96	0.768	0.895	1.847	1.677
	4.631	1.463		
1.031	1.358	1.13	0.608	0.86
1.422	1.335	1.872	1.087	1.125
0.985	1.006	1.343	0.982	0.955
2.53	0.159	1.372	1.61	
	1.852		1.559	0.284
1.096	1.439	0.764	1.238	0.959
0.784	0.496	0.742	2.296	1.509
0.619	0.766	0.838	3.054	
0.635	2.208	2.149	0.65	0.62
1.101			1.737	0.883
0.719	0.911	1.707	1.281	0.582
0.881	0.96	0.789	3.534	2.149
0.38	1.282	1.704	2.678	1.226
1.133	1.634	1.375	1.592	1.175
0.953	0.567	0.354	1.845	2.387

1.61	0.509	0.759	0.347	0.096
1.603	0.928	0.363	0.913	1.157
1.179	0.5			0.549
1.003	1.026	1.153	0.613	0.761
			0.831	0.256
0.72	0.11	0.799	0.534	1.259
0.886	0.887	0.649	1.182	1.495
	0.496		1.363	
		1.768		0.401
1.334	0.998	1.088	1.422	2.02
0.804	0.842		1.268	
0.286		0.532	0.73	0.884
0.03	15.217	0.316	0.221	0.055
0.997	1.514	0.857	1.595	1.054
1.169	1.482	0.927	1.475	1.422
2.189	2.258		1.643	0.925
0.79	0.734	1.36	0.919	1.345
0.815	4.153	1.183	0.744	0.477
1.471	3.264	1.033	1.186	0.529
		0.507		
0.49	0.26	0.553	0.405	0.929
1.299	0.802	0.93	0.697	1.192
	0.163	0.216	0.119	
0.721	0.635	3.41	1.175	0.841
0.951	0.818	1.467	1.502	1.593
0.61	2.846	3.085	0.939	1
1.333	0.932	0.998	1.126	1.055
1.685	0.88	0.995	0.524	0.631
1.105	1.154	1.212	0.918	1.123
0.268	0.256	0.36	0.21	0.203
0.872	1.45	1.22	1.766	0.652
			0.563	
0.7	2.608	3.246	1.125	0.907
0.656	1.845	1.233	0.637	0.179
1.123	1.611	1.06	1.273	1.431
0.491	0.189	0.461	0.29	0.408
0.996	0.863	0.489	1.856	1.32
0.909	1.038	0.608	1.104	1.098
0.952	1.063	1.309	1.28	1.512
1.002	1.147	1.58	1.288	1.994
0.793	1.329	0.943	1.554	1.654
0.873	1.15		0.62	0.889
0.54	0.939	0.486	2.195	1.702
	1.587			
1.051	1.308		1.657	0.747

1.505	1.453	1.318	0.522	0.258
0.96	1.094	0.708	0.869	0.849
0.828	0.941	1.469	1.277	0.646
0.996			1.206	0.518
0.953	1.07	1.22	2.31	2.062
0.641	1.414	1.29	2.569	0.291
0.913	1.799		2.157	0.633
0.641	5.618	0.81	0.817	0.676
0.714	0.794		0.824	0.956
0.253	0.647	1.793	1.525	0.405
0.885	1.561	1.117	0.574	0.706
0.893	0.693	0.886	1.661	1.86
0.827	1.48	1.028	1.352	1.489
0.585	1.12	0.519	0.571	0.662
0.704		0.711	2.537	1.213
1.094	1.585			1.119
1.059	0.944	1.758	1.454	0.903
1.289	0.463	0.384	0.408	0.983
0.67	0.585	0.328	0.937	1.127
0.75	1.774	1.396	1.1	1.077
	1.264	1.731		
1.132	0.948	0.569	0.673	1.025
1.129	0.84	0.935	0.663	1.379
0.846	1.131	1.232	1.8	1.655
	2.266	1.141		0.797
1.134	0.989	1.765	1.083	1.256
1.068	1.113	1.57	1.17	1.081
0.926	1.411	2.043	0.954	1.133
1.296	0.817	1.225	1.55	1.307
			0.616	
1.227	0.823	0.718	0.836	0.783
0.724	1.959	1.375		0.885
0.613	1.589	1.728	0.332	0.584
1.034				1.271
0.711	1.566	1.037	0.713	0.698
0.99	1.11	0.587	0.816	1.168
	0.413	0.393	0.695	0.718
0.492	1.451	1.266	1.055	
0.821	7.369		0.413	0.433
1.358	0.88	1.065	1.638	1.338
0.849	1.411	0.786	1.289	0.975
1.215	1.173	0.739	0.752	0.825
1.335	0.47	0.542	2.534	1.275
0.802	1.246		1.27	1.121
0.942	0.895	0.902	1.314	0.673
0.957	0.86	1.476	1.047	0.832
0.979	1.47	0.845	1.29	1.489
0.869	1.538	1.436	0.674	1.281
0.622	3.553	1.546	0.832	0.802

			1.53	
1.317	0.966	1.335	0.946	1.369
1.366	0.549	0.816	2.133	1.488
0.972	4.268			0.491
1.542	0.715	1.223	0.716	0.64
1.013	2.342	2.154	0.878	0.734
0.61	0.633	2.229	1.345	1.702
0.931	1.806	0.122	1.704	0.705
1.197	0.961	0.391	0.827	2.082
1.191				0.767
0.817	0.632	0.745	0.869	0.689
1.289	1.02	0.995	0.879	0.85
0.895	0.625			
1.185	1.132	0.362	0.571	1.043
	2.976		1.528	0.911
1.343	0.865	0.186	0.466	1.28
0.965	0.687	1.504	1.703	1.554
1.033	1.166	1.309	0.509	1.041
0.89			0.998	0.51
		2.941		
	1.608	0.213	1.536	
3.135	0.658	0.863	1.681	1.497
1.229	2.659		0.655	1.077
1.133	1.118	1.317	0.68	0.989
1.236	1.162	0.185	0.808	1.335
1.014		0.864		0.865
	1.181	1.723	1.116	1.32
1.337	1.211		0.796	1.43
0.673	0.744	1.181	0.517	0.964
1.152	1.252	0.592	1.369	1.141
0.326	0.147	0.44	0.313	7.486
0.916	0.907	1.307	1.022	1.158
1.272	1.069	0.156	0.28	1.338
1.08	1.048	0.684	1.176	0.786
1.481	1.249	1.335	1.674	1.537
0.487	0.63	3.217	0.898	0.502
0.77	1.634	1.039	1.11	1.241
0.696	0.918	5.153	1.256	0.049
1.102	0.774	0.976	1.219	1.198
1.18			2.025	1.337
0.861	1.026	1.462	1.027	1.03
0.572	1.081		0.855	1.359
0.944	0.795	0.35	0.485	0.681
0.423	2.738	6.788	1.075	0.861

0.954		1.03		0.921
1.292	1.105		0.912	1.544
1.106	0.891	1.238	0.918	1.172
1.255	1.221	1.601	0.966	0.651
1.025	1.022	1.223	1.411	1.525
1.051				0.762
1.278	1.53	1.465	1.357	1.496
1.065	0.968	1.08	0.904	1.397
1.116	0.818	0.88	0.397	0.717
0.848	2.137	1.292	0.286	
1.005	0.902	0.771	1.067	1.522
1.066	1.244	0.561	1.054	1.399
0.453	1.854		1.74	1.448
1.003	1.105	1.12	0.77	1.212
1.151	1.497	1.296	1.746	1.196
0.878	2.048	2.707	0.537	1.165
1.061	2.853	0.681	0.494	0.48
	0.806			
0.418		0.237	3.369	2.545
0.844	1.154	1.313	0.972	0.749
1.226	1.503	0.951	1.138	1.257
1.42	1.327	0.227	0.675	1.09
0.584	1.139	0.206	0.515	1.642
0.732	2.211	1.583	1.677	0.395
1.176	0.705	0.405	0.822	0.959
1.817	1.045	0.252	1.233	1.176
1.213	0.965	0.333	0.876	1.402
0.591	1.924	0.254	0.07	0.69
0.681	0.956		0.685	0.912
1.567	0.597	0.019	0.472	1.141
1.215	0.906	0.598	0.664	1.035
	5.465	0.646	0.806	0.983
1.643	4.189	0.664	0.774	0.716
1.218	1.017	1.071	1.807	1.685
1.261	1.56	1.243	1.569	0.925
	2.601		0.571	1.009
1.356	0.927	0.356	0.854	1.116
0.858	0.753	0.968	0.97	0.825
	1.412	1.934		2.68
0.98				
1.391	1.546	0.597	0.688	0.448
0.97	0.805	0.615	1.007	0.626
0.91	1.597	1.2	0.869	0.735
1.215	0.829	1.023	1.172	2.064
	1.275	1.201		
0.984	0.928	1.041	0.392	0.699
1.806	0.646	0.58	1.713	0.742

0.373			0.358	0.281
0.714	0.871	0.692	1.21	1.34
0.997	1.577	1.612	1.143	1.097
1.564	0.866	0.331	0.556	1.47
0.694	1.009	1.705	1.337	1.425
1.186	0.896	1.004	0.843	1.109
0.991	0.791	0.674	0.45	
		0.036		
1.385				0.718
1.139	0.794	0.653	0.988	1.117
0.605	1.029	0.896	0.689	0.809
1.488	0.996		0.807	0.996
1.095	1.059	0.787	0.831	0.905
0.858	0.925	1.176	1.355	1.053
1.063	0.732	0.399		0.357
		0.481	0.896	1.107
0.713	1.105	1.855	1.479	0.87
1.095	1.021	1.456	1.047	1.192
0.568	1.717	0.58	0.637	0.367
0.695	1.04	1.18	0.476	0.532
0.005	1.136	1.313	1.978	2.293
1.158	0.815	1.97	0.965	
0.959	0.845	1.306	1.272	1.193
1.193	0.882			1.504
0.876	0.745	0.725	0.463	0.579
1.143	1.04	0.775	0.654	0.823
1.579	0.924	0.397	0.854	1.202
0.927	1.664		0.998	1.136
0.686	1.181	1.123	2.434	0.823
0.996	0.928	1.593	0.919	1.015
0.703	0.246	0.261	0.413	1.727
0.447	1.045	0.417	0.344	1.497
1.333	1.075	0.527	0.737	1.683
1.24	1.155	1.326	1.801	1.369
0.747	3.122	2.904	1.137	0.513
1.031	0.852	0.849	0.943	1.216
0.672	2.437	1.602	0.782	0.761
1.366	0.529	0.58	1.092	1.183
0.975	1.396	1.234	1.01	0.79
0.602	2.061	7.428		0.77
1.135	0.882	0.744	0.978	0.945
1.323	0.698	1.13	0.138	0.715
1.002	1.506	1.175	1.403	0.933
1.083		0.844	1.62	1.944
0.9	0.994	0.846	1.361	1.013
1.152	0.64	1.183	0.765	0.947

0.849		0.801	0.921	1.214
1.183	0.765	0.783	0.59	1.074
0.824	2.406	0.621	0.27	1.25
1.122	1.095	0.791	1.203	1.134
0.99	0.144	0.09	0.059	0.014
0.736	0.804	0.58	0.862	1.131
1.004	0.834	1.027	0.621	1.487
1.191	1.115	1.084	1.184	0.923
0.983	1.352	1.313	1.201	1.915
1.713	0.877	0.299	0.759	1.251
0.812	2.012	1.033	2.261	1.099
0.748	0.919	0.849	0.696	1.37
1.008	0.838	0.911	0.613	0.93
				0.441
0.812	0.432	1.364	1.139	0.417
1.777	0.762	0.962	2.419	2.104
1.177	0.984	0.702	1.021	1.377
0.578	0.827	0.814	0.569	1.361
0.811	0.623	1.007	0.671	0.847
1.488	0.669	0.399	0.634	0.495
1.495	0.861	1.574	1.94	1.296
0.522	0.806	0.949	1.946	1.665
0.992	1.255	1.074	1.017	0.84
0.559	2.827		1.504	0.709
0.834	0.533	0.897	2.244	1.371
0.977	1.164	1.229	1.088	0.996
0.796	0.94	0.973	1.668	1.187
0.247			0.717	0.349
0.936	0.817	0.897	1.134	1.208
1.454			0.742	0.624
1.337	1.651	1.711		
2.236		1.738	1.769	1.021
0.921	1.404	0.426	1.175	1.03
0.656	1.517	0.806	1.502	0.728
1.337	0.964	0.878	2.206	1.379
0.726	0.541	1.105	1.295	2.034
0.938	0.9	1.097	0.819	1.329
0.351	1.029		0.574	0.406
0.881	2.041	0.725	2.028	1.739
0.953	2.246	0.746	1.494	1.531
0.972	0.838	1.053	0.558	1.117
0.963	0.917	1.392	1.169	0.847
0.279	1.747	1.106	0.891	
0.583	3.068	2.011	0.816	0.807
1.084	0.827	1.095	0.746	1.189
0.961	1.57	1.264	0.888	0.973

1.028	0.442	0.883	1.483	0.886
0.626	0.996	1.187	0.997	0.883
1.498	0.865	0.847	0.421	1.744
0.82	5.01		0.794	0.536
1.016	0.903	0.477	0.939	1.263
1.032	0.73	1.054	1.158	1.079
0.677	2.18	1.098	0.46	0.859
1.072	0.533		1.122	1.189
0.806	0.67	0.928	2.811	1.475
1.705			0.765	1.309
1.061	1.389	0.913	0.488	0.579
1.441	0.275			0.325
0.732	0.982	1.592	0.883	1.268
0.742			2.99	1.656
1.374	0.899	0.983	1.105	1.243
1.15	1.349	1.45	0.944	0.962
1.089	1.032			2.352
1.192	0.926	1.135	1.862	2.052
1.16	0.955	0.691	1.219	1.29
1.184	0.751	1.381	0.937	
0.423	0.692		0.779	
0.991	1.24	1.091	1.181	0.919
1.176	1.054	1.869	1.17	0.735
0.992	1.854	0.694	1.487	0.533
0.457	2.743	4.568	1.341	0.499
1.128	0.91	1.482	0.884	1.062
0.737	1.385	0.91	1.053	0.972
0.981	0.92	1.521	1.32	1.348
1	0.992	1.123	1.647	2.055
1.064	0.471	1.085	0.299	0.395
1.148	0.935	0.646	1.885	2.423
1.035	0.488	0.715		1.182
0.761	2.177	1.234	1.291	1.041
1.353	0.657	1.163	1.282	1.427
1.085	2.654	1.448	1.374	0.516
	1.498	2.265	0.248	0.662
0.607	0.785	1.221	2.172	2.114
0.773	0.783	1.786	0.658	1.08
0.703	0.887	0.598	1.143	1.257
0.614	0.764		0.674	0.435
1.438	3.046	1.779	1.11	0.83
1.532	0.687	1.829	2.257	1.942
1.052		0.905		0.897
1.02	1.383	1.678	1.394	1.418
0.885	1.737			0.296
1.161	1.241	1.242	0.821	0.66

0.995	0.662		0.99	1.09
	0.982	1.129		
	2.287			
1.051	1.223	1.556	1.217	1.257
	1.242		2.327	1.073
1.116	2.052	0.104		0.976
1.354	0.631	0.992	0.943	1.117
1.537	1.321	1.544	0.203	0.228
1.195	0.261	1.1	1.918	1.497
0.536	0.605	0.334	0.614	1.664
	2.065		0.22	
1.11	0.816	1.353	0.734	0.967
1.136	2.263	1.483	0.637	0.694
1.209	0.844	0.562	0.831	0.968
1.025	2.327	0.464	0.417	0.661
1.126	1.012	0.581	1.013	1.239
0.686	0.422	0.462	0.181	0.753

M_TXD_2	M_ZGH_3	M_ZJ_4	M_LFH_5	M_LCL_6
		0.854		
0.725	0.576	1.217	1.366	0.805
1.935	0.867	1.369	1.013	1.63
0.947				
0.208	3.462	0.649	1.558	0.456
1.012	1.497	0.88	1.489	0.946
0.769		1.463	1.817	1.494
0.876	1.207	1.022	0.848	1.171
0.809	0.796	0.454	0.543	1.276
1.141	0.933	1.801	1.233	1.224
1.296	0.928	0.734	1.029	1.241
0.481	1.42	2.293	1.377	0.487
1.313	0.805	1.467	1.448	1.569
0.44	1.084	1.444	1.637	0.934
0.174	0.844	0.242	0.384	0.698
			2.287	
1.01				
1.202	1.306	0.757	0.557	0.815
0.564	1.149	0.929	1.028	1.438
0.069	0.343	0.707	0.531	4.532
0.473	0.389	1.002	1.698	2.139
1.002	1.107	0.608	1.386	1.182
1.025	0.65	0.931	0.992	1.234
	0.997	2.781	0.556	0.656
1.302	0.784	0.666	1.266	0.789
		0.007		
1.16	1.319	1.157	0.834	0.99
3.205	0.39	2.308		
1.409	1.874	1.208	1.035	1.315
0.938	1.745	0.669	1.298	1.361
1.547				
0.361	1.301	1.75	1.228	0.636
0.563				
1.497	0.855	1.511	1.462	0.781
0.637	2.248	0.626	0.741	1.107
2.535	2.188	0.961	1.517	0.132
2.33	0.909	1.044	1.505	1.344
				0.035
8.041	0.834	0.786	1.371	0.455
1.317	1.257	1.886	1.708	1.381
0.688	1.528	1.007	1.239	1.039
1.894	1.302		1.455	1.021
0.899	1.855	1.172		0.507
		0.962		

0.089				
				0.778
1.408	0.814	1.571	0.782	0.646
1.168	1.397	1.008	0.82	0.898
		0.952		0.832
1.183			0.71	0.71
2.854				
0.145	0.731	1.295	0.817	0.247
1.515	1.172	1.493	1.701	1.414
1.357	1.14	0.736	1.204	1.76
0.594	0.792	1.905	1.196	0.707
0.943	1.013	0.88	1.252	1.155
1.408	1.293	0.722	1.275	1.236
1.12	1.482	0.541	1.029	0.635
1.011	2.217		0.002	1.053
1.901	0.661	0.669	1.243	1.766
0.867	0.765	1.225	0.839	0.06
0.645	1.081	1.24	0.614	0.953
0.84	1.35	1.285	0.644	0.946
1.546	1.264	0.872	1.612	1.647
0.78	0.943	1.048	1.157	2.059
0.006	1.994	0.004		
0.788	2.466	0.856		1.269
0.32	0.3	0.165	0.277	1.608
0.914	1.068	1.521	1.041	1.083
0.598	0.452	1.859	0.568	1.704
1.011	1.312	0.582	0.967	0.556
1.595	1.194	1.154	1.229	1.856
1.142	0.971	0.694	0.863	2.09
1.341	0.231	0.286	0.687	1.442
8.301	1.468	1.653	0.405	
0.249	0.501	0.175	5.29	0.547
0.329	1.448	1.28	1.756	1.064
0.037	1.429	1.558	0.386	0.378
0.046	0.111	1.018	4.711	0.106
0.83	0.798	0.505	0.986	0.966
7.393	0.663	0.763	1.104	0.254
		0.533		
0.056	1.688	1.773	0.672	1.947
0.586	1.042	0.462	1.329	1.504
		0.91	1.325	1.297
0.164	0.943	1.199	0.564	1.186
1.102	0.713	0.793	1.214	1.382
0.854	0.992			

0.502		1.566	0.701	
0.899				
0.424	0.326	1.587	1.017	0.788
0.584		2.177		
1.484	1.617	0.802	1.191	1.246
1.204	1.229	0.829	0.927	1.311
1.091	2.223	1.047	0.76	0.951
2.227	0.783	0.8	1.349	1.298
4.857	0.438	0.608	1.122	1.487
1.206	0.989		1.002	1.025
0.899				
2.013		0.62	0.476	0.794
0.821	1.679	0.359	1.1	0.591
1.214	1.302	0.947	0.899	1.187
0.887	1.169	0.773	0.9	
0.65	1.339	0.924	1.31	0.935
1.475	0.359		1.234	0.564
0.963	1.069	1.252	0.886	1.365
		3.029		0.858
0.847	1.172	0.975	1.218	1.382
1.19	0.863	1.646	0.497	
1.377		0.782		0.975
0.752	0.931	0.656	0.946	0.752
1.008	0.903	1.172	1.643	0.854
0.268	1.387	1.912	1.646	0.914
1.147	0.917	0.69	1.408	1.05
0.081	0.78	1.486	1.254	0.804
0.925	1.019	1.071	1.411	0.721
		0.833	0.692	
	1.502			0.551
0.899				
			0.182	1.041
		0.686		0.886
1.101	0.873	1.403	0.678	1.196
0.687	0.586	0.71	0.727	2.025
1.162	1.047	0.819	1.41	1.152
2.789	0.552	2.605	1.911	0.685
0.807	1.142	0.954	1.067	0.807
1.751	0.733	0.875	0.96	1.122
0.808	0.37	2.727		0.809
1.123	1.684	0.873	1.989	2.188
0.823	1.261	2.141	1.246	0.442
				0.818
1.217	1.141	0.936	1.074	1.133
2.296	0.865	1.171	1.045	1.356
0.947	0.588	1.08	1.196	1.123
1.279	0.617	0.26	0.703	1.028
0.819	0.709	1.88	0.818	0.598
0.566	0.234	0.58	1.341	1.365

	0.294	3.121		
1.041	1.406	1.606	0.982	0.743
1.212	1.265	0.43	1.268	1.41
1.522	2.351	0.709	1.257	1.734
	1.19	1.061		
1.663	1.268	0.769	0.752	0.57
0.77	0.693	0.626	0.7	0.668
0.721	0.719	1.258	0.859	1.162
0.679				
1.017	1.005	0.866	0.96	1.515
		1.775		
1.343	1.482	0.762	1.279	1.41
0.515		0.404		
1.11	0.863	1.139	0.945	1.249
1.46	1.455	1.331	0.707	1.011
1.334	0.55	1.638	0.588	0.816
4.911	0.477	2.062	0.473	0.718
1.652	0.515	1.142	0.468	0.706
0.59	1.852	1.359	0.7	0.473
0.242				
0.55	0.8	1.03	1.18	2.246
1.3	1.436	1.125	1.19	1.656
1.296	0.837	0.693	1.042	0.996
1.19	1.362	0.58	0.874	0.805
0.809	1.378	2.089	0.631	0.794
1.203	1.311	0.927	1.34	1.138
1.226	0.827	1.371	0.947	0.958
	0.578			0.484
0.98	0.894	1.03	1.122	1.372
0.824	0.964	0.612	1.209	
0.821	0.881	0.978	1.239	1.133
2.193	1.072	1.081	0.722	0.419
1.018	0.819	1.278	0.834	1.261
1.122	1.207	0.491	1.075	0.617
0.346	1.375	2.191	1.332	1.252
3.64	0.818	1.818	1.621	0.42
1.173	0.841	0.551	1.006	1.232
0.33	0.831	2.405	0.96	0.934
1.108	1.092	1.216	0.837	1.244
1.446	1.083	1.017	0.984	1.144
1.566	0.622	1.064	1.359	1.165
1.139	1.34			
1.867	1.054	1.317		0.769
0.741	1.239	1.496	1.104	0.771
0.716	1.277	1.433	1.415	1.42
0.768	0.894	1.749	0.912	1.255
0.971	1.036	0.639	0.803	0.993
0.941	0.784	0.755	1.175	1.268
2.006	1.151	0.945	0.872	1.079
0.937	1.636	1.343	0.777	1.143

0.971	0.855	1.926	0.598	0.489
1.519	0.514	2.74	0.233	0.32
1.103	1.036	0.951	1.292	1.412
1.132	0.006	2.319	0.346	0.01
0.595	0.307	3.308	1.185	0.919
0.918	0.476	1.674	0.964	0.679
1.087	0.736	1.578	0.491	0.917
0.205	1.729	1.236	0.205	0.312
	0.901	0.67	0.338	
0.791	1.44	0.935	0.819	0.766
0.819	0.822	1.078	1.005	0.935
	0.771	1.298		
1.922	0.609	1.062	0.911	1.601
	1.015			
0.092	1.463	2.141	1.461	0.642
0.853	0.892	1.308	0.595	1.148
1.864	0.67	0.095	0.815	1.236
3.052	0.965	1.936	1.718	1.872
0.477		0.979		
1.797	0.994	1.199	0.833	0.837
0.978	0.733	0.656	1.414	1.153
1.144	1.02	0.894	1.25	1.185
1.01	0.761	1.338	1.13	0.991
1.285	0.65	0.508	1.683	1.315
0.907	1.692	0.485	1.074	0.572
2.161	1.056	1.159	0.62	0.914
0.894	0.74	1.135	0.766	0.648
0.758	1.105	0.836	1.113	1.225
	0.264	0.613	0.437	
1.301	0.523	0.934	1.316	1.611
	0.126	0.062		0.105
0.878	1.236	1.011	0.839	0.869
		2.528	1.71	0.696
		1.092	1.352	1.17
0.905	0.797	2.419	0.75	0.802
1.195	1.566	0.316	0.669	1.063
1.183	1.714	0.676	0.909	1.266
0.683	1.365	1.245	0.976	0.83
1.104		2.12	0.866	0.403
1.211	0.655	0.819	1.352	1.14
1.088	0.602	1.486	0.818	1.069
0.836	1.039	1.431	2.075	1.162
0.641	1.001	0.983	1.554	1.297
0.707	1.56	1.49	0.608	1.358
0.627	0.234	1.135	0.089	0.1
0.614	0.358	1.004		0.647
0.855	1.321	0.995	1.065	0.935
6.95	0.465			

1.135	1.405	0.76	0.862	0.973
1.662	1.042	0.836	0.855	1.75
1.168	1.214	0.911	1.797	1.848
0.817	1.04	0.949	1.752	0.98
1.285		1.407		0.518
0.636	1.849	1.908	1.444	0.194
1.076	1.01	1.314	0.584	1.408
0.653	0.857	1.122	1.351	2.268
1.507	0.872	0.629	0.919	0.637
1.045	0.837	0.477	0.418	0.809
0.135	1.894	0.698	0.433	0.869
	1.026	1.016	1.393	0.205
		0.564	0.898	
0.675	0.629	0.8	1.798	1.554
0.015	0.061	1.282	2.633	1.794
0.805	1.373	0.883	0.67	0.709
0.581	0.972	0.745		
2.536	2.262	4.955	0.802	1.073
1.516	1.539	0.951	1.262	1.636
1.156	1.082	0.997	0.737	1.057
0.56	0.316	1.309	0.524	0.622
2.872			1.438	
1.18	2.774	1.518	0.299	0.41
0.835	0.661	1.938	0.649	1.186
0.712	1.126	1.247	0.991	0.925
0.723	0.626	0.364		
				0.361
0.491	1.619	1.267	1.553	1.221
		1.539		0.919
0.945	0.917	0.54	0.756	0.733
1.802	1.156	0.862	1.192	1.375
0.945	1.177	1.295	0.735	0.874
1.832	2.26	1.211	0.632	0.559
1.111	0.052		0.059	
3.365	0.328	0.09	0.091	0.98
0.702	1.074		1.223	1.929
	1.367			
0.613	1.239	1.203	1.223	1.057
1.799	1.282	0.894	0.899	1.436
1.101		0.996		
0.899	0.939	0.971	1.662	1.306
0.716	1.071	0.748	1.31	1.31
0.795	1.28	1.045	0.786	0.974
0.893	2.207	1.194	0.865	1.316
1.571	1.595	2.058	0.31	0.275
1.143	1.056	1.679	0.856	0.842
0.407	1.132	1.398	0.953	1.211
1.832	1.393	1.378	0.945	0.383
2.057	0.93	0.907	0.913	1.187

1.666	0.115	1.214	0.148	1.197
0.871	1.254	0.985	0.863	1.182
1.67	2.21	1.276	0.815	0.658
0.577	1.599	1.352	1.131	0.745
1.069	1.202	0.492	1.181	0.436
1.245	0.674	1.522	0.791	1.17
0.174	0.2	4.174	4.95	
1.092	1.239			
0.866	0.937	0.901	0.796	0.901
0.923	1.231		0.813	0.807
0.917	0.89	0.954	1.226	1.482
1.283	1.072	1.228	0.791	0.821
0.974	1.245	1.729	0.957	1.028
2.778	1.132	0.136		0.52
0.563	1.952	1.916	1.518	
1.446	0.956	0.806	0.772	1.129
1.408	0.787	0.569	1.136	1.263
1.215	0.944	1.009	1.168	1.27
1.086	0.36	1.736	0.516	0.762
8.062	0.903	1.441	0.355	0.602
		3.106		
1.093	1.183	1.292	0.346	1.021
1.947	1.192	1.171	0.845	0.875
0.835	1.847	1.116	0.862	0.895
0.976	0.994	1.45	0.753	0.948
0.928	0.761	0.301	0.309	0.596
0.882	1.272	1.153	0.872	1
1.784				0.581
0.95	1.338	1.354	1.092	0.754
0.455	0.654	0.783	1.478	1.388
0.912	0.811	1.474	0.81	0.891
2.318	0.857	2.086	0.648	0.806
1.641	0.781	1.389	1.74	
0.584	2.367	2	1.228	1.002
0.136	3.331	0.891	1.373	0.175
0.557	0.844	0.638	0.809	0.598
1.844	1.757	0.818		0.639
0.112	0.067		1.503	1.932
0.812	1.055	0.904	0.568	1.322
0.585	2.096	0.111	0.467	0.627
1.607	0.662	0.481	0.889	1.257
0.948	1.405	0.943	1.195	0.547
0.46	1.057	1.502	1.074	0.34
1.632	2.504	1.149	0.435	0.679
	0.485	0.622		0.795
1.404	0.904	0.893	1.091	1.289

0.772	0.341	1.11	0.895	0.491
		2.028	0.602	
0.361		1.978	1.886	0.454
0.88	0.938	1.067	0.614	1.391
0.915	0.701		0.988	1.429
1.005	1.194	0.305	0.594	0.588
		0.634		
0.535	1.412	1.441		1.229
1.244	0.102	0.865	0.379	1.766
0.389	0.295	3.246	0.147	0.358
1.015	0.42	0.93	1.201	1.095
1.701	0.91	0.565	1.17	0.839
2.839	1.008	2.034	0.465	0.852
1.931	1.946	0.78		0.944
0.825	1.137	0.653	1.15	0.785
0.464	0.813	1.004	1.184	1.34
0.972	0.856	1.323	1.125	1.268
2.562	1.3	0.382	1.548	1.628
0.671	0.321	0.592	1.239	1.928
1.153	1.083	0.966	0.68	1.6
1.757		0.579	0.377	0.461
0.602	0.713	0.73	0.971	1.912
	0.72		1.324	0.82
1.817	0.835	0.568	0.875	1.692
0.714	0.77	1.037	1.666	1.243
1.052		0.122		
0.273	0.679	0.321	0.827	1.073
0.606				0.347
0.748	0.678	0.789	0.255	0.964
1.295	0.889	1.188	1.474	0.546
0.289	1.144	3.868	0.771	0.511
		3.668		
0.154	0.909	2.782	1.075	0.843
1.721	1.169	1.039	0.505	0.878
1.058	1.066	0.62	0.661	0.603
1.528	1.46	1.2	0.767	1.015
1.446	0.439	0.414	0.989	1.283
0.486	0.933	0.948	1.713	1.008
0.892	0.606	2.293	0.763	
1.447	0.642	0.685	0.835	
1.08	0.929	0.574	1.045	0.838
		3.274		
2.142	8.537	1.116		
14.125	1.672	4.509	0.261	0.223
0.944	0.984	1.527	0.999	0.81
0.764	1.212	0.769	1.054	1.257
0.85	1.447	0.795	1.035	1.22
0.7	0.603	0.613	0.653	0.464
0.655	1.509	0.862	1.743	1.207

1.015	1.196	1.241	1.332	1.261
2.582	1.043	0.885	1.695	1.005
0.465	1.5			
1.32	1.062	0.726	0.168	0.692
0.773	0.565	2.037	1.009	1.018
1.068	0.604	0.509		
0.852	0.768	2.114	0.888	1.027
		0.998		
5.315	0.999	0.824	1.11	0.774
1.217	0.578	0.543	0.89	1.335
0.997	1.096	0.591	1.581	1.074
1.163	0.844	1.11	0.91	0.939
1.284	1.115	0.853	0.966	1.334
	0.685			1.167
0.898	1.163	1.207	1.025	0.464
0.756	1.092	0.826	1.033	1.394
1.386	0.849	0.627	0.859	1.015
2.107				
0.66	0.884	0.929	1.171	1.016
0.439	0.74	1.081	0.951	0.674
0.305	0.156	0.089	0.574	0.293
8.078	0.387	1.34	2.159	0.407
1.055	1.467	1.574	0.743	0.752
1.089	1.308	1.174	0.694	1.13
1.464	1.205	1.213	0.83	0.911
2.104	1.057	0.664	0.74	1.359
1.37	1.418	1.036	1.17	1.413
0.208	2.024	1.762	0.916	0.848
2.211	0.851	0.88	1.83	1.7
1.263		1.38	1.001	0.834
1.254				
1.402	1.332	1.582	0.905	1.564
0.598	0.31	2.426	1.146	1.034
0.432		0.537	0.437	0.842
0.499	0.681	1.366	0.409	1.12
1.439	1.24	1.937	1.07	0.577
	1.088	1.246	1.054	1.065
		1.732	1.202	
1.031	0.896	0.982	1.017	1.399
1.122	1.678	1.301	0.807	0.507
0.767	1.001	0.54	1.039	1.212
1.011	0.82	1.237	0.959	0.823
1.838				
0.448	0.915	2.049	0.707	0.933
0.458	1.532	0.273	0.338	1.289
	1.5	0.936	0.514	0.877
		1.609		1.336

0.753	1.468	1.561	1.228	0.897
0.987	0.967	1.192	0.692	1.384
0.261	0.268	2.059	0.406	0.8
1.503	1.384	1.206	1.478	1.196
0.876	1.336		2.372	0.708
0.757	1.323	1.047	1.749	0.706
0.772	1.189	1.555	1.243	0.854
0.812	0.932		1.329	0.444
0.706				
0.424		1.167	0.712	
4.334	1.109	1.423		
		0.925		0.746
0.418	1.439	2.034	0.795	0.756
1.044		1.705		0.859
	2.165	1.781	2.663	0.949
0.83	1.263	1.512	1.089	0.961
1.393	0.039	1.137	0.726	0.857
1.122	1.192	0.957	1.148	1.243
1.331	1.447	0.469	0.887	0.824
1.888	0.839	0.743	1.567	0.892
1.17	1.802	0.791	1.234	1.799
0.847	0.793	0.946	0.974	1.401
0.514				1.473
	0.121	0.238	0.148	
0.957	1.149	1.111	0.904	0.992
1.198	1.027	1.055	0.896	1.112
1.105	0.962	0.939	1.788	
0.965	1.608	1.553	0.488	1.027
0.026	1.769	1.037	1.253	1.035
0.771	0.867	1.101	1.145	0.971
0.796	0.634	1.287	1.049	0.582
0.802	1.061	0.739	0.934	1.272
1.015	0.907	1.121	0.867	1.87
0.996	0.788	0.542	0.848	1.13
1.135	0.657	1.486	0.903	1.12
	0.216	1.007	0.103	0.221
0.893	1.228	0.565	1.345	1.272
0.707	0.68	0.566	0.858	1.483
0.495	1.771	1.334	0.752	0.775
0.943	0.338	1.698	1.182	0.685
1.638	0.577	0.899	0.708	0.719
0.989	0.541	0.26	1.2	2.898
0.759	0.985	0.896	1.581	1.067
1.521	0.896	0.849	1.478	1.137
1.007				0.773
1.107	0.834	1.624	0.953	1.046
0.825	1.077	0.963	0.783	0.847

0.72	0.845	0.745	1.147	0.762
0.402	1.709	2.248	0.464	1.256
1.187	1.292	0.614	0.875	0.83
1.22	0.877	1.75	1.28	0.892
3.814		1.189		
1.205	0.591	1.098	1.318	1.449
0.741				2.048
		1.058		
4.631	0.65	5.122		
0.545	0.403	0.711		0.369
1.284	0.967	1.84	0.484	1.118
3.195	0.939	0.856	1.055	0.305
1.257	1.51	0.891	1.046	1.262
0.823	0.837	0.511	0.719	1.013
0.786		2.089	0.522	0.593
0.794	1.281	0.668	1.062	1.724
1.251	0.928	0.697	1.268	1.261
0.672	0.906	1.537		1.141
1.07	1.357	1.201	0.86	0.674
0.872	1.053	1.309	0.783	0.856
1.113	0.881	1.401	1.253	1.21
0.576	0.87	2.442	1.513	0.69
0.333	0.482	1.611	0.777	1.439
0.968	0.314	1.013	0.872	1.039
0.896	1.341	0.416	1.529	0.857
		0.053		
1.039	0.857	1.118	1.039	0.976
0.756	1.227	1.649	0.487	1.057
2.364	1.676	0.921	0.416	1.179
0.973	1.223	1.104	0.275	1.13
1.333				
2.645	1.698	1.035	0.816	0.696
1.338	1.072	0.91	1.615	1.363
1.809	1.696	0.983	1.145	0.74
1.236	1.705	1.729	1.608	1.385
0.79	1.022	1.131	1.01	1.045
0.585	1.058	0.273		1.05
1.32		0.117	1.707	1.56
0.394	0.644	1.204	1.69	1.434
1.728	0.862	0.289	0.723	1.592
2.148	1.323	0.522	0.875	1.914
0.555	0.451	0.863	0.967	1.19
0.944	1.385	0.634	0.687	1.045
1.072	1.148	1.254	1.075	1.247
1.623	0.955	0.357	1.068	1.409
2.79	1.041	0.696	0.542	0.746
1.305	2.042	1.735	1.592	1.301

	0.113	2.565		
1.002	0.716	1.621	1.099	1.071
2.18			0.579	1.196
			0.635	
1.312	1.202	1.112	0.764	0.817
0.466	1.523	0.9	1.019	1.019
0.941	1.509	1.087	1.015	1.295
1.447				1.874
0.828	1.418	1.613	0.929	0.683
1.02	0.272	2.03	0.804	0.354
0.736	0.614	1.542	0.843	1.203
2.507	0.691	0.755	1.349	0.82
1.827	0.764		1.868	0.731
1.198	0.602	1.871	1.093	0.978
0.359	1.299			0.387
0.97	0.833	0.73	0.917	1.418
		1.38		
		0.202	0.299	
0.759	1.52	1.461	1.146	0.976
1.027		2.278		0.079
4.61	0.461		0.702	
2.628	2.017	1.805	0.002	0.001
1.349	1.063	1.591	0.665	1.109
0.465				1.062
0.607	1.351	1.071	0.926	1.362
		0.99		
0.856	0.735	1.222	1.284	1.898
0.985	1.262	1.079	1.756	1.107
1.023	0.47	1.532	1.218	0.933
1.115	1.137	0.752	0.688	0.959
2.093	0.757	1.03	1.172	1.315
0.179	0.2	2.284	0.674	1.278
		0.822		
1.046	0.863	0.631	0.604	0.761
0.786	1.589	1.86	0.536	0.824
1.333	1.74	1.224	1.227	1.496
1.368	1.036	0.783	1.364	1.235
0.503	0.313	0.185	0.341	0.302
0.938	0.753	0.865	0.516	
1.262	1.397	0.94	0.714	0.933
1.153	0.957	1.134		0.434
1.523	0.784	0.53	0.916	0.976
0.737	1.313	1.073	1.131	1.053
	0.333	1.775	0.408	0.461
2.608				
0.987	1.17	0.883	1.104	0.887
1.427	0.975	0.957	1.092	1.516
0.67	1.22	0.096	0.135	0.391
0.527	1.51	0.93	1.044	1.191

1.035	1.247	1.408	1.136	1.181
1.983	1.487	0.982	1.451	1.237
1.758	1.311	0.783	0.949	1.098
1.974	1.588	0.829	1.193	1.096
0.837	1.255	0.974	1.132	0.886
0.794	1.638	0.709	0.763	0.77
1.176	1.373	0.544	1.258	0.709
	0.91		0.764	
		1.151		
1.347	0.682	1.122	1.31	1.113
0.942	0.604	0.516	1.275	1.241
0.699	1.085		0.784	0.595
	3.62	0.374		3.582
0.863	0.399		5.479	0.932
1.423	0.86	1.024	0.818	0.945
0.761	0.437	0.254	0.987	0.508
0.204				
1.024	1.065	1.568	0.974	0.843
1.339	1.269	0.958	0.064	1.554
0.979	1.082	1.196	0.941	1.054
		1.007		
0.405	1.295	0.739	1.344	1.244
0.464	0.786	0.96	1.792	0.045
0.877	1.843	1.095	0.512	0.527
1.221	1.646	0.749	1.376	1.475
1.124	0.871	1.365	1.21	1.3
1.004	0.934	0.577	0.237	1.387
1.304	1.295	0.857	1.263	1.415
1.126	1.059	0.579	1.095	1.327
1.274	0.949	0.731	1.07	1.287
2.039				
	0.57	2.503	1.977	0.932
0.457		2.928	1.196	0.895
0.52	1.148	3.066	0.379	0.31
0.792	0.741	0.117	1.415	
0.714		0.125		
	1.232	0.755	1.036	
0.623	1.634	1.355	1.143	0.847
1.161	0.874	0.609	0.718	1.583
0.877	1.215	1.202	1.115	1.3
0.793	0.443	1.604	0.827	0.987
2.86	0.077	0.737	0.384	0.092
	0.906	1.692	1.119	0.806
2.572	0.11	0.088	1.206	0.204
1.005	0.924	1.251	0.881	1.335
0.761	1.493	0.837	1.053	1.142
1.419	1.558	0.363	0.671	1.159

1.11	1.341	1.799	0.27	0.947
	1.351	0.637	0.657	1.422
1.514	1.618	1.239	0.795	1.159
	1.209			
0.921	0.995	1.057	1.017	1.098
1.086	1.099	1.212	0.994	0.873
1.351	0.738	0.407	0.756	1.111
0.779	0.723	0.701	0.941	1.274
0.307	1.247	0.943	1.66	1.243
0.378		1.607		
0.025	0.038			
1.183				
2.081	0.487	0.828	1.587	0.381
	1.233			
		0.381	1.801	1.562
1.089	1.48	0.628	0.923	1.336
5.258	0.469	1.698	0.572	
	1.081	1.323		
1.033	2.162	1.31	1.131	1.649
1.034	1.397	0.836	1.225	1.244
	0.794	0.697	0.561	0.476
0.345	0.634	0.177		
1.42	0.253	0.863	1.132	1.194
0.232	0.785	0.715	0.369	1.148
	1.143	0.839		
1	0.807	1.07	1.02	1.175
3.024	1.018	1.242	1.117	1.453
0.581	0.685	1.503	1.016	0.699
0.764	1.147	1.589	1.017	0.549
1.066	0.854	1.453	0.892	0.776
		0.612		
2.302				
	1.605			
0.681	0.503			0.274
0.449	0.895	1.104	2.102	1.163
1.803	0.881	1.717	0.703	0.856
0.607	0.631	2.337	1.063	
1.213	1.892	1.61	0.803	0.347
3.244			1.03	0.67
1.144	0.413	0.802	1.137	0.563
	0.409		1.416	1.541
		1.339		0.789
0.733	0.878	0.977	1.099	1.195
0.541	1.015	1.192	1.987	0.583
		0.8		1.019

2.61	0.75	0.329	1.391	0.789
1.251	1.271	1.244	1.026	0.98
0.659	0.81	1.555	1.073	0.868
1.573	0.719	0.701	1.377	1.148
0.799	0.835	1.209	1.368	1.243
0.69	1.286	1.664	1.189	0.974
0.01	1.134	2.326	1.375	
1.566	0.69	1.063	2.153	1.137
1.03	1.341	0.986		1.719
1.654				
1.249	1.518	1.234	1.716	1.198
0.738	1.47	1.133	0.379	0.424
0.253	1.143	1.485	1.054	0.712
0.669				0.905
1.104	1.011	1.483	1.205	1.01
0.596	0.639	0.326	0.744	1.227
0.496		0.785	2.271	
1.096	1.451	0.909	0.764	1.292
		1.726	0.901	
1.287	1.271	1.329	0.787	0.909
0.11				
0.987	1.127	1.53	1.458	1.403
0.86	1.897	1.741	0.613	1.13
0.899	0.953	0.885	0.807	0.709
1.124	1.755	1.197	0.894	0.924
0.825	0.293	0.975	0.984	0.804
0.832	1.037	0.977	1.275	1.325
0.699	0.963	0.943	0.959	1.56
1.001				
0.323	1.025	0.8	0.663	0.88
0.949	1.058	0.831	1.048	1.127
1.167		0.853		1.452
1.935	0.854	0.446	1.004	1.407
0.911	0.569	0.133		0.61
0.924	1.091	1.673	0.755	0.819
0.708	1.56	2.092	1.315	0.744
0.743	0.909	0.961	1.011	0.873
		1.572		0.84
0.856		1.015	1.805	1.207
0.509	0.593	0.844	1.558	1.714
0.926	0.507	0.689	1.22	1.384
0.707		0.629		
0.685	1.366	0.97	1.664	1.07
0.178			0.223	0.382
		0.73		
0.226		3.728	1.204	0.506

3.169		2.081	0.447	
1.619	2.656	1.213		
2.411	2.205	0.536	0.714	0.285
0.801	0.804	1.236	1.594	1.099
0.658	1.735	1.033	0.68	1.352
1.278	0.174	1.515	0.395	0.625
0.361	0.701	1.742	1.3	0.762
0.685				
1.056				
1.163	0.016	1.039	1.208	1.166
1.81	1.386	0.995	0.451	1.068
	1.222	1.665		
2.136	0.286	0.535		0.504
1.712	1.622	0.907		
1.767	0.521	1.274	1.517	1.473
1.378	1.117	1.635	0.82	1.075
1.075	1.127	0.774	1.376	1.121
	1.101	0.768		
1.879	1.307	1.049	1.464	1.006
0.681	1.109	1.32	0.729	0.655
2.609	0.376	0.268	1.944	
0.692	1.352	1.937	0.98	0.527
0.905	1.28	0.925	1.184	0.8
1.042	0.844	0.661	1.222	
0.608	0.473	1.128	0.518	0.546
0.93	0.867	0.899	1.401	1.275
1.05	1.156	1.68	0.744	1.02
		0.645		
0.399	1.747	1.268		1.276
1.309	0.564	1.914	1.145	0.883
2.066	2.273	0.063	0.099	0.143
1.315	0.811	1.252	0.471	1.066
2.129	1.205	1.033		0.935
0.039	0.028	0.007	0.404	0.416
0.945	1.463	1.541	0.786	0.564
		1.504		
0.438	1.554	0.566	1.01	1.01
2.082	0.787	0.728	1.638	0.567
0.326	0.757	1.743	2.453	0.344
0.892	0.954	1.101	0.679	0.861
0.554		1.326	1.126	0.98
0.826	0.736	0.466	0.427	0.99
0.469	1.378	2.341	1.613	0.756
	0.552	2.751		
0.368	0.833	0.435	0.996	1.134
0.685	0.404	0.177	2.068	1.764

1.156	1.098	1.405	1.145	0.896
0.629	1.316	2.575	2.041	0.876
1.258	1.206			
0.946	1.623	0.926		
1.294	1.172	1.046	0.902	0.917
	1.755	0.667	0.731	1.057
0.755	0.958	0.67	1.13	0.885
1.161	1.017	0.55	0.609	
0.316	1.891	2.215	1.002	0.906
0.876	0.213	0.835	1.413	1.154
0.763	1.305	0.46	0.123	0.103
0.078	1.441	2.501	2.528	0.331
0.104		3.826	1.069	0.662
		0.925	0.461	0.656
0.534	1.379	0.745	0.609	1.732
1.801	1.642	1.215	1.033	1.166
0.449		3.166	0.783	0.866
1.37	1.79	1.221	0.34	
0.601	0.645	0.385	0.526	1.464
0.562	0.211	0.11	0.22	0.169
	1.424	0.842	1.174	0.943
0.017	0.043	2.477	0.133	1.384
0.181	1.123	1.303	1.231	0.851
0.749	1.103	1.333	1.828	1.033
2.008	0.914	1.473	1.707	0.91
1.686	0.615	1.546	1.354	1.167
0.827	0.915	0.499	0.499	0.685
0.501	0.756	0.838	0.869	1.044
0.506				0.577
1.624	1.053	1.089	1.527	0.42
0.667	0.874	1.05	1.077	1.542
	1.082	0.941		
1.982	0.596	1.373	0.682	0.742
0.925	0.702	1.821	0.917	0.824
2.341	0.482	1.189	0.722	1.759
1.458	0.113	0.387	1.67	0.976
0.561	2.51	0.612	1.709	1.438
0.862	1.289		0.016	0.696
0.922	1.549	1.388	0.678	0.789
1.608	0.726	0.362	0.844	1.348
		0.541		1.375
0.964	0.586	1.303		0.825
0.817	1.057	1.421	0.926	0.891
0.379	0.202	0.24	0.406	
0.891	0.999	0.623	1.181	1.177
1.624	1.74	1.243	0.607	0.761

1.484	0.827	1.039	1.548	1.417
0.897	0.587	0.736	1.371	1.117
0.771	1.597	1.489	1.449	1.11
		1.514	0.994	0.977
0.879	0.561	1.34	0.854	
0.929				
		0.913		0.696
0.502	0.449	2.645	1.051	0.788
1.136	1.497	0.956	0.815	0.857
		1.635		0.905
0.763	1.188	0.949	1.216	1.577
2.892	2.335	2.735		0.722
2.052	1.584		0.4	1.102
0.909	0.646	1.514	0.868	0.902
1.324	0.472	1.052	0.564	0.956
0.267	0.353	3.073	0.051	1.172
	0.202	1.994	0.645	0.244
0.968	1.408	1.319	0.807	1.131
1.134	0.73	1.171	1.143	1.035
1.267	1.116	0.665	0.975	0.843
	0.506	1.586		0.804
1.535	0.799		0.751	0.584
0.292	0.422	1.168	0.393	1.047
2.181	0.805			1.287
1.31	0.453			
0.985	0.715	0.589	0.969	0.783
0.735	0.434			0.954
1.196	0.902	0.12	1.486	0.902
0.306	1.138	0.583	1.397	0.751
0.289	1.334	1.664	0.892	0.71
0.767	1.396	0.311	0.544	0.858
0.587	1.005	1.213	1.144	1.131
1.031	0.8	1.499	1.461	1.098
0.8	1.368	1.03	1.076	1.095
	1.666			
0.152	0.607	2.28	0.608	0.236
1.392	1.354	1.83	1.057	0.067
0.632	1.13	0.564	0.755	0.853
1.705	0.999	1.1	0.994	0.957
2.138	0.828	0.699	1.564	1.68
0.62	0.9	0.638	0.219	0.781
	1.889	3.036		1.125
2.6		1.454	0.583	
0.931	1.255	0.55	1.188	0.547
0.62	1.138	0.934	1.025	1.175

0.854	0.22	1.485	0.729	0.927
0.259	0.401	0.847	2.001	0.811
0.686	0.817	1.509	0.76	0.857
0.726	0.662	0.981		1.221
1.279			1.274	1.351
	0.1			
1.166	1.05	0.409	0.883	1.187
0.328	1.482	1.276	0.987	1.271
1.717	0.822	1.948	0.426	0.635
1.863	0.892	0.44	0.732	0.615
0.723	1.267	1.167	0.779	0.818
0.984	0.949	1.004	0.799	0.932
1.129	1.142	1.193	2.023	1.444
1.557	1.215	1.355	0.754	0.864
0.885	0.586	1.811	0.567	0.888
0.923	0.655	1.069	1.541	0.786
1.112	1.481	0.658	1.824	1.516
1.004	1.622	1.001	0.682	1.21
0.656	0.635	0.805	1.793	1.561
	0.437			
1.083	0.983	0.756	1.116	1.522
0.579	0.633	0.878	1.392	1.484
1.35	2.379	0.916	1.162	1.414
0.889		0.428		
1.032	1.227	0.433	1.351	0.856
	0.829	4.384	0.627	0.37
1.656	1.279	1.165	0.859	0.849
0.894	0.918	0.864	1.002	1.071
0.884	1.079	1.781	0.985	1.668
0.5	0.995	0.949	1.162	0.974
0.336	0.233	1.677	0.551	0.52
1.152	1.536	0.737	1.565	1.059
0.652	0.52	0.359	0.348	0.657
0.824	0.434	1.172	1.273	0.851
0.643	0.934	2.129	0.961	0.852
0.561	0.443	0.695	0.655	0.729
0.644	0.784	1.93	2.13	1.225
1.317	1.185	1.388	0.789	0.98
0.678				1.008
1.464	0.966	1.45	1.846	1.317
1.219	1.068	1.777	0.99	1.116
	1.205			
	0.063		0.016	0.087
1.135	2.226	0.227	0.439	0.31
1.827	0.792	0.996	0.912	0.953
1.38	1.519	0.952	1.042	1.583

0.637	0.867	0.628	1.503	1.05
0.921	1.112	0.839	1.439	0.733
0	1.015	0	1.277	1.429
0.298	1.493	1.93	1.093	1.487
2.262	1.008	1.54	1.316	0.793
		1.256	0.713	0.76
0.987	1.159	0.754	1.123	1.016
0.216	1.571	2.54	0.685	0.882
0.792	0.446		0.577	1.388
0.7	0.974	1.458	0.537	1.212
0.703	1.165	1.052	0.761	1.147
1.401	0.224	0.244	0.816	0.325
1.405	0.869	1.29	1.044	1.062
1.479	0.468	1.673	0.699	0.695
0.657		1.221		0.876
0.978	1.362	1.364	0.792	1.158
0.581	1.111	0.66	1.103	1.269
1.034		0.723	0.753	1.66
0.759	0.353	0.753	1.551	1.086
	1.462	1.362		
0.865	1.795	1.168	1.26	0.967
1.086	0.83	0.665	0.585	1.111
1.025	1.152	1.481	1.109	0.752
	0.835	0.979	1.044	0.416
1.95	1.194	1.031	0.906	1.051
0.424	1.001	1.876		
0.843	0.139	2.035	0.172	1.28
0.589			1.143	1.181
0.714	0.713	0.746	1.294	0.932
1.933	0.991		1.075	0.784
0.809	1.635	1.491	0.826	1.227
1.263	2.895	1.165	1.405	1.35
0.534	1.038	1.516	1.064	1.178
2.462	0.722	0.561	0.665	1.023
0.656	0.626	0.793	0.747	0.633
0.847	0.793	1.514	0.746	0.755
1.059	0.733	1.136	0.98	1.155
2.381	0.582	0.281	0.401	0.423
0.799	0.874	0.674	0.829	1.014
1.997	1.032		1.335	2.366
0.651	0.61	0.663	3.397	0.622
0.574	1.264	0.936	1.034	1.437
0.153	2.917	0.893	3.515	1.404
1.015	0.495	1.073	1.08	1.089
1.525	0.861	0.758	0.668	1.617
		1.01		
0.218	0.735	2.966	0.951	0.599
0.245	2.084	0.418	0.768	0.844

1.049	0.339	0.882	2.002	1.217
2.124	0.75	0.751	0.605	1.876
1.859	1.279	1.009	0.881	1.167
1.362	1.789	0.223	0.12	0.943
0.881	0.666	1.312	1.284	0.994
1.116	0.904	1.022	1.312	0.94
0.376	1.841	0.338	1.489	0.366
1.22	1.106	1.607	0.846	1.156
	0.457	0.993	0.82	0.785
		0.799	1.205	0.722
0.568	0.797	0.862	1.143	1.005
2.345	1.06	0.651	1.319	0.785
1.292	1.425	0.66		
1.152		1.297	1.99	0.124
	0.605	0.779	0.513	1.851
1.188	0.46	2.062	1.253	0.099
0.646		1.123	1.656	2.617
0.817	1.353	1.251	0.856	0.854
0.136	1.978	0.253		
1.701	1.335	0.518	1.132	0.631
0.916	0.964	0.79	1.11	1.054
1.865	1.187	0.767	1.833	0.918
3.001	0.701	0.31	0.486	0.53
0.748	0.571	0.379	1.079	1.024
0.79	0.842	0.858	1.053	1.239
1.1	0.681	0.686	0.149	0.635
0.866	2.313	0.66	0.75	0.65
0.929	0.894	1.324	1.913	1.016
1.758	1.137	0.701	1.037	1.131
0.28	0.217	0.263	0.159	0.198
	1.047			
1.204	1.101	0.905	1.295	1.162
1.121	1.406	0.848	0.919	1.045
1.984	0.623	2.098	1.057	0.69
0.796	0.349	0.877	1.581	0.959
0.905	2.153	1.062	0.929	1.078
0.558		0.941	0.427	
0.935	0.913	2.02	0.422	0.52
0.648	1.829	1.04	1.086	0.594
0.969	0.878	1.118	0.897	1.74
1.27	0.845	1.342	0.27	0.903
1.265	2.919	0.118	0.884	0.973
1.148	0.546	0.776	1.18	1.574
	0.472	0.178	3.262	1.081
1.47	1.023	0.863	1.067	0.967
1.394	1.263	0.668	1.059	1.102
1.024	1.662	0.709	1.105	0.979

0.22	0.547	0.833	0.991	1.306
0.497	0.808	1.197	1.085	1.366
0.94	1.301	1.318	0.519	0.736
0.813	0.587	1.246	0.654	0.98
1.246	1.309	0.611	1.239	1.104
1.863	1.259	1.245	1.088	0.536
1.181	1.245	0.776	1.186	1.302
1.212	1.099	0.595	1.05	0.75
1.156	1.26	1.043	0.855	0.867
8.218			0.463	0.448
1.214	0.718	0.756	1.202	0.797
0.362	1.535	1.965	1.472	1.02
	0.515	0.787	1.344	1.65
0.838	1.675	1.433	0.525	0.997
0.558	1.167	0.839	1.212	0.748
0.861				
0.828	1.782	1.284	0.932	0.927
0.407	1.531	0.951	0.734	0.977
2.067	1.017	0.921	1.102	0.768
1.156	0.798			0.613
0.664	0.586	1.832	0.758	0.891
0.977	0.176	1.462	1.577	1.217
0.843	0.622	2.522	0.63	1.241
1.451	0.818	1.051	0.919	0.953
1.602	0.785	0.866	1.117	1.188
9.056	1.542	0.461	0.873	0.539
0.855	0.954	0.684	1.087	1.315
0.523	0.598		1.247	1.324
0.521	0.768	2.003	3.296	0.569
0.75	0.822		1.589	0.861
		0.515	1.668	1.156
0.679	0.957	0.986	1.056	1.134
0.997	0.844	1.02	1.392	1.369
0.849	1.736	1.402	0.427	0.541
1.509				
1.492	1.125	0.973	1.212	1.068
0.704	1.337	1.748	1.343	1.095
1.126	0.29	0.837	1.198	1.124
0.538	1.426	0.819	1.281	1.562
0.047				
	0.425	0.953	0.62	
0.981	0.899	0.759	1.281	1.396
1.046	0.969	1.43		0.799
2.853	0.175	0.413	0.906	0.442
9.399	0.445	0.724	0.996	0.789
0.501	1.437	2.654	1.247	0.565
1.192	2.331	1.567	0.829	1.088
2.381	2.087	0.487	0.52	
1.084	1.265	1.588	1.014	0.876
0.888	0.769	0.85	1.112	1.292

0.905	0.907		1.185	
1.001	1.228	0.493		0.632
2.154	1.366	0.967	1.61	1.58
0.913	0.655	0.975	0.797	0.803
1.199	1.189	1.024	0.898	1.255
0.887	1.011	0.962	1.27	1.294
1.306	0.825	1.504	2.079	1.353
0.966				1.989
1.214	0.88	1.526	1.162	0.736
1.027	1.308	0.878	0.934	1.196
1.011	0.676	2.294	0.811	1.295
		1.911		0.847
1.782	1.938	2.494	2.006	0.378
0.186	0.147	0.217	1.446	1.015
1.799	0.925	0.624	1.241	0.815
1.159	0.757	0.755	0.909	1.239
0.429	0.441	1.139	1.334	0.85
0.564	1.198	0.946	1.848	1.54
1.916	1.285	1.522	1.083	0.571
1.288	1.811	0.649	0.682	0.956
1.091	0.395	1.054	1.371	0.75
0.719	1.236	1.758	0.551	0.908
1.019		0.984	0.15	1.78
0.767	0.937	1.172		0.581
1.247	1.164	1.097	0.967	0.854
6.572	1.244	1.35	0.774	0.834
1.008	0.676	1.291	0.737	1.08
1.173	2.374	1.326	1.143	1.057
0.725	1.161	0.799	1.359	1.662
1.42	0.581	1.299		
1.425	1.576	0.613	0.936	1.196
		1.358		
0.858	1.031	0.596	1.302	1.197
0.473	1.318	1.553	0.723	0.674
1.18	0.906	0.957	0.953	1.173
1.332	1.118	0.801	1.228	1.273
1.019	1.01	3.76	0.784	0.343
1.096	0.976	0.842	1.434	1.203
0.941	0.946	1.034	0.433	1.288
0.667	1.022	0.686	0.64	
0.261	2.586	1.781	1.911	2.003
0.142	0.89	1.876	0.685	0.84
1.129	0.65	0.512	0.852	0.799
0.314	0.923	1.78	0.979	0.574
0.431	0.815	0.827	0.816	1.052
0.273	0.461	1.995	1.752	0.51
1.036	1.337	0.642	1.049	1.047
	1.326			
2.11	0.796	1.003	1.276	0.3
		0.983		1.086

0.48		0.86		0.58
1.044	3.195		0.746	
1.808	0.876	1.013	1.453	1.392
0.978	0.979	0.849	0.961	0.879
0.991	1.046	1.265	1.002	0.995
3.025	0.827	0.474	1.415	0.542
1.083	1.254	0.944	1.356	1.373
1.3	0.15	1.788	0.433	0.172
0.513				
0.576	1.483	0.891	1.366	0.704
0.839	1.969	0.663	1.626	1.305
0.877	0.548	2.221	1.108	1.243
0.285	1.055	1.305	1.334	1.243
0.225	0.633	2.134		
4.334	0.695	0.441	1.075	0.824
2.415	0.5	0.579	1.164	0.691
2.497	0.994	1.528	2.25	0.976
1.227	1.403	0.892	1.324	1.375
	1.055	1.619		
0.98	1.313	0.684	0.736	1.393
	1.334	2.068	0.132	0.083
0.53	1.008	1.39	0.964	1.388
0.799	0.867	0.605	1.003	1.261
0.791		0.79		0.67
0.668	0.75	1.169	1.026	1.048
1.249	1.166	0.595	1.171	1.577
0.921	0.844	0.958	0.656	1.341
2.233	2.213	1.201		0.685
1.124	0.784	0.803	1.11	1.285
0.413	1.164	1.645	1.18	0.911
0.636	0.707	1.205	1.098	1.079
7.956	0.528	0.341	0.722	0.496
1.051	0.904	0.842	1.214	1.295
1.039	0.591	1.244	0.603	0.75
0.859	1.162	0.47	0.979	1.258
0.485	0.853	1.945	1.123	1.145
1.468	0.974	0.786	0.834	0.714
1.101	0.807	0.531	0.506	0.658
2.241	0.584	1.847	1.449	0.473
	1.614	1.007	1.105	1.157
2.203	0.883	0.335	1.275	0.845
0.687		0.971	0.885	0.743
0.39	0.327	0.515	0.391	0.446
1.172	0.917	0.919	1.138	1.361
0.209		1.783	1.043	
7.803	0.58	0.695	1.207	0.394
1.414	1.205	0.663	0.986	1.06

0.459	3.984	0.368	0.625	3.003
0.993	0.864	1.571	0.773	1.194
1.679	0.48	0.454	0.916	0.967
1.532	0.895	0.834	1.261	1.309
0.297	0.393	0.251	0.635	1.497
		1.241		
7.016	0.369	0.39	0.509	1.009
0.103	0.602	0.002		0.113
	0.587	1.868	0.922	
1.26	0.511	1.342	2.163	0.907
1.963	1.354	0.907	1.631	1.427
0.871	1.743	1.34	1.083	0.719
0.527	1.65	0.691	0.933	0.754
1.268	1.126	0.945	1.274	1.315
1.414	1.573	0.817	1.078	1.32
1.022	1.218	1.187	0.733	0.853
0.997	0.906	0.582	0.983	1.931
1.232	1.484	1.446	0.772	0.959
1.184	1.769	1.077	1.109	1.29
1.844	1.083	1.553	1.676	1.197
0.726	1.597	1.575	1.509	0.774
0.229	0.365	1.115	0.927	0.584
1.017	0.934	1.144	3.565	0.79
1.843	0.242	0.094	1.424	0.249
1.164	1.005	1.325	1.134	1.111
3.703	0.746	2.523	0.282	0.606
0.455	0.95	1.103	0.72	0.848
0.637	0.858	1.134	1.304	1.084
1.092	0.736	0.792	1.528	1.577
5.807	0.794	0.727	0.941	0.442
0.46	1.419	0.64	1.142	1.996
0.859	0.716	1.648	1.247	1.197
0.547	0.385	1.922	1.021	1.058
1.086	1.503	1.021	1.2	0.963
0.785	1.147	0.7	0.918	1.21
1.045	0.782	0.636	1.03	1.3
1.2	0.812	0.782	1.176	0.925
1.501	0.497	1.222	0.923	0.826
1.257	0.238	1.322	0.778	1.104
		0.564		
1.451	1.08	0.542	0.438	1.155
0.097	0.196	0.67	1.073	0.308
1.181	1.767	0.283	0.58	2.076
0.715	1.397	0.717	0.981	0.9
4.275	0.334	0.3	1.056	0.373
0.915	0.963	0.728	1.396	1.247
0.58		1	0.77	1.125

1.035	0.737	1.258	1.155	1.126
0.672	0.363	0.914	0.826	0.785
0.844	1.15	0.802	1.204	1.284
	0.878	1.799		1.046
0.787	1.251	1.081	0.862	0.915
0.873	1.065	0.815	0.866	0.999
1.395		2.232	1.941	0.272
1.771	0.77	1.154	0.623	0.863
1.043	1.477	0.592	1.176	1.588
1.261	0.954	1.26	0.857	0.982
0.925	0.854	1.073	1.061	1.078
1.163	0.705	1.599	1.26	1.439
1.365	0.766	0.881	0.944	1.197
0.89	0.489	1.197	1.178	1.507
0.994	0.728	0.905	1.08	0.796
0.446	0.224			
1.261	0.978	0.536	0.764	0.545
0.837	1.515	0.53	0.571	1.455
1.79	1.347	0.951	1.757	1.409
0.507	0.43	0.565	0.794	0.8
1.061	1.635	0.405	0.65	0.655
0.938	0.946	0.932	1.307	1.097
1.076	1.587	1.043	1.568	1.276
0.157	0.629	0.827	1.866	1.54
1.639	1.956	1.991	0.242	0.161
1.239	1.086	0.701	0.859	1.093
1.278	2.077	2.795	0.715	0.345
1.121	1.432	0.877	1.669	0.941
0.811	3.436	1.713	1.095	
0.82	0.615	1.066	2.124	0.723
1.249	1.719	1.192	0.583	0.822
1.245	0.642	1.203	1.744	1.309
1.262	0.894	1.373	0.837	0.668
0.687	0.64	1.108	0.387	1.341
1.033	0.646	1.635	0.909	0.834
1.403	1.192	1.166	1.171	1.476
1.024	1.143	0.908	1.286	1.176
1.34	0.954	1.664	1.108	1.516
1.645			0.902	2.447
0.923	0.957	1.421	0.814	1.007
1.425	2.258	0.641	0.822	0.452
0.473	0.793	1.072	0.57	1.11
1.06	1.135	1.229	1.008	0.825
0.709	0.942	1.004	1.104	1.221
0.755	0.382	0.573	0.595	0.671
1.341	0.842	0.633	1.564	1.739
1.143	0.979	0.987	0.761	1.104

1.051	0.954	1.109	0.91	0.926
1.707	1.284	0.813		0.727
0.399	0.901	0.462	0.915	1.055
1.374	1.311	1.889	1.368	0.894
0.964	1.045	0.954	0.645	0.632
0.873	1.192	0.562	0.653	0.544
1.06	1.25	0.688	1.239	1.09
1.214	1.007	1.141	0.797	0.974
0.799	1.252	1.866	1.273	0.865
1.056				
1.813	0.464	0.722	1.003	1.345
0.111	0.494	0.9	1.405	0.372
1.204	1.453			1.132
0.779	1.3	1.08	1.098	1.277
0.935				
1.341	1.76	1.249	0.696	0.721
		1.117		
0.715	1.105	1.002	1.02	1.136
1.182	1.025	0.985	1.47	0.731
0.358	0.169	2.14		0.3
0.903	0.834	0.903	0.921	1.168
1.163	1.24	0.971	0.739	0.742
1.702				
5.19	1.08	0.939	1.186	0.578
1.096	1.106	1.286	0.957	1.071
0.949	1.168	1.006	1.219	0.832
0.353	1.741	0.854	2.287	1.049
0.829	1.41	0.945	0.959	0.88
0.984	0.629	1.4	0.478	0.727
1.157	0.869	1.033	1.041	1.208
1.262	0.614	1.577	0.968	1.289
0.771	0.893	0.451	0.967	1.085
0.57		1.931	0.993	0.818
0.95	1.436	1.038	1.096	1.086
1.22	1.094	0.695	1.147	1.7
1.453	1.388	1.206	0.667	0.839
0.109				
4.586	1.281	0.223	0.309	0.179
0.725	1.209	1.3	1.038	1.195
0.568	0.595	1.935		0.856
0.731	1.588	0.977	1.255	1.36
1.062	0.63	0.566	0.562	0.852
		1.074	3.18	
2.603	0.978	0.69	1.147	0.69
0.824	1.196	1.167	0.814	0.809
2.242	0.873	1.056	1.507	1.297

0.814	1.455	0.469	1.042	0.932
1.294	1.599			0.974
1.332	0.339	0.866	1.116	1.071
0.164	1.725	1.088	1.888	0.645
1.164	1.058	0.908	0.862	0.941
1.907	0.753	1.537	0.719	0.607
1	0.911	0.996	0.997	0.784
0.961	0.805	1.191	1.479	1.332
0.875	1.133	1.227	1.31	1.51
1.864	1.365	0.932	0.753	0.542
2.53	2.751	0.382		0.379
1.305	0.967	1.037	1.444	1.382
0.971	0.918	1.193	0.636	1.068
	1.227	0.995	1.091	0.459
0.765	0.88	1.504	0.991	1.033
1.06	2.107	0.51	0.71	0.037
1.237	1.02	1.664	2.028	1.404
0.428	0.868	0.84	0.909	0.688
0.825	0.788	1.199	0.568	0.654
1.13	1.034	1.623	0.442	1.851
2.595	0.808	1.048	0.528	0.807
	1.994	1.341		1.364
0.542	0.992	0.902	1.777	1.082
0.506	1.164	1.08	1.029	1.072
0.798	1.343	0.705	0.901	1.269
0.968	0.363	1.138	0.886	0.987
0.518	1.006	1.176	0.47	1.49
1.026	0.565	0.743	1.116	0.915
4.519	0.76	0.766	1.095	0.458
0.947	0.586	0.868	1.142	1.449
0.964	1.569	0.623	1.808	0.679
5.242	0.224	0.703		0.851
1.17	0.697	0.763	1.046	1.4
0.684	1.161	1.091	1.154	0.924
1.842	0.863	1.214	0.913	0.358
0.591	1.533	0.909	1.445	0.997
1.343	1.541	0.314	0.591	1.018
1.512	1.06	0.471	1.124	1.085
0.743	1.274	0.673	0.818	0.509
1.37	1.817	0.52	0.935	0.532
0.835	0.857	0.638	0.603	0.901
0.674	1.131	0.864	0.523	0.613
1.04	0.621	0.461	1.458	1.454
1.105	1.491	0.582	0.583	1.025
		0.096		

0.822	0.856	0.928	1.388	0.957
	1.208			1.003
	0.93			
0.696	1.303	0.921	0.802	0.742
0.17	1.056	0.496	0.056	
2.077	0.954	0.896	0.741	0.437
1.362	0.585	0.88	1.295	1.141
1.03	1.25	0.335	0.762	1.083
2.156	2.423	0.737	0.702	0.74
0.72	0.928	0.667	0.781	0.755
0.75	0.871	1.325	1.439	0.732
1.273	1.433	0.655	0.848	1.125
0.668	1.535	0.754	1.401	1.063
1.035	1.045	0.992	1.058	1.119
0.737	0.542	1.185	1.298	0.845
1.207		0.738		
1.256	1.697	1.334	0.539	0.685
0.283	1.224	0.874	0.835	1.806
2.141	1.481	1.174	0.926	1.183
1.032	1.352	1.773	1.543	0.705
0.988	0.818	0.54	1.194	1.21
1.824	1.043	0.855	1.329	1.485
0.437	0.699	0.676	1.298	1.721
0.418				0.664
1.636	1.762	0.439	1.182	1.232
0.88	0.827	1.279	1.274	0.966
1.493	1.291	0.794	1.064	1.033
0.808	1.409	1.413	0.97	0.946
0.827	0.845	1.094	1.459	1.496
0.871	1.234	1.153	3.63	0.89
0.44	1.299	1.621	0.814	0.468
0.697	1.068	0.96	1.429	1.282
0.728	0.582	3.533	0.736	0.517
0.156	0.474	1.526	1.885	0.807
1.057	0.687	0.466	0.414	0.285
1.343	1.369	1.197		
1.069	1.17	0.754	0.991	1.201
0.551	0.622	0.524	0.751	0.87
0.653	0.8	0.522	1.111	0.65
1.235	0.7	1.111	0.747	1.033
0.958	0.757	0.713	2.531	0.527
	0.942	0.214		1.439
1.03	1.148	1.089	1.43	1.603
0.723	1.794	1.317	1.578	0.987
0.988	0.632	0.417	0.996	0.974
0.37	1.483	0.81	4.248	0.892
0.906	0.825	2.006	0.625	0.213
9.735	1.365	0.929	0.546	

1.072	0.876	0.706	0.941	0.777
0.776	1.091	0.928	0.803	1.09
0.825	1.997	0.462	0.898	2.629
0.791	0.181	0.002	0.89	0.727
1.968	1.92	0.892	0.529	
1.292	1.805	0.43	1.003	1.322
0.531	0.927	1.157	1.575	1.08
1.11	1.323	0.488	0.574	1.229
0.615	0.979	1.077	1.513	
1.001	0.633	0.95	0.904	0.818
	1.559	1.019		
1.383	1.434	0.646	0.844	1.373
0.922	0.666	1.455	1.357	0.881
0.562	1.639	2.849	0.537	0.331
1.212	0.912	0.357	0.517	1.294
1.488	1.586	1.458	1.491	1.442
0.972	1.035	0.601	0.647	1.2
0.292	0.528	0.202	0.577	1.128
1.006	0.793	0.639	0.964	0.713
0.721				
8.367	1.217	1.731		
0.66		0.495		0.601
1.674	0.977	0.853	1.435	1.337
0.274	0.354	2.057	1.451	0.802
5.048	1.308	0.98	1.121	1.256
0.329				
0.498	0.726	0.851	1.518	0.751
1	0.857	0.97	0.923	1.346
1.411	1.529	1.315	0.919	1.13
0.925	1.033	0.871	1.3	1.787
1.732			0.375	0.757
0.569	0.457	2.283		
	1.29	2.047		
1.025	0.885	1.115	1.031	1.217
1.204	1.567	1.15	0.759	0.922
1.373	0.685	0.623	0.575	0.833
0.849	2.08	0.9	0.662	1.059
0.096	1.561	0.514	1.173	0.075
1.354	0.815	0.827	0.993	1.161
0.723	1.027	0.724	0.737	0.934
1.696	0.915	0.74	0.506	0.921
1.363	0.83	0.741	1.011	1.556
0.007	0.638	0.867	0.478	0.032
1.054	1.468	0.849	1.258	1.066
1.359	0.944	1.107	1.192	1.175
	0.05	1.504	0.205	2.048
1.545	1.121	1.034	1.397	0.814

0.15	1.626			
1.473	0.953	0.998	1.841	1.217
0.376	0.71	1.335	0.872	0.926
0.792	1.112	1.002	0.989	0.739
1.049	1.099	1.282	1.376	0.928

1.1	1.103	0.826	1.239	1.159
1.402	1.47	1.259	1.411	1.389
1.031	0.665	0.584	0.959	1.042
1.176	0.841	0.863	1.793	0.909
1.22	0.492	1.051	0.615	1.528
1.281	0.951	0.872	1.395	0.767
1.207	0.974	0.974	1.322	1.119

1.031	0.974	1.023	0.82	1.56
0.776	1.398	2.349	0.705	0.605
1.937	0.562	0.939	0.27	0.469
9.349		0.272		
0.824	1.093	1.55	0.537	1.429
0.323			1.668	
0.598	0.872	0.947	1.212	2.229
1.205	1.467	0.653	0.876	1.031
0.988	1.51	0.797	0.753	1.123
3.184	1.165	1.042	1.226	0.776
0.508	0.91	0.6	0.199	
1.805	0.921	1.126	0.703	0.726
0.922	1.342	0.679	1.758	1.134
2.328	1.039	1.08	1.034	1.02
0.639	2.077		1.326	1.12
1.271	1.65	1.105	0.83	0.884
0.838	0.982	1.183	1.666	1.029
1.188	0.793	1.318	1.503	1.22
1.997	1.076			1.327
0.222	1.793	1.635	1.187	1.325
0.792	0.585	1.192	1.545	0.874
0.95		0.943	1.15	
0.951	0.778	0.973	1.368	1.137
0.302	1.819	0.795	1.855	1.15
0.88	1.596	0.82	0.669	0.855
0.584	0.561	0.811	0.914	0.477
		0.154	1.054	0.286
			0.526	0.371
2.642	1.409	1.136	1.197	0.933
	0.867	2.104		0.873
2.306	1.565	1.25	1.206	1.72
0.704	1.242	0.919	1.026	0.97
1.326				
1.641	0.405	1.739	0.685	0.558
2.062	2.475		0.75	0.575

0.933	0.495	1.653	1.368	0.948
1.507				
0.848	0.75	0.598	0.67	0.682
1.266	2.237	0.935	0.594	0.849
2.78	0.53	0.651	1.228	0.353
0.705	0.506	1.108	1.093	0.848
0.6	1.409	1.301	1.486	1.165
0.916	1.031	0.746	1.017	1.153
2.67				
1.379	0.273	0.503	1.3	1.171
1.528	1.363	0.919	1.399	1.295
0.857	1.029	1.951	1.584	0.637
0.781	0.969	0.591	0.605	1.017
1.077	1.234	0.756	0.667	1.06
0.827	1.125	0.886	0.997	1.186
1.181	1.663	1.272	0.698	1.072
0.935	0.982	0.617	0.798	0.957
1.41	1.014	0.864	0.832	0.447
0.346	0.555	1.433	1.286	1.491
2.76	0.426	0.423	0.601	0.663
0.644	1.426	1.336	1.327	0.741
0.756	0.937	0.941	1.241	1.176
1.454	1.696	1.146	1.713	0.788
0.817	0.402	0.437	0.313	0.41
1.188	1.471	0.709	0.976	0.854
1.138	0.875	0.903	1.493	1.253
2.475	1.866	3.402	2.722	0.538
0.395	1.425	1.106	1.742	1.294
1.131	1.03	1.284	1.175	1.067
0.869	1.292	1.447	0.961	0.986
0.273	1.031	3.317	1.296	0.984
13.905	0.4	0.821	0.053	0.498
9.27	0.744	0.674	0.893	0.438
1.312	1.294	1.24	1.037	1.937
1.061	0.999	1.003	1.14	1.314
		1.679		
1.156	1.463	0.815		0.801
1.678	0.73	1.323	1.05	0.734
0.963	1.027	0.795	1.304	0.873
1.206	0.822	0.571	0.948	0.748
0.606	0.153	1.239	1.579	0.801
1.549	1.517	1.184	0.951	0.873
0.43	1.354	1.548	1.189	1.114
1.062	0.815	1.189	0.954	1.067
2.136	0.584	0.227	1.367	0.702
2.834		1.852	0.345	

0.89	1.012	0.895	1.135	1.334
0.831	1.188	1.436	1.292	1.251
0.875	1.059	1.072	1.354	0.923
0.863	2.167	0.487	1.157	1.754
1.22	1.246	0.705	1.071	0.94
1.201	0.838	0.71	1.694	1.061
1.164	0.493	1.485	1.108	1.179
0.33	0.613	0.702	2.518	0.551
1.208	0.414	0.197	1.06	0.98
	2.391	0.686		1.196
1.023	0.93	1.116	0.719	0.844
1.072	1.215	0.982	1.415	1.563
0.943	1.218	1.166	0.851	1.008
1.165	0.868	1.23	1.668	1.502
0.571	1.636	2.954	0.698	0.331
		1.705	0.804	
0.173		0.776	2.017	
	0.518			
1.051	1.416	0.555	0.801	1.277
1.295	1.47	0.704	1.332	1.317
			0.324	0.291
2.599	1.888	0.203	1.747	1.044
0.877	1.317	0.881	1.097	1.462
1.038	0.937	0.75	1.235	1.746
0.698	0.548	0.768	0.782	0.696
1.77	0.69	0.756	1.031	0.853
1.102	1.075	0.565	0.837	1.031
0.849	1.474	1.989	1.333	1.072
1.722	1.523	0.474	0.481	0.557
0.779	0.594			0.032
1.241				
4.535	0.964		1.107	1.128
1.262	2.295	1.33	0.723	0.791
0.951	0.229	0.585	0.179	0.121
4.34		1.764	0.648	
0.382	1.21			
0.78	1.198	1.189	1.436	1.365
0.656	1.253	1.318	2.076	0.916
1.068	1.294	0.829	0.674	0.868
1.193	0.288	2.009	0.74	1.081
1.082	1.426	0.791	0.899	0.671
4.289				
3.832	0.58		1.275	0.926
0.767	0.345	0.742	2.124	1.812
1.636	0.946	0.426	0.454	0.652
1.02	1.085	0.246		2.05
0.35		5.547	0.371	0.261
0.26	1.181	0.744	1.164	0.615

0.041	0.082	0.531	1.874	1.237
1.065	1.411	1.24	1.466	0.915
0.992	0.539	0.871	0.894	
4.552	1.029	1.151	0.343	0.277
1.255		1.299	0.704	
1.113	1.269	1.265	0.915	1.057
1.333	1.041	0.692	1.119	1.164
0.507	0.854	0.411	0.594	1.076
2.215	2.849	0.161	0.217	1.638
1.421	1.502	1.453	0.769	0.891
1.061	1.155	0.864	1.576	1.169
1.675	0.607	0.708	1.041	1.865
1.212	0.904	0.82	1.335	1.114
0.662		2.038	2.007	0.967
			1.117	
0.51	0.276	2.74	1.849	0.728
0.886	0.488	0.734	0.437	0.217
0.937	0.86	0.798	0.903	0.672
1.872	0.453	0.545	1.088	1.103
0.538	0.779	1.147	0.694	1.307
1.787		0.432	1.246	1.243
2.126	0.049	0.513		
0.481		0.568		0.536
	0.218	0.093	0.139	
0.415	1.047	1.397	1.324	1.259
0.816	1.3	0.585	0.842	1.082
1.467	1.356		1.048	
1.187	0.873			
0.899				
0.572	1.78	0.422	0.712	1.324
1.339	1.135	1.15	0.812	1.695
				0.458
1.177	0.287	1.899		
1.253	1.12	1.31	1.007	0.74
0.985	1.19	0.809	1.456	0.85
1.172	1.312	1.088	0.771	1.35
0.158				0.444
0.481		0.587	3.541	
0.999	0.611	0.834	1.251	1.21
0.262	0.325	1.582	0.537	1.116
0.95			0.468	
0.607	1.284	0.622	1.76	1.122
0.715	0.969	1.888	1.402	1.301
0.867		0.403	0.707	0.75
0.462		0.512	0.928	0.65
1.313	0.766	0.968	1.498	0.89
1.269	1.185	1.29	0.982	1.194
1.456	0.694	1.448	1.649	1.099

0.719	0.743	0.542		0.962
3.016	0.334	0.423	1.003	0.613
0.669	0.651	0.762	0.492	
1.282	1.141	1.485	0.933	0.913
1.697				
0.799	1.491	0.077	0.151	0.696
1.431	0.809	1.686	0.906	1.094
3.213		0.475	0.962	0.193
0.884		0.328		0.352
1.176	1.716	1.167	1.252	1.675
	0.543	1.196		
3.77	0.652	3.037	0.04	0.346
6.186	0.086	0.478	0.251	0.937
1.476	1.05	0.975	1.223	1.054
0.451	1.144	0.907	1.398	1.339
1.672		1		1.263
0.903	1.304	1.368	0.229	1.029
0.423	1.43	1.308	0.728	1.136
0.333	0.764	0.62	1.17	1.019
	0.404	1.056	1.297	1.154
	1.011			0.797
2.727	1.557	1.769	0.536	0.535
	0.07			
1.309	1.001	1.184	1.181	1.45
2.5		1.304	0.6	0.401
2.619	1.383	1.568	0.806	0.542
1.162	1.228	0.626	0.763	1.012
1.02	0.587	0.583	0.889	1.069
1.148	1.14	1.325	0.887	1.043
1.764		0.533		0.764
0.876	0.831	0.733	0.984	1.229
12.232	2.216	6.247	0.243	0.234
3.178	0.823	1.115	1.188	0.814
		0.508		
0.355	0.95	0.853	1.149	1.14
0.587	0.607	0.329	0.515	1.652
0.852	0.656	1.44	1.739	1.052
12.414	1.64	3.765	0.237	0.687
0.678	0.366	0.389	1.055	0.745
0.815	0.505	0.809	1.122	1.151
0.776	1.036	0.905	1.136	1.226
2.104	1.558	1.207	1.154	0.915
0.911	0.766	1.548	1.79	1.273
	1.395	0.674		0.988
1.897	1.17	1.118	1.186	0.752
		0.99		
1.513	1.053	0.935	1.212	1.086

1.104	1.38	1.644	1.034	0.622
1.265	1.336	1.209	0.843	1.041
1.216	0.83	0.379	0.917	1.168
1.268	1.01	0.555	1.397	
1.071	1.765	1.084	0.991	1.3
1.057	0.337	2.302	1.94	0.875
0.516	1.946		1.527	0.66
0.572	0.676	0.505	1.251	0.774
0.746	0.401	1.849	0.406	
10.557	1.191	3.438	0.347	0.278
0.751	2.244	1.158	1.534	0.731
0.837	0.808		0.399	1.034
1.173	0.818	0.913	1.354	1.397
1.403	1.025	1.15	1.132	0.841
	0.906	1.003	0.694	
	0.116	2.003	0.069	0.037
1.096	0.804	0.767	1.279	1.222
0.916	0.485	1.901	0.181	1.031
0.37	0.603	1.001	0.754	0.881
0.484	0.817	0.456	1.069	0.981
	0.932			
0.844	0.974	1.733	0.864	1.286
1.218	1.097	0.995	0.656	1.12
1.406	0.7	0.426	0.857	1.072
	0.305	0.305	0.7	0.816
0.801	1.612	1.075	1.175	0.588
0.808	1.001	0.838	1.226	1.208
0.614	1.079	0.969	1.066	1.02
1.417	1.488	0.459	0.843	0.814
0.607	1.527	1.053	1.033	1.178
0.436	1.684	2.205	0.421	0.717
0.625	0.991	0.526	0.678	0.985
1.416	1.906	0.653	1.383	
1.231	1.167	0.782	0.471	0.876
0.716	0.673	1.416	0.442	0.584
6.033		0.299		
	1.211	0.364		
0.254	0.348	2.227	0.354	0.586
1.286	1.189	0.817	0.626	1.383
1.509	0.98	0.882	1.36	0.952
0.828	1.225	1.902	0.84	0.526
0.681	1.062	1.268	0.784	0.608
2.421	1.127	0.906	1.11	
1.231	0.819	0.58	1.198	1.061
0.926	1.071	0.722	1.104	1.332
1.242	0.967	0.916	1.048	1.175
1.772	0.403	0.604	0.179	1.324
0.526	0.939	0.911	1.464	1.023

		0.911		
1.635	1.553	1.286	1	0.86
1.418	1.328	0.394	0.82	0.856
		0.208	1.094	0.709
0.766	1.274	1.106	0.749	0.83
1.055	0.847	0.957	0.557	0.936
3.24	0.384	1.458		
1.51	1.935	0.408	0.933	0.843
0.858	1.562	1.832	1.585	0.879
1.524	0.831	0.839	1.25	0.706
0.954	0.711			1.435
0.588	0.808	0.433	1.302	0.603
1.318				
0.93	1.139	1.41	1.165	0.387
0.853	1.281			
0.761	1.377	2.073	0.935	0.897
1.273	0.488	1.51	0.876	0.897
1.209	1.297	0.718	0.794	1.114
0.874	1.459	1.205	0.766	0.916
0.498	1.12	2.778	1.69	1.016
1.585	1.453	0.754	1.275	1.143
0.38	0.569	2.731	1.077	1.088
1.477	0.847	0.372	0.912	0.973
0.794	0.373	2.078	0.839	1.088
0.858	0.525	0.818	0.686	1.531
	2.342	1.014	1.526	
0.919	0.705	1.206	0.718	1.046
1.025	1.328	1.276	0.697	0.932
0.964	0.79	1.672	0.774	1.045
0.267	0.495	0.118	0.21	0.241
1.016	1.055	0.918	1.349	1.337
0.813	0.333	1.726	1.233	0.398
0.849	0.884	1.2	1.741	1.299
0.85	0.285	1.056	1.428	1.16
6.665	0.661	0.93		0.388
0.632	1.122	1.121	1.19	1.012
3.07	0.74	1.319	0.813	0.755
0.708	0.64	0.944	1.3	1.036
1.861	1.514	0.682	0.953	0.484
1.704	1.284	1.049	0.907	0.775
0.869	0.756	1.561		0.903
0.723	2.474	1.518	0.569	0.663
0.639	1.117	0.692	0.778	0.734

	0.631			0.778
1.514	1.766	0.975	1.087	
0.933	1.326	1.366	1.149	0.922
0.658	1.005	0.733	0.881	1.218
1.435	1.521	0.976	1.143	1.298
0.418	0.775	1.456		
0.965	1.313	0.994	2.08	0.67
1.342	1.793	0.605	0.726	1.172
0.8	1.043	0.806	0.974	0.987
	0.143	0.561	0.879	
0.976	1.321			0.513
0.732	0.8	1.049	1.39	1.485
0.489	1.798	1.051	1.603	1.266
1.539	1.124	0.868	1.275	0.867
1.682	1.652	0.381	1.458	1.328
1.802	1.401	0.527	0.777	0.901
0.771	0.897	0.513	0.762	0.779
	0.042			
1.042	0.373	1.465	1.29	1.27
1.736	0.925	1.51	1.203	1.344
0.457	1.153	1.178	1.382	1.215
0.923	0.398	1.409	0.838	1.038
1.093	1.076	0.891	0.479	1.048
0.274	1.319	1.254	0.987	0.46
1.24	1.651	1.791	0.694	0.762
1.307	0.586	0.666	0.981	1.023
1.061	0.633	1.132	0.79	1.007
1.194		0.853	0.309	0.653
1.242	0.781	1.432	0.268	0.792
0.811	0.246	0.744	0.412	1.175
0.972	1.051	1.038	0.81	1.032
			1.343	1.178
0.946	0.47	1.199	1.322	1.82
1.789	1.503	1.2	1.337	1.613
1.031	0.593	1.425	2.323	0.568
		0.291		
0.851	0.563	1.47	0.689	1.055
0.04	1.331	1.379	0.756	0.647
1.707	2.166	1.178		2.242
		0.867		
0.292				0.701
0.789	1.573	0.686	0.812	1.04
0.899				
0.755	0.501	1.508	0.698	0.944
0.501	1.614	0.795	1.218	1.348
1.51	1.557	0.84	1.275	1.173
0.772	2.753		0.894	
1.032	1.004	1.567	1.465	1.381
0.685	0.698	0.917	0.545	1.048

0.133	0.137	3.259	0.285	0.317
1.125	2.249	1.377	0.81	1.056
0.995	0.631	0.649	1.047	0.892
1.4		0.386		
1.023	0.936	1.713	0.458	1.121
0.599	1.268	0.616	0.908	1.075
1.241	1.308	0.723	1.014	1.102
0.389	0.797	0.109	0.723	1.716
0.705	0.657	2.132	0.588	
1.69	1.104	1.052	0.974	1.088
0.593	1.553	1.478	1.834	1.036
1.3	0.823	1.242	0.127	0.912
1.694	1.231	1.192	0.715	0.896
1.328	0.989	1.039	1.293	1.766
1.12	1.218	1.437	0.239	1.061
1.107	0.816		0.609	
0.769	0.363	3.278	0.577	0.804
0.666	1.104	1.015	1	1.133
1.002	0.44	1.232	1.384	1.331
2.162	1.737	0.838	0.509	0.398
0.002	0.014	1.353	1.292	1.682
0.078	1.379	0.656	0.44	1.237
0.775	1.168	0.689	0.852	1.107
1.25		1.037		0.973
1.246	0.971	0.896	0.8	0.707
0.882	1.11	1.287	0.868	0.945
1.447	1.364	1.372	0.543	0.67
0.574	0.7	2.076	0.973	0.599
1.172	0.848	0.688	1.563	0.909
1.146	1.11	0.901	1.205	1.023
0.93	1.065	0.568	0.583	0.455
1.446	0.318	2.066	0.279	1.323
0.979	1.346	1.624	1.039	1.002
1.888	1.792	0.978	0.932	1.165
0.354	1.021	1.02	2.164	0.618
1.152	1.319	0.978	0.853	1.333
0.534	1.278	1.525	0.592	0.589
1.362	0.845	1.006	0.981	1.598
0.557	1.974	1.518	0.72	0.96
2.651	0.517	0.597	0.671	1.124
0.823	0.547	0.877	0.548	1.102
0.982	1.592	1.174	0.809	1.174
1.316	0.777	0.278	1.389	1.513
2.163	0.602	0.734	1.474	1.227
0.936	0.307	0.647	1.285	0.533
1.27	0.713	0.952	0.879	0.938

1.171	0.711	0.367	0.853	1.258
0.799	0.483	1.343	1.528	1.121
1.433	0.484	0.431	5.013	1.662
0.945	1.669	1.343	1.332	1.317
	0.979	0.999	1.025	0.055
1.477	1.177	1.262	0.987	0.98
1.085	0.935	1.177	0.927	0.901
0.977	1.025	0.693	1.165	1.121
1.156	1.32	1.204	0.466	0.929
0.834	0.963	0.979	0.907	1.114
1.173	1.408	0.949	1.194	0.843
0.389	0.738	1.565	1.385	1.082
1.06	1.185	0.741	1.418	0.957
1.399				0.41
0.864	0.607	1.354	1.309	0.885
0.872	1.173	0.194	1.034	2.045
1.631	1.021	0.896	1.516	1.089
1.403	1.278	1.365	1.443	1.295
1.044	0.88	1.244	0.925	1.152
0.127	1.3	0.74	0.572	1.972
1.005	1.086	0.934	0.986	1.323
1.135	1.452	0.477	0.588	1.189
3.074	1.015	1.115	0.651	0.746
0.949	1.59	0.99	0.826	0.842
0.979	1.292	0.773	1.044	0.676
1.112	1.205	1.061	1.123	1.261
1.418	0.925	1.48	1.656	1.077
1.334	0.493	0.425		0.736
0.957	1.233	1.336	0.78	1.145
0.928	0.607	0.494		0.861
		0.874		
		0.621	0.959	
1.244	2.279			1.056
1.598	0.566	1.963	1.068	0.861
1.014	0.84	0.842	0.793	1.13
0.664	0.585	1.029	0.99	1.014
0.67	1.595	0.906	0.66	1.769
1.058	1.555	1.438	0.677	0.723
0.848	1.332	1.669	0.979	0.909
0.327	2.48	0.887	1.545	0.365
2.322	0.852	0.745	1.275	1.367
0.826	1.304	1.268	1.08	1.04
0.899	1.316	1.129	1.41	1.198
1.076		0.784	1.053	
1.953	0.783	1.713	1.813	0.385
1.16	0.987	1.054	1.252	0.998
0.603	0.787	0.932	2.612	1.51

0.166	0.331	1.107	0.485	0.864
1.275	1.358	1.275	0.876	1.292
1.574	1.828	0.484	0.354	0.395
3.045		1.587		0.923
1.18	1.116	1.317	0.975	0.949
1.021	0.93	1.441	1.324	1.525
0.64	0.719	0.852	0.573	1.677
1.79	0.711	1.183	0.785	1.188
0.166	0.217	2.366	0.352	0.85
1.548	0.804	0.954	1.077	1.155
0.588	0.858	1.292	0.676	1.025
				1.088
0.808	1.507	0.865	0.845	0.802
		1.424	0.341	
1.327	1.09	1.149	1.332	1.221
0.78	0.902	1.078	1.257	1.285
0.899				
0.611	1.268	0.791	1.752	1.146
1.146	1.76	0.992	0.892	1.422
1.861	1.265	0.88	0.98	1
1.26		0.973	1.62	0.956
	0.997			0.854
0.669	0.845	1.084	1.325	1.097
1.023	1.007	0.893	0.904	0.896
1.32	0.506	1.164	2.274	1.664
0.121	1.651	1.459	1.085	0.48
0.979	0.784	0.811	0.75	0.928
0.855	0.913	0.983	1.873	1.018
0.99	0.753	0.715	1.072	0.788
1.182	0.976	0.866	1.191	1.263
0.455	1.932	1.097	0.928	0.758
1.801	0.991	0.925	1.51	1.782
0.754	0.937	1.754	0.618	1.143
0.427	1.174	1.245	1.172	1.11
1.072	0.82	1.002	1.3	1.119
1.383	0.836	0.746		0.513
3.78	0.243	1.559	0.643	0.446
0.777	1.314	1.153	0.739	1.106
0.964	1.566	0.89	1.383	1.222
0.883	0.952	1.317	0.745	1.451
0.371	0.412	1.283	0.553	0.601
0.756	0.756	1.646	1.121	0.886
0.08	0.443	1.221	0.358	1.628
1.387	0.788	0.828	0.773	1.167
1.092	1.329	0.777	1.625	0.874
	0.515	0.999	1.364	1.073
1.048	1.025	0.786	1.304	0.784

0.848	0.706	1.08	0.99	0.626
		1.733		
	0.424	2.243		
0.934	1.711	0.971	1.286	1.037
1.142	0.302	1.038	1.204	1.184
0.534	0.967	1.387	0.876	0.283
0.909	0.59	0.942	1.039	1.019
0.191	0.924	2.036	0.198	0.254
1.721	1.699	0.952	1.428	1.179
0.733	0.37	0.58	1.892	0.666
3.696				
1.083	1.346	1.151	0.846	0.936
0.911	0.884	1.167	1.14	0.819
1.319	0.812	1.43	0.909	0.813
0.693	0.561	1.926	0.953	0.81
1.008	1.473	1.338	1.096	1.07
0.684	0.393	0.186	0.443	0.837

M_ZYH_7	M_WSB_8	M_FSM_9	M_ZSH_10	A_SYS_1
0.799				
0.65	0.913	1.625	1.263	0.789
1.052	1.412	1.059	1.484	1.387
		0.188		
0.891	0.405	0.526	0.747	0.853
0.706	1.881	0.694	0.907	1.337
1.199	0.725	1.165		0.283
0.951	0.912	0.83	0.85	0.845
0.716	1.262	0.576	0.915	2.028
1.168	0.88	1.031	0.895	1.223
0.898	0.976	0.971	1.183	0.974
0.444	0.498	0.286	0.222	
0.921	0.687	0.765	1.216	1.002
1.26	0.58	0.825	0.48	0.834
1.269	0.926	0.822	1.112	0.728
2.525		0.133		
0.407	0.975			
1.135	1.158	0.987	1.08	1.679
1.182	1.363	0.682	0.741	0.935
0.136	0.247	0.161	2.745	2.75
0.547	0.499	0.293	0.902	0.617
1.734	0.604	0.579	1.226	0.842
1.044	1.027	0.848	1.091	0.892
1.631	0.369	0.54	0.529	1.278
1.142	1.129	1.562	1.072	0.973
0.958	1.168	1.249	1.202	1.088
		0.13		
1.078	1.115	1.059	0.818	0.539
0.923	1.473	0.909	0.94	0.581
	0.921		0.886	1.082
1.269	0.538	1.048	1.267	2.052
	2.397		0.925	
	0.447			
1.472	1.153	1.072	1.017	1.076
1.13	0.612	0.733	0.65	0.822
0.912	0.512	0.882	0.39	1.154
1.303	1.406	0.603	1.26	1.039
				0.028
0.358	0.466	0.633	0.232	0.237
1.138	1.741	1.079	1.028	0.111
1.973	0.867	0.613	0.936	0.925
0.441	0.742	0.926	0.681	0.724
0.935	0.505	1.248	1.239	0.914
0.484		0.954	0.263	0.815

			0.117	
	0.803			
0.805	0.629	0.769	0.496	0.951
0.891	1.211	1.532	1.073	1.201
0.818		1.345	1.498	1.556
1.074	1.574	0.937		0.78
		0.395		
0.605	1.675	3.245		0.058
1.292	1.367	0.763	0.687	1.19
1.592	0.725	1.214	1.197	0.958
0.926	0.791	1.019	1.058	0.836
1.22	0.816	1.061	1.311	0.778
0.983	1.319	0.765	1.258	0.772
0.608	1.675	1.529	0.686	0.551
1.153	1.277	0	0.883	1.034
		0.893		1.016
0.487	0.772	1.334	0.745	1.12
0.491	0.995	1.421	1.054	1.115
0.911	0.835	1.203	0.586	1.461
0.901	1.089	1.013	1.21	1.518
1.295	0.794	1.012	0.862	0.432
1.151	0.944	1.323	0.947	0.446
				0.002
0.361	0.152		0.145	1.284
	1.371	0.376	0.75	1.718
0.976	0.83	1.398	1.022	1.322
1.735	0.631	1.62	1.307	0.97
0.696	0.188	0.856	1.454	0.915
		0.515		
1.387	1.161	1.254	1.023	1.057
1.315	0.988	0.803	1.508	0.907
0.4	0.418	0.199	0.507	0.456
0.754	0.671	0.1		0.613
0.462	6.965	0.259	0.522	0.696
1.315	0.651	0.798	0.639	0.889
1.691	0.138	0.287	0.119	0.657
0.092	0.072	1.093	0.084	0.114
1.025	1.424	0.835	1.193	1.158
0.316	0.326	1.604	0.276	0.351
	0.573	1.658		
4.736	0.084	1.602	0.144	0.634
1.161	1.098	1.207	1.107	0.713
0.684	1.421	0.679	0.951	0.686
0.594	0.73	0.811	0.832	1.034
1.084	1.236	0.813	1.181	0.87
0.269		0.74	1.007	0.754

1.114		0.932	1.706	1.131
1.102	1.007	1.477	1.49	1.275
	0.391	0.957	0.352	1.62
1.243	0.973	1	0.759	0.881
1.016	0.819	0.834	0.697	0.636
0.617	0.544	0.668	0.611	1.227
0.898	1.282	1.536	0.795	0.603
1.034	1	1.061	0.9	0.883
1.057	1.016	0.914	0.957	1.203
0.348		1.216		0.802
1.007	2.136	2.066	0.662	1.042
1.384	1.132	1.126	1.213	1.251
0.983	1.018	1.252	0.687	0.537
0.47	1.107	1.036	0.965	2.997
1.106	1.141	1.251	1.068	1.165
0.859	0.618	0.768	0.968	1.059
1.089	0.924	0.898	0.883	0.848
0.9		0.885	0.887	1.672
0.791			0.63	0.501
0.981	1.06	0.643	0.897	1.078
0.892	1.514	1.01	1.872	0.954
1.255	0.368	0.471	0.44	1.122
1.126	1.165	1.012	1.32	1.105
2.204	0.414	0.801	0.892	1.87
0.913	1.139	1.003	0.951	0.686
1.147		1.025		0.616
1.014			1.089	0.972
		0.393		0.585
1.453		0.137	0.278	0.191
1.145	1.264	0.839	0.86	1.594
0.531	1.247	0.991	0.428	0.82
0.9	1.046	0.501	0.189	2.08
0.527	0.531	0.806	0.691	0.851
0.7	0.97	1.254	1.039	1.326
1.229	0.542	0.771	1.391	0.684
	0.803	1.091	1.474	0.943
1.68			1.029	
0.866	0.632	0.539	0.741	0.953
1.153	0.878		0.702	
1.111	1.179	1.09	0.915	0.813
0.58	1.392	1.282	1.027	0.904
0.902	1.188	1.135	1.535	0.791
1.261	1.38	0.909	1.191	1.199
0.958	0.502	1.118	2.152	1.371
1.042	0.772	0.699	1.268	1.053

				0.221
1.635	0.358	0.843	0.657	1.232
	0.964	0.77	0.997	1.065
1.784	1.053	0.634	1.054	0.492
0.853				
0.893	0.97		0.711	1.261
1.104	1.281	1.06	0.816	1.431
1.067	1.168	1.432	1.321	1.376
		1.117	0.978	0.616
1.128	1.253	1.178	1.215	0.835
		0.624		0.53
1.105	1.244	0.873	1.108	0.886
				2.604
0.958	1.168	1.149	0.938	0.759
1.011	1.309	1	1.021	1.19
0.619	1.11	1.052	0.65	1.267
	1.337	1.769	0.921	1.358
0.617	1.102	1.129	1.859	1.29
0.758	1.057	0.981	1.073	1.105
		0.546	0.846	1.898
1.058	1.591	0.492	0.615	0.543
1.033	1.799	0.752	1.051	0.817
1.093	1.203	0.996	1.368	1.194
1.205	1.295	1.181	0.943	0.922
0.658	1.164	0.73	1.546	0.836
1.222	1.038	1.087	1.24	0.942
1.109	1.16	1.095	1.413	1.488
	0.638	0.556		0.748
0.962	1.028	0.851	1.304	0.766
0.913	1.203	1.387	1.019	1.107
0.351	0.778	0.962	1.174	0.789
0.304	1.525	1.549	0.615	0.616
1.251	1.222	1.254	1.505	1.502
1.589	1.353	1.753	0.808	0.81
1.02	0.732	0.658	0.736	1.535
0.465	1.234	1.18	0.381	0.609
1.092	0.969	0.937	1.074	0.879
0.937	0.421	0.663	0.912	1.116
1.047	0.949	0.987	1.217	1.201
0.974	1.258	0.771	1.457	1.553
0.96	1.061	1.059	0.941	0.893
		0.831	0.571	1.262
0.568	1.042	1.587	0.615	0.901
1.066	0.855	0.806	1.023	1.396
1.225	0.86	1.169	1.169	0.737
1.449	0.914	1.016	1.506	1.658
0.923	0.96	1.155	1.343	1.549
1.336	1.179	0.986	1.6	0.799
0.705	1.303	1.158	0.889	1.19
0.955	0.824	1.043	1.149	1.232

1.014	1.218	0.833	0.919	2.58
0.862	0.213	1.344	0.828	1.396
0.945	1.142	1.101	1.03	0.766
0.373	0.482	1.66	1.267	2.569
	0.538	0.608	0.641	0.689
1.139	0.585	0.929	0.915	1.255
0.805	1.057	1.459	1.191	1.365
0.647	0.253	0.942	1.351	1.265
0.422	0.455	0.441	0.396	0.439
1.068	0.84	0.934	1.298	1.246
0.662	1.063	1.292	0.902	0.699
				0.69
1.179	0.705	0.486	0.831	1.472
		0.469		
2.66	0.944	0.28	3.214	0.708
1.041	1.252	1.041	0.622	1.606
0.794	1.676	0.162	1.636	1.797
0.193	0.2	0.179	0.144	0.762
0.625				0.781
1.089	0.969	1.187	1.249	1.351
1.257	0.293	1.494	2.928	0.388
1.158	0.677	0.841	1.588	1.141
1.058	1.103	1.425	1.151	1.157
0.618	0.587	0.611	0.606	1.351
0.883	1.095	1.319	1.05	0.915
1.036	0.396	1.051	1.282	1.585
0.799	0.878	1.952	1.051	1.351
1.194	1.015	1.027	1.229	0.765
1.217	0.277	0.567	1.935	0.461
0.998	0.839	0.915	1.309	1.323
2.39		0.084		1.585
0.853	1.066	1.02	0.854	1.178
1.851				
	0.725	1.168	1.623	0.749
0.785	0.845	0.948	0.765	2.377
1.049	0.729	0.856	1.138	1.452
1.407	0.573	0.504	1.012	1.045
0.718	0.868	0.909	1.247	1.09
1.207	0.702	1.116	0.785	2.662
1.177	1.003	0.745	0.72	0.985
1.121	0.423	2.38	1.567	1.693
1.042	1.131	1.586	1.148	0.769
1.316	1.106	1.011	0.935	0.73
0.66	0.875	0.721	0.721	1.801
0.847	0.521	0.797	2.563	3.297
			0.694	0.389
1.1	0.979	1.021	1.091	1.244
				0.386

0.895	1.184	1.079	0.834	0.609
0.755	1.252	0.62	1.67	0.872
0.678	0.866	1.092	0.856	0.637
0.877	0.804	0.688	0.677	0.943
0.769			1.003	1.631
0.971	0.7	1.779	0.522	0.691
1.468	0.893	1.187	1.377	1.03
1.292	0.63	0.838	0.516	0.605
0.58	0.848	1.067	1.462	0.682
1.108	1.481	1.325	1.13	0.794
0.876	0.354	0.282	0.442	0.369
0.236	0.239	0.219	1.65	0.3
	0.442	0.505	0.858	1.301
1.204	0.817	1.525	1.442	1.221
				0.594
0.164	3.51	2.444	0.078	0.799
0.959	1.17	0.976	0.91	1.462
	1.331	1.043	1.455	1.269
0.804	0.73	0.507	0.652	0.605
1.298	0.852	1.37	1.136	1.191
0.787	1.15	1.183	1.361	1.314
0.345		3.597	0.396	0.488
1.417		1.306	1.024	0.653
0.924	0.941	0.427	1.005	1.615
1.1	1.07	0.924	1.234	1.271
1.741	1.02	1.663	1.243	1.294
	1.301	0.843	1.432	1.759
			2.011	
1.47	0.812	0.883	0.923	0.676
0.461			0.551	1.383
1.072	1.402	1.124	0.982	0.654
0.994	1.028	0.81	0.888	0.918
1.144	1.072	1.244	1.108	1.123
0.404	0.564	0.458	0.948	1.098
0.069	0.127		0.221	4.99
0.196	0.106	0.078	0.08	
0.867	1.011	0.982	1.114	1.075
				1.319
1.455	0.735	0.834	0.683	0.687
1.163	1.165	1.088	1.217	0.908
1.174	1.179	1.128	0.999	1.073
0.748	1.192	0.604	0.696	1.263
0.977	0.739	1.304	1.157	1.545
0.623	1.185	0.413	1.058	1.965
2.391	1.486	1.629	1.445	2.083
0.91	0.922	1.383	1.036	1.498
0.932	0.968	0.554	0.863	0.643
0.863	0.739	0.421	0.959	0.976
0.835		1.227		1.031

0.981	1.066	1.187	1.022	1.779
1.299	0.905	0.916	1.157	0.712
0.351	0.433	0.505	1.151	1.188
2.009	0.967	1.191	1.264	0.648
0.603	0.892	3.956	0.785	0.92
0.97	1.04	1.07	1.518	1.655
0.271	0.276	0.315	0.452	0.651
1.247	0.852	0.833	1.101	1.337
0.747	1.555	1.519	0.897	1.029
1.479	4.43	0.7		0.718
1.168	1.1	1.251	1.24	0.93
0.932	1.138	1.099	1.04	1.267
0.778	0.968	0.679	1.555	1.151
		1.161	0.117	1.737
	0.458	0.345	0.351	
0.466	0.66	0.734	0.113	1.454
0.777	1.448	0.884	0.678	1.978
0.813	1.07	1.34	1.029	1.142
1.042	0.921	1.129	1.514	1.679
0.59	0.622	0.33	0.654	0.688
	0.769	1.248		0.75
1.101	1.127	1.101	0.875	1.276
1.221	0.954	1.352	1.206	0.763
1.041	0.782	0.649	1.058	1.076
1.019	1.291	1.589	0.988	1.601
0.896	1.591	0.821	0.674	0.251
0.926	1.067	1.114	1.372	1.441
0.952	0.897	1.07		1.115
0.856	0.914	0.878	1.04	1.373
0.792	1.258	1.426	1.295	1.115
1.162	0.863	1.147	1.355	1.32
0.625	1.054	1.179	0.7	1.092
1.172	1.339	1.042	1.082	0.713
1.354	1.077	1.013	0.975	1.094
1.38	0.498	0.216	0.195	0.564
	0.484	0.43	0.468	0.727
0.357		0.78		
				0.332
1.053	0.885	1.073	0.897	1.493
0.391	0.877	0.527	1.156	0.929
0.982	0.873	0.622	1.376	0.839
0.771	1.011	1.418	1.265	1.328
1.032	0.436	0.595	0.292	0.636
0.35	0.698	0.962	0.661	1.023
	0.567			
0.925	0.912	0.575	0.917	0.703
				1.05

0.802	1.104	0.729	0.905	1.266
1.384	0.565	0.508	0.571	0.364
1.181	1.37	1.031	0.683	1.164
0.551	1.942		0.847	
0.869	1.426	1.201	0.725	0.578
	0.203		0.204	
0.372	1.025	1.112	1.208	1.019
0.507	1.632	1.321	0.768	1.009
4.316	0.307	0.491	0.362	0.385
0.699	0.793	2.197	1.2	1.294
1.187	0.694	1.238	1.218	1.054
0.514	0.867	1.423	0.811	0.946
0.937	1.019	0.279	0.871	0.646
1.212	1.476	1.045	1.019	0.865
0.42	0.69	0.84	1.493	1.292
1.264	1.321	1.058	1.283	1.341
1.078	1.248	1.103	0.873	0.86
0.492	0.45	0.223	0.689	0.482
1.166	1.282	1.232	1.278	1.206
0.655	0.561	0.959	1.74	1.917
0.395	0.606	1.274	0.147	1.346
1.101	0.59	0.745	0.817	0.529
0.838	1.475	0.929	0.715	0.953
0.416	0.836	1.162	1.036	0.852
0.586	0.701	0.578	0.67	0.802
0.376	1.377	1.307	0.634	
0.719	0.542	0.846	1.219	1.138
0.937	0.772	1.326	1.525	1.115
0.853	0.261	0.351	2.095	1.857
	0.269	0.55	0.308	
1.361	0.418	0.485	0.307	0.697
0.982	1.077	1.456	1.789	1.177
1.586	1.277	3.913	0.811	0.776
0.91	1.301	1.359	1.041	1.141
0.104	1.007	0.801	0.679	1.637
1.654	0.71	1.037	0.771	1.014
1.035	0.958	1.028	1.074	1.111
1.156	0.811	1.585	1.581	0.755
0.778	1.514	1.307	0.799	1.117
	0.698	0.641		0.872
0.113		0.156	0.217	0.638
0.255	0.236	0.633	0.288	0.219
0.829	0.796	1.621	1.004	1.612
1.086	1.07	0.947	0.984	1.001
0.948	1.075	0.474	1.14	1.946
0.558	1.023	0.709	2.495	0.408
1.394	1.067	0.821	0.988	0.692

1.521	1.339	1.465	1.222	0.957
1.016	1.169	1.189	0.809	1.107
	0.941		0.576	
1.331	1.281	1.558	1.189	1.136
1.03	1.018	1.138	1.473	1.722
0.791				0.857
1.214	0.844	1.021	1.081	1.56
	0.595			0.687
0.976	1.194	1.269	0.671	0.861
0.928	0.93	0.809	1.167	1.117
0.787	1.512	1.195	0.943	0.617
0.769	0.889	1.033	1.483	1.387
1.095	1.175	1.052	1.344	1.359
	0.511	1.217	0.839	0.937
1.102	1.22	1.075	0.982	1.201
0.881	0.926	0.908	1.19	0.986
1.061	1.041	0.834	1.052	0.886
0.543				0.765
1.376	0.981	0.859	0.931	1.157
0.762	0.789	0.948	1.334	1.48
0.065	0.456	0.077	0.157	7.381
0.119	0.34	2.852	0.483	0.523
0.701	1.007	1.307	1.36	1.597
0.935	1.188	1.05	1.268	1.327
1.06	1.243	1.321	1.236	1.195
1.124	1.431	1.268	1.306	0.43
1.288	1.331	0.961	1.337	1.257
1.091	0.662	0.434	1.214	1.604
0.743	1.599	1.213	1.128	0.962
1.082	1.229	0.893	1.117	1.301
				0.323
1.115	1.094	0.858	1.148	1.201
0.89	0.421	0.652	1.09	1.33
	0.738	0.611	0.359	1.473
1.29	1.343	1.528	0.873	1.055
1.343	0.799	1.066	1.071	1.435
		0.892	1.18	1.217
1.167	0.299	0.273	0.188	0.553
0.724	1.137	1.052	1.259	1.021
0.85	1.288	1.03	0.788	1.212
0.919	1.214	0.773	0.66	0.902
0.846	0.854	1.876	1.303	0.909
1.395	0.646	0.877	0.554	1.431
1.669	0.422	1.47	0.384	2.628
2.447	0.268	0.442	0.201	
1.866			0.162	

1.023	1.051	1.212	1.255	1.944
0.791	0.972	1.202	1.049	0.989
2.198	0.554	1.897	0.958	1.293
1.08	1.338	1.16	1.131	0.799
0.676		1.784	0.885	0.813
1.527	1.155	1.499	1.516	1.298
1.059	0.897	0.979	0.992	1.377
1.186	1.667	1.822	1.639	
0.381		3.062		
0.938	0.593	1.033	1.16	0.19
0.259			0.197	0.239
0.616				1.378
1.332	1.007	0.983	0.953	1.698
1.229		0.801		0.939
1.622	0.658	0.146	0.164	1.008
0.92	0.769	0.657	0.804	0.873
	1.46	1.182	1.089	2.199
1.024	1.271	1.179	1.633	0.946
0.666	1.334	1.291	0.607	0.507
0.765	1.024	0.677	0.633	1.027
1.035	1.103	0.509	0.861	0.934
0.925	1.071	1.052	2.138	0.723
	1.265	0.815	0.466	0.512
0.964	1.256	1.101	1.182	1.392
1.102	1.149	0.895	0.939	1.381
0.772	1.155	1.421	1.534	1.683
1.077	0.858	0.376	1.224	1.369
1.207	0.473	0.402	0.924	1.148
1.081	1.106	1.155	1.298	1.144
0.952	0.958	0.918	1.742	1
1.105	1.068	0.809	1.05	0.824
0.646	1.183	1.194	0.738	1.095
0.706	0.353	0.543	0.896	0.877
0.957	1.179	1.156	1.199	1.836
8.776	0.347	7.562	0.731	0.854
0.881	0.832	1.121	0.751	0.673
0.802	1.068	1.42	0.899	0.387
0.872		1.005	0.905	1.53
1.093	1.246	1.802	1.216	0.942
				2.4
0.543	0.734	0.317	0.919	2.978
0.466	0.94	1.102	1.305	1.293
0.654	0.824	1.089	1.058	1.049
	1.051	0.809		0.702
1.324	1.266	1.225	1.225	1.405
1.568	1.028	0.916	0.979	1.45

0.982	0.844	0.673	0.487	0.957
0.921	1.097	0.747	0.57	1.649
1.189	1.28	1.181	0.965	0.946
1.296	0.742	0.608	0.861	0.914
0.713	0.574	0.907	0.283	
1.385	1.255	1.209	0.626	0.934
0.6	1.589		0.533	
			0.234	
	0.14	0.092	0.081	
0.8		0.829		
1.597	1.375	0.91	1.011	0.794
0.55	0.643	0.594	0.821	0.896
1.094	1.07	0.985	0.996	0.992
1.381	1.069	0.993	1.494	1.324
	0.771	0.785	0.851	0.917
0.957	1.494	0.415	2.201	0.857
1.035	1.455	1.084	1.146	0.713
0.936	0.419		0.747	2.036
0.674	1.109	1.203	0.782	1.029
0.976	0.956	1.099	1.335	1.344
0.986	1.266	0.968	0.967	0.96
1.889	1.121	0.789	2.582	0.875
0.644	0.48	0.786	0.773	0.988
0.45	0.251	0.467	0.629	0.586
0.706	1.104	0.884	1.02	0.994
0.727	0.767	1.035	0.914	1.146
0.559	1.206	0.461	1.36	0.622
1.099	0.794	1.221	0.819	1.627
0.877	0.92	1.193	1.027	1.219
		0.808		
1.097	0.986	1.274	0.816	1.293
0.958	0.826	0.967	0.788	0.925
1.468	0.507	0.841	0.486	1.29
1.444	1.12	0.933	1.726	0.875
1.14	0.924	1.482	1.63	1.532
0.877	1.112	1.026	1.416	0.689
1.361	1.356	1.516	0.76	1.045
0.916	0.448	0.611	0.814	0.799
0.567	0.865	0.967	4.277	0.976
0.444	1.64	0.932	0.874	0.519
0.647	0.312	0.533	1.146	0.727
1.045	0.915	0.844	1.29	1.092
0.982	0.784	1.196	0.612	1.283
1.104	0.88		1.258	0.689
1.002	1.13	1.271	1.283	1.161
1.413	0.755	0.564	1.505	0.461

0.28		0.276	0.811	1.129
1.417	0.873	1.133	1.379	1.358
0.75	0.768	0.96	0.525	1.234
			0.765	
1.935	0.499	2.287	0.577	1.489
0.977	1.073	0.704	0.688	0.994
1.492	1.366	0.98	1.337	1.334
1.391		0.94	1.292	0.843
0.933	1.07	1.224	1.113	1.264
0.463	0.723	0.937		1.191
1.258	0.968	1.141	1.458	1.964
0.519	1.085	1.391	0.594	1.035
1.055	0.357	0.667	0.227	1.312
1.048	0.896	1.198	1.074	1.212
0.362	0.468	0.497	3.263	3.315
0.919	1.002	1.023	1.307	0.938
	0.365	0.471	0.529	0.691
		0.38	0.281	0.296
1.315	0.758	0.851	0.782	1.207
0.687	0.759	1.007	0.076	2.136
1.408			1.216	
1.861	1.695	0.005	0.005	0.008
0.874	1.338	0.661	1.295	1.68
0.947	0.71	0.835	0.617	1.367
1.033	0.97	1.111	1.209	0.77
				0.751
1.262	0.613	0.525	0.901	0.647
1.103	0.694	0.889	1.581	0.968
0.985	0.893	1.314	1.158	1.398
0.75	1.346	1.079	0.911	1.65
1.088	0.613	1.299	0.742	0.929
1.672	0.535	2.024	0.953	1.139
				1.187
0.808	1.067	1.054	1.352	0.951
0.781	1.364	1.025	1.089	1.678
1.295	1.052	0.781	1.187	0.843
1.368	0.704	0.965	1.048	0.911
0.304	0.289	0.284	0.354	0.471
	1.878	1.101	1.21	0.783
1.206	1.007	1.403	1.338	1.065
1.176				1.158
1.066	1.864	0.828	1.348	1.676
1.074	0.896	0.86	1.052	0.901
0.683	0.422	0.298	2.906	0.324
		1.009		
0.88	1.164	0.961	1.065	1.567
0.659	1.094	1.117	0.791	0.605
1.269	0.299	0.66	0.529	1.213
1.054	1.2	1.129	0.835	0.735

1.028	0.933	0.89	0.564	0.651
1.397	1.104	1.205	1.187	0.877
0.861	1.432	1.125	1.006	1.11
0.856	1.643	1.452	0.887	0.683
0.899	1.061	1.642	1.687	1.096
1.424	1.306	1.077	1.074	1.04
1.005	1.017	0.761	0.856	1.12
	1.677	1.188	1.38	
0.997				0.732
0.865	0.623	0.985	0.818	1.081
1.236	0.95	1.095	1.243	0.869
1.381		2.991	0.725	0.263
0.405	0.444			
0.595	0.747	0.421	0.246	0.238
1.056	0.921	1.232	1.257	1.097
0.45	0.816	0.567	0.448	0.561
	1.146		0.807	0.693
1.431	0.726		1.023	0.814
1.277	1.3	1.386	1.036	1.203
0.321	1.24	0.93	1.026	0.829
1.041	1.019	1.133	1.203	0.963
		0.761	0.539	0.667
1.026	0.928	1.089	1.21	0.973
1.07	0.761	1.192	1.601	1.482
1.202	0.833	1.443		0.956
1.472	1.123	0.886	0.851	0.681
1.019	0.764	1.116	0.827	1.583
1.075	1.261	0.773	1.83	0.432
0.572	1.069	1.787	0.539	2.242
1.319	0.946	1.4	0.966	1.399
0.853	1.209	0.966	0.718	0.899
1.41	0.43	0.779	0.771	3.226
		1.025	0.427	1.232
0.879	0.768	0.866	0.687	1.125
2.39			0.462	0.414
	0.173			0.743
0.925		0.83	0.556	1.322
1.028	1.196	1.143	1.003	1.248
0.92	0.982	1.1	0.977	1.162
0.885	0.83	0.607	0.678	1
1.191	1.069	1.276	1.562	1.83
2.228	1.15	0.91	0.1	0.931
1.89	0.575		0.585	0.62
1.622	2.224	0.101	2.127	2.437
0.781	1.161	0.819	0.752	0.514
0.876	1.32	1.045	0.989	1.146
2.424	0.955	1.141	0.668	0.746

0.439	1.543	0.94	1.183	0.978
	0.87	1.05		
0.29	1.05	1.542	1.006	0.567
	1.186	1.094	1.301	1.216
1.031	0.735	0.783	0.99	0.778
1.249	1.202	1.044	1.321	1.51
0.719	0.566		0.752	1.108
1.12	1.681	1.278	1.261	0.456
1.646	1.044	1.026	1.032	0.966
			1.01	
	1.046	1.172		
0.332	0.795	0.558	0.811	0.863
	0.645		0.875	
1.618	1.045	0.648	0.318	0.95
0.754	1.309	1.017	1.452	1.107
1.252	0.652	0.636	0.12	1.432
0.7	1.072	1.012		1.051
1.335	1.073	1.37	1.352	0.987
1.024	1.465	0.854	0.964	0.786
0.788	0.24	0.268	0.367	0.429
0.187	0.26	0.596	0.558	
0.59	0.713	0.837	0.738	1.715
0.725	0.676	1.153	0.971	1.847
				0.87
0.867	1.067	1.085	1.318	1.155
0.708	0.667	1.455	0.69	0.803
1.376	1.01		0.987	1.32
0.911	1.139	1.085	0.825	1.051
0.922	1.196	1.483	1.723	1.453
0.627	0.194			
				0.741
				0.981
0.266	0.481	1.488	0.865	0.537
0.717	1.335	0.758	0.844	0.199
0.863	1.329	1.577	0.721	1.926
1.365		0.741	0.69	1.18
1.33	0.773	0.832	1.034	1.261
0.557				0.51
0.776	0.791	1.881	1.238	1.318
1.093	0.41	0.329	2.288	3.202
0.657		1.101	1.303	1.295
1.431	0.574	0.769	0.641	0.73
1.006	0.334	1.02	0.52	1.129
0.79				1.244

0.498	0.445	1.291	0.89	1.184
0.922	1.218	1.2	1.271	1.185
1.22	1.626	0.624	1.104	1.299
0.98	1.083	0.23	1.478	0.895
1.413	0.753	1.057	0.834	0.931
1.301	0.901	1	1.035	1.26
1.447	1.231	0.577	1.205	0.917
0.717	0.768	0.671	0.47	0.638
1.124	0.815	0.565	1.091	0.884
1.214	1.187	1.097	0.917	1.052
0.894	1.841	1.543	1.136	1.197
1.618	0.551	0.491	0.694	0.737
	0.856	0.947		1.216
0.976	1.285	0.963	1.039	1.088
0.75	1.37	1.432	2.216	1.054
1.365		0.718	0.656	0.806
	1.112			
0.716	0.978	0.615	1.634	1.358
0.976	1.247	1.243	1.46	1.009
1.418	0.942	1.122	1.613	0.793
1.105	0.639	0.895	1.102	1.057
0.807	1.262	1.227	0.516	0.906
0.931	1.115	1	1.003	1.264
1.161	0.737	1.142	1.021	1.212
0.906	0.895	0.787	0.496	1.054
0.798	1.043	0.937	0.988	1.055
0.508	1.259	0.619	0.82	2.108
			0.334	
1.802	0.846	1.631	0.542	1.302
		1.025		
0.832	0.879	1.38	1.495	0.567
0.383		1.68	0.819	0.865
1.093	1.139	1.284	1.101	0.824
1.287	0.887	0.69	1.205	1.079
0.916	2.115	1.232	1.274	1.426
0.801		1.164	1.161	1.358
0.795	1.007	1.018	0.9	1.032
0.54	0.706	0.563	0.875	0.958
0.753	1.574	1.148	1.113	0.831
				1.133
0.787	0.812	0.676	1.89	0.707
			0.323	0.182
			1.276	
1.506	0.35	0.559	0.434	0.871

0.543			0.77	1.073
	0.183	0.332	0.401	0.37
1.23	1.21	1.333	1.486	0.472
1.052	1.015	1.033	1.232	0.79
0.924	0.737	0.59	1.001	0.53
1.053	1.268	1.339	1.168	1.297
1.512	0.767	0.764	0.904	0.889
		0.772	0.961	0.762
	0.412			
	1.089	1.083	0.82	0.907
0.499	1.463	3.767	0.445	0.606
		1.216	0.835	1.111
0.255	3.02	1.355	0.261	2.455
		1.4	0.738	1.379
1.251	1.386	1.534	1.535	0.908
0.967	1.08	1.079	1.045	1.276
1.224	0.846	0.942	1.046	0.962
	1.125		0.761	1.026
1.426	1.21	0.859	1.548	0.866
0.504	1.007	2.012	0.864	1.436
0.415	0.172	0.249	1.44	1.128
1.527	0.846	1.015	1.022	1.342
1.058	1.154	1.019	1.084	0.897
	1.41	0.911		0.749
1.444	1.129	1.726	1.432	1.619
	1.178	1.363	0.69	
1.29	1.073	1.136	0.58	1.305
1.66	1.056	0.278	0.452	1.436
1.025	0.72	1.067	1.493	1.397
0.088	1.87	0.284	0.266	0.303
0.854	1.756	0.653	1.55	1.273
0.745	2.07	1.114	1.012	0.945
				0.427
1.236	1.003	0.883	1.101	1.224
0.558				
1.721	1.003		0.87	0.706
1.262	0.705	0.476	0.452	0.964
0.205		0.327	0.291	1.452
1.165	1.131	1.291	1.355	1.382
	0.749	0.869	1.146	1.455
1.2	1.165	1.511	1.626	0.691
1.806	0.341	0.691	0.408	0.395
0.934		0.403		1.098
0.71	0.943	0.935	0.667	0.693
0.793	0.96	0.801	0.844	0.916

1.024	1.079	0.998	1.368	1.685
1.845	0.431	1.438	0.915	0.938
		0.639		
				0.829
1.222	0.993	0.907	0.904	1.015
			0.764	
0.981	1.246	0.89	0.947	0.803
			0.683	0.907
2.061	0.23	0.657	0.503	
1.375	0.874	1.001	1.048	0.702
	0.057	0.731	0.975	
0.661	0.224	0.276	0.331	0.449
0.683	0.275	0.422	0.419	1.026
0.822	0.732	1.519	1.261	1.258
0.761	1.143	0.702	0.533	0.484
0.944	0.783	0.888	0.737	0.958
	0.401	0.896	1.957	1.05
1.563	1.123	0.805	0.86	1.175
0.313	0.568	0.529	1.293	0.319
0.217	0.252	0.062	0.171	0.092
1.04		0.717		1.616
1.818		0.031	1.278	1.294
1.384	0.123	1.751	1.1	1.074
1.099	1.089	1.13	1.02	0.885
0.838	0.865	0.878	1.305	0.691
1.31	1.361	1.293	1.512	1.507
1.014	1.594	0.921	0.882	1.334
1.167	0.883	1.056	1.503	1.201
1.822	0.33	0.434	0.354	0.584
0.495	1.098	1.713	0.613	0.934
0.318	1.397	0.869	0.989	1.298
0.822	0.852			1.086
1.017	0.807	1.623	1.337	1.353
0.829	1.131	1.582	1.327	1.078
0.477	0.465	0.607		1.124
1.754	0.384	0.752	1.05	0.559
1.221	1.239	0.654	0.976	0.569
0.524		0.018	0.837	0.01
0.531	0.911	0.899	1.044	1.232
			0.899	1.672
				0.823
1.401	1.127	1.635	1.268	1.624
1.073	0.913	1.347	0.989	1.518
0.665	1.309	0.816	0.499	1.238
1.473	1.332	0.92	1.012	1.417

1.259	1.053	1.443	0.897	1.107
1.253	1.033	1.27	1.671	1.041
1.225	0.819	0.858	0.793	0.995
1.312	0.57	0.708	0.584	0.972
1.046	0.874	1.576	1.106	0.93
	0.939	0.972		
		0.332	0.884	1.141
0.679	0.715	1.226	1.015	1.321
0.957	0.918	1.008	1.469	1.573
0.968	0.626	1.644	0.682	0.625
1.344	0.863	0.902	0.999	0.859
0.639	0.227	0.509	0.397	0.663
0.729	1.44	1.296	0.766	1.082
1.603	0.399	1.776	1.093	1.004
1.127	1.185	1.81	0.776	1.897
1.36	1.004	2.562		1.352
0.437				0.171
1.127	1.461	1.151	1.4	1.439
1.276	1.066	1.121	1.125	1.13
0.879	1.154	0.705	0.927	2.017
				0.937
0.789	0.794	1.094	1.093	1.346
1.542	0.691	1.93	1.144	1.153
	1.495	0.904		
	0.6	1.106		4.088
1.003	0.537	0.873	1.813	0.632
0.805	0.727	1.119	0.872	1.204
1.115	1.162	1.069	1.119	1.002
0.643	2.168	2.771	0.221	2.038
1.113	0.904	2.368	0.94	1.607
0.879	1.893	0.703	0.059	1.999
0.927	0.865	1.397	0.828	1.895
1.595	0.759	1.666	0.757	0.818
0.859	1.481	1.292	1.406	1.815
			1.309	1.414
0.449	0.219	0.156	0.145	0.371
0.836	1.369	0.873	2.02	0.918
0.828	1.015	1.449	1.053	0.995
1.147	1.142	1.585	0.963	1.304
1.052	1.025	1.384	0.82	0.572
1.001	1.443	1.573	0.88	1.32
1.436	0.546	0.56	0.628	1.342
1.508	1.602		0.328	1.119
0.902	1.109	1.045	0.816	1.432
1.968	0.669	0.789	0.915	0.656

1.048	0.796	0.811	1.473	1.736
1.454	0.764	0.671	1.48	0.811
1.263	0.929	1.805	0.898	1.151
1.128	0.582	1.443	0.903	2.224
	1.278			
			0.93	1.388
1.157	0.997	0.472	0.937	1.697
1.255	0.428	0.511	0.565	0.712
1.057	0.924	1.377	0.784	2.877
		1.391	2.084	1.52
0.654	1.132	1.103	1.14	1.314
0.867	1.038	0.947	0.75	1.29
1.115	0.843	0.853	0.945	0.726
0.787	1.059	1.09	0.81	1.028
0.738	1.116	1.567	1.432	1.667
0.776	1.021	1.308	1.298	0.643
1.27	1.365	1.102	0.651	0.543
1.002	1.394	1.195	1.118	1.11
1.202	0.806	1.505	1.427	1.213
1.5	0.692	1.165	0.735	1.444
1.746	1.3	1.358	1.603	1.086
0.764	1.117	1.065	0.413	1.135
1.292	1.161	1.111	1.321	0.859
	0.594	0.668	0.85	1.784
0.864	1.487	0.72	1.031	0.567
1.095	0.156	0.219	0.313	0.5
0.848	1.154	1.036	0.963	1.082
1.025	0.919	0.861	1.404	1.057
1.39	0.802	1.196	0.823	0.793
0.929	0.662	0.655	0.617	0.584
1.257	0.853	1.371	1.224	1.92
1.056	0.935	0.915	0.809	0.941
	0.432		0.369	0.209
0.885	1.223	1.092	1.149	0.614
1.156	0.981	1.311	0.783	1.452
0.563	1.331	0.58	0.599	1.656
1.421	0.661	1.06	0.523	0.621
1.172	1.074	1.053	1.081	1.337
	0.327			
0.761	1.471	0.721		0.865
0.826	1.164	1.522	1.35	1.058
1.463	1.171	1.306	1.443	1.375
		1.455		1.115
0.032		0.122		0.706
1.36	0.72	0.323	0.437	1.016
0.971	0.96	1.168	1.087	0.992
0.943	1.127	1.141	0.998	1.423

0.73	1.052	0.819	1.219	0.793
0.969	0.821	1.003	1.279	1.084
1.231	1.173	1.17	1.188	0.966
1.568	0.757	1.264	0.955	1.613
0.721	1.263	1.074	0.7	0.841
0.488			1.364	1.987
0.892	0.991	0.959	0.979	1.331
0.899	0.777	0.573	0.279	1.279
0.629	1.362	1.324	1.608	1.898
0.623	1.091	1.97	0.621	1.604
1.517	0.924	0.67	1.241	1.039
0.442	0.181	0.221	1.761	0.807
0.81	0.958	1.207	0.78	1.015
1.192	2.049	1.082	1.099	1.801
0.906	0.679	1.937	1.121	1.085
0.8	1.227	0.971	0.906	1.269
1.187	0.67	1.076	0.813	1.129
0.374	1.116	1.464	0.994	1.41
0.841	1.699	1.257	0.737	1.722
0.515				1.065
1.789	0.675	0.936	0.284	0.653
0.907	1.334	0.986	1.044	2.185
0.913	0.982	1.1	1.132	1.117
0.733	0.556	0.265	0.682	0.238
1.106	1.278	0.851	1.155	1.036
1.167	0.923	0.615	0.405	1.096
1.611	0.142	0.152	1.331	1.468
0.133	0.343			
0.69	0.866	0.617	0.761	0.576
				1.09
0.971	1.153	1.459	1.225	1.777
0.107	0.075	0.097	0.075	0.689
1.417	0.853	0.83	0.902	1.164
0.762	0.616	2.592	2.226	0.735
0.32	0.423	0.367	0.528	0.582
1.055	1.097	1.107	1.09	1.358
1.339	1.256	1.152	1.355	1.523
0.728	0.377	0.257	0.582	0.731
2.946	1.059	0.802	0.848	0.833
0.895	1.041	1.919	0.717	0.679
0.649	0.612	1.28	1.137	1
1.199	1.306	1.072	1.271	0.878
0.558	0.131	0.962		0.994
				0.865
0.782	1.2	2.11	1.254	1.148
0.922	1.483	1.192	1.262	0.924
0.899				
0.718	0.452	0.654	0.378	0.727
1.573	1.09	0.993	1.373	0.981

1.139	1.134	1.479	0.984	1.155
1.014	1.327	0.91	0.609	0.61
1.111	1.082	0.975	1.105	0.903
0.173	0.272	0.294	0.176	2.754
1.176	1.115	1.09	1.287	1.102
1.185	0.631	1.149	1.23	0.929
2.549	0.194	1.407	1.89	0.282
0.938	1.142	1.065	1.033	1.374
1.046	0.943	0.898	0.995	2.039
0.763		1.051		
0.947	1.264	0.651	1.133	1.351
0.618	0.522	1.157	1.313	1.235
0.503	2.199	0.262	1.66	1.288
1.143	0.619	0.302	2.122	0.601
1.453	1.904	0.103	1.266	1.843
	0.864	0.494	1.144	1.07
0.813	1.646	1.083	1.273	1.828
		0.182		2.765
0.753	1.363	0.498	0.563	1.898
1.193	0.625	0.821	0.934	1.207
0.984	1.156	1.528	0.966	1.14
0.468	0.516	0.678	0.511	0.825
0.639	0.626	0.649	0.643	0.729
1.064	1.111	1.088	1.196	0.865
0.166	4.604	0.475	0.639	0.661
0.966	0.996	1.283	1.333	0.825
0.837	1.116	1.246	1.155	0.939
1.011	0.978	0.974	1.086	0.923
0.218	0.201	0.266	0.22	0.277
			1.679	0.554
1.049	1.406	0.929	0.735	1.155
1.142	0.96	0.822	1.047	0.87
0.224	0.303	0.252	1.366	1.306
0.952	1.011	1.275	0.977	1.231
1.107	0.786	1.289	1.479	1.016
	0.607	0.793	0.873	0.466
0.725				1.096
0.693	0.522	1.977		1.116
0.797	1.28	1.608	1.241	1.474
1.362	1.29	0.672	0.778	0.576
0.951	1.2	1.483	1.477	1.481
0.802	0.045	0.623	0.807	0.944
1.036	0.913	0.855	1.575	1.226
1.181	0.628		0.784	1.963
1	1.023	1.009	1.045	0.941
1.26	1.349	1.129	1.618	0.643
0.864	1.168	1.143	1.624	1.035

1.554	1.282	0.74	3.184	1.262
0.447	1.134	1.093	1.947	2.334
1.17	1.055	1.173	1.49	1.566
0.794	0.906	0.683	0.856	2.623
0.944	0.788	1.104	0.865	0.727
1.026	0.787	1.029	1.16	1.304
1.188	1.161	1.118	0.913	1.128
0.611	1.202	0.534	0.536	1.344
1.059	1.236	1.089	1.062	1.39
0.204	0.409	0.24		0.278
0.806	0.993	0.991	0.882	2.036
2.082	0.932	0.342	2.438	0.617
0.993				0.285
1.183	0.623	0.463	1.244	1.19
0.897	1.499	1.554	1.371	1.061
	0.836	1.869	1.142	
1.412	0.58	1.02	1.19	0.957
1.018	0.351	0.582	0.468	1.202
	1.09	1.008	0.525	
0.972	1.402	1.413	0.972	1.009
0.716	0.355	0.727		0.787
1.195	1.13	1.654	1.778	1.169
1.027	0.739	0.948	1.12	1.401
0.693	0.906	1.184	1.388	1.312
0.866	0.921	1.089	1.158	0.699
0.496	0.663	0.675	0.478	0.429
0.862	0.971	0.666	2.344	1.415
0.342		0.363		0.776
0.553	0.525	0.485	0.574	1.188
0.801		0.684		0.597
1.135		0.967		0.852
1.098	0.872	0.811	1.292	0.799
1.189	0.952	0.999	1.11	0.849
1.104	1.011	1.227	1.211	1.687
1.173	0.642	0.988	0.868	0.871
1.079	0.918	0.978	1.131	1.219
1.185	0.915	1.134	1.412	1.289
1.436	0.987	0.649	0.859	0.809
	1.804			
				0.664
1.003	0.983	1.036	0.963	0.895
0.84	0.805	0.908	0.921	1.461
2.154	2.51	2.668	0.295	0.546
0.754	0.742	0.676	0.755	0.619
1.263	0.8	0.464	0.515	1.294
1	0.724	0.548	0.867	1.127
0.314	0.37	1.095	1.208	0.897
1.027	1.151	1.226	0.865	1.131
1.176	1.084	1.301	1.397	0.963

1.235				0.841
	0.192	0.786	1.145	2.18
1.23	0.876	0.992	1.598	1.221
0.956	1.326	1.03	1.174	1.264
	1.365		1.173	
1.142	0.923	0.962	1.161	0.95
0.833	1.274	1.467	1.576	1.014
1.323	1.033	0.401	1.474	0.574
0.956	1.144	1.054	0.497	0.5
0.848	0.817	1.163	1.116	1.437
0.667				1.025
0.352	1.355	1.391	0.44	1.721
0.328	0.535	0.537	2.424	0.483
0.463	0.549	0.473	0.682	0.775
0.562	1.071	1.383	0.656	1.075
1.011	0.864	0.768	1.52	0.603
0.836	0.817	0.911	0.727	0.677
0.732	1.024	1.092	0.719	1.006
1.19	1.374	1.535	0.866	1.251
0.888	0.618	0.793	0.674	0.609
0.799	1.196	1.172	0.554	1.299
1.252	1.358	1.182	1.061	1.38
	0.589	1.242		1.517
1.024	1.083	1.372	0.837	0.997
0.586	0.581	0.961	0.641	0.933
2.516	1.426	1.173	0.978	0.992
1.066	1.156	1.303	1.033	0.782
0.375	0.593	0.769	0.726	0.966
		0.324		1.162
1.19	1.025	0.849	1.021	1.099
1.253	1.115	1.212	0.806	0.638
1.567	0.681	1.005	1.183	1.799
0.885	0.958	0.983	0.834	0.75
1.087	1.036	1.211	1.331	1.033
1.515	0.041	1.092	1.8	1.361
1.131	0.965	1.158	1.071	0.823
1.217	1.186	0.349	1.237	0.644
0.651		0.623	0.739	0.882
2.062	1.38	1.186	0.339	1.449
1.178	1.041	0.878	0.157	1.103
0.902	0.926	0.639	1.317	0.85
1.485	0.373	0.455	0.657	1.316
1.168	0.56	0.395	1.973	1.007
0.39	0.349	0.596	1.087	1.052
0.866	1.083	0.903	0.983	0.73
	0.481	0.486		0.488
0.892	1.052	1.545	0.953	0.178
	0.854	1.051	0.877	1.414

	0.429	0.497	0.343	6.887
1.007			1.291	1.281
0.855	1.175	1.608	1.146	0.784
0.821	1.232	1.251	1.39	1.732
1	1.172	1.16	1.075	1.433
0.969	0.692	0.767	0.593	1.097
1.353	0.656	1.026	1.879	0.895
0.159	0.764	1.773	1.112	0.899
	0.715			0.781
1.502	1.047	1.058	1.192	0.645
1.716	0.891	0.959	0.809	0.86
1.115	0.687	0.686	0.843	1.421
1.118	0.715	0.515	0.412	0.837
	0.355			0.408
0.822	0.635	0.651	0.527	0.809
1.164	1.009	0.772	1.265	1.097
1.093	0.439	0.695	0.692	0.657
1.021	1.263			0.979
0.702		0.689	0.778	0.509
1.293	0.967	0.563	1.012	0.535
1.202		1.198	0.069	0.88
1.136	0.79	1.06	1.167	1.351
1.286	1.173	1.243	1.329	1.072
1.103	0.883	0.914	1.127	0.931
0.585	1.514	0.659	0.865	0.884
0.923	1.186	1.175	1.867	1.635
0.207	0.496	0.502	0.496	1.039
1.157	1.137	0.95	1.32	0.919
1.535	0.741	0.66	0.523	0.69
1.003	1.205	1.288	1.312	0.98
0.606	0.434	0.376	2.994	2.07
1.095	1.085	1.159	1.195	1
1.052	1.406	1.371	0.941	1.537
0.966	1.142	1.072	1.091	0.808
0.587	0.74		0.677	1.371
1.416	1.362	1.383	1.173	1.802
0.565	1.266	1.374	1.52	1.255
0.588	0.942	1.582	1.216	0.617
1.42	0.92	0.786	0.178	
0.899	0.847	1.399	0.851	0.739
	1.192	3.545	0.818	0.824
0.306	0.663	0.34	0.636	0.781
0.757	0.846	0.887	0.9	0.766
			0.706	1.041
0.377	0.419	1.224	1.153	0.443
1.039	1.362	0.861	1.756	0.995

0.553	0.477	0.449		3.598
1.108	1.161	1.167	1.182	1.551
0.535	0.614	0.463	0.616	0.574
1.186	1.366	1.372	0.772	0.836
0.511	1.933	0.832	1.086	1.109
				0.782
2.318	0.519	0.587	0.247	0.77
0.408	0.132	0.069	0.056	0.2
1.801	0.827	0.739		0.502
0.907	1.157	1.62	1.097	0.338
1.175	1.071	1.256	1.158	0.707
0.659	0.907	0.729	1.332	0.97
1.035	1.232	1.848	1.005	1.426
1.085	1.054	1.063	1.252	0.887
1.612	1.218	1.062	0.976	0.897
0.728	1.062	1.131	1.126	1.188
1.33	1.029	1.463	1.222	1.257
1.692	1.041	0.972	3.633	0.986
0.826	1.281	0.954	1.136	0.235
1.15	0.667	0.999	0.775	0.687
0.789	1.151	0.667	0.67	0.412
0.783	0.352	0.391	0.368	0.97
0.639	1.025	0.868	3.404	0.702
1.747	1.923	1.656	2.051	0.1
1.658	1.07	0.798	1.39	0.756
0.306	1.005	1.65	0.56	0.82
0.618	0.83	0.955	0.676	0.728
1.729	0.885	0.709	1.113	0.681
0.92	1.001	0.964	1.203	0.835
0.529	0.661	1.014	0.555	0.653
0.978	1.007	0.951	1.005	0.916
1.351	1.245	1.047	1.064	1.421
0.929	0.828	0.714	1.084	0.731
1.002	1.054	0.667	0.782	1.193
1.168	1.206	0.859	0.934	0.967
0.783	0.844	0.558	1.064	1.548
1.08	1.346	0.927	1.174	0.882
0.945	1.578	1.794	1.284	1.427
1.286	0.809	1.241	1.222	0.924
				0.57
1.303	1.628	0.648	1.317	1.306
0.039	0.132	2.997	0.192	0.511
0.588	0.909	0.699		0.604
1.224	0.648	0.832	0.923	1.275
0.42	0.708	0.447	1.372	0.427
1.085	0.935	0.831	1.006	0.773
	2.272	0.653	1.051	1.203

1.057	1.003	0.937	1.284	1.333
0.892	1.372	0.47	0.59	2.201
1.076	0.837	0.871	1.447	0.831
0.911	0.628		1.068	1.06
0.986	1.117	1.008	1.04	1.308
0.891	1.124	1.209	1.368	1.513
2.089	0.343	1.441	1.718	0.241
0.813	1.701	1.052	1.175	1.387
1.342	1.5	0.902	1.489	0.681
	0.752	1.163	0.596	1.131
1.119	1.197	1.278	1.08	1.062
1.182	0.935	0.996	1.1	1.243
0.853	0.992	0.944	1.213	1.119
1.135	0.884	1.241	1.59	1.019
0.928	1.196	1.297	1.077	1.214
0.991	1.03	1.298	0.845	1.322
1.479	0.78	0.581	0.71	1.194
0.992	0.863	0.927	0.917	0.479
0.454	0.437	0.396	0.223	0.311
0.423	1.285	0.626	1.437	1.579
1.144	0.974	1.095	1.409	0.588
1.059	1.16	1.158	1.334	1.712
0.792	0.858	0.27	0.288	0.575
0.709	1.284	1.319	1.115	2.034
0.689	1.241	1.126	1.269	0.732
0.596	0.485	1.344	0.497	0.965
1.024	0.701	1.298	0.905	0.538
	0.8	1.875	0.867	0.562
0.386	0.771	0.933	0.937	0.305
0.924	1.112	1.264	1.006	1.546
1.348	1.065	1.428	1.47	0.703
0.901	1.153	0.808	0.77	1.261
0.802	0.999	1.118	0.87	1.613
1.368	1.098	1.138	1.319	1.476
1.141	0.775	1.204	1.167	1.185
1.118	1.112	1.309	1.044	0.852
0.893	1.606	0.99	1.363	0.935
0.457		0.384		0.486
1.395			0.568	0.885
0.637	0.738	1.205	1.984	1.399
0.35	0.661	0.281	1.088	0.432
1.071	0.936	1.075	1.022	1.229
0.687	1.066	0.976	1.025	0.722
0.774	0.657	0.594	0.705	0.823
1.564	1.193	0.931	1.342	1.058
0.994	2.329	1.261	1.491	1.332

0.981	1.416	1.24	1.232	1.645
1.059	1.213	1.045	1.004	0.568
0.974	1.161	0.794	0.997	1.632
0.87	1.069	1.568	0.777	1.376
0.637	1.376	1.186	0.465	0.846
0.68	1.366	1.293	1.095	0.56
0.833	1.098	1.251	0.891	0.668
0.962	1.438	1.122	1.242	1.757
1.174	0.749	0.928	0.98	1.136
	0.66	0.832		
0.685	1.256	1.532	0.763	0.938
0.598	1.696	1.98	0.138	0.436
1.201	0.787	0.9	1.439	0.51
1.193	0.615	0.851	1.407	0.952
1.05	1.426	1.225	1.216	1.053
				1.248
1.234	1.073	1.409	1.007	0.821
1.212	0.857	0.747	1.116	0.755
0.372	0.485			0.576
1.217	1.015	0.9	0.823	0.828
1.059	0.964	0.921	1.014	1.514
				0.261
0.501	0.826	1.635	0.467	0.489
1.109	1.287	1.113	1.249	1.249
				0.791
0.953	1.021	1.022	0.894	0.618
1.143	0.738	0.666	0.827	0.574
				0.738
0.939	0.987	0.837	0.929	1.249
1.137	0.95	0.807	0.874	1.154
1.161	0.956	1.183	0.955	0.735
1.331	1.061	0.971	1.004	1.002
0.616	0.834	0.341	0.805	0.96
1.052	0.63		0.774	1.4
1.634	0.709	0.615	1.027	1.273
0.987	1.083	1.311	0.793	0.751
1.036	1.251	1.503	1.147	1.341
0.083	0.086	0.126	0.051	0.083
1.215	1.118	0.921	1.182	0.589
1.369	0.718	1.24	0.992	1.599
1.249	1.264	0.956	0.951	0.681
1.108	1.026	1.117	1.403	1.001
		1.175		0.584
1.076		0.819	1.103	0.816
0.974	0.949	1.054	0.967	1.386
0.615	1.432	0.986	0.752	0.844

0.944	0.98	1.11	1.698	0.992
0.925	0.909			0.864
0.717	0.384	1.716	1.048	0.607
1.791	0.304	0.416	0.364	0.746
1.132	1.239	0.993	1.475	1.241
1.083	0.725	1.166	1.39	1.004
1.272	0.887	1.374	1.255	0.842
0.971	1.246	0.733	1.161	0.94
0.99	1.117	1.718	1.294	1.015
0.606	0.905	1.042	0.633	1.305
0.439	0.507	1.183	0.598	1.519
0.954	1.089	1.176		0.728
1.137	0.751	0.776	1.206	0.689
0.773	1.091	1.191	1.002	1.386
2.075	1.1		3.323	0.54
1.27	0.972	1.047	0.943	1.544
1.13	1.21	0.981	0.888	1.053
1.174	0.382	1.136	0.508	0.915
0.875	0.933	0.278	1.493	1.76
0.776	0.772	0.993	0.775	1.013
0.998	1.646	0.474	0.902	0.782
1.264	1.206	1.445	1.246	1.406
0.859	0.622	1.484	0.465	0.806
0.951	1.518	1.119	0.55	0.872
0.991	1.132	0.816	0.88	0.908
1.042	0.689	1.016	0.873	1.106
1.299	0.995	0.915	0.912	1.808
2.095	1.45	1.102	1.145	0.726
1.079	1.422	1.379	1.566	2.265
0.645	0.748	1.508	0.746	0.481
1.05	1.147	1.213	1.087	0.995
1.019	1.114	1.193	1.092	0.948
				0.573
0.911	1.375	1.458	1.267	0.862
1.414	0.752	0.708	0.869	0.882
0.889	1.324	1.568	1.151	2.116
1.275	1.402	0.87	0.871	0.739
0.912	1.44	1.257	1.32	0.741
1.28	2.89			
1.03	1.746	1.076	0.927	0.693
0.744	1.204	2.182	0.783	0.871
1.166	1.306	1.301	1.238	0.93
1.256	1.246	0.725	0.358	0.664
1.109	1.186	1.738	0.689	0.748
0.559	1.432	1.154	1.013	0.831
1.53	1.351	1.224	1.227	1.173
1.408				

1.286	1.158	1.038	0.782	1.625
4.457	1.856	1.025	0.612	
1.021	0.869	0.943	1.32	1.203
1.171	2.879	0.174	0.119	0.048
0.679	1.004	0.843	1.028	0.868
1.236	1.299	1.136	1.558	0.99
1.594	1.609	1.431	1.39	1.572
1.053		1.577	0.913	1.287
1.304	1.865	1.344	0.894	0.766
1.217	0.711	0.877	1.091	0.873
0.259	1.077	1.089	0.985	1.108
1.049	0.576	0.498	0.715	0.617
	0.592			
1.187	0.945	0.985	1.15	0.694
1.018	1.09	0.945	0.898	1.113
0.757	1.131	1.095	0.792	1.57
1.028	1.238	0.99	1.552	0.825
0.625	0.864	1.133	0.836	1.42
1.002	0.886	1.012	1.183	1.067
1.155	1.35	0.914	1.031	1.084
1.016	0.766	0.876	1.089	0.859
0.66	0.971	1.371	0.862	0.711
0.408	0.463	0.443	0.646	0.647
	0.316	1.069		
0.933	1.095		0.896	1.132
1.354	1.403	1.283	1.081	0.6
1.074	1	0.921	1.109	
1.096	0.839	0.93	1.106	1.316
1.061	0.88	1.144	1.062	0.88
1.128	0.78	0.613	0.455	0.67
1.514	0.636	0.933	0.653	1.882
1.18	1	0.846	1.072	1.143
0.97	0.582	0.439	0.477	0.307
1.26	0.384	0.744	0.659	0.578
0.311	0.331	0.311	0.38	0.773
				0.226
	0.499	0.804	0.792	1.64
0.792	0.655	0.781	0.736	0.799
0.175	0.329	0.208	0.291	0.598
0.682	0.749	0.737	1.423	0.836
1.069	1.053	1.284	1.13	1.584
0.806	1.428	0.873	1.213	
0.862		1.505	1.307	0.689
1.483	1.195	1.161	1.084	0.503
0.919	0.645	0.667	1.277	0.849
0.825	1.076	0.702	2.144	0.915
0.749	0.824		0.492	0.44
1.543	0.271	0.988	1.763	0.889
1.514	0.964			0.488

0.984	1.172	1.315	1.253	1.056
0.889	0.903	0.975	1.24	1.352
1.244	0.868	0.62	0.747	0.712
0.014	0.466	0.948	0.213	0.339
1.194	1.441	0.88	1.434	0.82
0.643	1.209	1.149	0.561	0.233
1.021	0.797	1.775	0.838	0.582
1.577	0.721	0.796	1.252	0.858
	0.818	1.198		
0.858	1.016	1.172	1.245	0.851
	0.898			0.609
0.916	1.183	0.847	0.955	0.909
1.29	1.002	1.367	1.287	0.16
0.521	1.207	1.498	0.687	2.457
1.059	1.146	1.691	1.066	0.713
1.4	0.825	0.564	1.248	0.984
0.94	1.101	0.992	0.928	0.856
1.101	0.51	0.533	0.974	1.567
0.471	0.616	0.919	0.737	0.742
		0.943	1.423	0.657
	0.316		0.089	0.199
	1.088	0.936	1.533	1.305
1.33	1.169	1.313	1.329	1.129
0.663		0.195	0.586	1.671
1.516	0.956	0.755	0.929	0.904
	0.342			
0.368	0.599	0.586	0.567	0.866
0.951	0.693	0.682	1.178	1.013
1.284	0.841	1.004	1.185	1.284
0.811	0.984	0.805	0.88	1.266
0.524	2.684		0.853	0.554
1.079				0.502
0.931	1.198	1.497	1.153	0.516
0.757	1.043	1.387	1.283	1.694
0.4	1.795	0.897	0.878	1.248
1.624	0.852	0.993	1.201	0.846
1.806	1.393	0.043	0.56	0.827
			1.207	0.747
0.838	1.117	0.98	1.116	1.505
1.194	0.856	0.963	1.228	1.236
1.032	0.68	0.807	0.948	0.794
1.22	1.047	1.093	1.469	1.308
0.441	0.501	0.776	0.046	0.874
0.798	0.626	0.502	0.714	1.625
0.933	1.144	1.212	1.321	1.114
0.311	3.895	0.232	0.677	1.836
1.144	1.016	1.128	1.038	0.867

	0.271	1.384	0.581	1.414
1.289	0.857	0.837	0.786	0.608
1.005	0.594	0.663	0.688	0.901
1.032	1.259	1.128	1.396	1.297
0.734	1.536	0.923	0.746	0.937
0.991	1.25	0.951	1.112	0.921
1.065	1.519	1.173	1.356	0.862
1.212	1.457	0.732	1.078	1.209
1.031	1.109	1.022	1.022	0.76
0.604	1.046	1.651	1.071	1.403
0.995	1.241	0.525	1.014	1.185
1.019	1.501	1.536	1.568	1.451
0.717	0.85	1.045	0.772	0.931
1.316	0.493	0.461	0.957	2.62
0.094	0.251	0.924	0.465	0.378
	0.088	0.406		
1.636	0.837	0.573	0.835	0.849
1.016	0.386	0.543	0.579	
1.316	1.039	1.12	0.652	0.71
1.039	0.786	1.125	0.844	1.05
0.998	1.108	1.153	1.064	1.632
1.032	0.93	1.242	0.916	0.972
0.722	0.394	0.301	0.516	0.657
0.678	1.114	1.042	1.248	1.691
0.767	1.033	1.031	1.273	0.657
0.867	1.505	0.835	0.869	0.857
0.38	0.648	0.829	0.931	1.207
1.065	0.967	1.181	1.206	1.443
1.051	0.816	0.794	0.785	0.938
0.958	0.927	1.063	0.899	0.891
0.952	1.261	0.834	1.196	1.695
2.208	0.899	1.2	0.629	0.862
1.048	0.988	1.217	1.255	0.873
0.788	1.32			1.284
1.093	1.177	0.925	1.479	0.76
1.448	1.051	0.782	0.851	0.72
1.076	0.958	0.658	1.072	1.424
0.853	1.014	0.504	0.378	3.49
		1.017	0.571	0.619
	0.21			0.229
0.753	0.697	0.707	1.04	0.656
1.058	0.372		0.923	1.649
0.995	0.922	1.695	1.66	1.151
0.783	0.776	0.922	0.793	0.534
	1.253	1.447	0.587	
0.779	1.119	1.295	1.38	1.556
2.052	0.321	0.498	0.336	0.399

1.469	1.308	0.891	1.25	1.752
1.209				
1.477	0.806	2.576	1.06	1.079
0.583	0.467	0.897	1.093	1.548
1.173	1.691	0.455	1.796	0.56
1.085	0.965	0.95	0.853	1.417
2.387	0.691	0.922	0.633	1.21
1.058	0.873	0.971	1.237	0.987
	0.297			0.388
0.942	1.026	1.081	1.297	0.921
1.494	1.171	0.976	1.22	1.131
0.763	0.915	0.842	1.019	0.441
1.397	0.837	1.082	1.244	0.891
0.99	1.006	0.829	0.975	0.802
0.978	1.124	0.923	1.276	0.77
	0.949	0.773	0.834	1.345
1.123	1.637	1.346	0.698	1.825
0.921	1.252	1.681	0.72	0.231
1.316	1.551	1.141	2.081	1.128
0.667	0.37	0.6	0.634	0.306
1.155	0.716	0.904	0.64	1.246
1.115	1.246	0.97	1.101	0.778
1.228	0.776	0.681	1.441	0.952
1.16	2.158	1.092	0.527	1.88
0.754	1.414	1.281	0.728	0.757
1.091	0.882	1.158	1.337	0.883
0.512	0.564	1.229	0.523	0.984
1.591	1.002	0.767	1.115	0.563
0.91	1.623	0.971	1.096	1.501
1.186	1.136	1.108	1.298	1.349
1.107	0.391	0.351	0.549	1.213
0.679			0.023	0.607
0.166	0.267	0.976		
1.467	1.248	1.044	1.291	0.811
0.91	1.057	1.166	1.071	0.999
		0.819		
1.024	1.119	1.05	0.997	1.374
1.215	0.855	1.441	1.507	0.988
0.961	1.54	0.512	0.339	1.725
0.472	0.628	0.428	0.544	0.61
1.497	0.874	0.886	0.941	1.298
0.958	1.334	1.193	0.915	0.923
1.093	1.17	1.938	1.248	0.59
1.043	1.313	1.31	1.477	1.237
0.731	1.157	1.299	0.768	1.869
		1.158		0.202

1.034	1.372	0.974	1.199	0.637
1.218	0.546	0.759	0.728	0.062
1.406	1.034	0.915	0.702	0.991
1.439	0.826	0.491	0.725	1.376
1.134	0.984	1.259	0.94	1.266
1.053	0.669	1.027	1.227	0.729
	1.152	0.886	1.265	1.462
0.708	0.461	0.459	0.487	0.513
0.451	0.986		0.65	2.318
0.828		0.321	0.249	1.292
0.663	1.055	1.332	1.028	1.544
0.994	1.282	1.107	1.517	0.966
1.048	1.085	1.163	1.012	1.712
1.55	1.167	0.996	0.735	0.833
0.484	1.14	1.271	1.301	1.254
0.479	0.504			
				1.006
1.685	1.058	1.1	1.112	1.118
1.157	1.468	1.104	1.02	0.594
	0.298		1.65	0.525
0.819	1.243	1.043	0.956	0.736
1.085	0.966	1.088	1.072	0.982
1.153	0.936	1.034	0.955	0.649
0.853	0.735	1.464	1.701	0.75
0.874	1.375	0.572	0.762	1.514
1.149	0.962	1.271	1.319	1.568
0.85	0.911	0.827	1.092	0.949
0.866	0.982	1.691	0.61	0.581
0.93	2.196	2.459	0.527	0.624
0.761	0.553	1.37	0.965	0.775
1.029	0.996	1.135	1.028	0.947
0.21	0.701	0.703	0.105	0.155
0.755				
	3.593	1.676		0.384
1.222	1.033	1.06	1.005	0.839
0.584	0.824	0.868	1.046	0.906
1.417	1.149	1.099	1.138	1.145
1.036	1.022	1.268	1.179	1.59
1.115	1.104	1.024	1.077	0.902
1.409	0.579	0.615	0.468	1.348
0.622	0.816	1.097	0.978	
1.118	0.837	1.148	0.768	0.976
1.212	1.82	0.606	1.733	1.004
1.165	2.556	0.161	1.14	0.484
0.203	0.192	0.209	0.35	0.526
0.874	0.318	0.475	0.308	0.443

			0.094	0.617
1.12	0.941	0.791	0.592	0.576
1.062			0.727	0.645
0.733	0.795	1.533	0.537	0.648
1.204	1.626	1.429	0.187	2.579
0.995	1.039	1.029	0.987	1.208
0.6	0.838	1.244	1.232	1.287
0.873	1.035	0.84	1.078	0.93
0.198	3.038	0.199	0.203	0.221
0.462	1.139	0.862	1.29	1.184
1.079	1.116	0.062	0.857	0.859
0.972	1.255	1.827	1.009	0.718
0.986	1.058	1.397	0.961	0.93
1.117	0.419	0.298	1.405	1.004
0.583	3.041		0.461	1.803
0.654	0.189	0.519	0.704	0.804
0.417	0.475	0.747	0.464	0.337
1.185	1.326	1.057	1.127	0.829
0.536	0.927	1.052	1.024	0.893
1.452	1.002	0.915	0.926	0.974
0.957	0.936	1.027	1.069	0.804
0.428	0.777	0.563	1.412	0.786
0.101				0.105
1.02	0.407	0.564	0.454	0.72
1.43	1.186	0.85	0.89	0.795
0.771		0.83		
	1.071			
1.818	0.459	0.677	0.697	0.361
1.415	1.111	0.946	1.29	1.004
		0.244		
1.217	1.148	0.811	1.536	1.443
1.52	0.965	0.891	0.85	0.657
1.012	1.039	1.17	1.142	0.806
2.435	0.133		0.197	
0.676		1.291		
1.115	1.07	1.213	1.337	0.829
1.383	0.757	1.853	1.139	1.091
0.68	1.103	0.828	0.777	0.508
0.629	1.135	0.637	0.909	0.466
0.853	0.854	0.869	0.646	0.977
0.76	1.497	0.892	1.293	
0.741	2.143	0.98	0.066	0.194
1.347	0.596	0.351	0.704	0.765
1.611	1.416	0.937	0.707	0.837
1.262	1.3	1.326	1.537	0.967

0.73	1.428	1.31	1.354	1.496
1.052	1.905	0.468	0.413	0.553
0.19	0.942	0.935	1.605	2.354
0.816	1.132	1.268	1.074	1.302
0.251	0.066	0.056	6.476	3.09
1.179	2.102	0.582	1.46	1.4
1.245	1.301	1.361	1.162	1.246
			0.186	
0.184	0.39	0.548	0.427	0.293
1.317	1.124	1.314	1.505	0.849
1.142			0.561	1.622
0.611	1.29	2.468	0.395	0.062
0.078	1.285	0.121	0.067	
1.478	1.088	0.805	1.253	1.003
1.149	0.74	1.371	0.785	0.764
1.012	0.932	1.268	0.912	1.056
1.452	0.884	1.111	1.02	1.184
1.331	1.485	0.459	0.712	0.632
0.751	0.69	0.528	0.955	0.993
0.806	0.414	0.414	0.481	0.567
		2.647	0.882	0.811
0.5	0.913	1.345	0.564	1.349
1.347				
0.975	0.99	0.972	1.141	1.211
	0.839		0.648	1.675
0.824	0.774	0.967	0.921	0.562
1.269	1.458	1.092	1.206	0.778
0.803	0.639	0.664	1.189	1.054
1.227	1.459	1.204	1.134	1.332
0.868			2.247	1.344
1.139	1.038	1.017	1.283	0.958
0.192	0.166	0.555	0.224	0.206
0.785	0.728	0.96	0.582	0.744
				0.731
1.26	0.7	0.805	0.414	0.624
0.641	0.779	0.214	0.426	1.979
1.136	0.827	1.244	0.903	1.132
0.368	0.161	0.478	0.695	0.182
0.762	1.726	0.935	0.885	1.476
0.95	1.223	1.667	1.175	0.838
1.236	1.179	0.958	1.035	0.926
0.153	1.353	1.142	1.091	1.171
1.072	0.746	0.996	0.957	0.904
0.682	0.853		0.922	1.176
1.029	0.921	0.949	0.551	1.129
				0.708
1.019	0.75	0.916	1.452	1.459

1.126	1.475	1.062	0.908	1.56
0.977	1.188	1.263	1.18	1.33
0.795	1.056	0.619	0.819	2.023
1.728	2.684	0.864	0.99	
1.342	1.042	0.855	1.428	1.077
1.724	0.283	1.403	1.136	1.249
1.624	1.325	0.453	0.634	0.734
0.455	0.641	0.485	0.658	0.694
0.925	0.591	0.949	0.75	1.706
0.317	0.272	0.82	0.267	0.329
1.039	1.211	0.987	1.092	0.986
1.852	1.625	2.185	1.245	0.348
1.212	0.619	0.591	1.094	1.151
1.148	1.014	1.628	1.128	1.046
	0.788	0.973	0.887	1.359
1.355	1.175	1.401		1.383
0.863	0.806	1.361	0.681	0.776
1.173	1.144	1.459	1.575	1.326
0.822	0.629	0.593	0.742	0.702
1.378	1.085	0.737	1.027	0.874
1.227	1.087	1.135	1.141	1.523
0.933	1.162	1.509	1.265	0.883
1.154	1.016	0.484	0.668	1.192
	0.756	0.264		
1.537	1.049	0.633	0.645	0.752
1.003	0.978	0.897	1.099	0.857
1.158	0.83	1.086	1.11	0.983
1.266	1.407	1.228	0.918	1.019
				1.307
1.36	0.813	1.204	1.246	1.039
0.668	1.336	0.834	0.634	0.991
0.71	1.11	0.549	0.678	0.886
	1.407	0.358	0.28	1.38
0.741	1.415	1.873	0.862	1.276
0.882	1.04	1.482	0.734	2.069
0.632				
0.549		0.276		0.525
0.686	0.228	0.564	0.825	1.038
1.248	1.294	1.319	0.84	0.628
1.438	0.993	0.874	1.218	0.846
0.89	0.869	1.505	1.438	1.734
1.027	1.096	0.687	1.018	1.183
0.894	0.947	0.957	1.111	0.565
0.987	1.041	0.86	0.636	0.892
1.007	0.733	0.953	0.769	1.399
0.937	1.192	1.32	0.546	0.871
0.776	0.618	1.121	0.653	1.318
0.867	1.201	0.651	0.576	0.745

0.786	1.235	1.509	0.613	0.725
1.295	1.325	1.017	0.645	1.425
1.004	1.07	0.957	1.46	1.444
0.673	1.029	1.289	1.255	0.842
		0.162		
1.76	0.646	1.33	0.966	1.247
1.229	0.52	0.614	0.638	0.937
1.302	1.225	1.285	1.34	1.281
0.888	1.173	1.627	1.178	0.824
0.733	1.139	1.146	0.935	1.331
		0.823		1.18
1.307	0.812	0.877	1.53	1.112
0.951			0.843	1.537
1.077	0.986	1.037	1.037	1.357
0.233			0.37	
1.278	1.207	1.2	1.319	1.626
1.442	1.372	1.161	0.953	0.718
0.842	1.086	1.075	1.186	1.401
0.916	0.657	0.851		0.515
	0.636		0.637	
0.934	0.87	1.021	0.971	0.678
1.181	0.7	0.919	1.091	1.25
0.834	1.063	0.824	0.909	0.922
1.215	0.809	1.083	1.408	1.734
1.231	0.896	0.987		1.534
	0.883	0.577	0.482	0.234
1.143	0.762	0.86	1.839	1.191
0.805	1.038	0.938	0.955	1.224
1.064	1.34	1.056	0.833	1.208
0.623	1.256	0.298	6.198	0.353
1.1	1.2	1.248	1.169	1.042
1.426	0.743	2.109	1.34	2.605
1.007	0.754	1.046	1.014	0.999
1.21	1.07	1.451	1.387	0.949
0.685	0.577	1.466	0.37	0.446
1.222	1.022	1.024	0.694	0.844
0.681	0.861	1.297	0.918	0.616
1.383	1.273	1.298	1.124	0.977
1.1	0.708	0.846	0.533	0.982
1.033	0.991	0.892	0.778	1.281
	0.944	1.125	0.81	1.352
0.894	1.788	1.336	0.895	2.386
0.567	0.666	0.448	0.667	0.709

0.689	1.385	1.062	1.258	1.422
1.712	1.267	0.366	1.292	1.453
1.005	0.889	1.077	1.037	1.09
1.102	0.88	0.73	1.194	0.884
1.349	1.178	1.009	1.379	0.907
	0.947	0.784	1.036	1.141
0.902	1.373	1.238	0.915	0.569
1.155	1.029	1.132	1.095	1.331
0.791	1.37	1.162	0.989	1.279
0.705		1.177	3.505	0.753
	1.285	0.991	0.926	1.188
1.4	1.153	0.901	1.303	0.836
0.853	0.586	1.195	0.729	0.947
1.336	1.201	1.203	0.867	0.703
1.472	0.816	1.142	0.864	0.875
1.252	1.126	0.592	1.82	0.848
0.836	0.798	0.789	1.154	1.023
	1.269			
1.178	0.699	1.809	1.24	1.222
0.698	1.311	1.269	0.999	0.967
1.399	0.562	1.233	0.918	0.954
1.04	0.935	1.344	1.749	1.661
1.19	1.166	1.501	1.9	1.078
1.319		0.619	0.425	0.913
0.853	1.155	1.025	1.199	1.111
0.81	1.206	1.666	1.094	0.433
1.062	1.249	1.254	1.081	1.284
1.452	0.92	1.904	2.117	2.5
1.179	0.996	1.335	1.817	1.887
0.562	0.938	1.098	1.516	2.572
1.062	1.258	1.159	1.234	1.578
	0.528		0.613	
0.373	0.458	0.259	0.554	0.492
1.351	0.746	0.602	1.171	1.103
1.702	0.382	0.464	1.74	0.713
0.464		1.065	0.734	1.261
1.134	0.993	1.265	1.373	1.733
1.238	0.976	1.671	1.098	1.57
0.053	0.006	1.317	1.294	0.069
				0.962
	1.16	0.775		2.507
1.002	1.008	0.918	1.227	1.151
1.168	0.713	1.448	1.074	2.068
1.325	1.046	1.194	0.667	0.826
0.825	1.278	1.224	0.8	0.709
0.404		1.264	1.098	0.918
1.472	0.711	0.896	0.994	1.075
0.762	1.077	1.166	0.971	1.993

0.3	2.049	0.538	0.286	0.776
0.753	1.232	1.032	0.821	1.315
1.137	0.822	0.819	1.471	1.161
1.75	1.184	1.447	1.333	1.392
1.124	1.076	0.77	0.91	0.766
0.807	1.051	0.93	0.749	0.646
0.608			1.539	0.49
				1.709
0.796	0.513	0.668	1.384	1.579
1.044	1.024	1.352	1.407	1.496
				0.866
0.928	0.833	0.78	1.21	0.769
0.862	0.976	1.233	1.576	1.819
0.959	1.155	1.07	1.018	1.259
0.974	1.174	0.953	1.162	0.918
1.047	1.128	1.197	1.271	1.726
	2.343	1.404		
0.864	1.947	0.702	0.658	1.417
1.172	1.054	0.944	1.097	0.891
0.705		1.173	1.226	1.487
1.11	1.185	1.277	1.715	1.148
0.755	0.512	1.197	0.678	1.455
1.358	1.584	0.004	1.476	1.709
0.953	1.016	0.582	0.952	0.558
1.024	1.222	0.699	0.996	1.072
0.868	1.502	0.988		0.926
0.829	1.067	1.113	1.185	1.245
0.962	1.339	1.281	1.38	1.329
0.719	0.494	1.183	0.638	1.966
2.09	0.447	0.39	0.699	1.471
0.776	0.971	0.592	0.871	1.594
1.23	0.895	0.867	1.029	0.859
0.427	1.166	1.275	0.623	0.648
0.41	1.219	0.717	1.427	2.018
1.027	0.784	0.831	1.445	1.19
1.486	1.135	1.24	0.996	1.139
1.359	0.495	0.767	0.446	0.301
1.019	1.107	0.976	1.051	1.265
0.841	0.703	0.999	0.945	1.436
0.799	1.111	1.409	1.132	1.324
1.009	1.05	0.712	0.855	1.129
		0.613	0.744	0.579
0.741	1.453	1.001	1.025	1.267
0.677	1.344	0.869	0.882	1.691
0.903	0.445	1.095	0.683	0.929
0.641	1.209	1.03	1.065	0.834
1.074	1.219	0.647	1.046	1.815
0.948	0.895	1.126	0.911	1.142

0.806	1.034	1.115	1.531	1.224
1.134	1.321	1.329	1.066	0.704
0.705	0.794	0.738	1.312	0.543
1.188	1.184	1.102	0.795	1.153
0.287	2.251	0.034	0.024	1.822
0.661	1.326	1.219	1.322	1.356
1.107	1.144	1.089	1.297	1.24
1.203	1.362	1.27	0.926	0.812
0.493	1.471	0.564	0.384	0.334
0.911	1.265	0.953	1.712	1.513
1.52	0.757	0.548	0.607	0.487
0.884	0.96	1.053	0.843	0.715
1.008	1.002	1.024	1.214	1.442
	0.857	1.075	0.811	0.82
1.461	0.928	0.79	0.95	2.13
1.965	1.379	1.266	0.825	0.646
1.079	1.237	1.1	1.109	0.863
0.771	1.096	0.875	1.232	1.215
0.935	1.434	1.637	1.317	1.366
0.172	0.78	0.825	0.785	1.034
0.845	1.123	1.036	1.038	0.737
1.06	0.875	0.634	0.73	2.741
0.679	1.107	0.942	0.7	1.139
1.417	0.64	0.875	1.536	0.432
1.372	2.047	0.973	1.092	0.965
0.898	0.97	1.2	1.028	0.923
0.743	0.81	0.769	1.267	0.802
0.977	1.038	0.66	0.287	1.247
1.391	1.235	1.114	1.138	1.433
1.254	1.183	0.918		
	0.923	0.971	0.636	
1.365	0.632	0.396	0.786	0.379
1.039	0.895	1.166	1.198	1.37
1.626	1.392	1.486	0.744	1.033
0.954	0.953	1.112	0.908	1.161
0.778	1.387	1.176	1.097	1.139
0.863	1.315	1.016	1.142	1.436
1.002	1.058	0.943	1.253	1.578
0.561	0.972	1.095	0.595	0.358
1.527	1.149	0.934		0.931
1.013	1.187	1.087	1.008	1.25
1.247	0.975	1.181	1.181	1.106
1.128			0.543	1.086
0.738	0.354	0.688	0.802	0.476
0.831	1.264	1.024	0.803	0.81
0.884	1.162	1.154	0.842	0.874

1.355	0.778	2.068	1.221	1.423
1.243	0.841	0.92	0.825	1.201
1.57	1.054	0.962	1.651	0.854
0.667	1.061	0.772	0.33	0.285
0.986	1.481	1.114	0.994	1.694
0.881	1.221	0.718	0.954	1.098
1.1	1.504	0.807	1.458	1.911
1.189	1.123	1.189	1.076	1.227
1.125	0.641	1.792	0.743	1.004
1.298	1.28	1.214	1.648	1.048
0.85	0.086	1.271	1.147	1.892
0.825	1.168	0.681	1.573	1.691
0.818	1.28	1.028	1.051	1.163
1.359	0.365	1.335		0.822
0.997	1.565	1.151	0.953	0.485
1.055	0.712	0.819	1.009	0.965
0.64	1.02	1.186	0.963	1.017
1.016	1.117	1.214	1.273	0.765
0.738	1.615	1.479	0.715	0.848
0.896	0.769	1.205		0.949
		1.151		2.175
1.755	0.861	0.709	1.136	0.654
1.203	0.899	0.902	1.274	0.982
0.68	0.756	1.274	1.236	0.919
1.246	0.408	0.407	0.334	1.003
0.747	1.203	0.928	1.346	1.035
1.003	1.098	0.873	1.017	0.907
				0.783
0.973	1.104	1.011	1.183	0.938
1.147	1.402	1.168	1.032	0.694
1.896	0.963	0.474	0.982	1.858
1.44	1.048	1.404	1.376	0.815
0.831	1.179	0.934	0.939	1.559
1.021	0.988	0.943	0.848	0.913
1.12	1.163	1.116	1.365	0.801
	0.451	0.359	1.051	2.02
			0.222	0.018
2.107	1.319	1.346	1.047	1.538
0.83	1.698	1.082	0.786	0.885
1.053	0.88	1.105	1.717	1.795
0.586	0.335	0.393	0.336	9.18
0.955	0.597	0.426	0.639	0.686
1.22	1.134	0.251	1.319	1.075
0.965	1.634	1.131		
1.153	1.015	0.882	1.076	0.733
1.295		1.077	0.637	1.082
0.932	0.372	0.56	1.196	0.975

0.84	1.044	1.203	1.104	1.469
1.136	1.093	0.889	0.736	0.724
		0.747		0.606
1.656	0.543	1.173	0.975	1.624
0.816	1.006	1.207	1.395	1.143
0.807	1.14	1.17	1.023	1.834
1.145	1.41	1.07	0.863	0.611
3.073	0.558	0.656	0.742	0.787
			0.299	
1.106	1.004	1.269	1.198	1.336
0.785	0.797	1.024	1.074	1.114
1.141	1.17	1.336	1.479	1.689
0.581	0.846	1.178	0.946	1.348
1.672	0.864	1.41	1.225	1.37
0.77	1.476	1.933	1.652	1.187

A_CW_2	A_ZAM_3	A_LSQ_4	A_YSZ_5	A_GH_6
				1.037
1.037	2.073	1.902	1.195	1.648
1.199	1.046	1.37	0.015	0.446
0.881	0.828	0.553	0.91	1.94
1.261	0.77	1.707	0.828	0.674
			0.569	
1.024	0.965	0.773	1.061	1.035
1.217	1.16	1.491	1.525	0.5
1.097	0.868	0.825	0.692	1.036
0.874	0.785	0.885	1.305	1.55
0.365	0.368	0.309	0.393	1.743
0.81	1.455	1.013		1.269
0.684	0.522	0.366	0.57	1.354
1.311	1.82	0.41	1.629	5.009
1.434			0.111	
	0.849		1.534	
1.034	1.181	0.897	1.191	0.601
1.108	0.985	1.459	1.204	1.662
0.287	0.16	2.682	0.159	4.974
1.566	1.219	0.502	2.694	0.97
0.864	0.767	0.768	1.29	0.974
1.143	0.93	0.915	1.01	2.397
1.287	1.943	0.7	0.413	0.678
0.817	0.633	0.434	1.197	1.074
				0.005
1.172	1.321	1.047	0.896	0.844
		0.068	0.105	
1.051	0.902	1.177	1.27	0.251
1.01	1.095	1.178	1.05	1.835
		0.785		
0.842	1.855	1.309	0.687	0.279
			0.504	
1.401				2.507
1.028	1.412	0.826	0.885	1.393
0.536	1.58	0.628	0.801	0.722
1.219	0.685	0.474	0.945	
0.648	0.611	0.998	1.27	0.302
1.157				
0.369	0.399	0.474	0.725	0.698
0.123	0.168	0.965	0.745	
1.141	1.182	1.133	1.701	0.009
		0.781	1.345	
	1.219	0.649	0.953	0.55
	2.253	2.014		

		0.102		
		0.565		0.603
0.879	0.503	0.664	0.433	0.846
1.108	0.787	1.416	0.879	0.918
0.887	1.523	1.246		0.61
2.273		0.767	2.086	1.688
			0.268	
0.168		1.54	0.197	2.796
0.731	0.959	1.047	1.139	0.48
0.88	0.767	0.779	1.093	0.566
1.092	2.056	1.205	0.811	1.268
0.679	0.893	0.907	1.248	1.489
1.345	0.706	1.681	0.937	0.45
0.568	0.592	0.768	0.716	1.895
0	1.038	1.649	0	2.132
1.159	0.559	0.694		
0.996	0.453	0.76	1.291	0.854
1.32	1.212	1.276	1.006	0.88
0.414	1.278	1.228	1.086	0.543
0.891	1.646	1.497	0.897	1.242
1.066	1.016	0.918	1.162	1.13
0.744	1.219	1.457	1.436	1.351
3.063	0.003	0		
1.666	2.148	0.297	0.12	0.23
1.126	0.739	1.807	1.245	1.718
1.17	1.089	1.28	0.826	0.083
1.927	1.576	1.586	1.996	0.677
0.282	1.146	1.789	2.624	1.295
1.051	1.326	1.255	0.418	0.477
0.942	1.163	1.41	1.356	0.137
0.439	0.71	0.563	1.119	1.014
0.541	0.323			0.01
0.629	1.688	0.285	1.068	0.938
1.144	0.82	0.741	1.076	1.119
1.072	0.515	0.206	0.028	0.487
0.093	3.26	0.081	0.073	0.108
0.733	0.888	1.551	1.168	0.587
0.589	0.573	0.44	1.175	0.444
0.625			1.515	0.367
0.229	1.545	0.231	0.158	0.373
0.686	1.063	0.418	1.403	
0.578	0.707	1.086		2.901
1.2	0.929	1.311	0.67	0.794
1.018	0.89	0.947	1.433	0.928
0.76	0.885	0.663	0.558	

	1.345	1.154	0.761	
0.994	1.425	0.989	1.173	0.828
1.62	2.33	0.84		1.18
0.805	0.947	1.284	1.192	0.999
				0.43
0.983	0.901	0.805	0.956	1.799
0.701	1.554	0.614	2.052	0.855
0.705	0.615	0.779	1.29	1.172
0.917	0.461	0.784	1.129	0.946
0.912	1.307	1.347	1.275	0.94
			1.23	
1.131	1.158	0.751	0.871	1.304
1.017	1.039	0.967	1.084	0.983
0.928	0.883	0.99	0.79	2.127
0.496	0.862	0.782	0.84	
1.09	1.449	1.208	0.849	0.661
1.108	0.966	1.14	0.426	0.763
0.729	0.868	0.951	0.995	1.131
0.86	1.56	1.574	0.994	
0.807	0.642	0.938	0.614	
0.776	0.652	0.992	1.456	2.437
0.82	0.976	0.951	1.123	1.168
0.958	0.906		0.887	0.994
0.972	0.8	0.636	0.987	0.771
0.525	1.227	2.205	0.678	
0.977	0.787	0.854	1.152	1.659
		0.472		
0.775	0.706	0.358	1.429	
	0.339	1.026	1.382	
0.157			3.928	0.158
1.17	1.569	1.14	1.017	1.407
1.316	0.8	1.422	3.383	1.97
1.03	0.383	0.927	1.185	0.962
0.569	2.332	0.935	0.551	0.854
1.524	1.14	0.895	1.135	0.744
0.916	0.875	0.832	0.947	1.011
1.091	1.093	1.222	0.862	
	0.764	1.124	1.154	0.258
0.735	0.785	0.551	0.92	0.466
1.948	0.694	0.579	0.946	
0.886	0.943	1.382	1.306	2.129
1.196	0.972	1.455	0.898	1.293
0.843	1.338	1.233	1.58	1.101
1.188	1.047	0.673	2.017	1.292
1.225	2.246	1.341	0.589	1.119
1.141	0.993	1.159	1.294	1.942

	1.933			0.304
0.748	1.579	0.745	0.851	0.932
		1.144	1.084	1.29
0.934	1.053	1.507	0.612	0.123
	1.615			
0.826	1.19	0.932	0.764	
0.746	0.962	1.701	1.607	1.831
1.445	0.787	1.63	0.829	1.434
1.217	0.871	1.378	1.294	1.501
1.184				
1.117	0.8	1.141	1.076	0.369
0.852		1.097	0.878	2.299
1.109	0.774	1.049	0.959	1.76
1.041	1	0.829	0.966	0.739
1.257	0.891	1.325	1.476	0.678
0.373	1.857	0.185	0.552	0.391
1.117	1.058	0.404	1.018	
1.121	1.567	1.24	0.682	2.431
	1.298	0.914		
1.234	0.779	0.579	0.793	0.964
1.397	1.505	1.125	1.105	0.92
0.867	0.801	0.536	1.008	1.246
1.132	0.852	1.001	1.254	1.193
0.687	1.241	0.748	0.867	0.63
1.008	0.84	0.623	1.002	1.101
0.997	0.8	0.921	1.031	0.743
1.276		0.739		2.66
0.948	1.077	1.249	1.142	1.804
1.63	0.752	1.059	1.236	0.798
0.709	1.04	0.76	1	1.481
1.12	0.961	0.626	0.514	2.01
1.181	1.541	1.195	0.846	0.742
1.099	0.982	0.312	1.114	0.928
0.683	0.82	0.461	0.488	
1.157	0.366	0.817	0.937	0.568
0.721	1.198	1.098	1.091	2.305
1.592	1.09	0.534	0.637	0.838
1.123	1.282	1.117	0.953	1.21
0.831	1.009	1.411	0.919	0.327
1.076	0.885	1.104	1.244	1.322
1.322	0.613	1	1.276	1.882
0.931	1.263	1.052	0.712	0.633
0.833	0.839	0.655	1.157	0.466
0.966	0.549	0.695	0.838	
1.055	1.656	1.25	0.916	0.929
1.217	1.451	0.919	0.981	1.023
1.673	1.206	1.045	0.734	2.976
0.984	0.88	1.041	1.316	1.36
0.932	1.874	1.791	0.789	1.858

1.667	2.1	1.325	0.121	2.115
0.635	1.414	0.589	1.177	0.783
0.825	1.007	0.926	1.123	1.131
1.548	2.339	1.434		0.598
0.755	0.629	0.786		0.957
1.044	0.778	0.731	0.84	0.298
1.217	1.448	1.13	1.232	1.049
0.328	0.312	0.268	0.233	0.371
0.49	0.443			0.257
1.122	1.204	1.03	1.094	0.815
0.979	1.236	0.757	1.198	0.658
1.37	1.005			
0.854	1.127	0.912	1.579	1.034
				1.241
0.327	1.266	0.489	3.18	1.275
1.076	1.758	1.445	0.863	0.887
1.852	2.044	1.948	0.566	1.505
0.912	0.366	2.14	0.155	2.145
		0.933		
1.021	2	0.851	0.084	1.718
0.462	0.722	0.231	2.009	0.307
0.882	0.869	0.948	1.686	0.378
1.017	1.005	1.207	0.984	1.229
0.705	0.55	1.213	1.139	1.928
1.344	1.179	0.847	1.65	1.458
1.106	0.912	1.127	0.613	
1.429	1.527	1.217	0.591	0.619
1.029	1.049	1.085	1.316	1.508
0.818			0.024	3.608
1.091	1.839	1.073	1.113	0.638
		0.08		0.109
1.143	0.828	1.121	0.77	0.857
0.694		0.47	0.558	
1.34	1.1	0.611	0.883	1.848
0.575	2.891	1.08	0.514	0.488
1.05	1.322	1.146	0.966	
0.92	1.052	0.998	0.689	1.109
0.863	1.384	0.986	0.791	
1.068	0.725	0.756	0.687	0.396
1.374	0.61	1.102	1.136	1.511
1.347	1.539	1.434	0.987	0.815
1.128	0.986	0.827	1.561	0.418
0.991	0.917	1.21	1.393	1.261
1.877	1.856	1.648	0.626	1.223
0.826	3.28	0.786	0.514	1.376
0.849	1.654	2.017	1.26	0.81
0.949	0.93	1.058	1.013	0.762
	0.341		0.341	

1.248	0.928	0.941	1.073	1.567
1.037	1.165	1.105	0.657	
1.041	0.565	0.96	1.423	1.127
1.015	0.701	0.866	1.243	1.258
1.088	1.864	1.76		0.922
1.213	0.683	1.593	0.533	
1.227	1.131	0.813	0.45	0.518
0.582	0.546	2.026	0.691	1.883
0.9	1.272			0.58
1.103	0.884	0.716	1.615	2.184
0.624	0.242	0.259	0.305	0.993
1.323	1.746	1.065	0.166	1.716
1.446	0.92	2.076	0.842	0.797
0.847	1.006	1.355	1.224	1.804
1.313				
0.835	0.949	0.887	0.051	0.163
0.911	1.03	0.944	0.831	0.811
1.237	1.245			0.265
0.466	0.649	0.46	0.556	0.204
0.999	0.42	0.362	0.985	0.759
1.067	1.145	1.128	1.174	0.879
4.436	0.882	4.407	0.151	0.498
		1.051	1.361	0.445
1.169	1.352	3.267	1	0.73
1.208	1.136	1.129	0.451	0.622
1.232	1.084	0.914	1.139	
0.515	1.365	1.561		0.922
			1.326	1.123
1.073	1.03	0.713	0.849	1.77
1.092	1.467	0.649	0.372	1.211
0.942	0.997	0.613	0.592	1.199
0.606	0.91	0.801	0.875	1.246
1.333	0.995	0.766	1.104	0.64
0.385	1.819	0.28	0.793	1.39
	0.134	0.07	0.066	
0.191	0.12	0.094	0.082	0.047
	0.83	1.013		1.191
	0.858			0.942
0.863	0.898	0.751	1.278	
0.95	0.903	1.099	1.082	0.953
1.392	1.085	0.835		0.671
1.074	1.059	1.045	1.35	0.803
0.882	0.675	0.797	1.204	1.036
0.659	1.202	1.089		
0.992	1.859	0.863	0.606	1.571
0.219	1.841	0.152	0.286	0.612
1.316	1.703	1.22	0.761	1.103
1.246	1.468	1.519	0.83	1.299
1.179	1.105	0.789	1.305	0.439
0.436	0.652	1.488	1.202	1.223

1.172	1.74	1.808	1.033	0.641
0.858	1.052	0.791	1.127	1.269
0.467	1.79	0.303	1.098	1.084
1.093	1.141	1.486	1.05	1.925
1.104	0.721	0.926	1.079	1.684
1.302	1.606	1.277	0.952	0.774
0.172	1.013	0.599		1.415
1.209	1.295	1.505		1.5
0.724	0.467	0.967	1.929	2.094
0.733	0.8			0.899
0.793	0.984	0.923	1.208	1.492
1.1	0.857	1.025	0.955	0.877
1.426	1.34	1.106	0.555	1.215
0.894	0.768	0.727	0.901	0.2
0.592	0.756	0.323		
0.779	0.502	0.659	3.072	2.132
0.865	0.631	1.231	1.181	1.473
0.893	0.687	1.093	0.92	0.946
1.027	1.286	1.209	1.053	0.574
0.637	0.932	0.622	0.517	0.21
0.683		1.112	0.526	1.081
1.055	1.005	0.974	0.474	0.497
0.662	0.854	0.798	1.292	0.434
0.907	1.501	0.591	1.16	2.042
0.859	0.994	1.018		0.882
2.407	0.597	1.069	1.737	1.702
1.015	1.098	1.181	1.082	0.905
0.775	1.129		1.04	
0.974	1.075	0.925	0.766	0.782
1.345	0.594	1.193	1.059	1.19
1.171	1.643	1.337	1.056	0.781
0.785	0.9	1.249	1.003	0.605
1.141	1.341	0.51	1.693	
0.988	1.644	1.362	0.718	0.997
0.303	0.519	1.784	2.578	0.84
1.317	1.391	0.273	1.341	3.057
	1.794			1.548
0.175		0.102	0.791	0.332
1.599	1.499	1.173	1.064	0.955
0.617	1.334	1.759	1.089	3.369
0.744	0.552	0.822	1.564	0.73
1.482	1.895	1.727	1.018	0.661
0.839	0.826	0.547	0.447	1.423
1.895	1.119	0.69	0.689	0.985
		0.819		2.747
1.087	0.886	0.897	1.169	0.676
	1.519			1.122

1.116	0.656	1.838	0.618	0.439
	0.25	0.538		
1.291	0.694	1.059	1.023	0.243
0.973	0.933	0.899	1.155	2.155
		0.944		1.417
1.072	1.574	5.296	0.91	0.821
0.442				0.735
1.015	1.521	0.797	0.763	
0.989	0.576	1.71	0.484	2.19
0.339	0.371	0.252	0.291	0.442
0.935	1.537	0.964	1.16	2.307
1.435	1.379	1.56	0.77	0.776
1.298	1.053	0.84	1.044	1.068
0.933	0.821	0.554	0.754	3.584
1.738	0.834	0.493	1.43	1.226
1.201	1.169	1.039	0.903	1.077
1.18	1.249	1.383	0.643	0.934
1.085	1.045	1.733	0.934	1.467
1.057	0.899	0.282	1.207	0.947
1.258	1.107	0.917	1.03	0.456
1.587	1.332	1.526	0.655	0.361
0.848	0.173	0.281	1.09	0.274
0.955	0.974		1.18	1.153
0.81	0.737	1.133	0.664	
1.096	1.256	0.987	1.103	1.832
0.878	0.704	0.775	1.469	2.059
0.228	0.27	0.429	7.296	
0.735	0.741	1.994	0.782	2.603
0.916	0.505	1.948	1.303	0.91
	0.907	1.117		1.678
0.569	2.214	0.956	0.184	0.504
	0.466		0.279	
0.485	0.725	0.356	0.465	2.55
1.024	1.366	0.628	0.961	0.337
0.857	1.143	0.417	0.763	0.724
0.882	0.813	1.237	0.963	0.529
1.221	0.457	1.055	0.65	1.998
1.805	1.36	1.687	1.087	1.304
0.945	0.907	1.045	0.624	0.485
0.872	1.023		1.058	0.715
1.086	0.954	0.638	0.972	1.048
0.537		0.738		
0.138	2.148			
0.186	0.171	0.19	0.654	0.351
0.949	1.463	1.6	0.994	0.92
0.956	1.04	1.058	1.073	1.562
0.813	0.789	0.581	2.026	0.62
3.618	0.563	0.431	0.821	0.98
1.028	1.1	0.981	1.112	0.1

1.041	0.856	1.243	0.91	0.965
1.336	1.027	0.959	0.958	0.241
1.184				
0.576	0.748	1.029	0.958	
1.114	1.663	1.272	0.71	0.878
0.842	0.938			0.601
1.19	1.49	1.113	0.81	1.061
			0.428	1.037
0.714	0.577	0.767	0.931	0.494
1.066	1.044	1.248	1.199	1.479
1.027	1.39	1.311	1.552	0.385
1.035	1.19	0.882	1.087	0.705
0.995	1.305	1.015	1.277	0.746
0.962	0.565	0.916	1.164	
0.879	0.805	1.056	1.227	0.672
1.047	1.01	1.152	1.305	1.46
0.802	0.675	0.585	1.114	1.029
1.254				1.463
1.017	0.655	1.217	1.098	1.425
1.617	0.868		1.382	
0.392	0.532	0.762	0.454	0.381
0.199	0.39	0.35	0.094	1.53
1.216	1.458	1.386	1.1	0.984
1.488	1.109	0.833	0.667	1.12
1.152	0.989	0.751	1.065	0.599
1.747	1.074	0.376	1.378	
1.198	0.852	1.144	1.058	0.46
0.836	1.782	0.635	0.696	2.424
0.875	0.262	0.347	1.321	0.983
				1.262
1.232	1.095	0.972	1.12	
0.22		0.227	0.082	3.08
1.647	1.356	1.636	0.884	0.393
0.636	0.613	0.57	0.753	0.706
0.844	0.481	0.412	0.654	0.606
0.972	1.043	1.024	1.213	0.347
0.99	1.238	1.089	0.882	0.702
0.91		2.096		
	0.349	0.424	0.464	1.034
1.464	1.249	1.028	1.341	1.816
1.433	1.316	1.438	0.795	
0.687	0.693	1.214	0.978	2.682
1.066	0.984	1.241	1.036	0.795
0.5	0.901	0.638	0.602	0.85
1.654	1.993	0.188	0.798	1.441
0.406	0.343	1.643	0.869	0.956
1.193		0.126	1.425	

1.147	1.787	1.496	0.805	1.072
1.213	0.924	1.76	0.916	0.443
2.006	1.413	1.665	0.811	1.948
0.984	0.688	0.809	1.14	0.702
1.214	0.614	0.748	1.451	1.126
0.565	1.041	1.161	0.913	0.691
0.793	1.05	0.923	0.976	0.748
0.399	0.548	0.96	0.518	1.574
	0.507	0.391		1.272
1.272	2.475	0.707	2.086	0.626
0.24		0.211		
		1.324		
0.97	1.047	0.833	0.781	0.746
		0.707		0.625
0.662	0.595		0.584	
0.555	0.871	0.829	0.674	0.392
1.368	1.218	1.244	0.752	1.026
1.017	1.204	1.212	1.005	0.837
1.056	0.533	0.656	0.716	1.378
1.492	0.33	0.96	1.591	0.746
1.017	0.87	1.071	1.446	0.48
1	1.24	1.248	2.078	1.835
	3.551	1.305		1.388
0.104	0.138			1.83
1.049	1.014	1.277	1.126	0.688
1.185	1.427	1.071	0.86	1.09
1.22	1.17	1.229	0.512	0.364
0.99	1.177	1.008	1.202	0.724
0.776	0.792	0.572	0.817	1.102
1.126	1.788	1.627	0.862	1.318
1.028	1.715	1.644	1.361	1.251
1.153	0.844	0.991	1.157	1.595
1.193	0.664		0.66	1.504
1.206	1.228	0.968	1.303	0.945
1.427	2.223	1.178	0.684	0.483
0.832	0.247	0.217	0.154	0.202
1.133	0.786	0.909	1.44	0.864
1.23	1.321	1.195	0.595	1.398
			1.43	0.308
1.281	1.855	1.752	0.904	2.379
0.382	1.575			0.888
0.794	0.223	2.24	0.529	0.416
1.162	1.54	1.027	1.087	1.288
0.588	0.531	1.183	1.367	1.645
1.776	0.959	0.789		
1.196	1.24	1.199	0.835	0.915
0.993	0.984	0.933	1.573	0.895

0.918	0.447	0.751	0.833	1.318
0.864	1.352	0.659	0.711	0.683
1.11	0.877	1.007	1.235	1.155
0.807	0.89	0.853	0.918	1.315
	0.069		0.431	
1.658	1.078	1.416	1.006	1.129
			0.736	
				1.126
	0.107	0.102	0.206	
	0.503			
1.26	1.319	1.052	1.549	0.153
0.623	0.57	0.567	0.508	0.368
1.047	1.187	1.054	1.147	0.765
1.024	1.126	0.821	0.701	0.965
	2.415	1.698		
0.794	1.193	1.947	0.959	1.027
1.12	1.08	1.256	1.479	1.344
	1.424			0.091
1.263	1.307	1.82	0.574	0.773
0.88	1.082	1.068	1.004	0.843
0.904	1.094	0.891	1.35	1.559
0.423	1.301	0.608	1.882	1.159
0.474	1.146	0.968	0.622	0.709
0.971		1.173	1.155	1.312
0.957	0.862	0.978	1.39	1.573
			0.089	
		1.238		
0.835	2.322	1.125	0.999	0.447
0.441	1.647	0.938	1.301	1.598
0.895	0.563	1.175	0.744	1.171
1.33	0.99	1.579	0.785	1.078
	0.421		1.219	1.273
1.106	0.883	0.665	0.739	0.779
0.957	0.86	1.03	1.315	1.016
1.071	0.965	0.655	1.585	0.094
1.316	0.729	0.589	1.293	0.122
1.022	1.624	1.047	0.931	0.948
1.235	0.976	0.788	0.967	1.495
1.659	0.083	1.415	1.377	1.324
0.778	4.27	0.479	1.993	0.136
0.817	0.892	0.879	1.186	1.49
2.073	1.385	1.332	1.316	1.461
0.587	0.641	0.69	0.875	1.23
0.912	0.32	1.29	0.996	
1.186	1.119	1.16	1.005	0.003
0.429	1.091			
1.159	0.625	0.985	1.613	1.054
0.588	0.534	1.004	1.325	0.086

0.961	0.361	0.062		7.417
1.2	1.488	0.986	0.903	0.753
0.318	0.797	0.63	0.678	
		1.043		
1.38	0.971	0.439	0.934	1.148
1.223	0.977	1.153	1.225	1.374
1.205	1.155	1.041	1.15	1.026
			1.031	0.605
1.028	1.018	0.937	0.768	0.48
0.36	0.275	0.888	0.775	0.641
1.234	2.012	1.751	0.901	1.154
0.926	0.558	0.892	1.523	1.818
0.644	0.502	0.976		0.807
0.862	0.58	1.162	0.756	0.625
2.019	2.569	0.856	0.172	
1.161	0.861	1.754	1.526	0.994
0.49	0.811	0.447		
0.552	7.846			0.472
0.856	0.735	0.833	0.823	0.473
0.493	2.054	0.449	0.049	0.56
	1.091	0.687		0.523
1.791	0.015	1.789	0.003	
1.181	1.126	1.156	1.032	0.384
1.313	0.709	1.014	0.479	0.912
1.126	1.338	1.191	0.907	2.539
0.721	0.513	0.592	1.211	0.756
0.889	1.845	0.907	1.419	1.056
1.103	1.853	1.459	1.041	0.833
1.107	1.07	1.039	0.928	1.096
1.392	0.755	0.741	1.29	1.05
1.736	1.324	1.149	1.375	1.189
1.138	1.036	0.907	0.991	2.043
1.069	1.268	0.877	0.521	0.851
0.965	1.041	0.952	1.17	0.629
1.246	0.574	0.824	1.219	0.987
0.284	0.2	0.336	0.478	0.287
0.696	0.69	0.845	1.723	1.879
0.877	0.897	0.963	1.127	0.867
0.504	0.303	2.057		0.296
0.902	0.577	1.085	0.997	0.769
0.805	1.089	0.979	0.981	0.881
0.59	0.442	0.346	0.414	0.59
1.216	1.174	1.29	1.006	0.548
0.926	0.896	1.04	0.958	2.045
1.888	0.889	2.383	0.704	3.793
1.009	0.784	1.358	1.071	1.269

0.763	0.865	0.757	0.684	0.934
1.104	0.026		1.263	1.047
0.912	0.68	1.015	1.115	0.793
0.901	0.7	0.957	0.735	0.475
0.844	1.458	1.453	1.264	0.912
1.593	1.134	0.853	0.86	0.717
1.081	0.405	1.381	1.315	1.862
1.172	3.975	0.847	0.477	
	1.311			
1.141	0.674	0.903	0.949	0.773
0.666	0.815	1.421	1.09	1.44
0.426	1.173		1.533	2.211
0.721		0.471		0.395
0.346	0.339	0.301	0.454	3.674
1.101	0.978	1.308	1.11	1.386
1.044	0.413	0.692	1.196	0.261
1.524	0.378	0.997		1.133
				3.571
1.099	1.24	1.375	1.14	0.706
0.777	1.388	0.883	1.431	0.637
0.926	0.947	1.117	1.055	1.347
0.994	0.445			
	1.187	0.914	0.095	
0.928	1.648	1.495	0.218	2.151
	1.378	1.134	0.707	1.136
1.224	1.019	1.213	1.008	0.365
1.064	0.353	1.198	0.812	1.122
1.189	0.544	0.469	1.521	1.393
0.883	1.047	0.676	0.989	0.242
1.594	1.187	0.9	1.105	0.941
1.248	0.864	1.44	1.266	1.071
				0.913
0.807	1.375	1.193	0.359	1.111
0.823	0.727	0.418		0.902
0.822	1.122	3.238	0.363	0.739
0.841	0.773			1.326
0.942	1.087	0.568		2.089
	0.713	0.447	0.763	2.787
0.965	1.11	1.328	0.978	0.287
0.828	1.058	1.747	1.362	1.13
1.168	1.022	0.908	0.943	0.633
1.464	1.763	1.433	0.846	1.107
0.984	2.515	1.396	3.072	1.737
0.662			0.391	1.414
3.857	0.166	2.371	0.172	0.116
0.748	0.823	0.829	0.68	1.22
0.831	0.818	1.035	1.037	0.771
0.594	0.86	0.596	1.141	

0.997	1.346	1.145	0.76	1.306
1.257	1.249	1.512	1.598	1.482
0.476	0.535	1.049	1.076	1.751
0.66		1.236	1.191	0.404
0.663	0.919	0.63	0.779	1.265
1.117	1.397	1.16	0.867	0.819
0.895	1.065	1.353		1.559
1.52	0.412	2.042	1.074	1.068
0.904	1.042	1.105	1.417	1.345
1.632				0.92
		1.001		
0.672	0.607	1	1.196	2.5
			5.786	0.716
1.879	0.698	0.654	0.433	1.417
1.16	0.696	0.881	1.301	1.208
0.932	1.074	1.935	0.573	0.728
		1.094		
1.217	1.216	0.982	1.152	0.326
1.151	0.905	0.644	0.9	0.106
0.68	0.392	0.613	0.229	0.614
5.26		0.253	1.705	1.742
0.774	0.168	0.859	1.783	2.501
1.111	1.647	2.186	1.481	1.797
1.08	1.418	1.316	0.831	1.176
1.093	0.471	0.733	1.203	1.369
0.898	1.782	1.32	0.178	1.104
0.696	1.023	0.795	0.718	0.878
1.08	1.104	1.225	1.077	0.522
	0.748			
			0.429	3.162
			1.016	0.76
0.707	1.279	1.642		3.151
0.867	0.819	0.956	0.797	1.322
1.676	1.69	1.243	0.86	0.468
1.116	1.264	0.904	0.563	0.771
0.548	0.958	1.289	0.475	0.465
0.875	1.16	1.323	0.828	
0.5		1.164	0.632	0.931
1.041	1.242	1.742		
0.621	0.565	1.826	1.043	0.595
1.027	0.546	0.388	1.146	0.815
	0.561		0.995	

1.076	0.427	0.929	1.541	1.947
1.135	0.985	1.011	1.012	0.799
0.978	1.638	1.611	1.244	0.769
0.823	0.737	1.044	1.369	1.321
0.937	0.752	0.741	1.128	0.855
1.057	1.033	1.098	0.892	0.941
0.851	0.515	1.138	1.394	1.191
0.841	0.551	0.971	0.638	1.686
0.615	0.61	0.527	1.15	
				0.905
0.498	0.748	0.886	0.786	1.032
0.069	1.303	1.043	1.18	1.122
0.854	0.897	0.285	0.62	0.62
0.588	1.294	0.827		
0.747	0.844	1.383	1.282	0.703
1.257	1.265	1.126	0.993	2.747
	0.941	1.106	1.319	
		1.179		
1.316	0.936	0.595	1.148	2.11
			0.968	
0.745	1.226	1.376	1.033	0.826
1	1.512	1.193	1.607	0.127
1.234	0.742	0.792	0.75	0.592
0.823	1.117	0.775	0.898	0.222
0.864	1.007	1.078	1.007	0.642
1.49	1.09	0.697	1.625	1.047
1.079	0.627	1.313	0.95	1.234
1.199	1.572	1.257	1.186	1.1
		1.109		
0.991	1.619	1.531	0.449	0.666
			0.549	2.621
1.044	1.322	0.769	0.928	1.407
0.907	0.699	0.924	1.28	2.211
1.049	0.607	3.527	0.335	0.469
0.595	1.646	1.205	0.901	1.366
0.754	0.921	0.629	0.778	0.661
1.028	1.823	1.322	0.944	0.957
1.137	1.075	1.144	0.766	0.933
0.722	0.86	1.276	1.037	3.085
1.163	0.858	0.626	2.158	1.269
0.992	1.281	1.661	0.804	1.724
1.382	1.525	0.976	0.655	
1.069	1.485	0.998	1.387	0.301
			1.759	
0.97	1.122		1.287	1.634
0.889	1.552	0.943		1.149

	0.676		0.603	1.567
0.059	0.298			4.049
0.295	1.109	0.377	0.028	2.362
1.614	1.168	1.179	1.047	1.66
0.652	0.869	1.382	0.226	0.132
1.644	1.886	1.62	1.141	
0.938	1.724	1.299	0.758	1.202
0.576				
		0.706		
1.04	1.358	0.666	1.108	0.525
1.314	0.323	0.38	1.242	0.85
0.77	1	0.821	1.74	0.696
0.363	0.309	0.272	0.146	0.273
0.37	1.359	1.067		0.588
0.918	1.009	1.54	0.955	
1.118	1.125	0.988	1.021	0.614
1.066	1.073	1.028	1.237	0.926
0.496	0.885	0.609	1.293	
1.082	1.205	1.037	1.253	0.774
1.042	0.951	1.212	1.216	0.516
0.665	1.286	0.347	0.197	0.82
1.864	0.78	0.955	0.762	0.415
1.173	0.924	0.942	0.989	0.619
0.879		0.822	0.454	1.471
1.012	1.772	1.234	1.75	0.863
1.094	1.149	0.734	1.311	
0.643	0.91	0.79	0.635	0.853
0.234	1.353	1.274	1.246	0.364
1.001	1.96	1.389	1.145	1.485
5.325	0.147	0.18		0.22
1.15	1.142	1.135	1.565	0.377
0.534		0.719		0.535
0.084		0.435	1.265	0.462
1.077	1.16	0.805	1.005	0.418
1.518	2.269	1.324	0.345	1.674
0.765	0.42	0.896	0.938	2.408
				0.984
1.127	2.151	0.851	0.851	
0.93	1.077	1.72	0.984	0.898
	1.318	0.534	1.072	
1.135	0.819	1.177	1.4	1.139
0.703	0.653	0.269	0.557	1.442
0.695	1.505			
0.912	0.794	1.488	1.466	2.736
1.136	0.787	1.283	0.733	0.381

1.328	1.064	1.217	1.379	0.656
0.747	1.018	0.492	0.788	0.571
	0.69	0.988		
0.74	0.745	1.538	0.924	0.697
	1.485	0.943	0.607	
0.994	0.721	1.137	1.175	1.304
	0.648	0.871		
	0.809	0.355	0.832	1.11
1.054	0.948	1.065	1.116	1.191
	2.448	2.171	1.012	1.296
0.755	0.532	0.25	0.156	1.097
0.761	0.727	1.215		1.011
2.357	1.256	1.953	1.082	1.413
2.059	0.896	1.165	0.781	1.393
0.857	0.39	0.723	1.142	1.341
0.89	1.166	0.786	0.381	0.932
0.256	1.23	0.921	1.047	
0.869	0.465	1.164	0.571	
2.012	4.146	0.159	0.222	0.349
1.295	0.636		1.289	
1.803	1.559	1.356	2.134	1.358
0.928	1.113	0.224	0.961	0.763
0.976	1.265	1.352	1.001	0.979
0.711	0.847	0.465	0.695	1.025
0.641	1.292	1.655	1.316	0.709
1.325	2.457	1.239	1.122	1.419
1.551	1.702	1.437	1.105	1.713
0.619		0.247		
1.807	0.318		0.46	0.441
0.974	0.931	1.401	0.981	1.166
1.055				
0.46	1.106	0.719		1.079
0.72	3.147	1.782	0.725	1.888
0.613		1.202	1.476	0.237
1.979	0.576	2.016	0.514	0.142
1.041	1.873	1.146	0.84	2.277
6.898	1.481		1.327	1.307
0.963	1.164	0.886	0.617	0.654
1.124				1.592
	1.771			
1.08	0.895	1.081		0.638
1.516	1.246	1.663	1.115	0.574
				0.9
0.793	0.671	1.009	1.131	1.848
1.324	1.523	1.113	0.827	0.906

0.971	0.672	0.623	1.306	0.312
1.03	1.372	1.323	1.812	0.782
0.841	1.086	0.81	0.773	0.375
0.799	0.793	1.408		0.794
				1.368
	1.447	1.537	1.602	
	1.425	0.672		
			1.438	
1.187	1.361	1.177		1.238
1.054	1.065	0.931	0.78	0.68
1.365	1.023	0.687	0.97	
1.479	0.926	1.429	1.345	1.698
0.441	1.467	0.497	0.22	1.022
1.133	0.764	0.729	1.125	1.441
1.32	1.025	0.879	0.445	
1.177	1.457	0.86	1.086	0.875
0.421	1.238	1.39	1.083	0.107
0.205				0.454
1.179	1.117	1.127	0.691	0.554
0.907	1.035	1.036	0.693	0.628
1.053	0.681	1.252	1.305	1.491
0.753	0.358	0.401		
1.013	0.844	1.117	0.921	0.786
2.149	1.405	1.967	1.57	1.009
1.935	1.342	0.803	0.69	1.127
0.674				1.466
1.003	0.344	0.964	1.249	0.975
1.28	2.176	1.491	0.355	
0.869	0.819	0.908	1.022	2.288
1.378	1.206	0.187	0.728	0.752
1.183	0.753	0.62	0.976	0.863
1.005	1.094	3.463	1.59	0.94
0.92	1.336	1.577	0.844	1.091
0.767	0.653	0.752	0.564	1.671
1.29	1.179	1.502	1.132	1.196
	0.868	0.541	0.857	
0.33	0.323	0.214	0.137	2.057
1.173	1.997	1.259	1.412	0.581
1.356	1.423	0.73	1.32	0.68
1.21	1.004	1.085	0.912	0.408
1.025	0.921		1.045	1.013
0.939	0.823	1.569	0.943	1.995
0.94	1.086	0.571	0.269	0.585
1.223	0.584	0.459	0.586	0.921
1.166	0.931	0.923	0.89	0.363
0.893	0.951	0.874	0.823	1.293

1.371	2.123	1.201	0.829	1.111
				1.59
0.807	0.591	1.144	0.882	2.479
1.752	1.661	0.901	0.981	0.924
1.558	1.177	0.521	0.712	0.457
		1.096	1.21	0.7
1.436	1.027	1.928	1.255	0.595
0.932	1.451	0.457	0.919	1.511
2.028	0.963	0.504	0.944	0.493
2.075		1.777	0.555	0.338
1.001	1.137	0.689	0.857	0.802
1.136	1.077	1.408	0.982	1.154
1.034	1.128	0.856	1.259	1.473
0.852	0.661	0.87	1.019	
1.193	1.325	1.275	0.694	0.652
0.764	2.23	1.98	1.103	1.09
0.852	0.924	1.258	1.271	0.866
0.975	1.215	1.09	0.773	0.945
0.838	0.995	1.341	1.217	1.784
0.715	0.506	0.303		
1.171	1.066	1.244	1.254	1.547
0.672	1	1.374	1.431	1.579
1.307	1.126	0.898	0.943	0.12
0.978	1.355			1.046
1.055	0.623	1.206	1.167	1.099
0.476	0.348	0.328		0.83
0.865	0.799	1.038	0.917	0.832
1.052	1.448	1.189	1.236	1.46
1.056	1.076	0.597	0.544	0.857
0.785	0.517	1.015	0.907	0.874
1.398	1.637	0.941	1.849	1.164
0.502	1.179	1.495	1.209	1.824
0.429	0.494		0.607	0.403
1.075	0.694	1.527	0.564	1.798
0.714	2.138	1.309	0.629	0.923
0.736	0.656	1.696	0.986	2.395
0.91	0.694	0.611	0.645	0.88
1.063	0.879	0.861	0.73	1.556
1.519				1.643
			1.352	1.252
1.14	1.74	1.258	1.264	0.476
1.138	1.549	1.594	0.778	0.604
		0.839	0.784	
	0.081		15.015	0.21
0.48	0.313	1.185	4.815	0.552
0.956	0.937	1.312	1.28	0.677
0.931	0.982	1.039	1.18	0.717

1.641	1.06	2.456	0.777	1.813
0.879	0.804	0.673	1.662	0.592
1.004	1.107	0.895	1.622	0
1.033	2.014	0.988	0.785	0.937
1.678	0.754	0.609	1.384	0.517
0.908	2.452	1.459	0.579	
0.874	1.124	0.991	0.868	1.16
1.112	1.292	1.603	0.178	0.985
1.807	2.305	1.721	0.644	1.101
1.045	1.845	1.622	1.258	0.916
0.907	1.816	0.621	0.704	0.988
0.216	0.381	0.263	0.472	0.249
0.72	1.002	1.306	0.815	0.814
0.997	1.265	1.332	1.081	0.652
2.379	0.215		1.122	1.429
1.099	1.296	0.886	0.772	0.651
0.855	1.016	1.385	1.182	1.202
1.226	0.829	0.457	2.023	
0.515	0.412	0.473	0.78	1.141
1.002	0.65	0.961		
0.732	1.28	1.197	0.578	0.687
1.308	1.976	1.698	1.133	0.709
0.865	0.988	1.243	1.095	0.971
0.686	0.268		0.515	0.865
0.969	0.958	1.163	1.074	1.071
1.105	1.985	0.839	0.616	
1.071	2.129	0.131	1.562	0.19
	0.209		1.597	
0.949	0.722	0.904	1.131	2.002
		1.107	0.902	0.559
0.912	1.912	1.631	0.631	1.088
0.8	0.406	0.11	2.018	0.321
1.049	1.216	0.716	1.016	0.856
1.017	0.711	1.264	0.734	0.823
0.592	0.514	0.44	0.506	1.526
1.08	1.314	0.942	0.693	0.52
1.396	1.565	1.166	0.883	0.69
3.762	0.315	0.61	0.616	3.597
0.512	0.791	0.89	0.977	0.715
0.552	0.923		0.963	0.218
2.03	0.475	0.616	1.557	1.445
1.404	1.313	1.307	0.974	0.868
0.384	2.297	1.672	0.756	2.271
1.127	1.452	0.907	1.191	0.979
1.23	0.862	1.376	0.732	0.555
	0.358			
1.012	0.458	0.538	0.216	0.803
1.147	1.351	1.466	2.657	1.578

0.752	1.175	0.745	1.457	1.008
1.043	0.989	0.801	1.401	0.715
1.407	1.125	0.867	1.128	1.396
2.191	0.439	0.431	2.173	0.177
0.744	1.545	1.613	1.086	1.567
1.245	0.769	1.069	1.085	0.779
2.642	0.189	0.131	0.987	0.778
1.017	1.134	1.211	1.238	0.575
0.546	0.847	1.205	0.569	1.38
		0.83		1.974
0.841	0.742	0.992	1.212	1.24
2.02	1.488	0.65	0.571	1.463
	0.436			
			1.185	
0.473	0.232	0.292		3.573
1.772	3.293	1.619	0.743	1.845
1.615	1.159	0.398	2.025	
1.138	2.063	1.309	1.122	0.578
0.863	3.226			1.621
1.036	1.062	1.319	0.803	0.753
1.041	0.853	1.076	1.6	1.169
0.982	1.531	1.208	0.723	0.738
0.759		0.539	0.552	0.522
1.022	0.659	0.929	1.068	1.211
1.164	1.135	1.187	1.189	1.566
0.523	0.906	0.565	0.093	5.17
1.16	1.194	1.313	0.858	1.495
0.962	1.727	1.337	1.265	0.899
0.987	0.76	1.025	1.164	0.799
0.256	0.272	0.175	0.203	0.185
	0.921			
0.995	1.16	1.359	1.002	1.124
1.142	1.022	0.965	0.958	1.631
0.403	0.32	1.113	1.151	1.717
0.938	1.171	1.077	1.314	2.033
0.814	1.34	0.564	0.88	0.595
0.543	1.345	1.203		1.798
0.71	2.278			
0.5		2.157	0.375	0.288
0.728	1.079	1.148	1.074	0.682
1.148	0.94	0.612	1.251	1.781
1.264	1.669	1.288	1.071	0.438
	0.937			1.974
1.031	0.828	1.363	1.238	1.272
1.274	3.299		0.448	4.419
0.906	0.772	0.924	0.995	1.255
1.384	0.898	0.829	1.216	0.722
1.284	1.081	0.676	1.153	1.65

0.415	2.291	1.577	1.437	1.318
1.264	1.596	1.579	1.162	0.701
1.232	1.025	1.333	1.118	0.616
1.361	2.03	1.205	0.589	0.953
0.959	0.861	1.575	1.588	1.216
0.999	1.007	0.787	0.957	0.532
1.127	1.386	1.215	1.198	1.241
0.705	0.7	1.03	0.971	1.67
0.823	0.97	1.044	1.277	0.761
0.345	0.193	0.219	0.19	
1.078	0.755	1.119	1.343	1.205
0.393	0.833	0.263	1.893	0.638
0.64		0.546	0.642	
0.858	0.918	0.253	0.85	0.613
1.974	0.959	0.309	1.524	1.209
0.708		1.618	1.012	
0.969	1.234	0.874	1.136	0.641
1.121	1.344	0.624	0.988	3.033
			0.626	0.254
1.03	0.939		0.366	0.994
0.695	0.358		0.684	3.298
0.86	1.526	1.106	1.143	0.531
1.242	1.284	1.069	0.571	1.001
1.025	1.236	1.221	1.099	1.426
0.718	0.79	0.911	0.834	0.965
0.639	0.467	0.766	0.559	0.373
1.013	0.881	0.932	1.818	1.478
1.166	0.67		1.541	0.886
1.208	0.775	0.53	1.525	1.296
0.963	0.788	0.783	0.964	0.871
0.785	0.869		0.716	1.191
0.933	1.16	1.124	1.274	1.326
1.098	0.841	0.812	1.017	1.35
1.138	1.182	0.833	0.903	1.036
0.849	0.742	0.909	0.982	1.265
0.898	1.26	1.144	0.832	0.686
0.825	0.648	0.686	1.081	1.005
1.12	0.943	1.277	0.556	2.069
0.395		0.146	0.876	
0.398		0.317	0.507	0.628
0.91	1.022	0.802	1.066	1.251
1.279	1.074	1.217	0.778	0.989
0.309	0.545	2.593		1.18
0.821	0.822	0.526	0.769	0.037
0.605	0.684	0.979	0.829	
0.935	1.791	0.738	0.605	1.518
1.403	0.509	1.04		1.627
0.776	1.466	0.897	0.591	0.703
1.128	1.174	1.04	1.373	1.44

0.905			1.474	
	0.224	0.205	0.368	2.2
1.043	0.754	0.651	1.212	0.059
0.861	1.212	1.124	0.951	1.251
	0.925		1.066	
1.09	1.112	1.219	1.291	1.365
1.259	1.754	1.514	1.338	0.467
	0.602		1.143	1.44
0.858	0.51	0.685	1.391	0.397
1.01	1.143	1.235	1.098	1.89
0.791	1.266	0.712	0.787	0.534
0.894				
1.13	1.28	1.16	0.738	0.64
0.888	3.542	0.923	0.614	0.541
1.064	0.717	0.588	1.145	0.586
0.634	0.582	1.279	1.443	2.024
1.309	1.309	0.916	1.345	1.666
0.504	0.7	1.357	1.164	1.028
1.599	0.746	0.571	0.982	0.426
2.022	0.741	1.683	1.02	0.652
1.218	0.786	0.825	1.351	2.74
0.641	0.955	0.682	0.544	
1.128	1.569	1.519	0.852	0.492
1.414	1.256		1.088	0.407
1.309	1.431	1.218	0.466	1.282
0.575	0.711	0.466	0.729	0.385
1.091	1.584	0.932	2.32	0.806
1.052	1.722	1.139	0.828	0.623
1.352	0.882	0.772	1.789	1.35
	2.272			
1.178	0.813	1.376	0.556	0.565
1.193	1.215	1.41	1.435	1.766
0.741	1.281	0.982	0.931	1.438
1.084	0.896	1.045	0.932	0.97
0.778	0.976	0.893	1.232	1.057
0.041	1.746	0.936	0.408	2.937
0.965	0.753	0.772	1.178	1.208
0.818	1.175	1.196	1.364	0.802
	0.745	0.673	0.875	0.974
1.217	1.215	0.872	0.115	1.175
0.769	1.211	0.872		1.13
0.756	0.577	0.85	1.44	1.239
1.096	1.222	0.349	0.707	0.689
1.592	1.342	0.957	1.327	1.06
0.67	2.85	3.017	0.57	1.364
0.939	0.852	1.224	1.007	1.427
1.732	1.977			0.284
1.357	1.098	0.993	0.142	1.016
1.39		1.191	0.692	

0.766	0.847	0.442		
0.858		0.816	1.01	0.649
0.382	1.092	1.103	1.173	1.274
1.155	1.179	1.461	1.015	0.73
1.174	1.39	1.169	1.084	0.662
1.041	0.493	0.908	1.267	0.925
0.865	1.101	0.887	1.015	0.698
1.463	0.961	0.728	0.465	1.546
0.861		0.4		
1.031	2.06	1.318	0.944	2.087
1.265	0.915	1.161	0.976	0.967
	2.376	1.315		0.745
0.893	0.566	0.38	0.9	0.879
0.651	0.453	0.395		0.597
1.008	0.3	0.36	1.039	1.177
0.91	0.403	1.092	1.605	1.078
0.511	0.471	0.787	0.899	1.192
2.253	0.741	0.87	1.074	0.206
	1.163			
0.959	1.153	1.489	0.985	0.851
0.066	0.605	0.062	0.048	1.018
1.081	1	0.755	0.799	1.221
1.233	0.915	1.215	1.203	1.294
		0.777	0.708	2.645
1.31	0.957	0.976	0.933	1.133
0.732	0.969	0.852	2.027	
1.281	1.344	1.174	0.914	1.481
0.399	1.099	0.429	0.485	1.215
1.003	0.949	1.001	1.257	1.462
0.907	0.971	0.738	0.579	1.953
1.348	1.36	1.974	0.62	3.923
0.275	1.508	0.398	0.545	0.397
0.821	0.936	0.902	1.191	1.246
1.211	1.208	1.14	0.761	1.169
1.707	0.995	1.936	1.307	1.935
1.458	0.977	0.352	0.511	0.155
1.28	1.627	1.097	0.674	0.706
0.704	0.949	1.306	1.163	0.669
1.287	1.339	1.401	0.98	0.471
0.596	0.838	1.295	1.094	1.914
0.94	0.848	0.355	1.049	1.034
1.062	1.175	1.242	1.424	0.692
0.592	0.802	0.435	0.549	6.244
0.826	0.78	1.057	0.903	1.014
0.951	1.42	0.568		0.653
0.484	0.914	0.668	0.52	0.335
0.841	0.89	1.515	1.047	0.829

0.826	0.345	0.715		0.933
1.274	1.25	1.018	0.945	0.754
0.58	0.836	1.112	1.465	1.435
1.017	0.806	0.873	1.451	1.359
1.351	1.113	1.499	1.397	2.227
0.714	1.083	0.444	0.638	0.724
0.511	0.42	0.66	0.644	0.86
0.132	0.118	0.051	0.262	1.46
		0.533	0.441	
1.788	1.904	1.152	1.058	1.087
0.758	0.661	0.659	1.217	0.476
0.996	1.462	1.044	0.84	0.919
2.154	1.43	0.258	0.672	1.961
0.842	0.755	0.705	1.287	1.007
1.046	0.882	0.437	1.25	1.093
0.951	0.563	1.014	0.807	0.725
0.977	0.804	1.511	1.637	
0.926	1.24	0.941	0.679	1.163
1.052	0.755	1.309	1.596	0.331
1.438	1.454	1.187	1.028	1.475
0.832	0.968	0.863	0.461	1.192
0.699	0.594	0.511	0.34	2.262
0.656	0.655	0.78	0.538	1.625
1.667	1.272	0.952	1.719	0.807
0.679	0.923	0.981	1.675	0.872
1.924	0.755	0.637	0.871	0.387
0.683	0.549	0.456	0.37	0.571
1.013	0.931	0.892	0.894	1.468
1.337	1.351	0.935	1.265	0.877
0.649	0.525	0.946	0.68	0.556
1.05	2.531	1.323	1.156	2.174
1.193	1.639	0.82	0.908	1.01
0.71	1.216	0.242	1.08	1.408
1.092	1.466	0.974	0.868	0.75
1.033	0.991	1.176	1.089	1.25
1.194	0.922	1.545	0.954	2.075
1.148	0.864	1.175	1.61	1.286
0.537	0.583	1.063	0.602	1.941
1.551	1.061	1.163	1.638	1.318
	1.664			
0.631	0.94	1.024	1.439	0.607
0.705	0.246	5.128	0.827	0.717
0.637	0.367	0.84	0.796	0.904
1.309	1.029	0.967	1.395	1.247
0.255	0.085	0.567	0.422	0.166
0.904	0.618	1.046	1.074	1.239
1.254	1.504		0.925	1.314

1.401	1.328	1.137	0.828	1.447
1.565	0.841	0.968	0.721	1.471
0.879	1.125	1.037	1.423	1.207
0.516	0.997	1.159		0.957
1.175	1.1	1.167	1.094	1.025
1.065	0.993	1.183	1.151	0.876
1.937	0.338	1.765	2.572	0.14
1.144	1.263	1.754	0.959	0.739
0.776	0.772	1.178	0.992	1.593
	1.008	1.193	1.164	
1.215	1.337	1.272	0.935	1.338
1.463	1.136	1.17	1.115	1.21
0.962	0.894	0.979	1.471	1.226
1.241	1.355	0.974	1.39	1.496
1.25	1.365	1.31	1.24	0.84
		0.104		
0.862	0.416	0.943	1.074	0.949
0.798	0.815	1.262	1.266	1.376
0.789	0.724	1.197	1.35	0.892
0.594	0.544	0.658	0.353	1.083
0.464	1.683		1.374	
1.103	1.223	1.403	1.305	1.2
1.29	1.537	0.635	0.797	0.851
0.405	0.254	0.557	1.575	0.677
0.287	1.118	1.508	0.423	
0.641	1.13	0.542	1.556	
0.465	0.786	0.607	2.021	0.591
1.592	0.722	0.935	0.569	1.291
1.102	1.081	0.868	0.799	
1.028	0.674	0.817	0.679	1.629
1.151	0.988	1.057	0.858	0.605
0.87	1.29	1.673	1.818	1.475
1.531	1.218	0.921	0.831	1.085
1.622	2.764	1.387	0.96	1.04
1.706	1.128	1.133	0.977	0.632
1.201	1.121	0.78	0.98	0.816
1.171	0.863	1.226	1.023	1.481
0.502	1.152	0.84	0.986	0.763
				1.439
	1.171	2.642	0.309	
1.352	2.683	0.788	0.287	0.274
1.822	1.528	1.617	1.452	1.292
0.938	0.895	0.968	0.871	0.821
0.99	1.066	1.12	1.327	1.619
0.816	0.841	0.657	0.861	0.544
1.174	1.303	0.796	1.462	0.416
1.007	1.055	1.087	0.942	0.668

1.06	1.445	1.262	1.114	1.085
1.397	0.148	1.102	0.656	1.213
0.404	0.391	1.612	0.697	1.545
0.706	0.964	0.942	0.583	0.383
1.101	0.669	0.649	0.706	2.052
1.428	0.758	0.609	0.895	1.474
1.081	1.104	1.312	1.055	1.736
1.272	1.056	1.148	1.144	0.848
0.915	0.901	0.957	0.773	0.669
1.084	0.678	0.498		
1.184	0.373	0.874	1.147	0.813
0.368	0.26	0.578	1.746	0.616
0.604		1.406	0.964	0.338
0.91	1.191	0.674	1.452	1.008
	0.827	0.85		
0.97	0.934	0.895	1.038	0.493
0.954	1.029			0.757
1.071	1.081	1.283	1.115	1.219
0.849	0.733	0.705	1.284	0.976
		0.641		
0.985	0.995	1.213	1.567	
1.045	1.091	0.855	1.188	0.992
0.589	0.539	0.618	1.225	
1.093	0.948	1.331	1.153	0.766
	1.261	0.856		
1.308	1.181	1.374	1.45	0.882
0.768	1.292	1.129	0.68	2.634
1.296	1.081	1.134	1.171	0.828
0.758	1.006	1.172	0.761	0.665
1.109	0.855	0.607	1.288	1.446
1.386	0.713	1.16	1.329	1.38
0.861	1.183	0.893	3.259	2.003
0.749	1.24	0.819	0.551	
1.162	0.625	0.453	0.77	0.528
0.957	0.672	1.511	0.79	1.916
1.197		1.187	1.013	0.93
1.033	0.083	0.896	0.22	
1.363	1.385	1.359	1.198	1.45
1.131	2.417	1.267	0.593	0.989
0.923	1.099	1.241	1.083	1.348
0.782	0.853	0.563	1.366	1.538
		1.017		1.002
0.939	1.302			
1.1	0.824	0.985	0.916	0.857
0.78	0.846	1.497	0.847	1.603

0.799	0.931	1.33	1.632	1.281
	1.496	1.084		
0.452	0.881	0.833	0.859	1.68
0.673	0.465	0.205	0.581	1.015
0.827	1.068	1.126	1.221	0.946
1.063	1.108	1.06	1.096	1.101
0.812	1.057	0.829	1.245	1.585
1.422	0.835	0.741	0.92	0.802
1.239	2.136	1.497	0.973	0.748
1.437	0.827	0.757	0.863	0.614
0.261	1.036	0.591		0.945
0.785	1.046	0.858	1.065	1.596
1.182	1.195	1.255	1.019	0.993
	1.026	0.504	2.363	1.265
0.984	0.996	0.856	1.097	0.558
1.51	1.127	1.114	0.253	3.231
0.784	0.501	0.808	0.849	0.556
1.178	0.602	0.969	1.412	0.633
0.807	0.752	0.836	0.886	0.538
2.083	1.231	1.15	1.32	0.806
1.228	1.165	1.548	1.018	0.421
	1.453		1.126	1.311
0.872	0.924	0.853	1.044	1.745
0.838	0.947	1.038	1.078	1.573
1.125	0.884	1.193	0.861	2.111
1.105	1.884	1.454	1.037	1.513
1.16	0.682	1.4	2.015	1.497
1.404	1.351	1.71	0.83	1.117
0.631	0.907	0.941	0.825	1.252
0.902	0.429	0.965	1.119	1.432
0.832	1.178	0.844	2.057	1.784
1.244	1.02	1.31	1.278	0.842
1.075	0.776	0.59	0.92	1.115
1.579	1.357	1.006	0.574	0.984
1.051	1.537	1.603	1.184	1.821
2.243	0.867	0.605	1.185	0.712
	0.579	0.15		1.476
1.035	0.836	0.738	1.398	0.779
1.223	1.242	4.134	0.335	0.73
1.294	0.979	0.86	1.819	0.984
1.003	0.926	0.857	1.302	2.237
1.507	2.26	0.66		0.819
0.866	0.884	1.134	1.263	
1.77	1.219	0.472	0.868	0.978
				1.762

1.067	0.828	1.355	1.293	1.213
0.774	0.975			0.227
1.077	1.187	1.089	1.106	0.953
2.198	0.354	0.472	0.384	3.455
0.458	1.044	0.768	1.708	0.678
1.167	1.092	1.036	1.649	0.941
1.758	0.769	0.71	0.912	1.471
0.945	1.779	0.26	0.192	2.12
1.037	1.453	0.623	1.127	1.845
1.042	1.317	1.153	0.798	0.866
1.441	1.314	0.918	0.993	0.92
0.943	0.832	0.833	1.203	0.659
0.882				
0.973	1.08	1.455	1.241	1.554
1.2	1.344	0.831	0.748	2.68
1.61	1.779	0.776	1.104	
0.733	0.965	0.847	1.284	0.036
2.607	1.035	0.441	0.401	0.809
0.938	0.676	1.173	1.178	0.17
0.775	1.477	1.039	1.236	
0.768	0.815	0.907	1.235	1.362
1.175	0.607	0.779	0.86	1.265
1.331	1.019	0.711	1.614	1.578
	0.245			0.605
0.706	0.315		1.253	
0.94	1.265	1.897	1.43	0.568
1.147	1.088	0.543	1.055	
1.041	1.083	1.049	0.745	0.909
1.201	0.937	1.238	1.071	1.345
0.591	0.521	0.433	0.549	1.05
1.447	0.882	0.645	0.675	0.67
0.826	1.103	1.015	1.198	1.237
0.583	3.38		0.396	0.471
0.673	0.806	1.206	0.643	1.845
0.513	0.965	0.68	0.668	0.948
0.744	0.78	0.403		
1.243	0.585	1.362	0.605	1.181
0.715	0.541	0.536	0.927	0.562
0.853	1.141		2.298	0.159
1.258	1.231	1.304	1.072	0.94
0.485	0.281	0.584	0.679	
0.865	1.161	1.445		1.686
1.006	1.007	1.068	0.798	0.954
0.767	1.337	1.485	1.289	0.618
1.221	0.621	1.116	1.67	1.043
0.553	0.403	0.356	0.307	
0.792	1.489	0.343	0.745	0.248
0.555	0.02	0.887		0.071

2.328	1.389	0.778	0.886	1.28
1.029	1.113	1.131	1.106	1.558
1.471	0.669	0.616	0.772	1.723
0.505	1.045	1.109	1.625	1.466
0.953	1.164		0.295	
0.658	0.63	0.172	0.618	1.064
1.136	1.112	1.251	1.154	1.408
1.284	0.784	0.993	1.172	1.417
	1.287	0.584	1.16	0.454
0.959	0.822	1.254	0.976	1.048
		0.72	0.828	1.558
1.427	0.781	1.171	1.383	
1.123	1.339	1.295	0.721	2.405
0.468	0.422	0.425	0.408	1.697
1.339	0.913	1.175	1.495	1.887
0.989	0.93	1.07	1.838	0.279
1.166	0.895	0.816	1.081	1.869
0.439	0.194	1.193	1.141	2.028
0.665	0.674	0.687	1.267	0.657
0.67	0.925		0.84	
0.311	0.265	0.757	0.222	2.124
			1.92	
0.741		0.878		
1.091	1.591	1.175	1.26	0.649
0.35	2.901	2.226		1.095
	0.811	0.663	0.967	0.308
	0.595			0.732
0.776	0.624	0.676	1.277	1.196
1.041	1.907	1.521	0.973	1.264
1.175	1.587	1.126	1.081	0.302
1.128	1.033	0.737	1.212	0.701
0.145	0.182	0.718	1.902	0.734
				0.434
0.868	1.147	1.38	0.909	1.054
0.954	1.102	0.795	1.192	0.504
1.219	0.779	1.301	1.226	1.17
1.026	1.333	0.482	0.671	0.632
1.527	1.344	1.573	1.159	1.974
	0.557			
1.241	0.758	1.334	1.067	0.864
0.879	1.127	0.827	1.299	0.975
0.6	0.433	0.648	2.045	3.171
1.09	0.939	1.465	1.411	0.532
0.06	16.307	0.018	0.032	0.072
0.799	1.114	0.783	1.683	1.644
1.021	1.039	1.043	1.014	0.644
1.4	1.886	0.311	1.894	
1.2	1.049	0.929	0.973	1.331

	0.398	0.24	0.452	
0.578	0.535	1.059	1.049	1.272
0.541	0.68	0.528	0.694	1.338
0.892	0.717	1.992	1.806	1.148
1.261	1.235	1.311	0.829	1.334
0.987	0.983	1.217	1.392	1.101
0.961	1.038	0.922	1.425	0.268
1.268	0.618	0.907	1.539	1.015
0.9	0.91	0.98	1.198	1.299
1.733	1.21	1.202	0.669	1.097
0.679	0.52	0.626	1.453	0.999
1.382	1.315	1.514	0.815	0.566
1.109	0.871	0.97	0.781	1.777
1.208	1.587	0.934		
0.661	0.851	0.52	0.386	0.929
	0.103	0.088	0.201	0.176
1.228	1.173	1.224	0.672	0.707
0.494	1.895	1.292	1.166	0.711
0.522	0.615	0.437	1.351	2.35
1.152	1.209	0.654	1.079	0.758
1.145	2.113	1.555	0.837	1.136
0.741	1.389	0.343	1.485	1.197
0.854	0.619	0.437	2.209	1.587
0.868	0.271	0.751	1.062	0.944
1.348	1.835	1.377	1.068	2.351
0.837	0.851	0.835	1.337	0.521
0.476	1.003	0.511	1.225	2.425
0.85	0.978	0.924	1.02	0.394
0.931	0.739	0.978	1.012	1.304
0.997	1.137	1.206	1.129	1.149
0.772	0.753	1.566	1.129	0.4
1.018	2.282	1.048	0.554	1.643
1.073	1.497	1.505	1.099	1.64
0.616	0.777	1.788	1.074	1.008
0.876	1.308	1.162	1.575	1.062
1.085	1.011	0.824	0.942	1.875
0.85	1.507	0.817	1.494	0.466
1.417	1.123	0.963	0.533	1.232
			0.684	
	0.249		1.241	0.81
0.797	0.59	0.638	0.443	1.588
0.909	0.767			1.087
0.852	0.707	0.901	1.233	0.009
1.117	0.77	1.409	0.222	1.591
		1.408		0.702
1.033	1.287	1.028	0.852	0.445
0.365	0.609	0.289	1.31	0.452

1.351	1.915	1.111	1.629	0.609
	0.16			0.162
0.924	0.71	0.485	0.807	1.01
0.836	1.709	1.161	0.76	4.045
1.812	0.704	1.441	0.966	1.056
1.076	2.171	1.143	1.143	2.615
1.605	0.876	0.719	0.537	0.944
0.821	0.797	0.852	1.098	1.195
0.452				
1.473	0.739	1.196	1.684	1.619
1.069	1.022	0.993	1.524	0.297
1.993	0.4	0.704	0.856	0.721
0.928	1.273	0.695	1.178	1.259
0.627	0.411	0.498	1.173	1.083
0.989	1.443	1.429	1.204	1.457
0.591	1.343	0.92	0.623	
1.076	0.394	0.795	1.867	0.771
0.873	0.624	1.51	1.364	1.183
0.708	1.39		0.958	0.833
1.111	0.863	0.826	0.653	0.491
0.819	0.756	0.72	0.711	0.543
1.232	1.133	1.243	1.206	1.096
0.966	1.47	1.029	1.004	1.774
1.398	1.275	1.33	0.297	0.108
0.924	0.684	0.852	0.773	1.369
1.364	1.714	1.206	0.803	1.713
1.741	0.906	0.571	0.541	1.643
1.005	1.235	1.181	0.801	2.514
0.836	0.662	0.944	2.043	0.888
1.016	1.099	0.989	0.999	0.428
1.003	1.124	0.815	0.634	0.733
0.438	0.024		0.33	0.914
1.036	0.587	0.295	0.752	0.556
1.03	0.839	1.321	1.155	0.294
1.131	1.35	1.145	1.04	1.085
			0.391	
1.548	1.217	0.928		0.632
1.279	1.37	1.333	1.321	0.823
1.205	1.127	0.758	1.215	1.4
1.047	0.635	0.545	0.949	0.862
0.902	0.733	0.539	0.396	1.628
1.081	1.026	0.898	0.841	0.921
0.279		1.694	0.555	
1.041	1.334	1.133	0.994	0.764
1.306	1.432	0.939	0.85	1.535
0.314	0.414			0.097

1.189	1.057	1.097	1.432	1.719
1.04	0.555	0.577	0.914	1.657
0.861	0.872	1.24	1.207	1.119
1.289	0.637	1.562	0.768	
1.116	1.186	1.469	0.955	0.792
1.513	0.663	0.666	1.085	1.454
0.863	1.402	1.147	1.245	0.327
0.49	0.44	0.488	0.507	1.485
0.596	0.487		0.873	
0.228	0.293	0.959	0.789	
1.23	1.611	2.766	0.93	1.033
1.13	1.083	1.346	1.042	0.858
1.357	1.222	1.28	0.894	0.774
2.075	0.839	1.246	1.001	1.016
1.389	1.023	0.533	0.685	0.436
	1.678	0.64		
				3.016
			0.841	1.307
1.139	0.984	0.877	0.699	1.248
0.83	0.633	0.834	1.466	0.843
		1.895		
1.408	1.191	1.335	1.344	0.319
0.895	1.041	0.822	0.837	1.327
0.92	0.811	1.326	1.216	1.277
1.08	1.043	1.265	0.769	1.882
0.825	0.479	1.234	1.657	1.494
0.917	0.856	1.021	1.334	1.663
0.906	1.627	0.894	0.741	0.757
1.149	0.719	0.292	1.032	1.036
1.207	0.952	2.451		1.508
0.57	0.862	1.422	1.146	0.854
0.961	1.213	1.06	0.874	0.629
0.763	0.233	0.128	0.268	0.044
				0.567
0.828	0.642	0.667	1.15	1.212
1.038	1.294	1.288	1.038	1.699
1.312	1.682	1.337	0.721	1.154
1.144	0.95	1.01	0.782	1.032
0.907	0.835	1.053	0.913	0.38
0.914	1.182	0.385	1.566	1.054
0.155	0.25	0.166		
0.794	0.745	0.765	1.037	
1.028	0.735	0.744	1.608	1.596
0.688	0.571	1.335	1.397	1.403
1.68	0.848	0.335	0.654	1.074
0.185	0.201	0.248	0.271	2.355
0.455	0.508	0.204	0.518	1.057

0.81	0.805		1.418	1.329
0.848	0.775	0.843	0.843	1.589
1.13	1.328	1.116	0.818	0.729
0.46	1.078	0.641	1.135	1.75
1.801	0.188	0.087	0.117	
0.871	0.986	1.12	1.109	0.989
0.824	0.657	0.975	1.145	1.783
1.355	0.751	1.091	1.098	2.032
1.381	0.123	2.044	0.118	1.285
0.988	1.006	1.257	0.93	0.357
1.267	1.247	1.047	1.186	1.608
0.33	0.521	1.739	0.836	0.953
0.999	1.101	1.173	1.294	1.245
	1.904	1.386	1.151	
0.256				
0.503	0.66	0.318	0.319	1.872
0.651	0.313	0.401	0.715	0.189
1.229	1.02	1.202	1.095	1.604
1.809	0.549	1.182	1.423	1.629
1.468	1.205	1.458	1.327	1.96
1.921	0.778	1.535	1.186	1.891
0.824	1.431	0.757		1.893
				0.074
0.514	0.757	0.294	0.654	1.299
0.895	0.839	1.138	1.404	1.13
				0.438
0.94	1.034	0.375	0.279	1.721
1.228	1.304	0.983	0.987	0.742
	0.424		0.73	0.975
1.34	1.085	0.78	0.734	0.683
0.639	0.481	1.049	0.903	0.962
1.065	1.038	1.094	1.022	1.251
		0.122	0.149	1.287
0.843	1.202	0.335	0.502	
1.072	1.249	1.324	1.264	1.169
1.764	1.269	1.43	1.568	1.605
2.549	0.418	0.599	0.742	1.013
0.844	1.094	1.067	0.702	1.537
0.795	0.761	0.928	0.92	0.854
0.469	0.414	1.304	1.757	3.687
	0.445	0.186	1.535	
1.237	1.239	0.488	0.713	0.555
1.29	0.609	0.983	0.866	0.993
1.075	1.699	1.499	1.406	0.008

1.156	1.416	1.778	1.298	1.907
0.702	1.385	1.263	0.951	2.24
0.494	0.758	1.266	1.871	0.416
1.031	0.857	0.902	0.943	0.806
0.08	0.098	0.084	0.148	1.064
1.28	1.389	0.793	1.262	1.048
1.125	1.393	1.313	0.904	0.751
0.455	0.501	0.467	0.49	0.52
1.196	1.262	1.461	1.188	0.106
0.657	0.667	0.366	0.963	
1.868	0.633	0.607	0.972	
0.03	0.025	0.759	0.043	1.036
1.381	1.128	0.762	1.107	1.398
1.39	1.247	0.991	1.049	1.951
		1.78	0.952	
0.986	0.74	0.926	0.663	0.265
1.439	1.379	1.547	1.348	1.52
0.687	0.642	0.624	1.534	1.869
0.488	0.583	0.502	1.7	2.601
0.615	0.646	1.182		
0.277	2.226	0.52	0.746	2.902
1.516		1.072		1.073
0.918	1.005	1.376	1.29	0.619
		0.456	0.367	
0.502	0.975	0.46	1.03	1.742
1.023	1.118	0.882	1.434	1.505
0.959	0.726	0.732	0.829	0.835
0.943	0.994	1.386	1.328	0.799
1.207	0.479	1.07		
1.15	1.11	0.988	1.156	1.432
0.189	0.3	0.205	0.337	0.233
0.646	0.517	0.771	0.92	0.93
1.088	1.065	1.068	0.723	2.28
1.436	0.72	1.918	1.616	1.658
0.971	1.107	0.978	1.136	1.417
0.387	0.575	0.343	0.55	0.451
0.971	0.655	2.046	1.58	1.059
1.161	0.978	1.235	1.519	1.766
1.064	1.434	1.493	1.062	0.919
1.155	1.039	1.247		0.39
1.072	1.042	1.387	1.003	1.577
1.211	1.85	2.147		1.364
1.897	0.741	0.674	1.352	0.726
	0.58		0.779	1.334
0.98	1.58	0.991	0.903	0.525

0.687	1.056	1.433	1.227	0.711
1.157	1.515	1.119	1.018	0.938
0.996	0.618	1.223	1.027	1.562
1.191			0.696	0.444
1.245	0.974	0.895	1.251	0.465
1.441	0.579	1.041	0.951	2.723
0.92	1.345	0.33	0.556	0.542
1.003	0.613	0.592	1.362	1.296
1.077	1.32	1.519	0.676	1.96
0.413	0.867	0.365	0.284	0.742
0.798	1.027	1.21	0.763	1.213
1.14	0.561		0.669	0.698
1.296	1.057	0.922	1.07	1.461
0.82	2.653	0.998	1.276	2.017
1.2	1.101	0.818	0.283	
0.052	2.588	1.145		1.009
0.709	0.868	1.657	1.517	1.711
2.441	1.29	0.897	0.958	0.684
0.776	0.771	0.886	0.589	15.551
1	0.717	1.193	1.511	1.55
1.372	1.335	1.144	0.875	0.58
0.831	1.01	1.645	1.009	0.534
0.781	0.719	0.779	0.813	1.358
0.266	0.595		0.876	7.396
1.085	0.892	0.892	0.985	0.714
1.027	0.921	1.153	1.163	1.389
1.091	0.86	1.046	0.998	1.246
1.519	1.251	0.663	0.828	0.928
0.97				
1.274	1.186	1.097	1.423	1.319
1.042	1.489	1.027	0.498	1.901
2.046	1.147	2.246	0.605	1.483
		0.219	1.577	1.284
1.181	1.686	1.274	0.592	0.478
1.561	1.862	1.146	0.838	1.49
0.149				
0.673				1.283
0.829	0.758	0.363	0.39	0.269
1.276	0.65	0.681	1.432	0.456
1.299	1.378	0.887	1.049	1.477
1.363	0.956	0.559	0.812	1.184
1.103	1.267	1.278	1.136	0.523
0.923	0.487	0.673	0.982	1.838
0.688	0.516	0.895	0.582	2.414
1.08	0.795	1.108	1.036	1.244
1.204	0.888	1.653	1.219	0.974
0.921		1.607	0.159	1.476
1.153	0.82	0.843	0.979	1.518

1.032	1.266		1.033	0.216
0.862	0.784	0.698	0.728	0.412
1.344	0.81	0.448	0.978	0.789
	0.116	0.334	1.094	1.38
1.097	0.633	0.969	1.516	0.314
0.835	1.082	0.748	0.917	1.032
			0.121	
1.232	1.002	1.049	0.288	1.085
0.863	0.847	0.597	0.841	1.364
1.059	1.191	1.352	0.852	0.238
0.717	1.265	1.077	0.561	0.603
1.635	1.437	1.526	1.252	2.066
			0.955	
1.123	1.085	0.739	0.84	0.972
	0.475			
1.225	1.284	1.119	0.978	0.315
			0.26	
1.169	1.587	1.394	0.996	0.374
1.142	1.161	0.791	1.281	1.498
1.129	1.157	0.887	0.961	0.781
0.903		0.429	0.54	0.944
0.445		1.288		0.712
			1.37	
0.977	0.617	0.653	0.975	1.304
1.044	1.808	0.538	0.633	0.938
1.104	0.972	1.013	0.846	1.924
1.625	2.293	1.048	0.657	0.872
1.5			1.205	1.329
	1.44	1.878	0.527	1.044
1.034	1.666	1.759	0.623	0.579
0.913	1.002	1.618	0.65	0.969
1.615	1.531	1.311	1.085	0.607
0.482	0.278		0.163	0.297
1.292	1.412	1.231	1.138	2.07
0.867	1.643	1.429	0.237	0.968
0.943	1.606	0.92	1.055	0.875
1.346	1.741	2.144	1.265	1.313
0.43	0.582	0.438	1.196	1.112
1.253	1.218	1.265	0.889	1.827
2.339	0.627	0.424	1.149	1.137
1.315	1.7	1.899	1.103	2.176
0.997		1.275	0.73	
0.891	1.005	0.663	0.959	1.079
0.892	1.035	0.796	1.204	0.816
0.896	0.958	0.851	0.635	0.859
0.711	0.844	0.483	0.924	0.726

1.51	0.953	1.162		0.525
1.25	1.173	0.385	0.93	0.307
1.201	0.916	0.958	0.926	0.46
0.95	0.88	1.185	1.146	1.63
0.885	1.042	1.102	1.258	0.454
0.918	0.811	0.772	1.098	
0.9	1.33	1.098	1.105	1.621
1.054	0.876	1.157	0.914	0.647
1.27	1.283	1.36	1.053	1.591
	0.811	0.746		1.092
0.793	0.935	1.487	1.467	
1.007	1.295	0.907	0.911	1.519
0.758	1.65	0.856	0.511	1.445
0.683	0.621	1.433	1.029	1.26
0.832	1.319	1.001	0.933	0.466
0.786	0.533	0.651	1.297	0.594
0.968	0.715	0.962	1.12	0.401
				3.631
0.735	0.589	1.158	0.911	1.137
0.646	1.44	1.567		1.622
0.994	1.089	0.953	1.22	0.979
1.209	1.65	1.321	0.811	1.027
1.444	1.673	0.728	0.365	0.89
0.775	0.596	0.289	0.447	0.737
1.009	1.142	1.145	1.013	0.369
1.622	0.947	0.755	1.111	2.248
1.336	1.918	1.165	0.923	0.759
1.515	0.737	1.161	0.275	0.574
0.719	1.775	1.536	0.511	
1.773	2.425	1.377	0.961	1.272
1.269	1.3	1.15	1.003	1.155
	0.818		0.931	
0.674	0.54	0.572	1.57	1.908
0.689	1.053	0.715	0.697	0.221
0.508	1.276	0.184	0.479	
0.671	0.968	0.811	0.745	1.506
1.431	1.599	1.072	0.882	1.145
1.684	1.6	0.992	0.915	0.995
0.068	1.184	2.042	1.636	1.209
1.829		0.71		0.632
0.885	1.2	1.036	0.999	0.763
1.307	1.396	0.947	0.746	1.13
0.83	0.93	0.9	0.898	1.544
0.737	0.623	1.035	0.967	0.756
	1.028	0.769	1.114	
0.856	0.753	0.939	0.918	0.801
1.277	1.288	1.593	0.923	0.955

0.427	2.236	0.385	0.114	0.326
0.875	0.905	0.674	1.281	0.8
0.933	0.602	0.849	0.812	0.844
0.73	1.628	0.984	0.8	0.792
0.828	1.002	1.267	1.116	1.92
1.166	0.783	1.111	1.045	1.329
1.081	0.728	1.988	1.981	2.07
0.46	0.588	1.426	0.715	0.524
1.077	0.99	1.292	0.948	0.874
0.726	1.957	1.406	0.739	2.522
1.791	1.222	1.382	0.859	0.911
1.003	1.005	1.061	0.826	0.593
0.857	0.884	1.514	1.066	0.7
0.851	1.128	1.015	1.08	0.876
	0.792	1.774	1.251	0.439
1.009	1.398	0.923	1.284	0.695
1.151	1.098	1.036	1.182	1.368
	1.003	0.952		
1.422	1.86	1.123	1.467	1.816
1.864	2.58	2.057	0.605	0.28
1.218	1.845	1.276	1.442	0.924
0.678	2.358	0.106	1.077	1.296
1.192	0.859	1.066	1.289	1.099
0.774	0.788	1.281	1.092	0.838
0.913	3.89	1.2	1.211	0.817
1.167	1.347	1.28	1.128	1.089
0.549	1.118	1.748	0.516	0.807
0.365	1.384	1.158	1.091	0.787
0.732	0.553	1.045	1.25	2.027
0.861	0.923	0.947	1.089	1.245
0.978	0.213	0.674	0.44	0.331
1.137	1.991	1.141	1.083	0.699
0.886	0.95	0.958	0.892	0.841
1.192	0.91	0.962	0.974	0.338
0.984	0.555	0.462	1.005	1.588
1.201	1.355	1.114	1.271	0.872
1.205	1.08	0.594	0.557	0.956
1.317	1.115	1.28	1.451	1.014
0.952	0.941	0.942	0.716	0.809
0.325	0.61			0.634
1.411	1.597	0.998	1.097	1.416
0.898	0.766	1.221	0.965	0.787
0.332		1.463	1.154	2.035
0.94	0.911	0.661	1.119	1.062
1.136	0.43	1.087	1.093	1.094
0.981	1.136	1.602	0.987	1.304

1.072	0.828	1.507	1.365	0.613
1.115	1.795	1.279	0.829	1.978
1.361	0.582	1.579	1.21	0.615
1.13	1.096	1.165	1.043	1.449
				2.02
0.056	1.116	2.278	1.102	1.699
1.087	1.282	1.193	1.23	1.365
1.006	1.012	1.178	0.864	0.705
1.045	0.888	1.08	1.226	1.316
1.223	0.965	0.567	0.944	0.942
1.358	1.358	0.88	1.016	1.046
0.965	0.817	0.924	0.764	1.826
0.912	2.214	1.543	0.699	2.397
0.917	0.674	0.97	0.978	0.62
	0.766	1.103	0.952	
1.792	1.559	0.735	1.162	0.886
0.778	0.747	0.575	1.748	0.093
1.262	0.99	0.967	1.591	0.65
0.893	1.095	1.287	1.209	0.54
0.946	1.203	1.148	0.869	0.692
1.079	1.308	0.071	1.396	1.342
0.837	0.935	1.427	1.35	0.437
0.815	0.881	0.458	1.199	0.91
1.029	0.571	0.751	0.88	
1.023	0.708	0.61	0.555	1.443
1.195	0.986	0.669	1.26	0.872
0.711	1.154	1.321	0.9	1.346
0.639	0.782	0.923	1.173	1.557
0.326	0.206	1.172	1.057	1.495
1.276	1.384	1.053	1.028	0.657
1.065	0.739	1.471	1.088	1.425
			0.763	
2.321	0.313	0.645	1.089	0.307
1.202	1.09	1.254	0.963	0.67
1.6	1.14	1.759	0.671	1.785
1.278	1.348	1.006	1.256	1.087
1.407	1.337	0.69	1.158	1.574
1.145	0.972	0.832	0.984	0.586
1.364	1.243	1.081	1.383	0.503
1.207	1.379	1.835	0.557	1.512
	0.91	0.458	0.933	0.359
1.095	1.018	1.042	0.839	0.813
0.963	0.958	1.481	1.06	0.889
			1.094	1.233
0.381	0.377	1.124	0.382	1.59
1.078	1.073	0.971	0.919	1
1.039	1.339	1.395	1.417	0.606

2.168	1.097	1.603	1.486	2.146
1.239	0.648	1.224	1.061	0.847
0.401	1.441	2.182	0.962	0.598
0.686	0.288		0.48	2.108
1.18	1.525	1.07	0.959	0.423
0.993	1.116	1.121	0.943	1.867
0.88	1.097	2.089	0.574	0.424
0.972	1.117	1.154	0.692	1.073
2.233	1.128	1.439	0.514	0.797
0.665	0.676	1.138	1.369	0.418
0.59	1.037	1.079	0.84	0.968
1.058		1.022	1.074	
1.329	1.245	1.118	1.314	1.607
1.571		0.523	1.071	
1.118	0.746	1.405	1.378	1.408
0.742	0.716	0.573	1.017	1.123
0.582	0.758	1.35	0.932	1.135
0.993	1.141	1.08	1.275	
0.9	0.603	0.785	0.651	0.493
	0.878		1.896	0.593
	1.029		1.264	0.882
0.984	0.89	0.881	0.887	1.503
0.982	0.939	0.822	1.381	0.726
0.638	1.05	1.075	1.164	2.115
0.472	0.585	0.247	0.633	1.081
0.888	0.977	1.012	1.263	1.707
1.045	0.924	1.095	1.648	1.314
	1.076			
1.086	0.904	1.992	1.209	1.3
1.611	1.365	1.524	1.279	1.042
1.33	0.688	0.941	2.517	0.172
0.922	0.841	0.914	0.873	0.161
0.665	1.13	1.163	1.067	0.791
0.895	1.065	1.008	1.447	1.13
0.842	1.222	1.03	1.22	1.063
1.424	0.317	0.381	1.587	0.697
0.052	0.875	2.236		0.683
1.092	0.555	0.985	1.482	0.723
1.094	1.392		0.879	0.485
1.357	2.053	1.756	0.92	0.713
0.68	0.42	0.447	0.27	0.597
0.715	0.714	0.655	0.579	1.728
1.411	0.878	0.116	1.261	0.592
0.538		1.394	1.054	0.873
0.813	0.686	1.227	1.621	0.852
1.139	0.563	1.443	1.276	2.156
0.936	1.105	0.945	1.129	3.515

1.11	1.321	1.263	1.332	
	0.436		1.683	
0.915	0.943	1.195	1.225	1.426
0.55	0.909	1.489		2.725
1.227	1.182	0.577	0.968	0.452
0.977	1.446	1.206	1.377	1.567
0.867	1.005	1.129	1.634	0.485
1.305	1.124	1.222	1.131	0.492
1.747	1.972	1.821	1.014	3.07
			1.938	
1.202	1.048	1.032	1.047	0.632
0.963	0.942	0.769	0.971	0.87
0.968	0.96	1.33	1.212	0.479
0.937	1.236	1.075	0.854	0.803
0.956	1.447	1.008	1.044	0.686
1.226	1.33	2.949	0.887	2.402

A_CL_7	A_WCE_8	A_WHQ_9
0.938	1.177	1.066
1.8	1.964	0.818
0.119	0.013	
		0.647
0.872	1.076	4.282
1.896	1.137	1.019
	0.226	
0.887	1.313	2.008
1.727	0.531	2.023
0.841	0.989	1.561
1.426	1.165	1.234
0.236	1.197	3.037
1.072	1.388	0.621
0.267	1.665	1.54
1.432	0.649	0.282
	0.107	
2.051	0.527	0.602
0.975	1.343	1.095
	0.227	3.262
0.706	0.758	1.471
0.845	1.151	1.992
1.238	1.334	0.897
0.184	1.878	
0.837	1.322	0.972
	0.108	7.22
0.89	0.703	1.115
		0.15
0.104	0.183	
1.247	0.844	0.33
0.954	0.422	
	1.838	0.588
3.359		
		1.55
1.462	1.612	0.571
1.894	1.188	0.963
0.519	0.3	1.285
0.27	0.238	0.126
0.506	0.338	2.431
0.087	0.493	0.176
1.34	1.137	0.019
0.624	1.644	
0.675		
	0.595	

		1.144
0.635		3.424
0.499	1.645	3.127
		1.139
1.523		1.317
2.371	1.118	0.941
		1.303
0.456		
	2.163	3.602
0.756	0.63	0.827
	0.233	
0.641	1.045	0.624
1.262	1.174	1.344
0.379	0.486	0.418
0.938	0.975	0.641
1.117	1.372	1.499
1.218	1.748	1.022
1.32	0.677	1.3
1.2	0.626	1.61
1.539	0.86	0.984
0.63	0.544	0.098
0.645	1.296	0.657
		4.483
1.223	0.21	0.982
4.209	1.095	0.49
1.262	1.215	1.677
0.388	0.619	0.231
1.143	1.937	0.578
0.611	0.332	0.936
2.008	1.014	0.498
0.93	1.021	2.758
		0.224
1.894	0.794	0.749
0.609	0.931	0.687
0.008	1.627	10.842
0.055	0.964	2.245
2.29	1.145	0.703
0.362	0.459	1.046
1.561		0.927
0.079	0.485	
1.16		1.189
2.913	0.645	1.246
1.506	1.266	0.552
0.462		1.363

0.818	0.972	
1.735	0.964	1.033
0.35		1.579
0.898	0.767	0.844
1.332	1.149	1.408
1.394	1.608	1.851
0.829	0.767	0.208
1.03	1.068	0.893
1.79	1.247	0.552
	1.547	
1.078	0.847	0.96
0.709	0.9	0.831
1.055		1.062
0.731	0.971	0.818
0.723	1.153	
1.141	0.861	1.231
0.564		
0.965	1.283	1.581
0.861	0.589	1.778
	0.845	
1.787	1.563	0.979
1.809	1.239	0.969
0.328	1.259	1.054
1.315	1.097	1.254
	1.131	0.846
1.117	1.301	1.217
		3.063
0.415	1.144	1.573
2.482		
		0.539
1.084	0.888	1.228
0.581	2.386	0.37
0.442	0.507	0.841
0.491	0.44	0.483
2.132	1.388	1.426
1.469	0.483	0.584
0.511		
0.21	0.745	
0.934	1.775	2.608
1.321	2.098	
1.02	1.147	0.938
0.416	0.99	0.895
1.449	1.139	0.755
1.798	1.288	1.398
1.61	0.868	1.082
2.187	2.199	1.059

1.38	0.339	2.017
0.713	0.816	
0.509	0.468	0.415
0.433	0.122	0.042
0.306		0.809
0.779		
1.144	1.423	0.835
2.255	0.894	0.877
		1.257
1.317	1.062	0.689
0.263	0.288	0.292
3.023	1.305	0.136
1.436	1.182	1.249
1.228	0.678	1.665
2.023	0.642	1.451
1.267	0.834	0.647
2.559	1.241	1.39
1.626	0.946	1.265
0.485		
0.885	0.99	
0.295	0.347	0.375
1.354	1.072	0.949
1.089	1.244	0.945
1.003	1.02	1.513
1.105	1.103	1.161
1.304	1.184	1.83
0.279	2.639	1.862
1.149	0.88	0.833
1.996	0.925	0.937
1.33	1.548	1.262
1.138	1.039	0.735
1.868	1.036	1.277
0.836	1.616	1.145
0.347	0.872	2.074
0.553	0.541	0.805
1.356	0.884	0.431
0.232	0.703	4.337
0.984	1.204	1.056
		0.378
1.403	1.527	1.296
0.544		
0.984	0.809	0.839
0.772	0.869	0.865
	0.533	
0.996	1.26	1.642
1.646	1.033	1.6
1.045	1.127	0.6
1.233	0.945	1.626
1.146	0.966	0.679

1.725	1.444	1.361
2.208	1.128	1.912
1.672	1.234	1.238
1.806	0.488	1.043
0.401		2.461
0.703	1.164	2.046
1.635	1.032	1.57
1.593	2.539	2.262
0.169	0.584	
1.322	0.793	1.912
1.969	1.151	1.533
1.561	1.667	0.372
0.62	0.296	0.305
1.516	1.117	1.047
2.037	1.118	0.528
0.236	2.489	2.17
	1.641	1.469
0.57	0.918	1.222
2.283	0.714	1.639
0.472	0.838	1.238
1.14	0.927	0.914
1.416	1.34	0.962
1.536	1.28	1.848
0.963	0.74	1.434
0.483	0.843	1.214
1.351	1.15	1.055
1.01	1.199	1.012
0.661	1.741	1.347
1.375	1.16	1.278
	1.241	0.868
1	1.052	0.86
0.716	0.744	0.817
	0.546	
0.903	0.683	0.796
0.818	0.718	1.714
1.105	0.699	0.722
1.037	1.166	1.317
1.532	0.798	1.227
0.863	1.174	0.294
1.288	1.483	0.4
0.996	0.849	1.221
2.703	0.926	1.312
1.019	0.896	1.694
1.161	0.892	1.202
0.158	0.366	0.704

1.021	1.317	1.001
		0.187
0.374	1.06	0.279
0.781	1.079	0.849
0.626	0.854	1.275
0.573	0.657	0.755
0.888	0.552	0.434
0.478	2.036	2.763
1.749	1.14	0.64
1.388	1.201	1.034
0.167	1.218	3.833
2.065	0.28	3.854
1.215	1.319	1.515
1.077	1.708	0.876
0.012	1.731	1.58
1.434	0.758	2.257
0.239		0.493
0.22	0.402	0.234
0.256	0.683	1.176
1.786	0.832	0.97
0.626	0.463	0.914
0.235	0.28	0.441
1.074	0.478	1.18
1.372	0.854	0.887
0.968	1.04	0.971
1.212	0.785	2.383
	0.684	
0.794	1.198	1.925
0.696		
1.006	1.114	1.014
1.004	1.566	1.885
1.489	0.925	1.289
1.469	1.445	6.115
		3.346
		0.095
1.119	1.079	
0.52		
0.44	0.799	0.282
0.985		
0.808	1.087	0.777
0.565	1.008	0.784
1.607	0.484	
1.411	0.424	0.452
1.193	0.481	1.138
1.114	0.667	0.771
1.131	1.364	0.961
0.576	1.013	1.354
1.156		0.909

1.311	0.733	0.931
0.689	0.99	1.036
1.538	1.432	4.527
1.54	1.478	0.735
1.039	1.203	0.532
1.236	0.746	0.983
1.315	4.465	0.106
1.864		
1.252	0.874	1.182
1.224	0.873	2.44
1.599	1.206	0.506
1.249	0.895	1.221
0.863	1.219	0.881
0.585	0.449	3.244
		2.621
0.803	1.346	1.598
1.516	1.937	0.833
0.762	1.277	1.242
1.391	1.134	0.982
0.847	1.213	1.241
0.32		
0.889	1.206	1.032
1.046	0.765	1.055
1.597	1.891	2.526
0.584	1.062	1.325
2.352	2.218	0.16
		1.139
1.365	1	1.31
	0.706	
1.319	0.96	1.576
1.1	1.082	0.687
1.198	0.781	0.857
0.937	0.92	2.365
0.499	0.432	
0.783	0.732	0.608
1.12	1.047	2.512
0.349	3.018	1.361
1.971	1.663	1.646
0.227	0.085	0.899
1.166	0.908	1.301
3.474	1.072	0.341
1.205	0.926	1.156
1.181	0.838	0.564
0.257	1.568	4.254
1.333	1.022	0.811
		1.885
0.195	0.553	0.299
0.911		

2.071	0.48	0.561
0.644	0.5	6.062
1.276	1.221	0.747
	0.798	
0.76	0.654	0.558
	2.014	7.545
1.239		0.728
1.53	1.499	0.228
0.189	3.061	
1.188	1.244	1.958
1.466	0.701	0.898
1.287	1.362	0.694
0.864	0.801	0.715
1.316	0.969	1.047
1.086	2.078	1.186
1.183	0.991	1.056
0.409	0.295	0.438
0.26	0.658	0.587
0.531	0.663	0.752
2.502	0.795	0.656
0.944	1.924	10.585
0.464	1.446	
0.238		
1.83	1.094	0.69
	2.04	0.365
1.546	1.385	0.558
0.077		
2	2.29	4.564
2.827	1.182	2.046
0.432		
0.281	0.921	
0.166	2.624	1.133
2.063	0.905	1.311
0.769	0.66	0.661
1.13	0.571	0.776
0.736	0.967	0.259
0.665	1.276	1.658
0.785	1.156	0.648
0.8		1.218
1.443	1.116	0.685
0.478		1.79
0.409	0.288	
0.257	0.171	0.406
1.102	0.859	0.599
1.249	1.2	1.135
0.979	1.844	1.761
0.572	0.863	0.729
0.602	1.256	1.194

0.518	0.699	0.638
0.842	0.854	1.138
0.858		
2.073	0.806	1.434
1.375	0.999	1.105
		0.68
0.869	0.97	1.834
0.166	1.3	1.413
0.545	0.518	0.775
1.75	1.051	1.125
	0.267	0.441
1.43	1.152	2.025
0.714	0.715	0.242
1.028	1.116	1.726
1.13	1.111	0.946
1.075	0.907	1.067
0.714	1.668	0.098
0.594	0.654	1.959
5.947	0.129	7.601
0.96	0.85	0.151
1.243	0.741	1.482
0.693	1.05	0.507
1.484	0.685	0.989
1.882	0.533	2.096
0.539	0.546	0.574
0.555	0.906	2.979
0.557	0.575	0.121
0.824		
1.296	0.577	1.021
0.251	1.196	
0.693	0.65	0.472
0.884	2.168	2.205
0.163	0.382	1.371
1.067	0.24	0.46
0.911	1.057	1.61
0.664	0.662	
	0.854	3.483
1.44	0.991	0.541
1.275	0.894	0.558
1.246	1.239	0.494
1.545		
0.347	1.864	3.618
2.287	0.324	2.421
0.942	0.911	0.763
	1.103	

1.077	0.823	0.626
1.697	1.051	1.131
0.815	1.212	0.755
0.484	0.502	0.832
1.262	1.004	
1.334	0.989	0.614
0.882	1.146	1.758
0.939		
1.66	1.336	0.501
1.402		1.132
0.835	1.139	1.47
0.661		
0.288	1.118	
0.792	0.878	1.765
1.31	1.302	0.631
1.259	0.95	0.812
0.935	1.584	0.447
0.97	0.76	1.028
0.067	0.079	0.185
1.377	1.19	0.713
1.506		0.371
	0.983	3.123
1.254	0.928	1.252
1.043	1.23	1.399
1.151	0.868	0.969
0.871	1.065	1.55
1.554	1.266	3.535
1.071	0.932	0.999
1.253	1.384	1.613
1.382	1.215	1.094
0.333	0.59	0.576
1.89	2.003	4.659
1.109	0.372	1.391
	0.234	0.523
1.201	1.671	0.851
1.179	1.143	1.324
1.049	0.71	1.683
1.288	1.447	1.636
1.583	0.559	1.85
1.655	2.134	0.699
0.918	1.517	0.897
1.659	1.412	0.997
0.452		0.814
1	0.823	1.13
1.105	0.835	1.506

0.681	1.968	1.258
0.407	0.862	
1.116	1.236	1.03
0.81	1.027	1.832
0.458		0.447
0.799	1.285	0.879
2.028		
	1.581	1.02
		0.172
0.539		1.936
0.986	1.113	1.148
0.516	0.679	4.028
0.738	0.621	0.743
1.355	1.308	2.366
1.52	0.715	1.087
1.399	1.198	0.471
1.184	1.273	0.251
0.477		2.152
1.179	0.978	1.214
1.389	1.199	1.485
1.123	1.194	1.445
0.575	0.549	0.658
0.361	1.667	2.111
0.623	0.472	
1.463	1.091	0.571
0.032		6.124
	0.04	1.995
1.085	0.688	0.922
1.103	0.642	1.63
0.85	0.941	1.464
1.356	1.015	1.202
1.21		
1.015	0.756	1.089
1.11	0.934	0.66
0.877	1.036	0.686
0.344	0.744	0.77
1.525	0.929	0.711
	1.217	1.05
0.44	0.787	0.508
1.156	0.327	1.631
2.077	1.03	1.077
0.674	0.975	1.816
0.5	0.949	0.746
1.288	1.481	0.246
0.893	0.985	0.763
1.103	0.831	0.738
1.468	0.826	0.974
	0.908	0.555

0.293		1.506
0.853	0.885	1.178
0.46	3.131	
0.862	0.861	
0.609	0.909	0.402
0.98	1.174	1.401
0.981	0.646	0.939
0.571		0.768
0.705	0.925	1.272
0.628	0.578	1.886
1.136	1.154	0.808
0.991	1.481	0.655
	0.913	
1.217	1.178	1.733
0.499	0.705	0.594
1.326	1.313	0.614
6.064		0.49
1		0.33
0.56	0.973	1.222
1.367	0.938	2.441
0.425		
0.004	1.488	2.009
1.228	0.883	1.034
0.185	1.56	
0.895	0.923	1.143
0.839	1.69	1.123
1.055	1.06	1.801
1.163	1.013	1.208
1.408	0.883	0.934
1.387	1.087	0.845
0.477	1.008	0.512
1.123		
1.651	1.289	0.938
1.062	0.738	0.814
0.23	0.213	0.501
1.845	0.956	1.556
0.418	0.386	0.188
1.987		1.093
0.882	0.69	1.06
1.489	2.8	1.128
0.839	0.804	0.687
1.16	1.223	1.181
0.209	0.927	
1.25	0.795	1.163
0.928	1.111	0.644
3.842	1.494	0.832
1.184	1.514	0.986

0.78	1.125	1.419
1.084	0.241	0.053
0.952	0.903	0.468
0.131	0.352	0.178
1.5	0.831	0.458
0.663	0.797	0.764
1.928	1.374	1.08
1.05	0.915	0.345
1.011	1.469	0.886
1.984	1.423	0.498
1.903	1.533	0.204
	0.427	1.182
0.314	2.834	2.132
1.466	1.45	1.271
2.491	1.06	0.885
1.916	2.256	0.221
		0.729
1.208	0.685	0.799
0.425	0.51	0.516
0.912	0.926	0.825
0.857		
1.967		1.582
0.261		1.547
1.301	0.59	1.133
	0.135	0.099
0.488	1.362	0.988
2.219	0.592	0.854
1.21	0.764	1.004
1.039	1.249	0.604
1.329	1.182	0.845
		0.62
0.332		
0.542	1.025	1.454
0.991	0.493	2.47
	0.362	0.442
0.589	1.138	2.385
0.571	1.319	0.976
1.816	1.051	1.227
1.068	0.772	0.725
0.976	1.028	0.665
1.581	1.323	1.248
2.241		1.376
0.021	0.053	1.034
0.47	1.295	1.032
1.23	0.903	0.836
0.631	0.401	

1.455	1.603	1.491
	1.074	
0.67	0.53	1.211
1.098	0.505	0.744
0.872	1.428	1.266
0.777	1.612	2.164
	1.722	0.59
1.955	0.877	0.51
0.341	1.304	1.476
0.737		
1.431	1.234	0.249
0.714		1.027
0.135	1.797	0.847
1.144	0.976	1.356
0.784	0.566	1.55
1.396	0.86	0.853
0.297	0.275	0.203
0.075		
0.142	1.569	4.732
		2.164
1.539	2.536	1.514
1.604	1.164	1.145
	1.104	
1.15	1.28	0.658
1.026	1.478	0.575
1.047	1.388	1.766
0.573	1.856	1.908
1.46	1.103	1.023
	0.741	0.896
0.107		0.557
		1.365
2.417	0.755	0.396
1.166	0.531	0.342
0.973	0.718	0.437
0.526	1.365	1.638
0.9	1.081	1.993
0.33		
1.532	0.845	0.678
2.511		
1.325		0.924
0.43	0.954	
0.117	1.194	2.058
1.831		

1.411	1.084	1.162
1.256	0.889	1.26
1.671	1.033	0.7
1.596	1.467	0.852
0.874	1.142	0.448
1.017	1.005	0.926
0.122	0.145	0.511
0.632	1.558	1.43
		0.504
	0.876	
0.758	0.719	1.001
1.996	1.004	1.717
0.224	1.579	4.08
1.685	0.97	
1.27	1.129	1.166
2.629	1.449	1.985
0.512	1.527	
		0.301
1.607	1.697	2.13
	1.368	0.949
0.79	0.754	1.32
0.291	0.489	
0.71	1.016	1.89
0.869	0.589	1.041
0.983	0.844	1.471
0.981	1.926	1.273
0.743	1.406	1
1.159	1.146	1.068
0.751	1	2.506
	0.87	
0.385	0.571	0.634
1.264		0.788
1.005	0.799	0.537
	1.042	0.982
1.242	0.96	1.577
0.993	0.902	0.616
1.844	1.047	1.086
0.824	0.451	0.59
1.149	1.054	
0.86	1.07	0.84
0.924	1.112	0.444
1.314		0.866
0.891	1.741	0.558
0.206	1.378	
0.526		

1.737	1.701	1.111
0.973	0.93	1.014
	0.111	
1.696	0.71	1.103
0.427	1.406	1.246
1.006		3.042
0.491		2.981
0.696	0.871	
0.687	0.928	
		0.625
2.539	0.977	0.479
0.943	0.737	
0.309	0.29	0.226
0.586	0.918	2.252
0.69	1.363	0.284
1.358	0.678	1.729
0.395	0.461	0.554
1.185	1.199	1.616
1.543	1.46	1.759
0.867	1.204	1.149
0.658	0.969	1.114
	1.289	0.709
1.032	0.751	
1.309		
0.678	0.998	1.437
0.303		
0.319		0.685
1.604	0.797	1.201
5.806	0.243	0.175
1.031	0.483	0.215
1.252	0.793	0.779
1.173	0.044	0.347
1.371	0.888	1.011
	0.836	
0.928	1.06	0.973
0.973	1.049	1.283
0.497	0.426	4.894
1.19	0.86	1.411
0.508	1.01	1.247
1.064	1.172	0.981
0.244	2.082	
0.546	1.008	
2.687	0.622	0.654
1.205	1.46	0.19

1.562	0.792	0.158
0.378	0.697	
0.605	0.911	
1.15	1.721	1.25
1.776	1.071	1.043
2.678	1.714	0.407
0.683	1.287	1.228
2.328	0.818	2.559
0.078	1.244	3.52
0.277	2.8	
1.046	1.421	0.563
1.302	1.722	1.214
0.64	1.009	0.374
0.566		
1.104	0.995	1.103
	0.645	7.73
2.492	1.934	0.817
	1.393	
0.011	1.302	
0.311	1.107	1.818
0.932	0.632	0.606
0.661	0.991	0.681
1.055	0.926	0.607
1.675	0.758	0.888
1.593	1.733	0.854
1.373	0.766	1.176
1.25	1.612	0.291
1.501	0.481	2.004
0.926	1.182	0.67
0.69	1.263	
0.739	1.113	1.904
1.066	0.885	0.376
0.001		2.211
0.548	1.054	1.988
2.51	1.023	0.5
1.073	0.733	0.578
0.788	0.916	0.901
4.667		2.977
1.386	1.761	1.106
1.138	1.033	1.129

0.191	0.377	0.533
0.912		
1.943	1.396	0.393
0.18	0.918	0.693
1.139	0.605	0.281
1.226		
2.503		1.292
0.522	1.778	
1.777	0.799	0.81
0.258		
0.828	0.728	1.385
0.247		
0.868	0.981	
0.474	1.32	1.872
1.735	0.483	0.859
1.448	1.044	0.508
	1.604	5.961
1.311	0.959	1.554
1.03	0.918	1.662
1.412	1.204	0.707
0.463	2.004	3.953
1.434		
0.656	0.8	0.436
1.657	1.273	0.824
	0.675	0.752
1.007	1.417	0.803
0.616		
1.242	1.943	2.427
0.765	0.627	1.507
0.228	0.808	1.927
0.305	0.358	
1.109	1.088	0.732
0.378	0.977	1.117
1.515	1.039	1.257
0.33		1.677
0.082	1.223	7.549
1.187	1.523	1.262
1.545	0.952	1.556
0.565	0.807	0.857
1.379	0.935	0.495
0.957	1.467	0.691
0.225	1.331	2.501
0.995	0.352	
0.789	1.526	1.691
0.606	1.096	1.279

2.026	1.196	1.717
1.208	2.098	
1.774	1.521	0.571
1.103	1.131	0.298
0.385	1.079	1.619
		0.114
0.304	0.418	0.533
0.235	1.354	2.574
0.846	0.424	1.334
0.89	0.694	0.539
1.777	0.962	1.625
1.41	0.995	1.34
0.506	1.004	0.75
1.25	0.927	0.311
1.25	1.216	2.112
1.082	0.978	0.513
0.522	0.753	0.411
1.13	0.709	0.83
1.084	1.71	1.014
2.366		0.726
	1.043	
1.626	1.642	1.131
0.033	0.067	0.405
1.694		1.873
1.275	0.734	0.5
	1.586	3.539
0.573	0.887	1.266
1.679	1.147	1.231
0.517	0.909	0.363
0.364	1.204	0.968
0.475	0.365	0.927
1.22	1.005	0.573
	0.411	0.407
0.814	1.462	1.281
0.868	0.825	1.229
1.753	1.503	1.166
0.249	1.869	0.792
0.705	1.045	1.2
0.895	1.357	1.899
1.138	1.426	
0.613	0.213	0.559
0.794	0.808	0.504
0.4		
0.137	0.137	0.239
0.396	0.212	2.517
0.906	0.868	1.805
0.658	0.801	0.928

1.382	1.274	1.055
0.987	0.994	1.551
0.921	1.12	0.792
0.374	0.651	0.246
0.987	0.826	1.081
1.157	0.632	0.914
1.539	1.122	1.227
0.067	1.508	0.531
0.748	0.574	
1.263	0.34	0.708
0.976	0.42	1.695
0.204	0.158	6.534
1.038	0.89	0.595
1.563	0.609	0.98
0.354	0.796	1.111
1.154	0.77	2.2
1.207	1.612	1.283
1.311	0.348	
3.019	0.687	1.534
0.966	0.749	1.103
2.127	0.759	1.303
1.407	1.069	0.959
	1.261	
1.122	0.442	0.608
0.467		1.096
0.962	1.538	2.217
0.123	0.332	
0.819	0.691	0.585
	0.873	1.392
0.681	0.528	0.432
0.791		0.165
0.53	1.337	0.903
0.852	0.757	0.73
0.206	1.072	2.401
0.699	1.028	1.845
0.953	0.845	0.875
0.626	4.76	2.941
1.022	0.904	0.498
0.268		0.391
0.741	1.292	0.231
1.337	1.175	0.749
0.487	0.65	
1.177	0.821	1.889
0.421	0.083	0.281
		2.046
	1.163	2.58
0.395	0.426	0.523

0.964	1.259	0.595
0.665	0.768	0.477
0.411	0.483	0.645
2.386	0.162	2.093
1.453	1.066	0.962
0.558	1.13	0.586
0.67	1.479	0.223
1.369	0.735	0.918
1.888	0.858	1.305
1.368	1.554	0.84
0.402	1.091	0.834
0.855	1.596	0.271
0.804	4.732	0.496
	0.875	0.697
0.653	0.196	0.232
0.761	0.609	0.851
0.169	0.313	1.782
1.464	0.694	1.556
1.677	1.338	0.643
0.958	0.502	1.085
0.693	0.581	5.538
0.878	0.945	0.506
1.528	1.388	0.933
0.788	0.581	0.611
1.103	1.447	0.287
1.428	1.006	0.507
1.123	0.773	0.622
0.241	0.168	0.184
1.247		
0.927	0.962	0.562
1.231	0.909	1.149
2.099	1.669	0.925
1.191	1.411	0.893
0.935	1.061	1.074
0.41	0.714	2.381
0.335		
0.383	0.491	
1.155	0.906	1.381
1.4	1.036	0.527
0.932	0.513	1.844
0.878	1.033	1.052
1.803	1.719	0.835
1.025	1.06	0.371
1.358	1.301	0.948
0.706	0.998	0.744
1.066	1.27	1.445

1.635	0.812	0.466
0.523	0.959	0.501
1.256	0.82	1.674
1.3	1.136	1.094
0.777	1.171	0.466
0.986	1.038	1.508
1.009	0.703	0.61
0.874	1.369	1.137
1.611	0.914	1.181
0.979	1.885	1.625
	0.208	0.434
	1.019	1.641
1.091	1.52	1.697
1.588	0.675	1.069
0.909		
1.638	1.559	1.49
1.524	1.356	2.345
0.711		2.499
	1.03	1.458
0.402	0.394	0.384
1.115	1.364	1.225
0.591	1.289	1.777
1.206	0.733	1.093
0.774	0.705	0.467
0.133	0.454	1.083
1.207	1.62	0.61
0.433	0.938	0.521
0.833	1.618	2.744
	0.318	0.479
1.162	1.391	0.437
1.174	1.265	1.429
1.112	1.393	1.501
1.129	1.185	2.266
0.891	1.666	1.252
0.805	1.25	1.327
1.321	1.677	2.544
0.809	1.037	0.256
3.882		0.619
	1.302	8.008
1.249	1.016	1.258
0.576	0.957	2.377
0.554	0.271	0.807
0.06	0.214	1.382
0.646	0.609	1.783
1.038	1.04	1.222
0.394	0.383	0.432
0.631	1.059	1.652
1.328	1.127	1.008

1.29	1.013	0.787
0.224	1.875	4.906
0.229	0.384	0.584
1.495	1.003	2.251
0.997	0.634	
1.263	1.258	1.453
0.48	0.278	0.312
0.906		
0.615	0.577	2.275
1.21	0.977	0.49
0.803	0.992	3.256
	0.74	0.951
0.54	0.546	0.664
2.23	5.988	0.719
0.307	0.621	0.675
0.934	1.642	0.462
1.85	1.171	1.216
0.56	1.14	0.25
0.815	0.884	1.788
0.869	0.534	0.622
1.181	1.669	1.563
0.544	0.872	1.153
0.88	0.674	0.428
2.337	0.696	0.43
0.654	1.123	0.726
0.428	0.334	2.298
1.322	0.532	1.249
0.334	0.596	
0.611	0.853	0.876
1.44	1.044	0.434
0.38	0.125	0.215
		1.476
0.557	0.813	0.685
0.714	0.891	1.878
1.075	1.423	1.223
1.21	0.997	1.158
0.849	0.775	0.593
1.032	1.08	1.144
1.402	1.49	0.786
2.779		2.338
0.235	0.154	1.275
0.628	1.688	1.107
1.829	1.171	1.933
0.298	1.632	7.956
1.465	1.394	2.457
1.814	0.627	1.383
1.102	1.378	0.83
0.654		3.113
0.063	0.736	0.29
	0.33	

	0.811	0.956
0.232		0.729
1.167		
0.845	0.451	0.191
1.897	0.835	1.397
1.421	1.008	1.135
1.104	1.039	1.494
0.337	1.383	0.986
0.122	2.532	3.612
0.759		0.684
1.078	1.134	0.624
0.892	0.889	0.47
0.887	1.026	0.835
0.099	1.412	2.42
	1.489	2.298
0.529	0.104	1.241
1.879	2.617	0.414
0.2	0.665	0.435
	0.281	0.503
1.064	0.735	0.673
0.116	5.773	
1.023	1.259	1.531
2.135	1.454	0.934
		0.609
1.235	1.659	1.937
1.555	1.159	0.362
2.112	1.119	1.21
1.512	1.153	2.809
1.465	1.456	1.409
0.324	1.122	0.682
0.768	1.075	0.5
0.214	0.427	0.251
1.261	1.239	1.545
1.319	1.03	1.619
1.609	1.271	0.33
0.953	1.108	2.733
0.913	0.357	1.949
1.617	0.752	0.682
0.441	0.922	0.211
1.293	1.272	
0.544	1.29	0.816
0.611	1.893	
4.418	4.672	4.28
0.536	0.747	0.554
		1.901
0.673	0.539	1.844
1.793	0.985	1.331

0.215	0.824	0.251
1.262	1.199	1.659
0.859	0.761	0.539
0.731	1.44	0.47
3.319	1.269	0.454
0.234		3.03
0.257	0.595	1.127
0.053	1.38	7.286
		2.001
0.552	1.613	0.377
0.194	0.299	0.26
0.926	0.621	2.044
1.816	0.566	1.35
0.974	1.057	0.911
0.821	0.72	0.573
3.454	0.84	1.003
0.322	0.672	0.673
	0.618	0.156
0.492	0.59	0.921
0.446	0.867	1.577
0.204	2.751	4.875
1.12	2.589	1.441
0.269	0.096	0.116
1.035	0.958	1.416
0.843	1.138	0.639
0.688	0.721	0.931
1.358	1.688	1.836
1.263	0.969	0.561
0.841	0.737	1.097
1.084	0.7	0.618
0.841	0.976	1.236
0.853	2.22	3.111
0.966	1.097	0.856
1.293	1.238	1.19
1.431	1.889	0.546
1.303	1.403	0.72
0.481	1.132	0.792
0.942	0.88	1.781
2.636	1.233	0.769
3.143	0.586	0.306
0.57		1.589
1.316	1.428	1.018
0.959	0.562	5.208
1.065	1.389	0.803
1.085	1.108	1.349

1.158	0.975	1.441
1.316	1.541	0.731
1.523	1.089	0.897
0.464	1.244	1.5
1.303	1.065	1.81
1.392	0.808	1.251
0.33	0.188	0.276
1.647	1.536	1.497
1.004	0.807	0.603
0.857		
1.27	1.071	0.733
0.8	1.296	0.466
1.869	1.562	1.221
1.186	1.196	1.157
1.471	1.379	0.951
0.145		
1.056	0.789	1.989
1.486	0.844	0.973
0.627	0.681	0.367
0.344	1.047	0.394
1.773	0.874	0.884
1.165	1.091	0.807
0.75	0.711	0.711
0.851	0.628	0.621
0.684	0.327	0.242
1.987	1.396	0.983
2.051	0.419	1.672
0.93	1.539	0.482
0.364	0.918	
0.472	1.718	0.835
1.26	0.737	1.073
0.393	0.146	0.269
1.163	1.03	2.803
1.244	0.883	1.227
1.295	0.861	1.673
0.564	1.048	0.962
1.019	1.061	0.728
1.189	0.767	0.722
0.725		
0.633	1.084	1.461
0.983	0.247	0.989
2.04	1.766	0.79
1.265	0.994	1.361
0.846	1.175	0.99
5.115	1.549	2.533
0.24	0.478	0.355
1.591	1.005	1.221

1.463	1.045	1.372
1.207	1.051	1.211
2.05	1.875	1.506
0.385	0.881	0.994
1.153	0.949	1.094
0.708	1.341	0.652
0.928	1.363	0.335
1.381	0.799	1.81
1.052	1.178	1.49
1.62	1.212	1.216
0.07	1.31	0.845
1.257	1.279	1.346
1.452		
1.136		0.61
		0.907
1.236	1.27	0.769
1.068	1.07	2.631
1.583	1.428	2.921
0.681	0.786	
1.432	0.676	1.793
		1.445
0.57	0.379	0.759
1.137	0.82	0.701
0.829	1	0.878
0.425	1.184	0.511
	3.868	0.797
1.112	0.645	1.847
0.753	1.305	6.697
1.41	1.336	1.028
0.983	0.896	0.809
1.283	0.75	1.372
0.526	0.705	1.166
0.284	0.923	2.927
1.336	0.824	0.495
1.669	0.879	1.245
2.252		
0.088		1.343
1.047	1.052	1.165
0.27	1.142	0.948
0.9	1.128	0.821
1.018	0.958	1.218
1.355	1.053	1.586
1.122	0.854	0.532

2.635	1.223	0.512
	0.589	0.757
0.855	1.62	0.53
0.108	1.54	5.503
1.626	0.983	1.08
1.74	0.989	1.871
0.892	0.931	0.854
0.572	0.992	1.433
0.462	0.43	0.311
0.562	1.154	2.492
1.564	0.795	1.407
1.084	1.107	1.52
0.896	0.741	0.982
0.685	0.217	
0.851	1.106	2.527
1.135	0.386	1.179
0.669	0.68	1.012
1.801	1.382	0.279
1.121	0.713	5.35
0.629	1.494	0.681
0.905	0.576	1.043
1.581	1.846	0.637
1.435	1.457	0.962
0.593		
1.043	1.396	0.923
1.164	1.185	1.27
1.706	0.643	1.548
0.881	0.485	
1.284	0.888	1.031
0.54	0.735	2.352
1.098	1.005	0.931
1.569	0.705	0.985
0.486	0.098	
1.408	0.986	1.003
0.557	1.192	2.103
1.227	1.034	1.037
0.793	1.318	0.665
1.296	0.855	0.851
1.275	0.738	0.55
0.439	0.851	1.364
0.577	0.827	1.102
0.603	1.463	1.711
1.249	1.107	0.689
2.205	1.109	0.849
0.358	0.93	0.396
0.642	0.936	1.009

1.249	1.415	0.85
0.775	0.846	
1.584	0.885	2.164
0.261	1.314	2.435
0.846	0.662	1.443
1.616	1.339	0.977
2.164	1.368	1.356
1.847	0.36	0.636
1.715	1.09	0.753
0.733	1.28	1.903
1.262	0.731	0.836
0.19	0.836	0.778
1.23	1.551	1.088
1.485	1.509	2.062
0.373		
1.419	0.735	1.161
0.234	0.543	1.424
0.465	0.958	1.111
1.203	0.736	
1.61	1.093	3.113
1.717	0.508	1.797
0.838	1.259	1.111
0.167		
1.008	1.054	0.748
0.937	1.223	1.617
0.645	0.933	1.158
0.26	1.373	0.982
0.416	1.286	1.09
0.899	0.797	1.514
		1.604
0.276	1.448	2.182
0.25	0.409	0.541
0.877	0.456	2.05
0.753	1.455	0.918
0.268	0.458	0.408
0.963	1.106	5.981
1.4	1.133	1.742
0.595	0.594	0.788
1.19	0.823	
0.827	0.842	0.717
1.066	0.597	1.615
2.51	1.221	1.348
0.157		
1.688	0.669	2.086
	0.933	

0.543	0.425	0.864
1.508	1.076	1.272
0.267	0.646	0.927
0.693	1.244	0.853
0.493	0.1	0.622
0.804	0.364	0.504
0.724	1.4	0.756
1.344	0.685	0.94
1.14	1.306	1.042
0.19	0.172	
1.45	1.258	1.452
0.446	2.281	2.236
2.107	0.97	1.158
0.172	0.328	0.437
1.428	1.064	1.321
2.993	2.58	1.528
0.736	0.576	0.567
2.966	0.983	
0.223	0.477	0.788
1.356		
1.557	1.376	
0.803	0.881	0.23
0.234	1.699	
0.651		0.295
0.238		
0.425	0.96	0.918
0.809	1.282	0.257
0.453	0.412	0.332
0.907	1.26	1.337
3.465	0.315	0.368
	1.084	1.122
1.1	1.09	0.926
1.276	1.027	1.924
1.575	0.921	0.26
1.104	0.98	1.368
1.872	0.803	1.973
1.158	0.883	1.133
1.71	1.205	1.152
1.292	1.292	1
0.921	0.885	1.041
1.02	0.619	1.99
1.156	2.076	2.421
1.284	0.878	0.879
0.924	0.156	0.752
0.791	0.935	0.84

0.125	1.301	4.798
0.876	1.422	0.845
0.536	1.94	2.897
2.501	0.67	1.949
1.177	0.705	1.445
1.054	1.061	0.712
0.623	0.358	0.268
1.665	2.282	0.633
1.443	1.261	0.547
0.705	1.899	0.599
1.04	1.558	1.585
1.004	0.763	1.125
0.418	1.061	2.306
0.681	0.669	1.027
0.643	1.146	2.011
0.157		0.286
0.355	1.068	2.457
0.12	1.609	0.867
1.234	1.431	1.119
0.876	0.868	1.103
0.877	1.217	0.625
3.505	0.318	2.51
1.726	0.953	1.438
1.288	1.162	0.882
1.134	1.296	0.869
1.119	1.008	
1.034	0.805	1.23
0.879	1.331	0.901
0.715	1.059	0.685
0.978	0.865	1.153
0.756	0.783	0.777
1.189	1.251	1.238
1.345		0.898
1.516	1.228	0.779
0.448	0.915	1.237
1.795	1.104	1.529
1.1	1.807	0.721
0.309	0.48	0.347
0.344	0.725	0.377
0.308		
0.017	0.023	0.24
0.162	1.116	0.498
0.502		
1.855	1.489	2.294
0.183	0.861	0.573

1.36	1.039	0.6
1.752	0.939	0.679
3.679	1.215	1.49
0.704	0.941	
		0.79
0.303	0.734	0.931
1.119	1.119	1.734
	0.988	
1.176	1.656	0.267
0.345	0.434	0.109
1.025	0.4	2.207
1.04	1.07	1.217
0.907	1.786	1.691
1.635	1.115	0.994
1.022	0.695	1.217
0.965	1.127	0.598
0.369	1.087	0.719
1.653	0.653	0.551
0.675	0.826	3.127
0.602	1.137	1.924
1.18	1.247	0.683
1.244	0.913	1.152
0.146		1.853
0.878	1.233	0.924
1.309	1.332	0.676
1.064	0.714	0.646
0.827	1.017	0.419
1.133	0.953	0.571
1.57	1.12	2.23
1.45	0.467	1.216
0.237	0.023	0.049
	0.175	
0.072	0.127	0.121
0.962	0.937	1.054
	0.892	0.582
1.025	1.878	1
1.417	0.427	0.703
0.73	1.123	1.215
0.394	0.59	0.692
0.344	1.908	1.534
1.06	0.535	0.92
0.819	0.644	0.392
1.329	1.148	1.228
1.213	1.228	0.862
		2.395

1.637	1.208	0.476
0.676	0.734	1.161
0.771	1.132	1.096
1.043	1.261	
	0.217	
0.922	0.748	1.011
0.789	0.557	1.233
1.082	1.979	0.921
0.388	1.99	5.316
1.023	3.201	1.084
0.355	0.259	4.913
1.152	0.527	0.279
0.505	0.854	0.335
1.354	0.882	1.15
0.598	0.946	0.518
0.228	1.157	1.423
	1.948	
	1.963	
0.762	1.273	1.591
0.472	0.588	0.513
1.618		2.248
0.086		
0.717	0.93	1.379
1.32	1.237	0.465
1.025	1.255	1.34
1.177	1.343	1.003
1.076	1.35	0.986
0.82	1.124	0.899
0.439	0.94	2.072
1.197	1.018	
0.607		
1.203	0.707	0.683
0.87	0.512	0.622
0.17	0.276	0.215
	0.724	0.782
0.721	0.822	0.429
0.815	1.142	0.728
1.043	0.878	1.063
1.192	0.754	1.366
1.096	1.172	0.672
1.1	1.196	1.348
0.085		0.805
1.345	0.795	0.839
0.85	1.462	0.478
1.566	0.401	0.343
0.489	0.661	0.067
0.38	0.346	4.424
0.294	1.617	5.462

	0.848	0.526
0.526	1.37	0.995
4.341	1.215	0.549
0.826	1.001	0.73
2.212	0.221	1.451
1.161	0.971	1.187
1.137	2.28	1.017
1.702	1.253	0.621
0.228	2.663	0.613
1.067	0.5	0.987
0.916	1.112	0.928
1.653	1.204	0.238
1.13	1.381	0.418
0.963	0.409	1.813
		1.062
0.156	1.672	3.42
0.455	0.503	0.392
1.909	1.078	0.426
1.775	1.486	0.293
1.534	1.927	0.919
1.725	1.331	0.583
3.956	2.073	1.931
	0.444	11.655
0.222	1.305	2.409
1.318	1.135	0.742
0.272		5.266
1.449		
1.105	0.882	0.959
0.459	0.793	0.391
	0.396	
1.23	1.256	1.608
0.737	1.306	0.556
0.803	0.763	1.304
0.063		
0.345	2.756	
1.076	1.163	0.581
1.027	1.307	0.812
0.473	3.456	0.155
0.768	0.971	3.991
0.725		
1.742		
0.832	1.173	0.171
1.503	0.587	1.106
0.916	1.25	1.16
0.14	0.262	0.241

1.442	0.778	0.732
1.491	2.443	0.713
1.817	1.125	1.471
1.527	1.173	1.373
1.258	0.614	3.622
3.49	0.321	0.578
0.824	0.677	1.156
0.45		
0.197	0.133	0.05
0.384	1.311	
	0.718	0.38
0.474	0.125	0.288
1.12	1.097	0.806
0.899	1.681	0.769
		0.694
0.306	1.356	1.23
0.95	1.429	0.499
0.86	1.041	1.642
0.677	0.596	0.461
1.176	2.084	0.34
0.63	0.489	0.457
1.5	3.809	1.798
0.519	1.065	1.11
0.94	0.929	0.642
1.064	1.055	1.615
1.429	0.728	0.464
1.168	0.473	
1.61	1.259	0.888
0.282	0.24	0.354
0.851	0.801	1.277
		2.335
0.176	1.306	1.194
2.392	1.597	0.279
0.741	1.919	0.413
0.408	0.866	0.128
2.912	2.08	0.227
1.122	1.446	0.561
1.205	1.262	1.17
0.475	0.675	0.581
0.455	0.824	0.288
1.019	0.846	
0.424	1.32	1.502
0.501	1.656	
1.245		0.589

1.524	0.763	1.328
1.182	0.814	1.22
1.666	1.534	0.761
		0.624
0.11	0.38	0.321
0.942	1.367	0.471
0.254	0.436	1.371
0.574	0.82	0.552
1.522	1.657	1.428
0.216	0.249	0.62
1.112	0.849	1.335
1.214	0.738	0.707
0.465	0.643	0.789
1.333	1.199	1.935
0.708	0.708	0.818
1.155	1.504	
1.014	1.292	0.466
1.688	2.027	1.925
0.392	0.507	0.631
1.498	1.014	0.465
1.109	1.06	1.091
0.852	0.673	0.944
0.887	1.938	1.596
1.294	1.042	
0.736	0.794	0.968
0.952	1.192	1.418
0.579	1.505	1.224
0.672	0.817	1.186
1.511		
0.927	0.81	1.929
1.34	1.229	
1.515	1.64	0.568
1.294	1.641	1.147
0.88	0.88	0.732
1.263	0.947	2.079
		0.448
	0.892	2.884
0.612	1.753	3.203
0.491	0.822	0.714
1.109	1.122	0.86
1.349	0.699	1.782
0.762	0.48	1.611
	1.002	
0.476	1.567	1.872
1.084	1.636	0.395
0.969	1.104	0.59
1.348	2.444	0.343
0.603	1.299	0.944

0.523	0.548	0.58
1.111	0.574	1.312
	1.278	
1.549	1.074	1.464
1.021	1.111	2.225
		0.104
1.034	1.028	1.244
0.371	1.653	2.138
0.473	0.297	1.314
0.985	1.938	
1.806	2.518	0.752
1.034		0.689
1.527	1.411	0.844
		1.945
0.509	1.088	1.607
	1.037	
1.376	1.142	
0.612	0.952	0.781
1.182	0.976	1.285
0.551		
	0.967	1.462
1.145		2.096
0.341	0.33	0.373
0.454	0.835	
1.19	0.844	0.838
0.952	1.249	0.787
1.02	0.722	1.142
0.497	0.523	0.163
1.32	0.672	0.77
1.648	1.012	0.883
0.852	1.2	0.817
0.306	0.24	0.504
0.787	0.872	0.995
0.927	1.225	2.46
0.597	1.846	0.642
0.874	0.948	0.13
0.383	0.393	
1.014	1.359	0.886
0.782	0.579	0.258
0.831	1.015	0.919
0.311		0.119
1.555	1.095	1.413
0.768		
2.175	0.632	1.544
0.593	0.672	0.746

1.597	0.776	
0.335	0.606	1.588
0.809	0.907	1.385
1.489	1.458	1.387
0.774	0.631	0.531
0.759	1.506	
0.606	1.513	0.469
0.591	0.427	1.132
1.414	1.122	0.87
1.7	0.499	0.696
0.57		1.172
1.162	1.402	1.292
0.332	0.959	0.582
1.243	1.363	0.883
0.674	0.685	0.304
0.907	0.774	1.019
1.766	0.797	1.388
		0.442
0.833	0.583	
0.968	0.875	0.776
0.65	1.644	1.241
1.664	1.112	1.261
1.748	0.918	1.065
	2.749	3.372
1.037	0.763	1.849
1.315	1.297	0.272
1.432	0.799	1.213
1.996	0.69	0.85
1.509	0.753	1.234
1.36	0.662	1.063
1.543	0.93	1.32
0.427	0.007	0.013
0.231	1.273	0.392
0.321	0.634	1.202
0.177		1.672
0.449		3.58
1.282	0.947	1.407
0.912	0.674	1.524
0.42	0.876	0.172
1.269	0.434	1.722
1.105	1.118	1.407
0.572	1.11	0.855
0.4	0.603	0.293
0.466		
1.233	0.813	1.398
1.31	0.735	1.173

1.91	2.346	2.102
0.399	0.774	0.986
1.406	1.302	2.391
1.874	0.791	0.661
1.303	1.41	0.806
1.064	1.27	0.821
2.888	2.036	0.746
1.353	0.916	0.963
1.266	0.79	0.865
0.983	1.025	0.558
0.499	0.975	0.534
1.442	0.98	1.706
1.31	1.091	1.005
1.095	1.071	0.728
0.81		0.632
1.147	0.835	1.464
1.075	1.252	1.338
0.525		1.026
1.876	1.39	0.881
2.392	1.401	
1.1	0.948	1.112
1.558	0.934	
1.451	1.279	1.576
0.967		1.044
1.278	0.814	1.335
1.634	0.919	0.836
1.174	1.419	1.545
0.868	1.152	2.018
0.832	1.189	0.8
1.091	1.104	1.446
2.275	0.468	2.169
0.688	0.712	1.831
1.016	0.855	1.352
0.424	0.407	0.443
0.35	1.874	
0.972	0.802	0.982
1.3	1.412	2.787
1.167	1.292	0.172
0.616	0.776	1.598
0.374	0.648	0.598
1.656	1.129	1.158
1.177	0.963	0.913
0.94	1.084	
1.148	1.143	
1.43	1.844	1.877
1.094	1.084	1.299

2.276	1.039	
1.549	1.683	0.915
0.113	0.208	0.307
0.923	0.978	0.964
		0.596
3.442	2.045	0.814
1.52	0.956	1.133
1.223	0.854	1.2
0.968	1.044	0.587
0.878	0.909	1.544
1.345	0.979	1.477
0.619	1.652	0.544
0.909	1.714	0.887
1.85	0.649	0.952
2.471		
1.284	1.75	1.593
0.358	0.465	0.315
0.87	0.428	0.674
1.798	0.729	0.639
1.324	1.203	1.346
1.269	1.131	2.627
0.854	0.856	1.45
0.938	1.965	1.293
1.08	1.388	
0.363	1.65	
1.109	0.925	0.678
0.87	0.853	0.874
1.073	1.182	0.762
1.244	2.671	7.539
0.797	0.72	1.011
2.041		0.777
	1.337	
0.1		0.517
1.081	1.036	1.318
0.513	1.01	0.956
0.933	1.15	1.058
0.852	1.631	0.067
1.123	1.081	1.241
1.226	0.371	2.108
1.376	0.466	0.321
0.163		0.156
1.045	0.996	1.062
1.292	1.24	0.872
1.42	2.194	1.791
0.611	1.659	1.566
1.489	0.818	1.274
0.882	0.956	0.633

0.981	1.113	0.554
1.3	0.529	0.709
1.757	0.63	0.826
0.42	0.697	0.595
0.846	0.609	0.984
0.513	1.236	
1.748	1.633	1.408
1.078	0.603	1.151
0.343	1.816	0.723
1.54	1.075	0.408
1.789	1.287	1.581
1.902	0.305	
1.409	0.964	1.472
1.325	1.019	0.363
0.926	1.42	1.436
0.988	0.801	1.329
0.516	0.685	0.485
0.874	0.992	0.38
1.361	1.1	0.772
1.591		
1.367	1.665	1.785
1.07	1.163	1.227
1.419	1.706	0.459
0.242	3.154	3.418
1.329	1.118	1.487
0.883	1.803	0.582
1.357	1.179	1.369
0.328	0.314	0.292
1.929	0.328	1.398
0.461	0.515	0.205
1.336	0.702	0.792
0.372	2.019	1.474
1.019	0.973	0.888
0.888	0.691	0.579
	0.179	
	0.499	0.424
1.258	0.812	0.853
1.479	0.906	0.96
3.303	1.253	1.042
0.211	1.568	0.956
0.64	1.132	1.784
		1.184
0.926	1.054	0.693
	1.21	2.057
1.737	1.617	1.458

1.516	1.063	
0.458		
0.706	1.065	1.126
0.907	1.566	0.389
1.312	1.126	2.455
1.345	1.151	0.9
1.349	0.833	1.879
0.085	0.103	0.509
2.094	0.762	0.514
0.2	0.691	0.616
1.292	0.851	1.391
0.939	1.077	1.292
1.726	0.921	1.579
0.971	0.99	1.91
1.227	0.7	0.665
3.172	0.959	0.927
