# Supplementary material for: What Are the Reliable Plasma Biomarkers for Mild Cognitive Impairment? A Clinical 4D Proteomics Study and Validation
Source: Mediators Inflamm. 2024 May 27;2024:7709277. doi: 10.1155/2024/7709277 (PMC11178428; doi:10.1155/2024/7709277)
Supplement: Supplementary 6 — Power Analysis Simulation Result. [file 7709277.f6.pdf]

Effect_size	Sample_size	Statistical_power
1.5	5	0.549385598
1.5	10	0.886970202
1.5	15	0.97743189
1.5	20	0.996099693
1.5	25	0.999391177
1.5	30	0.999911987
1.5	35	0.999988024
1.5	40	0.999998449
1.5	45	0.999999807
1.5	50	0.999999977
1.5	55	0.999999997
1.5	60	1
